# Biodiversity of the abyssal and hadal zone in the Nova Canton Trough (Central Pacific Ocean; 2751–7989 m): a visual inventory

**DOI:** 10.3897/BDJ.14.e204436

**Published:** 2026-09-15

**Authors:** Denise J. B. Swanborn, Alfredo Marchiò, Giulia La Bianca, Megan Cundy, Travis Reynolds Jr, Melanie S. Stott, Brett C. Gonzalez, Heather A. Stewart, Alan J. Jamieson

**Affiliations:** 1 Inkfish LLC, Perth, Australia Inkfish LLC Perth Australia; 2 Minderoo-UWA Deep-Sea Research Centre, School of Biological Sciences and Oceans Institute, The University of Western Australia, Perth, Australia Minderoo-UWA Deep-Sea Research Centre, School of Biological Sciences and Oceans Institute, The University of Western Australia Perth Australia; 3 Kelpie Geoscience Ltd, Edinburgh, United Kingdom Kelpie Geoscience Ltd Edinburgh United Kingdom

**Keywords:** Baited camera, baseline survey, deep-sea biodiversity, marine imaging, morphotaxa, submersible surveys, video-based observations

## Abstract

Comprehensive biodiversity inventories of abyssal and hadal ecosystems remain rare due to the financial and logistical challenges associated with operating in the deepest parts of the ocean. However, deep-sea exploration programmes are increasingly generating extensive video datasets offering new opportunities to document biodiversity across broad depth ranges.

Here, we present the most extensive visual biodiversity inventory assembled from an abyssal-hadal system to date, using imagery collected in the Nova Canton Trough (Central Pacific Ocean), combining observations from 99 baited lander deployments and 16 submersible transects conducted between ~ 3000 m and ~ 8000 m depth. Across more than 1500 hours of lander video and 136 hours of submersible video, ~ 30,000 individual organisms belonging to the kingdoms Animalia and Protista were recorded and grouped into 325 morphotaxa, spanning a wide range of benthic and demersal taxa. The integration of baited and non-baited visual platforms provided complementary observations of mobile scavengers and sessile or low-mobility benthic taxa.

Combined observations reveal a depth-structured transition from a diverse abyssal biota through a broad abyssal-hadal transition zone to a distinctive hadal assemblage. Notable findings include the first population of hadal snailfish (*Pseudoliparis* sp.) reported outside a subduction trench, high density and diversity of xenophyophores across the depth range and several putative bathymetric range extensions, including first hadal records for several groups within the Animalia and Protista. At phylum level, these include live Brachiopoda and direct observations of protists belonging to the Cercozoa. The orders Corallimorpharia (Phylum Cnidaria, Class Hexacorallia) and Nacromedusae (Phylum Cnidaria, Class Hydrozoa) are extended by over 1200 m, with the former being the first hadal record. The families Amathillopsidae (Phylum Arthropoda, Order Amphipoda), Candelabridae (Phylum Cnidaria, Order Anthoathecata), Hesionidae (Phylum Annelida, Order Phyllodocida), Onuphidae (Phylum Annelida, Order Eunicida) and Umbellulidae (Phylum Cnidaria, Order Scleralcyonaceae) are also depth-extended, with the first three representing first records at hadal depths.

The Nova Canton Trough also differs from most previously studied hadal systems in being a tectonically quiescent fracture zone rather than a subduction trench. This makes it an important reference system for evaluating whether depth-structured faunal zonation is consistent across different geological contexts. Its position in the central Pacific provides another important biogeographic relevance connecting existing hadal studies focused on the north and south Pacific.

By combining observations with curated reference imagery and identification notes, this study establishes a biodiversity baseline for the Nova Canton Trough and provides a visual framework to guide future taxonomic identification, ecological studies and targeted biological sampling, particularly at lower abyssal and hadal depths where biodiversity remains most poorly resolved.

## Introduction

Inventories of deep-ocean biodiversity, particularly at hadal depths (> 6000 m) remain sparse due to financial, technical and logistical constraints that have historically delayed sampling efforts at depth relative to shallower systems. Existing information on faunal communities in the oceans' deepest geomorphological features is often focused on specific taxa, for example, fish (Jamieson et al. 2021a), isopods (Brandt et al. 2024), amphipods (Ritchie et al. 2015), copepods (Bonk et al. 2025), glass sponges (Marchiò et al. 2025b) and annelids (Gonzalez et al. 2025). These types of studies often collate data from one or more features at broad geographic scale, but with sparse records within individual locations. More comprehensive single-location studies are emerging to examine hadal diversity (Brandt et al. 2020, Brandt et al. 2026), but many are typically limited to one type of sampling strategy (e.g. Jamieson et al. 2021b, Zhang et al. 2021, Jamieson and Weston 2023). Another challenge is that biological studies at hadal depths have often focused on the very deepest parts (e.g. Gallo et al. 2015) or within the confines of the hadal depth range (e.g. Blankenship et al. 2006), making it difficult to resolve patterns in diversity and community structure relative to surrounding areas and across continuous depth ranges.

These challenges are compounded by the remoteness of hadal features, which drives the cost of vessel time. The technical constraints at large distances from the surface also limit the use of conventional sampling gear and require specialist, often bespoke equipment. Most modern research vessels do not carry enough wire to deploy collection gear such as trawls, corers and grabs to hadal depths, resulting in a dearth of physical samples to underpin taxonomic inventories. As such, many modern hadal studies are performed using video-based systems, on either free-fall landers or submersibles that can cover larger areas of seafloor and document a far greater number of animals (e.g. Jamieson et al. 2021a, Jamieson and Linley 2021, Zhang et al. 2021, Jamieson et al. 2022, Jamieson et al. 2023, Jamieson et al. 2026, Stewart et al. 2026, Swanborn et al. 2026a). Increasingly, deep-sea expeditions are generating extensive archives of imagery capturing both biodiversity and habitat context. In many cases, organisms documented in these datasets are identified using morphotaxa frameworks, based on observable morphological characteristics rather than formal taxonomic identifications (e.g. Simon-Lledó et al. 2019, Swanborn et al. 2025a, Jamieson et al. 2026). High-quality imaging and video surveys also offer empirical advantages over extractive methods like trawling. As camera systems mounted on submersibles or other subsea platforms are non-destructive, they facilitate more accurate *in-situ* observations, including those of behaviour and habitat associations. Annotated datasets provide comprehensive census and environmental data, while serving as a permanent, verifiable record of the regional biota.

Image-based identifications are nonetheless constrained by existing knowledge about fauna in the region, image quality, environmental conditions and the visibility of diagnostic characteristics and are ideally used in conjunction with physical sampling (Hanafi-Portier et al. 2021, Bribiesca-Contreras et al. 2022, Swanborn et al. 2026b). Numerous morphologically cryptic species exist in the deep sea (Vrijenhoek 2009, Brandt et al. 2012, Brandt et al. 2026), meaning that true taxon richness is almost certainly higher than visual inventories alone can capture. Vice versa, morphologically distinctive life stages of a single species may also exist, which risks inflating diversity through assigning different names to genetically equivalent organisms (Torres et al. 2014).

Ideally, both types of data would be collected concurrently, but until the challenges of collecting physical samples at the same frequency as in shallower waters are addressed, video-based data will continue to play a large role in establishing the ecology of hadal systems (Jamieson et al. 2026, Swanborn et al. 2026b). When combined with a consistent identification framework and transparent annotation protocols, such datasets serve to better understand the species pool of the area and form the basis of future research where physical sampling strategies can be augmented by such morphotaxa inventories. Despite its potential, visual data remain significantly under-exploited due to a lack of automated analysis tools, requiring researchers to manually process vast image libraries and terabytes of video footage. This annotation bottleneck, constrained by the need for expert observers, standardised protocols, and substantial funding, is a primary challenge in marine ecological monitoring (Game et al. 2025) and can benefit from imagery-based guides as reference.

Here, we present the first integrated video-based biodiversity inventory of the Nova Canton Trough, combining observations from baited lander deployments and submersible transects conducted across water depths ranging from ~ 3000 m to 8000 m. The Nova Canton Trough, located in the Central Pacific Ocean, represents a deep geomorphological feature extending to depths below 8000 m. Unlike the majority of hadal systems studied to date, which occur in seismically active subduction trenches, the Nova Canton Trough is a tectonically quiescent fracture zone providing an opportunity to examine whether depth-structured faunal zonation documented from subduction trenches is consistent across different hadal geological contexts. Despite its considerable depth, the biological communities inhabiting this feature have remained undocumented since its discovery over 60 years ago (Menard 1964, Rosendahl et al. 1975). The trough also occupies a geographically key position between the better known hadal zones of the northwest and the southwest Pacific (Gallo et al. 2015, Linley et al. 2017, Brandt et al. 2020, Zhang et al. 2024, Swanborn et al. 2025a, Swanborn et al. 2025b, Brandt et al. 2026, Jamieson et al. 2026).

The objectives of this study are to document morphotaxa richness and depth distribution of observed taxa across the full abyssal-hadal depth gradient of the Nova Canton Trough and to provide a curated visual reference to support future taxonomic identification, ecological research and targeted biological sampling within the region. In doing so, this study represents the most extensive visual biodiversity inventory of a hadal system to date, establishes a biodiversity baseline for one of the least documented hadal systems in the central Pacific and highlights the value of video-based approaches for resolving biological patterns across the full depth range of abyssal and hadal ecosystems.

## Materials and methods

### Study site

**Figure 1.**
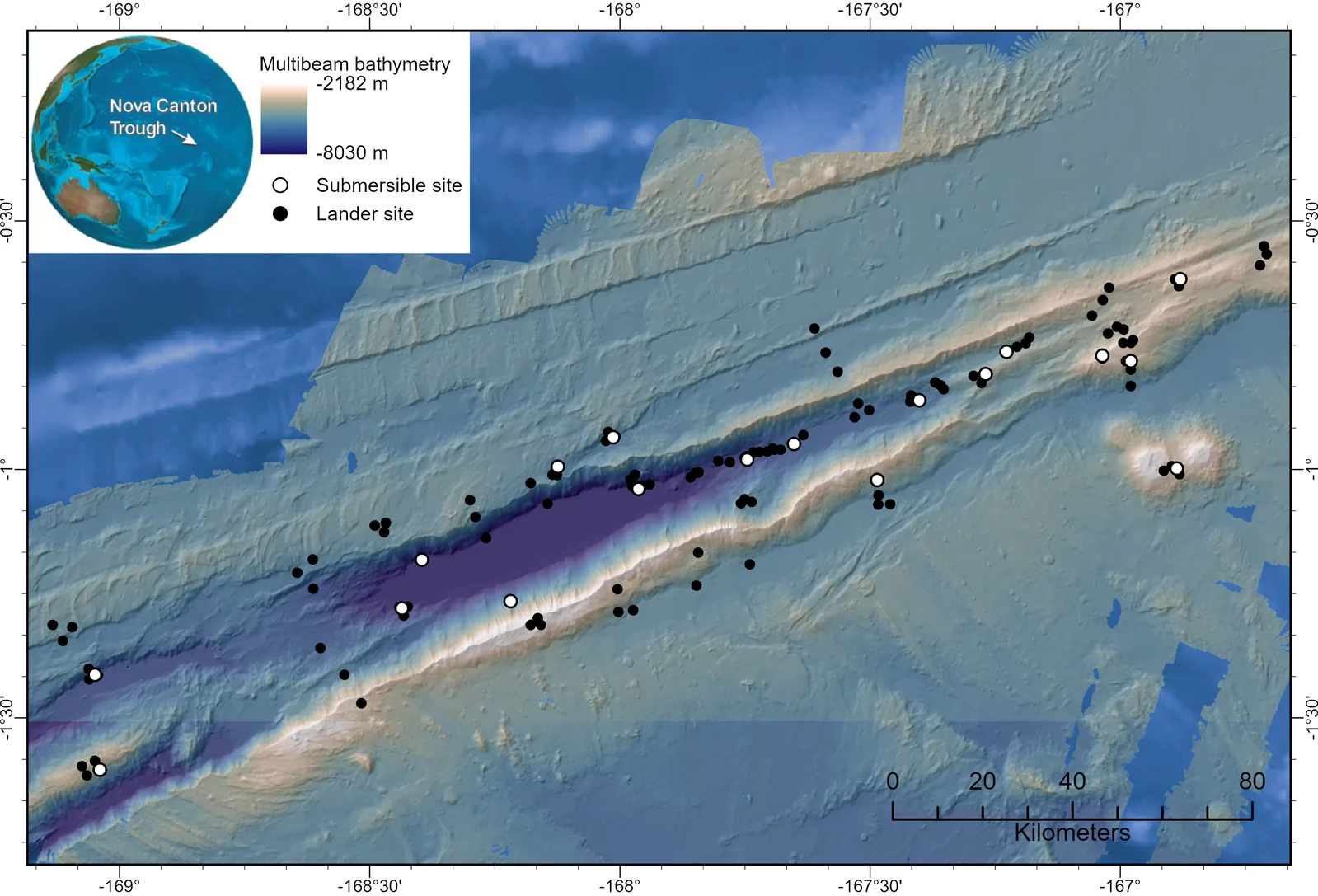
The Nova Canton Trough, with high-resolution multibeam bathymetry mapping and sample sites acquired during the Inkfish Expedition conducted in 2024. Black circles indicate baited lander deployment locations and white circles indicate submersible dive locations. Seafloor topography is shown using a multibeam bathymetry grid with depth ranging from 2182 to 8030 m. Areas outside the multibeam survey coverage are shown using a background regional bathymetric grid from the GEBCO Compilation Group (2025). Deployment details are presented in Suppl. material 1. The location of the Nova Canton Trough in the central Pacific Ocean is indicated using the inset.

The Nova Canton Trough, first described in detail in 1964 (Menard 1964), is located in the Central Pacific Ocean, west of the East Pacific Rise, between the Line Islands and Phoenix Islands (Fig. 1). It trends east-northeasterly between approximately 2°S and 2.2°N, extends for ~ 800 km and is typically 10-30 km wide, widening to the west-southwest. Despite its name, the Nova Canton Trough was first thought to be an abandoned spreading centre (Larson 1997), but has subsequently been shown to be a major fracture zone (Taylor 2006) comprising areas of deep, flat seafloor exceeding 8000 m depth flanked by large, asymmetrical ridges forming the opposing limbs of the fracture zone. The southern limb of the fracture zone is the steepest and highest relative to the surrounding seafloor, shoaling at around 2160 m depth. Much of the tectonic history is preserved on the seafloor as evidenced by the bathymetric expression of multi-transform fracture zones, spreading fabric and volcanic edifices (Jamieson et al. 2024).

### Data collection

Data for this study were collected during the Nova Canton Trough Expedition conducted aboard RV *Dagon* as part of the Inkfish Open Oceans Programme (Jamieson et al. 2024). The expedition comprised four 3-week long legs conducted between 21 February and 25 May 2024. The sampling sites for Legs 1 and 2 were all located within the faulted bounds of the trough with the shallowest (2751 m) located to the northeast and the deepest (7989 m) within the flat plain between the fracture zone limbs. The sampling sites for Legs 3 and 4 were located both within the fracture zone and across the surrounding region to provide context. All sampling sites are presented in Suppl. material 1. A hull-mounted EM124 multibeam echosounder (MBES, Kongsberg, Norway) was used to acquire a total of 194,761 km^2^ of bathymetric data (see Jamieson et al. 2024 for system details).

**Baited landers**

Four autonomous free-fall lander vehicles, rated to full ocean depth, were deployed during the expedition: Magna, Cranch, Omma and Onyx (MA, CR, OM and ON, respectively) . These landers descended to the seafloor by virtue of a negatively buoyant ballast weight, a single steel cube of 126 kg, that is jettisoned at the end of the seafloor operation. Once the ballast is released, the landers return to the surface through uplift from positively buoyant syntactic foam (TG39/11,500, Trelleborg Applied Technologies). Further details on landers are provided in Jamieson et al. (2024).

MA, CR and OM were equipped with a scientific payload as follows: High-Definition (HD) video camera (IP Multi SeaCam 3105; Deep Sea Power and Light, San Diego, CA) facing a baited frame, illumination is provided by a single diffused LED lamp (LED SeaLite, Deep Sea Power and Light, San Diego, CA) and a second identical camera placed on the bait arm facing outwards from the lander to gain a more detailed images of local fauna. ON was deployed twice and only baited with a small fish trap. The lander cameras collected visual data on the benthic and demersal fauna, as well as on seafloor habitat and substrate. Landers were also outfitted with amphipod traps, results of which are discussed in Wainwright et al. (2026).

Conductivity, temperature and depth (CTD) data were recorded at 16Hz throughout each deployment using an SBE49 FastCAT (Seabird Electronics, Bellevue, WA). All lander systems are powered by three 24 V lead acid batteries (SeaBattery, Deep Sea Power and Light, San Diego, CA).

**Figure 2.**
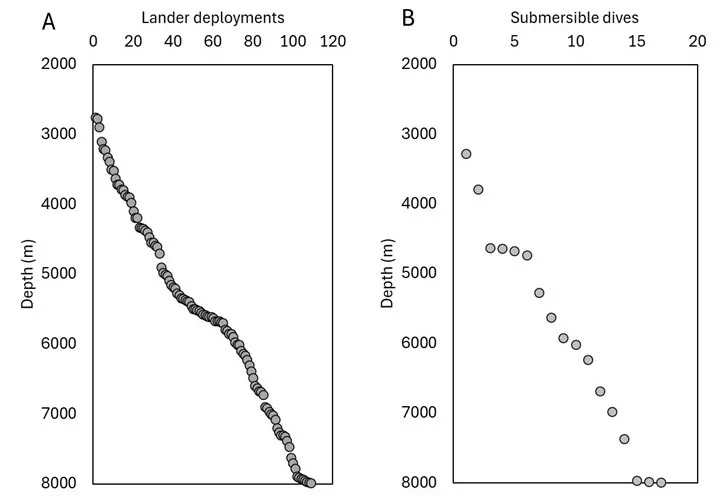
Maximum depth distribution of lander deployments (A) and submersible dives (B), order from shallowest to deepest.

In total, there were 110 scientific lander deployments in the Nova Canton Trough. Of these, 99 were systematically analysed due to camera failures on 11 deployments. Here, cameras recorded no footage or only recorded for a part of the lander's bottom time. Over 1500 hours of seafloor video footage were collected across the main and arm cameras (equating to 58 days of continuous video combined). The mean bottom time was 7.88 ± 1.8 hours. The vertical depth range of lander deployments was 5232 m (2751 to 7983 m water depth). The mean lander depth intervals were 48.4 m ± 44.6 s.d. across this range (Fig. 2a).

**Submersible**

Submersible surveys were conducted using the human-occupied vehicle *Bakunawa*, a 11.4 metric tonne two-person submersible vehicle (Triton 36 00/2; Triton Submarines LLC, US; Jamieson et al. 2019). The titanium sphere, with an internal diameter of 1.5 m and a thickness of 90 mm, featured three acrylic viewports for observations and to take additional images from inside the vehicle *ad hoc* using an iPhone 15. The exterior was equipped with three high-definition video cameras (IP Multi SeaCam 3105, DeepSea Power & Light) used to record footage of the surrounding seafloor. The submersible’s exterior was illuminated by ten 15,500-lumen LED lights (LED-1153-A3-SUS, Teledyne Bowtech). Depth and temperature were recorded through using the same SBE49 FastCAT instruments as the landers.

In total, 16 submersible transects were completed. The bottom time for each dive was ~3.4 ± 0.66 h, resulting in ~ 50.9 on bottom transect hours, producing 152.9 h of HD video data of the seafloor across three cameras used for visual accounts of biodiversity and associated seafloor habitat and substrate. The vertical depth range of the submersible dives was 4727 m (3273 m to 8000 m water depth). The mean submersible dive depth intervals were 295.4 m ± 253.2 s.d. across this range (Fig. 2b). Due to technological limitations of the submersible tracking system, we cannot establish exact distances covered during each dive.

An overview of deployment metadata can be found in Suppl. material 1.

### Biological Observations

An initial morphotaxa reference library was compiled to form a visual identification guide for the abyssal and hadal biota of the Nova Canton Trough and ensure consistent identification across deployments and platforms by multiple annotators. All identifications were derived from review of video footage obtained from baited lander deployments, submersible dives and high-resolution imagery captured from inside the submersible.

Video footage was then reviewed in detail to generate morphotaxon occurrence records using EventMeasure (SeaGIS Pty Ltd, version 6.48) and BIIGLE 2.0 (Langenkämper et al. 2017). Baited-lander footage was annotated in EventMeasure and aligned with the World Register of Marine Species (WoRMS). When the lander was on the seafloor (bottom time), annotations were made using MaxN (also known as N_max_) which is a conservative metric of relative abundance and refers to the maximum number of individuals of each taxon observed at a single point in time (Priede et al. 1994). Initial submersible dive annotations were made in EventMeasure and subsequently imported into BIIGLE to facilitate label checking and standardisation using the Largo tool, which allows annotations assigned to the same label to be viewed in a regular grid, enabling efficient review, re-labelling or deletion. Submersible dive footage was annotated only when the submersible was on bottom.

During annotation, visible organisms were identified to the lowest possible taxonomic rank using morphological characteristics. Where taxonomical identifications were not possible, organisms were classified using a morphotaxa framework in which visually distinct organisms are classified according to consistent morphological characteristics observable in video imagery. These included body shape, size, colouration, movement and other behavioural traits where visible. The level of taxonomic precision achieved was indicated using the open taxonomic nomenclature signs recommended for image-based identifications by Horton et al. (2021). Taxonomic designations were accompanied by suffixes indicating the level of identification (e.g. “fam.”, “gen.”, “sp.”). The designation “indet.” was used when a single observed morphotype could not be reliably identified beyond a given taxonomic level because diagnostic characteristics were unresolvable from the imagery, whereas "inc." was used when characters were visible, but identification remained uncertain. The designation “spp.” was used when multiple visual forms were observed within a given taxonomic rank, but lacked sufficient detail to be consistently separated across all annotated imagery and therefore grouped (e.g. “Mysidae spp.”). To ensure consistency between annotation platforms, a defined annotation vocabulary was applied within the BIIGLE project. Submersible annotations were first reviewed using the SMarTaR-ID label tree (Howell et al. 2019) and then re-labelled with the same taxonomical labels as *per* the WoRMS classification applied in EventMeasure. Thus, the same naming convention was used across both lander and submersible datasets.

The final annotated datasets for the submersible and lander were combined to provide an overview of morphotaxon occurrences across the Nova Canton Trough and the surveyed depth gradient. As the submersible *Bakunawa* did not possess a tracking system, exact horizontal positions along the dive track could not be reconstructed. However, observation depths were assigned directly from the CTD measurements. Raw CTD data were first cleaned using a centred running median to reduce noise, then resampled at 1 Hz. Bottom time was identified automatically from the cleaned CTD profile, based on reduced vertical velocity and the vehicle remaining within a defined depth range for a minimum duration; this was later confirmed by checking the videos. Each biological observation was matched to the CTD measurement corresponding to its annotation timestamp. Depth values, therefore, represent time-matched CTD estimates. Remaining uncertainty is mainly related to timestamp resolution, possible clock offset between video annotations and CTD data and short-term vertical movement of the vehicle.

For the purposes of this biodiversity inventory, presence-absence data were used as an analytical metric to integrate observations from baited landers and submersible transects despite differences in detection probabilities. The full lander and submersible datasets are made available on OBIS and GBIF.

## Results

### Overview

The current visual inventory comprises 325 organismal morphotaxa across 16 phyla, 24 classes, 52 orders and 70 families, spanning abyssal and hadal depths (2751–7989 m). Of these, 62.2% of morphotaxa were observed only during submersible dives (n = 202), 12.3% only during lander deployments (n = 40) and ~ 25.5% across both (n = 83), highlighting the complementary nature of these platforms for biodiversity characterisation. In total, ~ 24,500 individual organisms were annotated on submersible data and ~ 4,500 on lander data.

The most morphologically diverse phyla were Echinodermata (n = 86), Cnidaria (n = 72), Porifera (n = 48), Chordata (n = 45) and Arthropoda (n = 29), of which Cnidaria and Porifera were predominantly represented in submersible imagery, reflecting greater detection of sessile fauna during transects and Chordata and Arthropoda predominantly in lander imagery, reflecting bait-attending behaviour around landers. The majority of observations were in the Kingdom Animalia, although protists were also recorded. Observations in the Kingdom Animalia are discussed first by phylum, followed by observations of protists.

A complete overview of all morphotaxa, their bathymetric ranges, deployment occurrences and platform detection is provided in Table 1. Additional reference imagery and identification notes for each morphotype are provided in Suppl. material 2.

### Phylum Annelida

**Figure 3.**
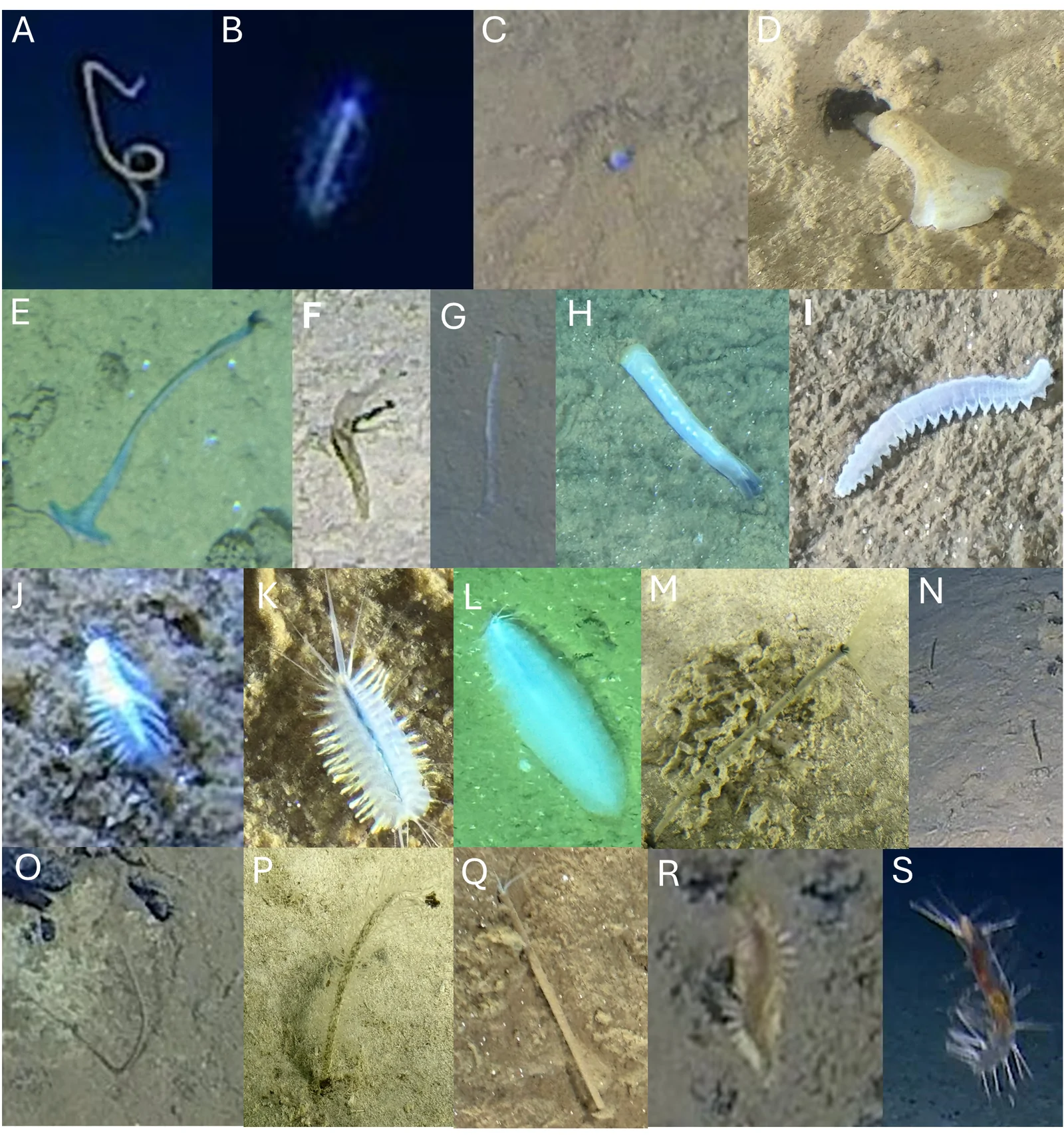
Annelida morphotaxa observed, with approximate depth of shown observations. **A** Annelida spp. 1, 6006 m; **B** Annelida spp. 2, 5294 m; **C** Echiuroidea spp., 6672 m; **D** Echiuroidea fam. indet. 1, 7964 m; **E** Echiuroidea fam. indet. 2, 7985 m; **F** Echiuroidea fam. indet. 3, 4337 m; **G** Echiuroidea fam. indet. 4, 5600 m; **H** Echiuroidea fam. indet. 5, 7369 m; **I** Hesionidae gen. indet. 1, 6672 m; **J** Macellicephalinae spp., 7369 m; **K** Macellicephalinae gen. indet. 1, 7369 m; **L** Admetellinae gen. indet., 6672 m; **M** Sabellida fam. indet. 1, 3791 m; **N-P** Sabellida/Terebellida spp.; **Q** Onuphidae gen. indet., 6672 m; **R** Acrocirridae spp., 6974 m; **S** Flabelligeridae spp., 4675 m.

Annelids (Fig. 3) were amongst the most widely distributed groups observed, occurring across the full depth range of the Nova Canton Trough (Table 1). Due to organism size, most annelids were restricted to higher-level identifications, although several morphotaxa were identified with depth-restricted distributions. A total of 11 morphotaxa and seven species groups were identified across at least five orders (Echiuroidea, Eunicida, Phyllodocida, Sabellida and Terebellida), comprising at least five families. We have replaced observations of the class “Polychaeta” with “Annelida” or “annelids” where appropriate, because “Polychaeta” is a traditional, non-monophyletic grouping rather than a currently supported natural taxonomic unit. As a result, observations are discussed at the order level rather than at the class level, as for other phyla.

At higher taxonomic levels, Annelida spp. 1 (Fig. 3A) and Annelida spp. 2 (Fig. 3B) exhibited the broadest bathymetric ranges (2771–7970 m and 2771–7987 m, respectively) and were most frequently encountered (37 and 67 lander and submersible deployments). Annelida spp. 1 was used for individuals with elongated and cylindrical bodies, with swimming pattern characterised by looping through the water column. Annelida spp. 2 was used when chaetae were visible, but the observation could not be conclusively resolved to one of the known groups (e.g. Polynoidae, Acrocirridae or Flabelligeridae).

#### Order Echiuroidea

Echiuroids or ‘spoon worms’ (Fig. 3C–H) were largely confined to lower abyssal and hadal depths (4337–7987 m). Echiuroidea fam. indet. 1 was highly abundant throughout the deepest part of the trough (5186–7987 m, 10 deployments) and Echiuroidea fam. indet. 3 was seen in the lower abyssal, albeit less frequently (4337–6006 m, 2 deployments). Echiuroidea fam. indet. 2, 4 and 5 were only observed on single submersible dives or lander deployments in narrow depth intervals (7985, 6673 and 7369 m, respectively). The group of Echiuroidea spp. was seen between 5186 m and 7987 m, but was too obscured by sediment burial to identify further.

#### Order Eunicida

Over 200 individuals from the family Onuphidae gen. indet., belonging to the subfamily Hyalinoeciinae and commonly known as ‘quill worms’ (Q), were observed lying horizontally on the sediment surface during a single submersible dive between 6651 m and 6680 m, with palps and prostomium visible protruding from the anterior end of the tube.

#### Order Phyllodocida

Within Phyllodocida, hesionid worms (Hesionidae gen. indet. 1; Fig. 3I) were highly abundant during a single submersible dive (6610–6683 m, ~ 1000 individuals), but were not observed elsewhere.

Polynoid scale worms (Polynoidae, Macellicephalinae; Fig. 3J, K) were most frequently recorded at hadal depths (5667–7987 m, 13 deployments). Some images of sufficient quality were assigned to Macellicephalinae gen. indet. 1, whereas others were too small or distant from the cameras and were assigned to Macellicephalinae spp. Admetellinae (gen. indet.), another subfamily within Polynoidae, was also recorded during a single submersible dive between 6663 m and 6677 m (Fig. 3L).

#### Order Sabellida

Sabellida-like morphotypes (Fig. 3M–P) spanned a broad depth range (3208–7978 m), although most observations were of small individuals that could not be conclusively assigned to a morphotaxon (Fig. 3N–P). One morphotaxa was identified at shallower depths within the dataset (3200–3800 m): Sabellida fam. indet. 1, representing individuals with a visible crown (Fig. 3M), which can be confidently placed within Sabellida; and Sabellida/Terebellida spp., comprising individuals appearing as small, sometimes curved sticks with no visible crown (Fig. 3N–P), which may alternatively belong to the family Melinnidae (order Terebellida).

#### Order Terebellida

The order Terebellida was represented by two morphotypes from two families: Acrocirridae spp. and Flabelligeridae spp. (Fig. 3R–S) observed from 4095–7022 m and 3885–6972 m, respectively, with the latter often seen swimming in the near-bottom water column.

### Phylum Arthropoda

**Figure 4.**
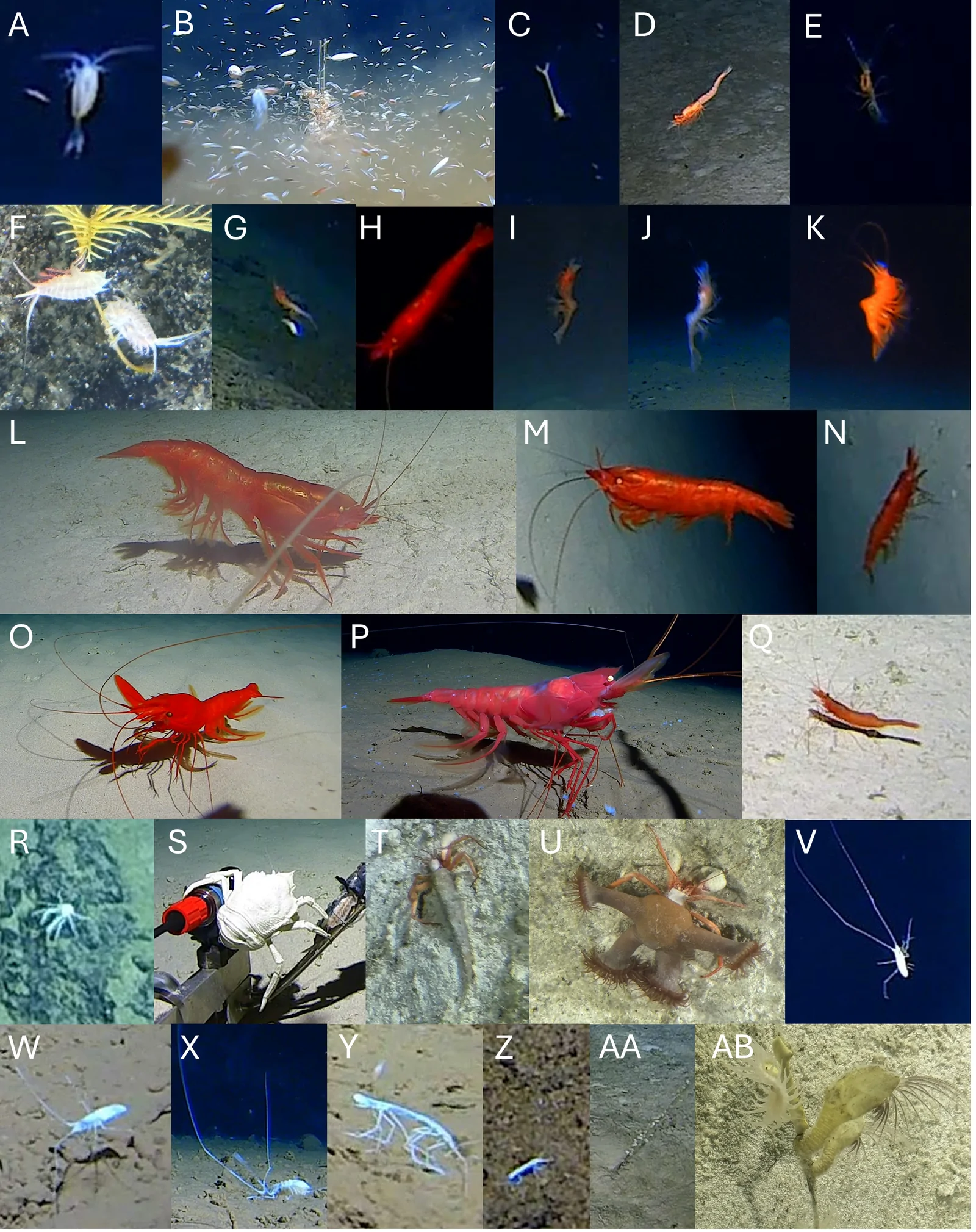
Arthropoda morphotaxa observed, with approximate depth of shown observations. **A** Copepoda spp., 6299 m; **B** Amphipoda spp., 6299 m.; **C** Cumacea spp., 6630 m; **D** Mysida spp., 3273 m; **E** Decapoda spp., 5686 m; **F** *Amathillopsis* sp. indet. 1, 6233 m; **G** Decapoda ord. indet. 1, 5809 m; **H** Acanthephyridae gen. indet. 1, 4368 m; **I** Acanthephyridae gen. indet. 2, 3712 m; **J** Acanthephyridae gen. indet. 3, 5023 m; **K** *Heterogenys* sp. indet. 1, 4469 m; **L** *Hymenodora* sp. indet. 1, 4095 m; **M** *Hymenodora* sp. indet. 2, 2751 m; **N** Benthesicymidae gen. indet. 1, 2751 m; **O** *Cerataspis monstrosus*, 2751 m; **P** *Benthesicymus crenatus*, 4450 m; **Q** *Hymenopenaeus* spp., 3494 m; **R** Munidopsidae spp., 4675 m; **S** *Munidopsis* sp. indet. 1, 3712 m; **T** Paguroidea spp., 3273 m; **U** Parapaguridae spp., 3273 m; **V, W** *Munneurycope* sp. indet., 6223 m and 6480 m; **X** Storthyngurinae gen. indet., 6672 m; **X** Ischnomesidae gen. indet. 1, 7893 m; **Y** Ischnomesidae gen. indet. 2, 6223 m; **AA** Scalpellomorpha spp., 3791 m; **AB** Alcockianum sp. indet. 1, 3791 m.

Arthropods were diverse and widespread, occurring across the full sampled depth range. The arthropoda were represented only by the subphylum Crustacea and a total of 27 morphotaxa within three classes (Copepoda, Malacostraca, Thecostraca). The Malacostraca were further represented by Decapoda in the superorder Eucarida and Amphipoda, Cumacea, Isopoda and Mysida in the superorder Peracarida (Fig. 4).

For several groups, including copepods (3208–7983 m; Fig. 4A), amphipods (3273–7987 m; Fig. 4B), cumaceans (5272–7022 m; Fig. 4C), mysids (2751–7985 m; Fig. 4D), and munnopsid isopods (2751–7798 m; Fig. 4W), their small size and limited diagnostic features in imagery restricted identification to higher taxonomic levels. These taxa were widespread and exhibited broad depth distributions, though multiple species are likely grouped within these ranges. In the case of the Amphipoda spp., large catches were made during the expedition using baited traps that are discussed elsewhere (Wainwright et al. 2026). For the purposes of image-based species identification, they have been taxonomically aggregated to Amphipoda spp. Contrastingly, one readily distinguishable morphotype was *Amathillopsis* sp. indet. 1 (Fig. 4F) that was observed on submersible dives between 5629 and 6228 m clinging to crinoids, xenophyophores and *Hyalostylus* sponges, usually in pairs.

#### Class Malacostraca - Superorder Eucarida

Highest diversity was observed within the order Decapoda (16 morphotaxa, including three spp. groups; Decapoda, Paguroidea and Parapaguridae). Several taxa exhibited broad depth distributions and high encounter frequencies, particularly the Benthesicymid *Benthesicymus crenatus* Spence Bate, 1881 (89 deployments; 3225–7470 m; Fig. 4P) and the Aristeid *Cerataspis monstrosus* Gray, 1828 (74 deployments; 2751–6673 m; Fig. 4O) that have depth ranges of 4245 m and 3922 m, respectively. The high frequency of observations of these two species and other decapod taxa largely reflected bait-mediated attraction to landers, but were also occasionally encountered during submersible transects. A second Benthesicymid (Benthesicymidae gen. indet. 1) was observed by a lander at 2751 m (Fig. 4N). Acanthephyrids (Fig. 4H–M) were also well represented within decapods and characterised by bright red colour with variation in rostrum shape and abdominal curvature. These included three indeterminate morphotaxa; Acanthephyridae gen. indet. 1, 2 and 3; 2751–4368 m, 3712–6094 m and 5023–6094 m, respectively (Fig. 4H–J). More recognisable taxa of Acanthephyridae comprised *Heterogenys* sp. indet. 1 (3100–6226 m; Fig. 4K) and *Hymenodora* sp. indet. 1 and 2 (3712–5186 m and 3387 m, respectively; Fig. 4L, M). Behavioural differences were also evident, including uncoordinated swimming patterns in *H.* sp. indet. 1 and A. gen. indet. 2 and 3.

One indeterminable morphotaxa in the genus *Hymenopenaeus* was observed between 3387 m and 6630 m (Fig. 4Q), as were Galatheoidea or ‘squat lobsters’; Munidopsidae spp. (3100–4734 m; Fig. 4R) and *Munidopsis* sp. indet. 1 (2751–4549 m; Fig. 4S). Hermit crabs of the superfamily Paguroidea and family Parapaguridae were observed at 3238–3238 m and 3100–4675 m, respectively (Fig. 4T, U). Paguroidea spp. were housed within a discarded conical shell, while Parapaguridae spp. were hosting a cluster of zoanthids (see also Fig. 8).

#### Class Malacostraca - Superorder Peracarida

The Isopoda were represented by two families (Munnopsidae and Ischnomesidae) and an indeterminable group between 5584 m and 7704 m. Ischnomesidae gen. indet. 1 were recorded between 7200 m and 7987 m in 13 deployments (Fig. 4Y) often interacting with xenophyophore structures, whilst a similar morph was seen at 7989 m. Ischnomesidae gen. indet. 2 were recorded between 5492 m and 6299 m by landers (Fig. 4Z). The diversity and small-bodied nature of the Munnopsidae is such that these were mostly restricted to the grouping of Munnopsidae spp. However, they were seen in 64 deployments between 2751 m and 7987 m, therefore spanning the entire depth range of 5236 m. A more readily identifiable morph, *Munneurycope* sp. indet 1, was seen between 5667 m and 6739 m 10 times by both lander and submersible and was seen swimming in the near-bottom water column (Fig. 4V, W), whereas munnopsids belonging to the subfamily Storthyngurinae (Fig. 4X) were seen on the seafloor in several lander deployments at similar depths.

#### Class Thecostraca

The class Thecostraca was represented in the order Scalpellomorpha by an indeterminable group at 4598 m and by *Alcockianum* sp. indet. 1 between 3690 m and 4614 m (Fig. 4AA, AB).

### Phylum Chordata

The Chordata were represented by three classes: Appendicularia, Ascidiaceae and Teleostei.

#### Class Appendicularia

**Figure 5.**
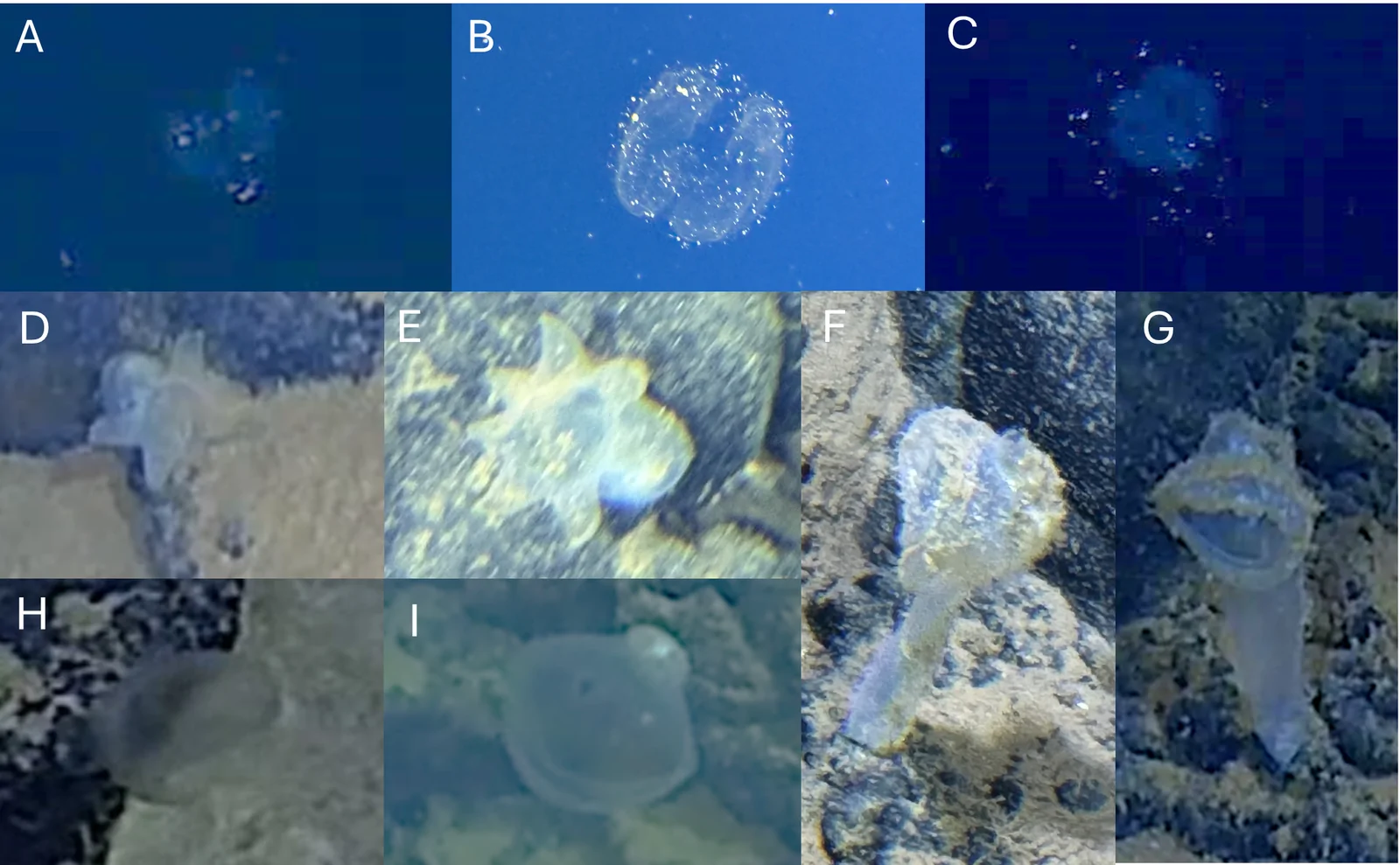
Chordata: Appendicularia & Ascidiacea, with approximate depth of shown observations. **A** Copelata spp., 4980 m; **B** Copelata fam. indet. 1, 4630 m; **C** Copelata fam. indet. 2, 5696 m; **D** Octacnemidae gen. indet. 1, 7161 m; **E** Octacnemidae gen. indet. 2, 7171 m; **FG** *Megalodicopia* sp. indet. 1, 6900 m; **H** *Megalodicopia* sp. indet. 2, 4700 m; **I** *Megalodicopia* sp. indet. 3, 5921 m.

Appendicularia (Larvaceans) were composed of three morphotaxa in the order Copelata: Copelata spp., Copelata fam. indet. 1 and Copelata fam. indet. 2 (Fig. 5A–C). Copelata spp. were too small and often too obscure to resolve a single morphotype, whereas if the housing were ovoid and showing two distinct chambers, an individual was classed as Copelata fam. indet. 1 and if the housing showed multiple chambers, it was classed as Copelata fam. indet. 2., sensu Jamieson et al. (2026). These could perhaps correspond to Oikopleuridae and Fritillariidae, but until higher quality imaging and/or physical samples occur, they are left at order level to avoid perpetuating very tentative identifications. Copelata spp. were observed in 39 deployments between depths of 2771 m and 7318 m, resulting in a large depth range of 4547 m, which may indicate more than one population. The more readily distinguishable Copelata fam. indet. 1 and 2 were observed at depths of 5023–5790 m (9 deployments) and 4980–6592 m (13 deployments), respectively. All groups were observed in both lander and submersible footage drifting in the near bottom currents.

#### Class Ascidiaceae

Five morphotypes of Ascidiacea (subphylum: Tunicata) were observed by submersible only (Fig. 5D–I). These all belonged to the order Phlebobranchia and the family Octacnemidae that comprised Octacnemidae gen. indet. 1 and 2; three times at depths of 5594–7161 m and once at 7172 m, respectively. Octacnemidae gen. indet. 1 and 2 were distinguished by the number of lobes, with five seen in the former and nine in the latter. Three morphotypes belonging to the *Megalodicopia* genus (*Megalodicopia* sp. indet. 1, 2 and 3) were seen respectively at 5580–7025 m (3 deployments), 4011–5287 m (3 deployments) and 5496–6969 m (5 deployments).

#### Class Teleostei

**Figure 6.**
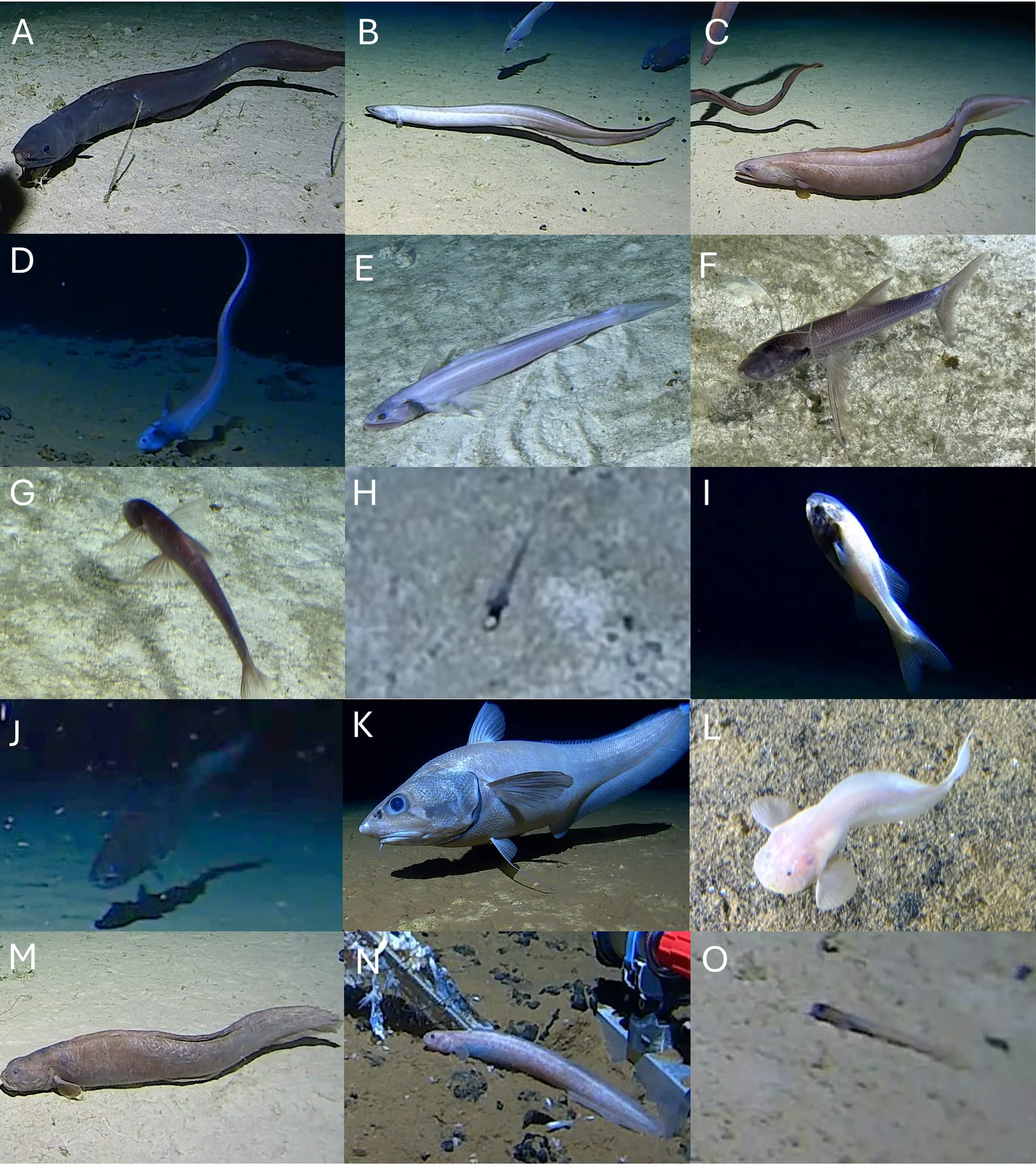
Chordata: Non-ophidiform Teleostei (bony fish), with approximate depth of shown observations. **A** *Ilyophis arx*, 3519 m; **B** *Ilyophis robinsae*, 4587 m; **C** *Ilyophis* sp. indet. 1, 3225 m; **D** *Ateleopus* sp. indet. 1, 5696 m; **E** *Bathysaurus mollis*, 3802 m; **F** *Bathypterois atricolor*, 3219 m; **G** *Bathytyphlops marionae*, 3799 m; **H** *Ipnops* sp. indet. 1, 4559 m; **I** *Abyssoberyx* sp. indet. 1, 5668 m; **J** *Coryphaenoides rudis*, 2771 m; **K** *Coryphaenoides yaquinae*, 5572 m; **L** *Pseudoliparis* sp. indet. 1, 6090 m; **M** *Pachycara* sp. indet. 1, 3791 m; **N** *Pachycara* sp. indet. 2, 5974 m; **O** *Pachycara* spp., 4546 m.

**Figure 7.**
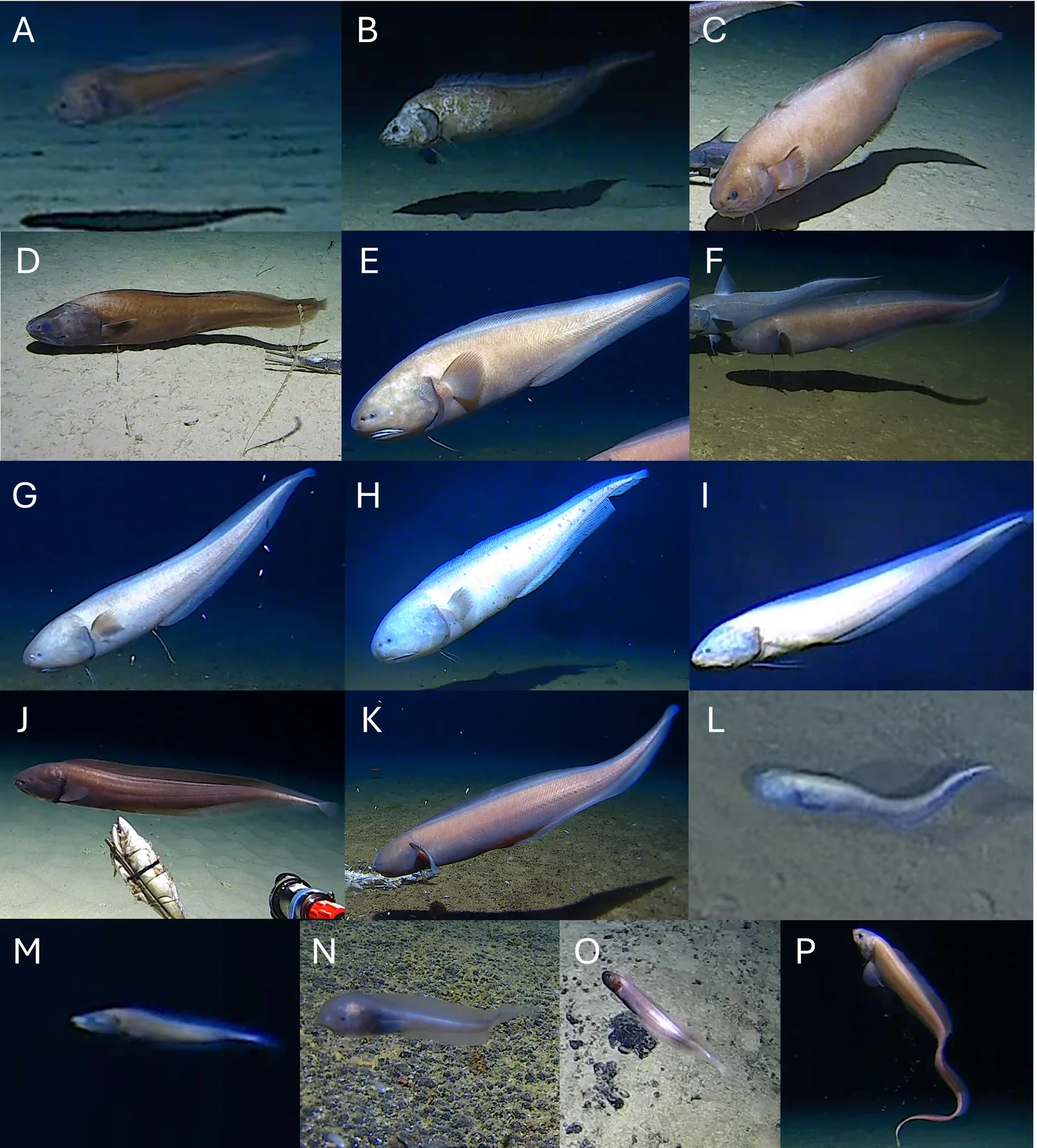
Chordata: Ophidiform-only Teleostei (bony fish), with approximate depth of shown observations. **A** *Acanthonus armatus*, 2771 m; **B**) *Abyssobrotula* sp. indet. 1, 3864 m; **C** *Barathrites iris*, 4469 m; **D** *Bassogigas walkeri*, 3208 m; **E** *Bassozetus* sp. indet. 1, 6223 m; **F** *Bassozetus* sp. indet. 2, 5552 m; **G**) *Bassozetus* sp. indet. 3, 6223 m; **H** *Bassozetus* sp. indet. 4, 5612 m; **I** *Bassozetus* sp. indet. 6, 4897 m; **J** *Bassozetus* sp. indet. 7, 2751 m; **K** *Bassozetus* sp. indet. 8, 6223 m; **L**; *Bassozetus* spp., 5600 m; **M** *Leucicorus* sp. indet. 1, 5809 m; **N** Ophidiidae gen. indet. 1, 5809 m; **O** Ophidiidae gen. indet. 2, 4700 m; **P** *Porogadus* sp. indet. 1, 3791 m.

The Teleostei (bony fish; Figs 6, 7) were represented by seven orders. The most diverse of these were the Ophidiiformes (cusk eels) with 15 species/morphotypes and four groups of ‘spp.’, all in the Ophidiidae family and, as such, were the most frequently encountered order (total depth range 2751–6480 m). The Ophidiidae family contained seven known genera and three unknown genera, with *Bassozetus* being the most diverse genus with seven morphotypes (Fig. 7E–L). The genus *Bassozetus* was generally found deep with a mean maximum depth of 6029 m ± 362 s.d. *Bassozetus* sp. indet. 1 (2751–6480 m) had the largest depth range of any species/morphotype in the survey; 3729 m. All *Bassozetus* observations were made by lander and 50% of them were also observed by submersible.

Three known ophidiid species were observed; *Acanthonus armatus* Günther, 1878 or ‘bony-eared assfish’ (2751–2771 m), *Barathrites iris* Zugmayer, 1911 or ‘iris cuskeel’ (4469–5974 m) and *Bassogigas walkeri* Nielsen & Møller, 2011 or ‘Walker’s cusk eel’ (2751–3494 m; ACD). Other indeterminate species were also observed; *Abyssobrotula* sp. indet 1. (3780–3892 m; Fig. 7B), *Leucicorus* sp. indet. 1 (4704–5921 m; Fig. 7M) and *Porogadus* sp. indet. 1 (3791–5609 m; Fig. 7P), while other less identifiable groups comprised Ophidiidae spp. (4635–6014 m), *Leucicorus* spp. (4126–6008 m), *Porogadus* spp. (4345–5079 m) and two morphotypes of Ophidiidae gen. indet. (3780–5854 m, and 4675 m, respectively; Fig. 7N, O).

The second most diverse groups were the Anguilliformes (Synaphobranchidae ‘cut-throat eels’ family; 2751–5612 m) and Aulopiformes (Bathysauridae ‘lizard fish’ and Ipnopidae ‘deep-sea tripod fish’ families) with three species/morphotypes and one spp. and four species/morphotypes, respectively. These two families were observed at 3803–4555 m and 3319–4560 m, respectively and were all from submersible deployments. The synaphbranchids were represented by *Ilyophis arx* Robins, 1976 (2751–4095 m; Fig. 6A), *I. robinsae* Sulak & Shcherbachev, 1997 (3885–5612 m; Fig. 6B), the morphotype *Ilyophis* sp. indet. 1 (2751–3519 m; Fig. 6C) and an unidentifiable synaphobranchid at 3273 m.

The known species of Aulopiformes comprised the bathysaurid *Bathysaurus mollis* Günther, 1878 or ‘highfin lizardfish’ (3791–4675 m; Fig. 6E) and the ipnopids *Bathypterois atricolor* Alcock, 1896 or ‘attenuated spiderfish’ (3273 m; Fig. 6F) and *Bathytyphlops marionae* Mead, 1958 or ‘Marion's spiderfish’ (3791 m; Fig. 6G). *Ipnops* sp. indet. 1 was observed between 3877 m and 4560 m and, while detailed images were not obtained, a white or reflective snout distinguished it from the other ipnopids (Fig. 6H).

The Gadiformes were represented by the macrouridae family with two species *Coryphaenoides* (*C. rudis* Günther, 1878 and *C. yaquinae* Iwamoto & Stein, 1974) and one spp. *Coryphaenoides rudis* or ‘Rudi’s rattail’ was the shallower of the two species, seen only at 2771 m (Fig. 6J), while *C. yaquinae* was the most frequently encountered species in the survey, having been sighted 65 times between 3387 m and 6673 m (depth range = 3286 m; Figure 6K). The Ateleopodiformes or ‘jellynose fish’ and Beryciformes were both represented by single morphotypes in the Ateleopodidae (*Ateleopus* sp. indet. 1; 4734–5696 m; Fig. 6D) and Stephanoberycidae or ‘pricklefish’ (*Abyssoberyx* sp. indet. 1; 4635–5921 m; Fig. 6I) families, respectively.

The Perciformes were represented by the Liparidae (snailfish) and Zoarcidae (eel pout) families by one morphotype and two morphotypes and an spp., respectively. The liparid *Pseudoliparis* sp. indet. 1 (6090–7985 m; Fig. 6L) was the deepest fish in the survey, the only one to be found exclusively at hadal depths and detected from both landers and submersible. Two indeterminate species of *Pachycara* (*Pachycara* sp. indet. 1 and 2) were recorded by lander from 3519–3791 m and 5383–5974 m, respectively, as well as the addition of the *Pachycara* spp. group (4546 m; Fig. 6O).

### Phylum Cnidaria

**Figure 8.**
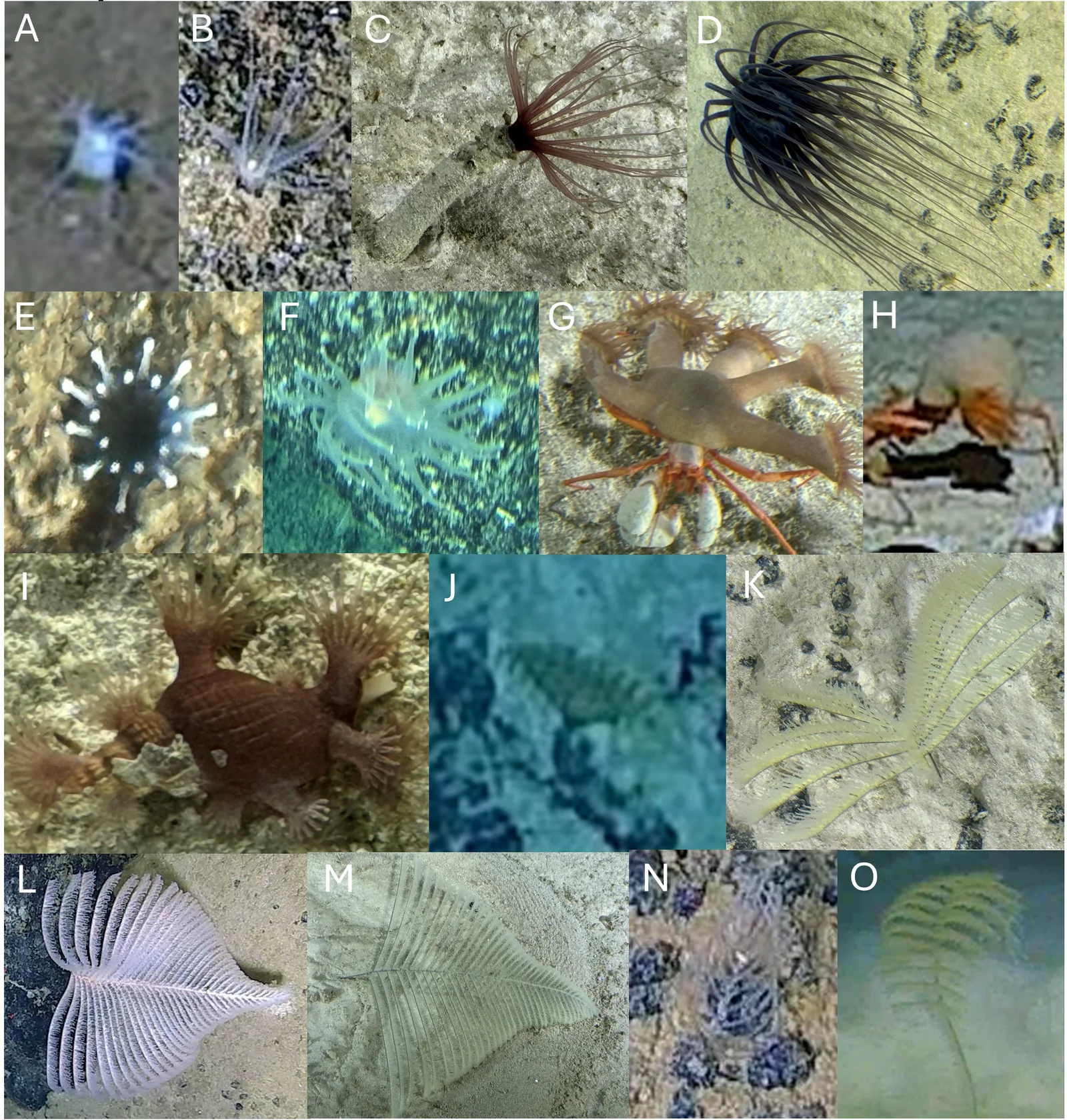
Cnidaria: Anthozoa, Hexacorallia: Ceriantharia, Corallimorpharia, Zoantharia Antipatharia, with approximate depth of shown observations. **A** Anthozoa ord. indet. 1, 7989 m; **B** Anthozoa ord. indet. 2, 6233 m; **C** Ceriantharia fam. indet. 1, 3219 m; **D** Ceriantharia fam. indet. 2, 4665 m; **E** Corallimorphidae fam. indet. 1, 6480 m; **F** Corallimorphidae fam. indet. 2, 4335 m; **G** *Epizoanthus* sp. indet. 1 (on the back of Parapaguridae spp.), 3272 m; **H** *Epizoanthus* sp. indet. 2 (on the back of Parapaguridae spp.), 4546 m; **I** Zoantharia gen. indet. 1 (on the shell of Neogastropoda), 3272 m; **J** Antipatharia fam. indet. 1, 3888 m; **K** Schizopathidae gen. indet. 1, 4633 m; **L** Schizopathidae gen. indet. 2, 4700 m; **M** Schizopathidae gen. indet. 3, 4635 m; **N** *Abyssopathes* sp. indet. 1, 6017 m; **O** *Bathypathes* sp. indet. 1, 4533 m.

**Figure 9.**
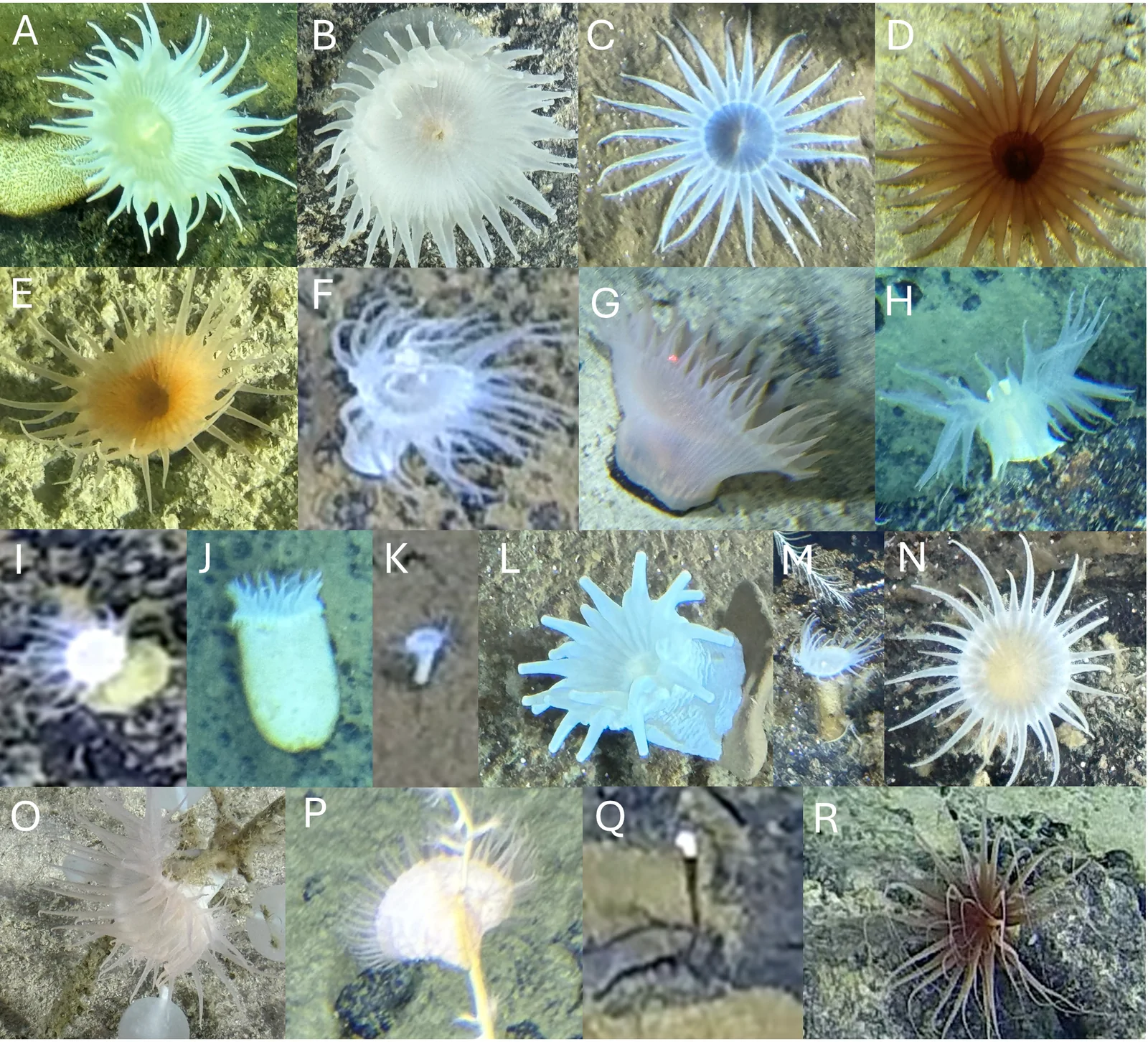
Cnidaria: Anthozoa, Hexacorallia: Actiniaria, with approximate depth of shown observations. **A** Actiniaria fam. indet. 1, 7400 m; **B** Actiniaria fam. indet. 2, 4585 m; **C** Actiniaria fam. indet. 3, 6969 m; **D** Actiniaria fam. indet. 4, 3240 m; **E** Actiniaria fam. indet. 5, 3219 m; **F** Actiniaria fam. indet. 6, 6017 m; **G** Actiniaria fam. indet. 7, 4700 m; **H** Actiniaria fam. indet. 8, 4700 m; **I** Actiniaria fam. indet. 9, 6227 m; **J** Rolling morphotype, Actiniaria fam. indet. 10, 6000 m; **K** Actiniaria fam. indet. 11, 7369 m; **L** Actiniaria fam. indet. 12, 7369 m; **M** Actiniaria fam. indet. 13, 6223 m; **N** Actinoscyphidae gen. indet. 1 (on *Chondrocladia*), 4603 m; **O** Actinoscyphidae gen. indet. 2 (on stalk of octocoral), 4529 m; **P** *Galatheanthemum* sp. indet. 1, 7985 m; **Q** *Relicanthus* sp. indet. 1, 4634 m.

**Figure 10.**
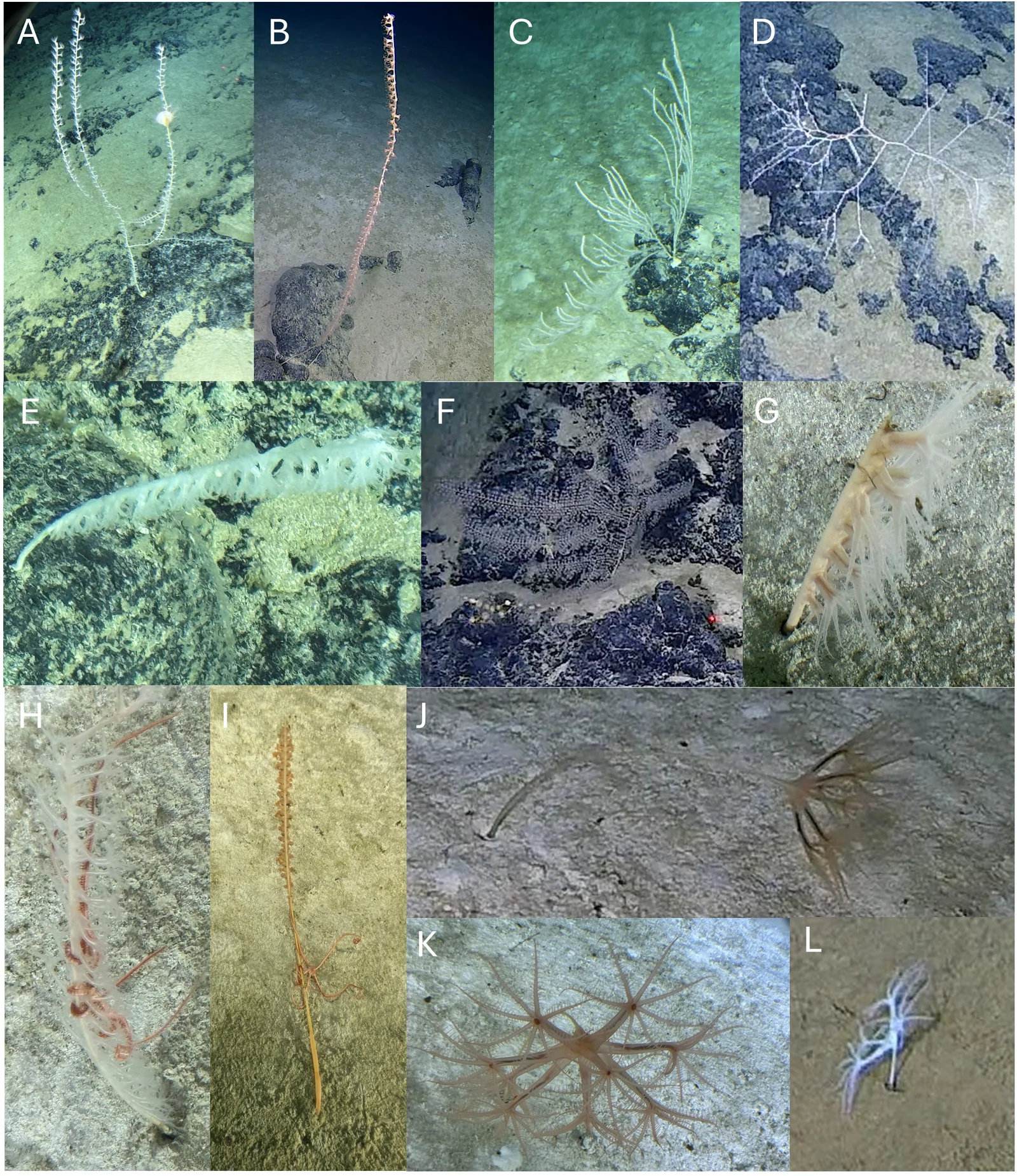
Cnidaria (Cont.) – Octocorallia, with approximate depth of the shown observations. **A** Octocorallia ord. indet. 1, 4675 m; **B** Octocorallia ord. indet. 2, 4734 m; **C** Octocorallia ord. indet. 3, 4635 m; **D** Octocorallia ord. indet. 4, 4619 m; **E** Octocorallia ord. indet. 5; **F** *Chrysogorgia* sp. indet. 1, 4359 m; **G** *Kophobelemnon* sp. indet. 1, 3799 m; **H** *Calibelemnon* sp. indet. 1, 3172 m; **I** *Scleroptilum* sp. indet. 1, 3238 m; **JK** *Umbellula* sp. indet. 1, 3273 m; **L** *Umbellula* sp. indet. 2, 6949 m.

**Figure 11.**
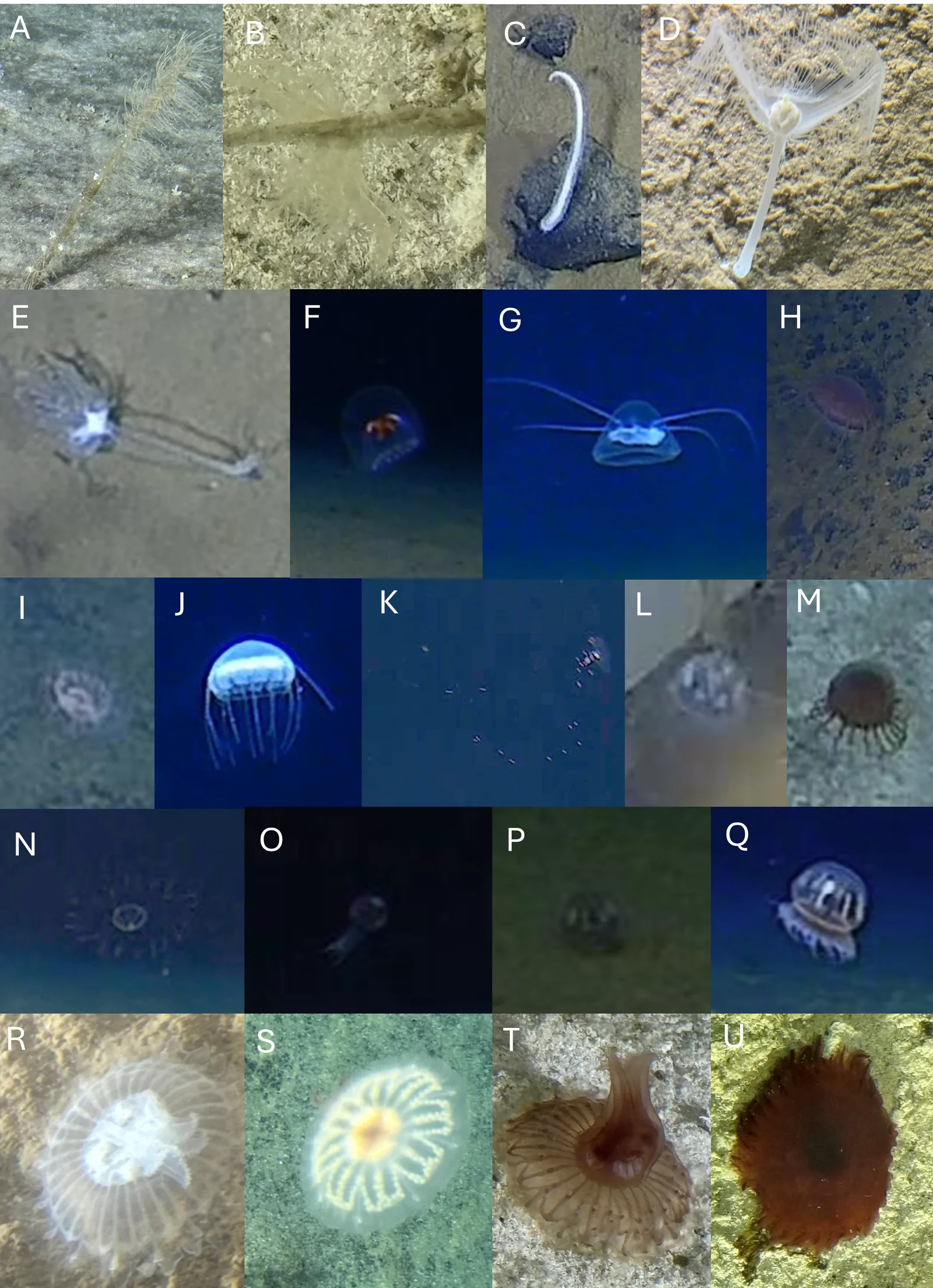
Cnidaria (cont.) Hydrozoa and Scyphozoa, with approximate depth of the shown observations. **A** Hydrozoa ord. indet. 1, 4630 m; **B** Hydrozoa ord. indet. 2, 3791 m; **C** Candelabridae gen. indet. 1, 6875 m; **D** *Branchiocerianthus* sp. indet. 1, 7670 m; **E** *Branchiocerianthus* sp. indet. 2, 6938 m; **F** Tiarannidae gen. indet. 1, 5492 m; **G** Aeginidae gen. indet. 1, 6911 m; **H** Aeginidae gen. indet. 2, 6012 m; **I** Cuninidae spp., 6226 m; **J** Cuninidae gen. indet. 1, 5790 m; **K** Siphonophorae fam. indet. 1, 3100 m; **L** Trachymedusae spp., 5272 m; **M** Trachymedusae gen. indet. 1, 4700 m; **N** Rhopalonematidae spp., 3519 m; **O** Arctapodema sp. indet. 1, 3791 m; **P** Rhopalonematidae gen. indet. 1, 6006 m; **Q** Rhopalonematidae gen. indet. 2, 5572 m; **R** Ulmaridae gen. indet. 1, 7987 m; **S** Ulmaridae gen. indet. 2, 6223 m; **T** Ulmaridae gen. indet. 3, 3237 m; **U** Ulmaridae gen. indet. 4, 3240 m.

Cnidaria were represented by the classes Hexacorallia, Octocorallia, Hydrozoa and Scyphozoa (Figs 8, 9, 10, 11).

Two indeterminate morphotaxa placed in the subphylum Anthozoa (Anthozoa ord. indet. 1 and 2, Fig. 8A, B) were frequently observed at lower abyssal and hadal depths. These appeared to be sediment dwelling. Anthozoa ord. indet. 1 was found between 7939 m and 7987 m embedded in soft sediment, whereas Anthozoa ord. indet. 2 ranged from 5653 m to 7987 m and was found embedded in more compacted substrate.

#### Class Hexacorallia

Hexacorallia were represented by organisms spanning the orders Antipatharia, Ceriantharia, Corallimorpharia, Zoantharia (Fig. 8) and Actiniaria (Fig. 9).

Ceriantharia were represented by at least two morphotypes (Fig. 8C, D), characterised by tentacles extending from a soft sediment tube. Ceriantharia fam. indet. 2 appeared very similar to *Relicanthus* sp. indet. 1 (Fig. 9Q) and was initially identified as such. However, in the specimens we observed, the tentacles are shorter, are not deciduous, maintain a constant taper and extend from the sides of a visible mouth; from it, short labial tentacles can be distinguished from the marginal tentacles supporting an identification as ceriantharia.

Corallimorpharians (Corallimorpharia) were infrequently observed, but included at least two morphotypes and were identified by the presence of the characteristic acrospheres. The first morphotype was partially embedded in sediment between 6480 m and 6669 m; it showed a dark, disc-like body and two whirls of five tentacles each, terminating in bright white acrospheres, suggesting its belonging to Corallimorpharia (Fig. 8E). Other possible identifications were considered and identifications as Actiniaria and Zoantharia were rejected due to the difference in tentacle morphology, lacking what we identified as acrospheres. The depth of the observation, well below the aragonite saturation horizon and carbonate compensation depth, discourage the identification as a scleractinian (Guinotte et al. 2006, Bostock et al. 2015). The second morphotype was translucent with white, reflective acrospheres at the end of the tentacles and found attached to rocks at abyssal depths (Fig. 8F). Of the four genera in the family (*Corallimorphus*, *Corynactis*, *Paracorynactis* and *Pseudocorynactis*), our specimens closely resemble the genus *Corallimorphus*, already known to be present in the abyssal zone of the Pacific Ocean (Fautin et al. 2002).

Zoantharians were represented by taxa associated with other organisms. *Epizoanthus* sp. indet. 1 and 2 (Fig. 8G, H) were observed on the shells of parapagurid hermit crabs between 3100 m and 4546 m depth, forming symbiotic associations with their hosts. An additional zoantharian morphotype (Fig. 8I) was observed attached directly to the shell of a neogastropod.

Black corals (Antipatharia) were primarily concentrated at lower abyssal depths and associated with rocky or hard substrates. Multiple harp-to-feather-shaped morphotypes were documented and tentatively assigned to the families Cladopathidae and Schizopathidae. Schizopathidae morphotypes were observed between 4031 m and 5293 m (Fig. 8K-M). Notably, Schizopathidae gen. indet. 3 (Fig. 8M) was observed attached directly to soft sediment rather than hard substrate. *Bathypathes* sp. indet. 1 (Fig. 8O) possessed a feather-like colony structure extending vertically in the water column. *Abyssopathes* sp. indet. 1 (Fig. 8N) represented the deepest antipatharian observed at 6018 m. This colony displayed a tightly branching funnel-like morphology and was found on nodule habitat.

Actiniarians (Fig. 9) represented the most morphologically diverse hexacorallian group, comprising at least 17 morphotypes spanning a range of body forms and colourations, in addition to indeterminate observations. Morphotypes were determined, based on tentacle number, width, length and arrangement, column morphology and column to disc ratio. Actiniarians were observed across the full depth range, though diversity appeared to vary across depth zones. Several taxa also appeared strongly associated with particular substrate types.

Actiniarians displayed depth zonation. Several actiniarian morphotypes appeared restricted to abyssal depths (< 6000 m). Actiniaria fam. indet. 4 and 5 (Fig. 9D, E) were both only recorded at 3219–3240 m depth and unique in their orange-red colouration and low abundance, while Actiniaria fam. indet. 2 and 7 (Fig. 9B, G) occurred between 4013 m and 5272 m and Actiniaria fam. indet. 8 (Fig. 9H) extended from 3818 m to 5402 m. A second group of morphotypes occurred on nodules around the abyssal hadal transition zone, including Actiniaria fam. indet. 6, 9 and 10 (Fig. 9F, I, J). Fam. 6 and 9 d,isplayed similar oral structures, but differed in their mode of attachment, whereas fam. 10 exhibited a distinctive rolling behaviour (Marchiò et al. 2025a); additionally, few individuals of fam. 10 with partially inflated bodies were observed in close proximity to fam. 9, suggesting that the two may represent motile and attached forms of the same species.

Several actiniarians morphotypes appeared predominantly hadal in distribution, including Actiniaria fam. indet. 1, 3, 11, 12 and 13 (Fig. 9A, C, K, L, M). These hadal morphotypes were generally smaller and bright white in colour and more abundant than their abyssal counterparts, with ~ 350 individuals of Actiniaria fam. indet. 13 observed. Actiniaria fam. indet. 9, 11 and 13 also strongly resemble sea anemones reported in Lemche et al. (1976) from 6758–6776 m in the South New Hebrides Trench, tentatively identified as “actiniid-like”. An organism resembling Actiniaria fam. indet. 3 and 4 was described in Lemche et al. (1976) from the same station, but identified as a brisingid asteroid. However, the number and thickness of the arms, together with the absence of spines, exclude its assignment to the order Brisingida. Instead, the clear separation between the appendages and the main body, as well as the presence of a visible mouth on the upper side of the animal, support the identification of both our morphotypes and the organism observed by Lemche et al. (1976) as sea anemones, likely with the column buried within the sediment.

Two actinoscyphidae morphotypes were observed attached to biological structures (carnivorous sponges and octocorals), rather than to the seafloor (Fig. 9N, M). Ca. 100 individuals of a probable *Galatheanthemum* morphotype were also observed (Fig. 9P), characterised by a cylindrical body and elevated oral disc with relatively short tentacles.

#### Class Octocorallia

Octocorals (Octocorallia) (Fig. 10) were observed primarily throughout the abyssal depth range of the Nova Canton Trough between 3100 m and 5981 m, with a single *Umbellula* morphotype extending into the upper hadal zone at 6949 m depth. Different colony morphologies were documented including branching, whip-like and sea-pen forms and specific morphologies appeared associated with specific habitat types.

Five indeterminate octocoral morphotypes were recognised (Fig. 10A–E), most occurring within a relatively narrow depth range between 4026 m and 5981 m. These included whip-like colonies with or without lateral branches (Fig. 10A, B), fan-shaped branching forms (Fig. 10C, D) and a form characterised by polyps alternately arranged laterally along the stalk (Fig. 10E). Along with a probable *Chrysogorgia* morphotype (Fig. 10F), these morphotypes occurred primarily on hard substrates and rocky outcrops.

Pennatulacean octocorals (‘sea pens’) were primarily restricted to abyssal depths between 3174 m and 4633 m and commonly associated with soft sediment habitat. Colonies tentatively assigned to *Kophobelemnon* sp. indet. 1 (Fig. 10G) and *Calibelemnon* sp. indet. 1 (Fig. 10H) were characterised by polyp-bearing structures extending from a central rachis, with ~ 350 individual *Calibelemnon* sp. indet. 1 observed during a single dive transect. A whip-like sea pen tentatively identified as *Scleroptilum* sp. indet. 1 was also identified in these habitats (Fig. 10I).

Umbellulid sea pens were amongst the more distinctive octocorals observed. *Umbellula* sp. indet. 1 (Fig. 10J, K) occurred across abyssal depths between 3100 m and 4337 m and was characterised by a long stalk terminating in a crown-like arrangement of polyps. *Umbellula* sp. indet. 2 (Fig. 10L) was recorded exclusively at 6949 m and displayed a more compact crown morphology with fewer, thicker branches and one of the few octocoral morphotypes extending into hadal depths.

#### Class Hydrozoa

Hydrozoans (Fig. 11) formed a diverse component of the benthopelagic cnidarian fauna occurring across nearly the full surveyed depth range between 3222 m and 7925 m depth. The hydrozoa were represented by the orders Anthoathecata, Leptothecata, Nardomedusae, Siphonophorae and Trachymedusae.

Hydrozoa ord. indet. 1 (Fig. 11A) and 2 (Fig. 11B) were indeterminate hydrozoans found attached to other organisms at ~ 3800 m depth.

Within the Anthoathecata, hadal hydroids belonging to the Candelabridae and Corymorphidae families were observed between 6241 m and 6976 m and 6792 m and 7620 m, respectively (Fig. 11C–E). Candelabridae gen. indet. 1 featured a short pedal attachment zone which sustains the elongated white main body covered in capitate tentacles, characterised by a thin shaft and enlarged tips. Of the three genera currently accepted within Candelabridae (*Candelabrum*, *Monocoryne* and *Fabulosus*), the morphology observed here appears more consistent with known *Candelabrum* observed in deep environments (Prudkovsky et al. 2023), although identification from imagery alone remains uncertain. In the Corymorphidae family, *Branchiocerianthus* sp. indet. 1 and 2 included upright stalked polyps with feeding structures extending into the water column, but these differed in stalk colouration.

Narcomedusae formed one of the dominant hydrozoan groups observed and displayed broad bathymetric distribution (3892–7966 m). Representatives of the Aeginidae family (Fig. 11G, H) were most prevalent and occurred at lower abyssal and hadal depths, with Aeginidae gen. indet. 1 observed across 17 deployments between 5686 m and 7987 m. Other Narcomedusae included members of the Cuininidae family (Fig. 11I, J) with one indeterminate group and one morphotype recognised that featured multiple tentacles extending from above the bell margin.

Trachymedusae were also widespread and occurred between 4980 m and 7987 m depth (Fig. 11L–Q). Trachymedusae fam. indet. 1 (Fig. 11M) had a distinctive red bell with short trailing appendages and was only observed once at 4700 m. Rhopalonematids (3387–6672 m) formed the dominant trachymedusan family and included several morphotypes with semi-translucent red bells with bright radial canals. A probable *Arctapodema* morphotype (Fig. 11O) was observed between 3791 m and 5667 m, whilst the other rhopalonemids extended into hadal depths.

Within the order Leptothecata, a probable member of the Tiarannidae family was recorded at 5492 m depth (Fig. 11F); and within the order Siphonophorae a single observation of Siphonophorae fam. indet. was recorded at 3100 m (Fig. 11K).

#### Class Scyphozoa

Scyphozoans were represented by four morphotypes all in the Ulmaridae family (Fig. 11R–U) which occurred across the full depth range surveyed (2751–7987 m). These medusae possessed broad gelatinous bell structures with relatively short marginal tentacles and occurred primarily on or close to the seafloor, frequently with tentacles and oral appendage extending upwards. The different morphotypes varied in umbrella shape, radial canal structure and pigmentation and displayed broad depth zonation. Ulmaridae gen. indet. 1 was the most abundant of these morphotypes (> 300 individuals) and found in the deeper parts of the Nova Canton Trough (5627–7987 m, Fig. 11R), whereas Ulmaridae gen. indet. 2 was found at a similar depth range, but in the water column (Fig. 11S, 5340–7005 m), Ulmaridae gen. indet. 4 at upper abyssal depths (3220–4518 m, Fig. 11U) and Ulmaridae gen. indet. 3 at lower bathyal to upper abyssal depth (2751–3237 m, Fig. 11T) and three or fewer individuals of these morphotypes were observed.

### Phylum Ctenophora

**Figure 12.**
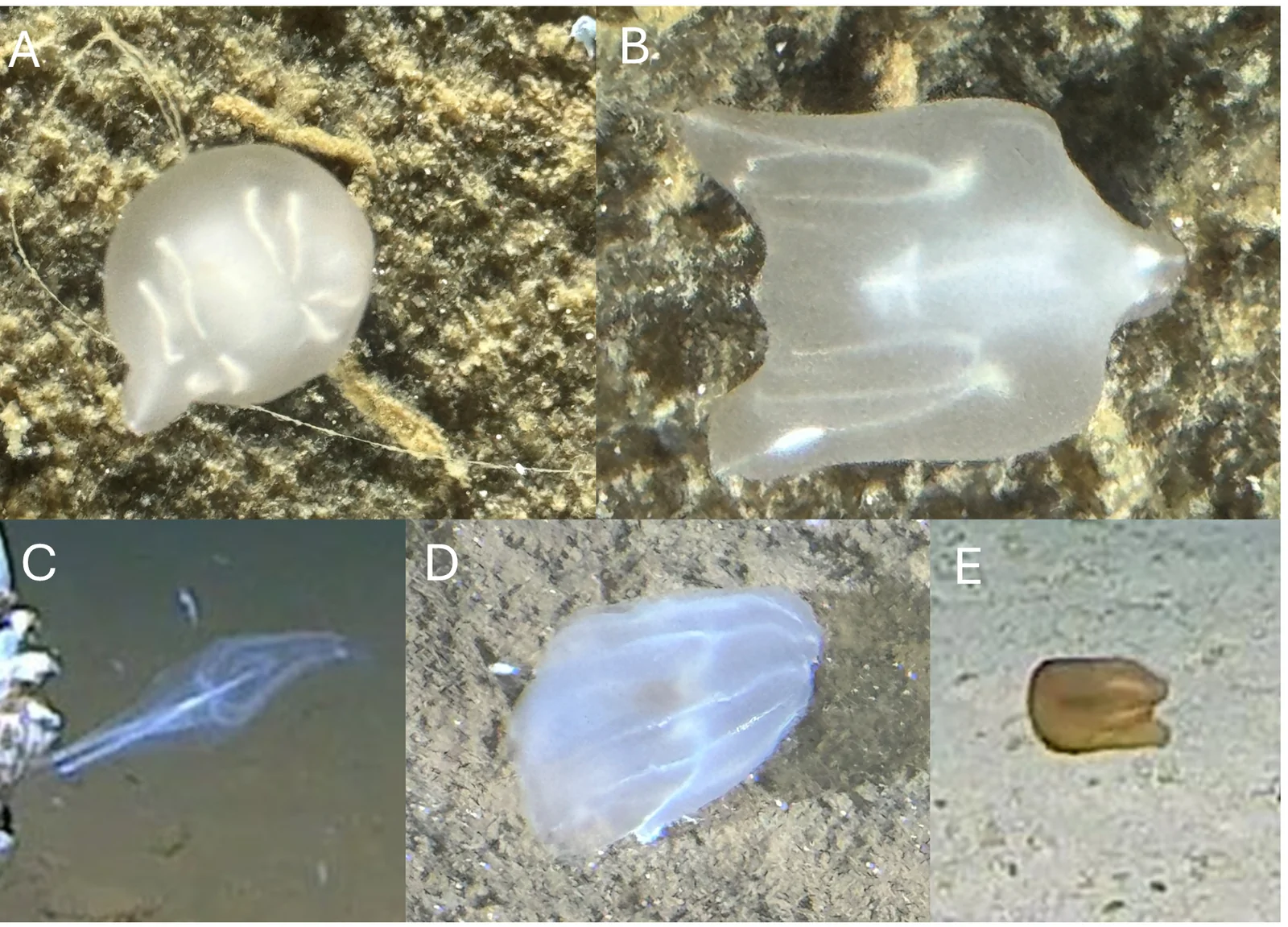
Ctenophora, with approximate depth of the shown observations. **A** Cydippida fam. indet. 1, 7483 m; **B** Cydippida fam. indet. 2, 6974 m; **C** Cydippida fam. indet. 3, 7022 m; **D** Cydippida fam. indet. 4, 6592 m; **E** Cydippida fam. indet. 5, 4587 m.

The Ctenophora (Tentaculata) (Fig. 12) were represented by five distinct morphotypes and an indeterminable group - Cydippida spp. – that were recorded across the entire depth range (2771–7989 m), but were too small to confidently delineate from others. All the five morphotypes belonged to the Cydippida order, (Cydippida fam. indet. 1–5; Fig. 12A–E). Interestingly, morphotypes Cydippida fam. indet. 1–4 were all found exclusively at hadal depths only with ranges of 6665–7484 m, 6592–6975 m, 6658–7022 m and 6223–7135 m, respectively. Therefore, Cydippida fam. indet. 1, 2, 3 and 4 had depth ranges of 819, 383, 364 and 912 m, respectively. Conversely, Cydippida fam. indet. 5 was seen in two lander deployments at 2771 m and 4587 m.

### Phylum Echinodermata

The Echinodermata were represented by five classes: the Echinoiddea, Asteroidea, Holothuroidea, Crinoidea and Ophiuroidea.

#### Class Echinoidea

**Figure 13.**
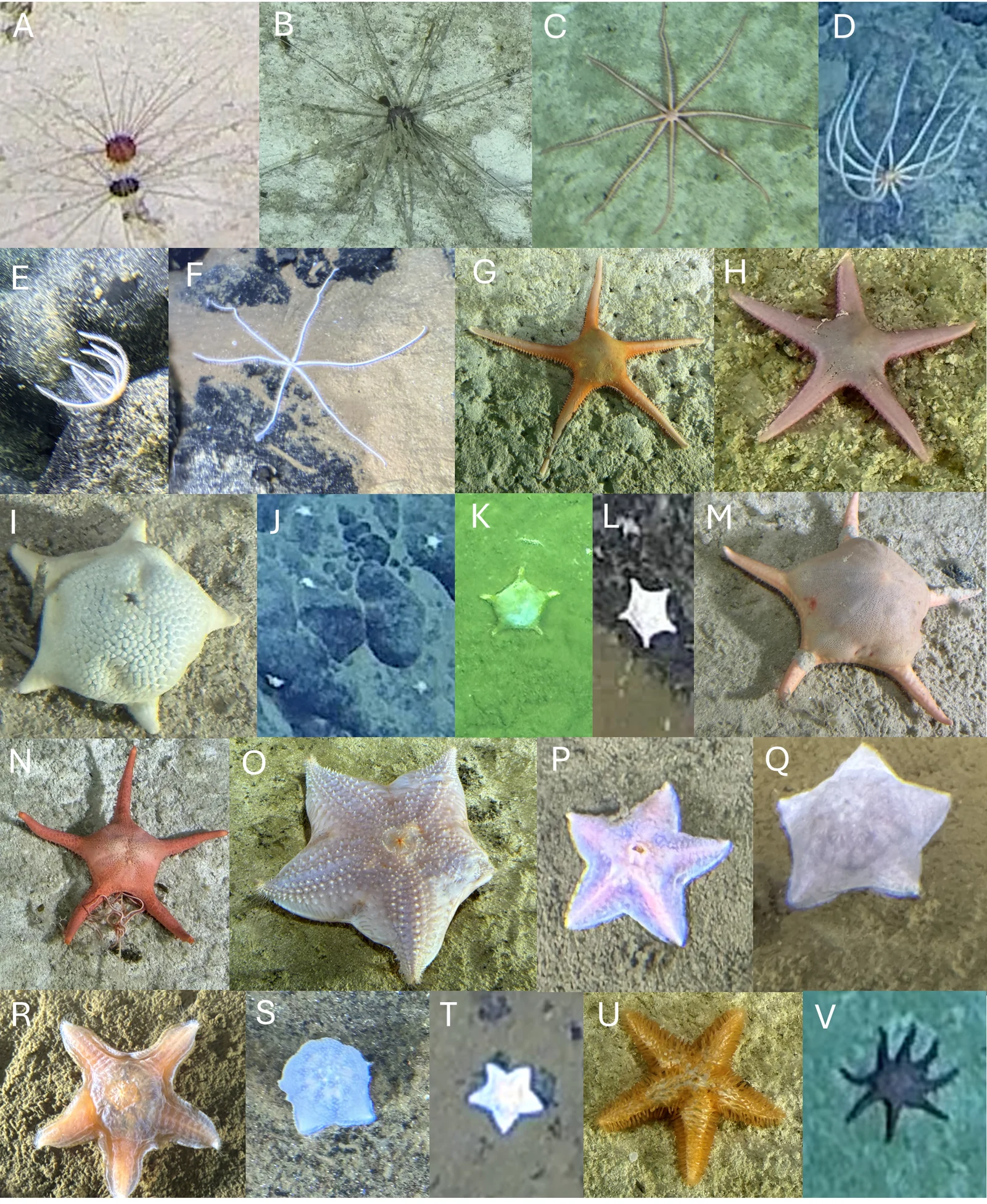
Echinoidea and Asteroidea, with approximate depth of the shown observations. **A** Aspidodiadematidae gen. indet. 1, 4337 m; **B** *Histocidaris* sp. indet. 1, 3804 m; **C** Brisingida fam. indet. 1, 4635 m; **D** Brisingida fam. indet. 2, 4635 m; **E** Brisingida fam. indet. 3, 5935 m; **F** Freyellidae gen. indet. 1, 6001 m; **G** Porcellanasteridae spp., 4700 m; **HI** Porcellanasteridae gen. indet. 1, 6700 m; **J** Astropectinidae gen. indet. 1, 3237 m; **K** Astropectinidae gen. indet. 2, 3740 m; **L** Goniasteridae gen. indet. 1, 4633 m; **M** Goniasteridae gen. indet. 2, 4635 m; **N** *Circeaster* sp. indet. 1, 3237 m; **O** *Lophaster* sp. indet. 1, 3237 m; **P** Solasteridae gen. indet. 1, 4051 m; **Q** *Hymenaster* sp. indet. 1, 5272 m; **R, S, T** *Hymenaster* sp. indet. 2, 7987 m, where S and T show inflation and deflation; **U, V** *Hymenaster* sp. indet. 3, 6000 m, inflated (U) and deflated (V).

Echinoids were comparatively uncommon and were observed primarily at abyssal depths between 3885 m and 5609 m on soft-sediment habitats from both lander and submersible-based observations (Fig. 13A, B). In addition to several indeterminate echinoid observations, two morphotypes were distinguished and tentatively assigned to the Aspidodiadematidae family (3894–4507 m, Fig. 13A) and the *Histocidaris* genus (3691–3804 m, Fig. 13B). These taxa possessed elongate spines, which were flexible in Aspidodiadematoidea gen. indet. 1 and rigid in *Histocidaris* sp. indet. 1.

#### Class Asteroidea

The Asteroidea spanned abyssal to hadal depths between 3236 m and 7986 m (Fig. 13C–V). Orders represented included the Brisingida, Paxillosida, Valvatida and Velatida.

Brisingid sea stars formed a conspicuous component of the abyssal fauna between ~ 3984 m and 6001 m. These taxa were characterised by elongate slender arms radiating from a comparatively small central disc and were either attached to hard substrate or found on sediment. Three indeterminate brisingid morphotypes and one freyellid morphotype were recognised. The freyellid morphotype was distinguished by the presence of exactly six arms, a characteristic primarily diagnostic for the genus *Freyastera*, but also reported for few *Freyella* species (Zhang et al. 2024).

Paxillosids were represented by members of the Porcellanasteridae and Astropectinidae (Fig. 13G–K). Porcellanasterids (Fig. 13G–I) occurred between 3822 m and 6952 m depth and included small individuals with broader bodies with inflated discs and short triangular arms, found on both rocky and soft substratum. Astropectinid morphotypes (Fig. 13J, K) were recorded at shallower abyssal depths and were characterised by robust pentagonal discs covered in small pores on the aboral side, displaying various degrees of inflation.

Valvatid asteroids were generally found on sediment or mixed substrates. Goniasterids (Fig. 13L–N) occurred at abyssal hadal depths between 3237 m and 4635 m and included pentagonal disc shapes and conspicuous marginal plates. Solasterids were less frequently encountered and included both a probable *Lophaster* morphotype (Fig. 13O) and an indeterminate solasterid morphotype between 3870 m and 4052 m (Fig. 13P), possessing eight arms.

Velatid asteroids were found between 4388 m and 7986 m and formed the dominant group of hadal asteroids. Three morphotypes of *Hymenaster* were identified in addition to indeterminate *Hymenaster* observations (Fig. 13Q–V) and displayed broad pentagonal bodies with an inflated, membranous appearance typical of the genus. These displayed bathymetric structuring with *Hymenaster* sp. indet. 1 occurring within abyssal to upper hadal depths between 4388 m and 5275 m, *Hymenaster* sp. indet. 2 extending from 6528 m to 7987 m depth and *Hymenaster* sp. indet. 3 occupying intermediate depths between 5818 m and 6944 m. *Hymenaster* sp. indet. 2 also resembled pterasterid asteroids reported in Lemche et al. (1976) from the Palau Trench axis at similar depth (8022–8042 m). In dive NC4_BK3_6900, *Hymenaster* sp. indet. 2 and 3 occurred in roughly equal proportions, raising the possibility that the smaller-bodied *Hymenaster* sp. indet. 3 may represent a juvenile form of *Hymenaster* sp. indet. 2. These *Hymenaster* individuals were observed in both inflated and contracted body forms (Fig. 13R–T and Fig. 13UV).

#### Class Holothuroidea

**Figure 14.**
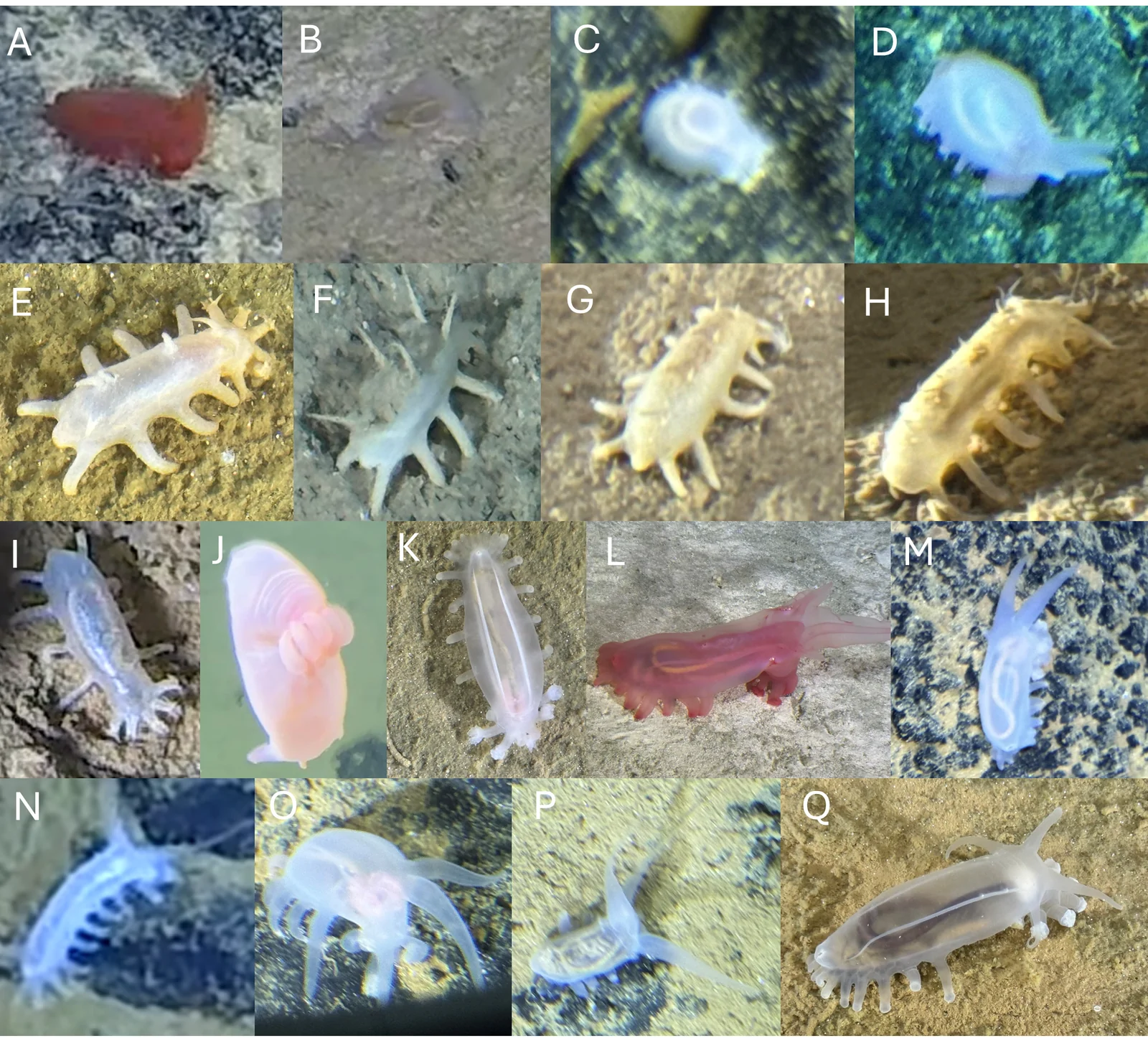
Echinodermata: Holothuroidea (Elpidiidae), with approximate depth of the shown observations. **A** Elpidiidae gen. indet. 1, 4723 m; **B** Elpidiidae gen. indet. 2, 4613 m; **C** *Amperima* sp. indet. 1, 5902 m; **D** *Amperima* sp. indet. 2, 5809 m; **E** *Elpidia* sp. indet. 1, 7369 m; **F** *Elpidia* sp. indet. 2, 6682 m; **G** *Elpidia* sp. indet. 3, 7987 m; **H** *Elpidia* sp. indet. 4, 7987 m; **I** *Elpidia* sp. indet. 5, 7989 m; **J** *Peniagone leander*, 6969 m; **K** *Peniagone* sp. indet. 1, 7369 m; **L** *Peniagone* sp. indet. 2, 4627 m; **M** *Peniagone* sp. indet. 3, 5836 m; **N** *Peniagone* sp. indet. 4, 5758 m; **O** *Peniagone* sp. indet. 5, 5761 m; **P** *Peniagone* sp. indet. 6, 5983 m; **Q** *Peniagone* sp. indet. 7, 7344 m.

**Figure 15.**
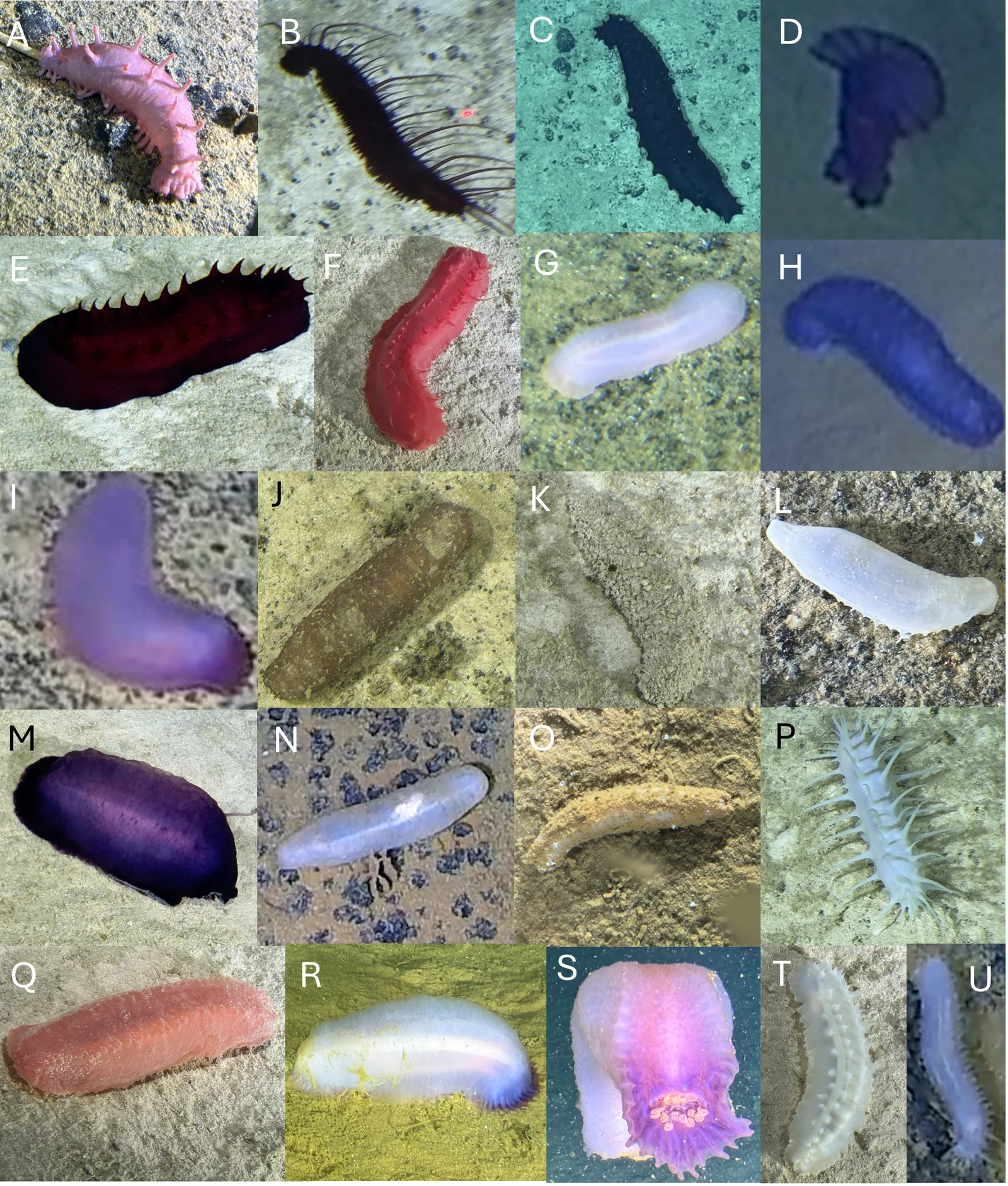
Echinodermata: Holothuroidea (cont.), with approximate depth of the shown observations. **A** Laetmogonidae gen. indet. 1, 6220 m; **B** *Psychronaetes* sp. indet. 1, 3760 m; **C** *Psychronaetes hanseni*, 4669 m; **D** *Enypniastes eximia*, 4482 m; **E** *Benthodytes* sp. indet. 1, 4630 m; **F** *Benthodytes* sp. indet. 2, 4424 m; **G** *Benthodytes* sp. indet. 3, 6228 m; **H** *Benthodytes* sp. indet. 4, 5600 m; **I** Psychropotidae gen. indet. 1, 5273 m; **J** *Mesothuria* sp. indet. 1, 5269 m; **K** *Mesothuria* sp. indet. 2, 3233 m; **L** *Mesothuria* sp. indet. 3, 6233 m; **M** *Benthothuria* sp. indet. 1, 4673 m; **N** *Molpadiodemas* sp. indet. 1, 5644 m; **O** *Molpadiodemas* sp. indet. 2, 7369 m; **P** *Oneirophanta* sp. indet. 1, 4304 m; **Q** *Paelopatides* sp. indet. 1, 4630 m; **R, S** *Paelopatides* sp. indet. 2, 6682 m; **T** Synallactidae gen. indet. 1, 4634 m; **U** Synallactidae gen. indet. 2, 6004 m.

The holothuroids (Figs 14, 15) were represented by four orders (Elasipoda, Holothuriida, Persiculida and Synallactida) comprising eight known families plus one without a designated family (*Benthothuria* sp. indet. 1) and one group, Holothuroidea spp., that were too small to classify beyond class.

The most diverse order was the Elasipoda that comprised four families (Elpidiidae, Laetmogonidae, Pelagothuriidae and Psychropotidae) (Fig. 14). Elpidiidae was the most diverse family with species and morphotypes in the *Amperima* (two morphotypes), *Elpidia* (five morphotypes plus *Elpidia* spp.) and *Peniagone* (seven morphotypes, one species and *Peniagone* spp.) genera. The two morphotypes of *Amperima* (*Amperima* sp. indet. 1 and 2) were seen at similar depths (5631–5902 m and 5248–5809 m) and both singletons observed during a single dive, distinguished by differences in the shape of the velum (*A.* sp. indet. 2 has much larger velum). The five morphotypes of *Elpidia* (*Elpidia* sp. indet. 1–5; Fig. 14E–I) were delineated by dorsal papillae morphology (number, length and arrangement). The *Elpidia* were particularly abundant > 6500 m albeit the vast majority were too small (~ 2 cm) and/or too far away to assign morphotypes, hence *Elpidia* spp. (5557–7987 m, > 5750 individuals). *Elpidia* sp. indet. 1 and 2 had broader depth ranges (5974–7532 m and 6094–7348 m, respectively), but did not extend into the deepest part of the fracture zone, while *Elpidia* sp. indet. 3–5 were only observed at lower hadal depths. It is unknown whether the differences in dorsal papillae shape and arrangements represent different developmental stages, but here they are cautiously assigned to morphotypes.

The *Peniagone* were represented by seven morphotypes as delineated by velum morphology. The most common was *Peniagone* sp. indet. 1 (> 1500 individuals, 6592–7987 m, depth range 1395 m; 14 deployments of both lander and submersible; Fig. 14K) and *Peniagone* sp. indet. 7 (6503–7345 m, depth range 842 m; three deployments from submersible; Fig. 14Q). *Peniagone* sp. indet. 2–6 were observed in numbers of 1–3 by submersible only and largely confined to depths between 5000 m and 6000 m (Fig. 14L–P). Amongst these observations were individuals too far or too small to identify, therefore assigned to *Peniagone* spp.; conversely the only Elpidiid identified to species level was *Peniagone leander* Pawson & Foell, 1986 that was seen in 14 deployments on both lander and submersible between 4980 m and 6969 m, often swimming freely in the near bottom currents (Fig. 14J).

In the Laetmogonidae family, three morphotypes were identified; Laetmogonidae gen. indet. 1 (5556–6221 m), *Psychronaetes* sp. indet. 1 (3760 m) and *Psychronaetes hanseni* Pawson, 1983 (4501–4670 m) (Fig. 15A–C). These were not especially common, observed as singletons in one or two submersible dives.

The only species observed in the Pelagothuriidae family was *Enypniastes eximia* Théel, 1882 between 4482 and 6299 m, observed twice from the submersible, swimming in the near bottom currents (Fig. 15D). Four morphotypes in the Psychropotidae family; *Benthodytes* sp. indet. 1–4 (Fig. 15E–H). They generally appeared in 2–4 submersible dives each with a total depth range of 4170–6228 m. Another Psychropotid morphotype, Psychropotidae gen. indet. 1, was observed between 5272 m and 6014 m on two dives. (Fig. 15I).

In the order Holothuriida, three morphotypes in the Mesothuriidae family were identified; *Mesothuria* sp. indet. 1, 2 and 3 (Fig. 15J–L). The first was seen on one dive at 5264–5270 m, whereas *Mesothuria* sp. indet. 2 and 3 were seen at 3233–6972 m and 4106–6645 m, with the relatively large depth ranges of 3739 and 2539 m, respectively. In the first instance, this large depth range may be attributed to the fact that this morphotype was delineated using sediment coverage, but this also prevented more accurate identification. *Benthothuria* sp. indet. 1 (no assigned family) was seen on four deployments of both lander and submersible between 3782 m and 4673 m (range = 891 m) (Fig. 14M). In the order Persiculida, two morphotypes belonging to the Molpadiodemidae family, tentatively identified as *Molpadiodemas* sp. indet. 1 and 2, were found, both restricted to hadal depths at 5645–6659 m and 6320–7961 m, as seen on three and five submersible dives, respectively (Fig. 15N, O). These also resemble *Hadalothuria wolffi* Hansen, 1956 and “*Hadalothuria*?” from Lemche et al. (1976), also in the order Persiculida.

In the order Synallactida, the Synallactidae family comprised four morphotypes; *Paelopatides* sp. indet. 1 and 2 (Fig. 15Q, R) and Synallactidae gen. indet. 1 and 2 (Fig. 15T, U). The two *Paelopatides* morphotypes spanned the lower abyssal depths and upper hadal depths of 4170–5749 m and 5270–6973 m and ranges of 1579 m and 1704 m, respectively. One individual *Paelopatides* sp. indet. 2 was perturbed by the presence of the submersible and ascended into the water column in view of cameras, providing detailed shots of the underside and mouth morphology (Fig. 15S). Synallactidae gen. indet. 1 and 2 were seen at 4435–4635 m and 4432–6004 m, therefore contrasting ranges of 199 m and 1572 m as derived from two and six deployments, respectively. A single morphotype from the Deimatidae family, *Oneirophanta* sp. indet. 1, was recorded on a single dive at 4304 m (Fig. 15P).

#### Class Crinoidea

Crinoids were observed across nearly the full depth range (3234–7964 m) and included representatives from the Comatulida (which include feather stars) and Hyocrinida orders.

**Figure 16.**
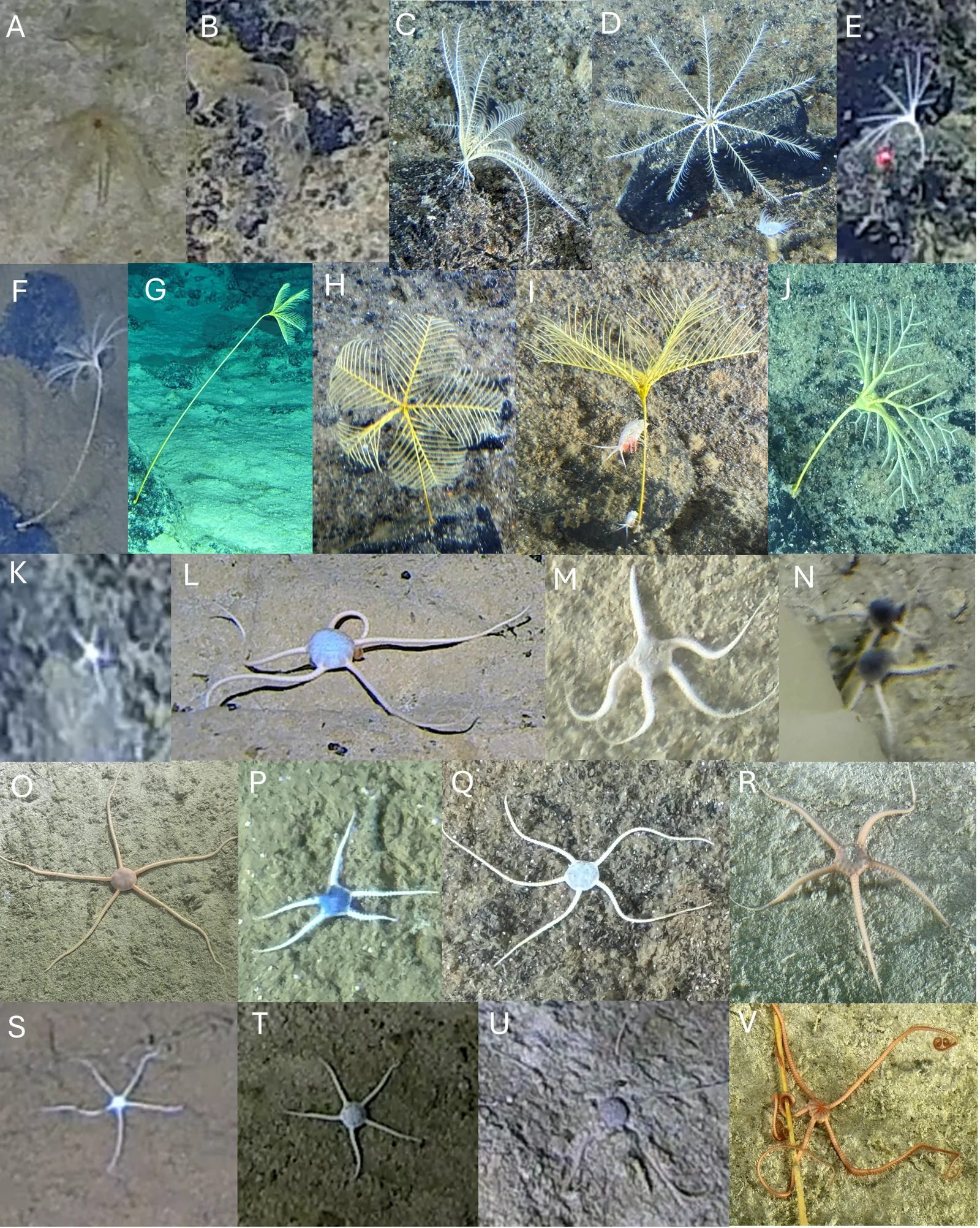
Echinodermata: Crinoidea + Ophiuroidea, with approximate depths of shown observations. **A** Comatulida fam. indet. 1, 3691 m; **B** Comatulida fam. indet. 2, 4585 m; **C** Pentametrocrinidae gen. indet. 1, 5648 m; **D, E** Bathycrinidae gen. indet. 1, 6228 m; **F** Bathycrinidae gen. indet. 2, 7983 m; **G** Hyocrinidae gen. indet. 1, 4397 m; **HI** Hyocrinidae gen. indet. 2, 6204 m; **J** Hyocrinidae gen. indet. 3, 6233 m; **K** Ophiuroidea spp., 6974 m; **L** Ophiuroidea fam. indet. 1, 5093 m; **M** Ophiuroidea fam. indet. 2, 4623 m; **N** Ophiuroidea fam. indet. 3, 4630 m; **O** Ophiuroidea fam. indet. 4, 3273 m; **P** Ophiuroidea fam. indet. 5, 6678 m; **Q** Ophiuroidea fam. indet. 6, 6233 m; **R** Ophiuroidea fam. indet. 7, 3802 m; **S** Ophiuroidea fam. indet. 8, 7919 m; **T** Ophiuroidea fam. indet. 9, 5264 m; **U** Ophiuroidea fam. indet. 10, 3239 m; **V** Ophiaplinthacata gen. indet. 1, 3273 m.

Shallower abyssal depths included two comatulid (“feather star”) morphotypes; Comatulida fam. indet. 1 (3234–3691 m, Fig. 16A) and 2 (4586–5265 m, Fig. 16B). Comatulids occurring at lower hadal depths included Pentametrocrinidae gen. indet. 1 (Fig. 16C), which occurred between 5659 m and 6222 m depth and was characterised by a compact crown with 10 undivided arms, stalk absent, whereas Bathycrinidae gen. indet. 1 (Fig. 16D, E) occurred between 4043 m and 6228 m and possessed a relatively short stalk supporting a crown of ten undivided arms. Other members of the Bathycrinidae family, Bathycrinidae gen. indet. 2, displayed a deeper depth range extending from 5262 m to 7964 m depth. Although observed once at shallower abyssal depths around 5000 m, these white coloured crinoids became increasingly abundant within the deepest parts of the fracture zone (Fig. 16F). Colonies possessed 10 undivided arms extending from elongate stalks.

Around lower hadal depths, stalked crinoids of the Hyocrinida order were increasingly abundant and were characterised by five arms and yellow colour (Fig. 16G–J). Several morphotypes were recognised in the Hyocrinidae family that differed in stalk length and pinnule arrangement. Hyocrinidae gen. indet. 1 (Fig. 16G) occurred between 4397 m and 5550 m depth and possessed a long stalk with a cup-shaped theca. Hyocrinidae gen. indet. 2 (Fig. 16H, I) was characterised by long pinnules up extending to nearly half the arm length and was only observed during a single dive between 6204 m and 6228 m depth, potentially representing a locally occurring form. *Amathillopsis* amphipods were frequently observed in association with this morphotype, clinging to stalks or filtration crowns suggesting elevated crinoid structures may provide important perching or feeding positions. Hyocrinidae gen. indet. 3 (Fig. 16J) occurred between 5383 m and 7496 m and was distinguished by asymmetrically branching arms with sparse elongate pinnules.

#### Class Ophiuroidea

Ophiuroids (brittle stars) were amongst the most broadly distributed echinoderms observed, occurring throughout nearly the entire surveyed depth range in abundance. Approximately 4000 individual ophiuroids were observed, of which the vast majority were too small to assign a morphotype and, therefore, collectively designated Ophiuroidea spp. (Fig. 16K). Of the remaining ones, 10 morphotypes were distinguished which displayed bathymetric structuring.

Several morphotypes appeared restricted to abyssal depths shallower than ~ 6000 m, including Ophiuroidea ord. indet. 1, 2, 3, 7 and 10 (Fig. 16L, M, N, R, U). Morphotypes occurring around the abyssal-hadal transition zone included Ophiuroidea ord. indet. 5 and 9, both characterised by compact discs with comparatively short arms. Deeper hadal forms, which were also more abundant, included Ophiuroidea 6 (~ 50 observations) and 8 (~ 330 observations). Compared to other morphotypes, these had lighter body colouration and longer, more delicate arms. The two forms differed primarily in disc appearance under illumination, with Ophiuroidea ord. indet. 8 having a noticeably brighter and more reflective central disc. It is uncertain whether these morphotypes represent distinct groups or variation within a single taxon, but they were retained as separate morphotypes pending further material or higher-resolution imagery.

One particularly distinctive ophiuroid was of the Ophiacanthidae family (Fig. 16V) with long coiling arms which were frequently observed entwined around stalked fauna at shallower abyssal depths between 3100 m and 3803 m (~ 100 observations).

### Phylum Porifera

**Figure 17.**
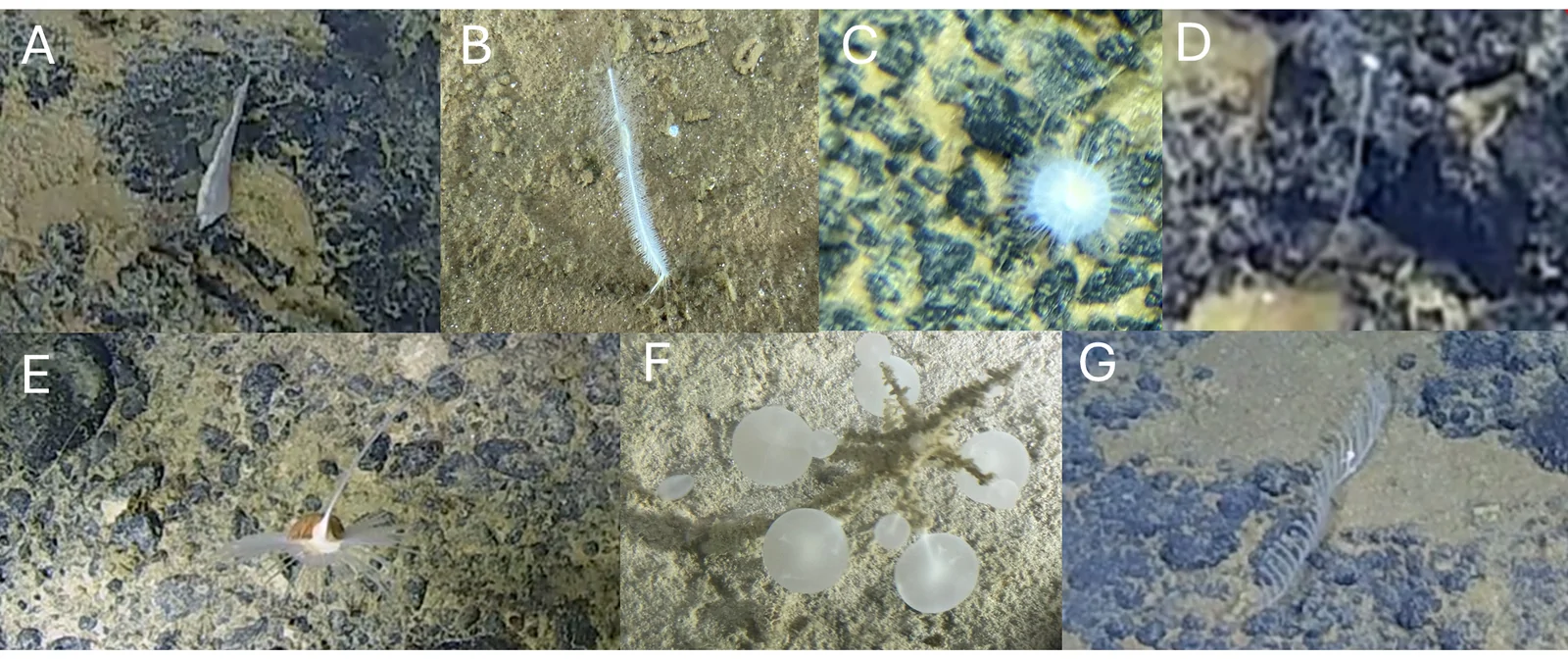
Demosponges. **A** Demospongiae ord. inc. 1, 5868 m; **B** Cladorhizidae gen. indet. 1, 6657 m; **C** Cladorhizidae gen. indet. 2, 5476 m; **D** Cladorhizidae gen. indet. 3, 6009 m; **E** Cladorhizidae gen. indet. 4, 5265 m; **F** *Chondrocladia* sp. indet. 1, 4603 m; **G** *Chondrocladia* sp. indet. 2, 5987 m.

**Figure 18.**
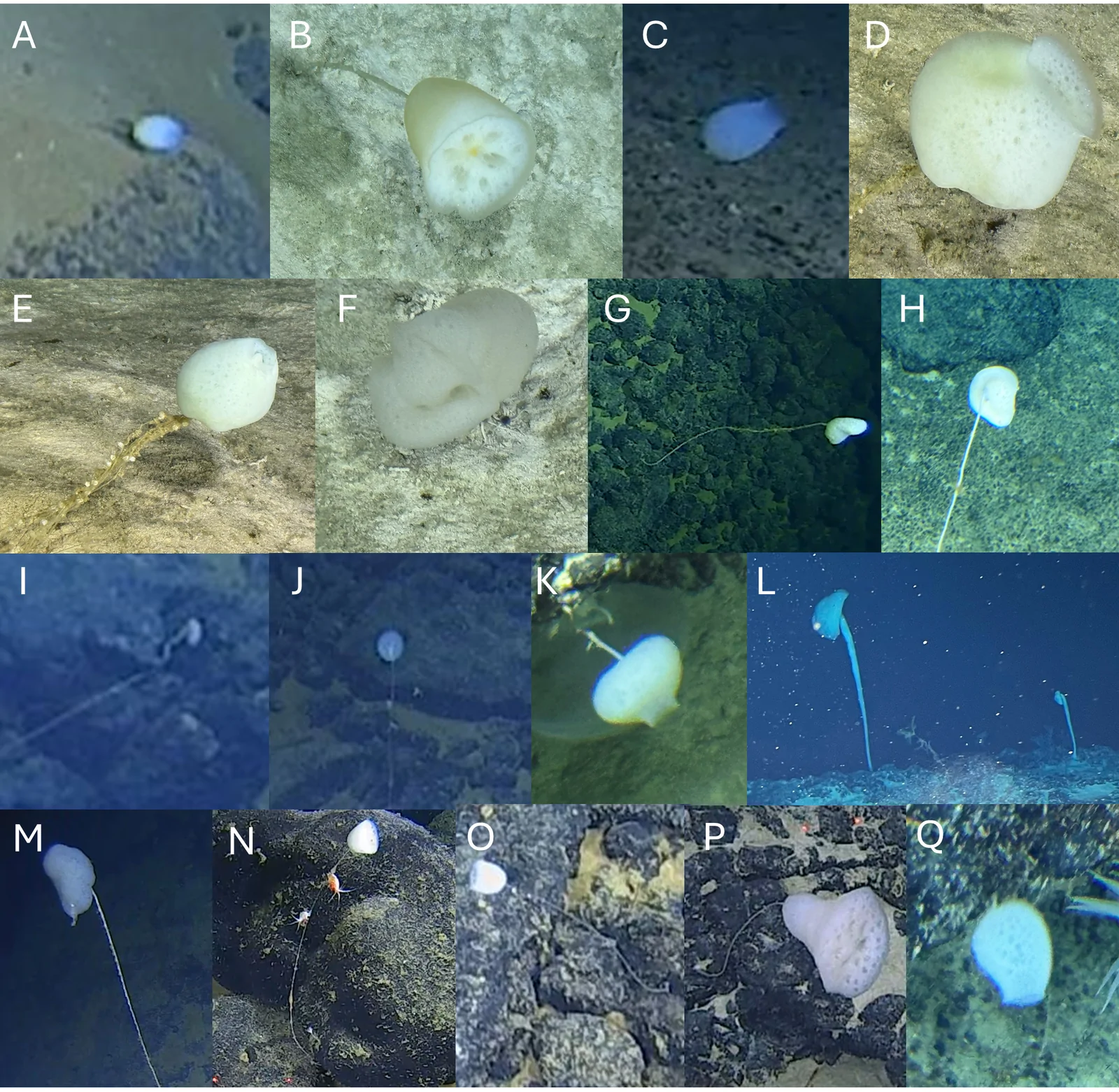
Stalked Hexactinellids. Approximate depths of shown observations have been indicated where possible. **A** Hexactinellida ord. inc. 5, 5434 m; **B** *Hyalonema* sp. indet. 1, 4624 m; **C** *Hyalonema* sp. indet. 2, 5272 m; **D** *Hyalonema* sp. indet. 3, 4625 m m; **E** *Hyalonema* sp. indet. 4, 4550 m; **F** Lyssacinosida fam. inc. 1, 3732 m; **G** Lyssacinosida fam. inc. 4, 5639 m; **H** Lyssacinosida fam. inc. 5, 5278 m; **I** *Caulophacus* gen. inc. 1; **J** *Caulophacus* gen. inc. 1; **K** Lyssacinosida fam. inc. 7, 4454 m; **L** Lyssacinosida fam. inc. 8, 3843 m; **M** Euplectellidae fam. inc. 6, 5400 m; **N** *Hyalostylus* sp. indet. 1, 5791 m; **O** *Hyalostylus* sp. indet. 2; **P** *Hyalostylus* sp. indet. 3, 4630 m; **Q** Hexactinellida ord. inc. 6, 4645 m.

**Figure 19.**
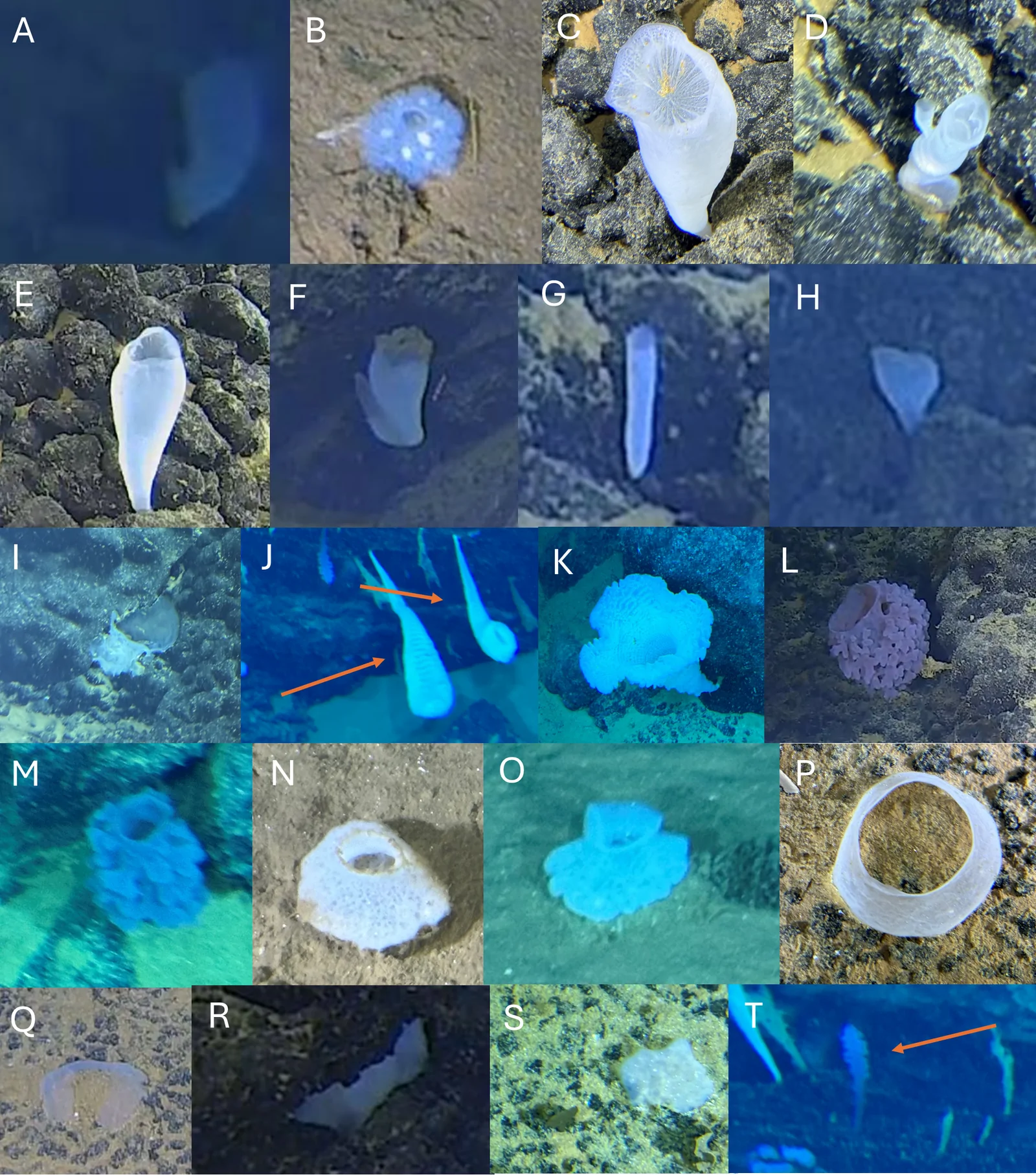
Other Hexactinellid morphologies. Approximate depths of shown observations have been indicated where possible. **A** Hexactinellida ord. inc. 2, 5557 m; **B** Lyssacinosida fam. inc. 3, 6676 m; **C** Euplectellidae gen. indet. 2; **D** Euplectellidae gen. indet. 3; **E** Euplectellidae gen. indet. 4, 5580 m; **F** Euplectellidae fam. inc. 5; **G** Euplectellidae gen. inc. 7. Funnel Morphologies; **H** Hexactinellida ord. inc. 4, 5702 m; **I** Euretidae gen. inc. 1; **J** Euretidae gen. inc. 2; **K** Euretidae gen. inc. 3, 5163 m; **L** *Periphragella* sp. inc. 1, 5500 m; **M** Periphragella sp. inc. 2. Nest Morphologies; **N** Lyssacinosida sp. fam. inc. 2; **O** Euplectellidae fam. inc. 1, 5643 m; **P** *Holascus* sp. indet. 1, 5997 m; **Q** *Holascus* gen. inc. 2, 5995 m. Uncommon morphologies: **R** Hexactinellida cls. inc. 1, 5277 m; **S** *Docosaccus* gen. inc. 1, 5994 m; **T** Farreidae gen. indet. 1, 5373 m.

Sponges belonged to the classes Demospongiae and Hexactinellida, comprising 258 observations (185 + 73) across 48 morphotypes (Figs 17, 18, 19). Notably, the study area represents the most diverse assemblages of abyssal-hadal hexactinellids reported to date (Marchiò et al. 2025b).

#### Class Demospongiae

Demosponges were the least abundant and diverse sponge class, with eight morphotypes distinguished. Seven morphotypes were assigned to the family Cladorhizidae (Demospongiae, Poecilosclerida), which comprises carnivorous sponges (Hajdu and Vacelet 2002). The only non-carnivorous demosponge showed a thin fan morphology, preventing confident assignment at the order rank (Fig. 17A). Carnivorous sponges dominated the demosponge fauna, comprising four morphotypes identified at family level with either erect (*Asbestopluma*/*Abyssocladia*-like; Fig. 17B, C) or umbrella-shaped morphologies (*Axoniderma*/*Cladorhiza*-like; Fig. 17D, E). The two remaining morphotypes were assigned to the genus *Chondrocladia* (ping-pong tree and lyra morphologies; Fig. 17F, G). Specimens that could not be reliably identified were grouped into a bin morphotype (Cladorhizidae spp.).

#### Class Hexactinellida

Hexactinellids (“glass sponges”) were the most abundant class, with 40 morphotypes and 185 observations. Seven morphotypes were identified at class rank, eight at order rank, 11 at family and 14 and genus. Two bin morphotypes were established for uncertain specimens: Hexactinellida spp., for poorlyresolved observations and *Hyalonema* spp., for short-stalked specimens on fine sediment lacking further diagnostic features.

Morphotypes were classified into seven morphological categories: Stalked (Fig. 18), Barrel (Fig. 19A–G), Funnel (Fig. 19H–L), Nest (Fig. 19N–Q), Irregular (Fig. 19R), Plate (Fig. 19S) and Erect (Fig. 19T).

From a taxonomic perspective, stalked morphotypes occur in both subclasses Amphidiscophora and Hexasterophora. All three amphidiscophoran families include at least one stalked genus, generally associated with soft sediments (the majority of Hyalonematidae; Monorhaphidae: *Monorhaphis*; Pheronematidae: *Ijimalophus*). Amongst these, *Hyalonema* is particularly widespread and is characterised by a stalk formed by twisted basal spicules supporting a relatively small cup-like body (Fig. 18B–E; Tabachnick and Menshenina 2002).

Within Hexasterophora, stalked forms are mostly represented by Lyssacinosida (Fig. 18F–P), particularly within the families Rossellidae (Fig. 18I, J) and Euplectellidae (Fig. 18M). These lyssacinosid stalked sponges can attain considerably larger sizes than hyalonematids. Certain species of *Caulophacus* (Rossellidae) may reach substantial lengths and body masses (Fig. 18I, J), together with large euplectellids, such as *Bolosoma* and *Saccocalyx*. In contrast, *Hyalostylus* (Fig. 18N–P) remains comparatively small, typically possessing a thin, untwisted stalk and a bell-shaped body superficially resembling *Hyalonema*. However, *Hyalostylus* differs in having a basiphytous attachment and occurring predominantly on hard substrates.

Short-stalked mushroom-like morphotypes (Fig. 18K–Q) may belong to several genera, including *Amphidiscella* (Euplectellidae) and *Sympagella* (Rossellidae). These taxa are often highly similar in external morphology and can be difficult to distinguish reliably to family level from imagery alone.

The barrel morphology (Fig. 19A–G) comprises erect sponges with a large atrial cavity occupying most of the body and terminating in an osculum, which may be either open or covered by a sieve plate. In these forms, a narrow pedunculate base expands into the main body, which may widen similarly to funnel-shaped morphologies, but subsequently narrows again towards the osculum, maintaining a constant diameter in the upper section. This morphology is particularly common, although not exclusive, to Euplectellidae and includes several “Venus’ flower basket” genera (*Euplectella*, *Regadrella*, *Corbitella* and *Dictyaulus*; Tabachnick 2002).

The funnel morphology (Fig. 19I–M) is characterised by a small attachment area from which a short peduncle expands into a broad atrial cavity that continuously increases in diameter as the sponge grows in height. This morphology is typical of some Euretidae (Reiswig and Wheeler 2002), such *Periphragella* (Fig. 19L, M), as well as other sceptrulophorans of uncertain taxonomic identity (Dohrmann et al. 2023), as *Lefroyella*, which may be represent the flowering specimen as in Fig. 19K.

Nest sponges (Fig. 19N–Q) possess a wide basal area that gradually narrows towards a smaller central osculum. Like stalked lyssacinosids, this morphology occurs in both Euplectellidae (Fig. 19O) and Rossellidae (Fig. 19P, Q). Nest morphotypes have been observed on fine sediments and nodule fields, but never on hard substrates.

Unusual morphologies include an irregular unidentified hexactinellid (Fig. 19R), a plate-like sponge identified as *Docosaccus* gen. inc. 1 (Fig. 19S), resembling some specimens collected from the Clarion-Clipperton Zone (Simon-Lledó et al. 2023) and an erect sponge assigned to the family Farreidae (Fig. 19T). This specimen exhibited a central cylindrical body with short, unbranched lateral tubes arranged in a two-dimensional configuration, rather than the radial branching typical of *Periphragella* (Reiswig 2002). With this morphology, the specimen was assigned to Farreidae, as we could not confidently identify it as either *Aspidoscopulia* or *Farrea*.

Glass sponges were first observed around 3700 m, but their highest abundance and morphological diversity occurred between 5000 m and 5700 m. Abundance peaked around 5200 m with 35 specimens, while morphological diversity reached its maximum value at approximately 5600 m with 14 morphotypes. Observations became sparse below 6000 m, with only three morphotypes observed at approximately 6600 m and a single nest sponge was recorded at approximately 6900 m, representing the deepest specimen in the explored area.

### Other Animalia

Several additional phyla within the Kingdom Animalia were observed only infrequently across the Nova Canton Trough with comparatively low morphotaxon diversity and, sometimes, restricted depth distributions. Many of these groups were only recorded from a limited number of deployments, suggesting sparse or patchy distributions within the study area.

#### Phylum Chaetognatha

**Figure 20.**
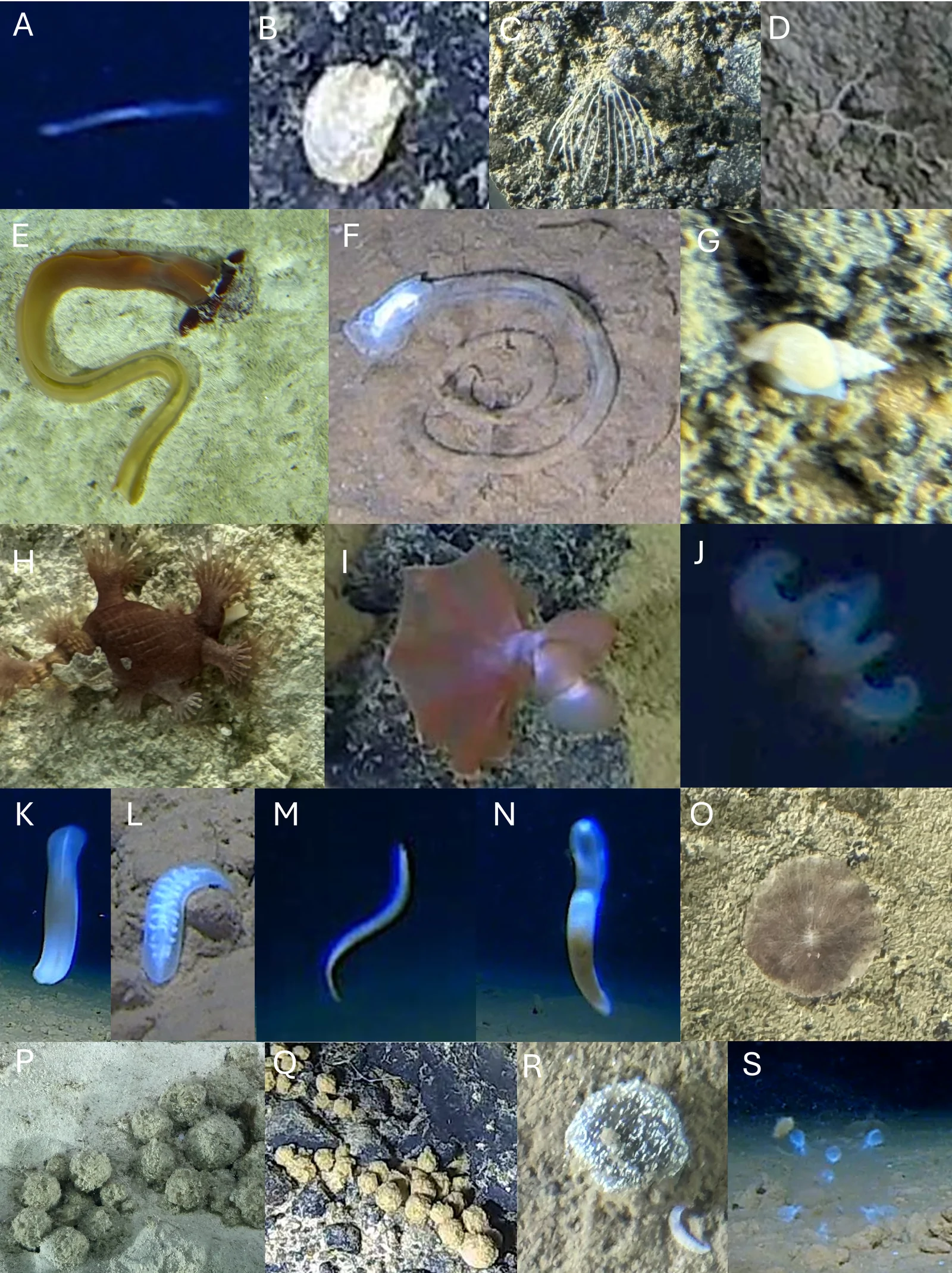
Rare or Low Diversity Phyla within Kingdom Animalia and Kingdom Protista, with approximate depth of shown observations. Kingdom Animalia: **A** Chaetognatha spp., 6006 m; **B** Brachiopoda spp., 6671 m; **C** Bryozoa cls. indet. 1, 5295 m; **D** Bryozoa cls. indet. 2, 3215 m; **E** Torquatoridae gen. indet. 1, 4536 m; **F** Torquatoridae gen. indet. 2, 6661 m; **G** Buccinidae spp.; **H** Neogastropoda sp. indet. 1, 3273 m; **I** *Cirroteuthis* sp. indet. 1, 6015 m; **J** Octopoda spp., 4549 m; **K** Nemertea cls. indet. 1, dorsal view, 7470 m; **L** Nemertea cls. indet. 1, ventral view; **M** Nemertea cls. indet. 2, 7304 m; **N** Nemertea cls. indet. 3, 5497 m; **O** Platyhelminthes spp., 5272 m. Kingdom Protista: **P** Radiozoa cls. indet. 1, 3804 m; **Q** Radiozoa cls. indet. 2, 5264 m; **R** Radiozoa cls. indet. 3, 6653 m; **S** *Tuscaridium* spp.

Arrow worms (Chaetognatha) (Fig. 20A) were regularly encountered, occurring across 72 deployments between 2751 m and 7621 m. Due to their small size, no specific morphotypes were resolved within this phylum and so there is likely considerable unresolved diversity within this group.

#### Phylum Brachiopoda

Brachiopods, characterised by a convex to flat shell, were recorded only in two submersible dives between 5586 m and 6671 m depth, possibly belonging to the order Rhynchonellata (Fig. 20B).

#### Phylum Bryozoa

Bryozoans were exclusively recorded from submersible imagery from three dives between 3215 m and 5295 m (Fig. 20C, D). Two distinct morphotypes were recognised: a fan-shaped upward branching form that appeared locally dominant in the deepest of three dives (Fig. 20C) and an irregularly shaped form predominantly associated with the shallowest dive (Fig. 20D).

#### Phylum Hemichordata

Enteropneust hemichordates (acorn worms) were recorded from lower abyssal to upper hadal depths. Two distinct morphotypes were recognised: one with a thick red-orange anterior body tapering to a yellow-green posterior end (Fig. 20E, 4429–4536 m) and one with a largely translucent body (Fig. 20F, 6661 m). These were observed making faecal trails and actively traversing the seafloor. Spiral and switchback faecal trails attributed to enteropneust activity were observed throughout the Nova Canton Trough (Swanborn et al. 2026 and see section Lebensspuren).

#### Phylum Mollusca

Molluscs were represented by isolated cephalopod and gastropod observations. Cirrate octopods (*Cirroteuthis* sp. indet. 1) (Fig. 20I) occurred across four deployments between 5151 m and 6015 m depth, whilst an indeterminate octopod was observed during a lander deployment at 4549 m depth (Fig. 20J). Gastropods occurred across much of the Nova Canton Trough depth range (3799–6678 m) and although many were too small to be classed into morphotypes, individuals likely belonging to the Buccinidae family (Fig. 20G) were observed from 5269–7203 m and an individual belonging to the Neogastropoda (Fig. 20H), characterised by a reddishbrown shell consisting of five whorls, was observed at 3273 m.

#### Phylum Nemertea

Three nemertean morphotypes were resolved (Fig. 20K-N), with behavioural characteristics contributing to their differentiation. Nemertea cls. indet. 1 (6480-7987 m) had a dorsoventrically flattened, smooth, featureless body with rounded terminal ends. Nemertea cls. indet. 2 (5367–7470 m) was narrower in form and displayed an undulating S-shaped swimming motion. While Nemertea cls. indet. 3 (5497–6094 m) exhibited a distinctive swimming behaviour characterised by the simultaneous flapping of both ends to propel itself upwards in the water column.

#### Phylum Platyhelminthes

Platyhelminthes were only recorded with confidence during one submersible dive between 5265 m and 5269 m and were represented by a rounded pink morphotype (Fig. 20O). See section ‘Unknown Fauna and Anthropogenic debris’ for a discussion of possible Platyhelminthes presence at hadal depths.

### Protists

Although Animalia comprised the majority of observations, protists formed a significant component of the abyssal and hadal assemblages in the Nova Canton Trough.

#### Phylum Cercozoa

Planktonic phaeodarians ('Minesweeper radiolarians’) in the genus *Tuscaridium* (Fig. 20O) (Adl et al. 2019), were observed across a broad depth range (2751–7987 m). These organisms construct mineral skeletons with needle-like pseudopods that likely aid buoyancy.

#### Phylum Radiolaria

Several unusual spherical clusters tentatively categorised as Radiolaria were observed across a broad depth range (Fig. 20P, Q, R). These ‘mudballs’ were observed in quantities up to hundreds at times. These are believed to be radiolarian-dominated detritus, formed by accumulating smaller radiolarian-sized spheres (A. Gooday, pers. comm.). Some aggregates appeared concentrated in depressions (Radiozoa cls. indet. 1, 3234–3804 m) or associated with larger features as rocks and boulders (Radiozoa cls. indet. 2, 5265–5295 m) (Fig. 20PQ). Darker forms observed between 6653 m and 6683 m (Fig. 20R) possessed white surface material, which was also abundant in the dive area and may have been accumulated by rolling around on the seafloor.

#### Phylum Foraminifera

**Figure 21.**
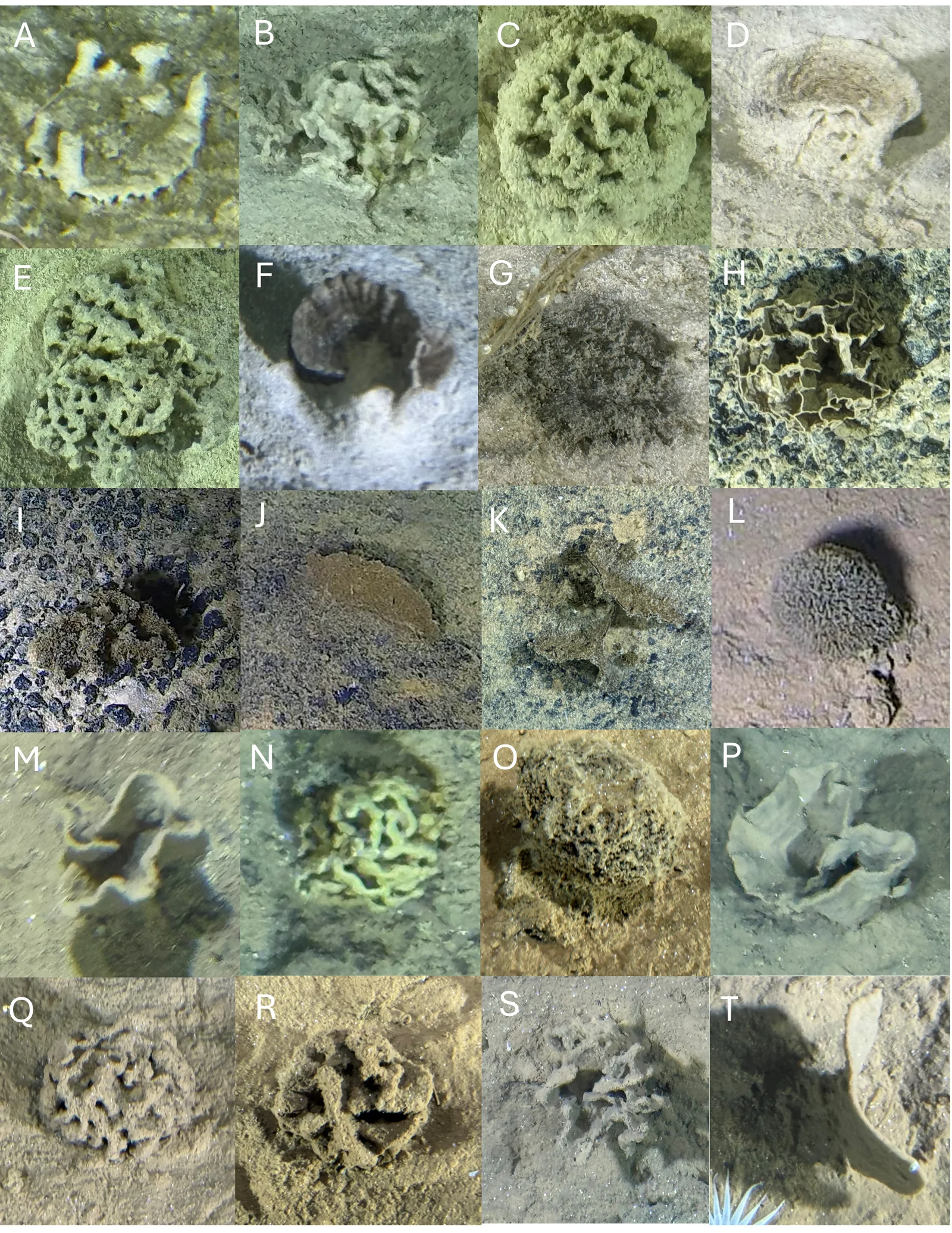
Foraminifera, Xenophyophorea: **A–E** = ~ 3200 m; **F–G** = ~ 4600 m; **H–I** = ~ 5300 m; **J–L** = ~ 6200 m; **M–T**= ~ 6700 m.

**Figure 22.**
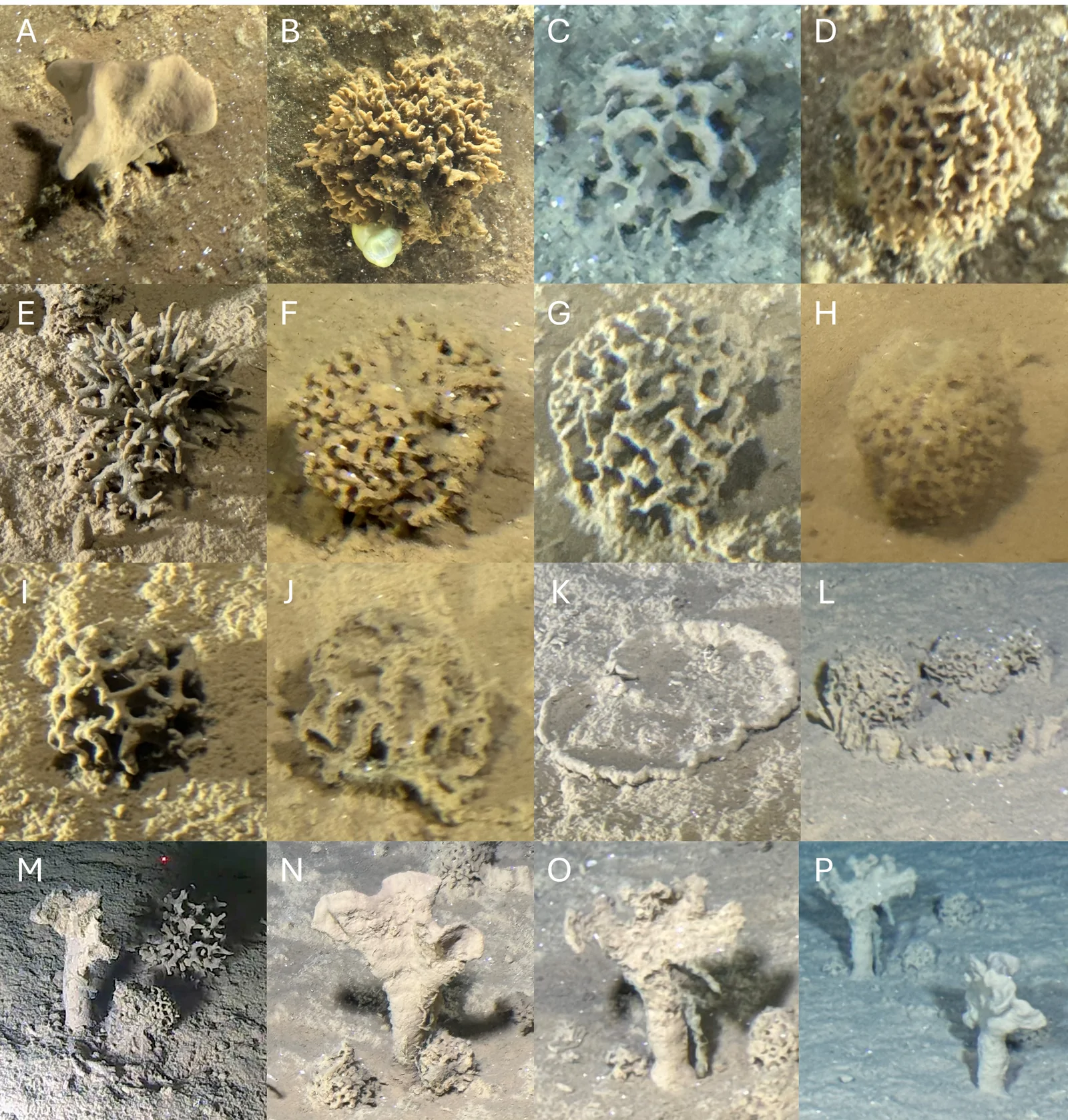
Foraminifera, Xenophyophorea (cont.): **A–D** = ~ 7400 m; **E–P** = ~ 8000 m.

Xenophyophores (Foraminifera, Monothalamea) formed one of the most visually conspicuous biogenic components of the benthos throughout the Nova Canton Trough (Figs 21, 22). These agglutinated foraminifera occurred across the full surveyed depth range and displayed substantial morphological diversity. Xenophyophores were especially abundant in the deepest part of the Nova Canton Trough at ~ 8000 m depth.

Due to their high abundance and the difficulty of distinguishing living individuals from empty tests, xenophyophores were not comprehensively annotated in the dataset. In addition, identifying xenophyophores from imagery is challenging because of their cryptic morphology. A selection of body forms are displayed here to showcase their abundance and diversity. Forms are presented in approximate order along the depth gradient to illustrate changes in morphologies with depth.

At shallow abyssal depths of around 3200 m, xenophyophores were represented primarily by rounded reticulate forms, although unusual lattice-like structures and shallow cup-like morphologies were also found (Fig. 21A–E).

At intermediate abyssal depths between ~ 4600 m and 5300 m (Fig. 21F–I), forms became increasingly elevated and three-dimensional in structure with changes in colouration representing substrate conditions. At depths of around 6200 m (Fig. 21J–L), xenophyophores appeared fragile, darker-coloured and included delicate flake-shaped structures (Fig. 21J) and intricate tightly-reticulated rounded structures (Fig. 21I–L). Around 6700 m (Fig. 21M–T), xenophyophores displayed high morphological diversity, containing rounded forms of varying degrees of reticulation (Fig. 21NOQRS), fan-shaped (Fig. 21T) and cup-shaped (Fig. 21MP) forms, some with thickened, elevated lobes, including a yellow-coloured morphotype (Fig. 21N).

In soft-sediment habitats at lower hadal depths of between 7400 m and 8000 m (Fig. 22), xenophyophores became particularly abundant and diverse. Compact rounded forms of varying degrees of reticulation (Fig. 22B–J), elevated stalked structures (Fig. 22M–P), as well as flattened, semi-circular forms (Fig. 22K, L) co-occurred. In the deepest part of the fracture zone, xenophyophores dominated the visible benthos with densities reaching approximately 20 individuals/m^2^.

Across the depth gradient, xenophyophore morphology appeared closely linked to changing sediment conditions, including sediment abundance, particle size and overall composition. The lower-relief abyssal forms were generally associated with fine-grained sediments and areas of mixed unconsolidated or partially consolidated substrates. Sites within the abyssal-hadal transition zone featured more areas of hard substrate, characterised by exposed bedrock outcrops, polymetallic nodule accumulations and thin sediment veneers. Reduced sediment accumulation in these areas may explain the prevalence of more "fragile" forms, as it may limit the development of larger forms. In contrast, hadal environments supported increasingly structurally complex morphologies constructed from fine, loosely consolidated sediments that accumulated within the fracture zone axis basin. Variability in colouration across the depth gradient may likewise reflect differences in incorporated sediment type and particle composition. Paler versus darker colours likely reflect a number of factors such as the clear delineation by water depth between observed calcareous or clay-dominated fine-grained sediments, which is attributable to the known Carbonate Compensation Depth (CCD), whereby carbonate deposition is precluded in waters exceeding 4000-4500 m (Bickert 2009). Additionally, whether dark coloured grains and fragments are locally available through weathering of volcanic bedrock and polymetallic nodules.

### Lebensspuren

**Figure 23.**
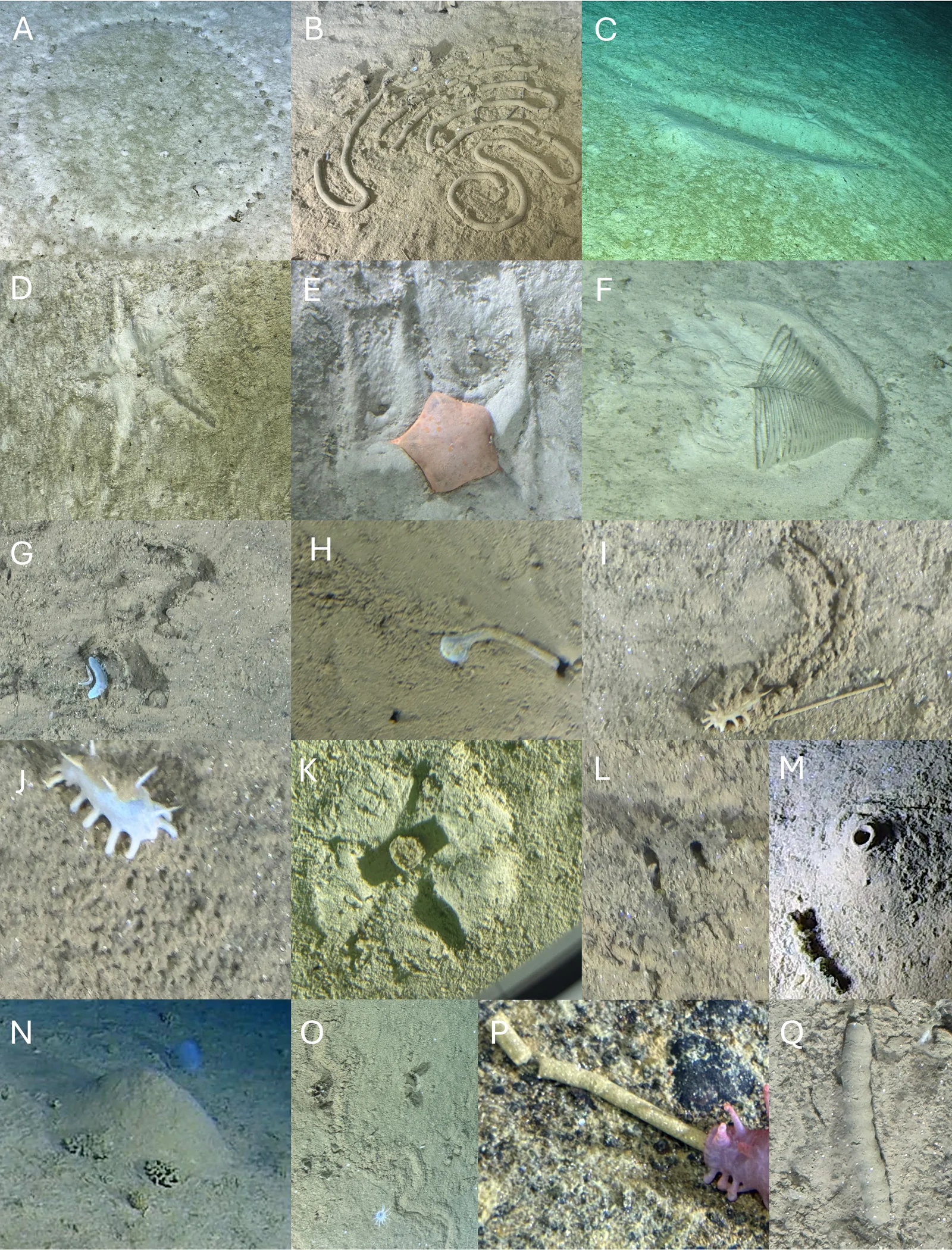
Lebensspuren examples, with approximate depths of shown observations. **A** Ring of burrows or ‘Fairy Circles’ of unknown origin, 3791 m; **B** Switchback faecal casts of enteropneust worms, 6682 m; **C** putative whale pits made by deep diving beaked whales, 3272 m; **D** Asteroids impressions, 3272 m; **E** in-situ burrowing of Asteroids, 4630 m; **F** mechanical erosion of surficial sediments by anchored Antipatharia, 4630 m; **G** shallow sediment turnover by Hesionidae annelids, 6682 m; **H** in-situ burrowing and feeding Echiuroidea, 7985 m; **I** Deep furrowing of surficial sediment by *Elpidia*, 6682 m; **J** perforated trail of sediment surface by *Elpidia* tube feet, 6682 m; **K** unknown perturbation, 3791 m; **L** infaunal burrows, 6233 m; **M** burrows with protruding chimney structure, 7987 m; **N** smooth mounds, 7987 m; **O** miscellaneous surface tracks, 6682 m; **P–Q** holothuroid faecal casts, 6233-6682 m.

In addition to observations of macro- and megafauna, a variety of lebensspuren and sediment disturbance features were observed reflecting activity of organisms on the seafloor (Fig. 23). A selection of recurring or unusual trace morphologies are discussed here that provide evidence of organismal activity even where the responsible fauna was not directly observed.

One of the most unusual patterns was the occurrence of circular arrangements of shallow burrows or ‘fairy circles’ of unknown origin (Fig. 23A) on relatively undisturbed soft-sediment plains. Eight instances were documented between 3220 m and 3799 m depth.

Spiral and coiled faecal traces produced by enteropneust worms were widespread across abyssal and hadal depths, often extending into intricate patterns (Fig. 23B).

A total of 63 large, elongate depressions were recorded on submersible dive NC2_BK1_3200 (Fig. 23C). There was no indication they were formed in chains and they were often grouped in small areas with no predominant orientation. The marks were elongate, approximately 60–100 cm long, 10–20 cm wide and 10 cm deep, with raised rims. Some were flat-bottomed or U-shaped in cross section and others had a central groove or were V-shaped in cross section. These pits are presumed to have been made by deep-diving beaked whales.

Multiple examples of sediment disturbance associated with locomotion and burrowing were documented. Asteroids left clear impressions within soft sediments resulting from resting (Fig. 23D) or active burrowing behaviour (Fig. 23E). Anchored antipatharians were observed generating sediment disturbance patterns around their attachment structures, likely produced by current-induced movement (Fig. 23F).

Examples of vermiform burrowing, feeding and locomotion-induced disturbances are shown in Fig. 23G, H. Hesionid annelids utilised burrows or depressions in dive NC1_BK2_6700, whereas echiurans were observed actively burrowing and feeding (Fig. 23H) within soft sediment habitats in the deepest part of the trough. Echiuran burrows were highly abundant and frequently observed without occupant (Swanborn et al. 2026).

Holothuroids generated some of the most extensive lebensspuren across the Nova Canton Trough. Locomotion activity produced extensive surface tracks, including deep furrowing of surficial sediment by *Elpidia* (Fig. 23I) and miscellaneous surface tracks of unknown holothuroid origin (Fig. 23O). Trails of regularly-spaced perforations produced by *Elpidia* tube feet were also recorded (Fig. 23J). Holothuroid faecal casts occurred frequently across soft-sediment habitats (Fig. 23P, Q), with cast morphology taxon-dependent.

Additional sedimentary structures included unknown perturbations (Fig. 23K), infaunal burrows (Fig. 23L), burrows with protruding chimney structures (Fig. 23M, N) and smooth mounds (Fig. 23N).

### Unknown Fauna and Anthropogenic debris

**Figure 24.**
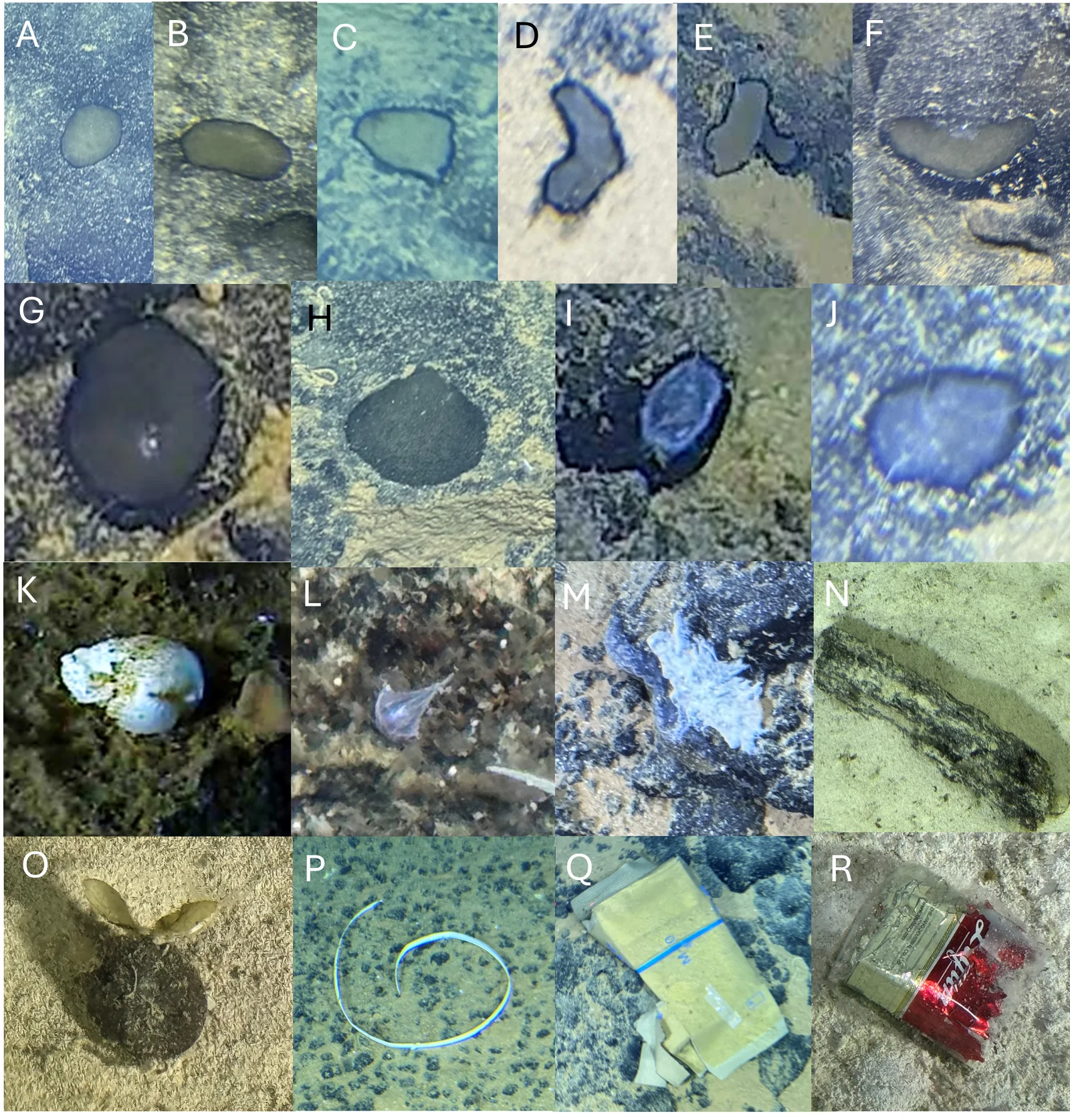
Unknown Fauna and Anthropogenic debris. **A–J** putative Platyhelminthes of various different morphologies; **K** Possible dead organism, closed anemone or discarded molluscan shell, 6233 m; **L** small tear-drop-shaped organisms, 6233 m; **M** possible disintegrated dead sponge, 6014 m; **N** wood, 3791 m; **O** plant debris, germinated seed, 3791 m; **P** plastic binding strap, 5921 m; **Q** discarded cardboard, 5921 m; **R** cigarette packet, 3273 m.

The dives NC3_BK2_6000 and NC3_BK4_8000 recorded an abundance of organisms seen adhering mostly to hard rock that were challenging to identify with confidence. Their body forms were small and generally oval to elongated flat and soft, with a darker frill around the perimeter of the body. These tended to be in the size range of up to 5 cm and exhibited body morphologies ranging from simply round through to more complex (see range in Fig. 24A–E). Larger body forms were seen with a similar frill and more organismal in appearance, that may be more mature individuals (Fig. 24F, G) and paler individuals, with a bluer body colour, were also seen. In the absence of physical samples, it is challenging to place these into a phylum, although, based purely on basic morphology, they appeared to mostly resemble Platyhelminthes (flat worms).

Other cryptic fauna comprised something that was either dead organism, closed anemone or discarded molluscan shell at 6233 m (Fig. 24K). The same dive revealed an abundance of very small tear-drop-shaped organisms scattered around the sediment surface that often reflected the light from the submersible, but were too small to identify (Fig. 24L). A possible disintegrated dead sponge was seen at 6014 m (Fig. 24M).

The only natural terrestrial debris was a section of wood and what appeared to be a germinated seed or other plant debris at 3791 m (Fig. 24N, O). The only anthropogenic debris noted from the study was a length of plastic binding wire, a cardboard box at 6014 m and discarded packet of cigarettes at 3273 m (Fig. 24P–R).

## Discussion

This survey of the Nova Canton Trough represents the most extensive visual biodiversity inventory produced for a hadal feature to date. Combining footage of nearly 100 baited lander deployments and 16 submersible transects spaced in high-resolution depth intervals across a continuous depth gradient (~ 2750–8000 m), the dataset integrates more than 1500 h of lander video and 136 h of submersible video to document 325 morphotaxa across 16 phyla of organisms. This inventory substantially exceeds previous visual surveys of hadal features, which have typically focused on narrower depth ranges, fewer deployments or a single sampling method (Lemche et al. 1976, Jamieson et al. 2026).

This inventory is also important because the Nova Canton Trough is a hadal fracture-zone system rather than a subduction trench, providing an opportunity to examine hadal biodiversity in a different geological context and significantly deeper than in previous studies in non-subduction hadal features to date (e.g. Weston et al. 2020, Stewart et al. 2026). This different geological setting is, therefore, an important point of comparison for future studies to understand whether hadal faunal structure is tied to depth alone or to the geological and disturbance context in which hadal depths occur.

Further, the combined use of baited landers and submersible transects captured a broader range of the fauna than either sampling method alone. Although both methods use video imagery, they do not sample equivalent biodiversity components. Baited landers are strongly selective for mobile bait-attending organisms and, therefore, effective for documenting scavenging or predatory taxa attracted to the bait or to the organisms attracted to the bait. However, they provide limited information on sessile or weakly mobile benthic fauna unless these occur within the field of view of the landers. Submersible surveys, on the other hand, proved more effective for visible benthic and benthopelagic fauna, with 62% of observations only captured on submersible video data. This is partly attributable to the fact that submersible transects can traverse multiple substrate types within a single dive and survey different communities associated with these substrate types. The multi-platform approach, therefore, expands the taxonomic and ecological breadth of the inventory and formal quantitative analysis of the data by platform is planned for separate ecological analyses.

### Assemblage Structure across the Depth Gradient

Observations revealed marked faunal turnover across the depth gradient of the Nova Canton Trough, consistent with established knowledge that depth is the dominant structuring gradient of abyssal-hadal biota, although depth is inevitably correlated with other ecologically relevant parameters (Brandt et al. 2025, Swanborn et al. 2025b, Wainwright et al. 2026). Overall, the Nova Canton Trough fauna shows a depth-structured transition from a diverse abyssal community to a distinctive hadal community, with the strongest changes occurring across the abyssal-hadal transition zone.

The lower abyssal (~ 4500–6000 m) zone showed highest apparent morphotaxa richness, both across lander-based and submersible observations. One of the most conspicuous parts of the bait-attending fish fauna was the high diversity of ophidiiforms, which included multiple *Bassozetus* morphotypes and other cusk eels. Other fish characteristics of lower abyssal depths were macrourid *Coryphaenoides yaquinae*, one of the most frequently recorded fishes in the dataset and zoarcids (*Pachycara* sp. indet. 2). The bait-attending decapod community was also characteristically composed of taxa ubiquitously occurring in these depths in the Pacific and extending across broad depth ranges (acanthephyrids - *Hymenodora* sp. indet. 1 and *Heterogenys* sp. indet. 1 – as well as *Cerataspis monstrosus* and *Benthesicymus crenatus)* (Swan et al. 2021). Submersible transects documented a richer benthic megafauna, which likely also reflects increased availability of hard substratum in this part of the fracture zone (Riehl et al. 2020), providing attachment opportunities for sessile filter- and suspension-feeding fauna that contained many of the sponge, octocoral, antipatharian, brisingid, crinoid and holothuroid morphotypes. Sponges showed high diversity in abyssal rocky outcrops, with stalked, barrel and funnel forms dominant in this environment (Marchiò et al. 2025b), highlighting the importance of tall body plans for better access to nutrients in the water column (Schönberg 2021).

Substantial faunal turnover was observed across the abyssal-hadal transition zone (AHTZ, ~ 5500–6500 m), although several bait-attending taxa persisted across the AHTZ. For example, ophidiid and macrourid fishes and decapods declined or disappeared here, with the exception of *C. monstrosus*, *B. crenatus* and *Hymenopenaeus* spp. This mirrors well-established depth zonation documented across abyssal-hadal fish communities globally (Linley et al. 2017, Jamieson et al. 2021a, Swan et al. 2021, Swanborn et al. 2025b). Submersible deployments also showed a shift in benthic megafaunal diversity, with an assemblage composed of distinct actiniarian morphotypes, hyocrinids, brisingids and several holothuroid taxa on areas of gravel, nodules and mixed hard substrata interspersed with fine sediment. Sponge morphological diversity decreased, with funnel euretid sponges becoming less abundant, while barrel and stalked forms from the order Lyssacinosida predominated.

Below the transition zone, hadal depths (> ~ 6000–6500 m) supported distinct fauna associated largely with finer, soft sediment in the deepest part of the trough. Lander deployments revealed the first population of hadal snailfish (29 deployments, 6090–7985 m) reported outside of a subduction trench (Jamieson et al. 2021a). Their presence confirms that hadal habitat in other geomorphological settings provides suitable habitat for liparids. The taxonomic identification of these individuals remains uncertain (Maroni et al. 2025b); although currently listed as *Pseudoliparis* s.p. indet. 1, it could belong to either *Pseudoliparis* or *Notoliparis*. Hadal submersible transects also documented several megabenthic morphotypes which appeared largely restricted to hadal depths, but were highly abundant in that depth range and, therefore, better interpreted as recurrent components of the hadal benthic assemblage. Elpidiid holothuroids were amongst the most conspicuous components and included several *Elpidia* morphotypes that could be highly abundant (numbers in the thousands) in the deepest part of the fracture zone, as well as two *Peniagone* morphotypes (1 and 7) of which > 1500 individuals were observed. Similar patterns were evident for Anthozoa cls. indet. 1 and several hadal actiniarians, as well as nemerteans and *Hymenaster* asteroids. Xenophyophores were also abundant and morphologically diverse, particularly in the deepest flat-bottomed plain of the trough axis.

Several high-level morphotaxa were also recorded across very broad depth ranges throughout the dataset, including mysids, munnopsids, copepods, chaetognaths, annelids, ophiuroids and appendicularians. These broad ranges should be interpreted cautiously, as many of these categories likely reflect image identification limitations and may, therefore, pool multiple taxa with differing depth ranges. Nevertheless, their repeated occurrence across the gradient is important for understanding the distribution of these higher-level faunal components.

### Patchiness and Local Abundance

Although the overall fauna was strongly depth-structured, the inventory also showed substantial patchiness in faunal distribution across deployments and in abundance. Several morphotaxa showed high local abundance despite restricted occurrence across deployments, suggesting local environmental conditions and biological interactions were particularly suitable for those taxa (McClain and Hardy 2010). The most striking examples were Hesionidae gen. indet. 1 and Onuphidae spp. reaching 988 and 210 observations, respectively, in the same dive around 6700 m. Such localised peaks in abundance are common in the deep sea, where habitat heterogeneity and bottom-up food supply can create small areas of unusually high faunal density against a background of low occurrence (Jumars and Eckman 1983). Several attached suspension feeders also showed restricted, but locally high abundance, including seapens *Calibelemnon* sp. indet. 1 (331 individuals, + associated Ophiacanthidae ophiuroids) and *Umbellula* sp. indet. 1 at 3200–4700 m. Several actiniarians morphotypes and hyocrinids were also found in high abundances across the abyssal-hadal transition zone ~ 6000 m. Around this depth, high abundances of putative rock-associated Platyhelminthes were also found. The presence of flat worms (Platyhelminthes) is plausible as they are known from hadal depths in the Kuile Kamchatka, Peru-Chile and Aleutian trenches (Beliaev 1989), with maximum depths of 9335, 6354 and 7298 m, respectively (Polycladida order). More recently in the Kuril-Kamchatka Trench, flat worm egg capsules from unknown species in the Tricladida order have been recovered from 6200 m (Kakui and Tsuyuki 2024). The identity of these organisms is, however, unresolved, as is their abundance within rugose substrata of the upper hadal zone in this region.

Rarity is a common trait amongst deep-sea life (McClain 2021) and other examples of patchiness were evident amongst ‘rare’ taxa that occurred only at low abundances in one or a few deployments. This was especially evident amongst sessile taxa, such as hexactinellid sponges, actiniarians and other cnidarians and crinoids, which can display considerable habitat specificity (Dijkstra et al. 2021). Patchy occurrence and distribution patterns were pronounced at ~ 4600–4700 m and ~ 6000 m where replicate dives in similar depth bands shared only a subset of morphotaxa. Some of this ‘rarity’ likely reflects real ecological heterogeneity and habitat-related hypotheses that require formal testing. Some patterns appear to be mediated by seafloor character and the patchy distribution of hard substratum in this depth range, suggesting that local habitat conditions may structure biodiversity strongly (e.g. Riehl et al. 2020, Mejía-Saenz et al. 2023), but it may also reflect diagnostic limitations of image-based identifications.

Whilst identifications reported here have been corroborated by taxonomists where possible, they have not been confirmed by physical samples. Groups with complex taxonomy, internal diagnostic characteristics and high morphological plasticity have recognised taxonomic identification challenges, as do small, highly abundant or burrowing organisms (Hanafi-Portier et al. 2021). Some apparent patchiness may, therefore, reflect taxonomic splitting on morphological plasticity or image quality variation. Vice versa, although several hadal species are known to have wide distributions (e.g. Weston and Jamieson 2022, Maroni et al. 2025a, Maroni et al. 2025b), some apparently broad distributions may, in fact, represent multiple cryptic species with narrower ecological ranges and so patterns discussed here are best interpreted as patterns in visual morphological diversity.

Patchiness was also evident in the distribution of lebensspuren. Some traces appeared widespread (e.g. holothurian surface traces), whilst others were restricted to certain dives and/or depth ranges. The putative whale-feeding marks are one of the more significant examples of localised lebensspuren and show striking similarities to those of Woodside et al. (2006) who described depressions to be 1–2 m long, 50 cm wide and ~ 10 cm deep with a central groove and raised rims to either side of the central groove. Auster and Watling (2009) described a similar morphology with the depressions being 0.5–1 m long and 20–40 cm wide, with a central linear depression up to ~ 10 cm deep with one end narrow and the other end a wider ovoid with a ridge of clumped mud. In the absence of direct observations of whales interacting with the deep seafloor, the exact process forming these marks is still speculative. Purser et al. (2019) detailed why all other known species from these depths are not large or powerful enough to create these marks. All previous reports of such marks eventually conclude that they are likely caused by deep-diving beaked whales, in most instances, Cuvier’s beaked whale. Of all beaked whales, only Cuvier’s beaked whale and Blainville’s beaked whale span the geographical range of these observations combined (MacLeod et al. 2006). The depth record for a Cuvier’s Beaked whale dive is 2992 m (Schorr et al. 2014).

### Notable Records, Range Extensions and Absences

Several records from the Nova Canton Trough appear unusual when compared with published records and global occurrence datasets and may represent depth extensions for several taxonomic groups, summarised in Table 2 or notable diversity.

#### Phylum

The deepest known species of Brachiopoda is *Pelagodiscus atlanticus* (King, 1868) (family Discinidae, class Lingulata) which are known from bathyal and abyssal depths to 5530 m in the northern Pacific Ocean (Beliaev 1989). However, only empty brachiopod shell cusps have ever been collected from hadal depths of down to 7600 m; therefore, no live Brachiopoda. Lemche et al. (1976), however, photographed Brachiopoda 6758–6776 m that were thought to belong to the family Phaneroporidae, though it is unclear whether these were alive or not. The Brachiopoda in this study (cf. Rhynchonellata) were seen at a depth of 5586–6671 m. Although some brachiopods were observed lying on sediment, most brachiopods observed in this study were seen attached to the edges of rocks and nodules and did not give the appearance of being empty shell cusps.

Nemerteans (ribbon worms) were recorded across 34 unique deployments between 5400 m and 8000 m. This observation warrants comment given the limited knowledge about this phylum at depth. Nemerteans have long been considered rare or absent from abyssal and hadal environments, a view only recently understood to reflect the difficulty of sampling this group rather than their true absence (Chernyshev and Polyakova 2019). For example, Beliaev (1989) reported only sampling fragments of Nemerteans below 6000 m. The first hadal nemertean was only described recently (Chernyshev and Polyakova 2018) and visual records from abyssal and hadal depths remain few (Jamieson et al. 2026). The consistency of nemertean occurrence across deployments, therefore, adds important support to the view that they are a recurrent, albeit under-sampled component of abyssal and hadal assemblages and highlights specimen collection as a priority for future work.

The protozoan phylum Cercozoa, including colonial aggregates in the water column tentatively identified as *Tuscaridium* to 7987 m, forms a notable component of the inventory. The group has so far been documented at hadal depths only through molecular proxies, such as eDNA derived from water (Xu et al. 2018).

#### Class

At class level, this study extends the known depth range of the Appendicularia to 7218 m from the previously record of 7176 m (Jamieson and Linley 2021), albeit by less than 50 m. However, the previous depth record was from the maximum sampling depth of the Java Trench (Jamieson et al. 2022), whereas in this study, their maximum depth is ~ 800 m shallower than the maximum sampling depth of the Nova Canton Trough, suggesting that ~ 7200 m could represent a true depth limit for the class rather than a sampling artefact.

Asteroidea are rarely reported from > 7500 m (Beliaev 1989, Jamieson 2015), but valvatid asteroids were a consistent part of the hadal benthic megafaunal community occurring across multiple dives to nearly 8000 m. This places the Nova Canton benthic observations amongst the deepest documented records of asteroids and indicates that asteroids can be a substantial component of hadal benthic assemblages in certain settings.

Glass sponges are rarely observed beyond abyssal depths, but in the Nova Canton Trough, three morphotypes of lyssacinosid sponges were observed deeper than 6500 m on soft sediment-dominated areas, exhibiting mostly nest morphologies; these observations represent some of the deepest known for the class (Marchiò et al. 2025b).

#### Order

At order level, this study also extends the known depth range of Narcomedusae from 6777 m (Jamieson and Linley 2021), to 7989 m (an increase of 1212 m). Likewise, the order Corallimorpharia (Hexacorallia, Corallimorpharia fam. indet. 1) is extended by 1362 m from 5320 m (Eash-Loucks and Fautin 2012) to 6682 m, representing the first record of the order at hadal depths.

Ctenophores are not a group typically considered common at hadal depths. To date, three Cydippida morphotypes are known from the trenches around Japan (Lindsay and Miyake 2007, Jamieson et al. 2026), Yap (Zhang et al. 2021) and one individual from over 10,000 m in the Kermadec Trench (Jamieson et al. 2023). Whilst those in this study do not represent a depth extension of the order, they exhibit unusually high diversity, matching the total number of morphotypes known from all other hadal records combined.

#### Family

Notable extensions at family level are those of the amphipod family Amathillopsidae, which were recorded here at 6228 m, clinging, in pairs, to other organisms such as crinoids. While this epibiotic behaviour is known in the family, this record extends their depth range by 669 m (Lörz and Horton 2021), representing the first hadal record for the family (Jamieson and Weston 2023).

In the octocoral family Umbellulidae, the observation of*Umbellula* sp. indet. 2 at 6949 m (superfamily Pennatuloidea) is also a notable extension, being ~ 600 m deeper than the deepest Umbellulidae occurrence currently returned by OBIS (6364 m; NOAA DSCRTP, 2026) and exceeding the sea pen depth range of 6100 m reported in literature (Williams 20111) by ~ 800 m. However, OBIS also includes an *Acanthoptilum* sp. specimen (Virgulariidae) from 7956 m (NOAA DSCRTP 2026) and the Nova Canton Trough observation therefore represents a probable ~ 600 m depth extension for Umbellulidae, rather than a global depth record for Pennatuloidea.

The annelid family Onuphidae (quill worms) has been characterised as extending to hadal depths, though confirmed hadal records are few. The deepest previously recorded species is *Leptoecia ultraabyssalis*, known from 6290–6330 m (Kucheruk 1977). Observations here at 6651–6680 m extend the confirmed depth range of the family by approximately 320–390 m and the individuals observed were present in locally high abundances, suggesting onuphids can be prevalent components of hadal communities rather than isolated records. Specimen collection and identification would solidify the taxonomic identification of these individuals.

The vertical distribution of the annelid family Hesionidae is not well-recorded in literature, but the deepest previously recorded occurrence of the family on OBIS is a specimen from 5325–5506 m (Earth Sciences New Zealand 2025). The Nova Canton Trough observations at 6700 m extends the known depth range of the family by approximately 1200 m and represent the first record at hadal depth. The high local abundance of hesionid individuals is particularly notable and specimen collection would be of considerable taxonomic and ecological interest, serving to confirm and characterise the family’s presence at hadal depths.

The Polynoidae subfamily Admetellinae has a known depth range of 3968–6860 m (Beliaev 1972, Gonzalez et al. 2025). Most knowledge of this group comes, however, from sampling grabs and trawls. While the Nova Canton Trough observations are not a depth record, they likely represent the deepest visual record for the group.

Tubed annelids were observed between 3208 m and 7978 m, with shallower observations putatively attributed to Sabellida (Sabellida fam. indet; Fig. 3M), while those at greater depths likely to belong to either Sabellida or Terebellida, specifically Melinnidae. While sabellids are known from far greater depths than those recorded in the Nova Canton Trough, current depth records for Melinnidae on OBIS show no samples below 6000 m. Specimen collection and identification would not only solidify the taxonomic placement of these curved tubed individuals, but would also likely represent a marked depth extension if they belong to Melinnidae.

The hydrozoan family Candelabridae was recorded at 6341–6876 m, substantially extending the known depth range for the family. Prior to this study, the deepest confirmed records for the family were from 4277–4278 m (Rybakova et al. 2020), making the Nova Canton Trough observation a potential extension of 2596 m and the first record of the family at hadal depths. Specimen confirmation would be needed to substantiate this record.

Ulmarid scyphozoans are also not commonly recorded at hadal depths; however, Ulmaridae gen. indet. 1 was particularly prominent at 8000 m (> 300 individuals) and had a near-hadal depth range (min depth = 5921 m). To date they have been recorded twice, once between 7848 m and 8662 m and again between 7984 m and 8228 m from either end of the adjoining New Britain and Bougainville trenches (Lemche et al. 1976, Gallo et al. 2015). However, those individuals were free-swimming.

#### Genus

The presence of the genus *Abyssopathes* (Hexacorallia) at 6018 m also ranks amongst the deepest records for the genus. The deepest published record is 5772 m (Molodtsova et al. 2023), although there are specimens from 6254 m listed in OBIS from the Tonga Trench.

#### Notable absences

Wainwright et al. (2026) included baited invertebrate traps on 102 baited lander deployments at ~ 100 m depth intervals on the same expedition. They recovered a total of 139,271 amphipod specimens that comprised 26 species identified using integrative taxonomy. Although most amphipods are not readily identifiable from images, there are several species that can be identified and, therefore, the absence of certain species was noticeable. For example, the ‘supergiant amphipod’ *Alicella gigantea* Chevreux, 1899, is the largest known amphipod (Jamieson et al. 2013) and has a depth range of 3890–8931 m in the Pacific, indicating that it may occupy around 59% of the world’s oceans (Maroni et al. 2025a). However, although it is known from 75 sites around the world, including the central, north and south Pacific, it was not detected in this study in either images or traps. The reasons for this remain obscure as the sampling effort in this study was considerably higher than in previous studies that record *A. gigantea* (Jamieson et al. 2013, Swanborn et al. 2025b).

Likewise, the predatory amphipod genus *Princaxelia* (Family Pardaliscidae) are often recovered in traps and readily identifiable in images due to their unique body form compared to scavenging lysianassoid amphipods (Jamieson et al. 2011). *Princaxelia* sp. are almost exclusively hadal endemics and are known from trenches directly north and south of the Nova Canton Trough with minimum depth ranges that overlap with the depths of this study (Kamenskaya 1995, Lörz 2010). Their apparent absence from the Nova Canton Trough remains unresolved.

#### Natural and unnatural debris

The submersible survey, based on 50.9 on-bottom transect hours, that produced 152.9 h of HD video data of the seafloor from 3273 m to 8000 m only encountered three items of anthropogenic debris which is encouraging low compared to other hadal features (Shimanaga and Yanagi 2016, Jamieson and Onda 2022, Abel et al. 2023). This is likely a reflection of how remote the Nova Canton Trough is to major industrial land masses. Similarly, the number of wood and plant debris was only two, which again suggests its remoteness is excluding transport from major coastlines, unlike other deep regions (Wolff 1976).

## Conclusions

The Nova Canton Trough inventory has broader coverage and resolution than previous hadal visual biodiversity studies, combining a broad depth range surveyed at high resolution using complementary platforms in a non-subduction hadal feature. The scale and resolution of the dataset help disentangle sampling effort from distribution patterns, as depth ranges and apparent rarities or absences previously established from single deployments or narrow sampling windows can now be evaluated against substantially greater effort than most prior hadal surveys. The survey revealed the Nova Canton Trough hosts both recognisable components of global hadal fauna and distinctive local assemblages, range extensions and absences.

At the same time, the inventory highlights the need for specimen collection and molecular work. Although image-based inventories are powerful for broad biodiversity baselines, they cannot resolve taxonomic uncertainty, cryptic diversity and potential pooling of taxa that likely underlie some of the patterns discussed. The inventory, therefore, also serves as a baseline for targeted specimen collection, particularly of range-extending and taxonomically uncertain taxa and for the continued development of standardised morphotaxon frameworks to enable cross-feature and platform comparisons.

Overall, this study highlights the value – and current scarcity – of high-resolution visual surveys across full depth gradients in hadal environments and makes the Nova Canton Trough, both as a hadal system and a geomorphologically distinct feature, an important reference point against which future surveys of hadal features can be compared.

## Supplementary Material

B4545FA0-150E-5F66-81C3-7368711D7320 10.3897/BDJ.14.e204436.suppl1 Supplementary material 1 Metadata of lander and submersible deployments included in this study Data type Deployment metadata File: oo_1673056.xlsx https://binary.pensoft.net/file/1673056 Denise J.B. Swanborn; Alan J. Jamieson

06646ED2-A575-52A0-86D0-80A53B9953EF 10.3897/BDJ.14.e204436.suppl2 Supplementary material 2 Nova Canton morphotaxa guide Data type Descriptions and imagery Brief description This document contains additional imagery of all morphotaxa observed in the Nova Canton Trough, along with identification notes. File: oo_1746328.pdf https://binary.pensoft.net/file/1746328 Denise J.B. Swanborn, Alfredo Marchiò, Giulia La Bianca, Megan Cundy, Travis Reynolds Jr, Melanie S. Stott, Alan J. Jamieson

## Figures and Tables

**Table 1.** Morphotaxa and depth ranges recorded from lander and submersible imagery across the Nova Canton Trough. For each morphotaxon, the table provides the minimum and maximum observed depths, total depth range, number of deployments in which the morphotaxon was recorded, platform type and associated figure panel where applicable

**Morphotaxon**	**Minimum Depth**	**Maximum Depth**	**Depth Range**	**No. Deployments**	**Platform type**	**Figure**
Kingdom Animalia						
Annelida - Annelida spp. 1	2771	7979	5208	37	both	Fig. 3A
Annelida - Annelida spp. 2	2771	7987	5216	67	both	Fig. 3B
Annelida - Echiuroidea - Echiuroidea fam. indet. 1	6592	7987	1395	10	both	Fig. 3D
Annelida - Echiuroidea - Echiuroidea fam. indet. 2	7309	7985	676	1	submersible	Fig. 3E
Annelida - Echiuroidea - Echiuroidea fam. indet. 3	4337	6006	1669	2	lander	Fig. 3F
Annelida - Echiuroidea - Echiuroidea fam. indet. 4	6673	6673	0	1	submersible	Fig. 3G
Annelida - Echiuroidea - Echiuroidea fam. indet. 5	7369	7369	0	1	iphone	Fig. 3H
Annelida - Echiuroidea - Echiuroidea spp.	5186	7987	2801	7	both	Fig. 3C
Annelida - Eunicida - Onuphidae - Onuphidae gen. indet.	6651	6680	28	1	submersible	Fig. 3Q
Annelida - Phyllodocia - Hesionidae - Hesionidae gen. indet. 1	6610	6683	73	1	submersible	Fig. 3I
Annelida - Phyllodocia - Polynoidae - Admetellinae gen. indet.	6663	6677	14	1	submersible	Fig. 3L
Annelida - Phyllodocia - Polynoidae - Macellicephalinae gen. indet. 1	7022	7989	967	2	both	Fig. 3K
Annelida - Phyllodocia - Polynoidae - Macellicephalinae spp.	5667	7384	1717	11	both	Fig. 3J
Annelida - Sabellida - Sabellida spp.	3208	7987	4779	8	both	Fig. 3M, N
Annelida - Sabellida - Sabellida fam. indet. 1	3800	3800	0	1	submersible	Fig. 3O
Annelida - Sabellida - Sabellida fam. indet. 2	3200	3800	6000	2	submersible	Fig. 3P
Annelida - Terebellida - Acrocirridae - Acrocirridae spp.	4095	7022	2927	32	both	Fig. 3R
Annelida - Terebellida - Flabelligeridae - Flabelligeridae spp.	3885	6972	3087	33	both	Fig. 3S
Arthropoda - Copepoda - Copepoda spp.	3208	7983	4775	48	both	Fig. 4A
Arthropoda - Malacostraca - Amphipoda - Amphipoda spp.	3219	7987	4768	19	submersible	Fig. 4B
Arthropoda - Malacostraca - Amphipoda - Amathillopsidae - Amathillopsidae gen. indet. 1	5629	6228	599	2	submersible	Fig. 4F
Arthropoda - Malacostraca - Cumacea - Cumacea spp.	5274	7022	1748	5	both	Fig. 4C
Arthropoda - Malacostraca - Decapoda - Decapoda spp.	3888	5836	1948	10	both	Fig. 4D
Arthropoda - Malacostraca - Decapoda - Decapoda ord. indet. 1	3888	5836	1948	8	lander	Fig. 4G
Arthropoda - Malacostraca - Decapoda - Acanthephyridae - *Hymenodora* sp. indet. 1	2751	5186	2435	34	lander	Fig. 4L
Arthropoda - Malacostraca - Decapoda - Acanthephyridae - *Hymenodora* sp. indet. 2	3387	3387	0	1	lander	Fig. 4M
Arthropoda - Malacostraca - Decapoda - Acanthephyridae - *Heterogenys* sp. indet. 1	3100	6226	3126	31	both	Fig. 4K
Arthropoda - Malacostraca - Decapoda - Acanthephyridae - Acanthephyridae gen. indet. 1	2751	4368	1617	21	lander	Fig. 4H
Arthropoda - Malacostraca - Decapoda - Acanthephyridae - Acanthephyridae gen. indet. 2	3712	6094	2382	15	lander	Fig. 4I
Arthropoda - Malacostraca - Decapoda - Acanthephyridae - Acanthephyridae gen. indet. 3	5023	6094	1071	4	lander	Fig. 4J
Arthropoda - Malacostraca - Decapoda - Aristeidae - *Cerataspis monstrosus*	2751	6673	3922	74	both	Fig. 4O
Arthropoda - Malacostraca - Decapoda - Benthesicymidae - *Benthesicymus crenatus*	3225	7470	4245	89	both	Fig. 4P
Arthropoda - Malacostraca - Decapoda - Benthesicymidae - Benthesicymidae gen. indet. 1	2751	2751	0	1	lander	Fig. 4N
Arthropoda - Malacostraca - Decapoda - Solenoceridae - *Hymenopenaeus* spp.	3387	6630	3243	19	both	Fig. 4Q
Arthropoda - Malacostraca - Decapoda - Munidopsidae - Munidopsidae spp.	2751	4342	1591	5	both	Fig. 4R
Arthropoda - Malacostraca - Decapoda - Munidopsidae - *Munidopsis* sp. indet 1	2751	4549	1798	4	lander	Fig. 4S
Arthropoda - Malacostraca - Decapoda - Paguroidea spp.	3238	3238	0	2	both	Fig. 4T
Arthropoda - Malacostraca - Decapoda - Parapaguridae - Parapaguridae spp.	3100	4675	1575	10	both	Fig. 4U
Arthropoda - Malacostraca - Isopoda - Isopoda spp.	5584	7704	2120	6	both	ns
Arthropoda - Malacostraca - Isopoda - Ischnomesidae - Ischnomesidae spp.	7989	7989	0	1	submersible	ns
Arthropoda - Malacostraca - Isopoda - Ischnomesidae - Ischnomesidae gen. indet. 1	7200	7987	787	13	both	Fig. 4Y
Arthropoda - Malacostraca - Isopoda - Ischnomesidae - Ischnomesidae gen. indet. 2	5492	6299	807	10	lander	Fig. 4Z
Arthropoda - Malacostraca - Isopoda - Munnopsidae - Munnopsidae spp.	2751	7987	5236	64	both	ns
Arthropoda - Malacostraca - Isopoda - Munnopsidae - *Munneurycope* sp. indet. 1	5667	6739	1072	10	both	Fig. 4V, W
Arthropoda - Malacostraca - Isopoda - Munnopsidae - Storthyngurinae gen. indet.	6299	6673	375	8	lander	Fig. 4X
Arthropoda - Malacostraca - Mysida - Mysidae - Mysidae spp.	2751	7798	5047	92	both	Fig. 4E
Arthropoda - Thecostraca - Scalpellomorpha - Scalpellomorpha spp.	4598	4598	0	1	submersible	Fig. 4Z
Arthropoda - Thecostraca - Scalpellomorpha - *Alcockianum* sp. indet. 1	3690	4614	924	2	both	Fig. 4AA
Chordata - Appendicularia - Copelata - Copelata spp.	2771	7318	4547	39	both	Fig. 5A
Chordata - Appendicularia - Copelata - Copelata fam. indet. 1	5023	5790	767	9	lander	Fig. 5C
Chordata - Appendicularia - Copelata - Copelata fam. indet. 2	4980	6592	1612	13	both	Fig. 5B
Chordata - Ascidiacea - Phlebobranchia - Octacnemidae - *Megalodicopia* sp. indet. 1	5580	7025	1445	3	submersible	Fig. 5FG
Chordata - Ascidiacea - Phlebobranchia - Octacnemidae - *Megalodicopia* sp. indet. 2	4011	5287	1276	3	submersible	Fig. 5H
Chordata - Ascidiacea - Phlebobranchia - Octacnemidae - *Megalodicopia* sp. indet. 3	5496	6969	1474	5	submersible	Fig. 5I
Chordata - Ascidiacea - Phlebobranchia - Octacnemidae - Octacnemidae spp.	4635	6873	2238	2	submersible	ns
Chordata - Ascidiacea - Phlebobranchia - Octacnemidae - Octacnemidae gen. indet. 1	5594	7161	1568	3	submersible	Fig. 5D
Chordata - Ascidiacea - Phlebobranchia - Octacnemidae - Octacnemidae gen. indet. 2	7172	7172	0	1	submersible	Fig. 5E
Chordata - Teleostei - Anguilliformes - Synaphobranchidae - Synaphobranchidae spp.	3236	3236	0	25	submersible	ns
Chordata - Teleostei - Anguilliformes - Synaphobranchidae - *Ilyophis arx*	2751	4095	1344	16	lander	Fig. 6A
Chordata - Teleostei - Anguilliformes - Synaphobranchidae - *Ilyophis robinsae*	3885	5612	1727	9	lander	Fig. 6B
Chordata - Teleostei - Anguilliformes - Synaphobranchidae - *Ilyophis* sp. indet. 1	2751	3519	768	4	lander	Fig. 6C
Chordata - Teleostei - Ateleopodiformes - Ateleopodidae - *Ateleopus* sp. indet. 1	4488	5696	1208	3	both	Fig. 6D
Chordata - Teleostei - Aulopiformes - Bathysauridae - *Bathysaurus mollis*	3803	4555	752	4	both	Fig. 6E
Chordata - Teleostei - Aulopiformes - Ipnopidae - *Bathypterois atricolor*	3219	3219	0	1	submersible	Fig. 6F
Chordata - Teleostei - Aulopiformes - Ipnopidae - *Bathytyphlops marionae*	3799	3799	0	1	submersible	Fig. 6G
Chordata - Teleostei - Aulopiformes - Ipnopidae - *Ipnops* sp. indet.	3877	4560	682	3	submersible	Fig. 6H
Chordata - Teleostei - Beryciformes - Stephanoberycidae - *Abyssoberyx* sp. indet. 1	4506	5686	1180	6	both	Fig. 6I
Chordata - Teleostei - Gadiformes - Macrouridae - Macrouridae spp.	3387	3387	0	1	lander	ns
Chordata - Teleostei - Gadiformes - Macrouridae - *Coryphaenoides rudis*	2771	2771	0	1	lander	Fig. 6J
Chordata - Teleostei - Gadiformes - Macrouridae - *Coryphaenoides yaquinae*	3233	6673	3440	65	both	Fig. 6K
Chordata - Teleostei - Ophidiiformes - Ophidiidae - *Abyssobrotula* sp. indet. 1	3780	3892	112	3	lander	Fig. 7A
Chordata - Teleostei - Ophidiiformes - Ophidiidae - *Acanthonus armatus*	2751	2771	20	2	lander	Fig. 7B
Chordata - Teleostei - Ophidiiformes - Ophidiidae - *Barathrites iris*	4469	5974	1505	15	lander	Fig. 7C
Chordata - Teleostei - Ophidiiformes - Ophidiidae - *Bassogigas walkeri*	2751	3494	743	5	lander	Fig. 7D
Chordata - Teleostei - Ophidiiformes - Ophidiidae - *Bassozetus* spp.	4013	5552	1539	5	both	Fig. 7L
Chordata - Teleostei - Ophidiiformes - Ophidiidae - *Bassozetus* sp. indet. 1	2751	6480	3729	43	lander	Fig. 7E
Chordata - Teleostei - Ophidiiformes - Ophidiidae - *Bassozetus* sp. indet. 2	4186	6299	2113	33	lander	Fig. 7F
Chordata - Teleostei - Ophidiiformes - Ophidiidae - *Bassozetus* sp. indet. 3	5272	6299	1027	17	lander	Fig. 7G
Chordata - Teleostei - Ophidiiformes - Ophidiidae - *Bassozetus* sp. indet. 4	5612	5612	0	1	lander	Fig. 7H
Chordata - Teleostei - Ophidiiformes - Ophidiidae - *Bassozetus* sp. indet. 6	3873	6090	2217	33	both	Fig. 7I
Chordata - Teleostei - Ophidiiformes - Ophidiidae - *Bassozetus* sp. indet. 7	2751	5673	2922	17	lander	Fig. 7J
Chordata - Teleostei - Ophidiiformes - Ophidiidae - *Bassozetus* sp. indet. 8	4472	6223	1751	2	both	Fig. 7K
Chordata - Teleostei - Ophidiiformes - Ophidiidae - *Leucicorus* spp.	4126	6008	1881	6	submersible	ns
Chordata - Teleostei - Ophidiiformes - Ophidiidae - *Leucicorus* sp. indet. 1	4704	5809	1105	14	both	Fig. 7M
Chordata - Teleostei - Ophidiiformes - Ophidiidae - Ophidiidae spp.	3918	5727	1809	3	submersible	ns
Chordata - Teleostei - Ophidiiformes - Ophidiidae - Ophidiidae gen. indet. 1	3780	5854	2074	12	lander	Fig. 7N
Chordata - Teleostei - Ophidiiformes - Ophidiidae - Ophidiidae gen. indet. 2	4675	4675	0	1	submersible	Fig. 7O
Chordata - Teleostei - Ophidiiformes - Ophidiidae - *Porogadus* spp.	4345	5079	734	4	submersible	ns
Chordata - Teleostei - Ophidiiformes - Ophidiidae - *Porogadus* sp. indet. 1	3791	5609	1818	2	lander	Fig. 7P
Chordata - Teleostei - Perciformes - Liparidae - *Pseudoliparis* sp. indet. 1	6090	7925	1835	29	both	Fig. 6L
Chordata - Teleostei - Perciformes - Zoarcidae - *Pachycara* spp.	4546	4546	0	1	lander	Fig. 6O
Chordata - Teleostei - Perciformes - Zoarcidae - *Pachycara* sp. indet. 1	3519	3791	272	2	lander	Fig. 6M
Chordata - Teleostei - Perciformes - Zoarcidae - *Pachycara* sp. indet. 2	5383	5974	591	2	lander	ns
Cnidaria - Anthozoa - Anthozoa spp.	3237	7986	4749	5	submersible	ns
Cnidaria - Anthozoa - Anthozoa ord. indet. 1	7939	7987	48	2	submersible	Fig. 8A
Cnidaria - Anthozoa - Anthozoa ord. indet. 2	5653	7987	2334	4	submersible	Fig. 8B
Cnidaria - Hexacorallia - Antipatharia - Antipatharia spp.	4013	5234	1221	2	submersible	ns
Cnidaria - Hexacorallia - Antipatharia - Antipatharia fam. indet. 1	3863	3889	25	1	submersible	Fig. 8J
Cnidaria - Hexacorallia - Antipatharia - Cladopathidae - *Abyssopathes* sp. indet. 1	5962	6018	56	1	submersible	Fig. 8N
Cnidaria - Hexacorallia - Antipatharia - Schizopathidae - *Bathypathes* sp. indet. 1	3827	4533	706	2	submersible	Fig. 8O
Cnidaria - Hexacorallia - Antipatharia - Schizopathidae - Schizopathidae gen. indet. 1	4634	4634	0	1	submersible	Fig. 8K
Cnidaria - Hexacorallia - Antipatharia - Schizopathidae - Schizopathidae gen. indet. 2	4031	5293	1261	3	submersible	Fig. 8L
Cnidaria - Hexacorallia - Antipatharia - Schizopathidae - Schizopathidae gen. indet. 3	4441	4635	194	3	submersible	Fig. 8M
Cnidaria - Hexacorallia - Zoantharia - Epizoanthidae - *Epizoanthus* sp. indet. 1	3100	3892	792	7	both	Fig. 8G
Cnidaria - Hexacorallia - Zoantharia - Epizoanthidae - *Epizoanthus* sp. indet. 2	4546	4546	0	1	lander	Fig. 8H
Cnidaria - Hexacorallia - Zoantharia - Zoantharia gen. indet. 1	3273	3273	0	1	submersible	Fig. 8I
Cnidaria - Hexacorallia - Actiniaria - Actiniaria spp.	4026	7983	3957	10	both	ns
Cnidaria - Hexacorallia - Actiniaria - Actiniaria fam. indet. 1	7325	7473	148	2	submersible	Fig. 9A
Cnidaria - Hexacorallia - Actiniaria - Actiniaria fam. indet. 2	4586	5269	683	3	submersible	Fig. 9B
Cnidaria - Hexacorallia - Actiniaria - Actiniaria fam. indet. 3	6654	6970	316	2	submersible	Fig. 9C
Cnidaria - Hexacorallia - Actiniaria - Actiniaria fam. indet. 4	3240	3240	0	1	submersible	Fig. 9D
Cnidaria - Hexacorallia - Actiniaria - Actiniaria fam. indet. 5	3219	3219	0	1	submersible	Fig. 9E
Cnidaria - Hexacorallia - Actiniaria - Actiniaria fam. indet. 6	5493	6017	524	2	submersible	Fig. 9F
Cnidaria - Hexacorallia - Actiniaria - Actiniaria fam. indet. 7	4013	5270	1257	4	submersible	Fig. 9G
Cnidaria - Hexacorallia - Actiniaria - Actiniaria fam. indet. 8	3818	5402	1584	4	submersible	Fig. 9H
Cnidaria - Hexacorallia - Actiniaria - Actiniaria fam. indet. 9	4078	6227	2149	4	submersible	Fig. 9I
Cnidaria - Hexacorallia - Actiniaria - Actiniaria fam. indet. 10	5948	6018	70	1	submersible	Fig. 9J
Cnidaria - Hexacorallia - Actiniaria - Actiniaria fam. indet. 11	7218	7984	766	3	submersible	Fig. 9K
Cnidaria - Hexacorallia - Actiniaria - Actiniaria fam. indet. 12	5512	7939	2428	5	submersible	Fig. 9L
Cnidaria - Hexacorallia - Actiniaria - Actiniaria fam. indet. 13	5684	6679	995	3	submersible	Fig. 9M
Cnidaria - Hexacorallia - Actiniaria - Actinoscyphidae - Actinoscyphidae gen. indet. 1	4603	4603	0	1	submersible	Fig. 9N
Cnidaria - Hexacorallia - Actiniaria - Actinoscyphidae - Actinoscyphidae gen. indet. 2	4529	4630	100	3	submersible	Fig. 9O
Cnidaria - Hexacorallia - Actiniaria - Galatheanthemidae - *Galatheanthemum* sp. indet. 1	6943	7986	1043	2	submersible	Fig. 9P
Cnidaria - Hexacorallia - Actiniaria - Relicanthidae - *Relicanthus* sp. indet. 1	4031	4635	603	3	submersible	Fig. 9Q
Cnidaria - Hexacorallia - Ceriantharia - Ceriantharia fam. indet. 1	3219	3803	584	2	submersible	Fig. 8C
Cnidaria - Hexacorallia - Ceriantharia - Ceriantharia fam. indet. 2	4665	4665	0	1	submersible	Fig. 8D
Cnidaria - Hexacorallia - Corallimorpharia - Corallimorphidae - Corallimorphidae gen. indet. 1	6480	6669	189	1	both	Fig. 8E
Cnidaria - Hexacorallia - Corallimorpharia - Corallimorphidae - Corallimorphidae gen. inc. 2	4336	5925	1589	3	submersible	Fig. 8F
Cnidaria - Octocorallia - Octocorallia spp.	3100	4755	1655	6	both	ns
Cnidaria - Octocorallia - Octocorallia ord. indet. 1	4063	5981	1918	5	submersible	Fig. 10A
Cnidaria - Octocorallia - Octocorallia ord. indet. 2	4026	4757	731	4	both	Fig. 10B
Cnidaria - Octocorallia - Octocorallia ord. indet. 3	4274	4407	133	2	submersible	Fig. 10C
Cnidaria - Octocorallia - Octocorallia ord. indet. 4	4619	4619	0	1	submersible	Fig. 10D
Cnidaria - Octocorallia - Octocorallia ord. indet. 5	4592	4758	166	2	submersible	Fig. 10E
Cnidaria - Octocorallia - Scleralcyonaceae - Chrysogorgiidae - *Chrysogorgia* sp. indet. 1	4359	4359	0	1	submersible	Fig. 10F
Cnidaria - Octocorallia - Scleralcyonaceae - Kophobelemnidae - *Kophobelemnon* sp. indet. 1	3217	3800	583	2	submersible	Fig. 10G
Cnidaria - Octocorallia - Scleralcyonaceae - Scleroptilidae - *Calibelemnon* sp. indet. 1	3174	3803	629	2	submersible	Fig. 10H
Cnidaria - Octocorallia - Scleralcyonaceae - Scleroptilidae - *Scleroptilum* sp. indet. 1	3238	4633	1394	2	submersible	Fig. 10I
Cnidaria - Octocorallia - Scleralcyonaceae - Umbellulidae - *Umbellula* sp. indet. 1	3100	4337	1237	5	both	Fig. 10J, K
Cnidaria - Octocorallia - Scleralcyonaceae - Umbellulidae - *Umbellula* sp. indet. 2	6949	6949	0	1	submersible	Fig. 10L
Cnidaria - Hydrozoa - Hydrozoa spp.	3233	7925	4692	16	both	ns
Cnidaria - Hydrozoa - Hydrozoa ord. indet. 1	3799	3804	5	1	submersible	Fig. 11A
Cnidaria - Hydrozoa - Hydrozoa ord. indet. 2	3791	3791	0	1	submersible	Fig. 11B
Cnidaria - Hydrozoa - Anthoathecata - Candelabridae - Candelabridae gen. indet. 1	6341	6876	535	2	submersible	Fig. 11C
Cnidaria - Hydrozoa - Anthoathecata - Corymorphidae - *Branchiocerianthus* sp. indet. 1	6792	7670	879	2	submersible	Fig. 11D
Cnidaria - Hydrozoa - Anthoathecata - Corymorphidae - *Branchiocerianthus* sp. indet. 2	6938	6938	0	1	submersible	Fig. 11E
Cnidaria - Hydrozoa - Leptothecata - Tiarannidae - Tiarannidae gen. indet. 1	5492	5492	0	1	lander	Fig. 11F
Cnidaria - Hydrozoa - Narcomedusae - Aeginidae - Aeginidae spp.	5492	7060	1568	2	both	ns
Cnidaria - Hydrozoa - Narcomedusae - Aeginidae - Aeginidae gen. indet. 1	5686	7987	2301	17	both	Fig. 11G
Cnidaria - Hydrozoa - Narcomedusae - Aeginidae - Aeginidae gen. indet. 2	6012	6012	0	1	submersible	Fig. 11H
Cnidaria - Hydrozoa - Narcomedusae - Narcomedusae spp.	3892	7966	4074	11	both	ns
Cnidaria - Hydrozoa - Narcomedusae - Cuninidae - Cuninidae spp.	4397	6226	1829	7	both	Fig. 11I
Cnidaria - Hydrozoa - Narcomedusae - Cuninidae - Cuninidae gen. indet. 1	4546	5848	1302	4	lander	Fig. 11J
Cnidaria - Hydrozoa - Siphonophorae - Siphonophorae fam. indet. 1	3100	3100	0	1	lander	Fig. 11K
Cnidaria - Hydrozoa - Trachymedusae - Trachymedusae spp.	4980	7987	3007	9	both	Fig. 11L
Cnidaria - Hydrozoa - Trachymedusae - Trachymedusae gen. indet. 1	4700	4700	0	1	submersible	Fig. 11M
Cnidaria - Hydrozoa - Trachymedusae - Rhopalonematidae - Rhopalonematidae spp.	3387	6672	3285	12	both	Fig. 11N
Cnidaria - Hydrozoa - Trachymedusae - Rhopalonematidae - *Arctapodema* sp. indet. 1	3791	5667	1876	4	lander	Fig. 11O
Cnidaria - Hydrozoa - Trachymedusae - Rhopalonematidae - Rhopalonematidae gen. indet. 1	4546	6299	1753	14	both	Fig. 11P
Cnidaria - Hydrozoa - Trachymedusae - Rhopalonematidae - Rhopalonematidae gen. indet. 2	6224	6229	5	1	submersible	Fig. 11Q
Cnidaria - Scyphozoa - Semaeostomeae - Ulmaridae - Ulmaridae spp.	6010	7987	1977	4	submersible	ns
Cnidaria - Scyphozoa - Semaeostomeae - Ulmaridae - Ulmaridae gen. indet. 1	5627	7987	2361	6	submersible	Fig. 11R
Cnidaria - Scyphozoa - Semaeostomeae - Ulmaridae - Ulmaridae gen. indet. 2	5340	7005	1665	3	both	Fig. 11S
Cnidaria - Scyphozoa - Semaeostomeae - Ulmaridae - Ulmaridae gen. indet. 3	2751	3237	486	2	both	Fig. 11T
Cnidaria - Scyphozoa - Semaeostomeae - Ulmaridae - Ulmaridae gen. indet. 4	3220	4518	1298	2	submersible	Fig. 11U
Ctenophora - Tentaculata - Cydippida - Cydippida spp.	3833	7987	4154	10	submersible	ns
Ctenophora - Tentaculata - Cydippida - Cydippida fam. indet. 1	6665	7484	819	2	submersible	Fig. 12A
Ctenophora - Tentaculata - Cydippida - Cydippida fam. indet. 2	6592	6975	383	4	both	Fig. 12B
Ctenophora - Tentaculata - Cydippida - Cydippida fam. indet. 3	6658	7022	364	3	both	Fig. 12C
Ctenophora - Tentaculata - Cydippida - Cydippida fam. indet. 4	6223	7135	912	5	both	Fig. 12D
Ctenophora - Tentaculata - Cydippida - Cydippida fam. indet. 5	2771	4587	1816	2	lander	Fig. 12E
Echinodermata - Echinoidea - Echinoidea spp.	3885	5609	1724	3	both	ns
Echinodermata - Echinoidea - Aspidodiadematoida - Aspidodiadematoida fam. indet. 1	3894	4507	612	3	both	Fig. 13A
Echinodermata - Echinoidea - Cidaroida - Histocidaridae - *Histocidaris* sp. indet. 1	3691	3804	113	2	both	Fig. 13B
Echinodermata - Asteroidea - Asteroidea spp.	3236	7141	3905	9	both	ns
Echinodermata - Asteroidea - Brisingida - Brisingida spp.	3984	4719	734	1	submersible	ns
Echinodermata - Asteroidea - Brisingida - Brisingida fam. inc. 1	4132	4635	504	3	submersible	Fig. 13C
Echinodermata - Asteroidea - Brisingida - Brisingida fam. inc. 2	4007	4635	628	3	submersible	Fig. 13D
Echinodermata - Asteroidea - Brisingida - Brisingida fam. inc. 3	5507	5935	427	2	submersible	Fig. 13E
Echinodermata - Asteroidea - Brisingida - Freyellidae spp.	4513	5932	1419	2	submersible	ns
Echinodermata - Asteroidea - Brisingida - Freyellidae gen. inc. 1	4641	6001	1360	2	submersible	Fig. 13F
Echinodermata - Asteroidea - Paxillosida - Porcellanasteridae - Porcellanasteridae spp.	3822	5591	1769	4	submersible	Fig. 13G
Echinodermata - Asteroidea - Paxillosida - Porcellanasteridae - Porcellanasteridae gen. inc. 1	5477	6952	1475	3	submersible	Fig. 13H, I
Echinodermata - Asteroidea - Paxillosida - Astropectinidae - Astropectinidae gen. inc. 1	3237	3237	0	1	submersible	Fig. 13J
Echinodermata - Asteroidea - Paxillosida - Astropectinidae - Astropectinidae gen. inc. 2	3741	4657	916	3	both	Fig. 13K
Echinodermata - Asteroidea - Valvatida - Goniasteridae - Goniasteridae gen. inc. 1	4634	4634	0	1	submersible	Fig. 13L
Echinodermata - Asteroidea - Valvatida - Goniasteridae - Goniasteridae gen. inc. 2	4413	4635	222	2	submersible	Fig. 13M
Echinodermata - Asteroidea - Valvatida - Goniasteridae - *Circeaster* sp. inc. 1	3237	3239	2	1	submersible	Fig. 13N
Echinodermata - Asteroidea - Valvatida - Solasteridae - *Lophaster* sp. indet. 1	3237	3239	2	1	submersible	Fig. 13O
Echinodermata - Asteroidea - Valvatida - Solasteridae - Solasteridae gen. indet. 1	3870	4052	182	2	submersible	Fig. 13P
Echinodermata - Asteroidea - Velatida - Pterasteridae - *Hymenaster* spp.	5288	7986	2699	5	submersible	ns
Echinodermata - Asteroidea - Velatida - Pterasteridae - *Hymenaster* sp. indet. 1	4388	5272	884	3	submersible	Fig. 13Q
Echinodermata - Asteroidea - Velatida - Pterasteridae - *Hymenaster* sp. indet. 2	6528	7987	1460	4	submersible	Fig. 13R, S, T
Echinodermata - Asteroidea - Velatida - Pterasteridae - *Hymenaster* sp. indet. 3	5818	6944	1126	2	submersible	Fig. 13U, V
Echinodermata - Holothuroidea - Holothuroidea spp.	4554	6950	2396	10	both	ns
Echinodermata - Holothuroidea - Elasipodida - Elpidiidae - Elpidiidae spp.	4313	7983	3670	30	both	ns
Echinodermata - Holothuroidea - Elasipodida - Elpidiidae - Elpidiidae gen. indet. 1	4104	4724	620	2	submersible	Fig. 14A
Echinodermata - Holothuroidea - Elasipodida - Elpidiidae - Elpidiidae gen. indet. 2	4613	4613	0	1	submersible	Fig. 14B
Echinodermata - Holothuroidea - Elasipodida - Elpidiidae - *Amperima* sp. indet. 1	5631	5902	271	1	submersible	Fig. 14C
Echinodermata - Holothuroidea - Elasipodida - Elpidiidae - *Amperima* sp. indet. 2	5248	5809	561	1	submersible	Fig. 14D
Echinodermata - Holothuroidea - Elasipodida - Elpidiidae - *Elpidia* spp.	5557	7987	2431	12	both	ns
Echinodermata - Holothuroidea - Elasipodida - Elpidiidae - *Elpidia* sp. indet. 1	5974	7532	1558	3	both	Fig. 14E
Echinodermata - Holothuroidea - Elasipodida - Elpidiidae - *Elpidia* sp. indet. 2	6094	7348	1254	5	both	Fig. 14F
Echinodermata - Holothuroidea - Elasipodida - Elpidiidae - *Elpidia* sp. indet. 3	7962	7987	25	2	submersible	Fig. 14G
Echinodermata - Holothuroidea - Elasipodida - Elpidiidae - *Elpidia* sp. indet. 4	7944	7987	44	3	submersible	Fig. 14H
Echinodermata - Holothuroidea - Elasipodida - Elpidiidae - *Elpidia* sp. indet. 5	7356	7987	631	3	submersible	Fig. 14I
Echinodermata - Holothuroidea - Elasipodida - Elpidiidae - *Peniagone* spp.	5636	7971	2334	6	both	ns
Echinodermata - Holothuroidea - Elasipodida - Elpidiidae - *Peniagone leander*	4980	6969	1989	14	both	Fig. 14J
Echinodermata - Holothuroidea - Elasipodida - Elpidiidae - *Peniagone* sp. indet. 1	6592	7987	1395	14	both	Fig. 14K
Echinodermata - Holothuroidea - Elasipodida - Elpidiidae - *Peniagone* sp. indet. 2	4213	4627	414	3	submersible	Fig. 14L
Echinodermata - Holothuroidea - Elasipodida - Elpidiidae - *Peniagone* sp. indet. 3	5538	5836	299	2	submersible	Fig. 14M
Echinodermata - Holothuroidea - Elasipodida - Elpidiidae - *Peniagone* sp. indet. 4	5124	5758	634	1	submersible	Fig. 14N
Echinodermata - Holothuroidea - Elasipodida - Elpidiidae - *Peniagone* sp. indet. 5	5762	5762	0	1	submersible	Fig. 14O
Echinodermata - Holothuroidea - Elasipodida - Elpidiidae - *Peniagone* sp. indet. 6	5566	5984	418	1	submersible	Fig. 14P
Echinodermata - Holothuroidea - Elasipodida - Elpidiidae - *Peniagone* sp. indet. 7	6503	7345	842	3	submersible	Fig. 14Q
Echinodermata - Holothuroidea - Elasipodida - Laetmogonidae - Laetmogonidae gen. indet. 1	5556	6221	665	2	submersible	Fig. 15A
Echinodermata - Holothuroidea - Elasipodida - Laetmogonidae - *Psychronaetes* sp. indet. 1	3760	3760	0	1	submersible	Fig. 15B
Echinodermata - Holothuroidea - Elasipodida - Laetmogonidae - *Psychronaetes hanseni*	4501	4670	168	2	submersible	Fig. 15C
Echinodermata - Holothuroidea - Elasipodida - Pelagothuriidae - *Enypniastes eximia*	4482	6299	1817	2	both	Fig. 15D
Echinodermata - Holothuroidea - Elasipodida - Psychropotidae - *Benthodytes* sp. indet. 1	4170	5304	1133	4	submersible	Fig. 15E
Echinodermata - Holothuroidea - Elasipodida - Psychropotidae - *Benthodytes* sp. indet. 2	4424	4754	330	2	submersible	Fig. 15F
Echinodermata - Holothuroidea - Elasipodida - Psychropotidae - *Benthodytes* sp. indet. 3	5461	6228	767	3	submersible	Fig. 15G
Echinodermata - Holothuroidea - Elasipodida - Psychropotidae - *Benthodytes* sp. indet. 4	4555	5990	1435	4	submersible	Fig. 15H
Echinodermata - Holothuroidea - Elasipodida - Psychropotidae - Psychropotidae gen. indet. 1	5273	5758	485	2	submersible	Fig. 15I
Echinodermata - Holothuroidea - Holothuriida - Mesothuriidae - *Mesothuria* sp. indet. 1	5264	5270	6	1	submersible	Fig. 15J
Echinodermata - Holothuroidea - Holothuriida - Mesothuriidae - *Mesothuria* sp. indet. 2	3233	6972	3739	5	submersible	Fig. 15K
Echinodermata - Holothuroidea - Holothuriida - Mesothuriidae - *Mesothuria* sp. indet. 3	4106	6645	2539	4	submersible	Fig. 15L
Echinodermata - Holothuroidea - Persiculida - *Benthothuria* sp. indet. 1	3782	4673	891	4	both	Fig. 15M
Echinodermata - Holothuroidea - Persiculida - Molpadiodemidae - *Molpadiodemas* sp. indet. 1	5645	6659	1014	3	submersible	Fig. 15N
Echinodermata - Holothuroidea - Persiculida - Molpadiodemidae - *Molpadiodemas* sp. indet. 2	6320	7961	1641	5	submersible	Fig. 15O
Echinodermata - Holothuroidea - Synallactida - Deimatidae - *Oneirophanta* sp. indet. 1	4304	4304	0	1	submersible	Fig. 15P
Echinodermata - Holothuroidea - Synallactida - Synallactidae - *Paelopatides* sp. indet. 1	4170	5749	1579	4	submersible	Fig. 15Q
Echinodermata - Holothuroidea - Synallactida - Synallactidae - *Paelopatides* sp. indet. 2	5270	6973	1704	6	submersible	Fig. 15R, S
Echinodermata - Holothuroidea - Synallactida - Synallactidae - Synallactidae gen. indet. 1	4435	4635	199	2	submersible	Fig. 15T
Echinodermata - Holothuroidea - Synallactida - Synallactidae - Synallactidae gen. indet. 2	4432	6004	1572	6	submersible	Fig. 15U
Echinodermata - Ophiuroidea - Ophiuroidea spp.	3208	7986	4778	55	both	Fig. 16K
Echinodermata - Ophiuroidea - Ophiuroidea ord. indet. 1	3387	5974	2587	2	lander	Fig. 16L
Echinodermata - Ophiuroidea - Ophiuroidea ord. indet. 2	4623	5270	647	2	submersible	Fig. 16M
Echinodermata - Ophiuroidea - Ophiuroidea ord. indet. 3	4600	4600	0	1	submersible	Fig. 16N
Echinodermata - Ophiuroidea - Ophiuroidea ord. indet. 4	3100	5271	2171	7	both	Fig. 16O
Echinodermata - Ophiuroidea - Ophiuroidea ord. indet. 5	5830	6678	848	3	submersible	Fig. 16P
Echinodermata - Ophiuroidea - Ophiuroidea ord. indet. 6	5594	7898	2304	6	submersible	Fig. 16Q
Echinodermata - Ophiuroidea - Ophiuroidea ord. indet. 7	3237	3803	566	2	submersible	Fig. 16R
Echinodermata - Ophiuroidea - Ophiuroidea ord. indet. 8	5482	7920	2437	8	both	Fig. 16S
Echinodermata - Ophiuroidea - Ophiuroidea ord. indet. 9	5264	6017	752	2	submersible	Fig. 16T
Echinodermata - Ophiuroidea - Ophiuroidea ord. indet. 10	3231	3239	7	1	submersible	Fig. 16U
Echinodermata - Ophiuroidea - Ophiurida - Ophiacanthidae - Ophiacanthidae gen. indet. 1	3100	3803	703	5	both	Fig. 16V
Echinodermata - Crinoidea - Crinoidea spp.	6222	6227	5	1	submersible	ns
Echinodermata - Crinoidea - Comatulida - Comatulida fam. indet. 1	3234	3691	457	2	submersible	Fig. 16A
Echinodermata - Crinoidea - Comatulida - Comatulida fam. indet. 2	4586	5265	679	2	submersible	Fig. 16B
Echinodermata - Crinoidea - Comatulida - Bathycrinidae - Bathycrinidae gen. indet. 1	4043	6228	2185	3	submersible	Fig. 16D, E
Echinodermata - Crinoidea - Comatulida - Bathycrinidae - Bathycrinidae gen. indet. 2	5262	7964	3322	5	submersible	Fig. 16F
Echinodermata - Crinoidea - Comatulida - Pentametrocrinidae - Pentametrocrinidae gen. indet. 1	5649	6222	573	2	submersible	Fig. 16C
Echinodermata - Crinoidea - Hyocrinida - Hyocrinidae - Hyocrinidae spp.	5683	7498	1815	4	submersible	ns
Echinodermata - Crinoidea - Hyocrinida - Hyocrinidae - Hyocrinidae gen. indet. 1	4397	5550	1153	3	both	Fig. 16G
Echinodermata - Crinoidea - Hyocrinida - Hyocrinidae - Hyocrinidae gen. indet. 2	6204	6228	24	1	submersible	Fig. 16H, I
Echinodermata - Crinoidea - Hyocrinida - Hyocrinidae - Hyocrinidae gen. indet. 3	5383	7496	2113	5	both	Fig. 16J
Porifera - Demospongiae - Demospongiae ord. inc. 1	5868	5868	0	1	submersible	Fig. 17A
Porifera - Demospongiae - Poecilosclerida - Cladorhizidae - *Chondrocladia* sp. indet. 1	4603	5292	689	2	submersible	Fig. 17B
Porifera - Demospongiae - Poecilosclerida - Cladorhizidae - *Chondrocladia* sp. indet. 2	5878	5988	110	1	submersible	Fig. 17C
Porifera - Demospongiae - Poecilosclerida - Cladorhizidae - Cladorhizidae gen. indet. 1	6658	6679	21	3	submersible	Fig. 17D
Porifera - Demospongiae - Poecilosclerida - Cladorhizidae - Cladorhizidae gen. indet. 2	5477	5975	498	2	submersible	Fig. 17E
Porifera - Demospongiae - Poecilosclerida - Cladorhizidae - Cladorhizidae gen. indet. 3	5420	6009	590	2	submersible	Fig. 17F
Porifera - Demospongiae - Poecilosclerida - Cladorhizidae - Cladorhizidae gen. indet. 4	5266	5266	0	1	submersible	Fig. 17G
Porifera - Demospongiae - Poecilosclerida - Cladorhizidae - Cladorhizidae spp.	4455	6672	2217	3	submersible	ns
Porifera - Hexactinellida - Hexactinellida ord. inc. 1	5278	5278	0	1	submersible	Fig. 18A
Porifera - Hexactinellida - Hexactinellida ord. inc. 2	5557	5557	0	1	submersible	Fig. 18B
Porifera - Hexactinellida - Hexactinellida ord. inc. 3	5702	5706	5	1	submersible	Fig. 18C
Porifera - Hexactinellida - Hexactinellida ord. inc. 4	5654	5693	39	1	submersible	Fig. 18D
Porifera - Hexactinellida - Hexactinellida ord. inc. 5	5434	5434	0	1	submersible	Fig. 18E
Porifera - Hexactinellida - Hexactinellida ord. inc. 6	4635	4656	20	2	submersible	Fig. 18F
Porifera - Hexactinellida - Hexactinellida spp.	3843	6018	2175	6	submersible	ns
Porifera - Hexactinellida - Amphidiscosida - Hyalonematidae - *Hyalonema* sp. indet. 1	3740	4624	884	3	submersible	Fig. 18G
Porifera - Hexactinellida - Amphidiscosida - Hyalonematidae - *Hyalonema* gen. inc. 2	4511	6008	1496	3	submersible	Fig. 18H
Porifera - Hexactinellida - Amphidiscosida - Hyalonematidae - *Hyalonema* sp. indet. 3	4560	4625	66	2	submersible	Fig. 18I
Porifera - Hexactinellida - Amphidiscosida - Hyalonematidae - *Hyalonema* sp. indet. 4	4551	4551	0	1	submersible	Fig. 18L
Porifera - Hexactinellida - Amphidiscosida - Hyalonematidae - *Hyalonema* spp.	4228	4627	399	3	submersible	ns
Porifera - Hexactinellida - Lyssacinosida - Lyssacinosida fam. inc. 1	3733	3733	0	1	submersible	Fig. 18M
Porifera - Hexactinellida - Lyssacinosida - Lyssacinosida fam. inc. 2	6657	6947	290	2	submersible	Fig. 18N
Porifera - Hexactinellida - Lyssacinosida - Lyssacinosida fam. inc. 3	6677	6677	0	1	submersible	Fig. 18O
Porifera - Hexactinellida - Lyssacinosida - Lyssacinosida fam. inc. 4	5640	5640	0	1	submersible	Fig. 18P
Porifera - Hexactinellida - Lyssacinosida - Lyssacinosida fam. inc. 5	4202	5279	1077	2	submersible	Fig. 18Q
Porifera - Hexactinellida - Lyssacinosida - Lyssacinosida fam. inc. 6	5386	5386	0	1	submersible	ns
Porifera - Hexactinellida - Lyssacinosida - Lyssacinosida fam. inc. 7	4454	5339	885	3	submersible	Fig. 18R
Porifera - Hexactinellida - Lyssacinosida - Lyssacinosida fam. indet. 8	3843	3957	113	1	submersible	Fig. 18S
Porifera - Hexactinellida - Lyssacinosida - Euplectellidae - Euplectellidae gen. inc. 1	5644	5644	0	1	submersible	Fig. 18T
Porifera - Hexactinellida - Lyssacinosida - Euplectellidae - Euplectellidae gen. inc. 2	5425	5645	219	1	submersible	Fig. 19A
Porifera - Hexactinellida - Lyssacinosida - Euplectellidae - Euplectellidae gen. inc. 3	5274	5804	531	2	submersible	Fig. 19B
Porifera - Hexactinellida - Lyssacinosida - Euplectellidae - Euplectellidae gen. inc. 4	5580	5580	0	2	submersible	Fig. 19C
Porifera - Hexactinellida - Lyssacinosida - Euplectellidae - Euplectellidae gen. inc. 5	5373	5692	319	1	submersible	Fig. 19D
Porifera - Hexactinellida - Lyssacinosida - Euplectellidae - Euplectellidae gen. inc. 6	5271	5594	323	2	submersible	Fig. 19E
Porifera - Hexactinellida - Lyssacinosida - Euplectellidae - Euplectellidae gen. inc. 7	5921	5921	0	1	both	Fig. 19F
Porifera - Hexactinellida - Lyssacinosida - Euplectellidae - *Docosaccus* sp. inc. 1	5994	5994	0	1	submersible	Fig. 19G
Porifera - Hexactinellida - Lyssacinosida - Euplectellidae - *Holascus* sp. indet. 1	5997	5997	0	1	submersible	Fig. 19H
Porifera - Hexactinellida - Lyssacinosida - Euplectellidae - *Holascus* sp. indet. 2	5994	5995	1	1	submersible	Fig. 19I
Porifera - Hexactinellida - Lyssacinosida - Euplectellidae - *Hyalostylus* sp. indet. 1	4656	5791	1135	3	submersible	Fig. 19L
Porifera - Hexactinellida - Lyssacinosida - Euplectellidae - *Hyalostylus* sp. indet. 2	5494	5629	135	1	submersible	Fig. 19M
Porifera - Hexactinellida - Lyssacinosida - Euplectellidae - *Hyalostylus* sp. indet. 3	3957	5481	1524	2	submersible	Fig. 19N
Porifera - Hexactinellida - Lyssacinosida - Rossellidae - *Caulophacus* sp. indet. 1	5270	5639	369	2	submersible	Fig. 19O
Porifera - Hexactinellida - Sceptrulophora - Euretidae - Euretidae gen. inc. 1	6014	6014	0	1	submersible	Fig. 19P
Porifera - Hexactinellida - Sceptrulophora - Euretidae - Euretidae gen. inc. 2	5134	5448	313	1	submersible	Fig. 19Q
Porifera - Hexactinellida - Sceptrulophora - Euretidae - Euretidae gen. inc. 3	5164	5164	0	1	submersible	Fig. 19R
Porifera - Hexactinellida - Sceptrulophora - Euretidae - *Periphragella* sp. inc. 1	5477	5639	163	1	submersible	Fig. 19S
Porifera - Hexactinellida - Sceptrulophora - Euretidae - *Periphragella* sp. inc. 2	5142	5594	452	1	submersible	Fig. 19T
Porifera - Hexactinellida - Sceptrulophora - Farreidae - Farreidae gen. inc. 1	5373	5373	0	1	submersible	Fig. 19U
Chaetognatha - Chaetognatha spp.	2751	7621	4870	72	both	Fig. 20A
Brachiopoda - Brachiopoda spp.	5586	6671	1085	2	submersible	Fig. 20B
Bryozoa - Bryozoa spp.	3233	4626	1392	3	submersible	ns
Bryozoa - Bryozoa cls. indet. 1	3225	5295	2070	3	submersible	Fig. 20C
Bryozoa - Bryozoa cls. indet. 2	3215	4634	1420	2	submersible	Fig. 20D
Hemichordata - Enteropneusta - Torquaratoridae - Torquatoridae gen. indet. 1	4429	4536	107	1	submersible	Fig. 20E
Hemichordata - Enteropneusta - Torquaratoridae - Torquatoridae gen. indet. 2	6661	6661	0	2	both	Fig. 20F
Mollusca - Cephalopoda - Octopoda - Cirroteuthidae - Cirroteuthis sp. indet. 1	5151	6015	864	4	both	Fig. 20I
Mollusca - Cephalopoda - Octopoda - Octopoda spp.	4549	4549	0	1	lander	Fig. 20J
Mollusca - Gastropoda - Gastropoda spp.	3799	6678	2879	4	submersible	ns
Mollusca - Gastropoda - Neogastropoda - Buccinidae - Buccinidae spp.	5269	7203	1933	4	both	Fig. 20G
Mollusca - Gastropoda - Neogastropoda - Neogastropoda fam. indet. 1	3273	3273	0	1	submersible	Fig. 20H
Nemertea - Nemertea spp.	7985	7987	2	1	submersible	
Nemertea - Nemertea cls. indet. 1	6480	7987	1507	22	both	Fig. 20K, L
Nemertea - Nemertea cls. indet. 2	5367	7470	2103	13	lander	Fig. 20M
Nemertea - Nemertea cls. indet. 3	5497	6094	597	4	lander	Fig. 20N
Platyhelminthes - Platyhelminthes spp.	5265	5269	4	1	submersible	Fig. 20O
						
Kingdom Protista						
Retaria - Radiozoa cls. indet. 1	3234	3804	570	1	submersible	Fig. 20P
Retaria - Radiozoa cls. indet. 2	5265	5295	30	1	submersible	Fig. 20Q
Retaria - Radiozoa cls. indet. 3	6653	6683	29	1	submersible	Fig. 20R
Cercozoa - *Tuscaridium* - *Tuscaridium* spp.	2751	7987	5238	8	both	Fig. 20S

**Table 2.** Putative bathymetric range extensions and notable deep records, with links to reference imagery. * represents first hadal occurrence, ^+^ indicates deepest observation not pictured in reference imagery due to image quality.

**Taxon**		**Previous recorded max depth (m)**	**Max depth (m), this study**	**Depth Extension (m)**
**Phylum**	Brachiopoda (Fig. 20B)	5530 (live); 7600 (shell) (Beliaev 1989)	6671	1141 (live)
	Cercozoa (Fig. 20S)	Hadal eDNA only (8727 m); Xu et al. 2018)	7987	unknown
**Class**	Appendicularia ^+^	7176 (Jamieson and Linley 2021)	7218	42
**Order**	Corallimorpharia* (Fig. 8E)	5320 (Eash-Loucks and Fautin 2012)	6682	1362
	Narcomedusae ^+^	6777 (Jamieson and Linley 2021)	7989	1212
**Family**	Amathillopsidae* (Fig. 4F)	5559 (Lörz and Horton 2021)	6228	669
	Candelabridae* (Fig. 11C)	4277–4278 (Rybakova et al. 2020)	6876	2596
	Hesionidae* (Fig. 3I)	5325–5506 (Earth Sciences New Zealand 2025)	6683	1177
	Onuphidae (Fig. 3Q)	6330 (Kucheruk 1977).	6680	350
	Umbellulidae (Fig. 10L)	6364 (NOAA DSCRTP 2026)	6949	585
