# Supplementary material for: Biodiversity of the abyssal and hadal zone in the Nova Canton Trough (Central Pacific Ocean; 2751–7989 m): a visual inventory
Source: Biodivers Data J. 2026 Sep 15;14:e204436. doi: 10.3897/BDJ.14.e204436 (PMC13598491; doi:10.3897/BDJ.14.e204436)
Supplement: Supplementary file 1 [file bdj-14-e204436-s002.pdf]

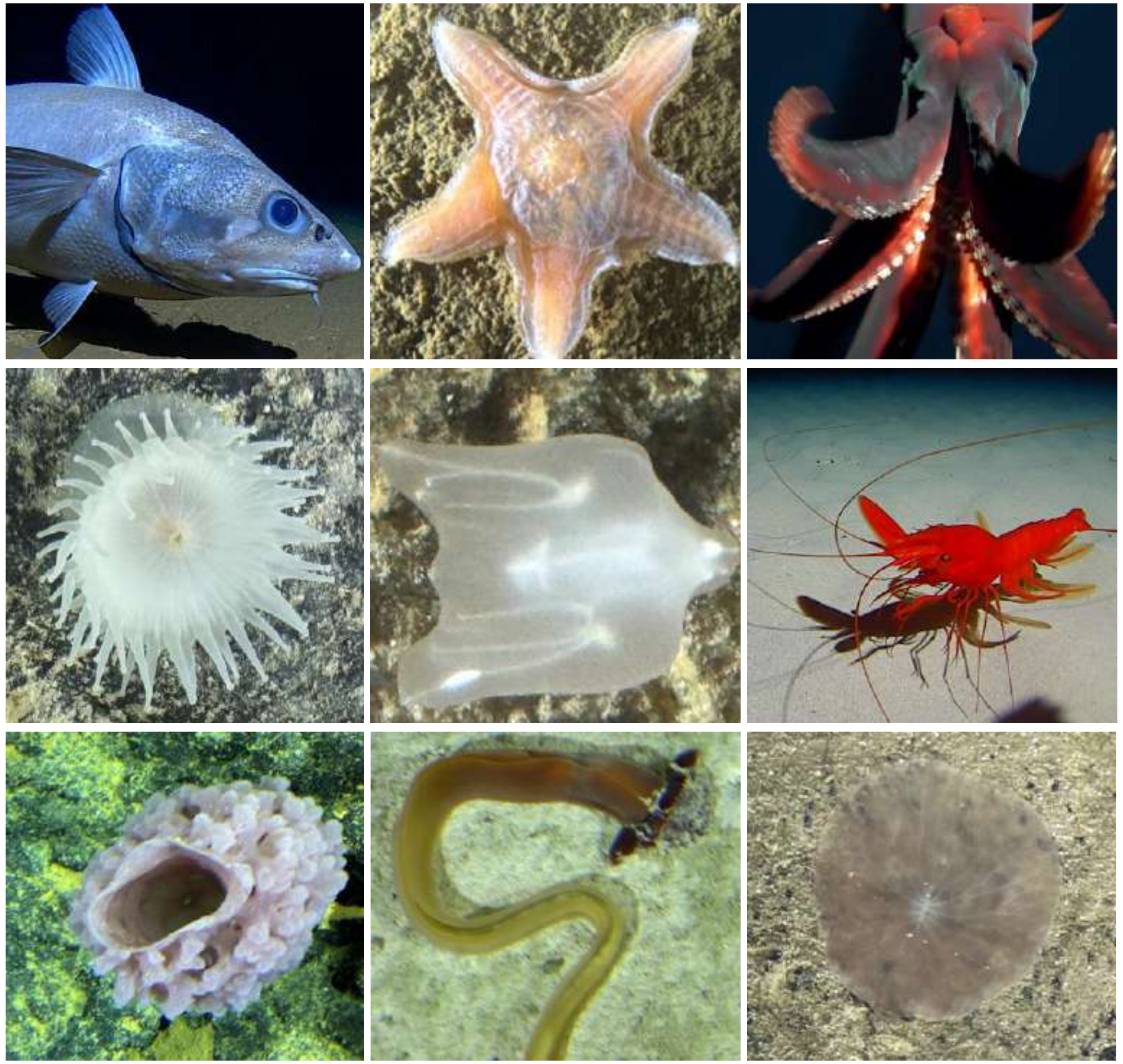

Nova Canton 2024

-Morphotaxa –

Supplementary Material

This document collates morphotypes observed during 99 lander deployments and 16 sub dives between ~3000 and 8000 m in the Nova Canton Trough. Morphotypes are sorted by phylum.

Class; Order; Family; Morphotaxa ON_signature (X)

(Note: morph numbers assigned based on this dataset only)

Deployment Code

Best pic 1

Best pic 2

Relevant morphological descriptors

Chordata

NC4_CR10_5000_NOR_0055

NC4_CR10_5000_NOR_0063

Appendicularia; Copelata; Copelata spp

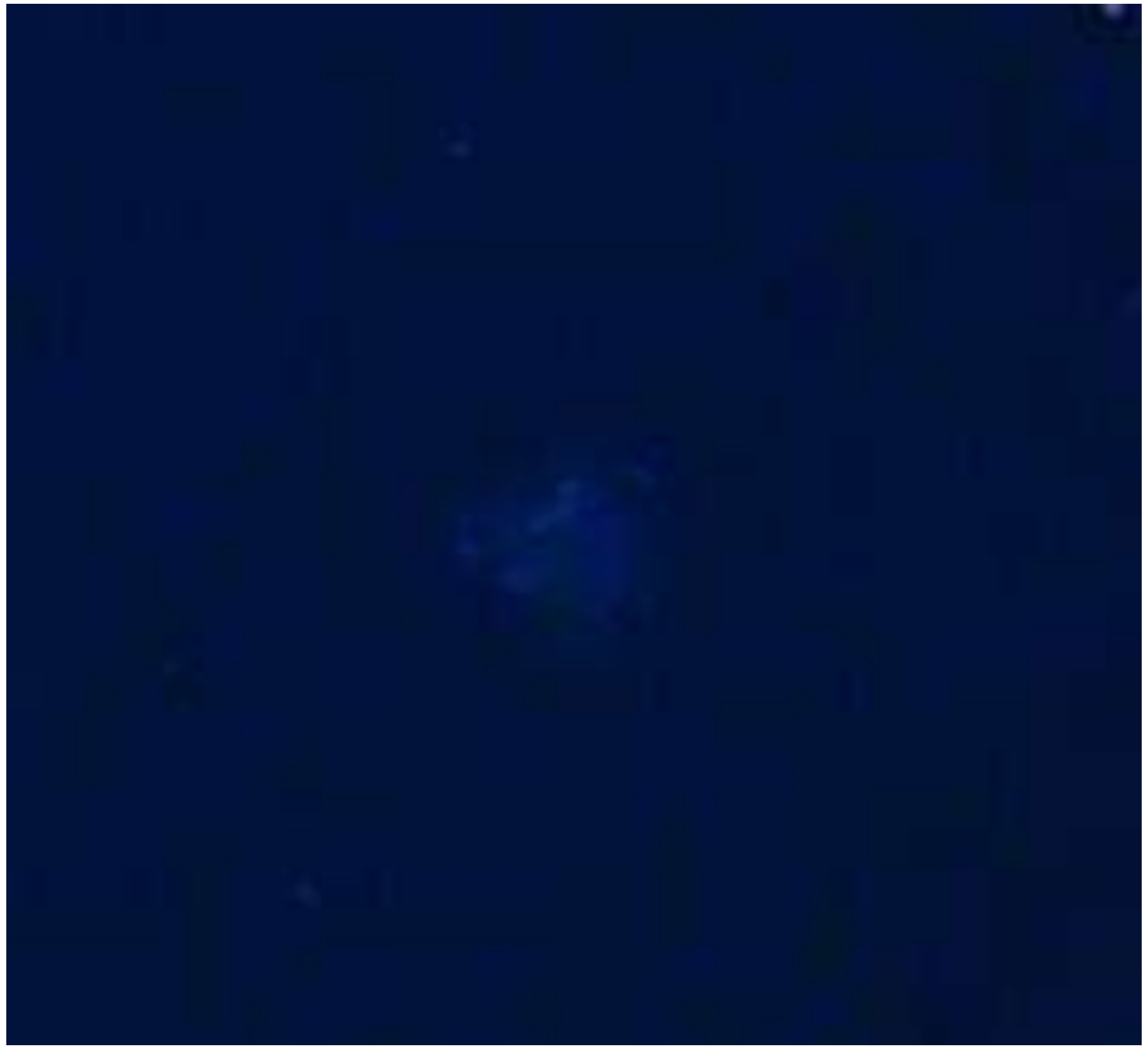
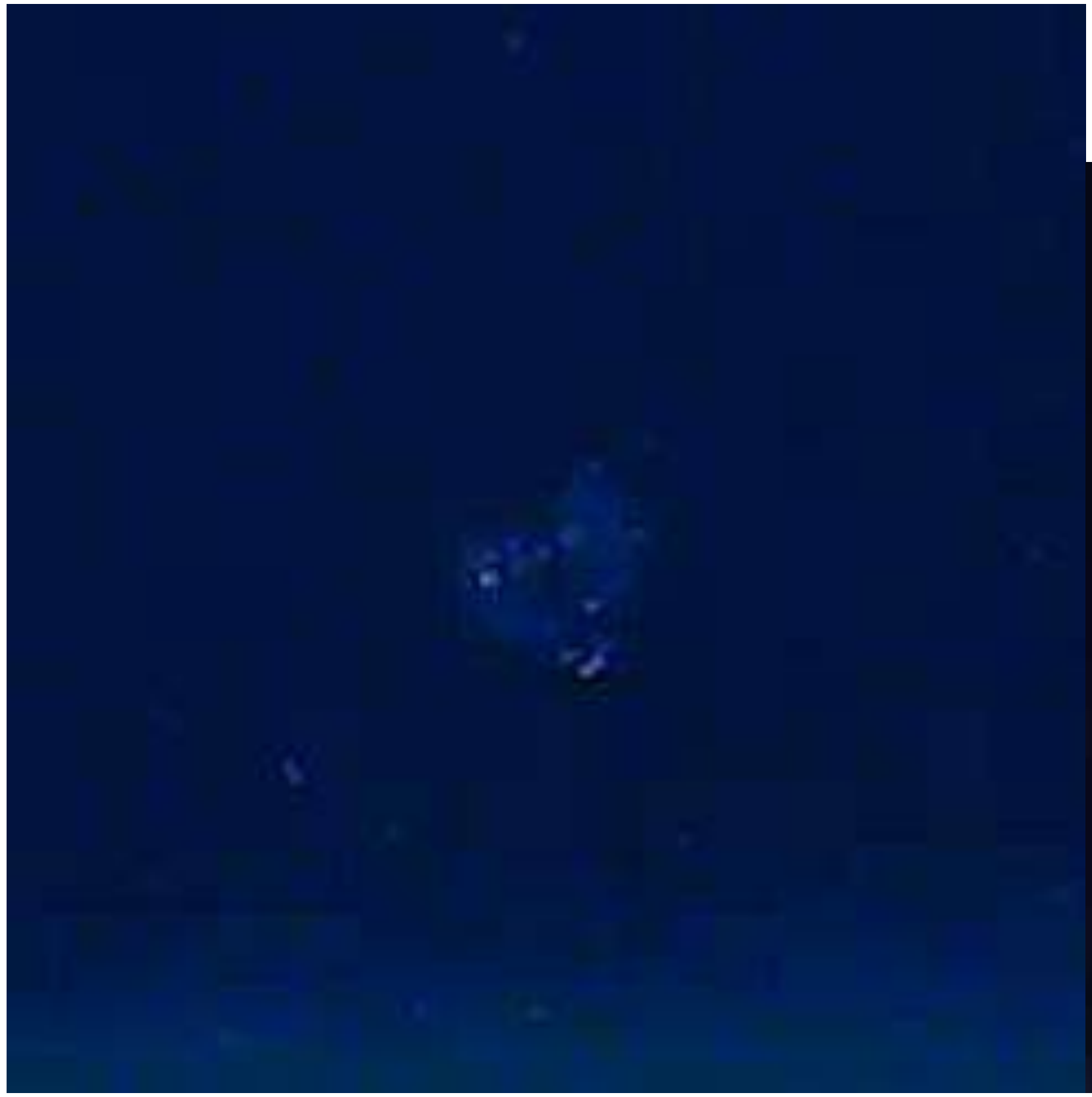
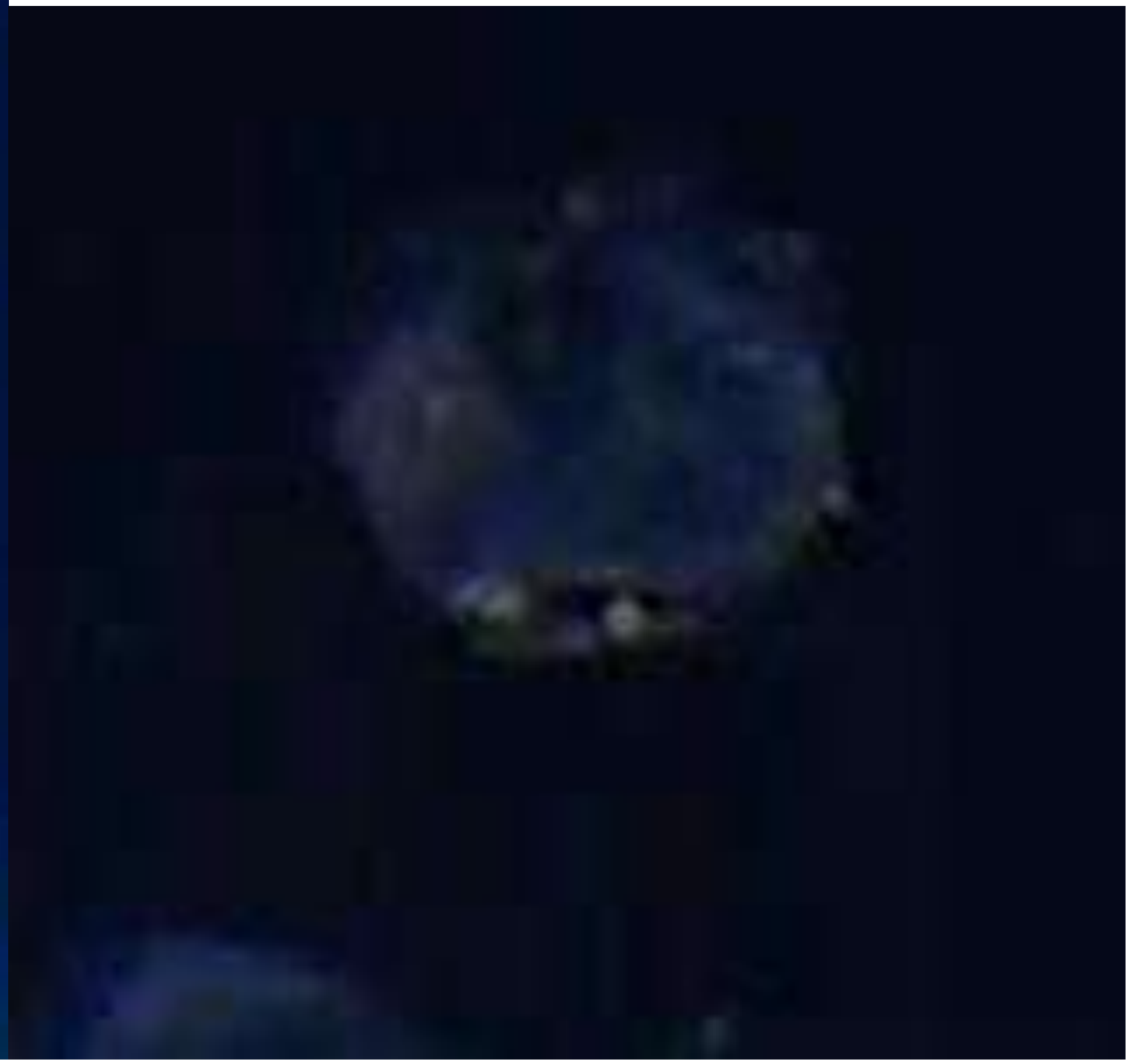

Small , translucent-white organism encased within a generally rounded, transparent gelatinous membrane. Particulate matter often observed attached to the gelatinous membrane. Observations likely the discarded membrane of either Fritillaridae or Oikopleuridae.

Appendicularia; Copelata; Copelata fam. indet. 1

NC2_CR2_3500_NOR_0071

NC2_BK5_4600_IMG_0416

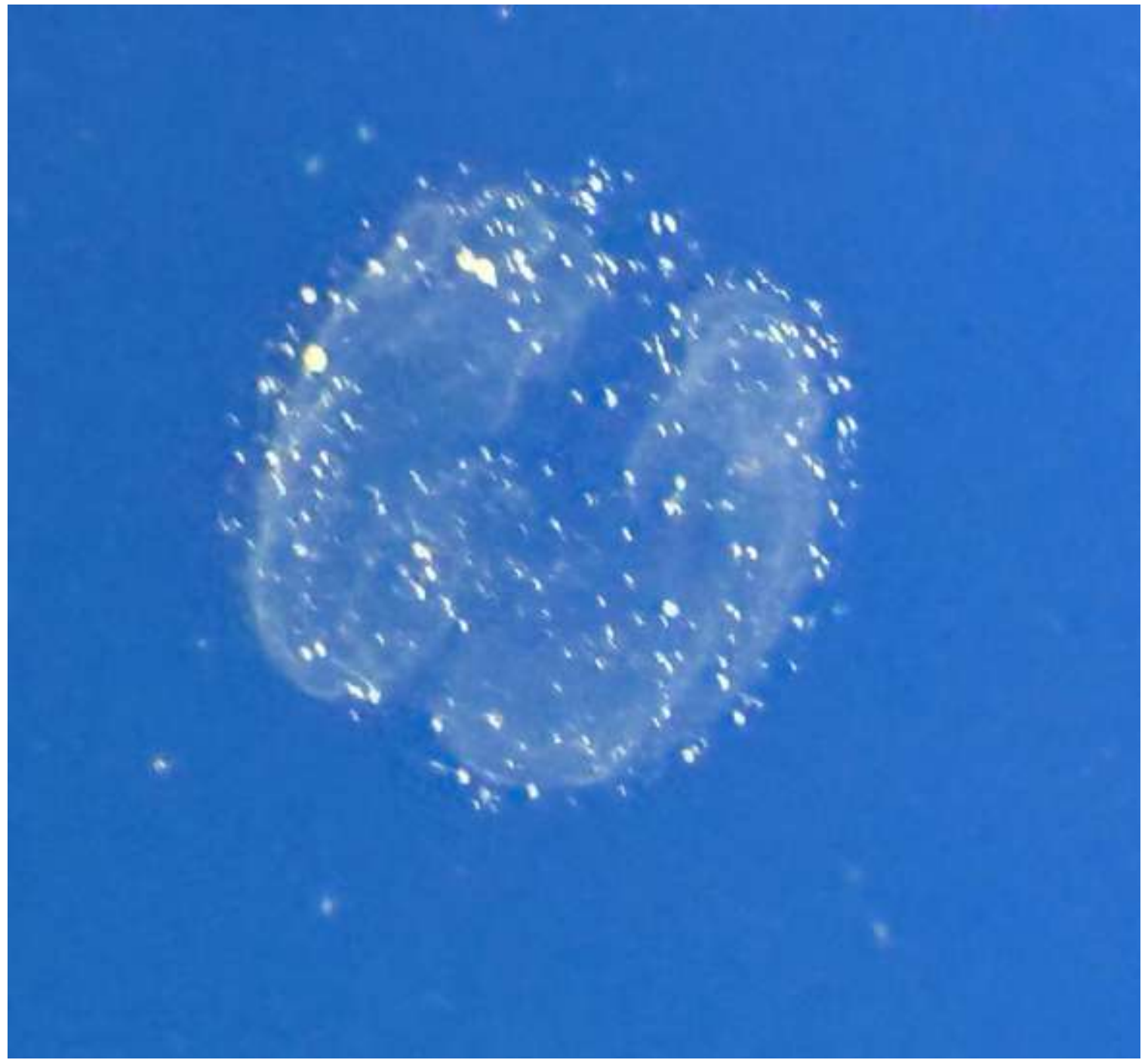

Small , translucent-white organism encased within a rounded gelatinous membrane consisting of two lobes reminiscent of tangerine slices. Particulate matter often observed attached to the gelatinous membrane.

Cf. Oikopleuridae

Appendicularia; Copelata; Copelata fam. indet. 2

NC4_MA11_5700_NOR_0114

NC3_CR4_5700_NOR_0062

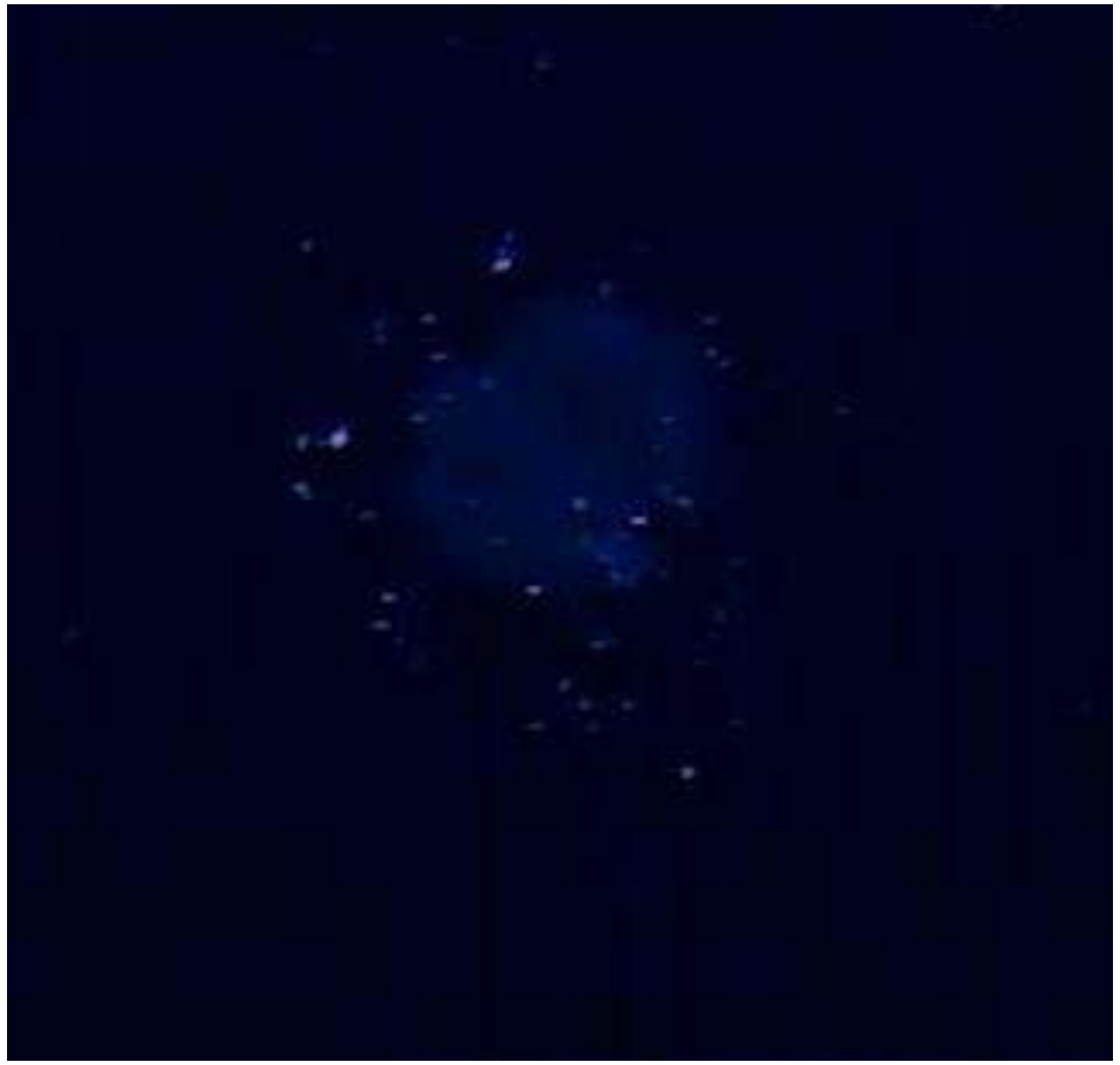

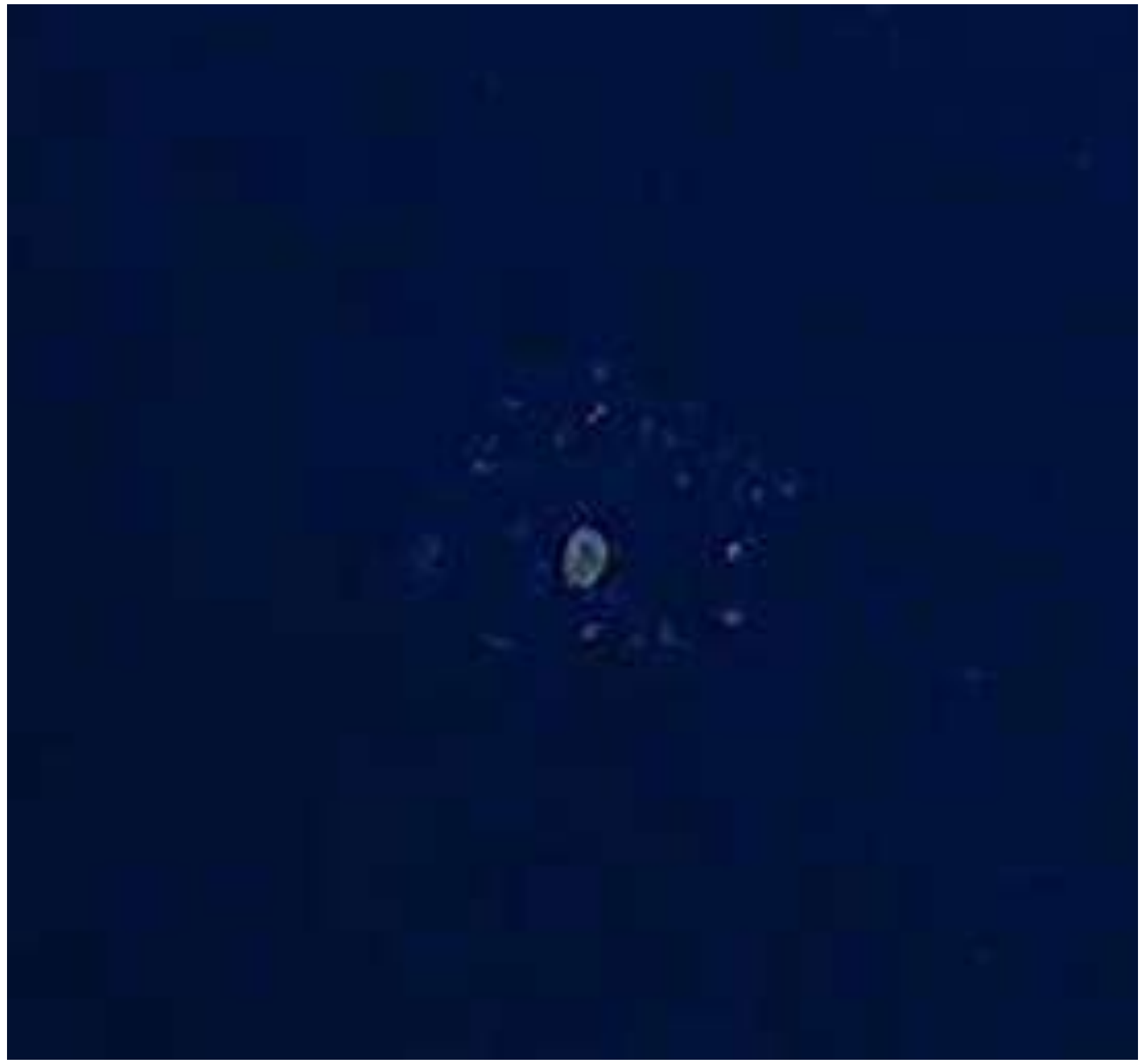

Small , translucent-white organism encased within a rounded gelatinous membrane. Particulate matter often observed attached to the gelatinous membrane.

Cf. Fritillaridae

Tunicata; Ascidiacea; Phlebobranchia; Octacnemidae; Octacnemidae gen. indet. 1

NC3_BK2_6000_NOR_0091

NC3_BK4_8000_NOR_0098

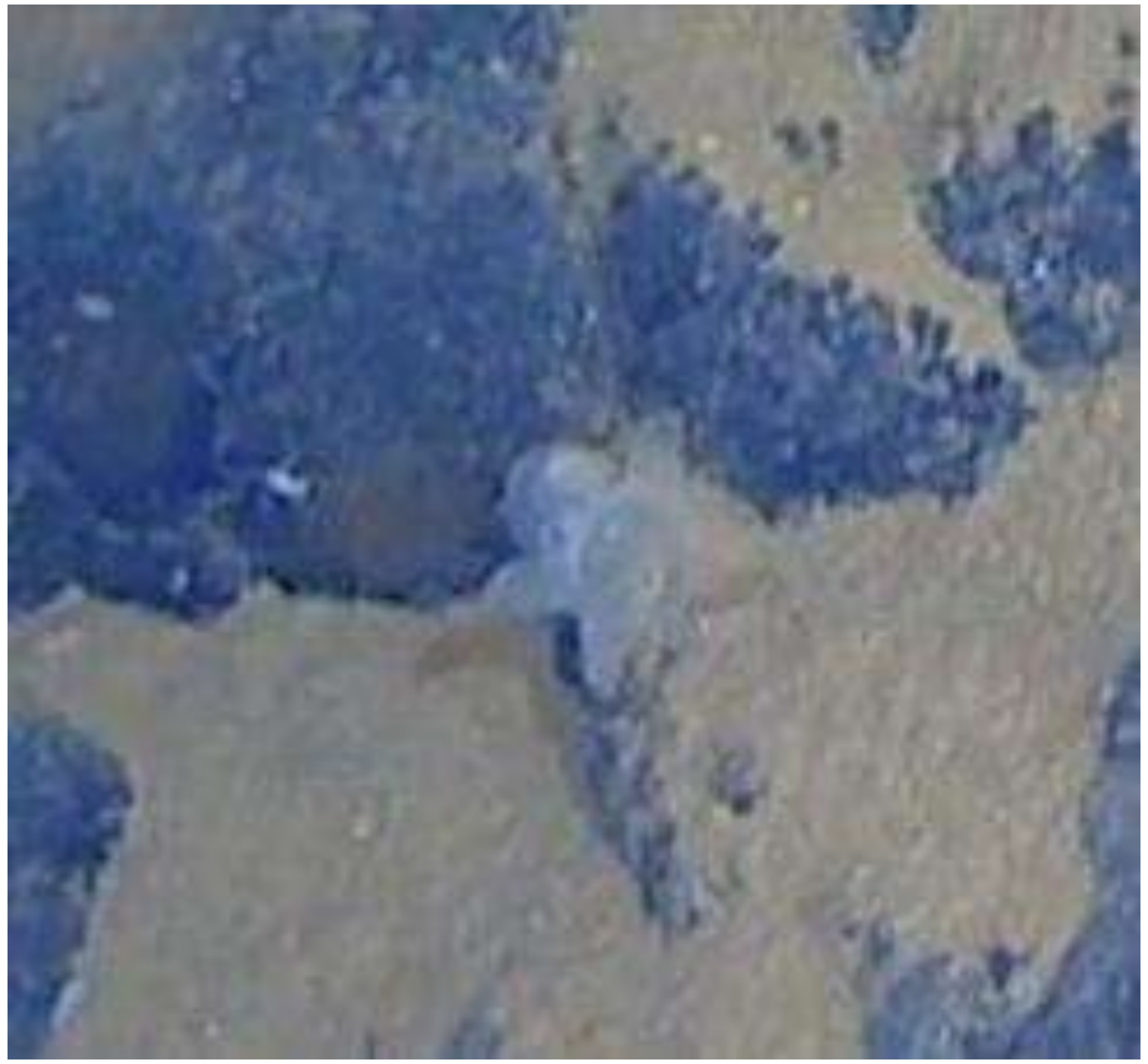

Small , translucent-white organism with a gelatinous texture often observed with particulate matter attached to the gelatinous membrane. 4-8 pedal-like lobes present on some individuals

Tunicata; Ascidiacea; Phlebobranchia; Octacnemidae; Octacnemidae gen. indet. 2

NC3_BK4_8000

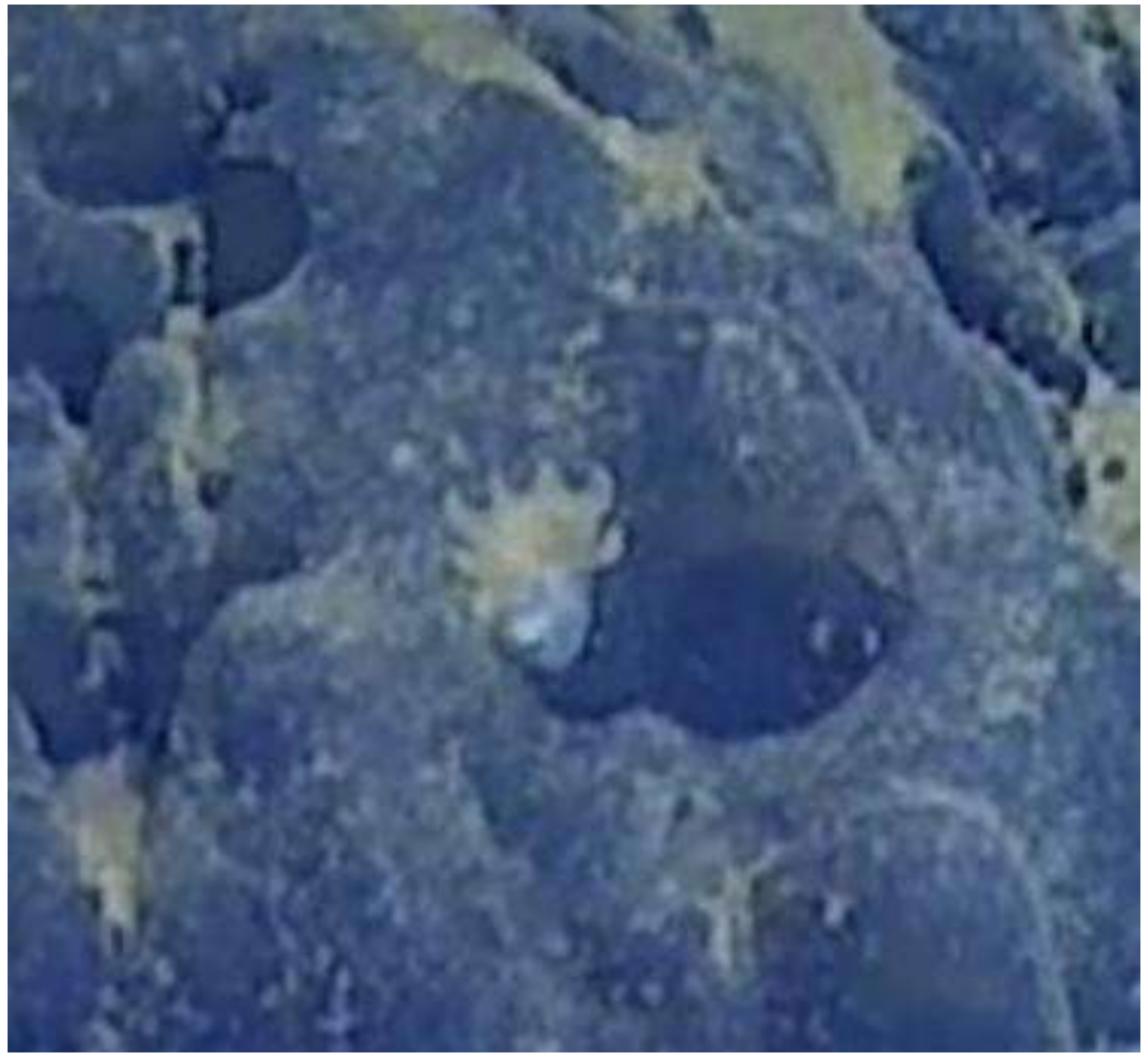

Small , translucent-white organism with a gelatinous texture observed with particulate matter attached to the gelatinous membrane. Cf octacnemus spp – 8 pointed tentacles with smaller one, closed upon approach submersible

Tunicata; Ascidiacea; Phlebobranchia; Octacnemidae; *Megalodicopia* sp. indet. 1

NC4_BK3_6900

NC3_BK2_6000

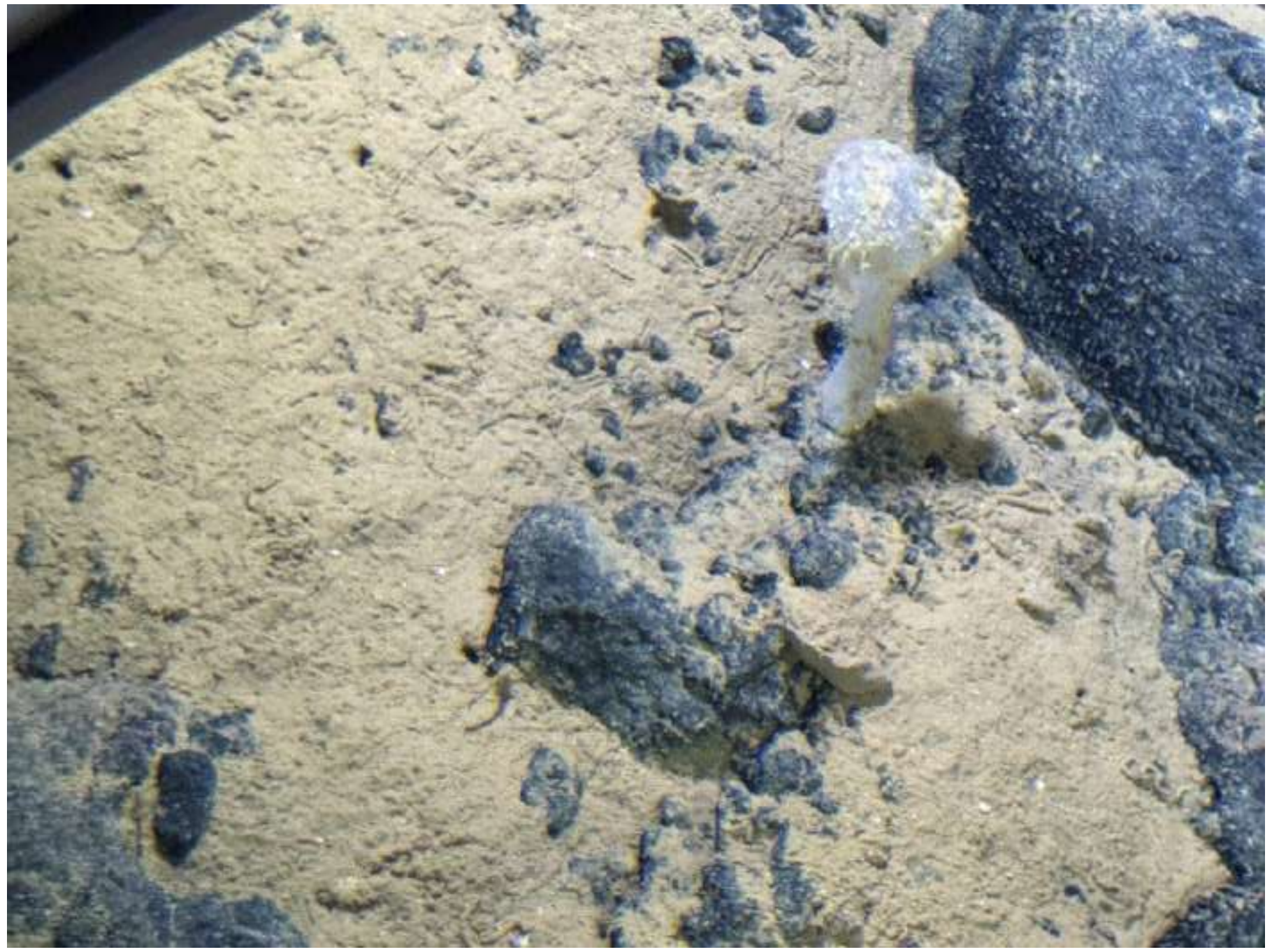

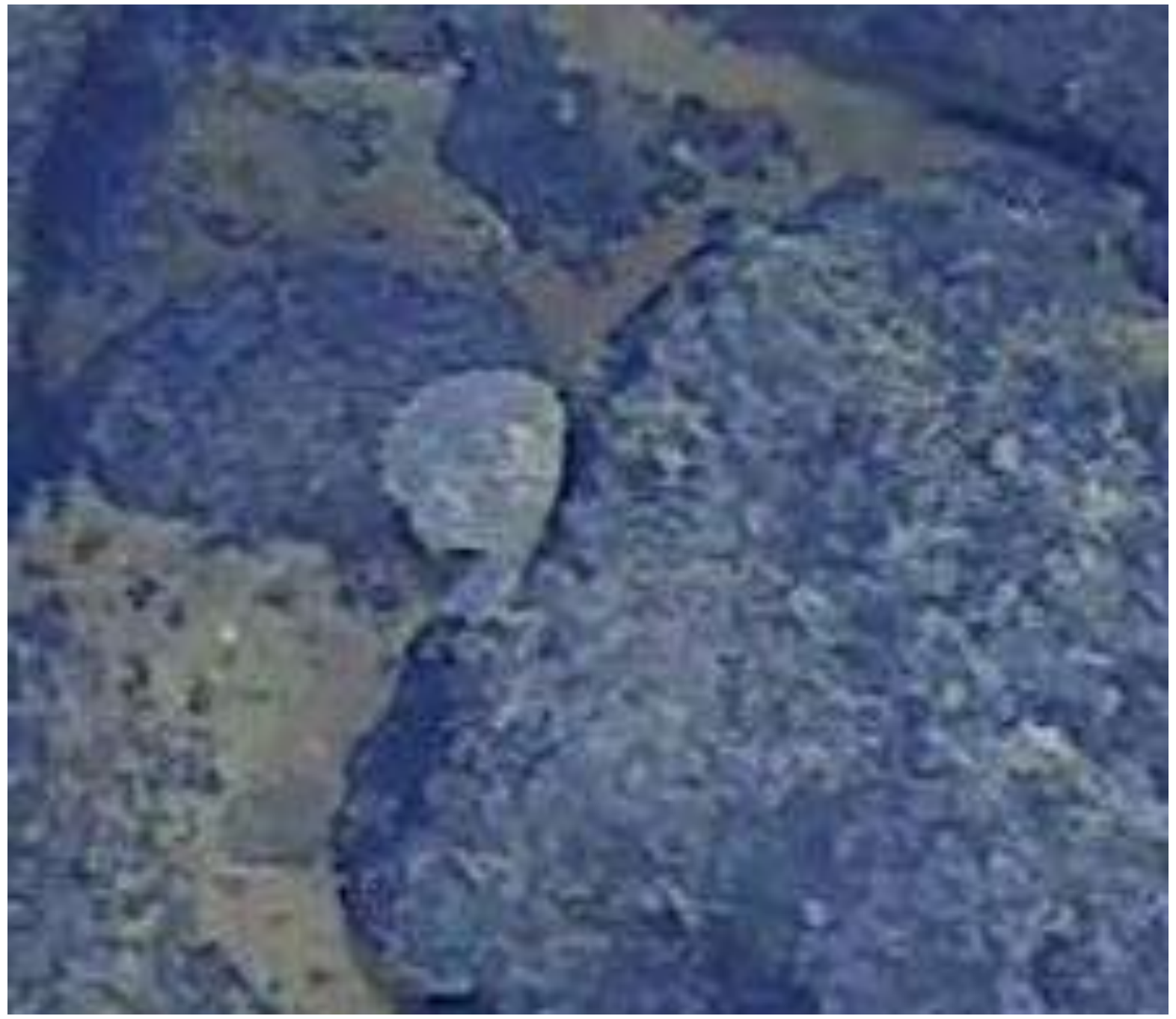

Stalked octacnemid

NC4_BK6_4700

NC4_BK4_4700

Tunicata; Ascidiacea; Phlebobranchia; Octacnemidae; *Megalodicopia* sp. indet. 2

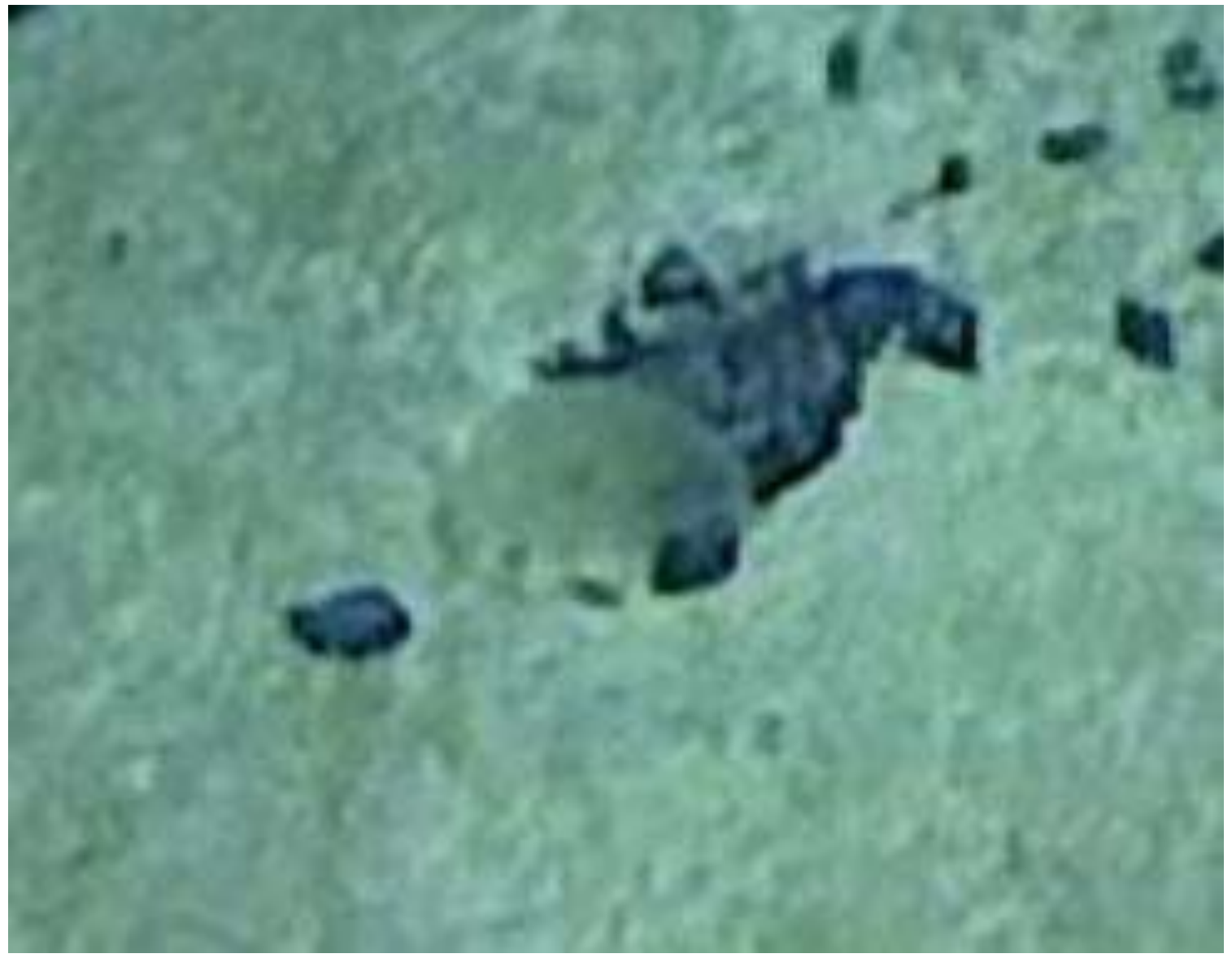

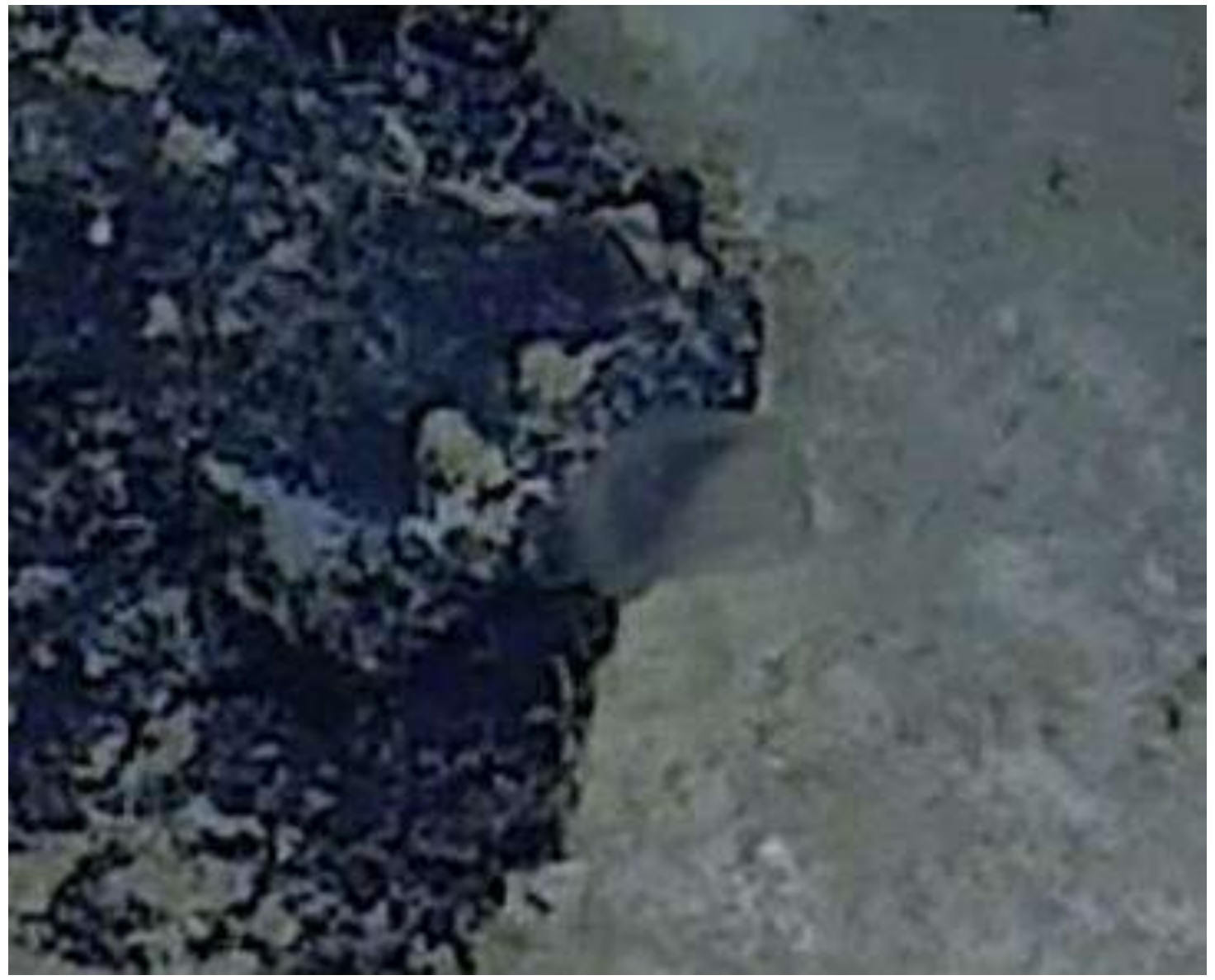

Small , translucent-white organism with a gelatinous texture attached to rocks. Short stalk, mouth, Slightly darker hue than other octacnemids observed. Small size.

Tunicata; Ascidiacea; Phlebobranchia; Octacnemidae; *Megalodicopia* sp. indet. 3

NC3_BK2_6000_NOR_0091

NC3_BK3_6000_NOR_0072

NC4_BK3_6900 NOR 0062

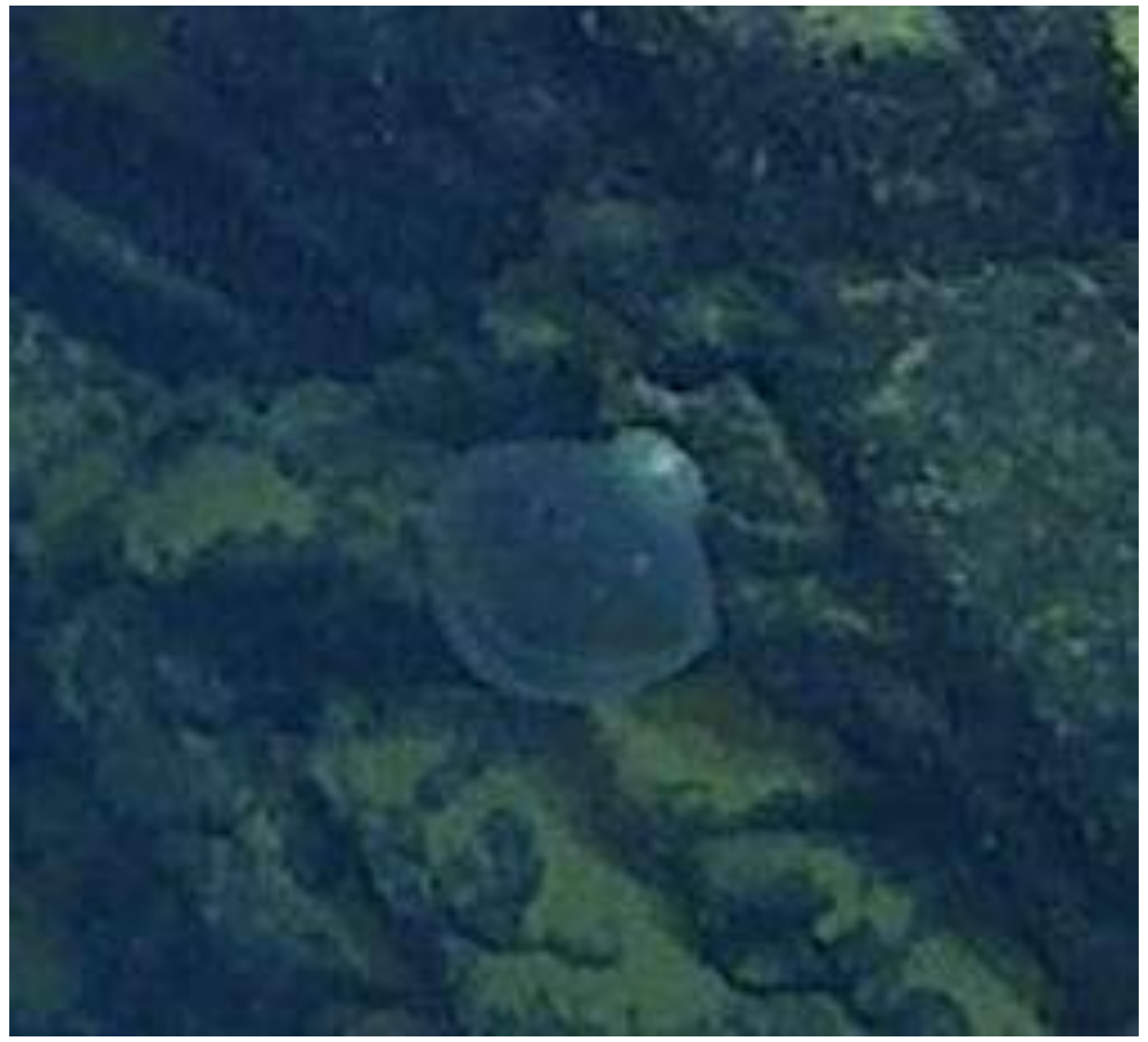

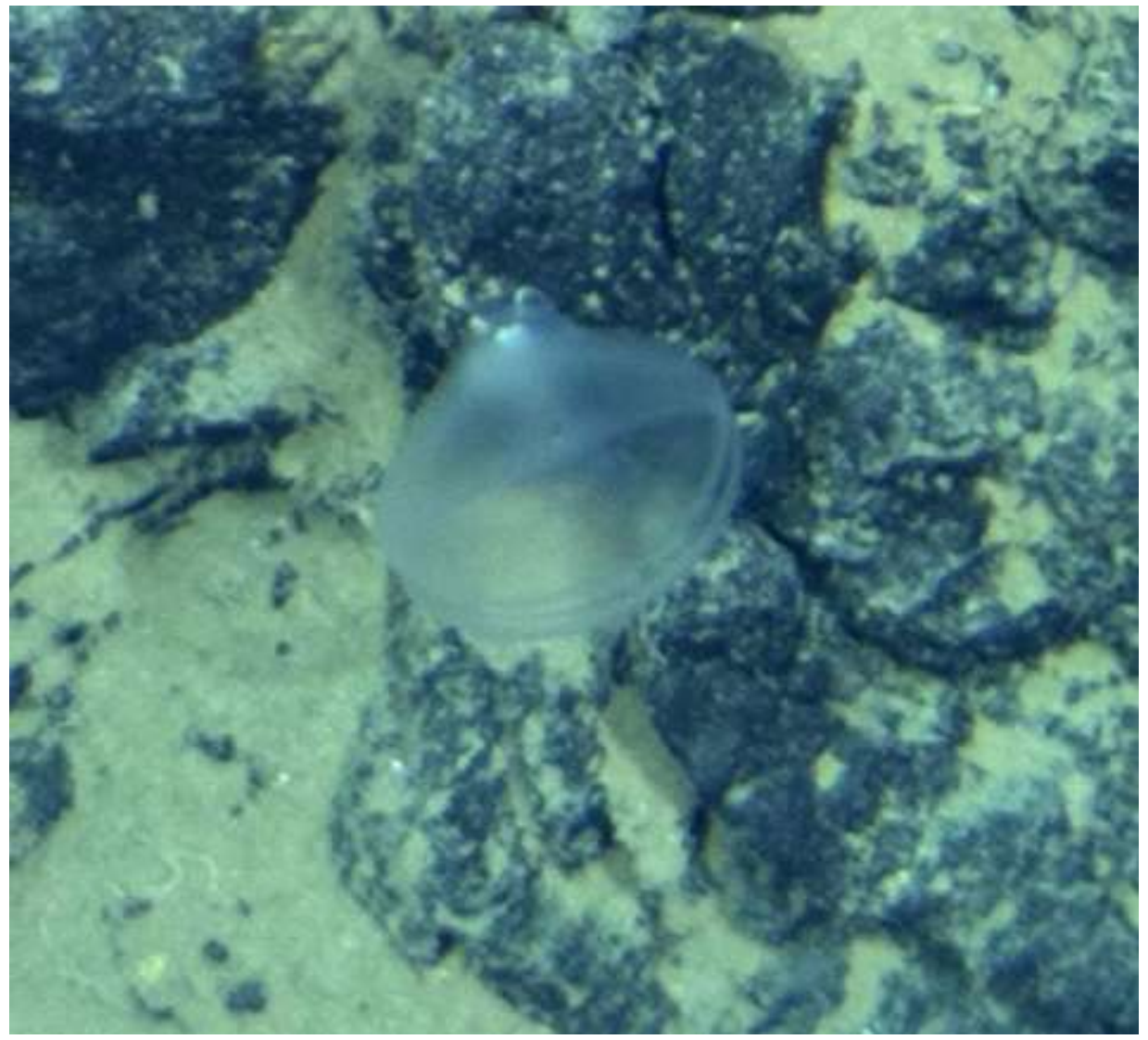

Small , translucent-white organism with a gelatinous texture often observed with particulate matter attached to the gelatinous membrane. mouth-like siphon present on the gelatinous membrane gives the organism a “pac-man” like appearance. Attachment to hard substrates is thick and short – white detail, size and shape makes it different from 2

Teleostei; Perciformes
Zoarcidae ; *Pachycara; Pachycara* sp. indet. 1

NC2_CR3_3800_NOR_0061

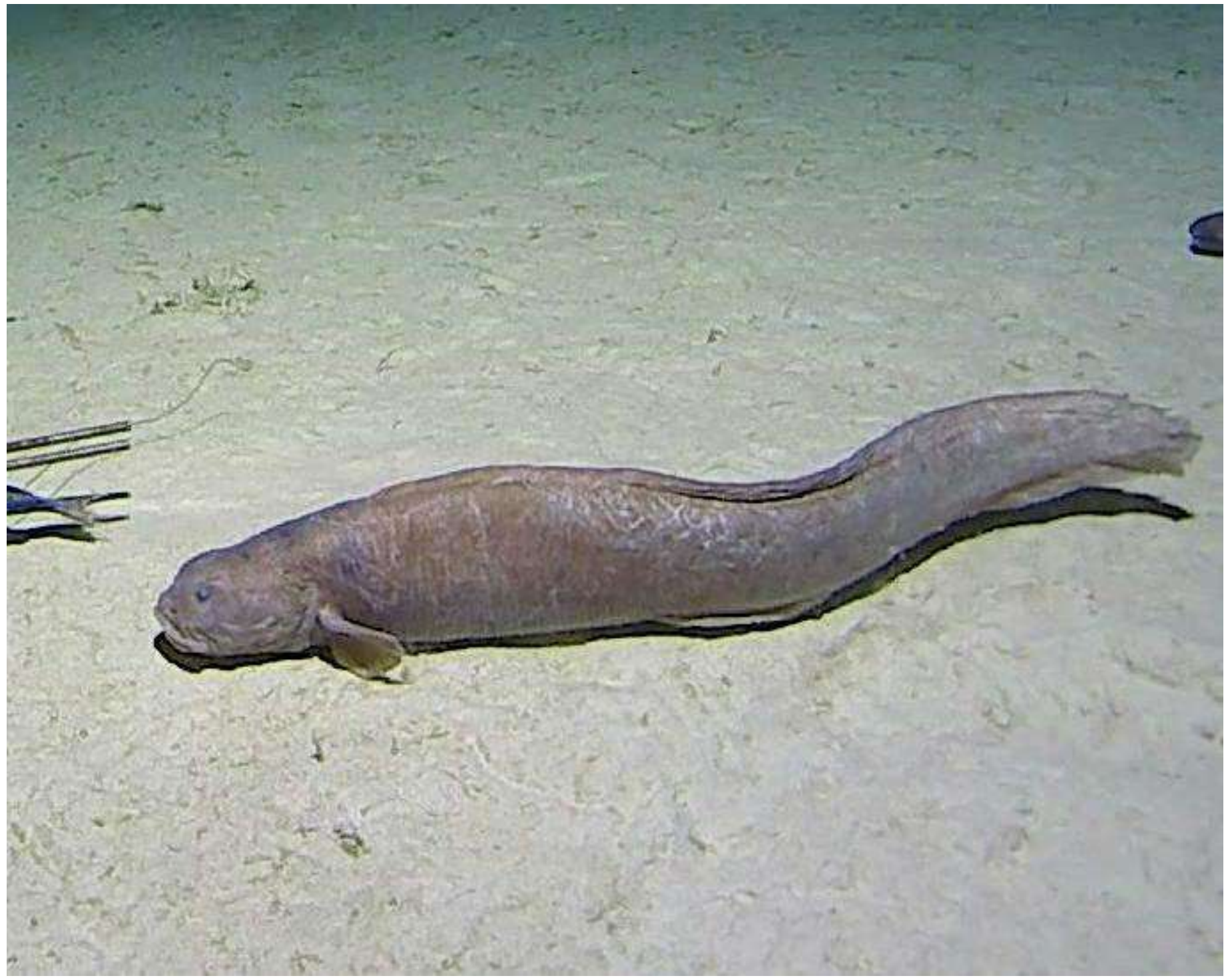

Skin is wrinkled and dull brownish in colouration. Body is robust. Dorsal fin begins behind pectorals and extends to the rounded tail tip. Eyes are small.

Teleostei; Perciformes
Zoarcidae ; *Pachycara;*
Pachycara sp. indet. 2

NC4_MA5_6000_NOR_0113
NC4_MA5_6000_NOR_0114

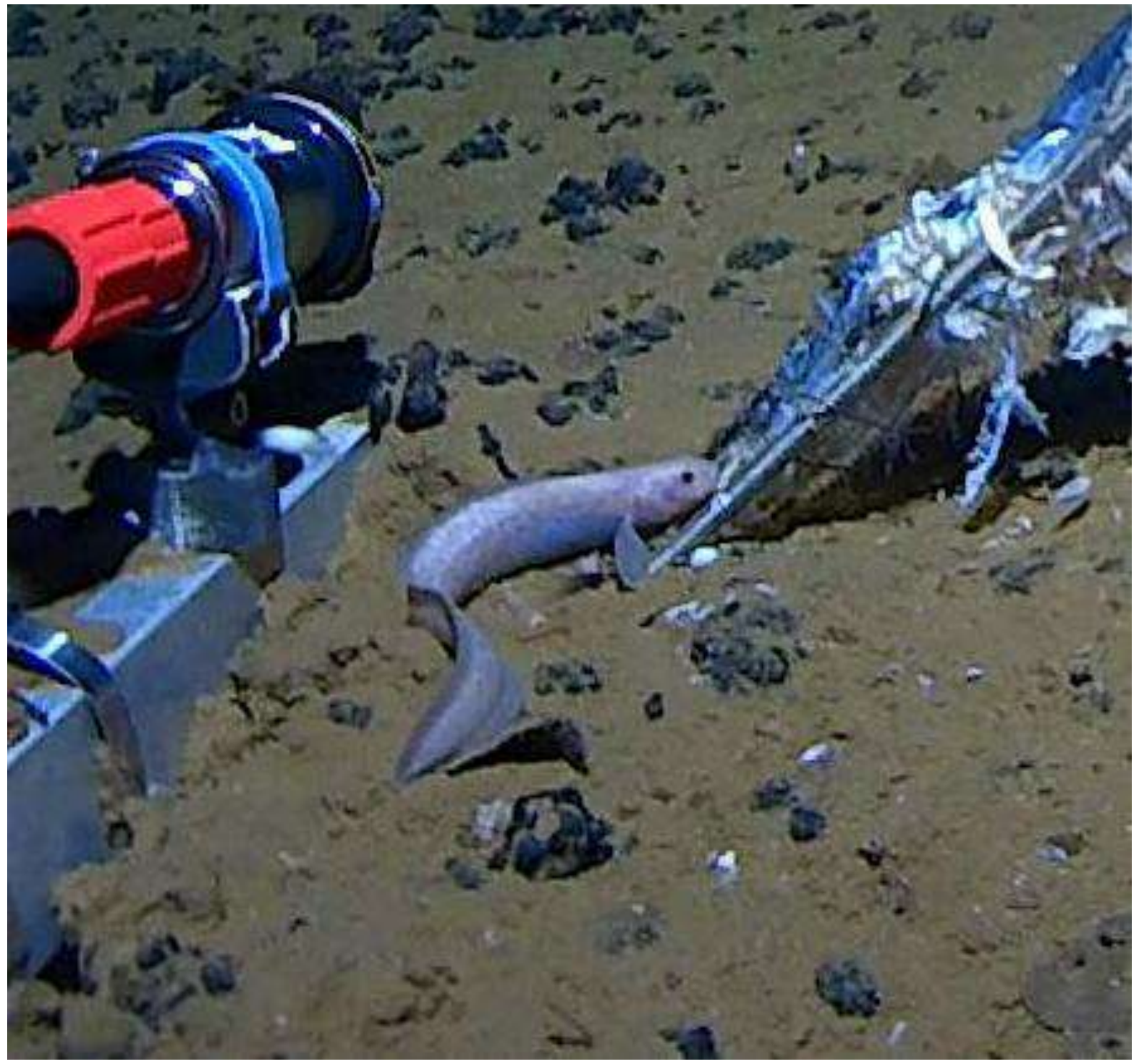

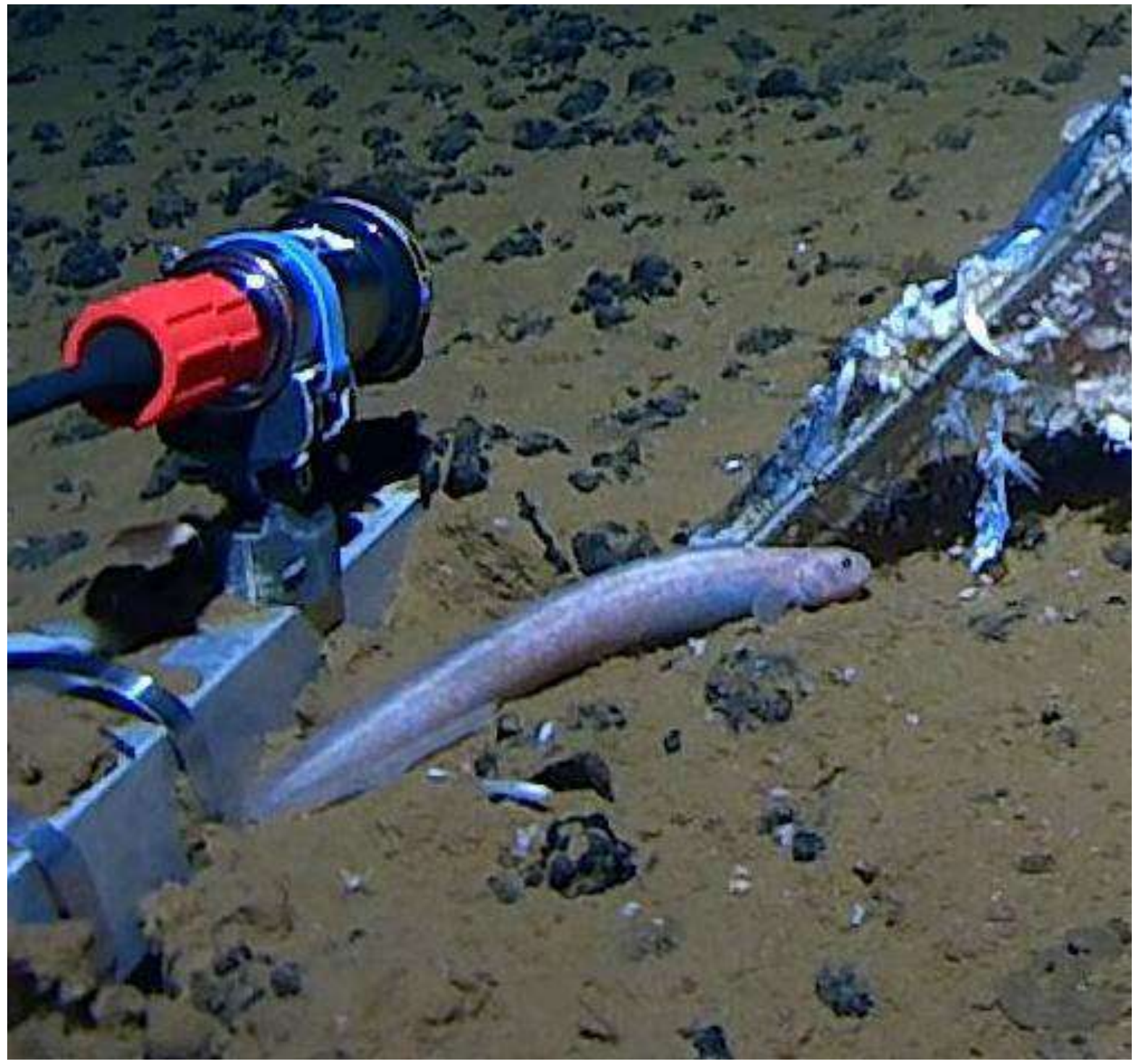

Skin is pinkish-purple in colour. Dorsal fin begins after the prominent pectoral fins. Large dark eyes relative to small sized Body.

Teleostei; Perciformes
Zoarcidae ; *Pachycara; Pachycara* spp.

NC4_OM11_4500_NOR_0108

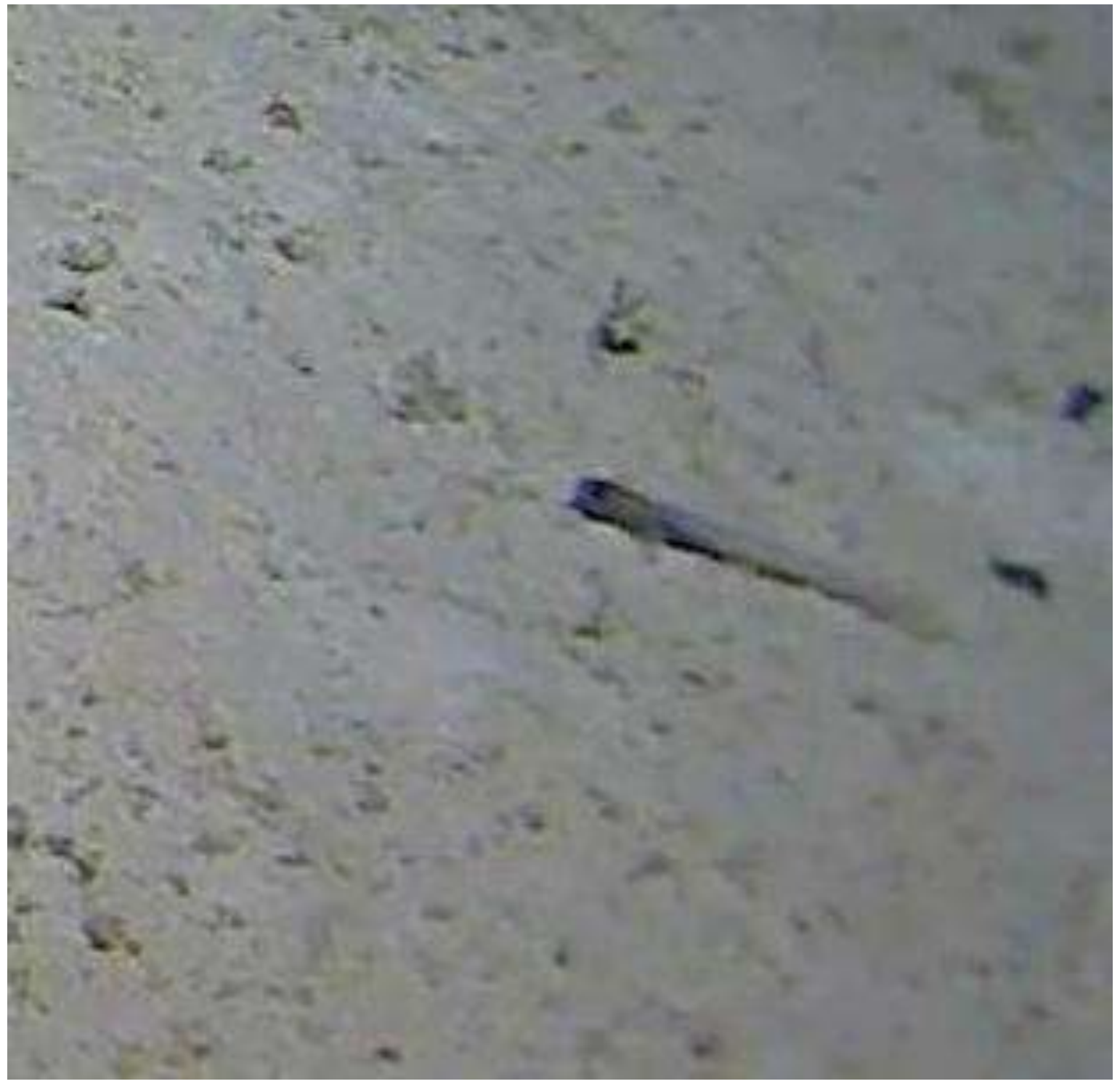

Skin is translucent. Small size with large dark eyes relative to small body. Smaller than Pachycara sp. 2 (possibly juvenile form of other Pachycara morphotypes)

Teleostei; Anguilliformes
Synaphobranchidae ; *Ilyophis;*
Ilyophis sp indet.1

NC3_CR7_3200_NOR_0090
NC3_OM7_3500_NOR_0092

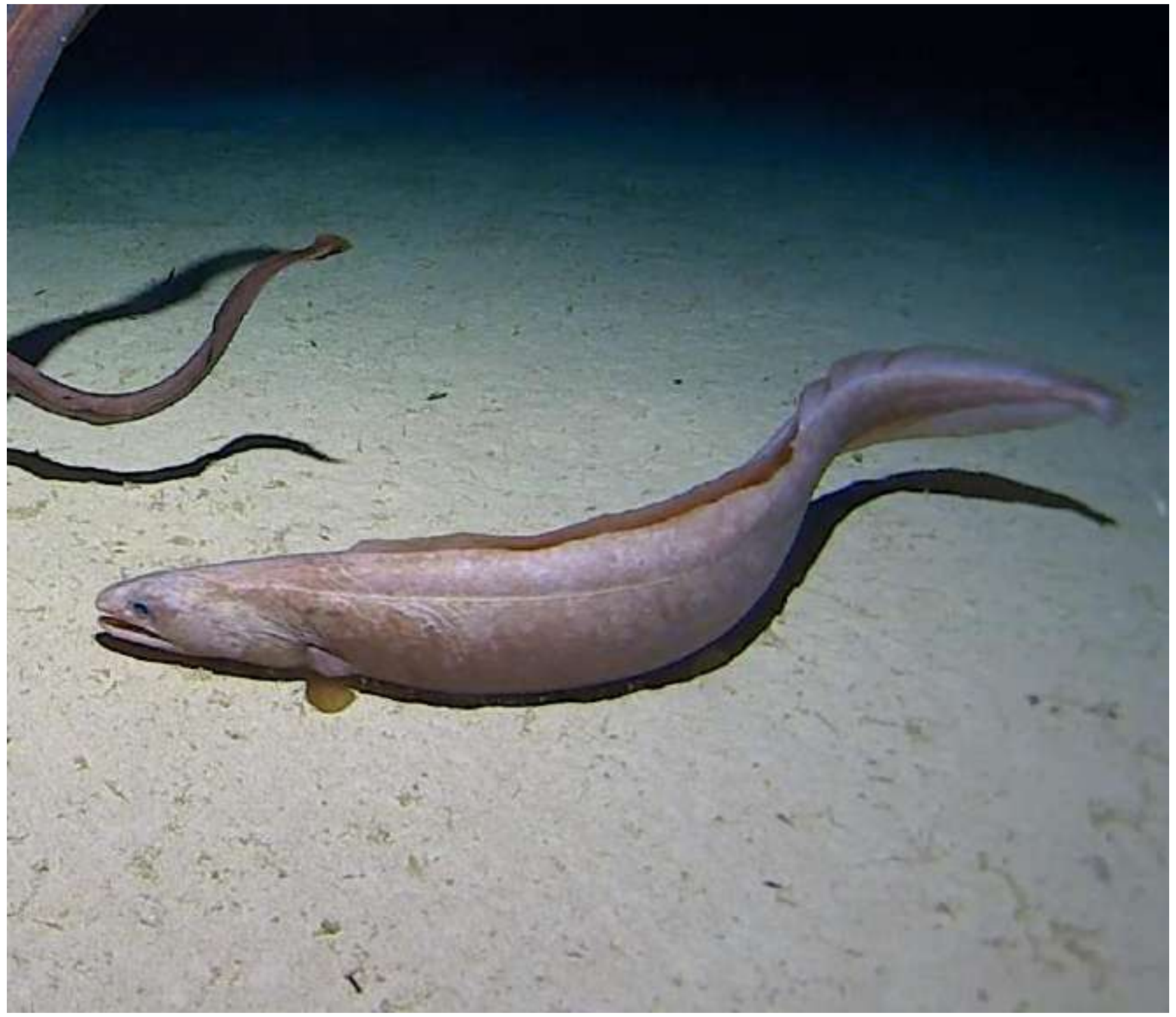
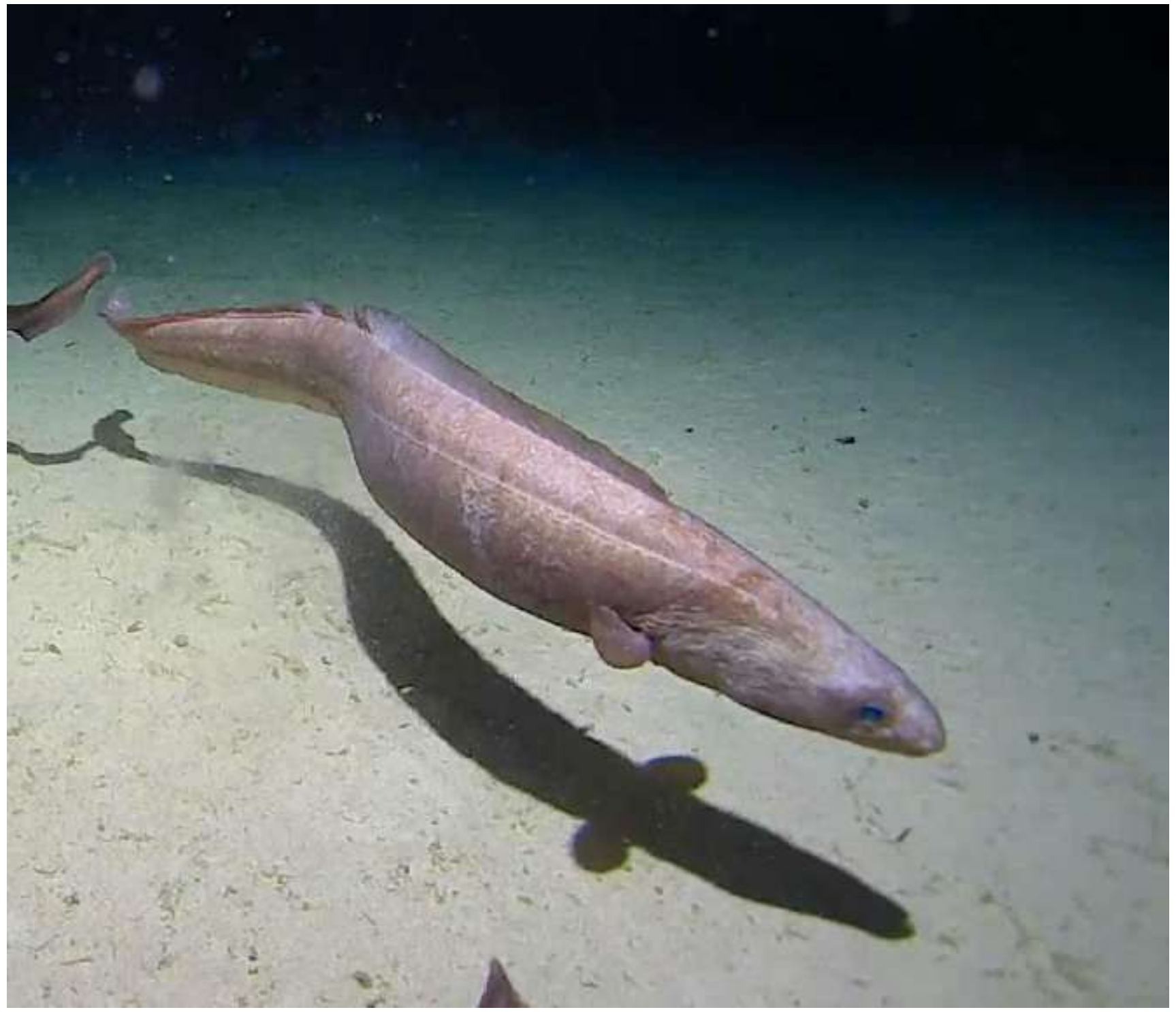
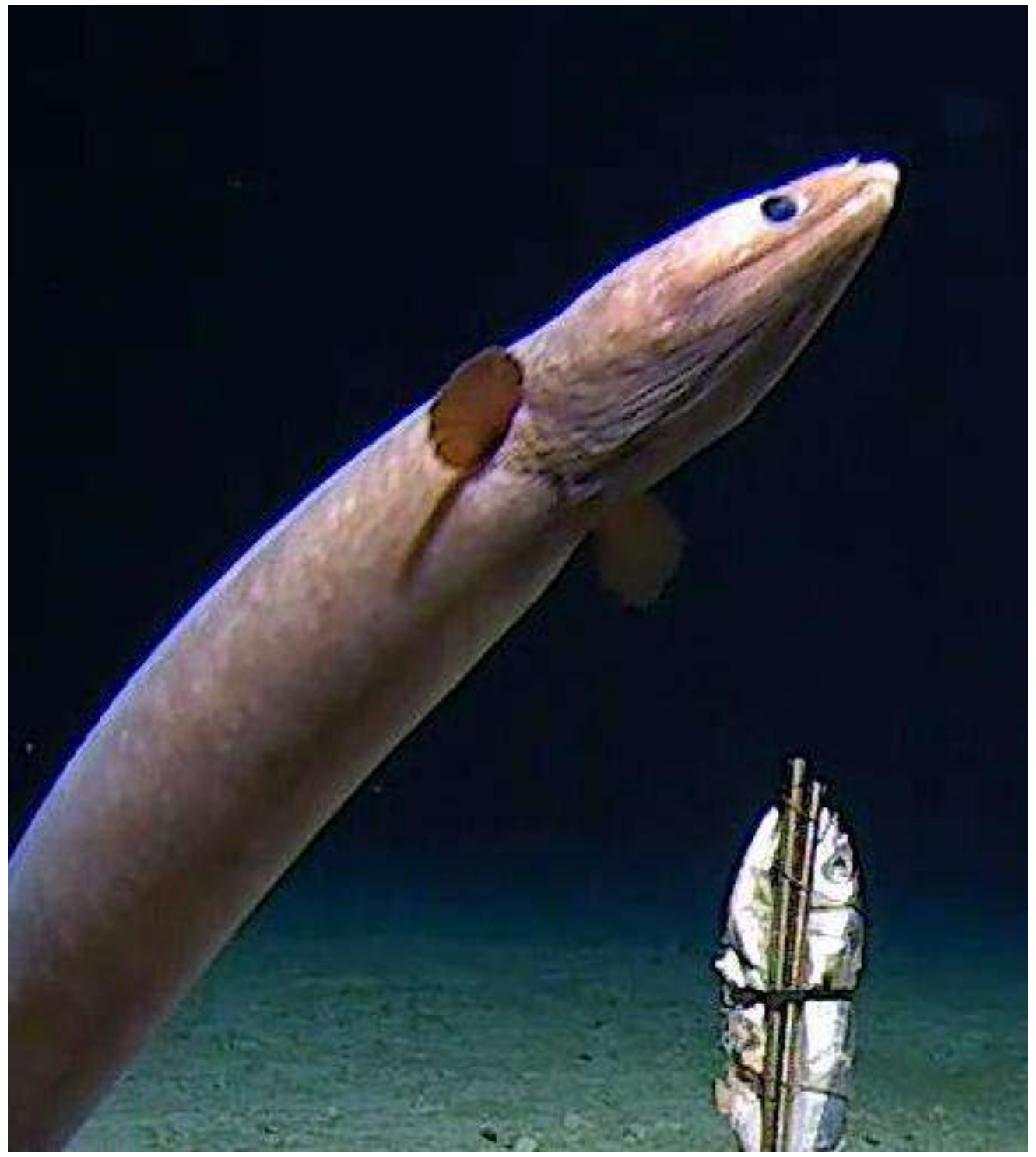

Pale, mottled pink in colour. Dorsal fin starts behind the pectoral fins and extends to the square tail fin. Anal fin extends to the tail fin. Body is elongated and tapers from the robust head and narrow snout. Slightly down curved snout tip projects beyond tip of lower jaw. *Notably larger and more robust than other Ilyophis species.*

Teleostei; Anguilliformes Synaphobranchidae ; *Ilyophis; Ilyophis robinsae*

NC3_OM6_4600_NOR_0040

NC3_OM6_4600_NOR_0041

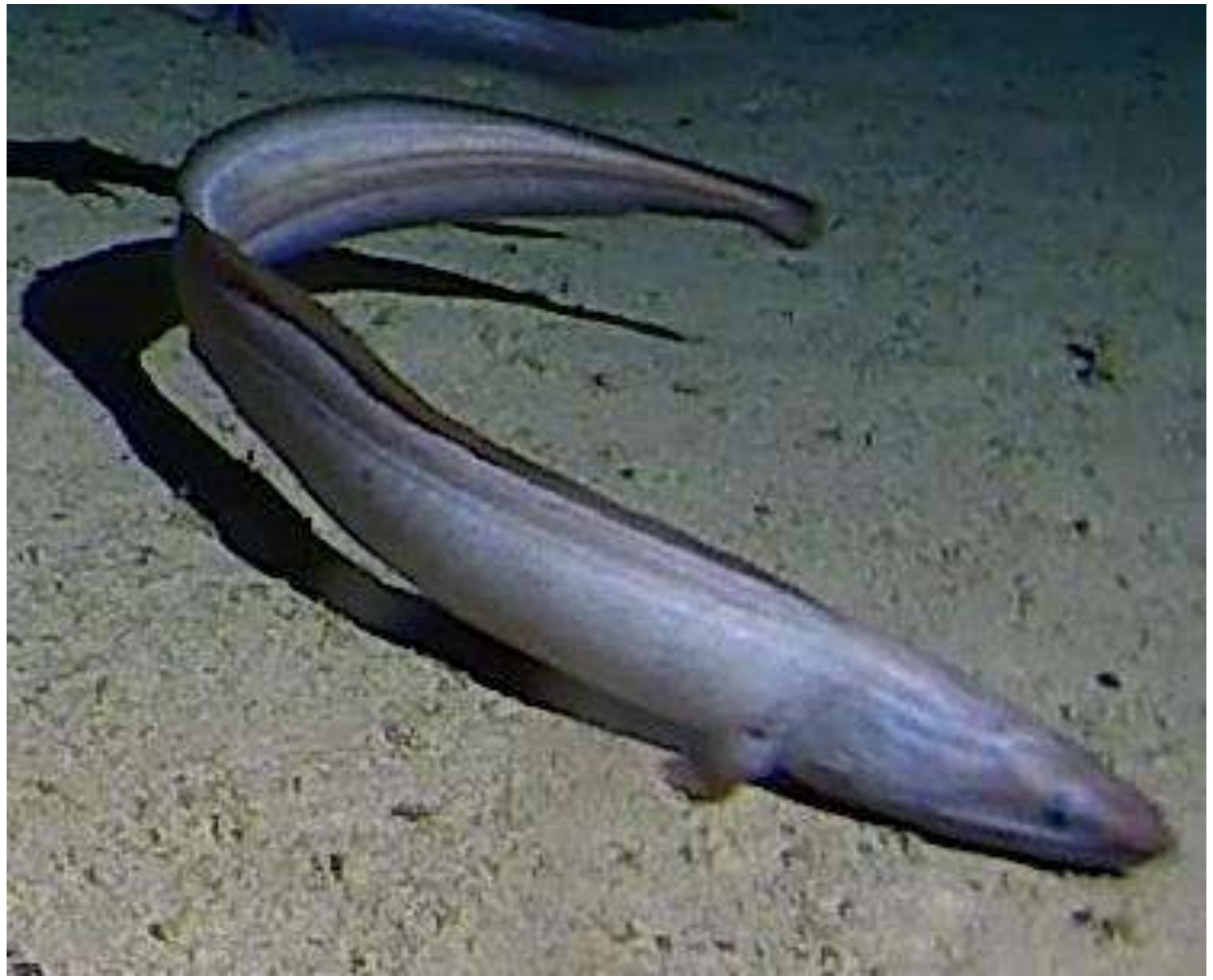

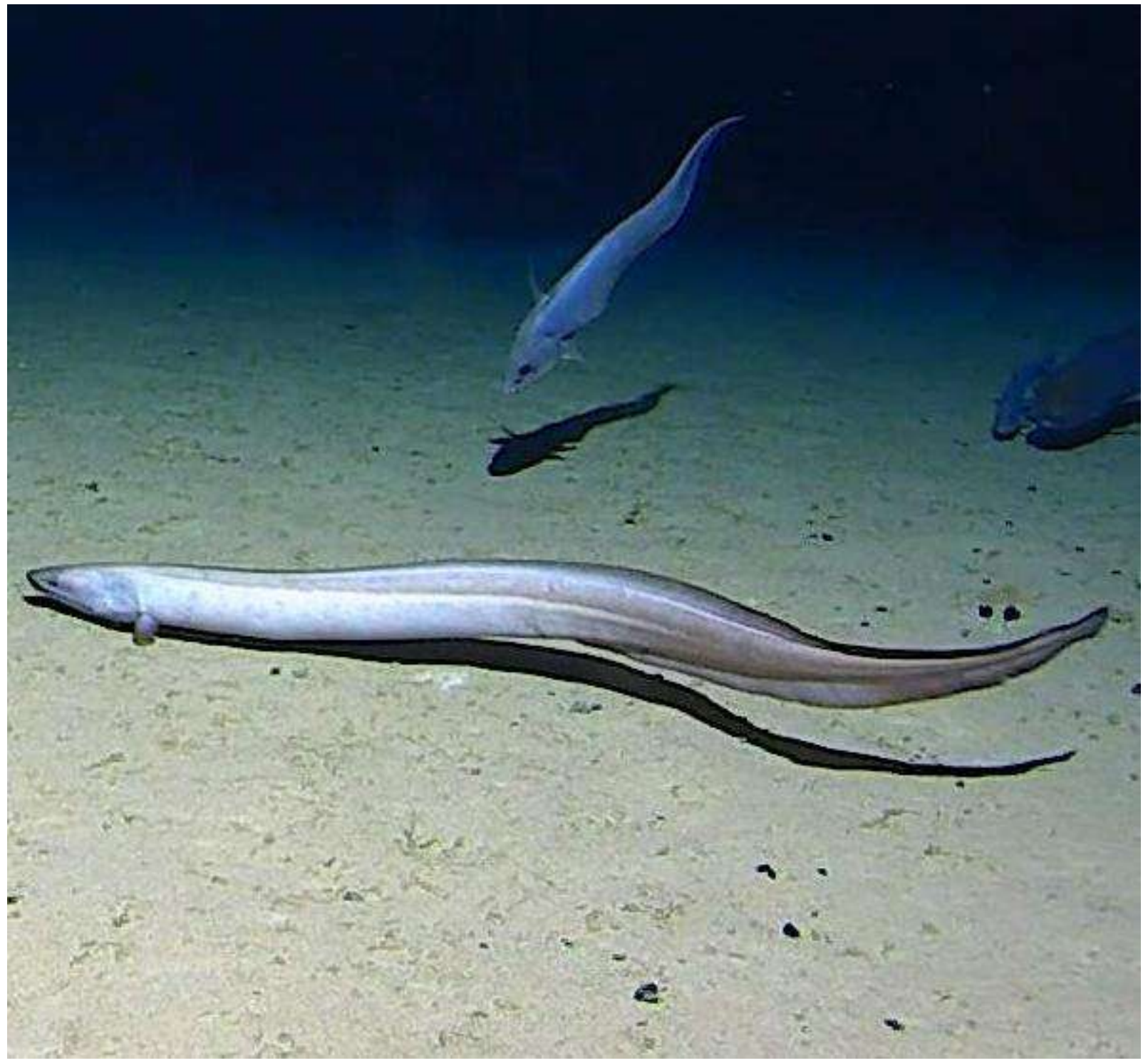

Pale in colour. Dorsal fin starts above the pectoral fins and extends to the square tail fin. Anal fin extends to the tail fin. Body is elongated and tapers from the narrow snout. Fleshy snout tip projects beyond lower jaw.

Teleostei; Anguilliformes Synaphobranchidae ; *Ilyophis;* *Ilyophis arx*

NC3_OM7_3500_NOR_0065
NC3_OM7_3500_NOR_0065

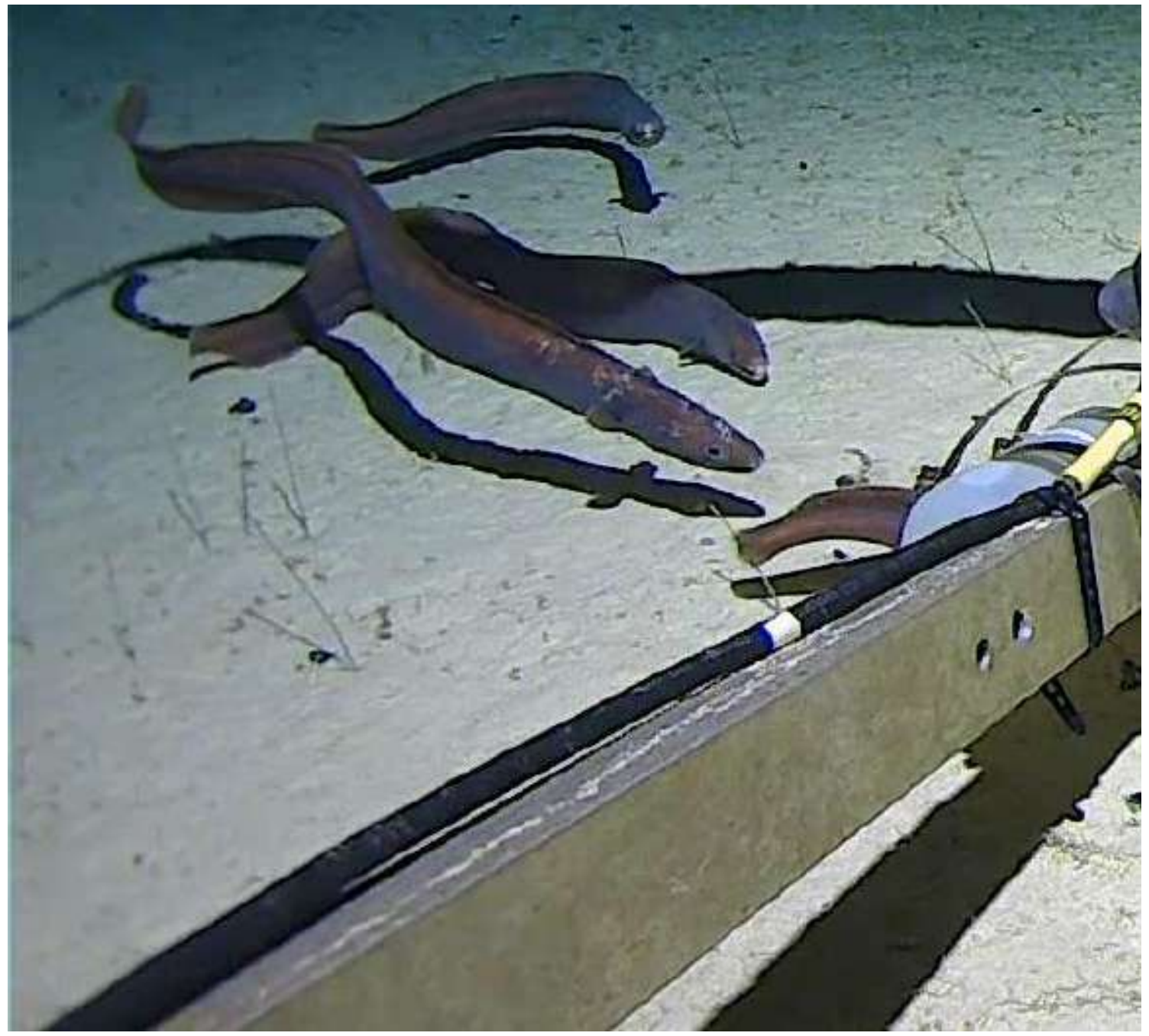
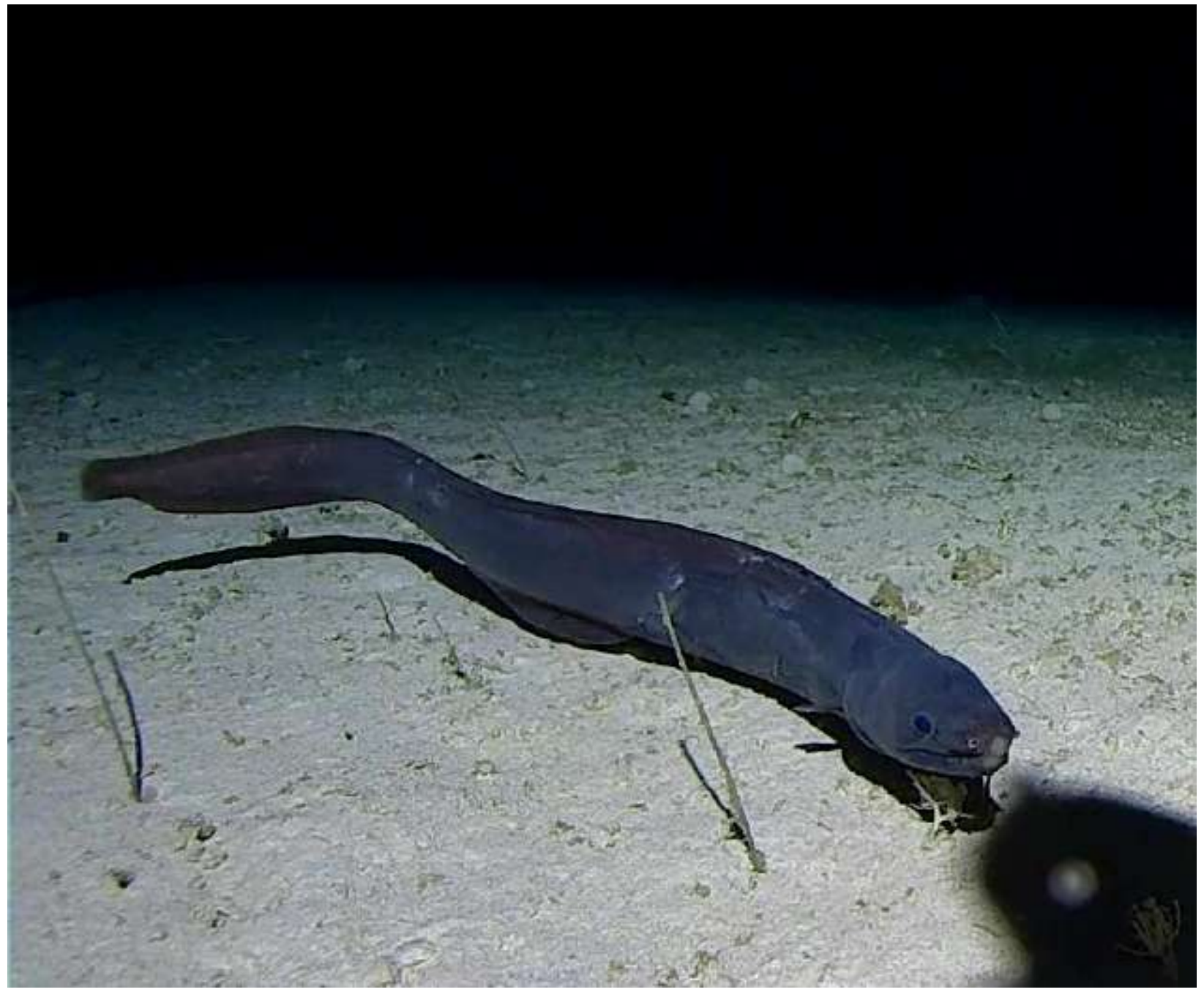

Skin is uniformly dark purple-brown in colouration. Dorsal fin starts above the pectoral fins and extends to the square tail fin. Anal fin extends to the tail fin. Body is elongated and tapers from the conical snout. Upper and lower jaws near equal in length.

Teleostei; Beryciformes
Stephanoberycidae ; *Abyssoberyx;*
Abyssoberyx sp. indet.1

NC3_BK3_6000_NOR_0080
NC4_MA9_6000_NOR_0080

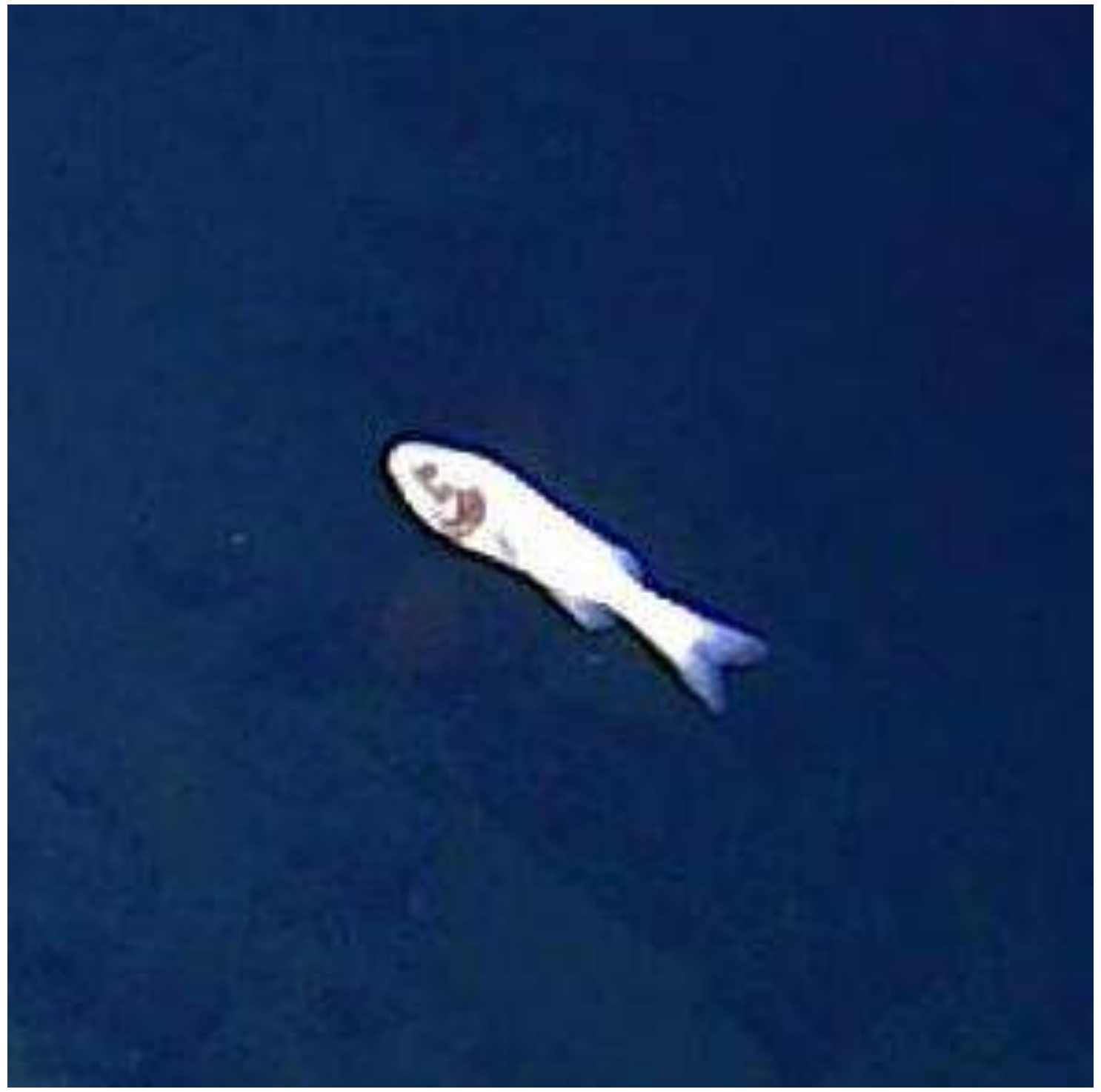

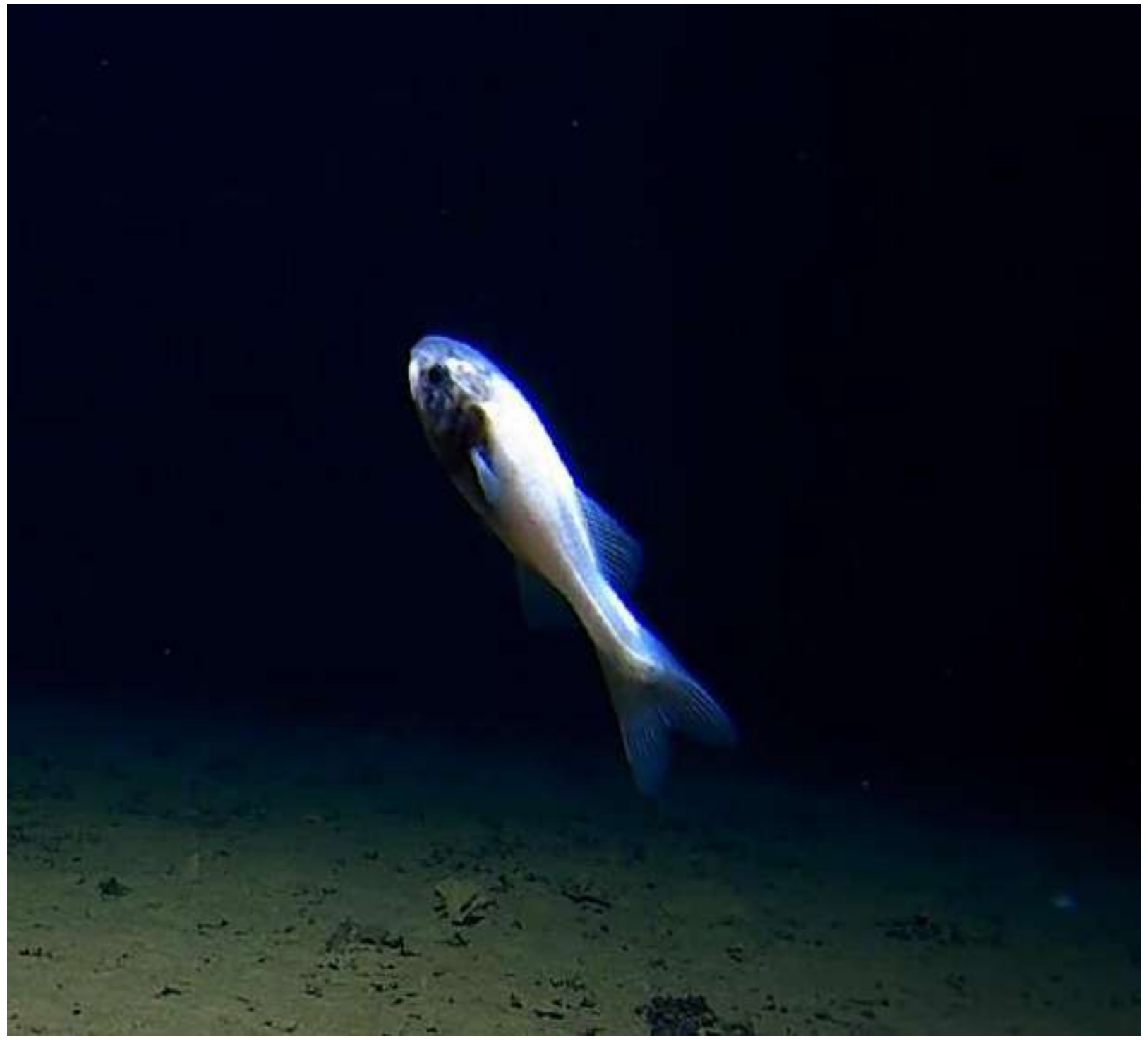

Skin is translucent. Eyes and gill cover appear dark. Dorsal and anal fins both triangular. Dorsal fin begins above the anal fin. Tail fin is forked with top and bottom lobes equal in length.

Teleostei; Ateleopodiformes
Ateleopodidae ; *Ateleopus; Ateleopus sp indet. 1*

NC4_MA11_5700_NOR_0043

NC4_MA11_5700_NOR_0044

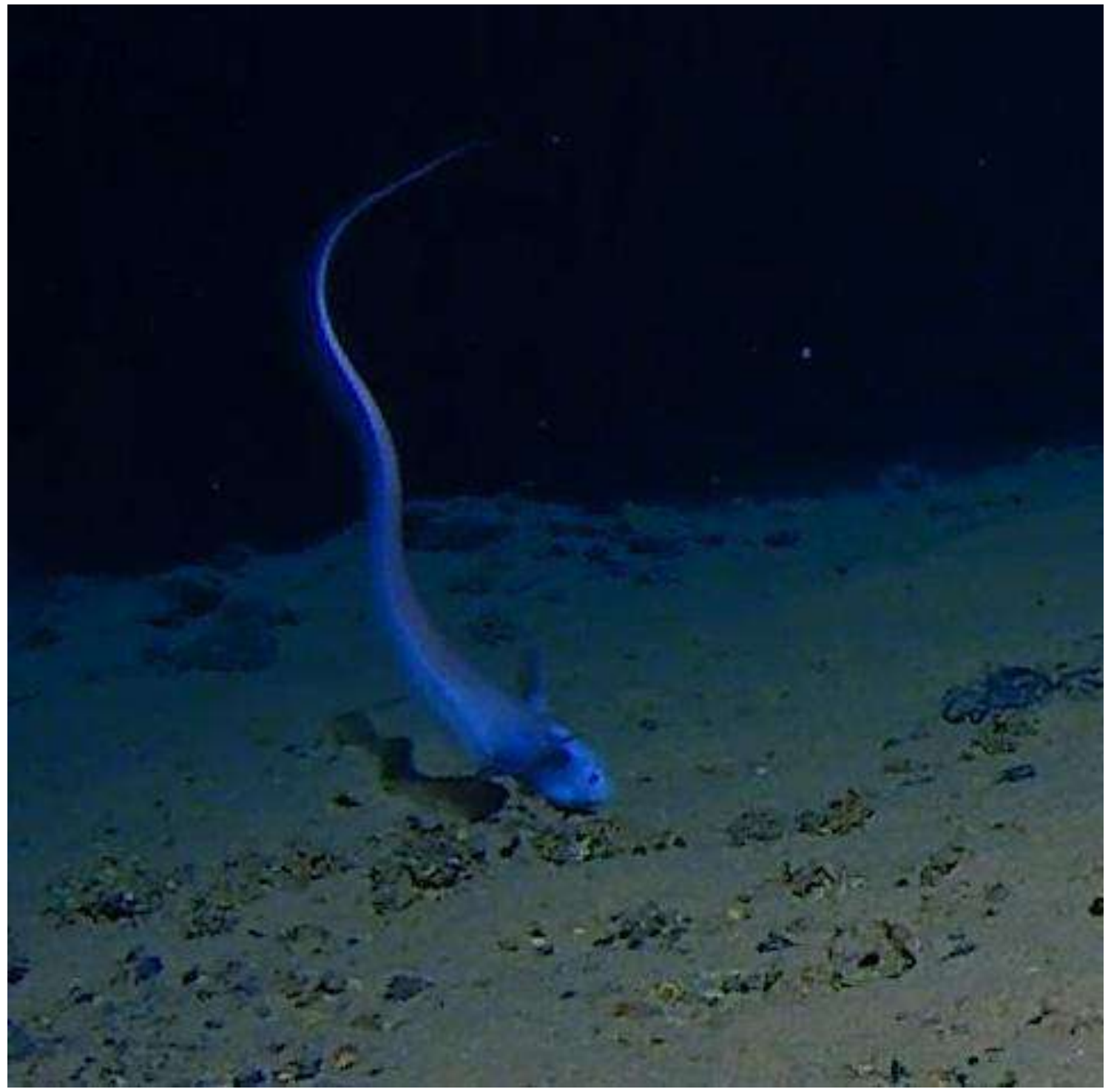

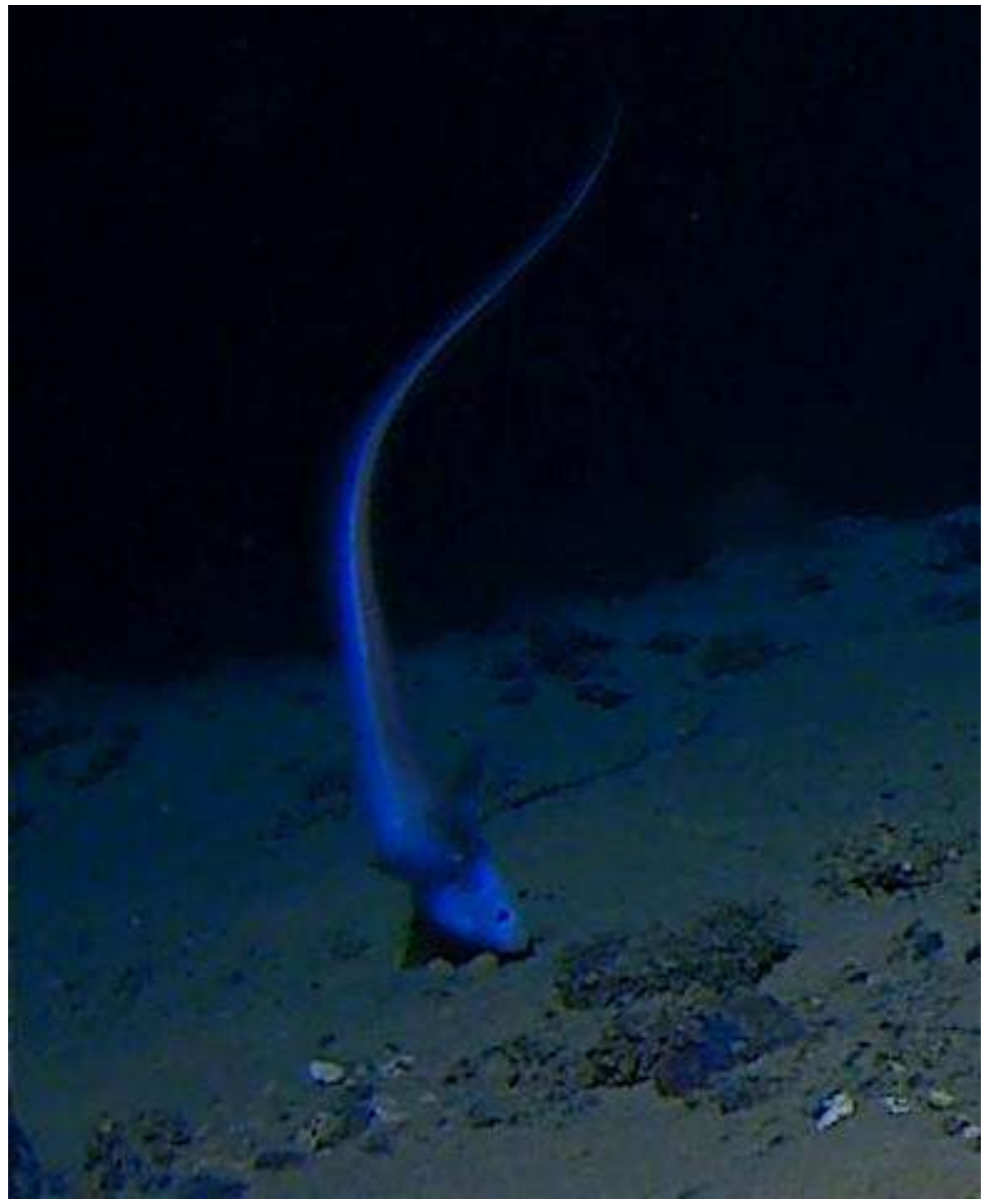

Anal fin extending along the tapered tail. Dorsal fin is positioned behind the head; large; triangular and high. Pectoral fins are relatively large and rounded. The snout is rounded and bulbous.

Teleostei; Aulopiformes
Ipnopidae ; *Bathysaurus; Bathysaurus mollis*

NC2_BK2_3800_IMG_7799
NC2_BK2_3800_IMG_7801
NC4_MA3_4550_NOR_0103

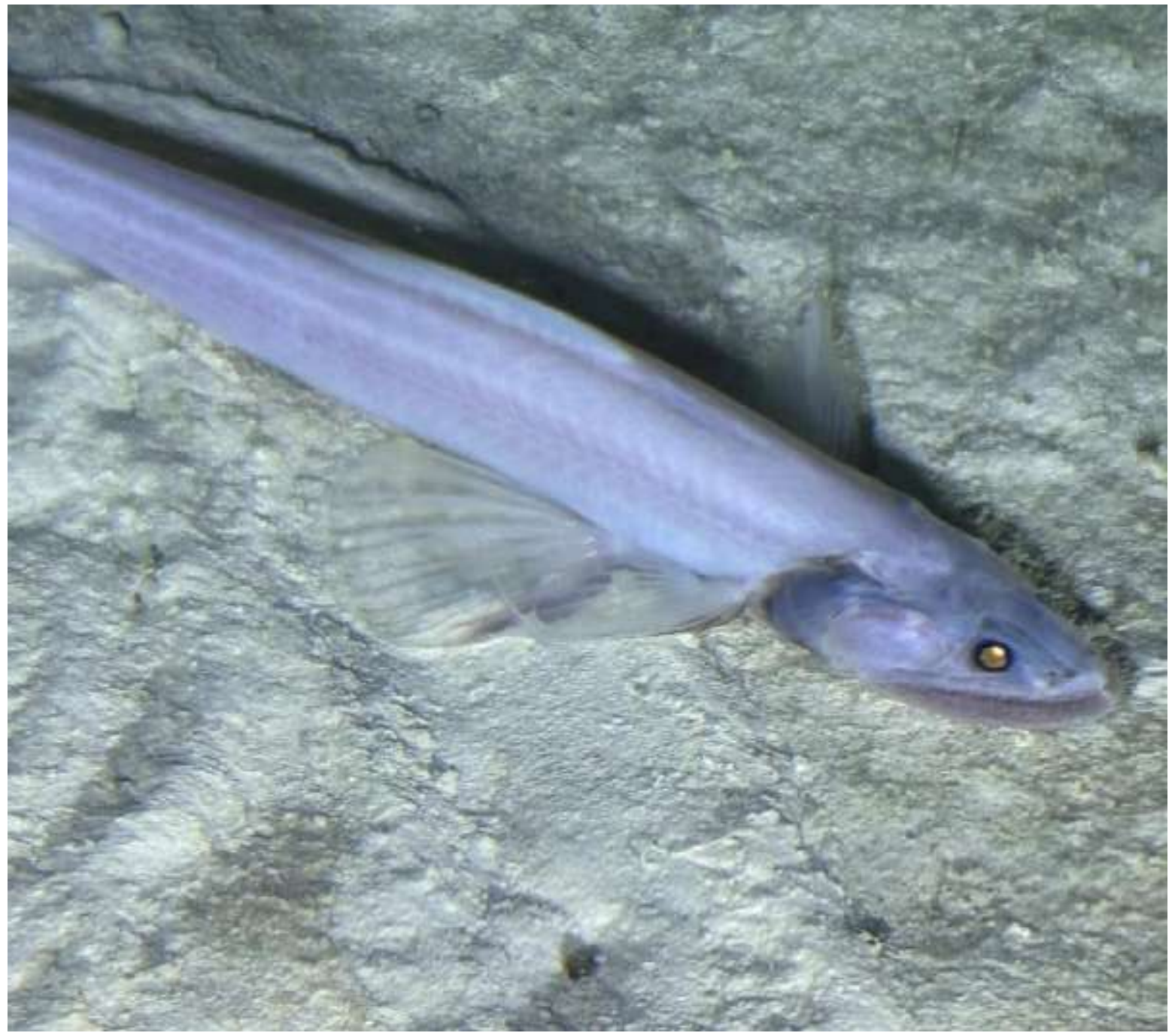
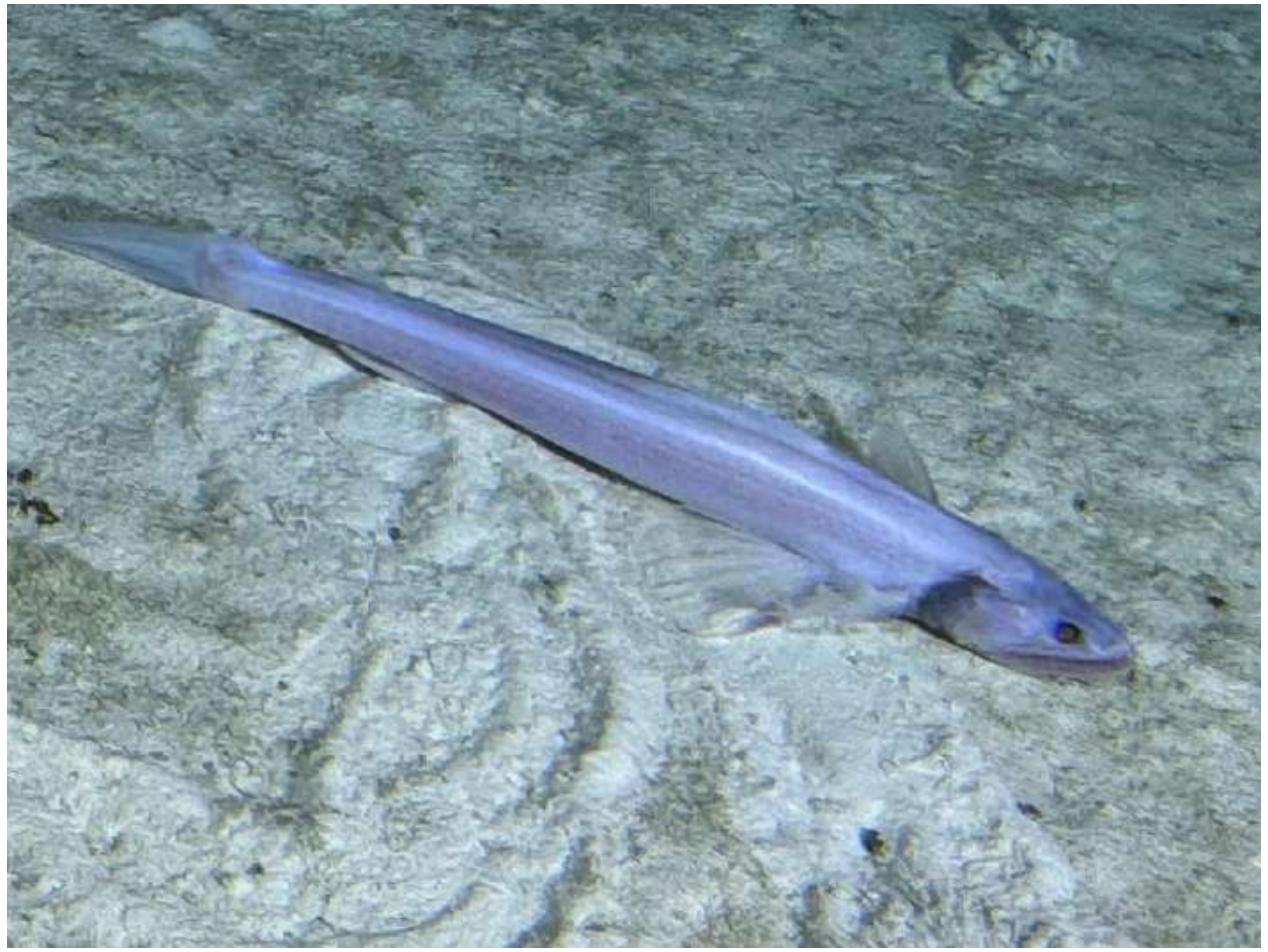
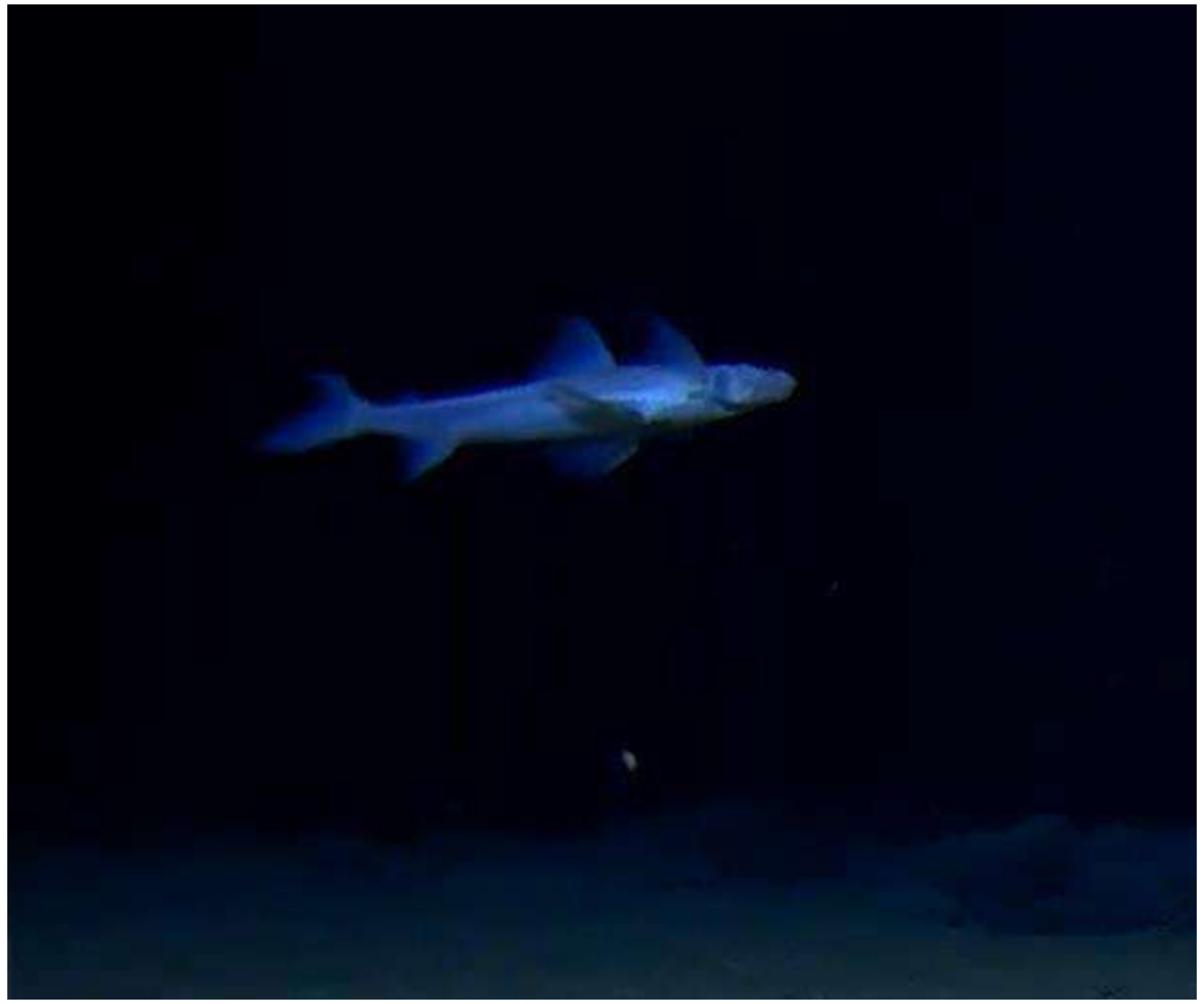

Uniform pale body. High, triangular dorsal and adipose fins. Wide, rounded pectoral fins and long pelvic fins. Anal fin begins roughly 3/4 length back. Tail fin is large and forked with lower fork longer than top. Eyes large and reflective on spoon shaped, flat head.

Teleostei; Aulopiformes
Ipnopidae ; *Bathypterois; Bathypterois atricolor*

NC2_BK1_3200_IMG_7470
NC2_BK1_3200_IMG_7476
NC2_BK1_3200_IMG_7487

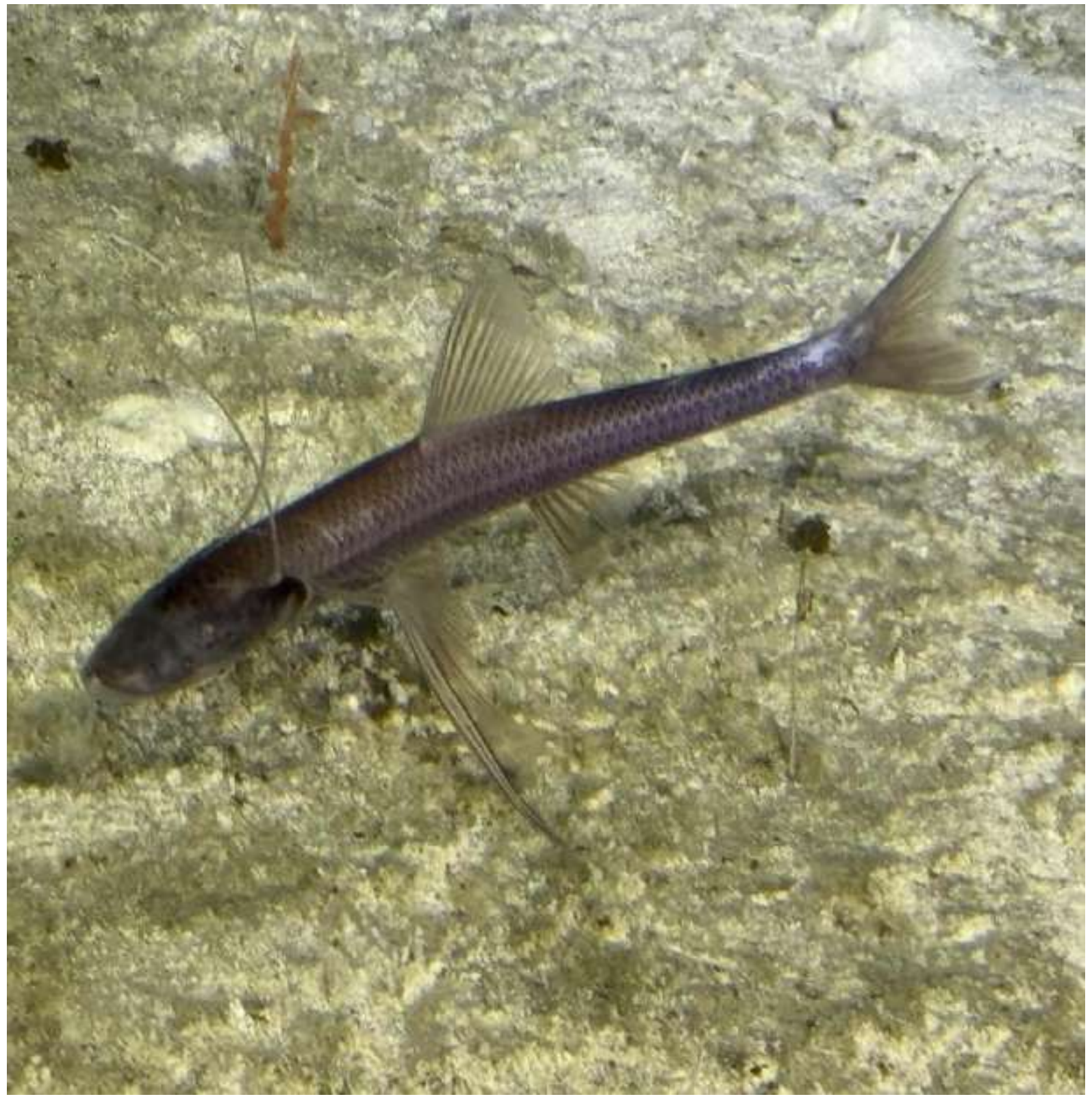
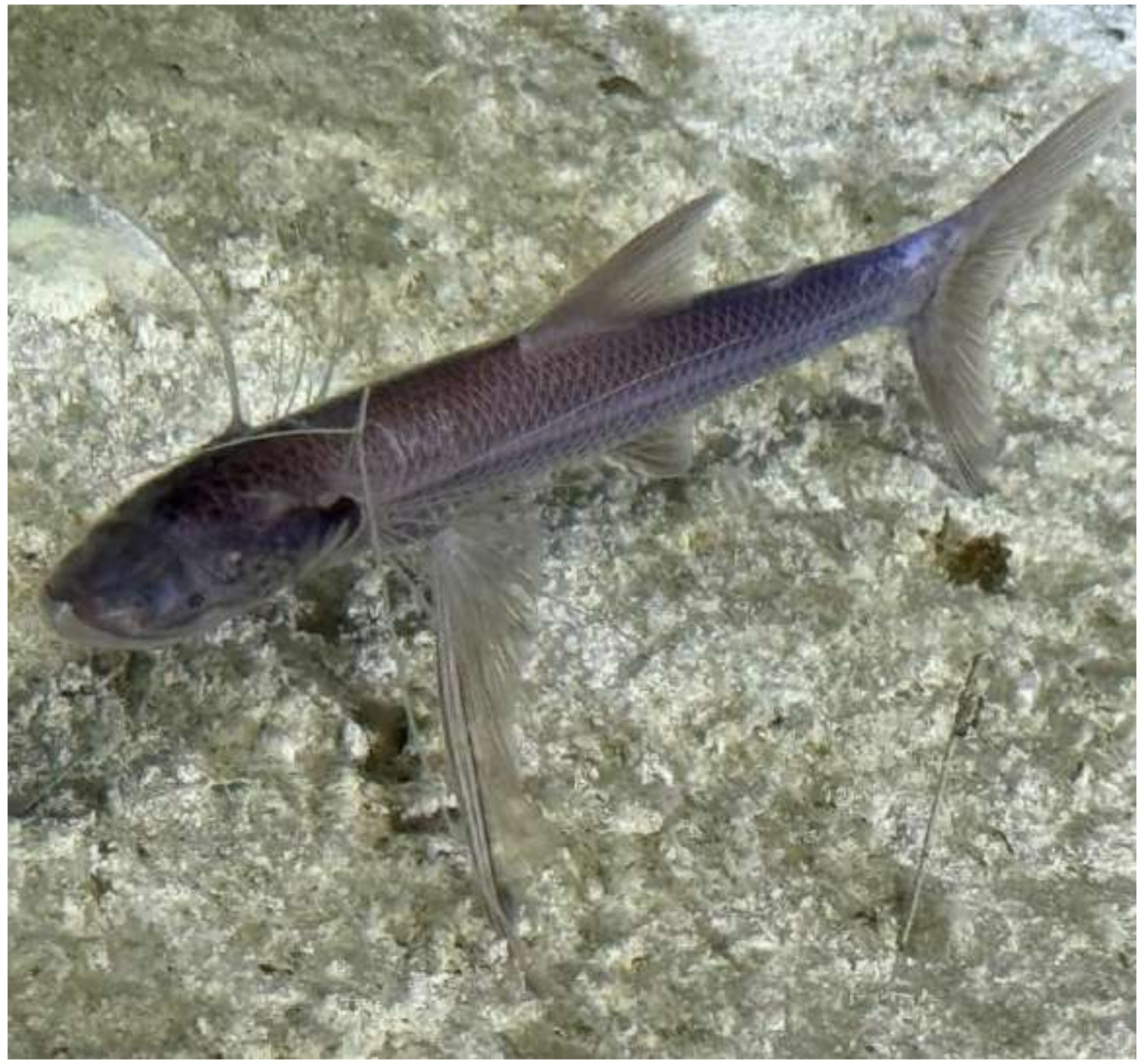
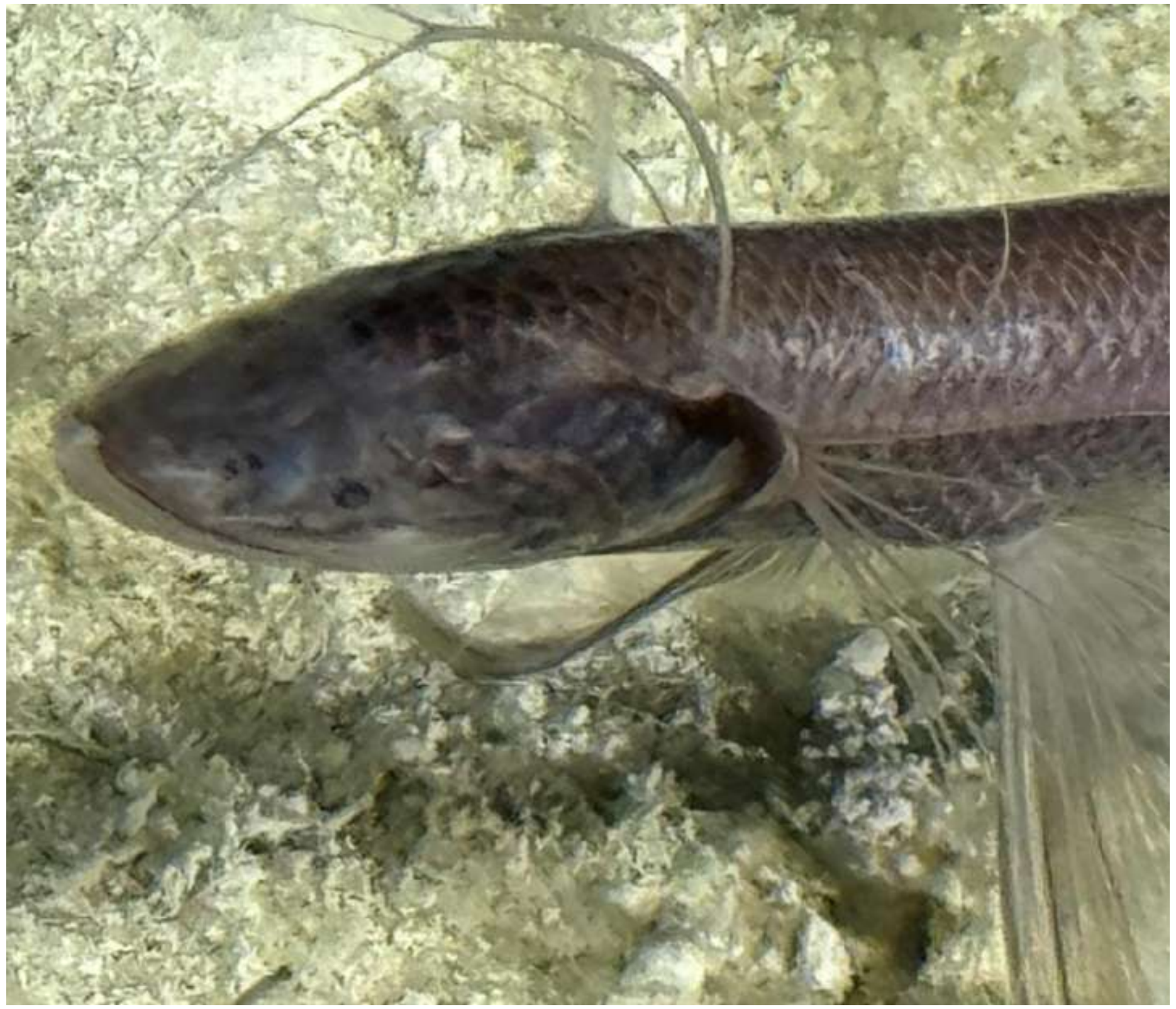

Uniform brown body. High, triangular dorsal, anal and caudal fins. Short pectoral fins and long pelvic fins. Anal fin begins roughly ½ length back. Tail fin is large and forked. Eyes small and vestigial on spoon shaped, flat head. Barbels extending forward from pectoral fins.

Teleostei; Aulopiformes
Ipnopidae ; *Bathyptyphlops;*
Bathyptyphlops marionae

NC2_BK1_3200_IMG_7766
NC2_BK1_3200_IMG_7777
NC2_BK2_3800_NOR_0071

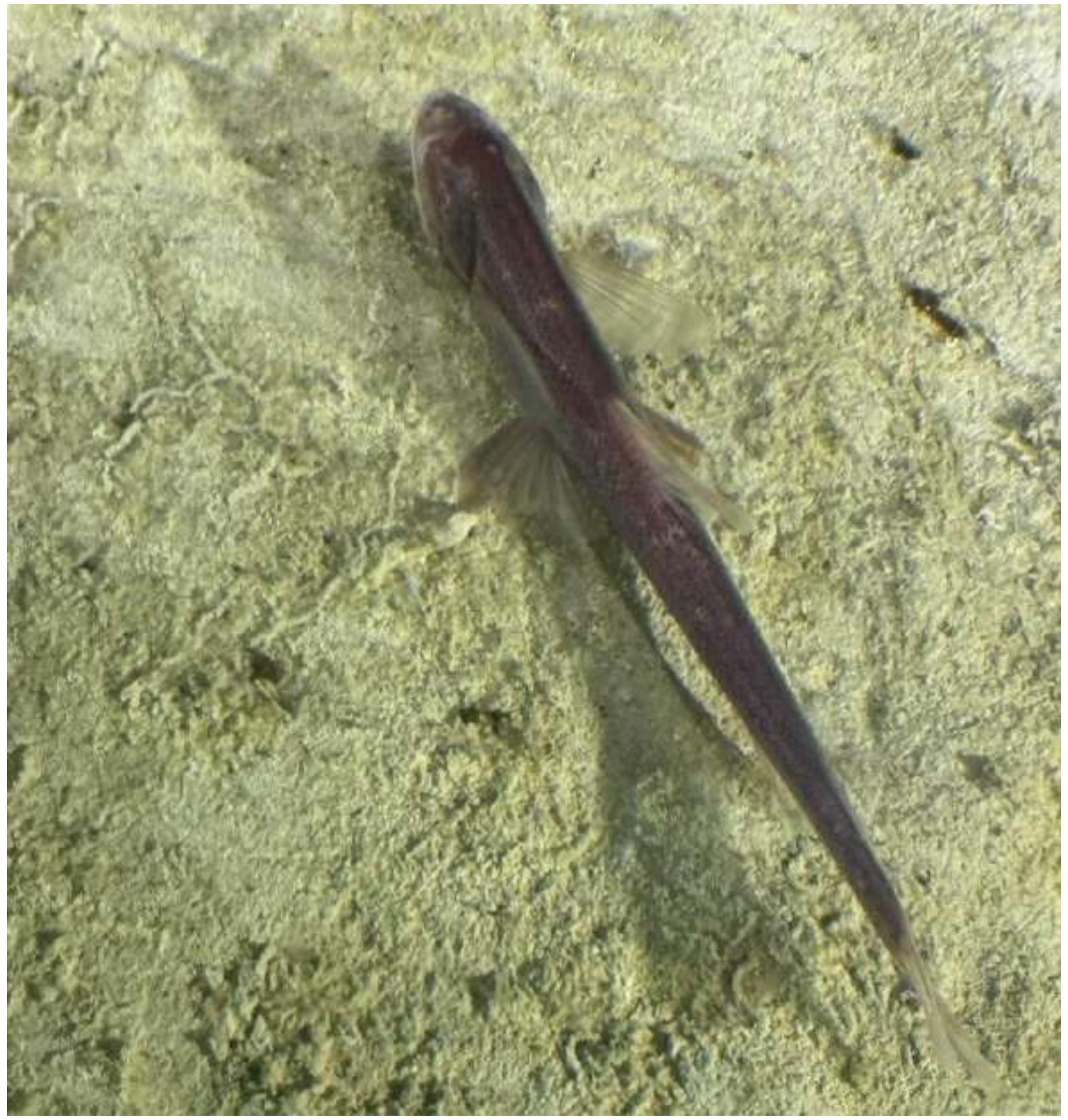
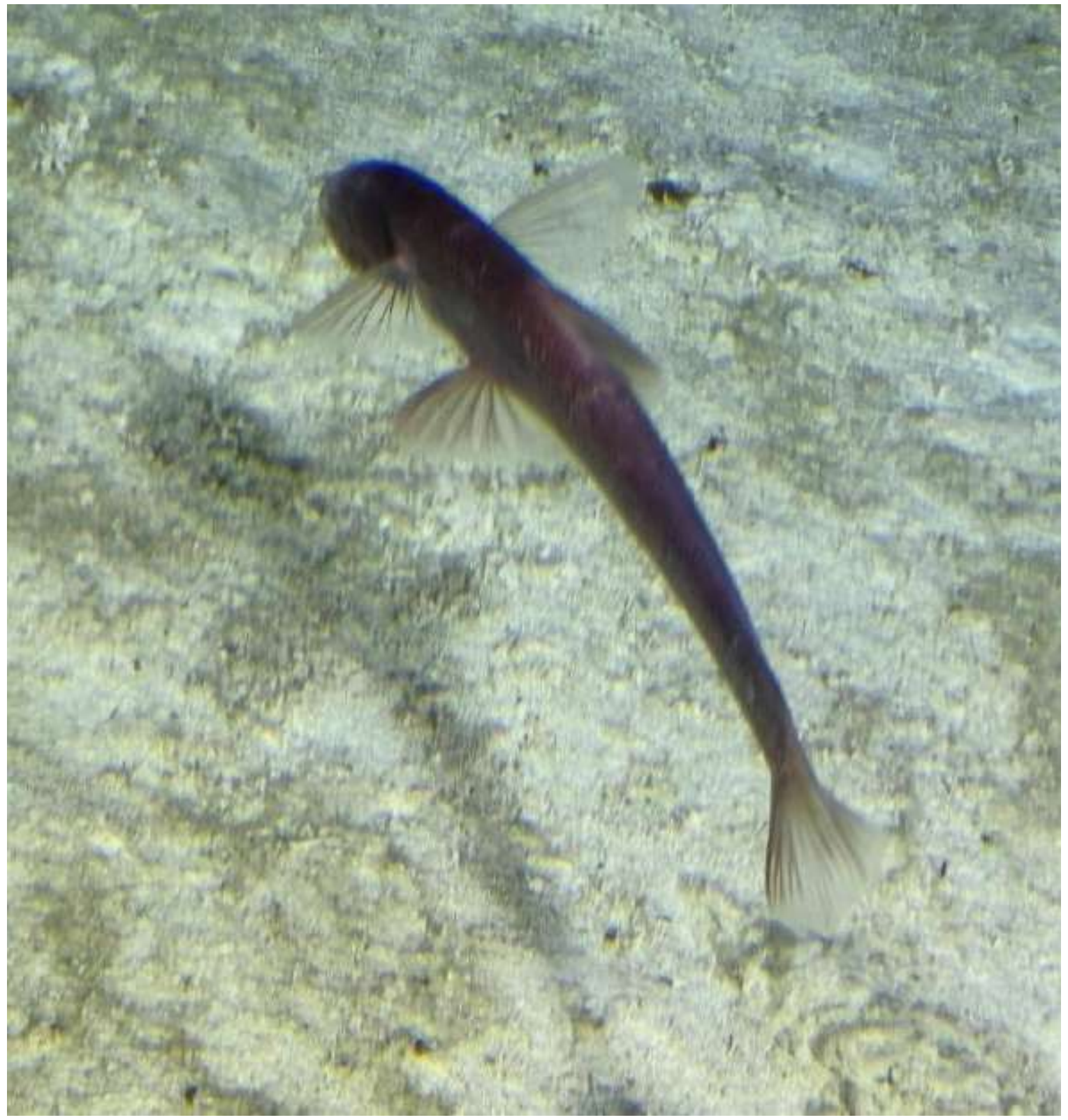
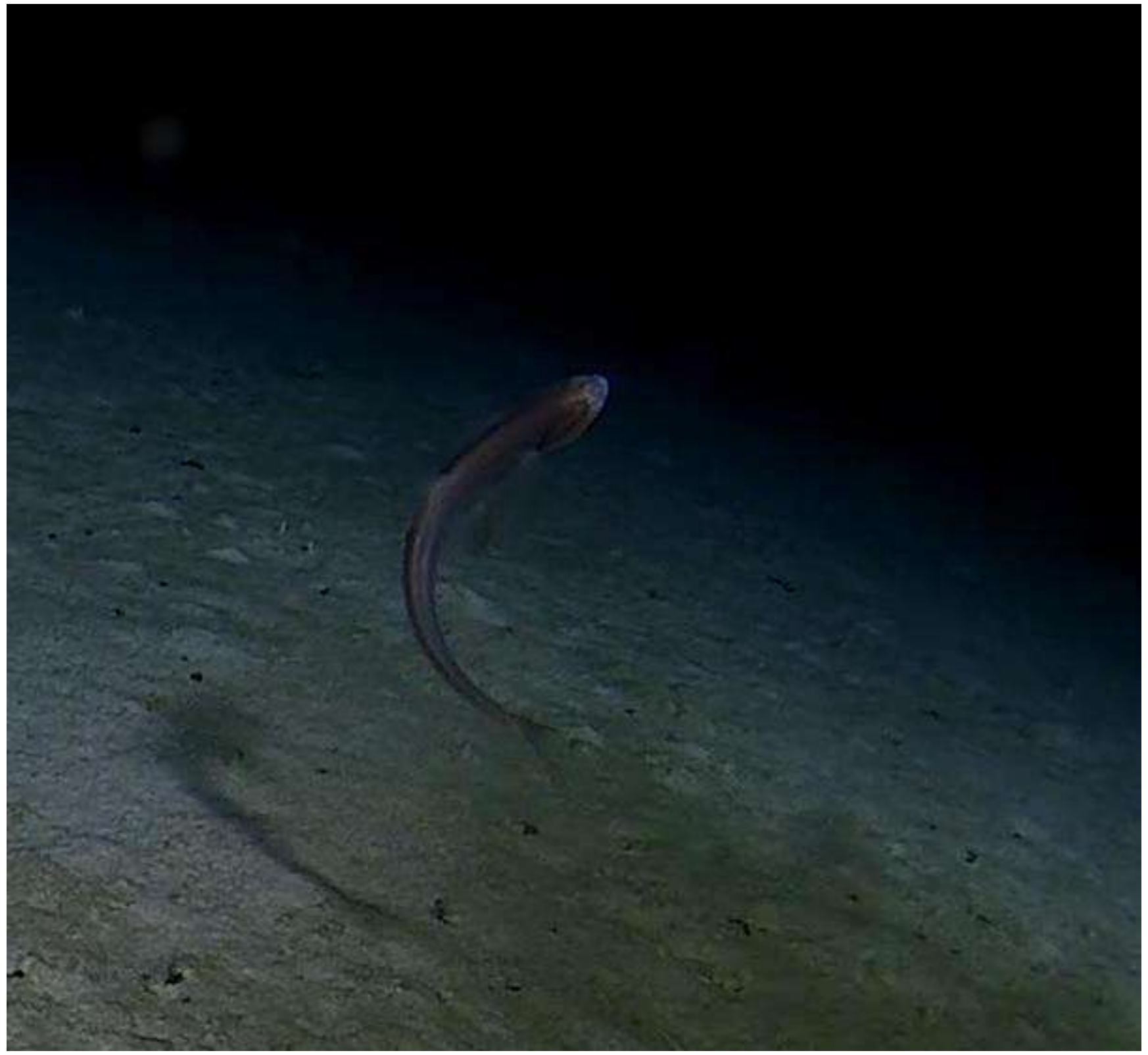

Uniform brown body. High, triangular dorsal, anal and caudal fins. Anal fin begins less than roughly ½ length back. Tail fin is forked. Eyes small and vestigial on spoon shaped, flat head.

NC4_BK2_4700_IMG_5304

Teleostei; Aulopiformes
Ipnopidae ; *Ipnops; Ipnops* spp.

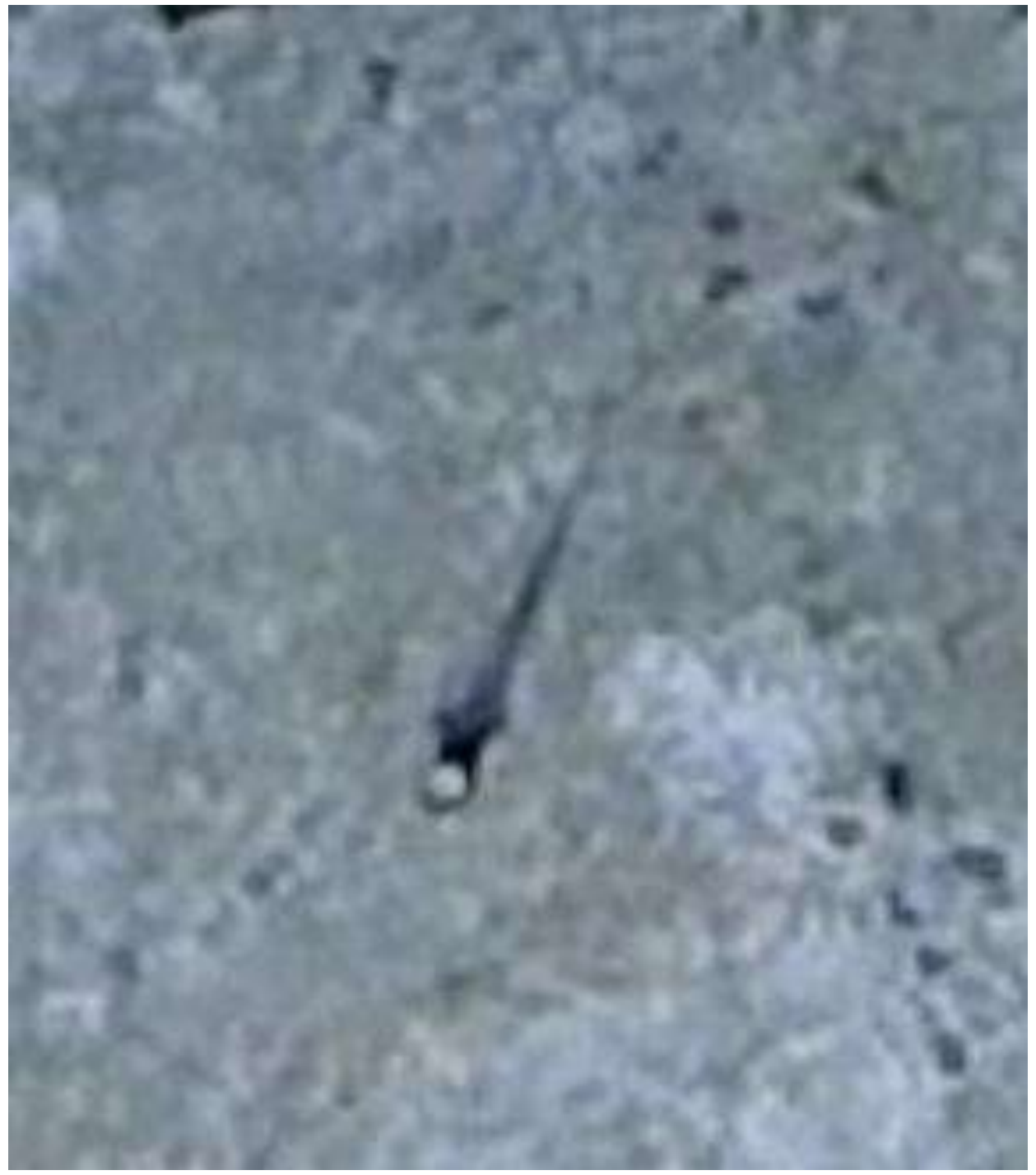

Greyish brown body with dark brownish purple head. Tail fin appears elongated and eel-like. Tail fin is forked. Eyes small and vestigial on spoon shaped, flat head.

Teleostei; Gadiformes
Macrouridae ; *Coryphaenoides;*
Coryphaenoides yaquinae

NC1_CR1_6200_NOR_0097
NC3_CR3_5500_NOR_0083
NC3_OM1_5600_NOR_0253

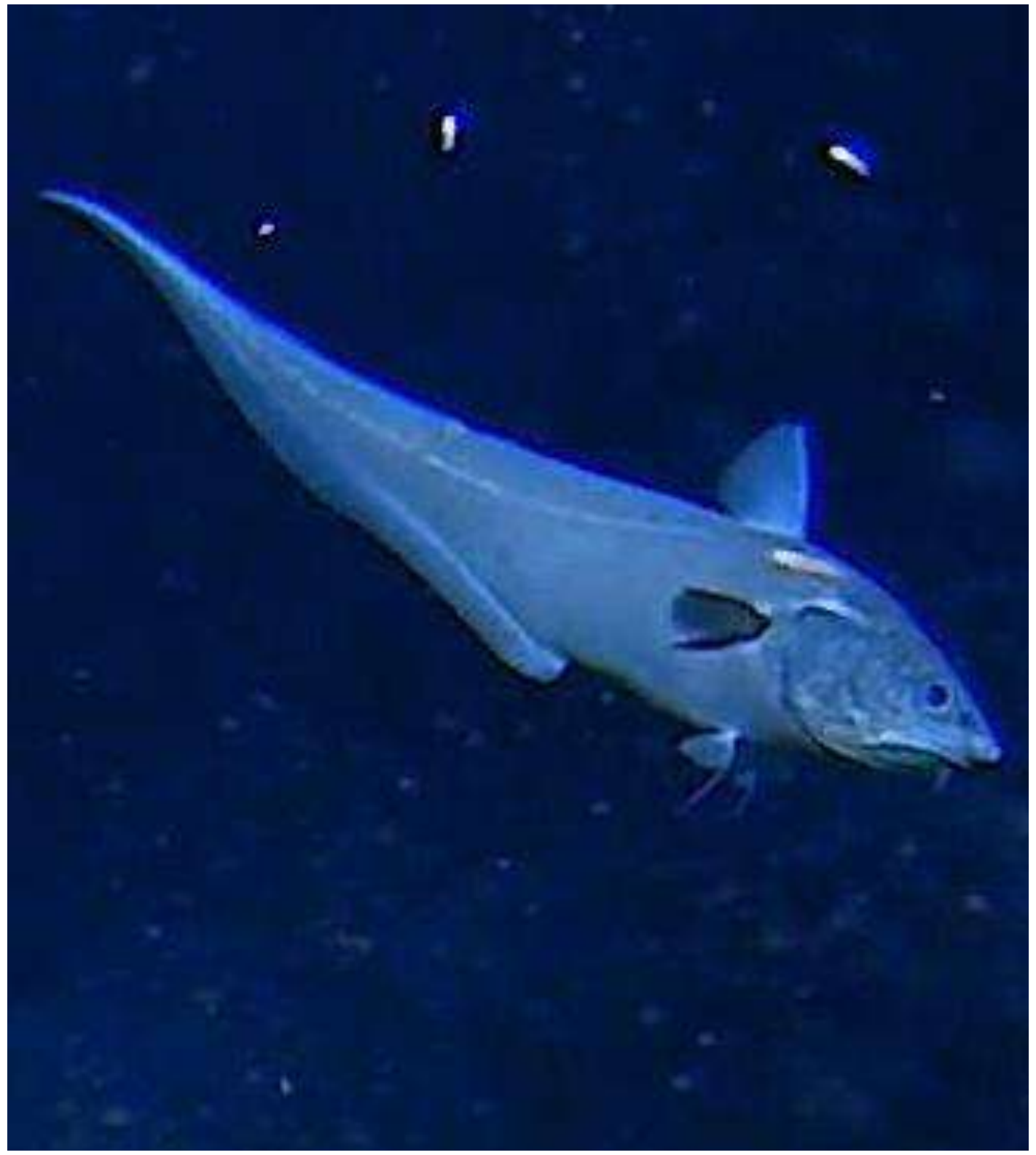

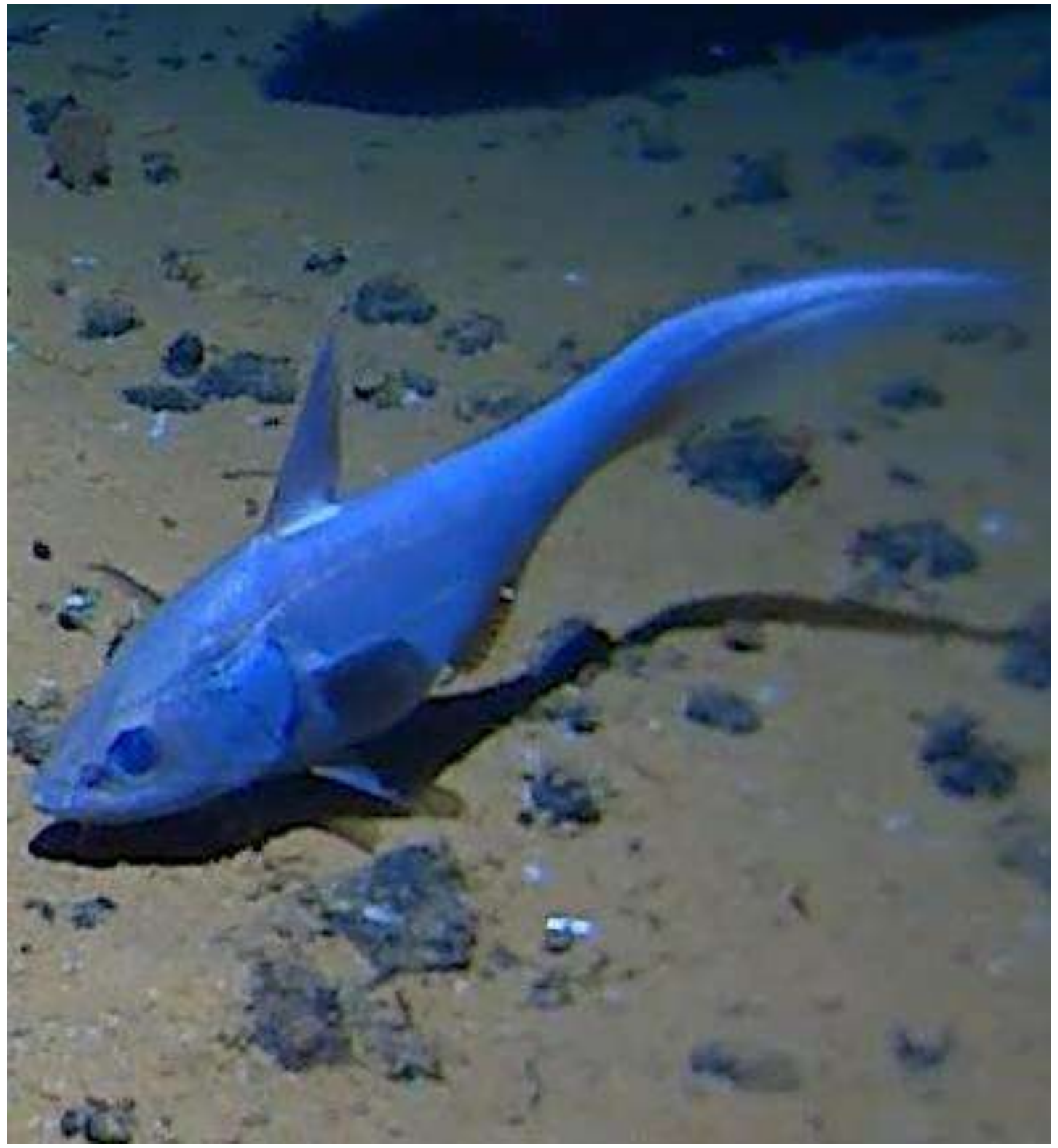

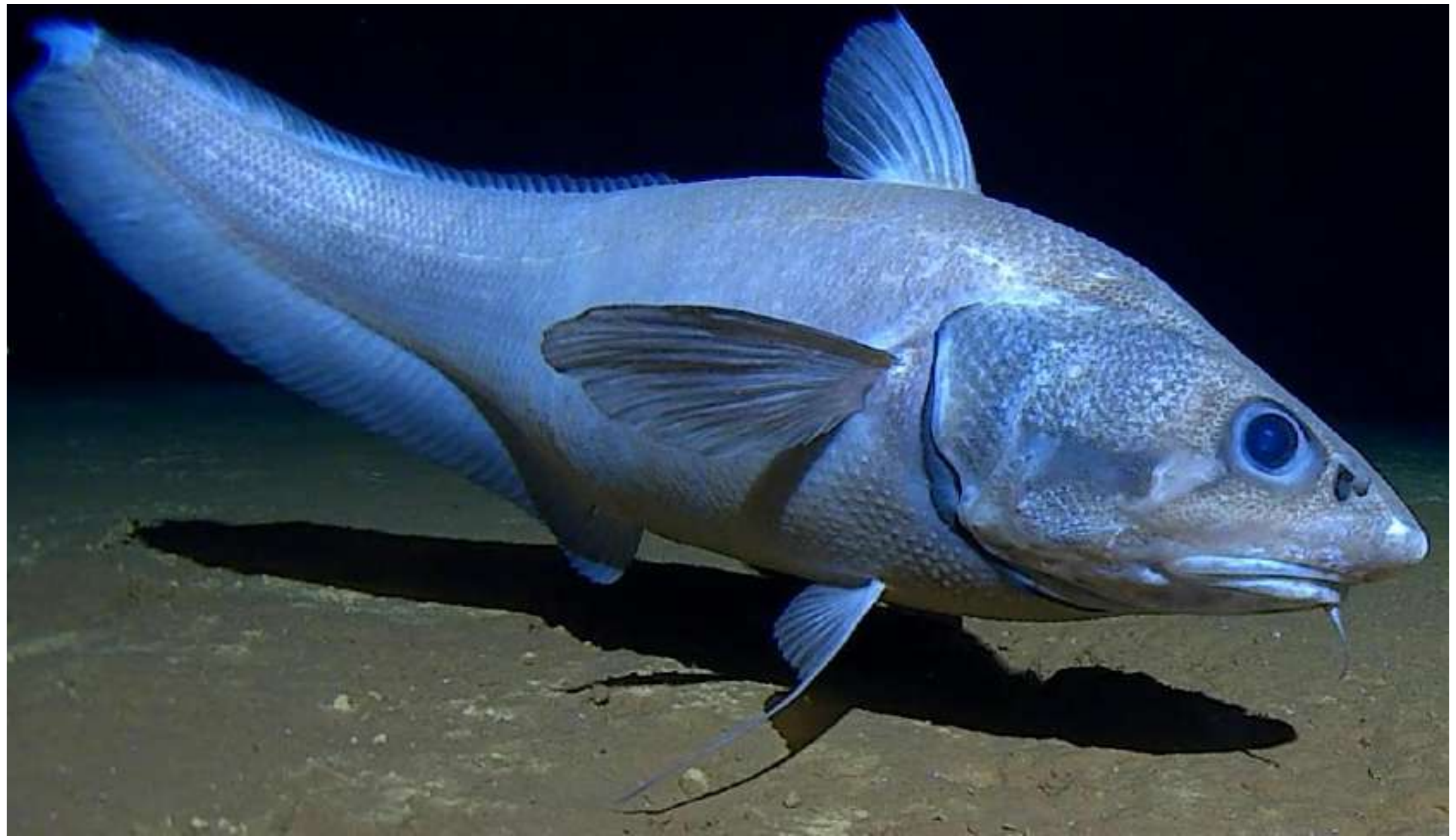

Uniform silvery body and rough skin texture. High, triangular dorsal fin, anal fin and caudal extend down the tapering tail. Anal fin begins less than roughly 1 head length back. Short, square, pelvic fins bear barbel-like protrusions. Large pectoral fins with rounded globular head, large eyes and pointed snout.

Teleostei; Gadiformes
Macrouridae ; *Coryphaenoides;*
Coryphaenoides rudis

NC3_OM8_2700_NOR_0130

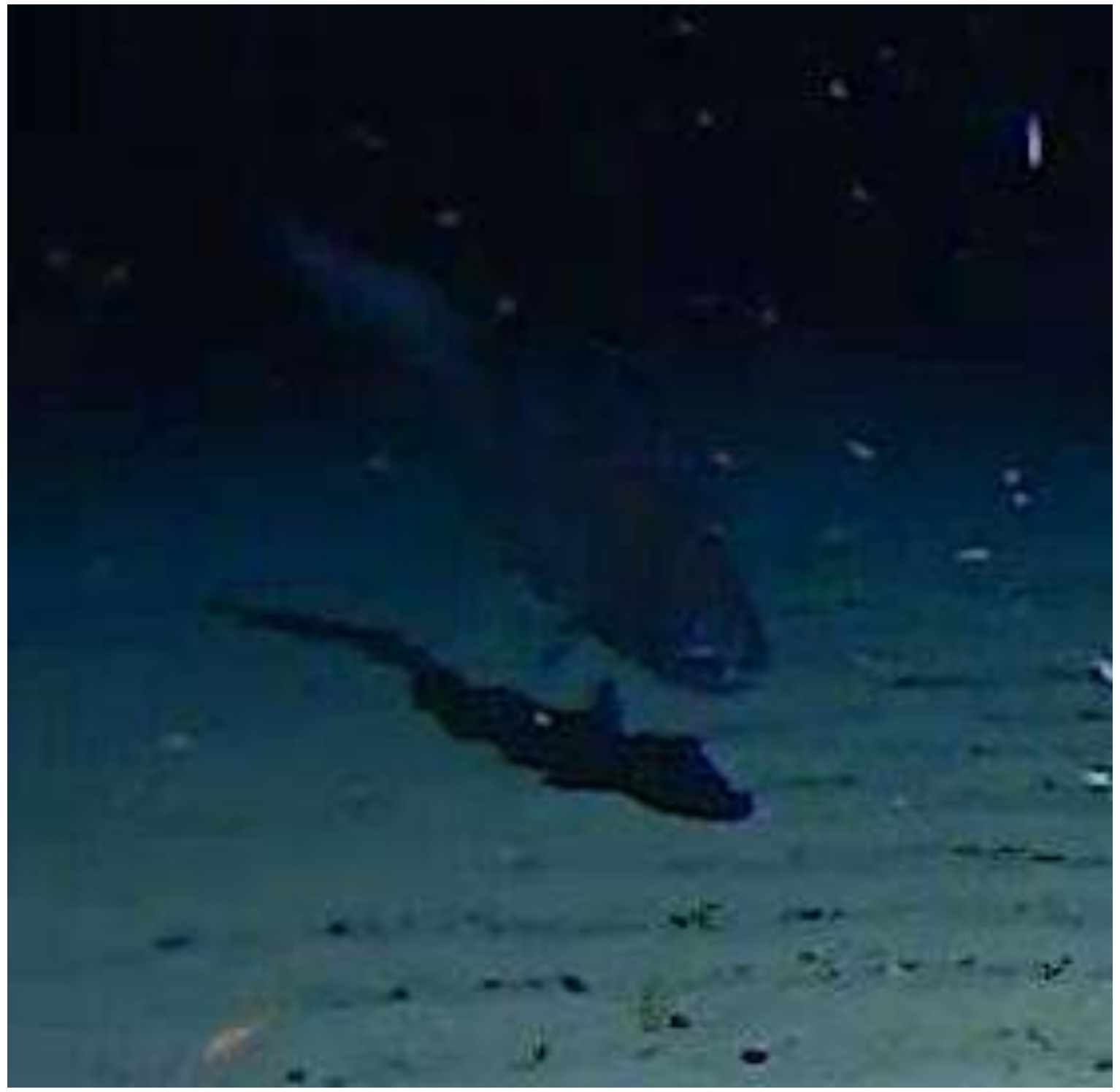

Uniform dark body and rough skin texture. High, triangular dorsal fin, anal fin and caudal extend down the tapering tail. Anal fin begins less than roughly 1 head length back. Short, square, pelvic fins bear barbel-like protrusions. Large pectoral fins with rounded globular head, large eyes and pointed snout.

Teleostei; Ophidiiformes
Acanthonidae; *Acanthonus; Acanthonus armatus*

NC3_OM8_2700_NOR_0101
NC3_OM8_2700_NOR_0101
NC3_MA8_2800_NOR_0121

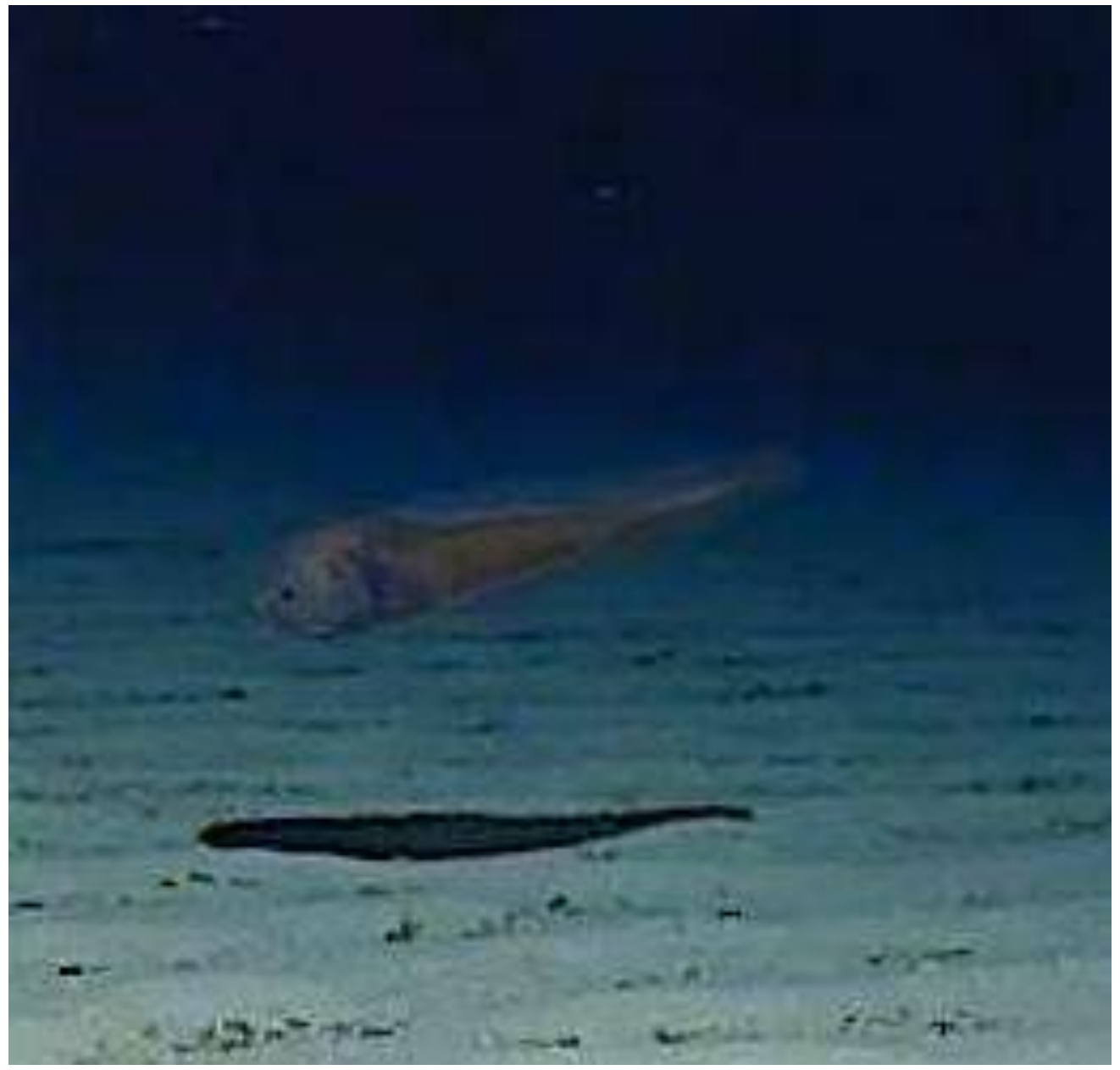 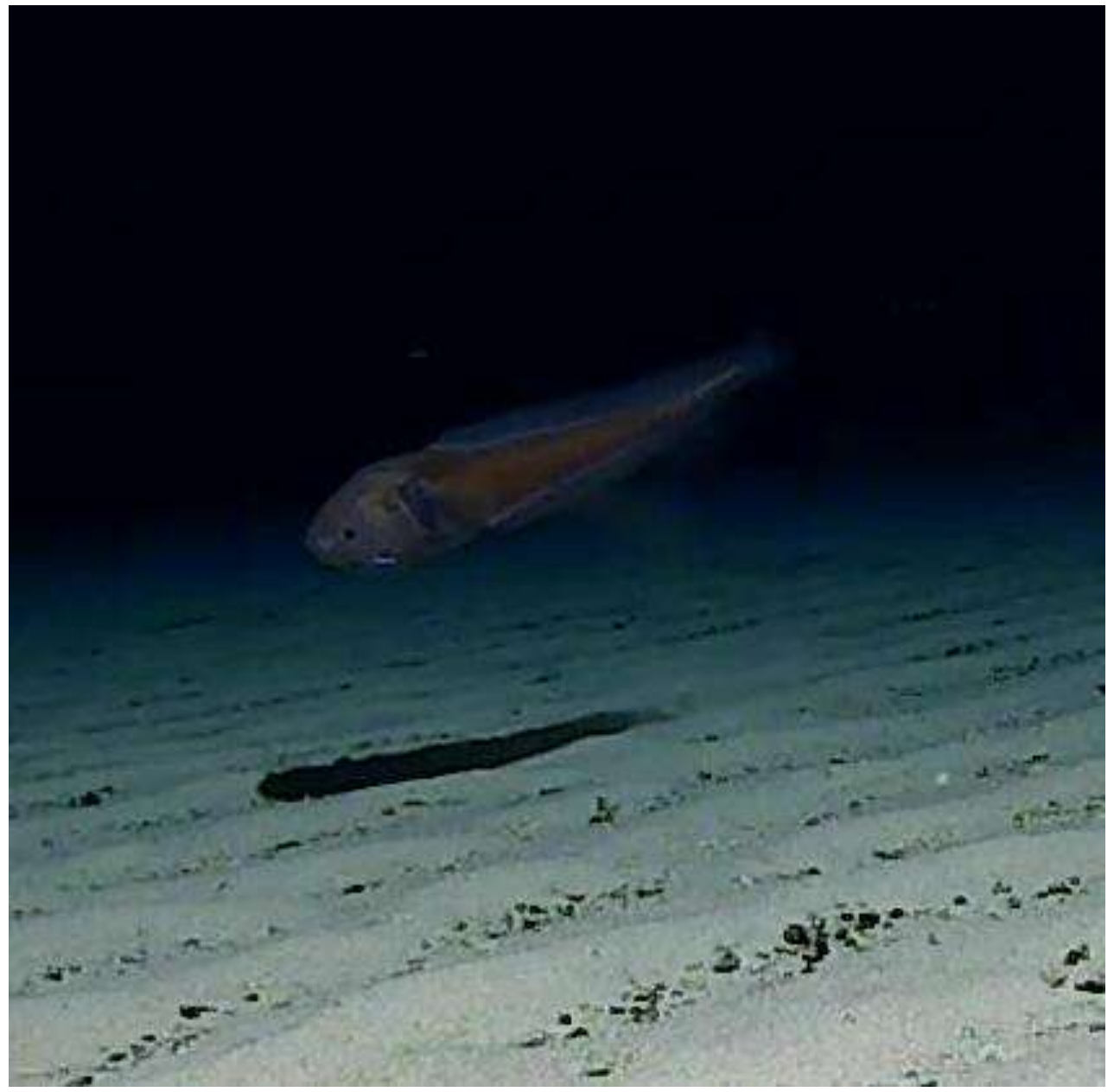 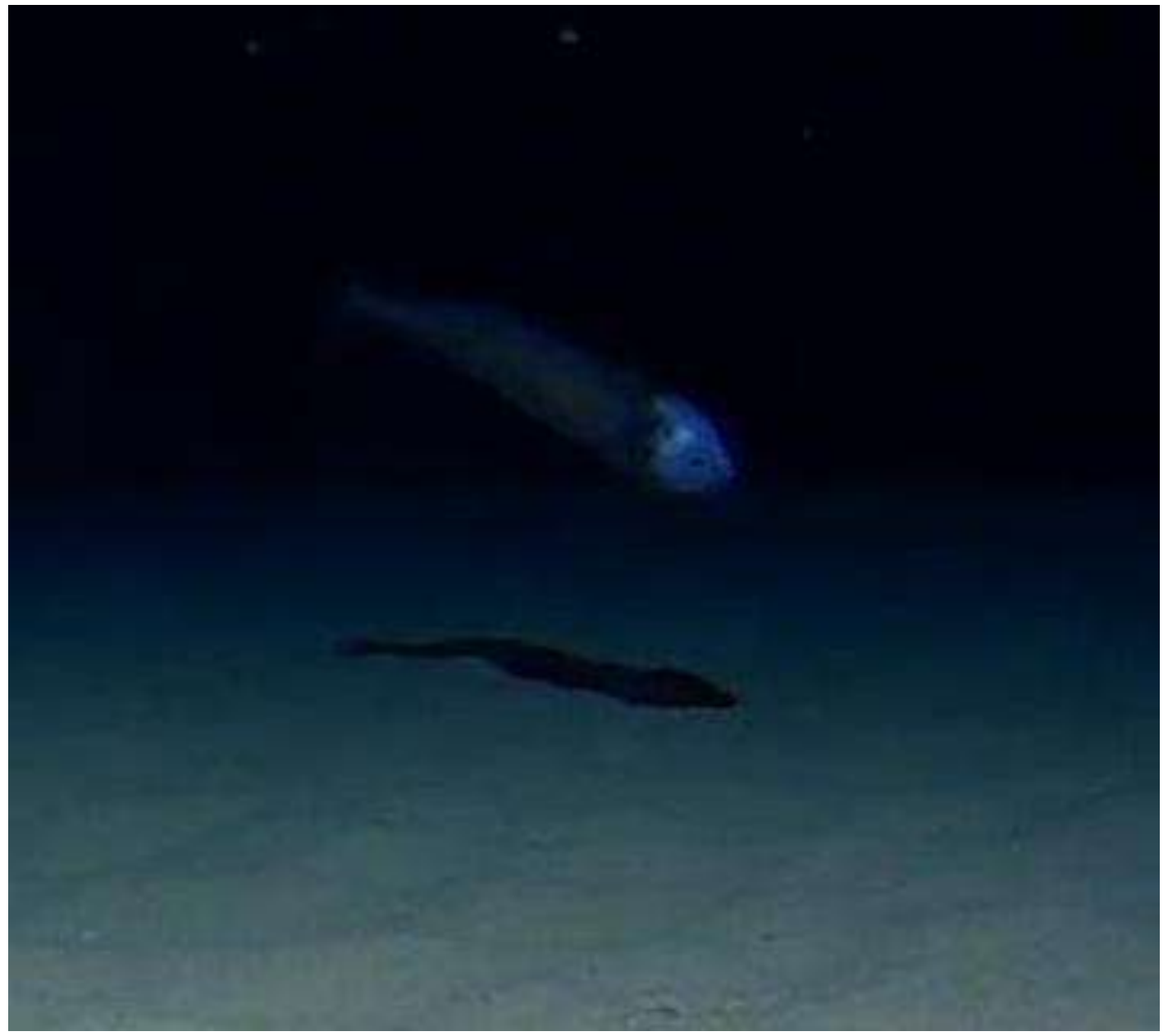

Brown coloured body with rounded, sometimes pale head. Transparent dorsal and anal fin extend to the tail with separate; rectangular tail fin. Anal fin begins less than roughly 3/4 head length back. Faint pectoral fins with rounded globular head and pointed snout.

Teleostei; Ophidiiformes
Ophidiidae; *Porogadus; Porogadus* sp. indet.1

NC2_CR3_3800_NOR_0068

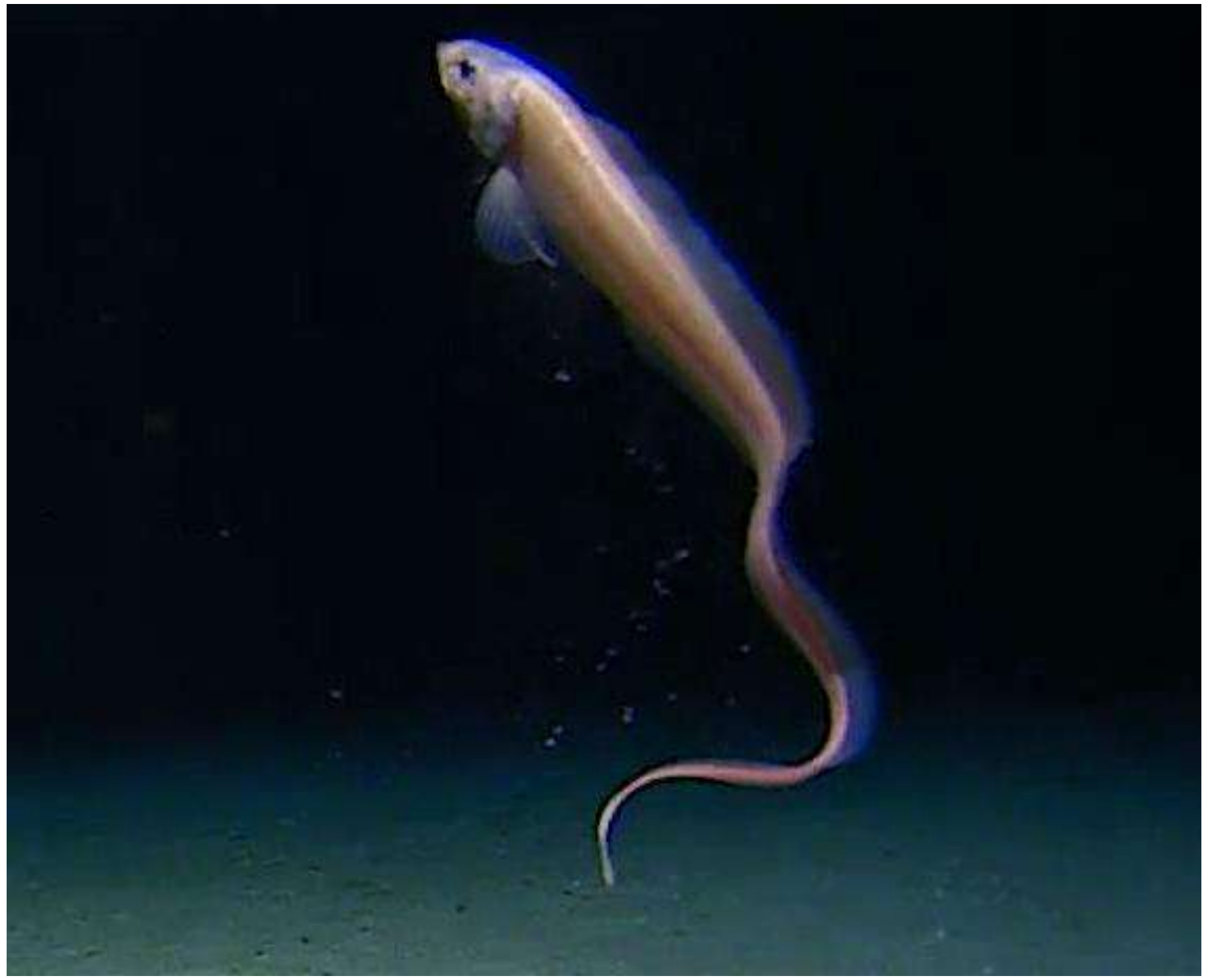

Dorsal fin begins at pectoral fins. Pectoral fins large and rounded with long; tapering tail and tailfin.

Teleostei; Ophidiiformes
Ophidiidae ; *Abyssobrotula;*
Abyssobrotula sp. indet. 1

NC2_OM3_3900_NOR_0098
NC4_CR11_3900_NOR_0031
NC4_MA10_3800_NOR_0041

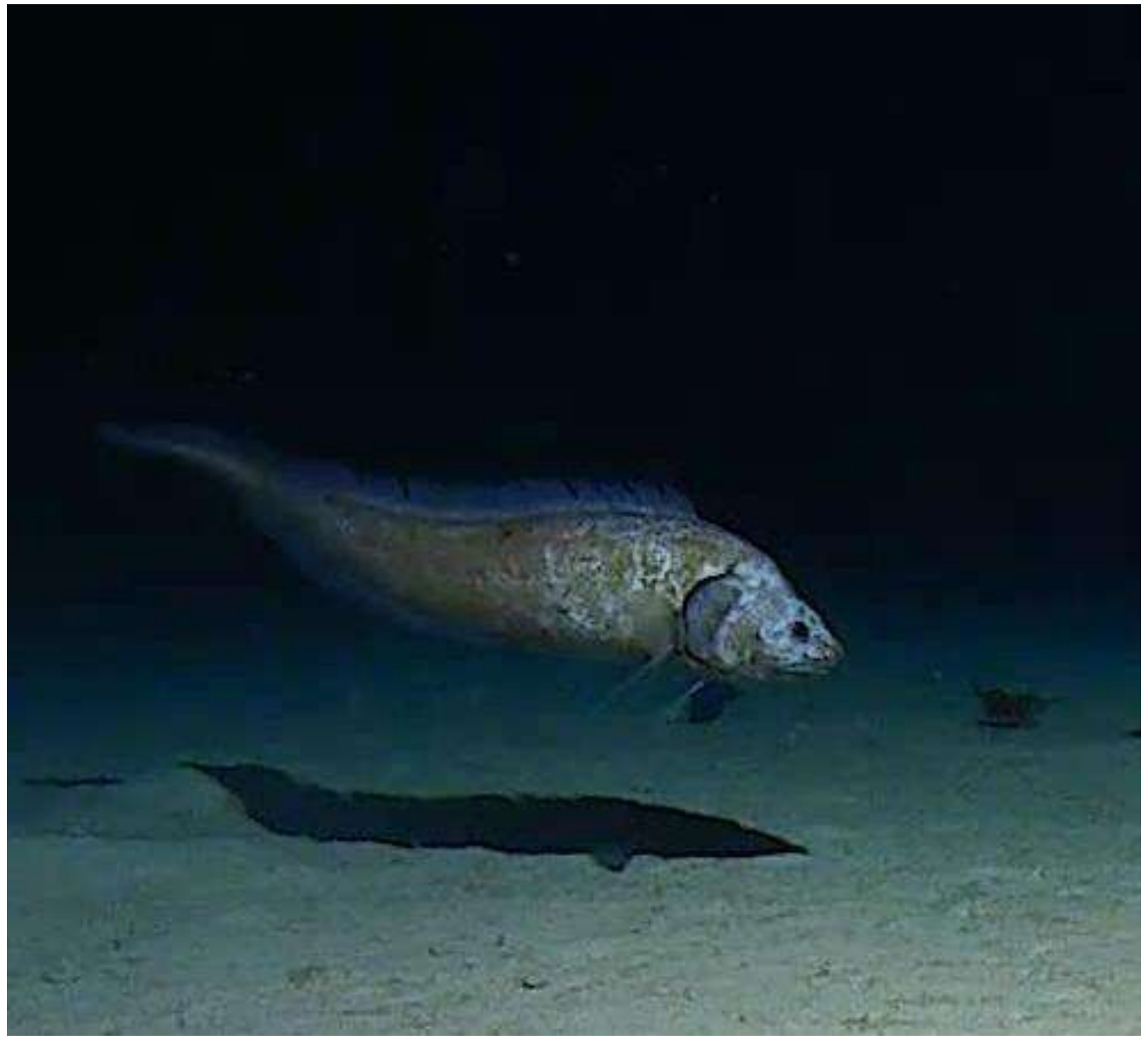

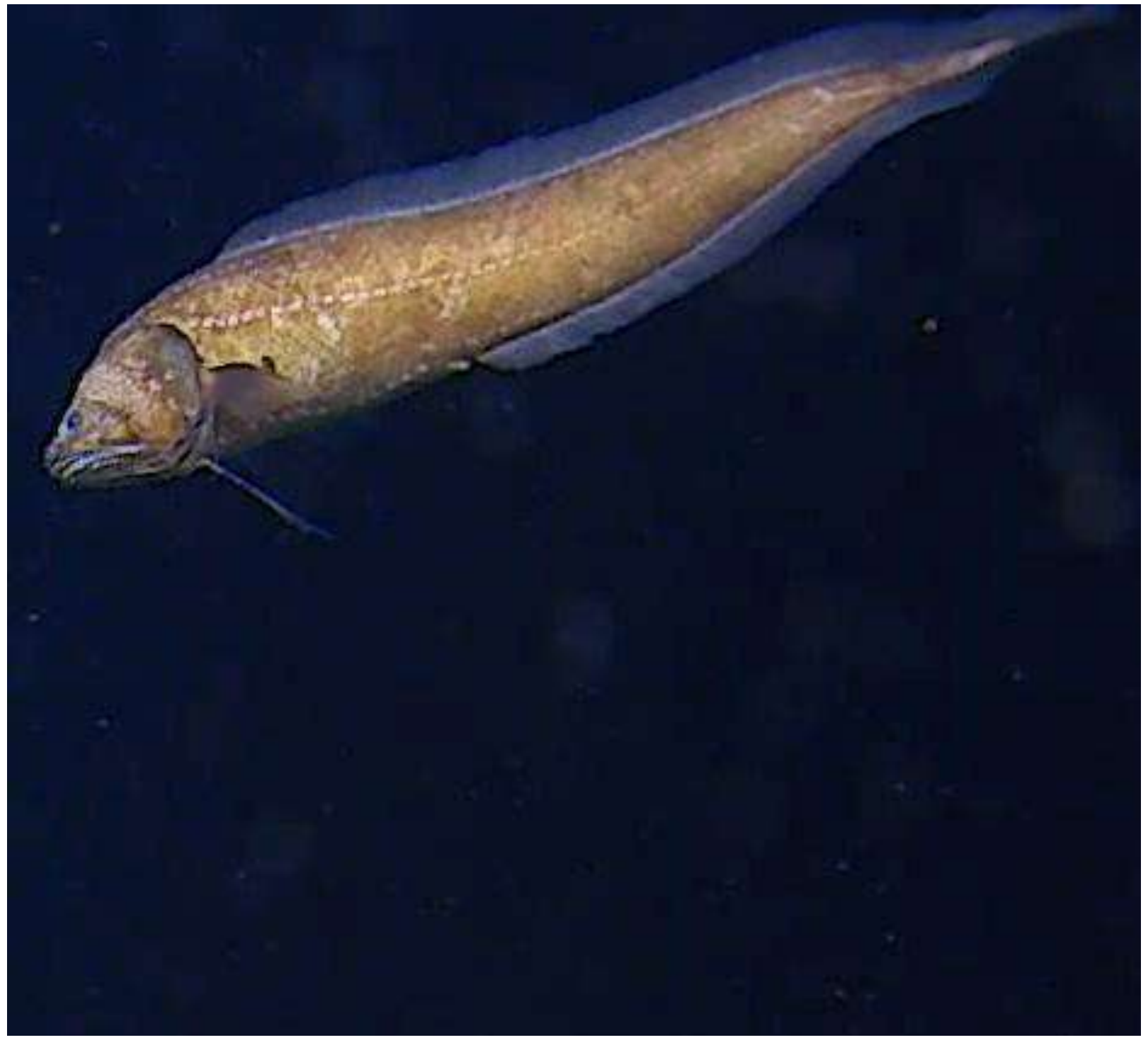

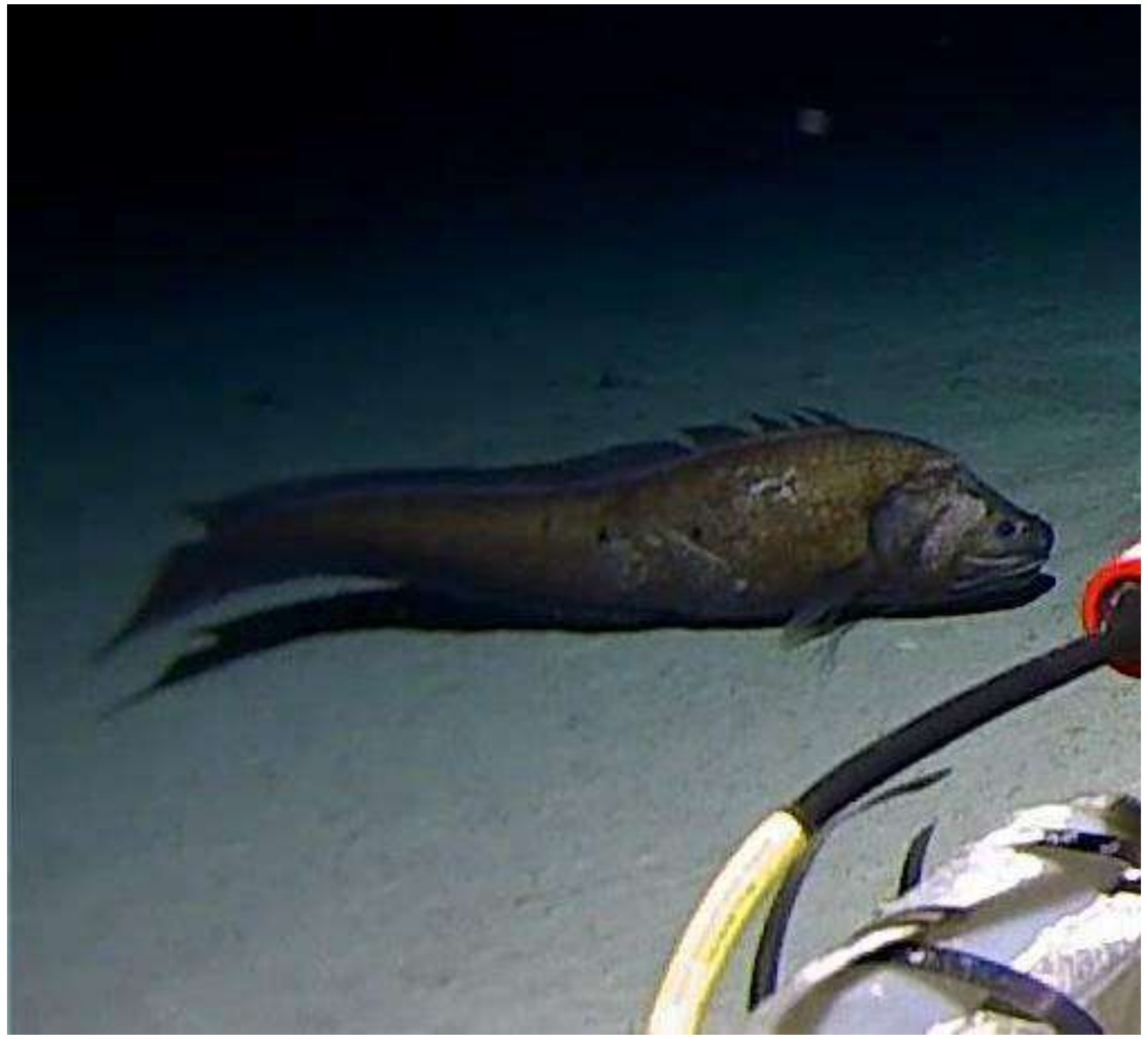

Mottled brown skin with large blotches around the head with white; rounded markings along the body . High dorsal fin (often damaged). Dorsal and anal fin extend toward the tapering tail/tailfin. Downward slope from dorsal fin to rounded snout.

Teleostei; Ophidiiformes Ophidiidae ; *Barathrites; Barathrites iris*

NC2_OM3_4900_NOR_0113

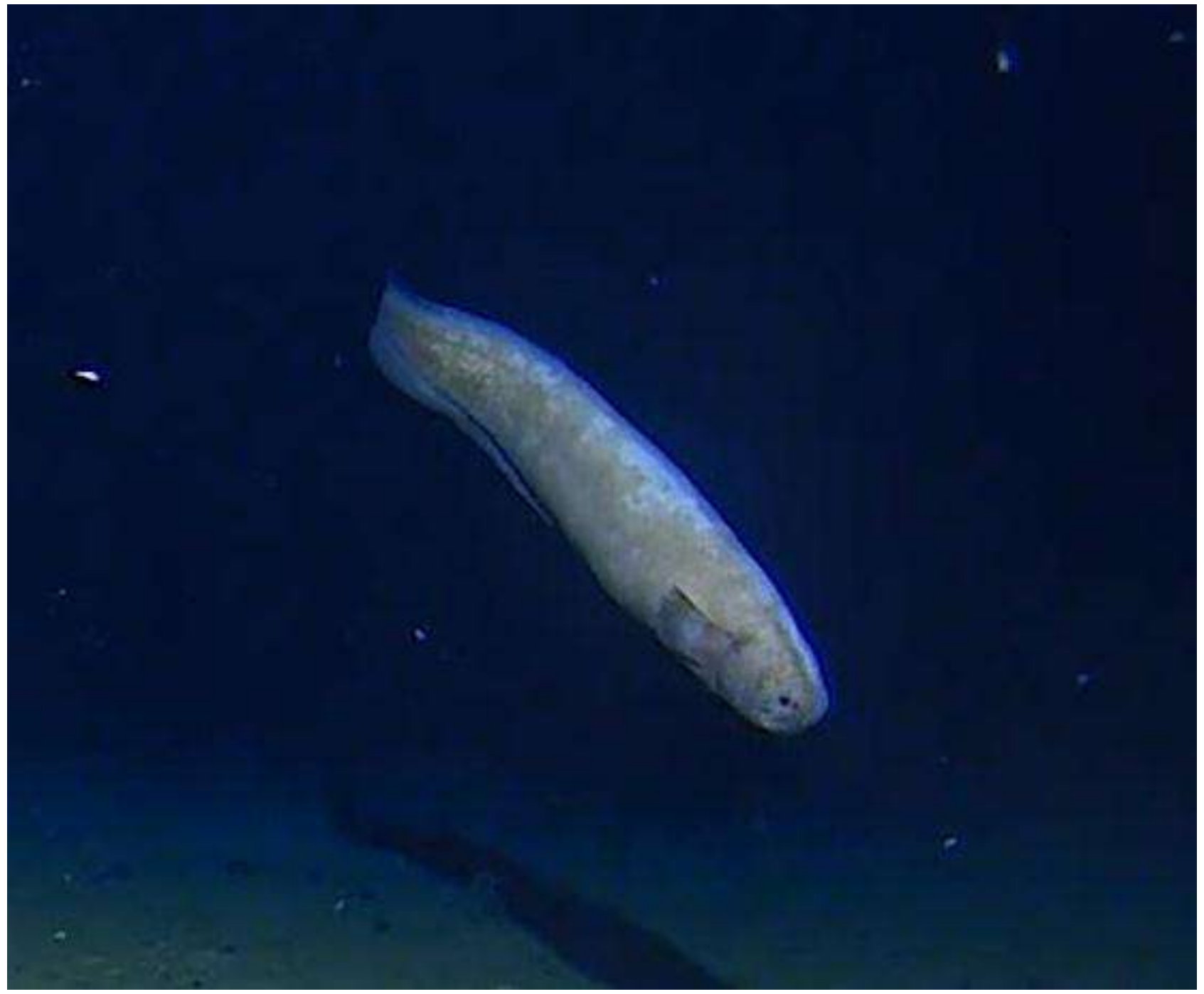

Mottled brown skin with large blotches around the dorsal fin. Dorsal fin starts ¼ of body length. Dorsal and anal fins extend toward the tapering tail/tailfin. Blunt; rounded snout.

Teleostei; Ophidiiformes Ophidiidae ; *Bassogigas; Bassogigas walkeri*

NC2_CR1_3200_NOR_0030
NC2_CR1_3200_NOR_0030

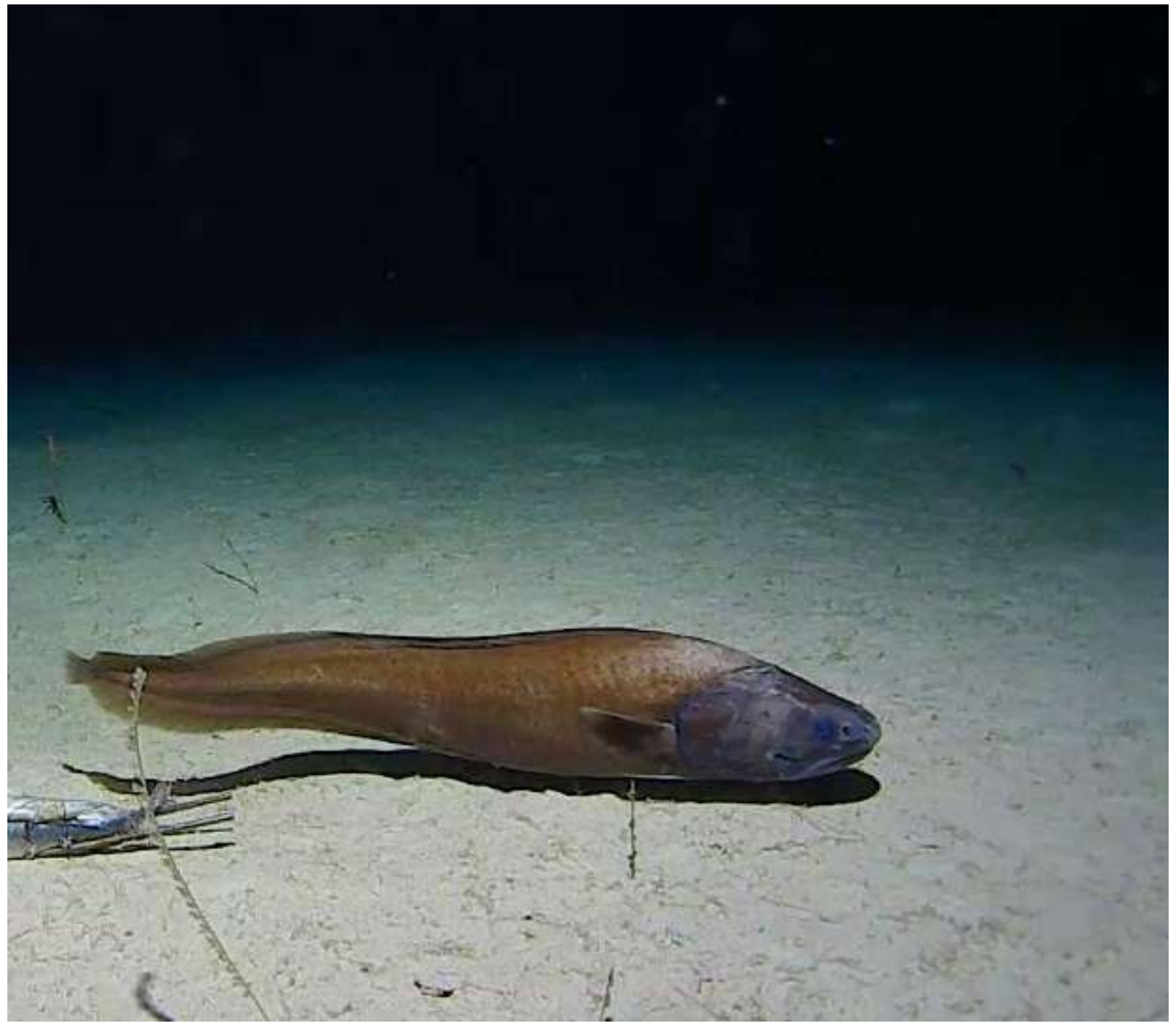

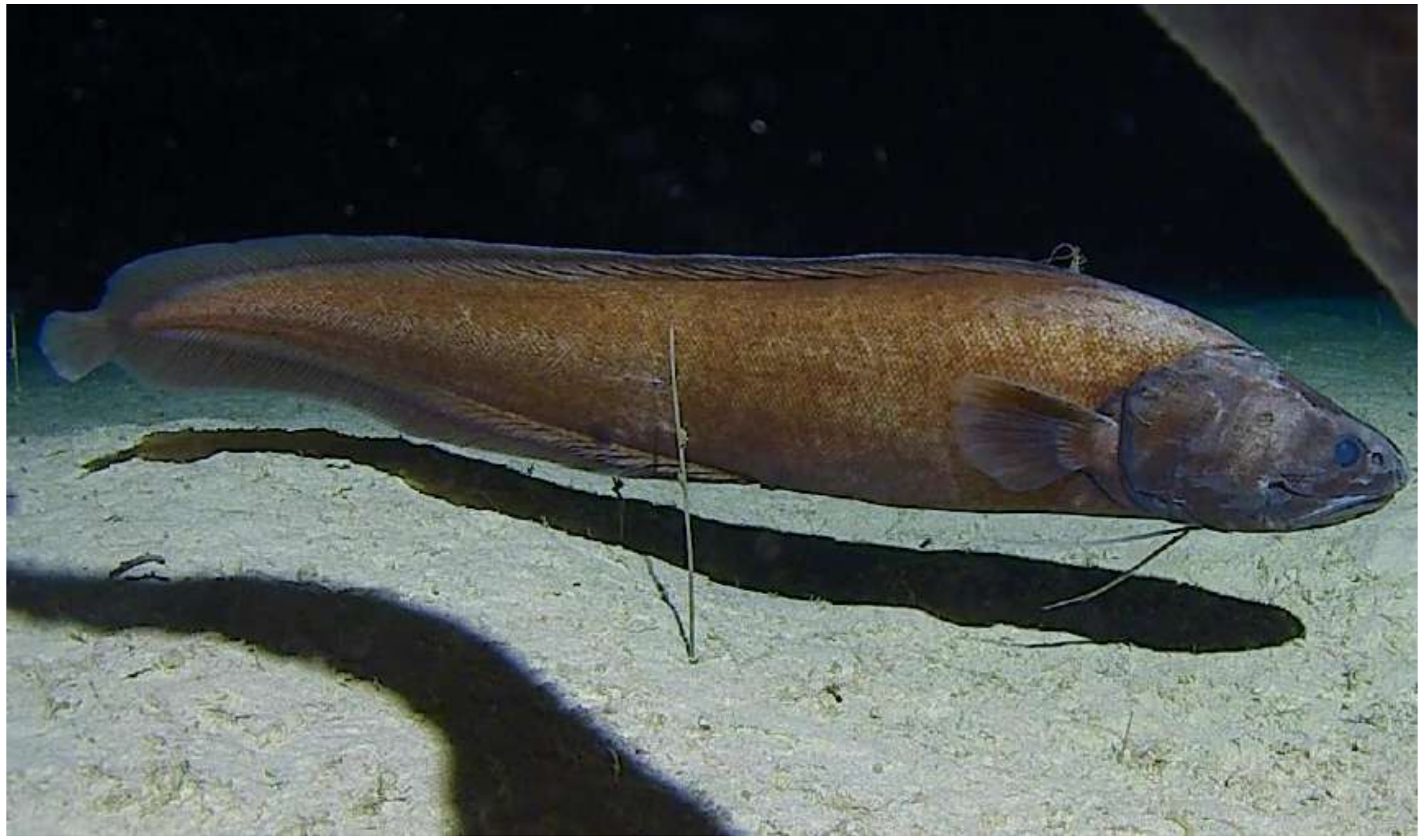

Brown coloured body with dark coloured head. Dorsal and anal fin extend to the tail with separate; square tail fin. Dorsal fin is short and held tightly against the body. Prominent pectoral fins with humped back that slopes toward the snout.

Teleostei; Ophidiiformes
Ophidiidae ; *Typhlonus; Typhlonus nasus*

NC4_MA1_5200_NOR_0051

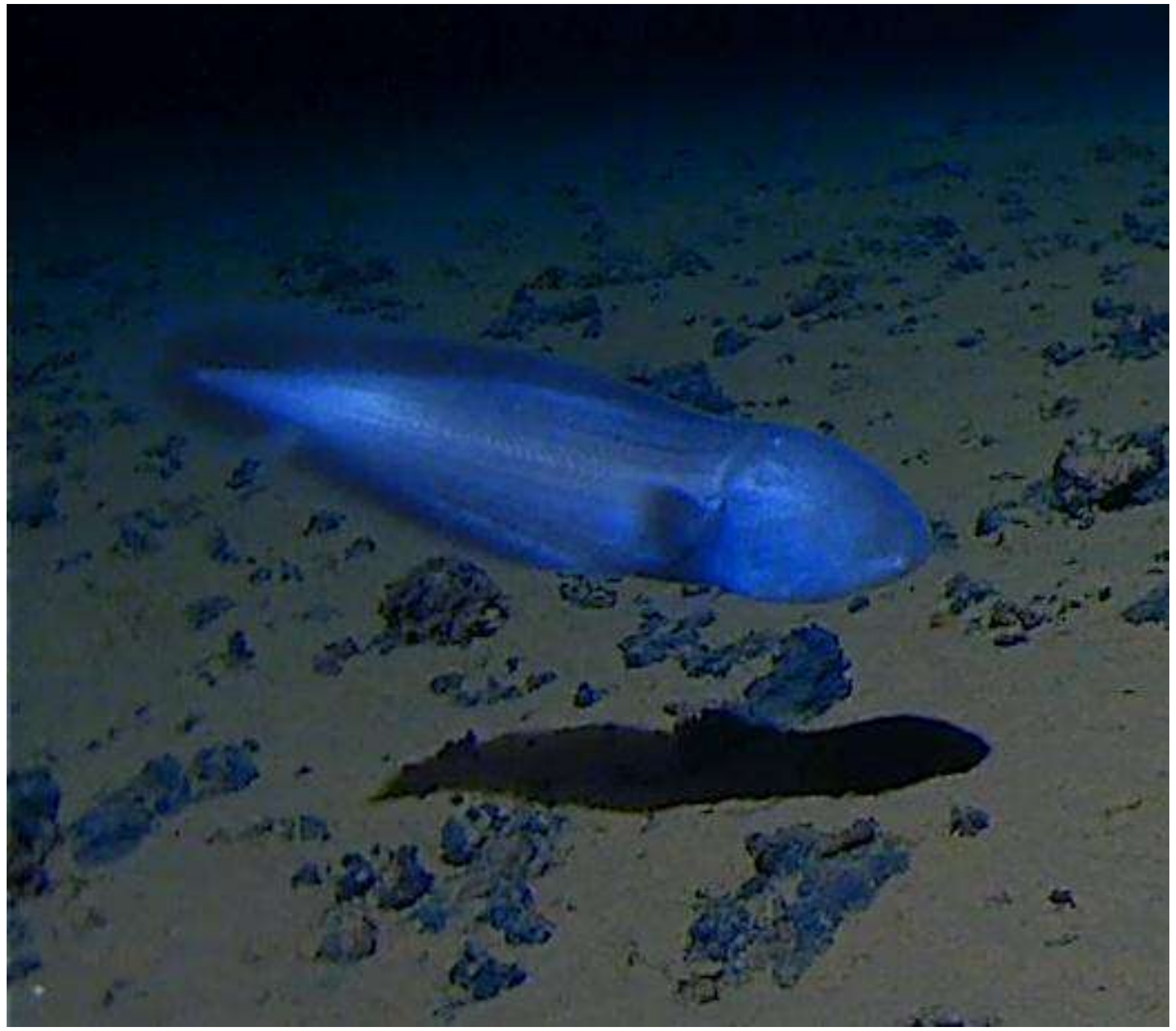

Uniform deep bluish coloured body and head. Dorsal and anal fin extend to the tail with rounded terminal end. Dorsal fin begins behind the head while anal fin begins 3/4 head length behind gill-slit. Prominent pectoral fins rounded. Eyes small and positioned nearer to the anterior end of the head.

Teleostei; Ophidiiformes
Ophidiidae ; *Bassozetus; Bassozetus* sp. indet.1

NC1_CR1_6200_NOR_0126

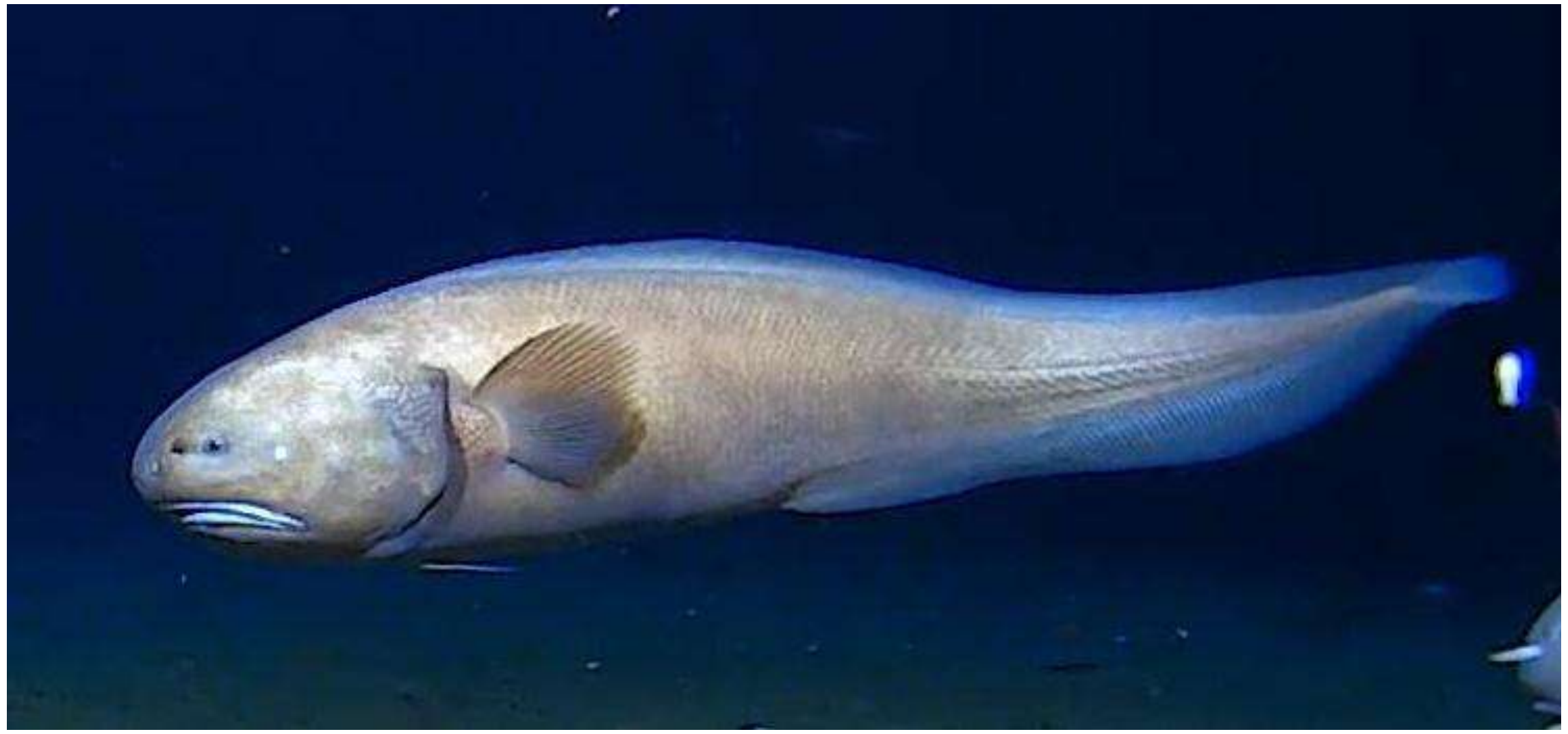

Uniform brown body with dark coloured scale pockets with fins mostly brown (fin tips may be transparent). Profile is straight. Transparent dorsal and anal fins extend to the tail with separate; rectangular tail fin. Dorsal fin is short and held tightly against the body. Prominent pectoral fins with almost pointed snout.

Teleostei; Ophidiiformes Ophidiidae ; *Bassozetus; Bassozetus* sp. indet. 2

NC1_CR1_6200_NOR_0068
NC3_OM5_5400_NOR_0049

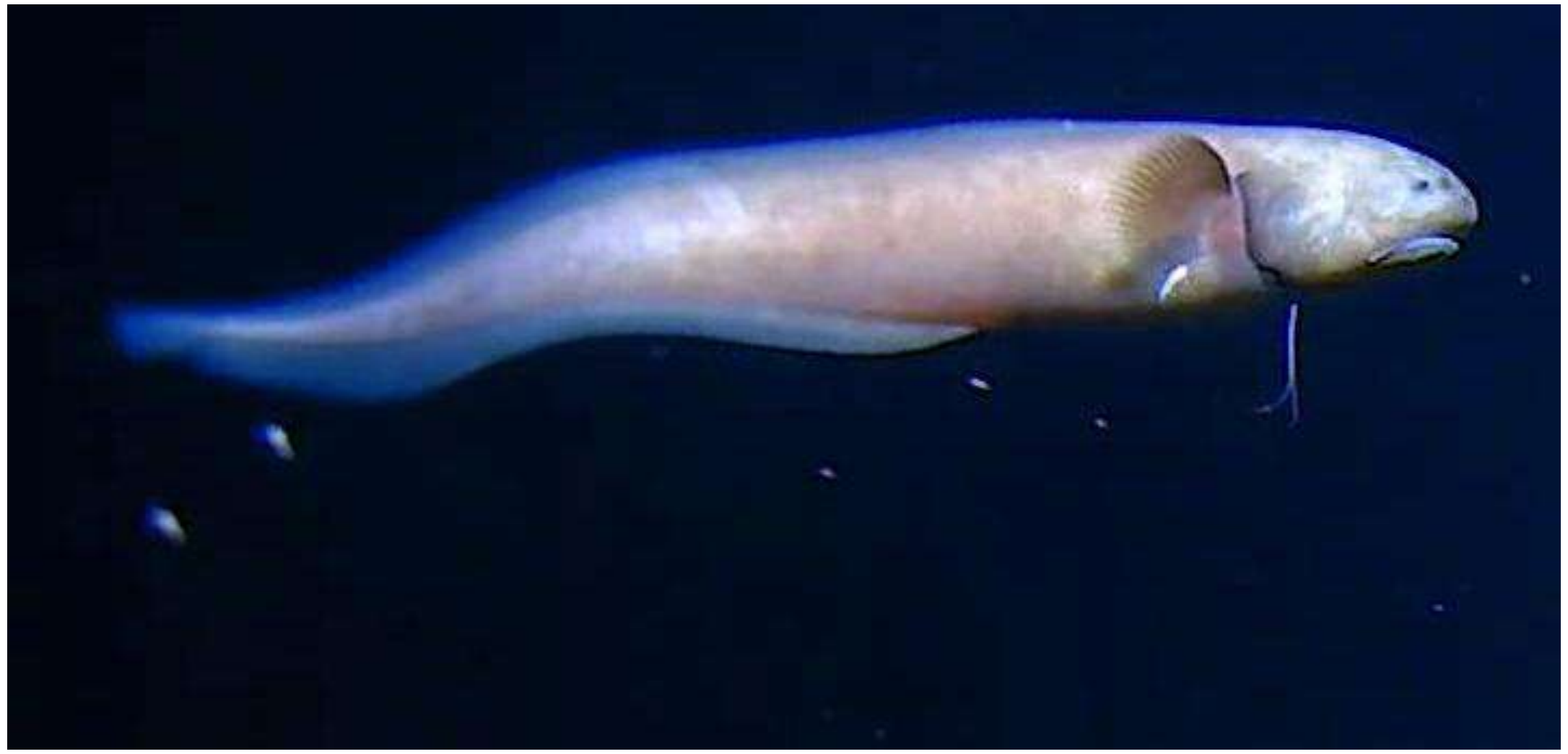

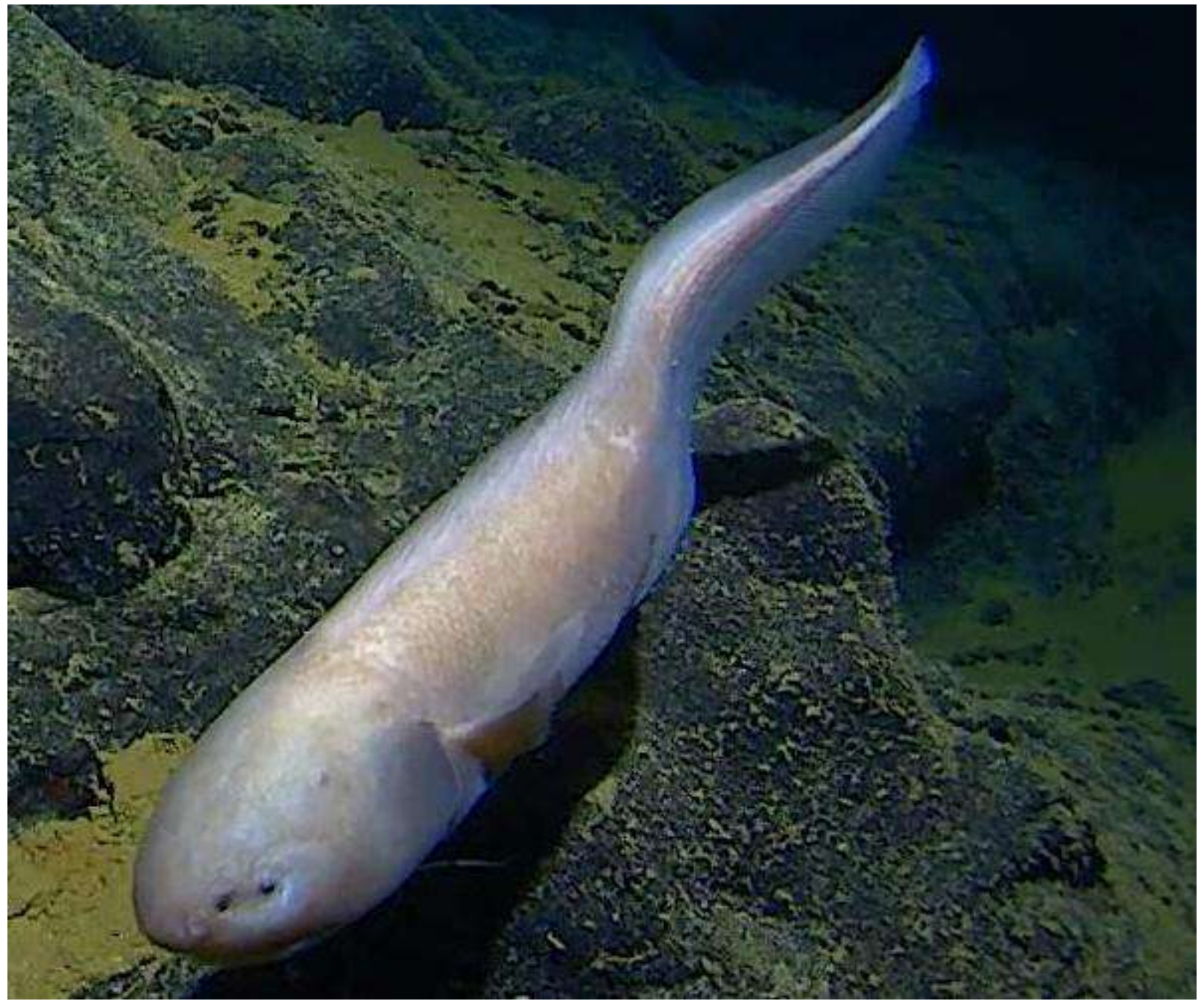

Transparent flesh. Profile is straight. Transparent dorsal and anal fin extend to the tail with separate; rectangular tail fin. Anal fin begins more than two head length back. Dorsal fin is short and held tightly against the body. More caudal fin rays than other Bassozetus. Prominent pectoral fins with rounded snout.

Teleostei; Ophidiiformes Ophidiidae ; *Bassozetus; Bassozetus* sp. indet. 3

NC1_CR1_6200_NOR_0067

NC1_CR1_6200_NOR_0067

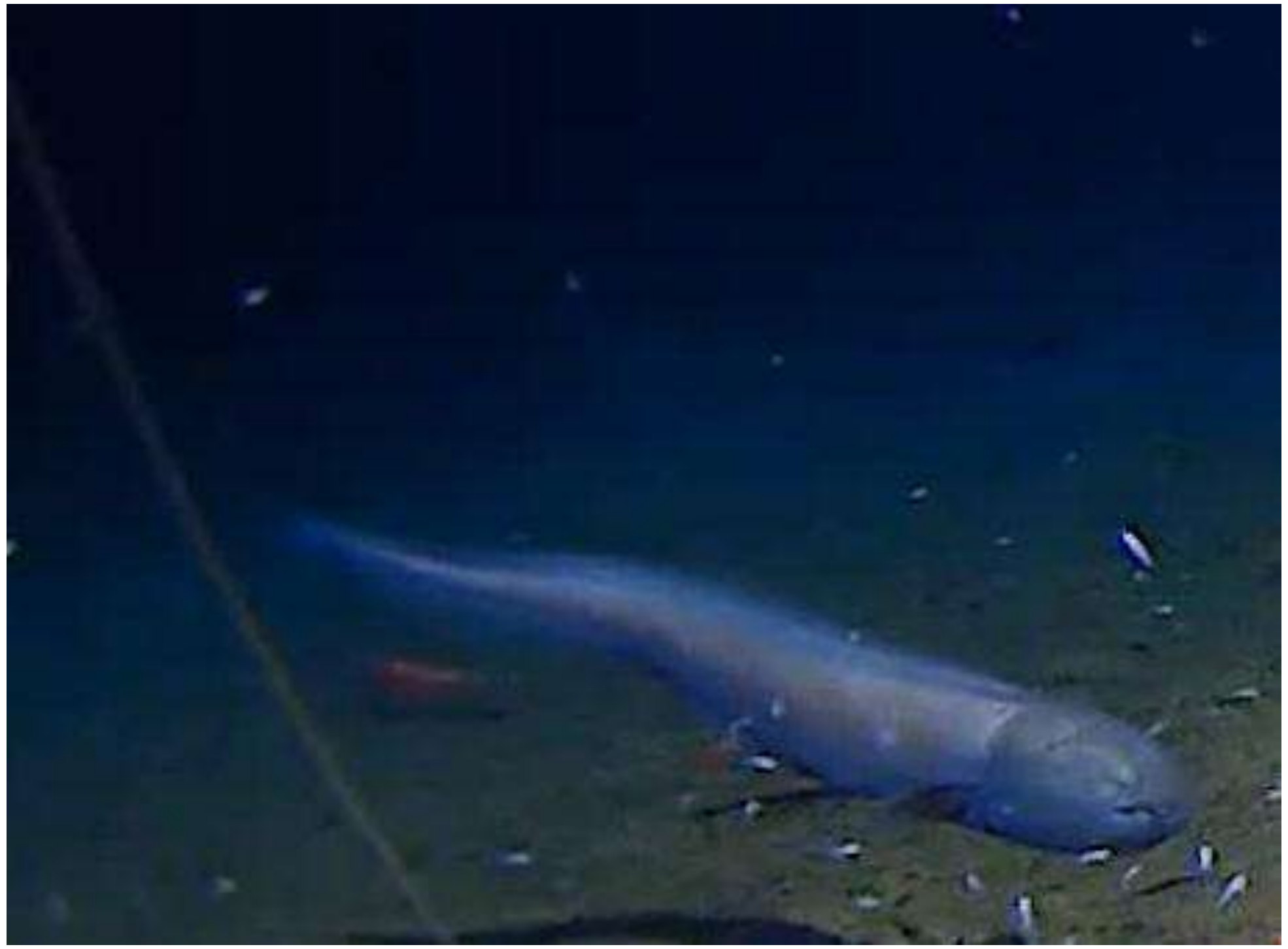

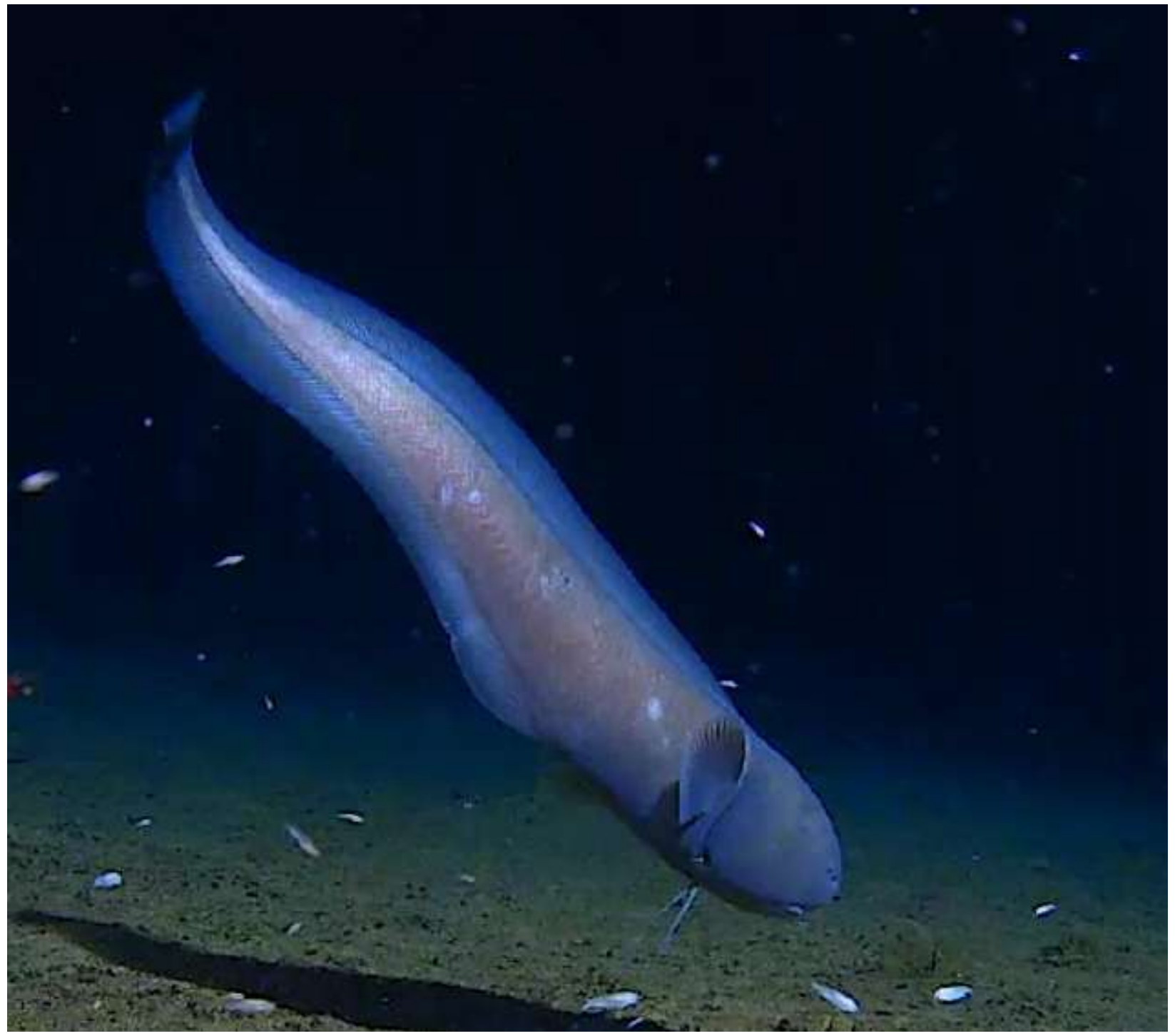

Dark brownish coloured body with greyish head. Transparent dorsal and anal fin extend to the tail with separate; rectangular tail fin. Anal fin begins less than roughly 1 head length back. Prominent; long pectoral fins with rounded globular head.

Teleostei; Ophidiiformes
Ophidiidae ; *Bassozetus; Bassozetus* sp. indet. 4

NC2_OM5_5600_NOR_0147
NC2_OM5_5600_NOR_0149
NC2_OM5_5600_NOR_0146

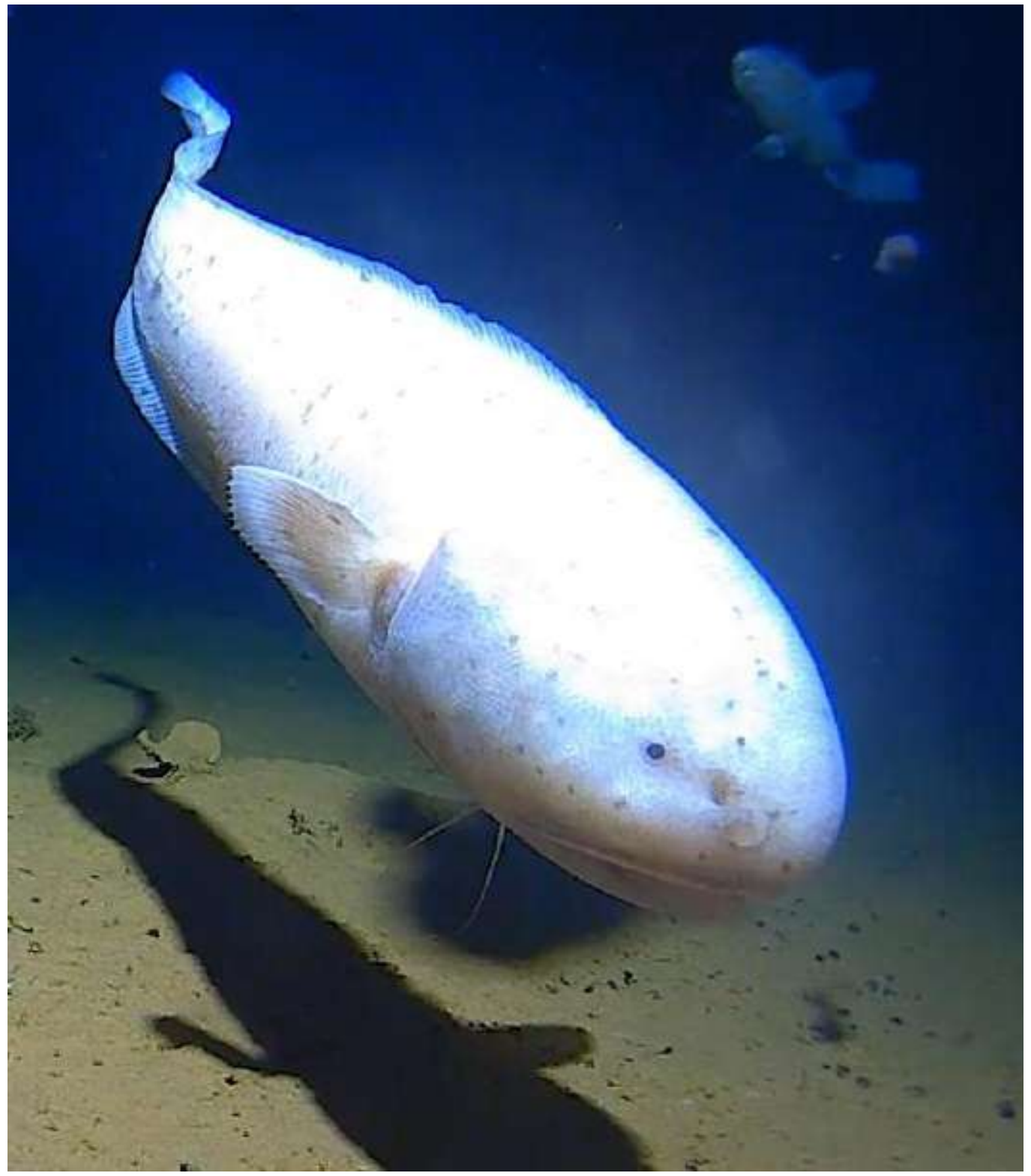
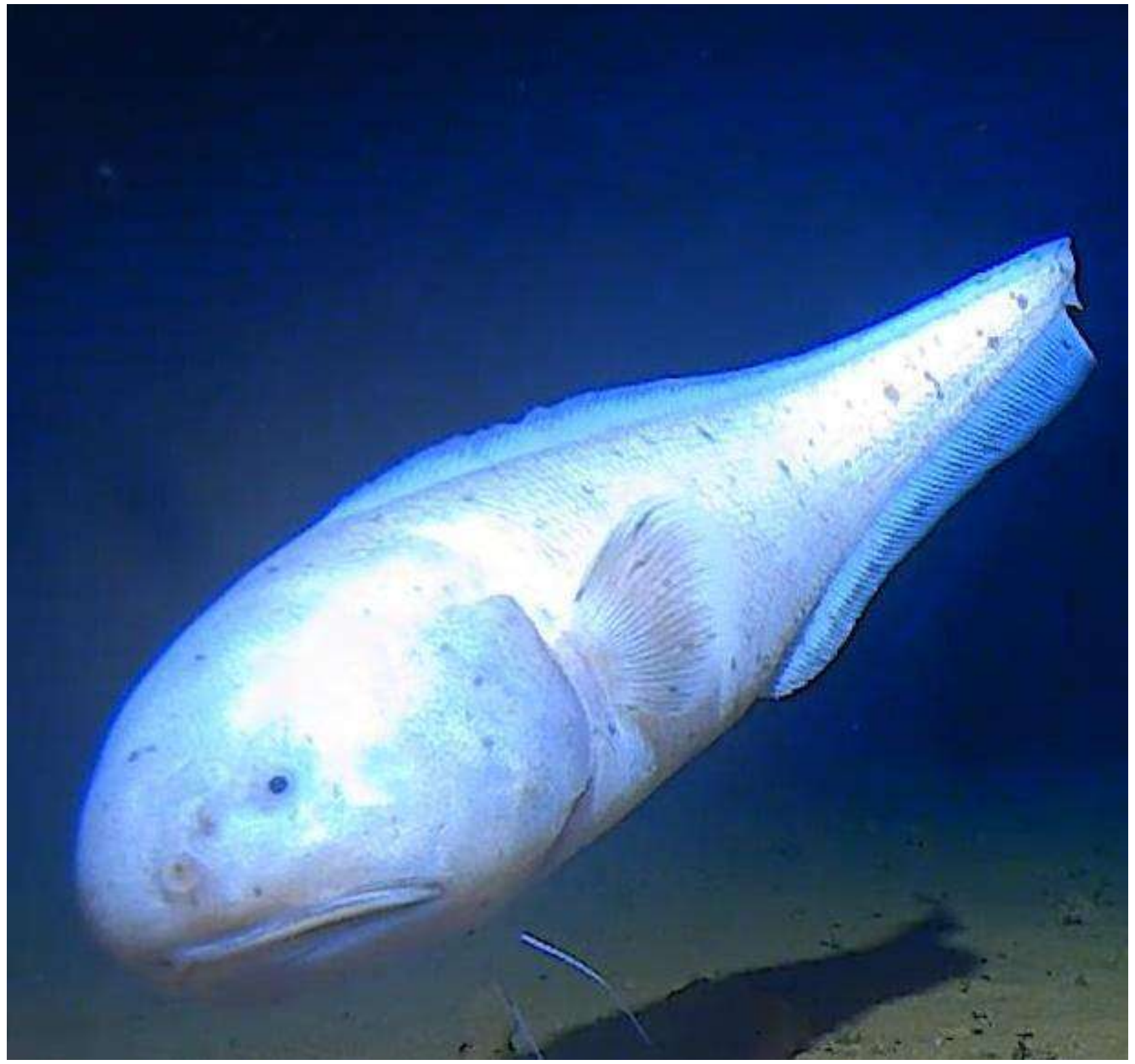
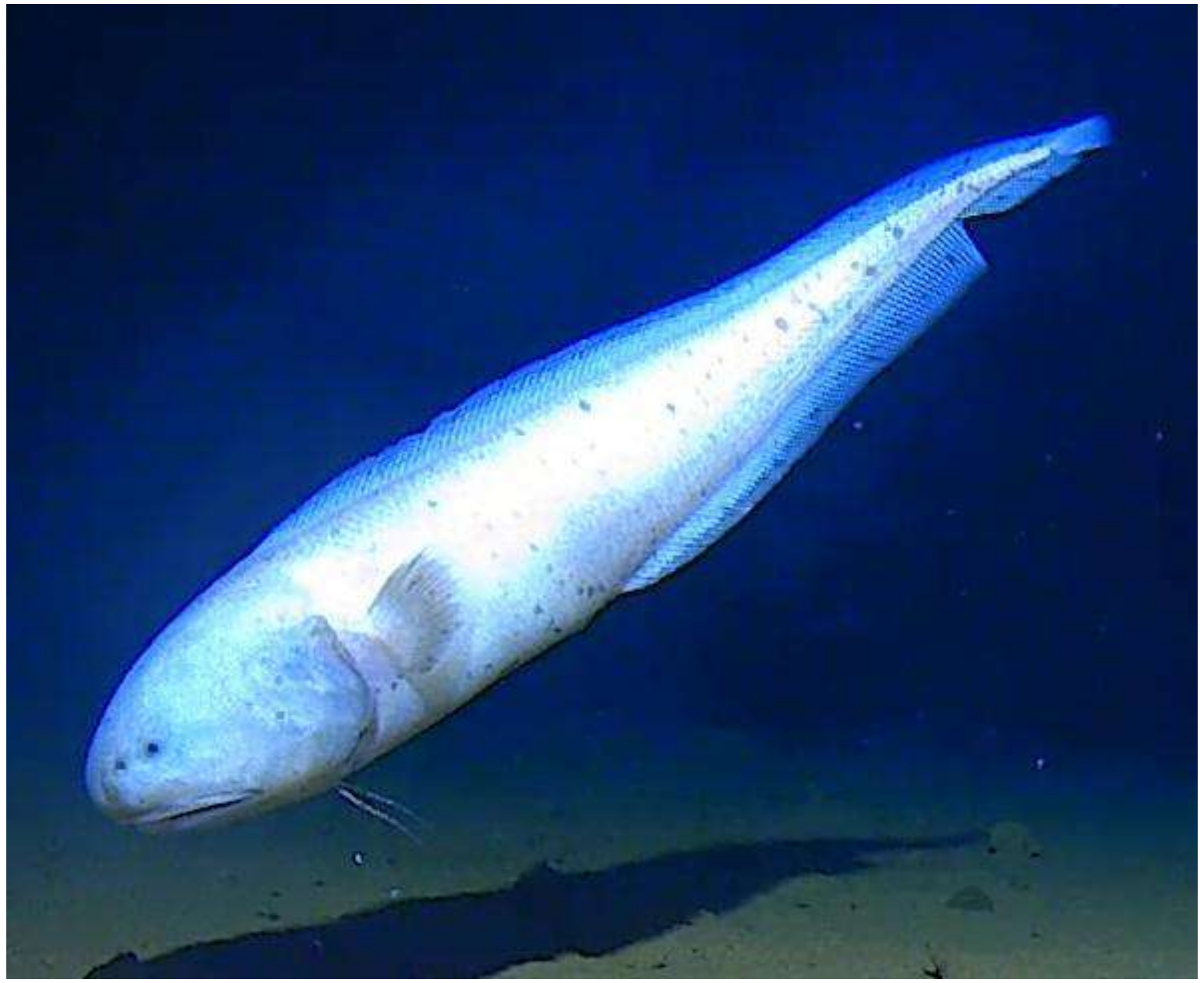

Pale grey with small dark spots. Pale grey dorsal and anal fin extend to the tail with separate; rectangular tail fin. Anal fin begins less than roughly 1 head length back. Prominent; long pectoral fins with rounded ovular head and body resembling a robust Bassozetus sp indet.2.

Teleostei; Ophidiiformes Ophidiidae ; *Bassozetus; Bassozetus* sp. indet. 6

NC2_OM9_4900_NOR_0110
NC2_OM9_4900_NOR_0111

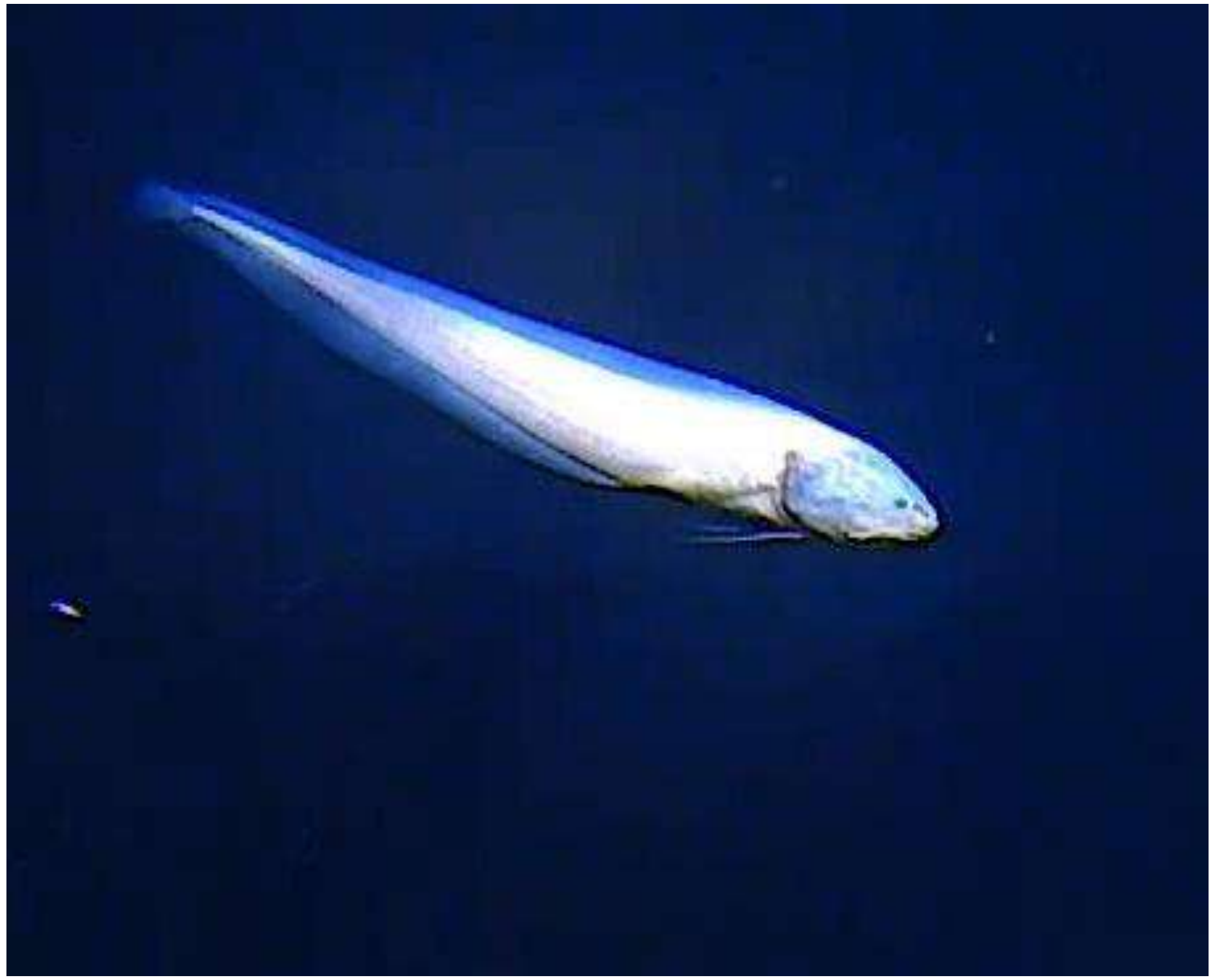

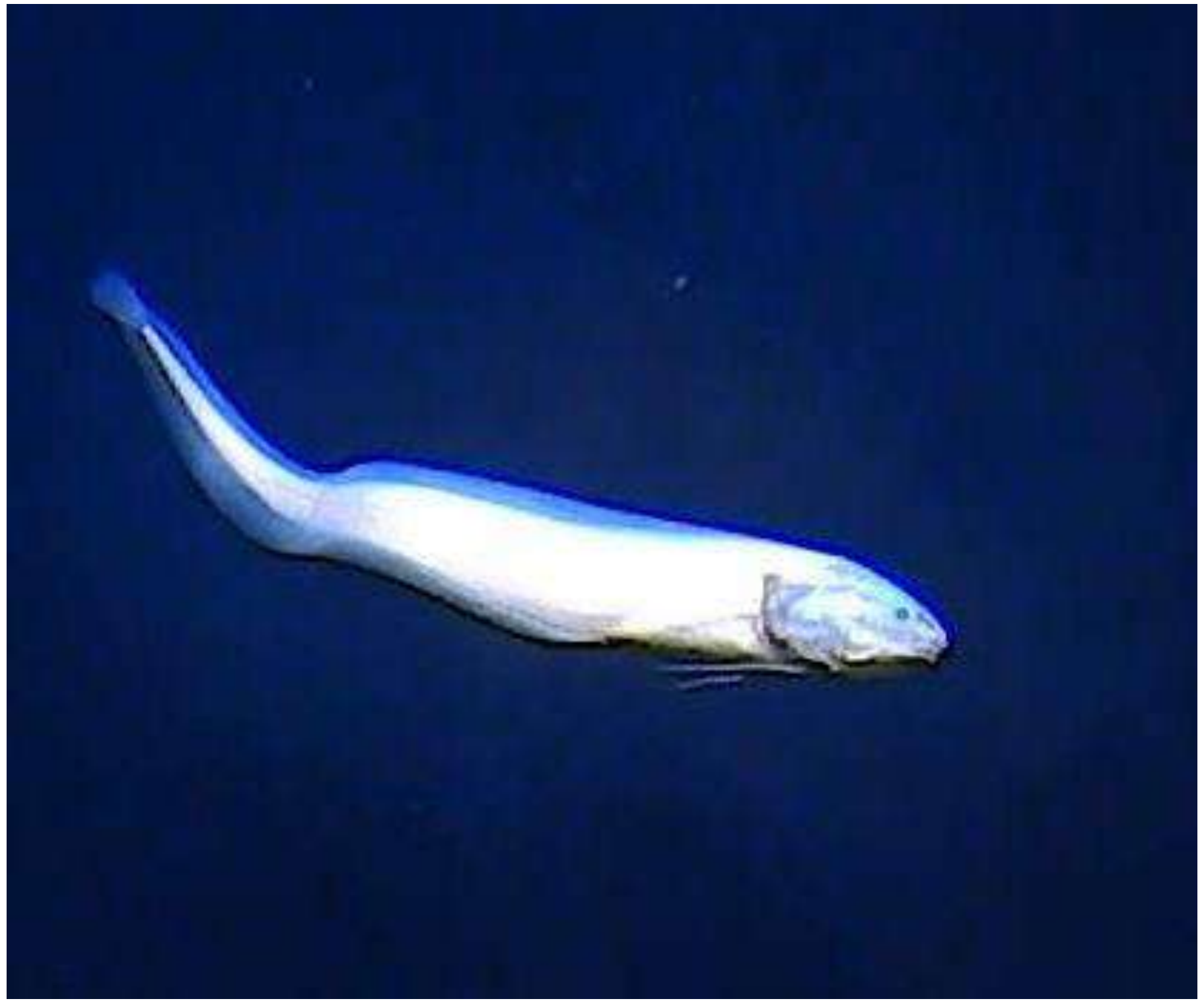

Pale coloured body with pale greyish head. Transparent dorsal and anal fin extend to the tail with separate; wedge-shaped tail fin. Anal fin begins more than roughly 1 ½ head lengths back. Short pectoral fins with ovular; pointed head.

Teleostei; Ophidiiformes Ophidiidae ; *Bassozetus; Bassozetus* sp. indet. 7

NC3_MA8_2800_NOR_0051

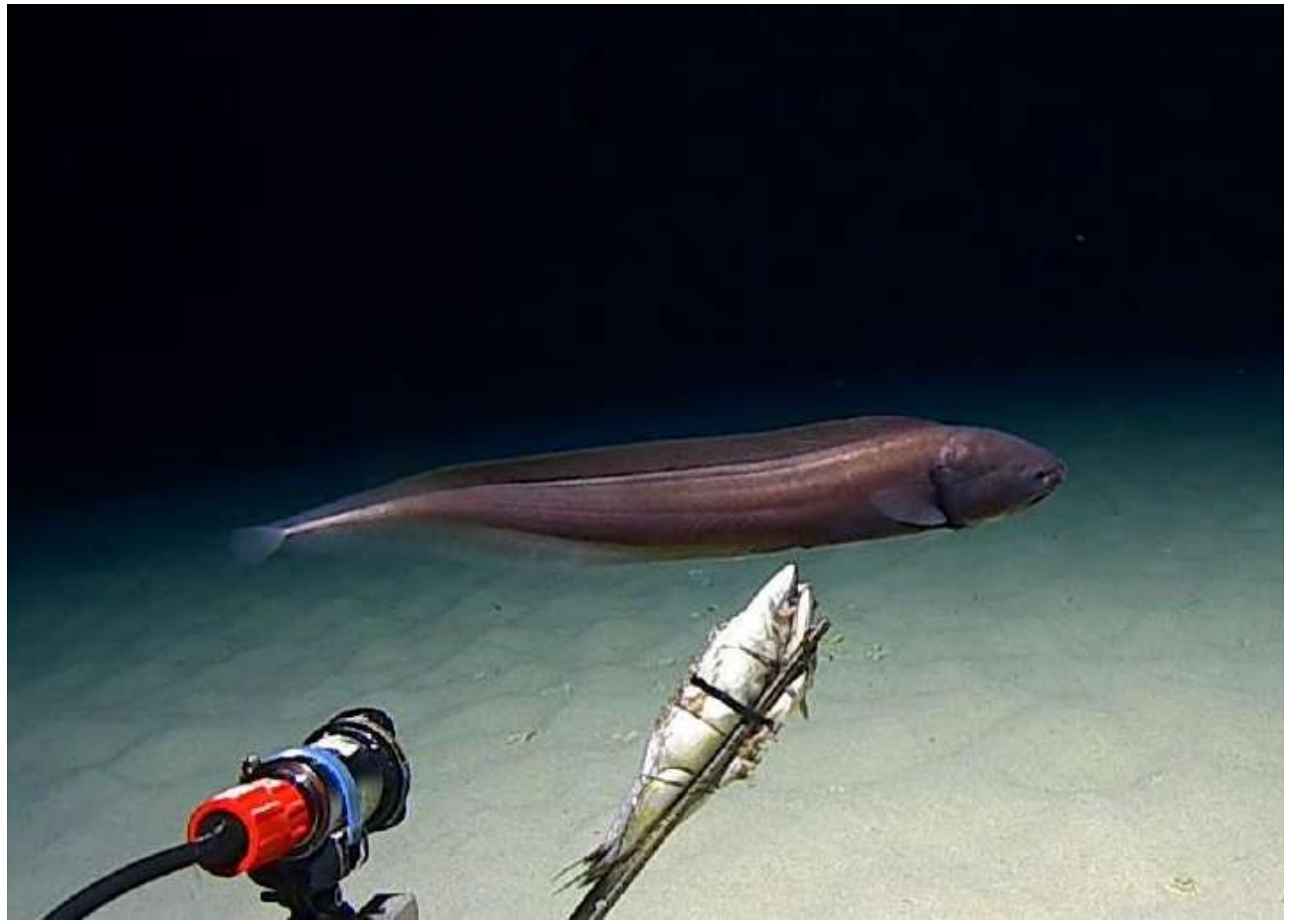

Dark bronze coloured body with dark brown head. Brown dorsal and anal fin extend to the tail with separate; wedge-shaped tail fin. Dorsal and anal fin become translucent toward tail tip. Tail becomes pale at terminal end. Anal fin begins roughly 1 ½ head lengths back. Short; rounded pectoral fins with more rounded triangular head.

Teleostei; Ophidiiformes Ophidiidae ; *Bassozetus; Bassozetus* sp. indet. 8

NC1_CR1_6200_NOR_0106

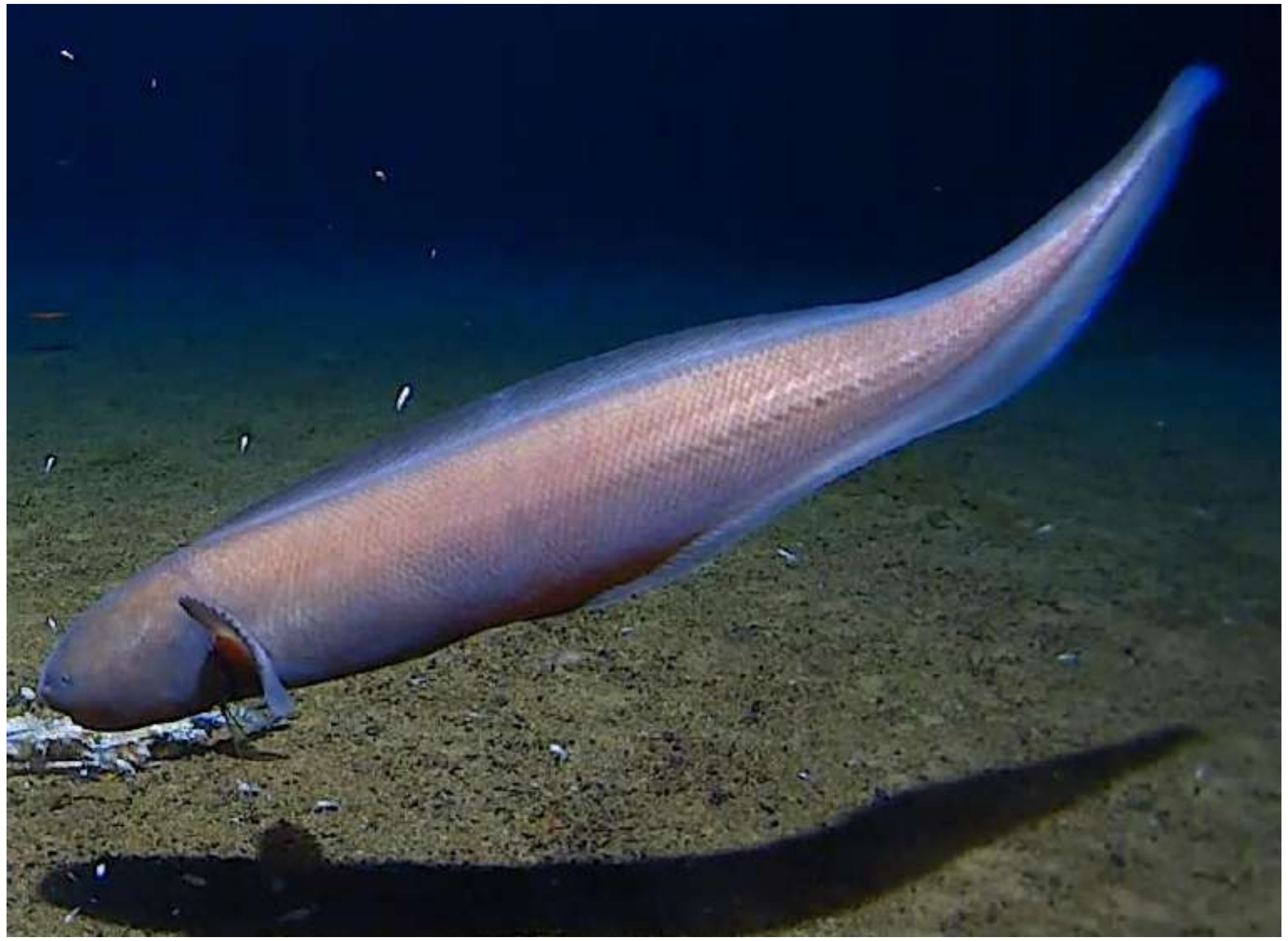

Brown coloured body. Brown dorsal and anal fin extend to the tail with separate; wedge-shaped tail fin. Dorsal and anal fins become translucent at fin edges. Anal fin begins roughly 2 head lengths back. Wide; rounded pectoral fins with more rounded/ ovular head.

Teleostei; Ophidiiformes Ophidiidae ; *Leucicorus; Leucicorus* sp. indet. 1

NC2_CR5_5500_NOR_0073

NC2_MA6_5800_NOR_0059

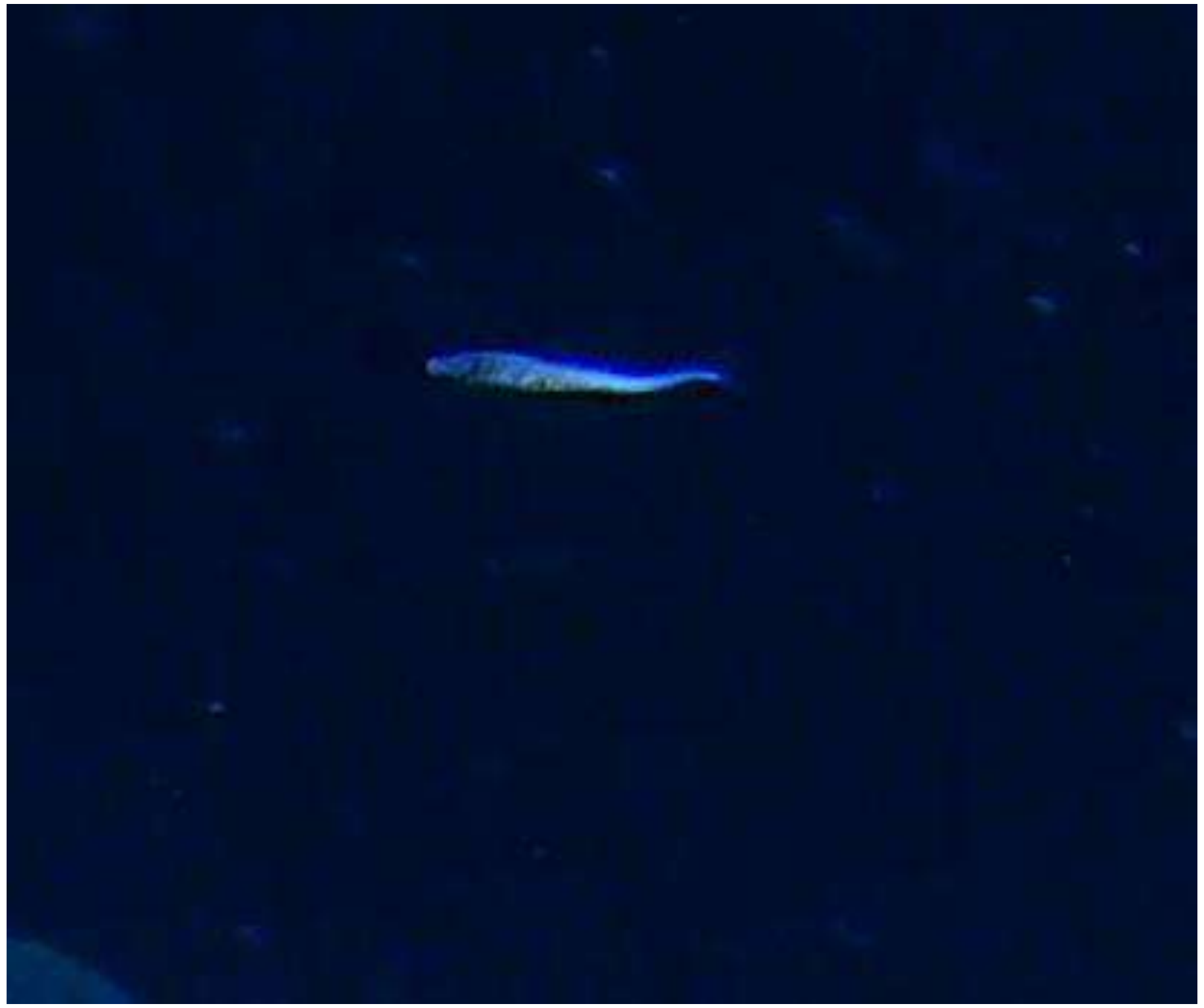

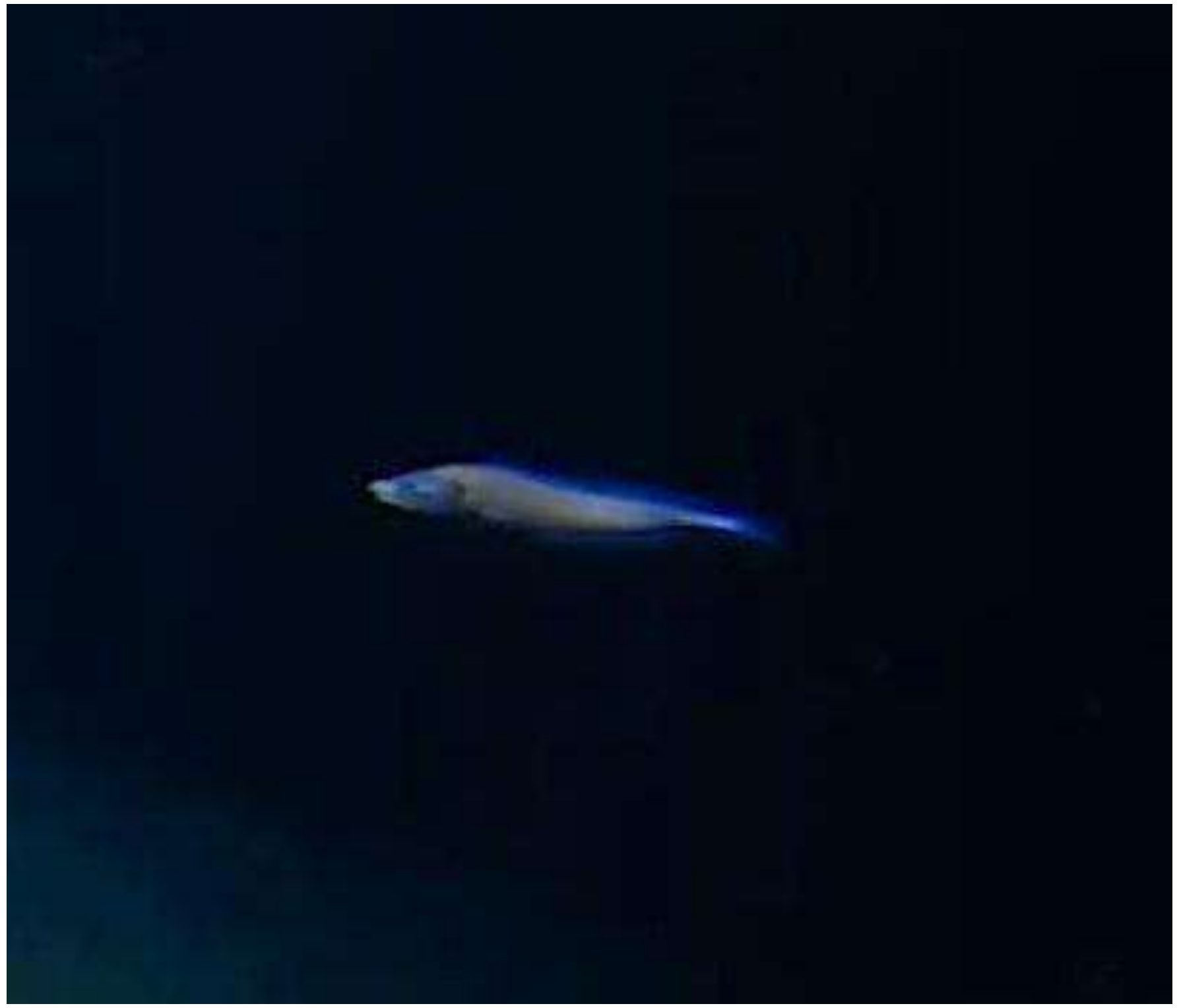

Dark coloured body with pale, concave head. Body is compressed Translucent dorsal and anal fin extend to the tail with separate; wedge-shaped tail fin. Anal fin begins roughly 1 head length back. Short pectoral fins with pointed and flattened head.

Teleostei; Ophidiiformes
Ophidiidae ; Ophidiidae *spp.*

NC2_MA6_5700_NOR_0060
NC2_MA6_5700_NOR_0115

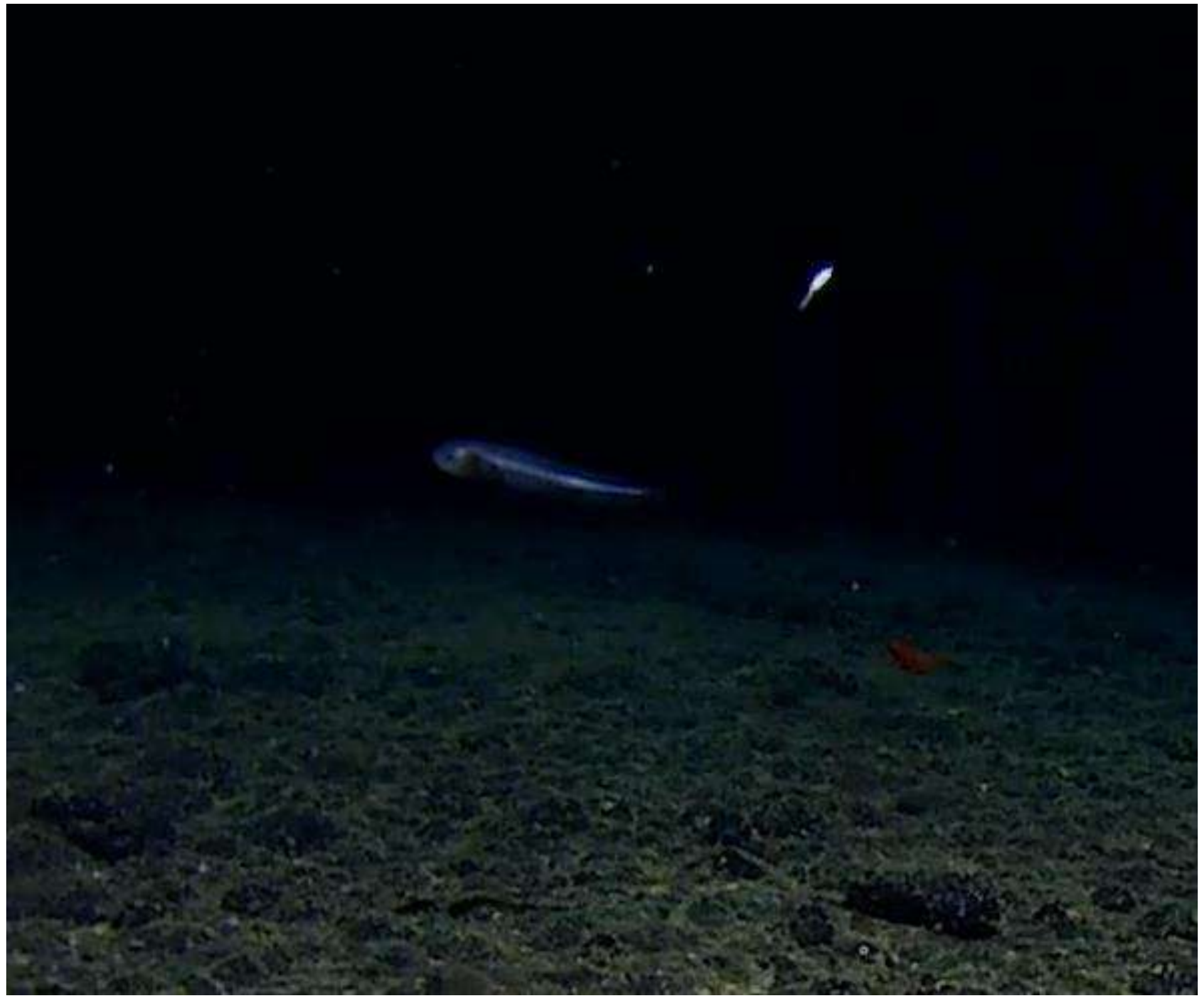
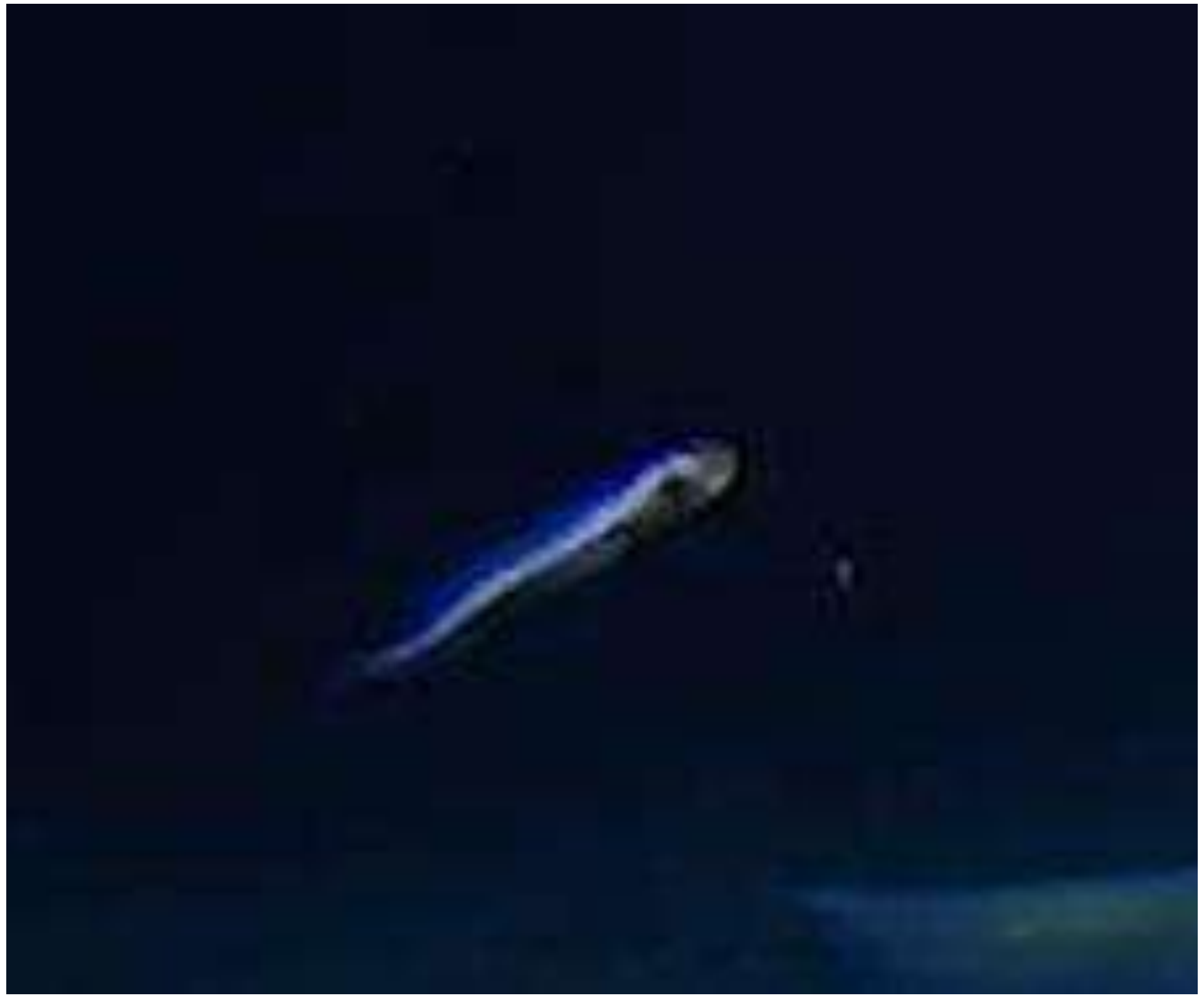

(possibly juvenile form of other Ophidiidae morphotypes)

Teleostei; Ophidiiformes
Ophidiidae ; Ophidiidae sp. indet. *1*

NC2_CR6_5800_NOR_0095
NC4_CR4_5100_NOR_0068
NC3_MA10_5200_NOR_0094

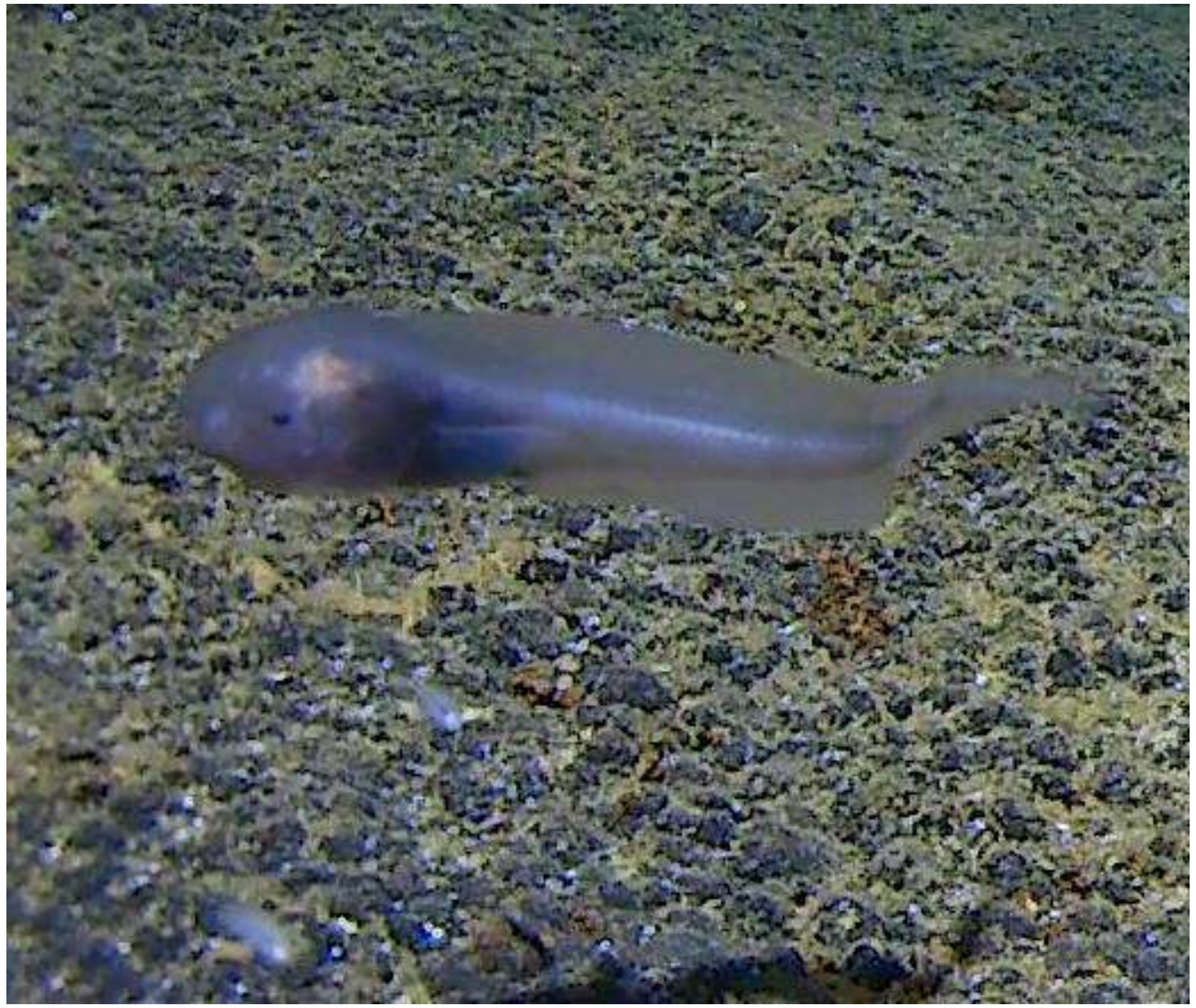

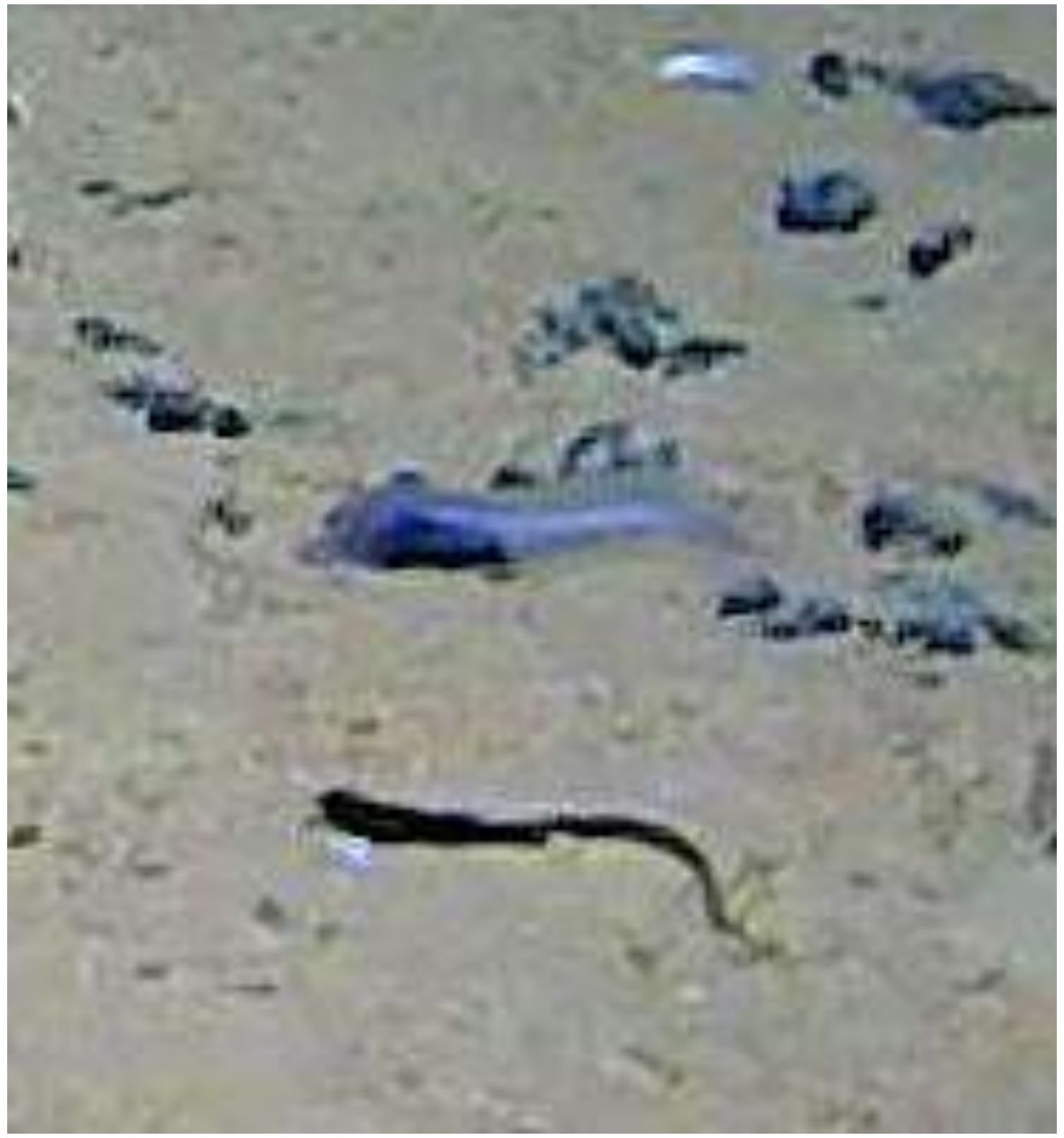

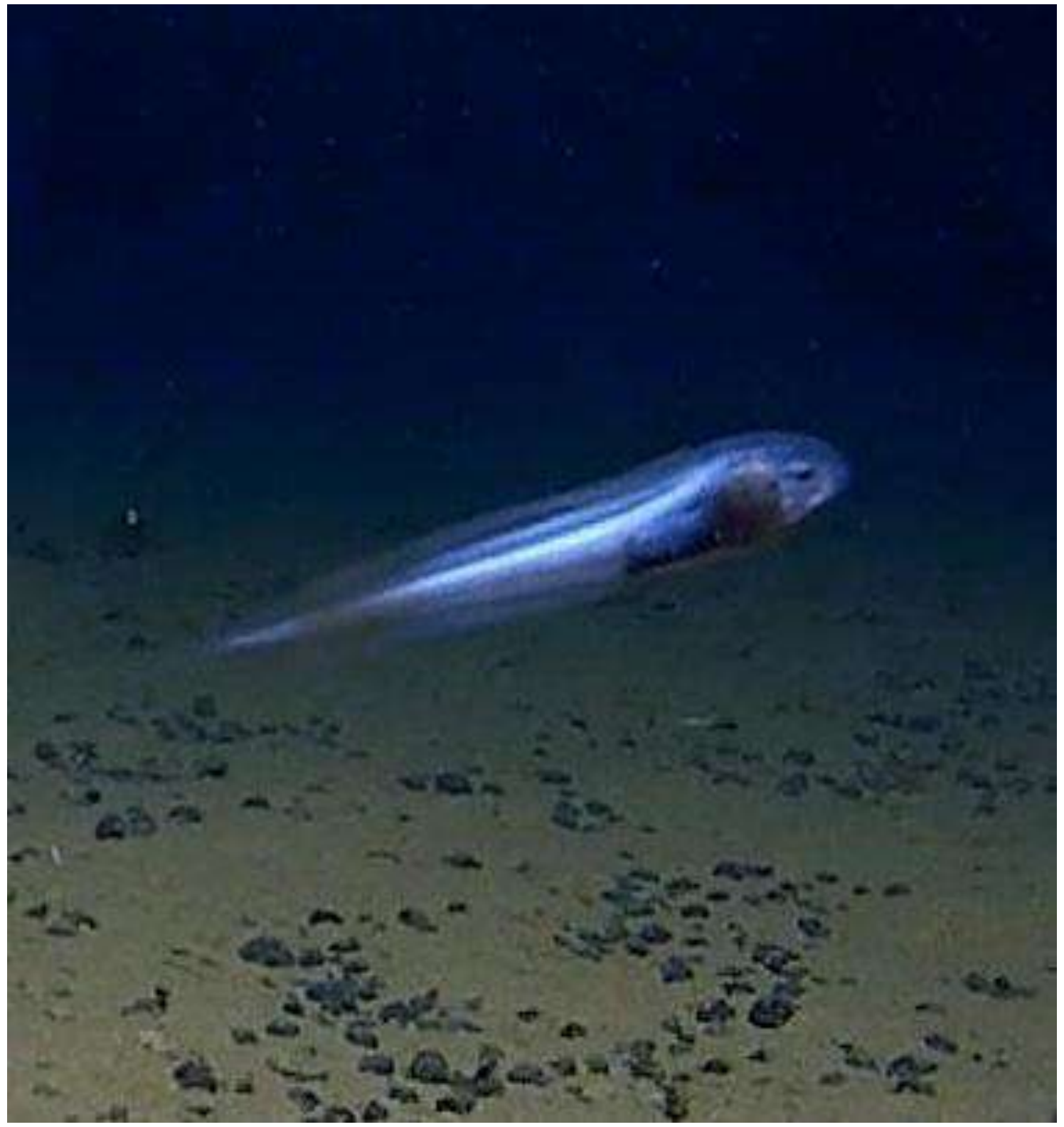

Silvery purple coloured body with dark brown head. Translucent dorsal and anal fin extend to the tail. Tail becomes pale at terminal end. Anal fin begins roughly 1 head length back. Short pectoral fins with more rounded head. Area between the gill slit and anal fin notably darker than surrounding anatomy.

Teleostei; Ophidiiformes
Ophidiidae ; Ophidiidae sp. indet. 2

NC4_BK4_4700_NOR_0066

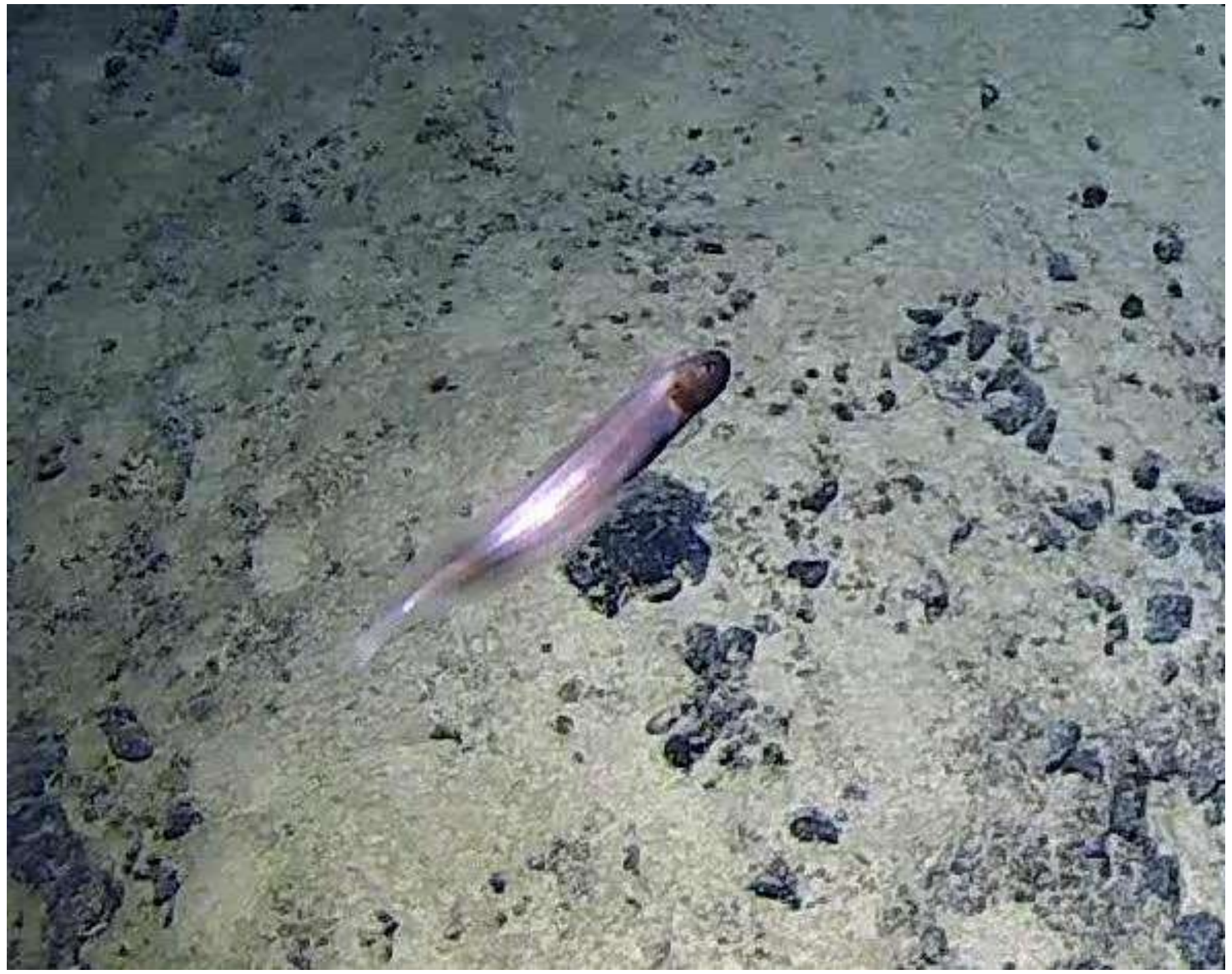

Silvery brown coloured body with dark brown head. Translucent dorsal and anal fin extend to the tail with separate; wedge-shaped tail fin. Tail becomes pale at terminal end. Anal fin begins roughly 1 ½ head lengths back. Short pectoral fins with more rounded head. May be a juvenile representative of other morphs

Teleostei; Perciformes
Liparidae ; *Pseudoliparis;*
Pseudoliparis sp. indet.1

NC1_BK1_6200_IMG_4695
NC1_BK1_6200_IMG_4715
NC1_CR2_6500_NOR_0099

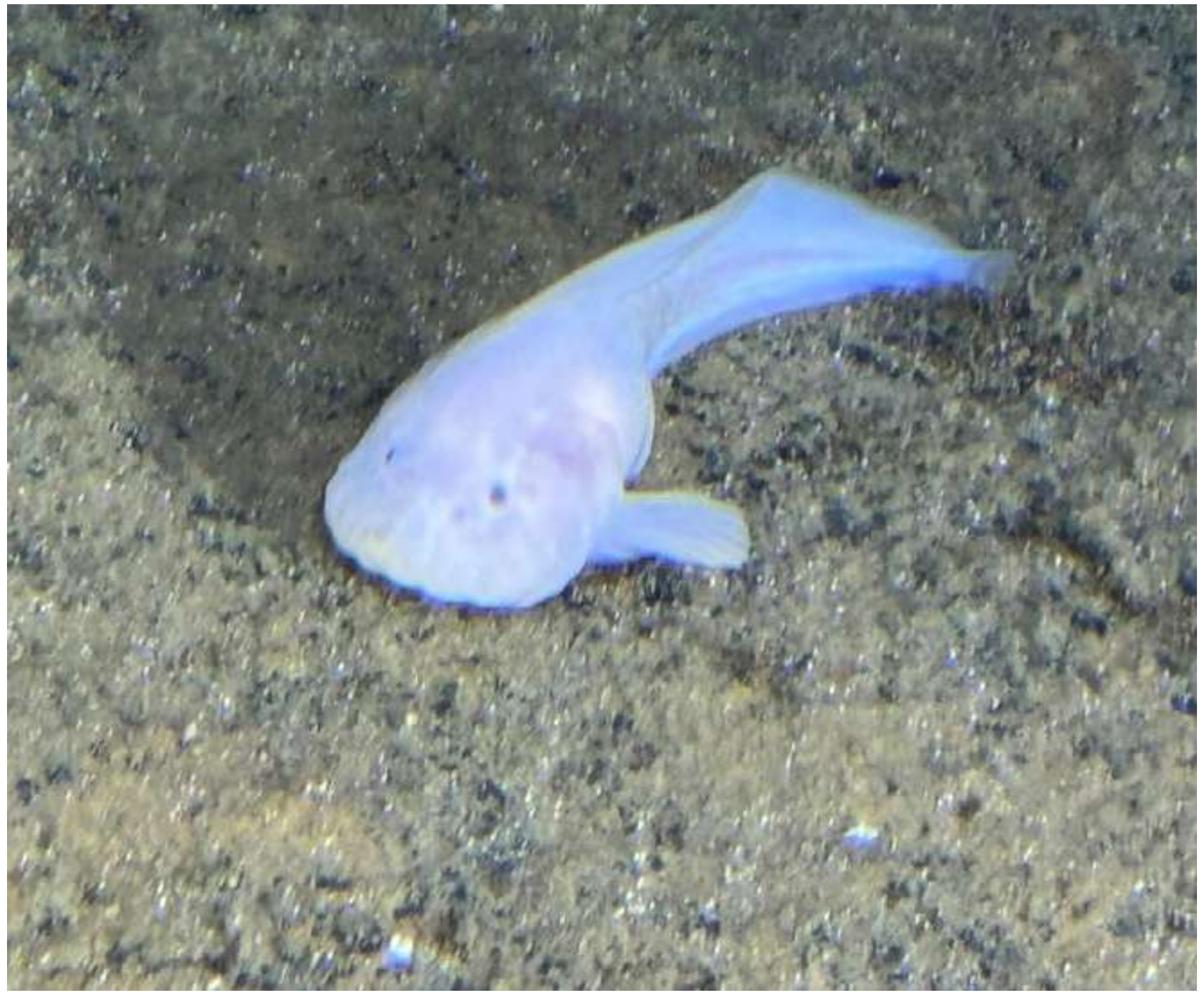
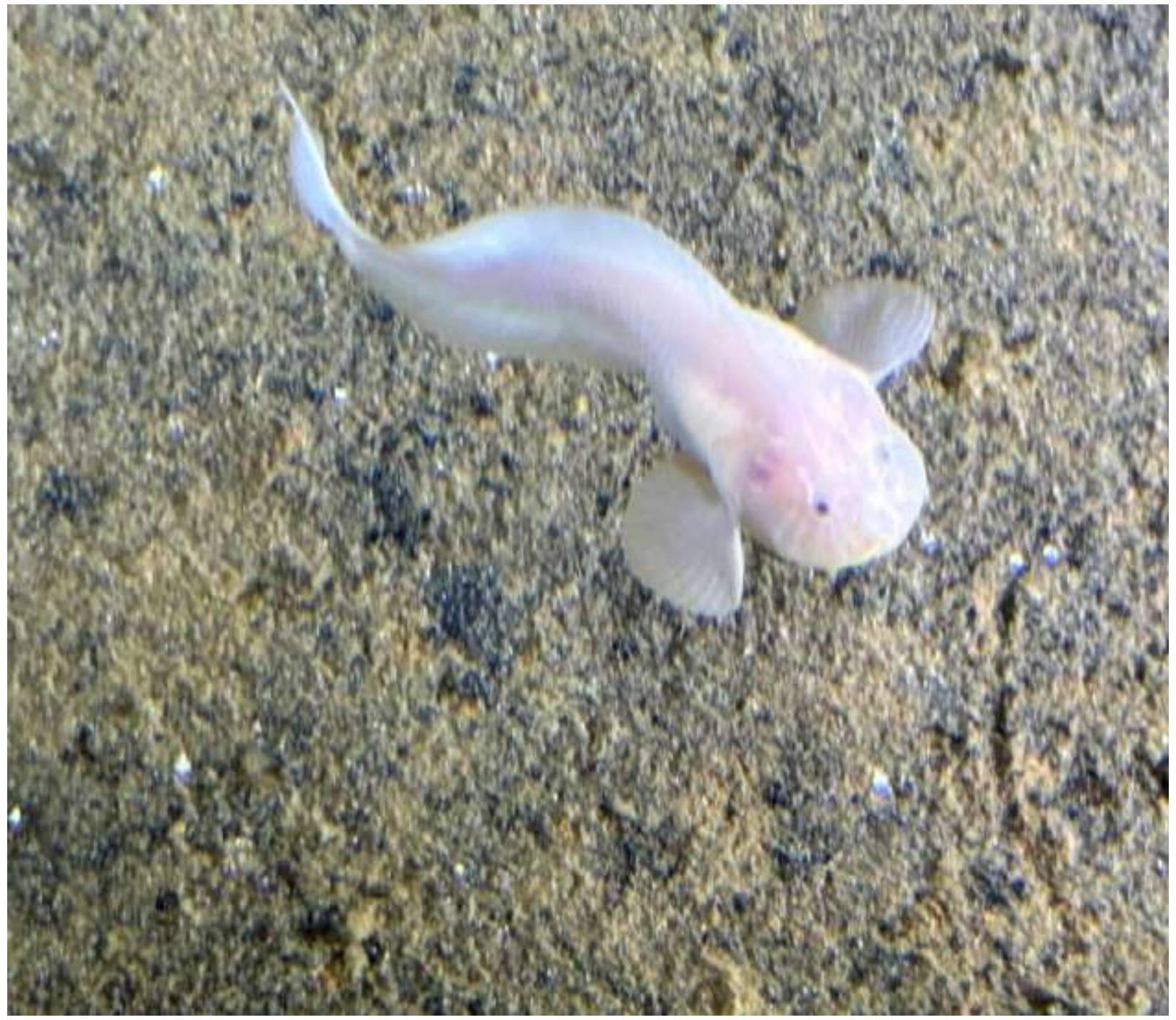
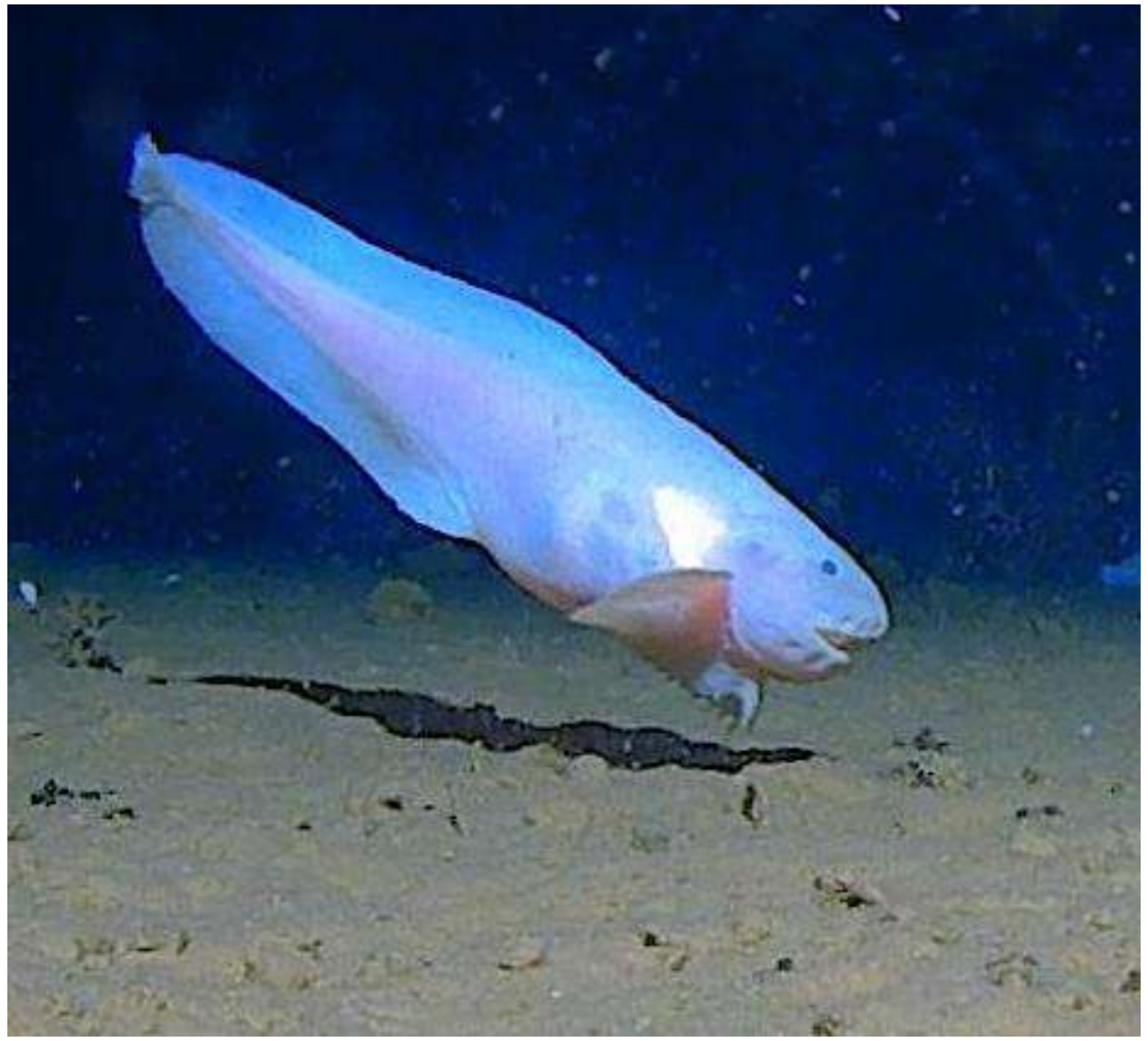

Atypical hadal snailfish body form; akin to Notoliparis kermadecensis; N. stewarti; Pseudoliparis swirei and P. belyaevi where the head is small and wide; small eyes; and the lateral profile anteriorly rounded. The head depth is about equal to body depth.

Echinodermata

Asteroidea; Brisingida; Brisingida fam. inc.1

NC4_BK2_4700_NOR_0071

NC4_BK2_4700_IMG_5214

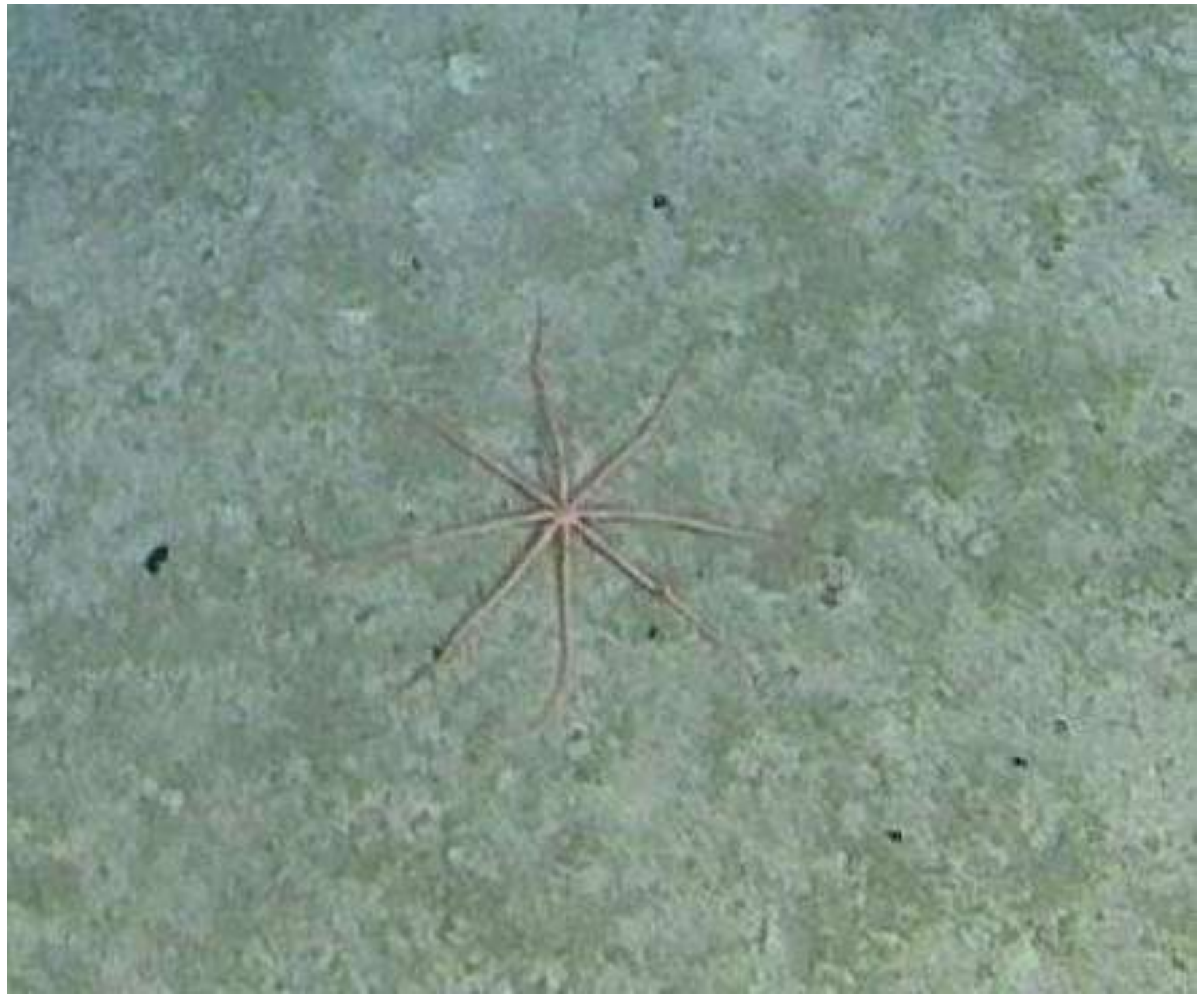

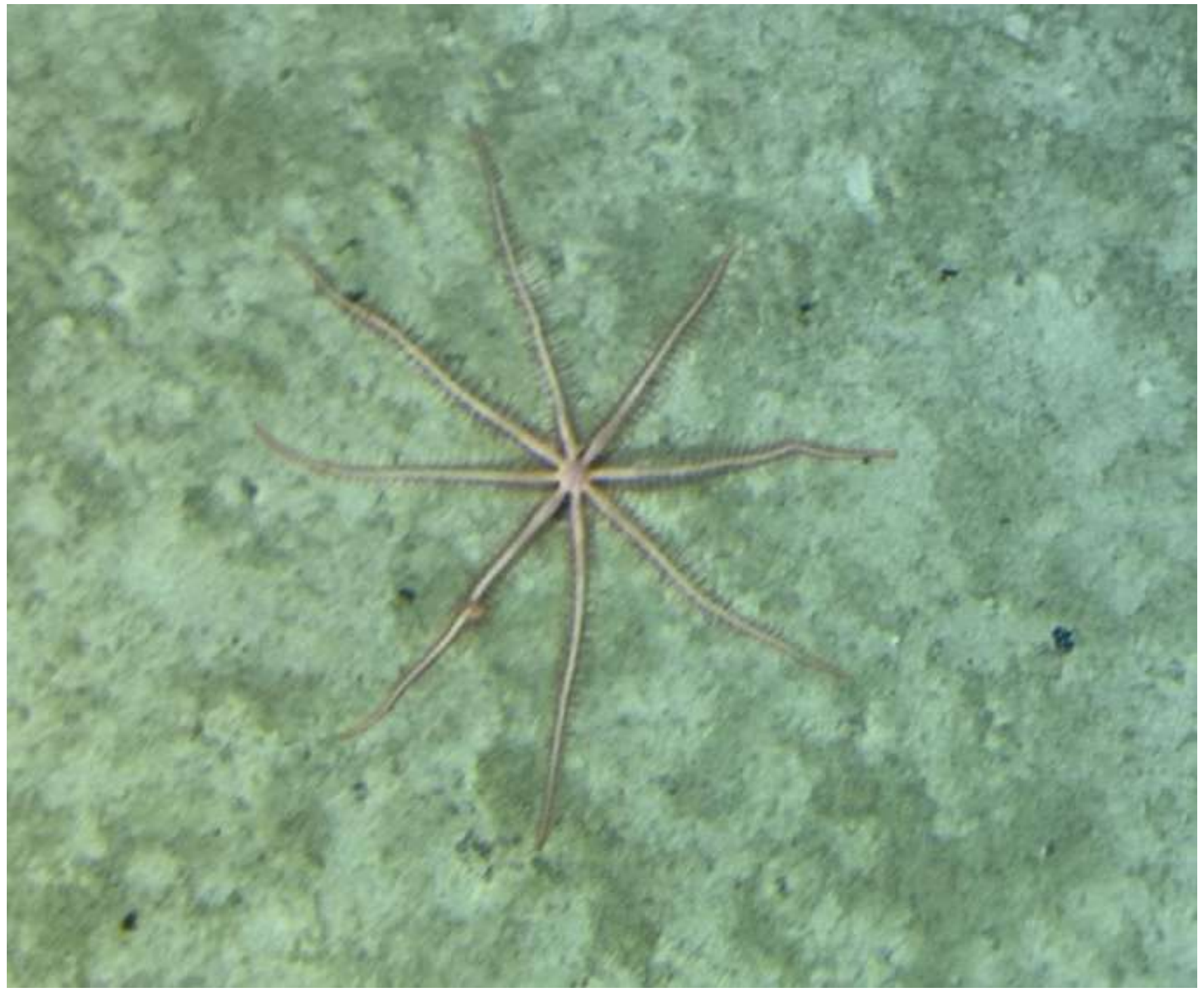

Pink ophiuroid-like appearance with small circular disc. Legs are long and number more than 5 with 8 arms observed.

Asteroidea; *Brisingida;* Brisingida fam. inc. 2

NC4_BK2_4700_NOR_0064
NC4_BK2_4700_IMG_5051
NC4_BK2_4700_NOR_0072

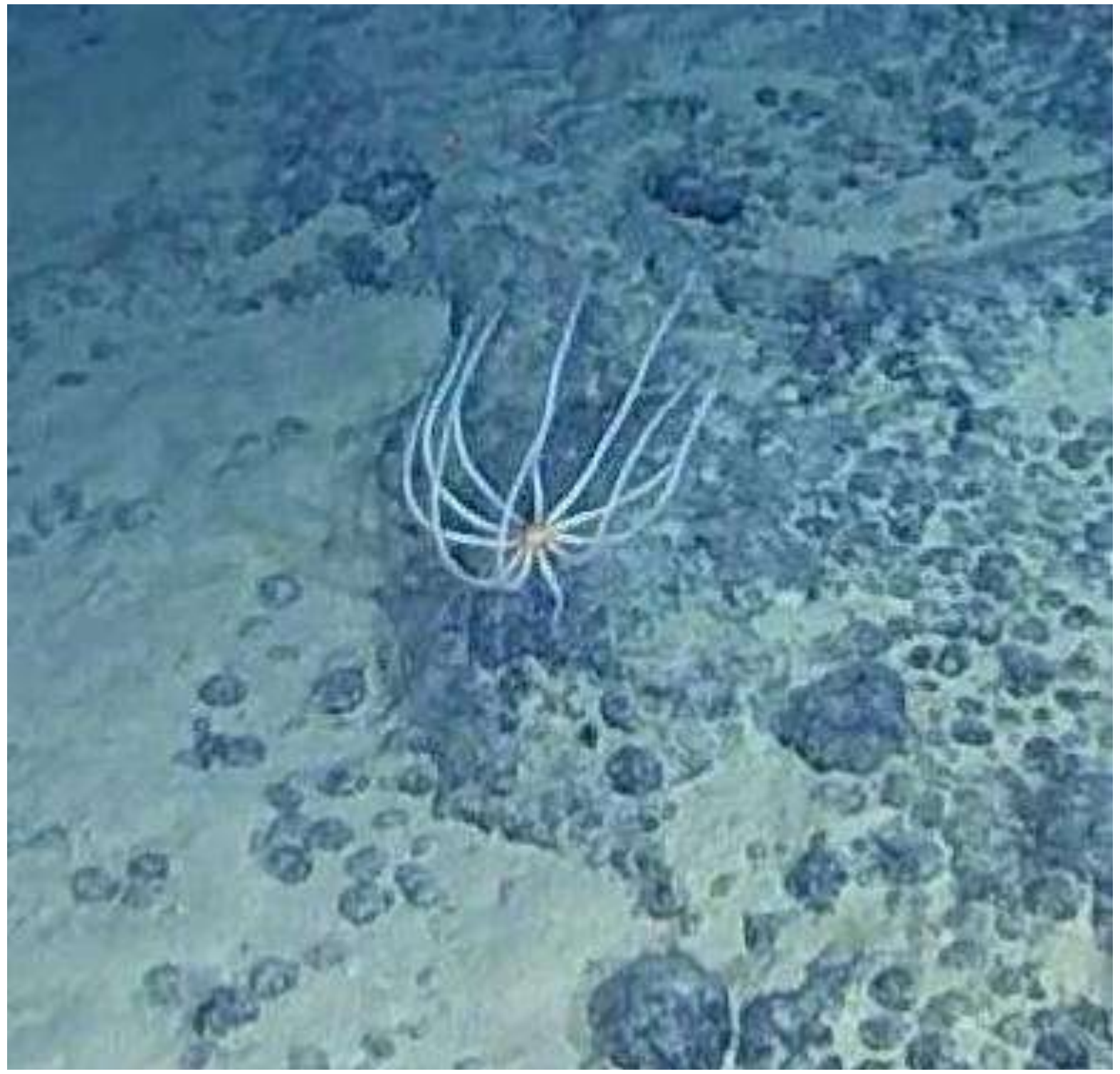
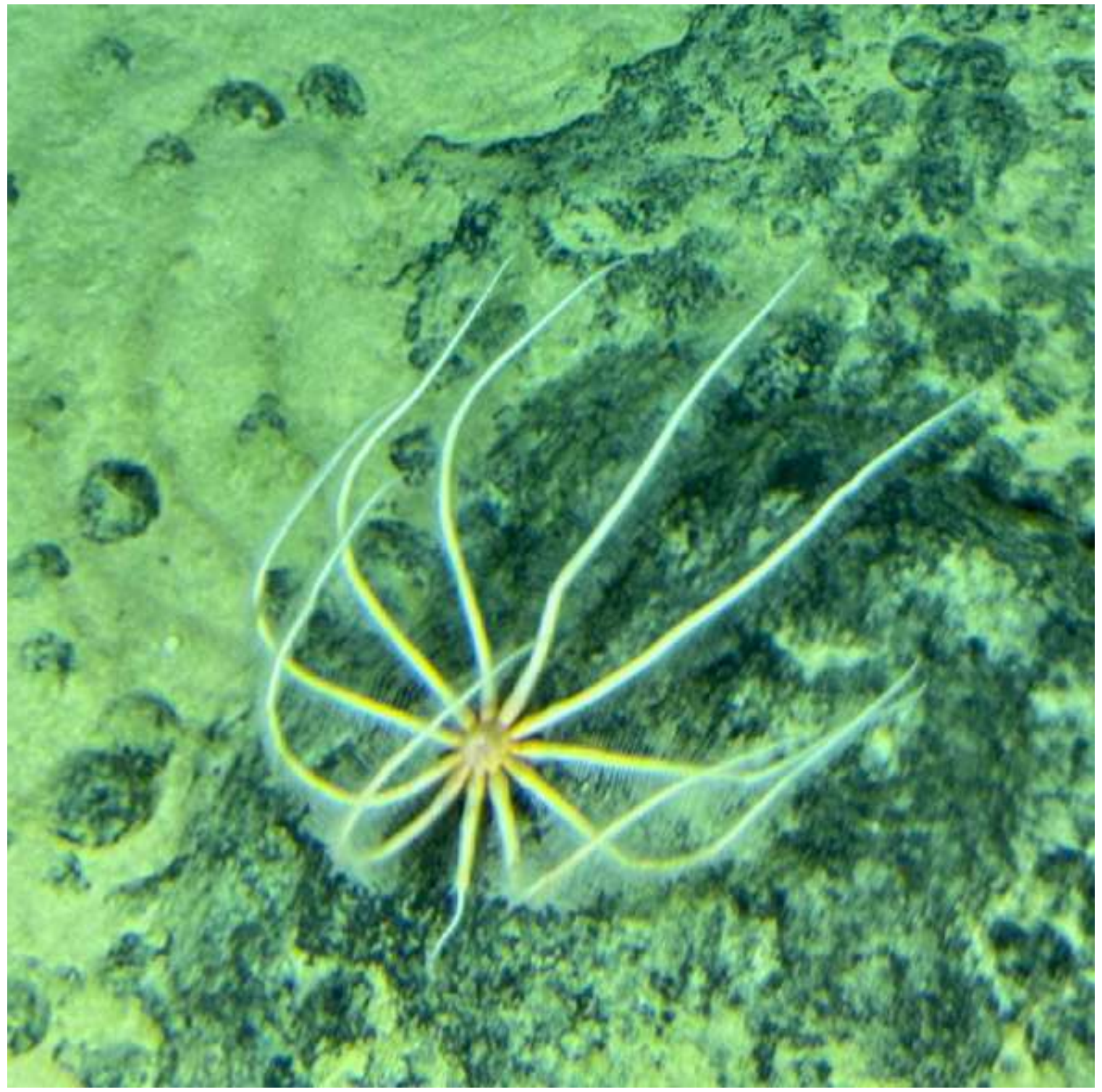
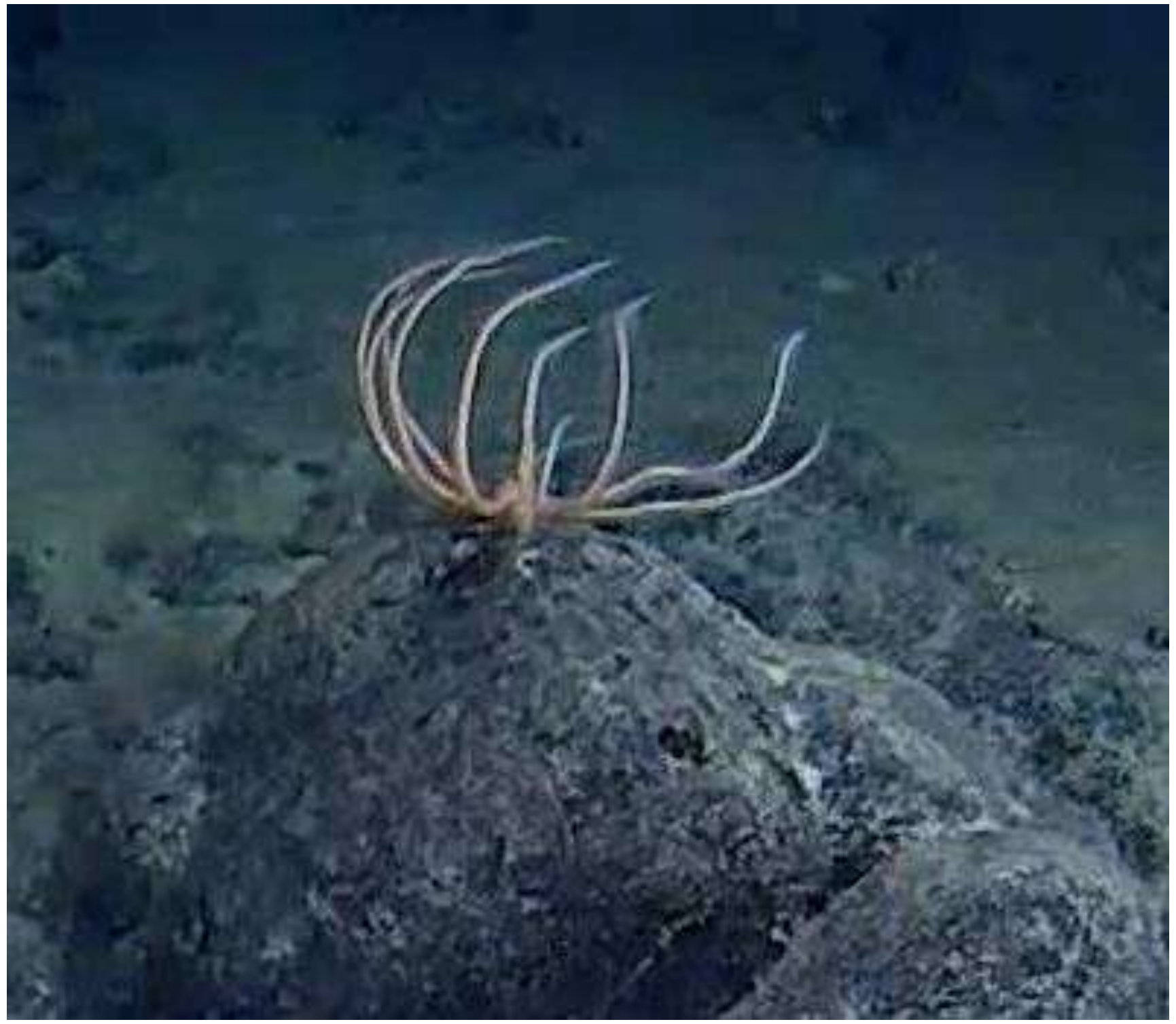

Pale, ophiuroid-like appearance with small circular disc. Arms are long, have feathery pinnules and number 11-12 arms.

Asteroidea; *Brisingida;* Brisingida fam. inc. 3

NC3_BK2_6000_IMG_2128

NC3_BK2_6000_IMG_2130

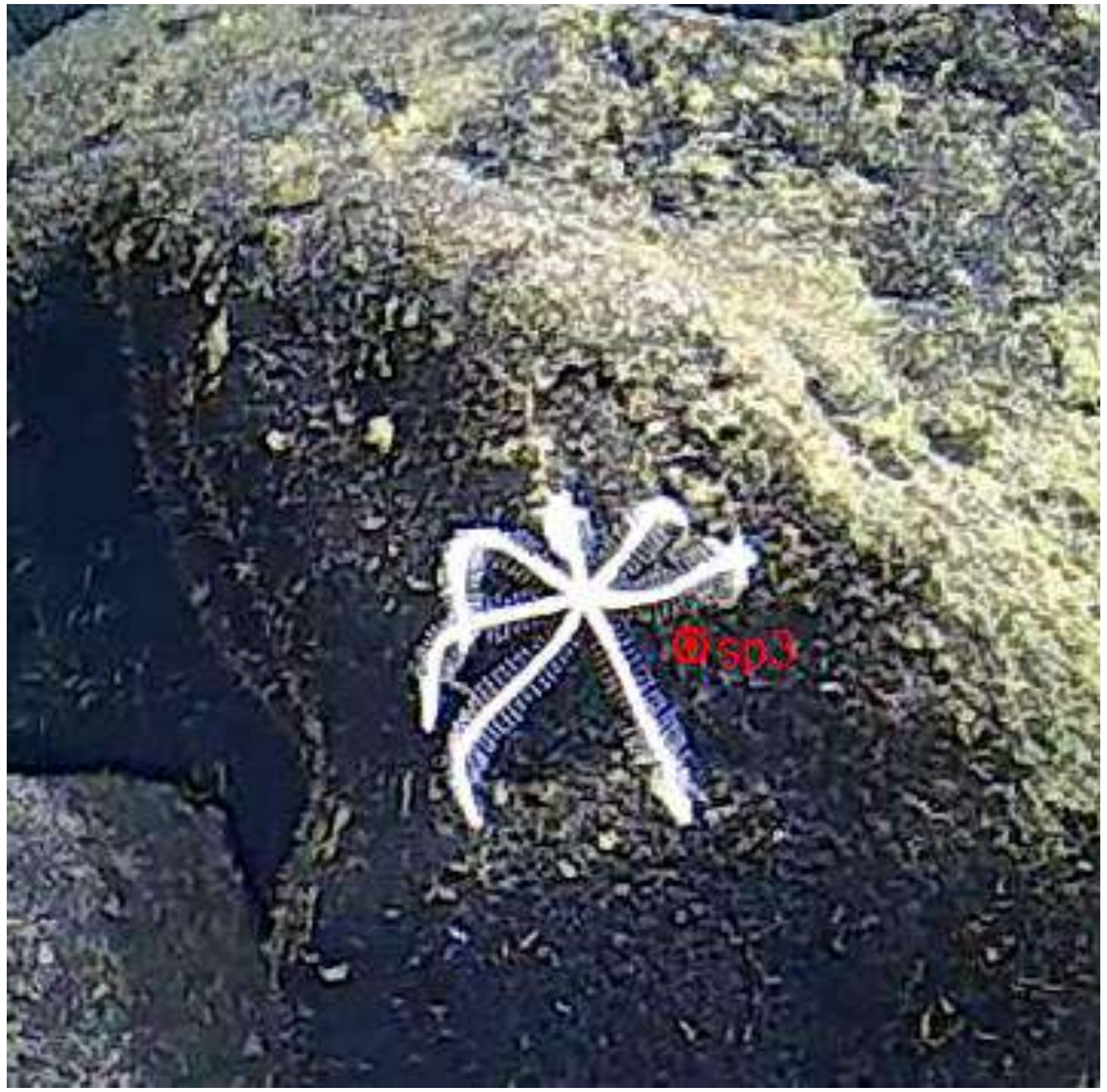

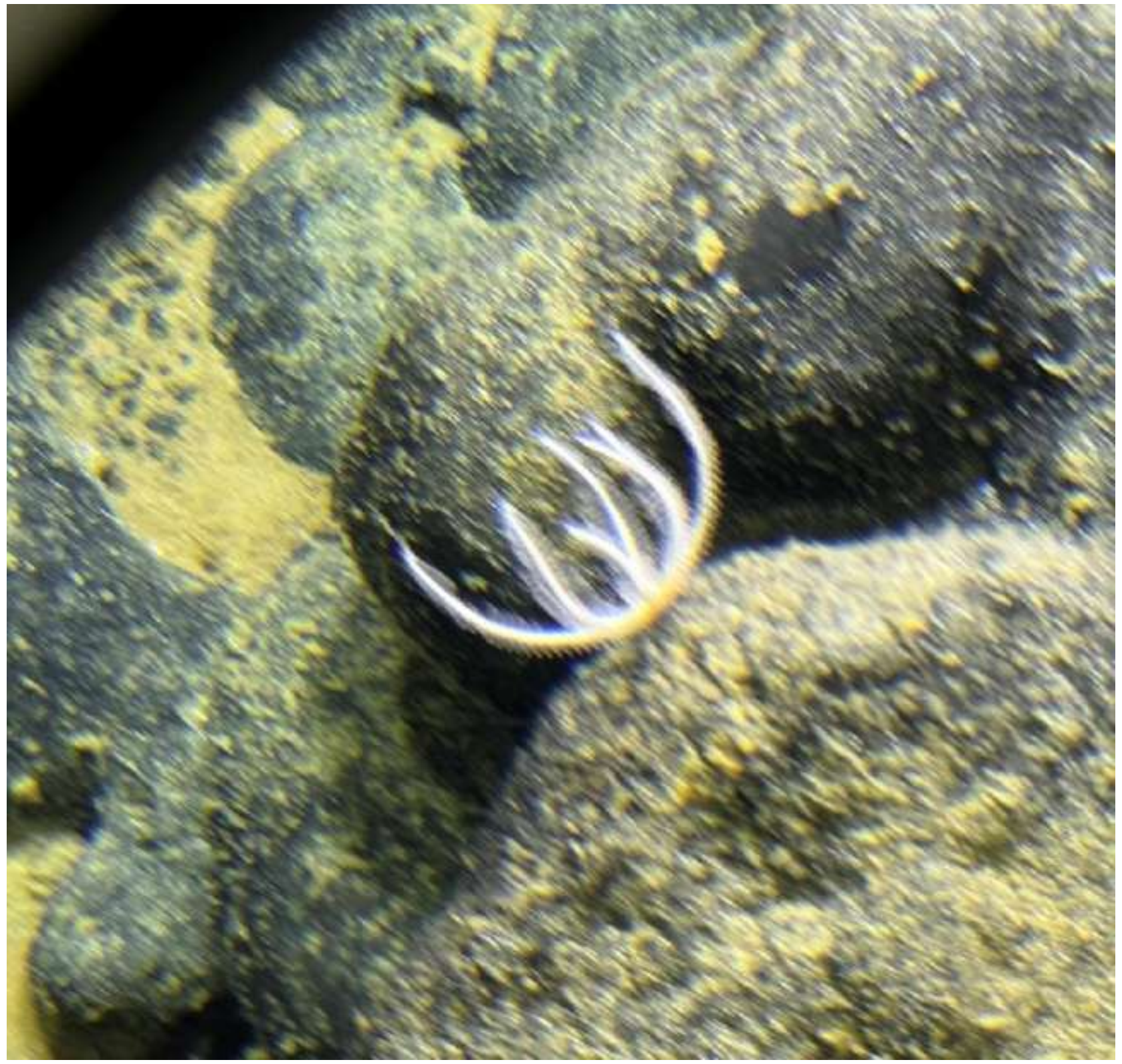

Pale, ophiuroid-like appearance with small circular disc. Arms are long in relation to the disc, have feathery pinnules and number 7 arms. Overall size appears small

Asteroidea; Brisingida; Freyellidae; Freyellidae gen. inc. 1

NC3_BK2_6000_IMG_2009
NC4_BK4_4700

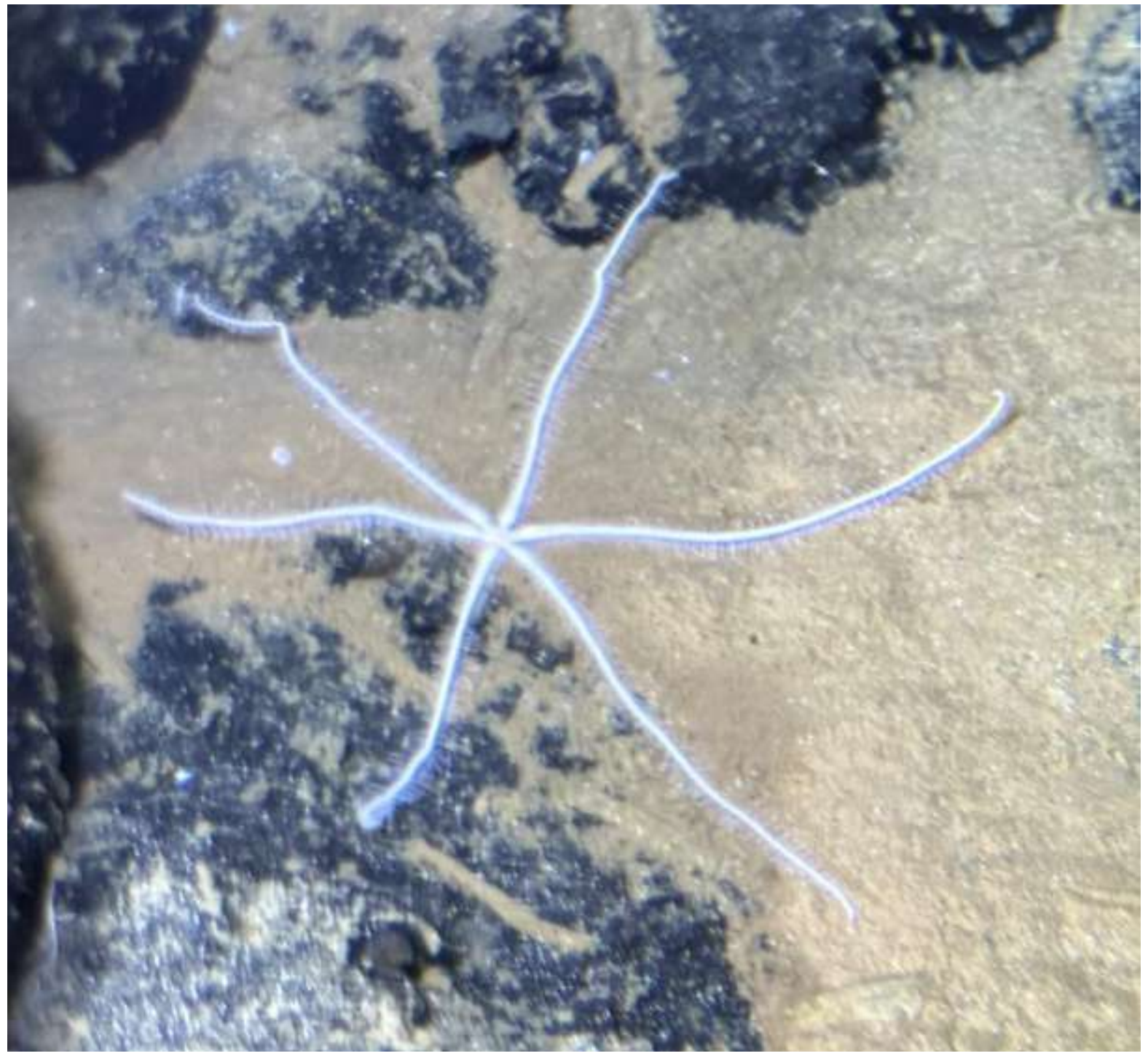

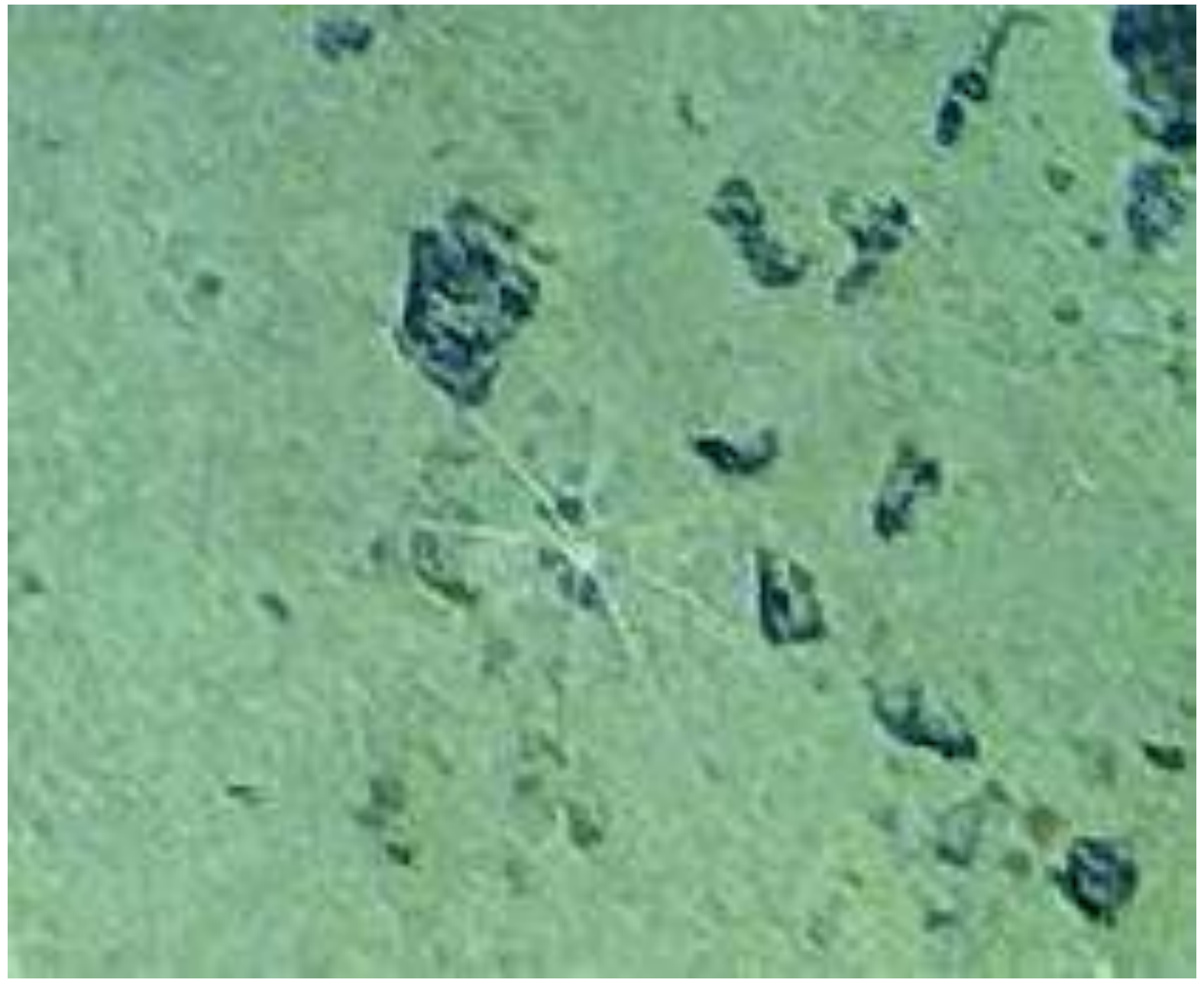

Pale ophiuroid-like appearance with small circular disc and 6 long, thin arms

Asteroidea; Paxillosida Porcellanasteridae; Porcellanasteridae spp.

NC4_BK3_6900

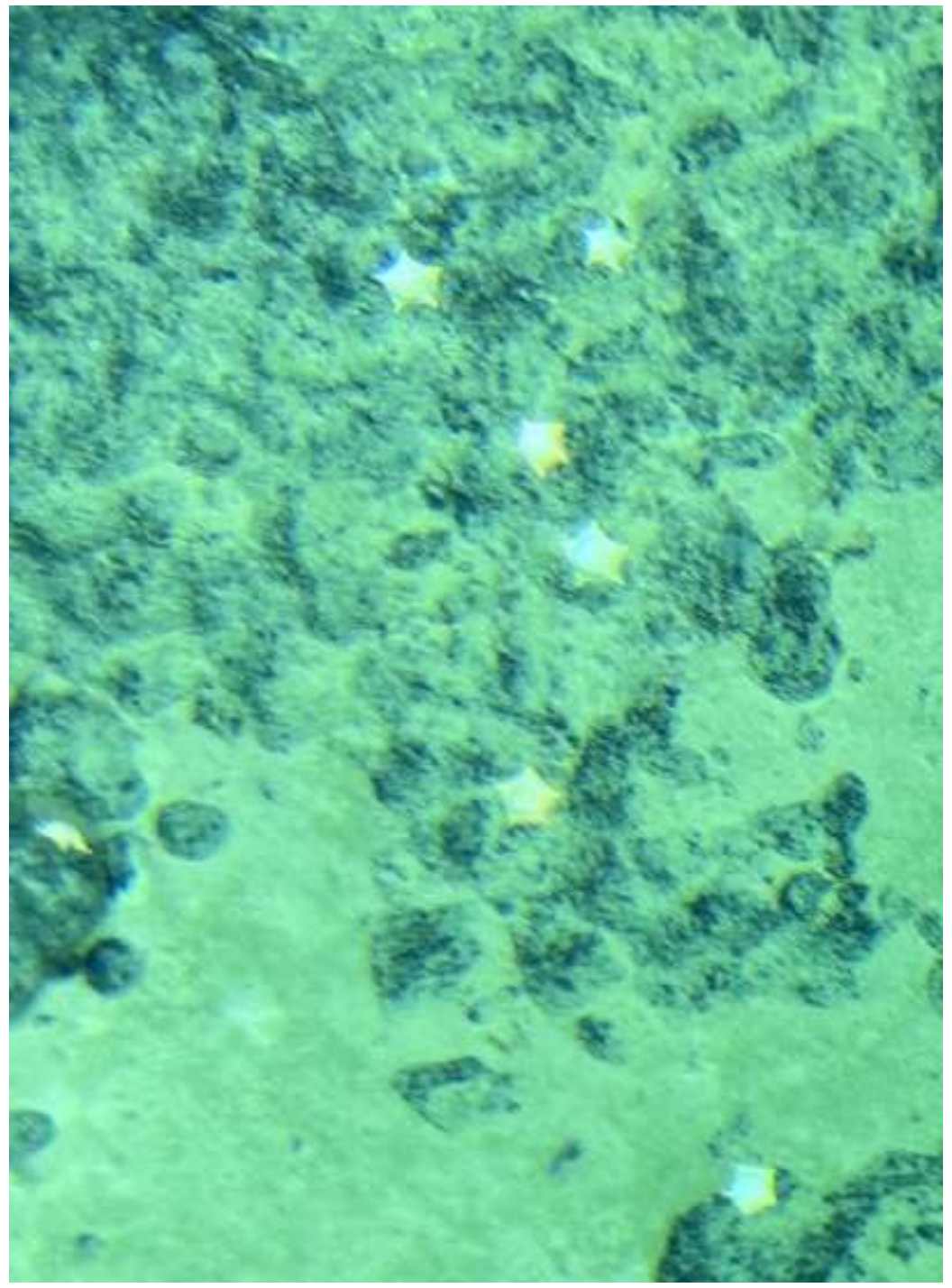

*High abundance, very small. ‘**chonkier**’ legs than sp1. Almost hymenaster like.*

Asteroidea; Paxillosida Porcellanasteridae; Porcellanasteridae gen. indet. 1

NC1_BK2_6700_NOR_0071
NC3_BK2_6000_NOR_0079

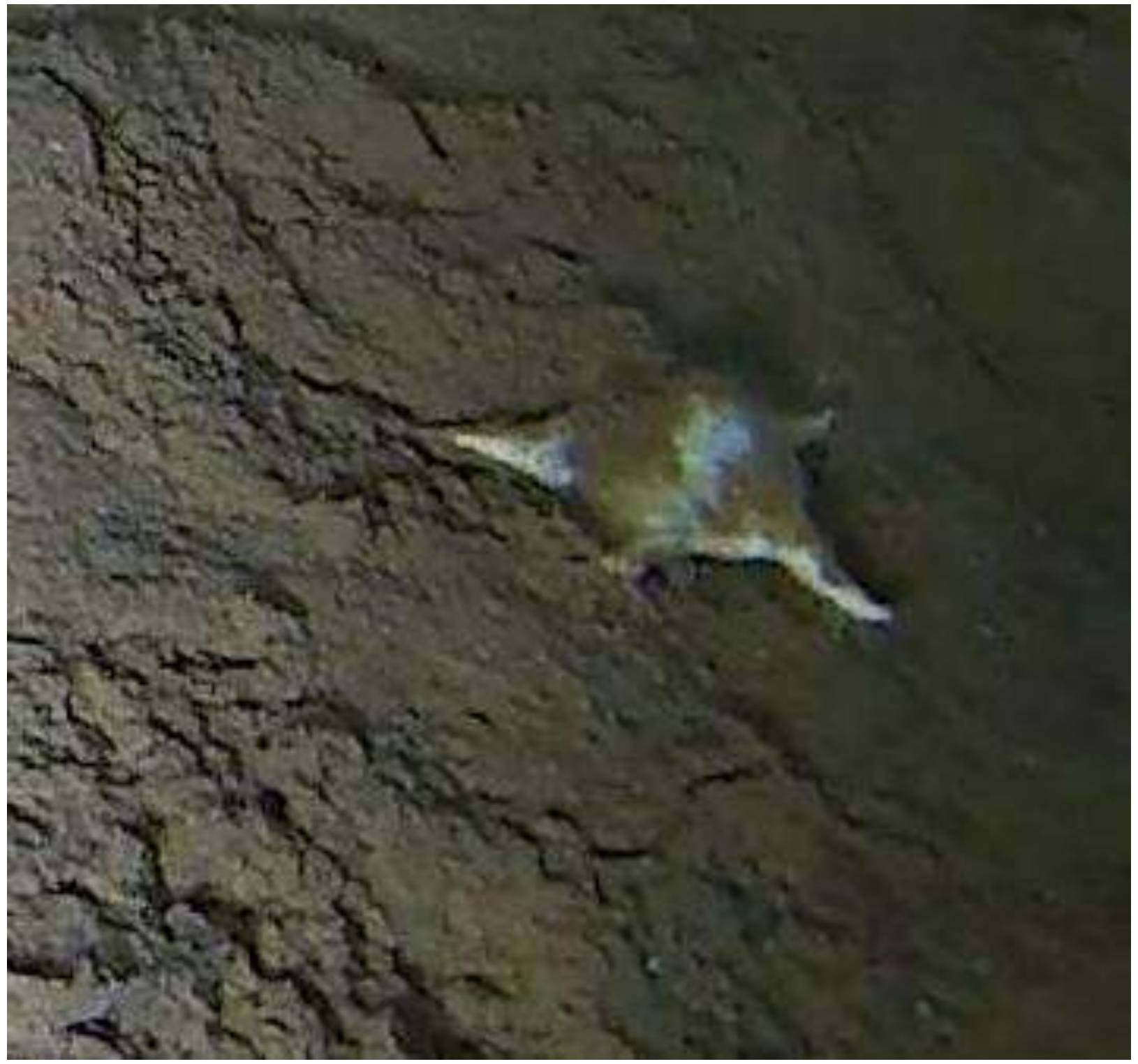
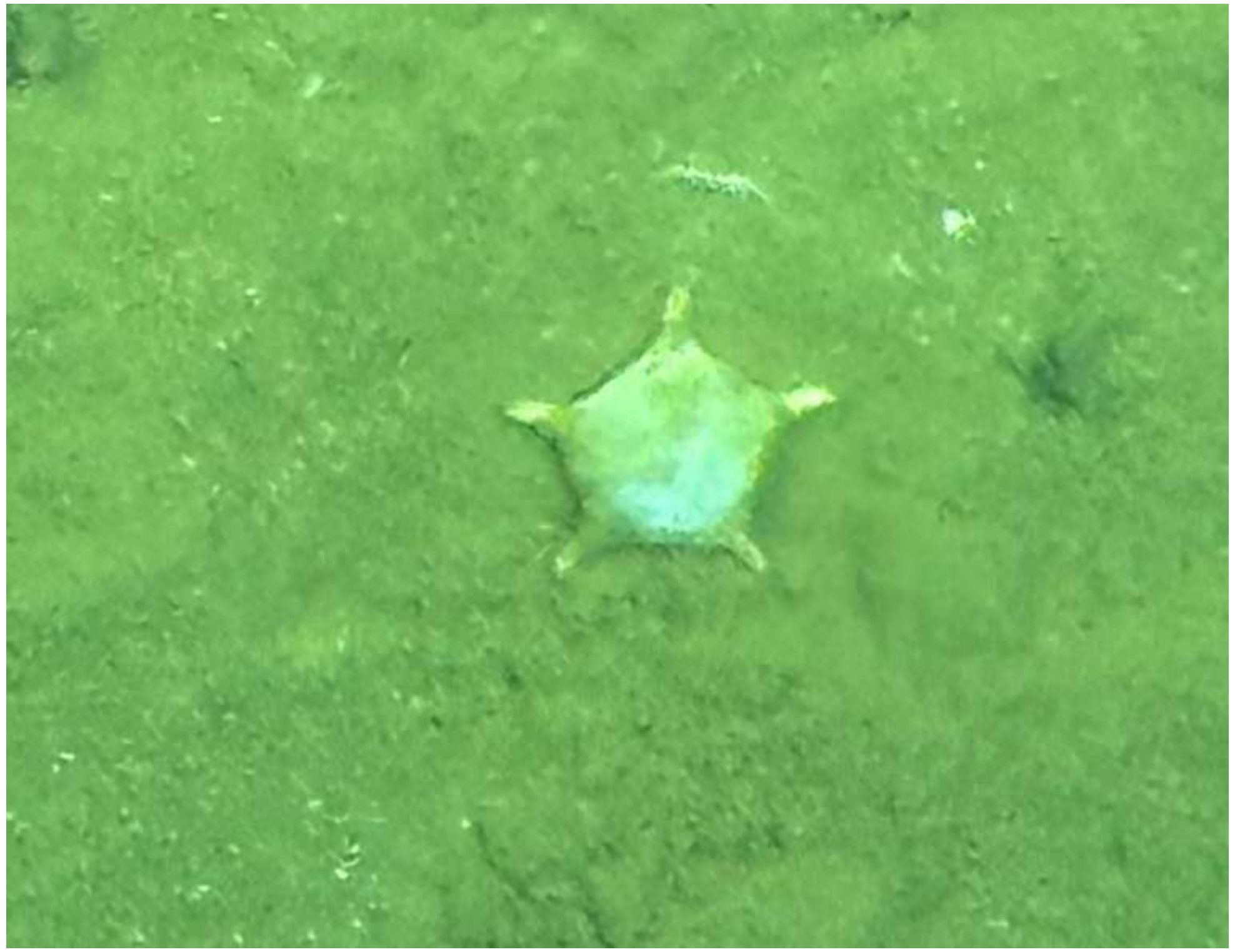
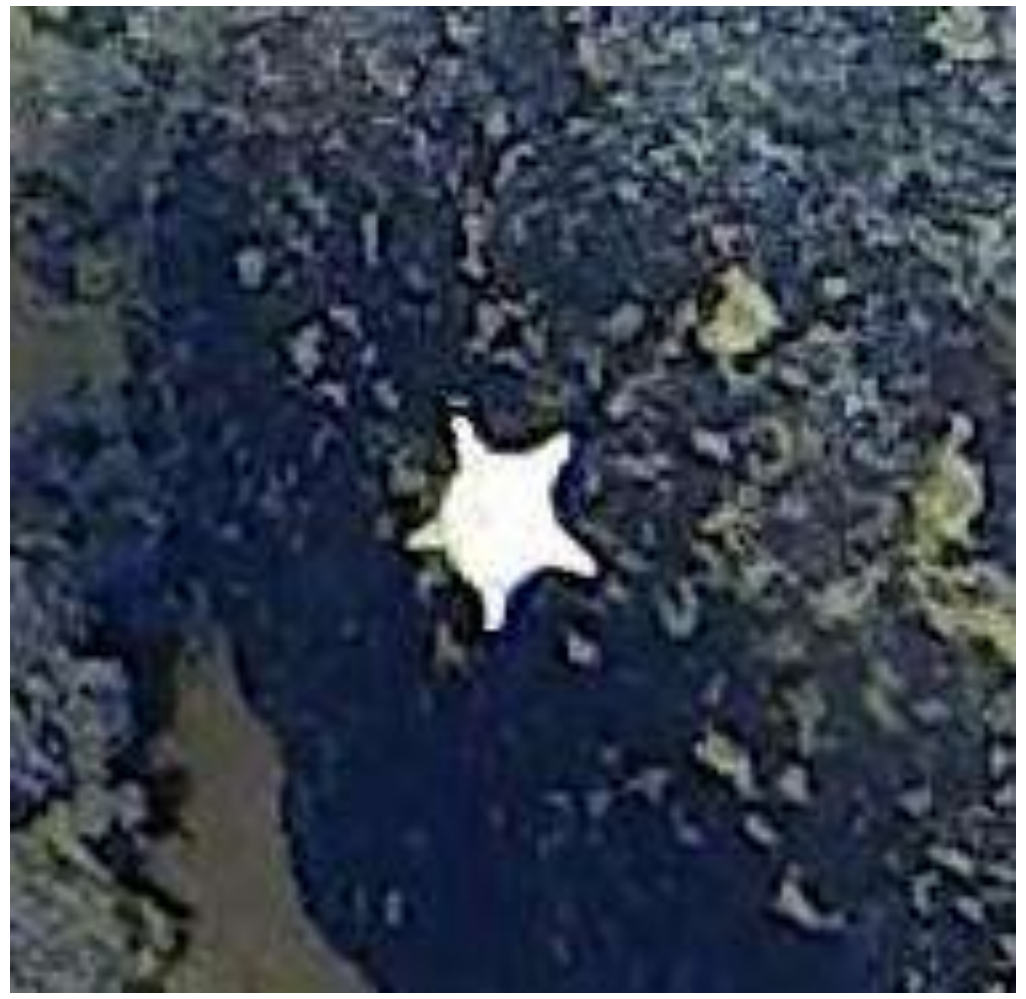

Pale stellate-shaped body with a dome-shaped disc covered in small pores on the aboral-side that are evenly dispersed over the disc. Limbs project from the robust disc as short thin arms.

Asteroidea; Paxillosida, Astropectinidae gen. indet. 1 (cf. *Dytaster)*

NC2_BK1_3200_IMG_7503

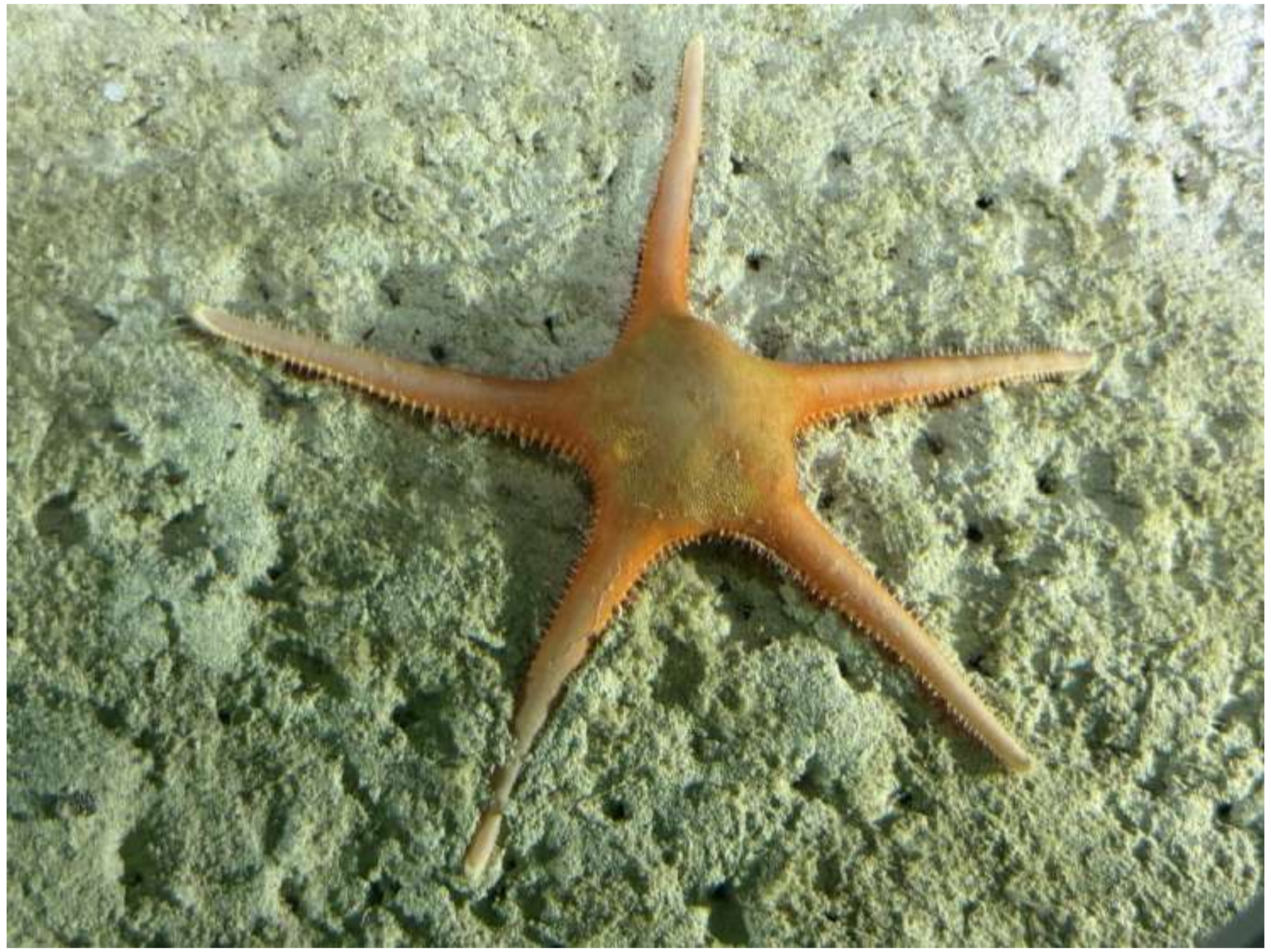

Yellowish-orange stellate-shaped body with a robust, pentagonal disc covered in small pores on the aboral-side that are dispersed over the disc. Limbs project from the paler, yellowish , pentagonal disc as orange, long thin arms that are distally off-white. Small, pale spines line the outer perimeter of the limbs.

Asteroidea; Paxillosida, Astropectinidae gen. indet. 2 (cf. *Persephonaster*)

NC2_BK2_3800_IMG_7898

NC4_CR11_3900_NOR_0101

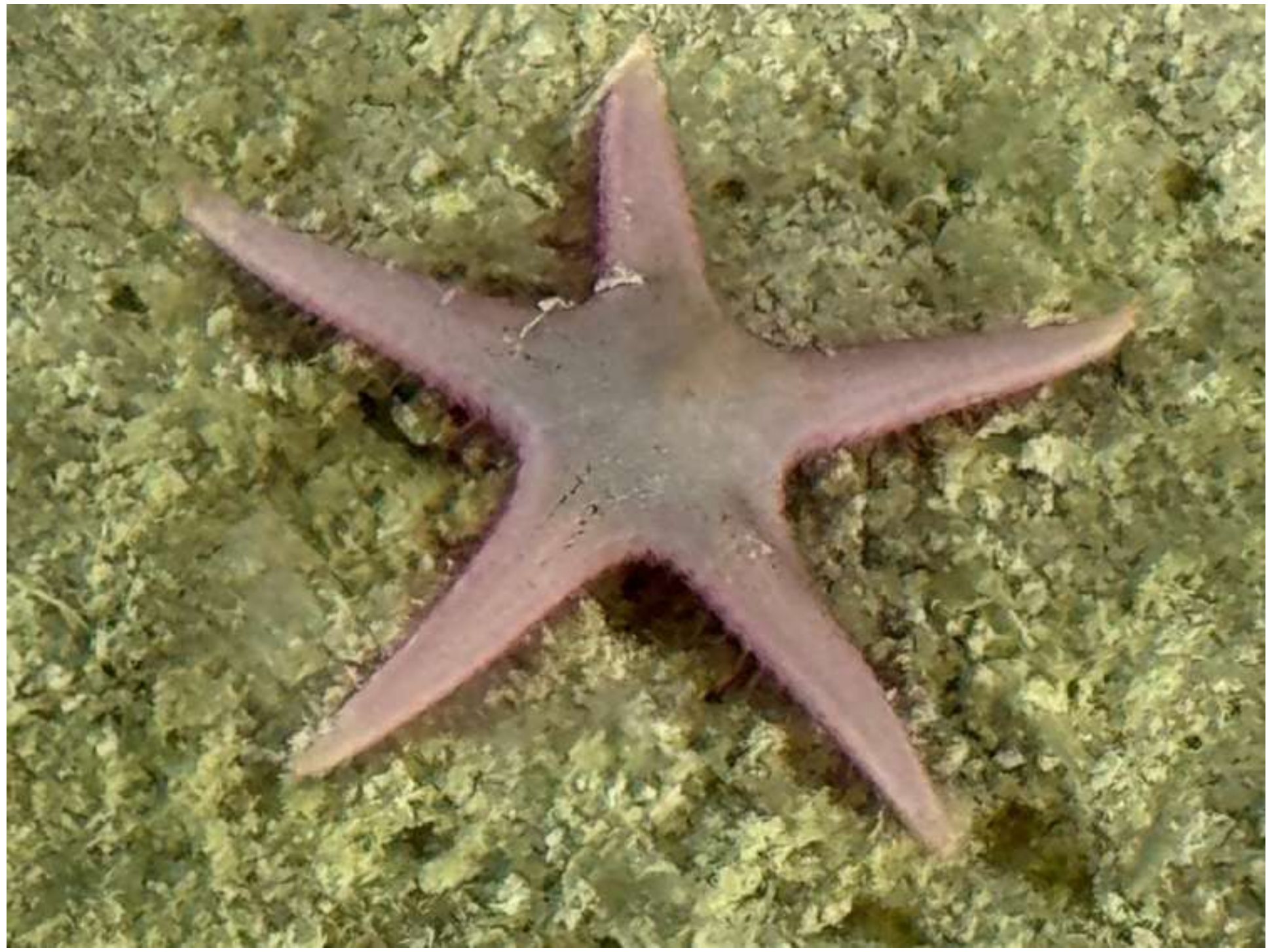

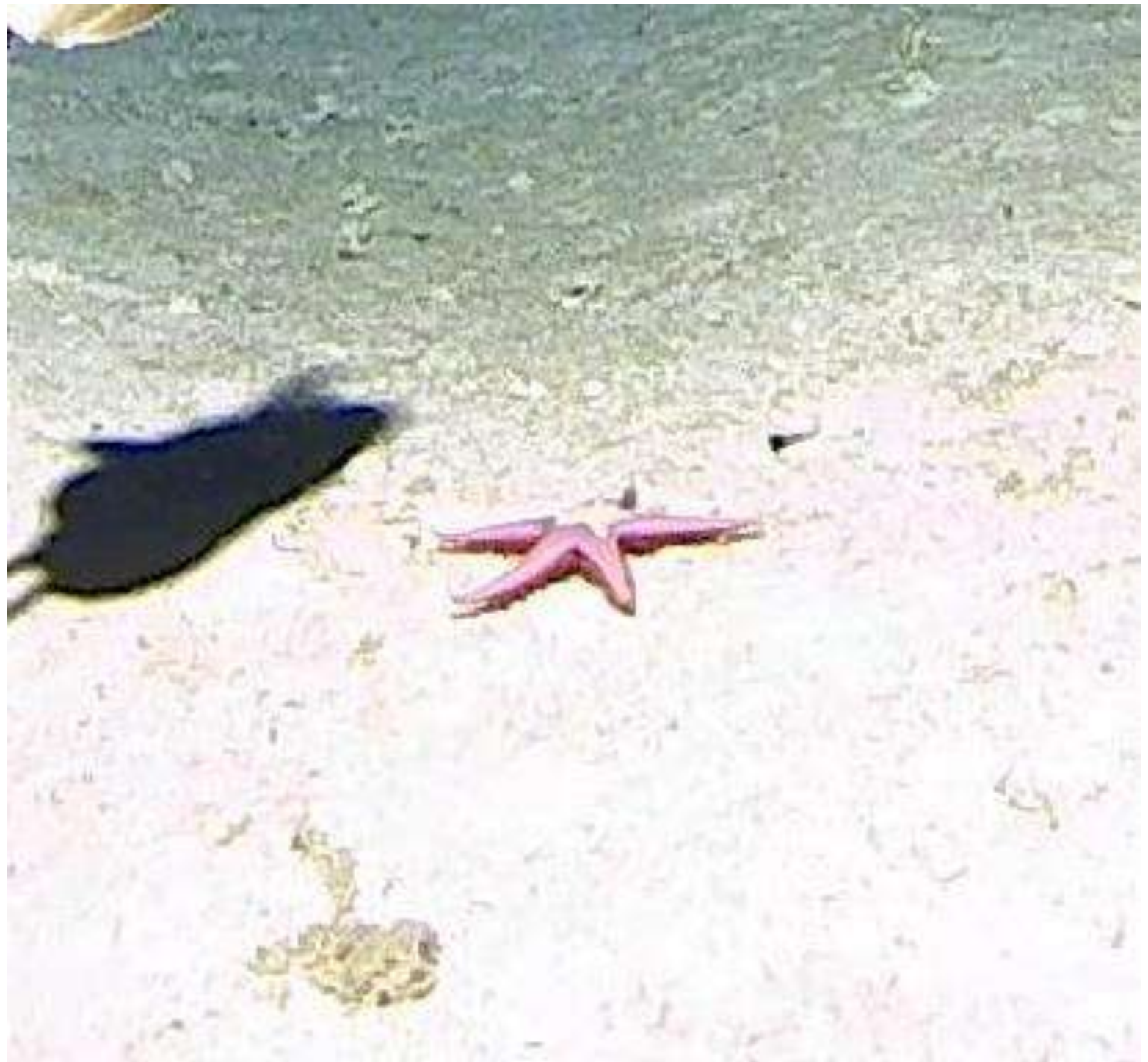

Dull purple-pink stellate-shaped body with a disc covered in small pores on the aboral-side that are dispersed along the base of the limbs. Limbs project from the darker, pentagonal disc as purple-pink arms. Long, thin, dark spines line the outer perimeter of the limbs.

Asteroidea; Valvatida, Goniasteridae, *Circeaster* sp. indet. 1

NC2_BK1_3200_IMG_7387

NC2_BK1_3200_IMG_7537

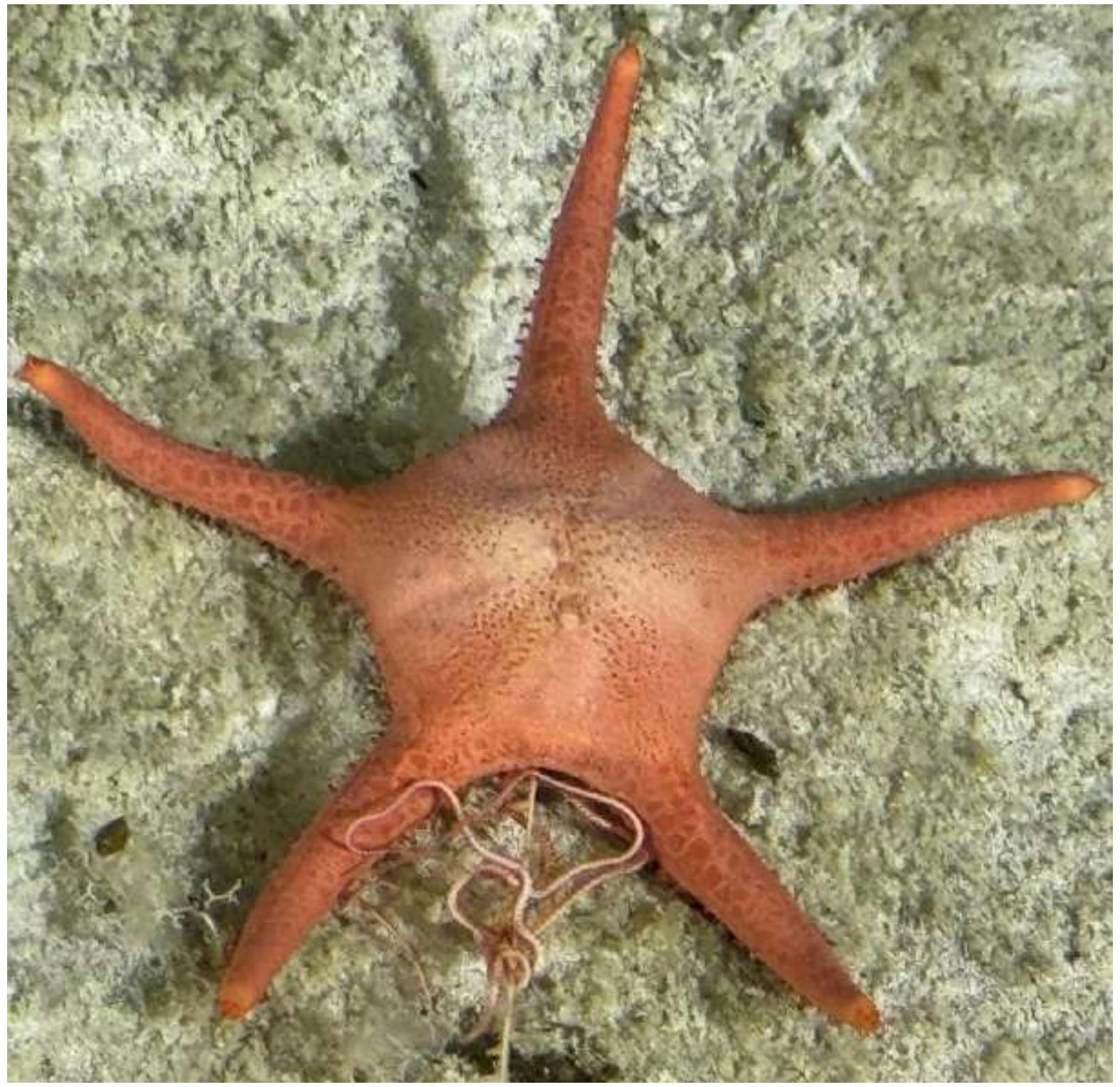

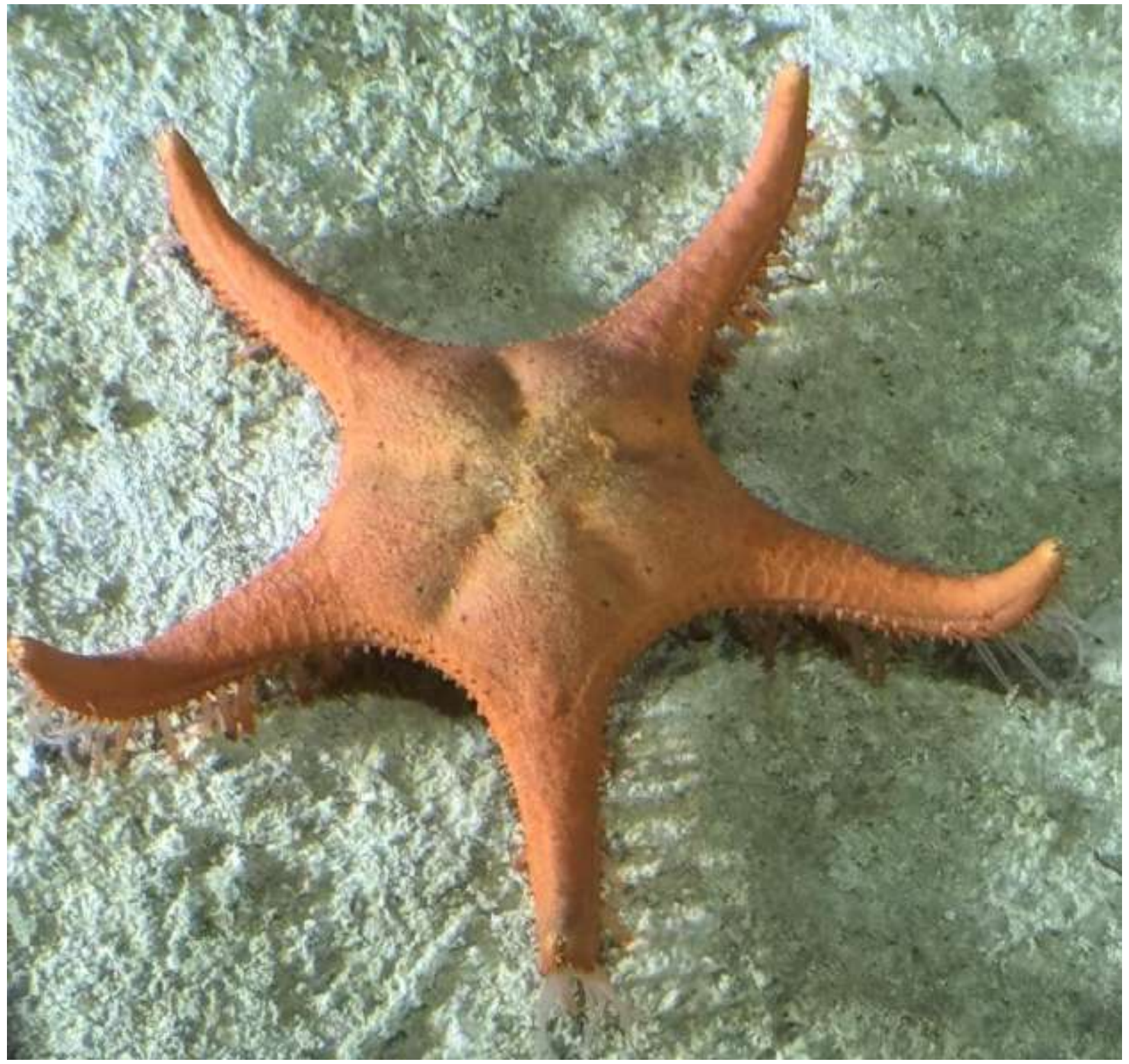

Red-pink body with a pale, pentagonal disc covered in small pores on the aboral-side that are dispersed over the disc. Limbs project from the pale, pentagonal disc as long thin arms that are darker than the disc. Small, dark spines line the outer perimeter of the limbs with long tube feet observed underneath.

Asteroidea; Valvatida;
Goniasteridae ; Goniasteridae gen. inc. 1
(cf. Bathycermastaster)

NC2_BK5_4600_IMG_0531

NC2_BK5_4600_IMG_0534

NC2_BK5_4600_IMG_0536

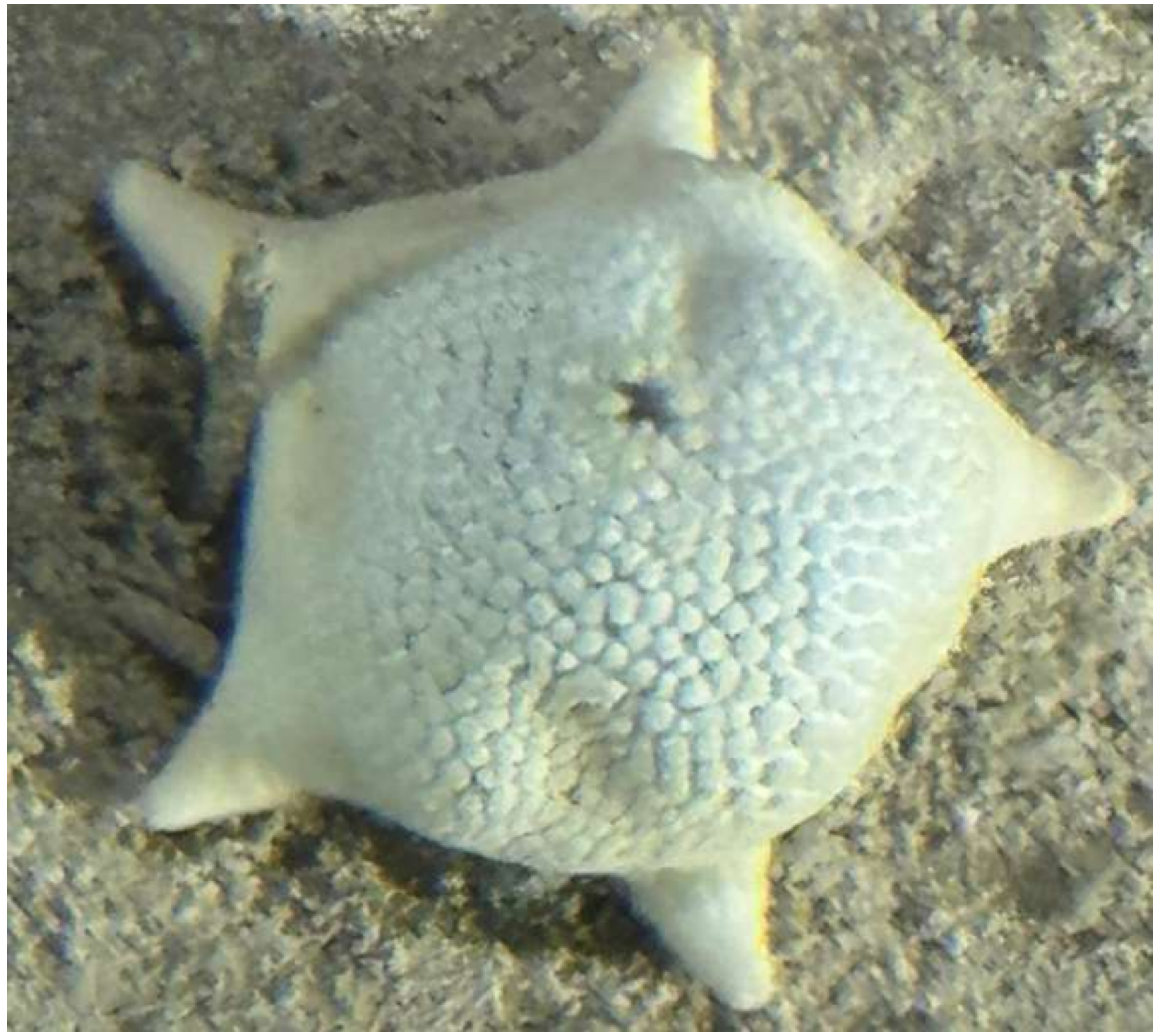

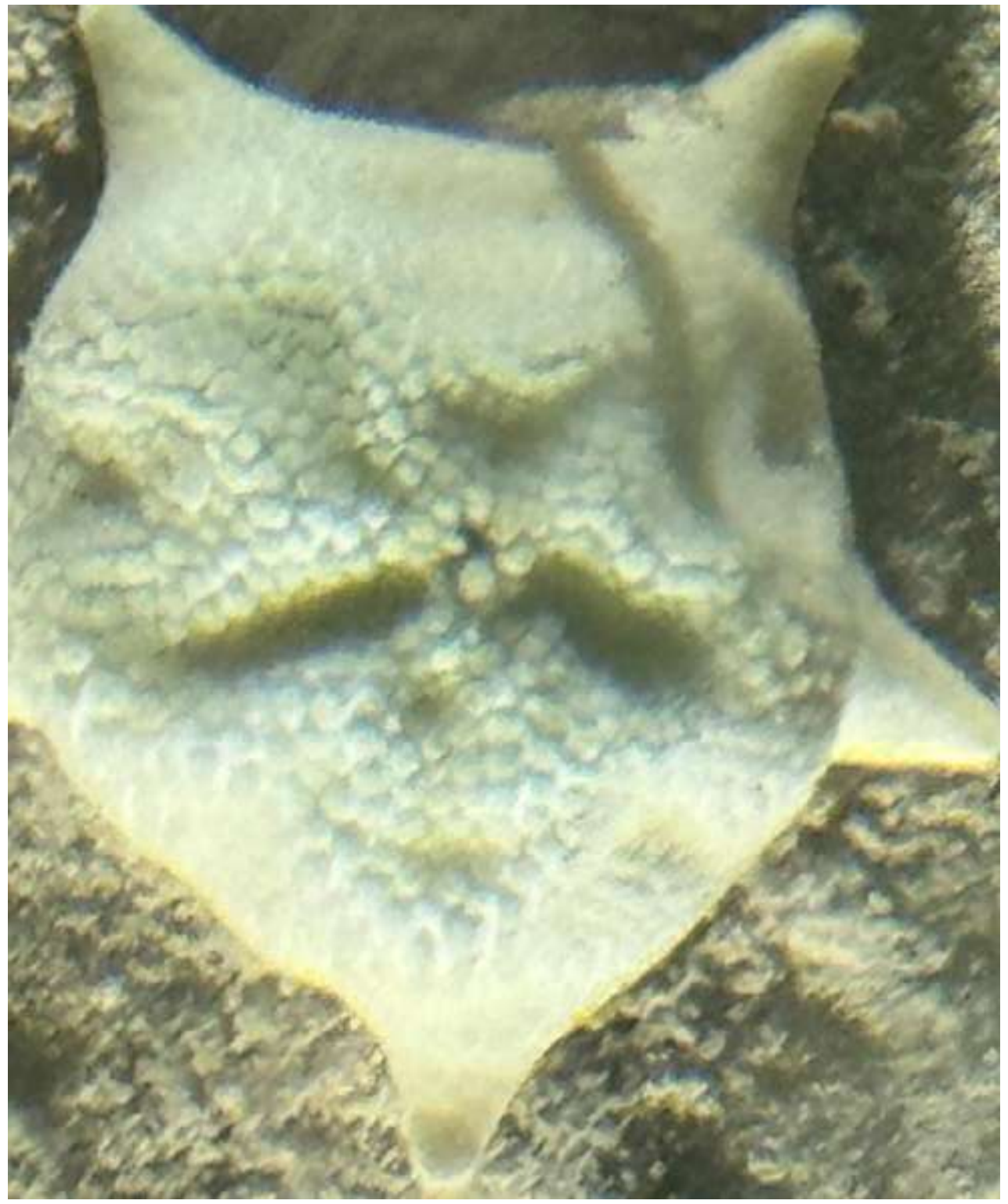

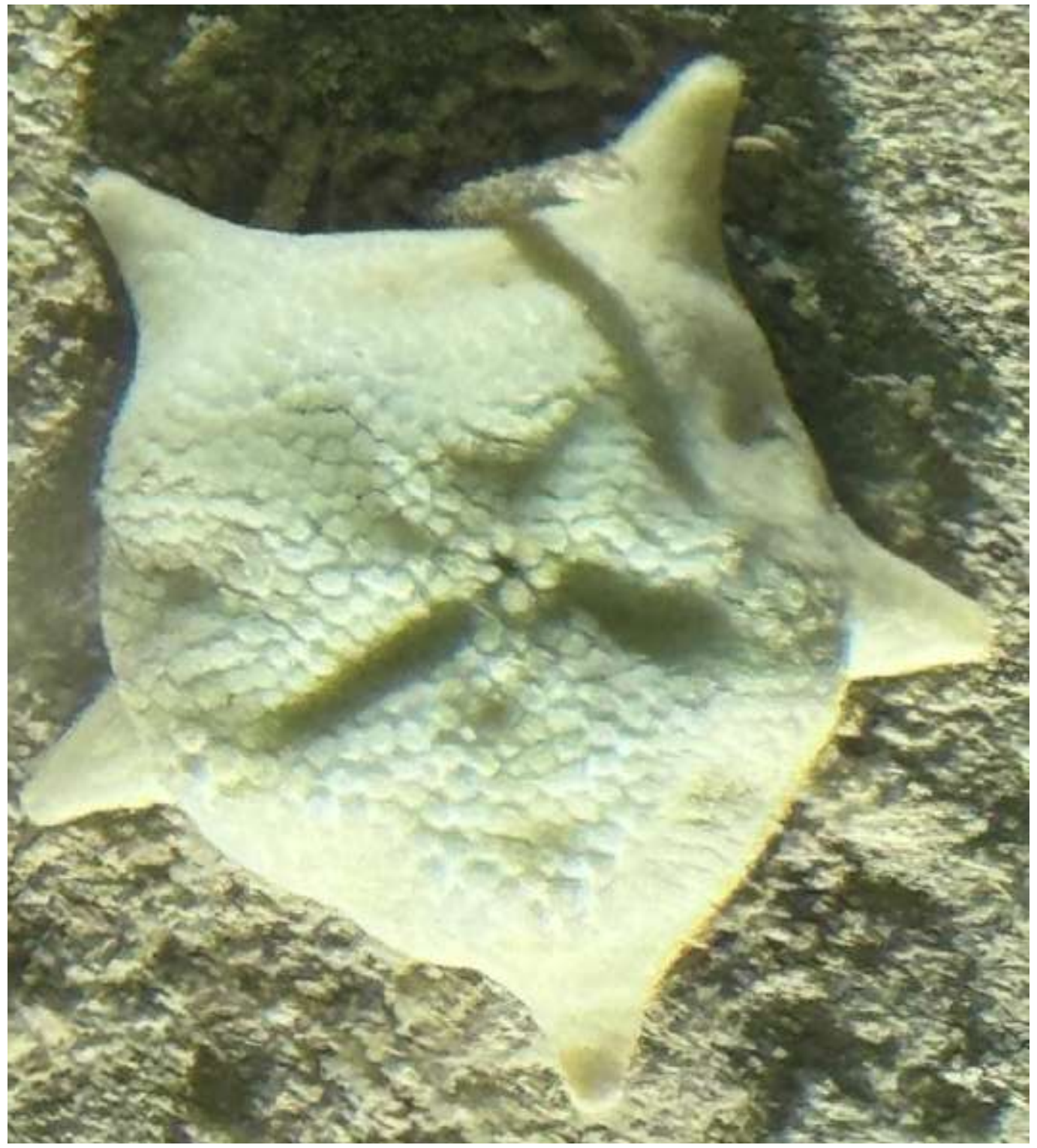

Pale stellate-shaped body with a dome-shaped disc covered in an uneven, knobbly surface on the aboral-side of the disc. Limbs project from the robust disc as short, thick arms.

Asteroidea;
Valvatida, Goniasteridae gen. indet. 2 *(cf. Bathycermastaster)*

NC2_BK5_4600_IMG_0294

NC2_BK5_4600_IMG_0390

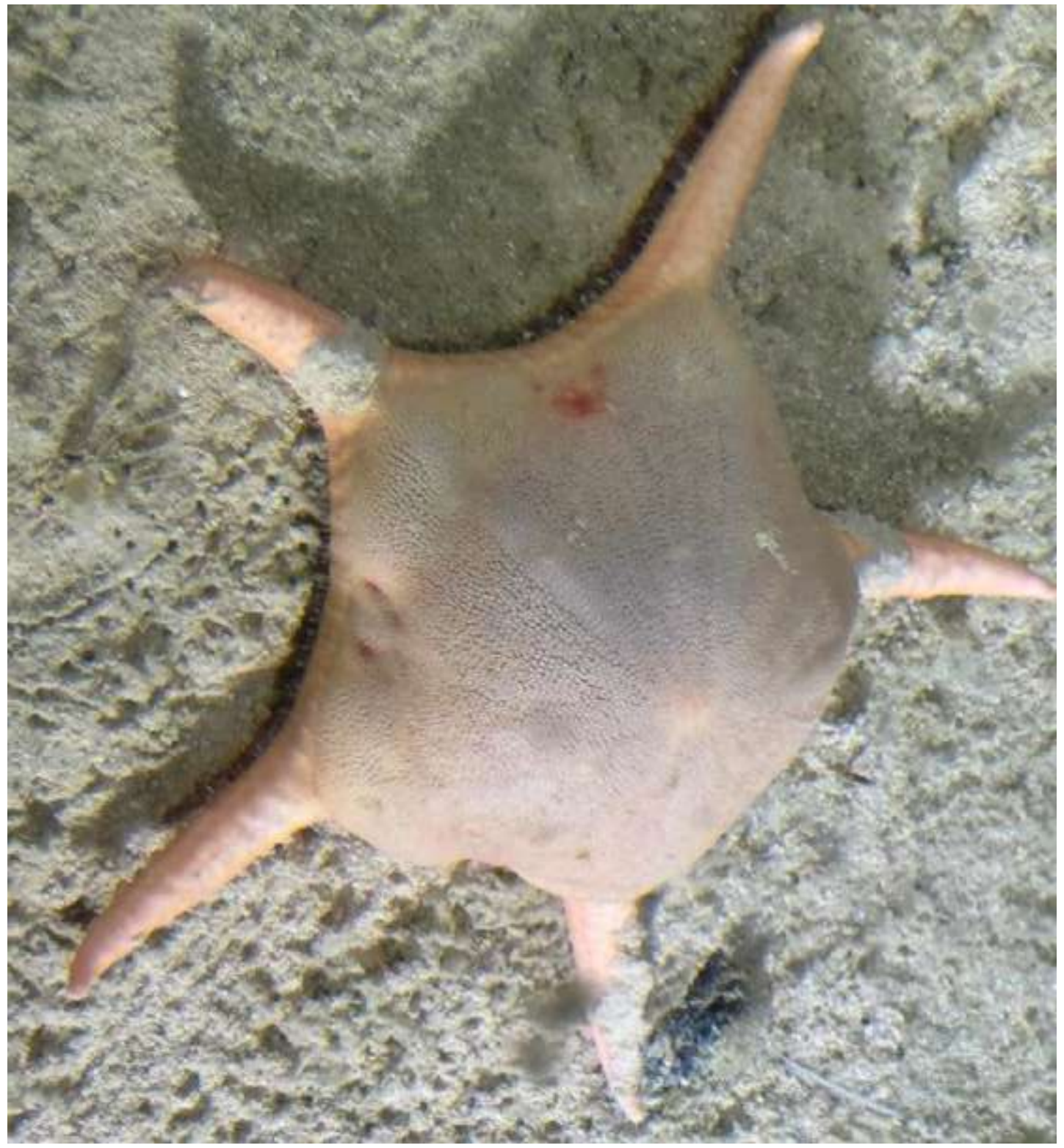

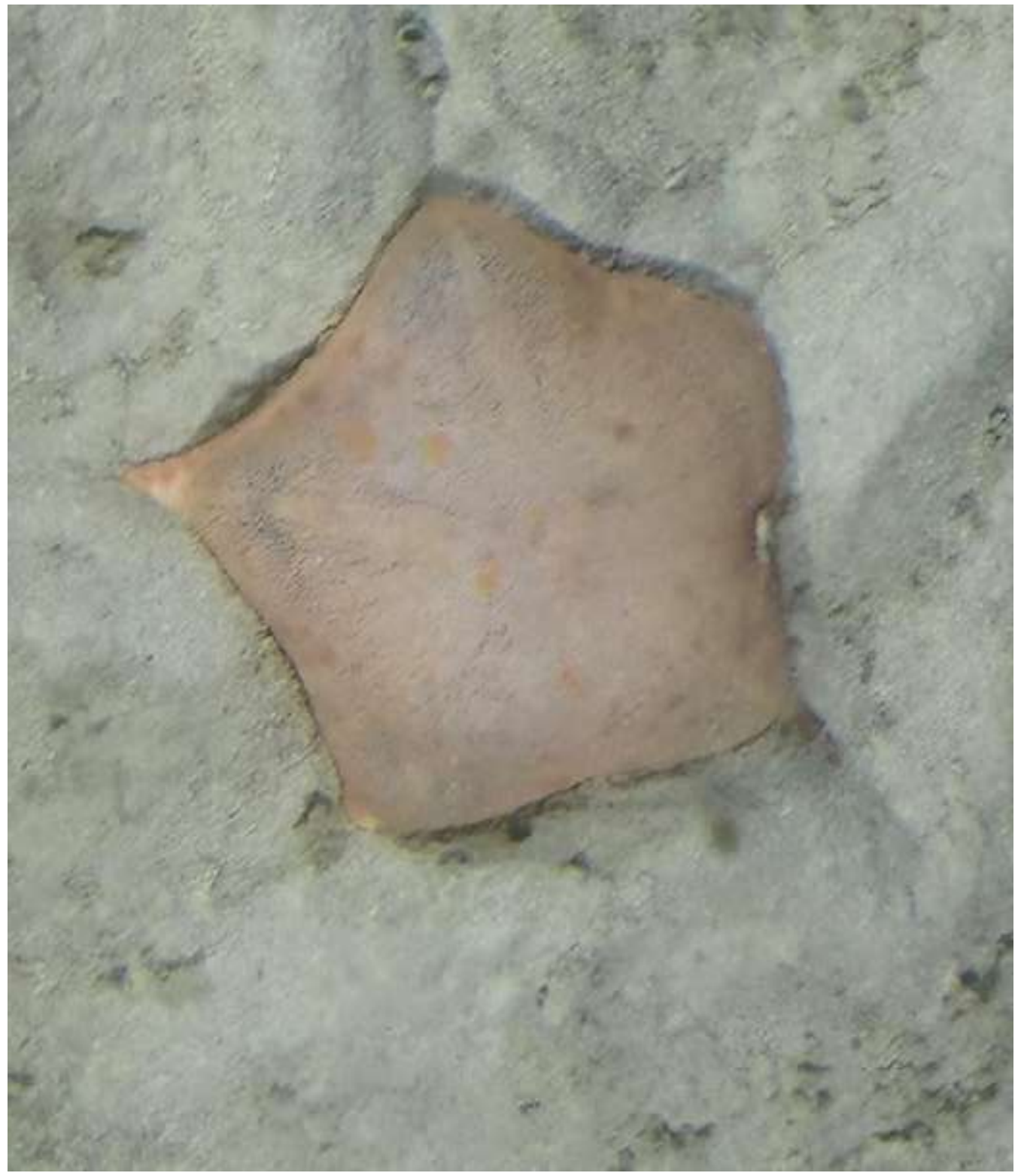

Pale pink stellate-shaped body with a dome-shaped disc covered in small pores on the aboral-side that are evenly dispersed over the disc. Limbs project from the robust disc as long thin arms.

Asteroidea; Velatida
Pterasteridae ; *Hymenaster;*
Hymenaster sp. indet. 1

NC2_BK4_5300_IMG_9644
NC4_BK1_5600_NOR_0065

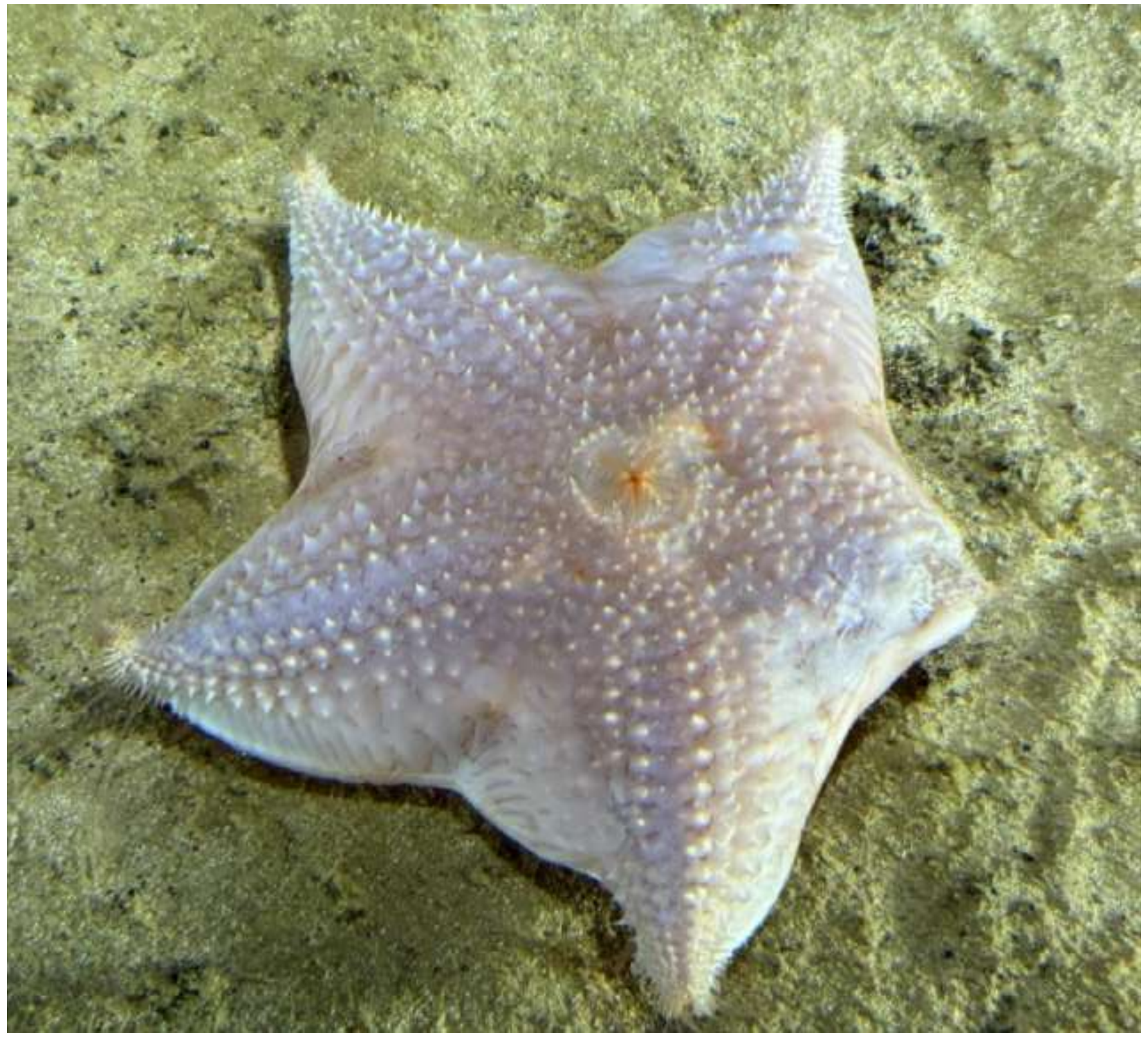

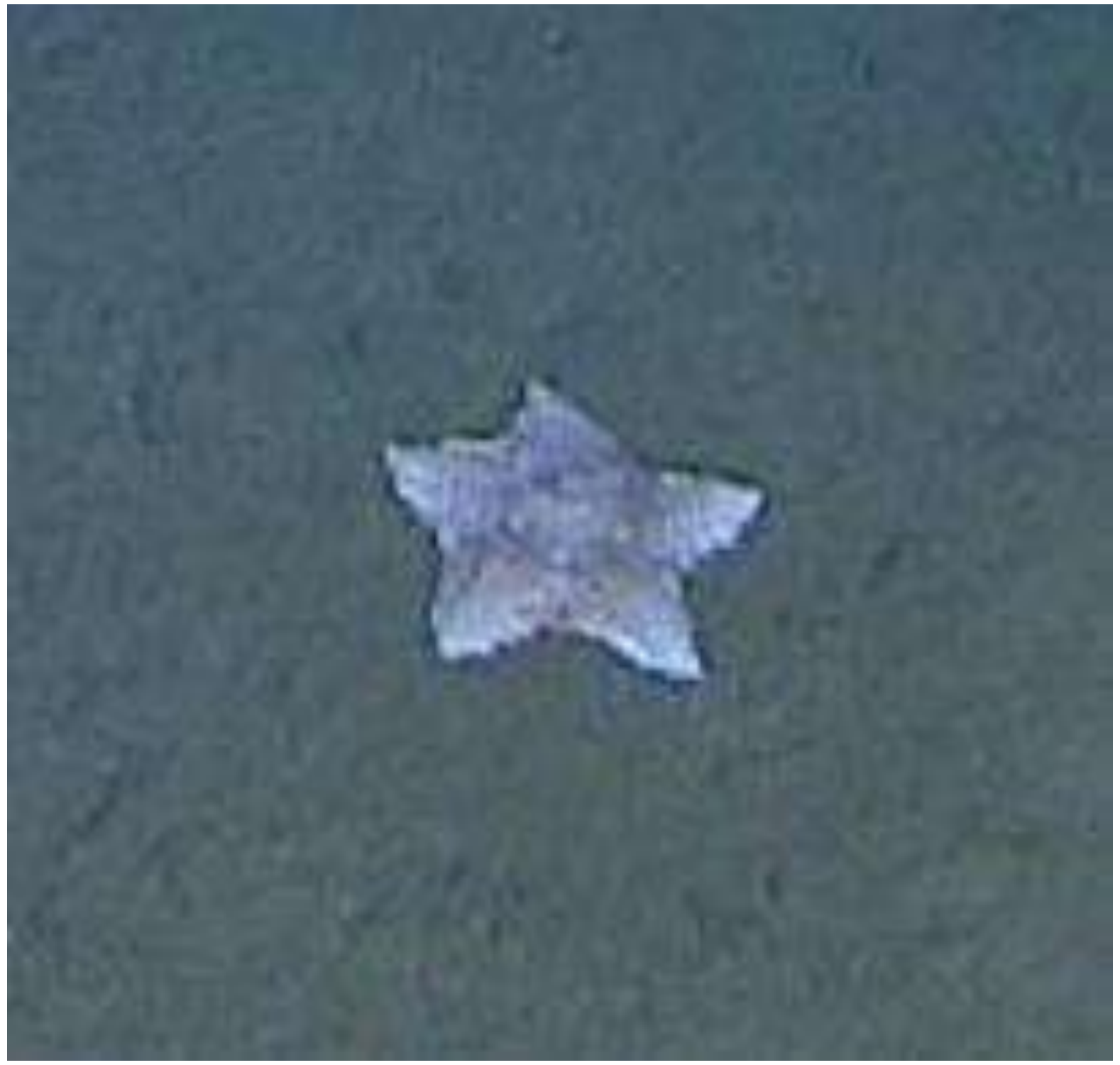

Gelatinous/ membranous pale stellate-shaped body covered in spine-like projections on the aboral-side that are evenly dispersed over the disc and along the length of the 5 limbs indicating the shape of the limbs. Spines appear thinner and more condensed on distal end of limbs. Webbing apparent between limbs.

Asteroidea; Velatida
Pterasteridae ; *Hymenaster;*
Hymenaster sp. indet. 2

NC1_BK3_8000_IMG_6315
NC1_BK3_8000_IMG_6630
NC3_BK4_8000_IMG_3779
NC3_BK1_8000_IMG_0646
NC3_BK1_8000_IMG_1072

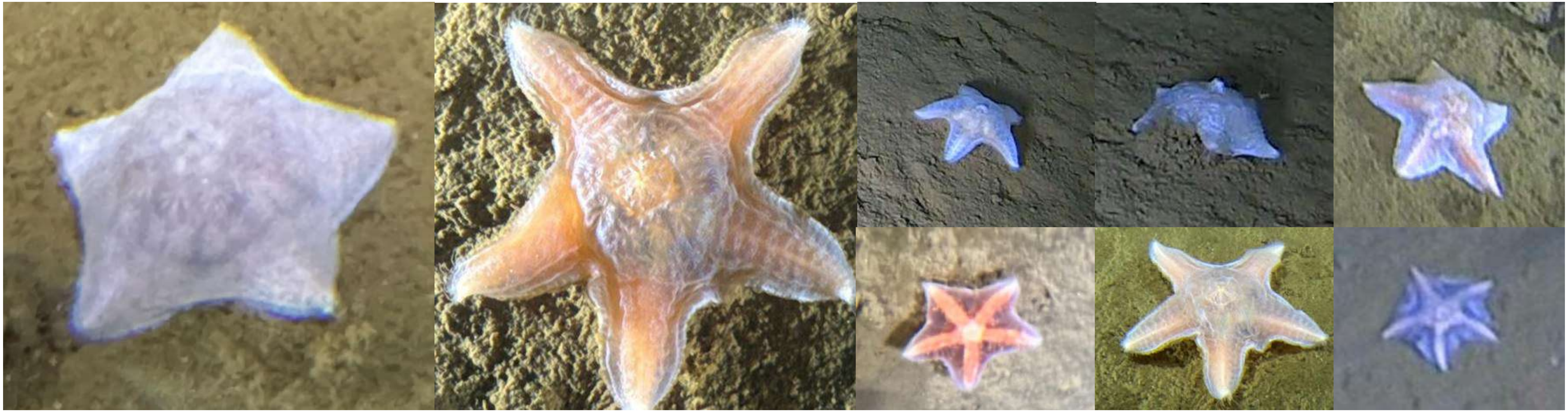

Gelatinous/ membranous pale-purple stellate-shaped body covered in small, sometimes with vaguely star shaped perforations on the aboral-side that are evenly dispersed over the disc. Some individuals present rough texture on the aboral-side that are evenly dispersed over the disc and along the length of the 5 limbs indicating the shape of the limbs. Webbing apparent between limbs. Ambulacral grooves are a deep red-orange colouration that becomes pale yellow on the distal portions of the limbs that matches the colouration of the stomach. Ventral side of webbing is a dark brownish colour.

Asteroidea; Velatida
Pterasteridae ; *Hymenaster;*
Hymenaster sp. indet. 3

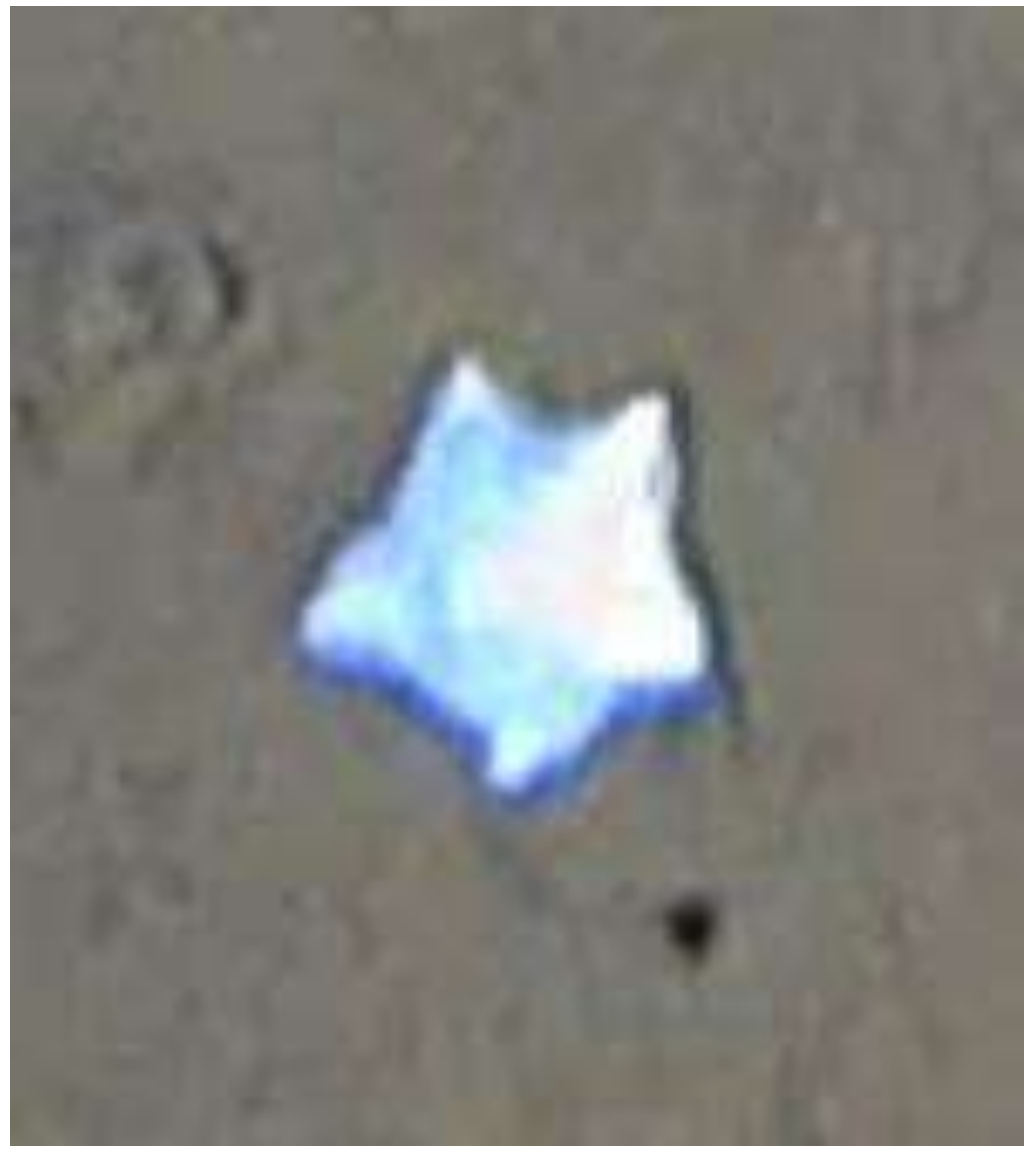

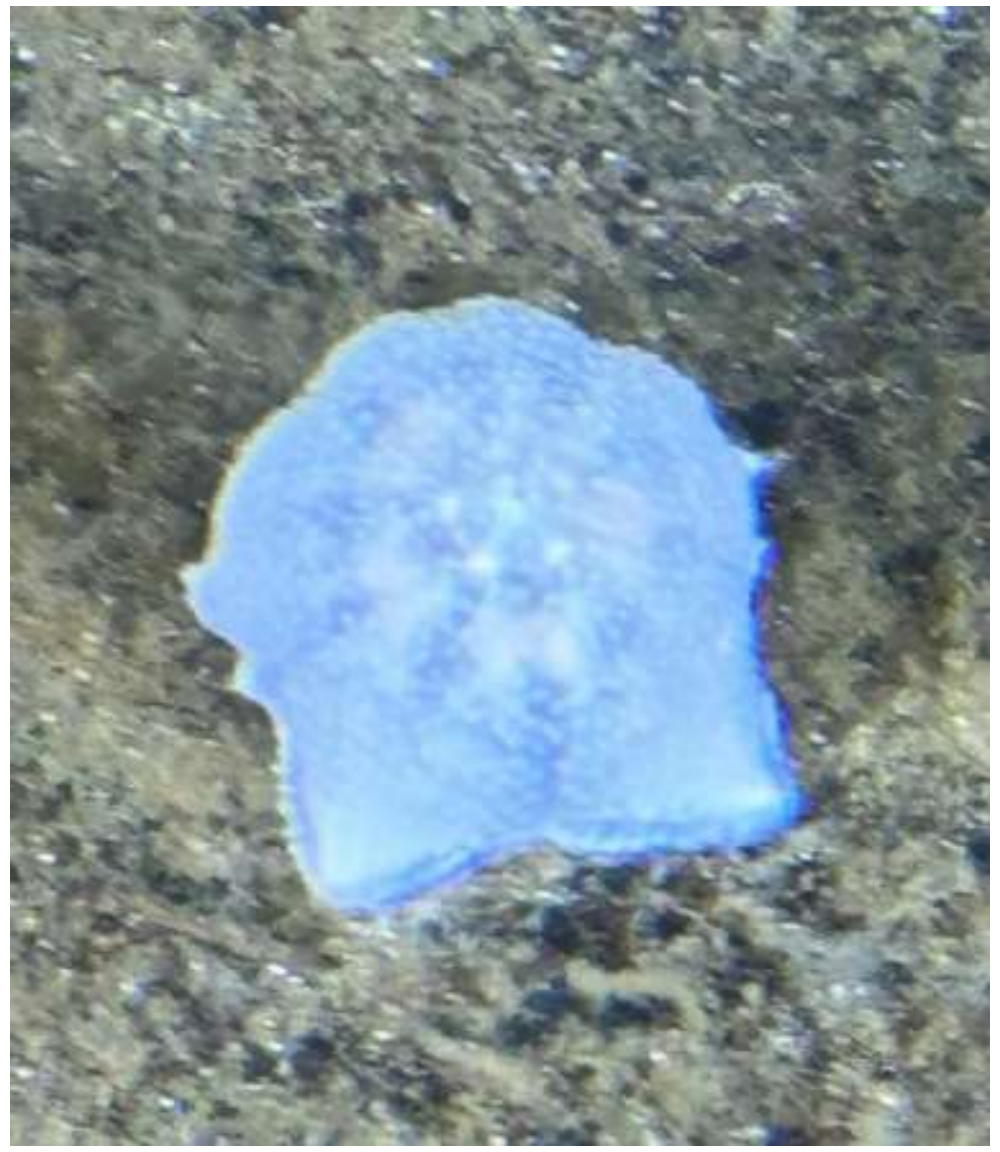

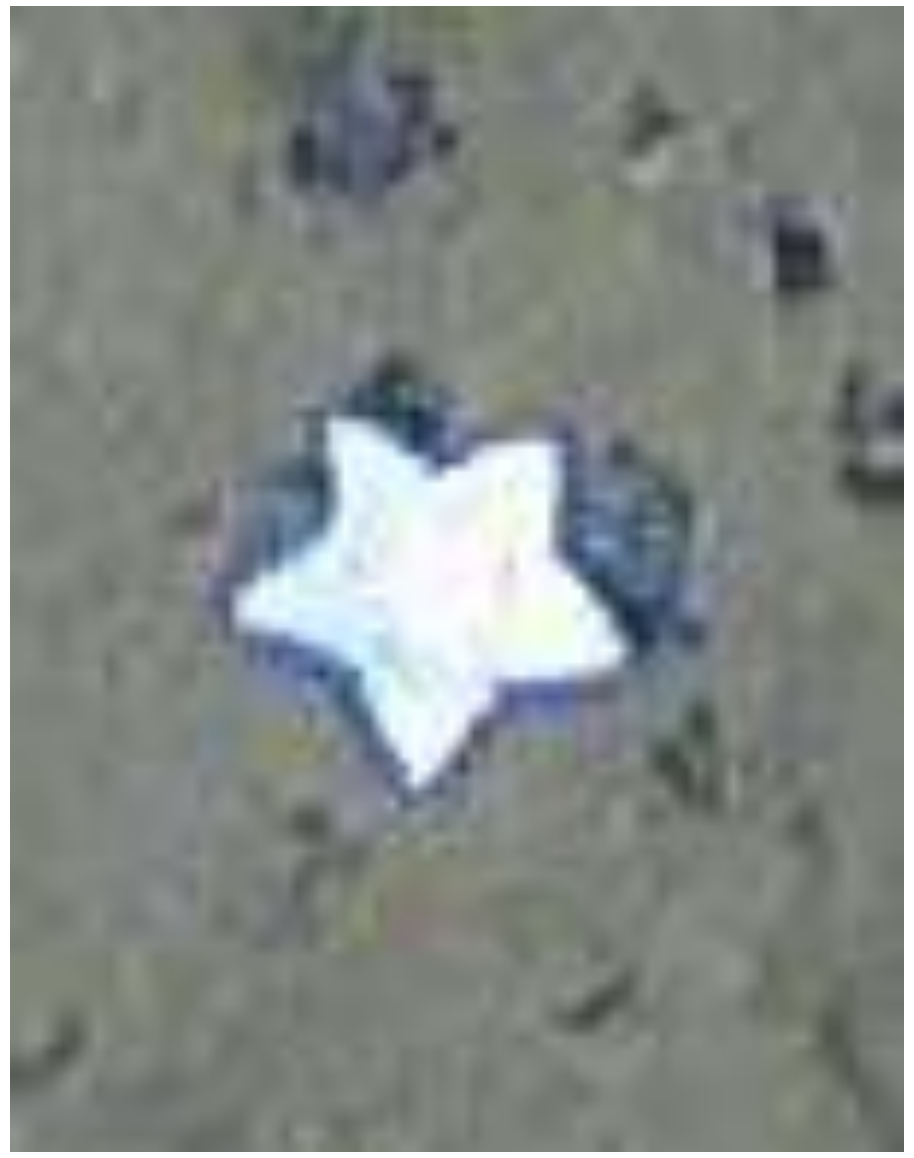

Very small white almost translucent body. Observed 'curled up' incl on soft sediemnt

Asteroidea; Valvatida; Solasteridae ; *Lophaster; Lophaster* sp. indet.1

NC2_BK1_3200_IMG_7419
NC2_BK1_3200_IMG_7415
NC2_BK1_3200_IMG_7577

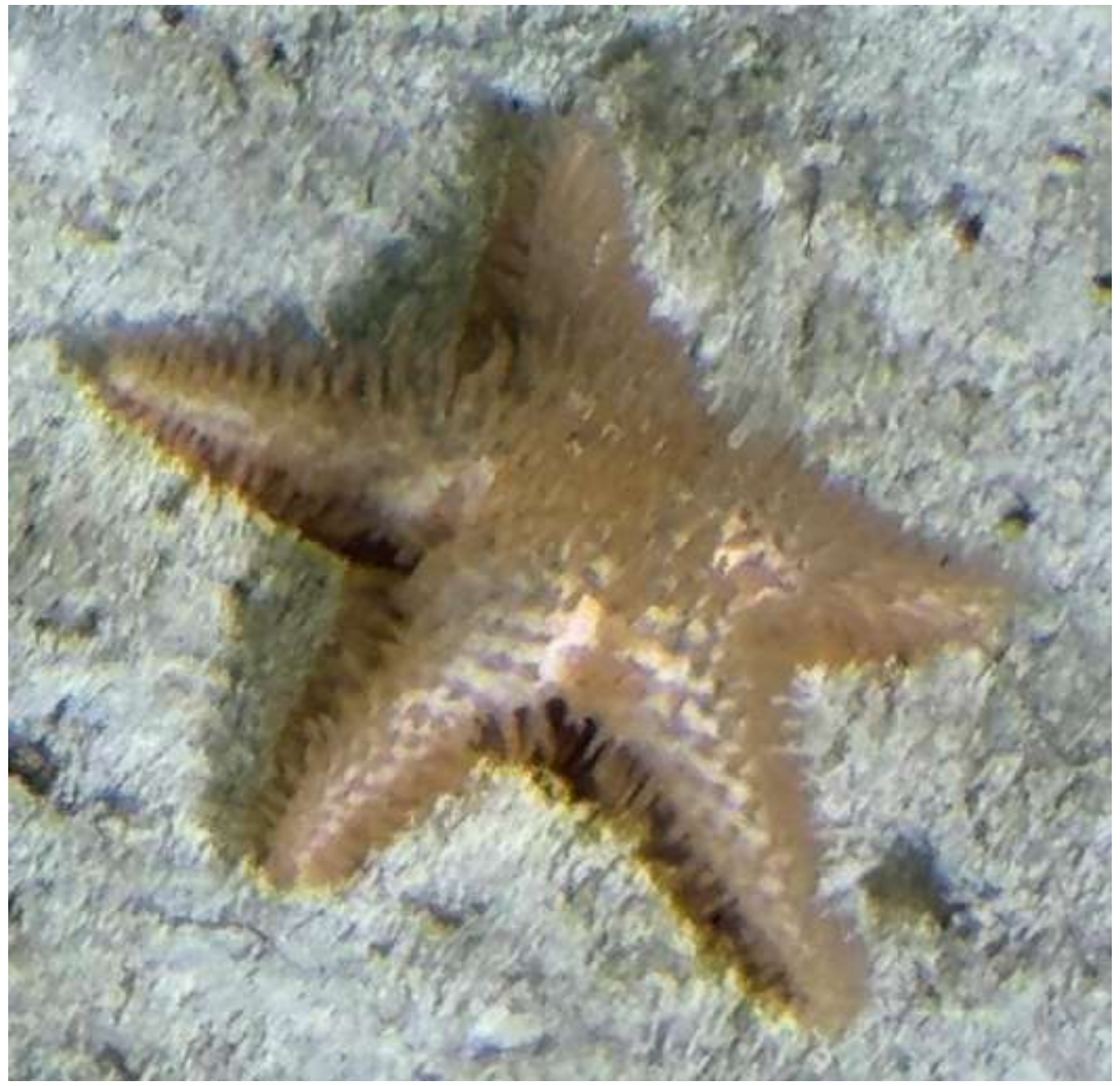
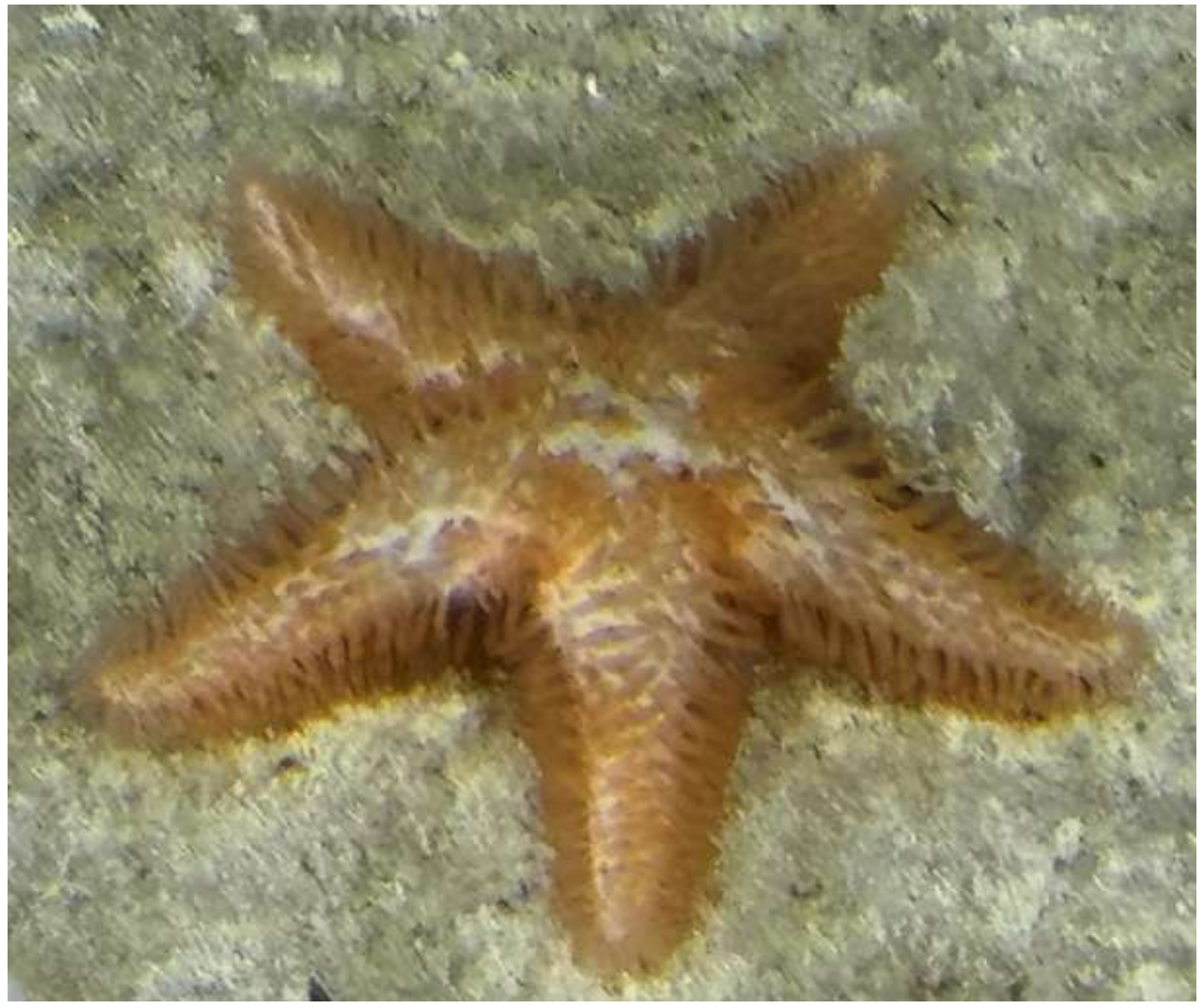
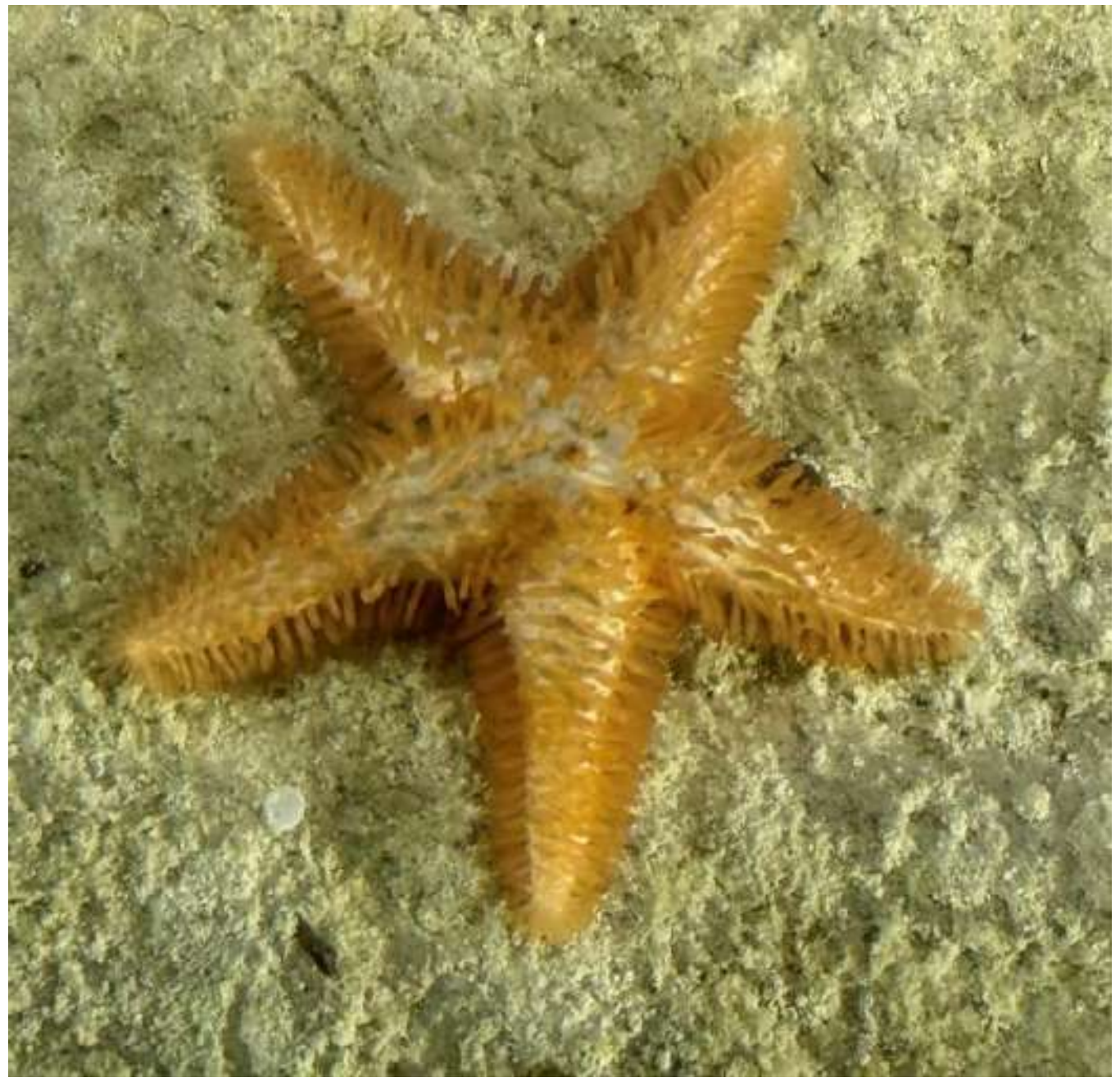

Pale stellate-shaped body covered in thick, golden-brown spines on the aboral-side of the disc and arms.

Asteroidea; Valvatida; Solasteridae ; Solasteridae gen. indet. 1

NC4_BK4_4700_NOR_0086

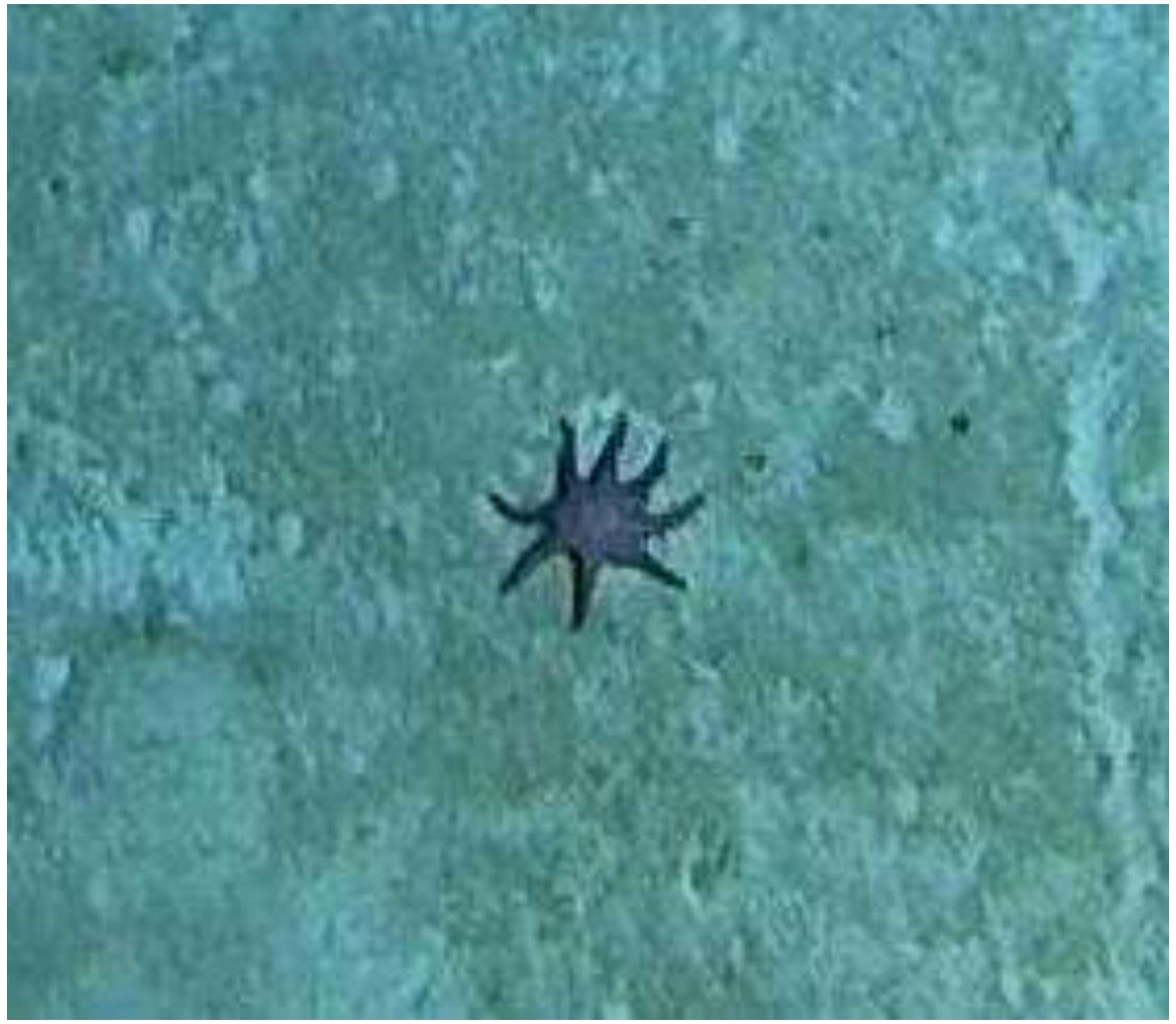

Dark, stellate-shaped body with wide disc and 8 arms.

Ophiuroidea; Ophiacanthida Ophiacanthidae ; Ophioplinthaca; Ophioplinthaca sp. indet. 1

NC2_OM2_3400_IMG_6832
NC2_BK1_3200_IMG_7557
NC2_BK1_3100_IMG_7389

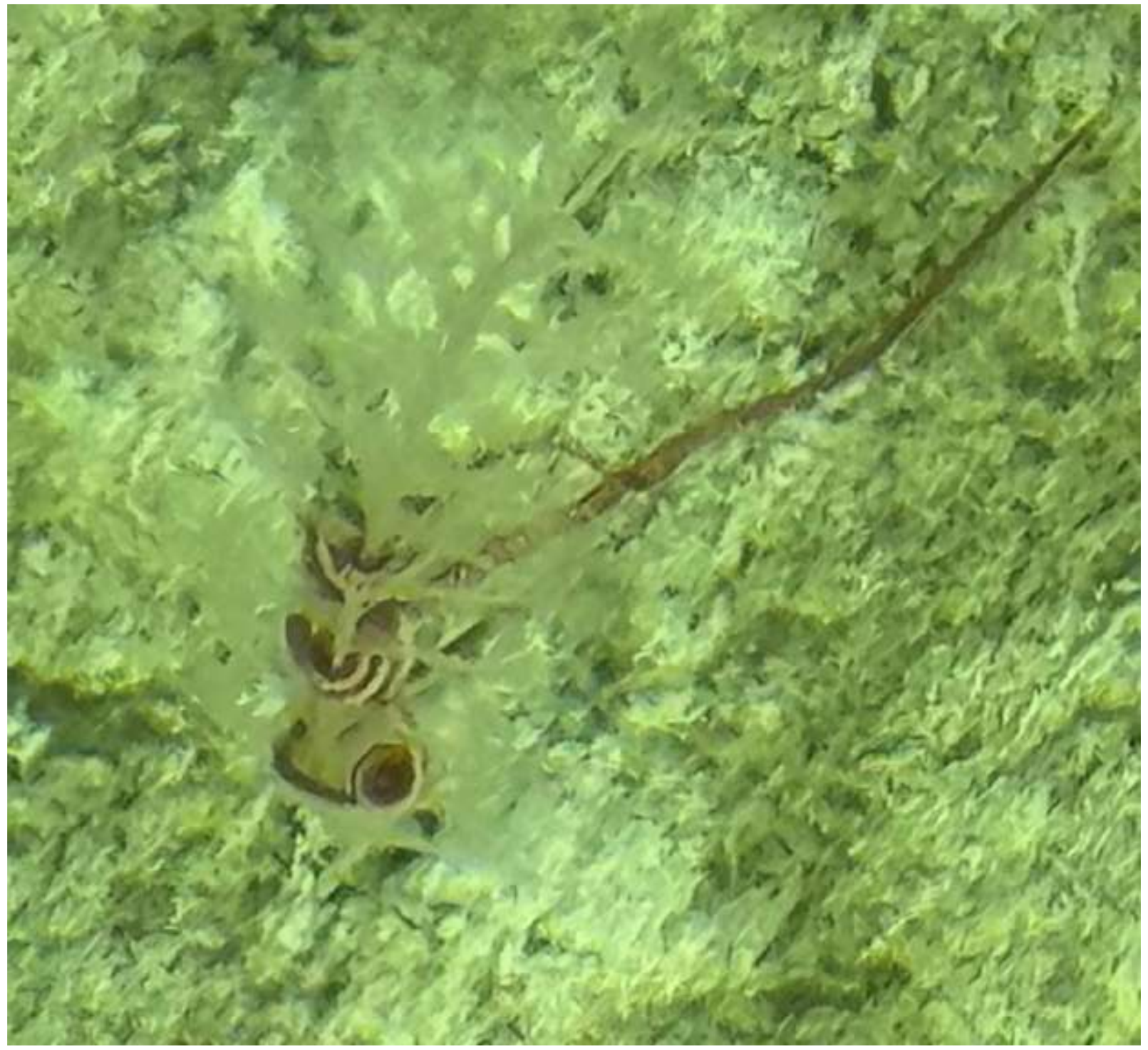
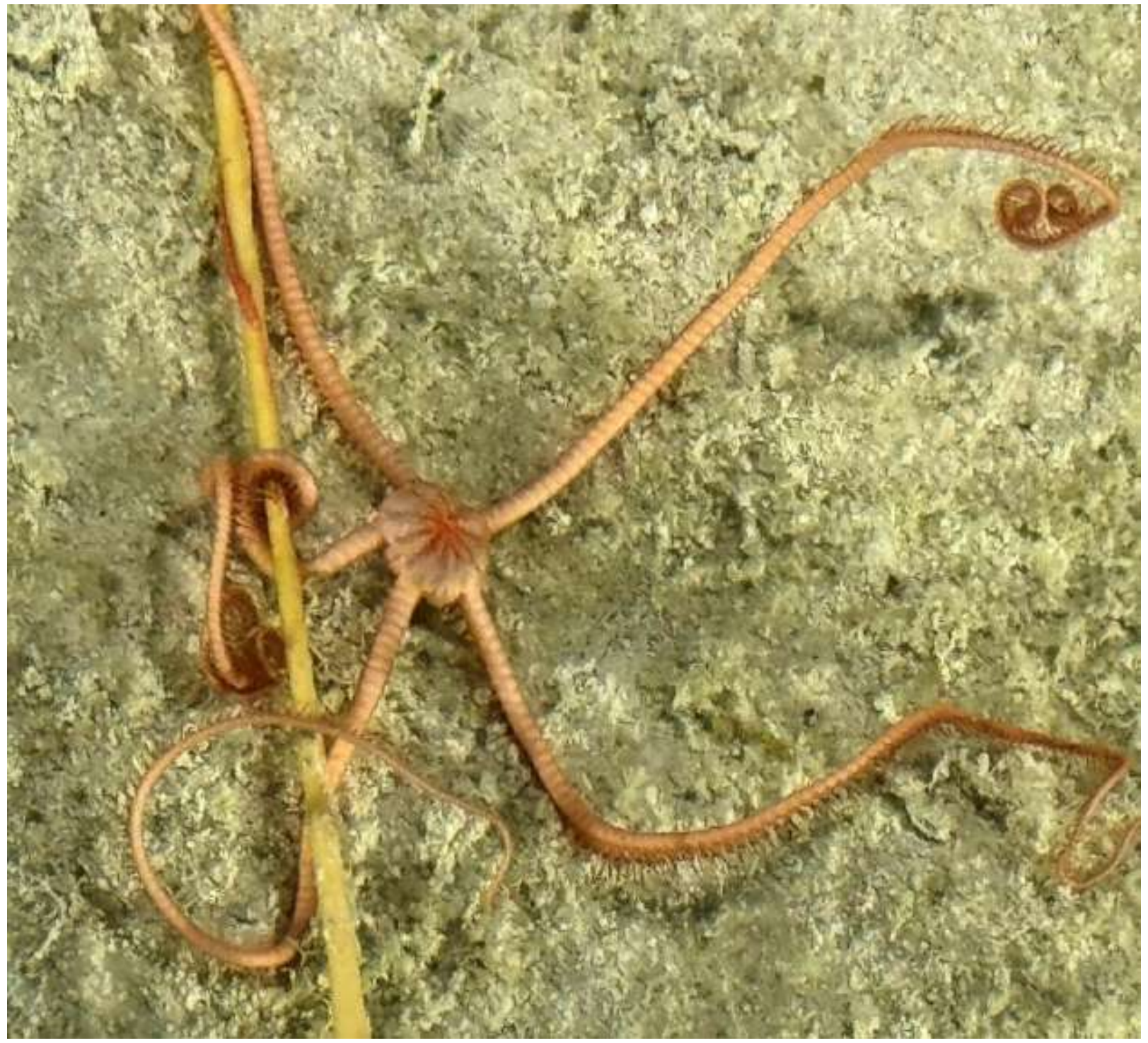
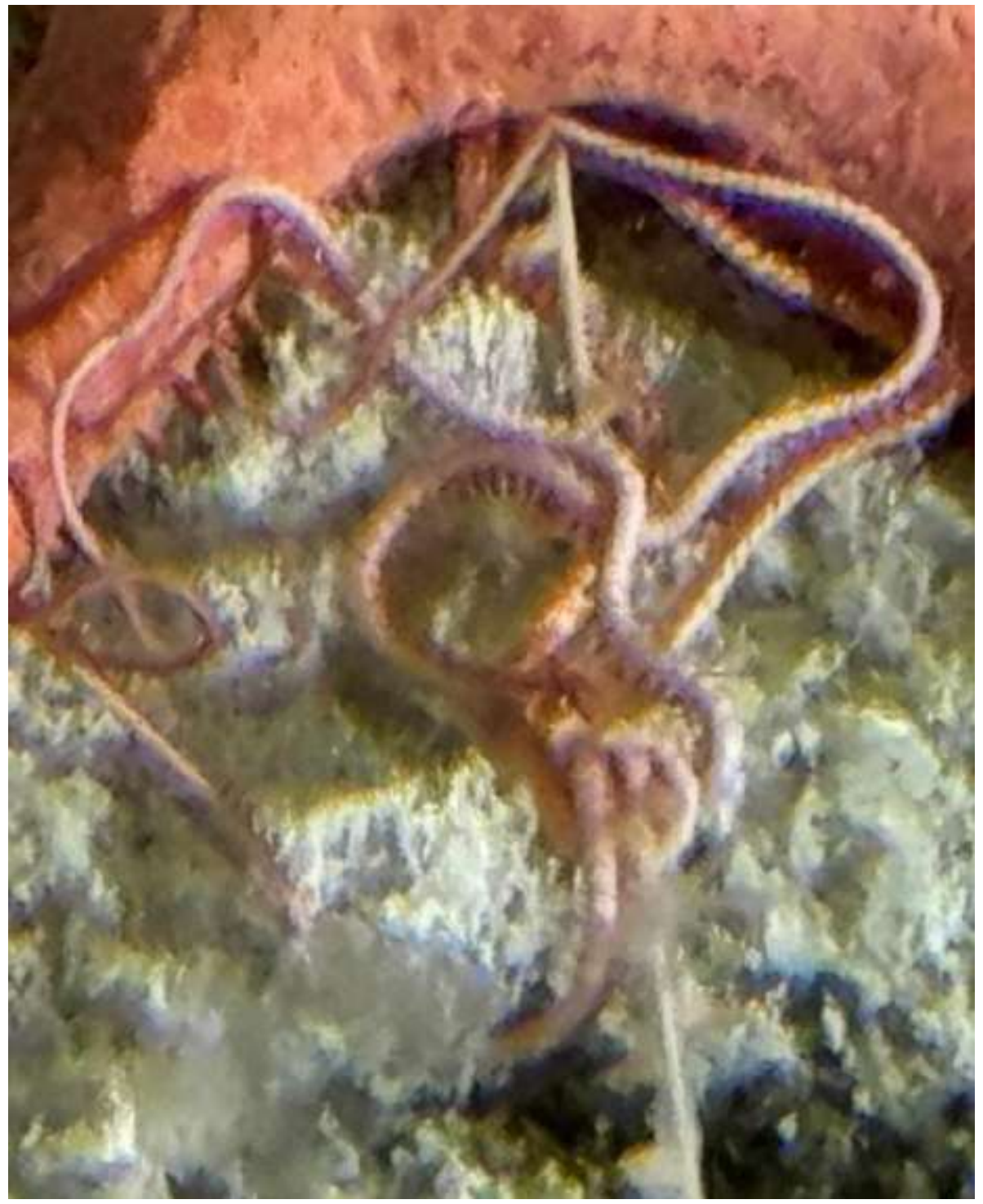

Body is a greyish pink with deep incisions present in the disc. Distally enlarged wedge-shaped lobes are present on disc. Long rough spines present on arms that are also long and thin.

Ophiuroidea; Ophiuroidea ord. inc.1

NC2_CR7_5100_NOR_0122

NC2_CR7_5100_NOR_0122

NC4_MA5_6000

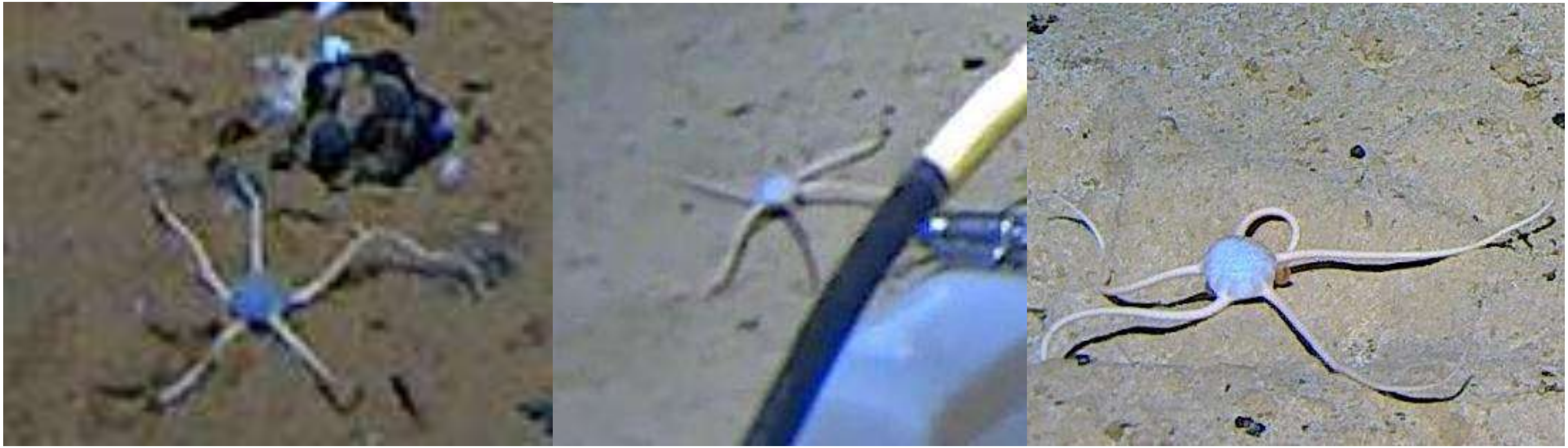

Disc is large and rounded, purple coloured and raised, with 5 thin arms present.

Ophiuroidea; Ophiuroidea ord. inc. *2*

NC2_BK5_4600_IMG_9927

NC2_BK5_4600_NOR_0081

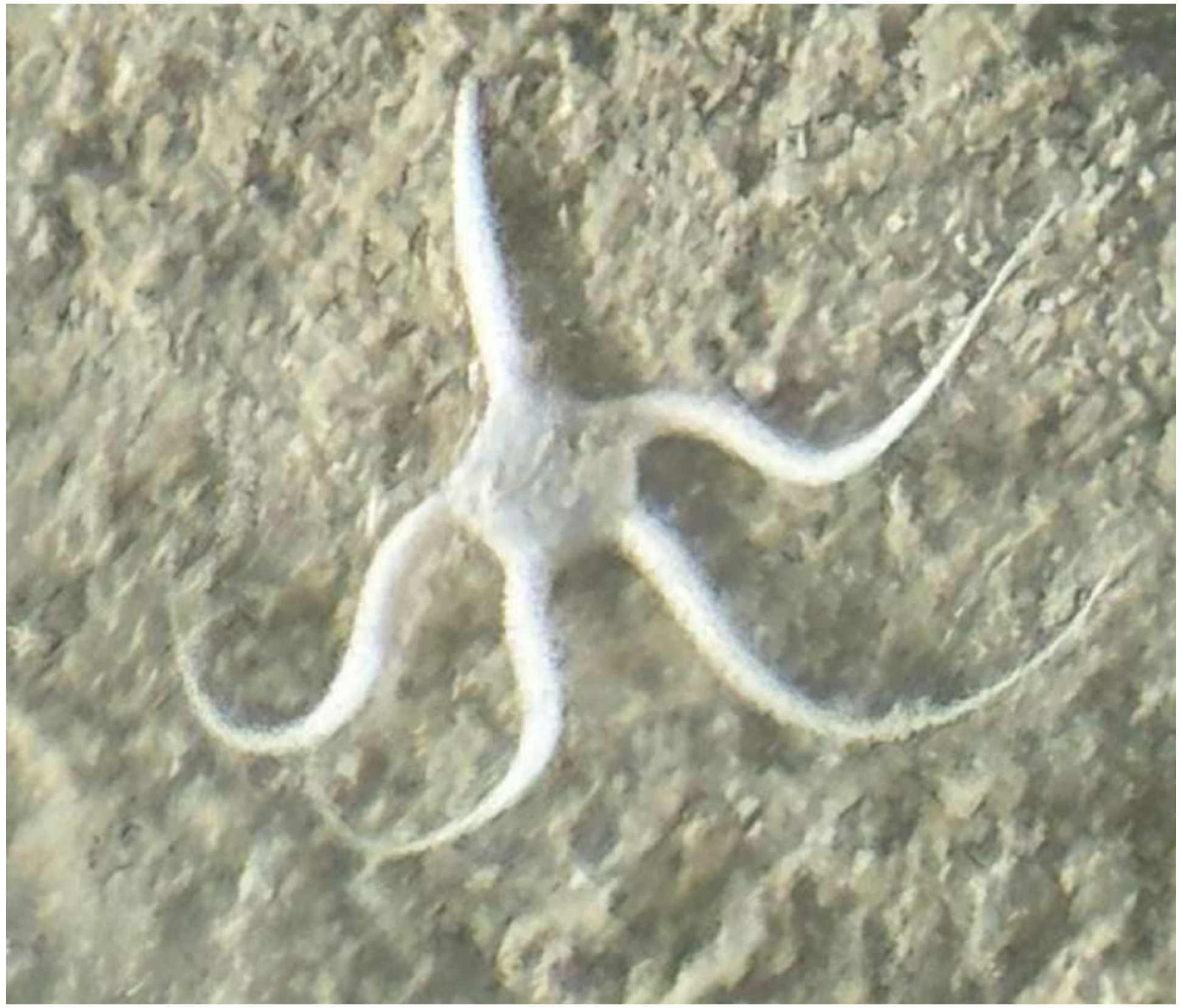

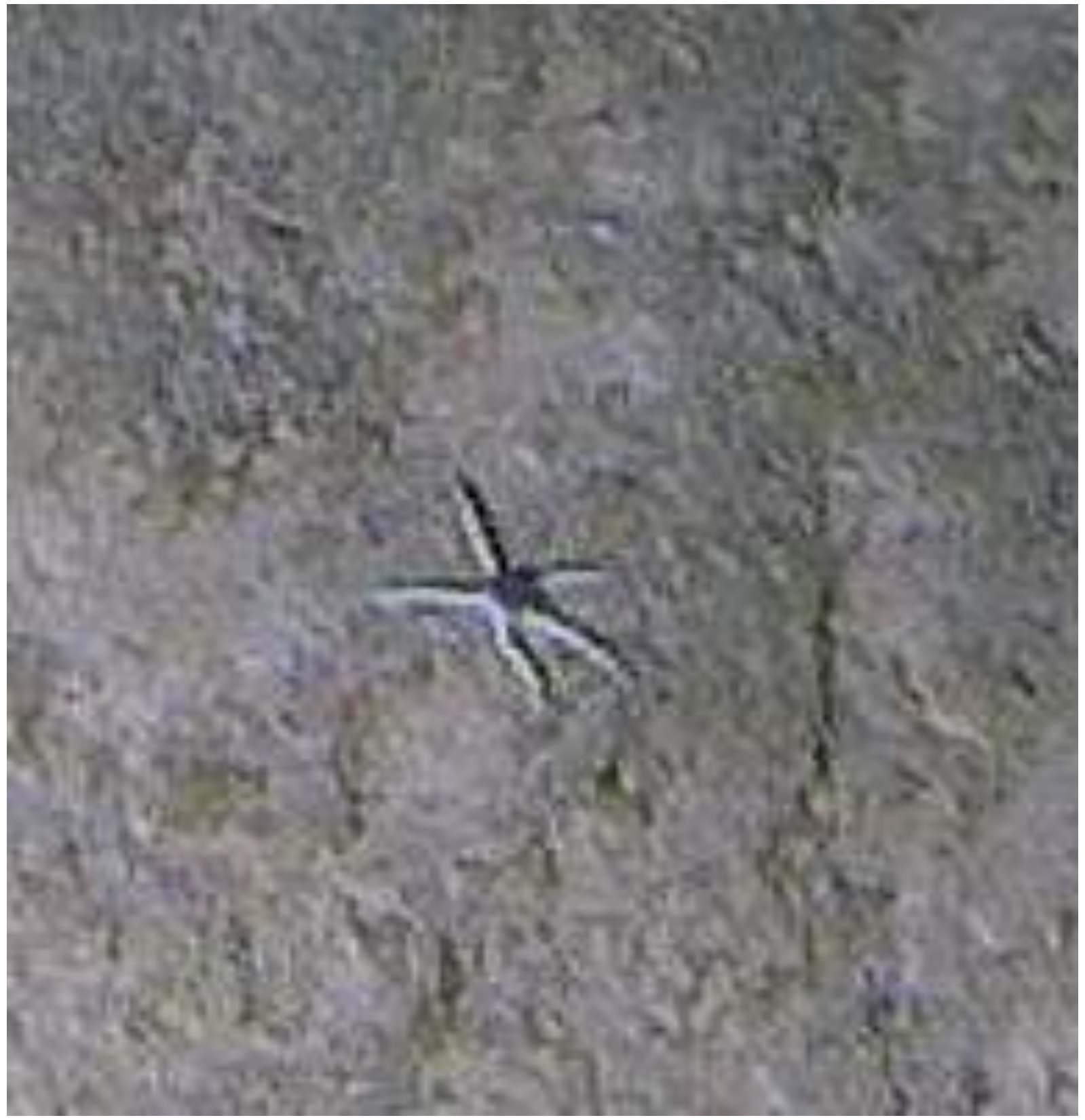

Disc is small and pentagonal with 5 relatively thick arms present.

NC2_BK2_3800_IMG_8341

Ophiuroidea; Ophiuroidea ord. inc. *3*

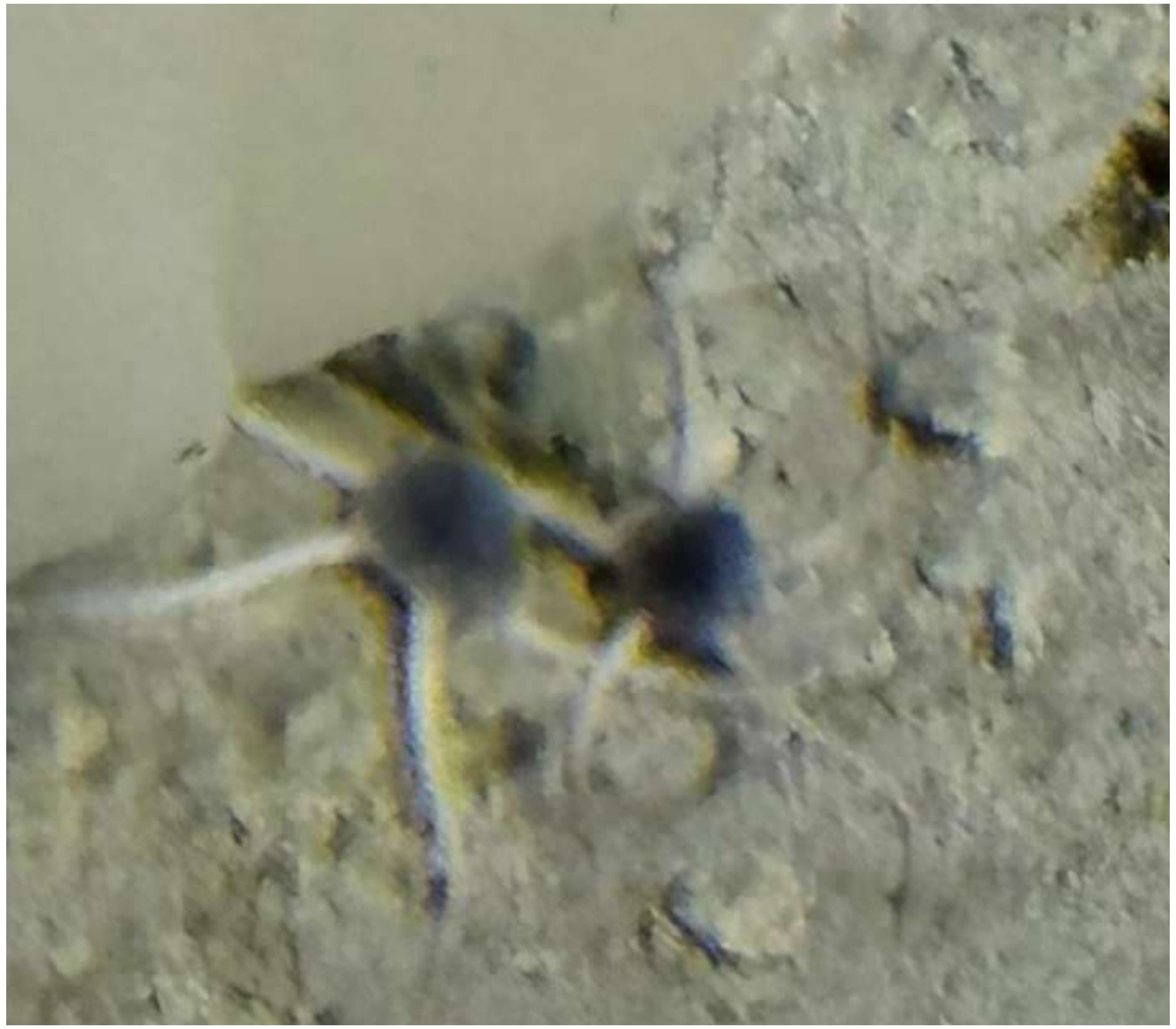

Disc is small, dark and pentagonal with 5 relatively thick arms present. One arm appears to be shorter than the others.

Ophiuroidea; Ophiuroidea ord. inc. 4

NC2_BK5_4600_IMG_0491

NC2_CR1_3200_NOR_0139

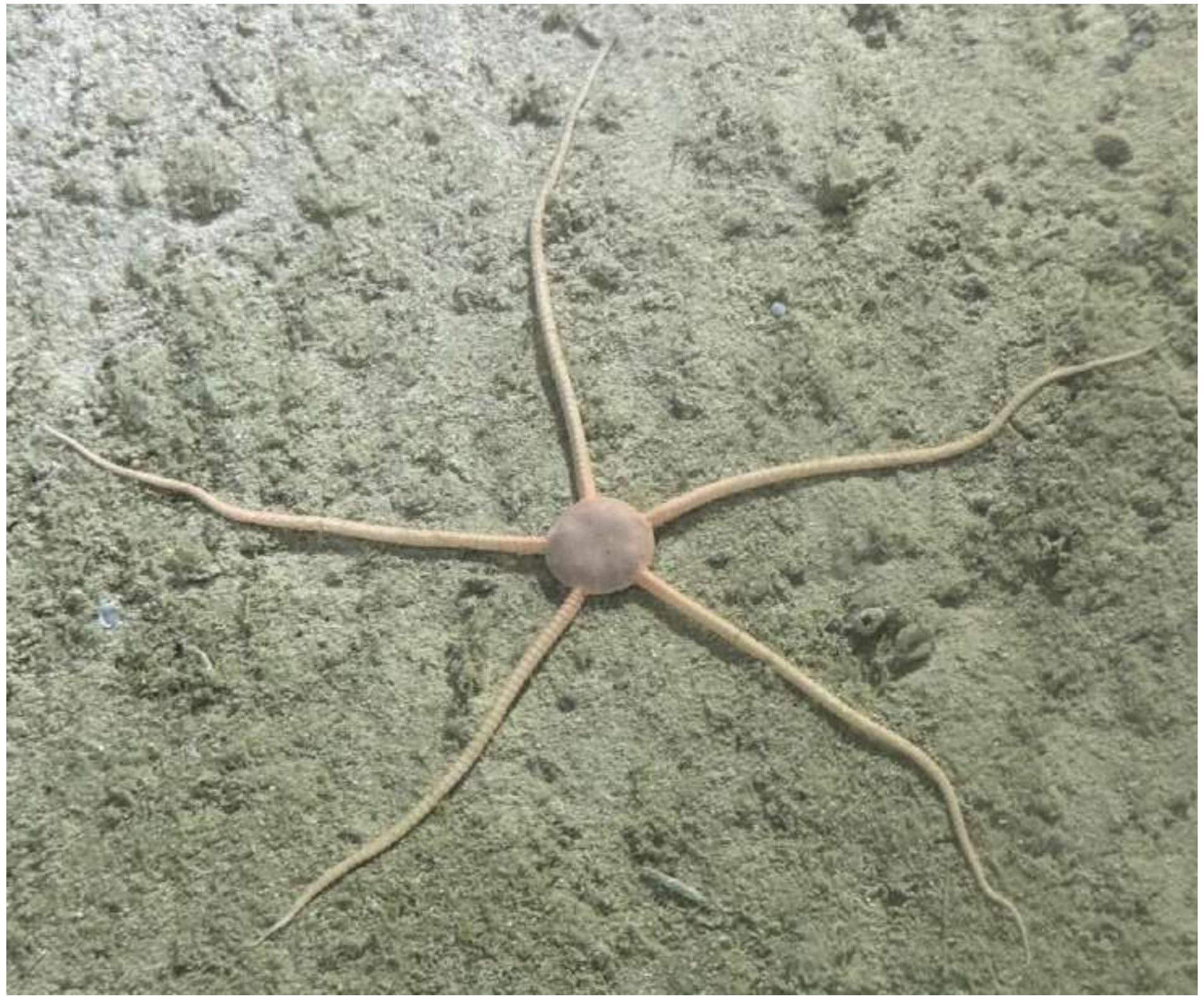

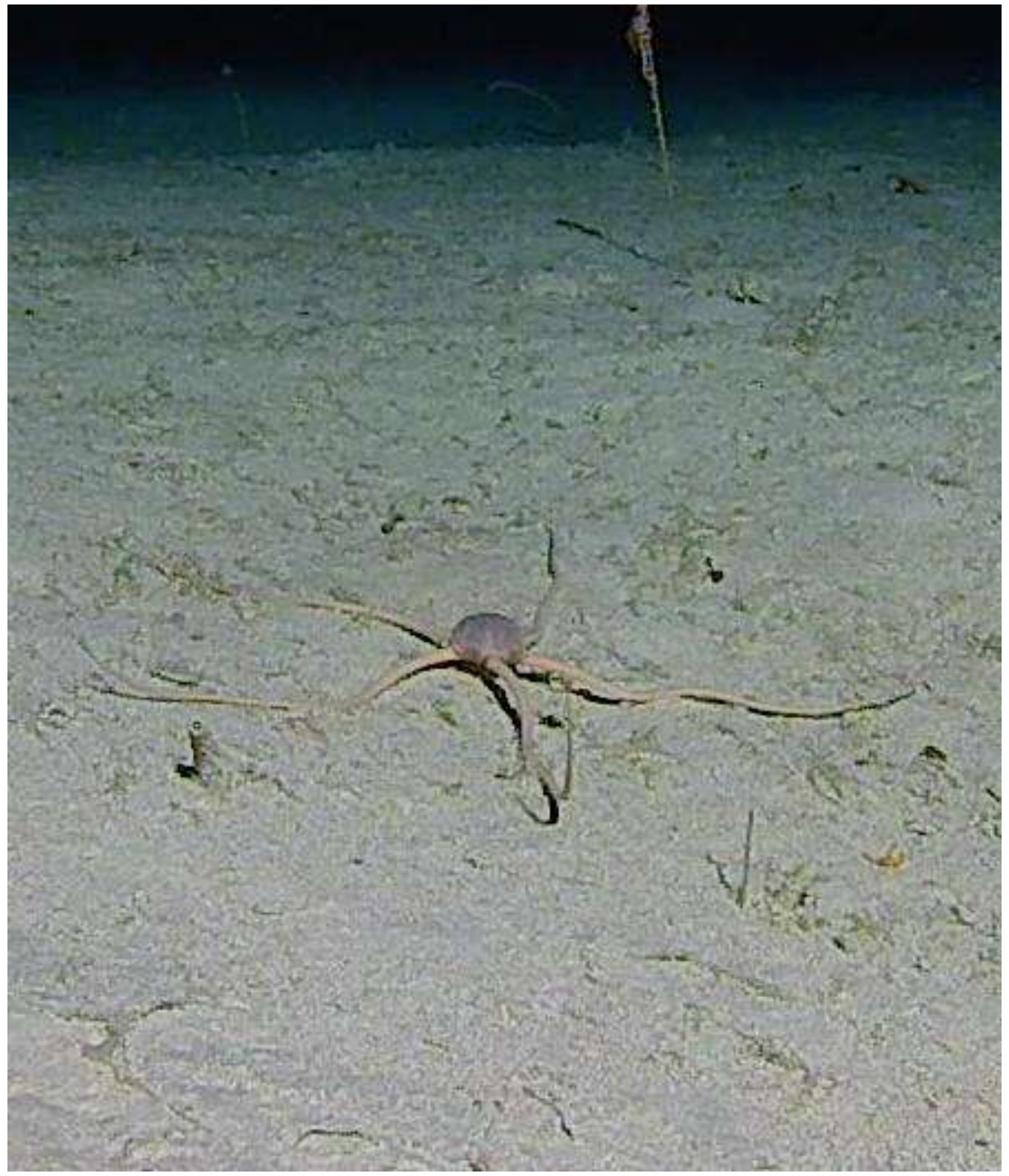

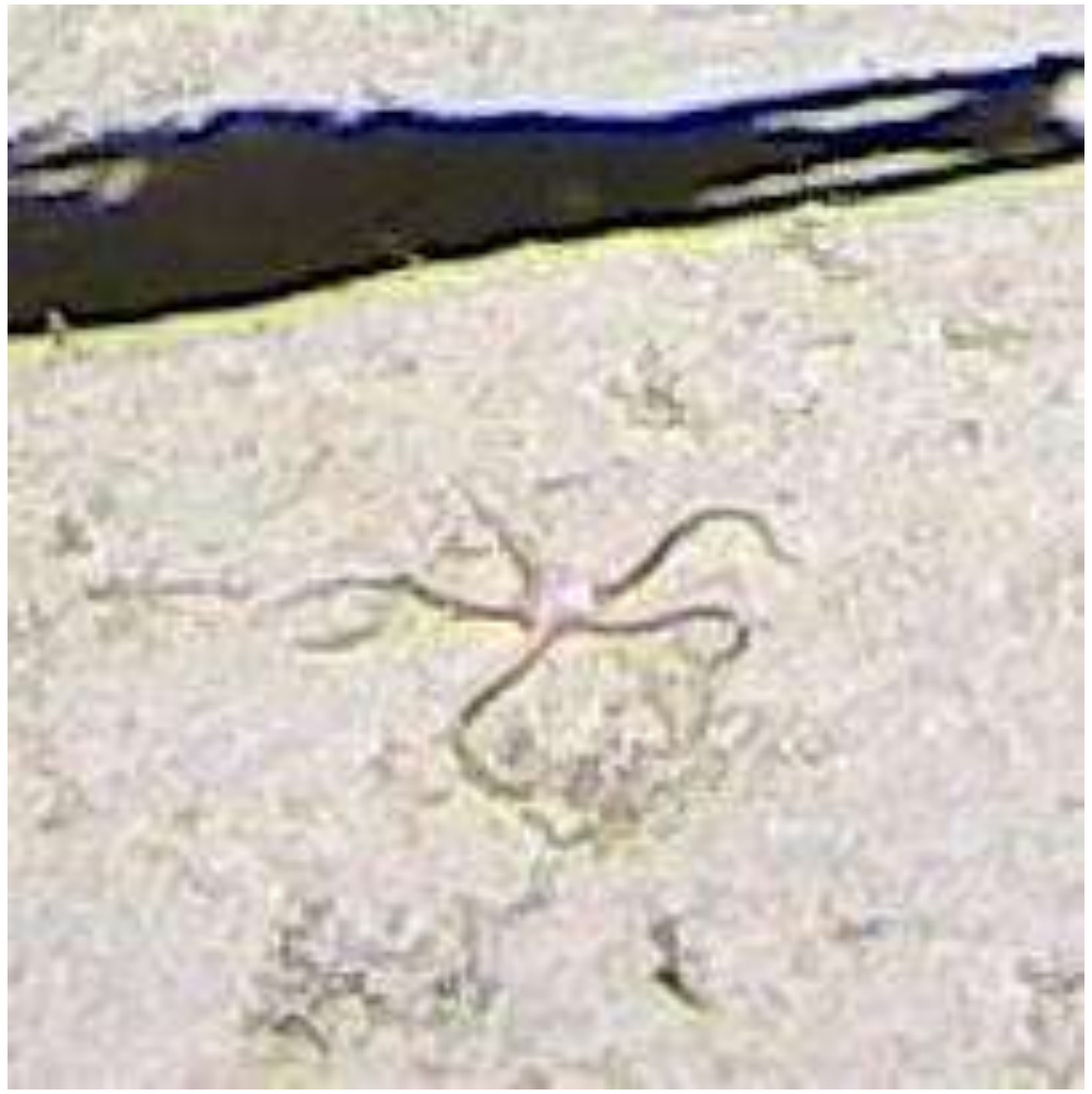

Disc is rounded and smooth with slight dome-like shape. Darker pink colour. Arms are long and thin with segmented plates along the limbs.

NC1_BK2_6700_IMG_4950

Ophiuroidea; Ophiuroidea ord. inc. 5

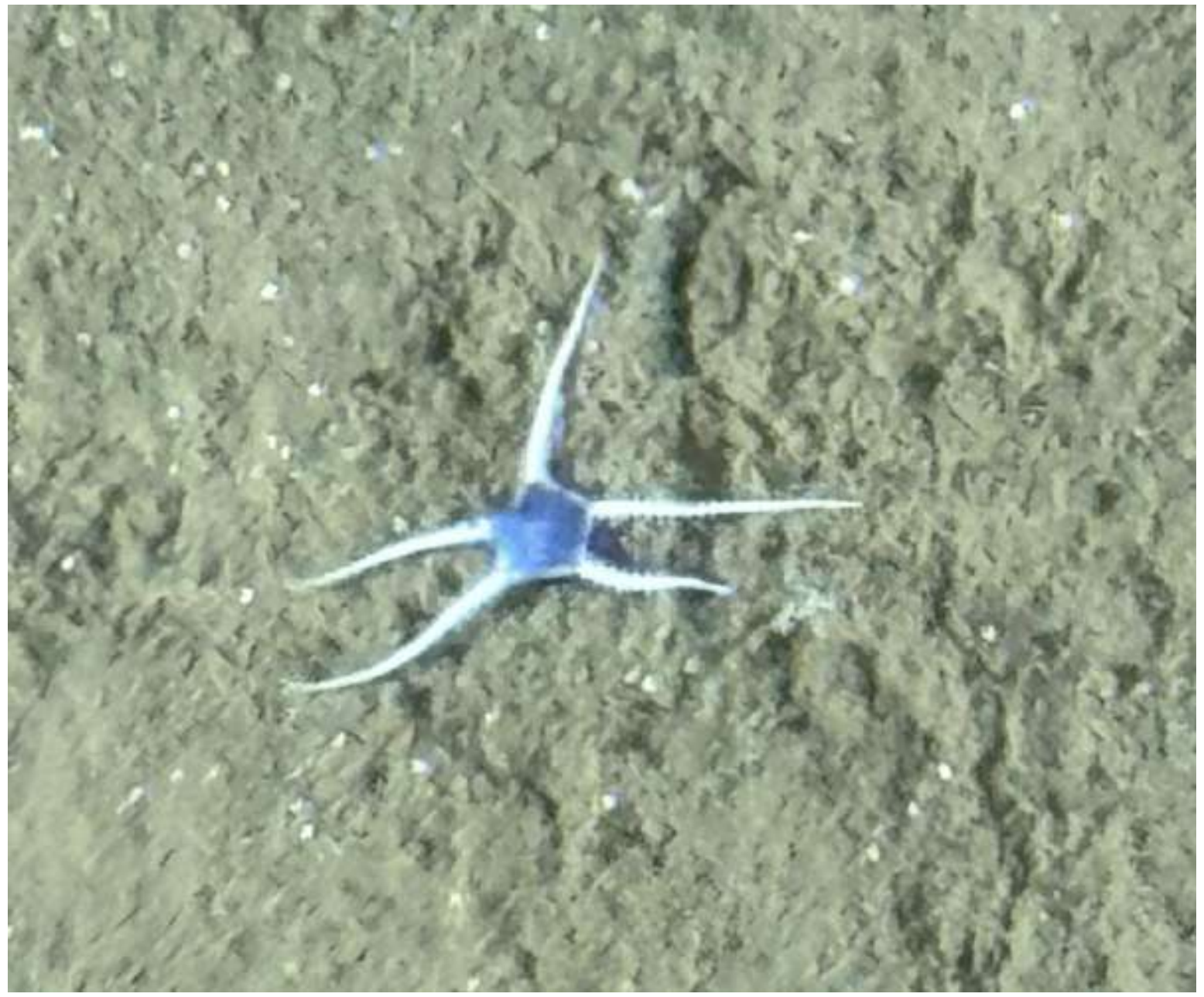

Arms are white with a greyish blue disc in situ. Disc is flattened and somewhat pentagonal

Ophiuroidea; Ophiuroidea ord. inc. 6

NC1_BK1_6200_NOR_0057
NC1_ BK1_6200 _IMG_4280
NC2_BK3_7400_IMG_9321

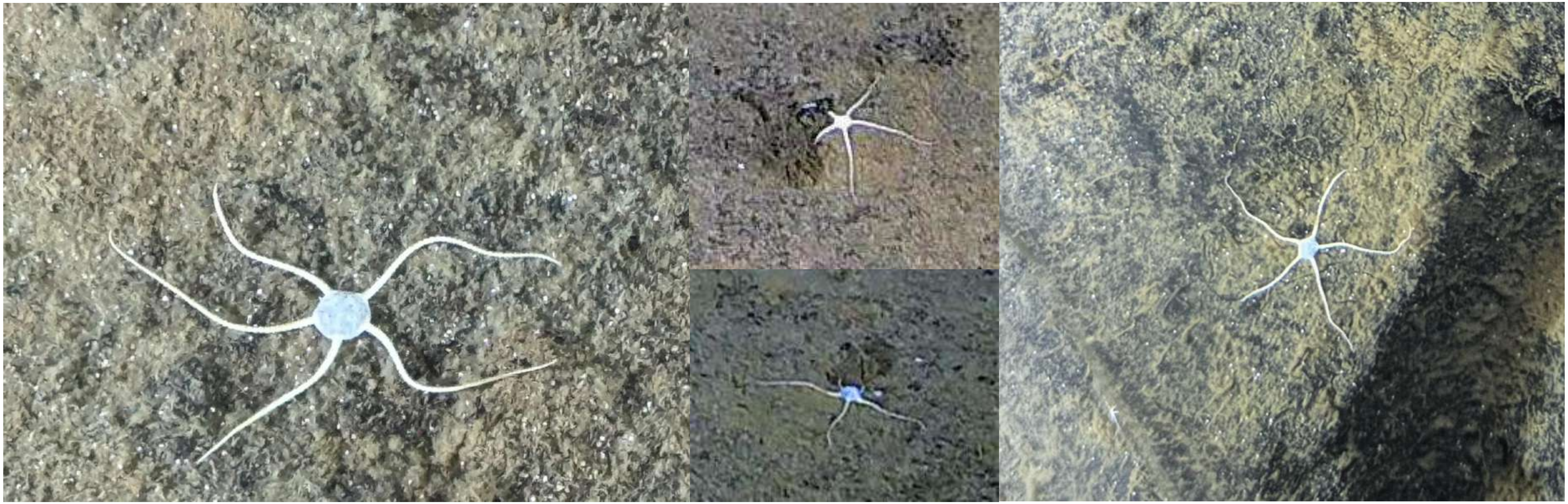

White; purplish/darker disc, slightly raised compared to arms.

NC2_BK2_3800_IMG_8079

Ophiuroidea;
Ophiuroidea ord. inc. 7

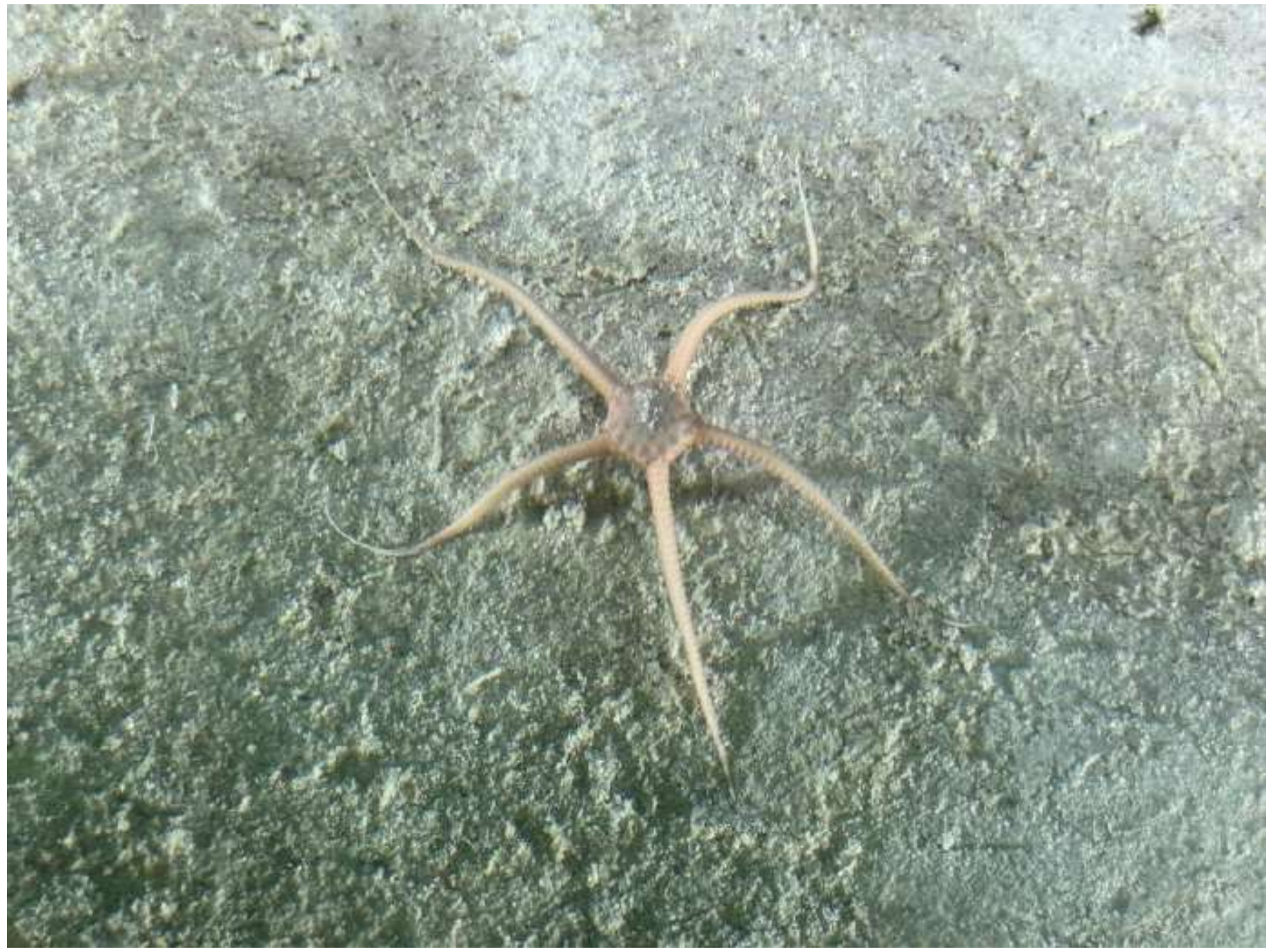

Ophiuroidea; Ophiuroidea ord. inc. 8

NC4_BK3_6900
NC3_BK4_8000
NC3_BK1_8000

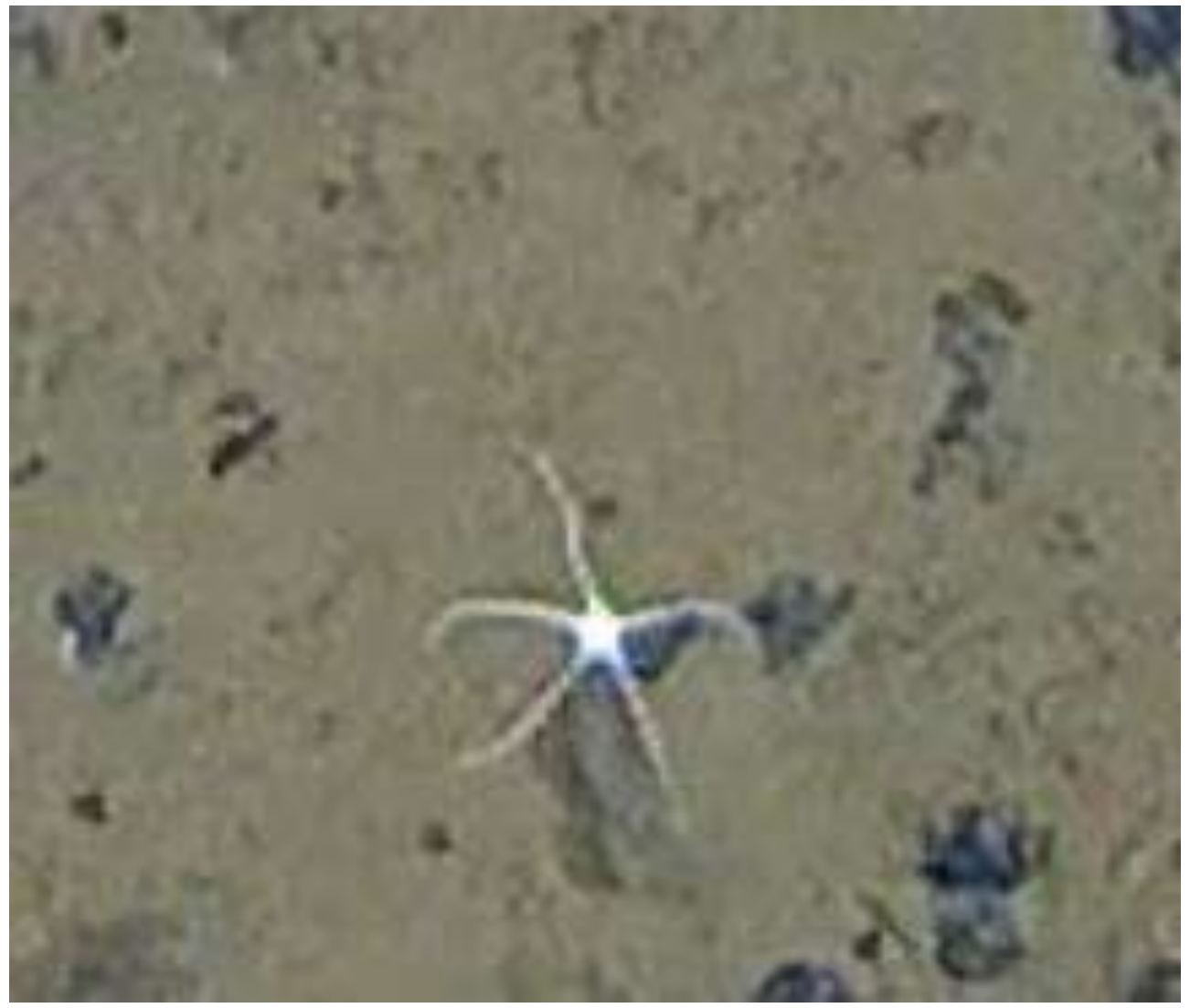

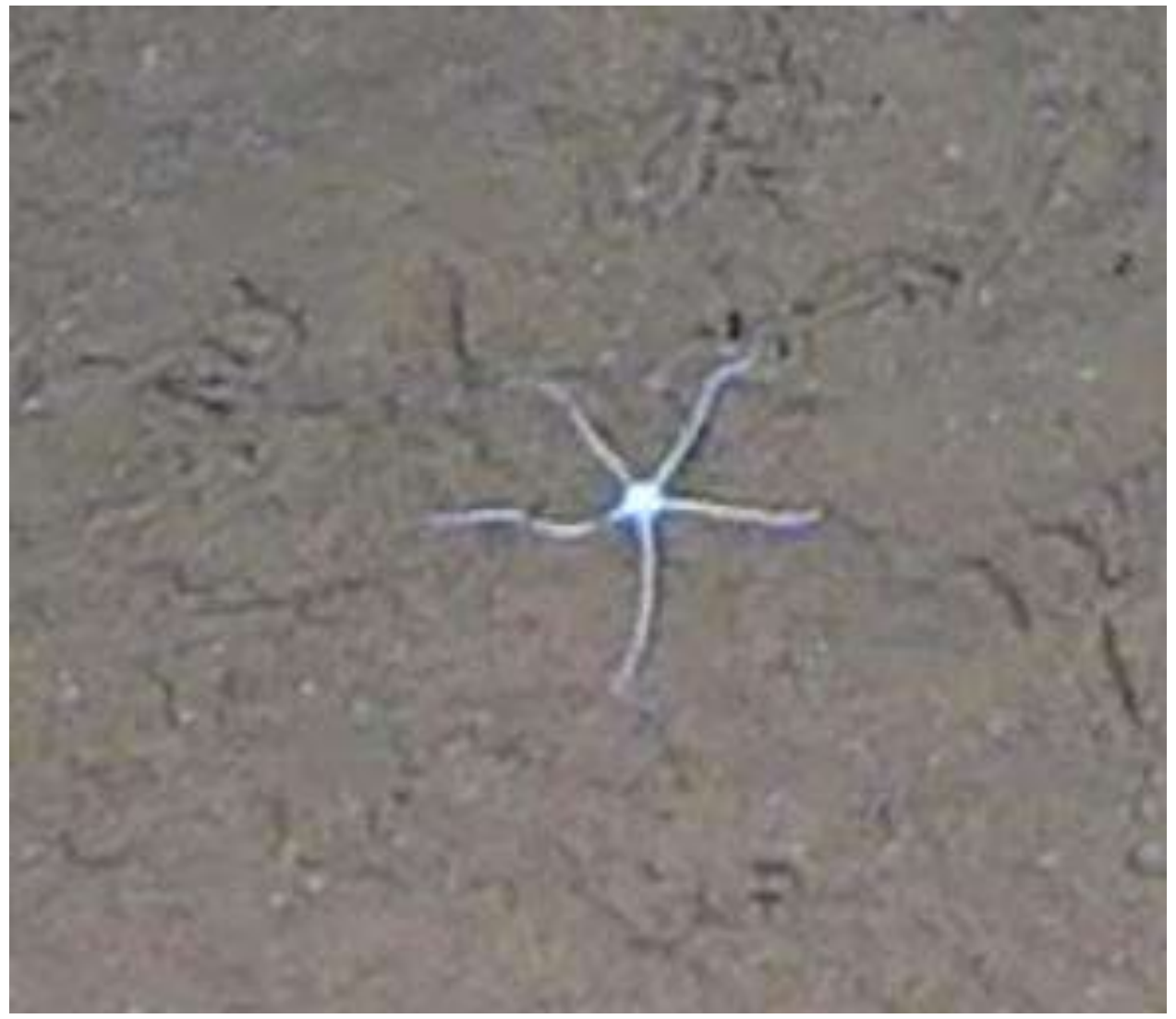

White; body a brigher colour white than arms (for sp6 body is darker). 2-3 cm wide, highly abundant on rocky substrate

Ophiuroidea;
Ophiuroidea ord. inc. 9

NC2_BK4_5300
NC3_BK2_6000

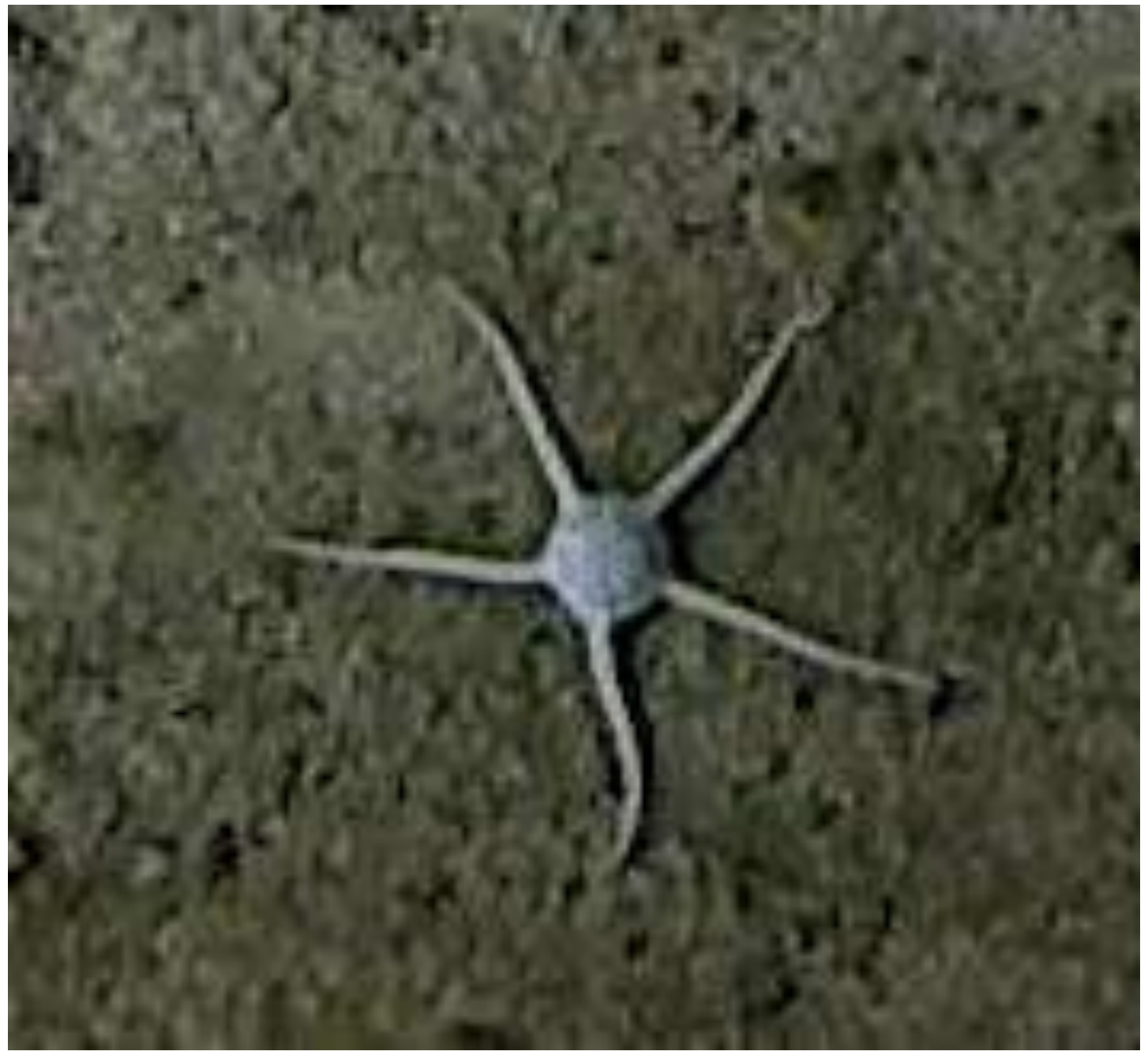

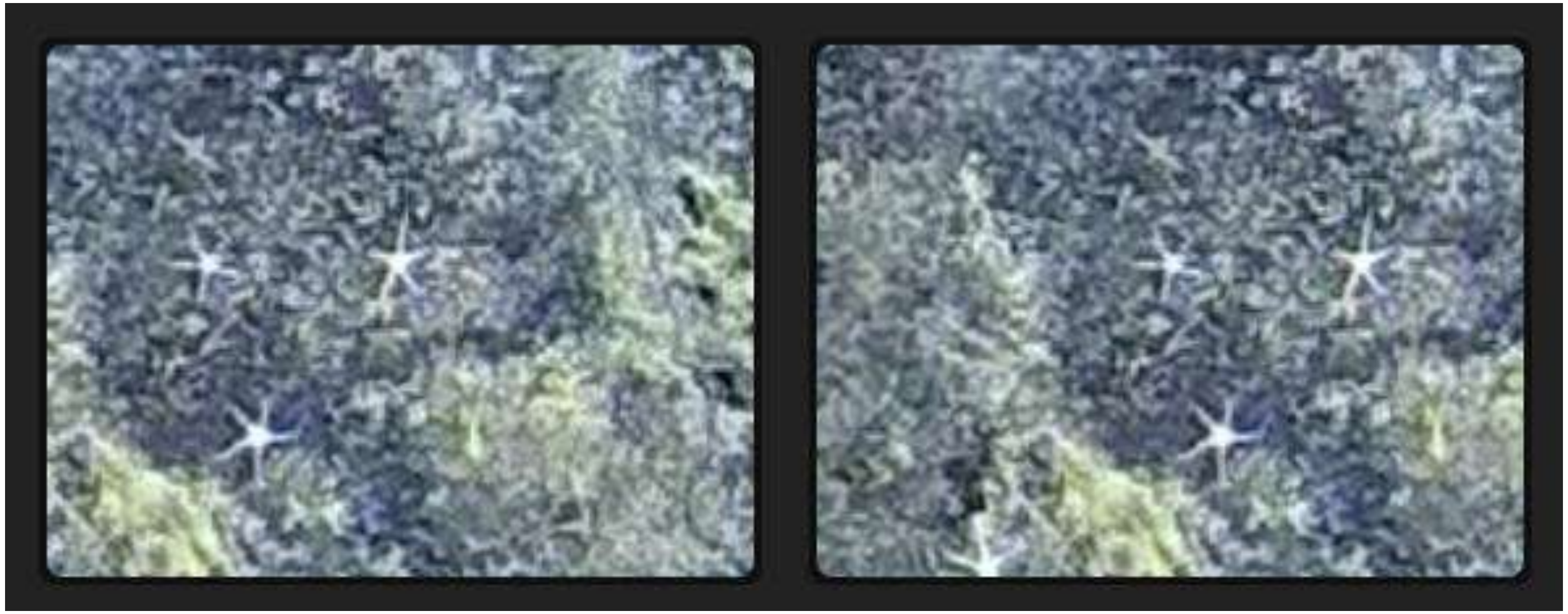

White; proportion arms to disc shorter than in 8 or 6.

NC1_BK1_3200

Ophiuroidea;
Ophiuroidea ord. inc. 10

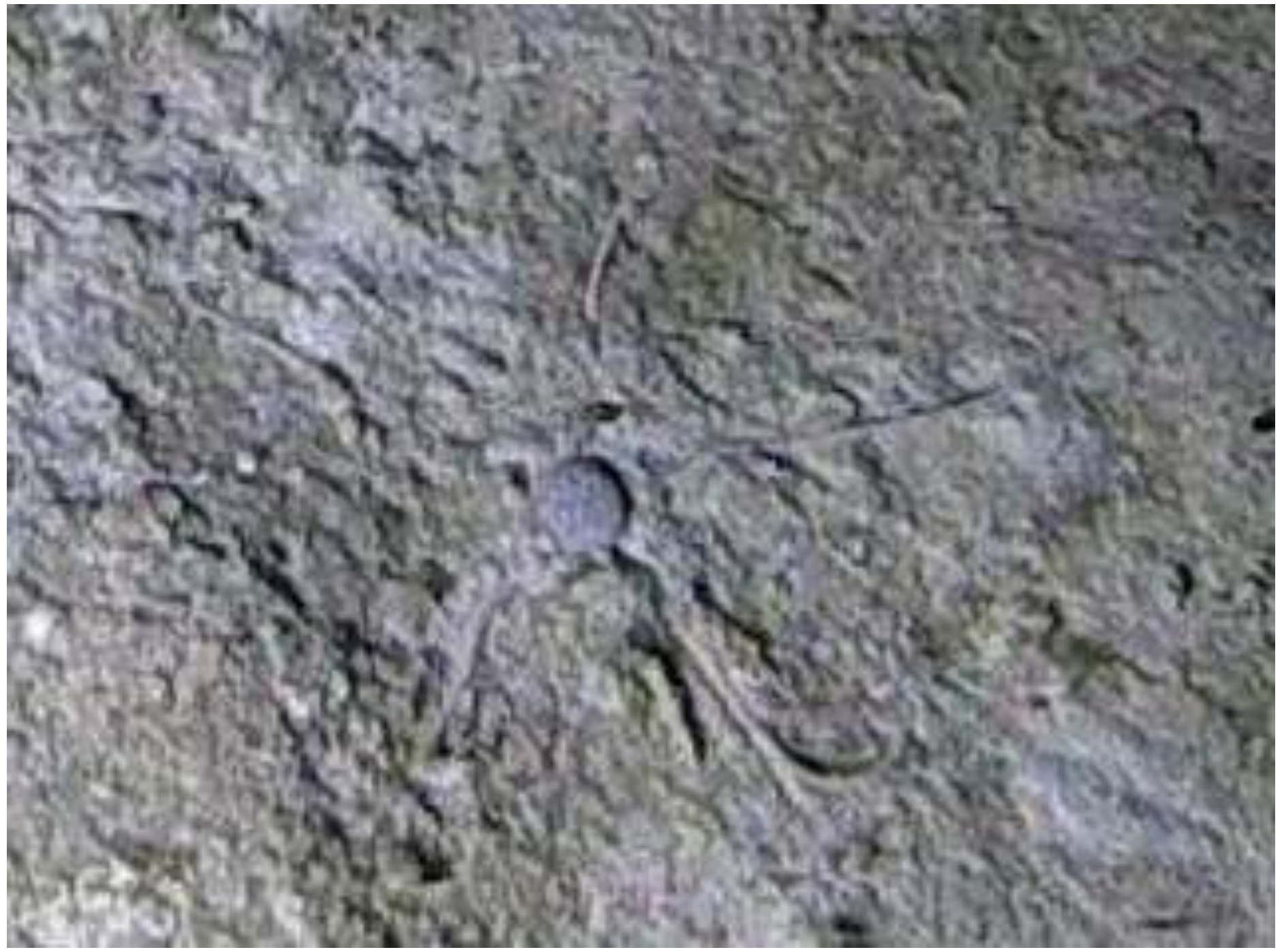

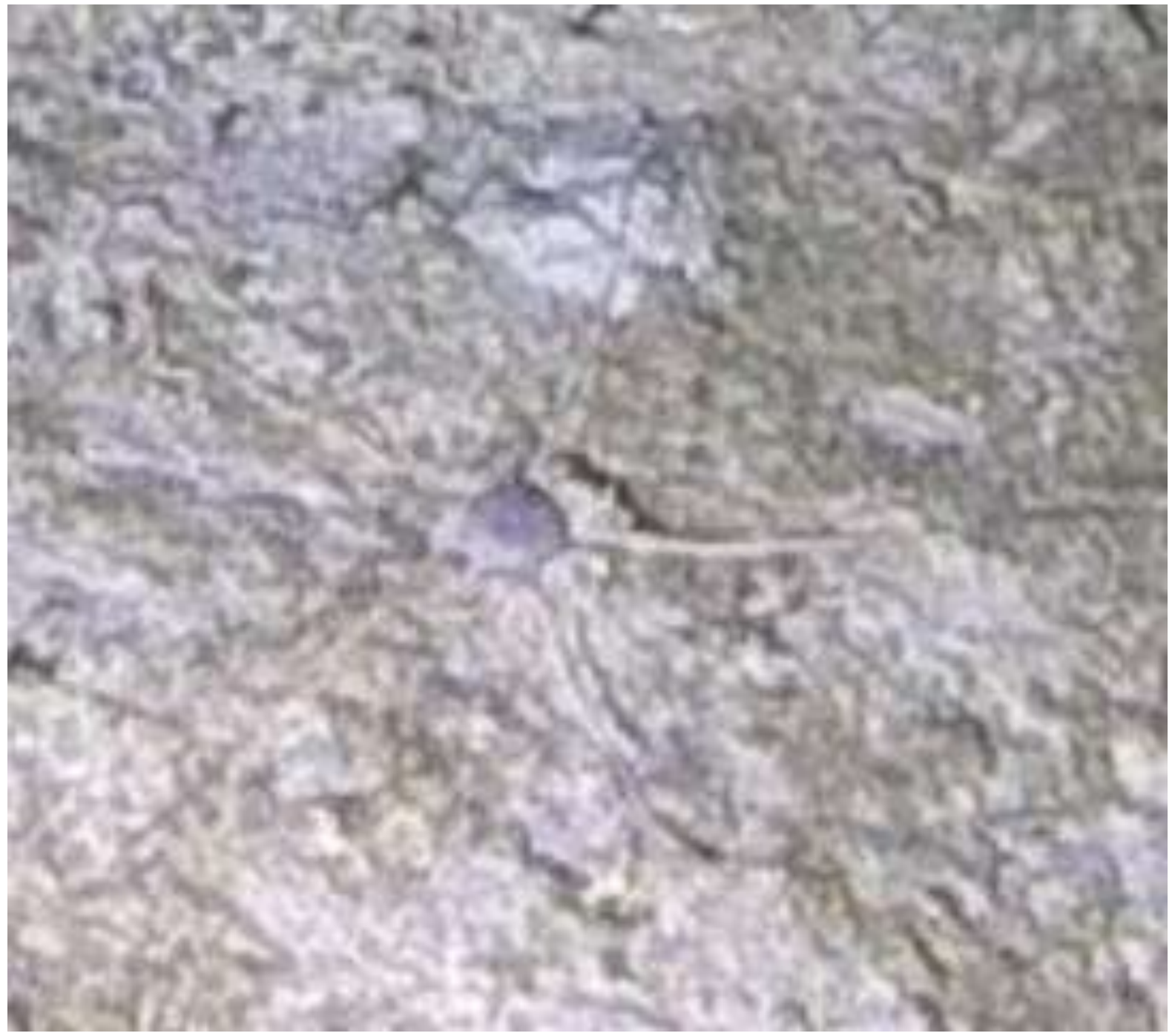

Light purple rounded body, found buried in sediment, elongated arms thinner than in 1.

NC4_OM5_6900

Holothuroidea; Holothuroidea spp.

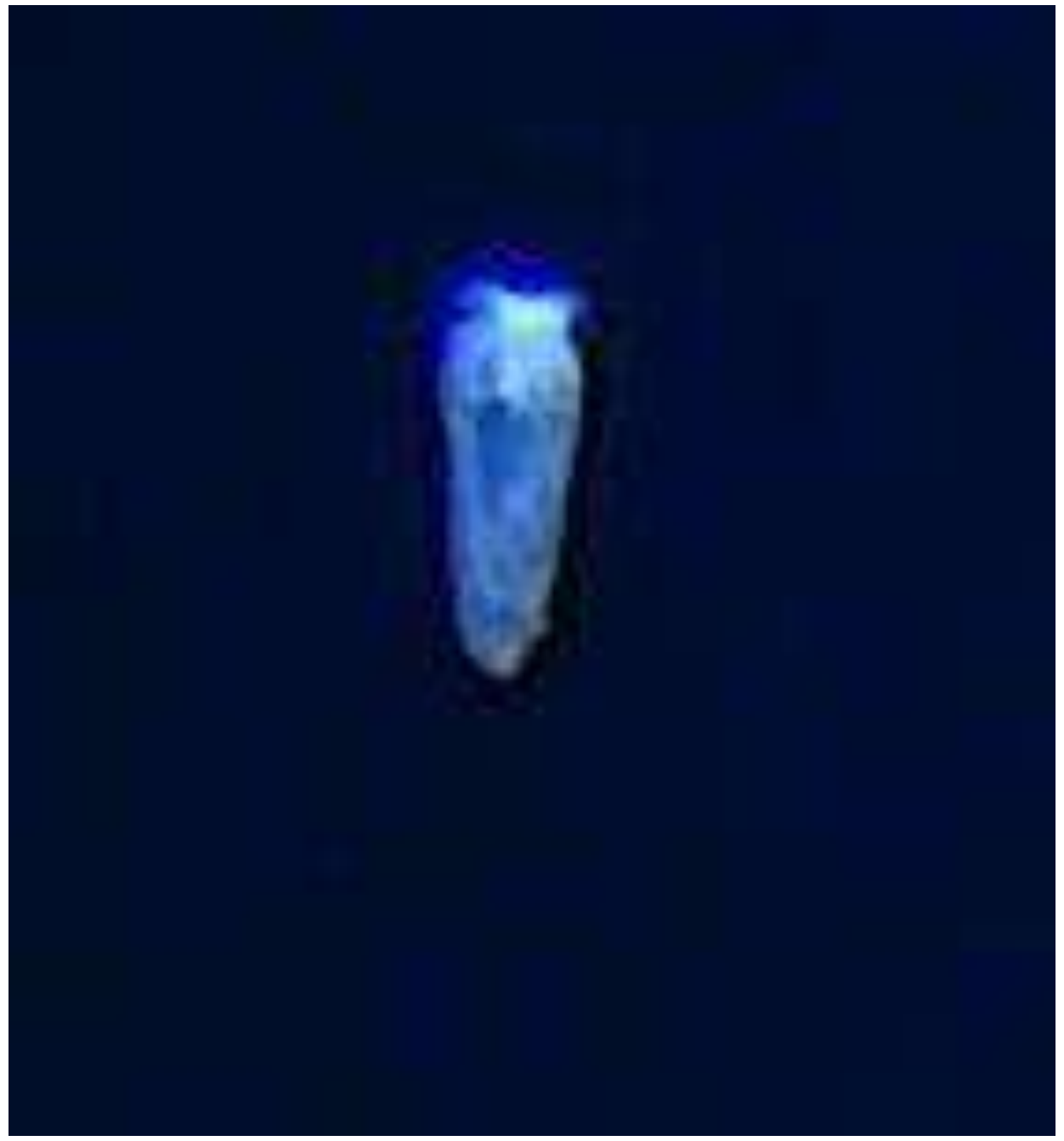

.

NC4_BK2_4700_NOR_0060

Holothuroidea; Elasipodida; Elpidiidae ; Elpidiidae gen. indet. 1

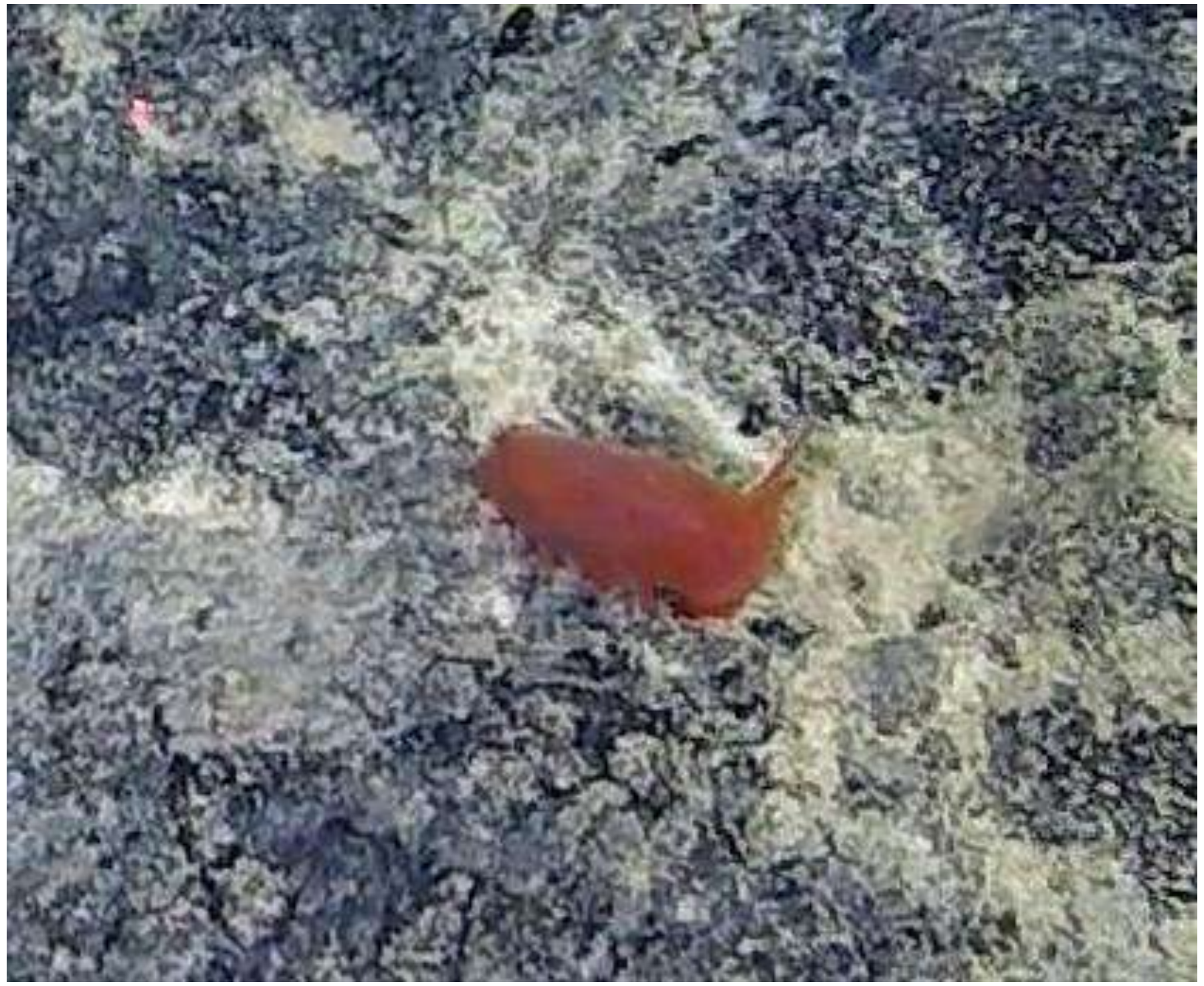

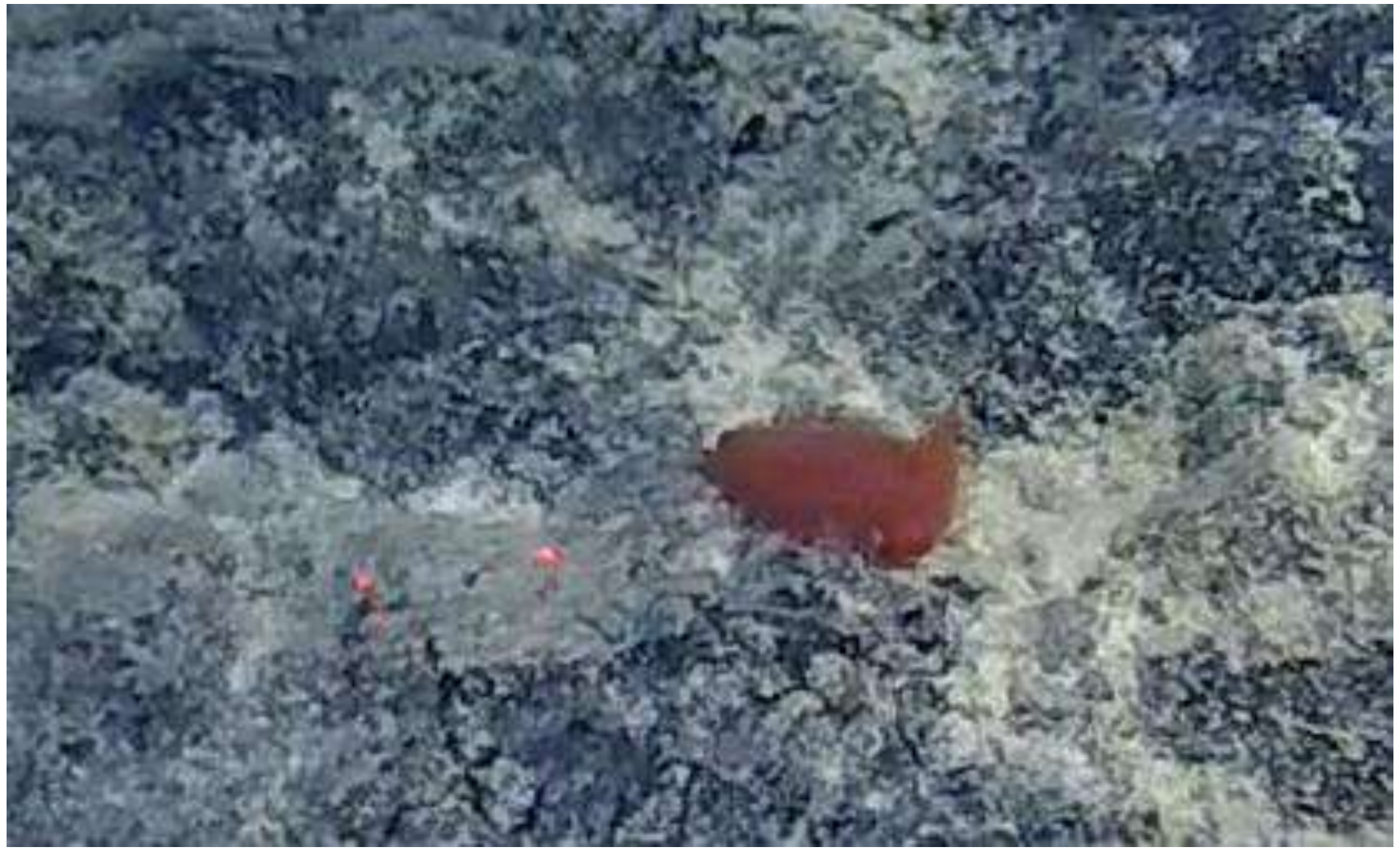

Pink colouration on ovoid Body. Vulum is built of two pairs of short, thin, vertically erect papillae. Oral disk is large and prominent. Tubefeet bordering posterior half.

NC2_BK5_4600_NOR_0066

Holothuroidea; Elasipodida; Elpidiidae ; Elpidiidae gen. indet. 2

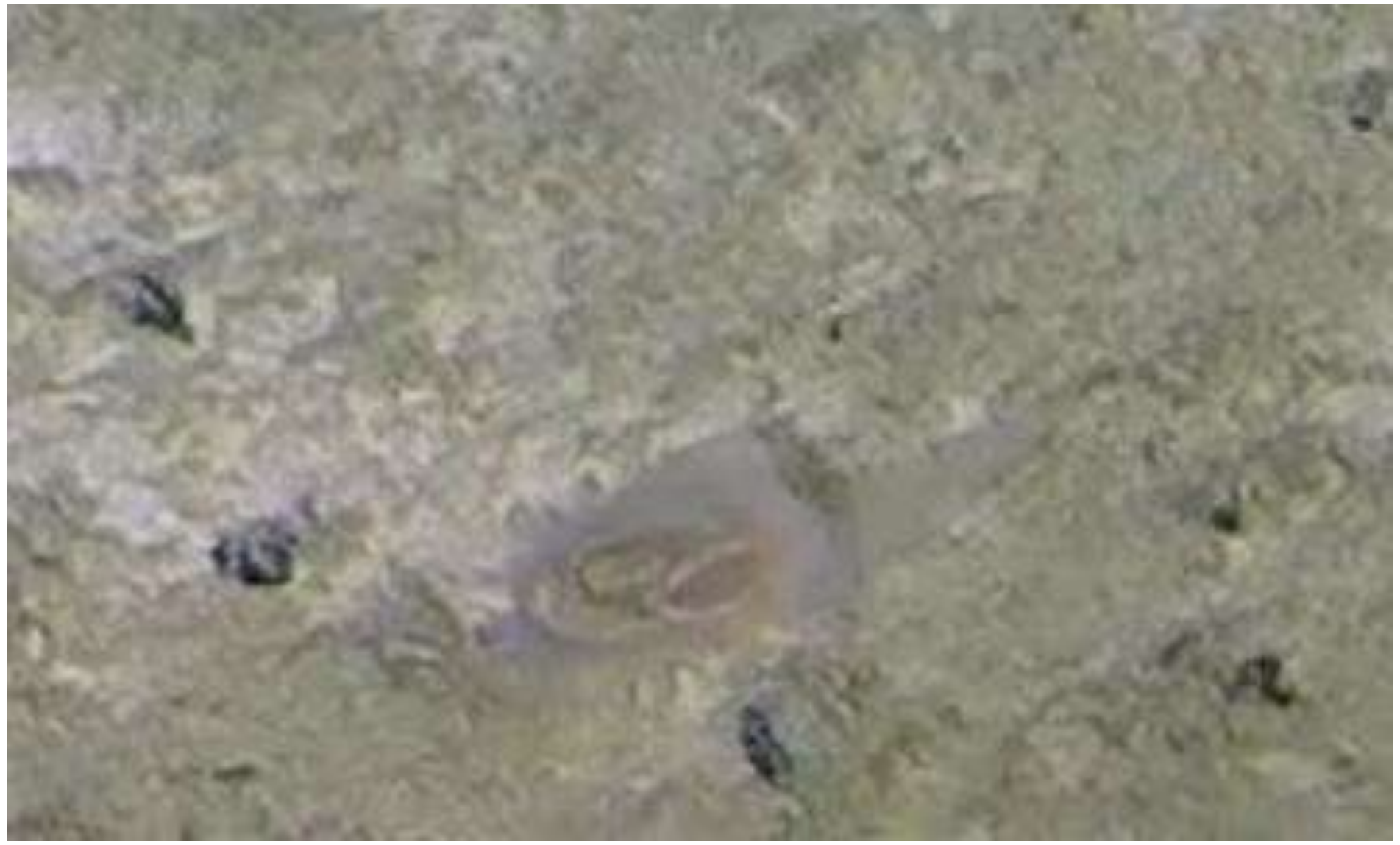

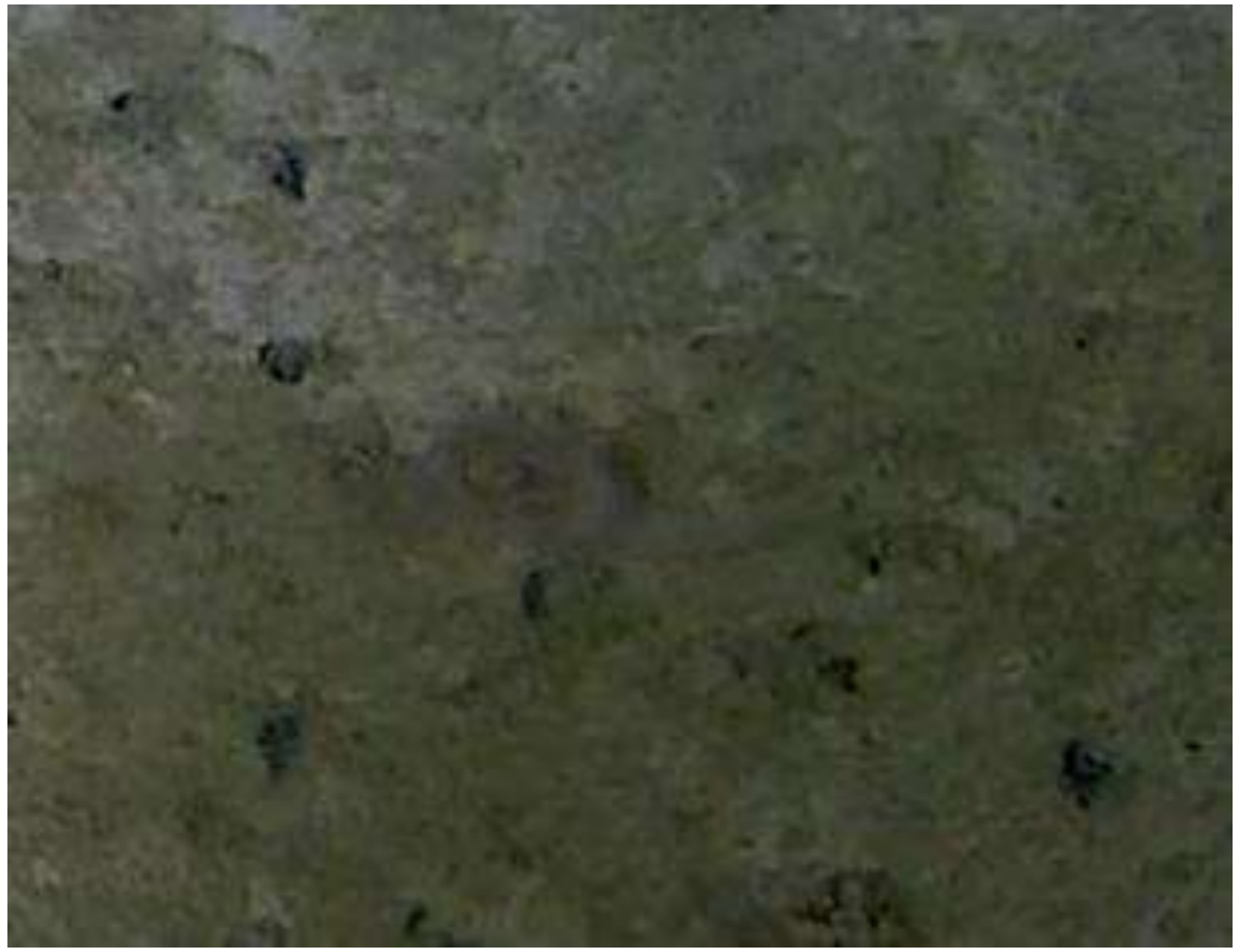

Pink translucent, one long papillae visible

Holothuroidea; Elasipodida; Elpidiidae; *Amperima* sp. indet. 1

NC3_BK2_6000_IMG_1998

NC3_BK2_6000_IMG_1999

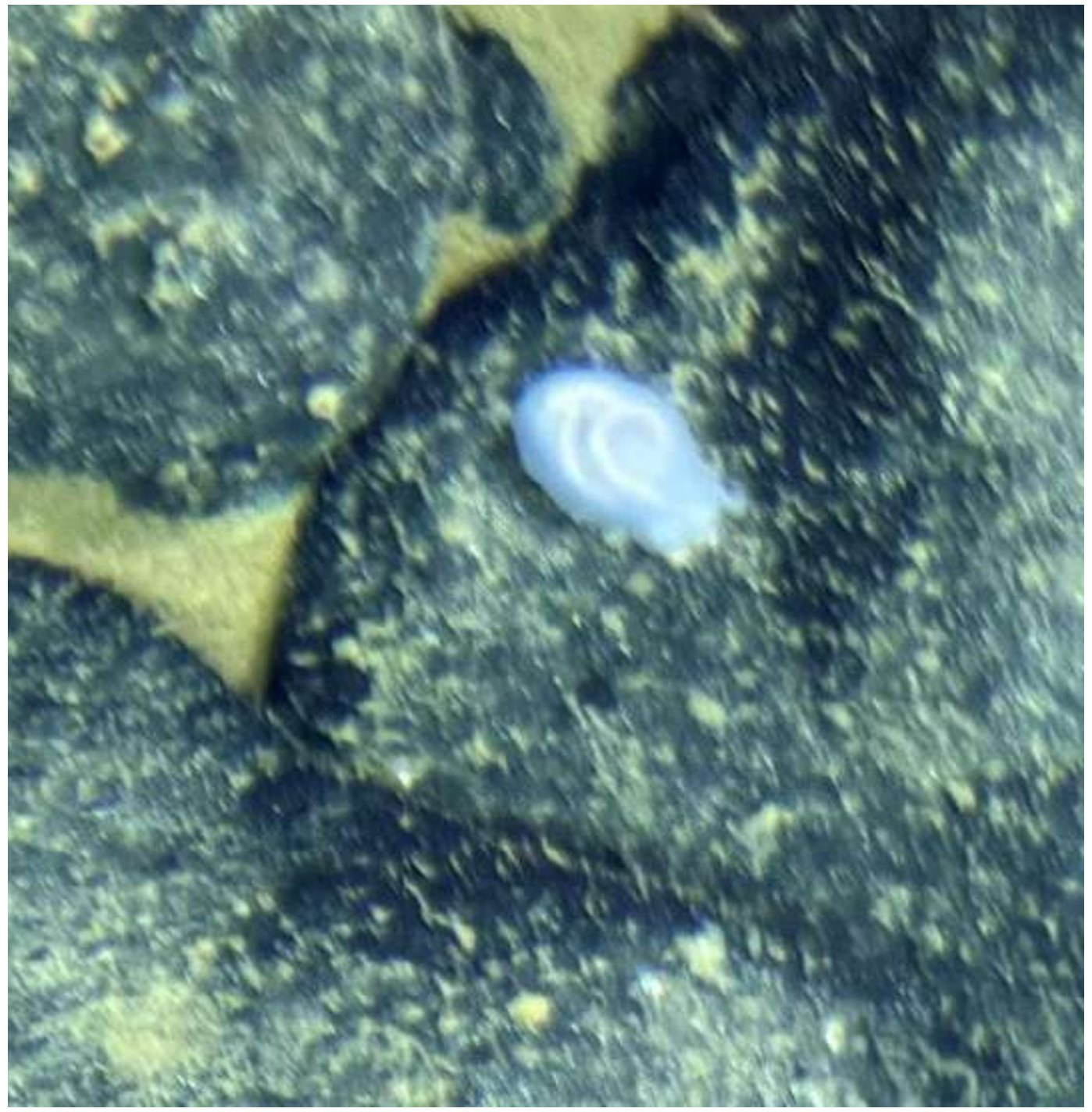

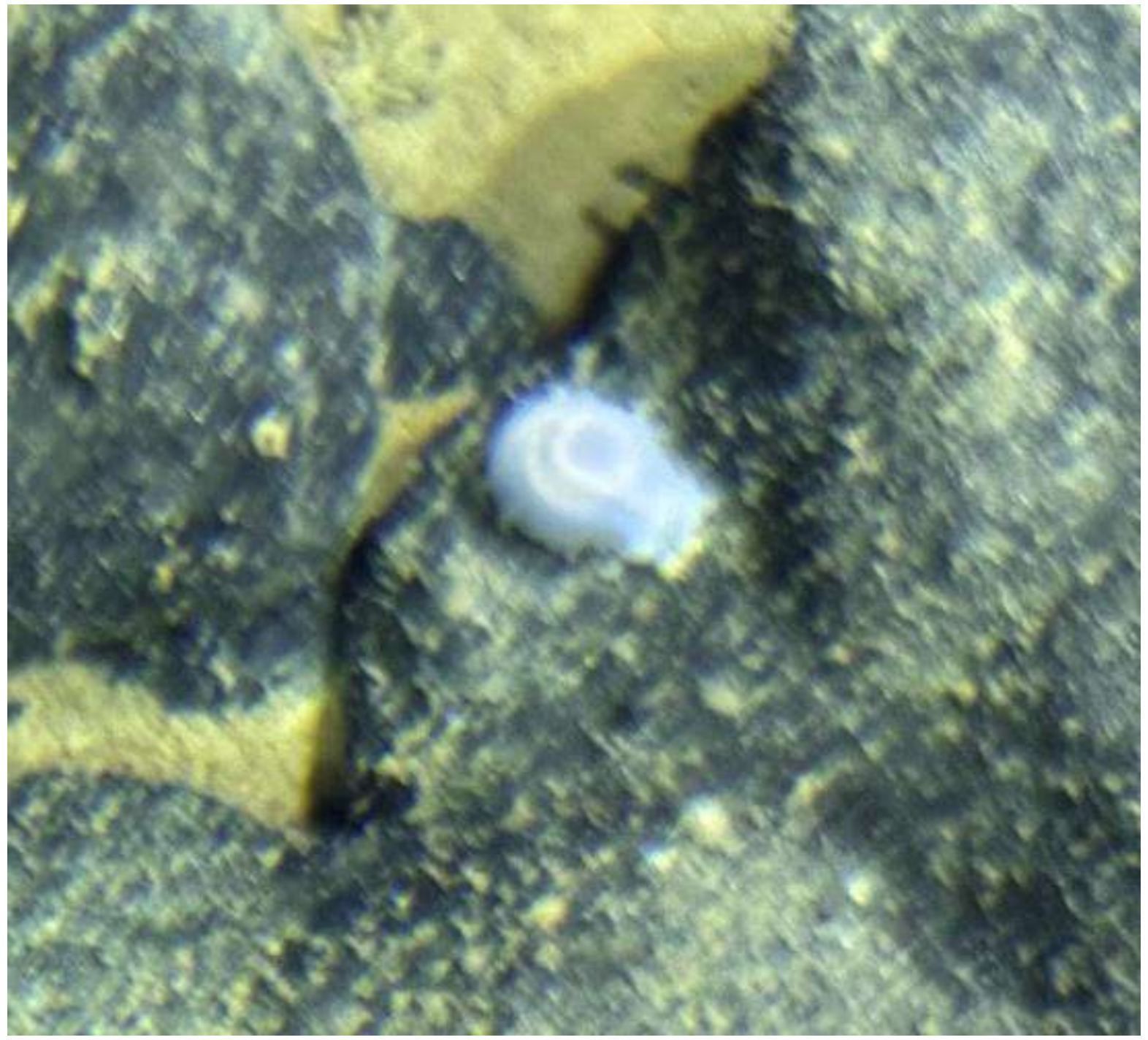

Skin is pale and translucent. Body is short and ovoid with bare; arched dorsal side.

Holothuroidea; Elasipodida; Elpidiidae; *Amperima* sp. indet. 2

NC3_BK3_6000_IMG_3388

NC3_BK3_6000_IMG_3389

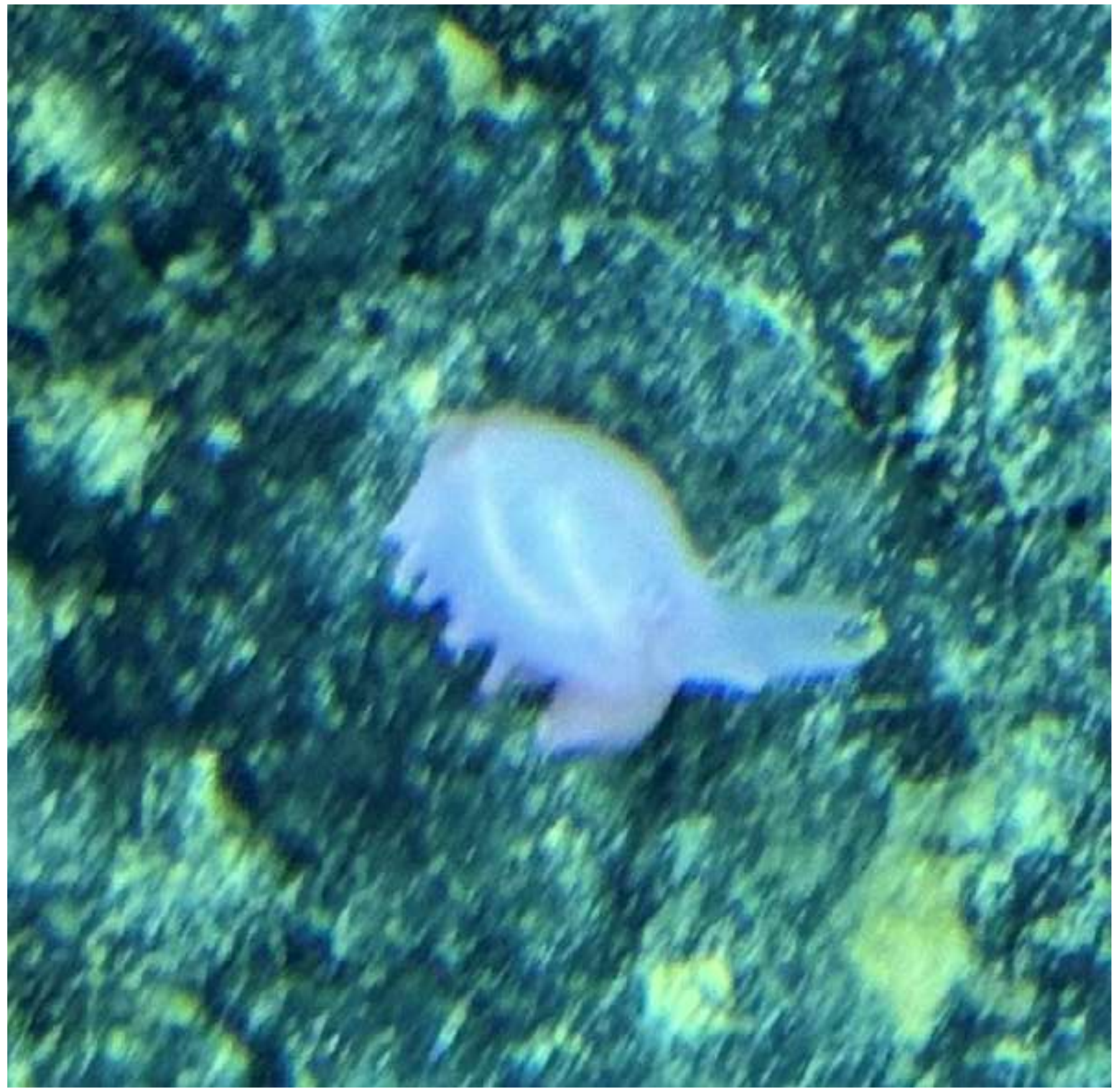
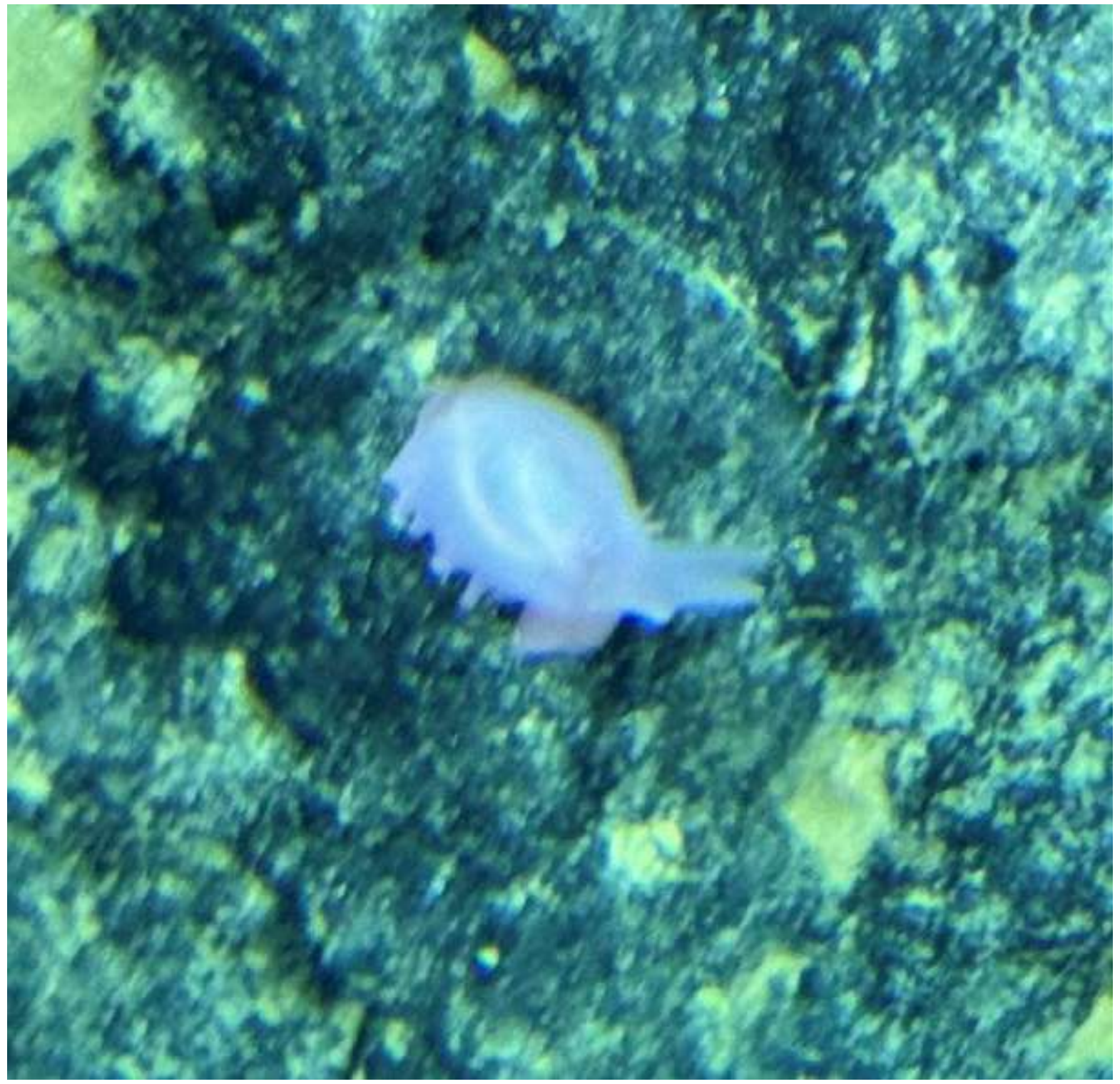

Skin is pale pink and translucent. Body is short and ovoid with bare; arched dorsal side. Vulum is built of 3 papillae. Very small individuals (~5cm)

Holothuroidea; Elasipodida; Elpidiidae ; Peniagone; *Peniagone leander*

NC4_MA5_6000_NOR_0042

NC4_BK3_6900_IMG_5694

NC4_BK1_5600_NOR_0068

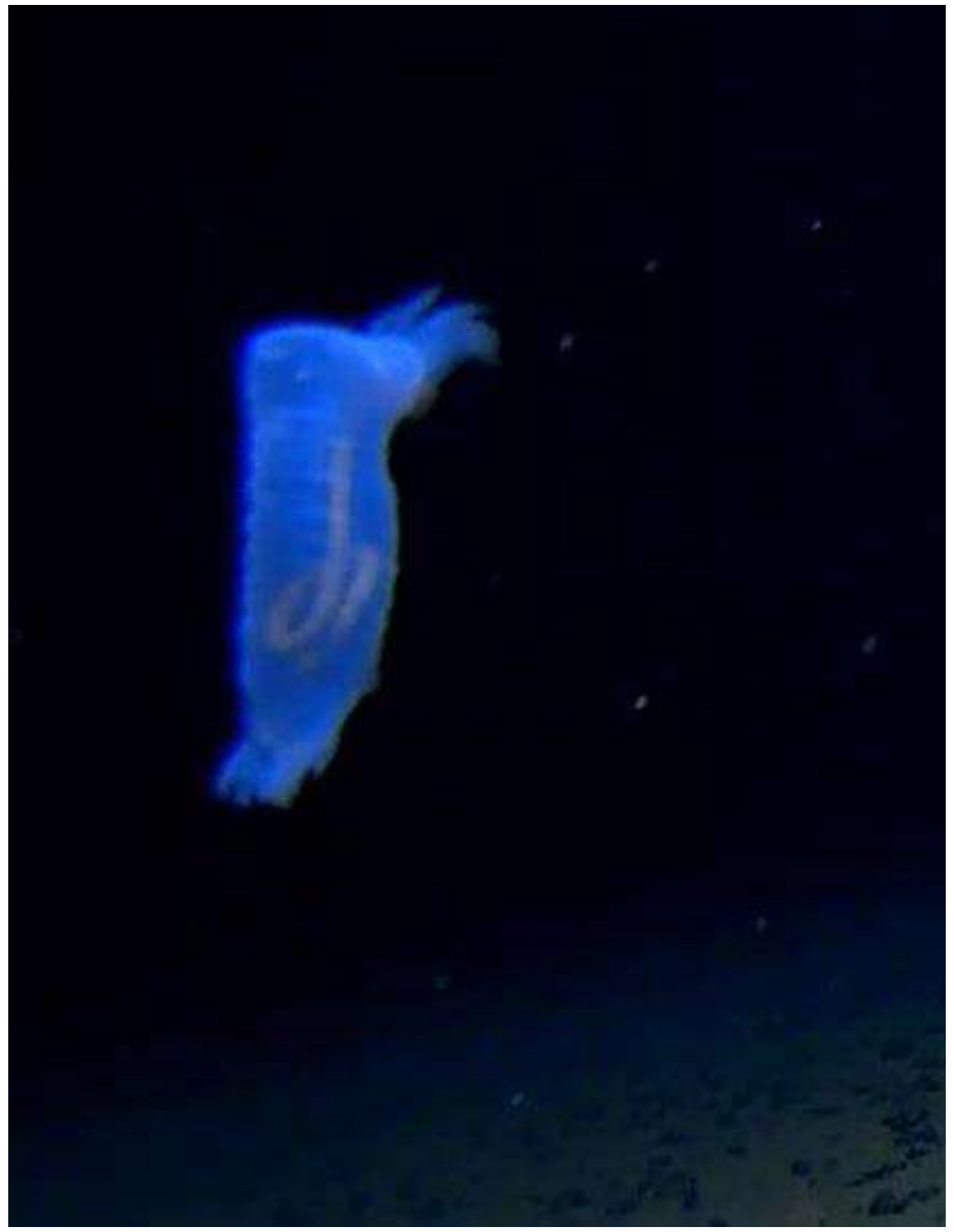

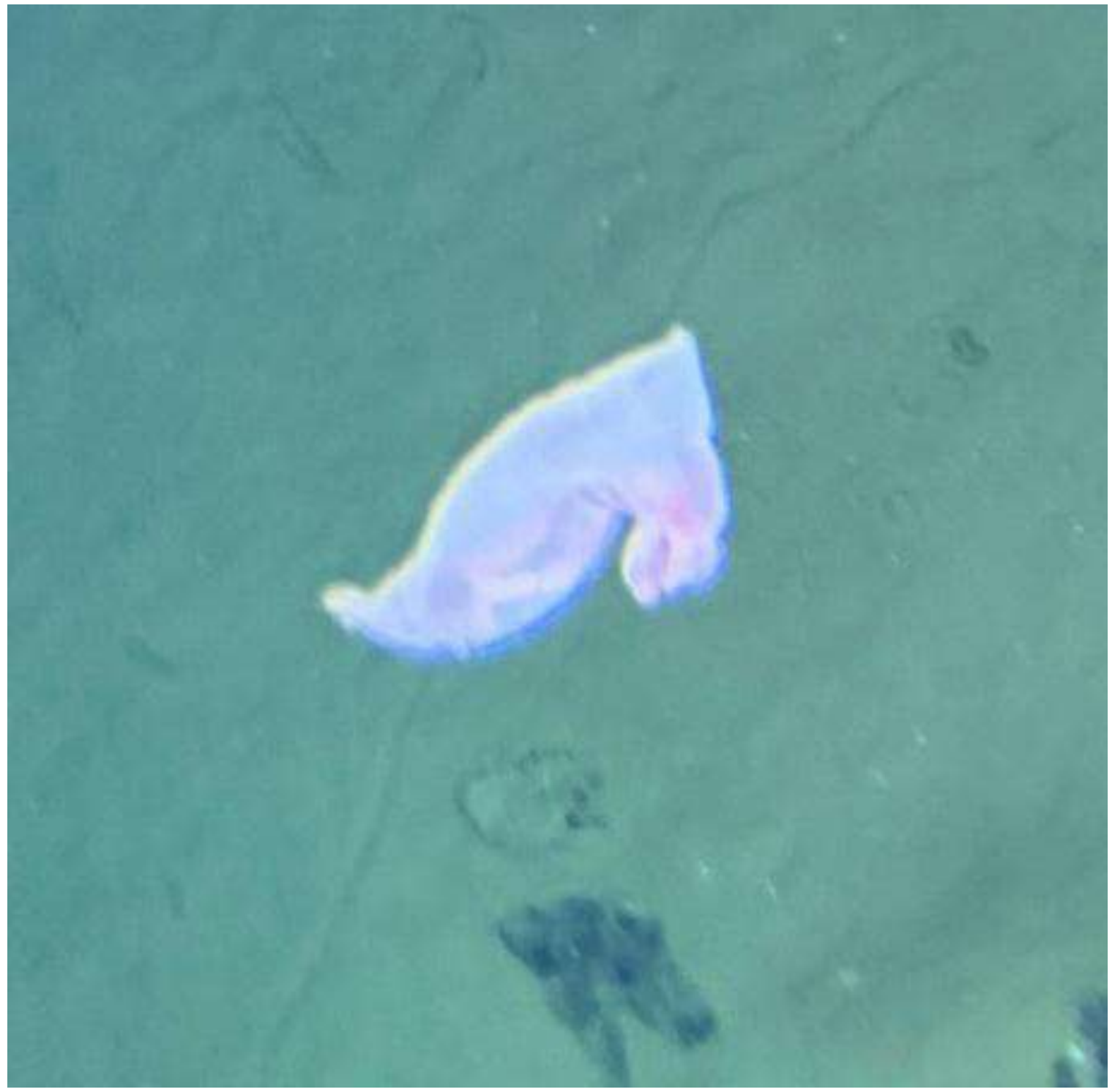

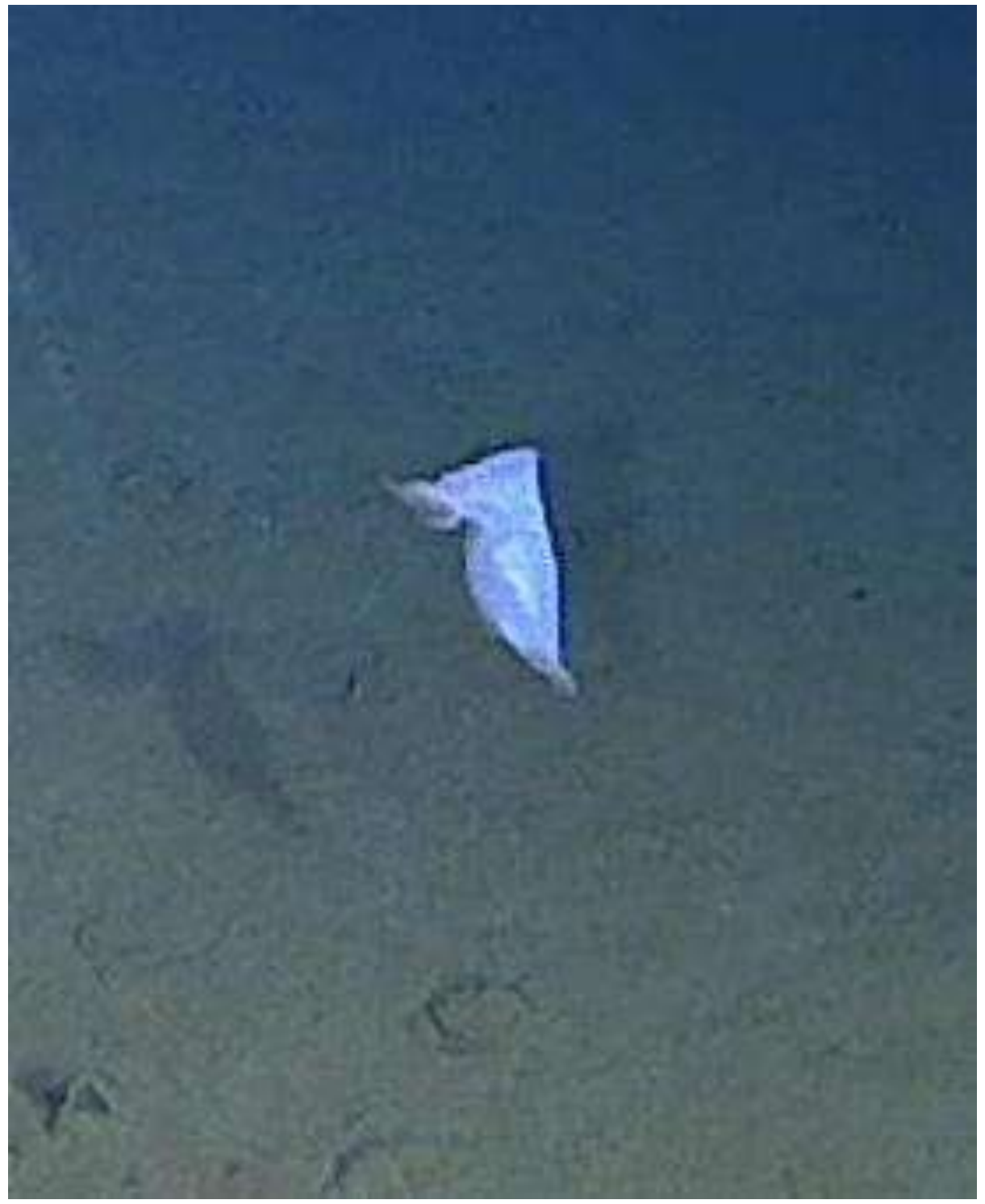

Skin is transparent. Body is short and ovoid. Velum is broad; with multiple projections. Ventral tube feet are restricted to the posterior portion of the body and for a fringe.

Holothuroidea; Elasipodida; Elpidiidae ; Peniagone; *Peniagone* sp. indet. 1

NC3_BK4_8000_IMG_4226
NC3_BK4_8000_IMG_4146
NC2_BK3_7400_IMG_8537

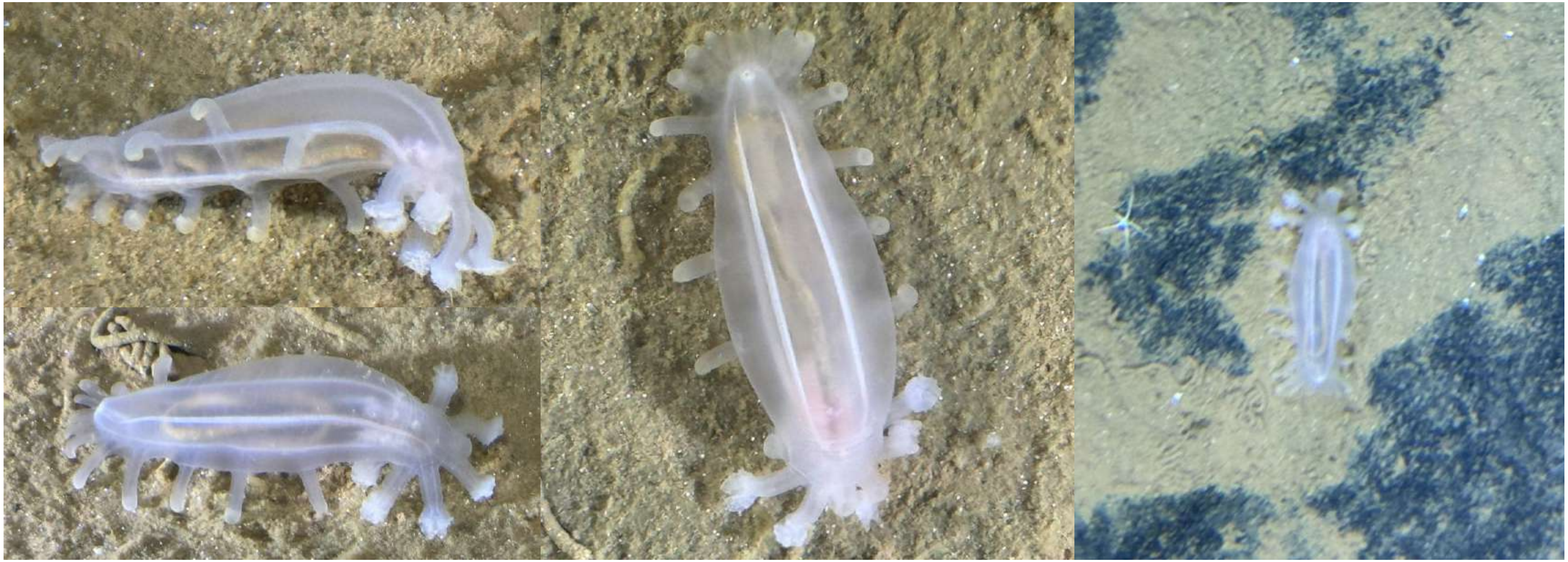

Skin is translucent/ white. Body is longer ovoid shape. 4 pairs of ventral tube feet occur along the sides. 3 pairs of tentacles line the mouth. Brim is present on the posterior end. No velum visible. Anus on dorsal surface

Holothuroidea; Elasipodida; Elpidiidae ; Peniagone; *Peniagone* sp. indet. 2.

NC2_BK5_4600_NOR_0074

NC2_BK5_4600_IMG_0242

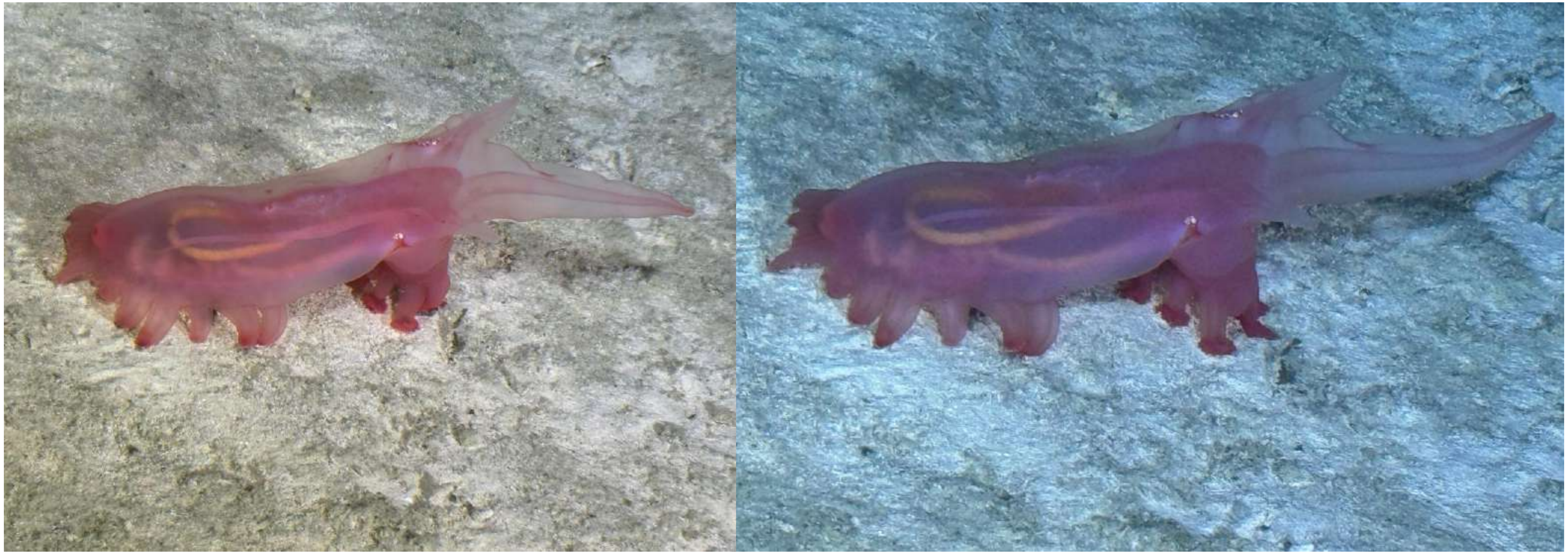

Pink colouration on ovoid Body. Vulum is built of two pairs of papillae with one pair of papillae much larger that the other. 6 pairs of Tubefeet bordering posterior half

Holothuroidea; Elasipodida; Elpidiidae ; Peniagone; *Peniagone* sp. indet. 3.

NC3_BK2_6000 _IMG_2177

NC3_BK2_6000_IMG_2178

NC3_BK2_6000 _IMG_2004

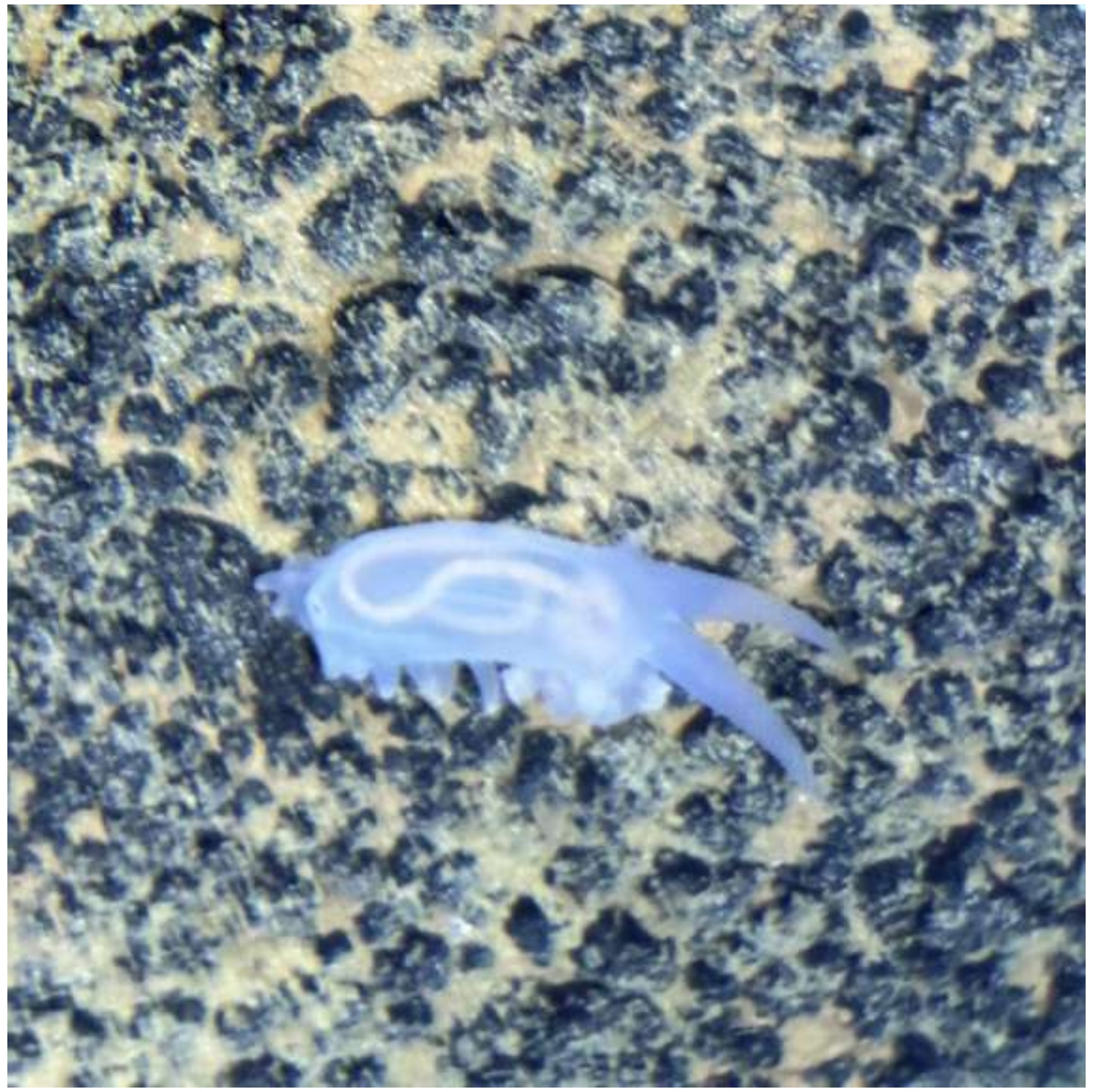
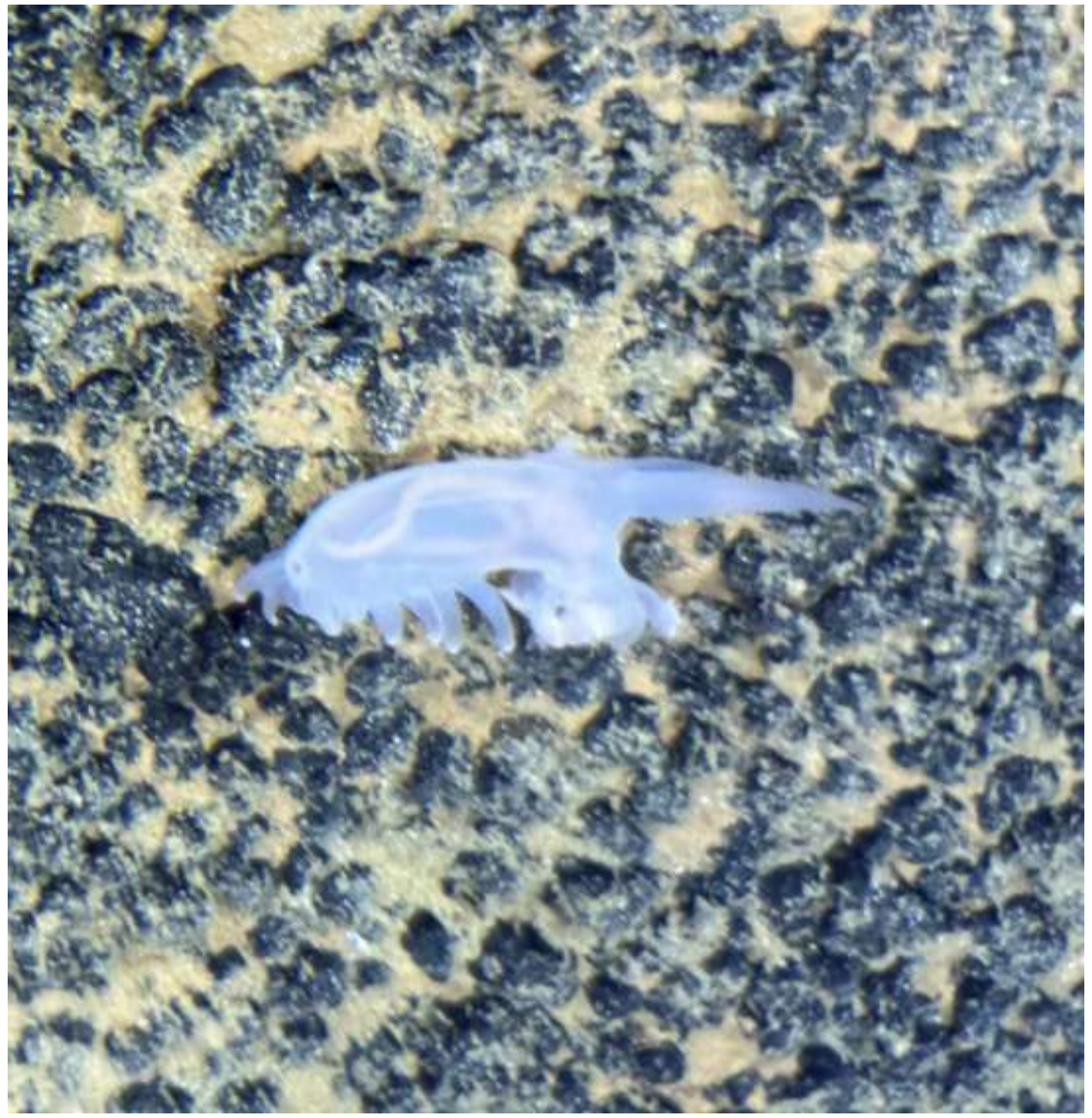
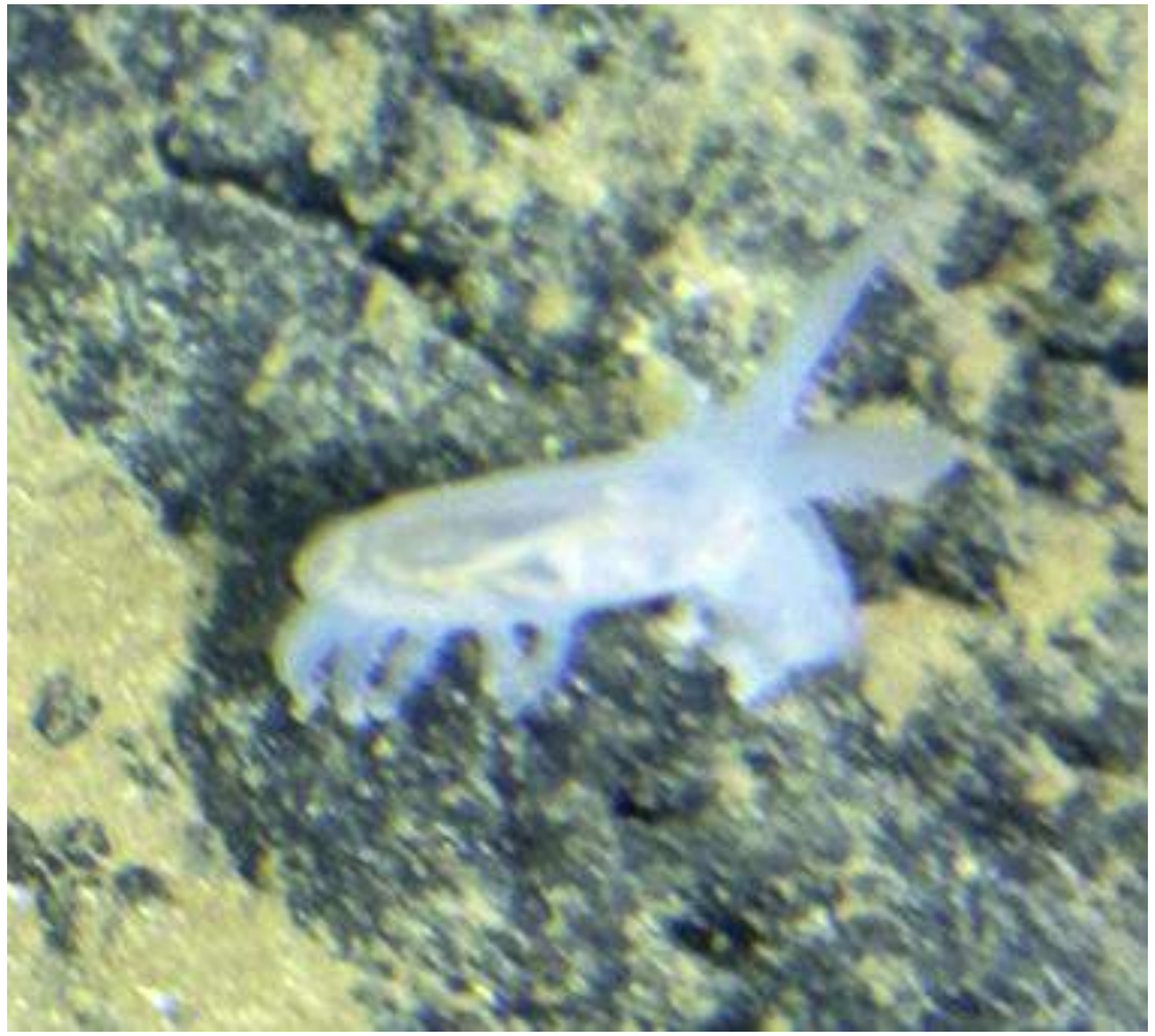

White/ translucent colouration on ovoid body. Vulum is built of 2 papillae. Horizontally positioned pair of papillae are longer. Tube feet bordering posterior half. Body size 15cm-20cm

Holothuroidea; Elasipodida; Elpidiidae ; Peniagone; *Peniagone* sp. indet. 4.

NC3_BK3_6000

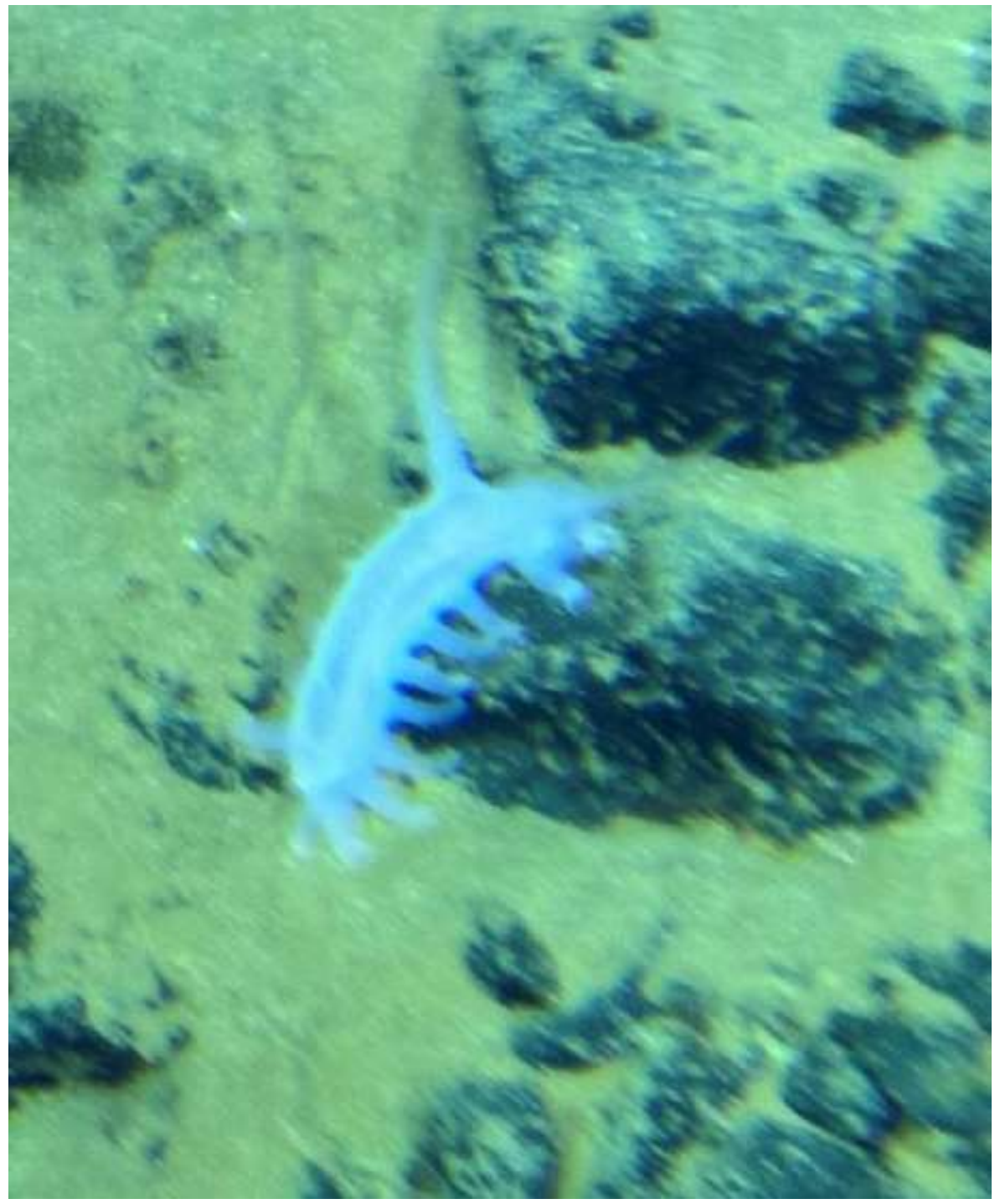

White/ translucent colouration on elongated body. Vulum is built of 2 papillae. Horizontally positioned pair of papillae are longer. 6 pair of tube feet.

Holothuroidea; Elasipodida; Elpidiidae ; Peniagone; *Peniagone* sp. indet. 5.

NC3_BK2_6000 _IMG_2004

NC3_BK2_6000 _NOR_0079

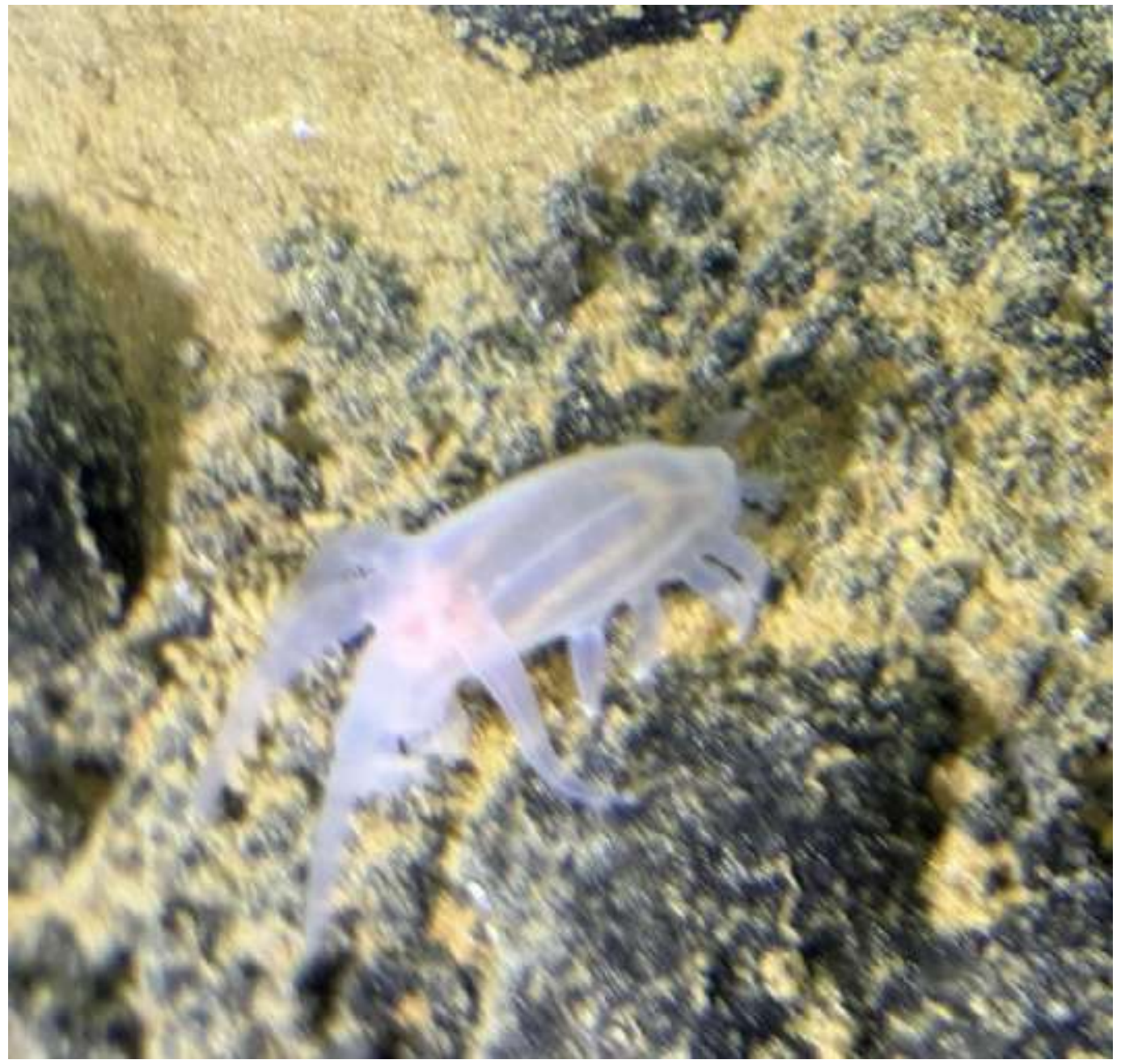

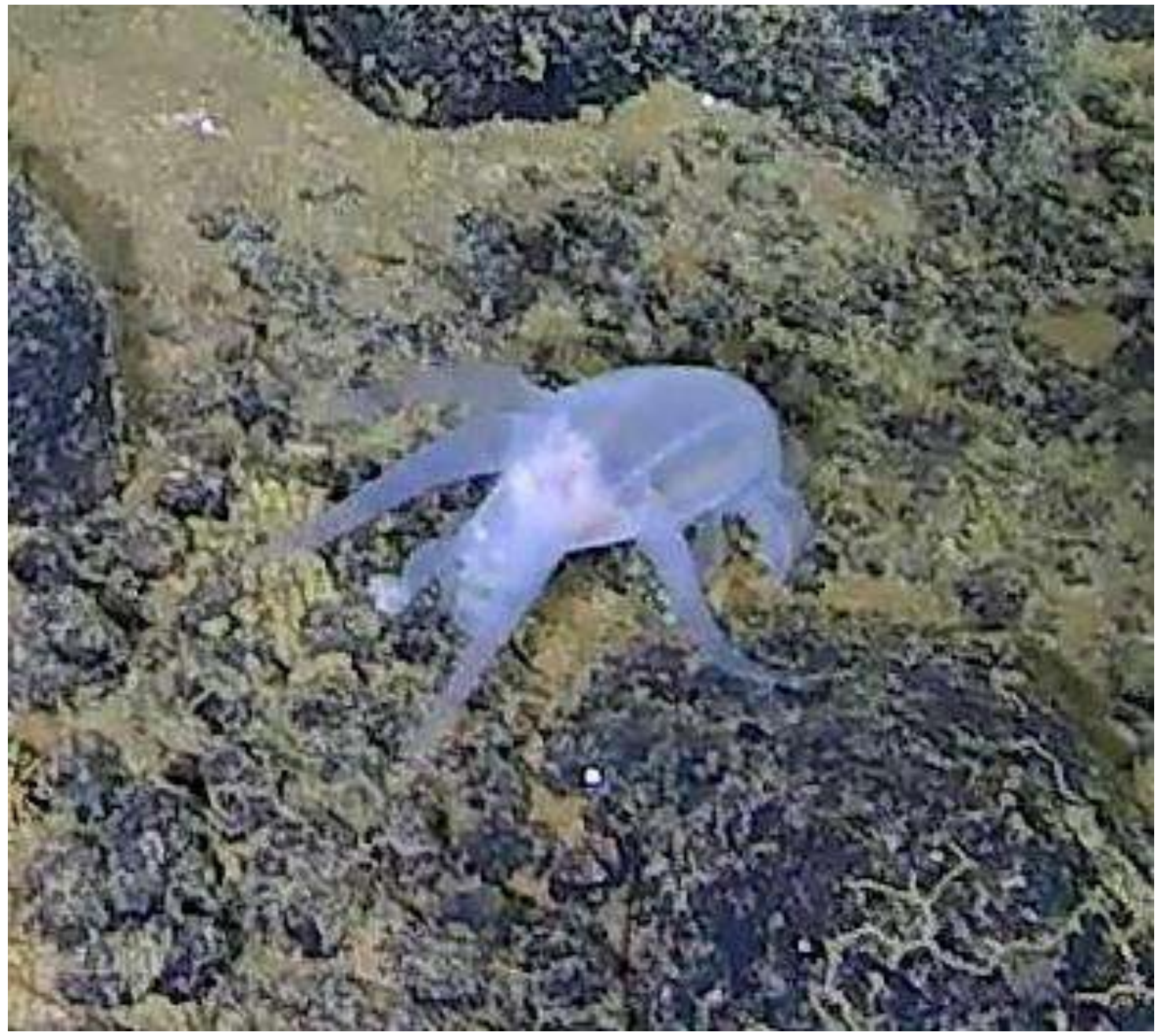

White/ translucent colouration on ovoid body. Vulum is built of 4 papillae. Horizontally positioned pair of papillae are longer. Tube feet bordering posterior half.

Holothuroidea; Elasipodida; Elpidiidae; Peniagone; *Peniagone* sp. indet. 6

NC3_BK2_6000 _IMG_1650

NC3_BK2_6000 _IMG_1648

NC3_BK2_6000 _IMG_ 1646

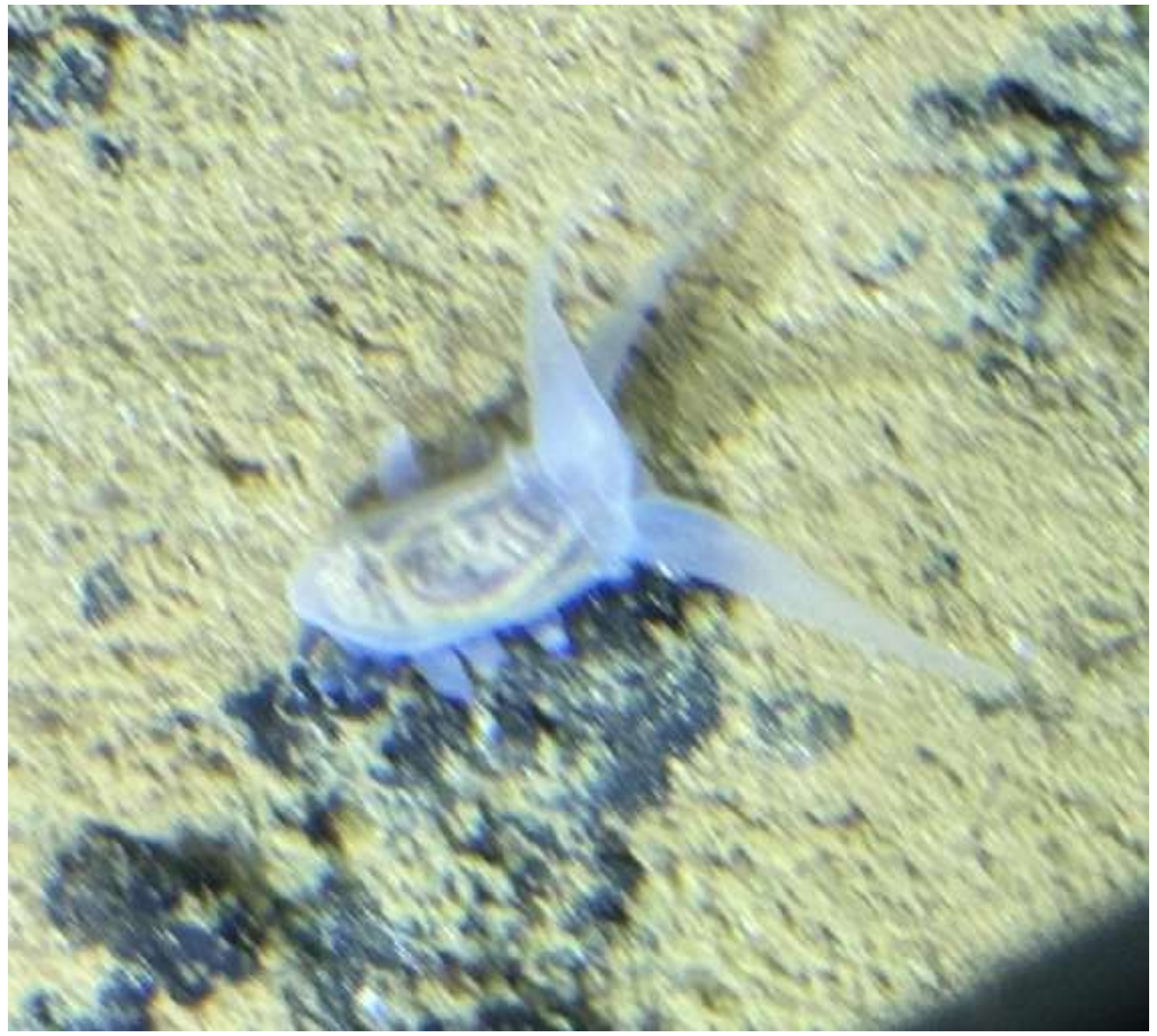

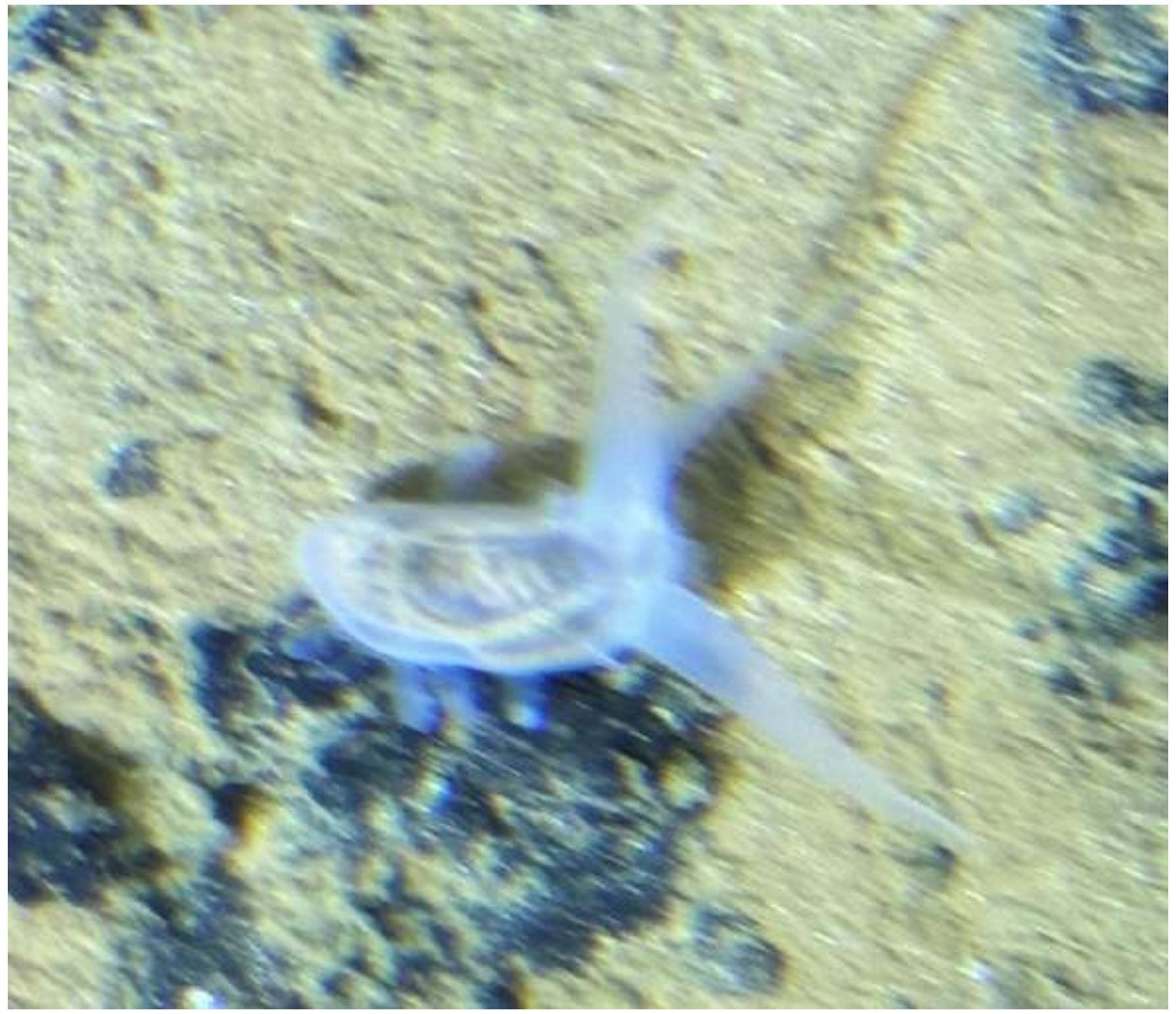

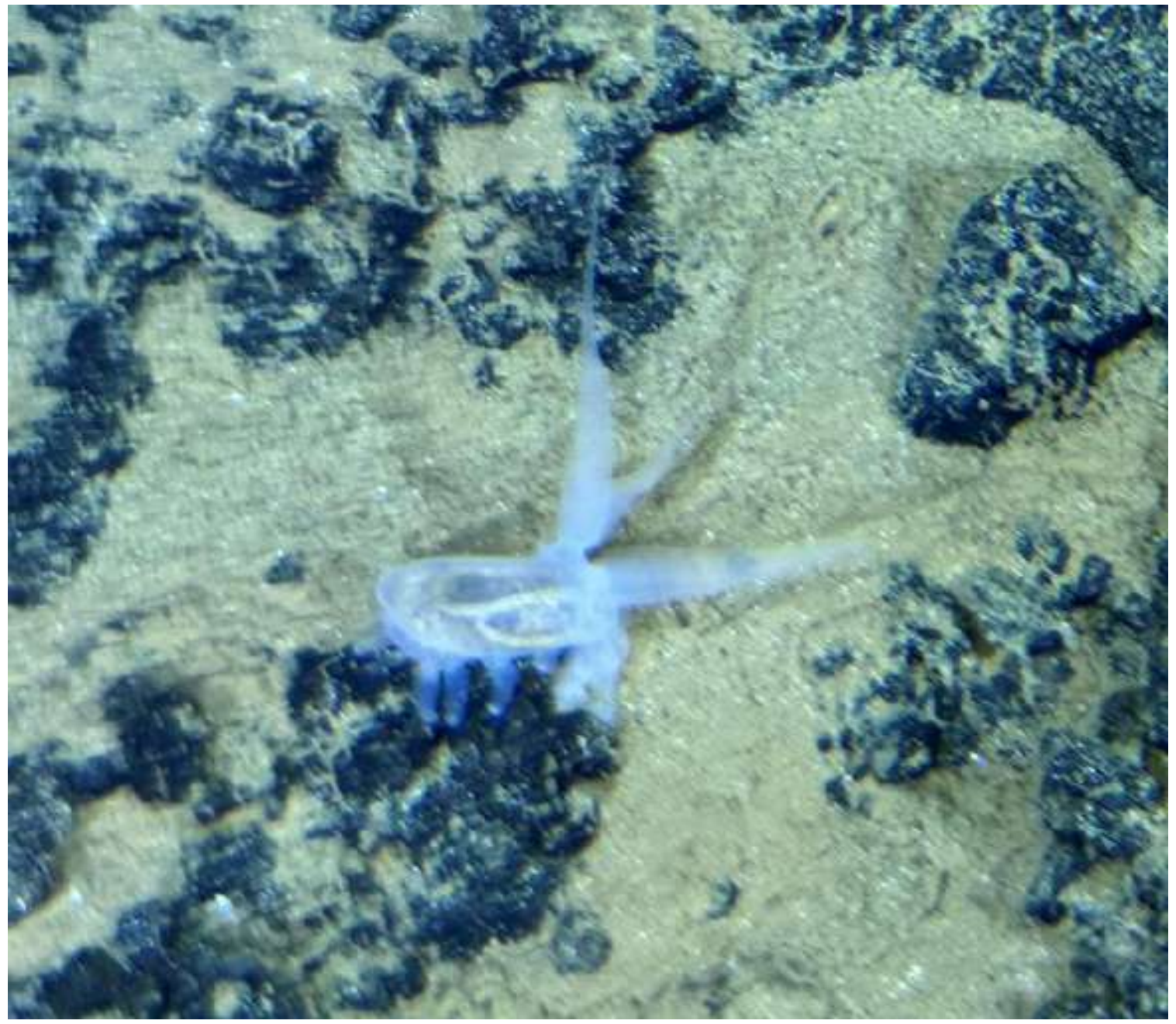

White/ translucent colouration on ovoid body. Vulum is built of three papillae. Horizontally positioned pair of papillae are longer. Tube feet bordering posterior half.

Holothuroidea; Elasipodida; Elpidiidae ; Peniagone; *Peniagone* sp. indet. 7

NC2_BK3_7400_IMG_8537

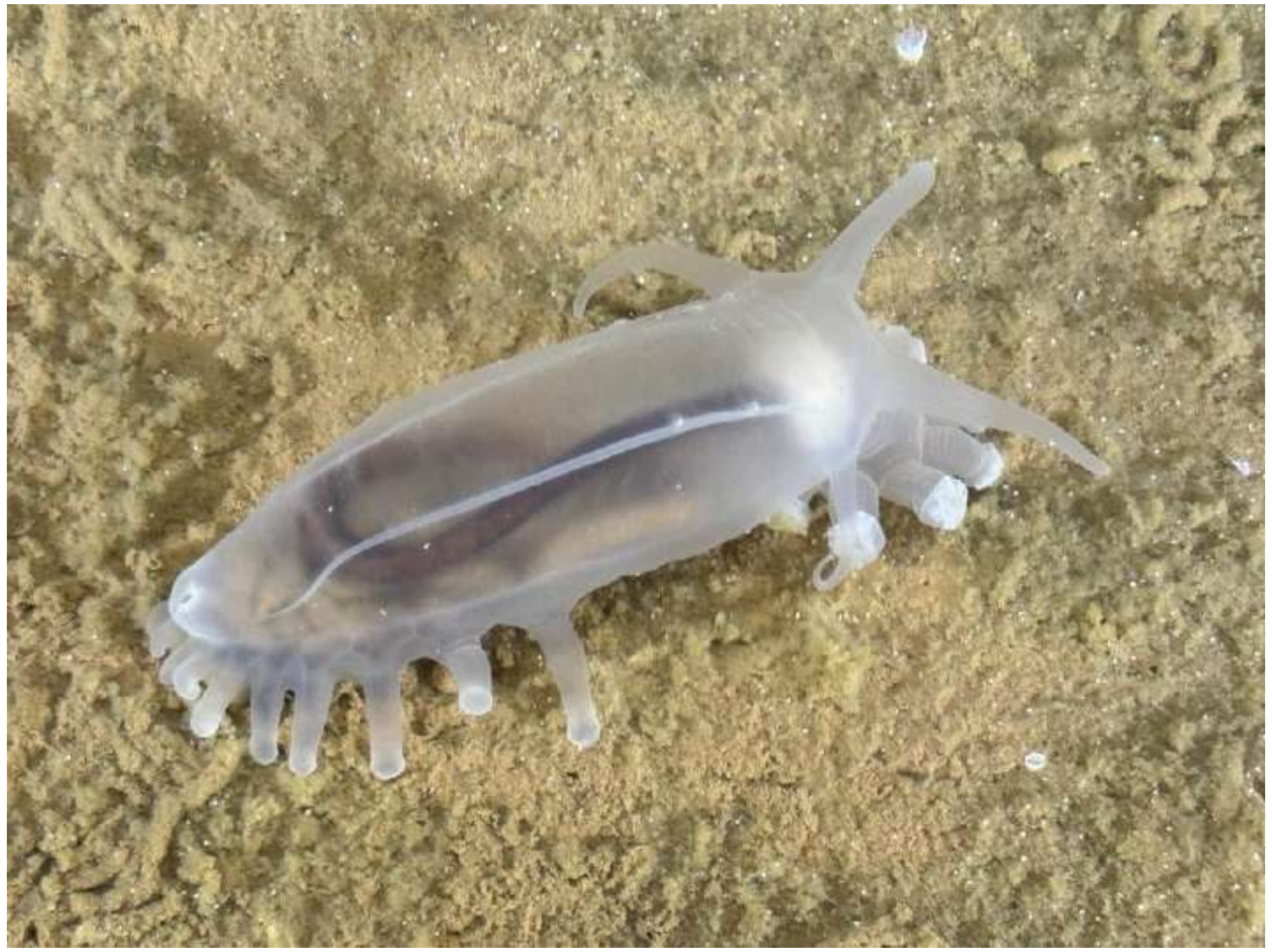

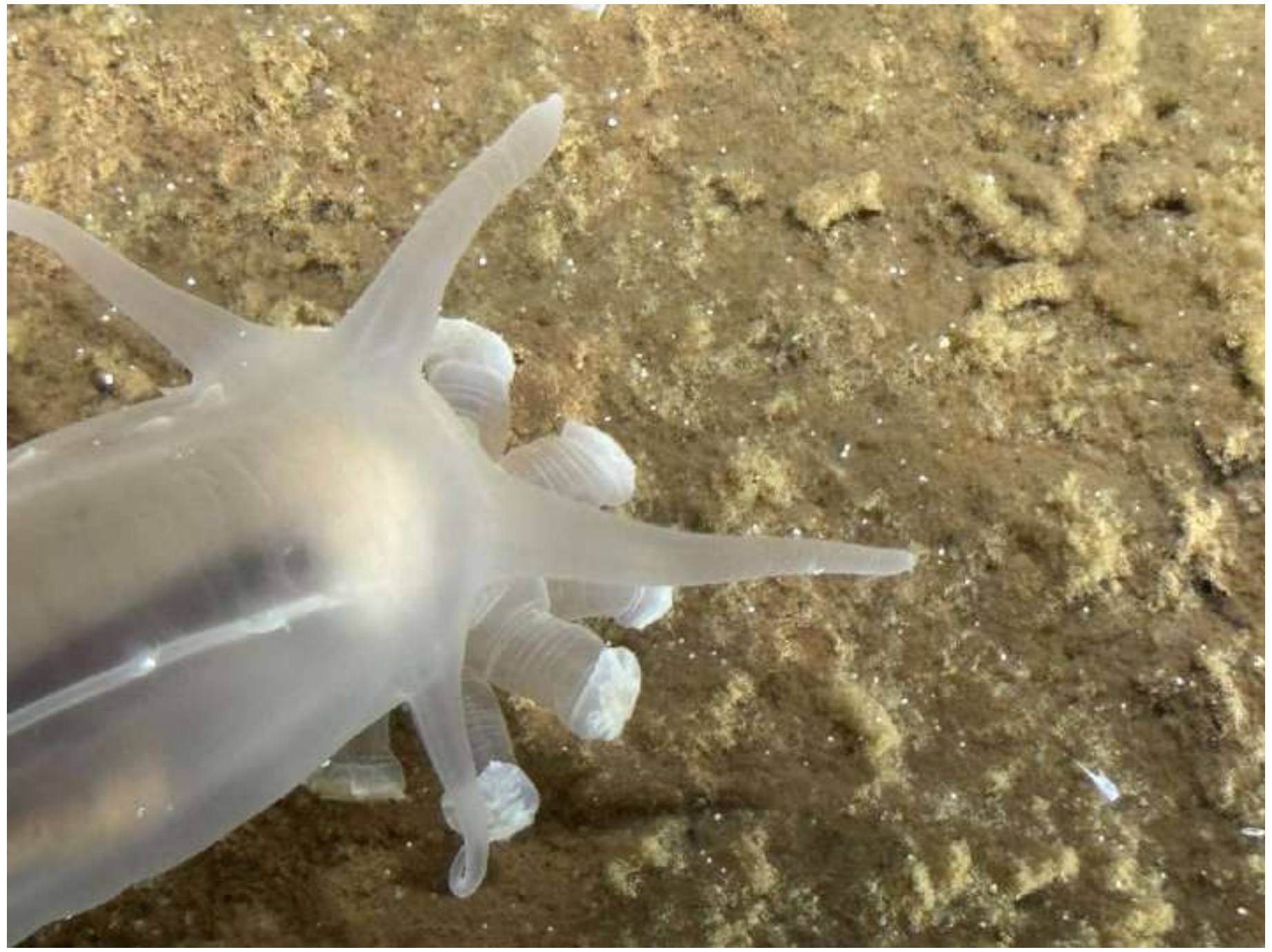

Skin is translucent/ white. Body is longer ovoid shape. 4 pairs of ventral tube feet occur along the sides, positioned towards the anus. 3 pairs of tentacles line the mouth. No brim on posterior end. Vulum build of two pairs of papillae.

NC2_BK3_7400_IMG_8929
NC2_BK3_7400_IMG_8942

Holothuroidea; Elasipodida; Elpidiidae ; *Elpidia; Elpidia* sp. indet. 1

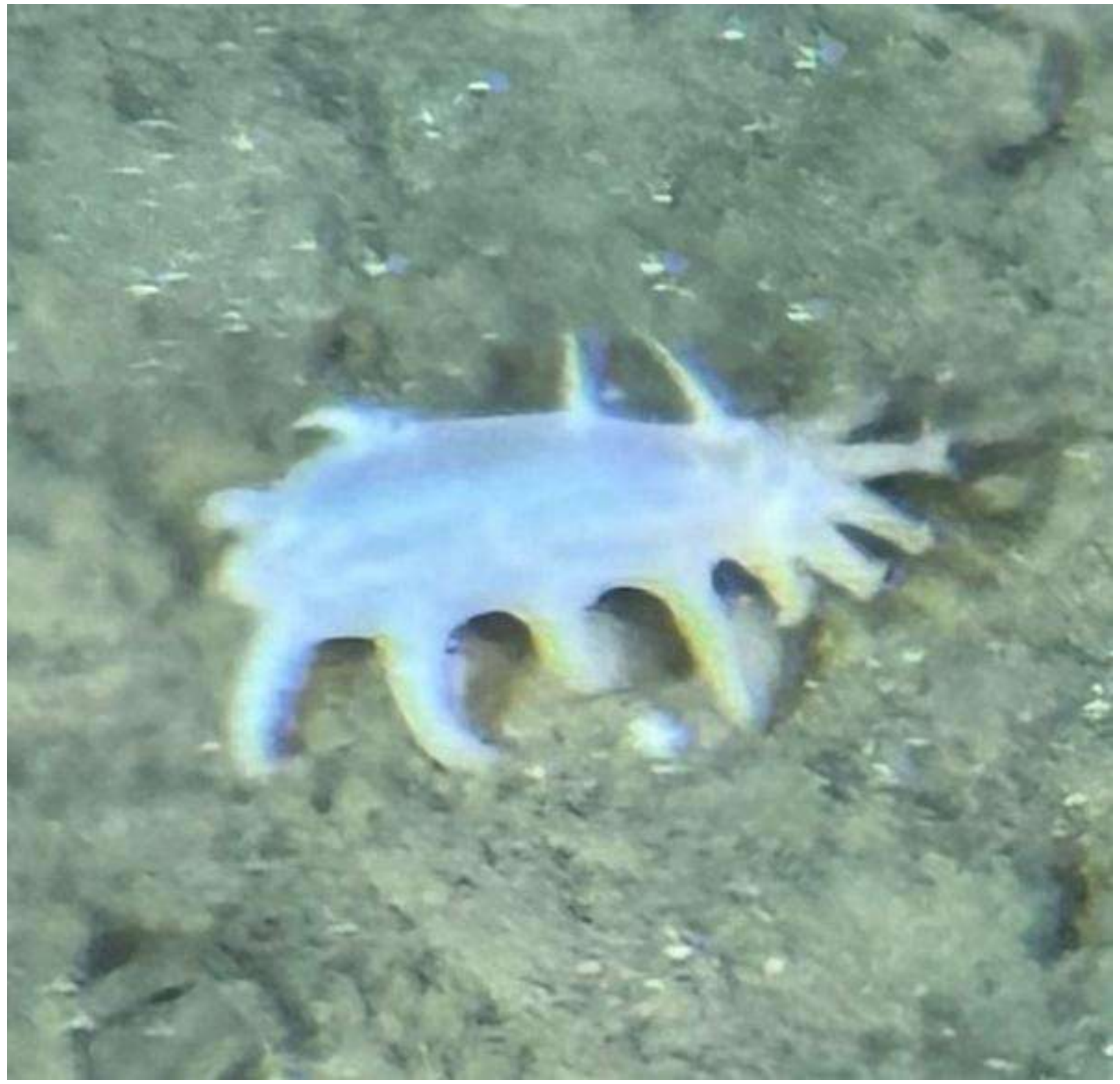
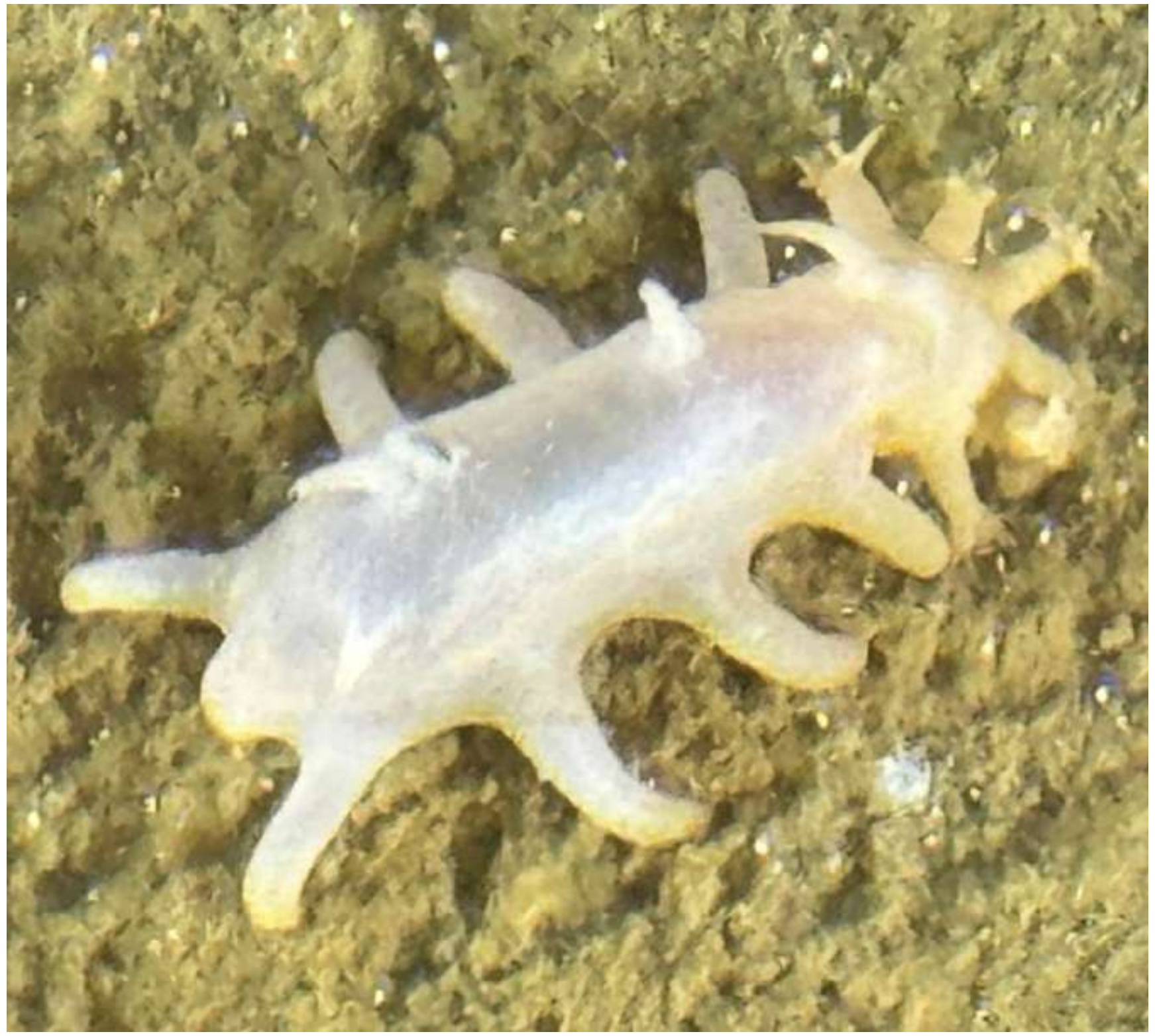

Skin is pale and translucent. Body is short and ovoid with 3 papillae along the length dorsal side. 6 pairs of tube feet with the posterior 4 pairs 2x as long as the anterior 2 pairs.

Holothuroidea; Elasipodida; Elpidiidae ; *Elpidia; Elpidia* sp. indet. 2

NC1_BK2_6700_IMG_4905

NC3_MA3_6100_NOR_0111

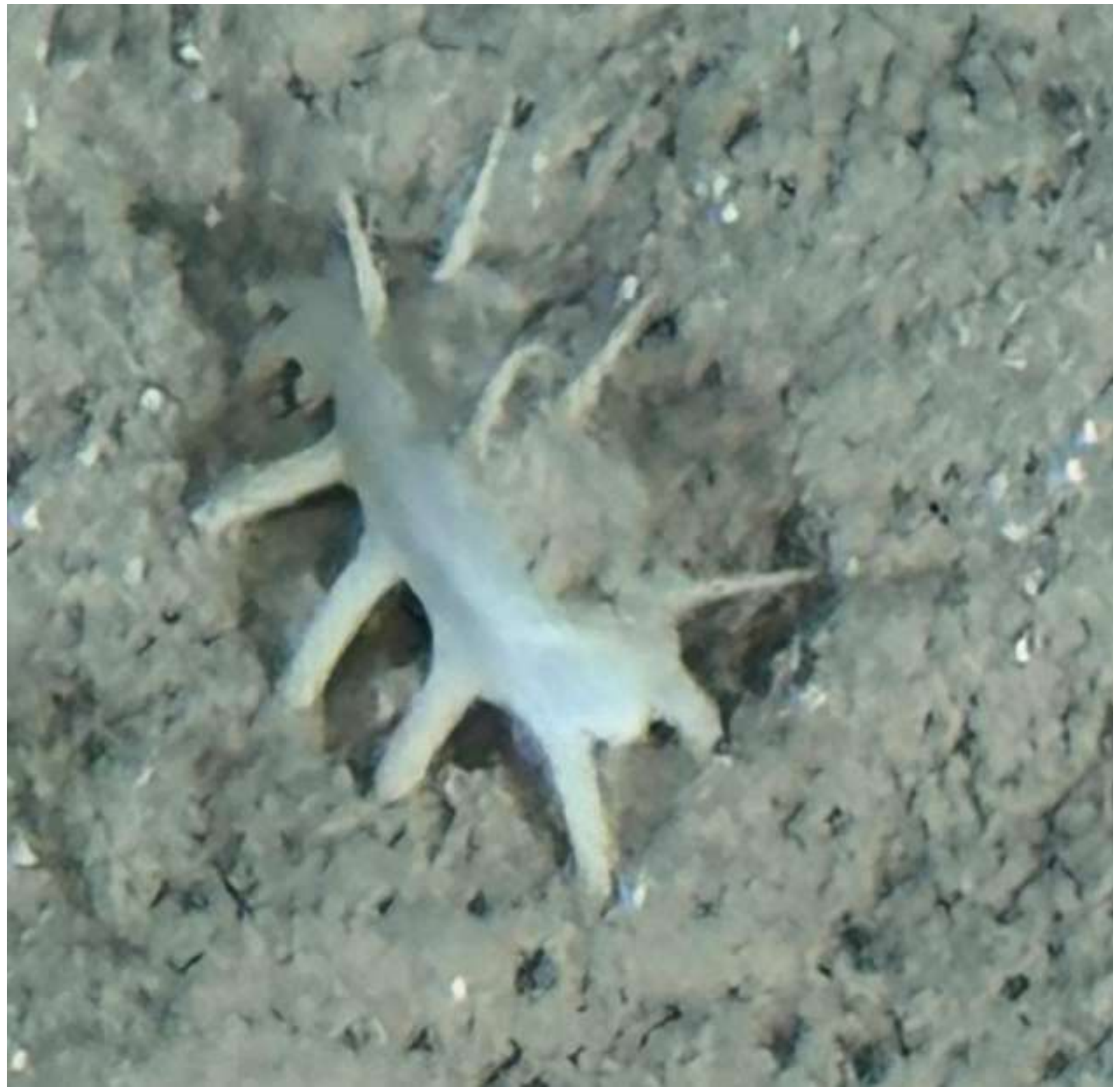

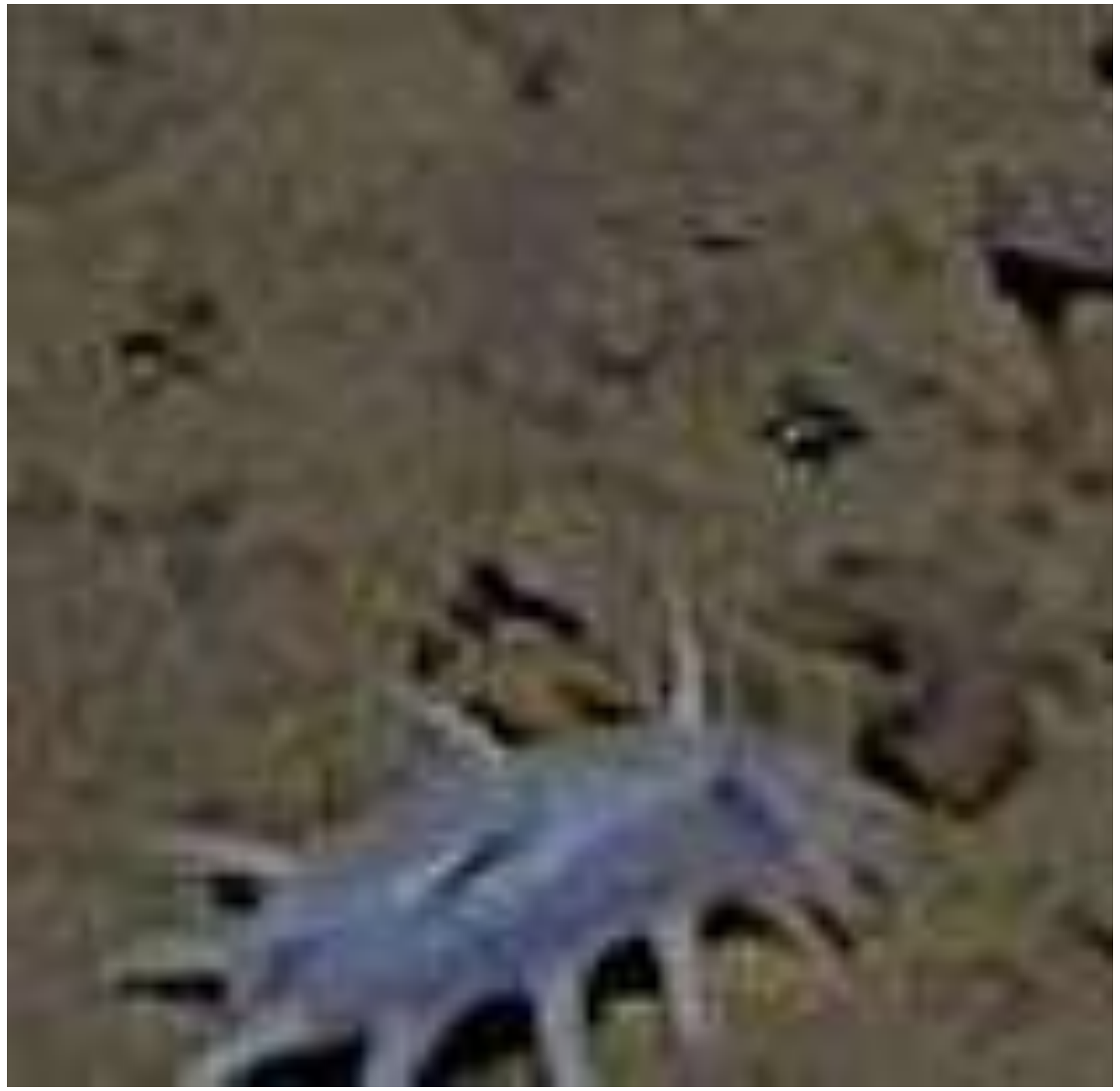

Skin is pale and opaque. Body is short and ovoid with arched dorsal side. 7 pairs of tube feet with the posterior 4 pairs 2x as long as the anterior 3 pairs. 3 pairs of long; conspicuous dorsal papillae present.

Holothuroidea; Elasipodida; Elpidiidae ; *Elpidia; Elpidia* sp. indet. 3

NC1_BK3_8000_IMG_6133

NC1_BK3_8000_NOR_0068

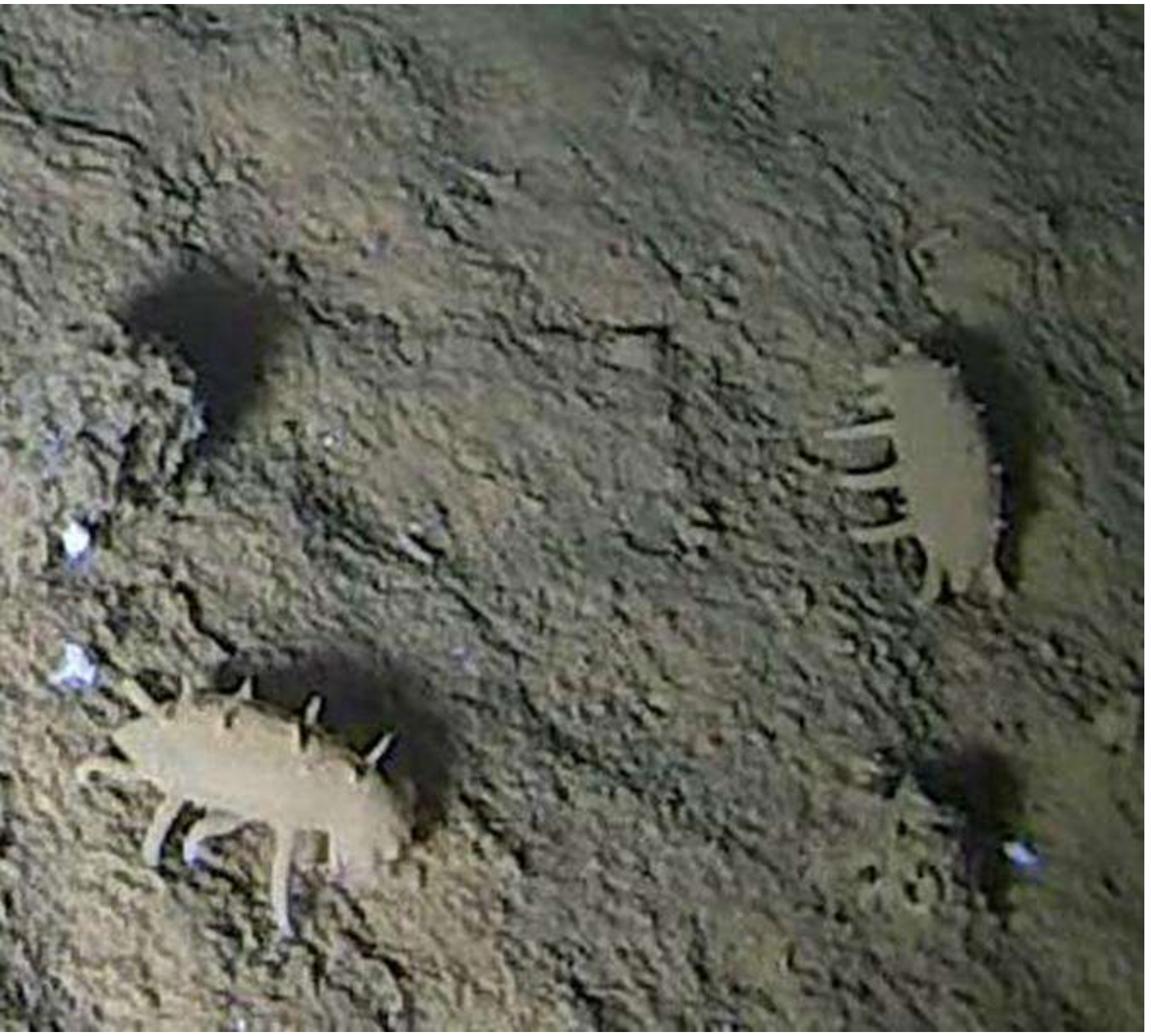

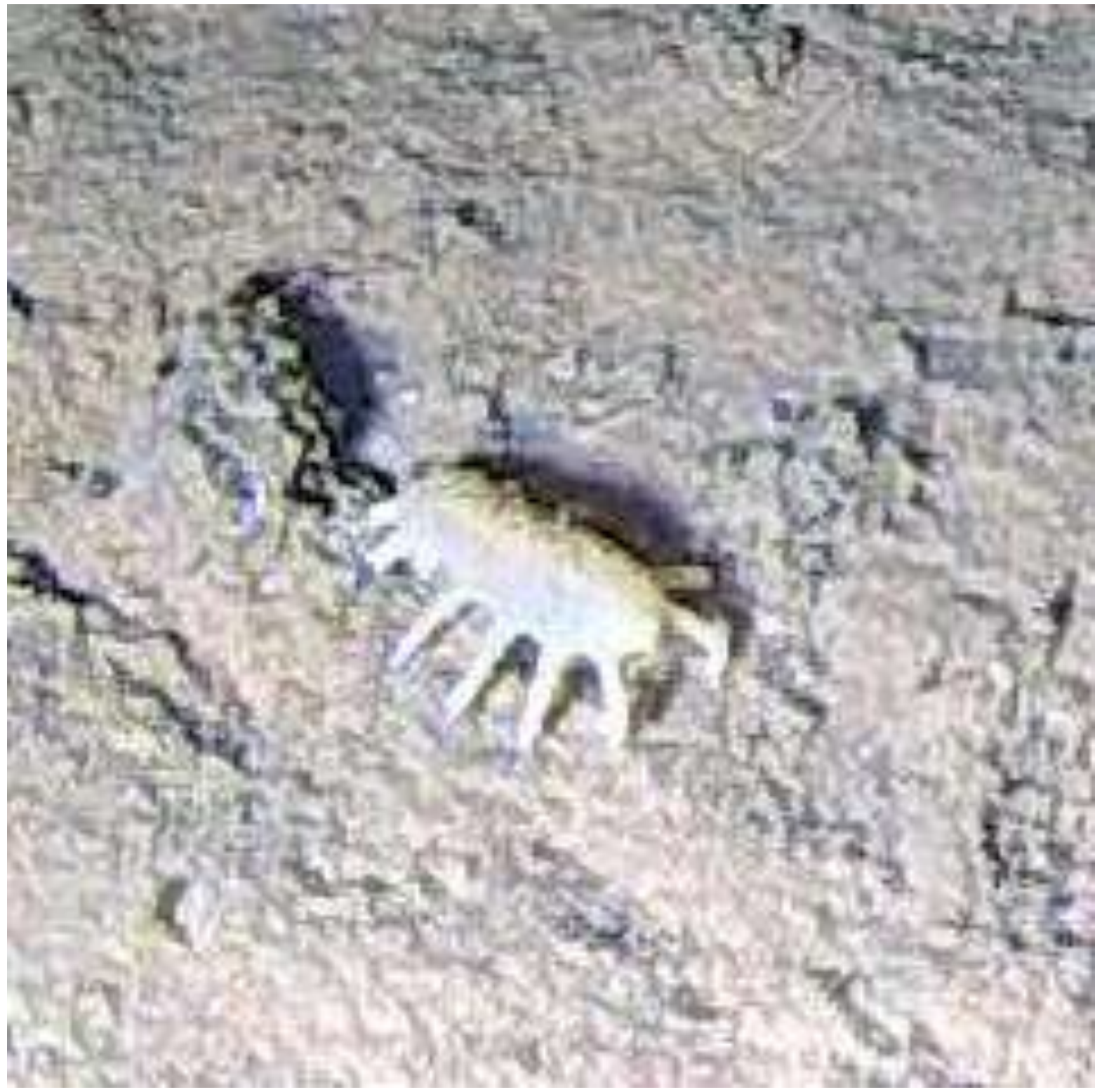

Skin is pale, translucent yellowish in colour. Skin may be covered in sediment. Body is elongate and ovoid with. 4 pairs of short, spike-like papillae observed on the arched dorsal side. 4 pairs of tube feet equal in length.

Holothuroidea; Elasipodida; Elpidiidae; *Elpidia; Elpidia* sp. indet. 4

NC1_BK3_8000_NOR_0079
NC1_BK3_8000_IMG_5953

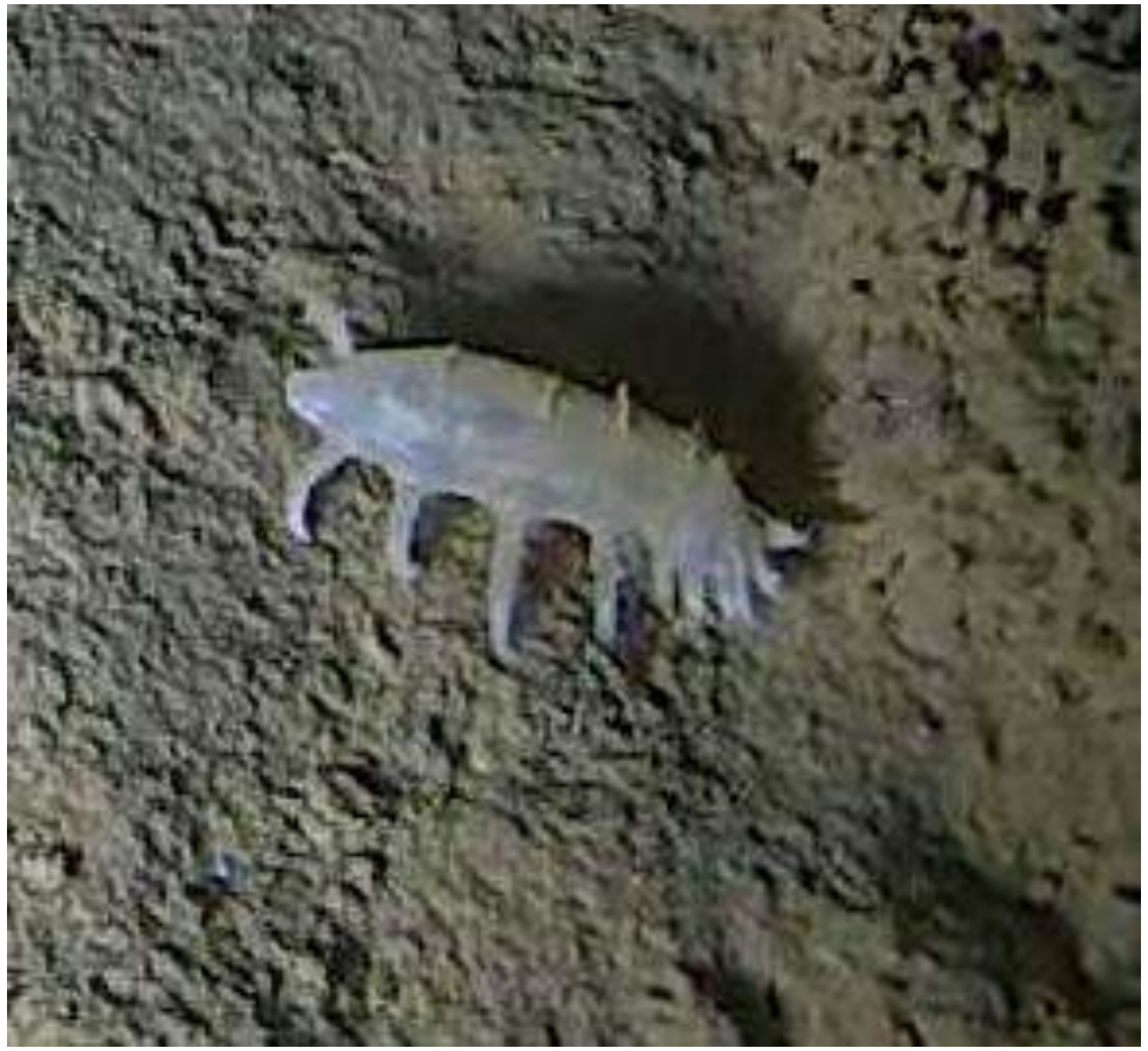

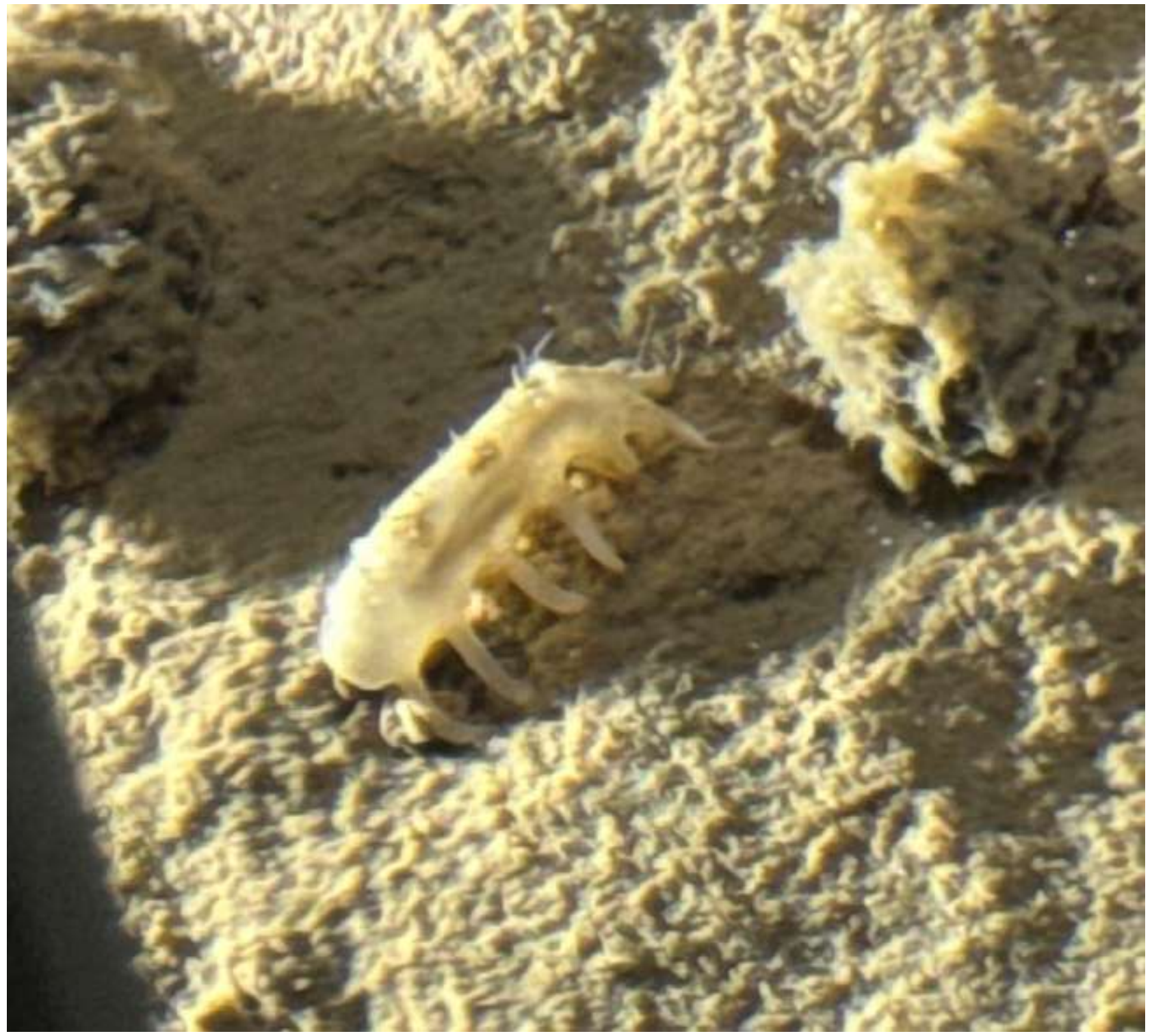

Skin is pale and translucent. Body is ovoid with a single row of 4 papillae/nubs running along arched dorsal side. 4 pairs of long tube feet equal in length.

Holothuroidea; Elasipodida; Elpidiidae; *Elpidia; Elpidia* sp. indet. 5

NC1_BK3_8000_NOR_66
NC1_BK3_8000_IMG_5953

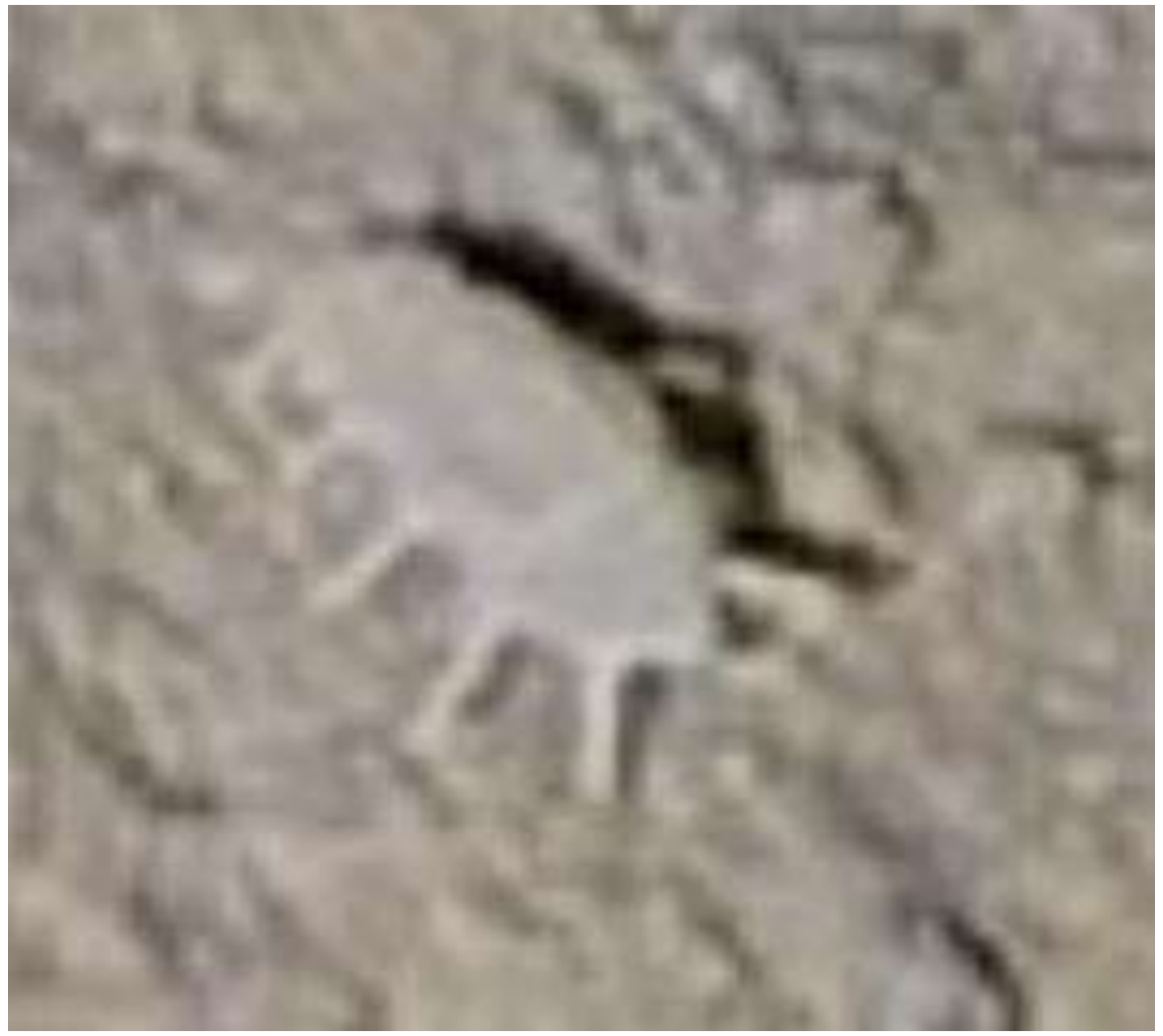

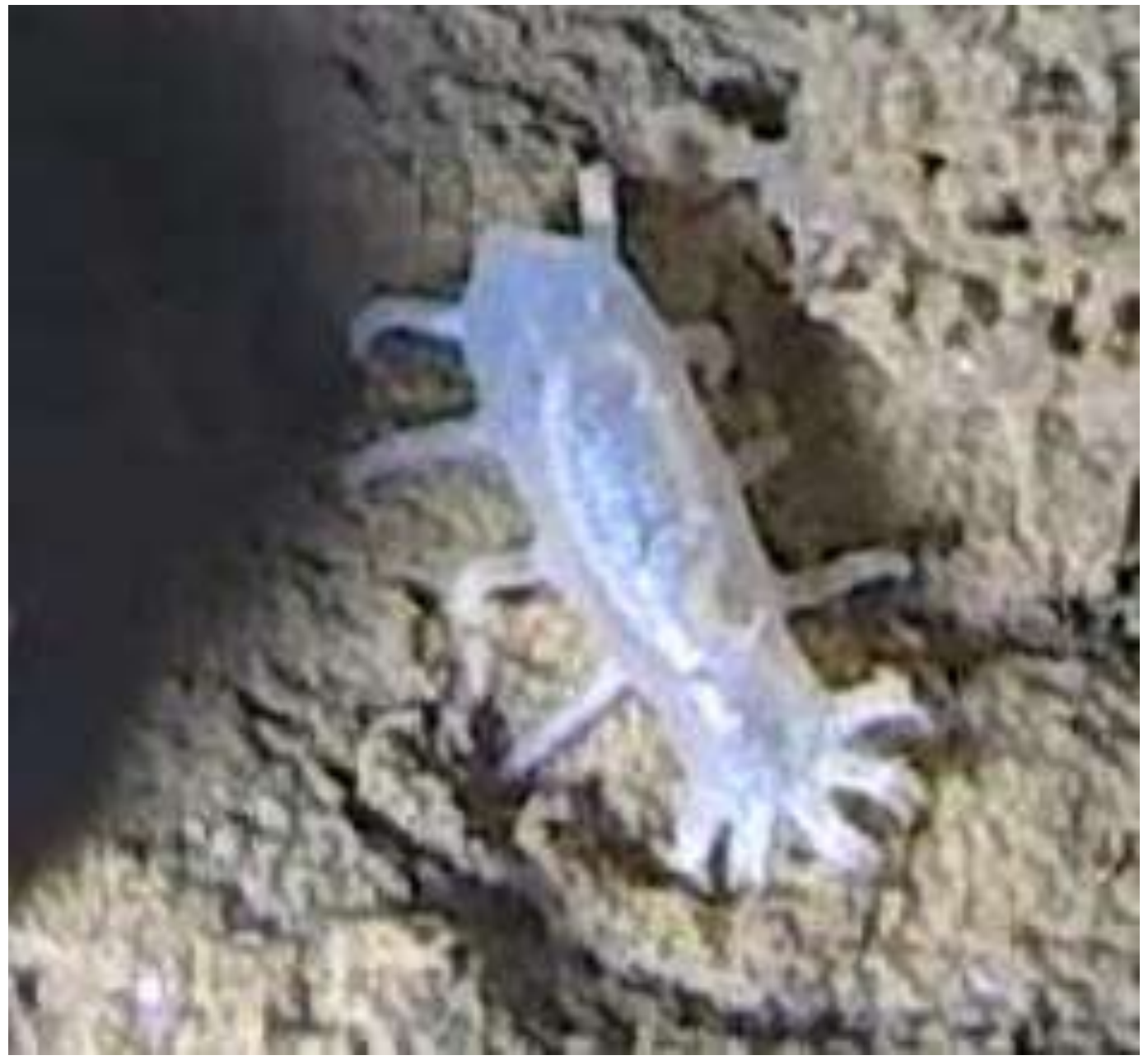

Skin is pale and translucent. Body is ovoid with significantly reduced or without observable papillae. 4 pairs of long tube feet equal in length.

Holothuroidea; Elasipodida; Pelagothuriidae; *Enypniastes; Enypniastes eximia*

NC3_OM1_5600_NOR_0201

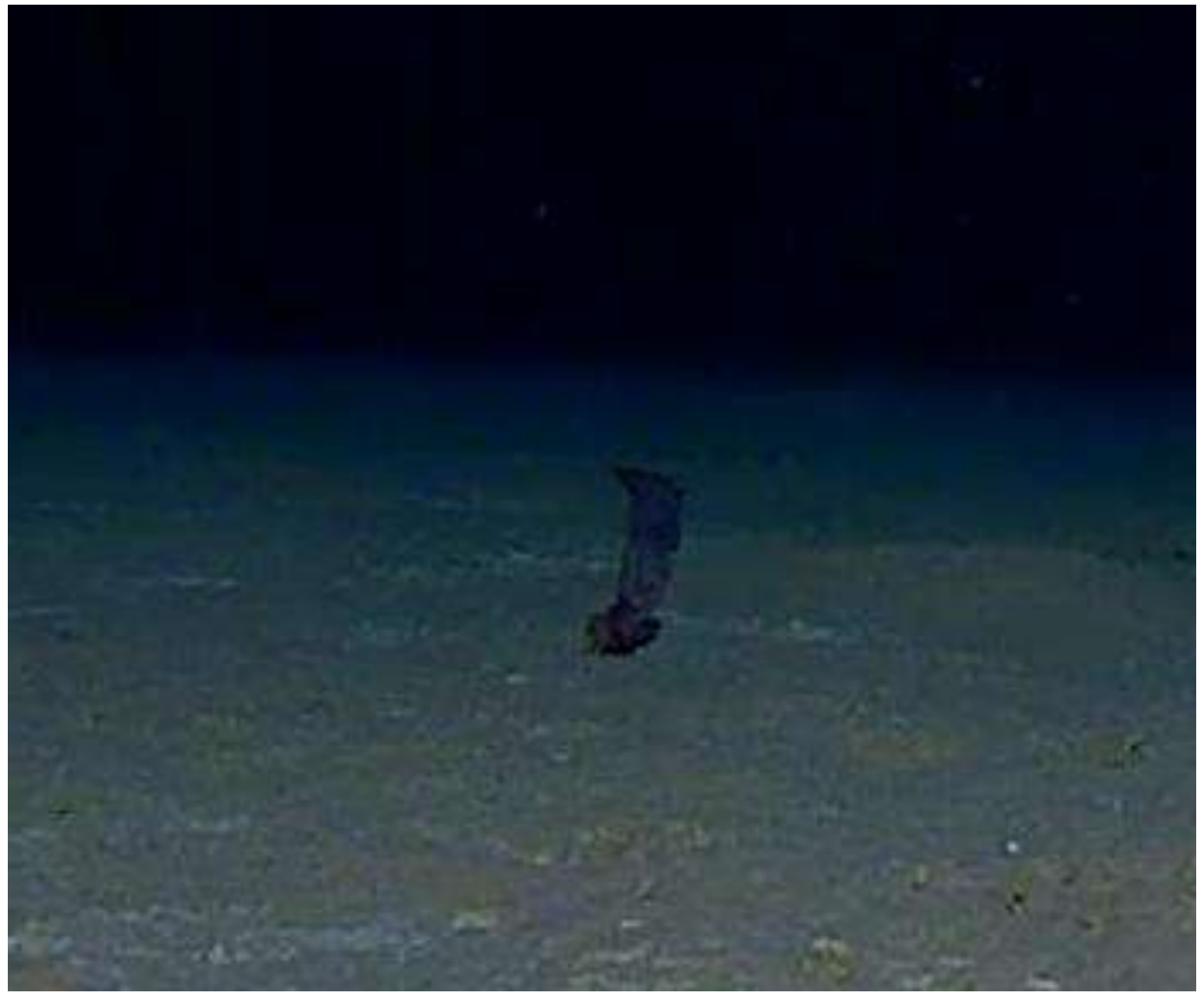

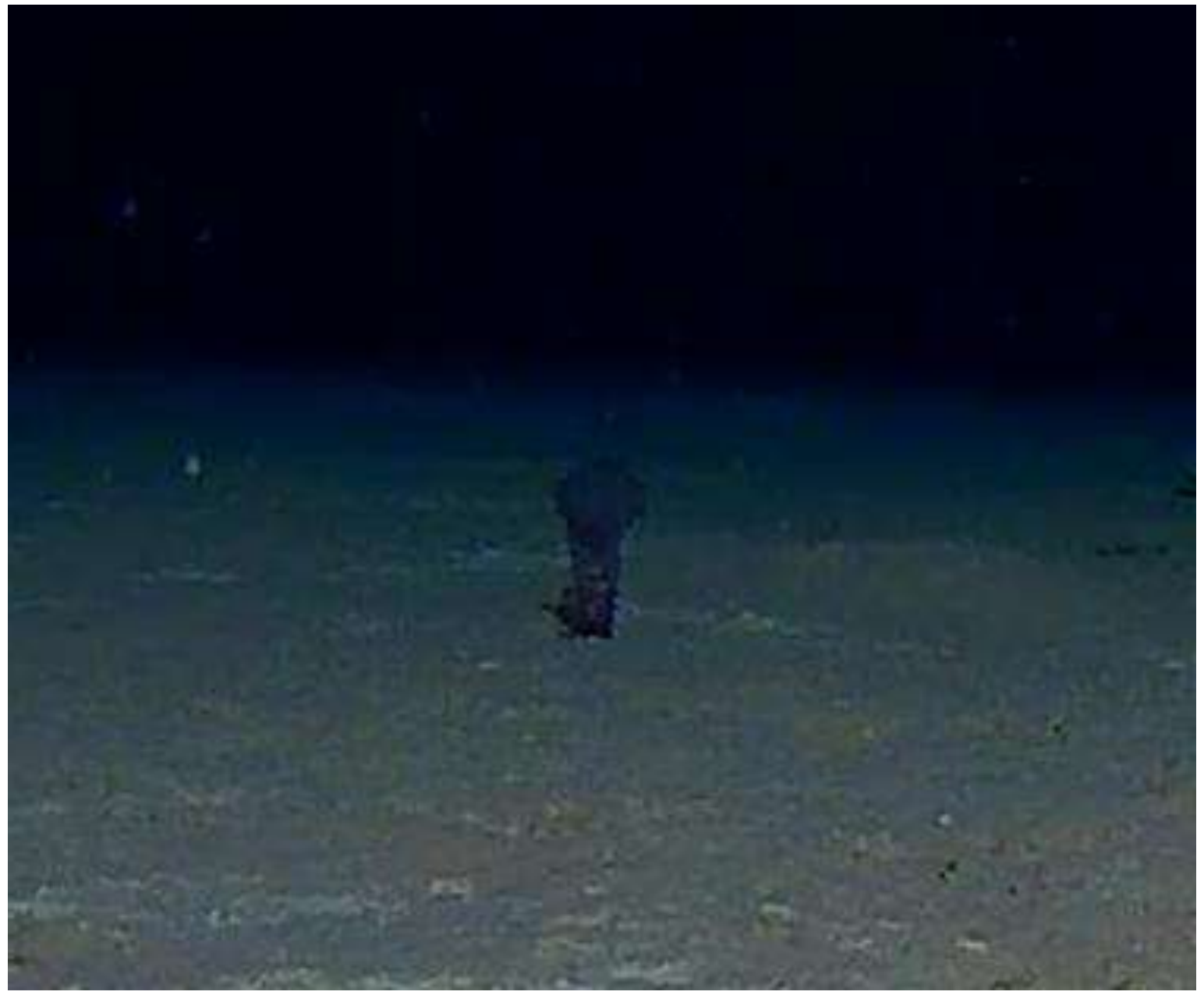

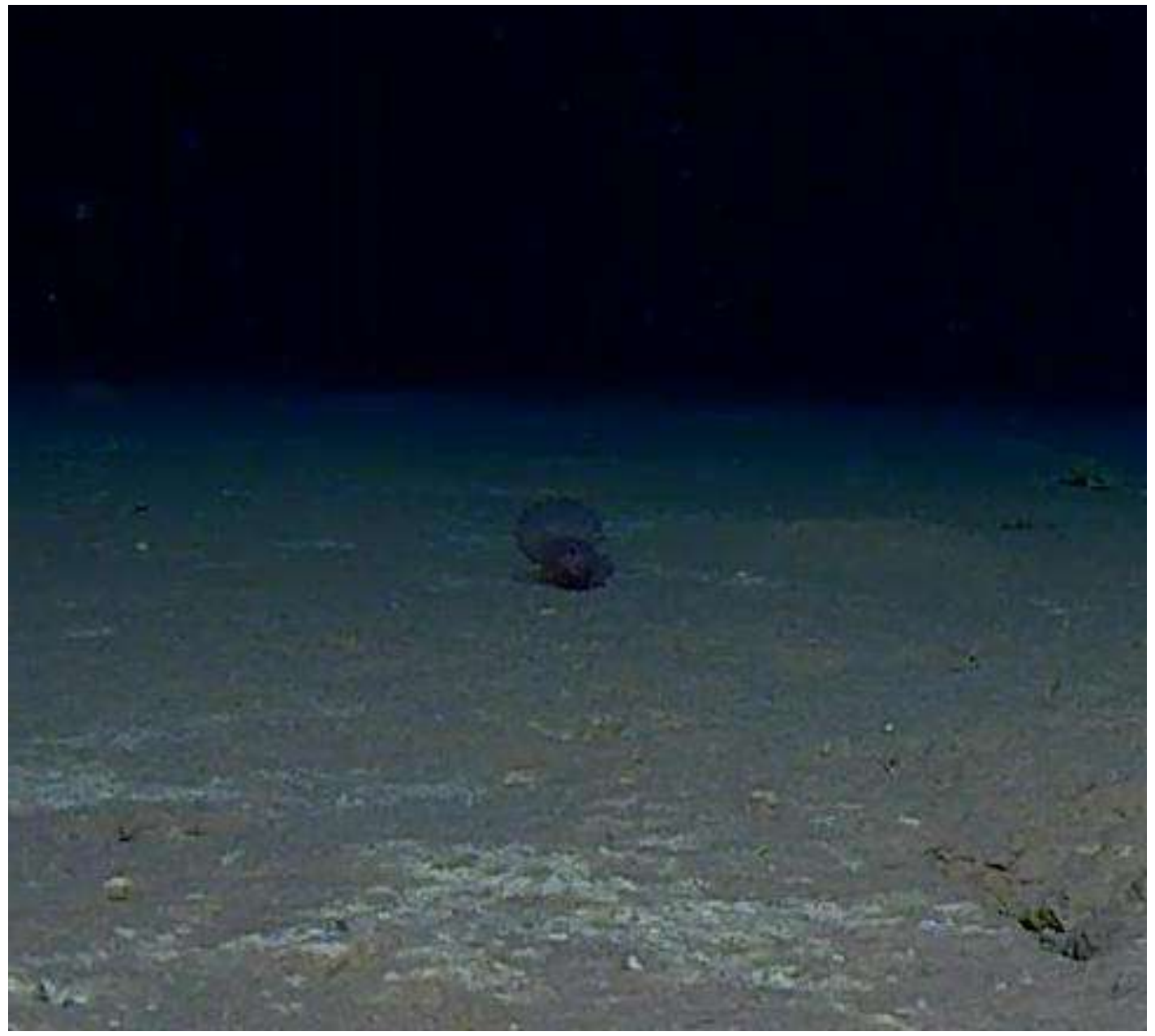

Skin is translucent/ dark purple. Body is longer ovoid shape. 4 pairs of ventral tube feet occur along the sides. multiple pairs of tentacles line the mouth. Brim is large on the anterior end.

Holothuroidea; Elasipodida; Psychropotidae ; *Benthodytes; Benthodytes* sp. indet. 1

NC2_BK5_4600_IMG_9999

NC4_BK4_4700_NOR_0072

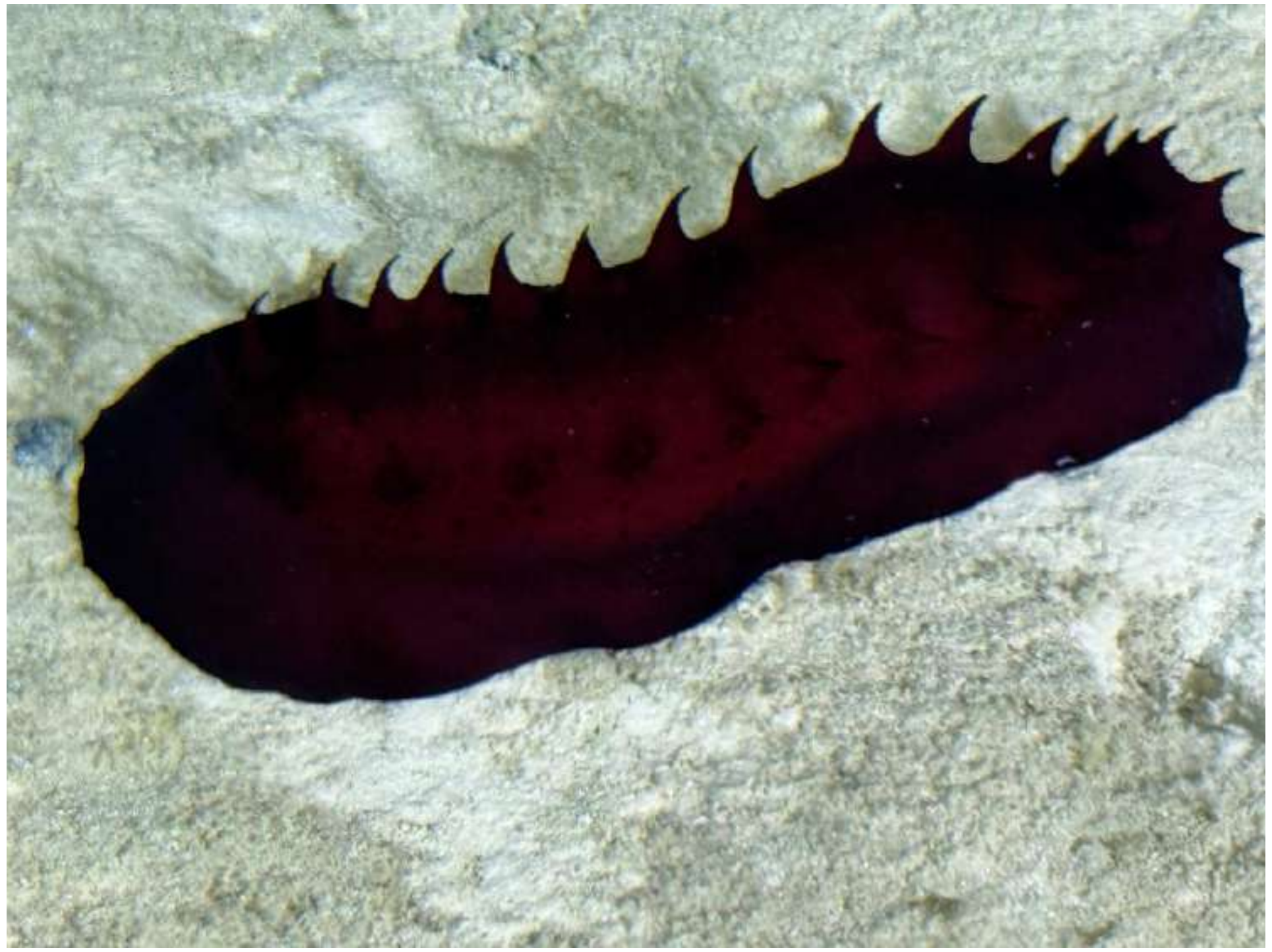

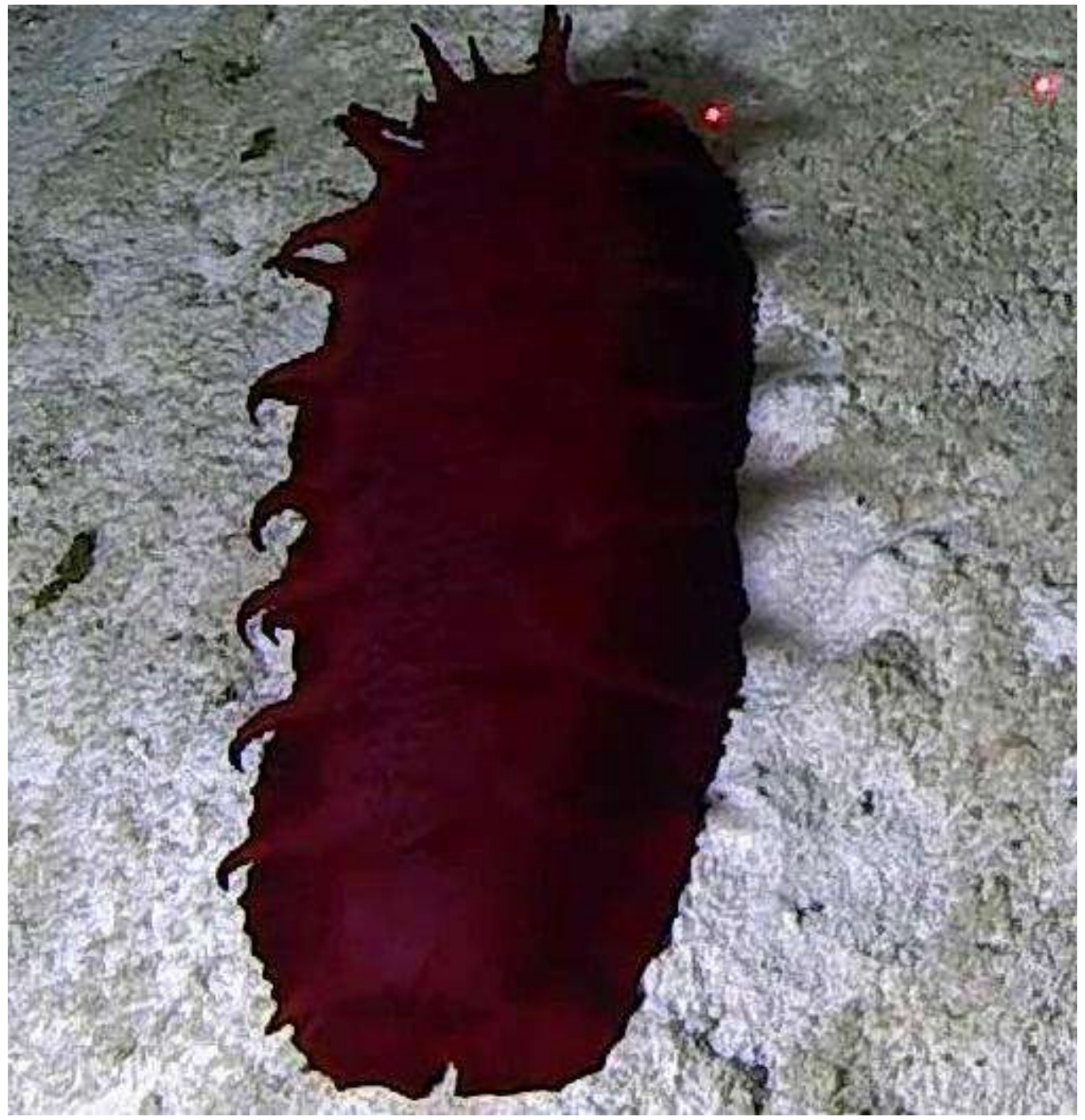

Colour is deep; dark red with flattened ventral side and arched dorsal side. Brim is well developed and extends around the body length. Roughly 10 pairs of thick; conspicuous dorsal papillae in two rows.

Holothuroidea; Elasipodida; Psychropotidae ; *Benthodytes; Benthodytes* sp. indet. 2

NC2_BK5_4600_IMG_0541
NC2_BK5_4600_IMG_9915
NC2_BK5_4600_IMG_9903

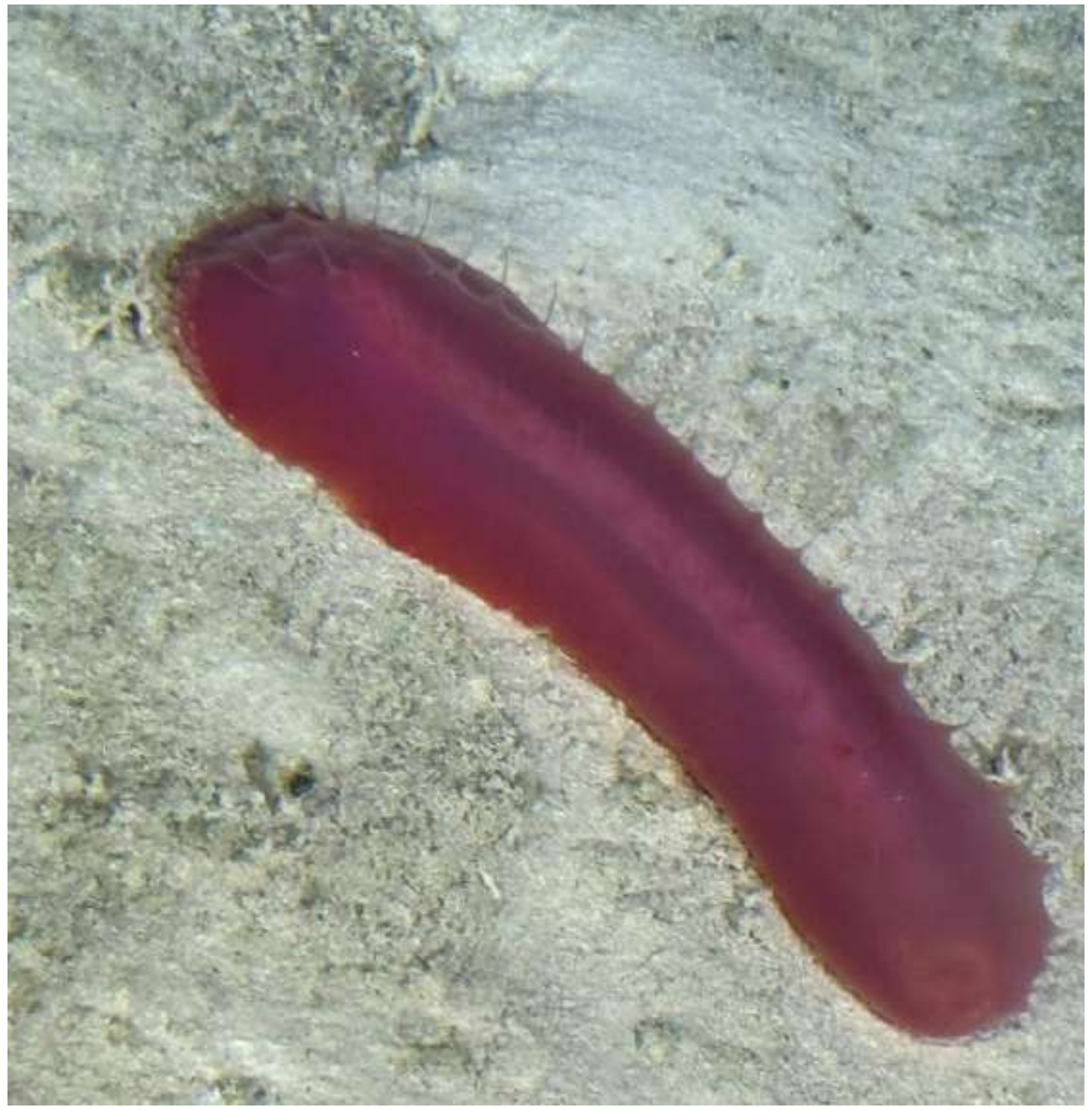

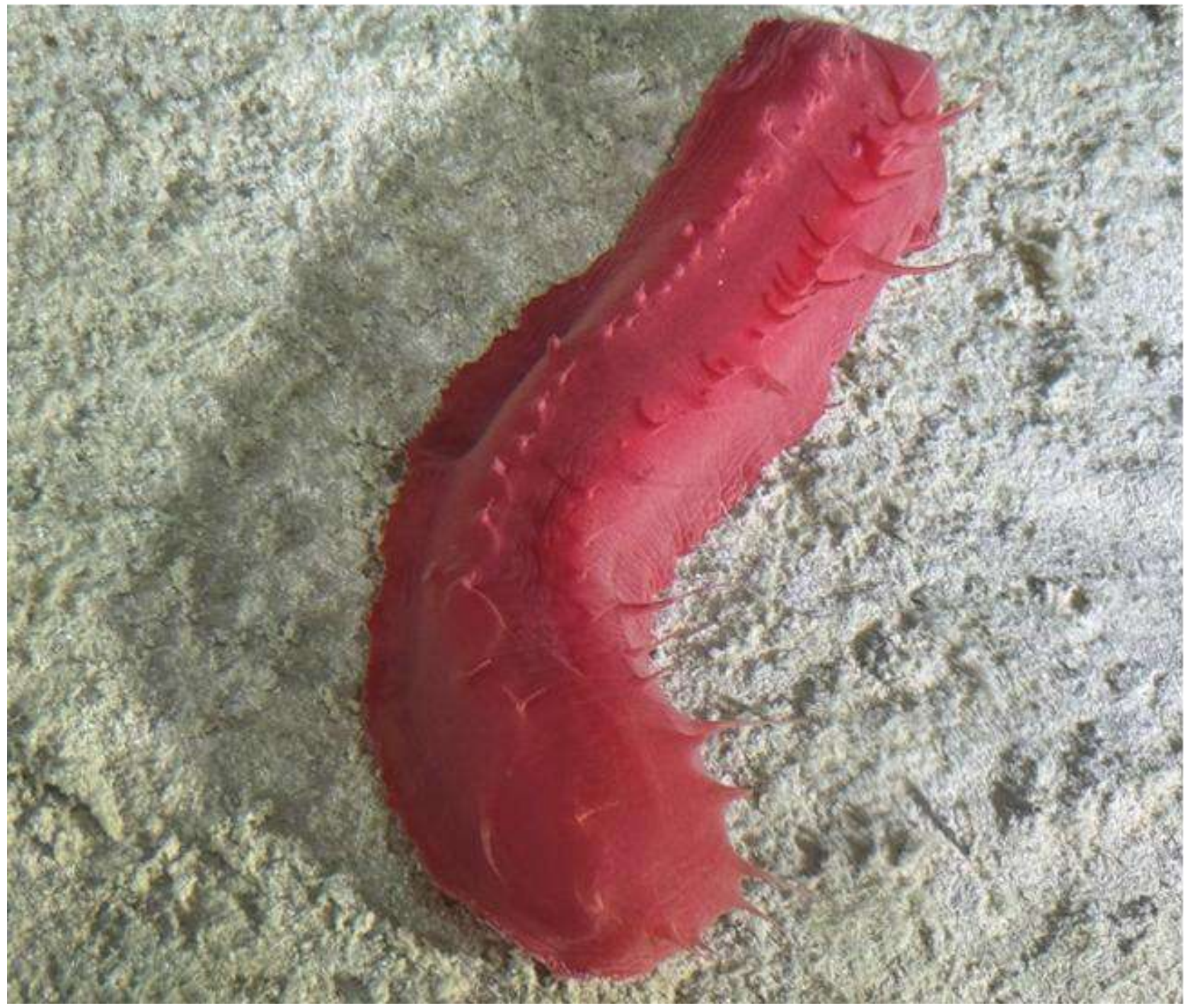

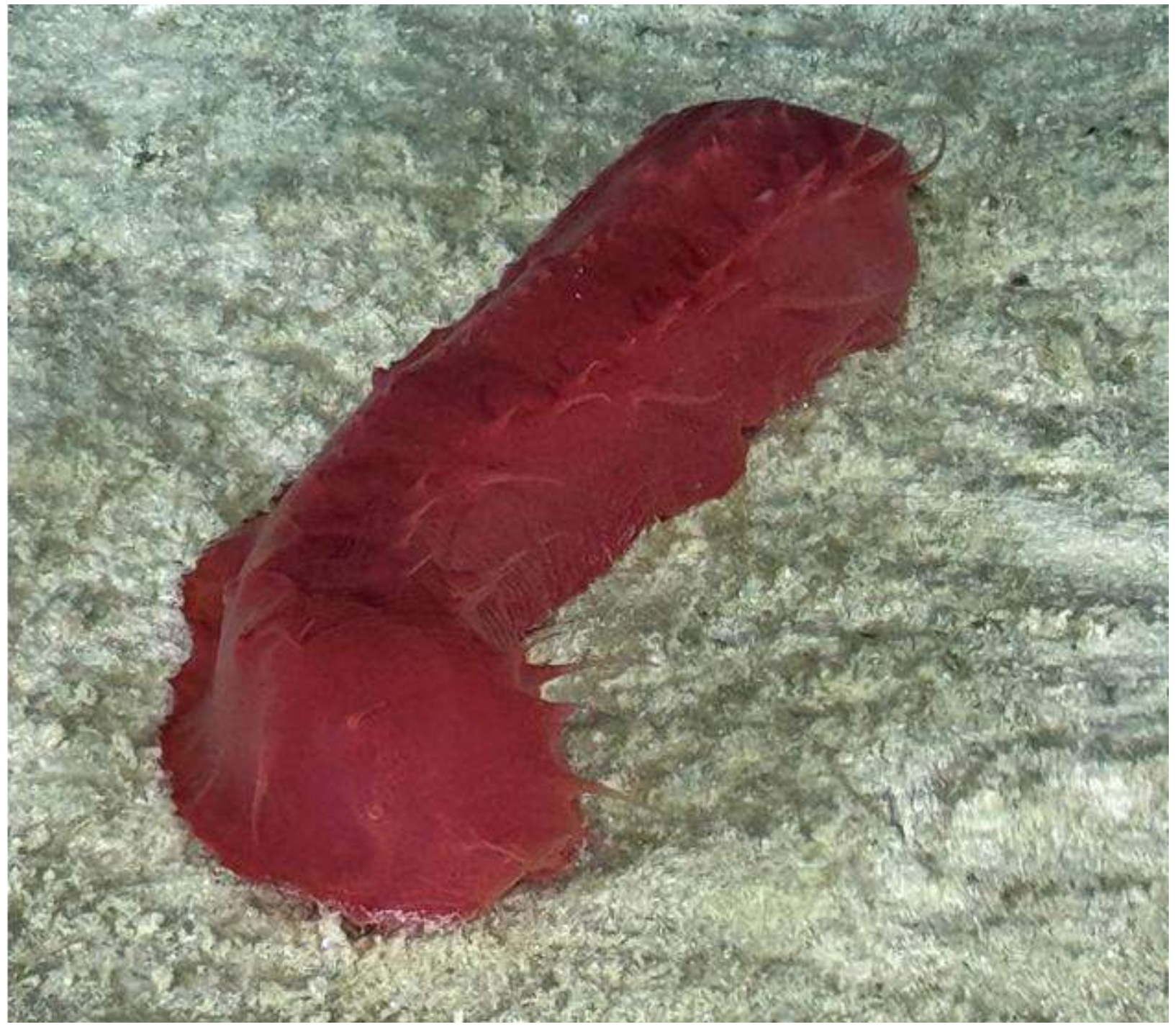

Colour is pinkish-red with flattened ventral side and arched dorsal side. Brim is less developed and extends to ¾ of body length. Roughly 20 pairs of thin; conspicuous dorsal papillae in two rows.

Holothuroidea; Holothuriida Psychropotidae ; *Benthodytes; Benthodytes* sp. indet. 3

NC1_BK1_6200_IMG_4407

NC1_BK1_6200_IMG_4409

NC3_BK2_6200_IMG_4412

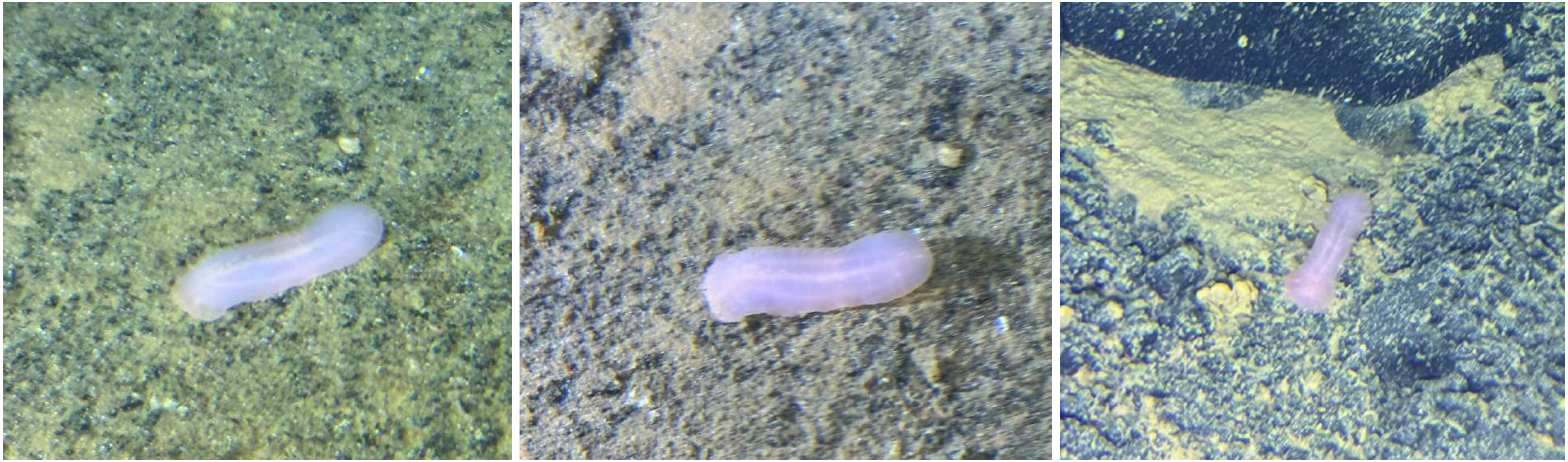

Translucent off-white/ pink colouration. Body is cylindrical with no apparent tube feet or dorsal papillae. Brim apparent on anterior end.

Holothuroidea; Holothuriida Psychropotidae ; *Benthodytes; Benthodytes* sp. indet. 4

NC4_BK1_5600__01_NOR_0070

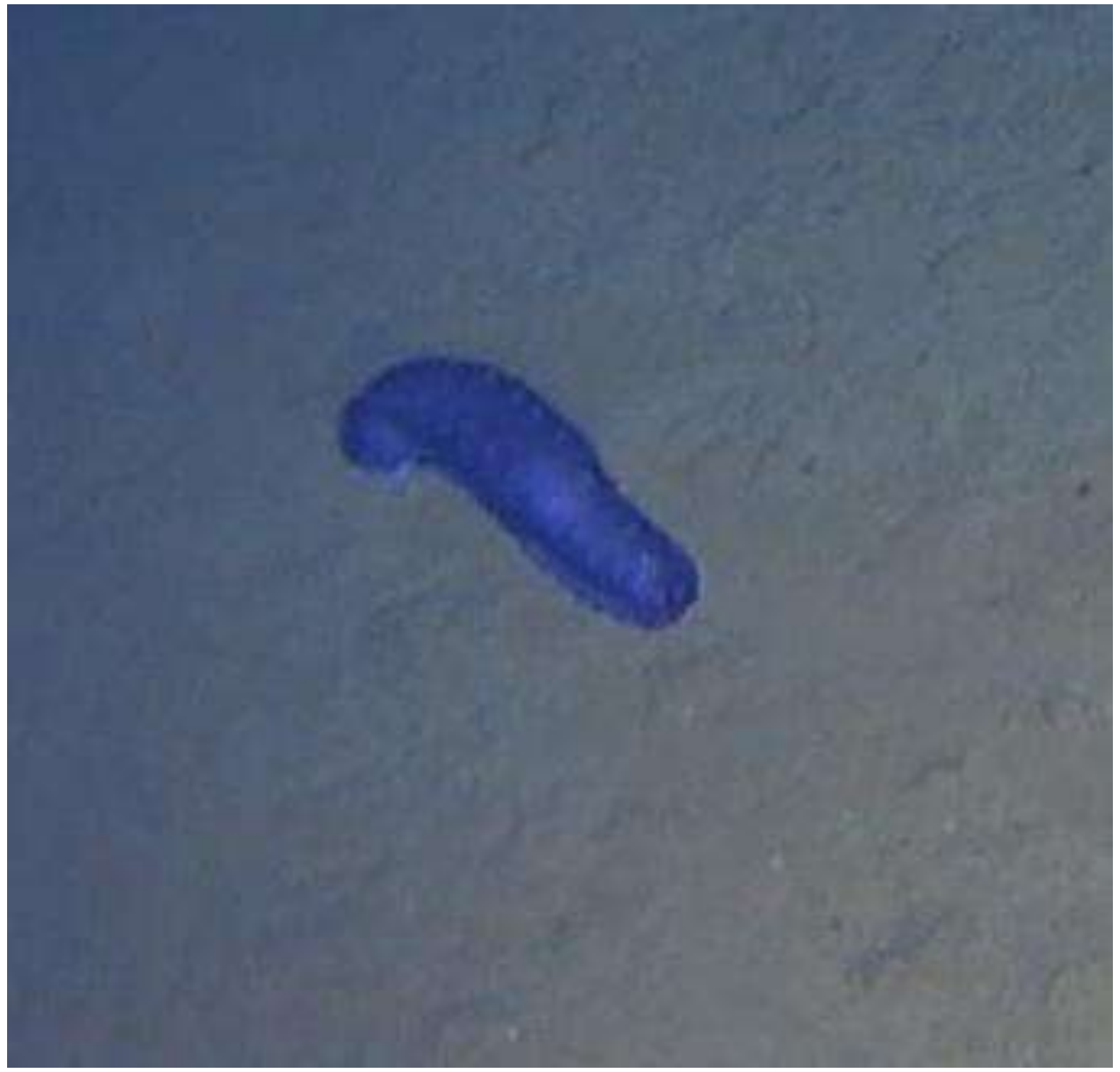

Skin deep purple in colour. Body is subcylindrical with distinguishable anterior lobe present. Two rows of short; blunt dorsal papillae. Anterior end has distinctive "head-like" oral disk.

Holothuroidea; Holothuriida
Psychropotidae ;
Psychoprotidae gen. indet. 1

NC1_BK2_6700_IMG_4939

NC1_BK2_6700_IMG_4941

NC2_BK4_5300_NOR_0063

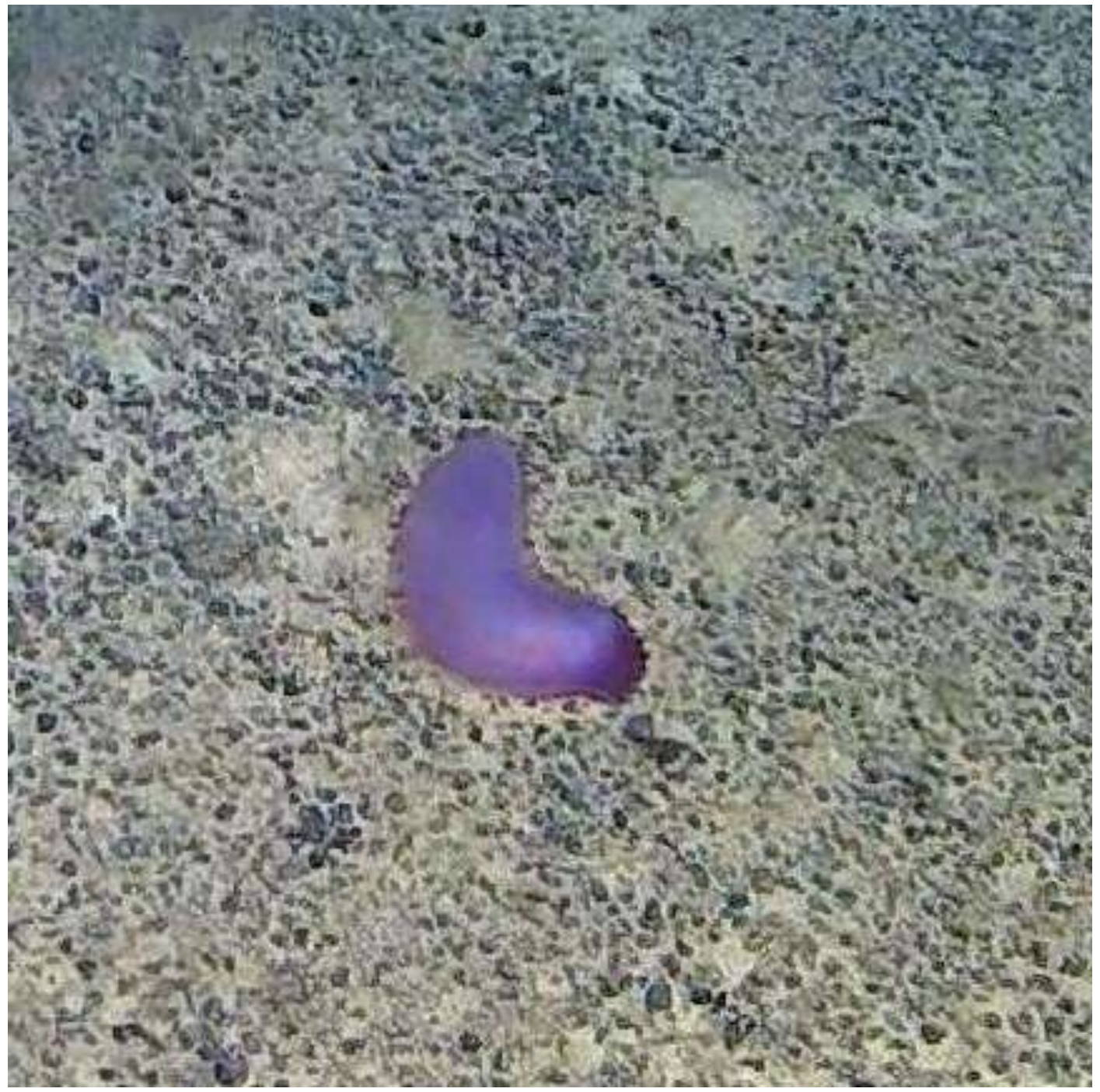

Body is whitish purple with deep lavender purple colouration on anterior brim. Skin has wrinkled texture. Ventral side is flattened while dorsal side is arched. Tube feet like tentacles near the mouth present underneath the anterior brim.

Holothuroidea
Elasipodida; Laetmogonidae; Laetmogonidae gen. indet. 1

NC1_BK1_6200_IMG_4821
NC3_BK2_6000_IMG_2013
NC3_BK2_6000_IMG_2008

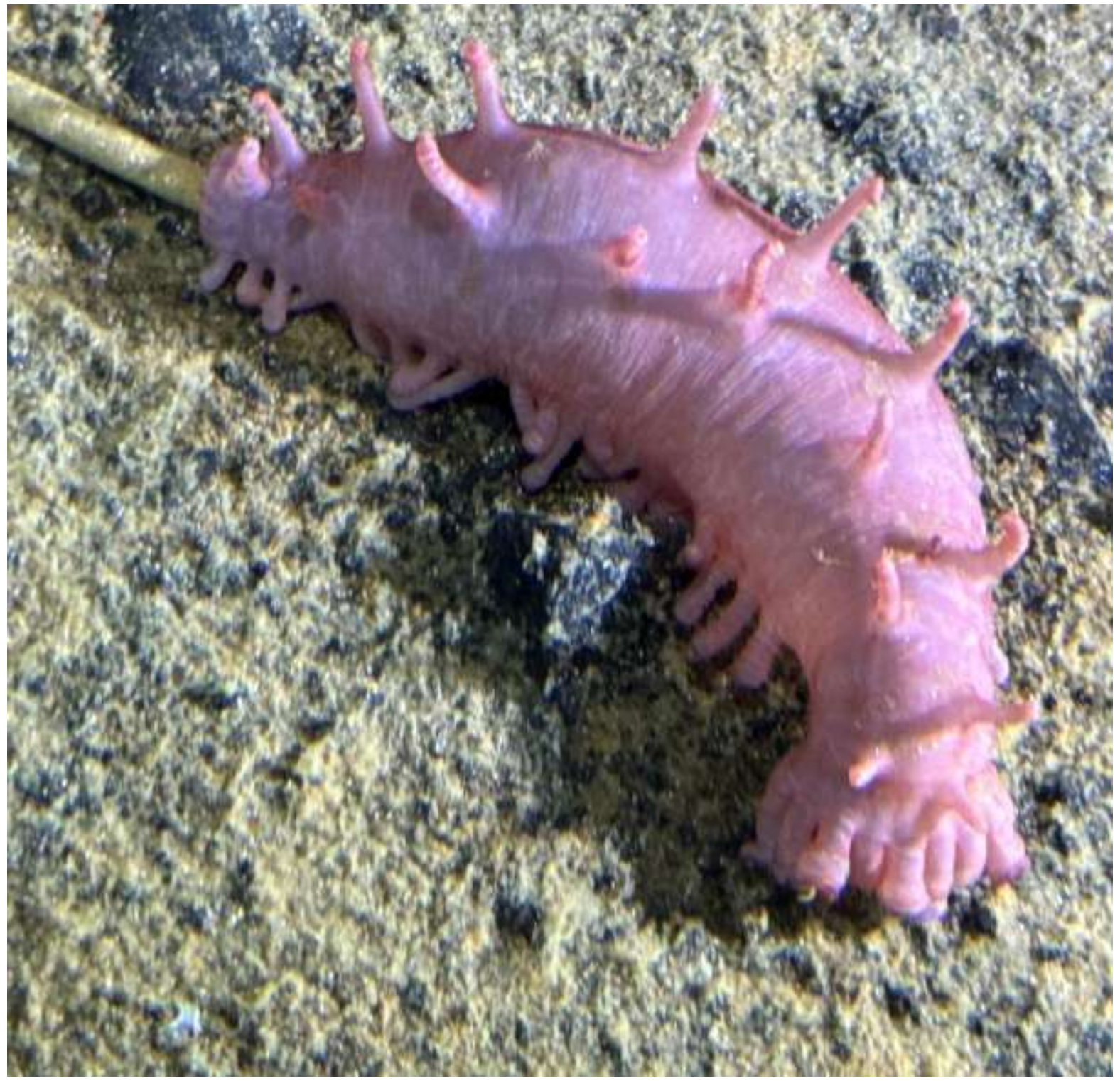
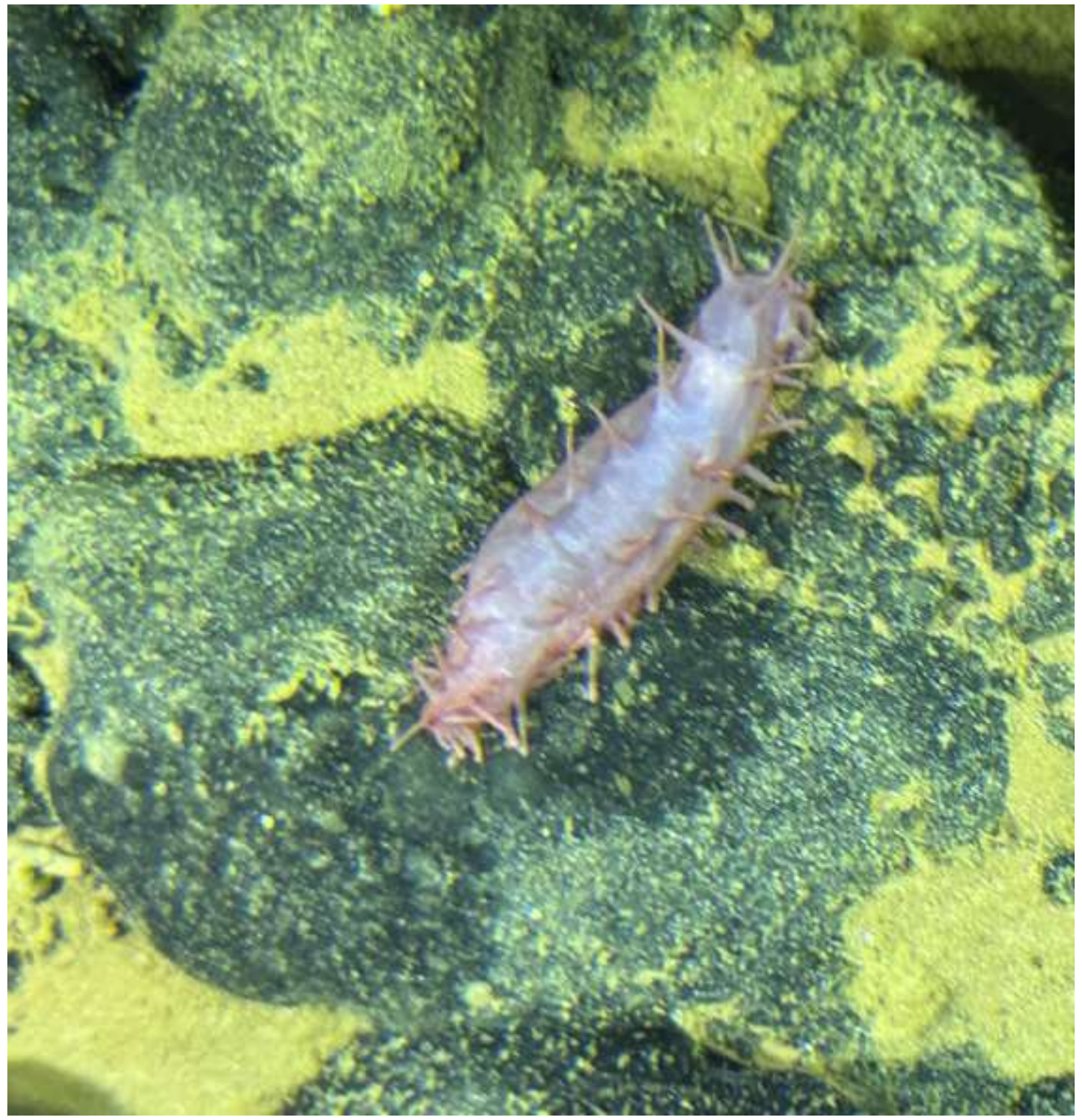
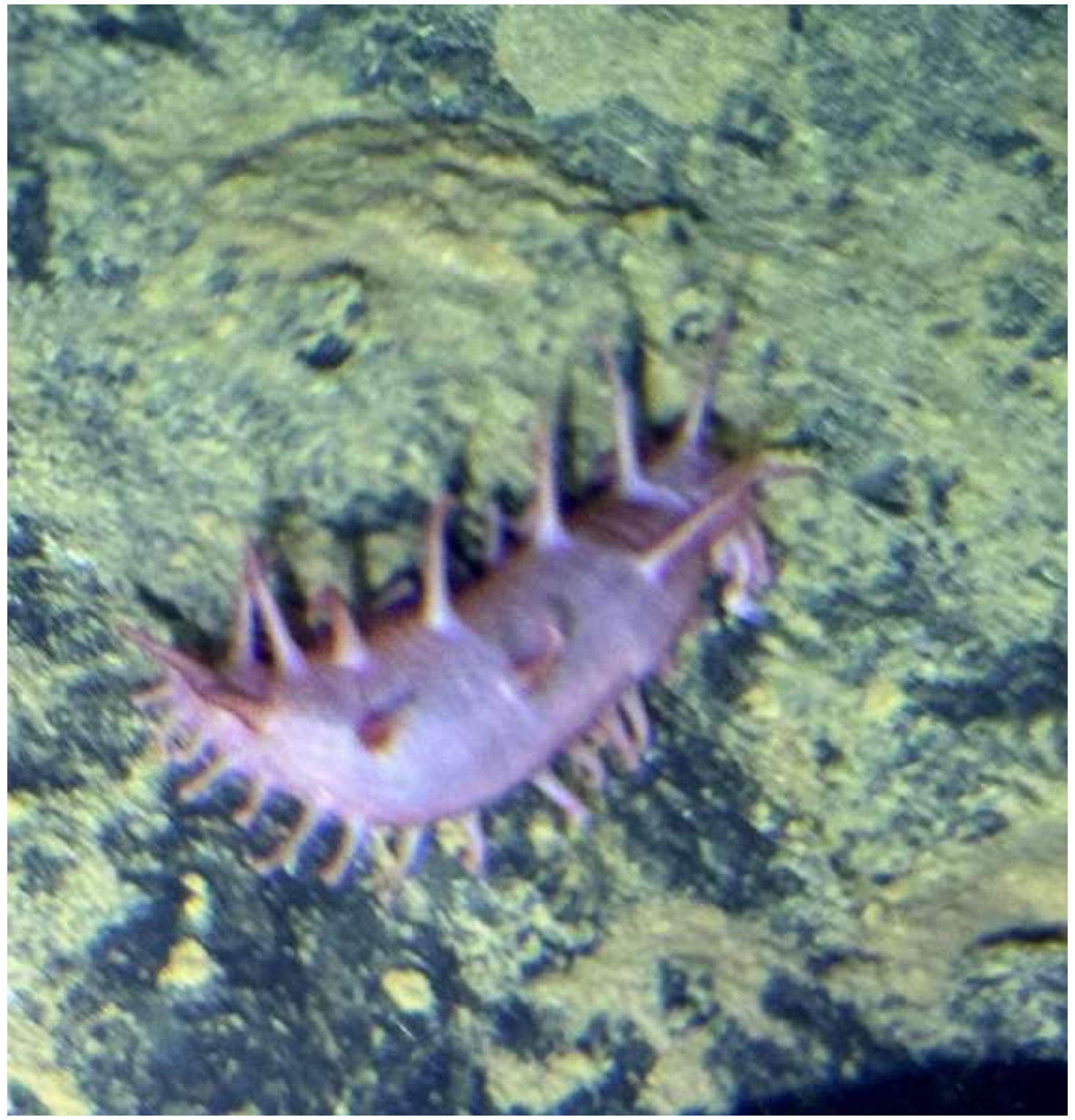

Pinkish purple to deep purple colour. Body is cylindrical with 8 pairs of short dorsal papillae arranged in two rows running parallel along the body to the posterior end. Multiple short ventral tube feet observed from the anterior end to the posterior end. Anterior end has distinctive "head-like" oral disk.

Holothuroidea; Elasipodida; Laetmogonidae; *Psychronaetes*
Psychronaetes hanseni

NC4_BK4_4700_IMG_6049

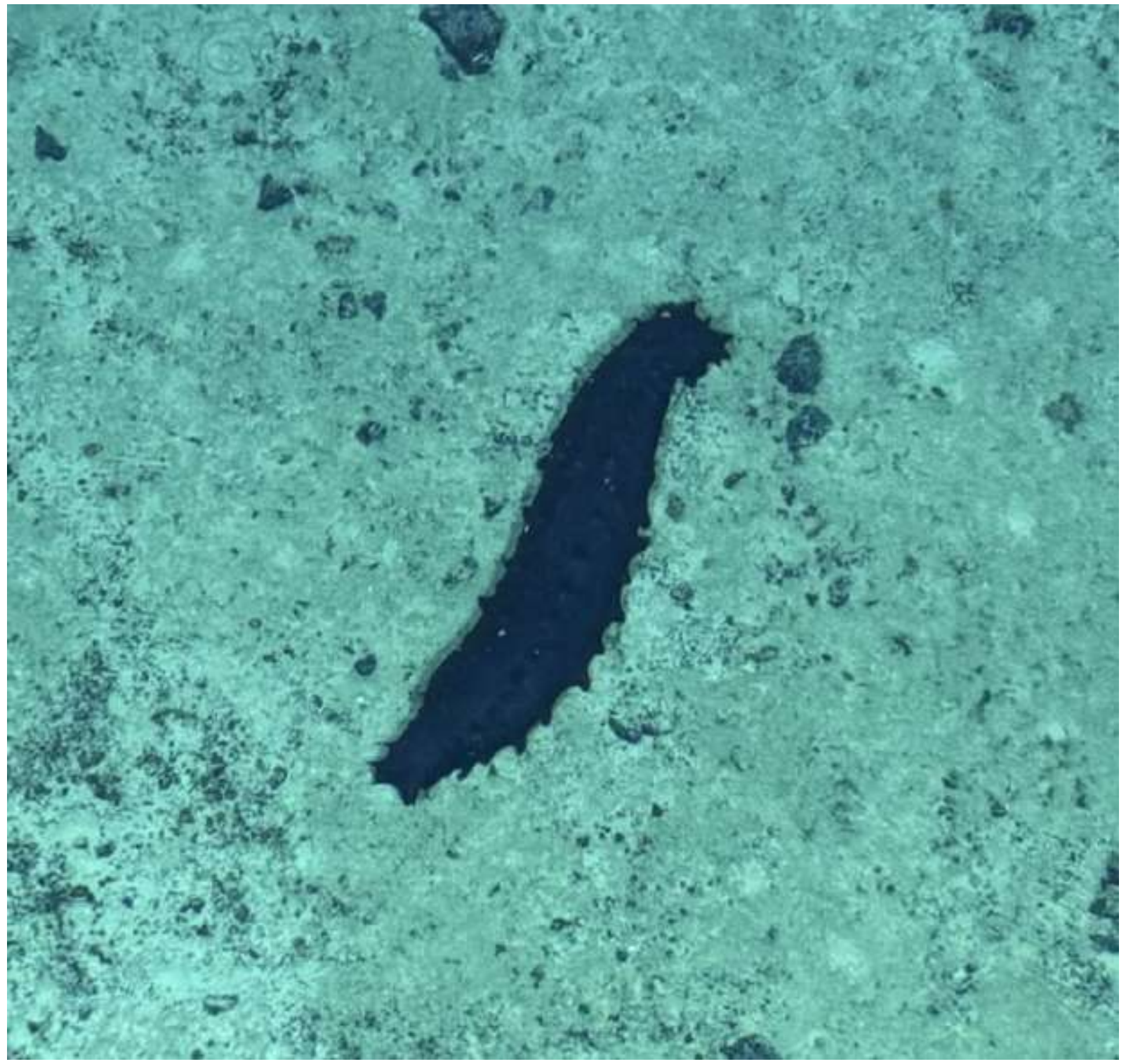

Dark-blue colour. Body is cylindrical with dorsal papillae that are short and thick and are arranged in three rows of papillae running parallel along the body that converge at the anterior/ posterior end. Anterior end has distinctive "head-like" oral disk.

Holothuroidea; Elasipodida; Laetmogonidae ; *Psychronaetes; Psychronaetes* sp. indet.1

NC4_BK4_4700_NOR_0094

NC4_BK4_4700_IMG_6183

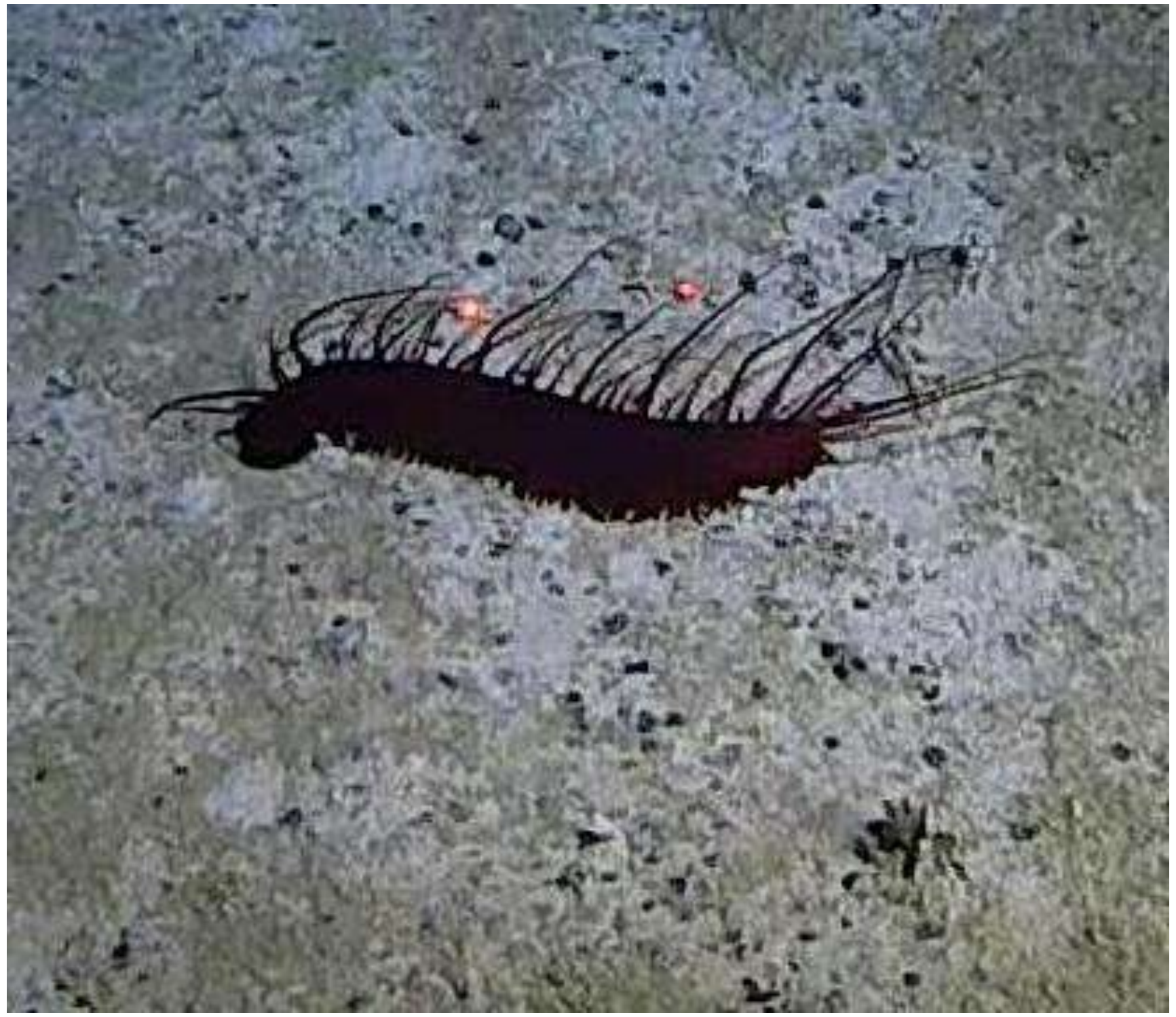

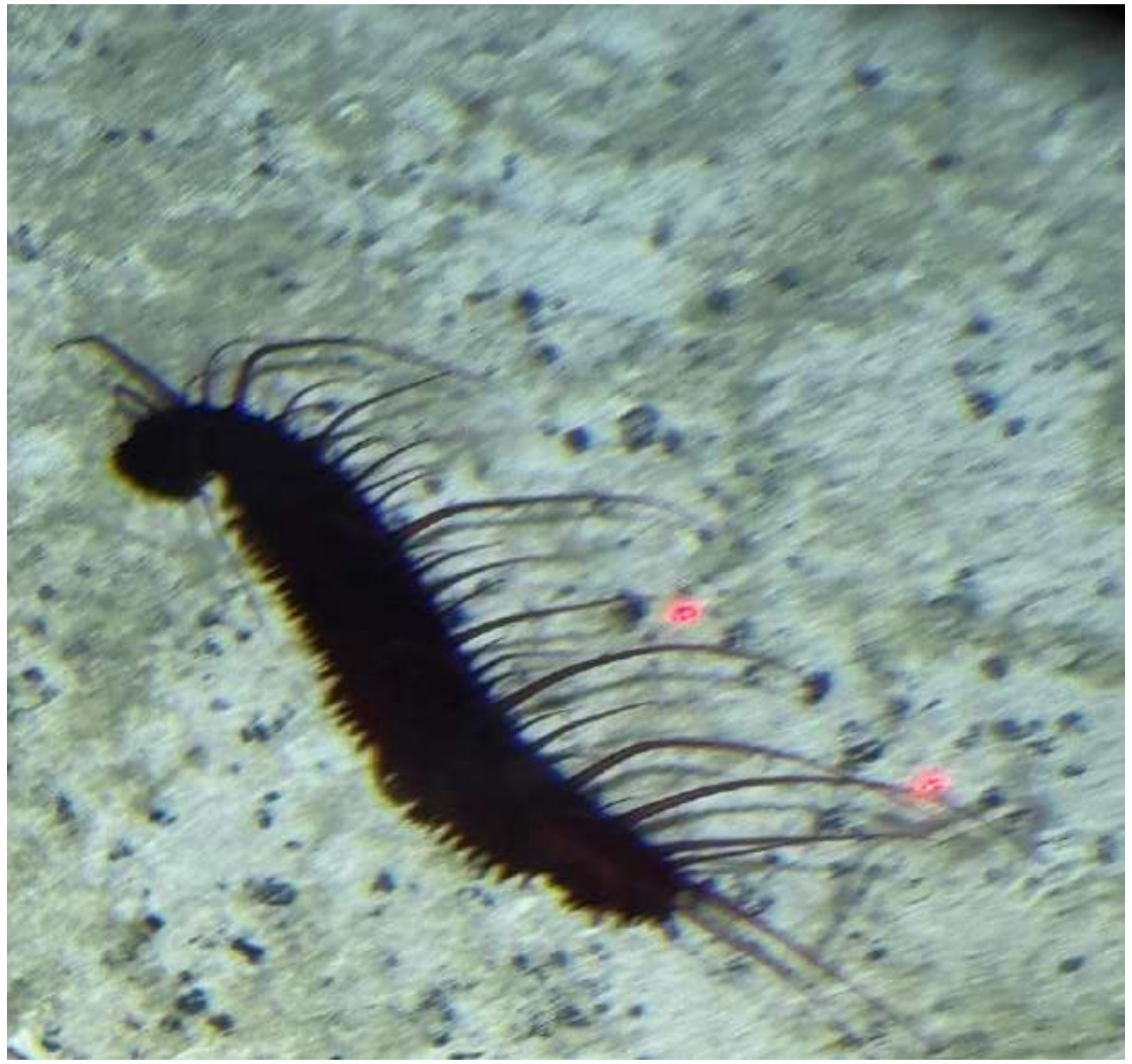

Deep; dark red colour. Body is cylindrical with posterior end resembling a "head" and fringed with short papillae. Dorsal papillae are long and are arranged in two rows running parallel along the body to the posterior end. Multiple short ventral tube feet observed.

Holothuroidea
Persiculida; *Benthothuria;*
Benthothuria sp. indet. 1

NC2_BK5_4600_NOR_0351

NC4_BK4_4700_IMG_6106

NC4_BK4_4700_IMG_6130

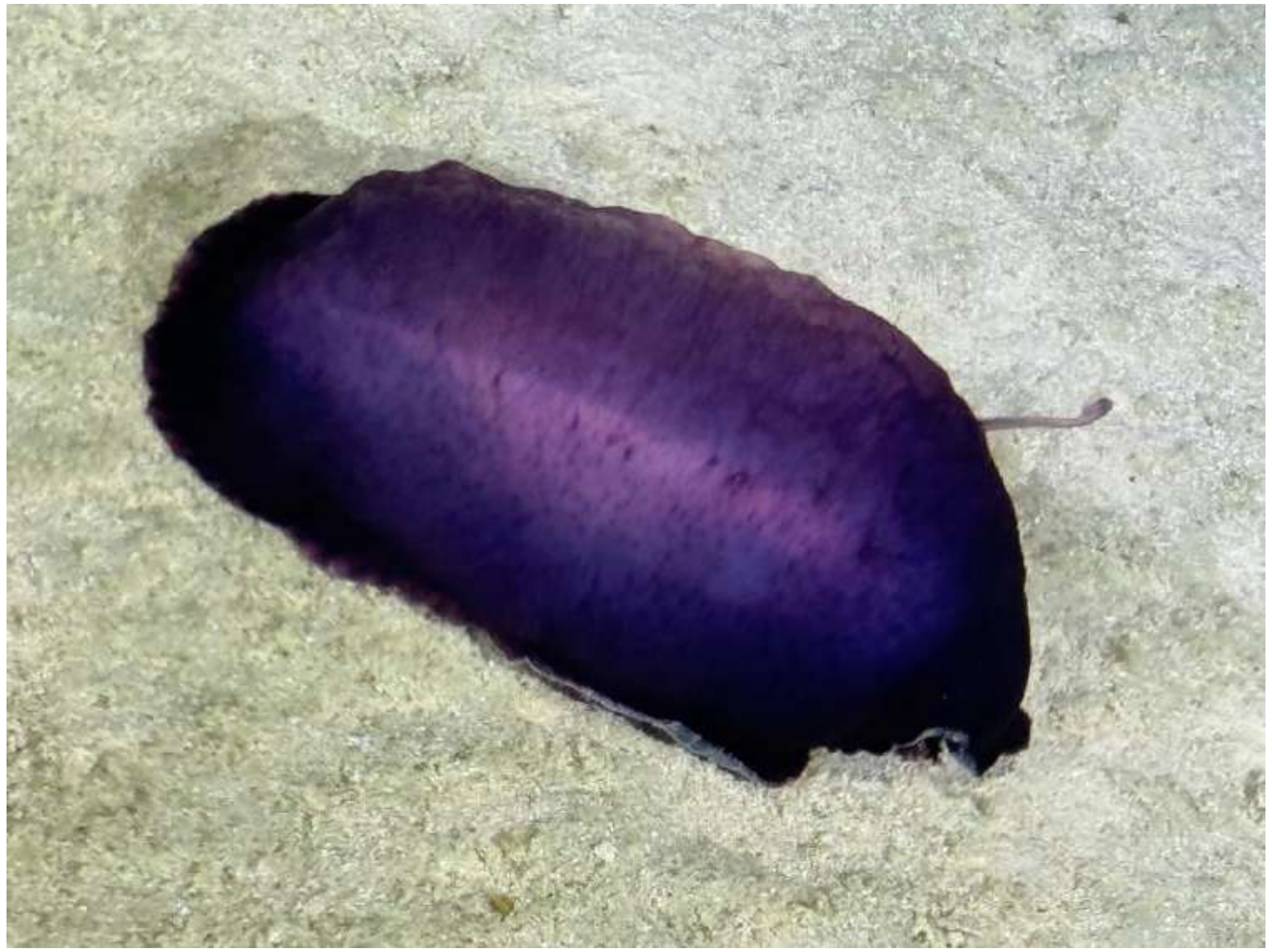

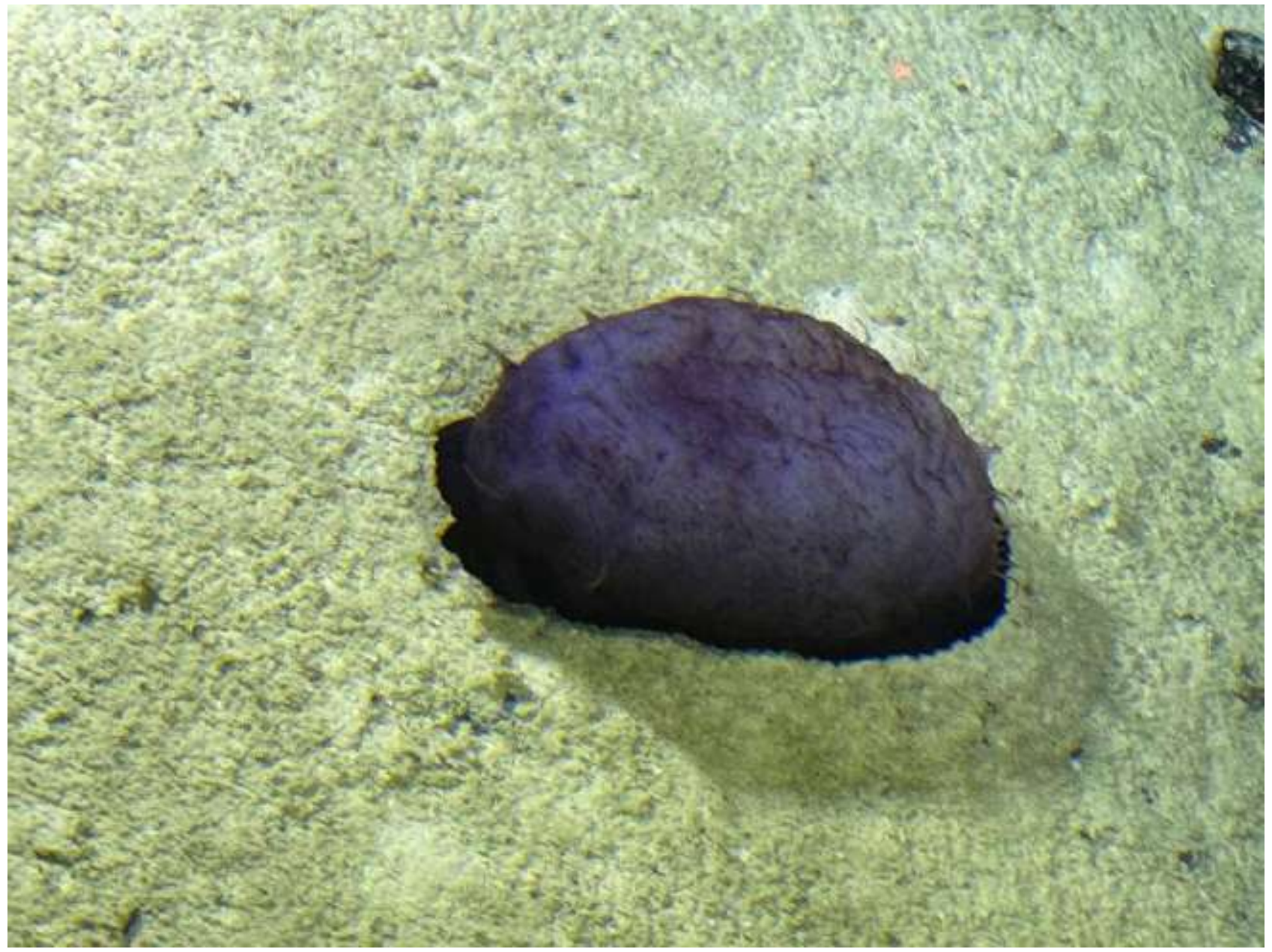

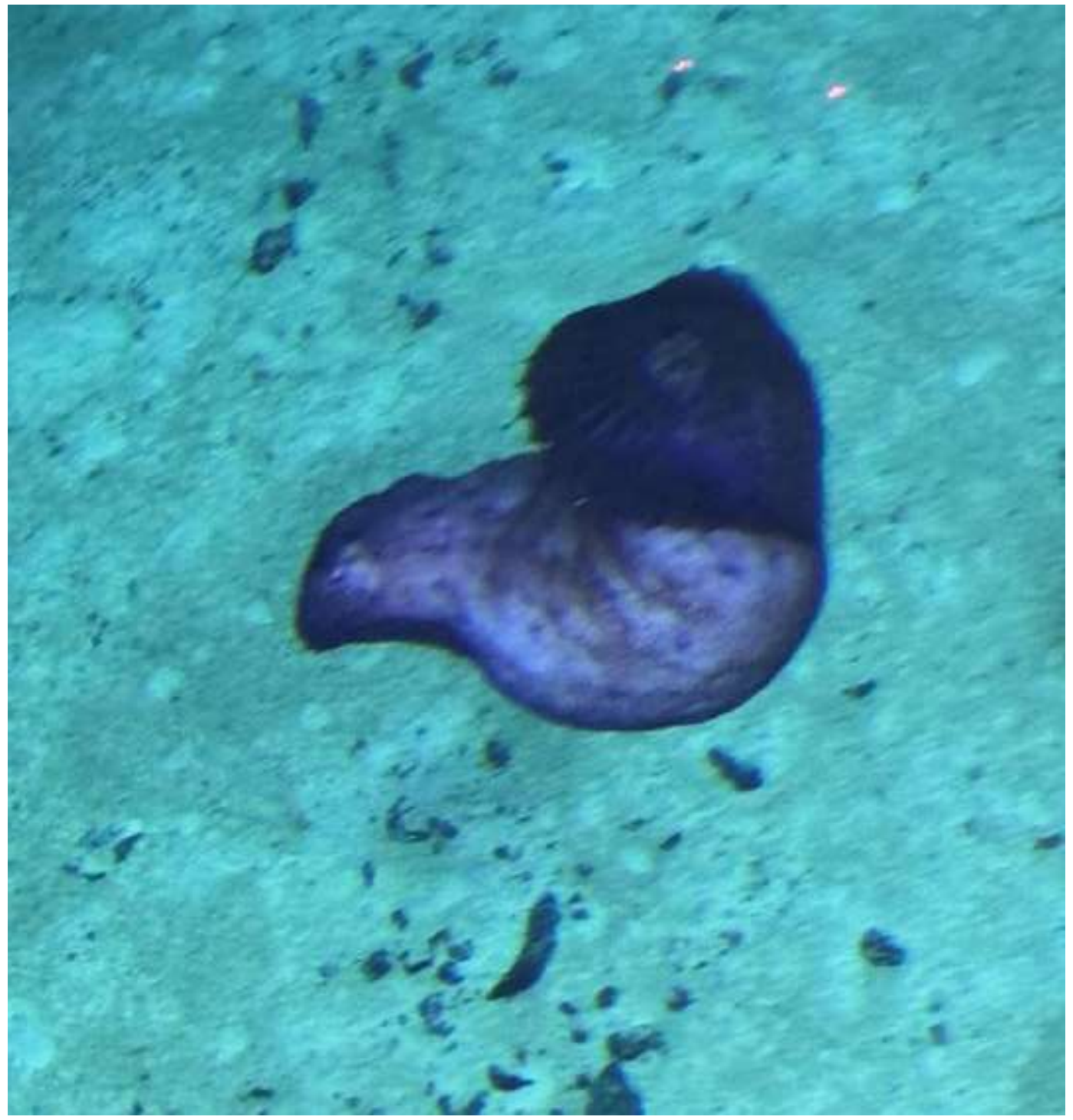

Body is purple colouration that fades to black on the brim. Skin has wrinkled texture. Ventral side is flattened while dorsal side is arched and rounded. Dorsal papillae observed with longer papillae observed on posterior end.

Holothuroidea
Synallactida; Deimatidae; *Oneirophanta* sp. indet. 1

NC4_BK4_4700_NOR_0074

NC4_BK4_4700_IMG_6121

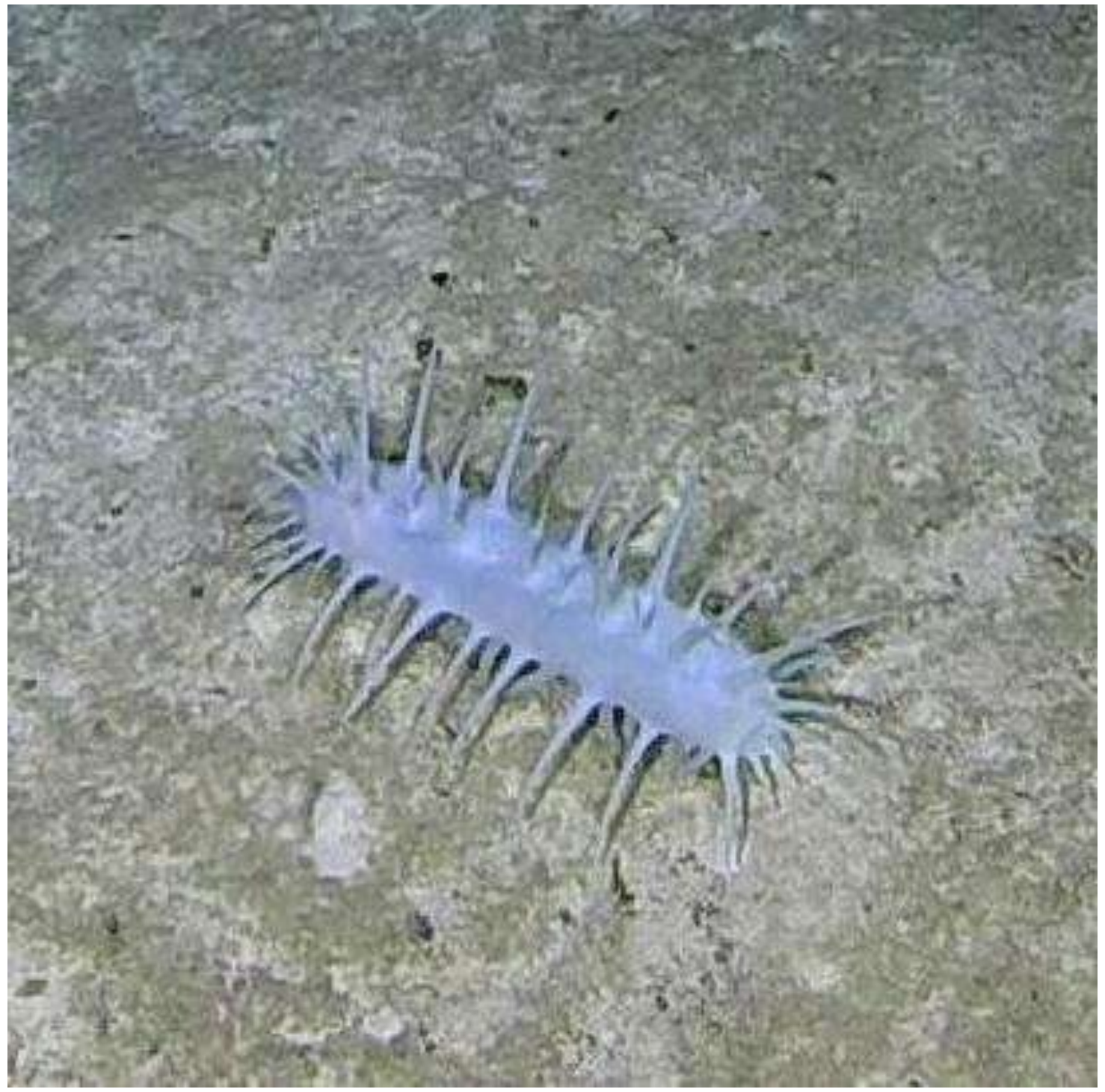

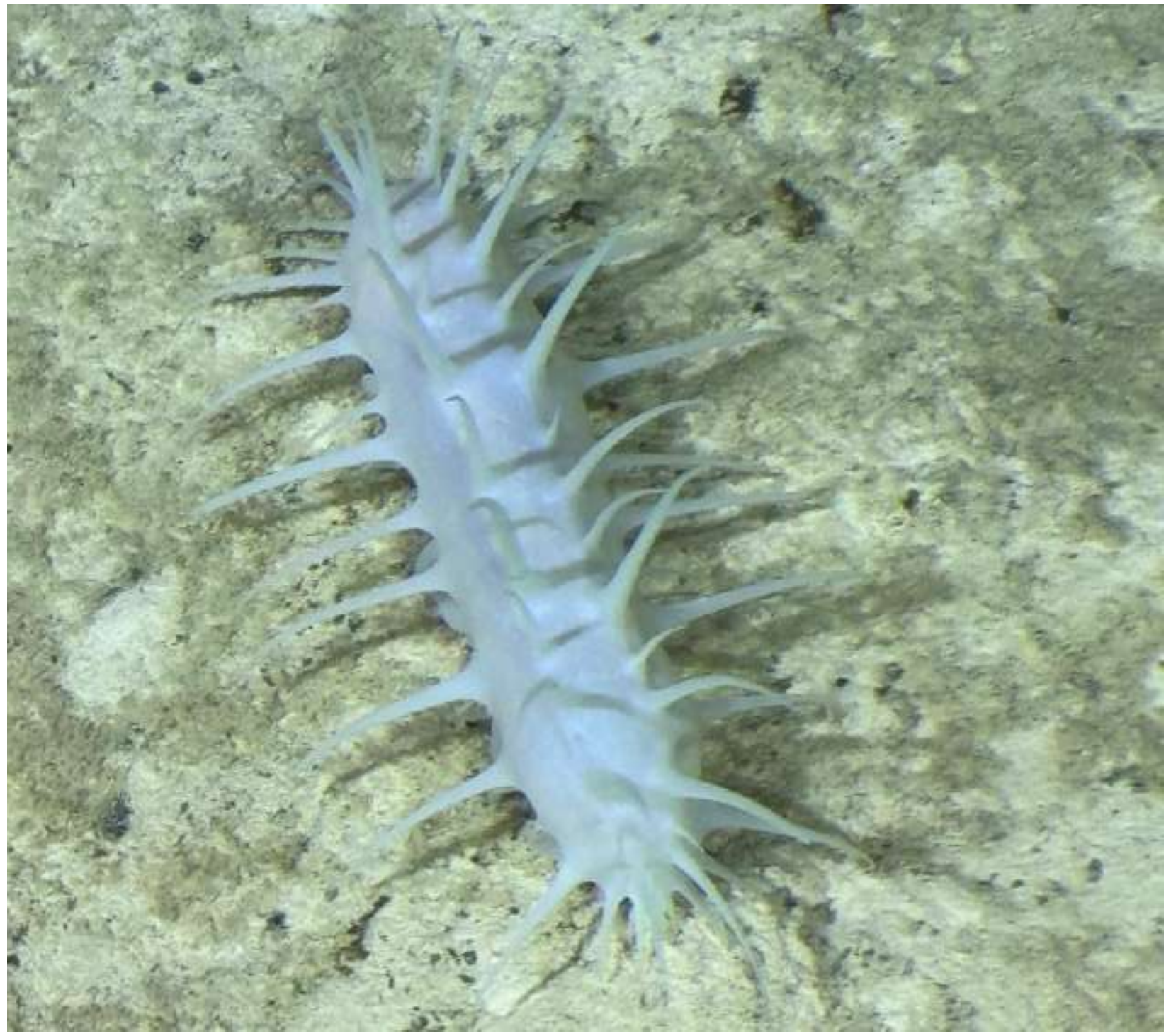

Pale; off white-grey colour. Body is cylindrical with dorsal papillae that are long and thick and are arranged in two rows running parallel along the body to the posterior end. Additional rows of papillae are present along the flank of the animal. Tube feet are observed near the posterior end of the animal.

Holothuroidea
Synallactida; Synallactidae; *Paelopatides* sp. indet. 1

NC2_BK5_4600_IMG_0548

NC2_BK5_4600_IMG_0315

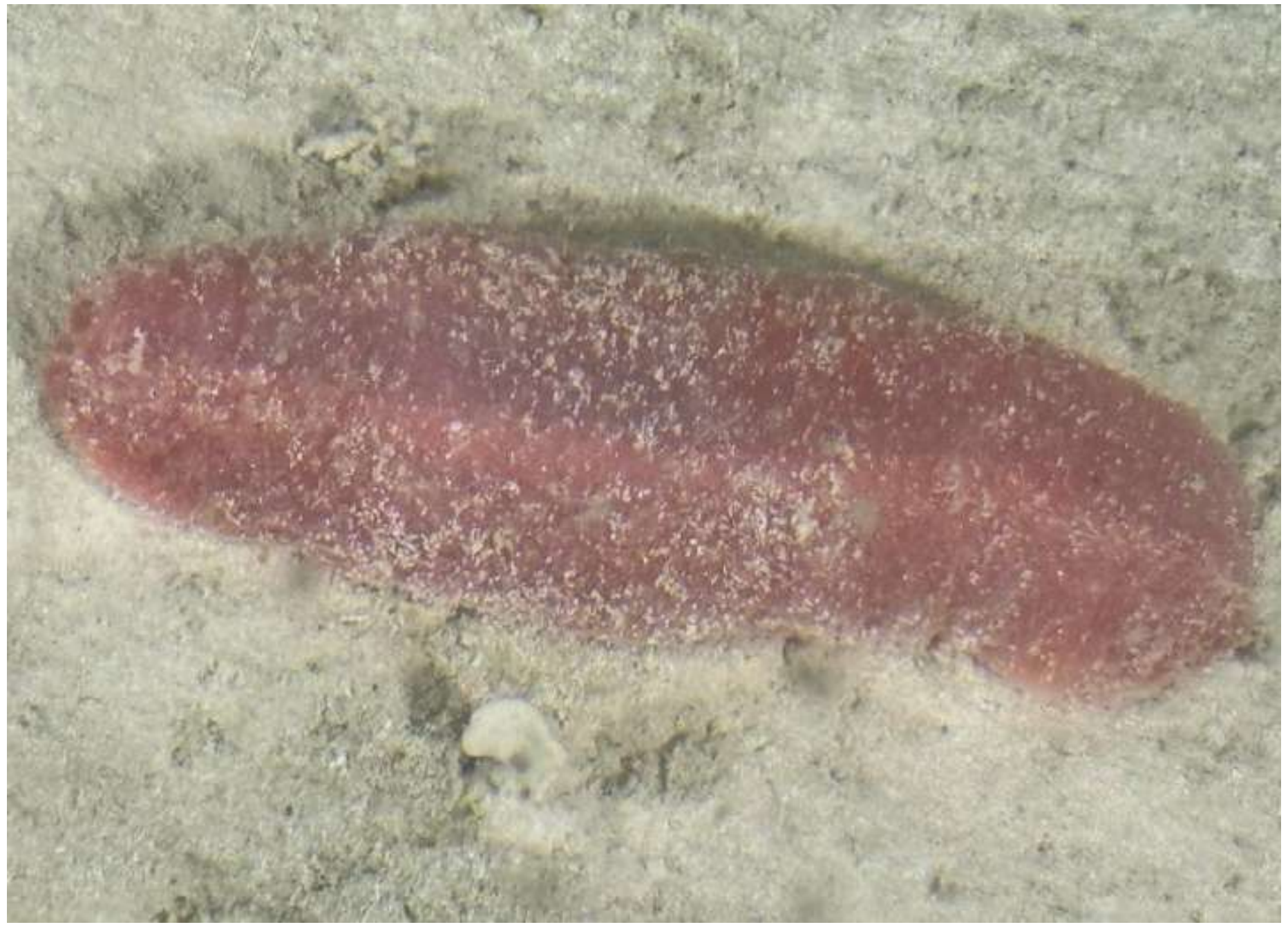

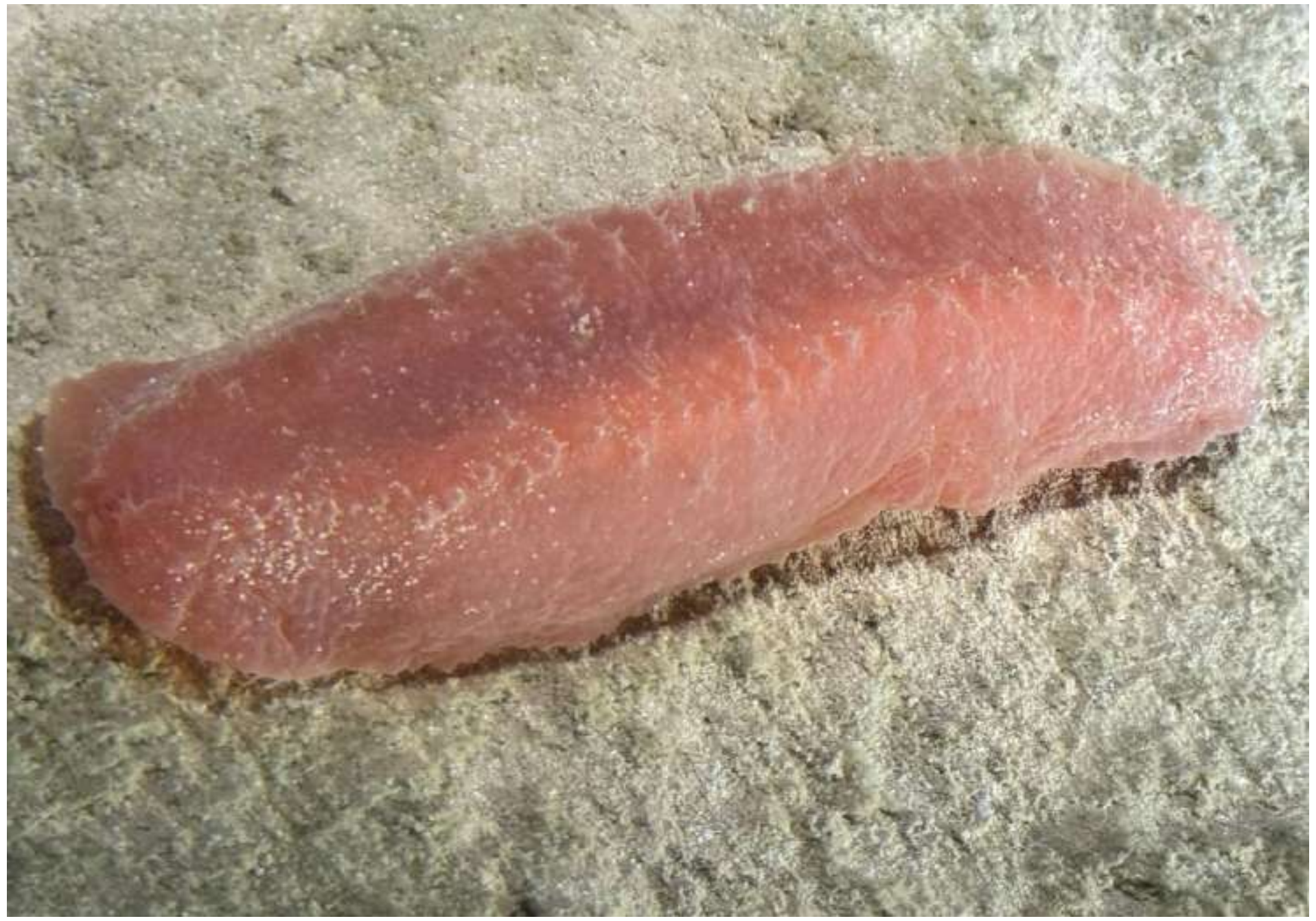

Pink colour. Body is cylindrical with dorsal papillae that are short and thin and are arranged in two rows of paired papillae running parallel along the body to the posterior end.

Holothuroidea
Synallactida; Synallactidae;
Paelopatides sp. indet. 2

NC3_OM5_5400_NOR_0042

NC1_BK2_6700_IMG_4918

NC4_BK3_6900_IMG_5932

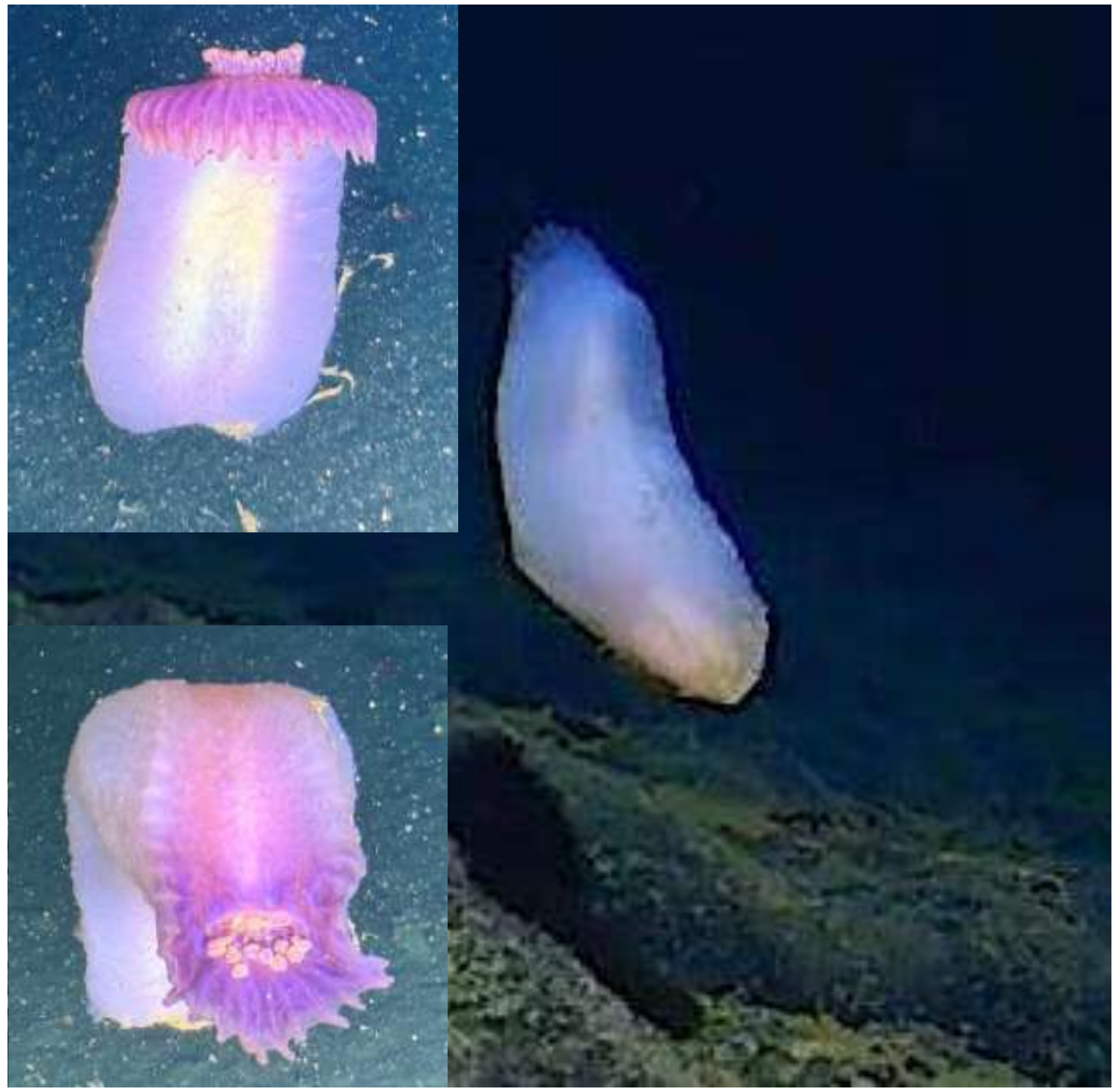

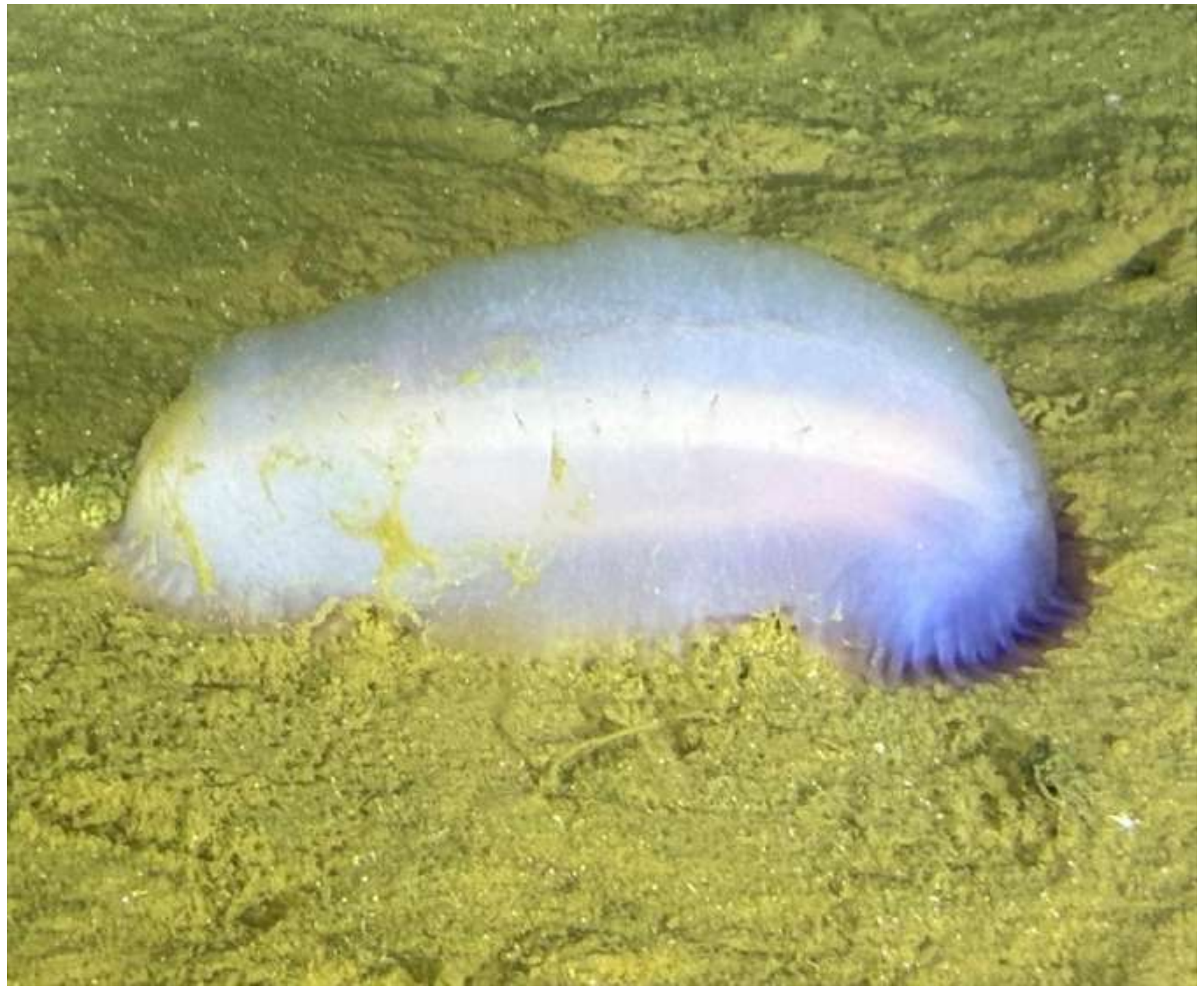

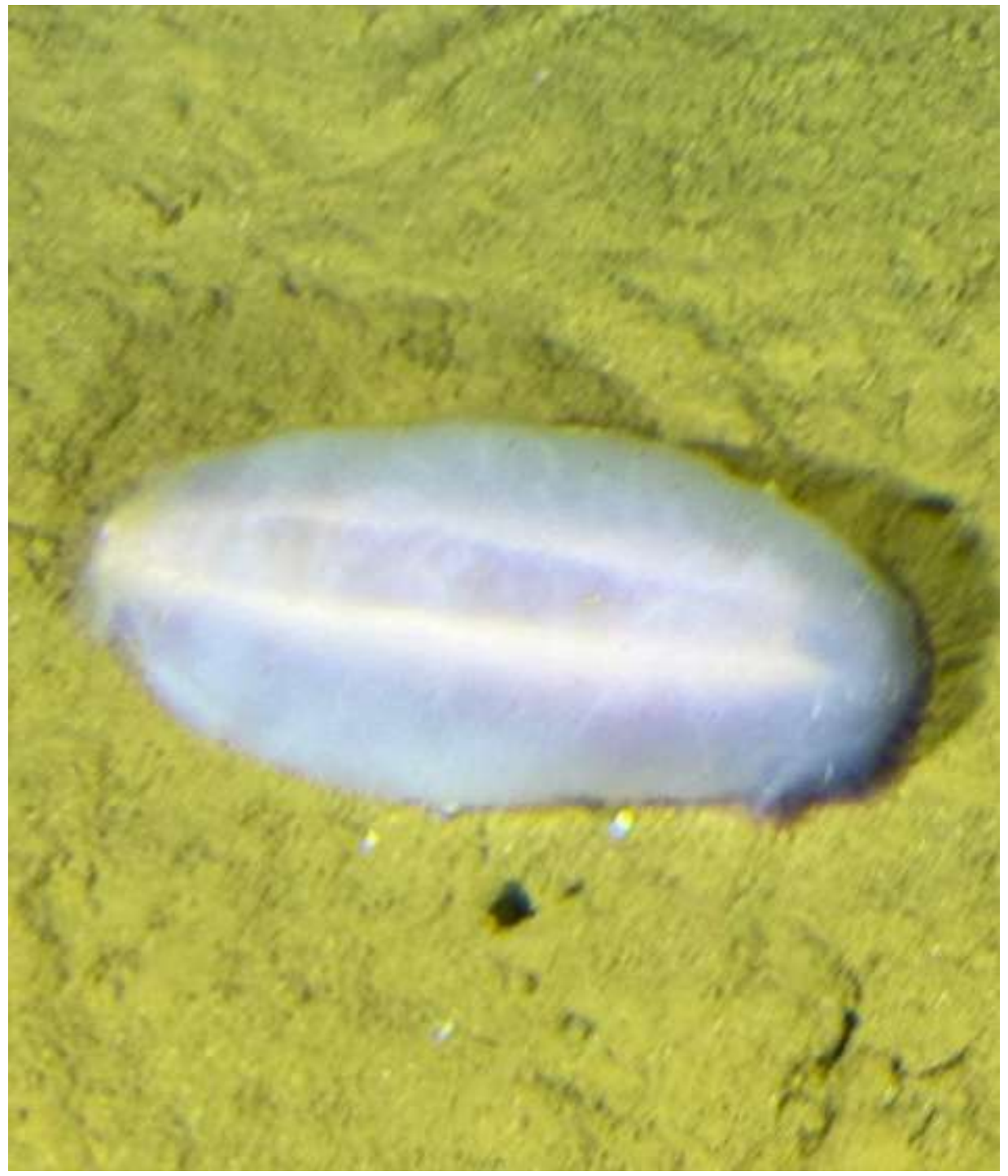

Body is opaque and whitish with faint blue colouration on anterior brim. Skin has smooth. Ventral side is flattened while dorsal side is arched.

Holothuroidea
Synallactida; Synallactidae; Synallactidae gen. indet. 1

NC2_BK5_4600_IMG_0428

NC2_BK5_4600_IMG_0432

NC2_BK5_4600_IMG_0317

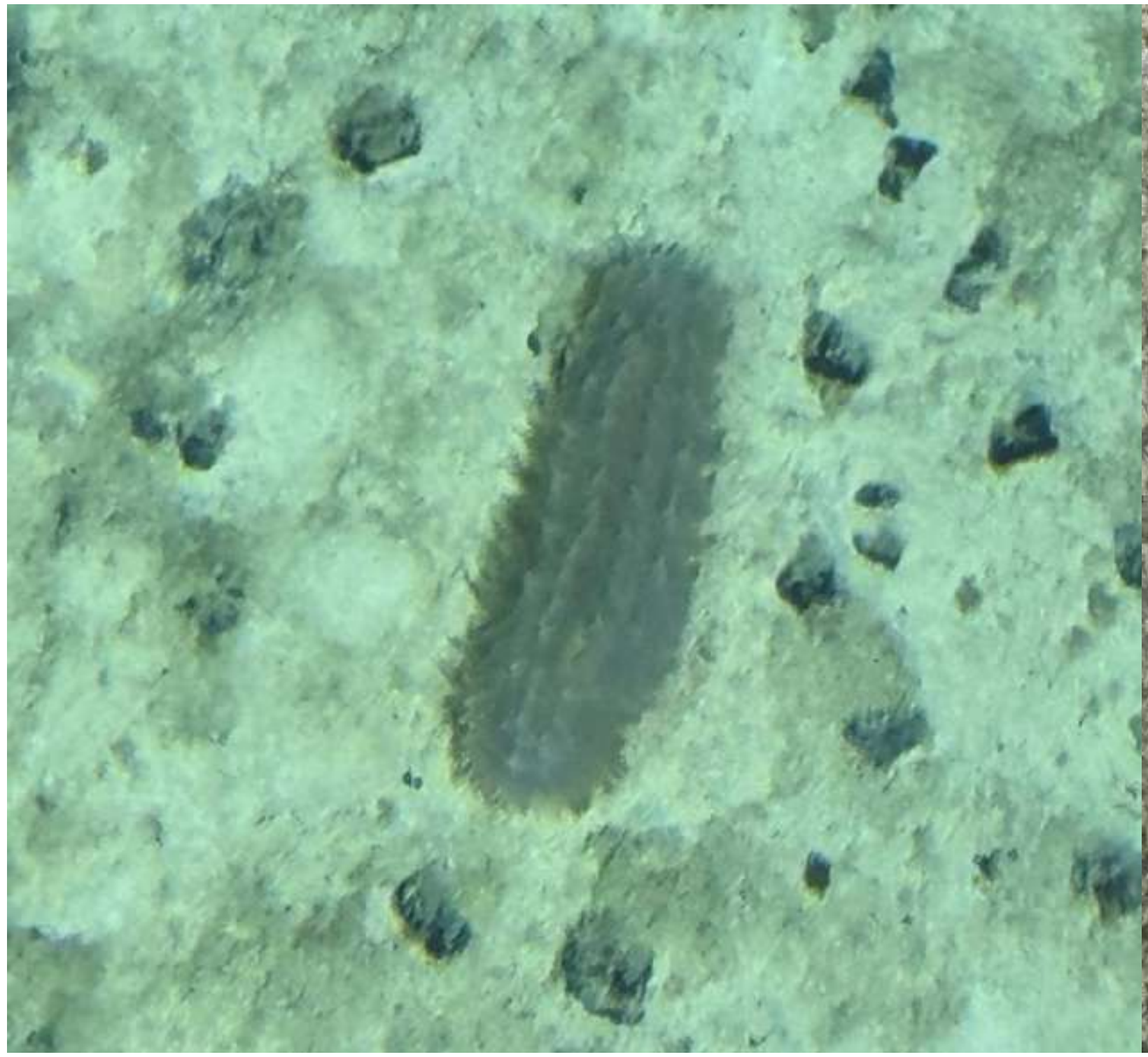
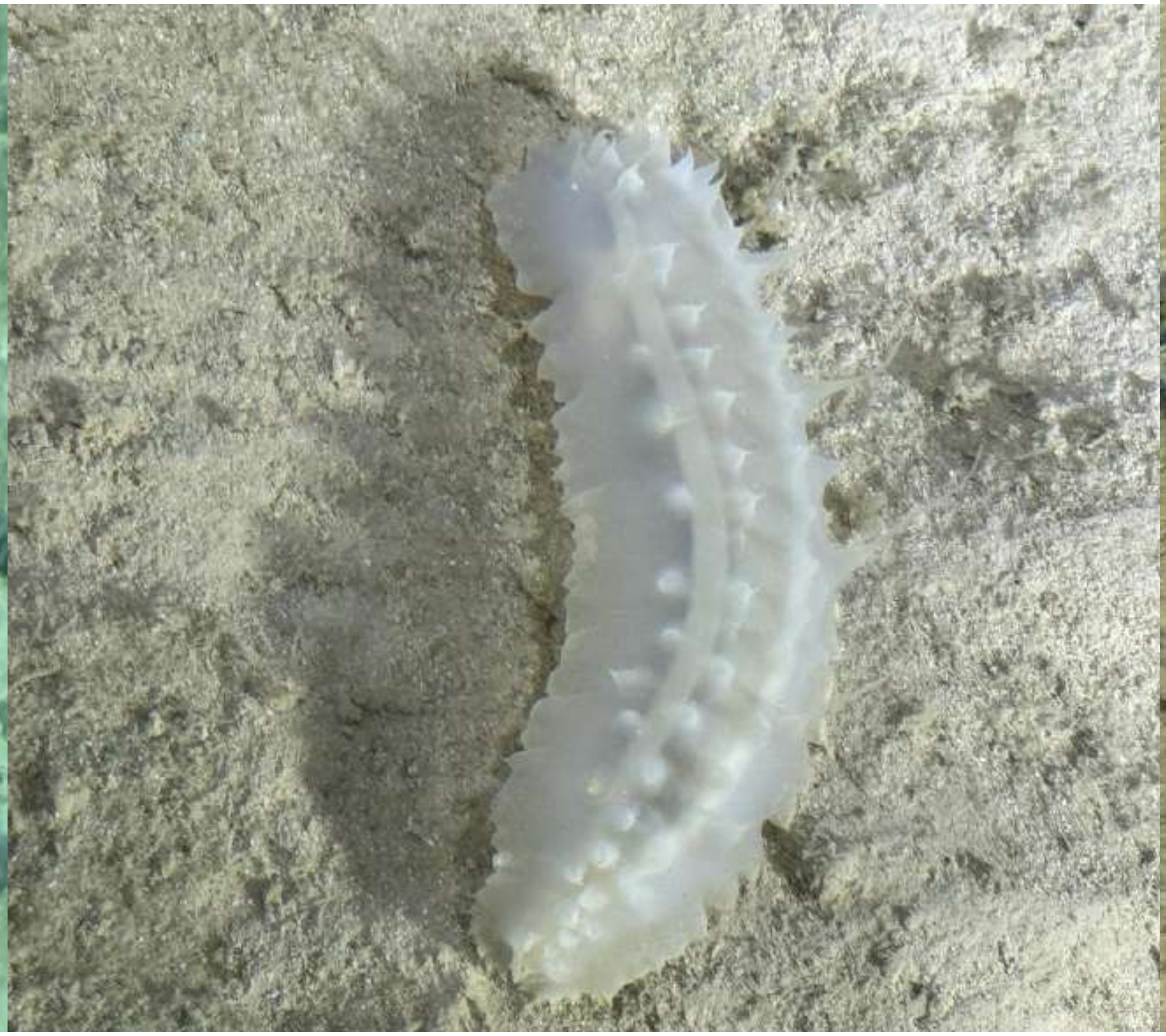
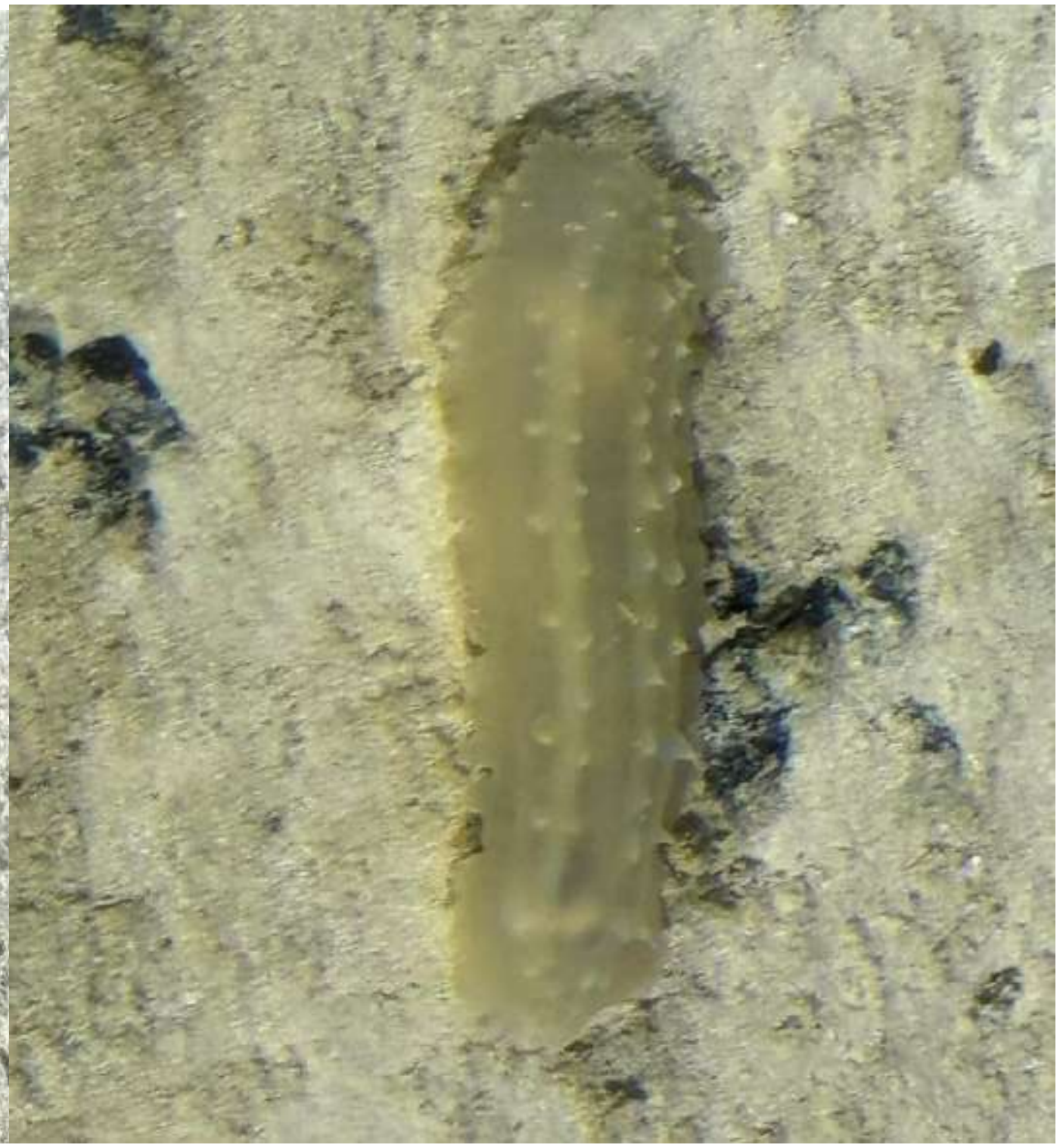

White/green/Grey colour. Body is cylindrical with dorsal papillae that are short and thick and are arranged in four rows of papillae running parallel along the body that converge at the posterior end. Brim is present around the animal and wraps around the anterior and posterior ends.

Holothuroidea
Synallactida; Synallactidae; Synallactidae gen. indet. 2

NC3_BK2_6000_IMG_2408

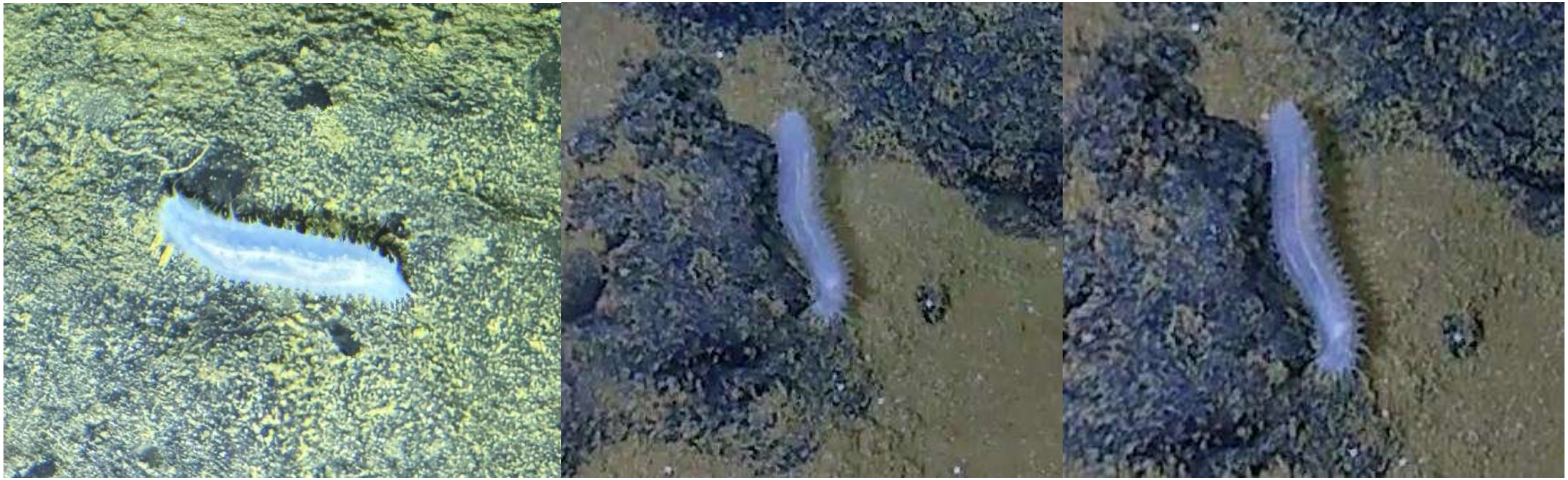

Skin transparent-white. Body is cylindrical with dorsal papillae that are short and thick and are arranged in two rows of papillae running parallel along the body that converge at the posterior end. Brim is present around the animal and wraps around the anterior end.

Holothuroidea
Persiculida; Molpadiodemidae, *Molpadiodemas* sp. indet. 1

NC3_BK2_6000_NOR_0060

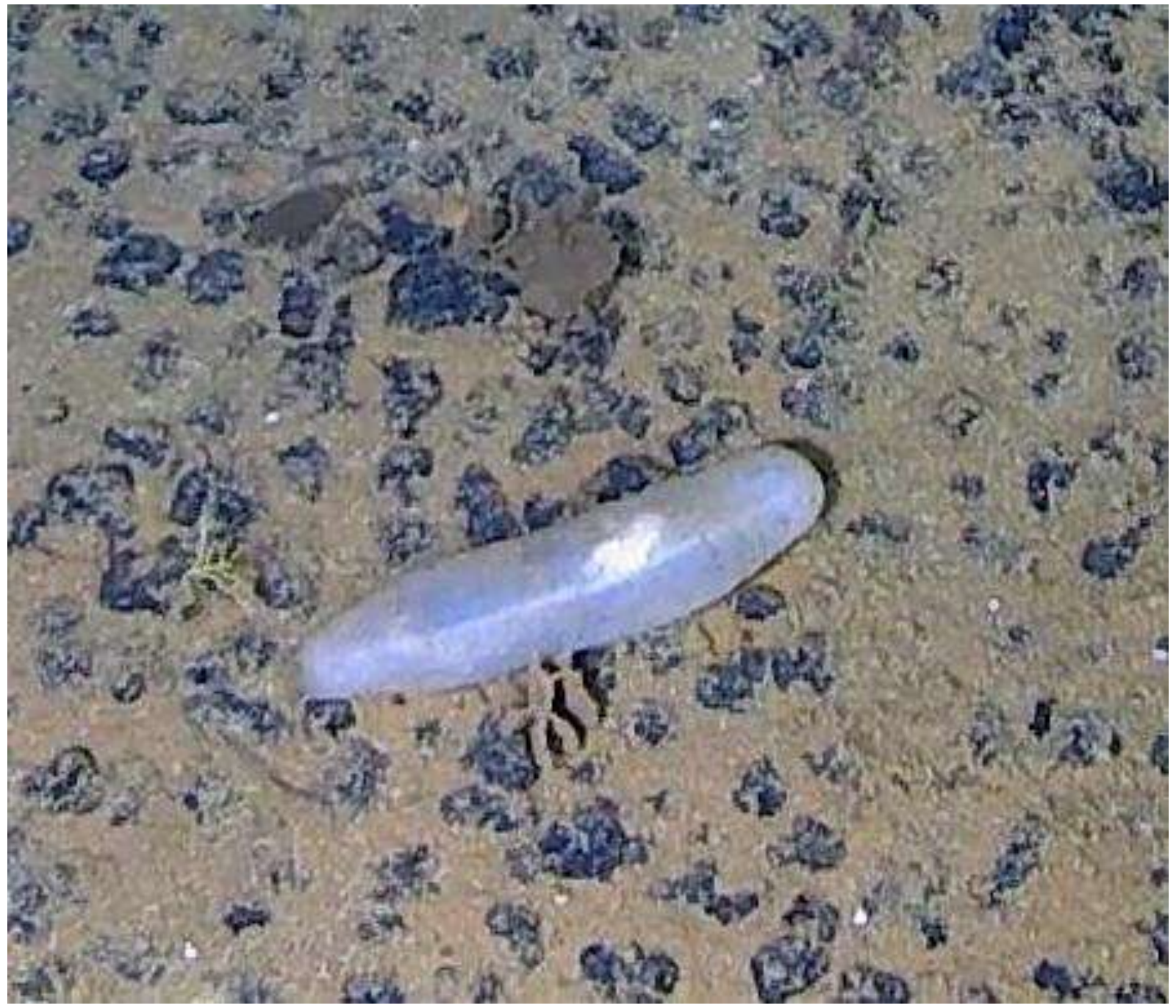

Skin transparent-white. Body is cylindrical with no dorsal papillae Brim is not present around the animal and wraps around the anterior end.

Cf. hadalothuria

Holothuroidea; Persiculida; Molpadiodemidae, *Molpadiodemas* sp. indet. 2

NC2_BK3_7400_IMG_8907

NC2_BK3_7400_IMG_9291

NC1_BK2_6700

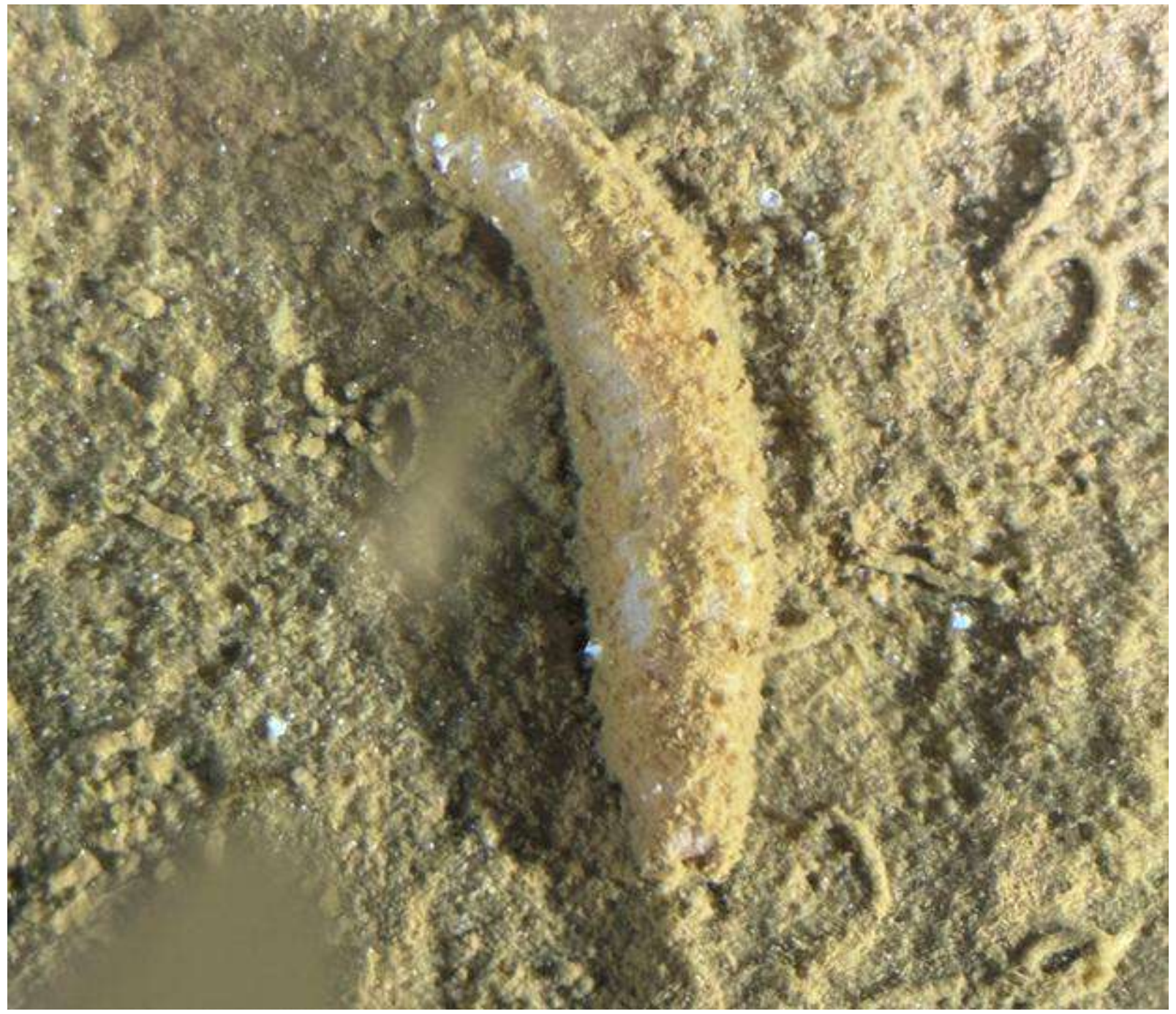

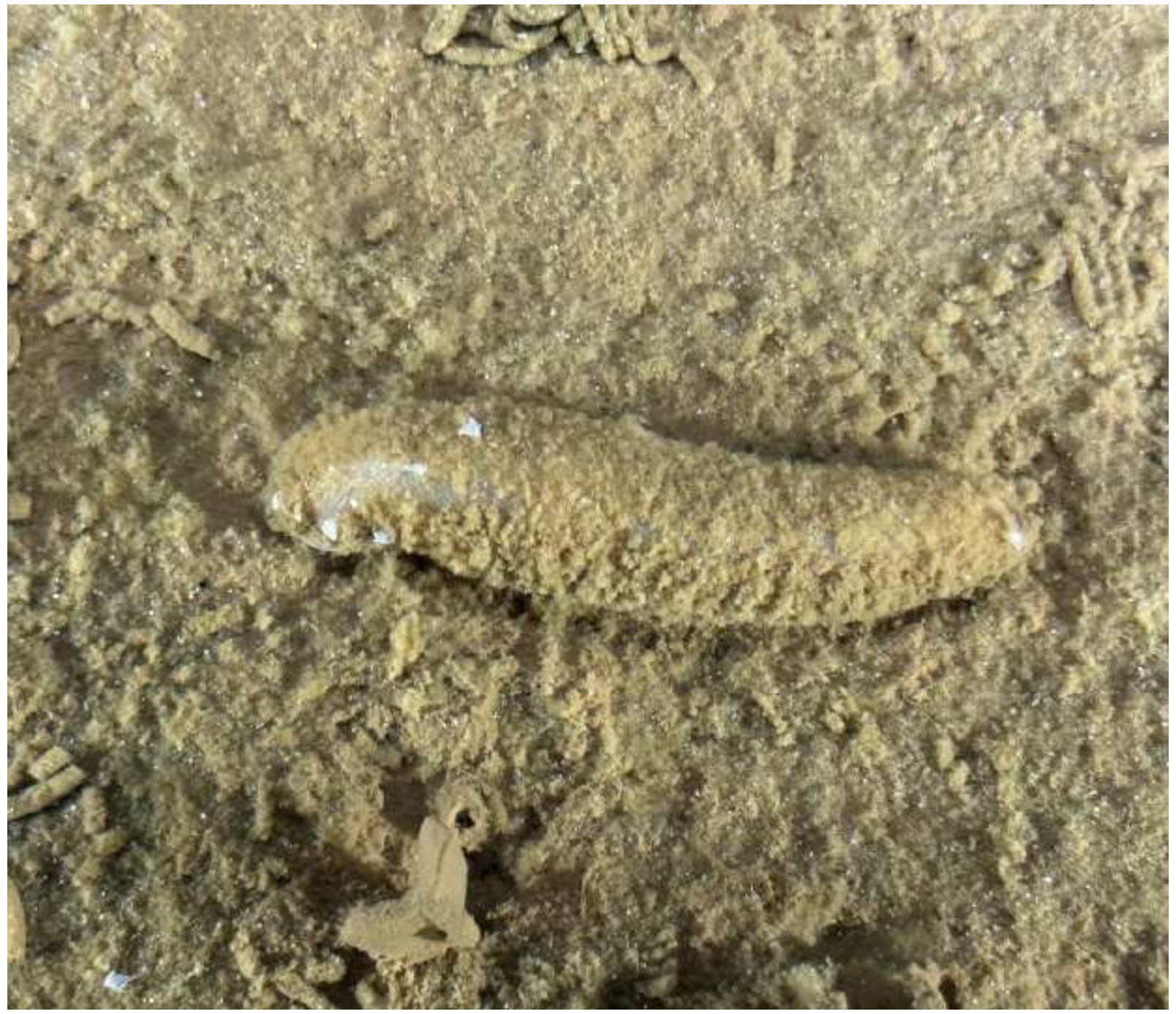

Off white colour. Body is cylindrical with no apparent tube feet or dorsal papillae. Observed coated in surrounding sediments. Possibly Meseres sp.

Holothuroidea; Holothuriida Mesothuriidae; *Mesothuria; Mesothuria* sp. indet.1

NC2_BK4_5300_IMG_9443

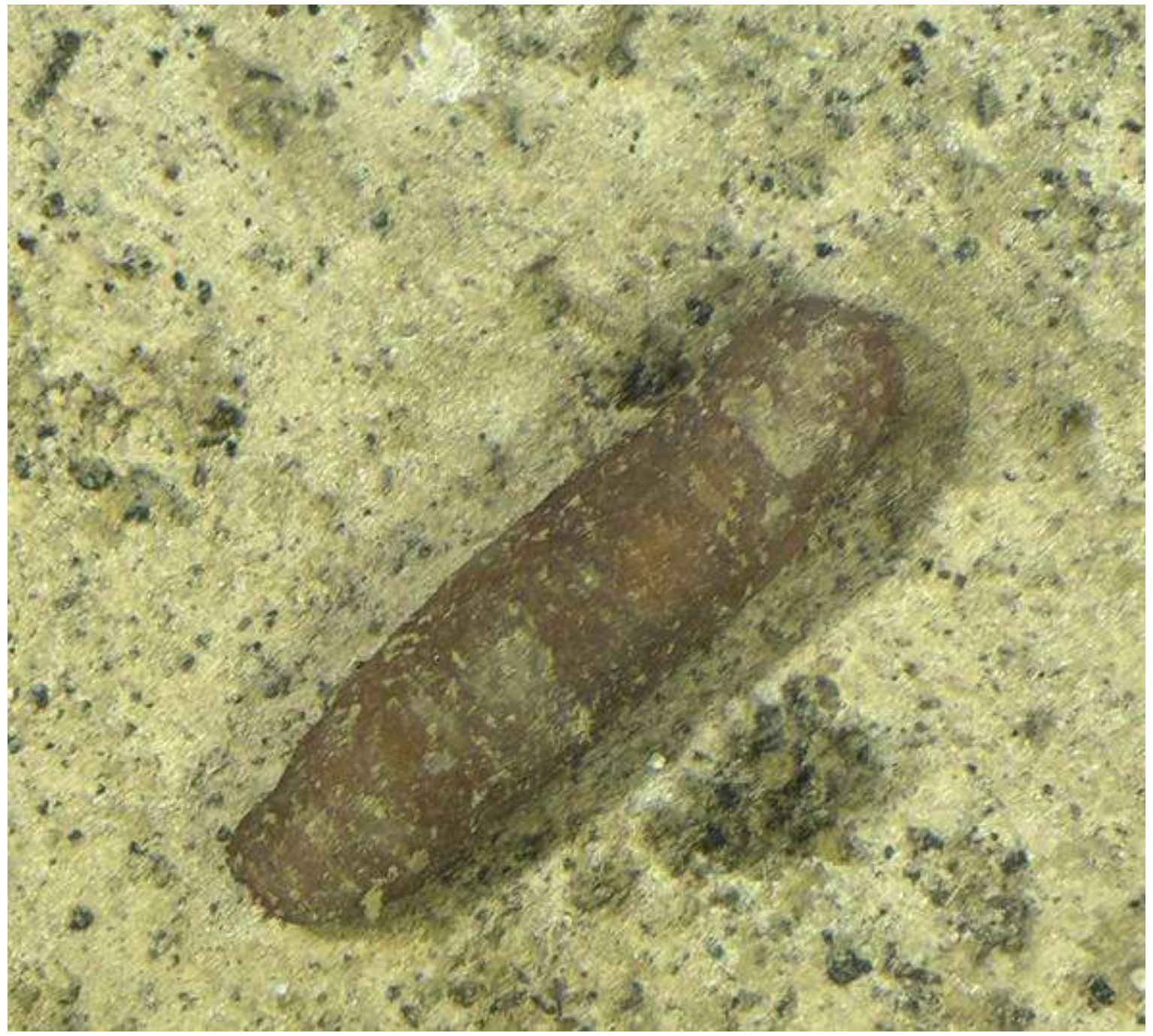

Brown colouration. Body is cylindrical with no apparent tube feet or dorsal papillae. Observed lightly coated in surrounding sediments. Possibly Meseres sp.

Holothuroidea; Holothuriida Mesothuriidae; *Mesothuria; Mesothuria* sp. indet. 2

NC2_BK1_3200_IMG_7017

NC1_BK1_6200

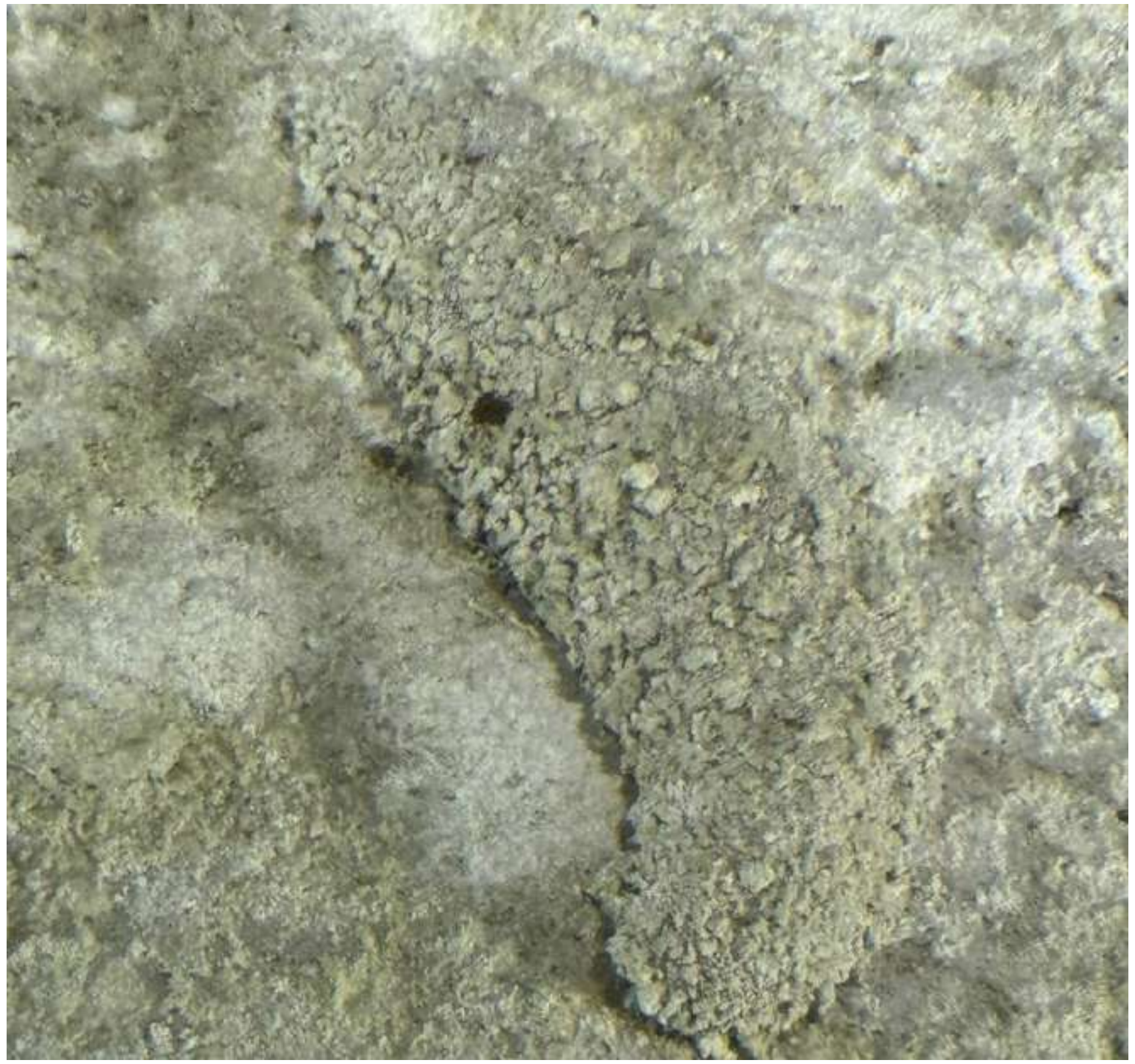

Body is cylindrical with no apparent tube feet or dorsal papillae. Observed coated in surrounding sediments.

Holothuroidea; Holothuriida Mesothuriidae; *Mesothuria; Mesothuria* sp. indet. 3

NC1_BK1_6200_IMG_4583

NC1_BK1_6200_IMG_4588

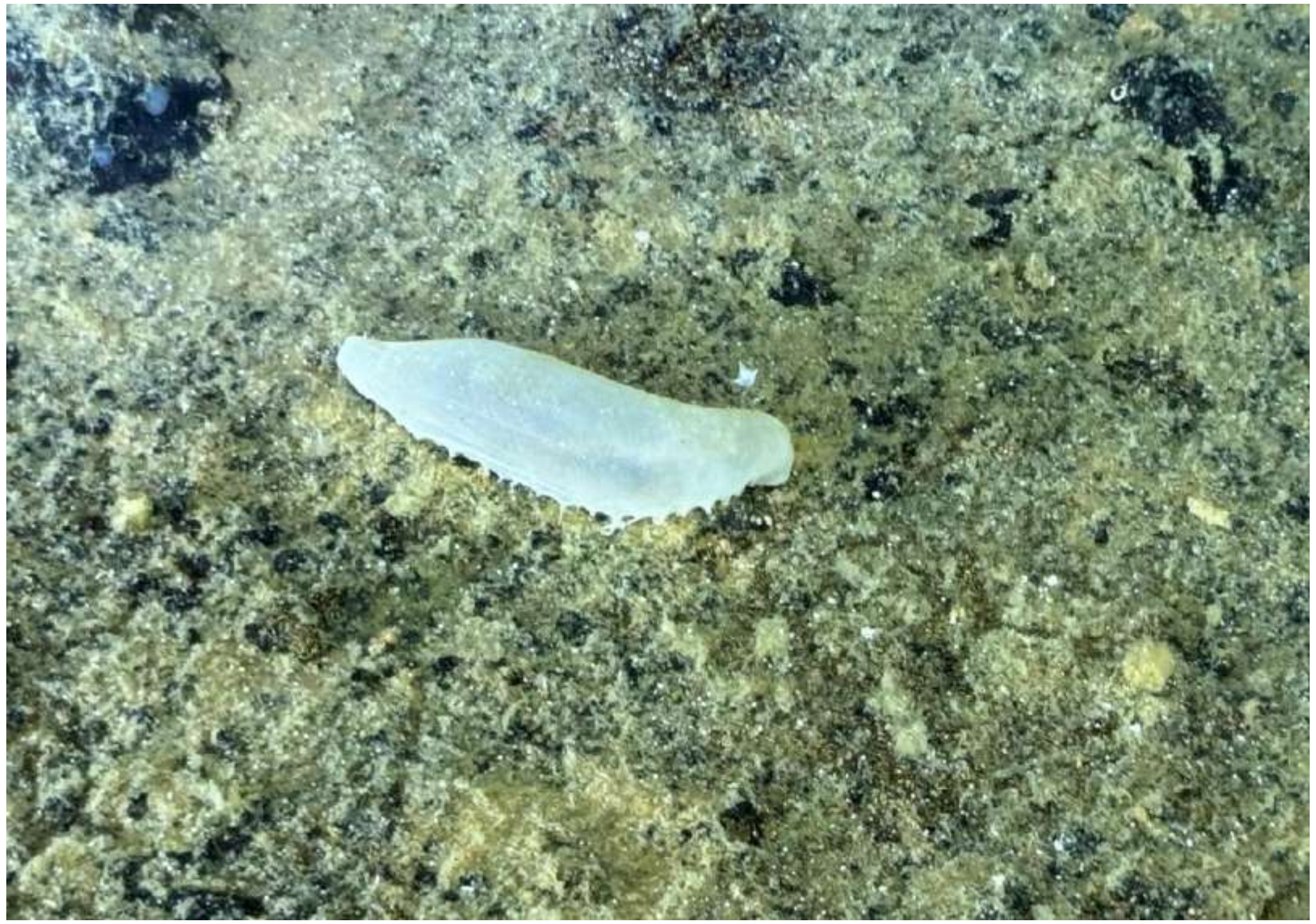

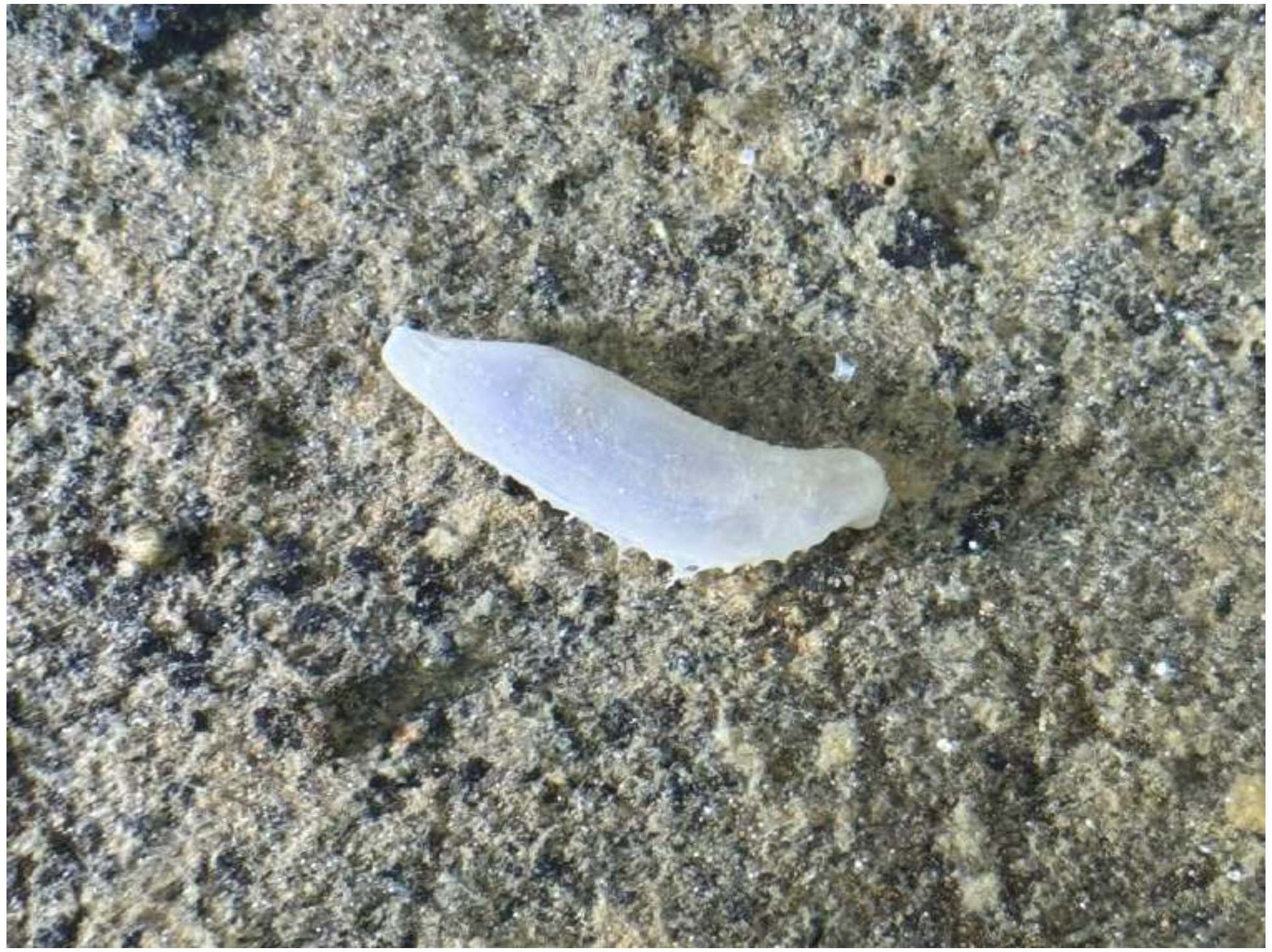

Translucent; white colour. Body is cylindrical with small tube feet and no apparent dorsal papillae.

Crinoidea; Comatulida; Comatulida fam. indet. 1

NC2_BK2_3800 – NOR_0085
NC2_BK1_3200 – NOR_0056

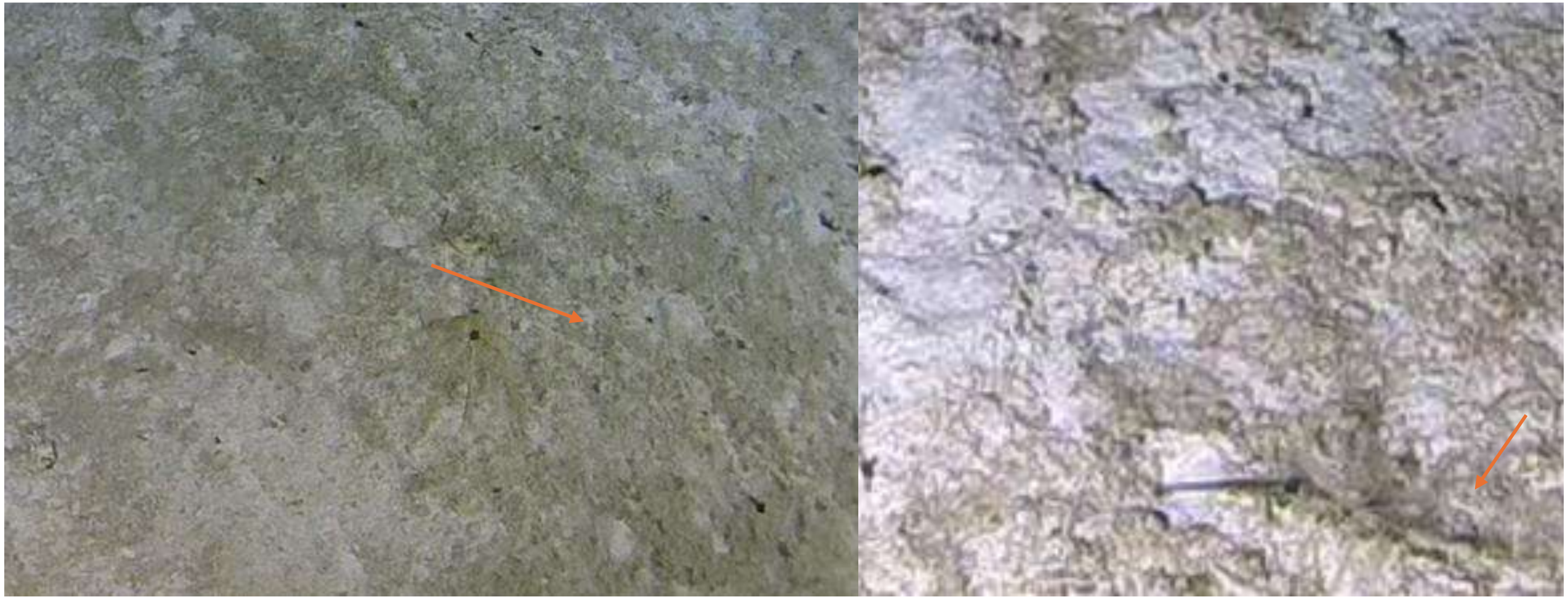

Feather star morphotype with dark calyx, sand coloured body. More than five arms, which suggest Thaumatocrinus. *However, structure of the arms, pinnules, and attachment are unclear.*

Crinoidea; Comatulida; Comatulida fam. indet. 2

NC2_BK4_5300 – NOR_0075

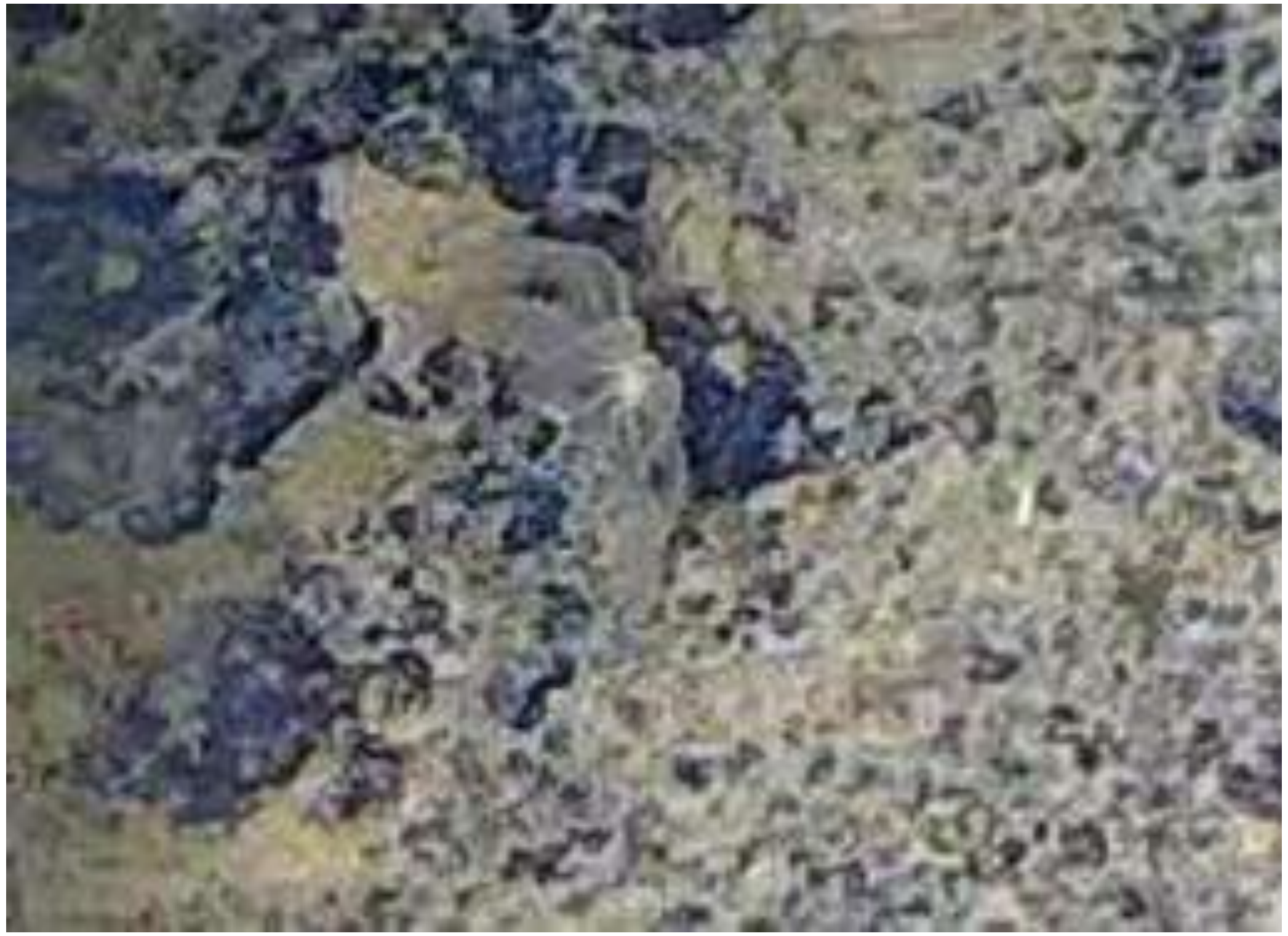

Feather star morphotype with light calyx and >5 number of arm, which suggest Thaumatocrinus. *However, structure of the arms, pinnules, and attachment are unclear.*

Crinoidea; Comatulida; Pentametrocrinidae gen. inc. 1

NC1_BK1_6200_IMG_4611

NC3_BK2_6000 – NOR_0085

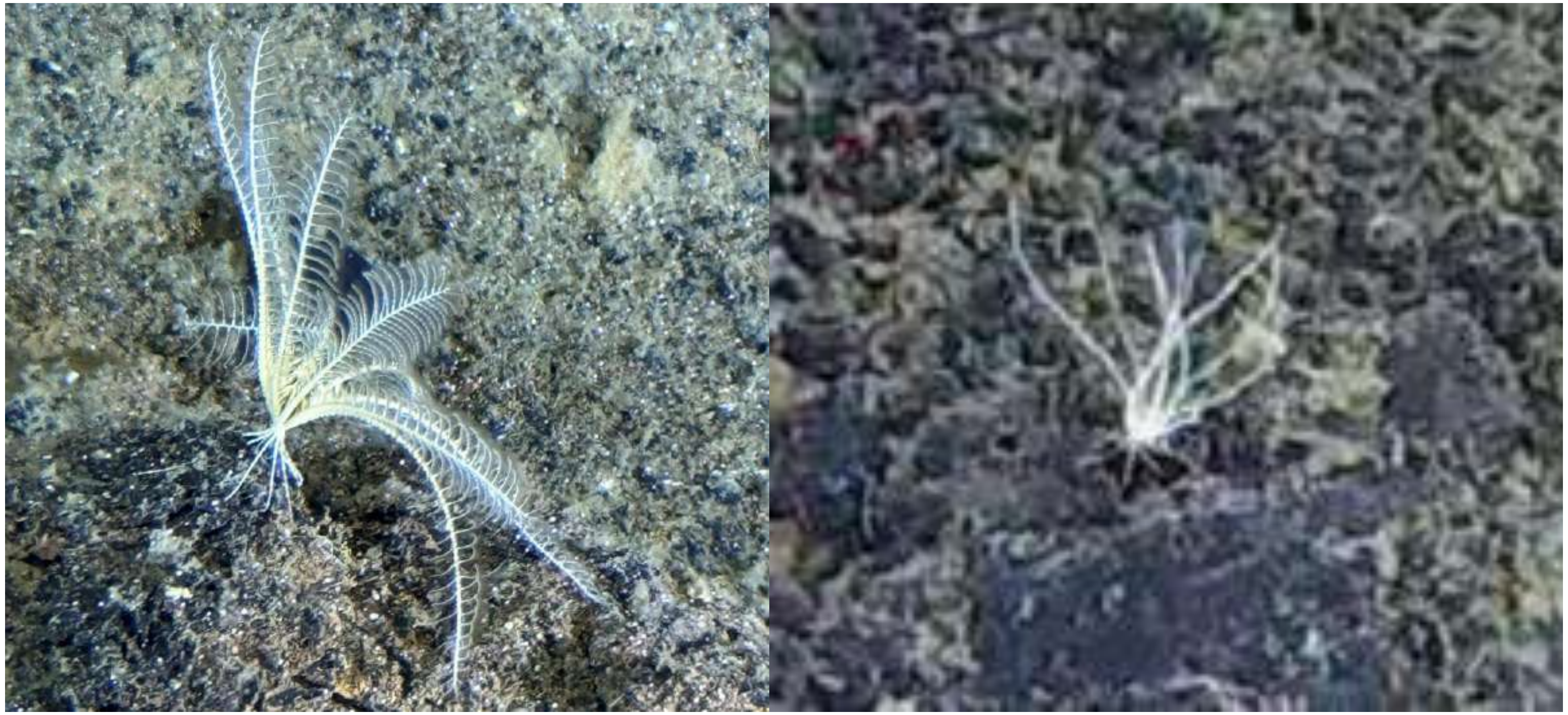

Feather star; white specimen without a segmented stalk and 10 undivided arms.

Originally annotated as Thaumatocrinus gen. inc. 1. Later changed to family level

Crinoidea; Comatulida; Bathycrinidae; *Bathycrinus* gen. indet. 1

NC1_BK1_6200_IMG_4128

NC1_BK1_6200_IMG_4635

NC1_BK1_6200_NOR_0068 ~ 01:30

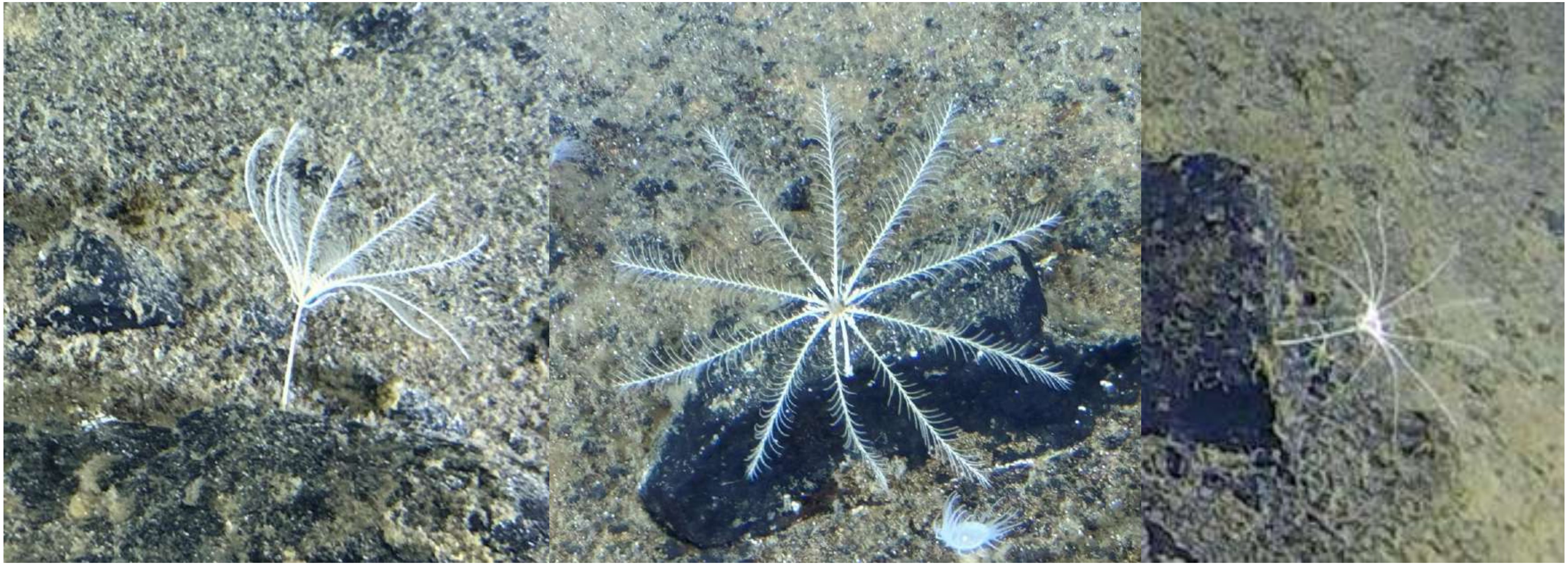

Stalked comatulid with homeomorphic short stalk with 10 undivided arms developing from a small calyx; arms carry short pinnules. Length of the arm and of the stalk are comparable. Vityazicrinus?

Crinoidea; Comatulida; Bathycrinidae; *Bathycrinus* gen. indet. 2

NC3_BK1_8000_IMG_0972

NC3_BK4_8000_IMG_3933

NC3_BK4_8000 – IMG_3901

NC4_BK3_6900

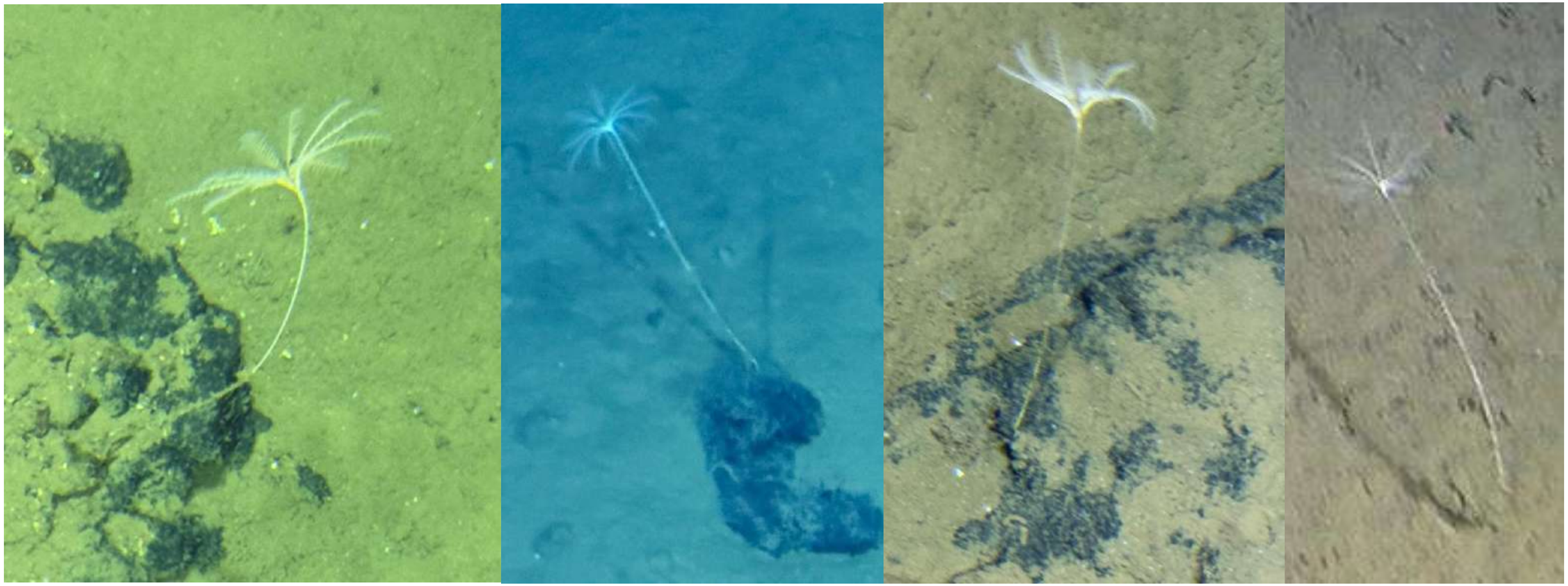

Medium to long stalk with 10 undivided arms developing from a small calyx, which looks bigger than Guillecrinidae sp. 1; arms carry short pinnules. Arms many times shorter than the stalk.

Crinoidea; Hyocrinida; Hyocrinidae; Hyocrinidae gen. indet. 1

NC2_BK5_4600_IMG_9781

NC3_BK2_6000_IMG_2420

NC3_BK2_6000_IMG_2411

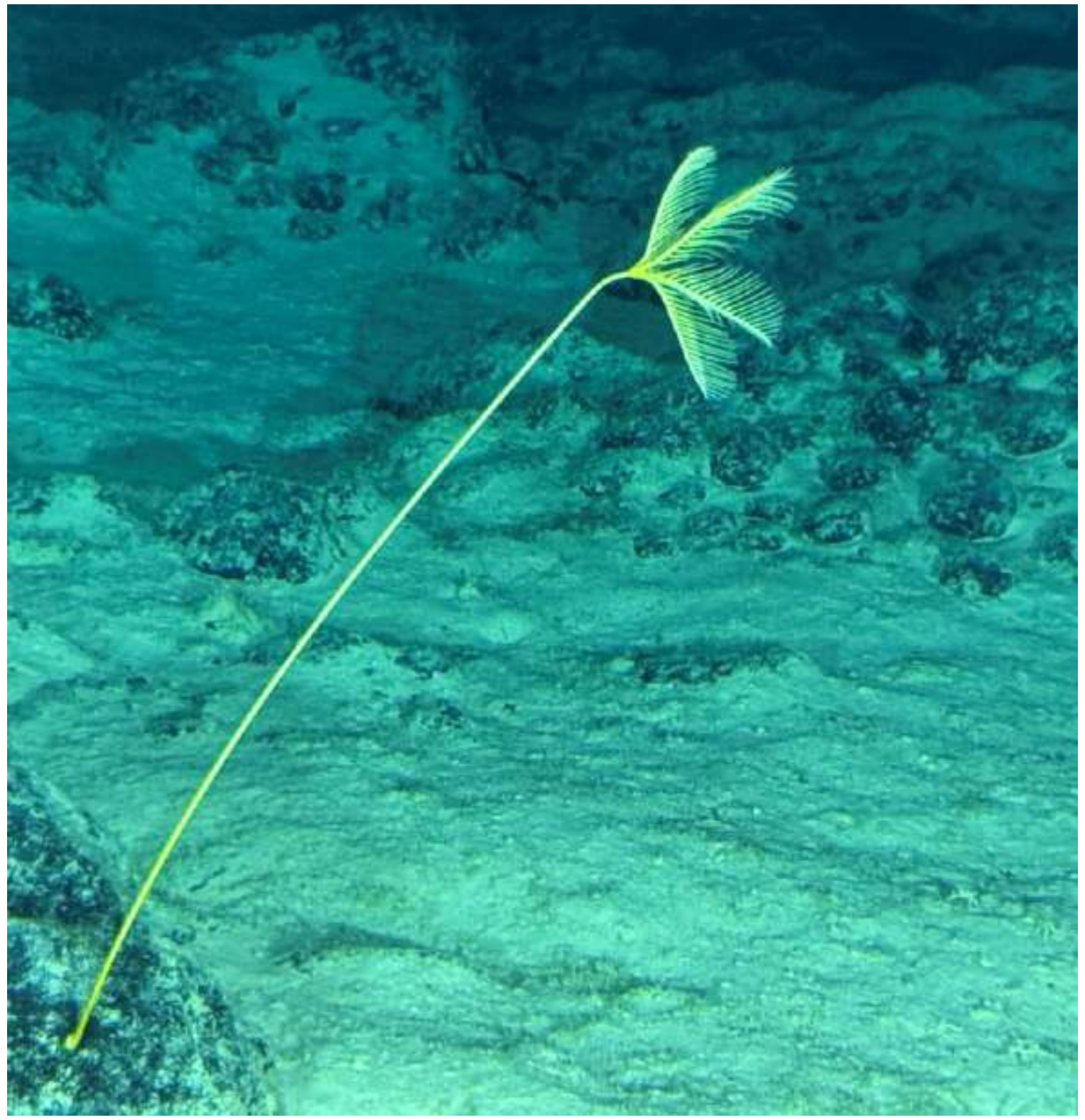

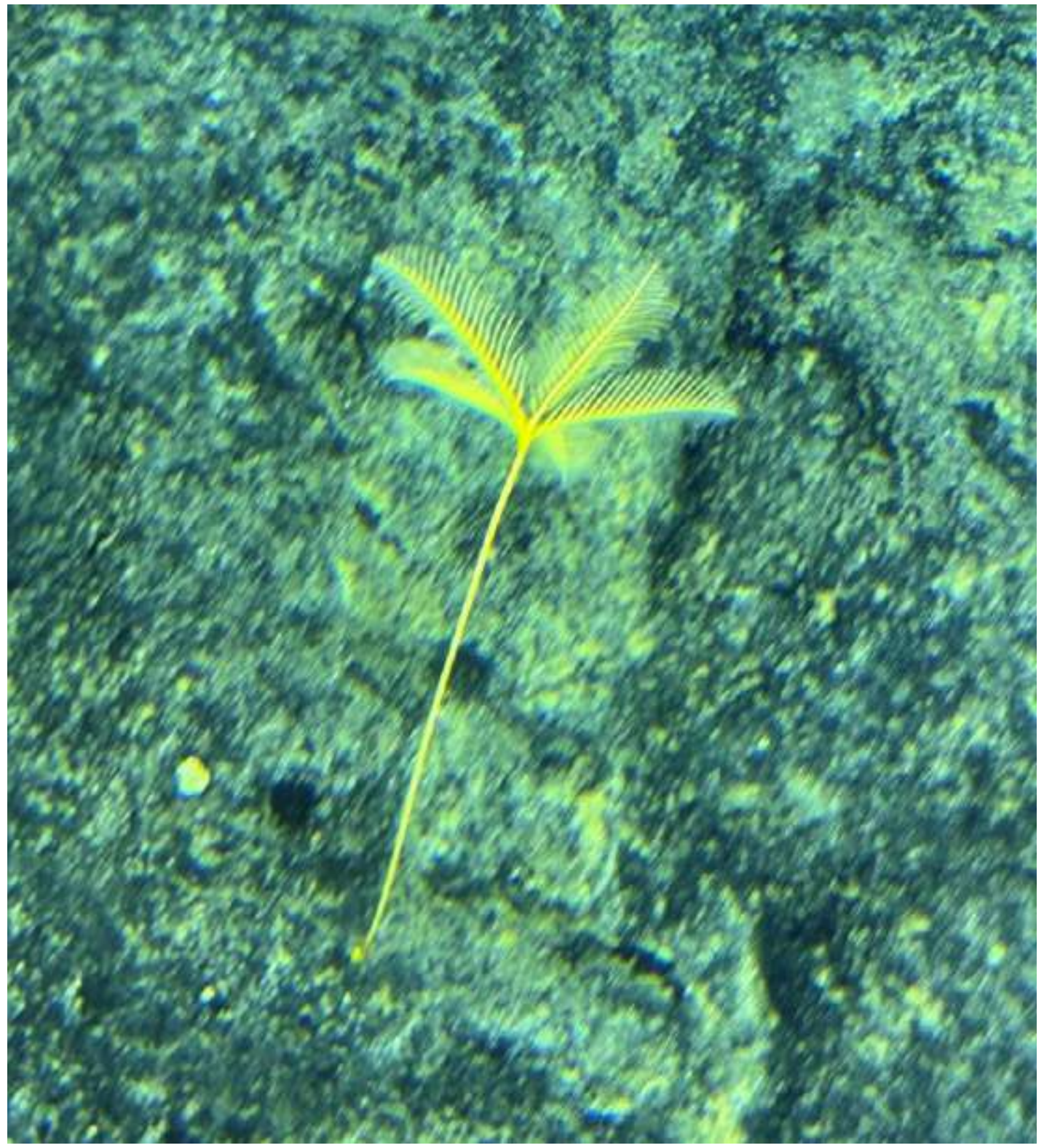

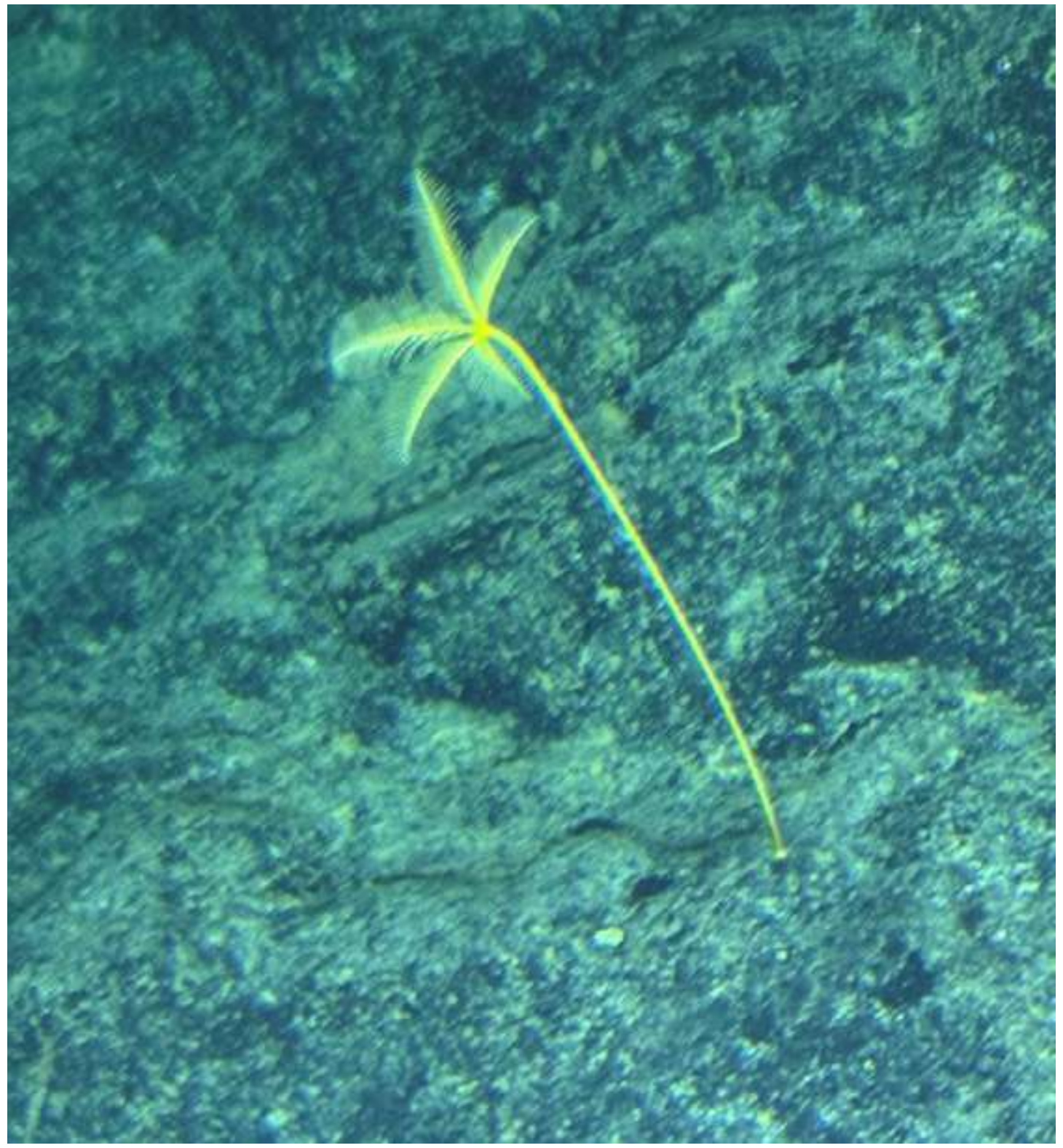

Yellow morphospecies with a homeomorphic long stalk with a cup-shaped theca. Five arms with medium-long thin and dense pinnules. Arm length is substantially shorter than the stalk; pinnules are proportionally much shorter than the arms. Hyocrinus?

Crinoidea; Hyocrinida; Hyocrinidae; Hyocrinidae gen. indet. 2

NC1_BK1_6200_IMG_4667

NC1_BK1_6200_IMG_4578

NC1_BK1_6200_IMG_4617

NC1_BK1_6200_IMG_4528

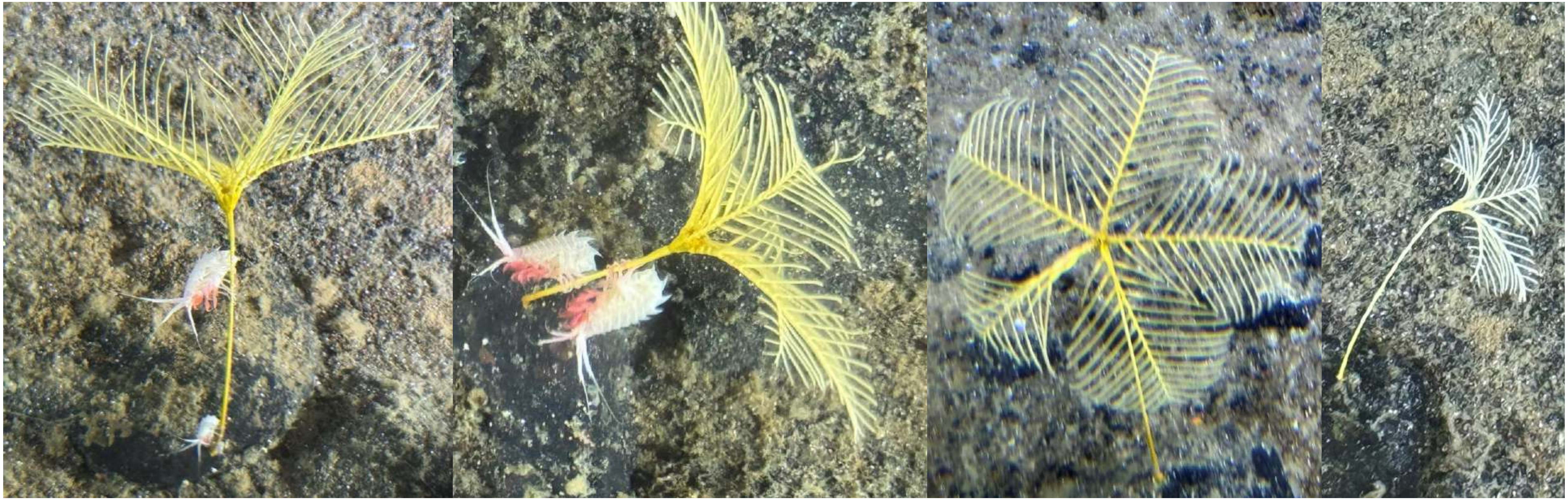

Yellow morphospecies with a homeomorphic medium stalk with a cup-shaped theca. Five arms with long pinnules. Arms are long between 1/3 and ½ of the stalk, while the pinnules cam get as long as almost half of the arm. Tiburocrinus *or* Parahyocrinus?

Crinoidea; Hyocrinida; Hyocrinidae; Hyocrinidae gen. inc. 3

NC1_BK1_6200_IMG_4444
NC1_BK1_6200_IMG_4264
NC1_BK1_6200_IMG_4268

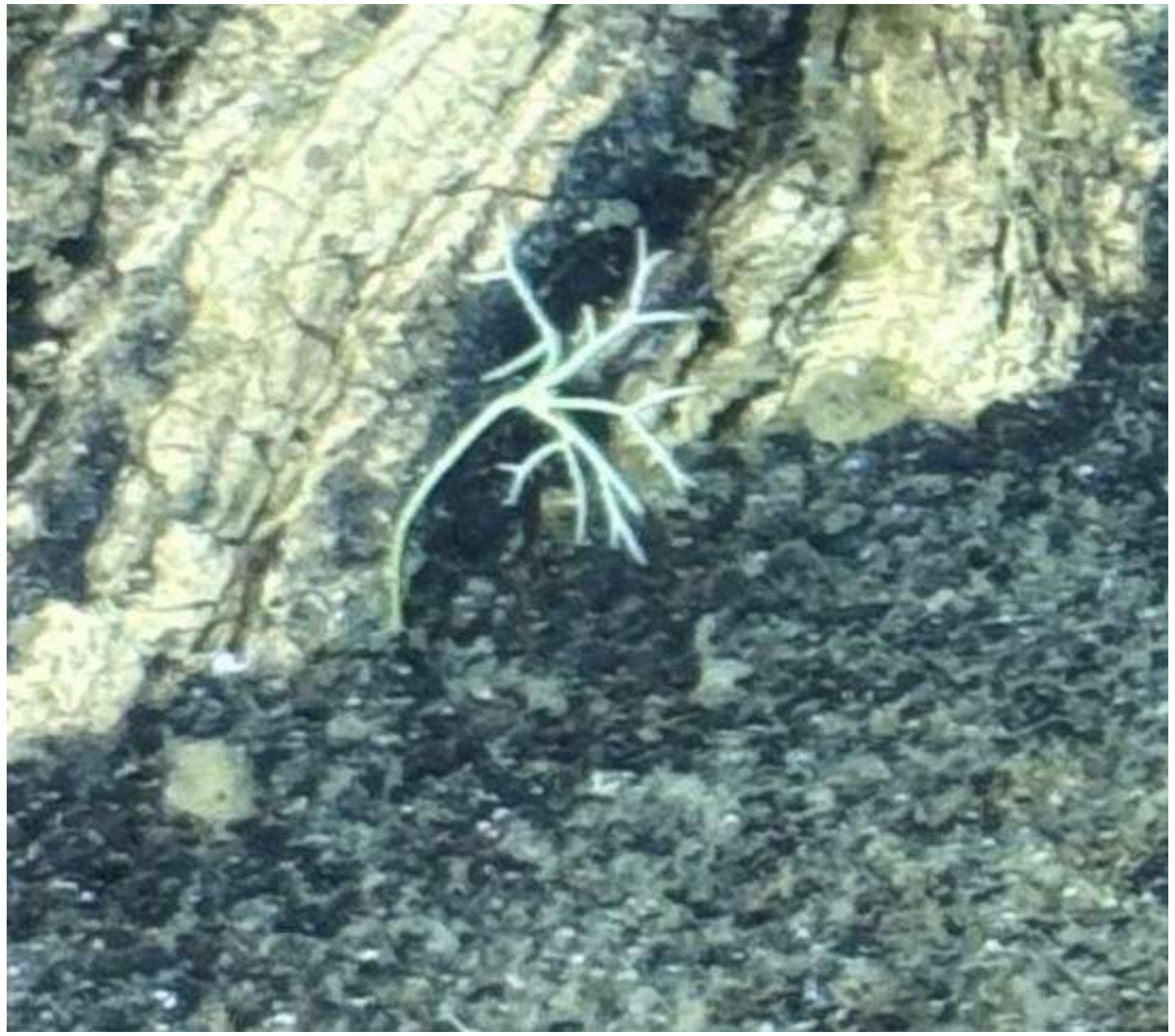
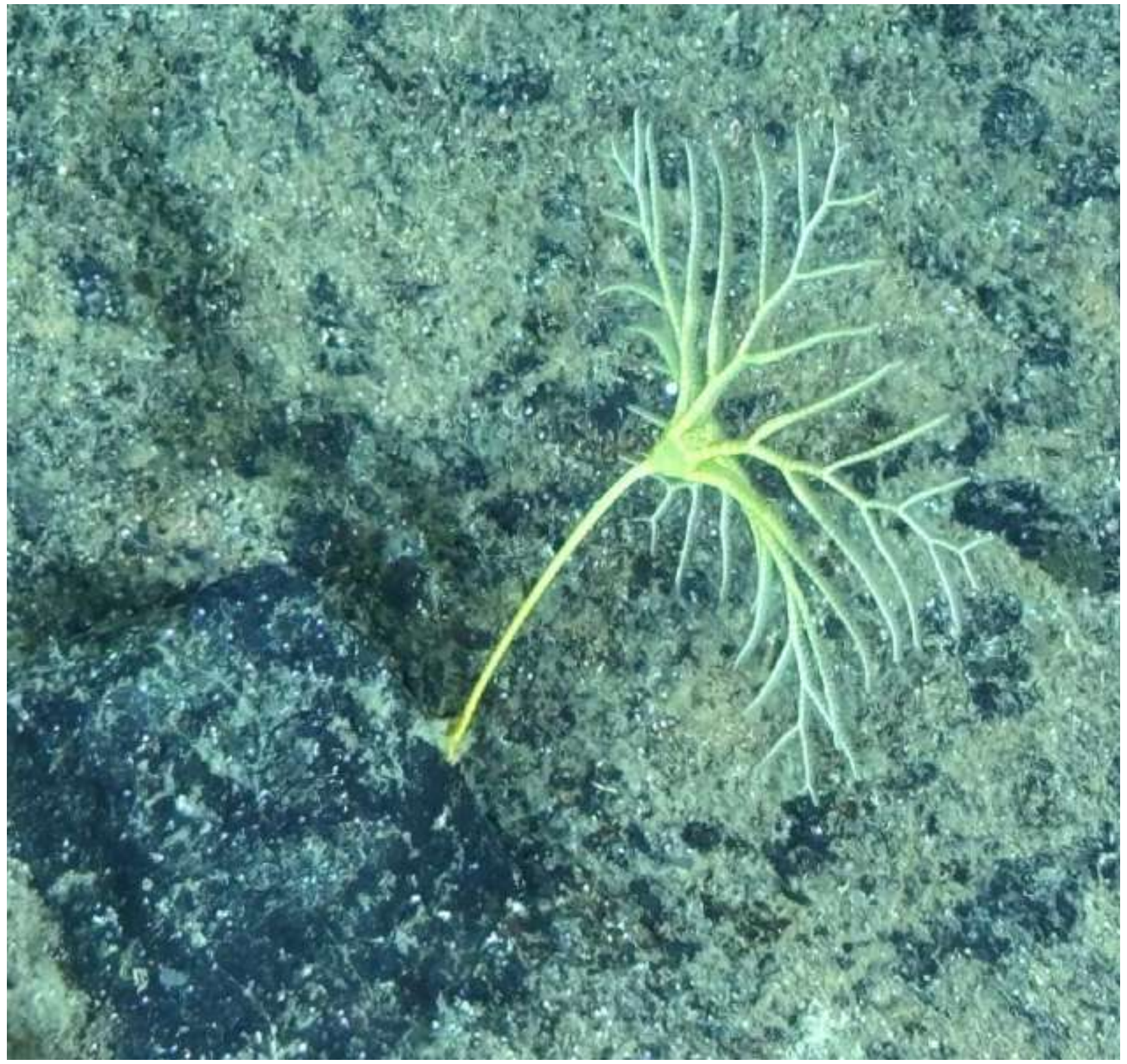
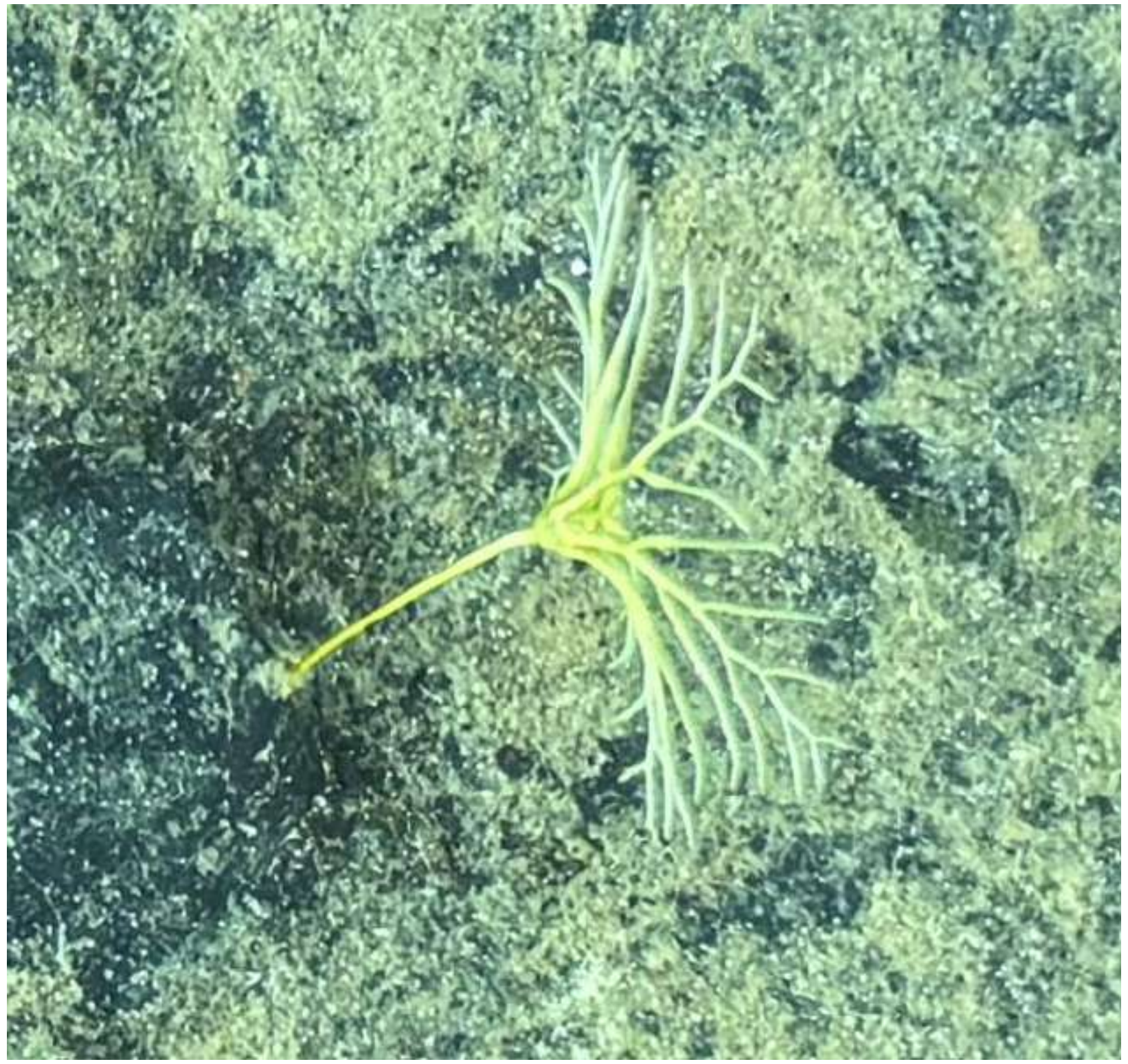

Yellow morphospecies with a homeomorphic medium stalk with a cup-shaped theca. Five arms that divide multiple times asymmetrically, each arm with sparce long pinnules. Likely a new species of Calamocrinus.

Left pic is a possible juvenile. SOME ONE WITH A MAC PLEASE RETRIEVE AN IPHONE PICTURE FROM NC3_BK1

Crinoidea; Hyocrinida; Hyocrinidae; Hyocrinidae spp.

NC1_BK1_6200 – NOR_00XX

NC1_BK1_6200 – NOR_0093

NC3_BK1_8000 – NOR_0094

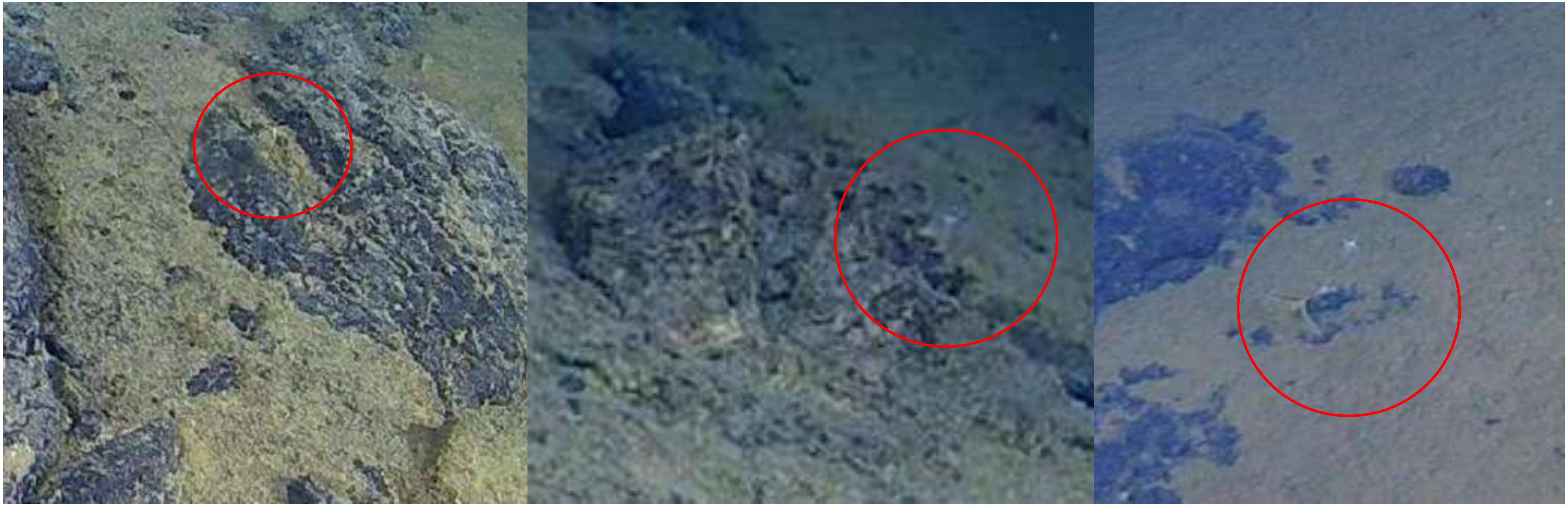

Yellow morphospecies with a short/medium stalk and five distinguishable arms. Pinnules not observed clearly enough in the picture so it is not possible to differentiate between sp. 2 and sp. 3

Crinoidea
Crinoidea spp.

NC1_BK1_6200_XX

NC1_BK1_6200_NOR_0101

NC4_BK1_5600 – NOR_0060

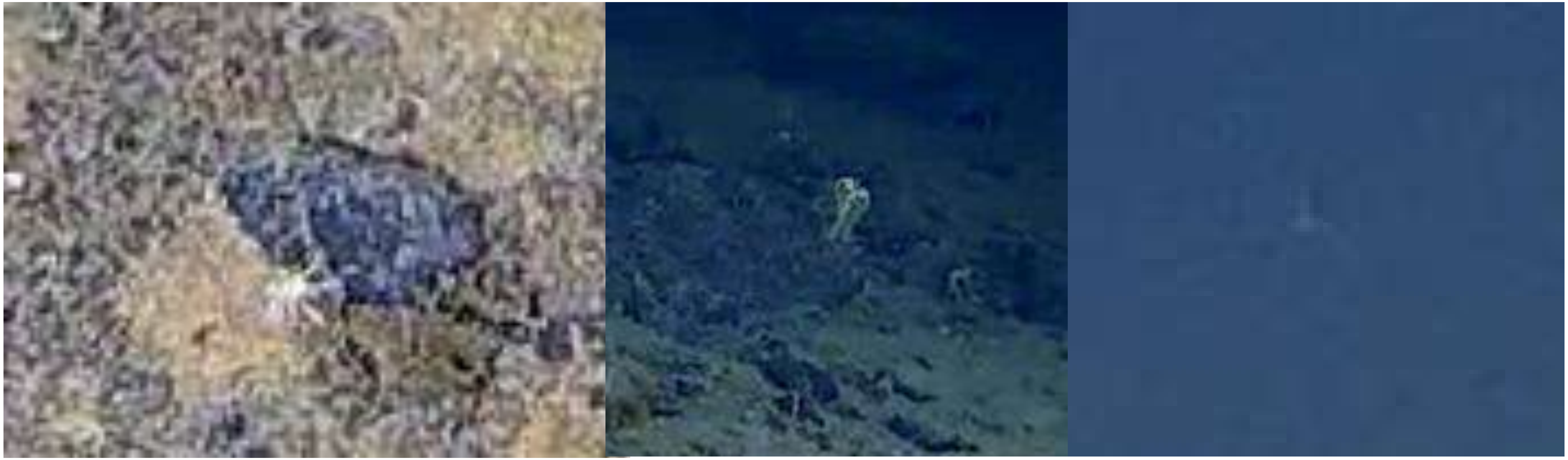

Crinoid but no distinguishing features visible that would allow for lower category classification, e.g. because too small to see (for example in dive NC1_BK1_6200).

Note: all crinoids in NC4_BK1_5600 don't have enough resolution

NC3_CR1_4400_NOR_0098

Echinoidea; Aspidodiadematoida; Aspidodiadematoida fam. indet. 1

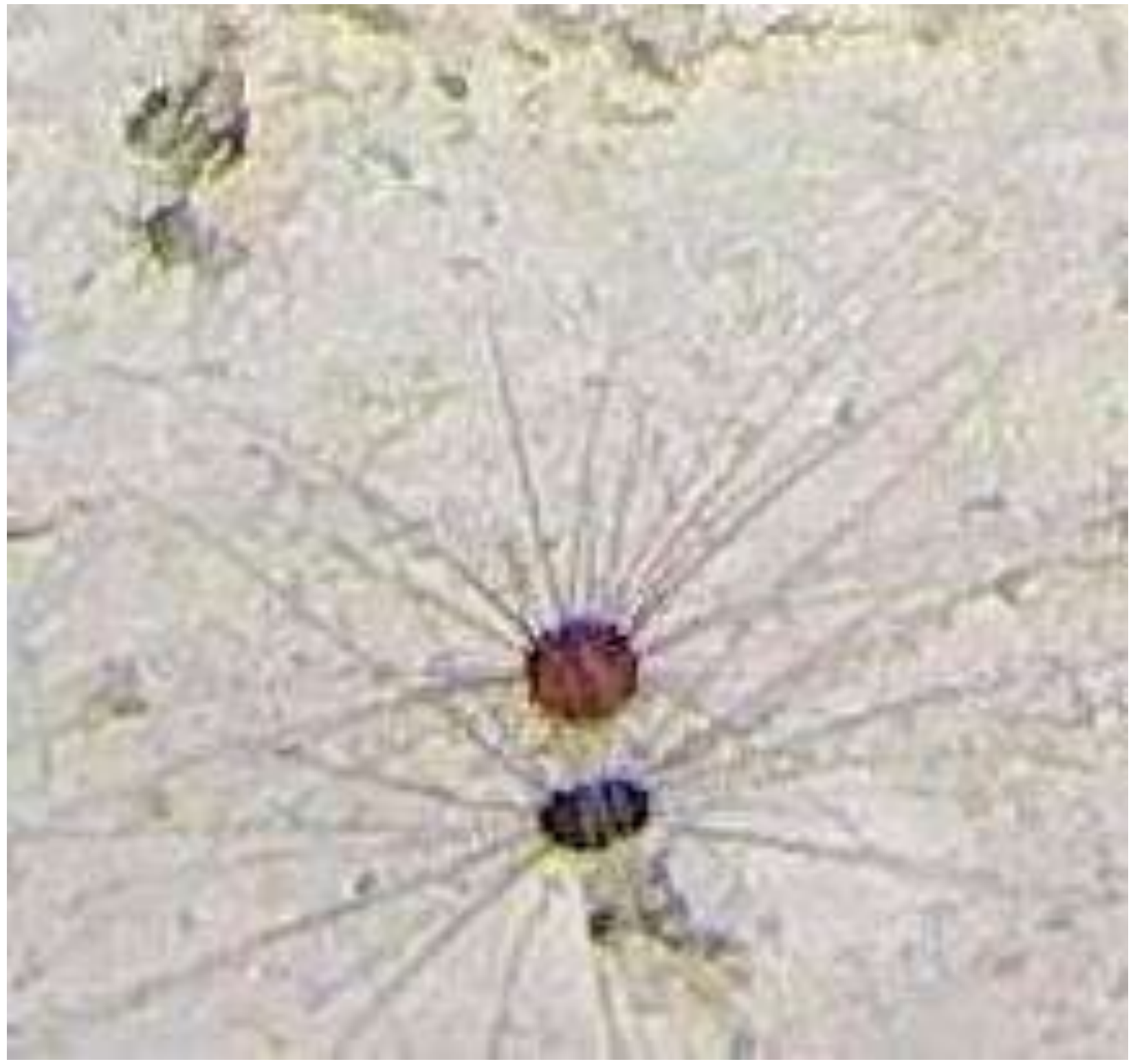

Round, fragile, dark red coloured body with thin, long and flexible primary spines, usually bent towards the seafloor.

Echinoidea; Cidaroida; Histocidaridae; *Histocidaris* sp. indet. 1

NC2_BK2_3800_IMG_7849
NC2_BK2_3800_IMG_7850
NC2_BK2_3800_IMG_7789

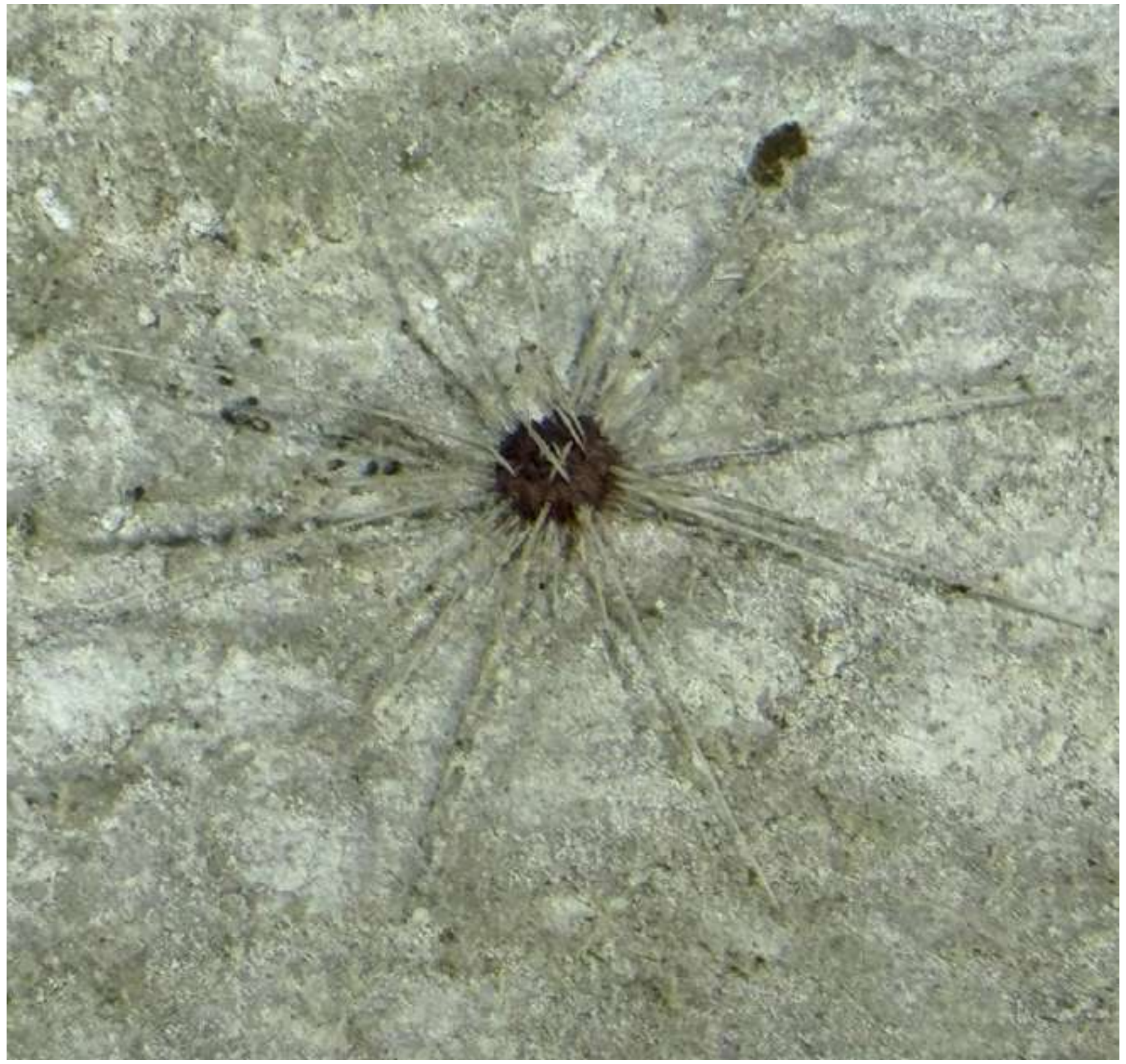
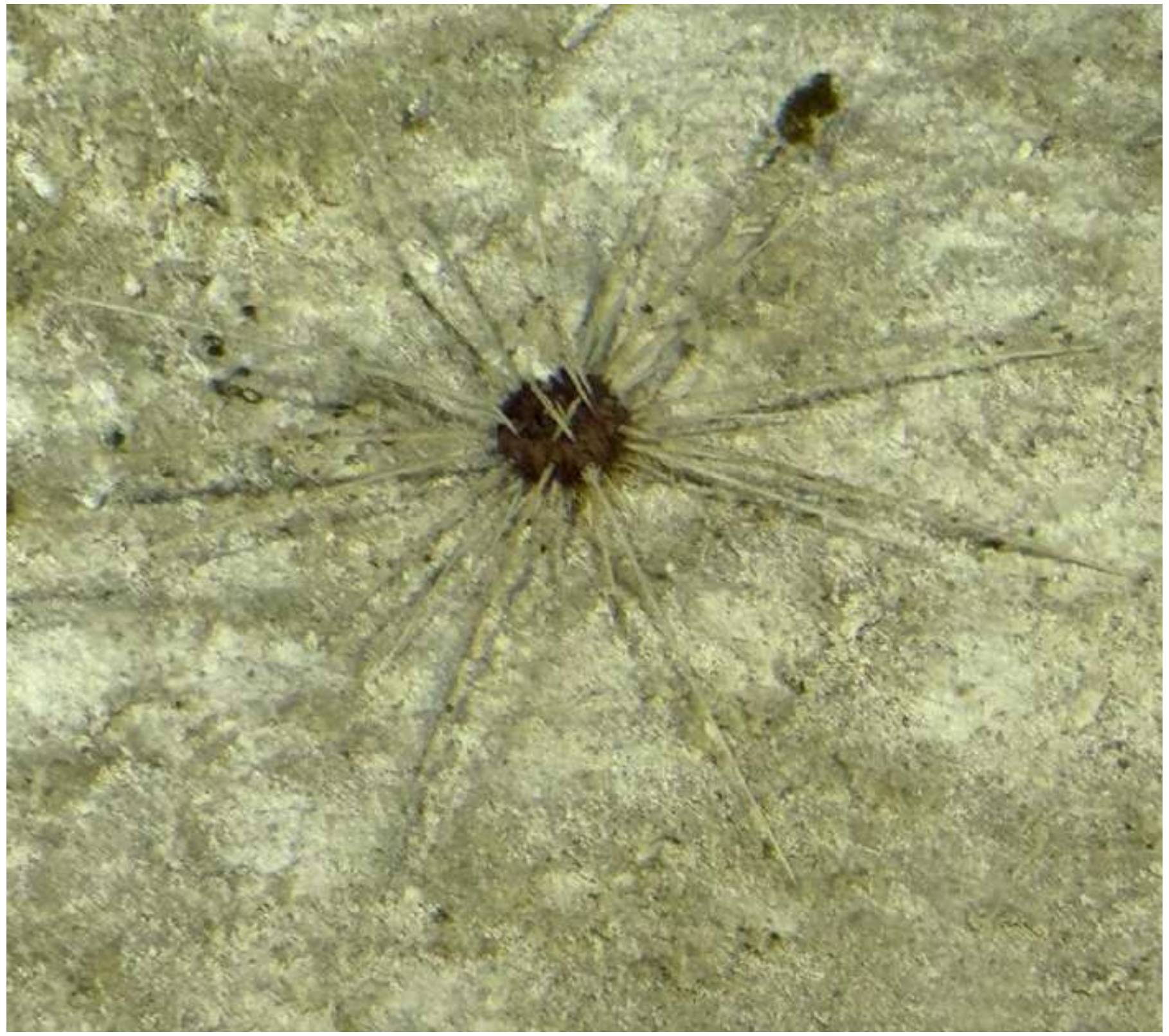
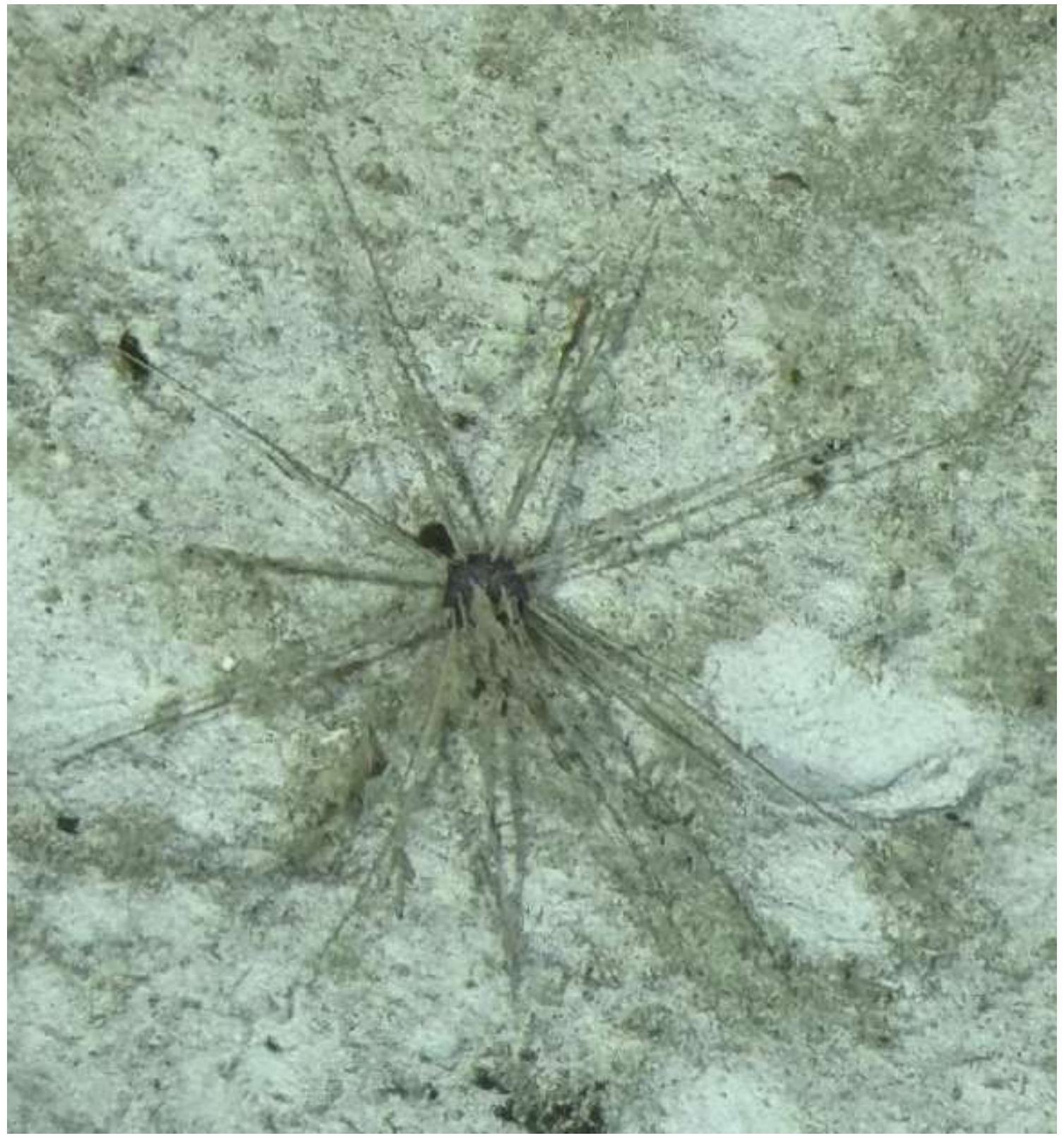

Round, dark coloured body with large, thick primary spines. Spines are rigid and almost do not bend under their own weight.

Echinoidea; Echinothurioida ; Echinothurioida fam. indet. 1

NC3_BK2_6000_NOR_0062

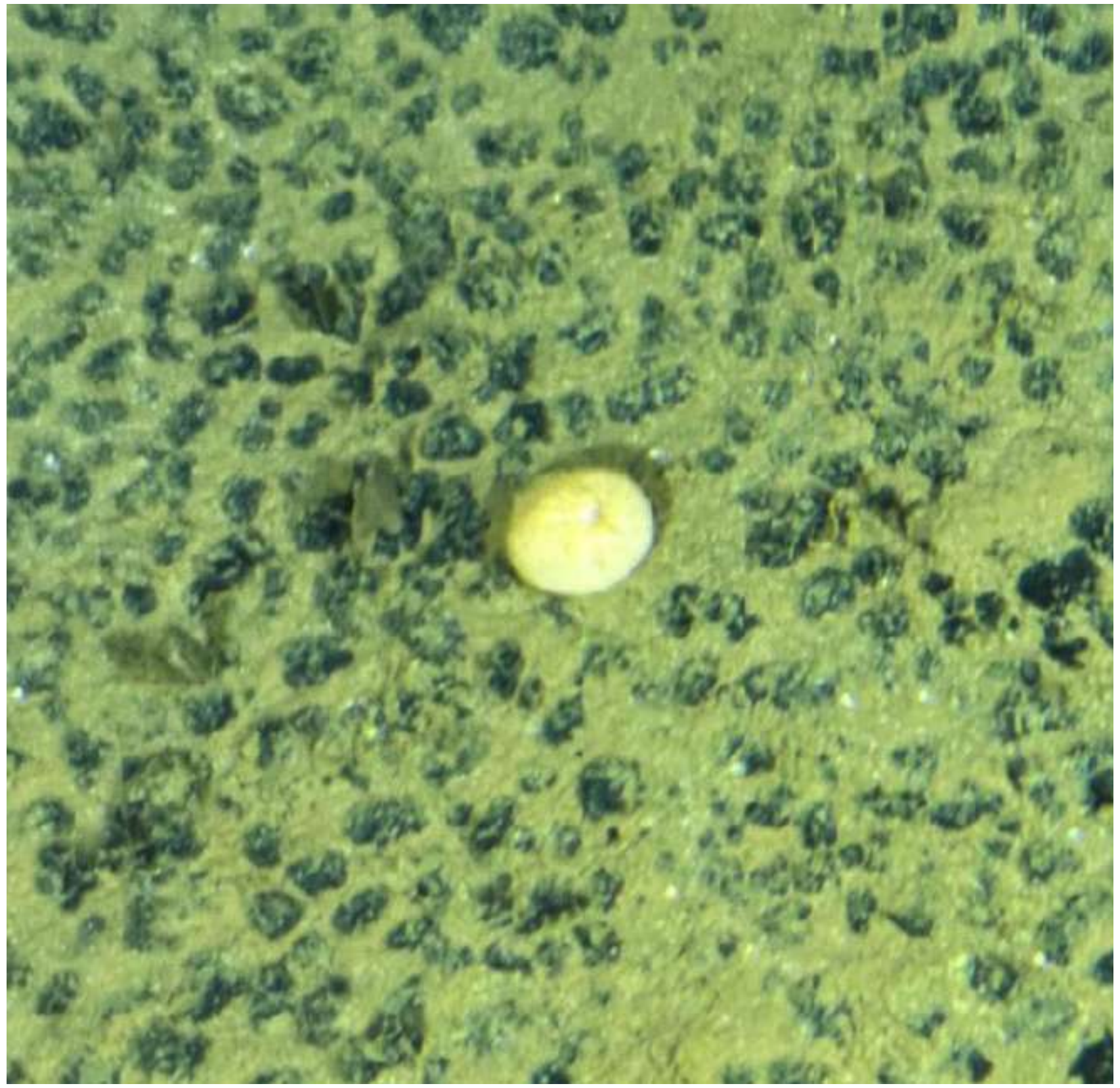

Body is rounded and with light tan colouration.

Mollusca

Cephalopoda; Octopoda; Octopoda fam indet.

NC4_MA3_4550_NOR_0060

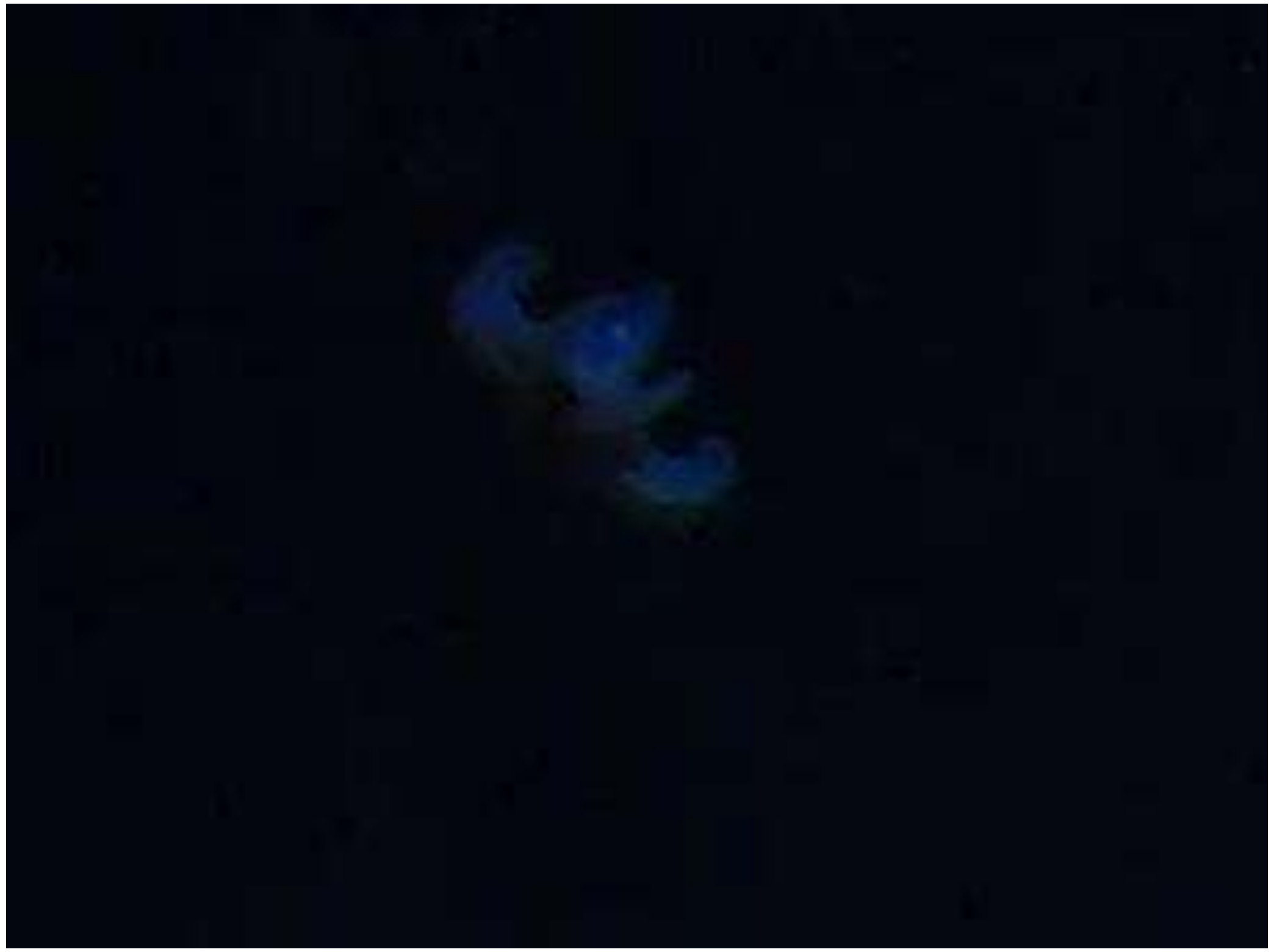

Body is dark in colour with. Fin-like extensions near the mantle are rounded. Robust tentacles lack apparent webbing and a held in curled position while moving through the water column.

Behavioural Diagnostic: Suspended drifting in a posture like an open umbrella, with arms and web outspread, distal ends slightly curved upon the aboral surface.

Cephalopoda; Octopoda; Cirroteuthidae; *Cirroteuthis; Cirroteuthis* sp. indet. 1

NC3_BK3_6000_IMG_2591
NC3_BK3_6000_NOR_0062
NC3_BK2_6000_NOR_1405

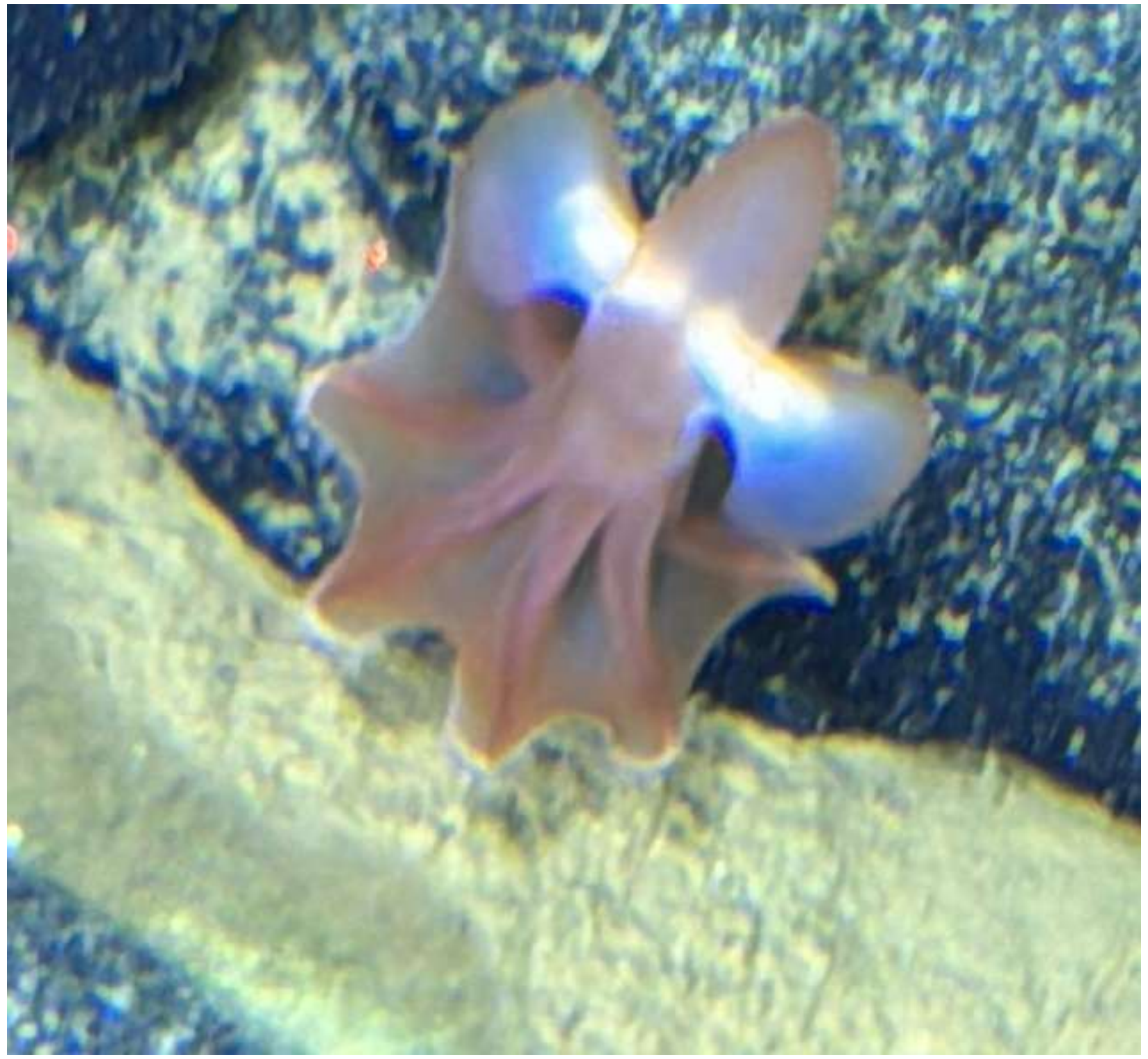
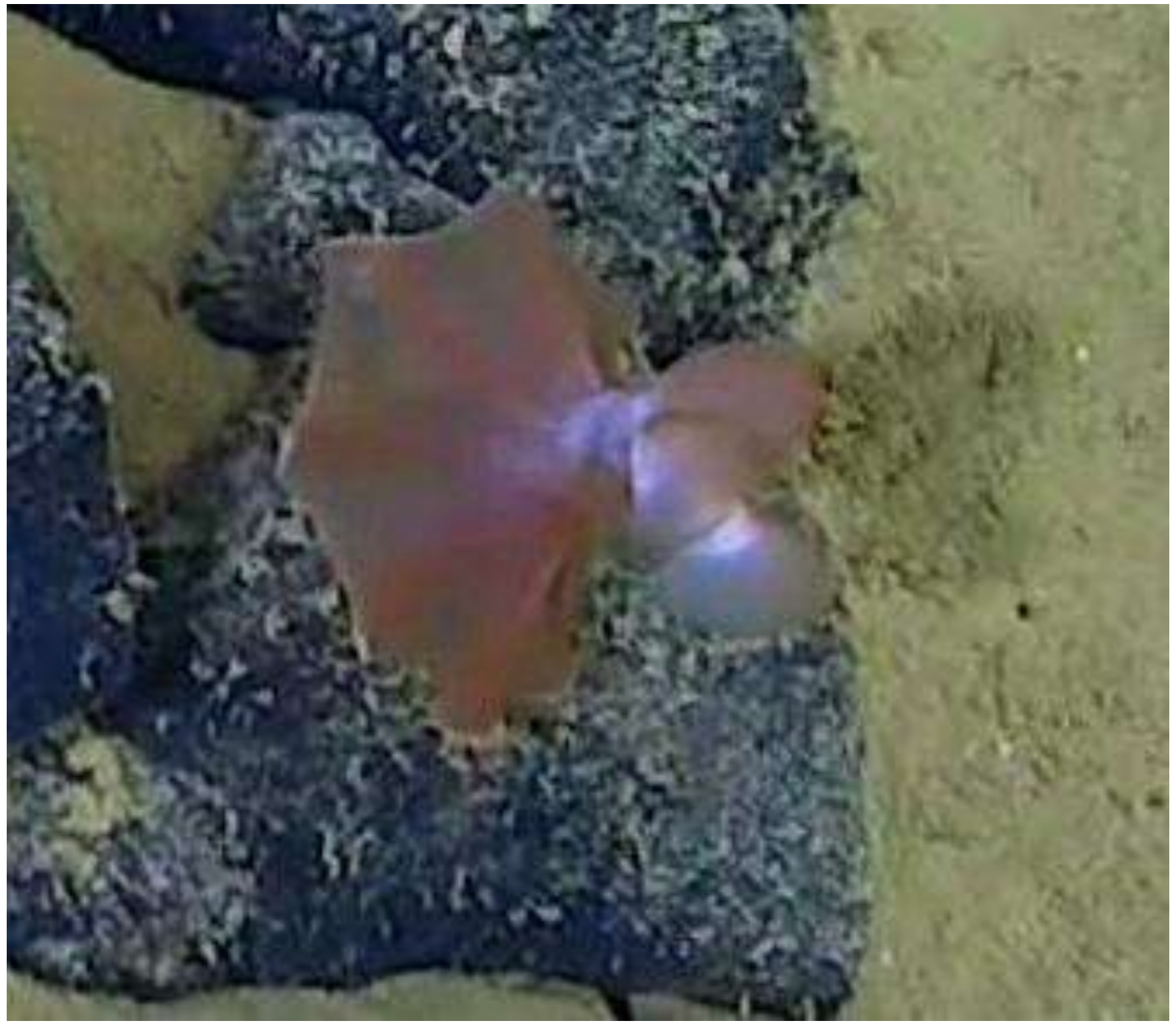
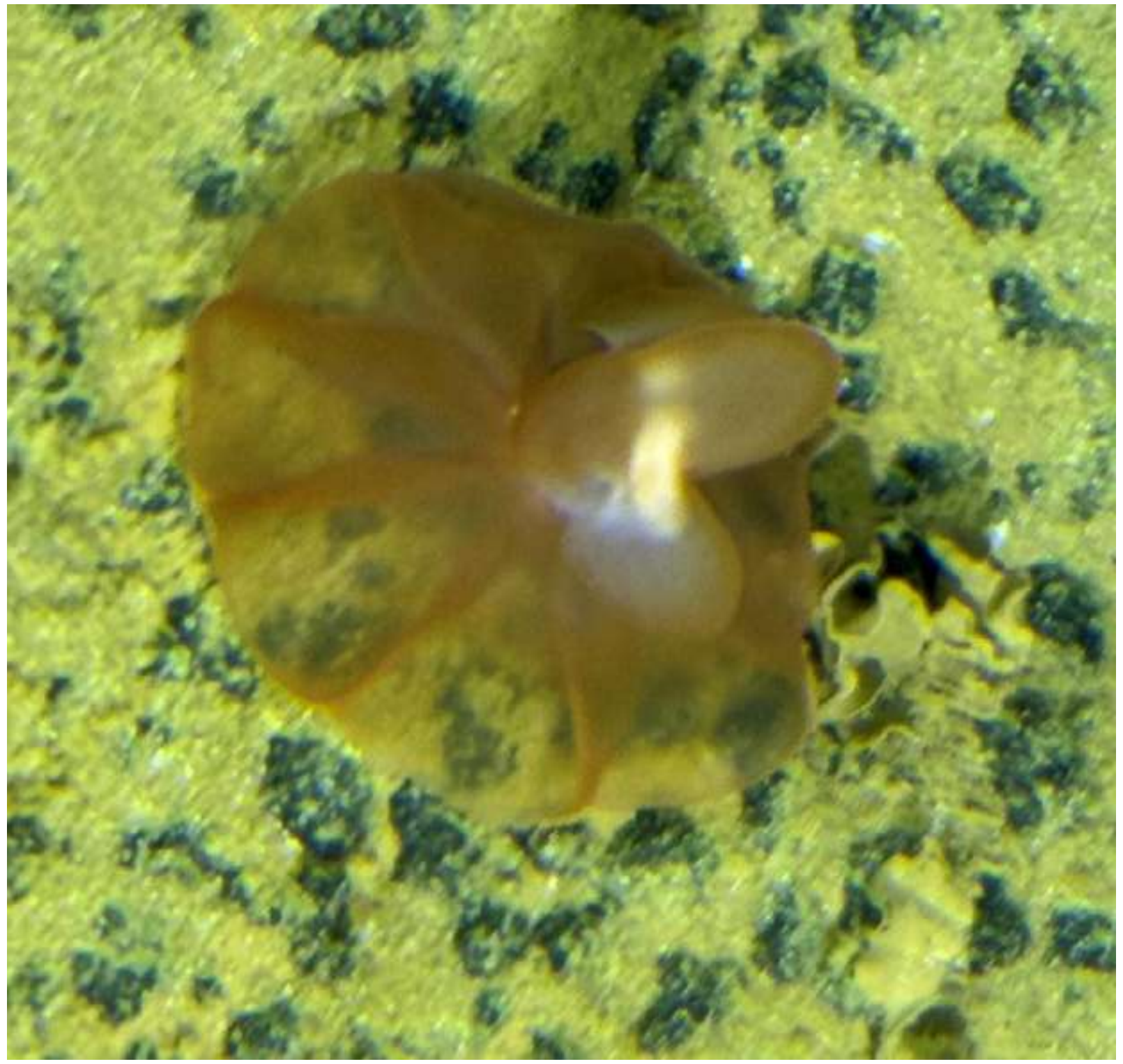

Body is semi-transparent and reddish purple in colour with pale coloration near the fin-like extensions. Fin-like extensions near the mantle are rounded. Short tentacles are webbed in semi-transparent skin. Resembles mulleri

Mollusca; Gastropoda; Neogastropoda; Buccinidae; Buccinidae spp

NC3_MA5_6000_NOR_0078
NC2_BK3_7400_IMG_8850
NC3_BK2_6000_IMG_2241

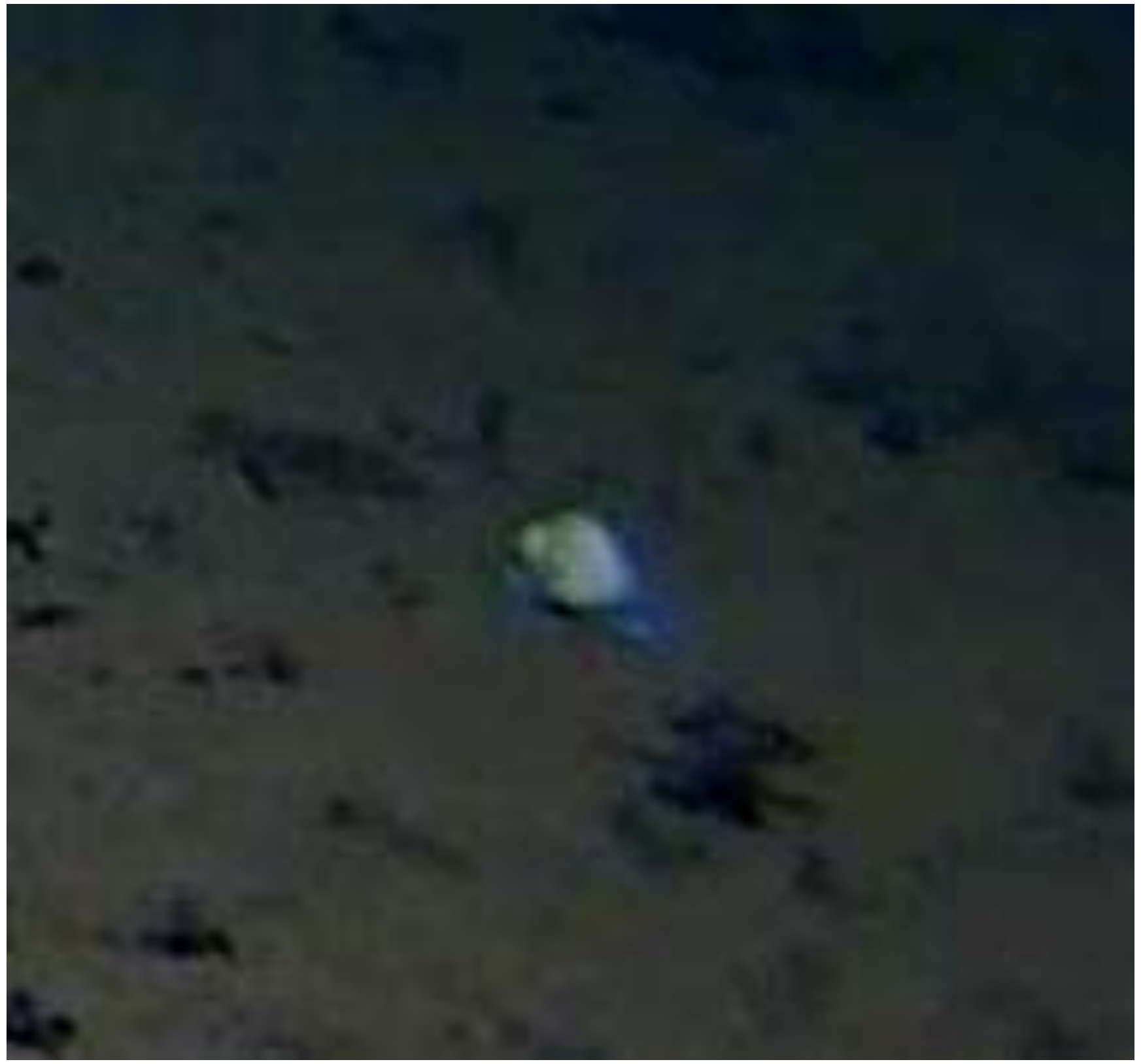
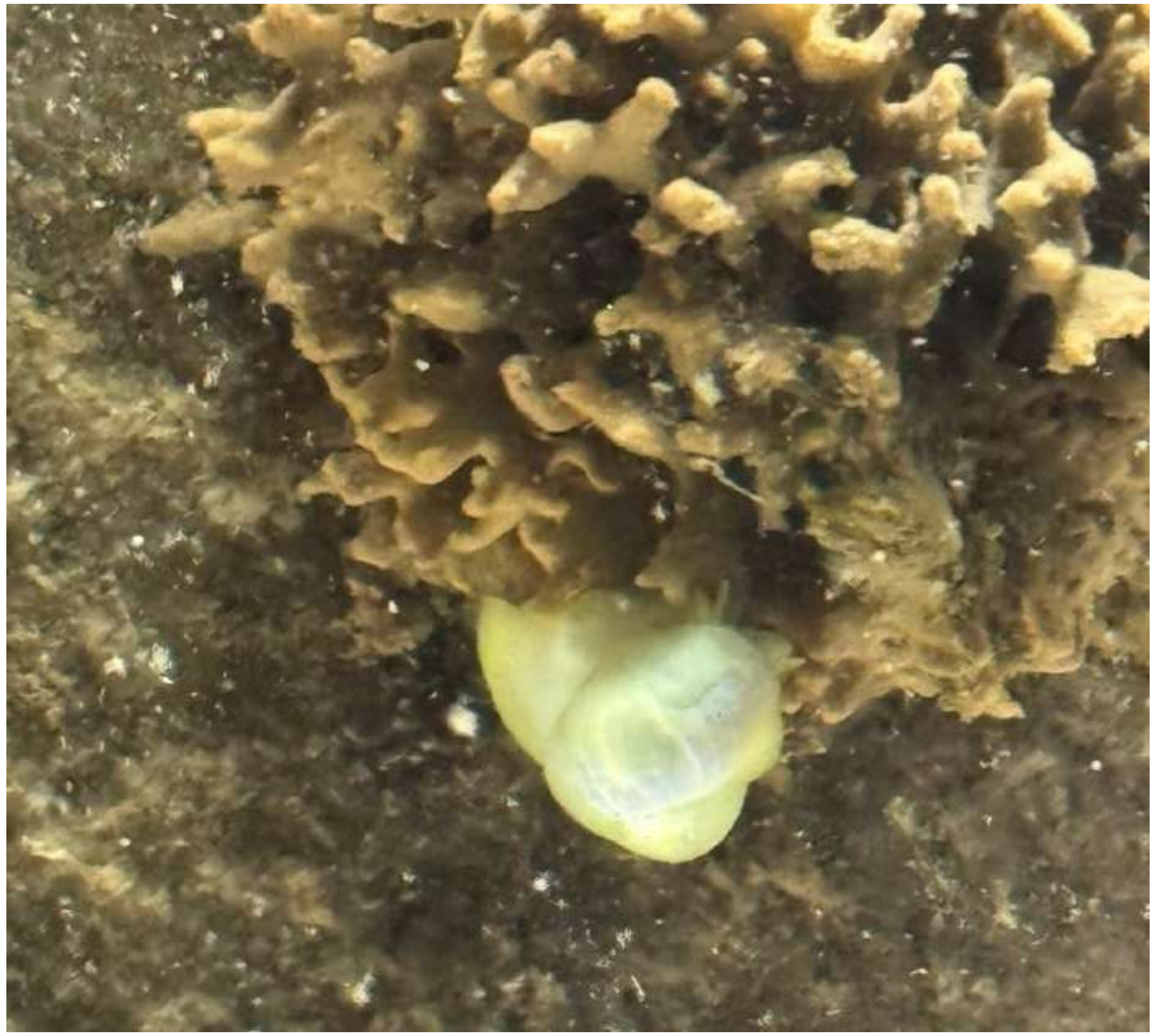
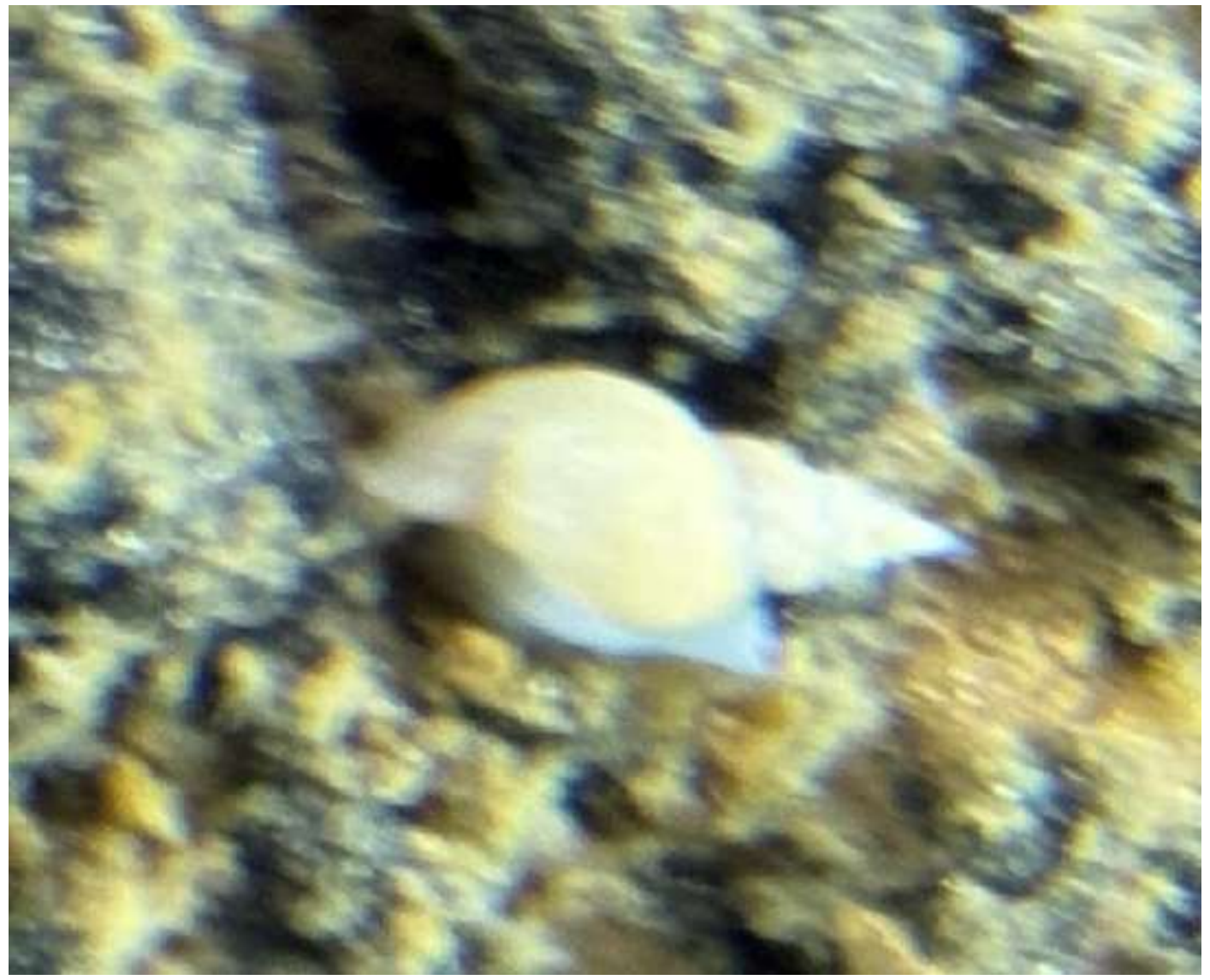

Foot appears pale. Spiralling shell is of pale yellow in colouration.

Mollusca; Gastropoda; Neogastropoda; Neogastropoda fam. indet. 1

NC2_BK1_3200_IMG_7631

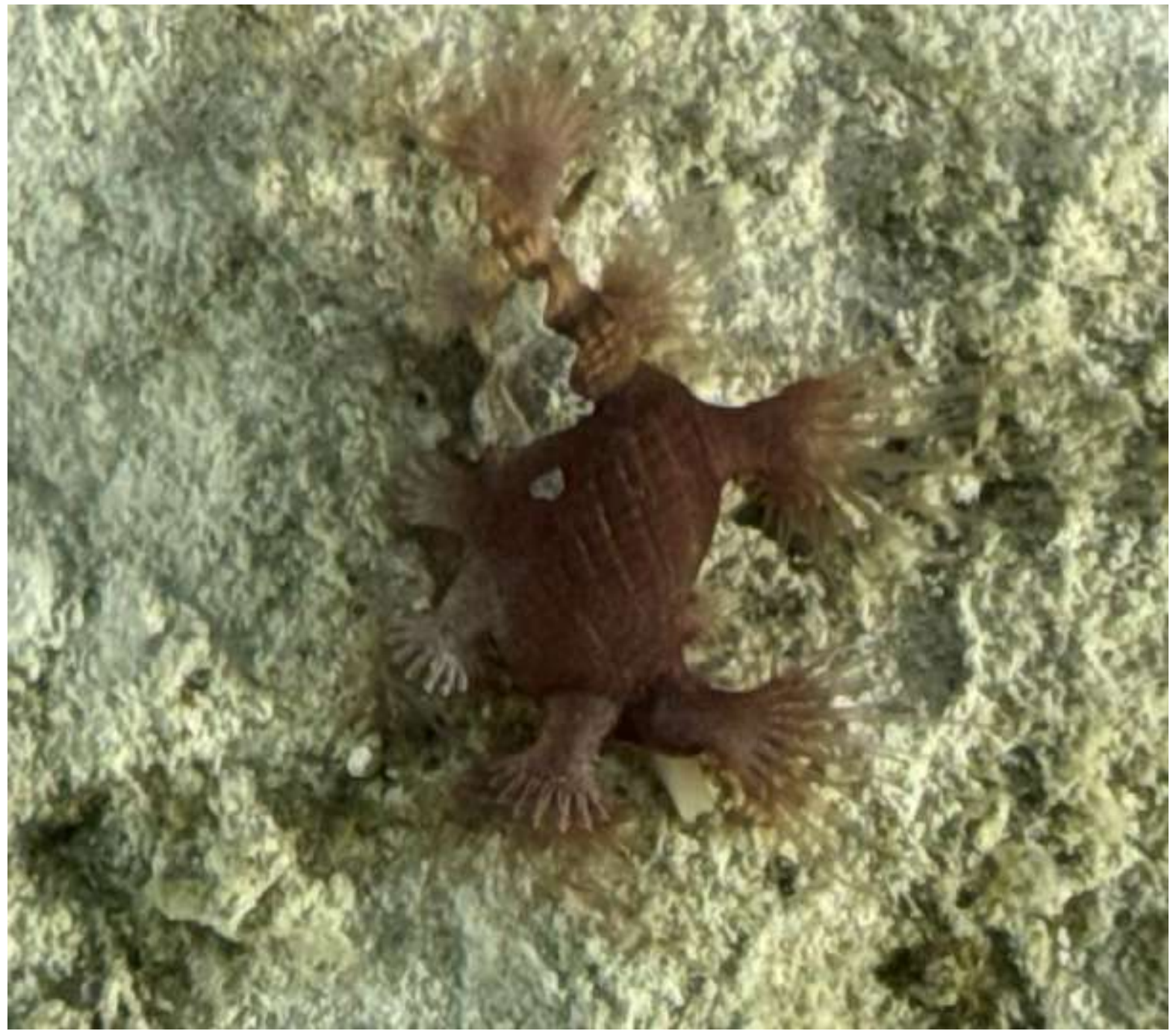

Siphon is pale in colouration. Spindle shaped shell is of reddish-brown colouration consisting of 5 whorls. Behavioural diagnostic: Shell is covered in zooanthids.

Brachiopoda

*NC3_BK2_6000_NOR_0059.avi*_0007047

NC3_BK2_6000_6_01_NOR_0065.avi_0007665

Brachiopoda ord. indet.1

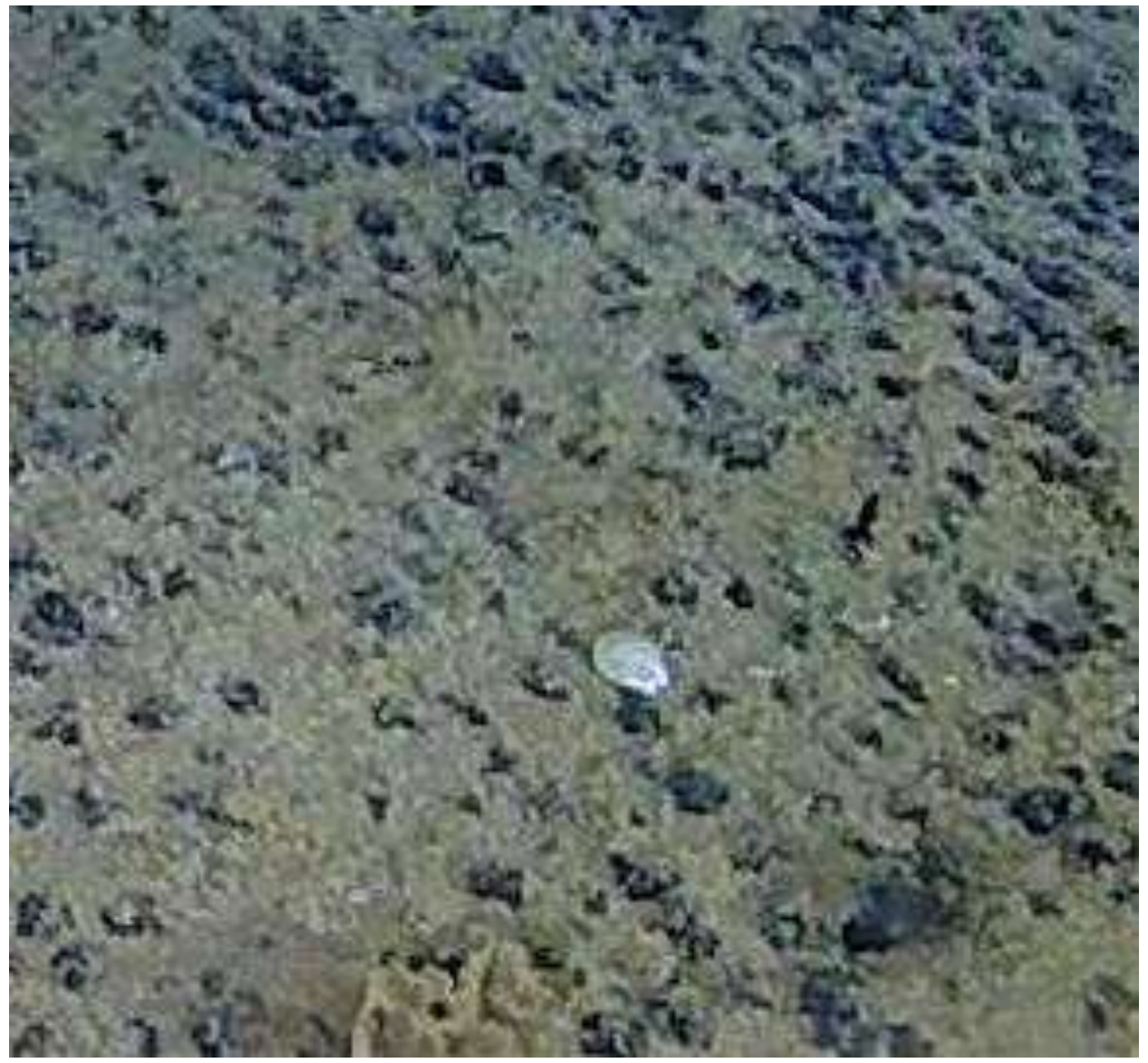

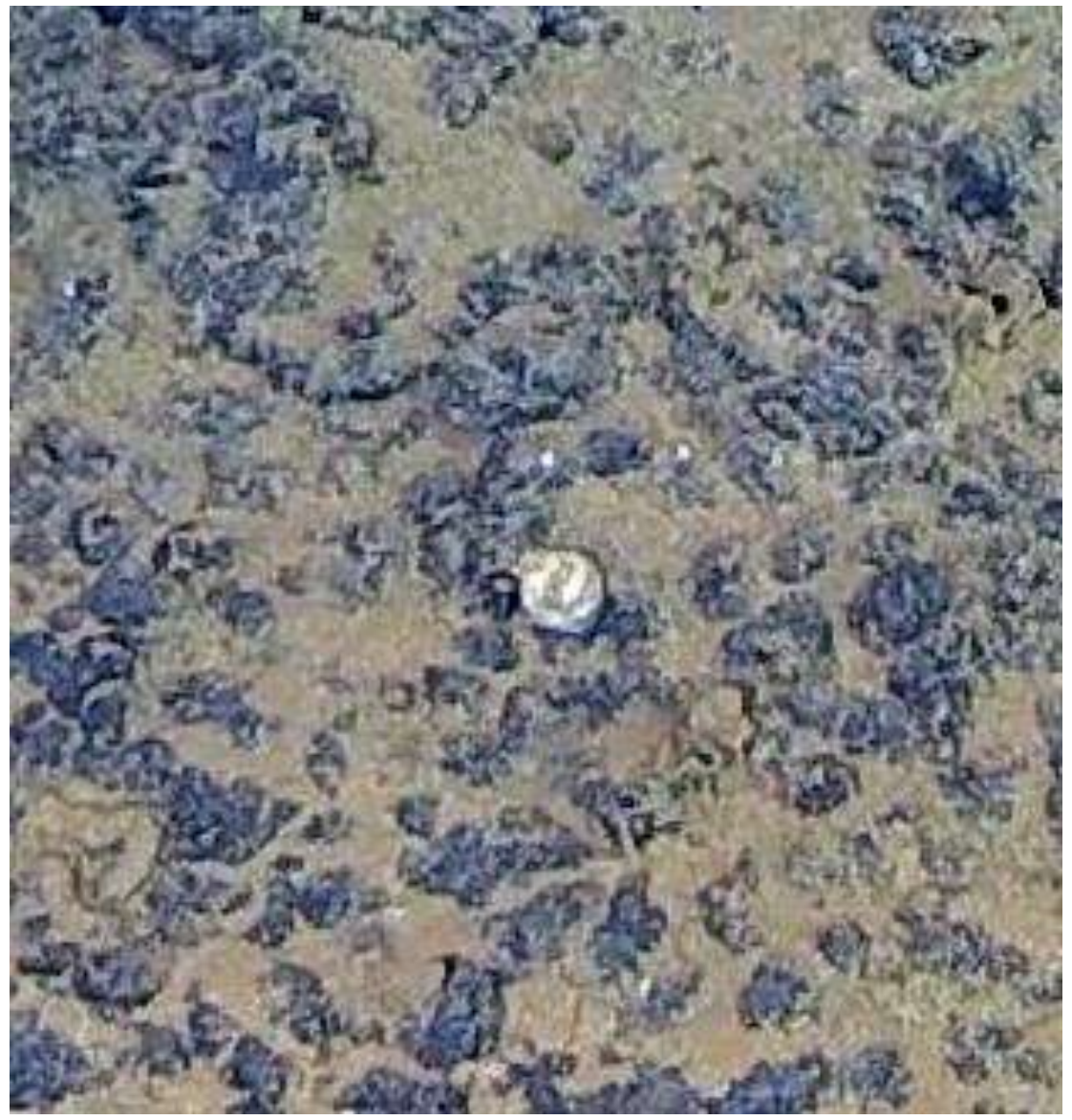

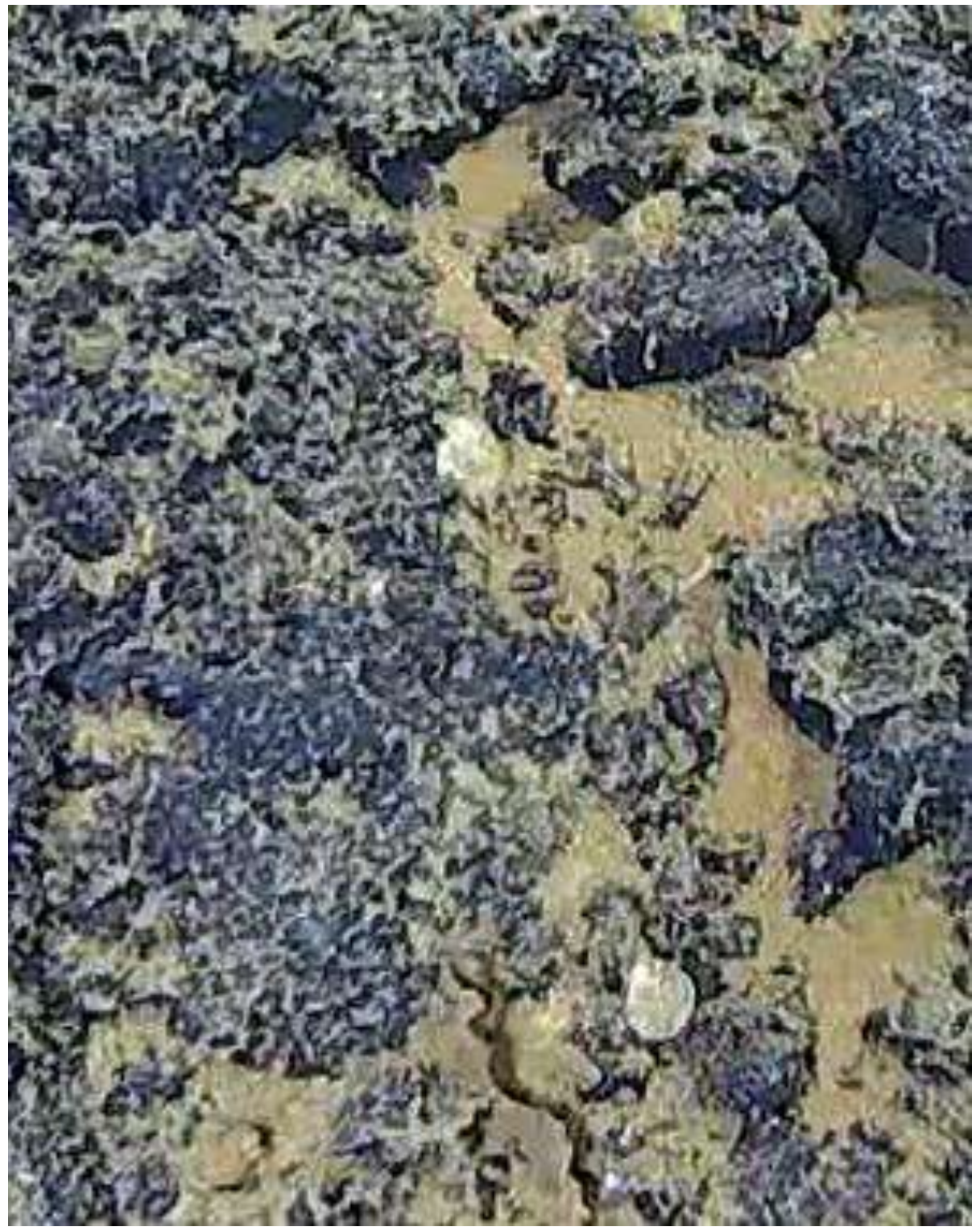

Convex to flat bivalve shell, round-ish to triangular shape, often seen light to white colour and at times covered in sand. Aff. Rhynchonellata

Arthropoda

Copepoda; Copepoda spp.

NC1_OM2_6000_NOR_0110
NC1_OM1_6300_NOR_0115
NC2_CR4_4100_NOR_0045

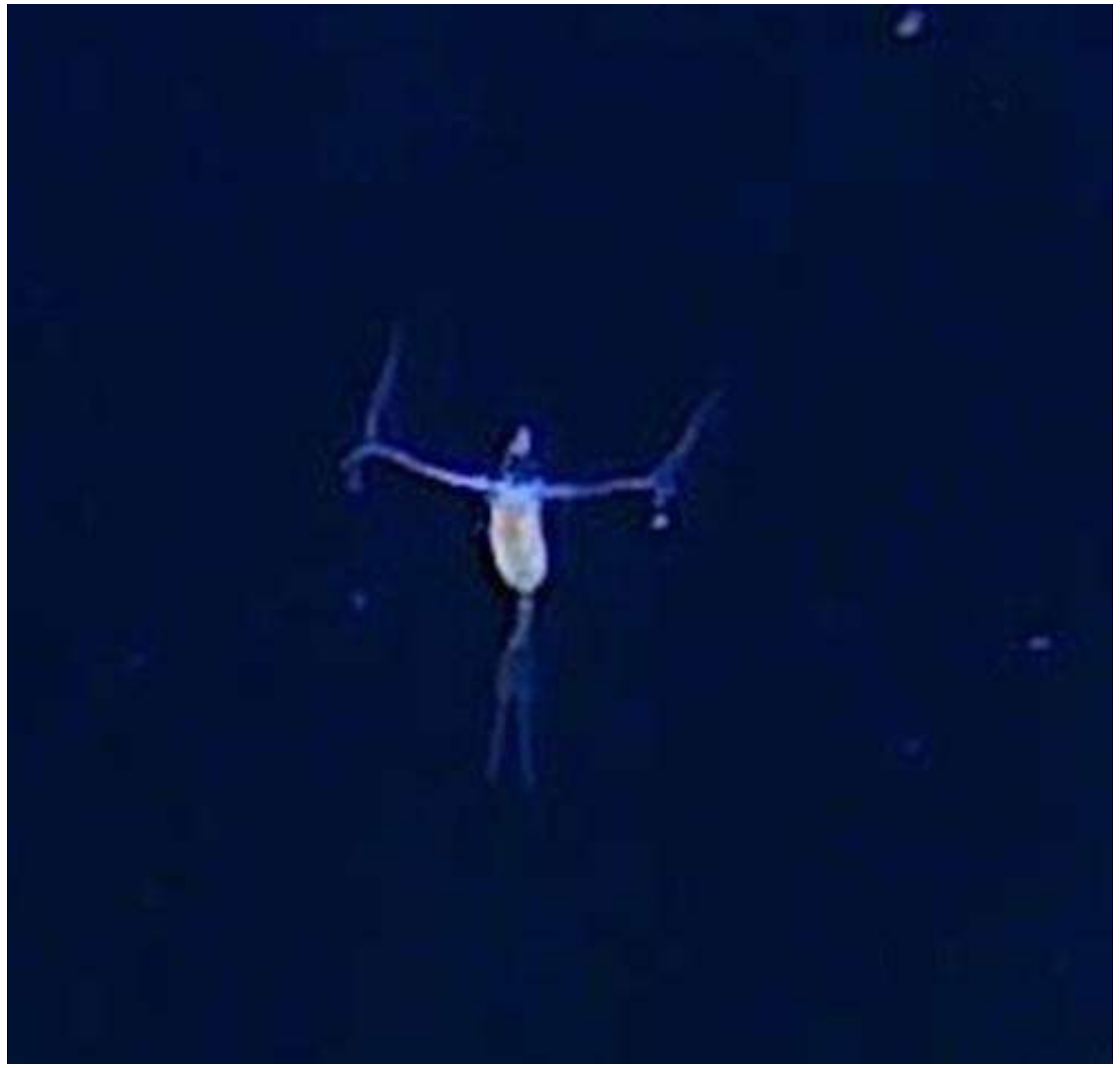
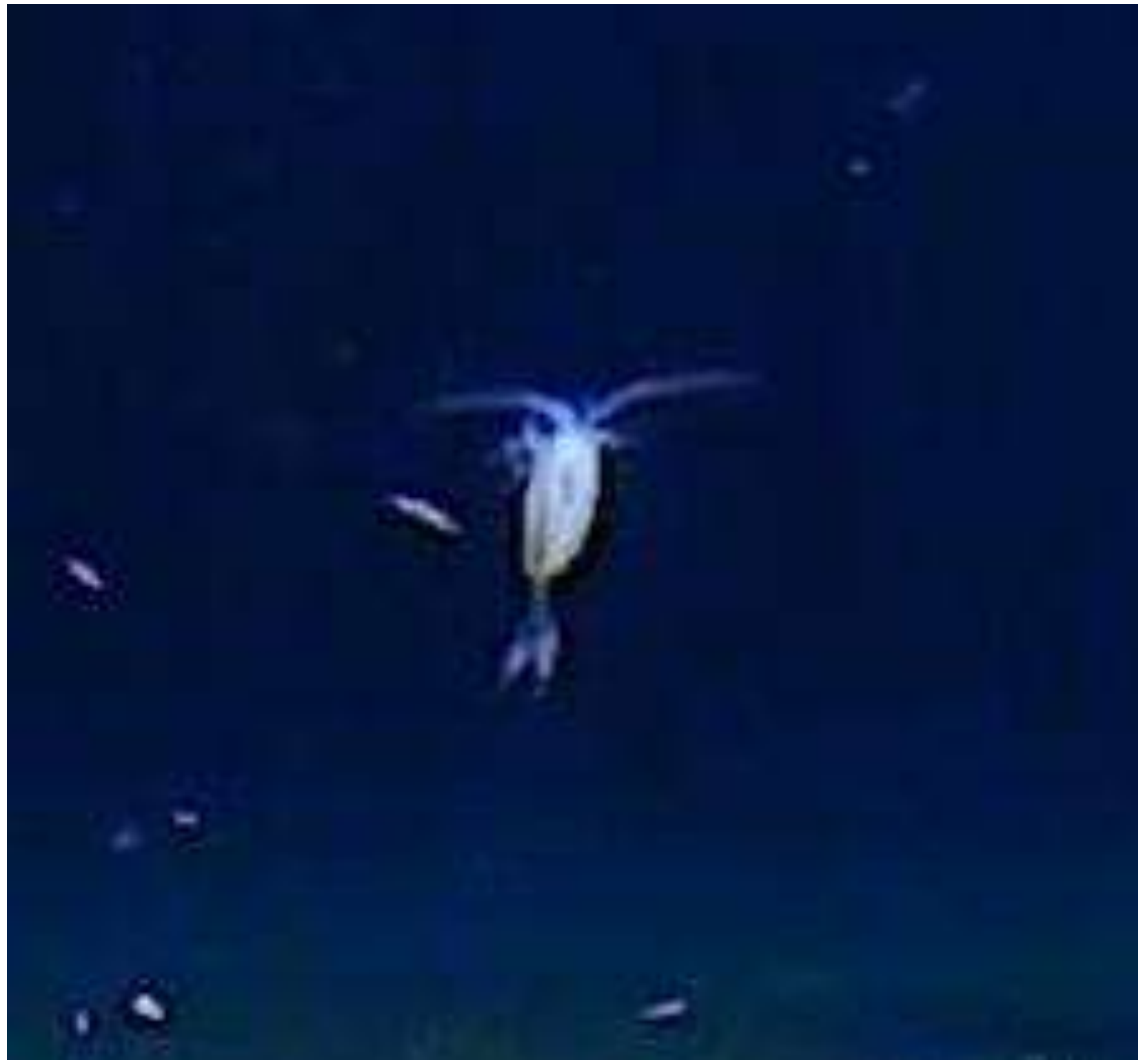
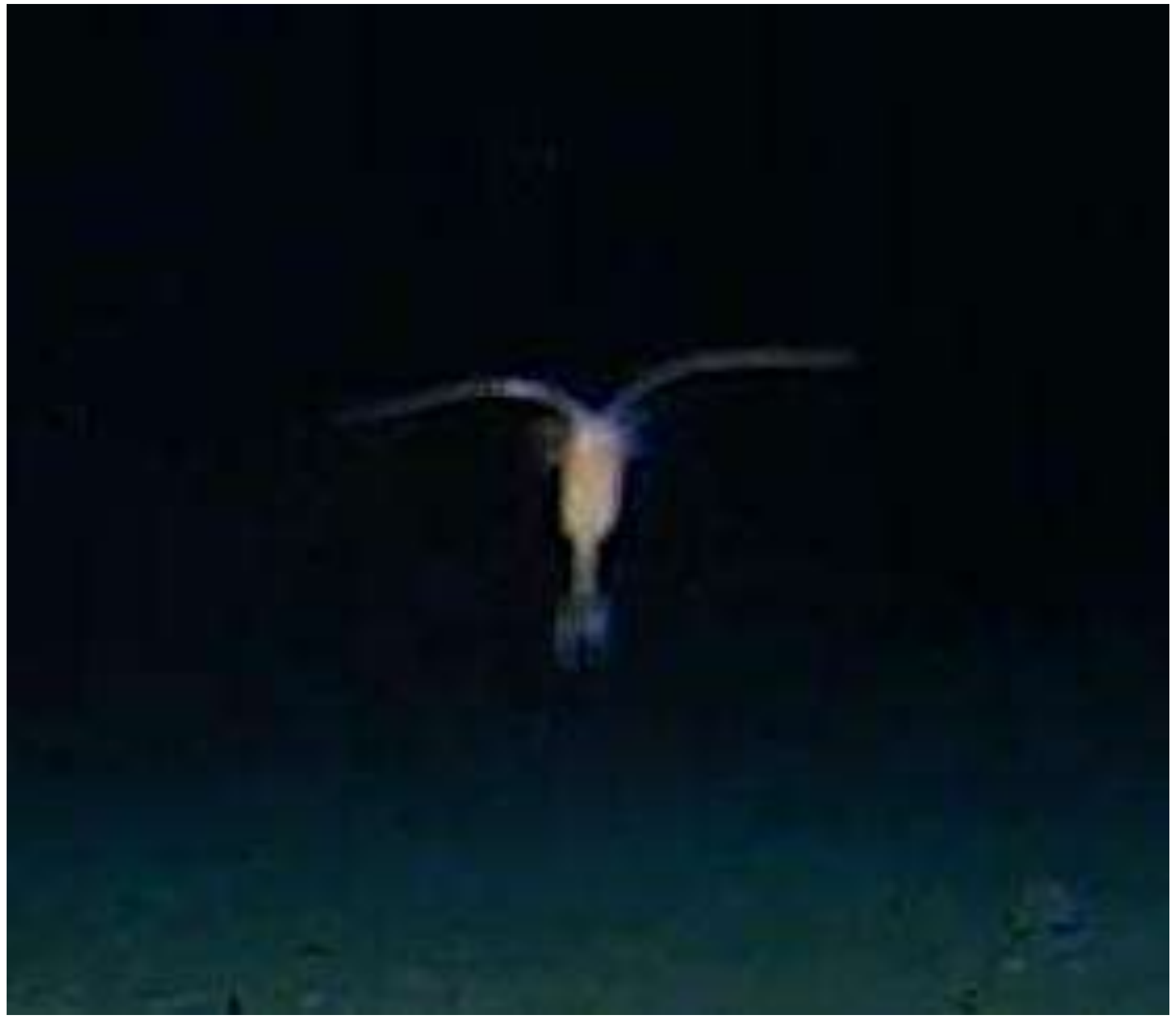

Body is ovoid and translucent. The tail is of a flattened, forked and translucent. The antennae are long and held horizontally from the cephalothorax.

Amphipoda; Amphipoda spp

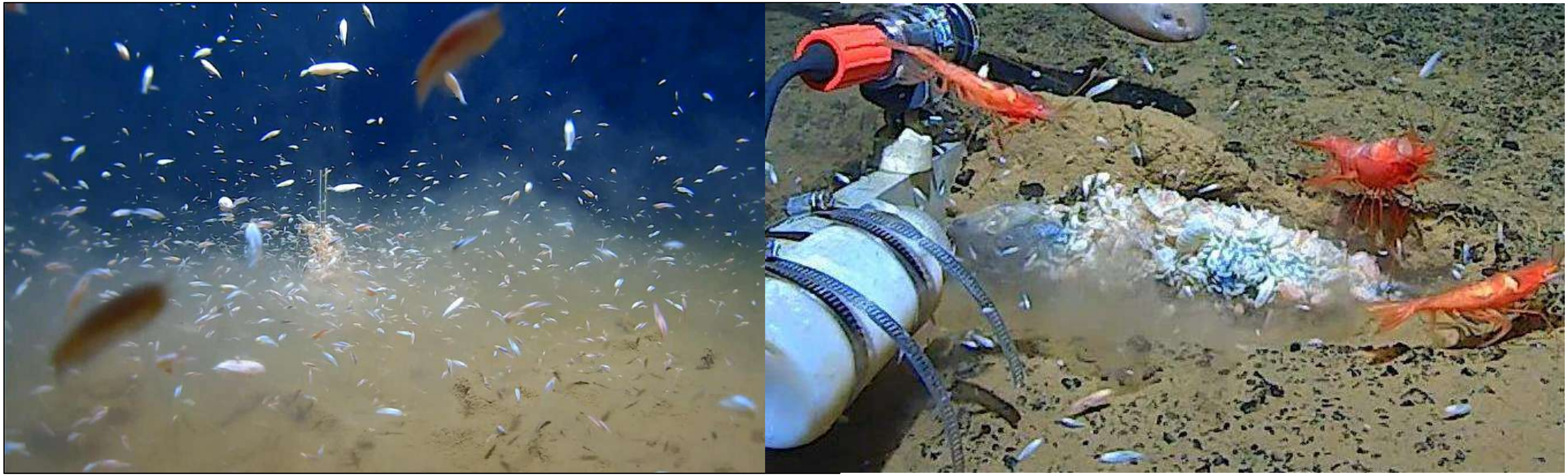

Amphipoda were attracted in high numbers to lander bait and were not identified to lower taxonomic level from video

Malacostraca; Amphipoda; Amathillopsidae; Amathillopsis; Amathilopsis sp. indet. 1

NC1_BK1_6200_IMG_4319
NC1_BK1_6200_IMG_4567
NC1_BK1_6200_IMG_4668

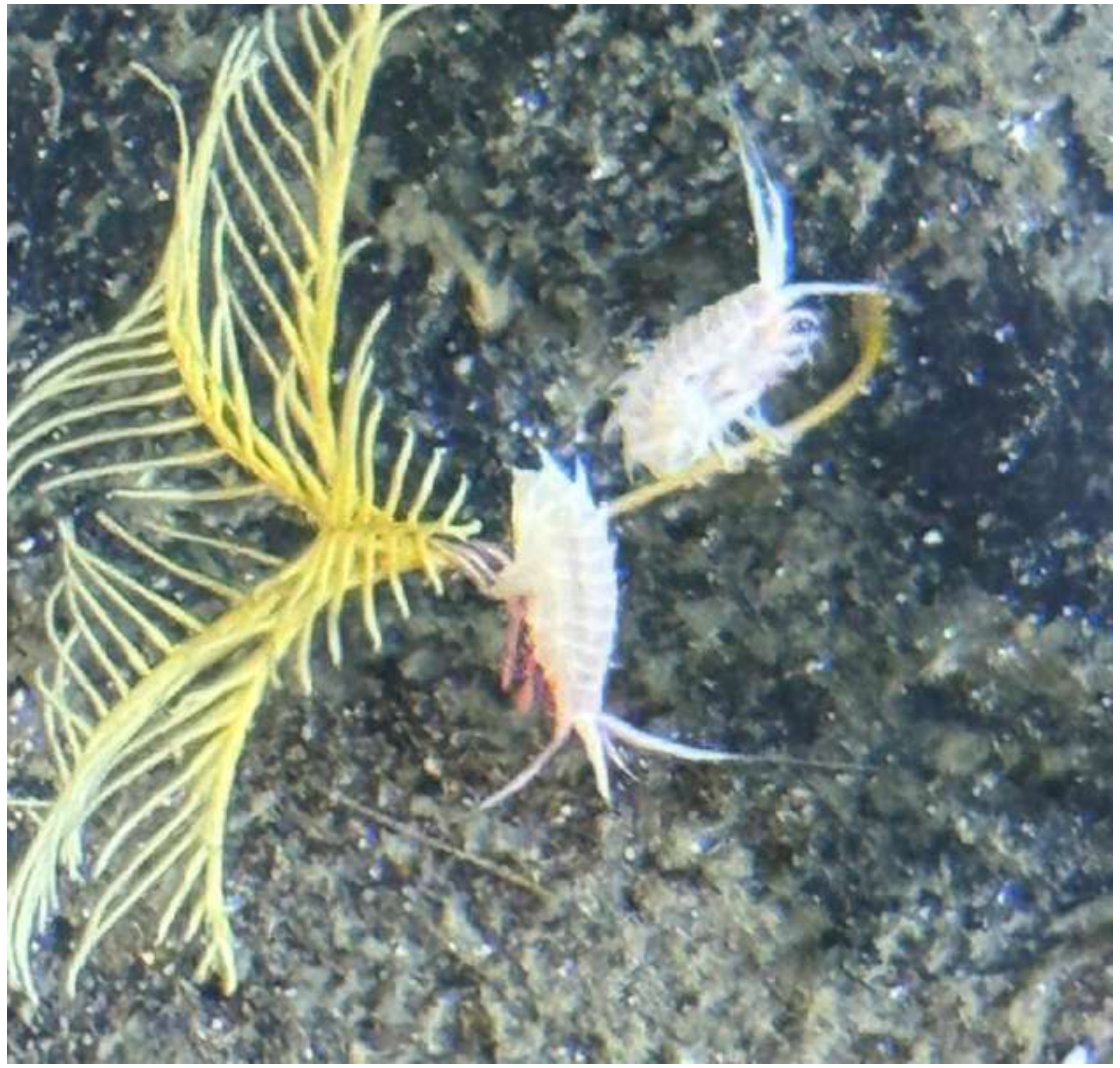

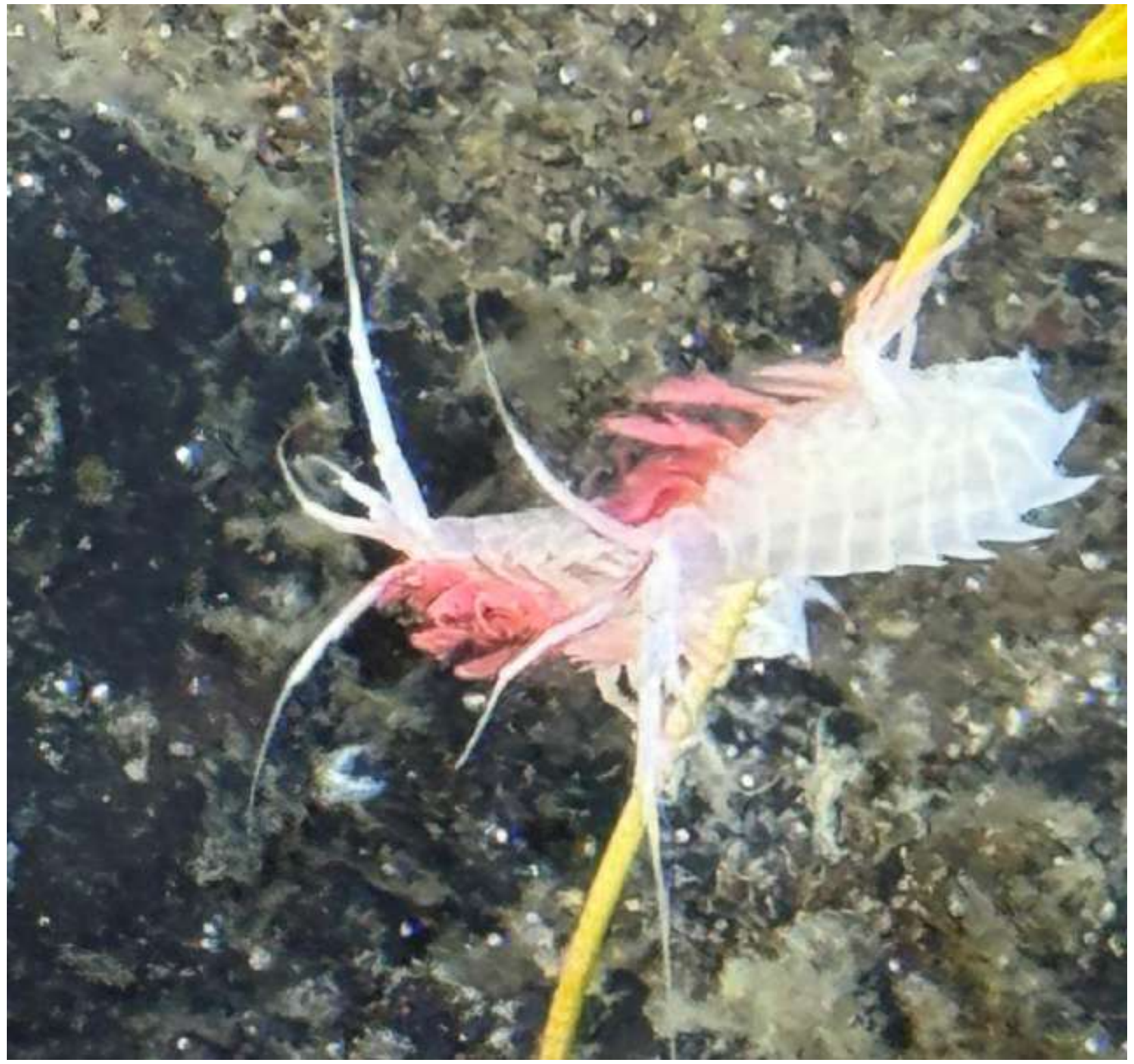

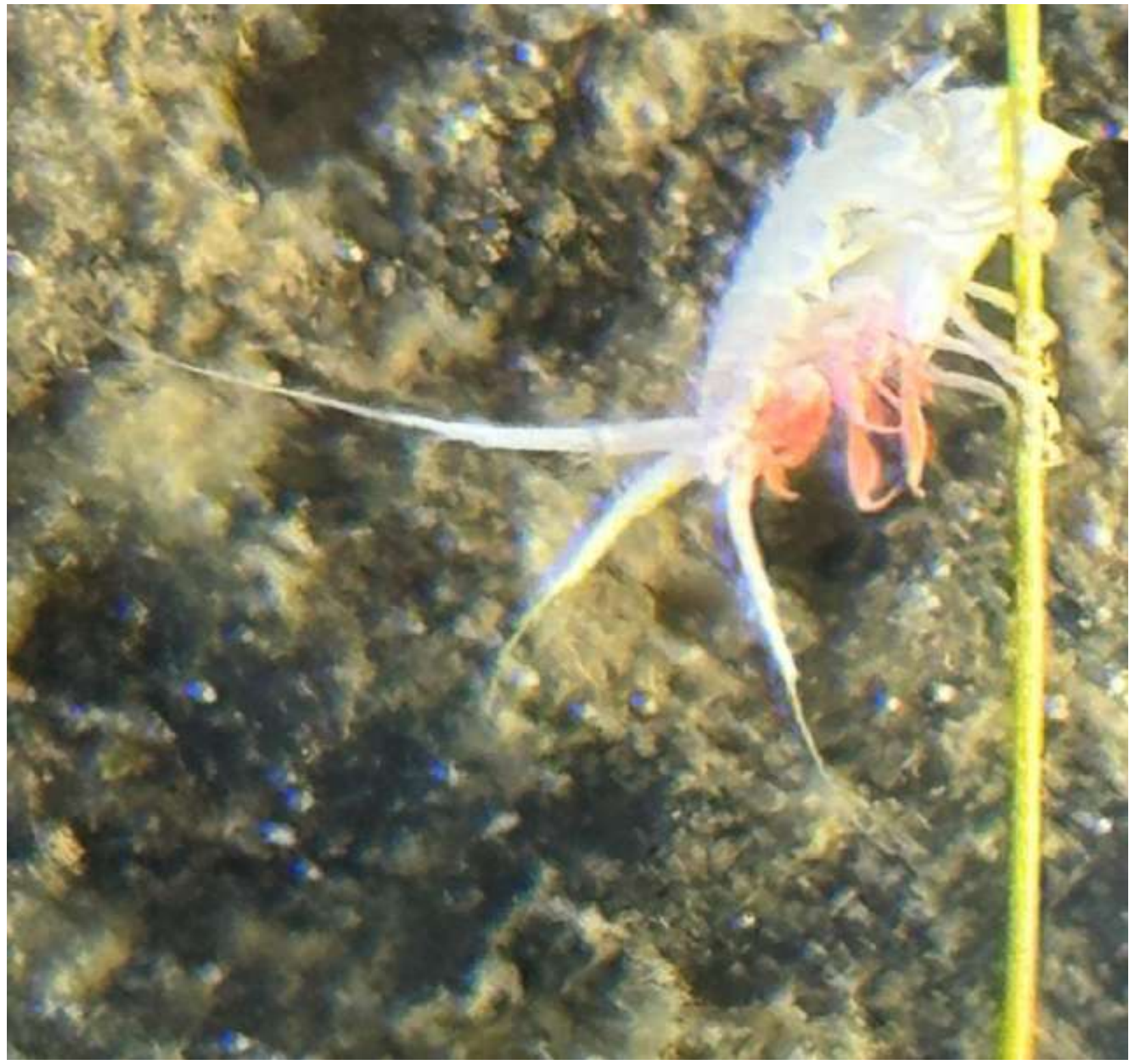

Bright white body with violet to reddish ventral appendages. Short rostrum, prominent, horn-like first antennae pair and posterior of the body strongly carinate with dorsal projections.

Behavioural diagnostic feature: cling to worm tubes and sponge stalks by posterior pereopods. Found in pairs.

Malacostraca; Cumacea
Cumacea spp.

NC1_OM4_7200_NOR_0031
NC1_MA3_6600_NOR_0084
NC1_CR8_7000_NOR_0102

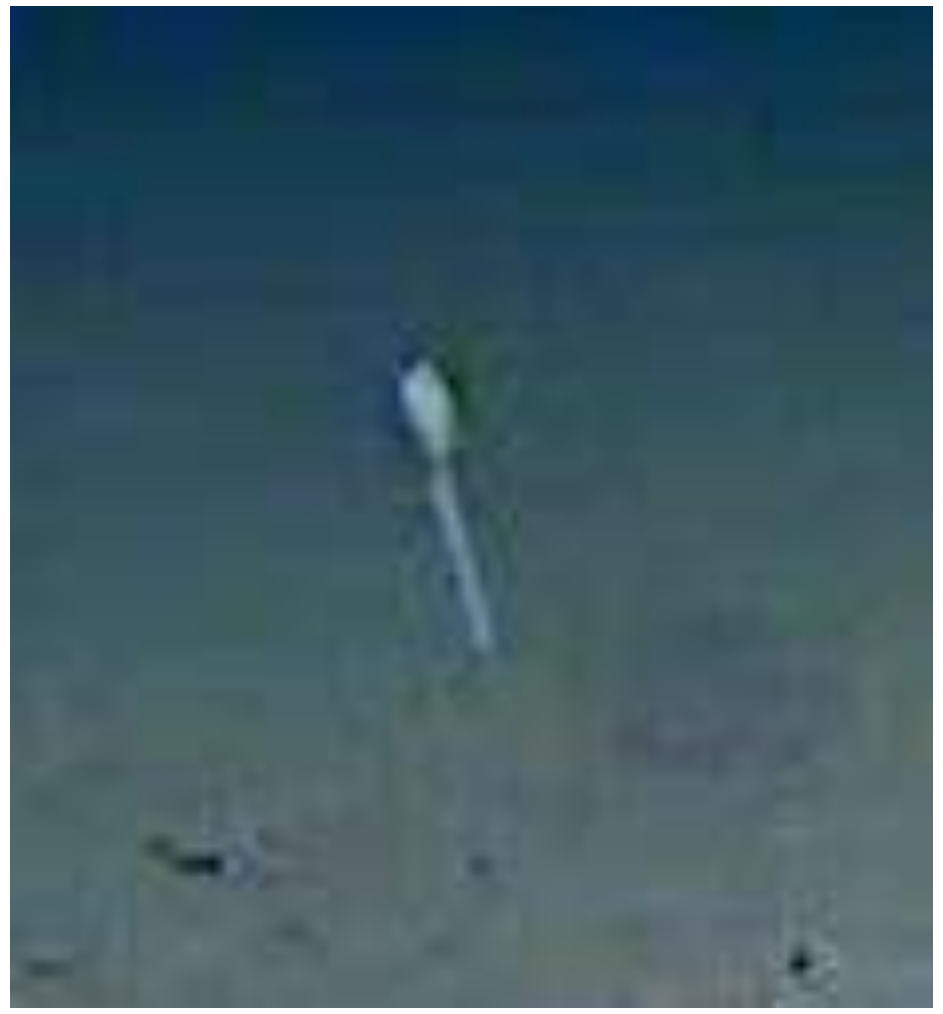
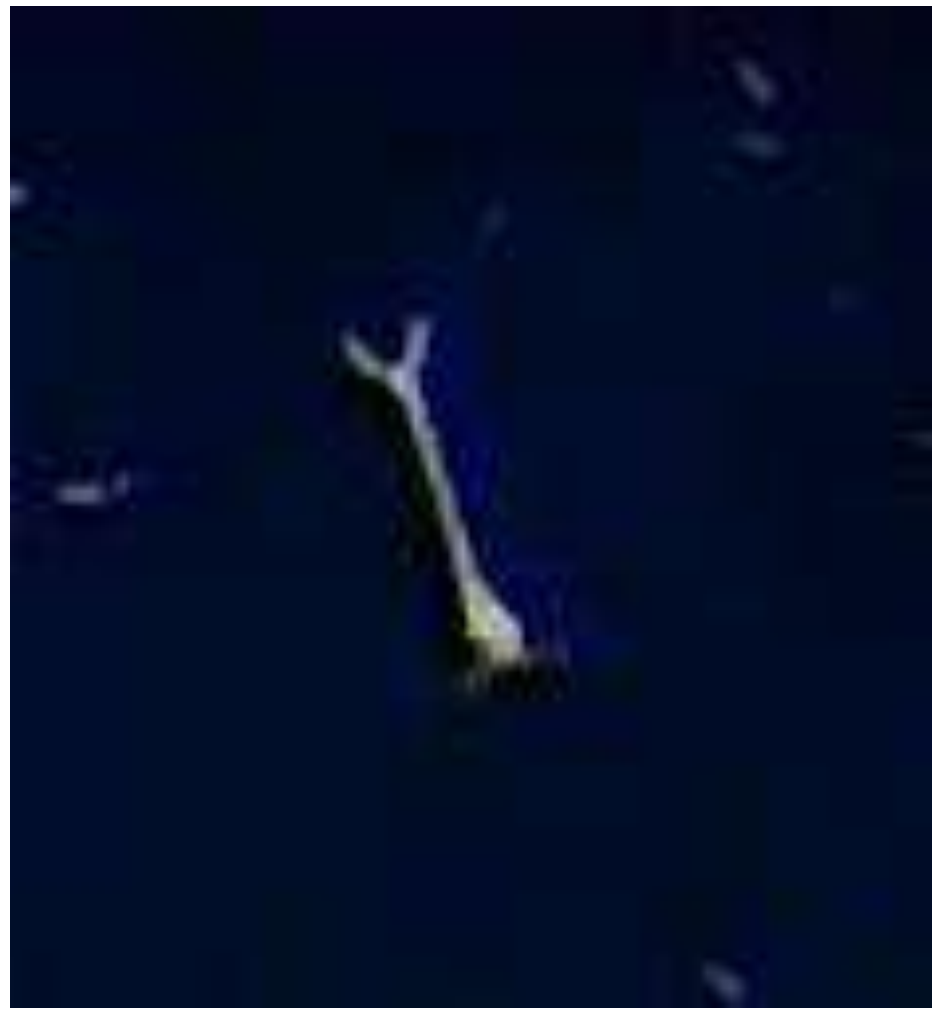
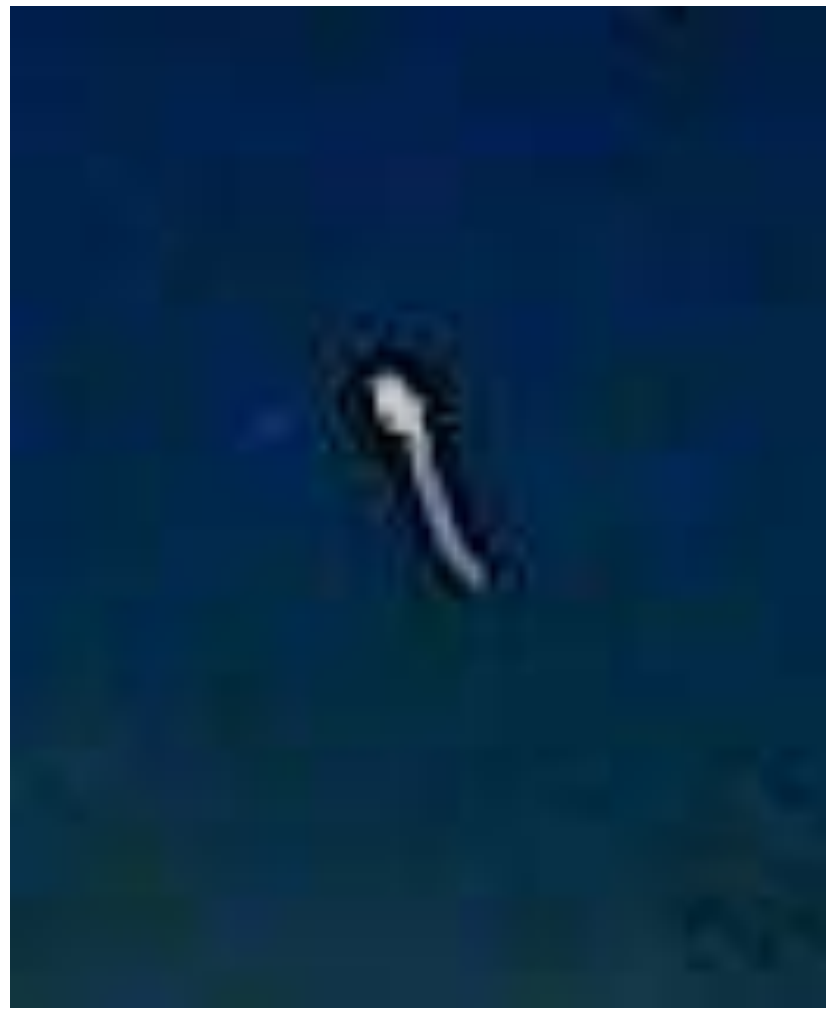

Small ovoid body with long slender tail (tail is forked but often can’t see this). Similar shape to a Mysid, however moves very differently through the water column.

Middle image: cf Leuconidae gen. indet.

Malacostraca; Decapoda; Acanthephyridae; *Heterogenys; Heterogenys* sp. indet. 1

NC4_CR9_5650_NOR_0099
NC4_OM3_4400_NOR_0096
NC2_MA8_4500_NOR_0074

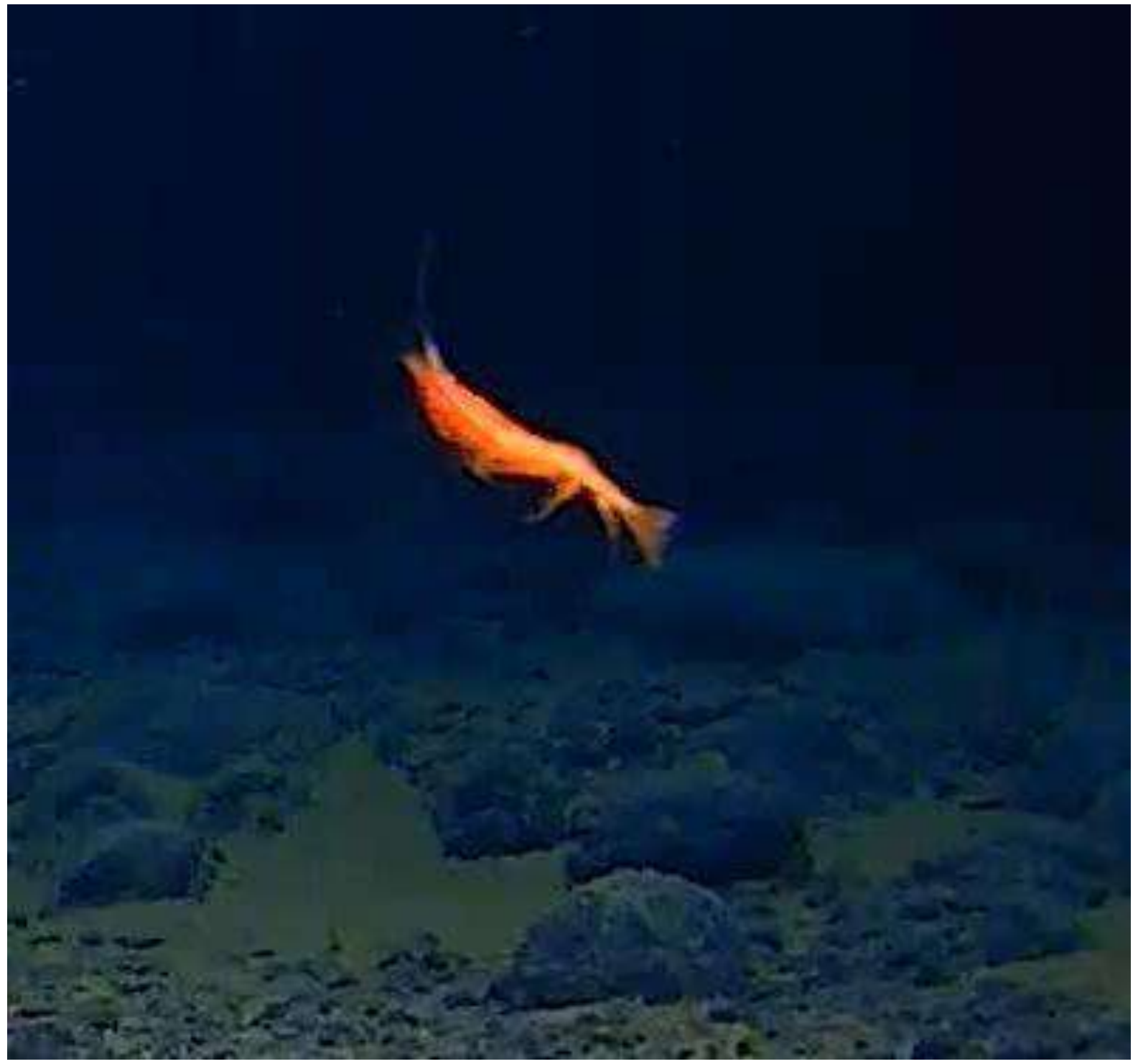

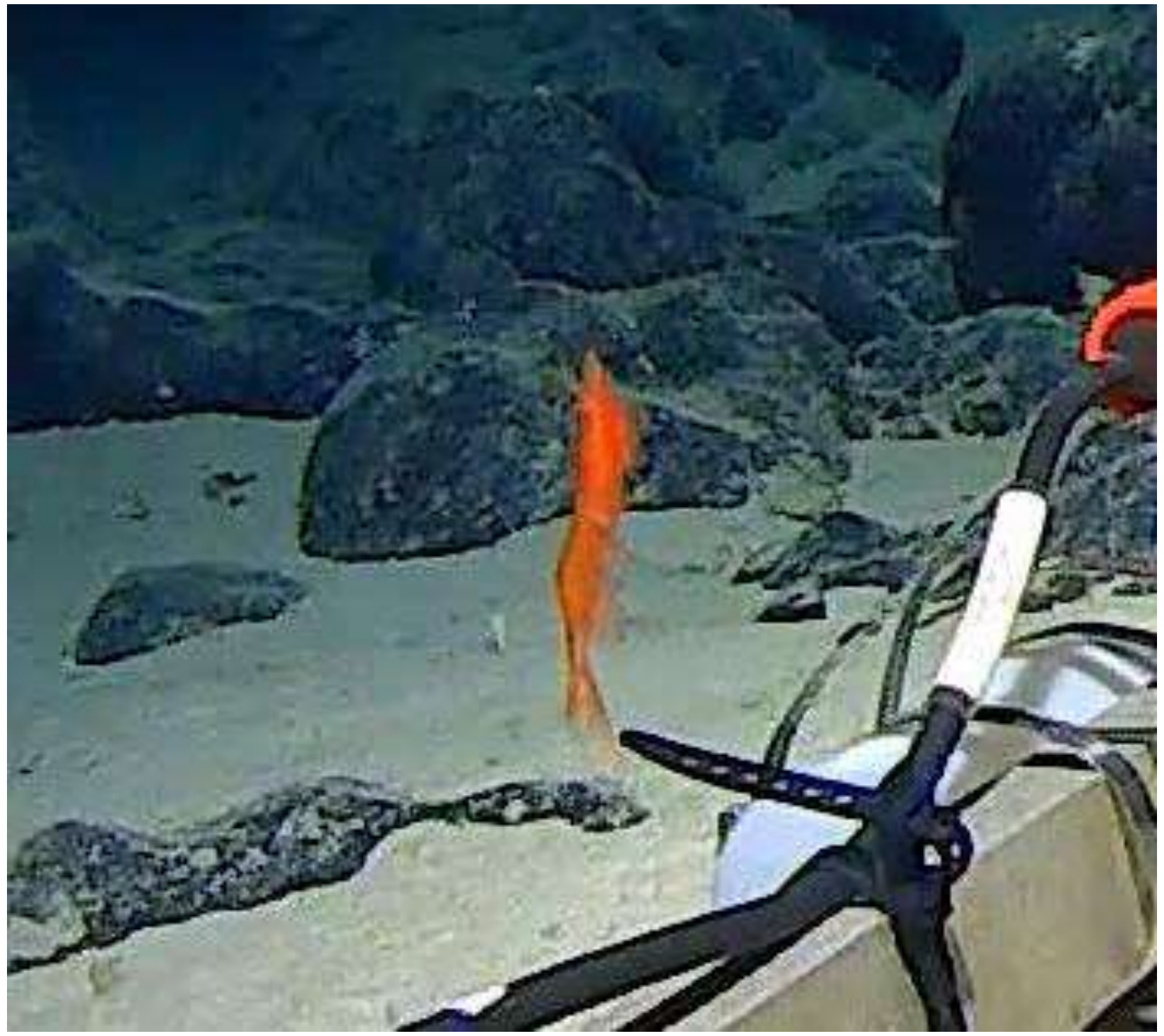

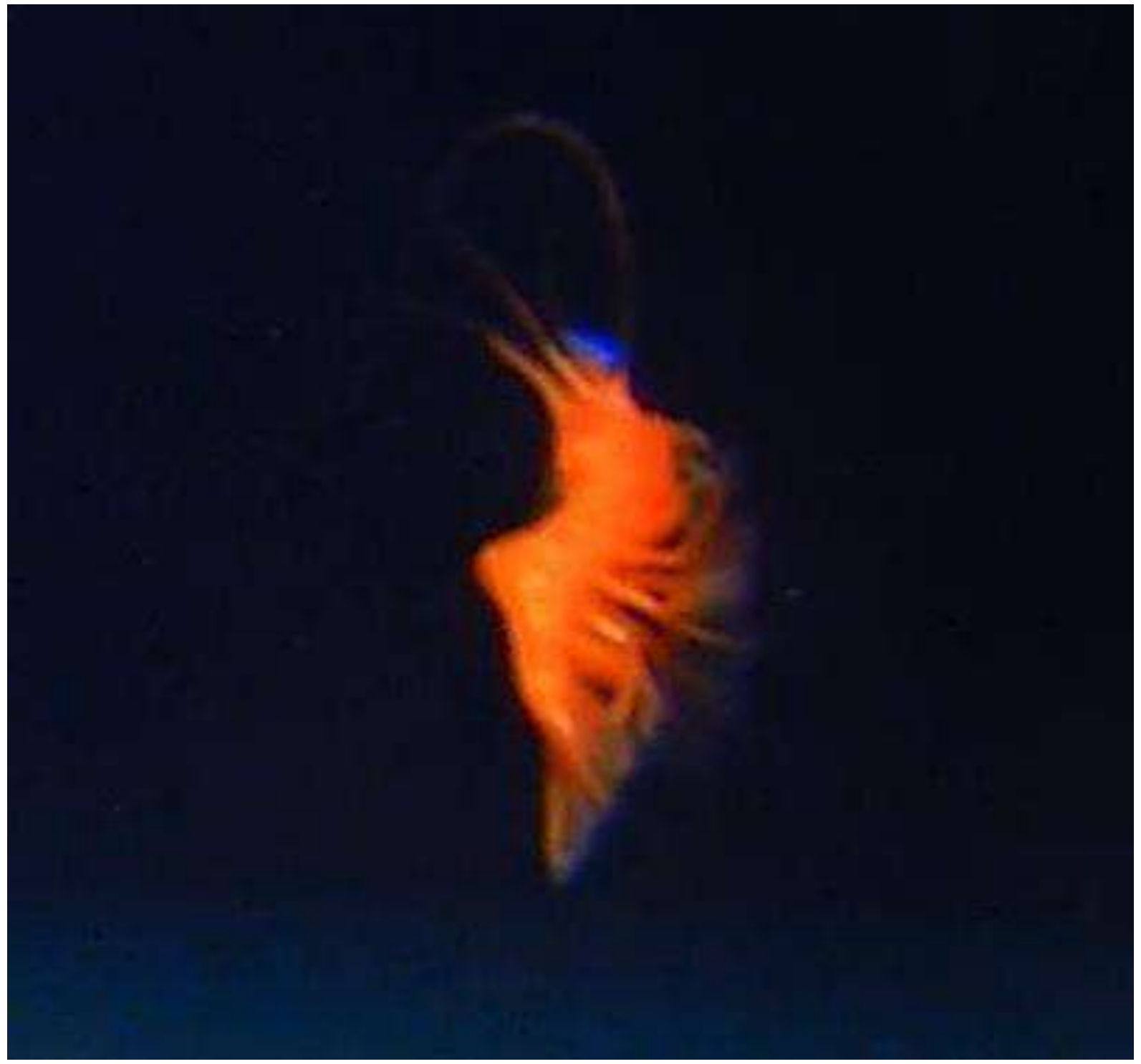

Bright red carapace wider than the yellow-orange abdomen and rostrum. Abdomen is moderately bent. Absent scaphocerites. Antennae long and pale in colour. Short abdominal appendages with wide 'tail fan'.

Behavioural Diagnostic: swim pattern is unbalanced.

***See video Clip**: https://uniwa.sharepoint.com/:v:/r/teams/EXT-MUDSRC/Shared%20Documents/Cruises/2024_02_Nova%20Canton/Data/Video%20Analysis/2_Imagery/Representative%20images/Arthropoda/Arthropoda_Malacostraca_Decapoda_Acanthephyridae_Heterogenys_sp1_NC2_MA8_4500_1_01_NOR_0074.avi_0005712.mp4?csf=1&web=1&e=aEkCfS*

Malacostraca; Decapoda; Acanthephyridae; *Hymenodora*; *Hymenodora* sp. indet. 1

NC2_CR8_4400_NOR_0026
NC2_MA8_4500_NOR_0023
NC2_CR4_4100_NOR_0029

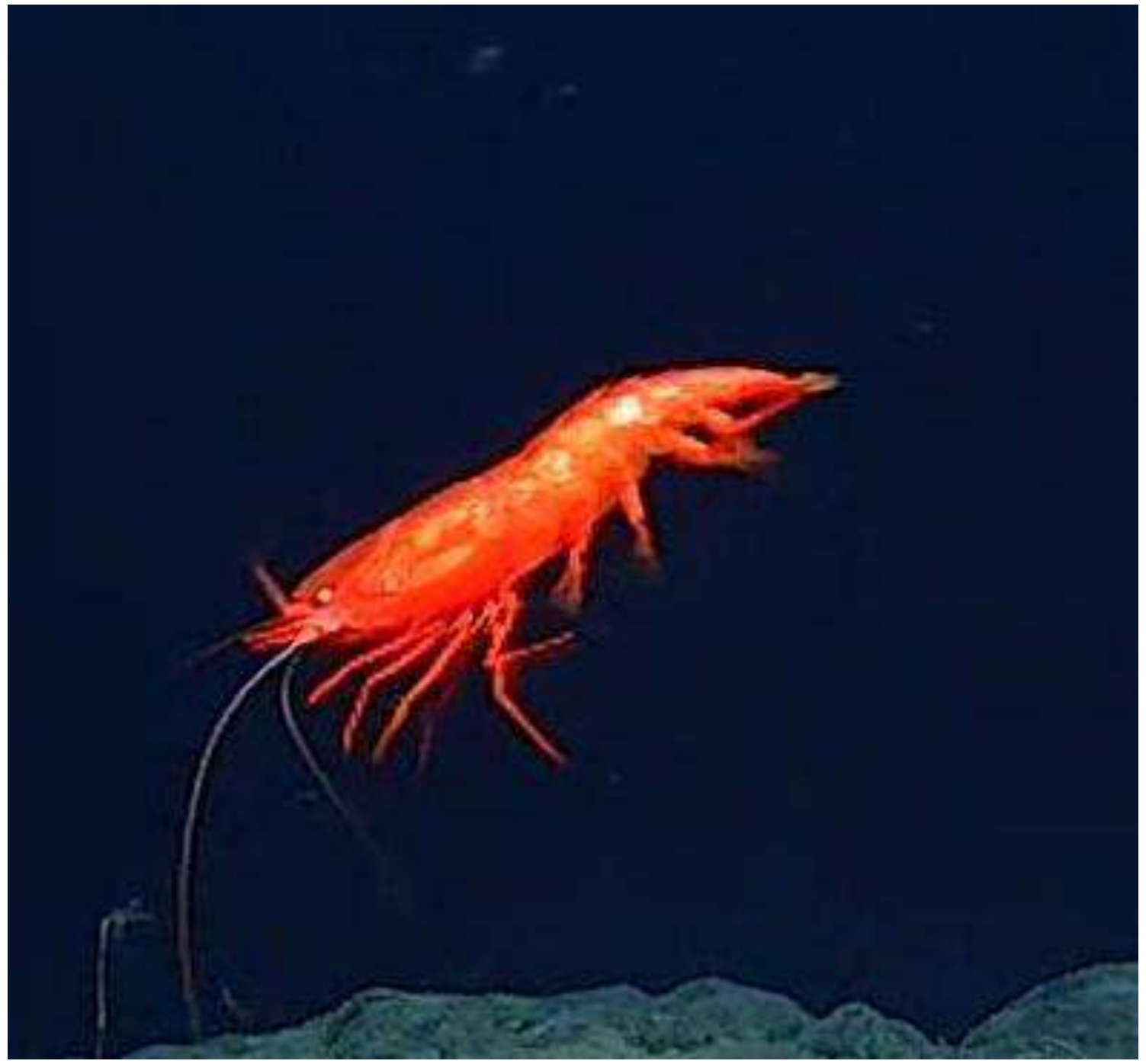
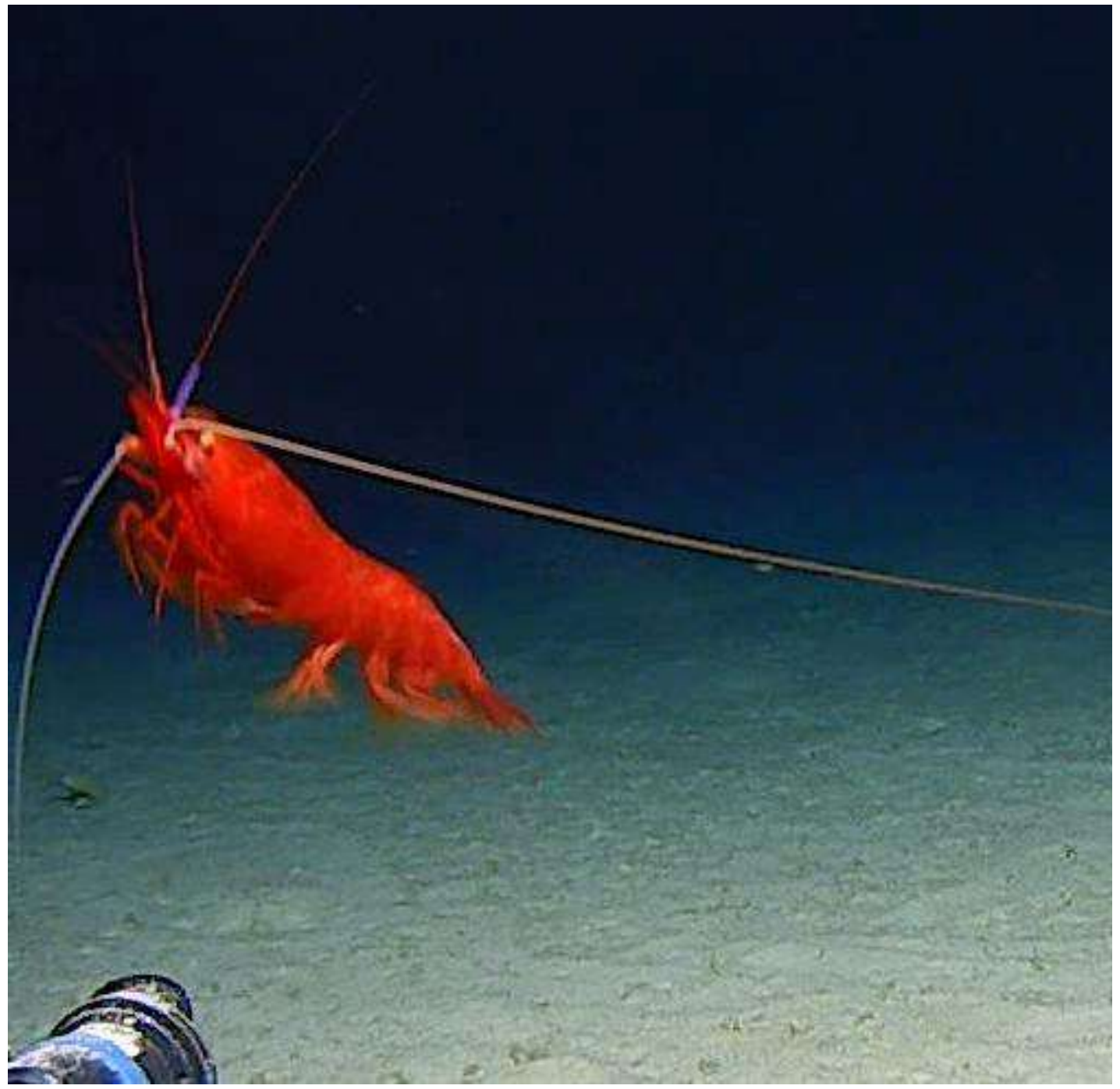
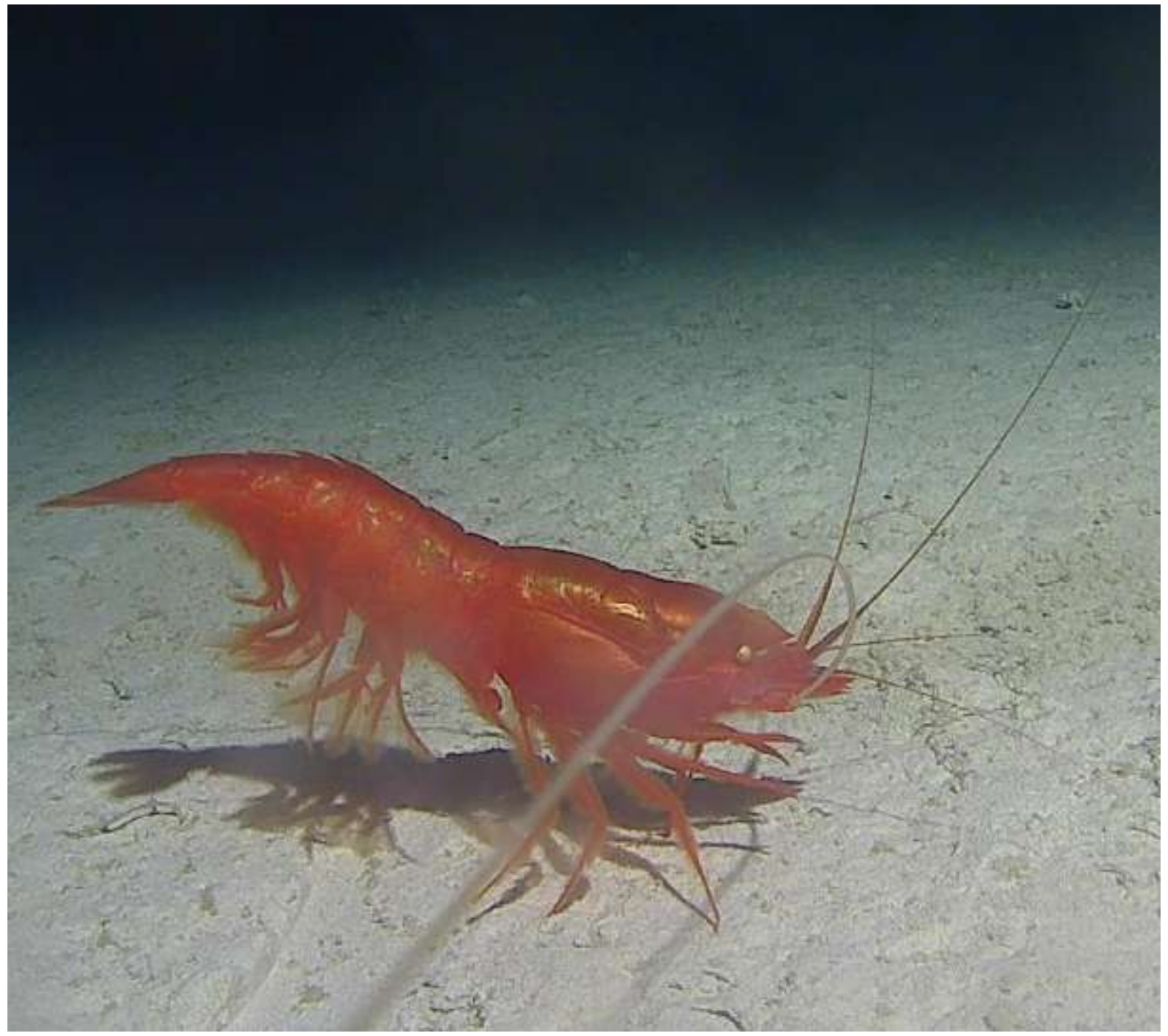

Uniformly bright red body with slightly dorsally darker patch around the centre of the body. Antennae long and white to yellowish white. Short abdominal appendages. No apparent scaphocerites.

Malacostraca; Decapoda; Acanthephyridae; *Hymenodora*; *Hymenodora* sp. indet. 2

NC2_OM2_3400_NOR_0038

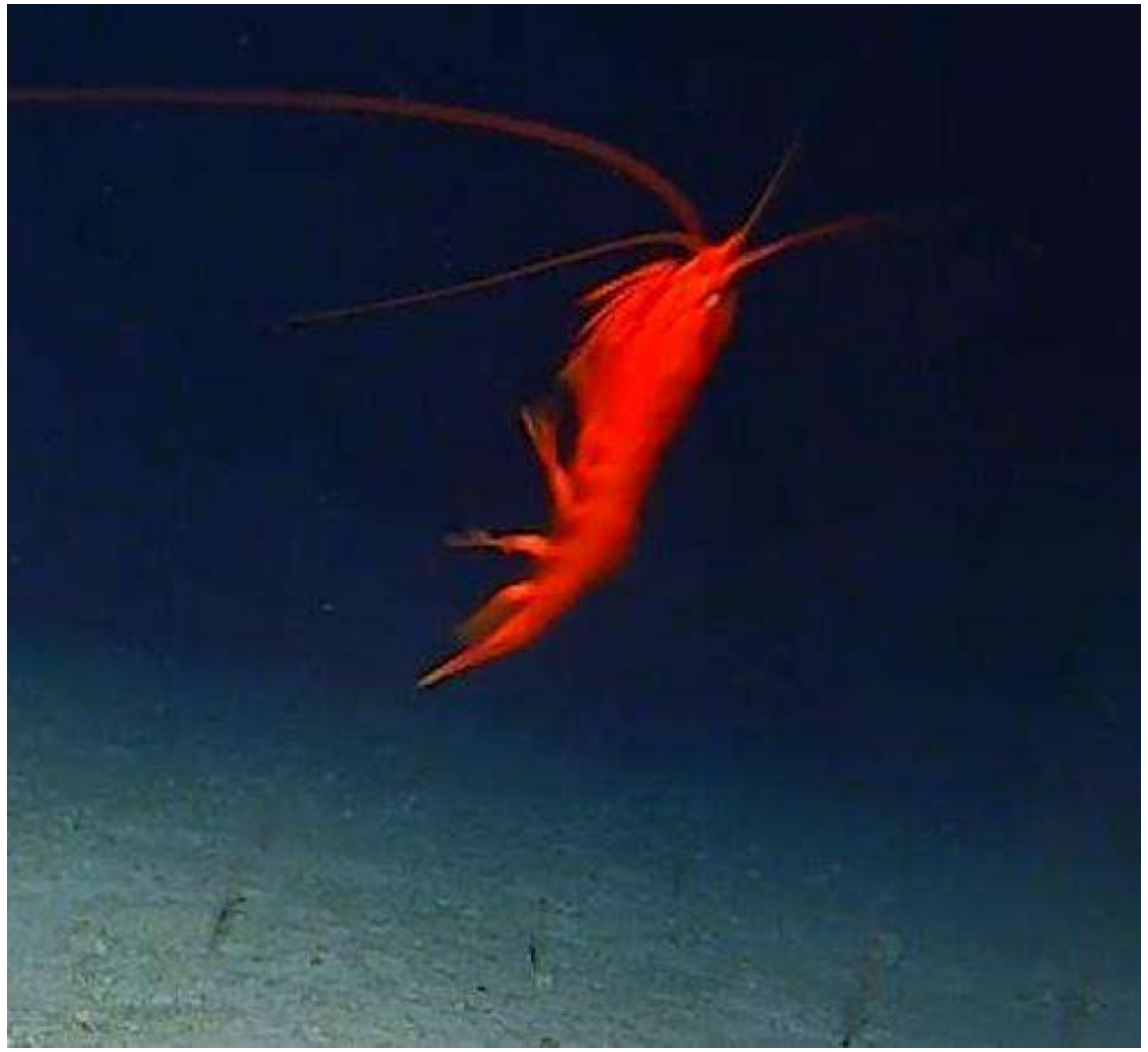

Uniformly bright red body with slightly dorsally darker patch around the centre of the body. Antennae long and red. Short abdominal appendages. No apparent scaphocerites.

Malacostraca; Decapoda; Acanthephyridae; Acanthephyridae gen. indet. 1

NC2_OM2_3400_NOR_0037
NC4_CR3_4400_NOR_0084

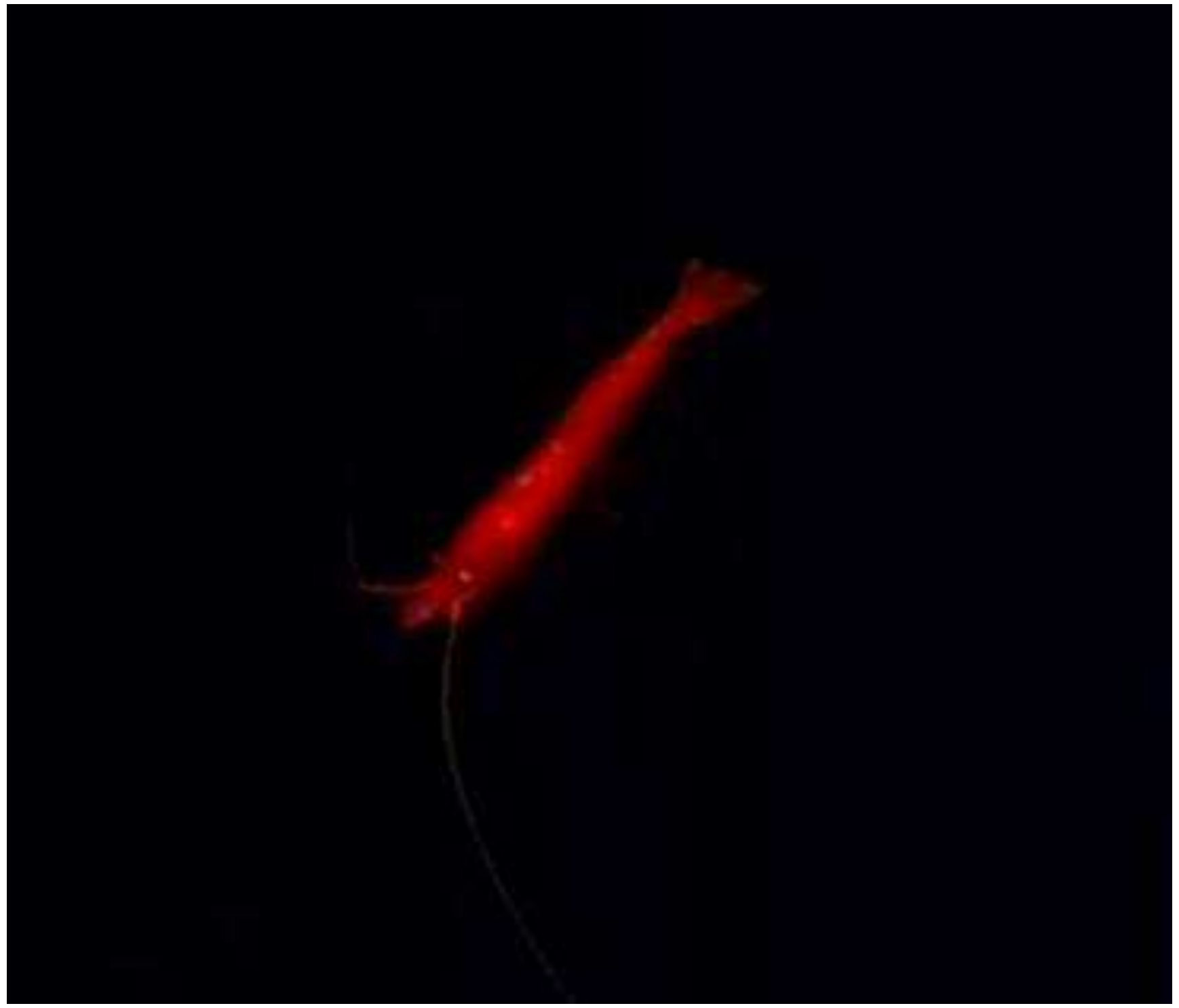

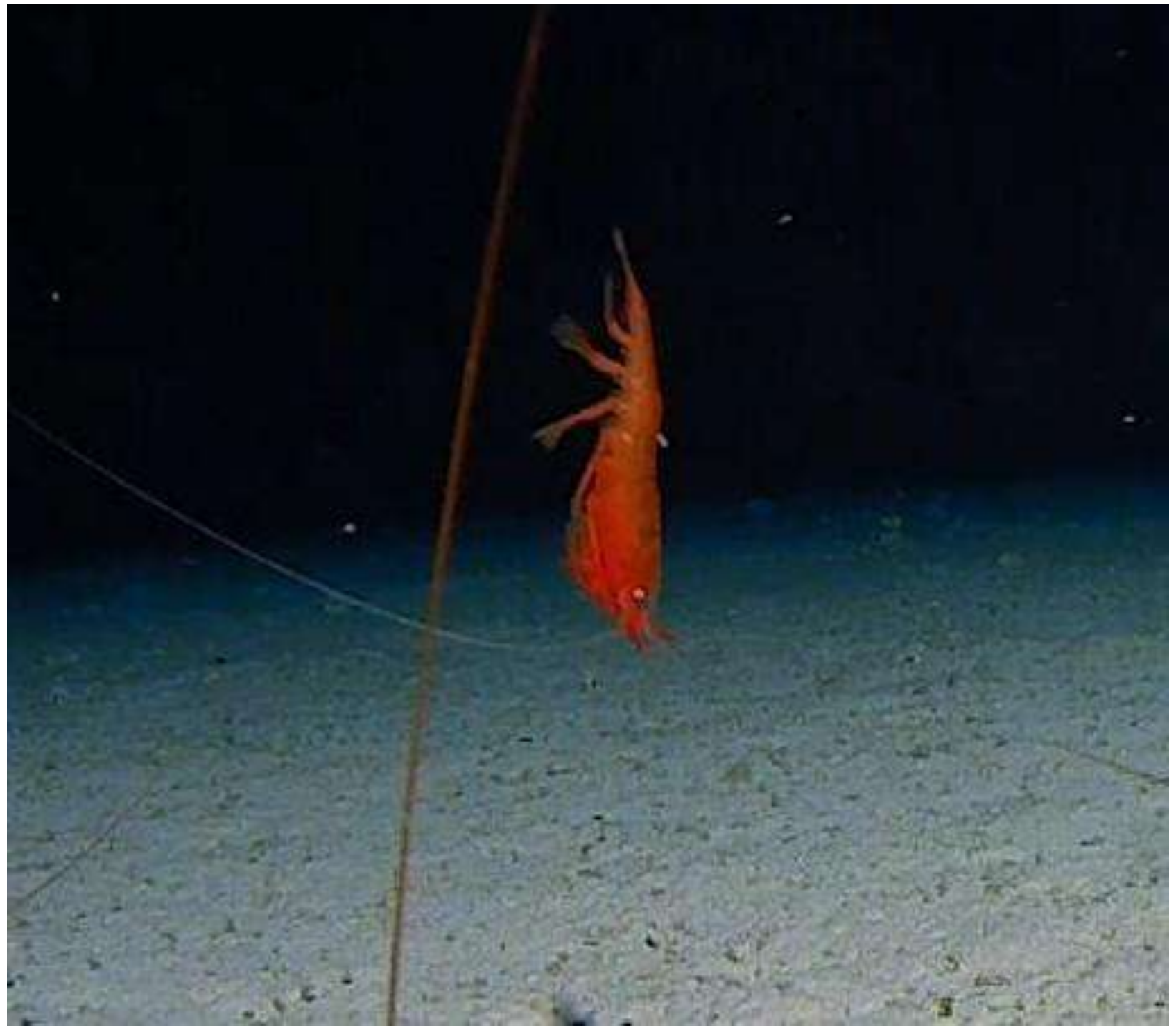

Bright red carapace wider than the abdomen. Rostrum is wide. Abdomen is moderately bent. Absent scaphocerites. Antennae long and pale in colour. Short abdominal appendages with wide 'tail fan'.

Malacostraca; Decapoda; Acanthephyridae; Acanthephyridae gen. indet. 2

NC2_MA3_3700_NOR_0103

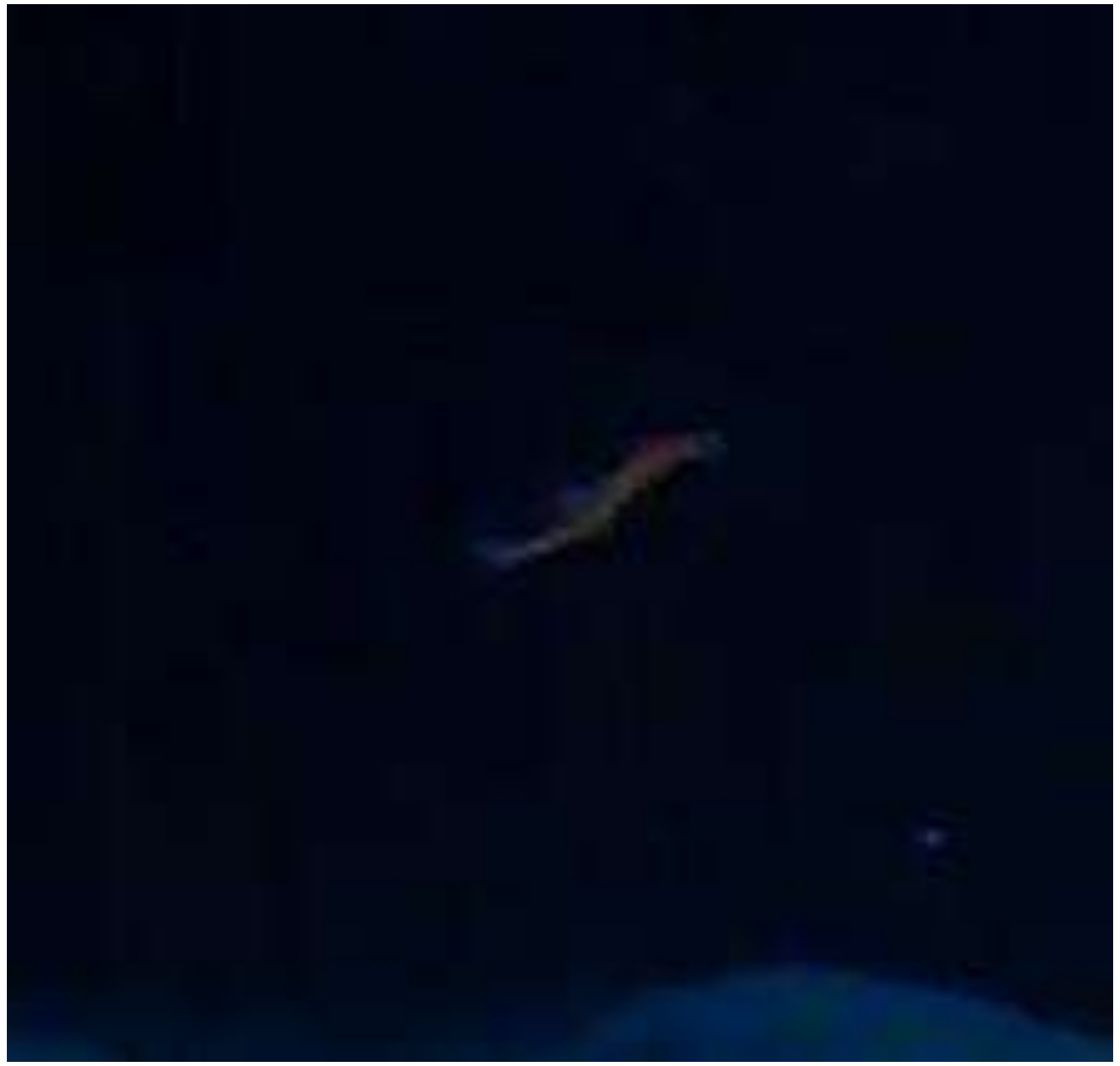

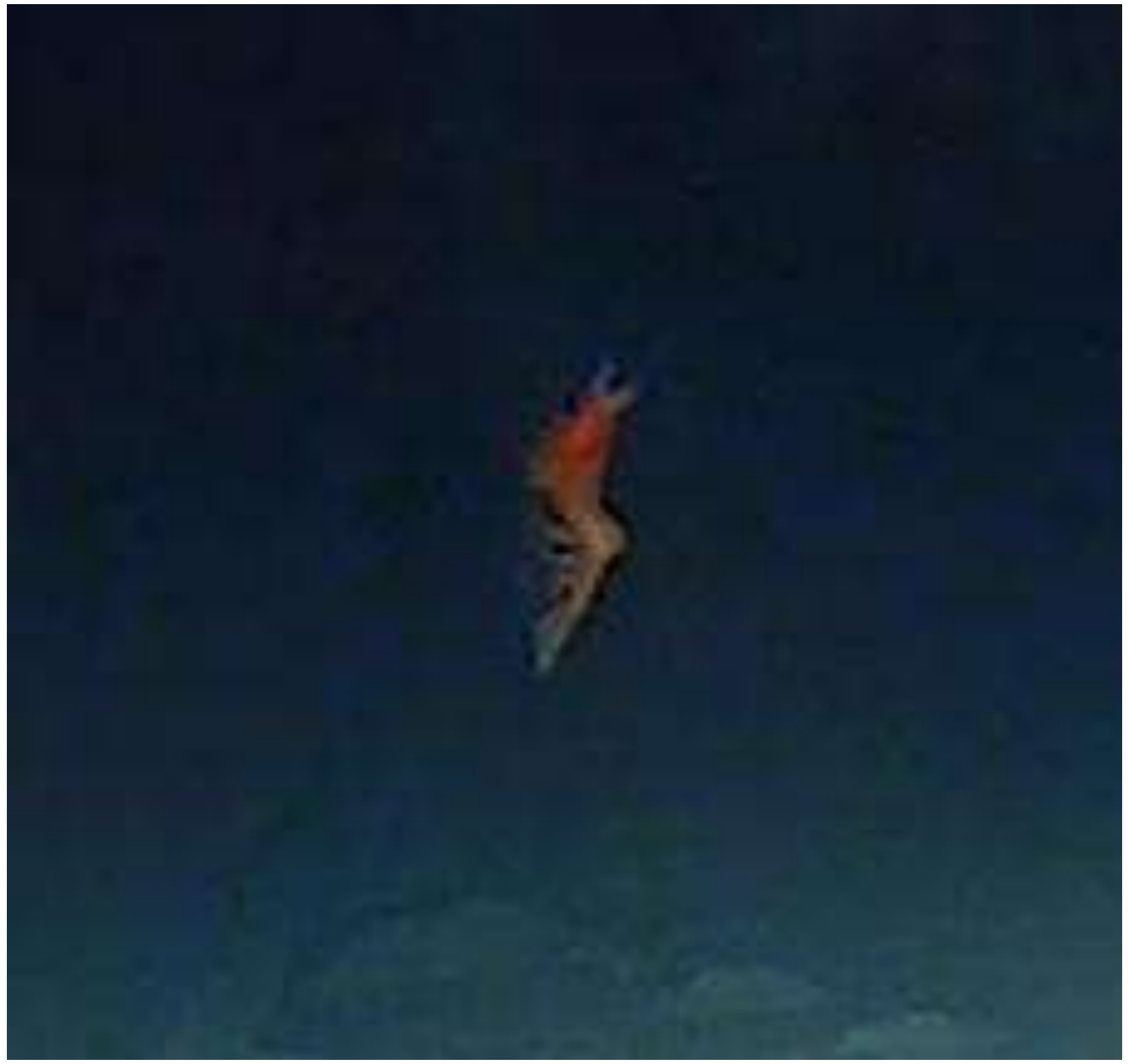

Bright red carapace wider than the yellowish abdomen and rostrum. Abdomen is strongly bent. Small, yellowish, scaphocerites. Antennae long and red. Short abdominal appendages with wide 'tail fan'.

Behavioural Diagnostic: swim pattern is uncoordinated drifting.

See Video: https://uniwa.sharepoint.com/:v:/r/teams/EXT-MUDSRC/Shared%20Documents/Cruises/2024_02_Nova%20Canton/Data/Video%20Analysis/Representative%20images/Arthropoda/Arthropoda_Malacostraca_Decapoda_Acanthephyridae_sp1_NC2_MA3_3700_1_01_NOR_0103.avi_0001521.mp4?csf=1&web=1&e=c9Ms1N

Malacostraca; Decapoda; Acanthephyridae; Acanthephyridae gen. indet. 3

NC3_MA3_6100_NOR_0052

NC4_CR6_5000_NOR_0046

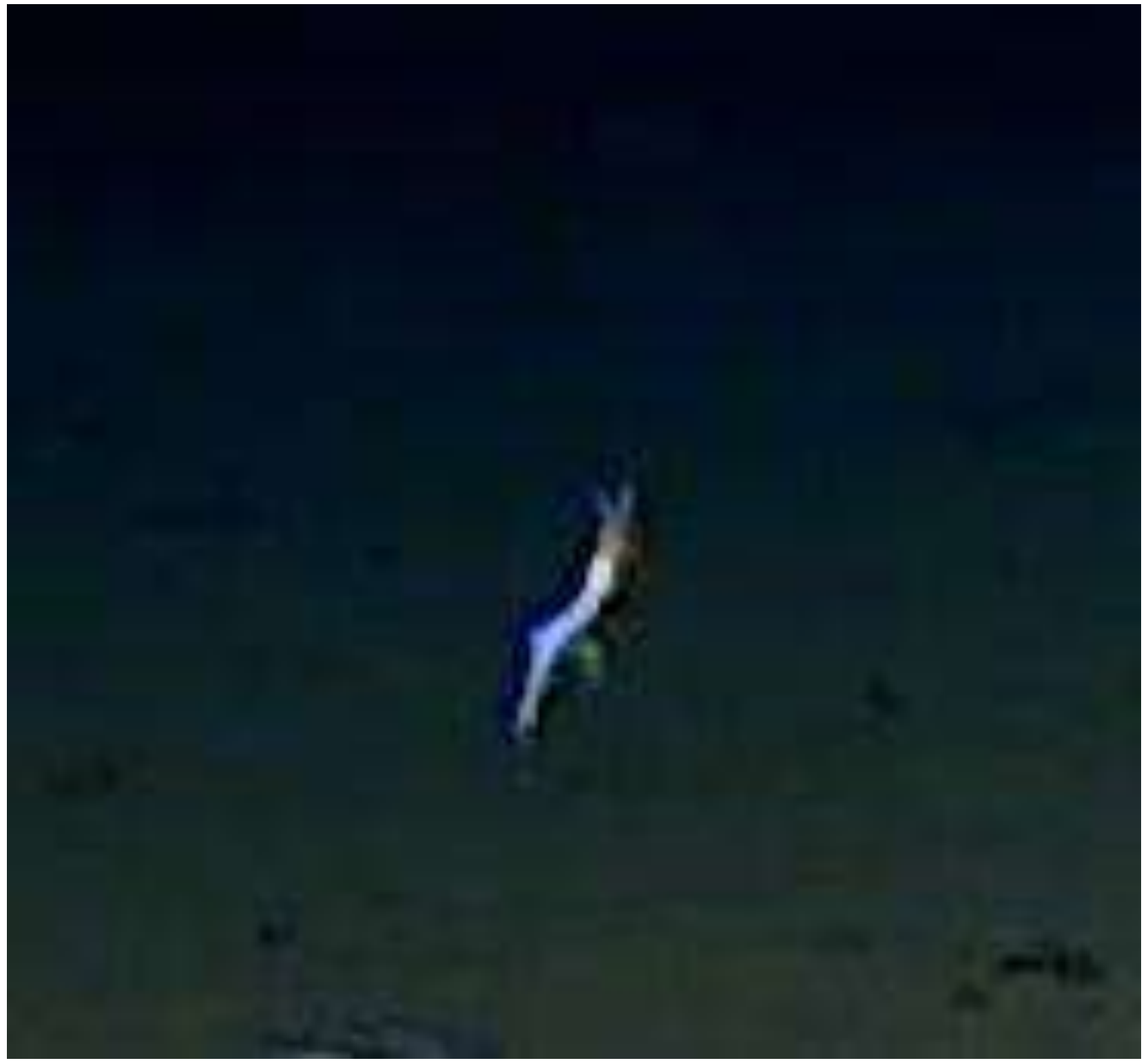

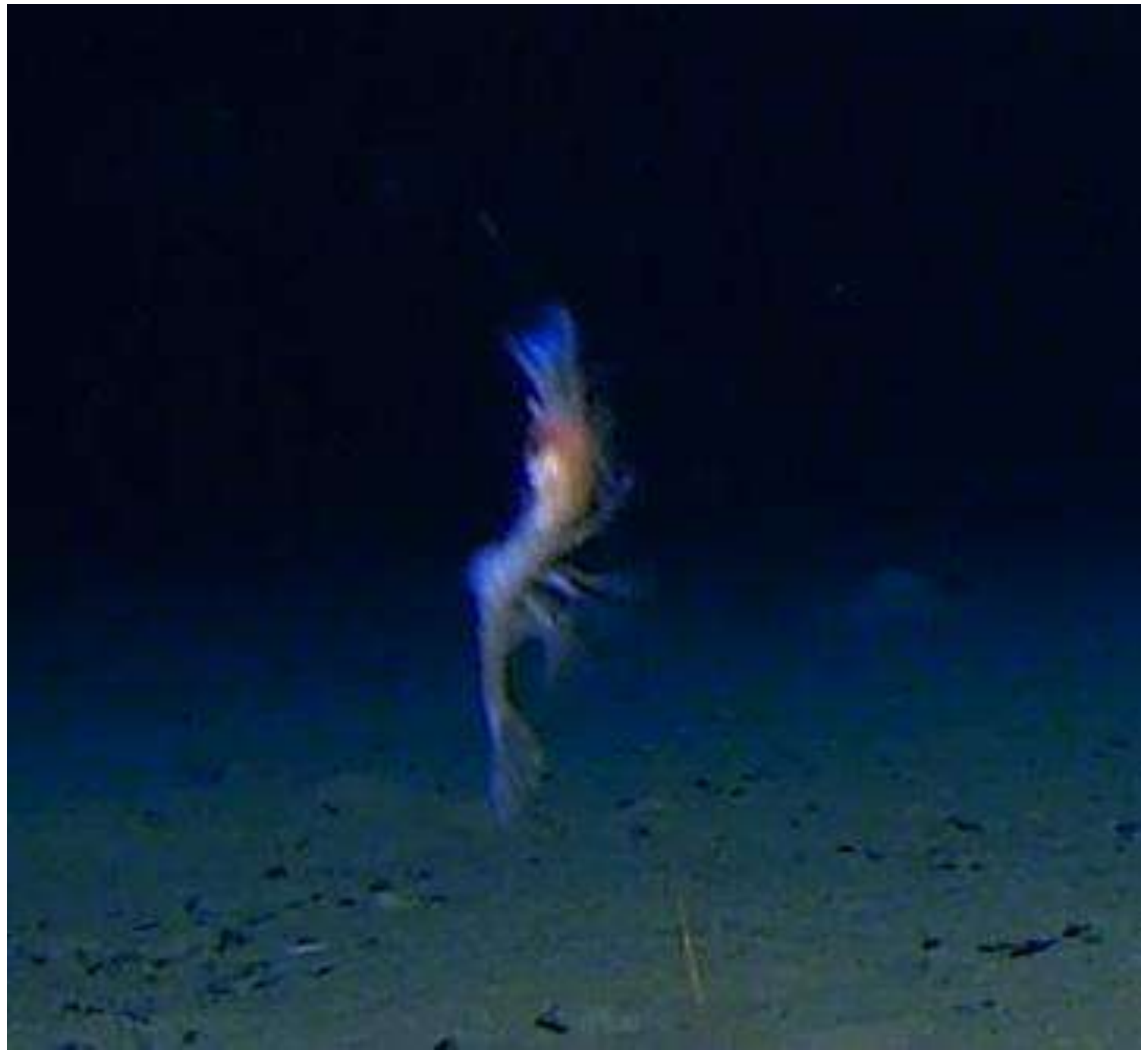

Translucent body with pinkish internal organs visible. Abdomen is strongly bent. Antennae long and transparent. Short abdominal appendages. Scaphocerites also transparent.

Behavioural Diagnostic: swim pattern is uncoordinated drifting.

Malacostraca; Decapoda; Aristeidae; Cerataspis; *Cerataspis monstrosus*

NC2_CR5_5500_NOR_0056
NC3_MA8_2800_NOR_0028
NC3_MA8_2800_NOR_0028

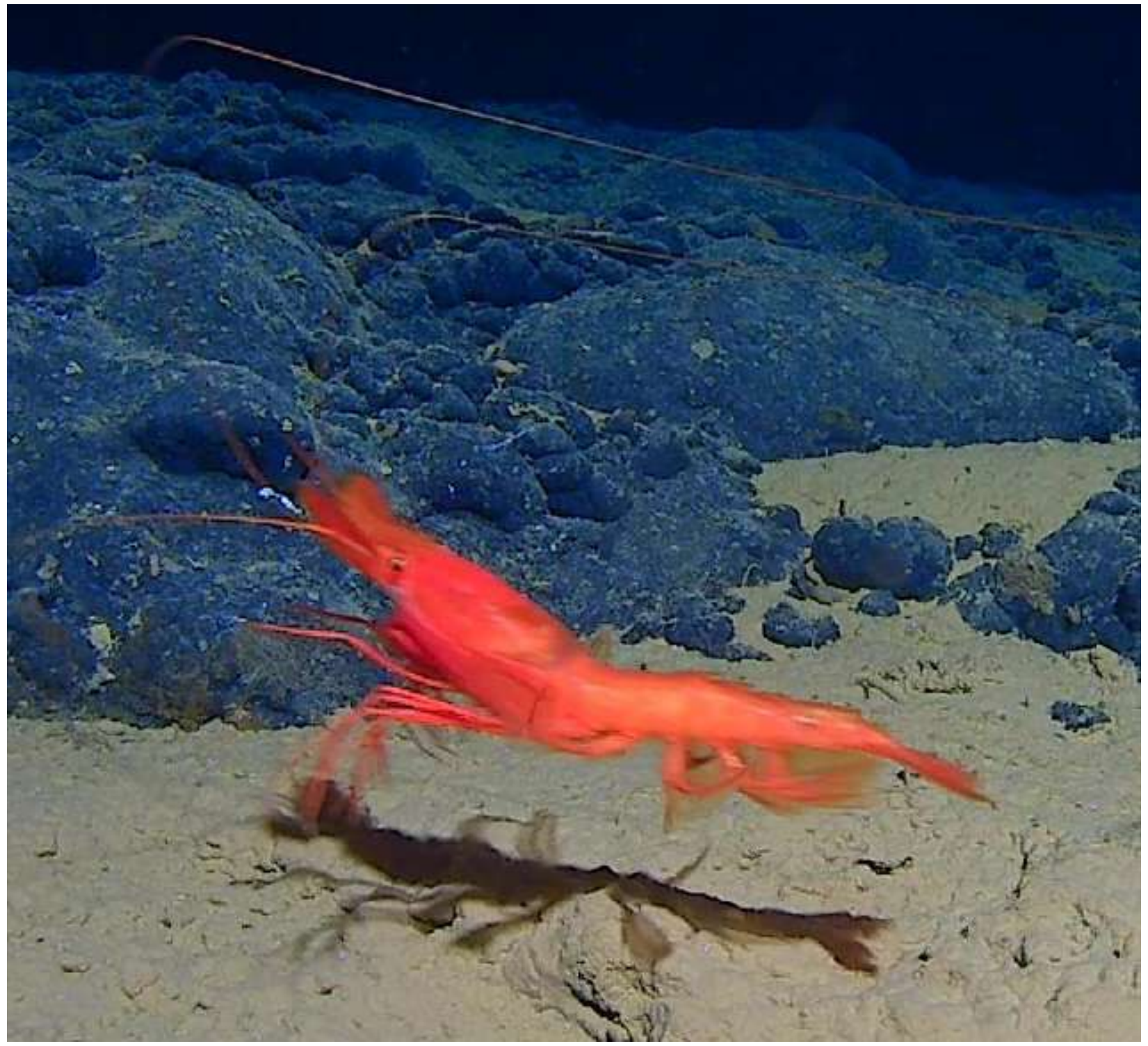
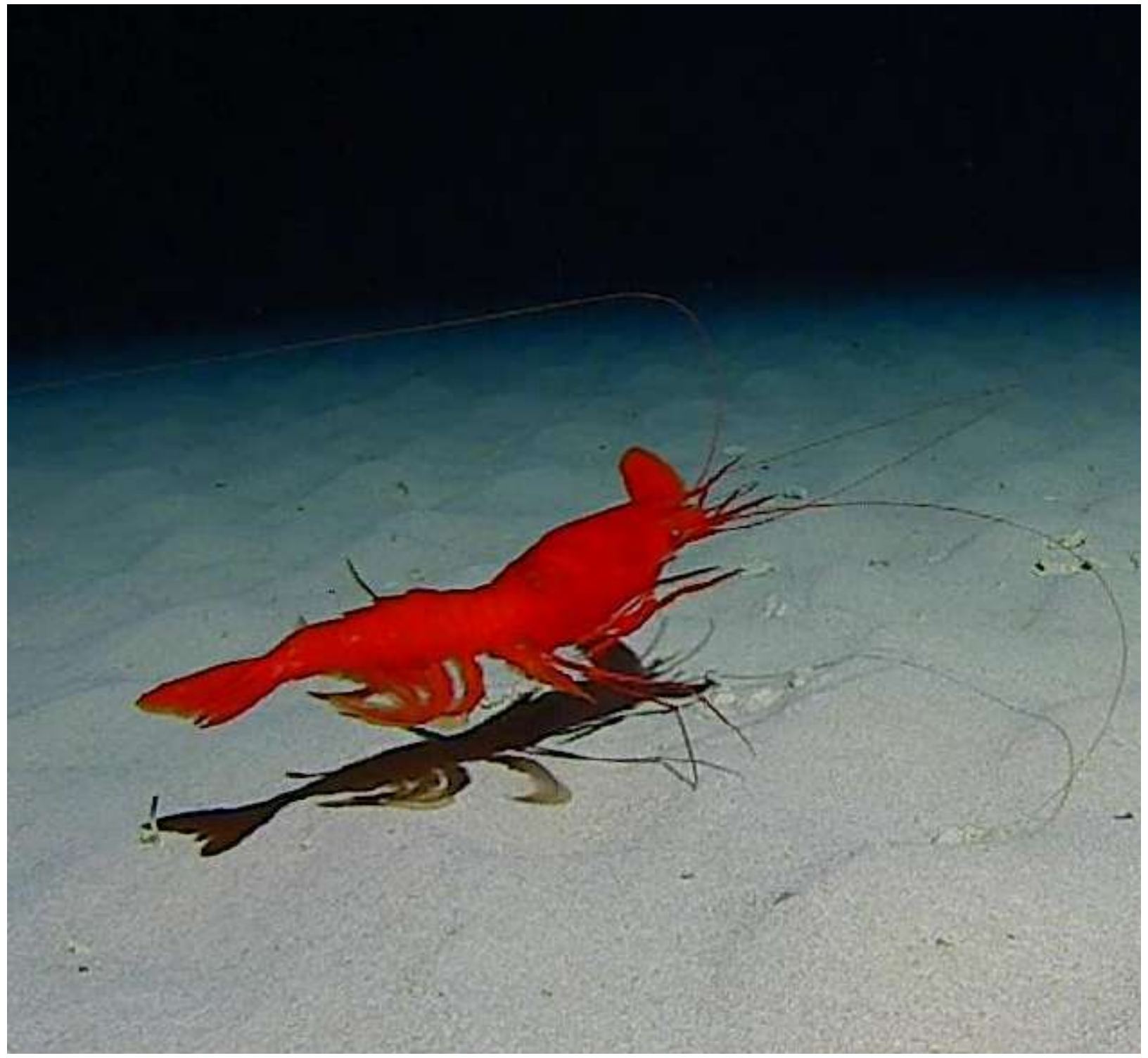
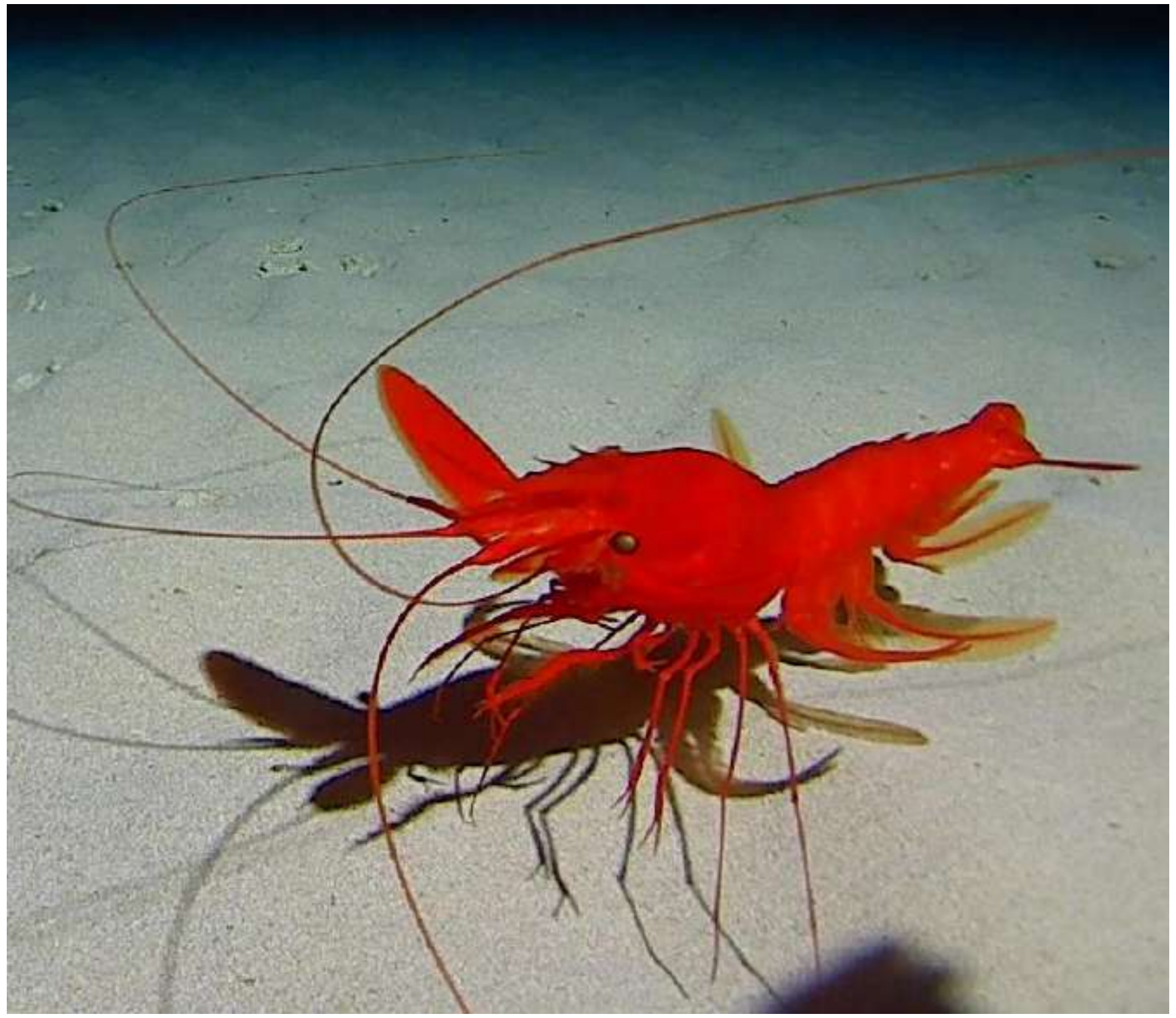

Bright red body. Carapace wider than the abdomen with elongate rostrum and large oval opaque-red, lateral facing scaphocerites. Carapace held in a slightly upward posture. Antennae long and red. Long abdominal appendages with wide 'tail fan'.

Malacostraca; Decapoda; Benthesicymidae; Benthesicymidae gen. indet. 1

NC3_MA8_2800_NOR_0055

NC3_MA8_2800_NOR_0135

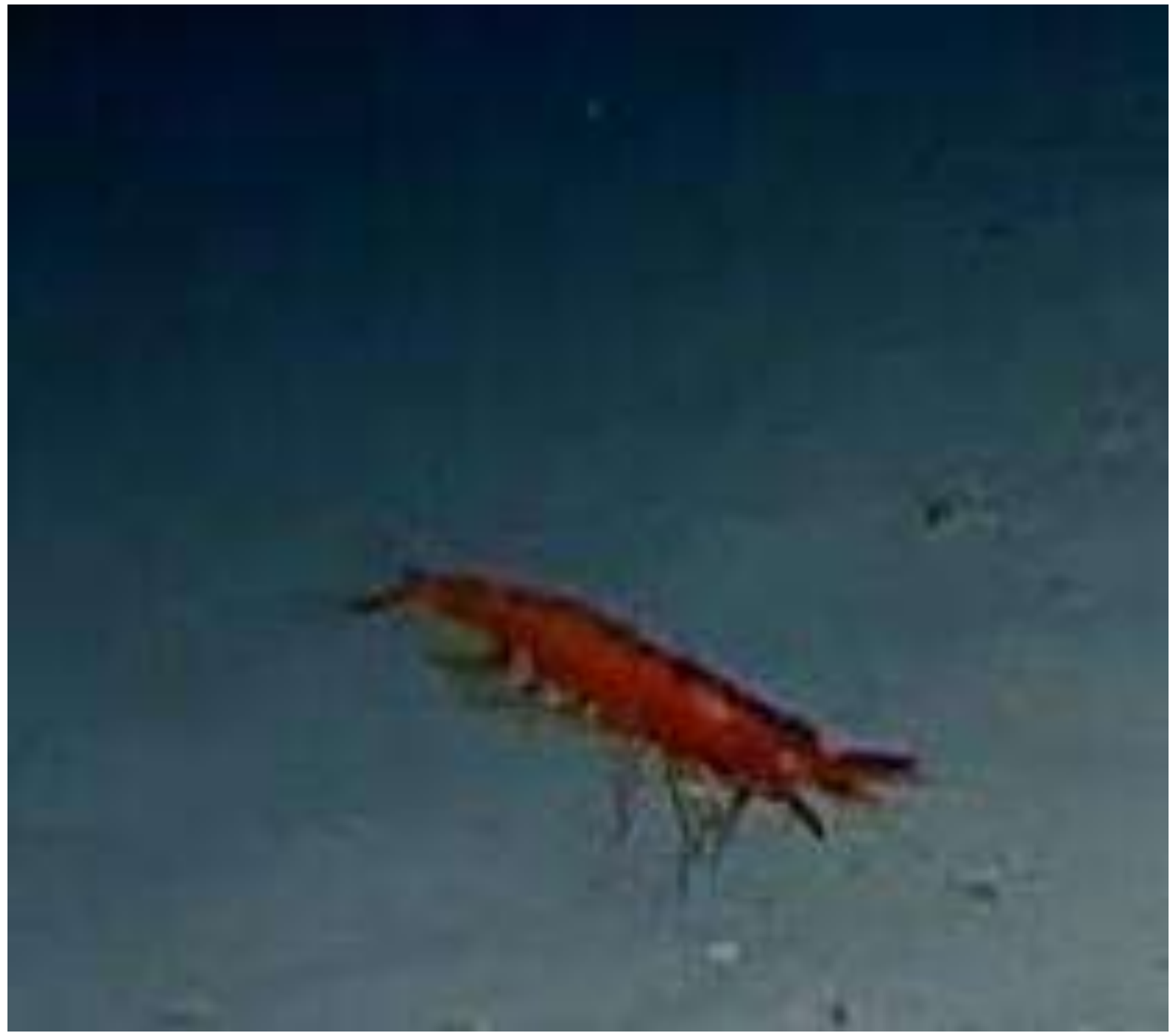

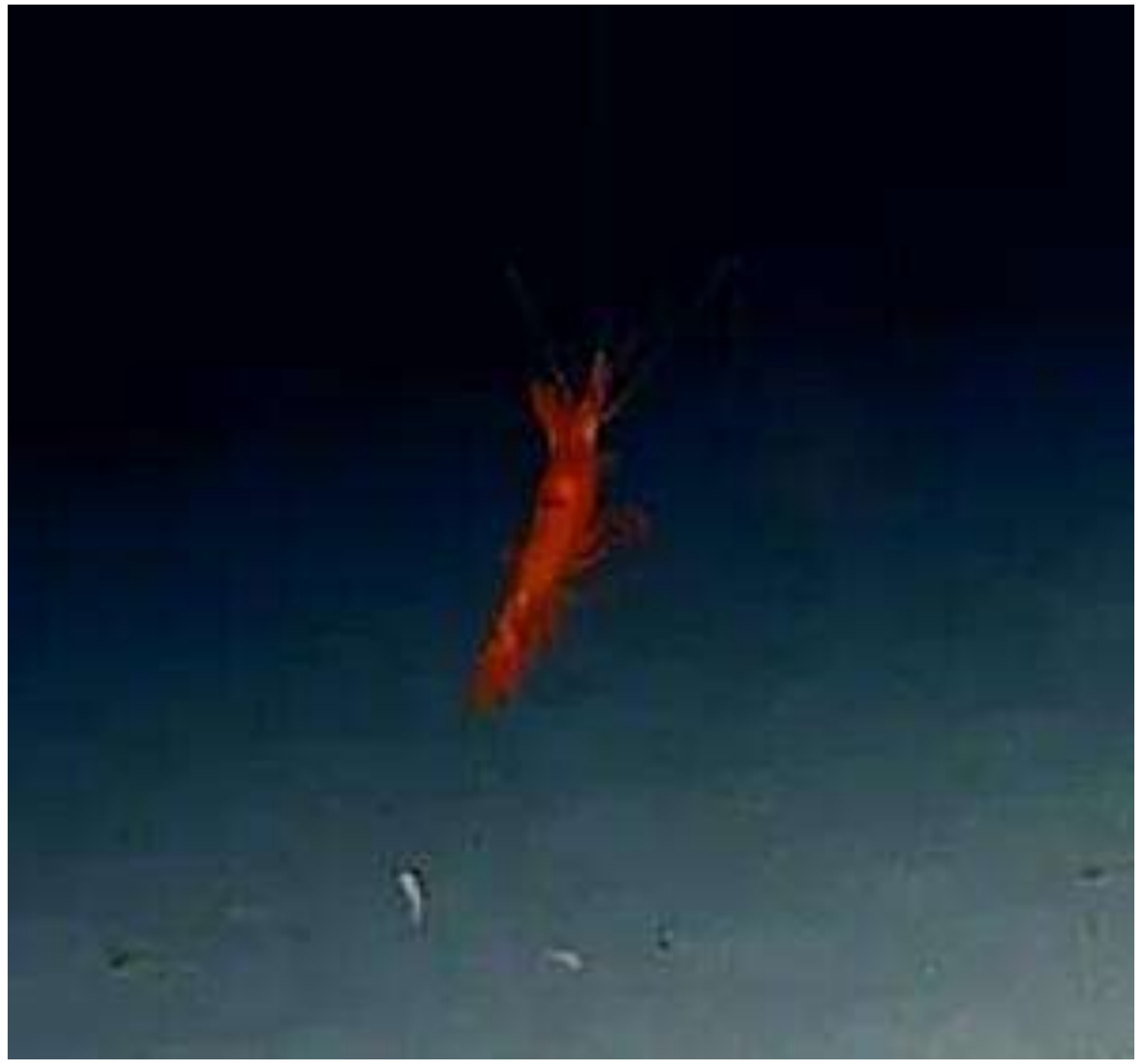

Red body. Short abdominal appendages. Large, red oval scaphocerites.

Malacostraca; Decapoda; Benthesicymidae; Benthesicymus; *Benthesicymus crenatus*

NC1_OM1_6300_NOR_0059

NC1_CR3_6700_NOR_0112

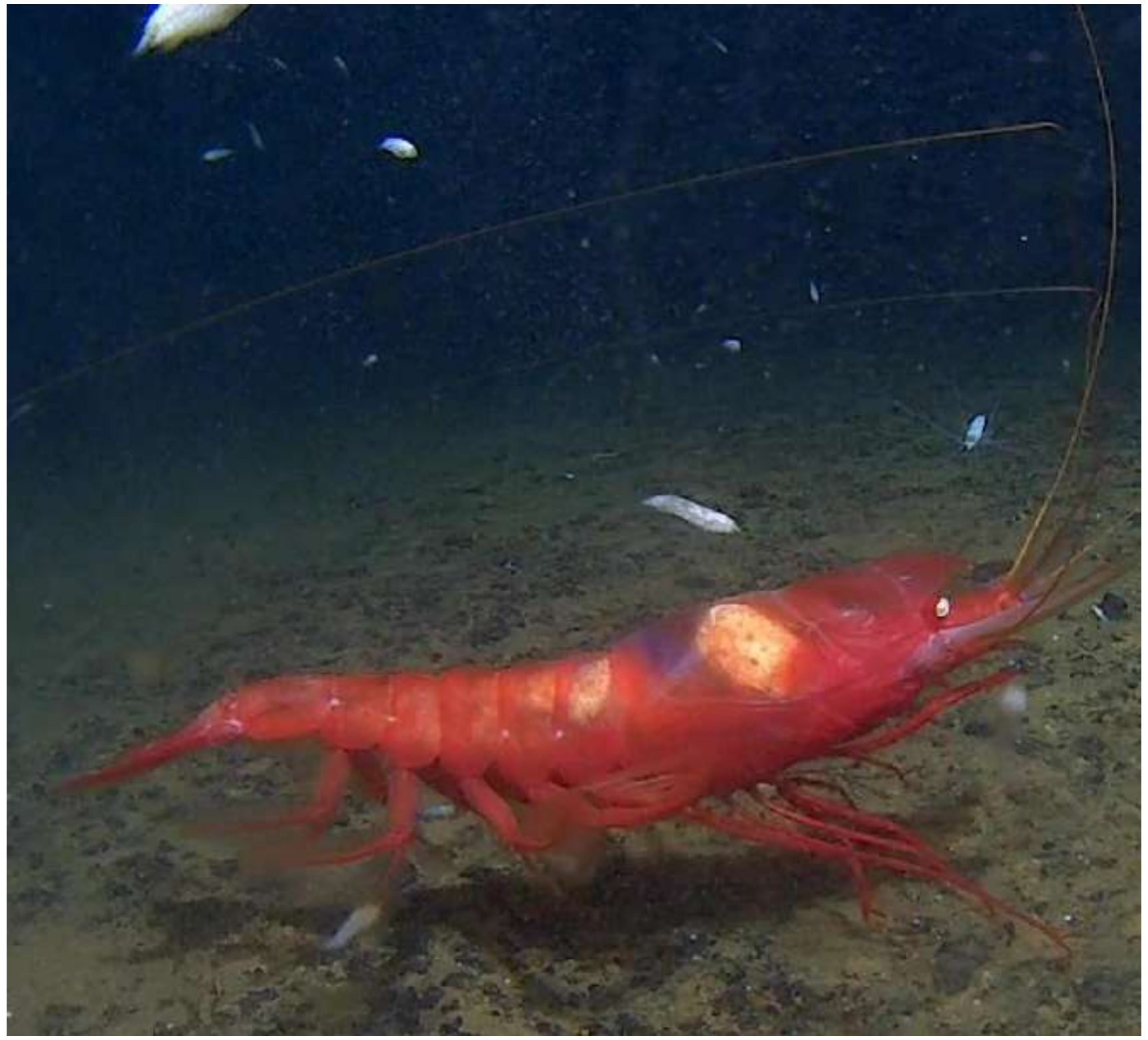

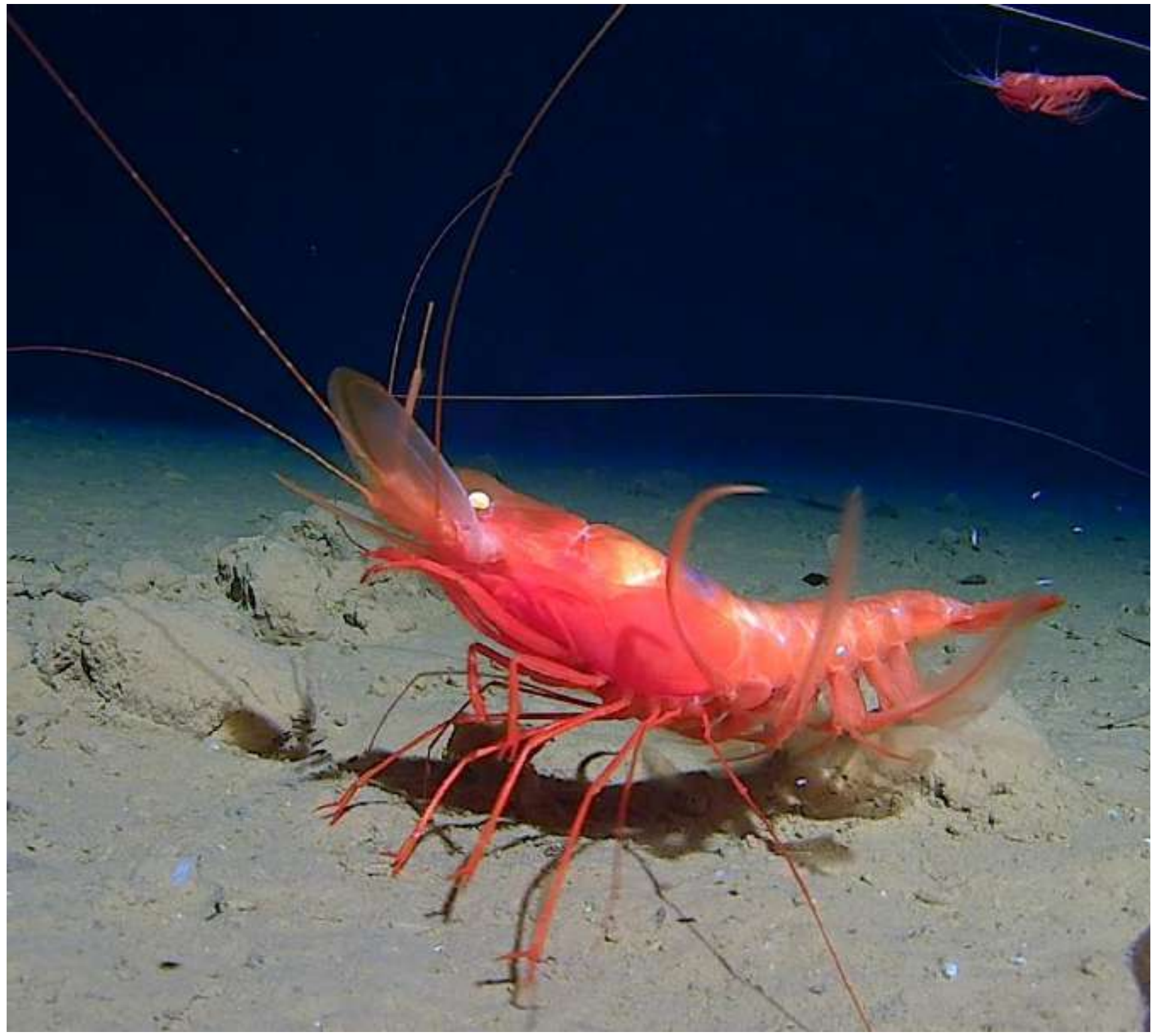

Red, translucent body with dorsally darker patch around the centre of the body. Long abdominal appendages. Antennae long and yellowish orange in colour. Large, white and semi-transparent oval scaphocerites.

Malacostraca; Decapoda; Galatheoidea; *Munidopsis* sp. indet. 1

NC3_MA8_2800_NOR_0055

NC3_MA9_3700_NOR_0096

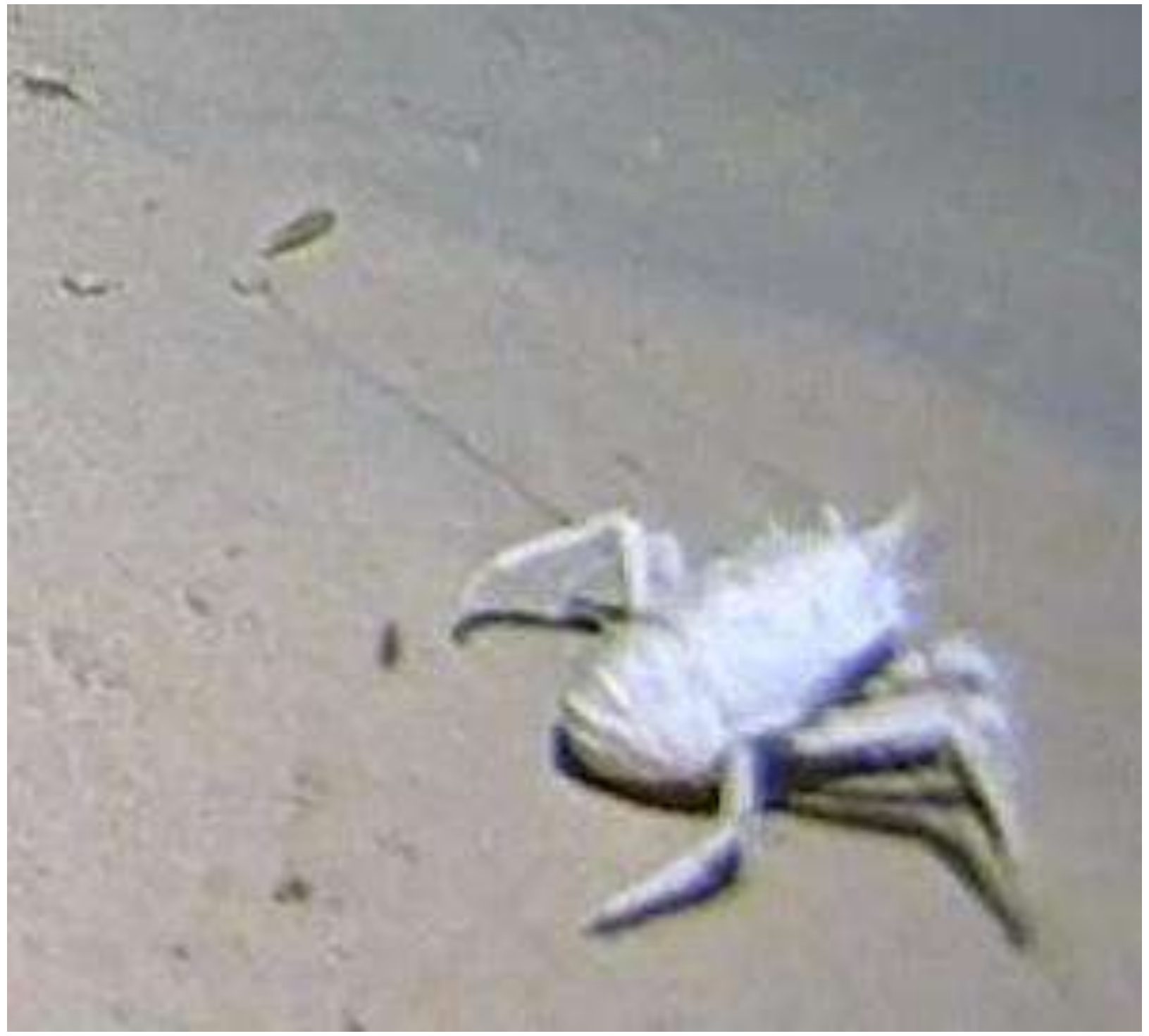

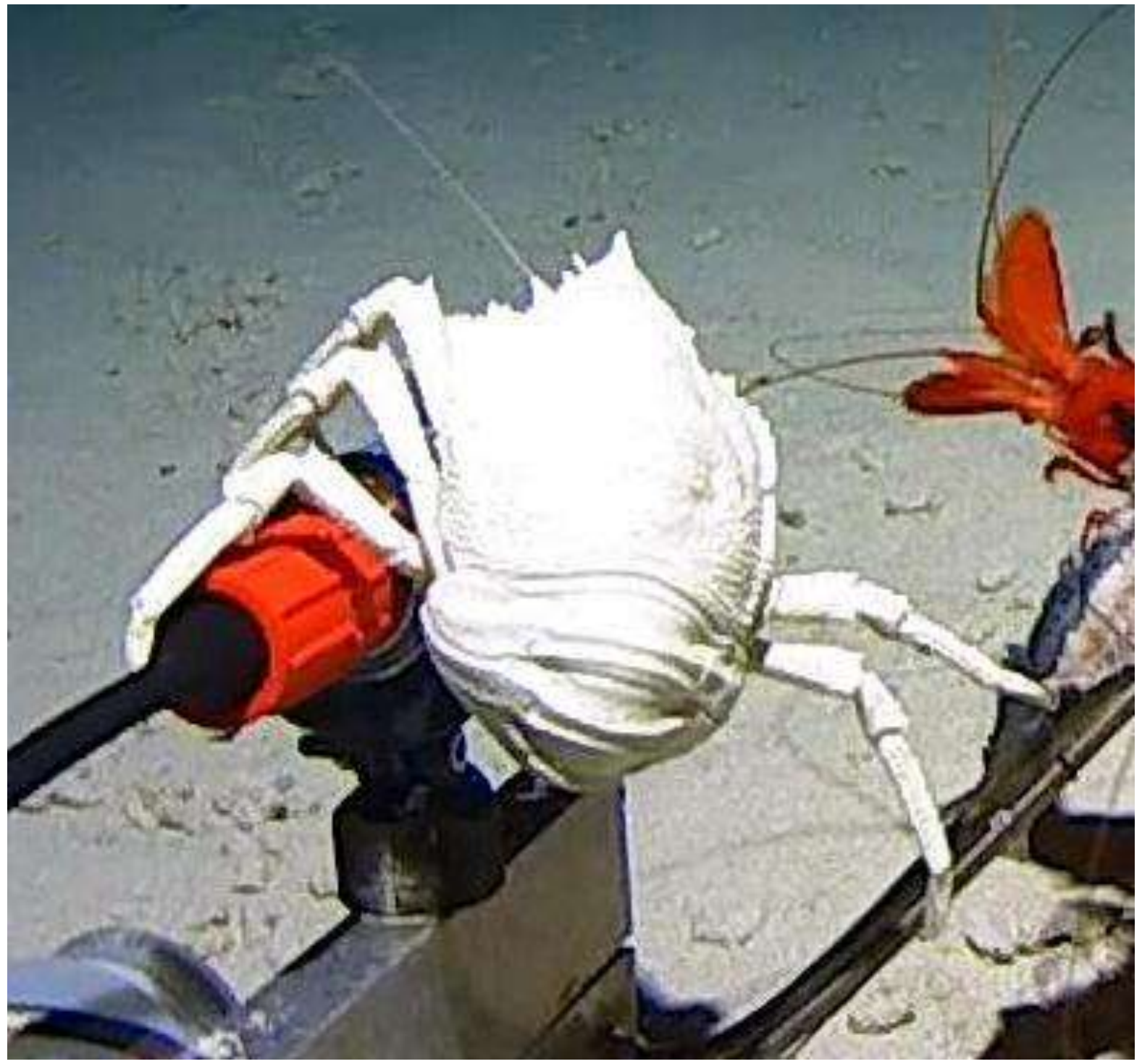

Pale-white squat lobster with 3 pairs of ambulatory appendages. Pincers are short and robust. Granules on the dorsal surface of body and pereiopods.

Malacostraca; Decapoda; Galatheoidea; *Munidopsis* spp.

NC4_BK4_4700_NOR_0059

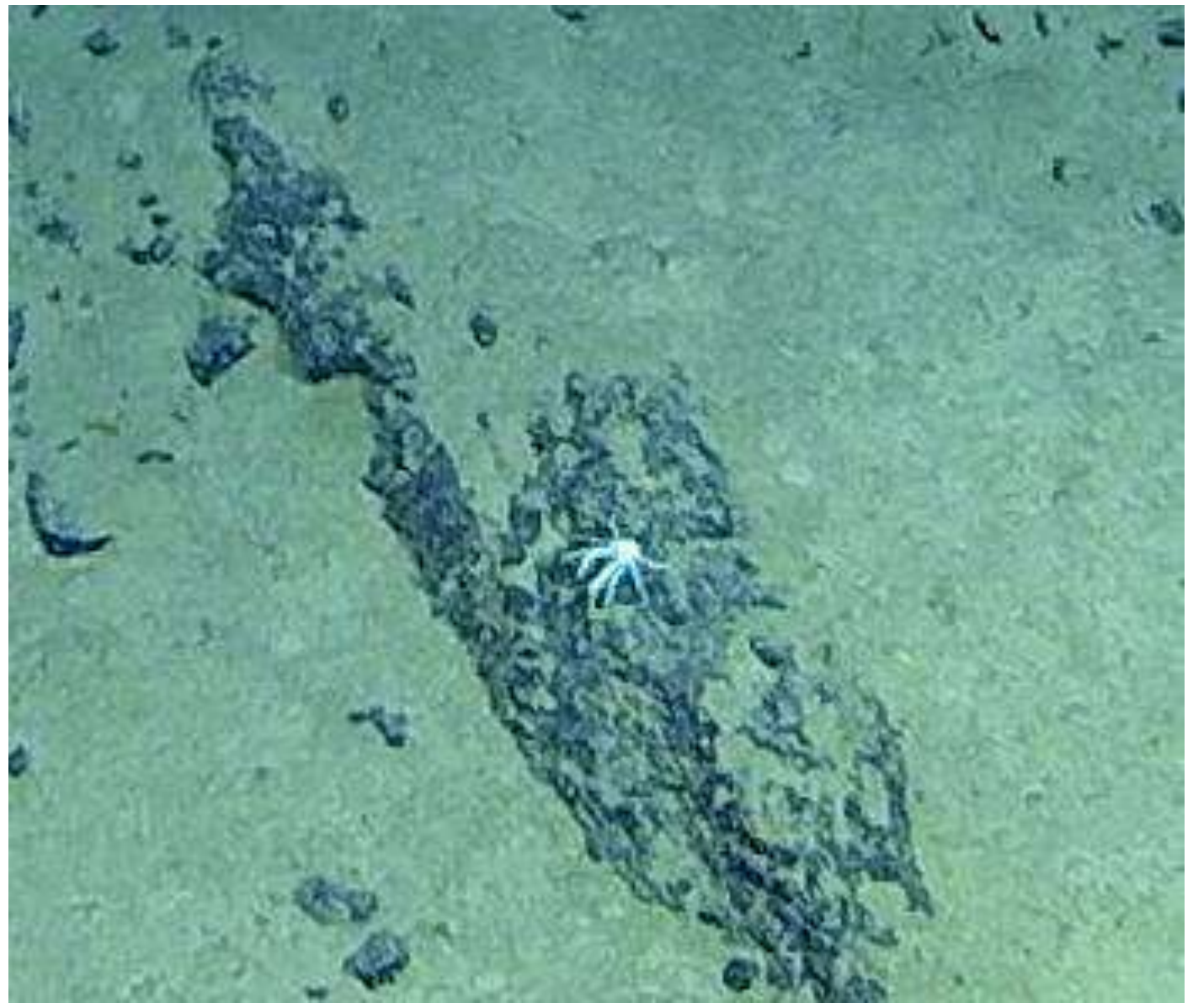

Pale-white squat lobster with 3 pairs of ambulatory appendages. Pincers are short and robust. Granules on the dorsal surface of body and pereiopods.

Malacostraca; Decapoda; Decapoda fam. indet. 1

NC2_MA6_5700_NOR_0115
NC2_OM7_5000_NOR_0045
NC4_OM9_5700_NOR_0029

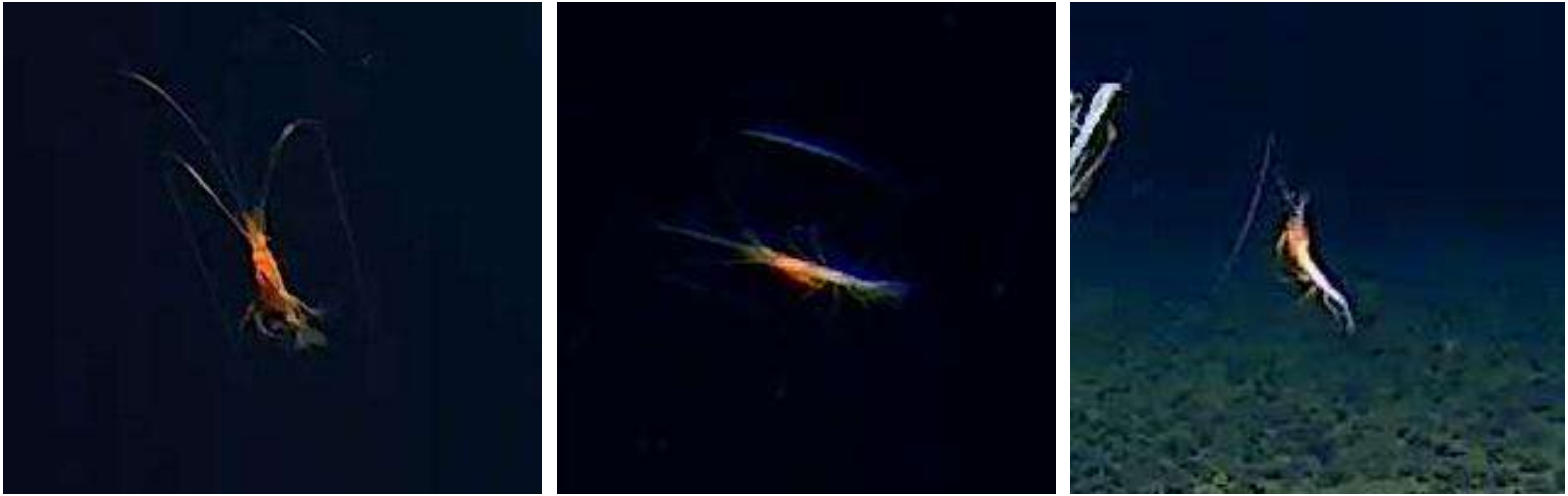

Bright orange-yellow body. Carapace slim and tapers to the abdomen with elongate rostrum and large oval opaque-yellow. Antennae extend roughly 2x the body length and yellow. Short abdominal appendages with wide, translucent ‘tail fan’.

Malacostraca; Decapoda; Solenaceridae; *Hymenopenaeus* spp.

NC2_BK2_3800_NOR_0073
NC2_CR2_3500_NOR_0042

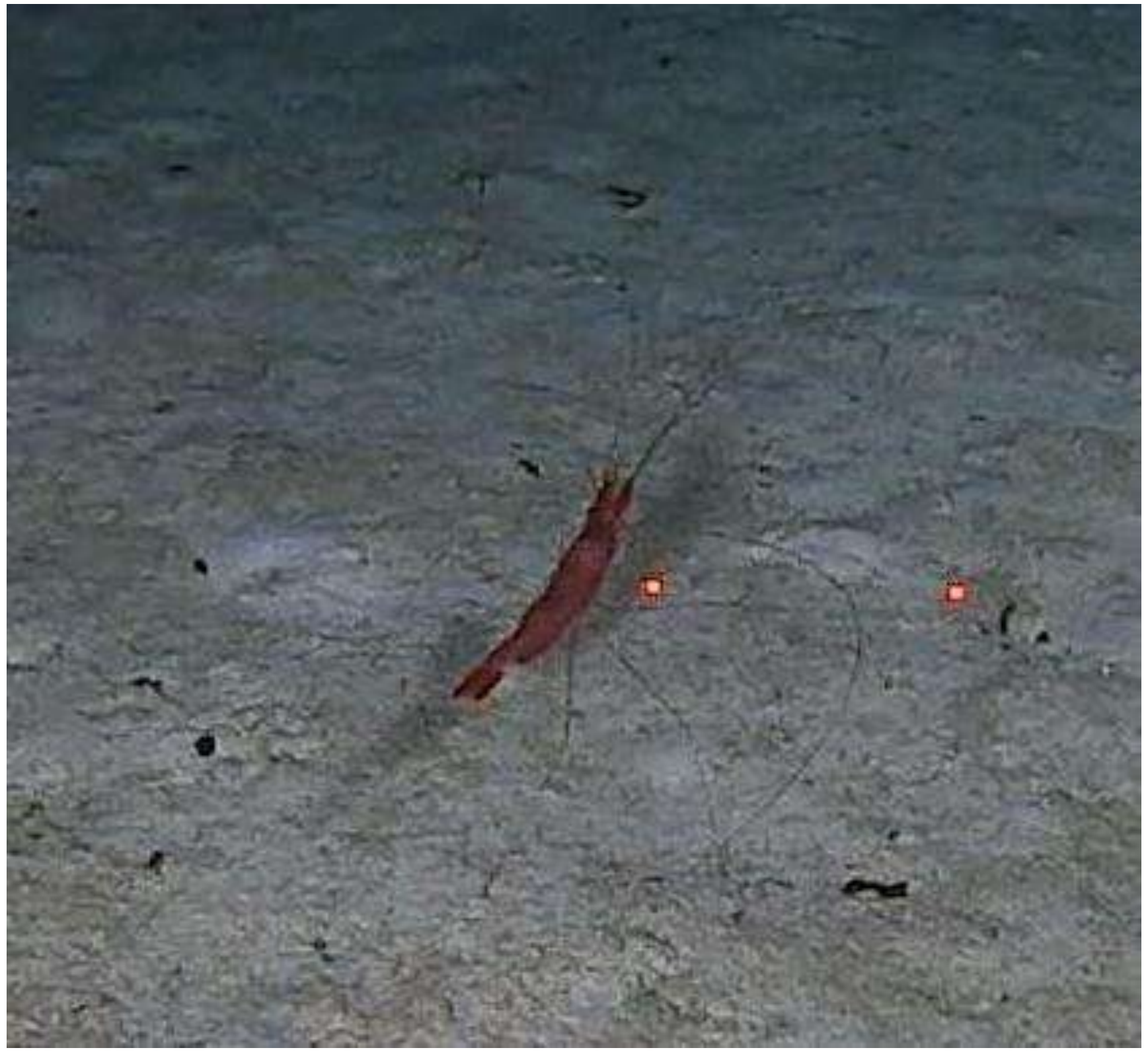

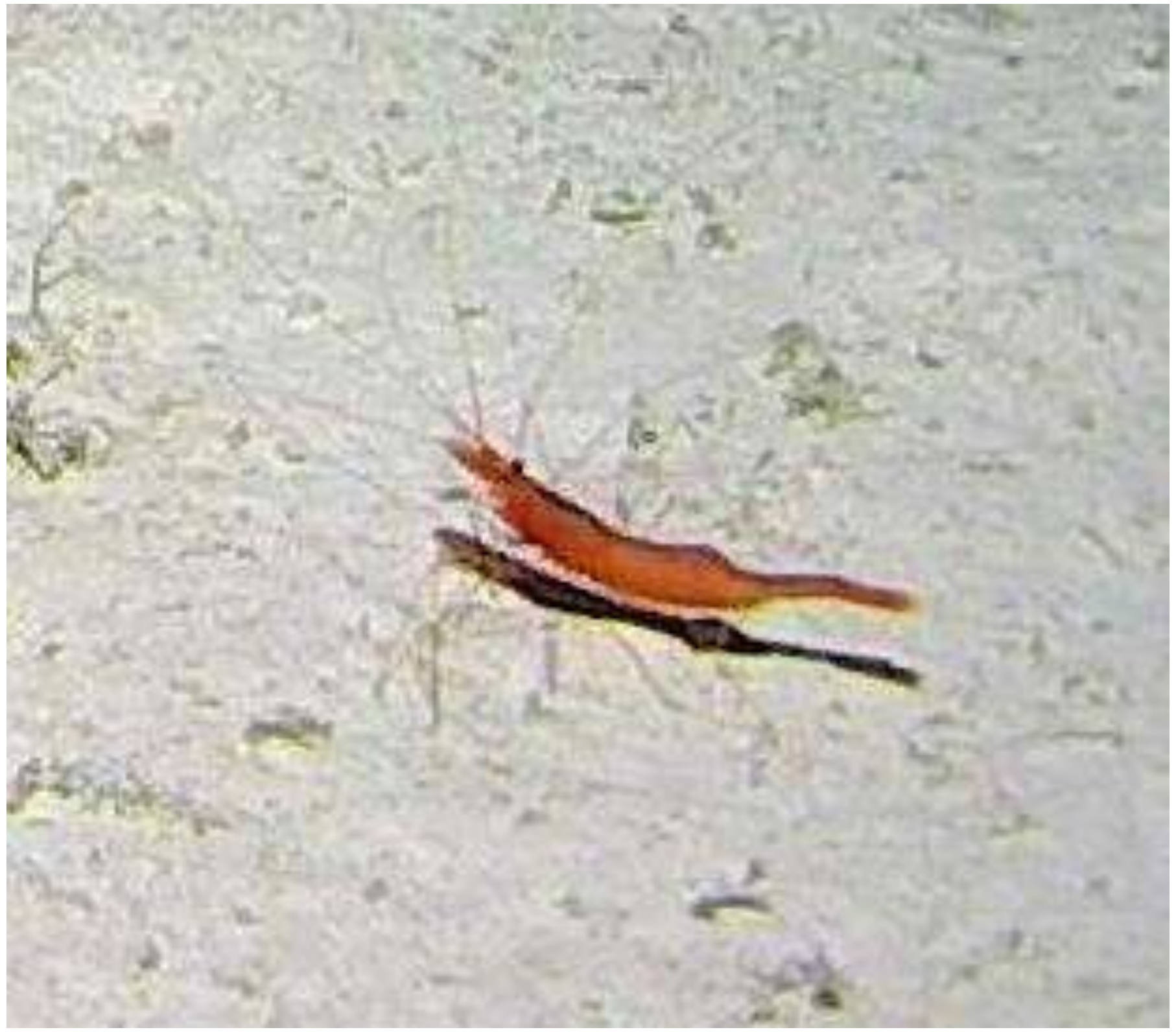

Red slender abdomen and short carapace with long forward-facing antennal scales. Extremely long, very slender ambulatory appendages. Narrow, 'cup' shaped posterior termination.

Malacostraca; Decapoda; Paguroidea; Paguroidea spp.

NC2_BK1_3200_IMG_7195
NC2_BK1_3200_IMG_7198
NC2_OM1_3100_NOR_0022

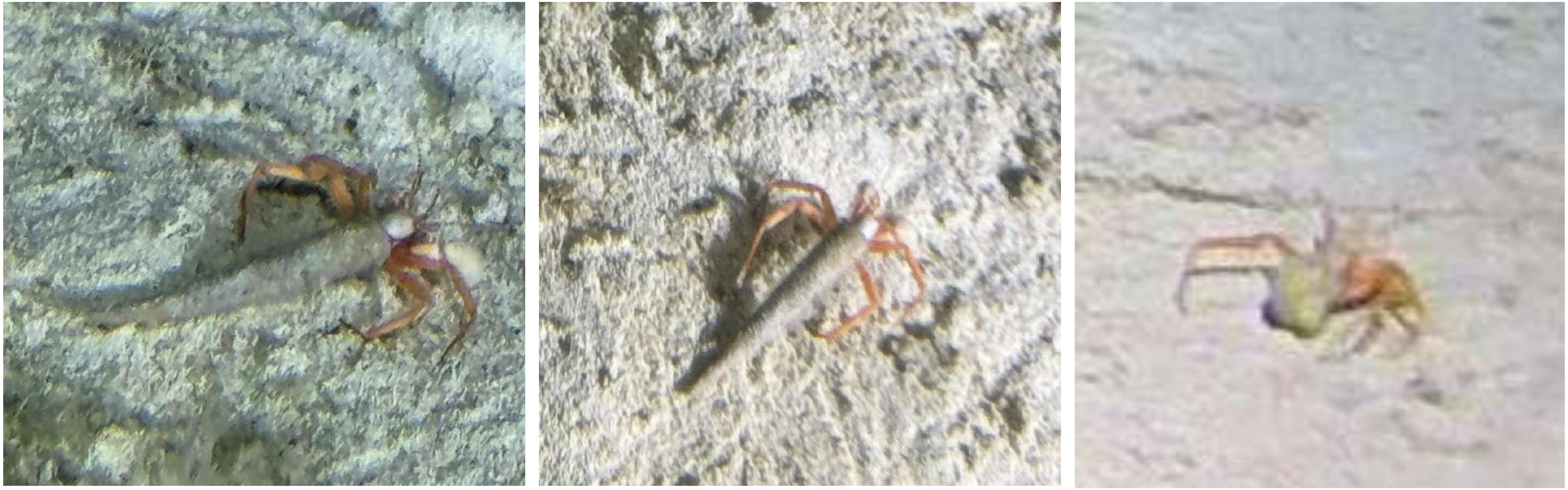

Orange-pink body with first 2 pairs of ambulatory appendages used for moving and visible. The left cheliped is smaller than the right.

Behavioural Diagnostic: Mollusc shells used as defence

Malacostraca; Decapoda; Paguroidea; Parapaguridae; *Sympagurus; Sympagurus* sp. indet. 1

NC2_BK1_3200_IMG_7410

NC2_BK2_3800_IMG_8104

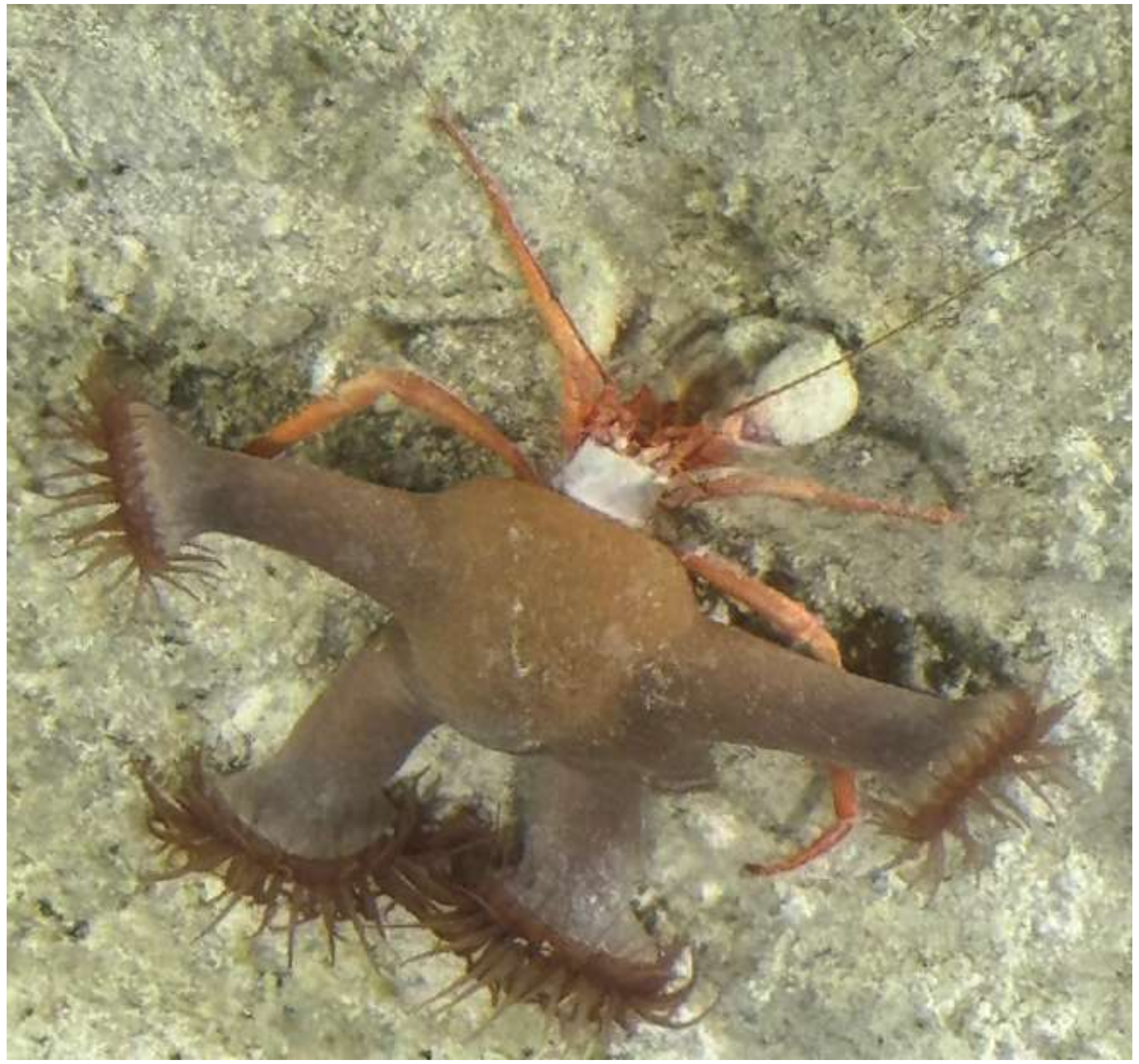

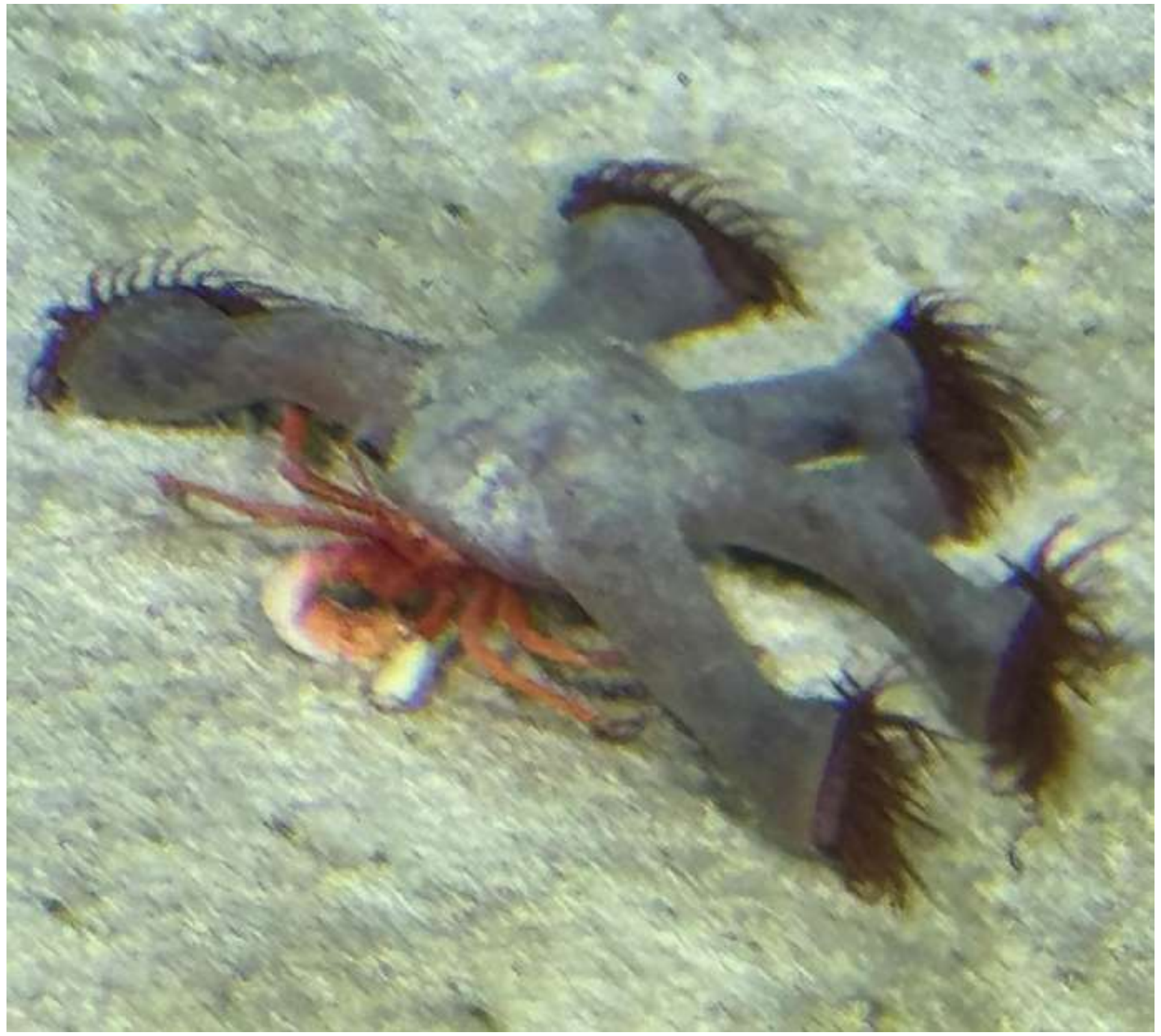

Orange-pink body with first 2 pairs of ambulatory appendages used for moving and visible. The chelipeds are large with the right cheliped larger than the left. Symbiotic, defensive relationship with zooanthids

Malacostraca; Isopoda; Ischnomesidae; Ischnomesidae gen. indet. 1

NC1_BK3_8000_NOR_0068

NC1_BK3_8000_IMG_6471

NC1_OM8_7900_NOR_0082

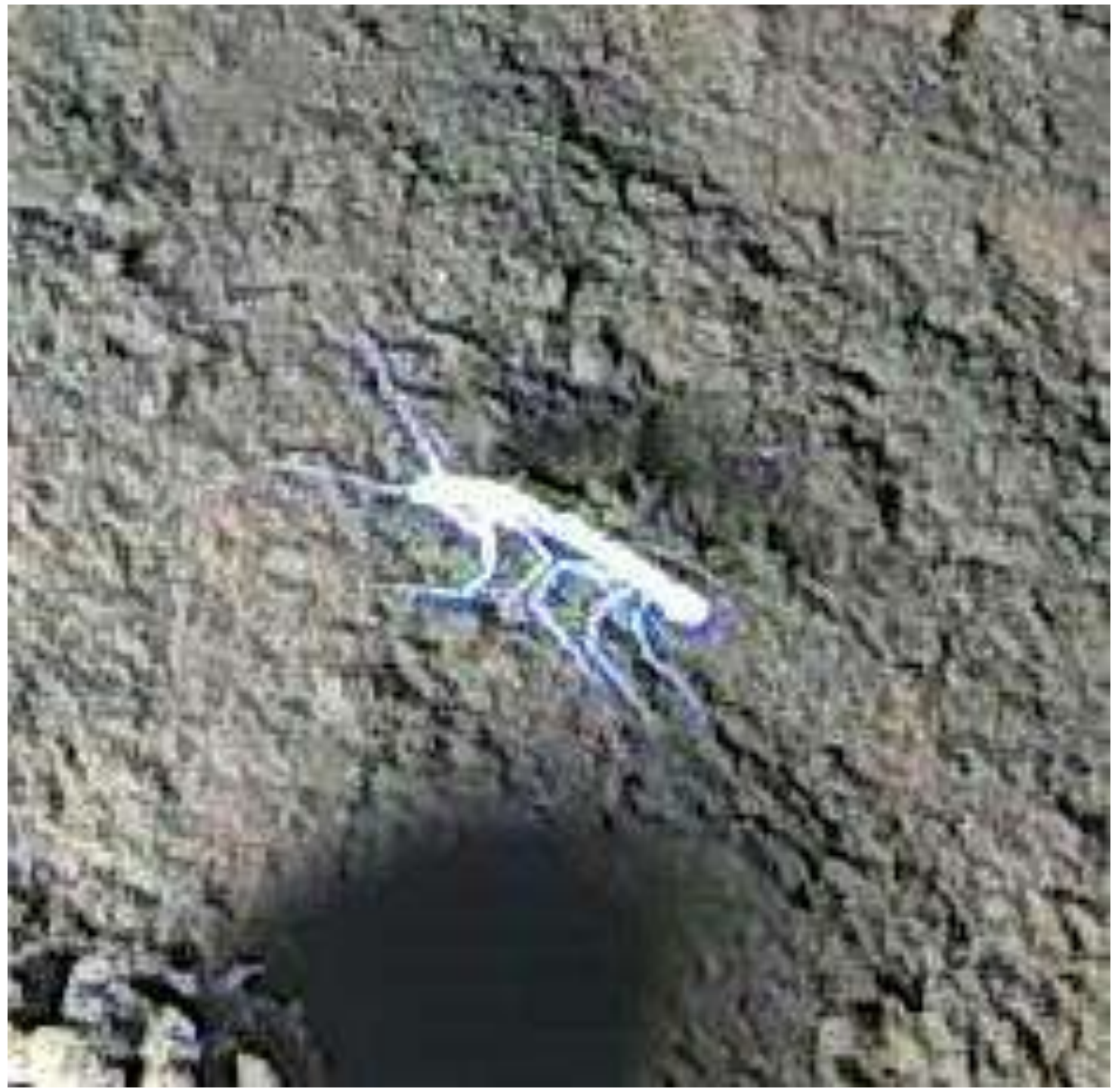

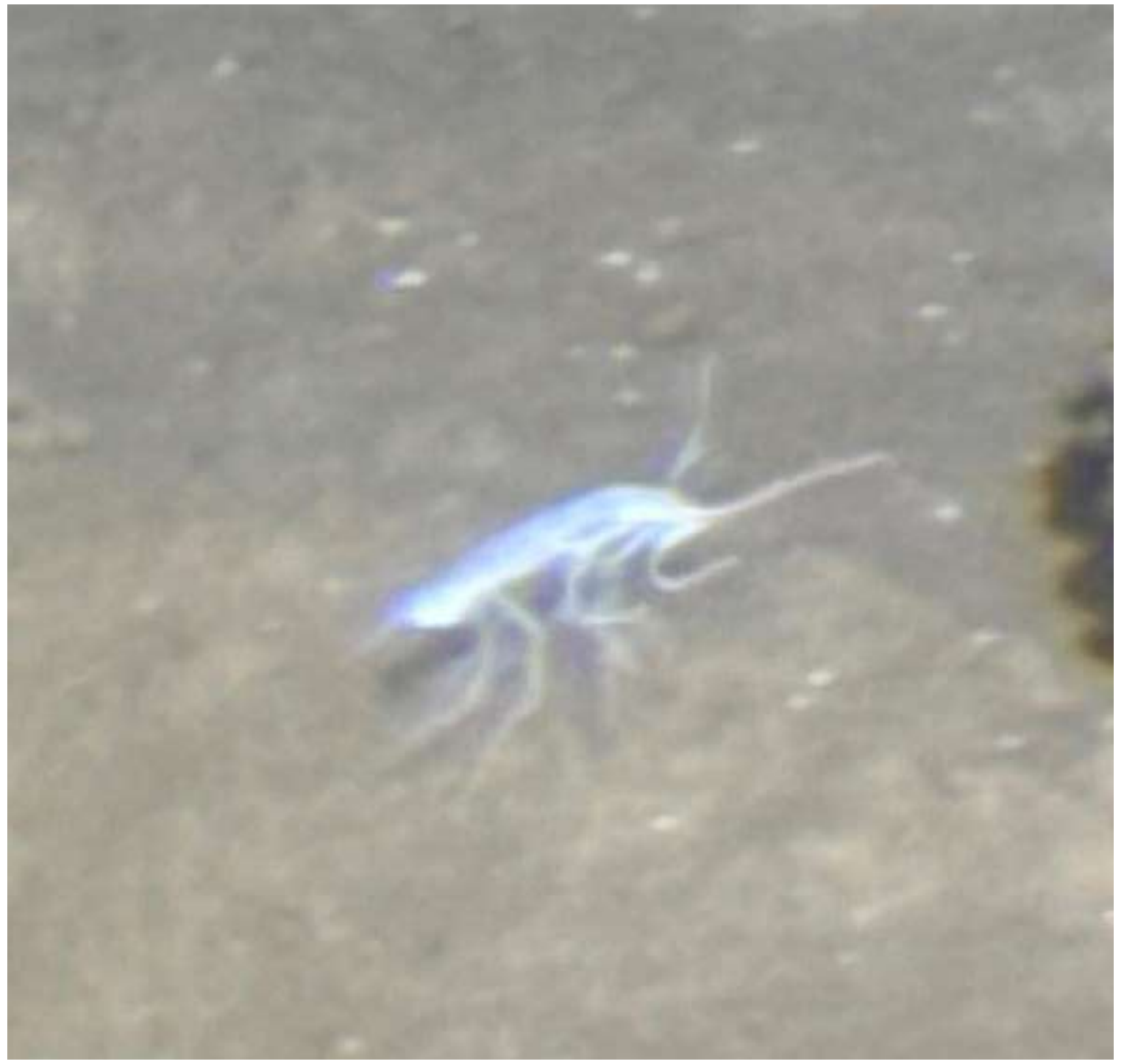

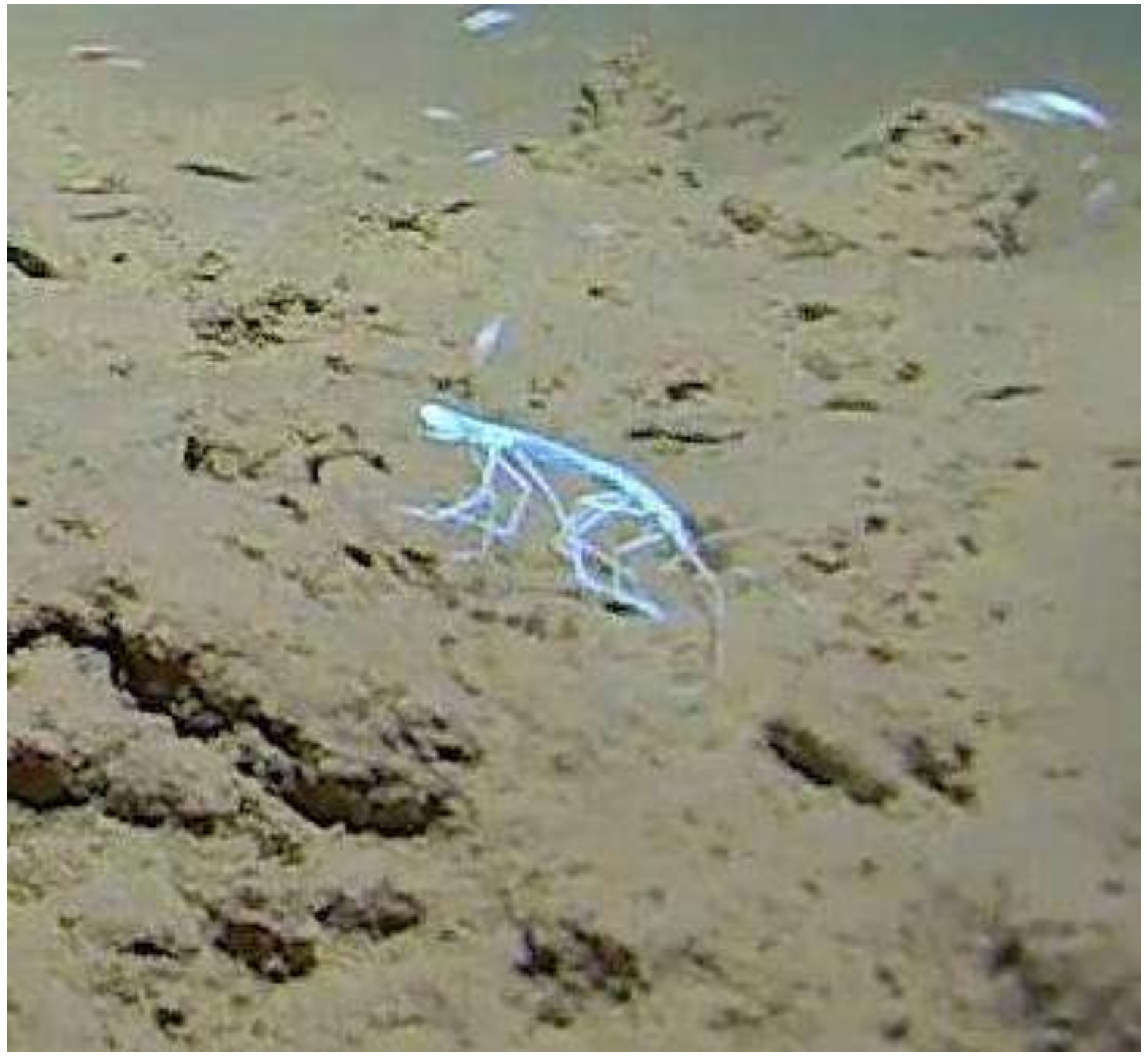

Body is white, elongated and narrow. Limbs long and slender with rounded 'tail-fin'.

Malacostraca; Isopoda; Ischnomesidae; Ischnomesidae gen. indet. 2

NC1_CR1_NOR_0030

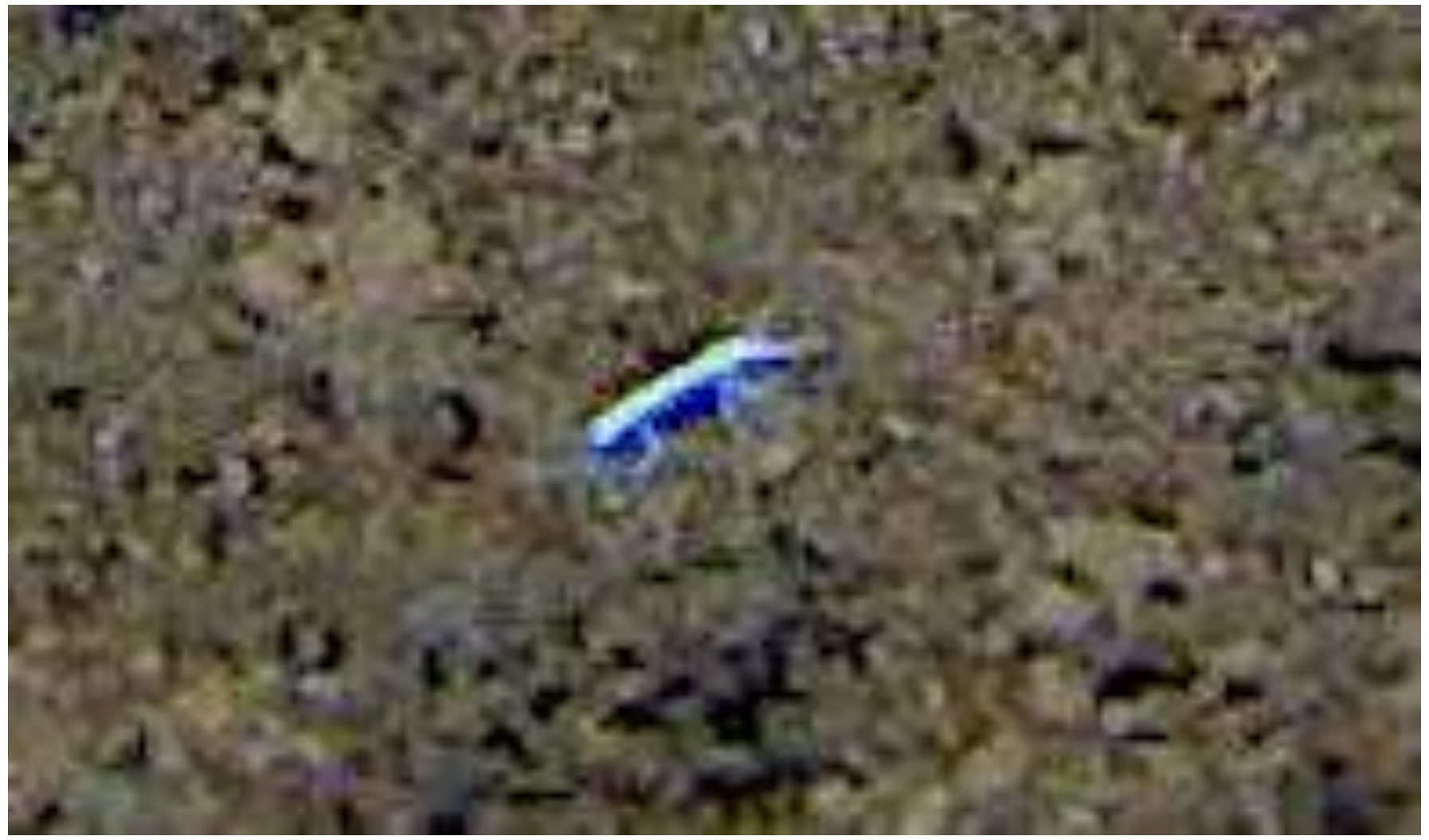

Body is white, elongated and narrow. Limbs long and slender with rounded 'tail-fin'.

Malacostraca; Isopoda; Munnopsidae; *Munnerycope* sp. indet. 1

NC1_CR8_7000_NOR_0100

NC1_CR8_7000_NOR_0101

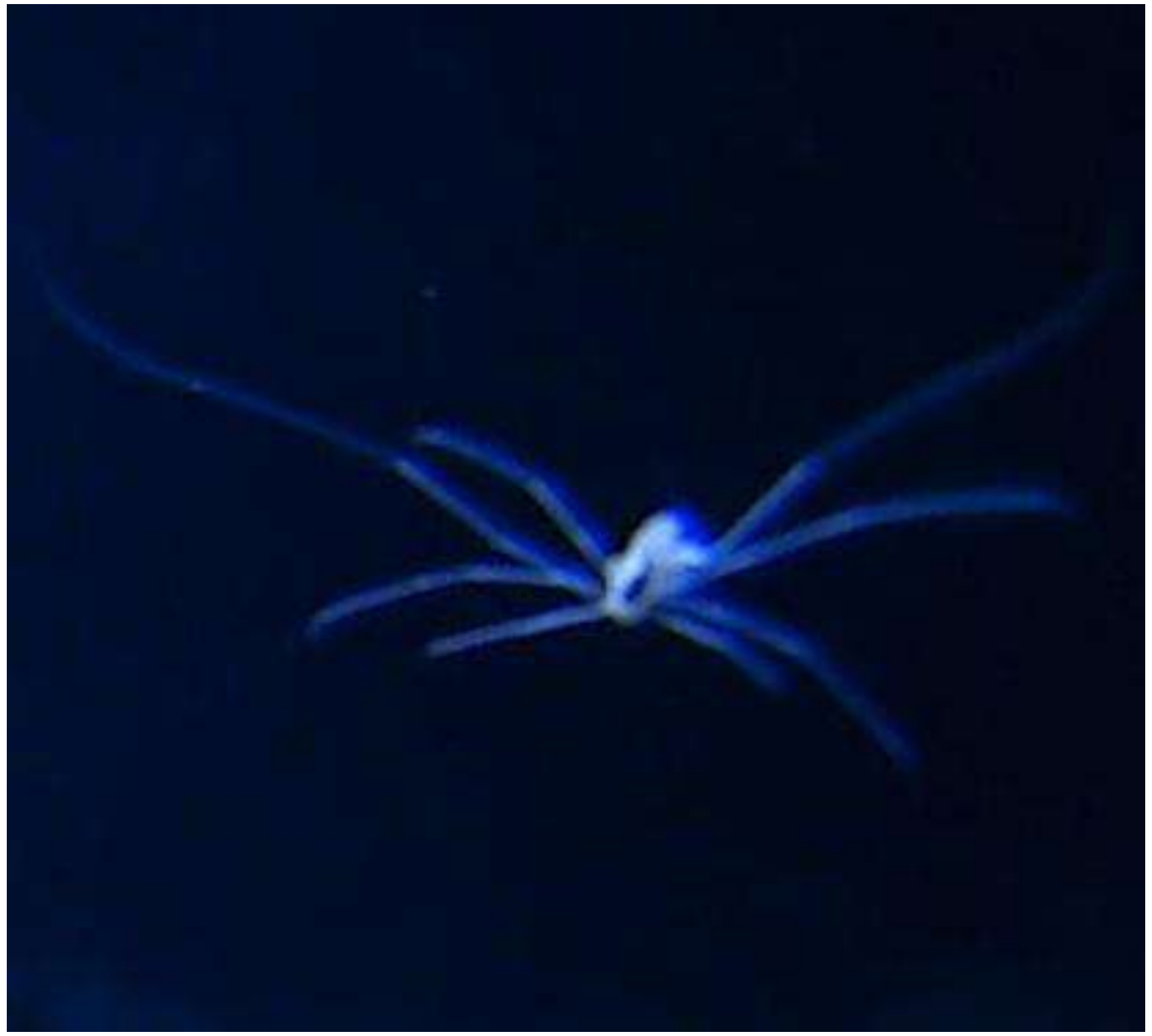

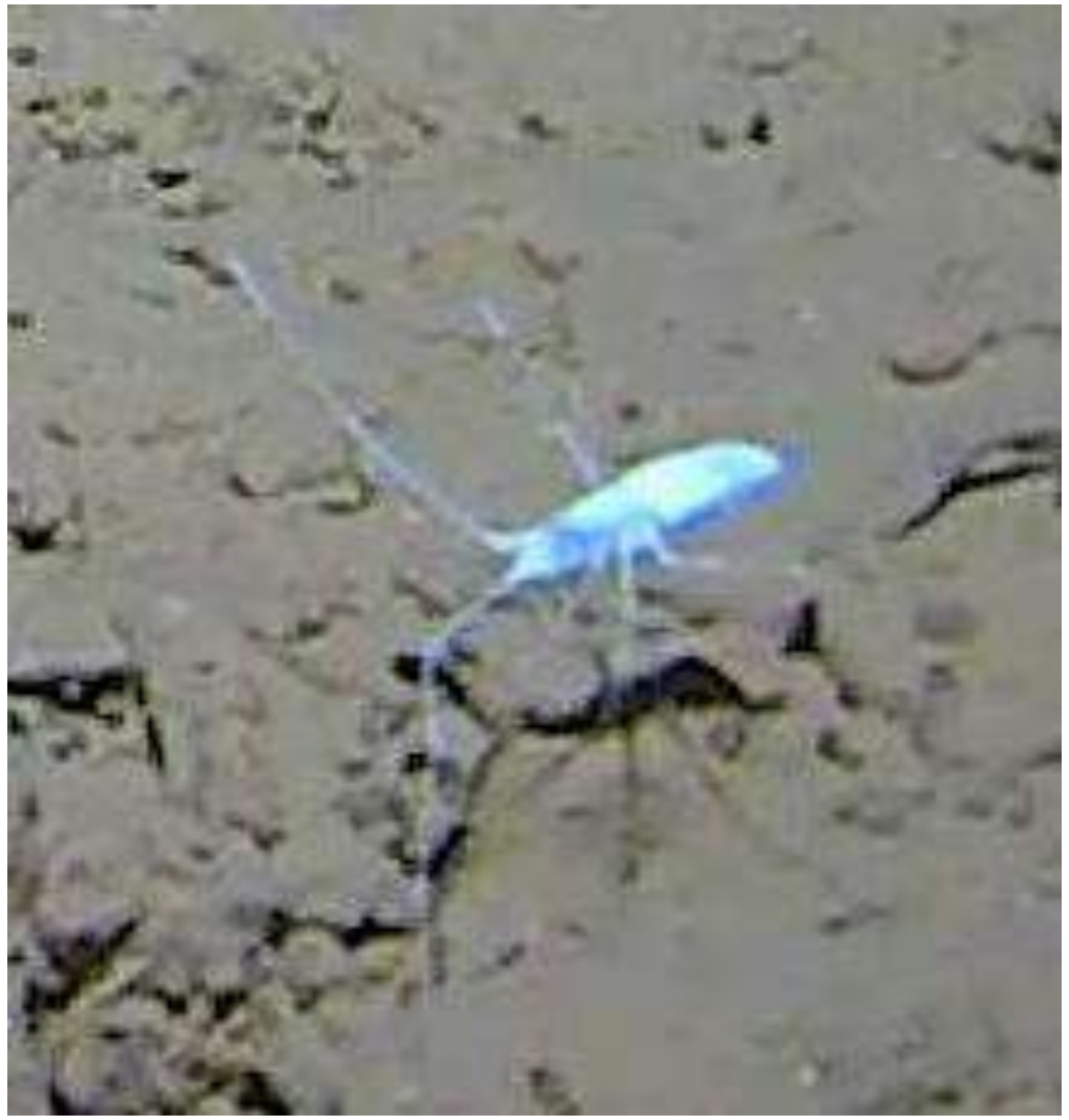

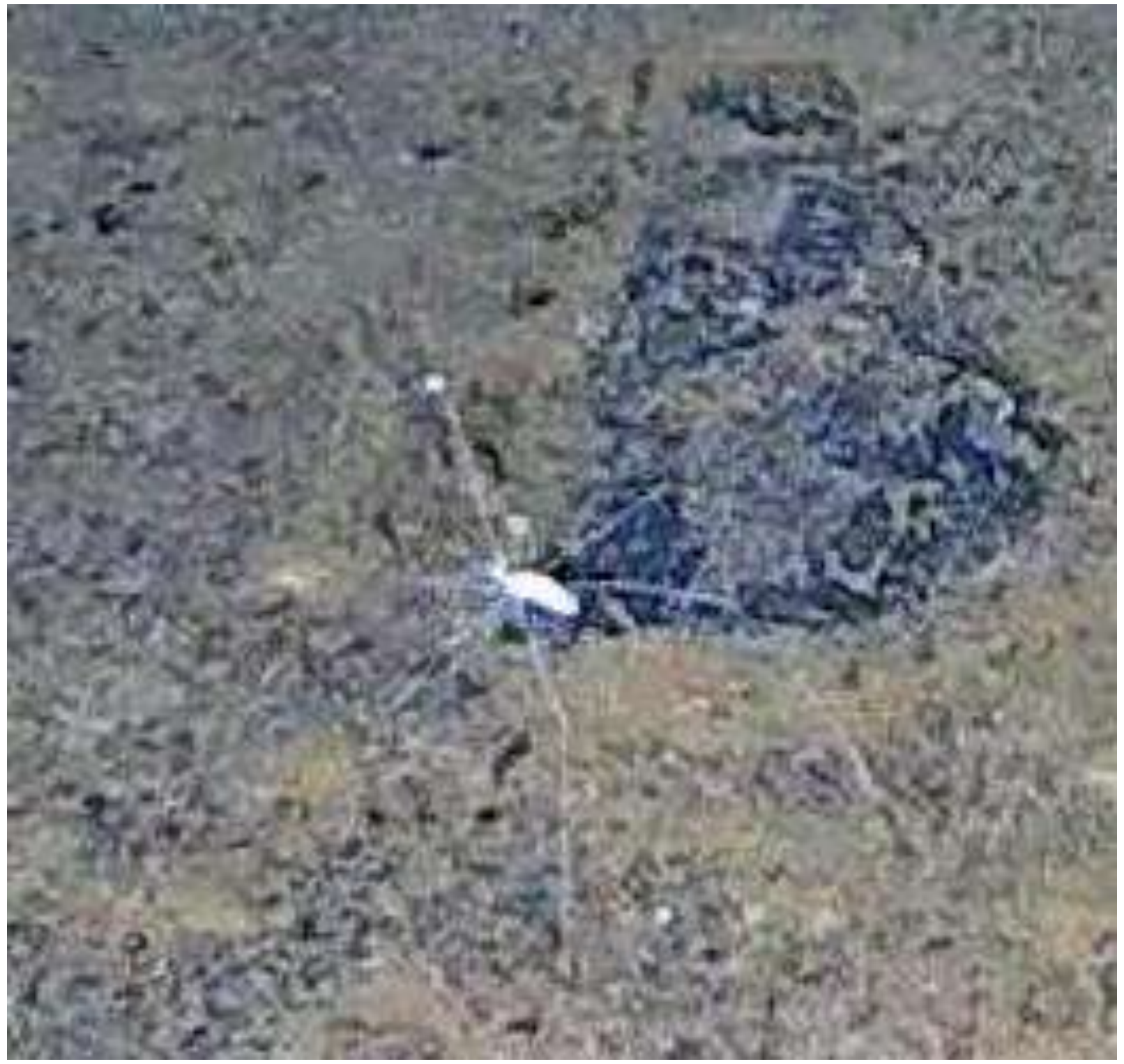

Body is white, ovoid and narrow. Limbs long and slender. Cf Munnerycope.

.

Malacostraca; Isopoda; Munnopsidae; *Storthyngurinae* gen. indet. 1

NC1_OM1_6300 _NOR_0076

NC1_CR2_6500_NOR_0065

NC3_OM3_6700_NOR_0045

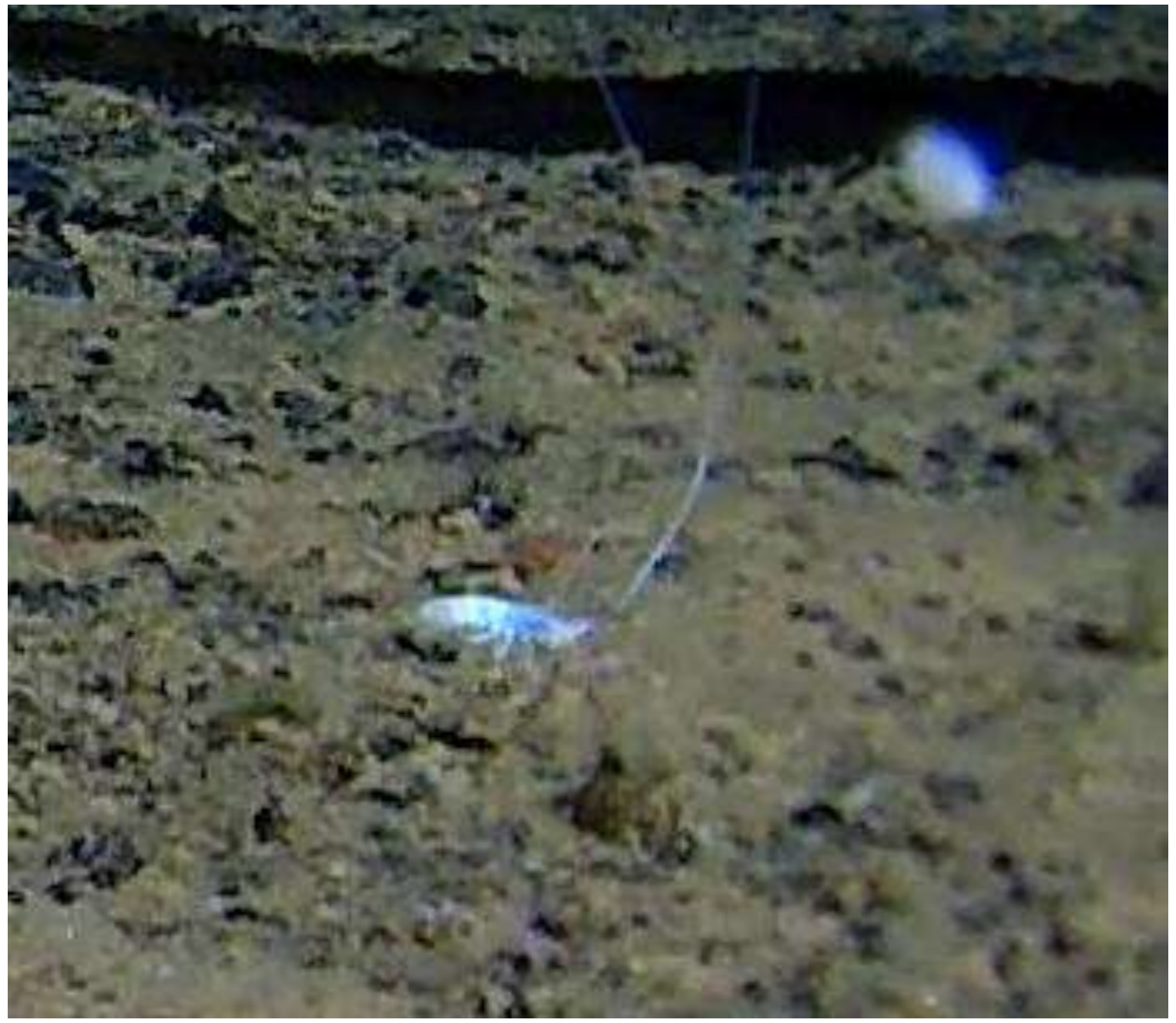

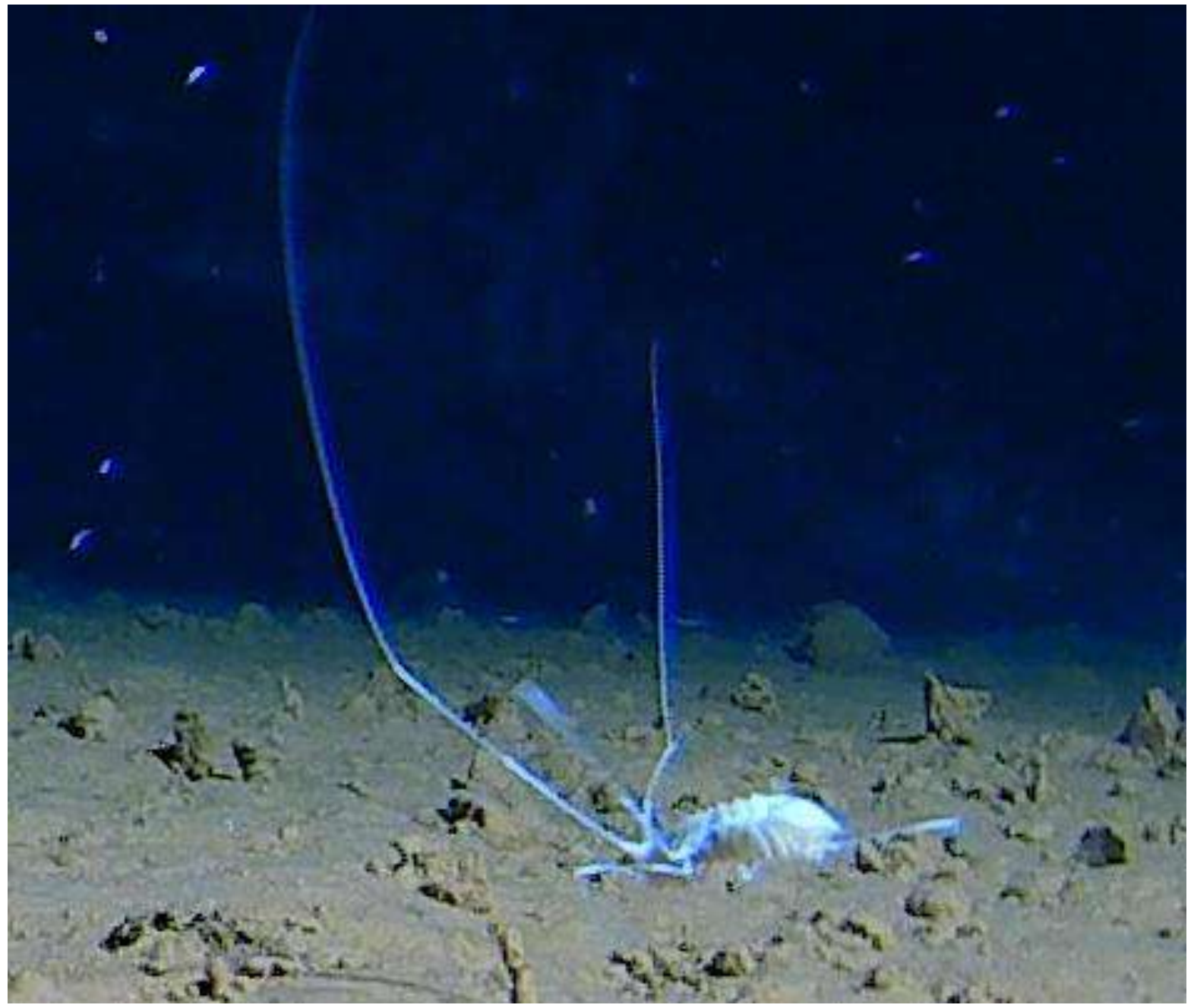

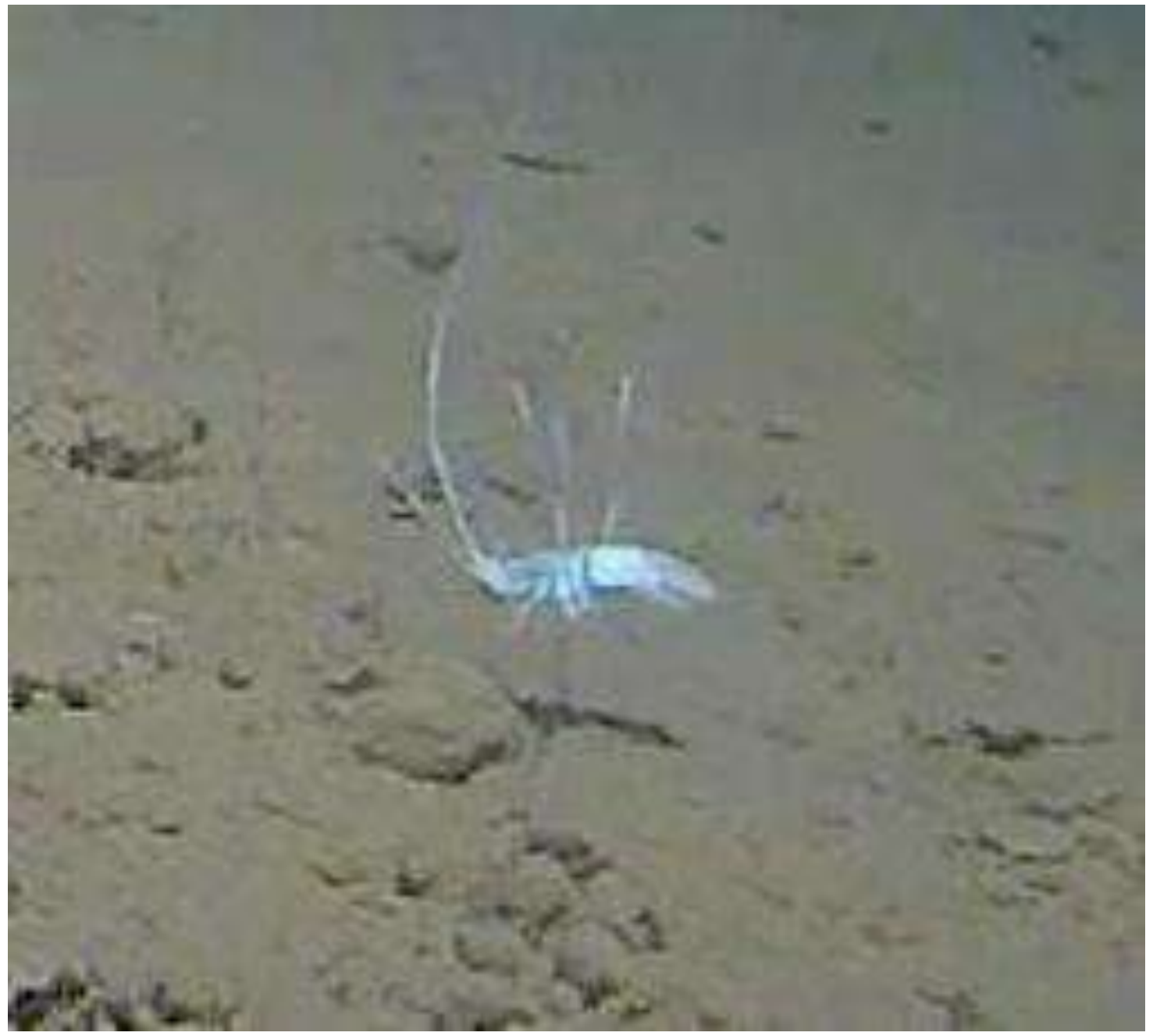

Body is white, ovoid and large. Ambulatory limbs are short while antennae are 2x-3x longer than the torso.

Malacostraca; Mysida; Mysidae; Mysidae spp.

NC2_BK1_3200_NOR_0050

NC1_OM2_6000_NOR_0108

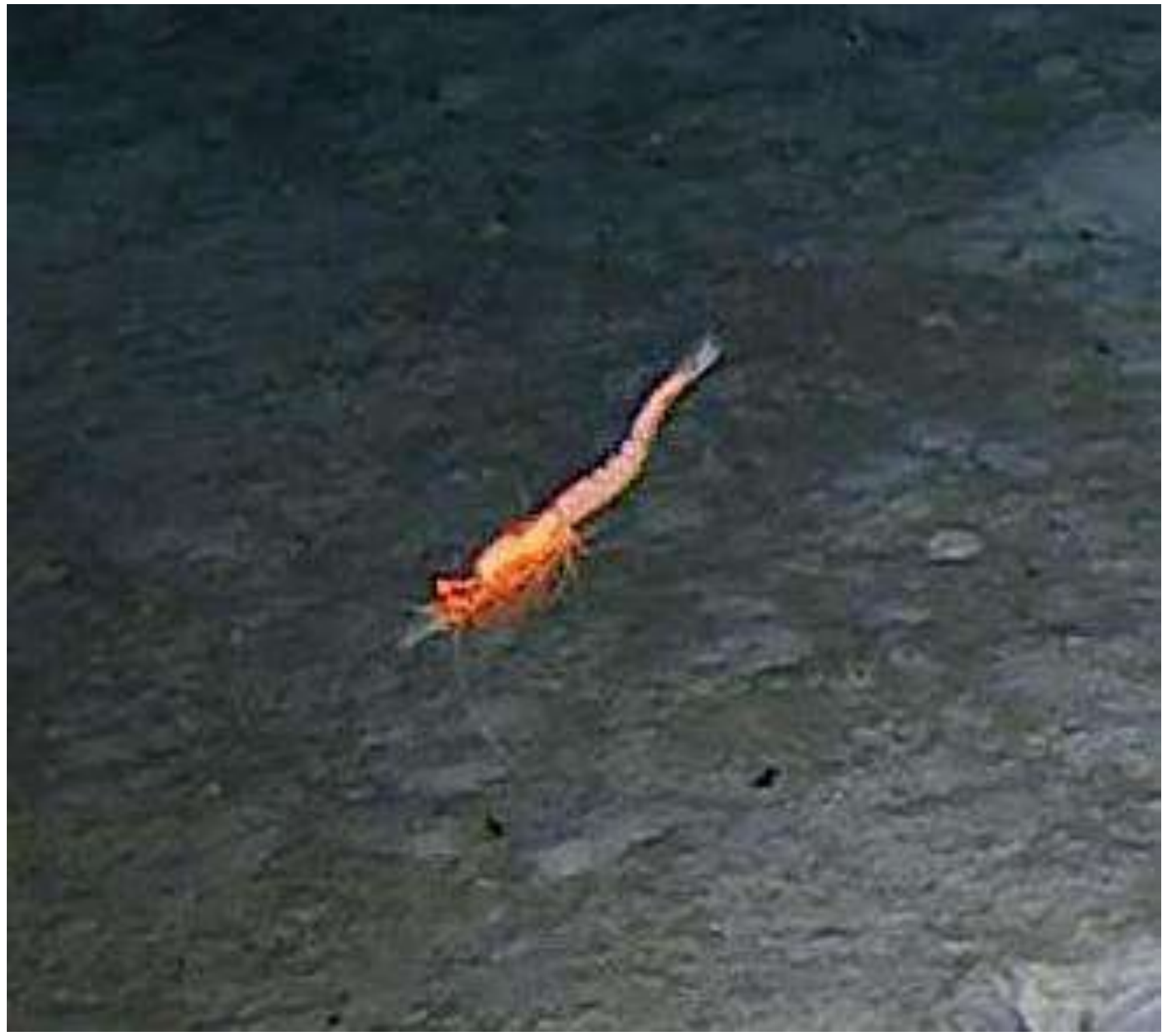

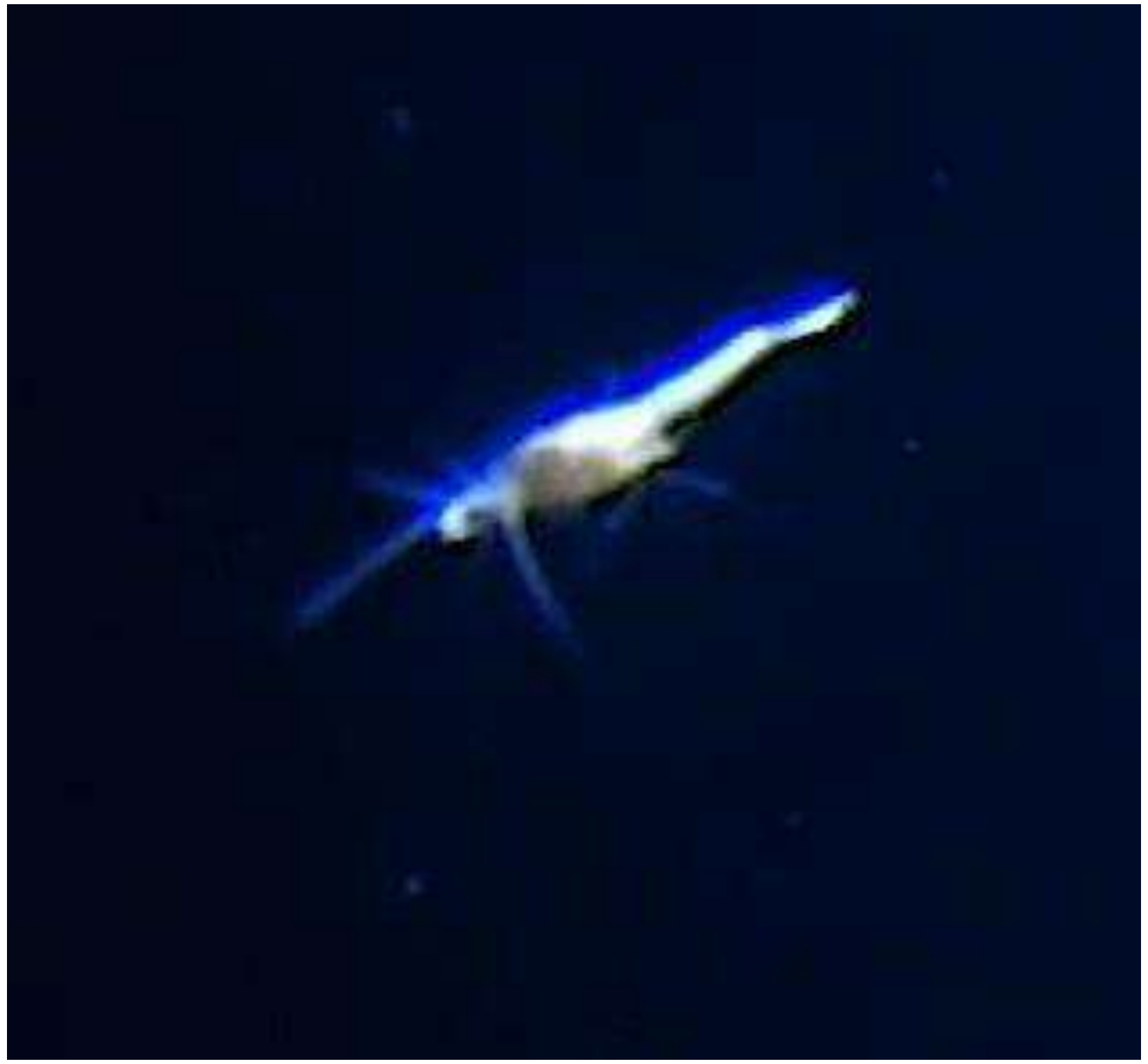

Body colour variable. Thick cephalothorax segmented from thin, tapering abdomen. Translucent ‘tail-fin’ observed.

Thecostraca; Scalpellomorpha; Scalpellidae; Alcockianum; *Alcockianum* sp. indet. 1

NC2_BK2_3800_IMG_8274

NC2_BK2_3800_IMG_8278

NC2_BK2_3800_IMG_8198

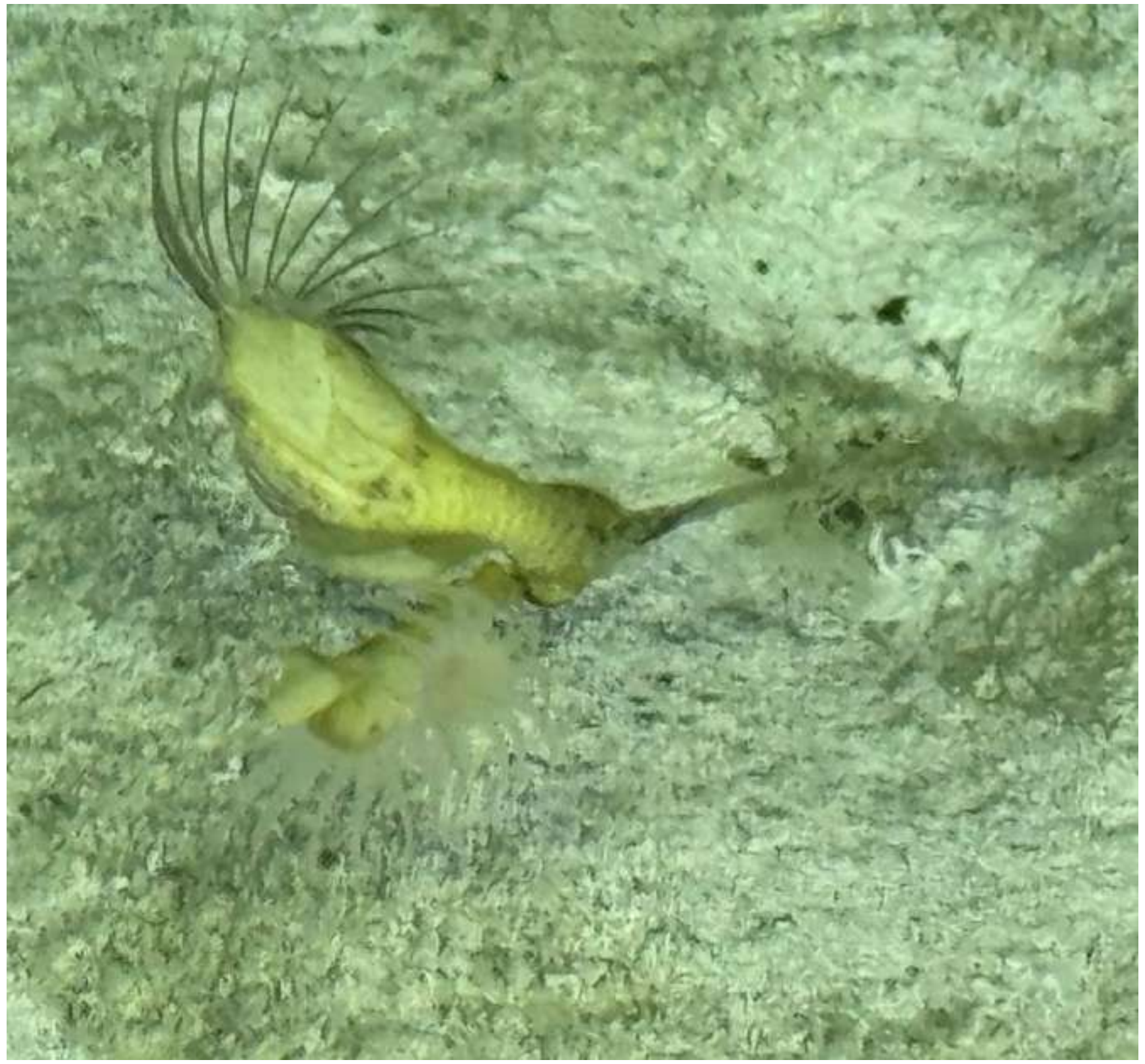
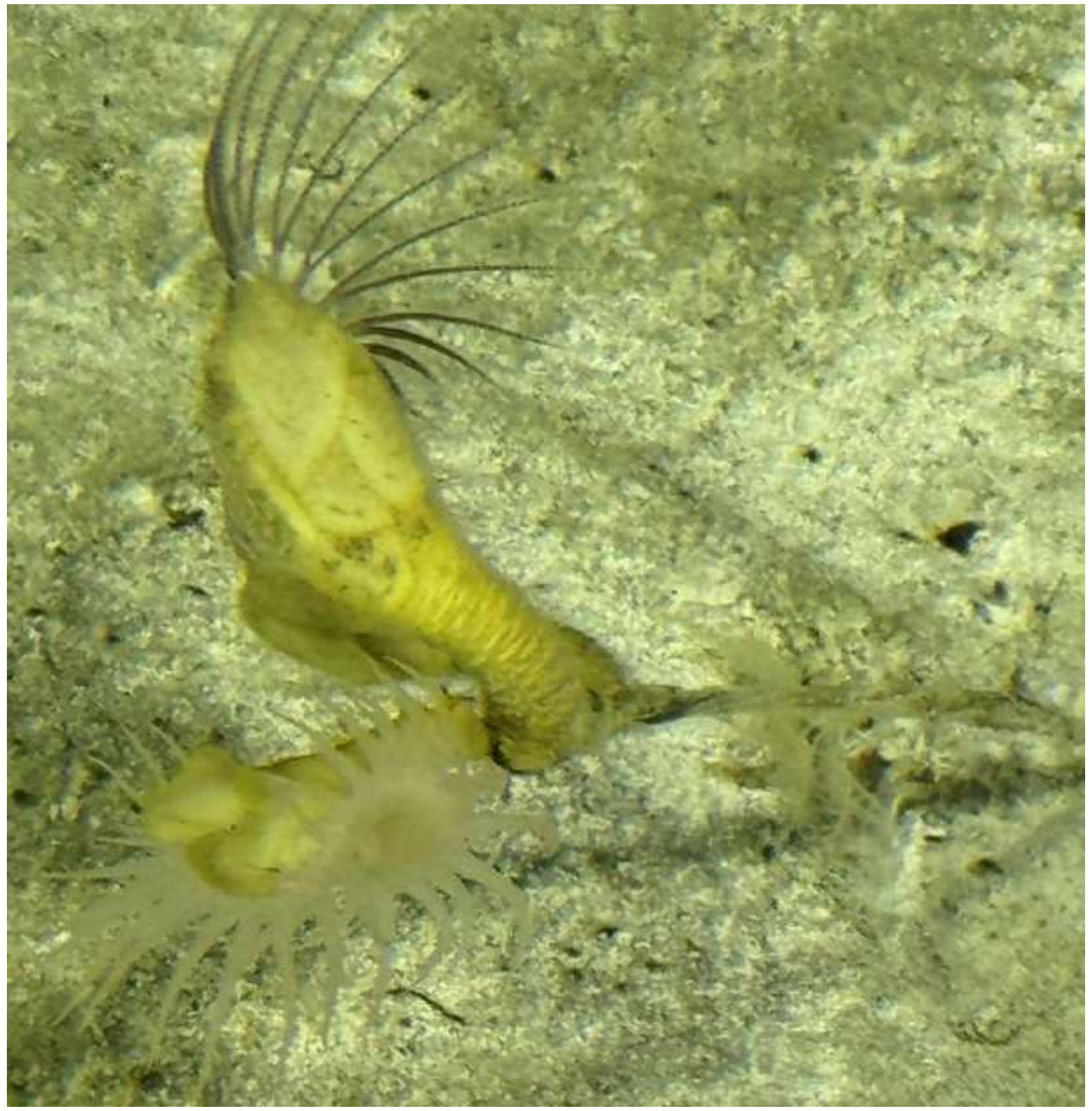
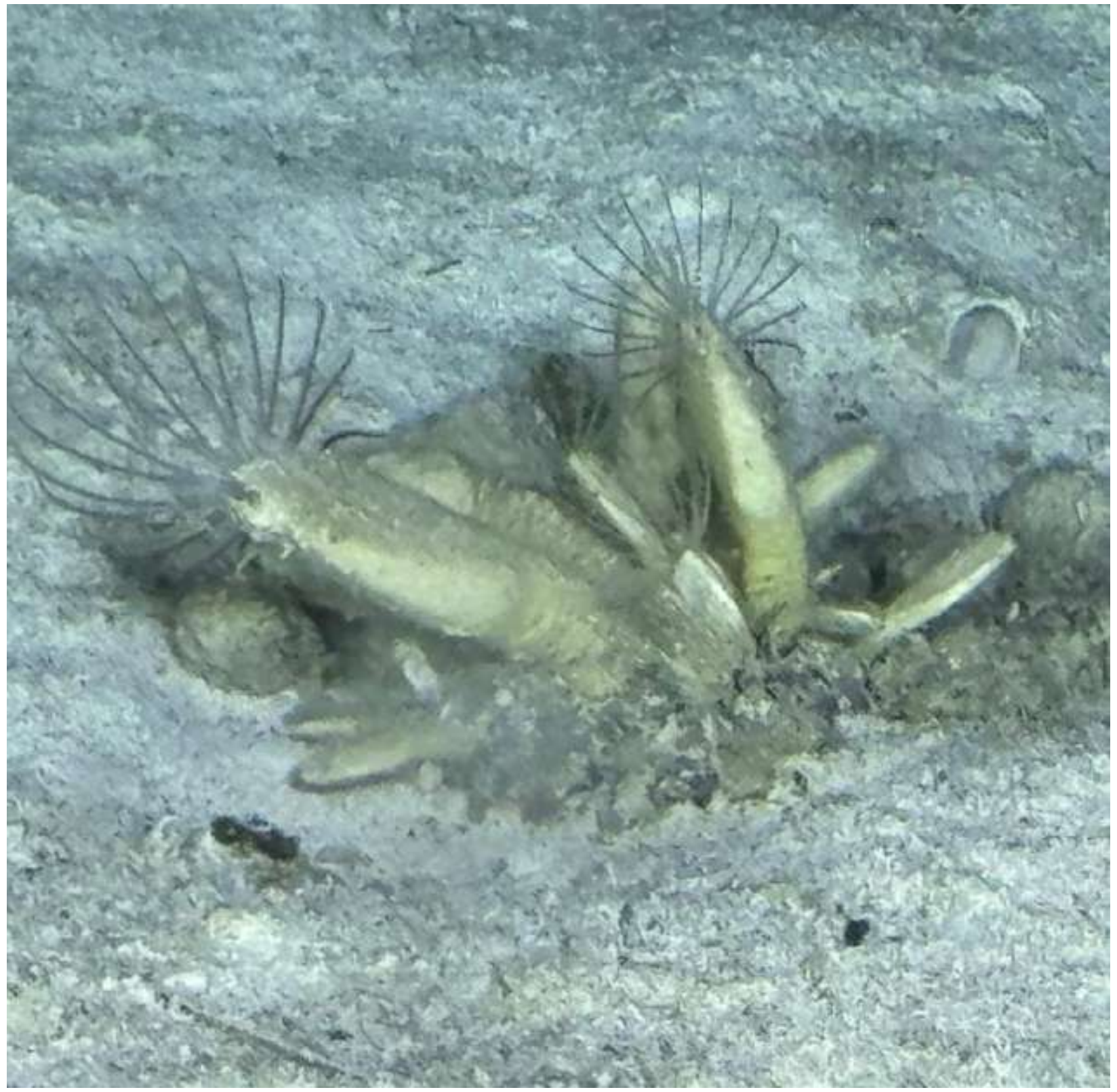

Pale, greenish-white body colour. Large, rounded, ovoid capitula with curved'stem'.

Behavioural Diagnostic: attaches to organic structures

Thecostraca; Scalpellomorpha spp.

NC2_BK5_4600

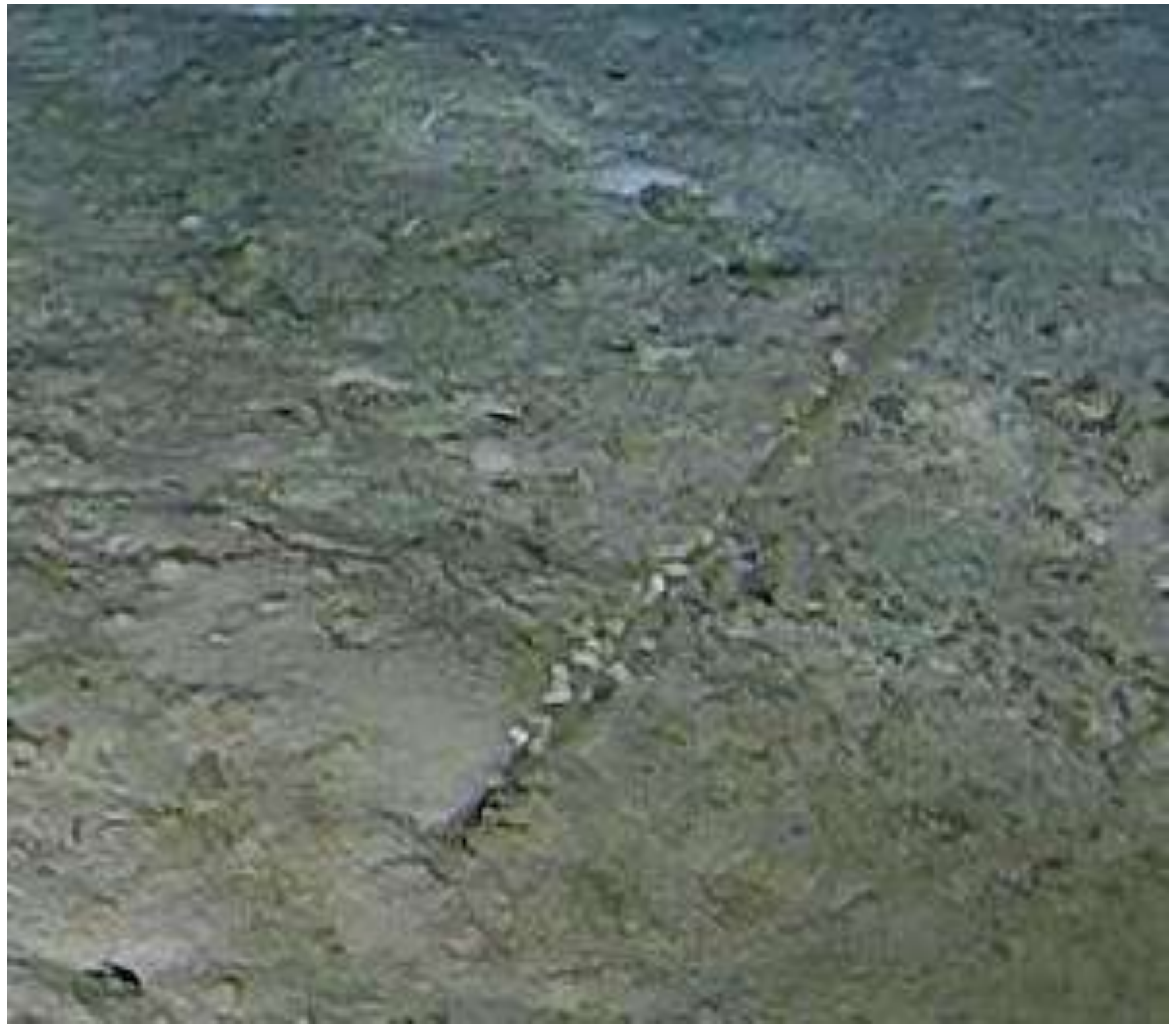

Pale, white body colour, multiple smaller individuals attached

Cnidaria

Anthozoa ord. indet. 1

NC3_BK1_8000

NC3_BK3_8000_IMG_3789

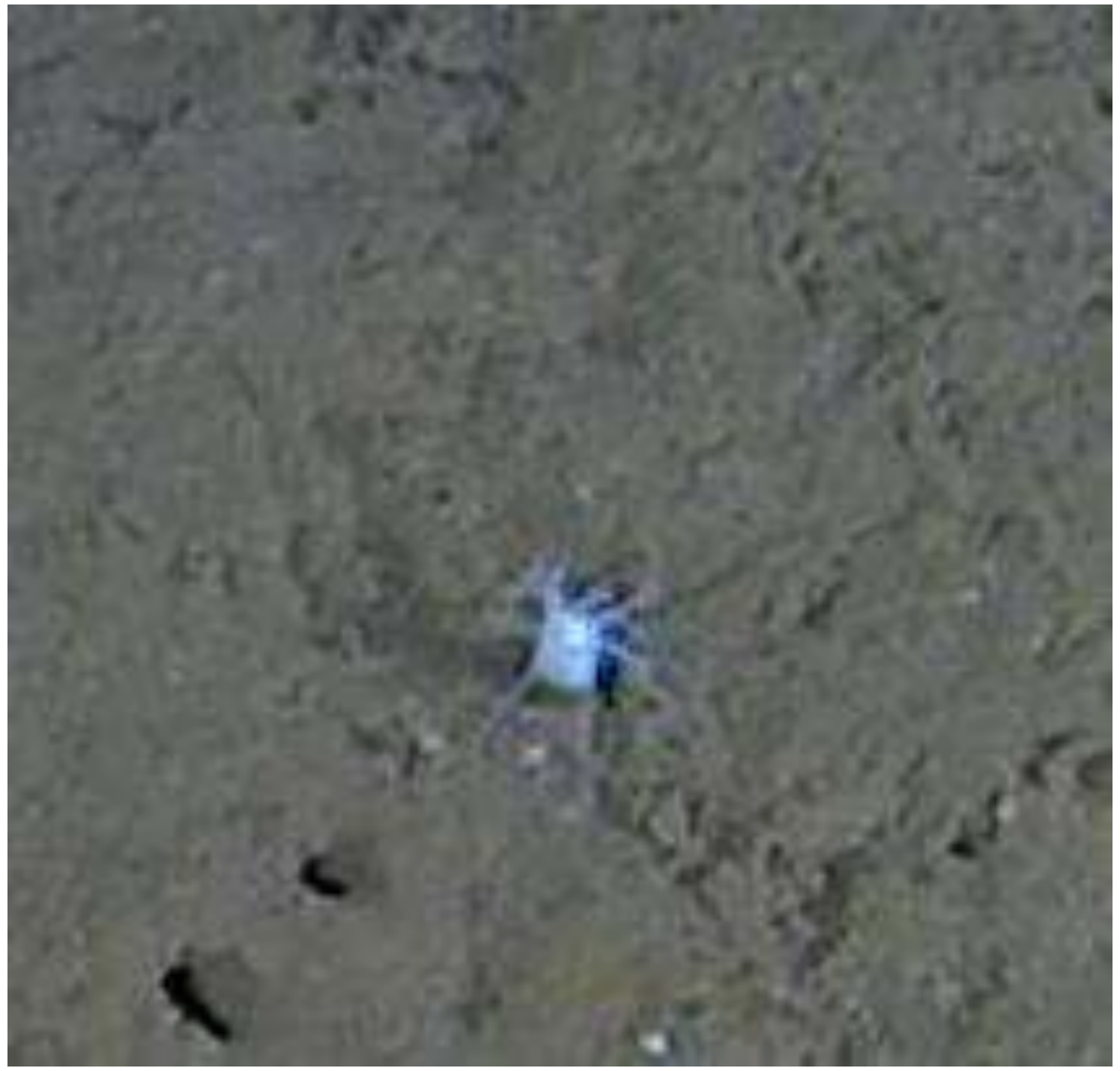

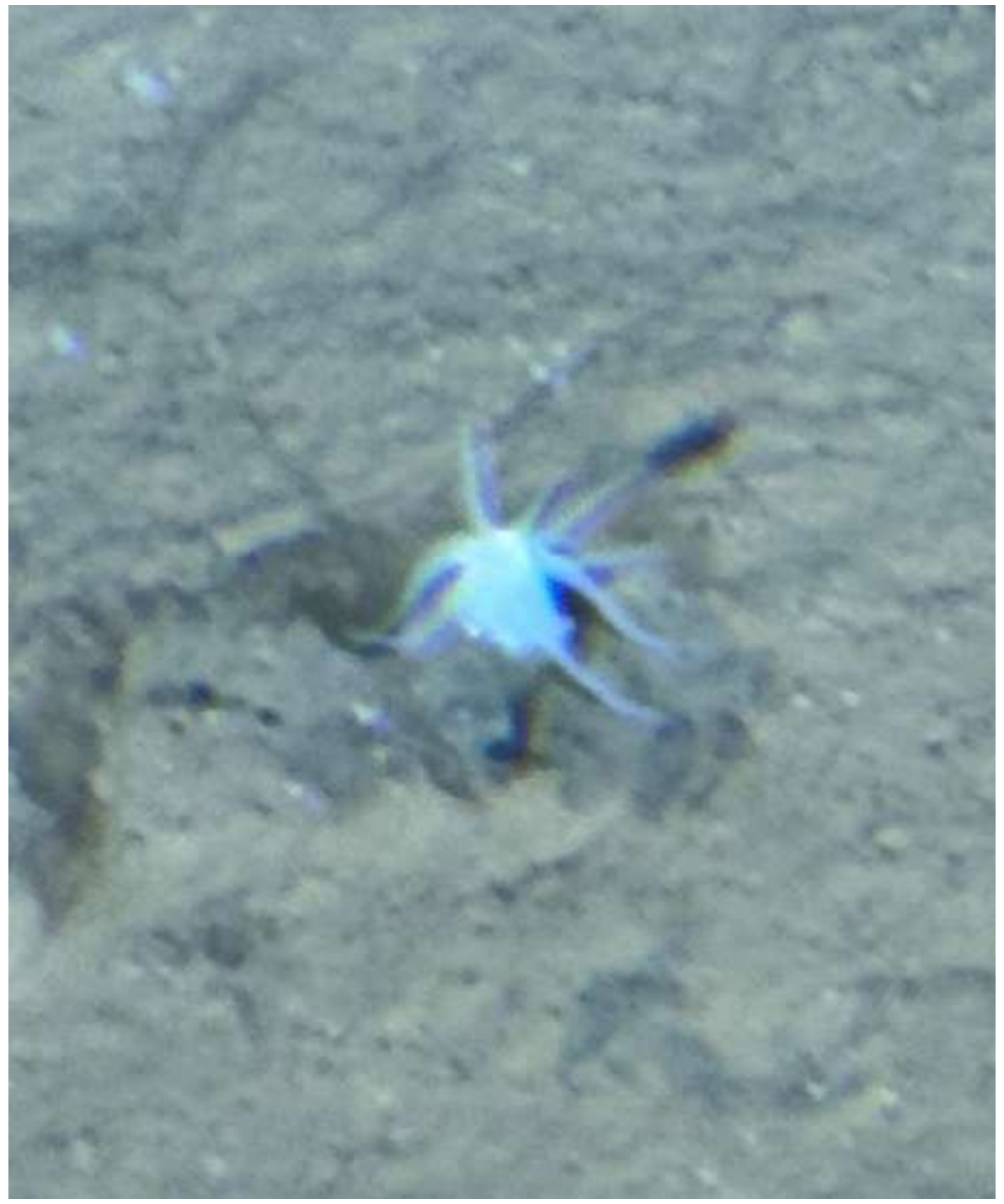

Found on soft substrate, fleshy body, long thin tentacles

Anthozoa ord. indet. 2

NC1_BK1_6200

NC3_BK2_6000

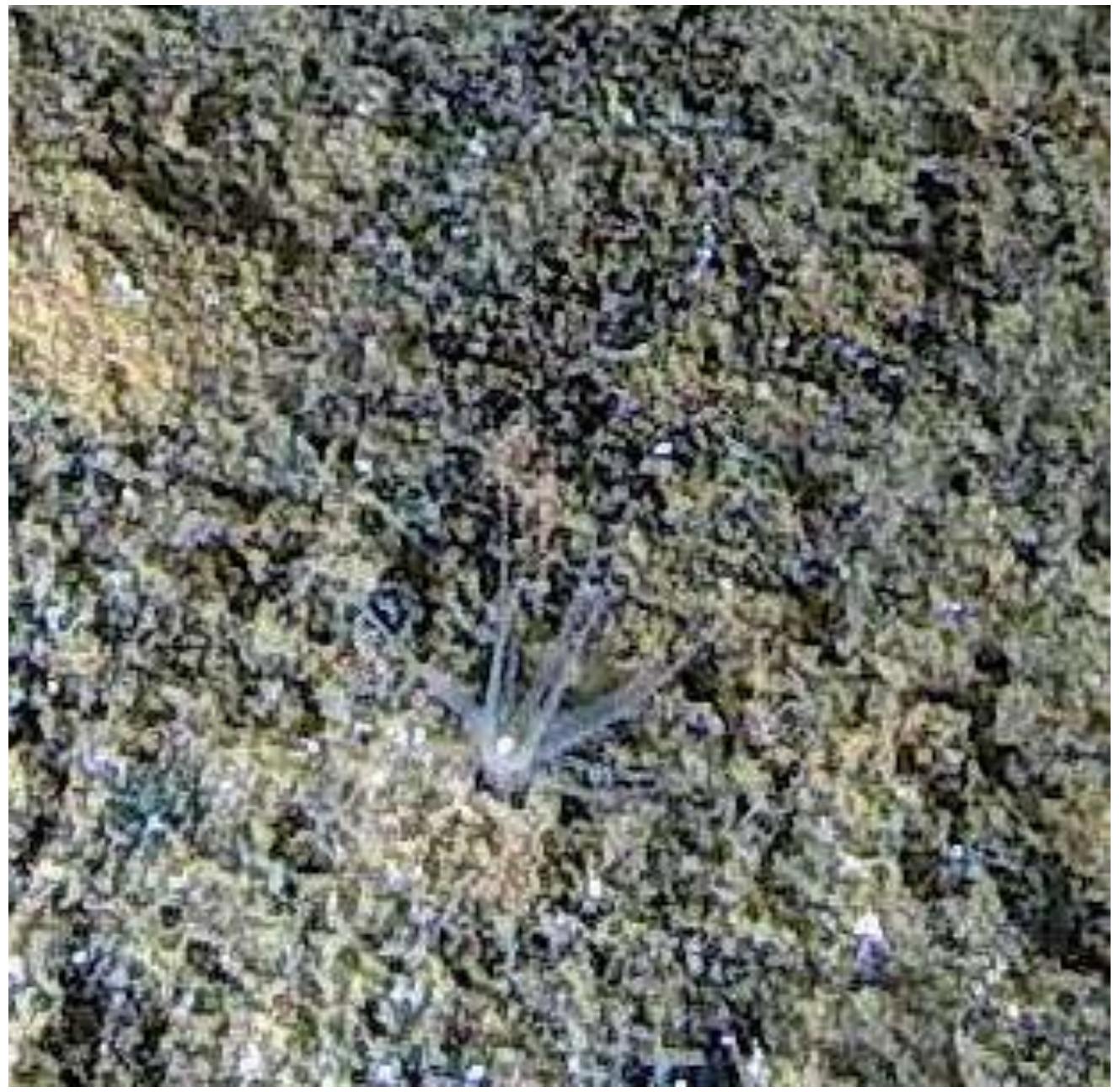

Found on harder substrate. White translucent, long tentacles

Hexacorallia; Actiniaria
Actiniaria fam. indet. 1

NC2_BK3_7400_IMG_8429

NC2_BK3_7400_IMG_9314

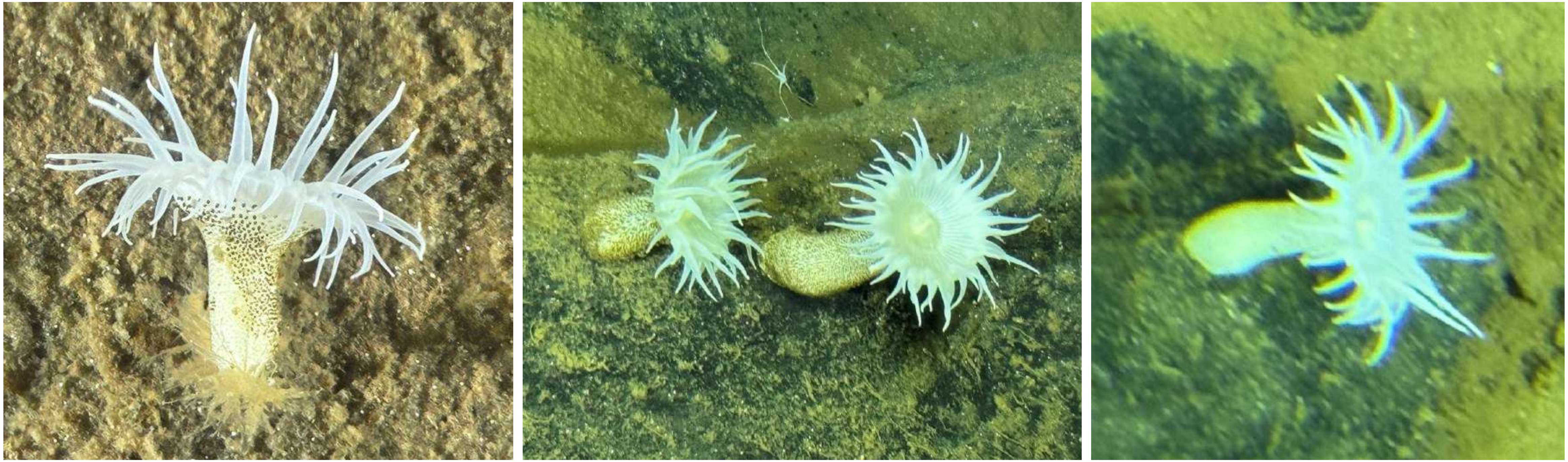

Found on both soft sediments and hard substrate. White-bluish medium tentacles; up to 48. White (?) column either smooth or with hair-like tubercles or verrucae; organization unclear. Mouth yellowish; siphonoglyphs and shape of the pharynx partially visible.

Hexacorallia; Actiniaria
Actiniaria fam. indet. 2

NC2_BK4_5300_IMG_9614

NC2_BK5_4600_IMG_9745

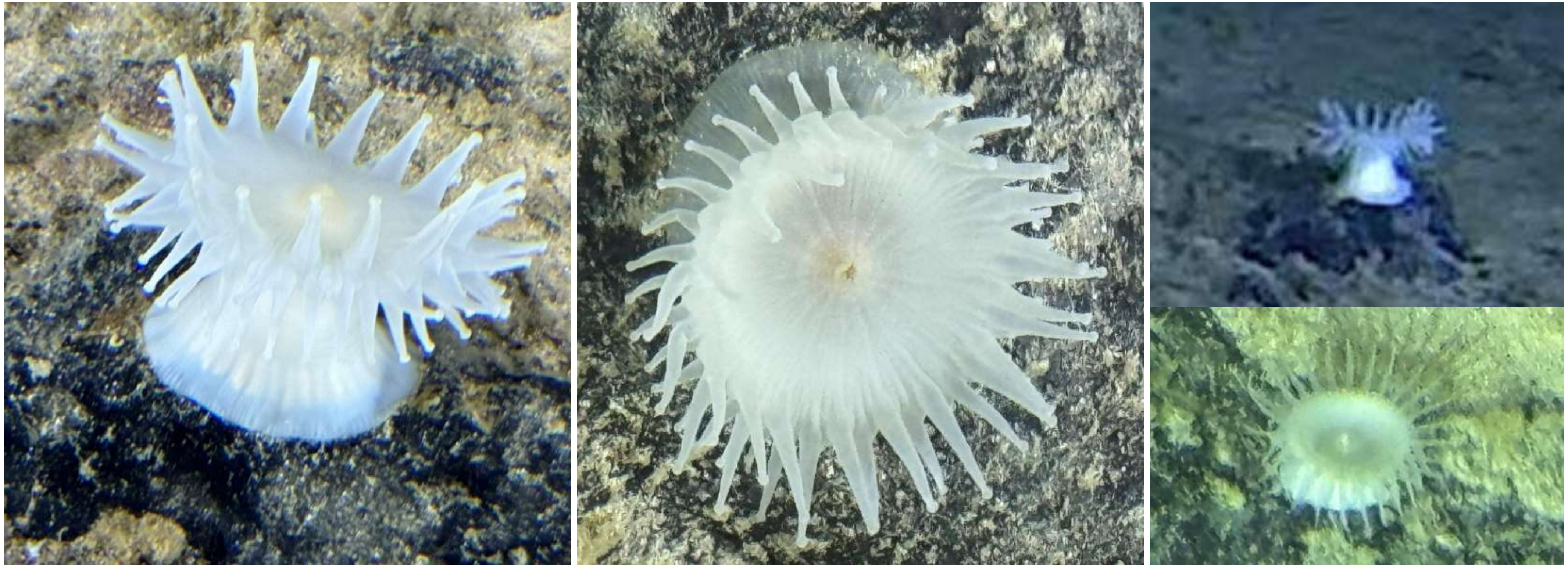

Found on hard substrate. White-bluish short tentacles; up to 60. White-bluish semitransparent column without secondary structures. Oral disc white; pharynx and siphonoglyphs not visible.

Hexacorallia; Actiniaria
Actiniaria fam. indet. 3

NC1_BK2_6700_IMG_4966

NC1_BK2_6700_IMG_5144

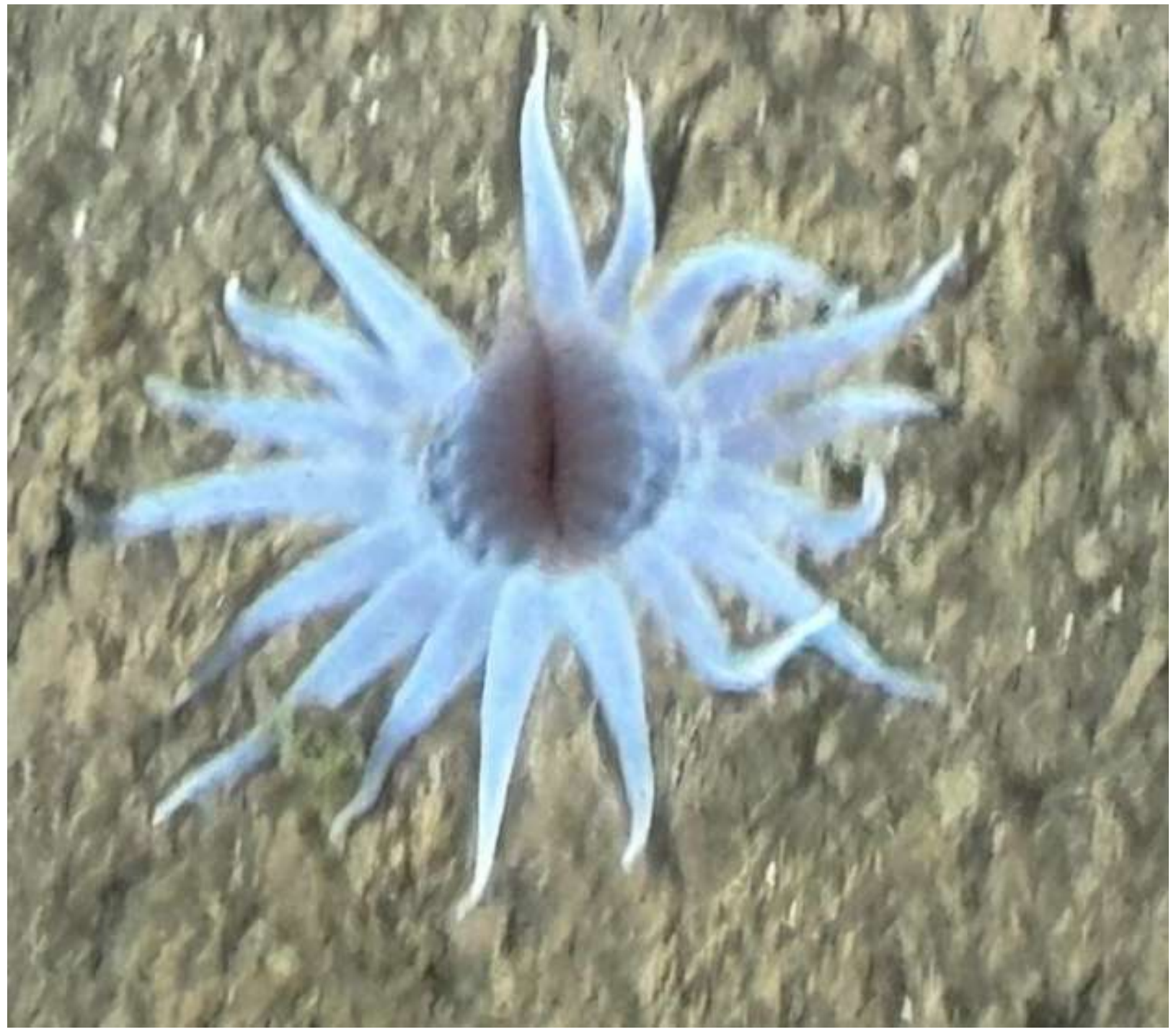

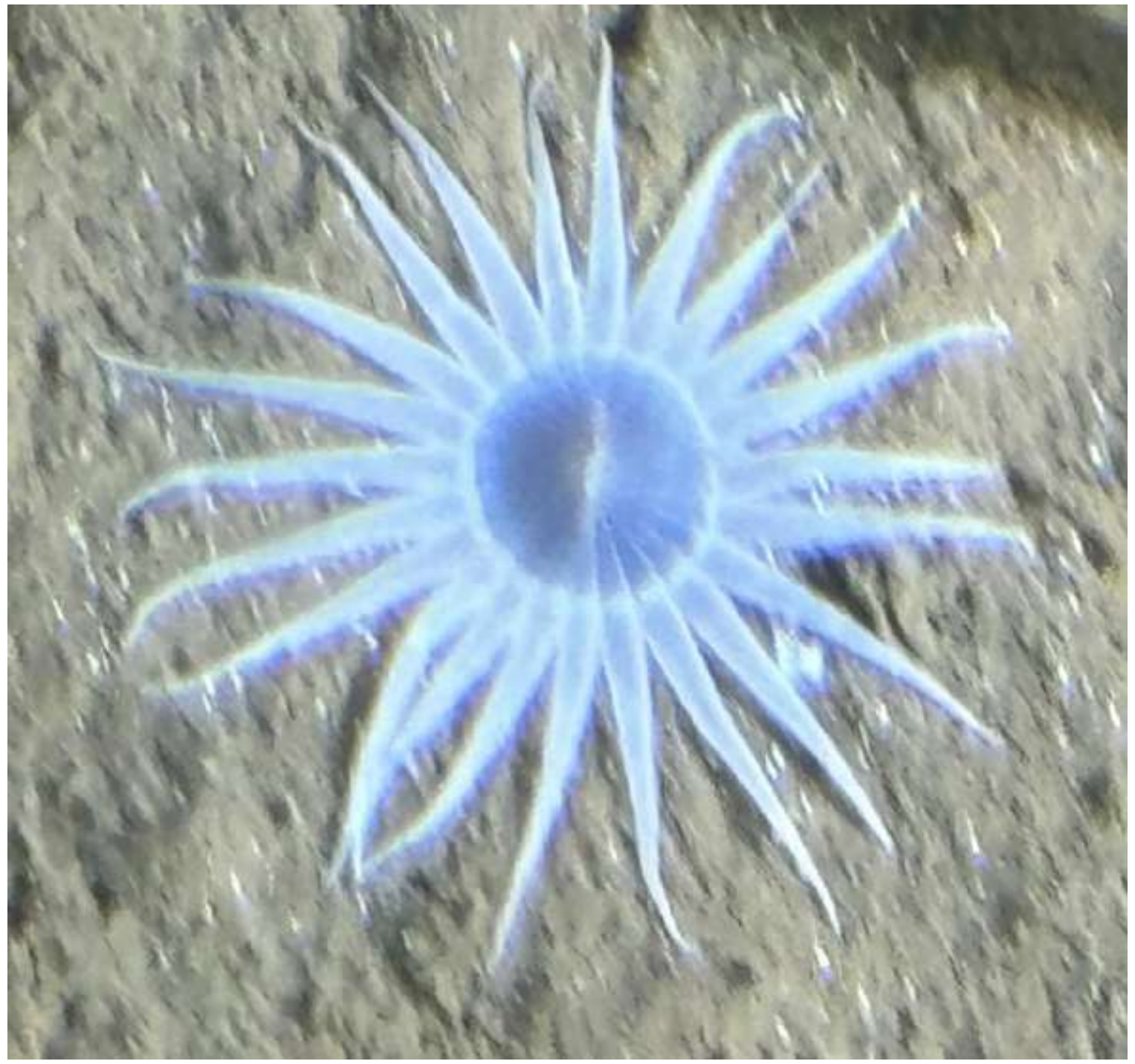

Found buried in fine sediment. White-bluish medium to long thick tentacles; up to 24. Column not visible. Blue oral disc; siphonoglyphs and shape of the pharynx visible. Presence of marginal structures suggested from the first picture?

Hexacorallia; Actiniaria
Actiniaria fam. indet. 4

NC2_BK1_3200_IMG_7605
NC2_BK1_3200_IMG_7608

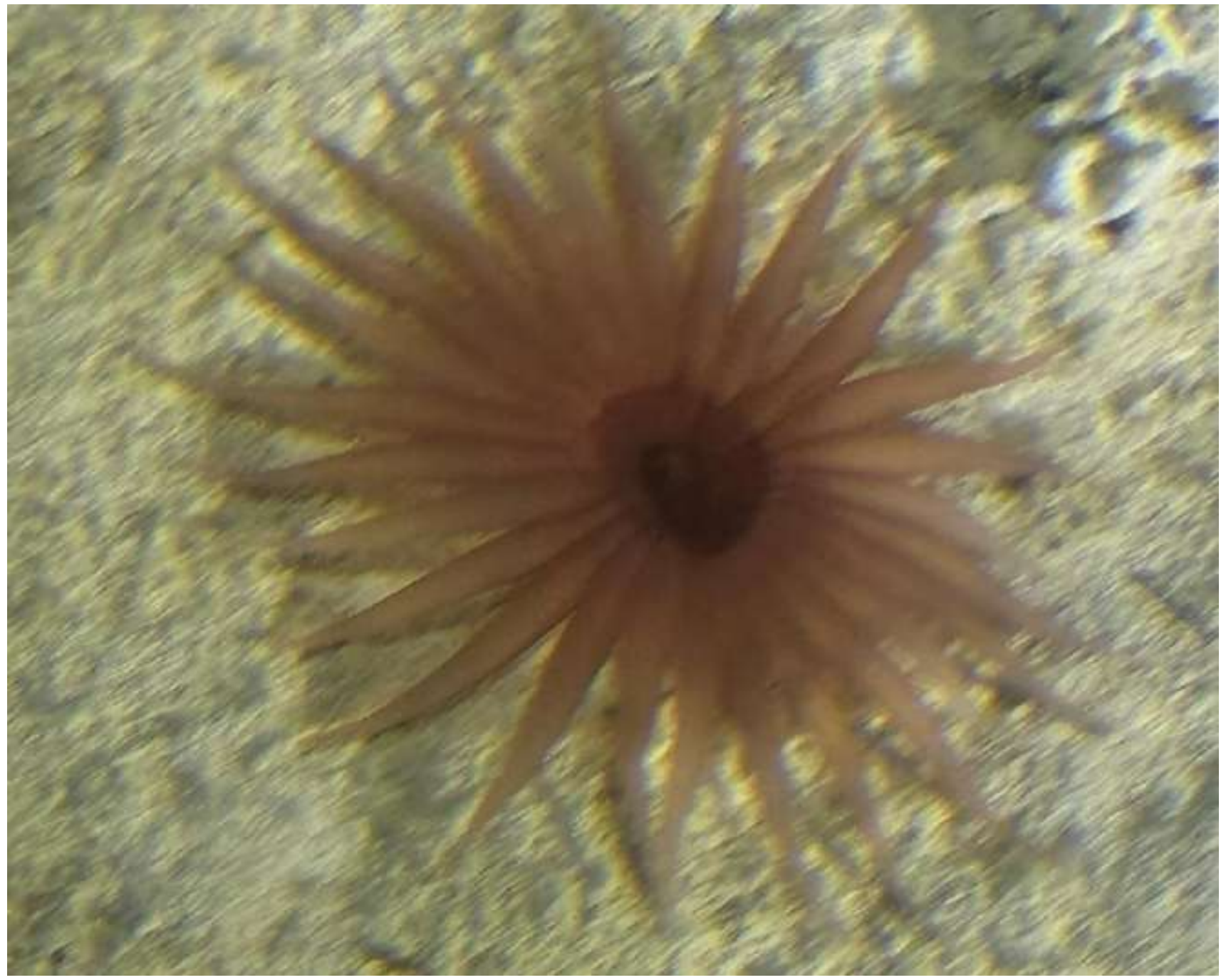

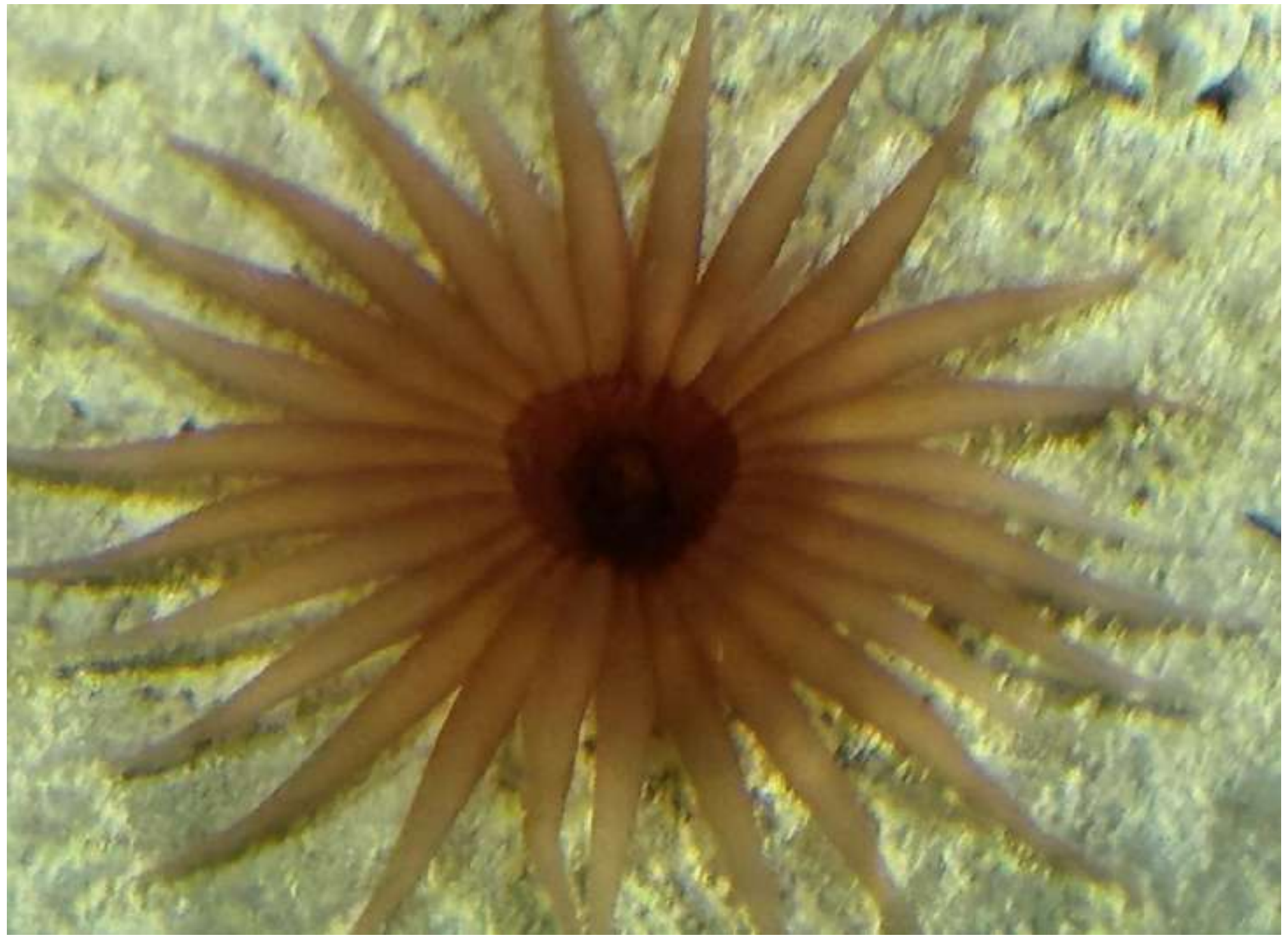

Found buried in fine sediment. Red-brownish medium to long thick tentacles; up to 30. Column not visible. Darker oral disc; shape of the pharynx not visible. Like Actiniaria fam. indet. 3; probably same genus or family.

Hexacorallia; Actiniaria
Actiniaria fam. indet. 5

NC2_BK1_3200_IMG_6799

NC2_BK1_3200_IMG_7220

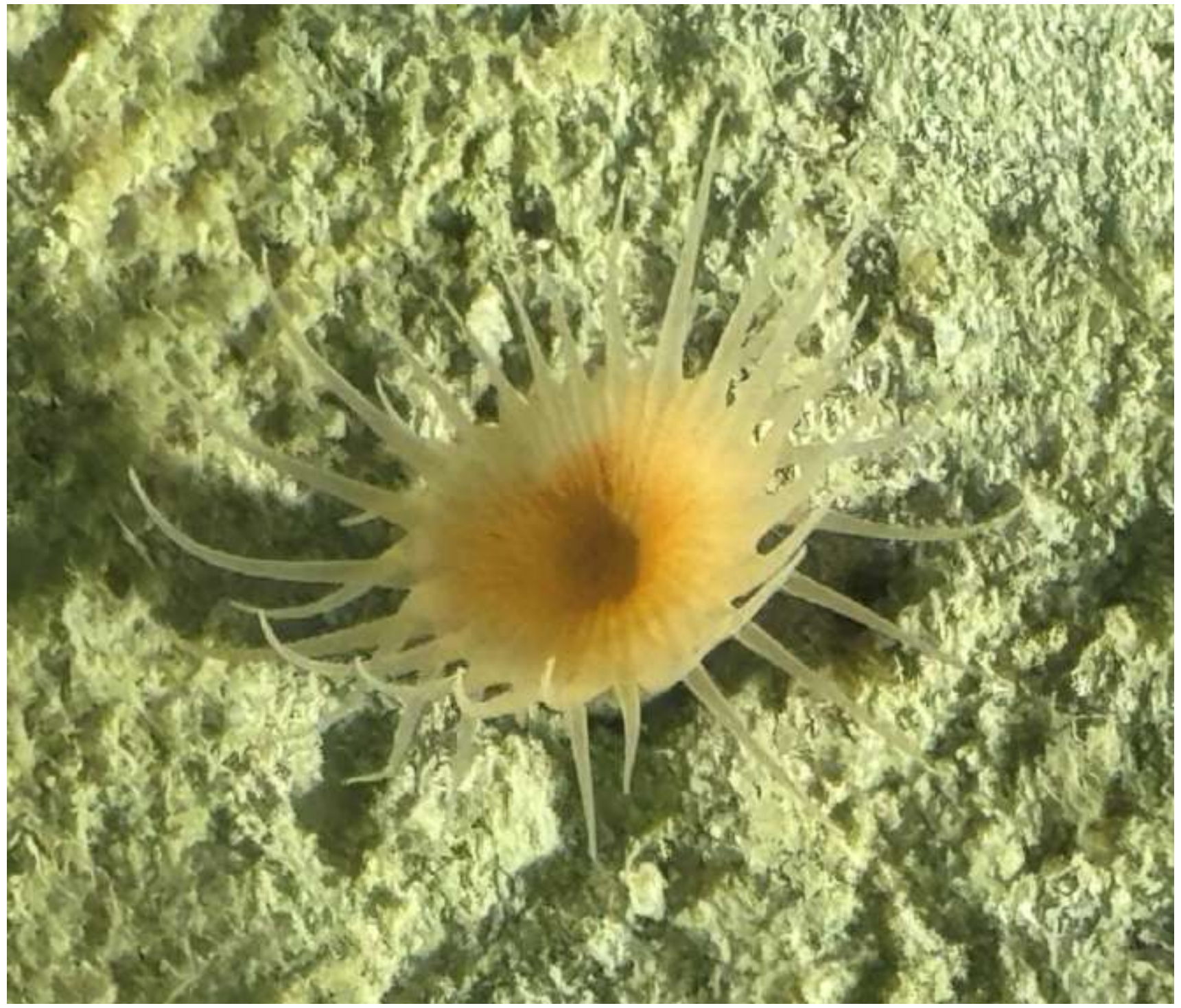

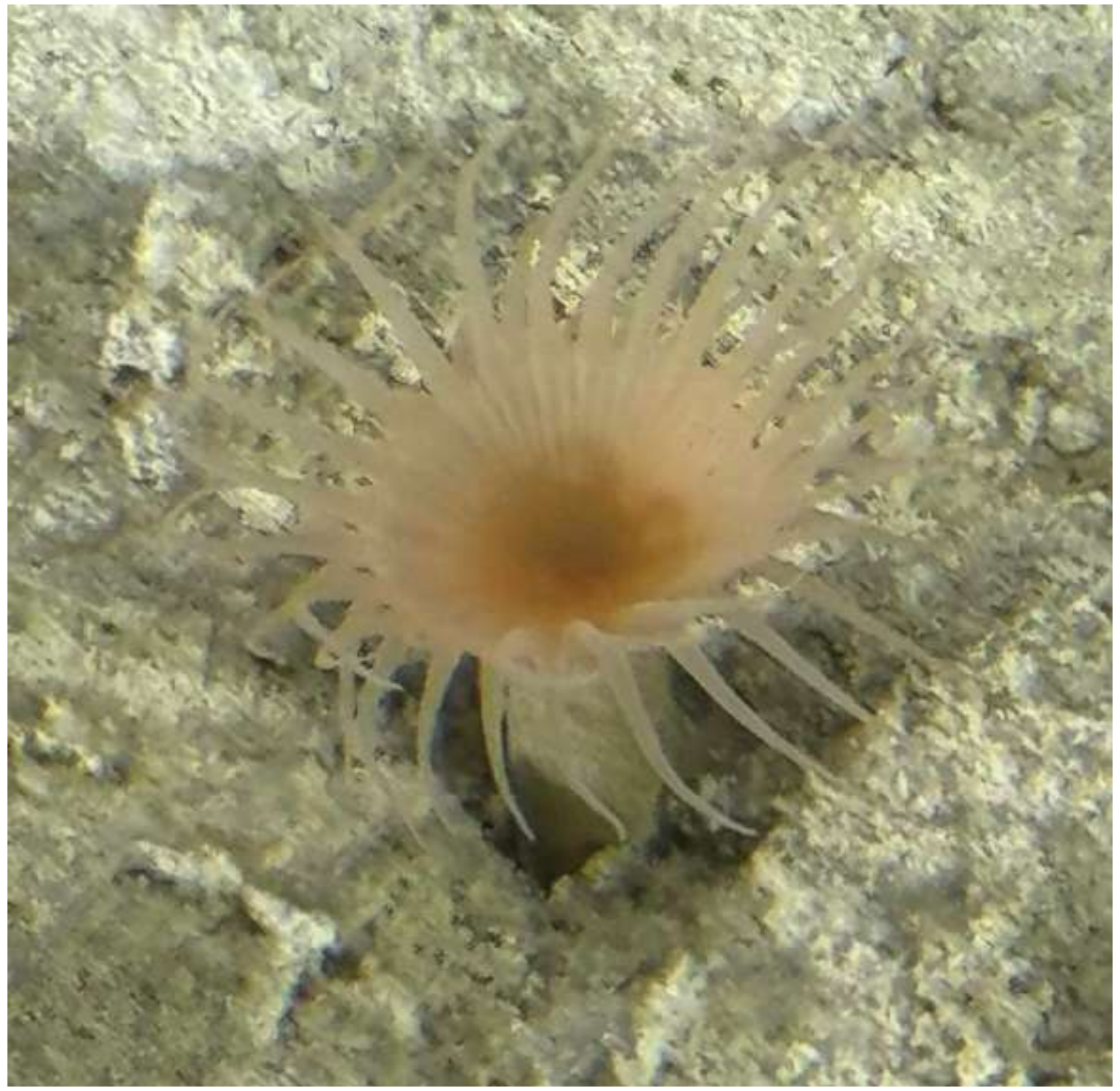

Found partially buried in fine sediment. Light orange medium to long tentacles; up to 50. Column sand-like; possibly covered in sediment. Orange oral disc; shape of the pharynx not visible. Edwardsiidae?

Hexacorallia; Actiniaria;
Actiniaria sp. indet. 6

NC3_BK2_6000_IMG_1549

NC3_BK2_6000_IMG_1552

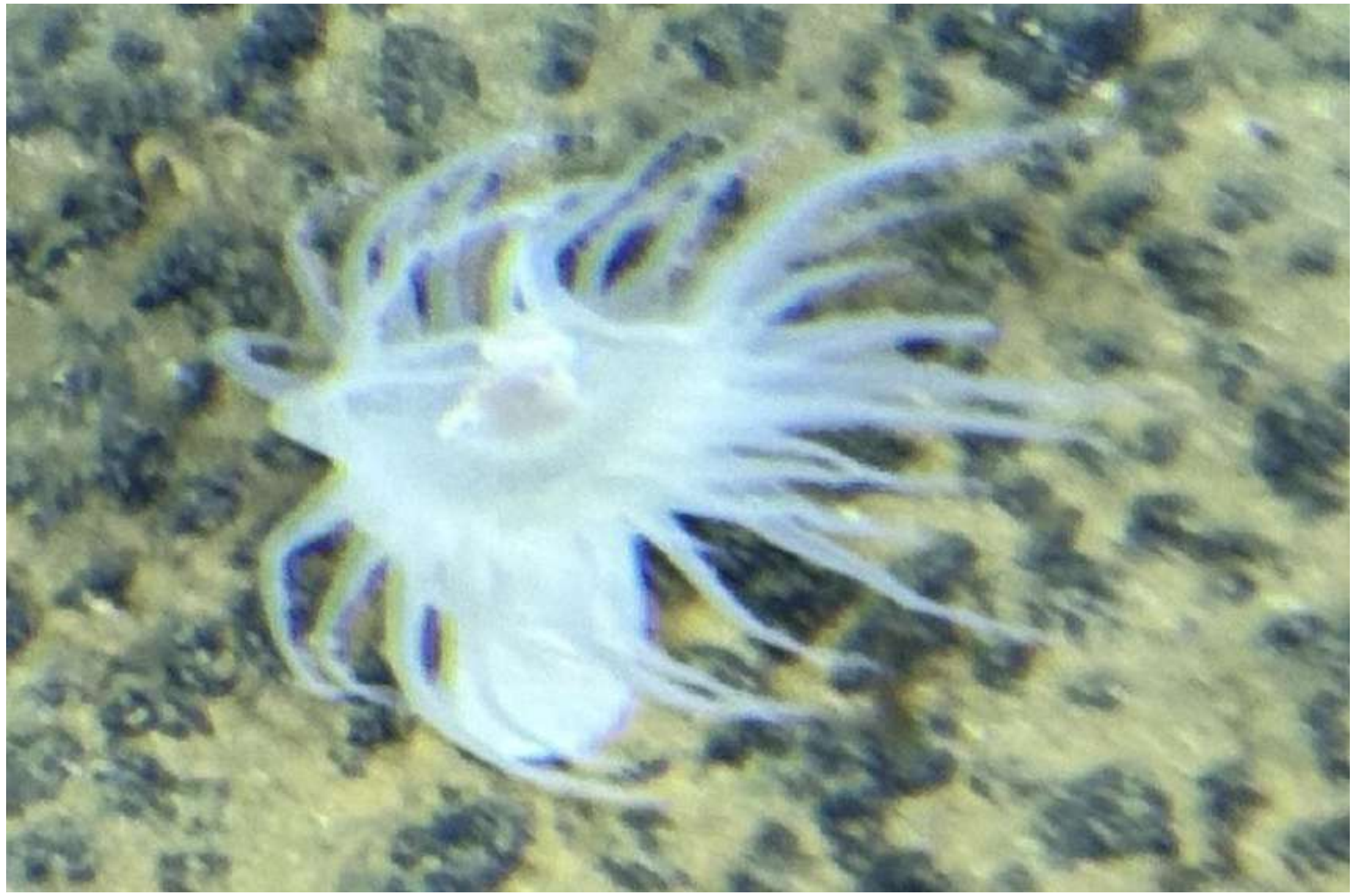

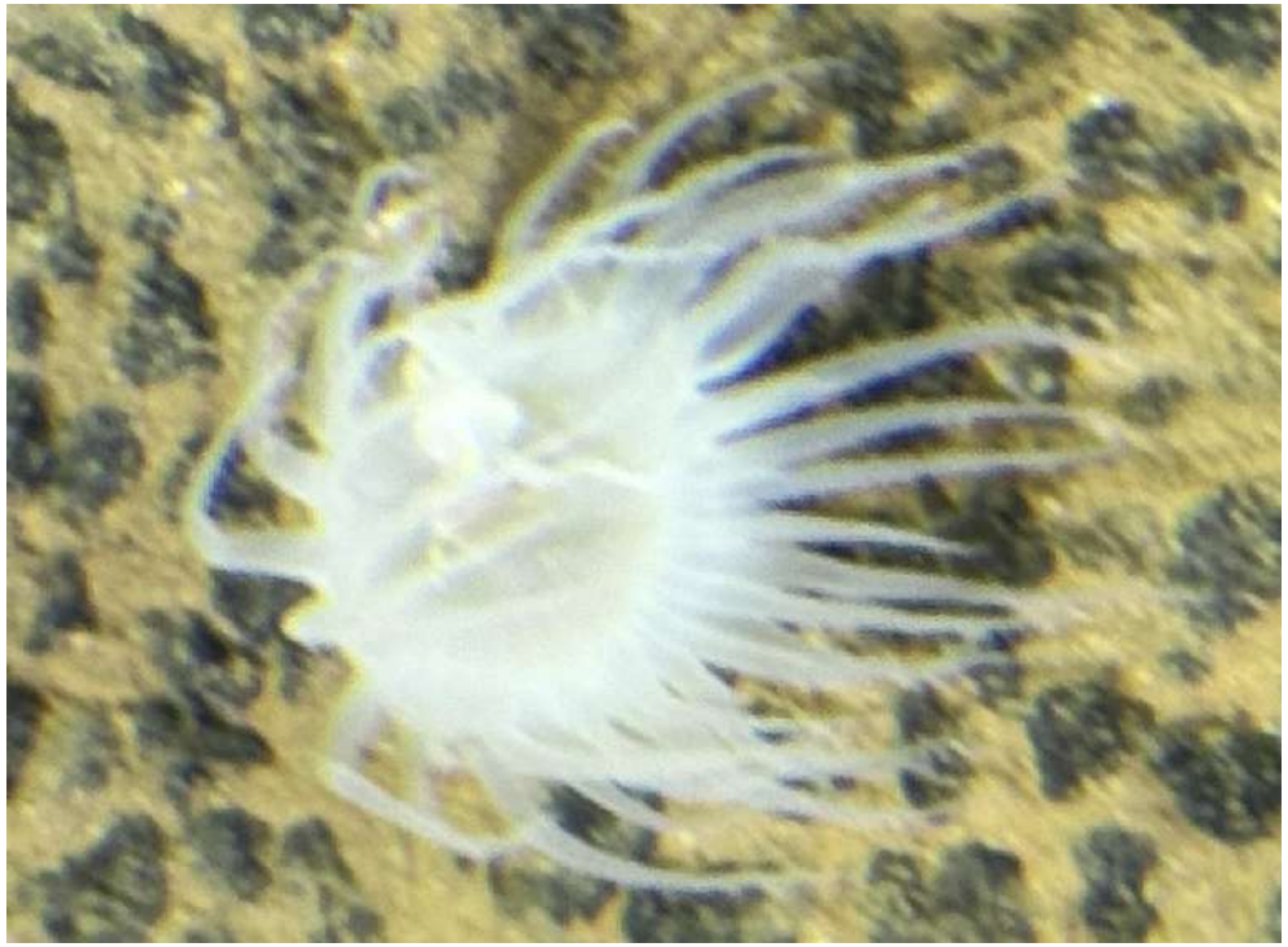

Found on nodules. White medium to long tentacles; possibly up to 60/70. Column white and smooth. White oral disc; borders of the mouth vertically developed; shape of the pharynx visible. Could be Actinernus.

Hexacorallia; Actiniaria
Actiniaria fam. indet. 7

NC4_BK4_4700_IMG_6059
NC2_BK4_5300_NOR_0077

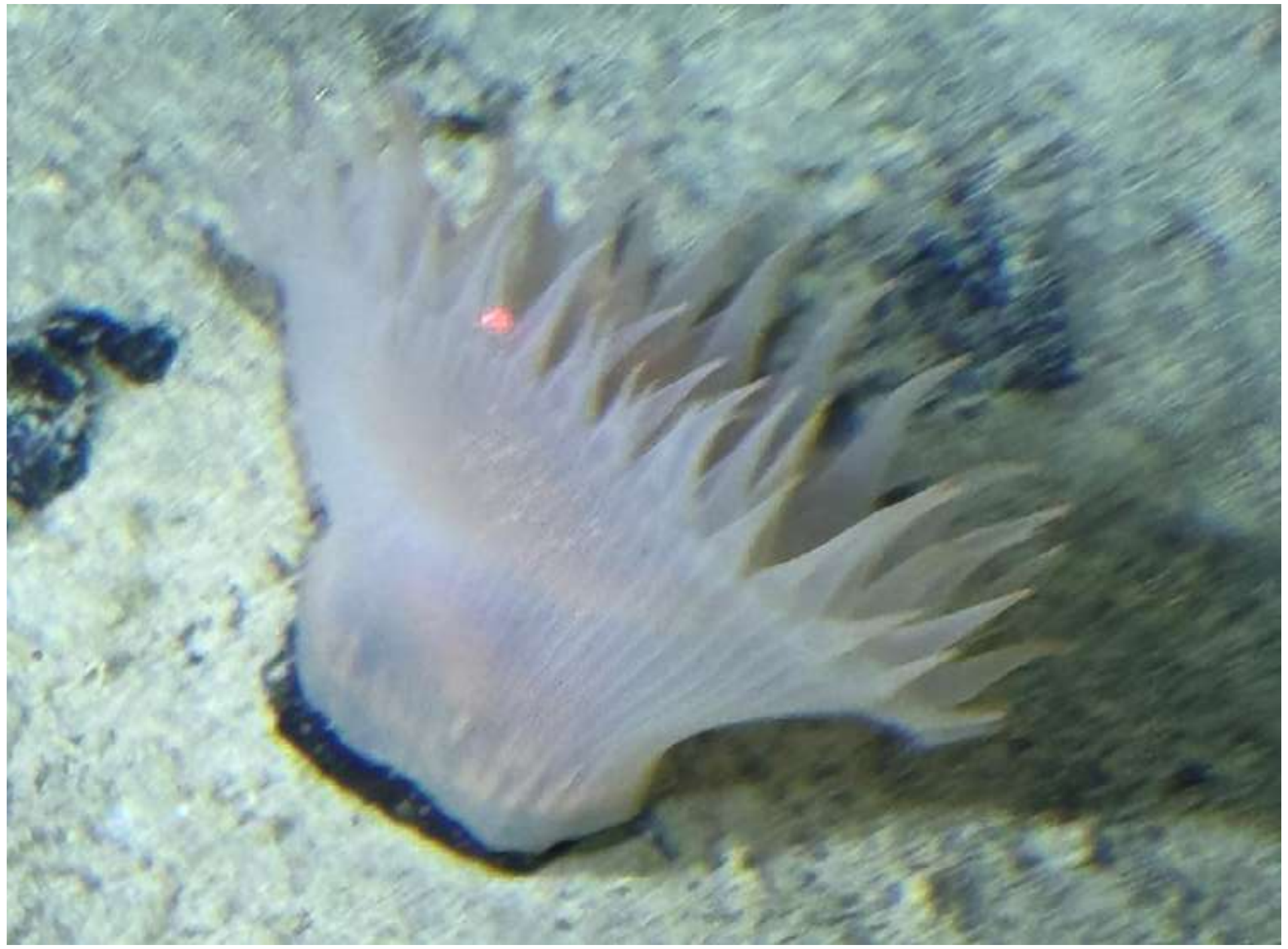

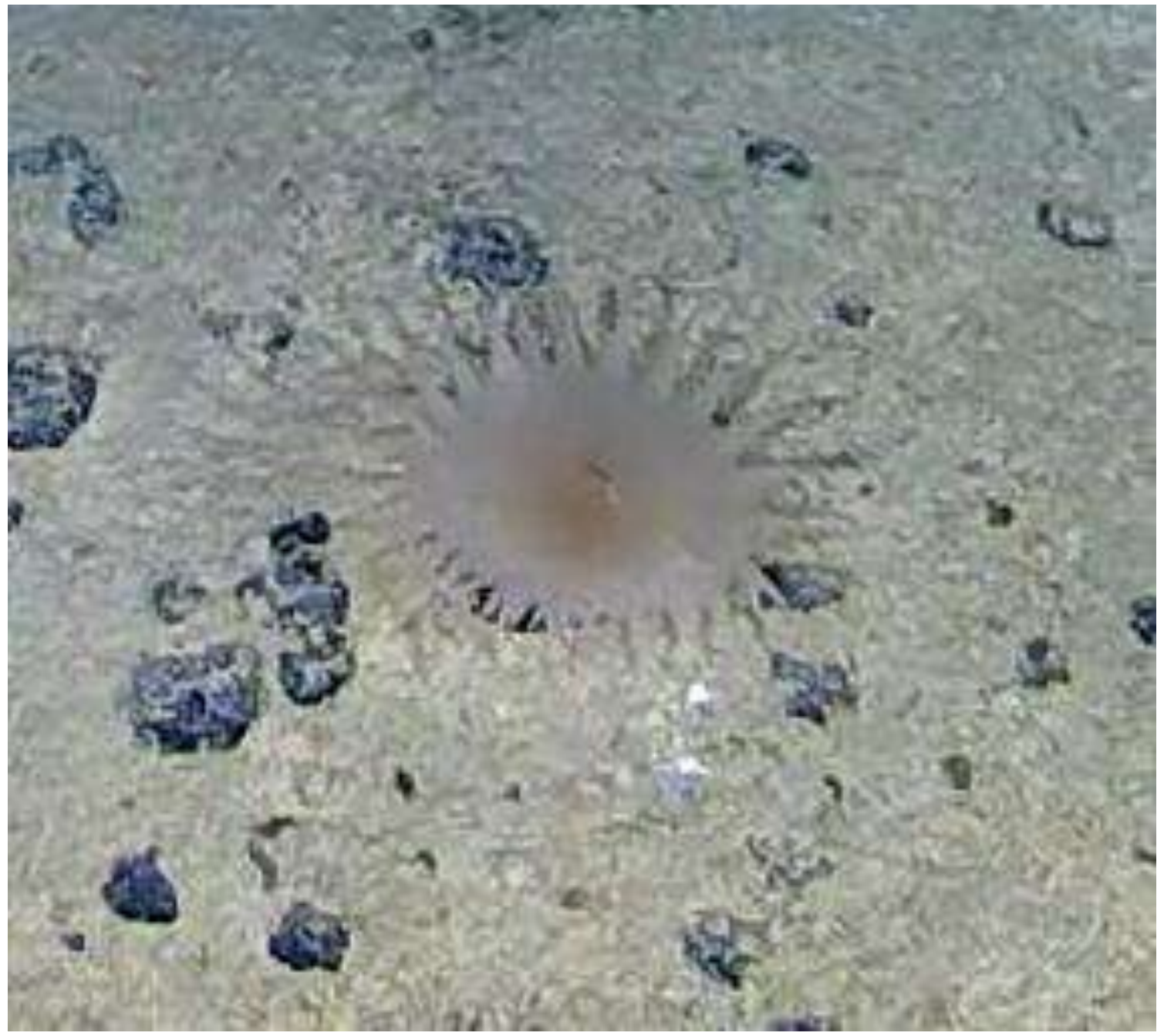

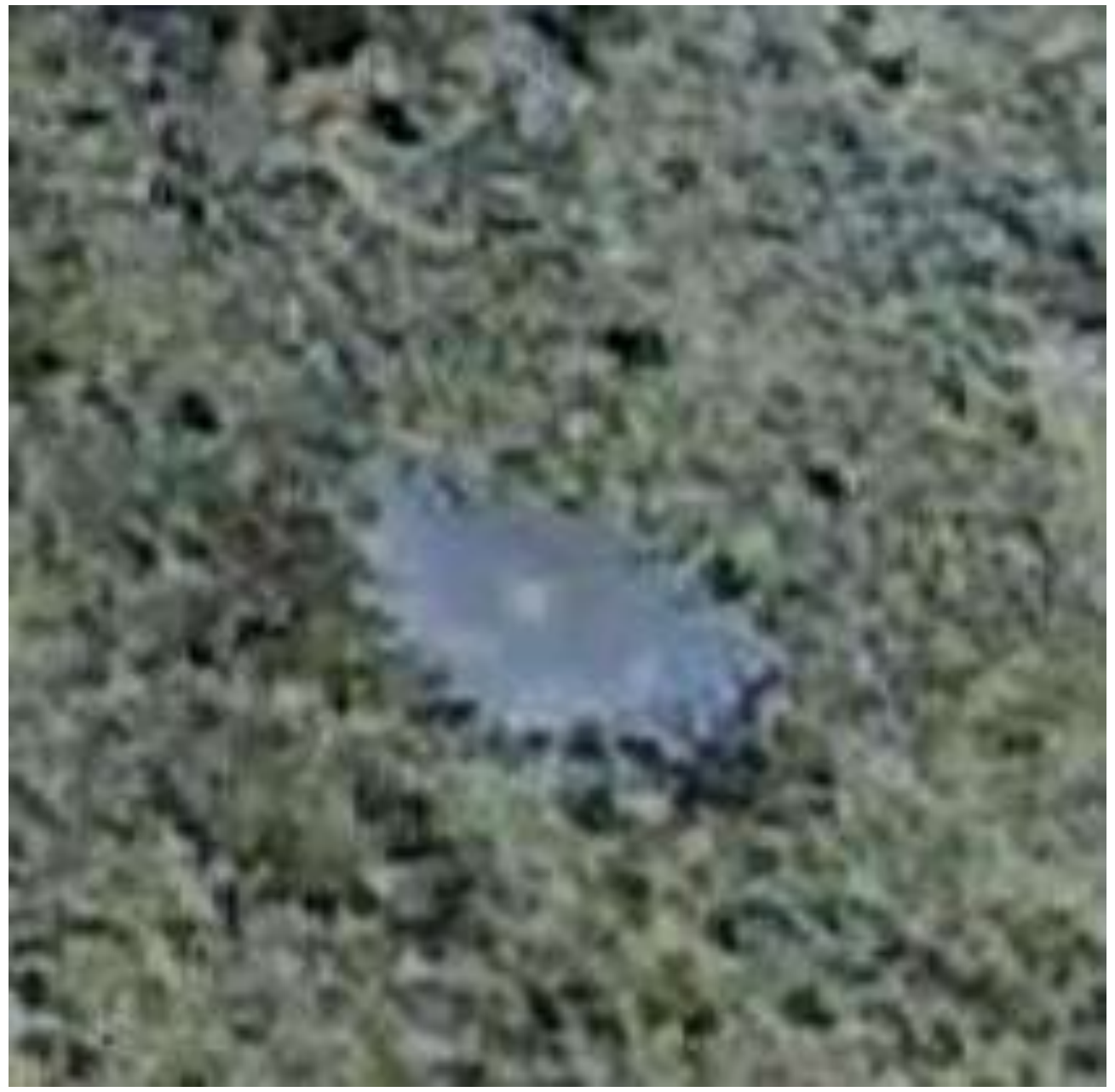

Found on nodules. Translucent short tentacles; possibly up to 60 (uncertain). Translucent column; with mesenteries visible.. White-Pinkish colour.

NC4_BK4_4700_IMG_6033
NC2_BK4_5300_NOR_0090

Hexacorallia; Actiniaria
Actiniaria fam. indet. 8

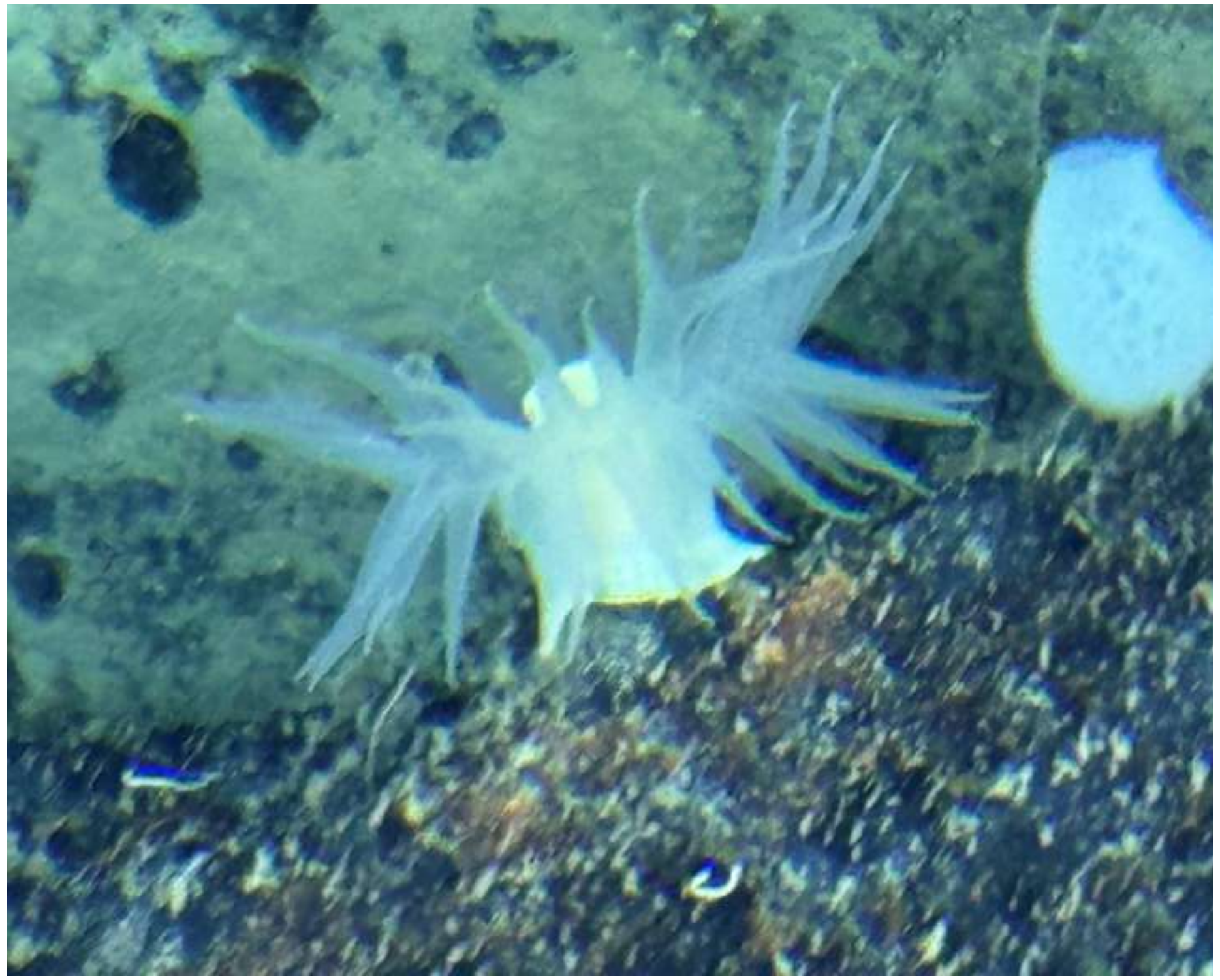

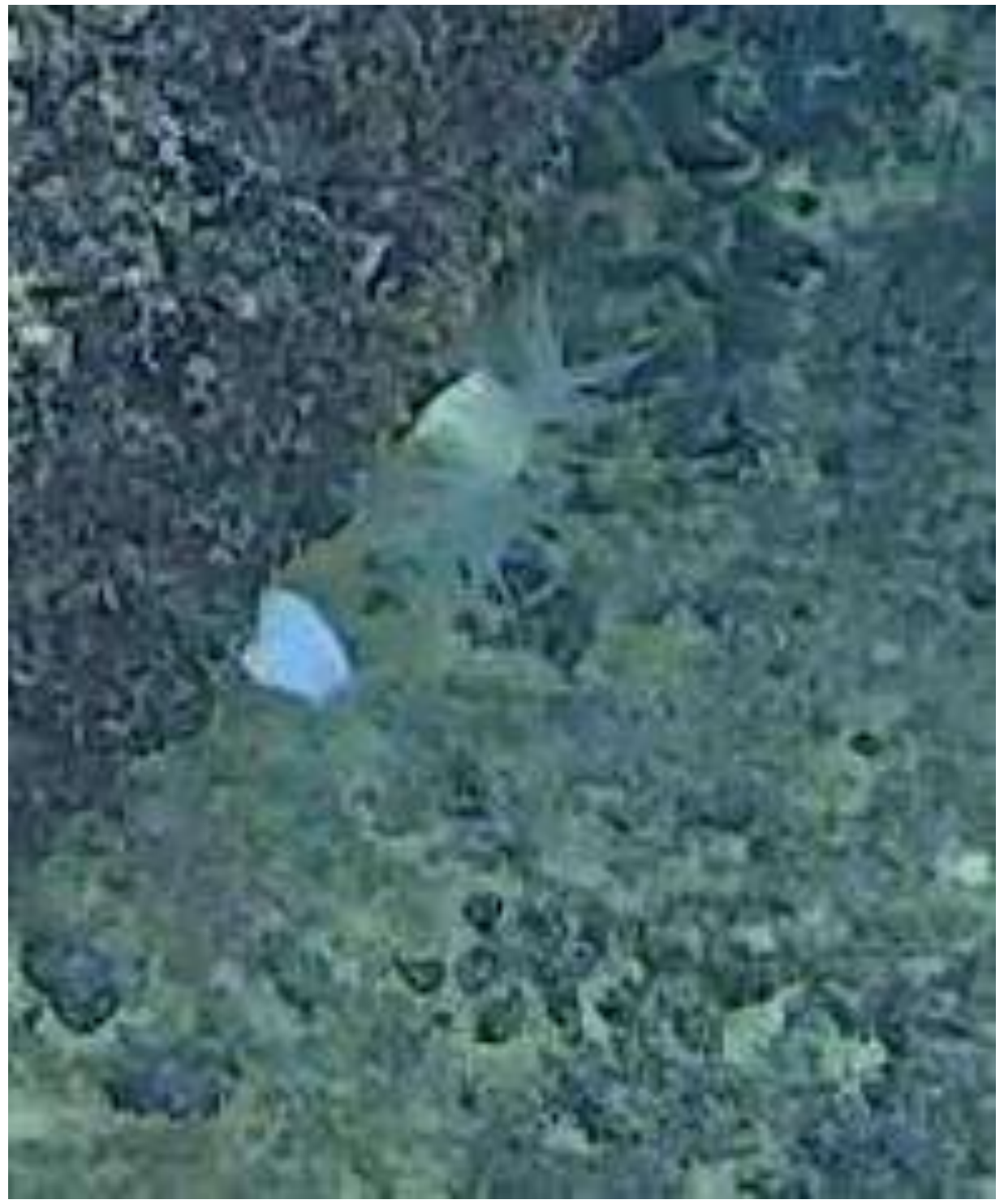

Found on hard substrate. Translucent short tentacles; possibly up to 40 (uncertain); longer than the height of the column. White column; with mesenteries partially visible. Oral disc not visible; but mouth vertically developed.

Hexacorallia; Actiniaria
Actiniaria fam. indet. 9

NC1_BK1_6200

NC3_BK2_6000

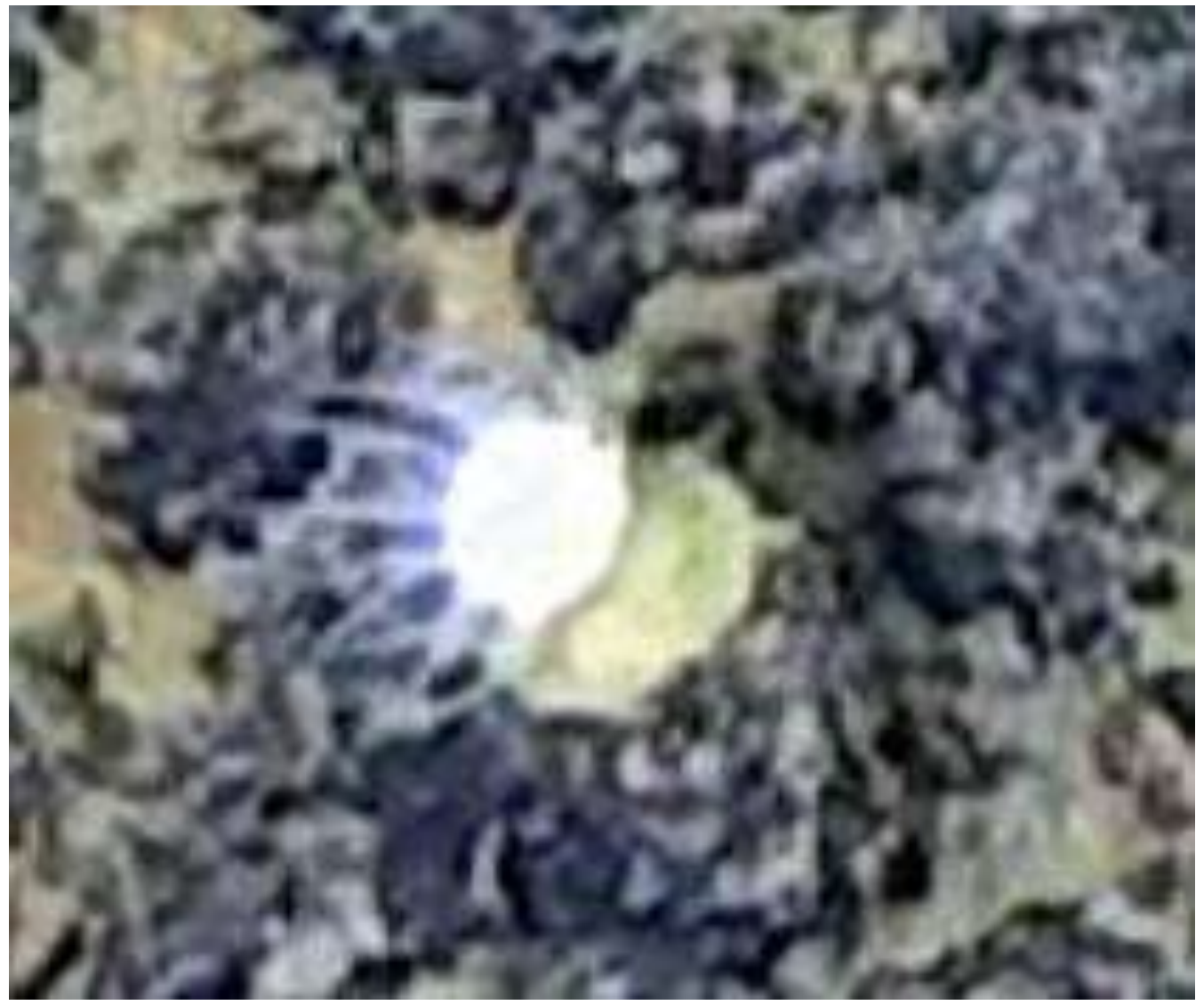

Stubby wide short base with distinct attachment to nodules, short length tentacles, one whorl, flat disc like sp. indet. 13, but distinct base.

Hexacorallia; Actiniaria
Actiniaria fam. indet. 10

NC3_BK2_6000_IMG_1360

NC3_BK2_6000_IMG_1364

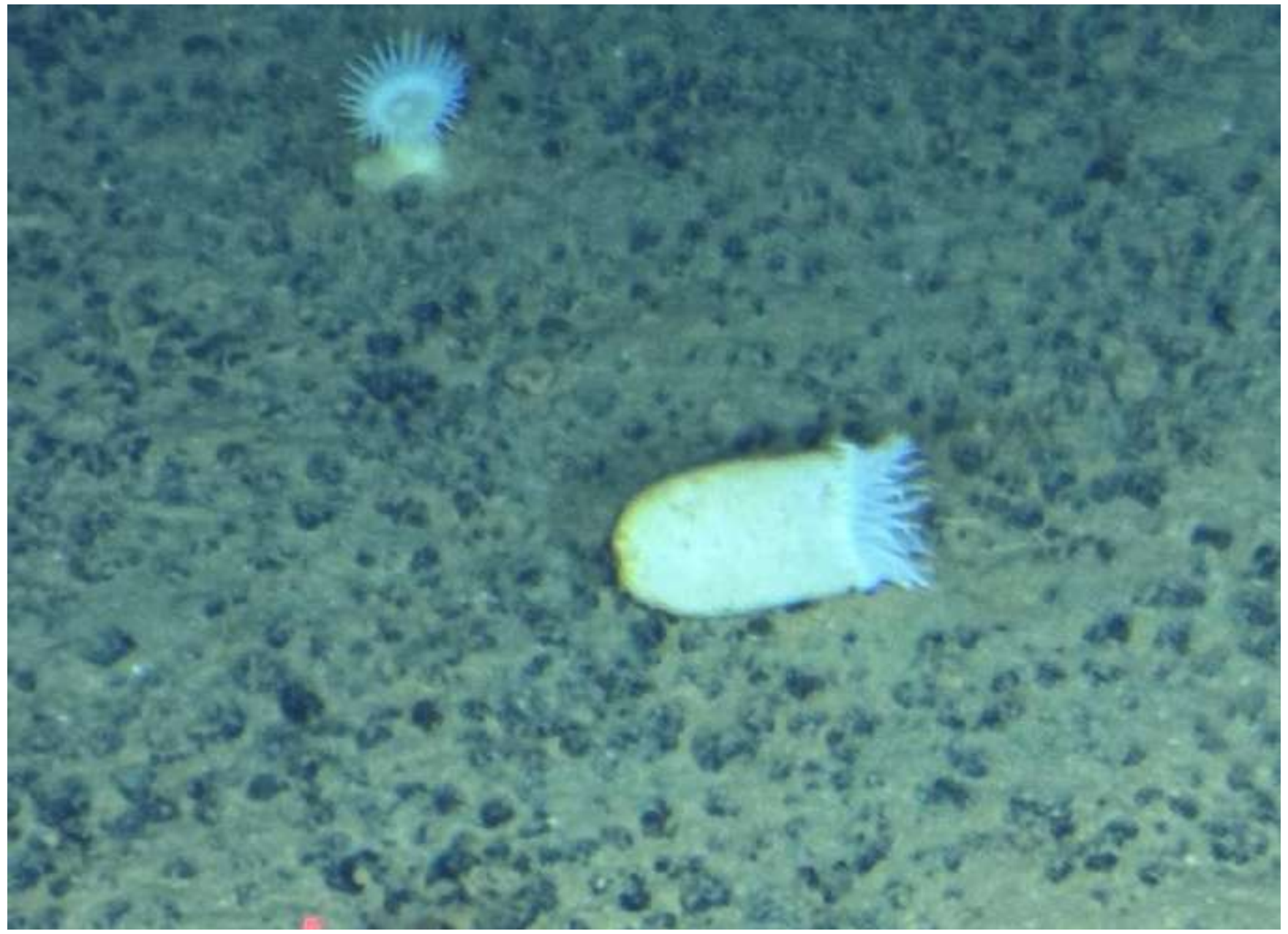

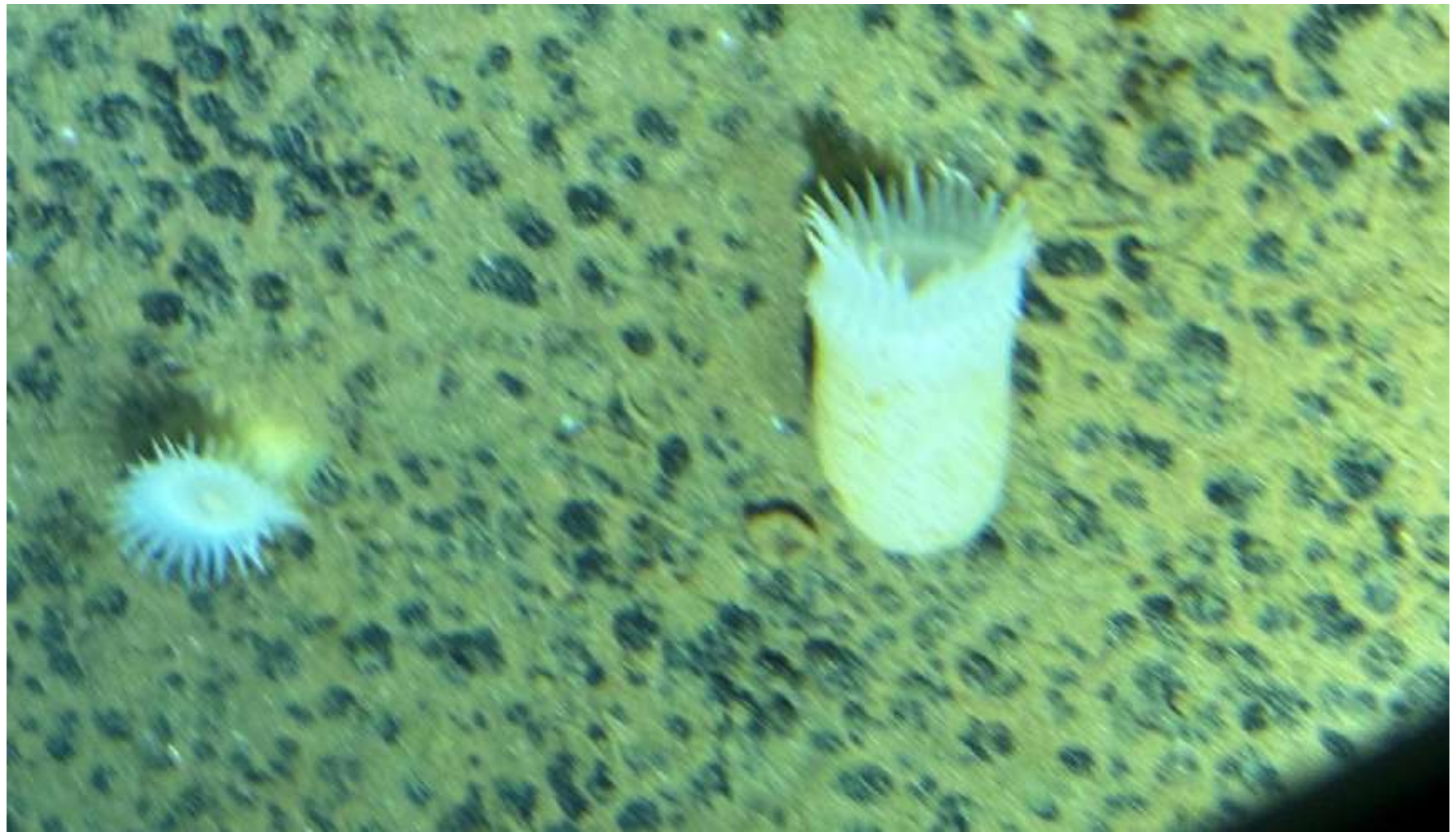

Found on nodules. White-bluish medium tentacles; up to 48. White column with verrucae or sediment material; organization unclear. Oral disc same colour as tentacles. Mouth yellowish. Can be found in a normal shape; attached to the seafloor; or in a cigar shape; for locomotion (Marchiò et al.; 2025)

“Rolling anemone” Superfamily Actinostolidae

Hexacorallia; Actiniaria
Actiniaria fam. indet. 11

NC2_BK3_7400

NC3_BK1_8000

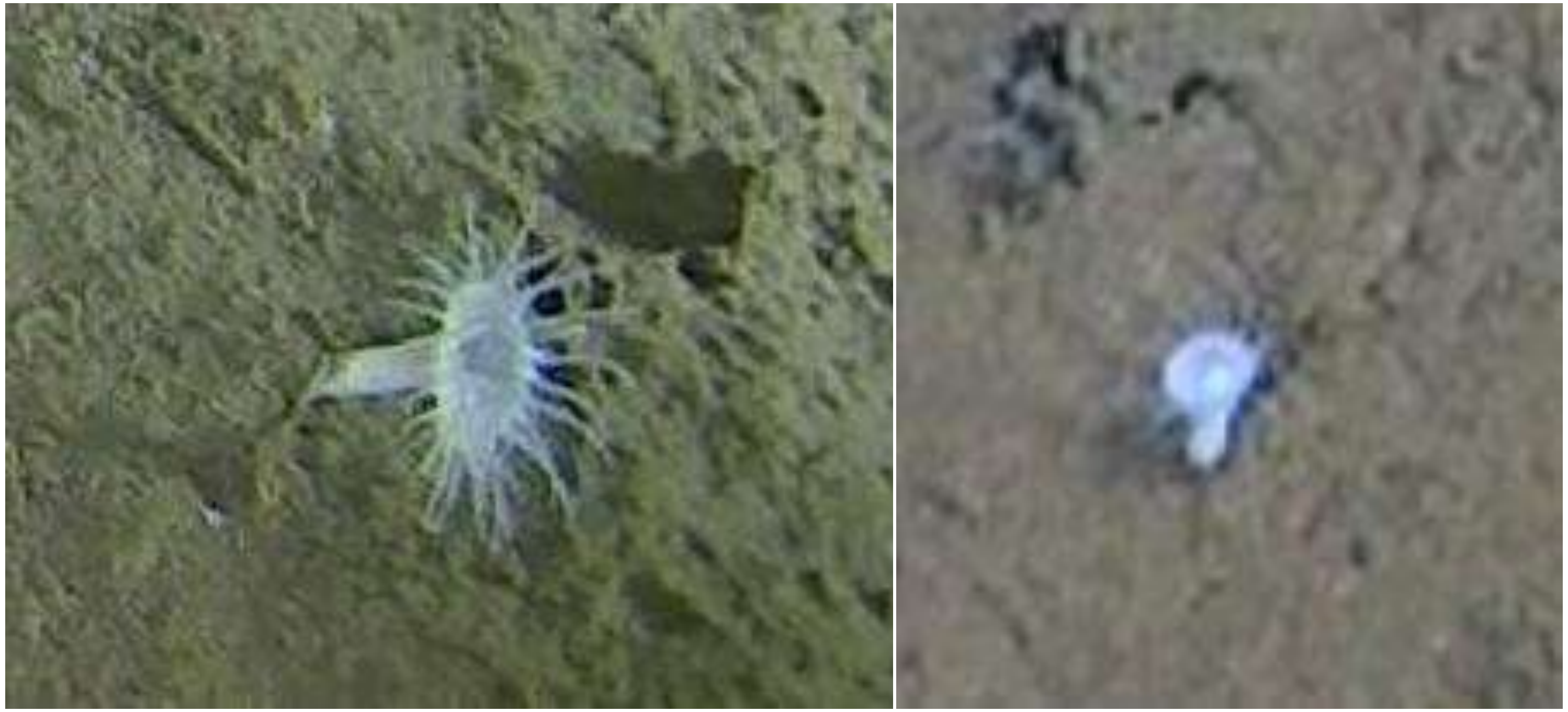

White-bluish medium thin tentacles; attached to a tube or sediment? Thin white column. Very small. Poss juvenile of 1/9?

Hexacorallia; Actiniaria
Actiniaria fam. indet. 12

NC2_BK5_4600_IMG_0400

NC4_BK3_6900

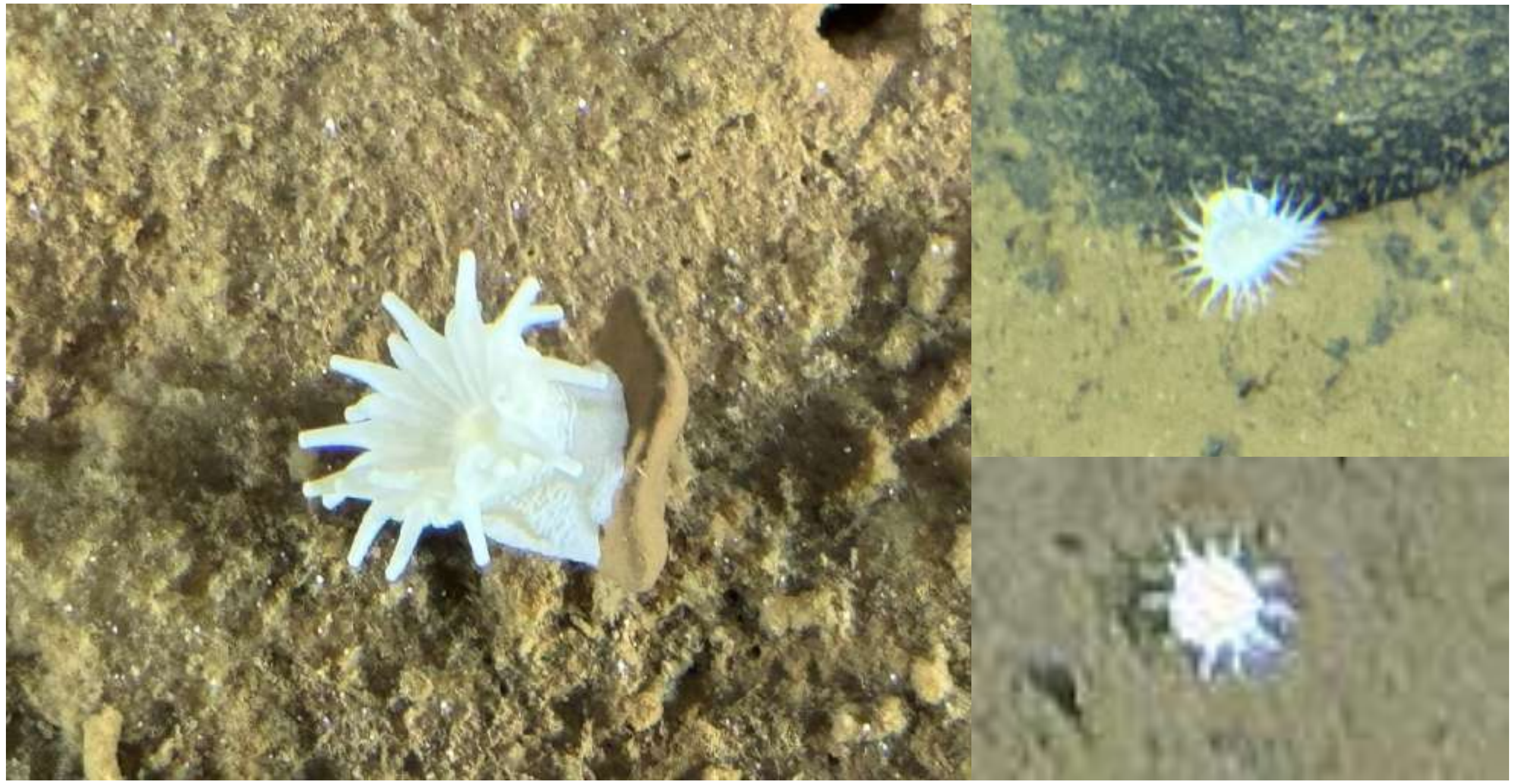

Stubby wide short base, medium length tentacles, one whorl. Found both on sand and rocks. Small,

NC1_BK1_6200_NOR_0057

NC1_BK2_6700_NOR_0080

Hexacorallia; Actiniaria; Actiniaria fam. indet. 13

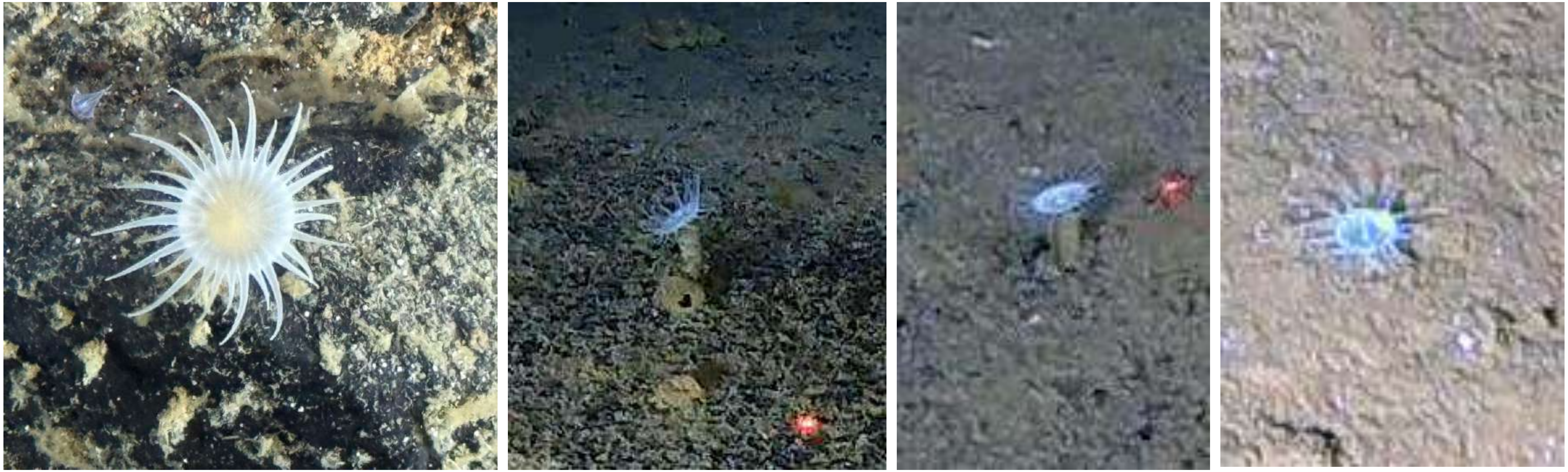

White-bluish oral disc and short tentacles; possibly up to 30. Column sand-like; possibly covered in sediment. Also found embedded in sediment. Very small (1-2cm)

Hexacorallia; Actiniaria; Galatheanthemidae; *Galatheanthemum; Galatheanthemum* sp. indet. 1

NC3_BK4_8000_NOR_0098

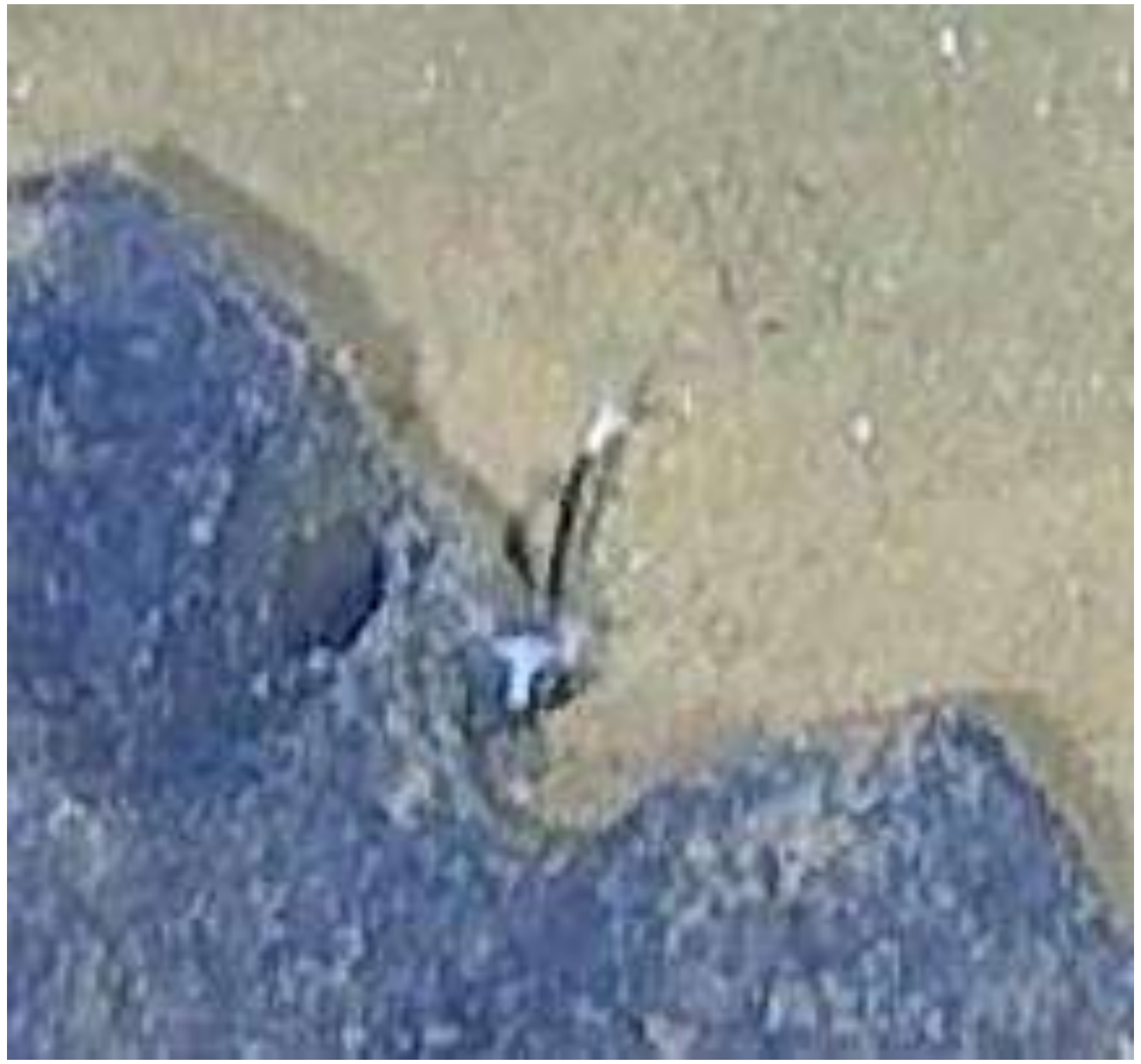

Found on hard sediments. White short tentacles. Column is dark and slender. Light White-bluish oral disc.

Hexacorallia; Actiniaria; Actinoscyphiidae
Actinoscyphiidae gen. indet. 1

NC2_BK2_3800_IMG_8290
NC2_BK5_4600_IMG_9884

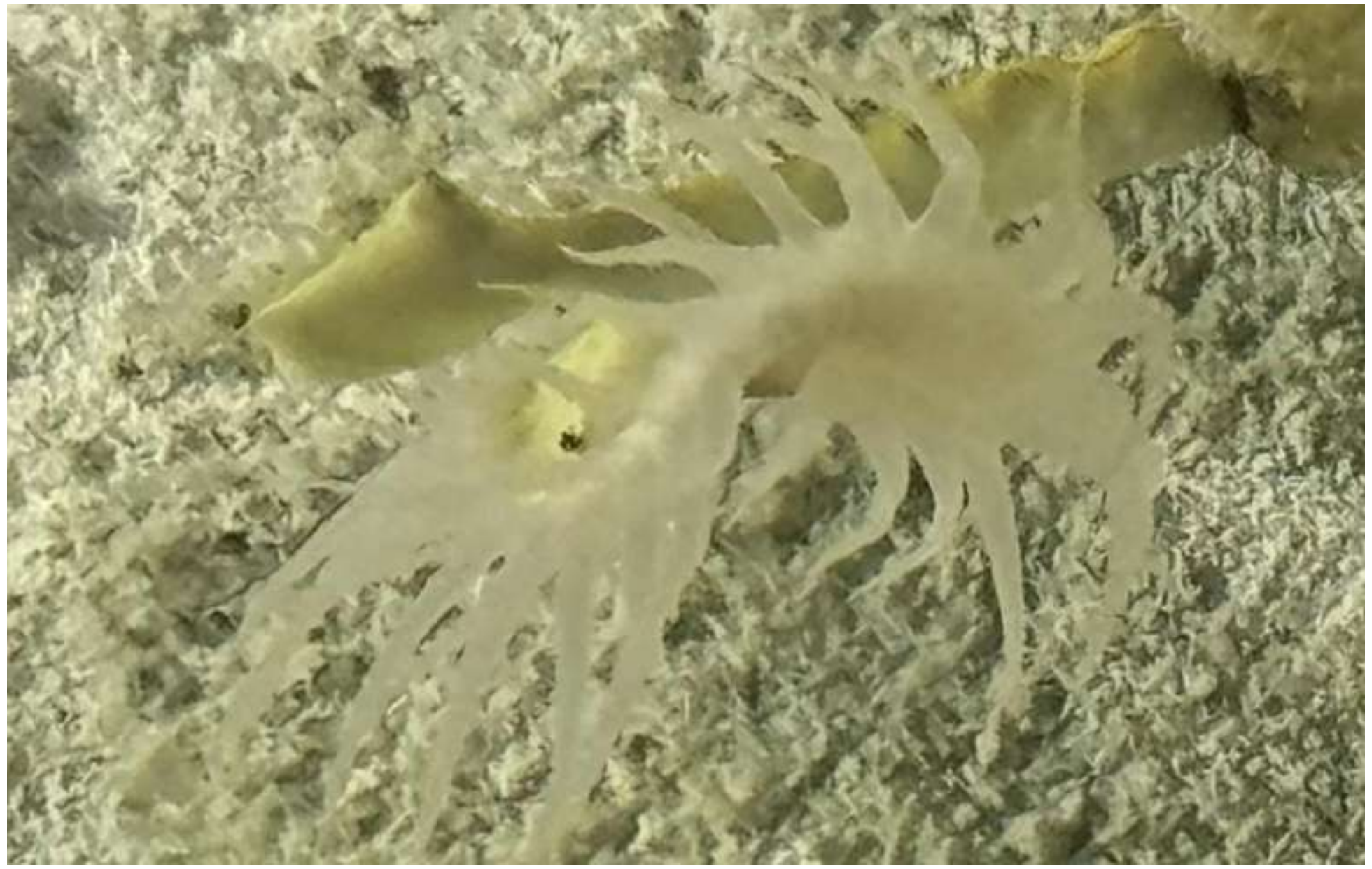

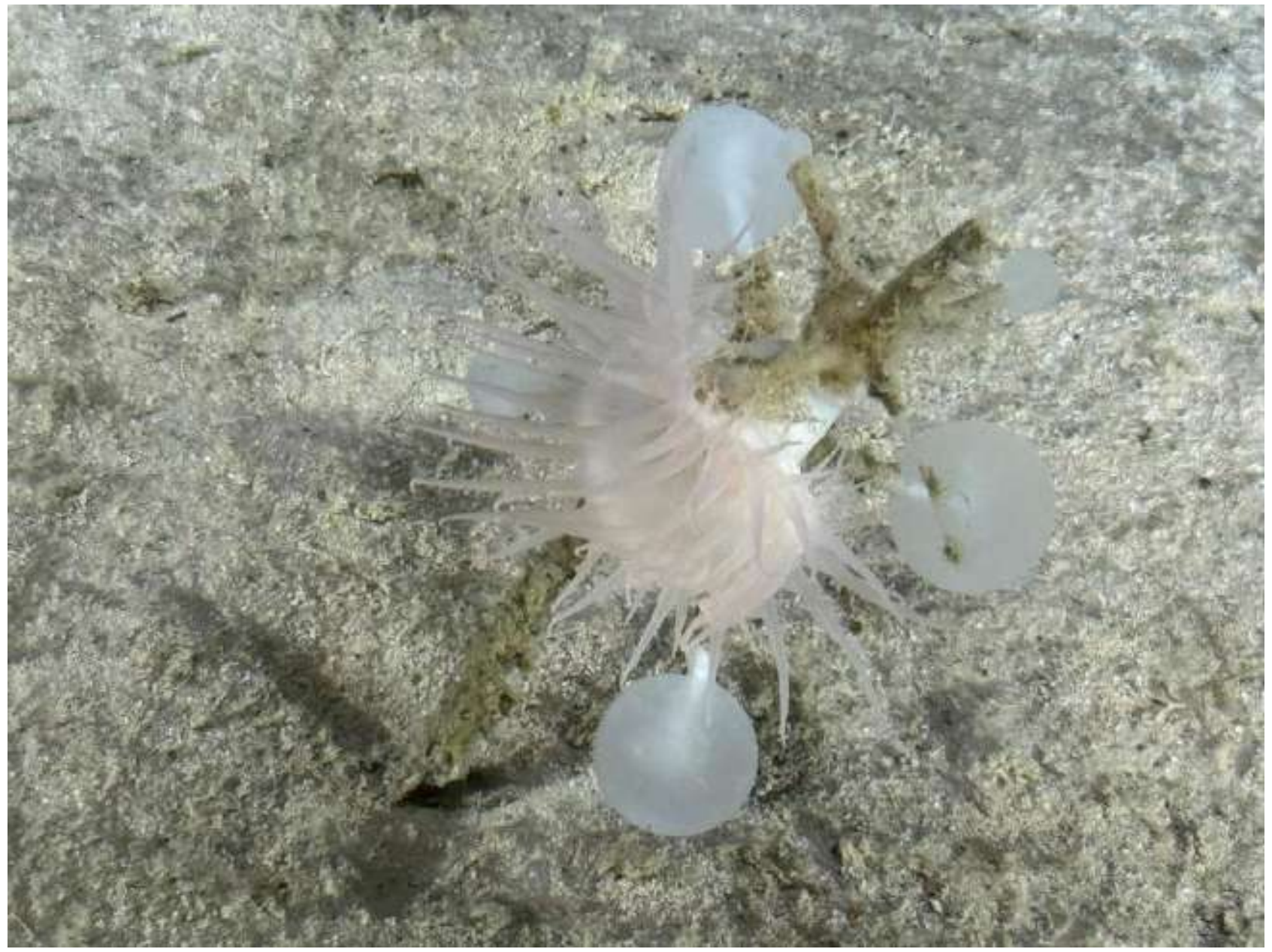

Epibiont on dead black coral. White medium tentacles; possibly up to 40. Column and basal disc not visible. Brownish oral disc; mouth not visible. Typical Venus flytrap shape; asymmetrical and recurve.

NC4_BK4_4700_IMG_6056

Hexacorallia; Actiniaria; Actinoscyphiidae; Actinoscyphiidae gen. indet. 2

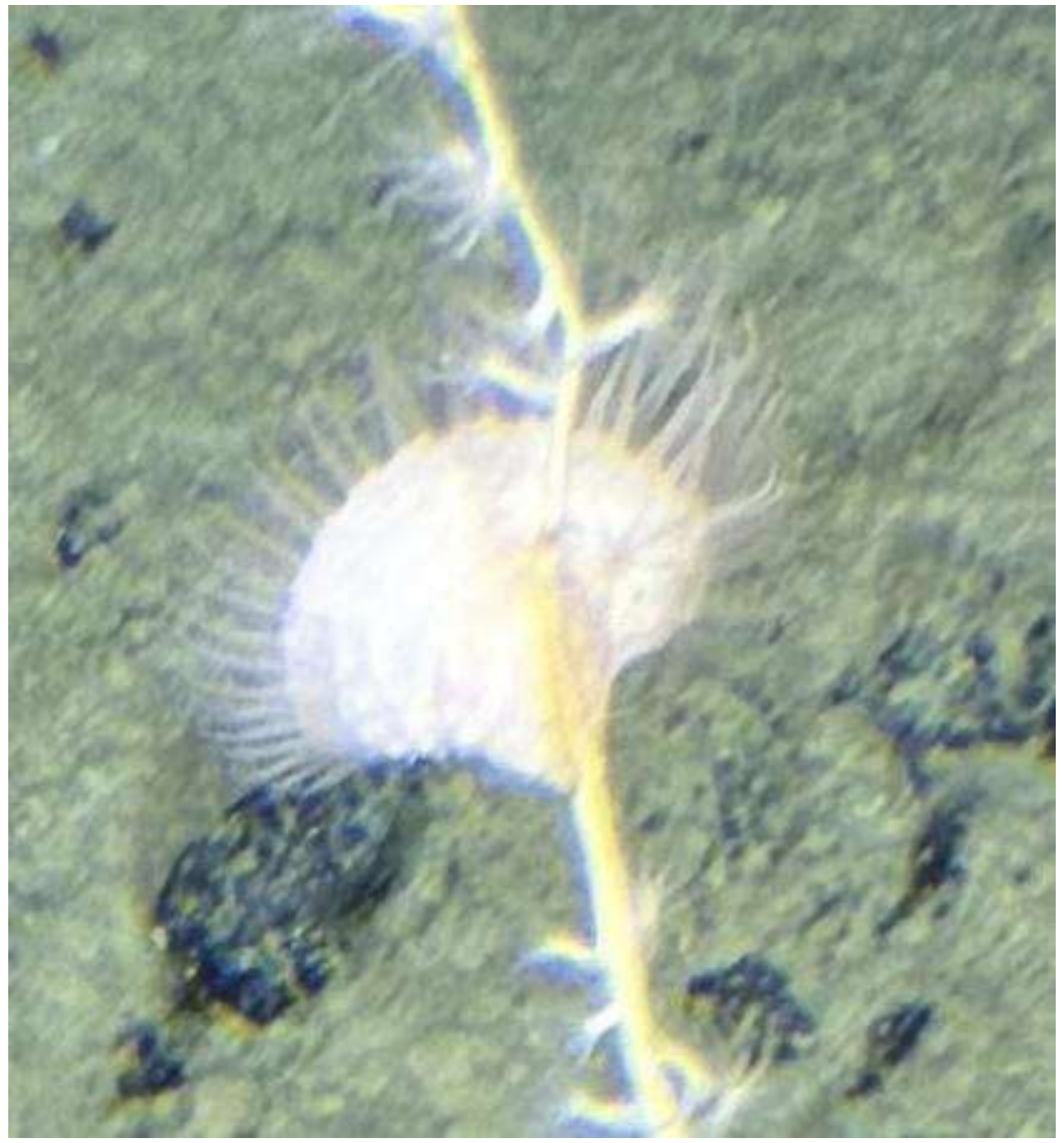

Epibiont on dying octocoral. Pink translucent short to medium tentacles; possibly up to 60. Light pink; reduced column. Oral disc not visible. Typical Venus flytrap shape; asymmetrical and recurve.

Hexacorallia; Actiniaria; Relicanthidae
Relicanthus; Relicanthus sp. indet. 1

NC4_BK4_4700_IMG_6076

NC4_BK2_4700_NOR_0065

NC4_BK6_4700_NOR_0044

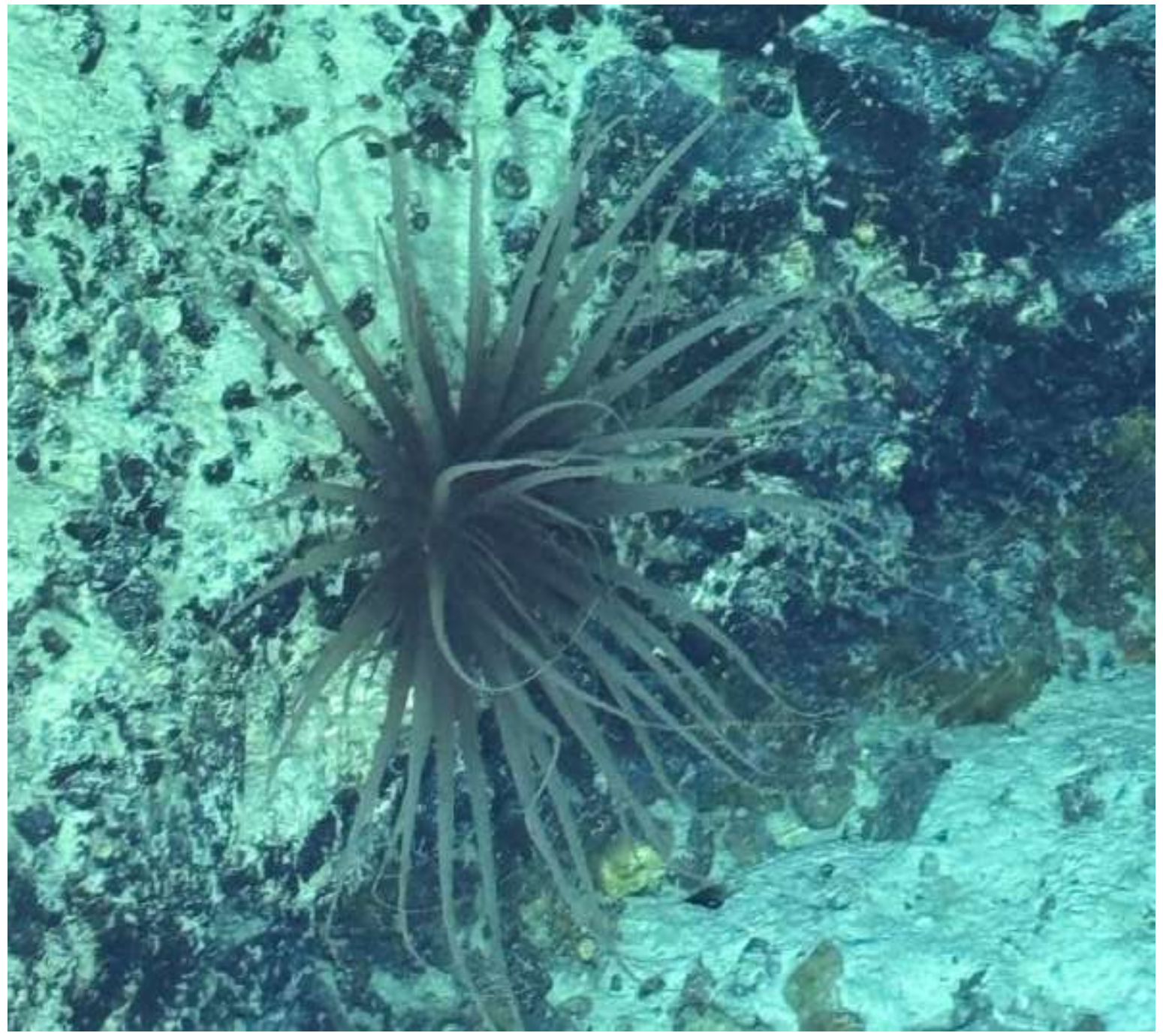
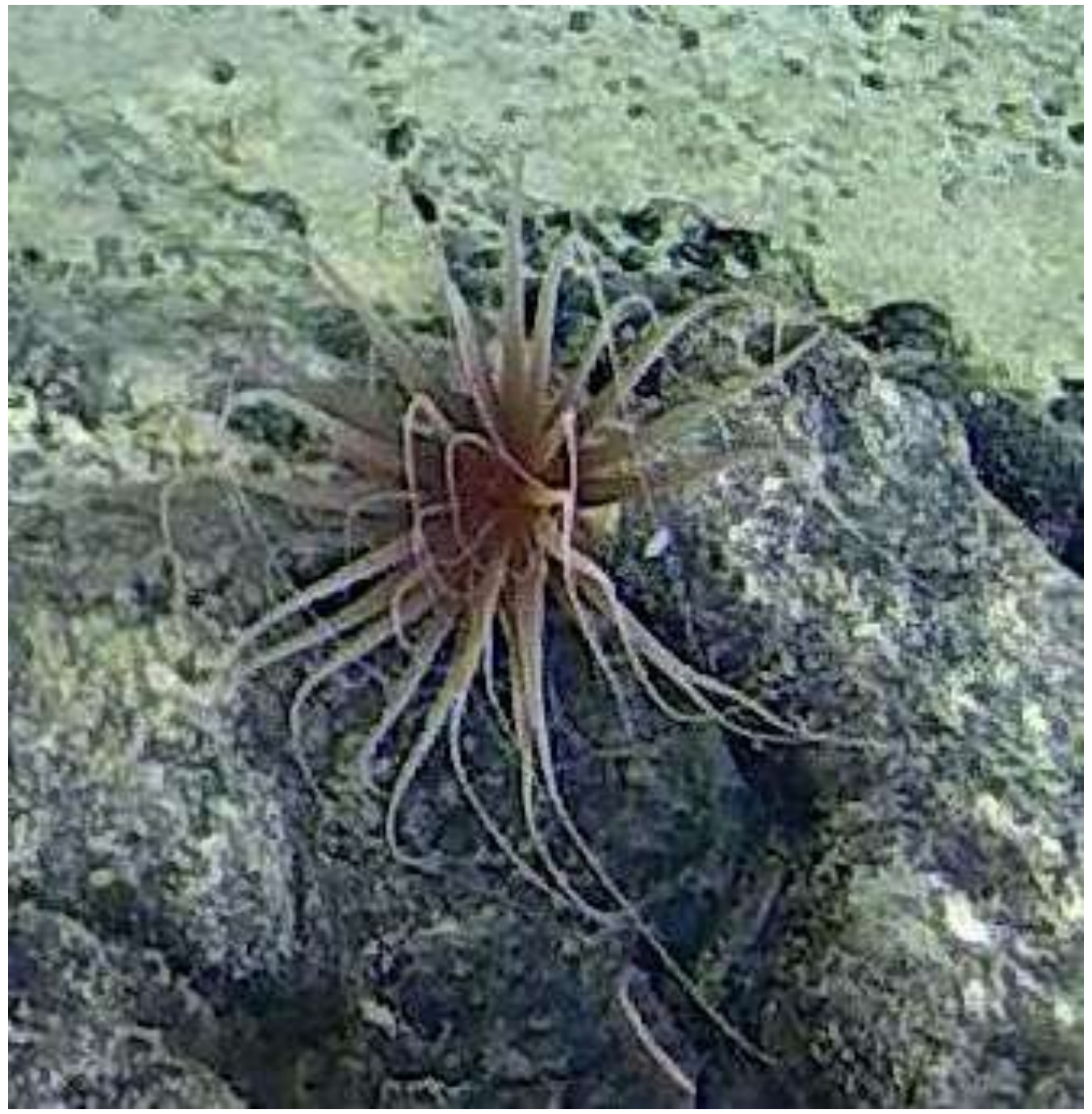
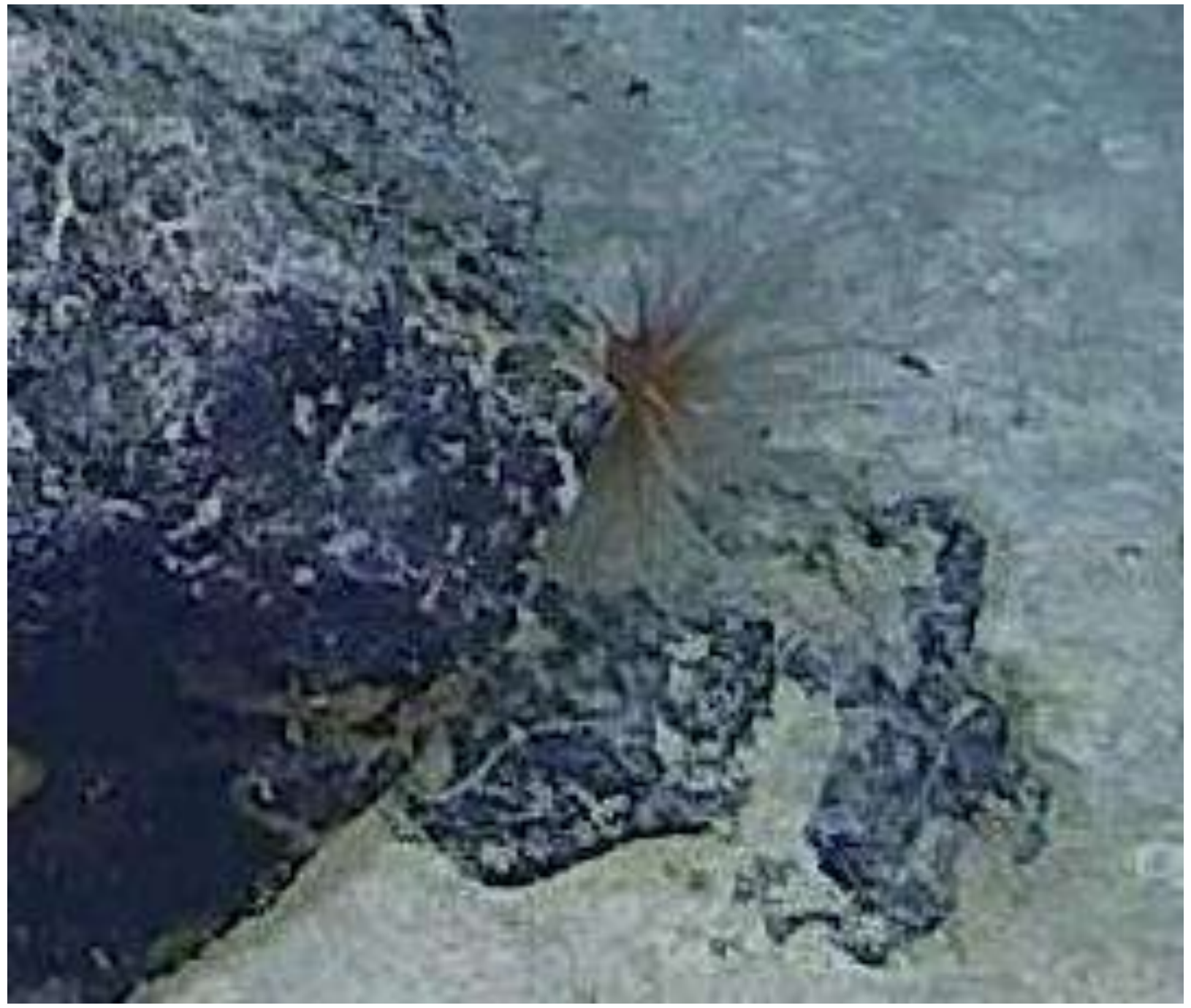

Epibiont on sponge stalk or large rocks. Whitish extremely strongly tapering; deciduous tentacles. Column smooth and stout; colour from pale pink to purplish-red. Basal disc adherent; same colour as column. Mouth with prominent; deep purple to maroon lips; actinopharynx same colour as oral disc. Our specimen are darker than usually reported; may indicate a new species in the genus; perhaps adapted to live on nodules?

Hexacorallia; Zoantharia; Epizoanthidae
Epizoanthus sp. indet.1

NC2_BK1_3200_IMG_7453
NC2_BK1_3200_IMG_7669

NC2_BK2_3800

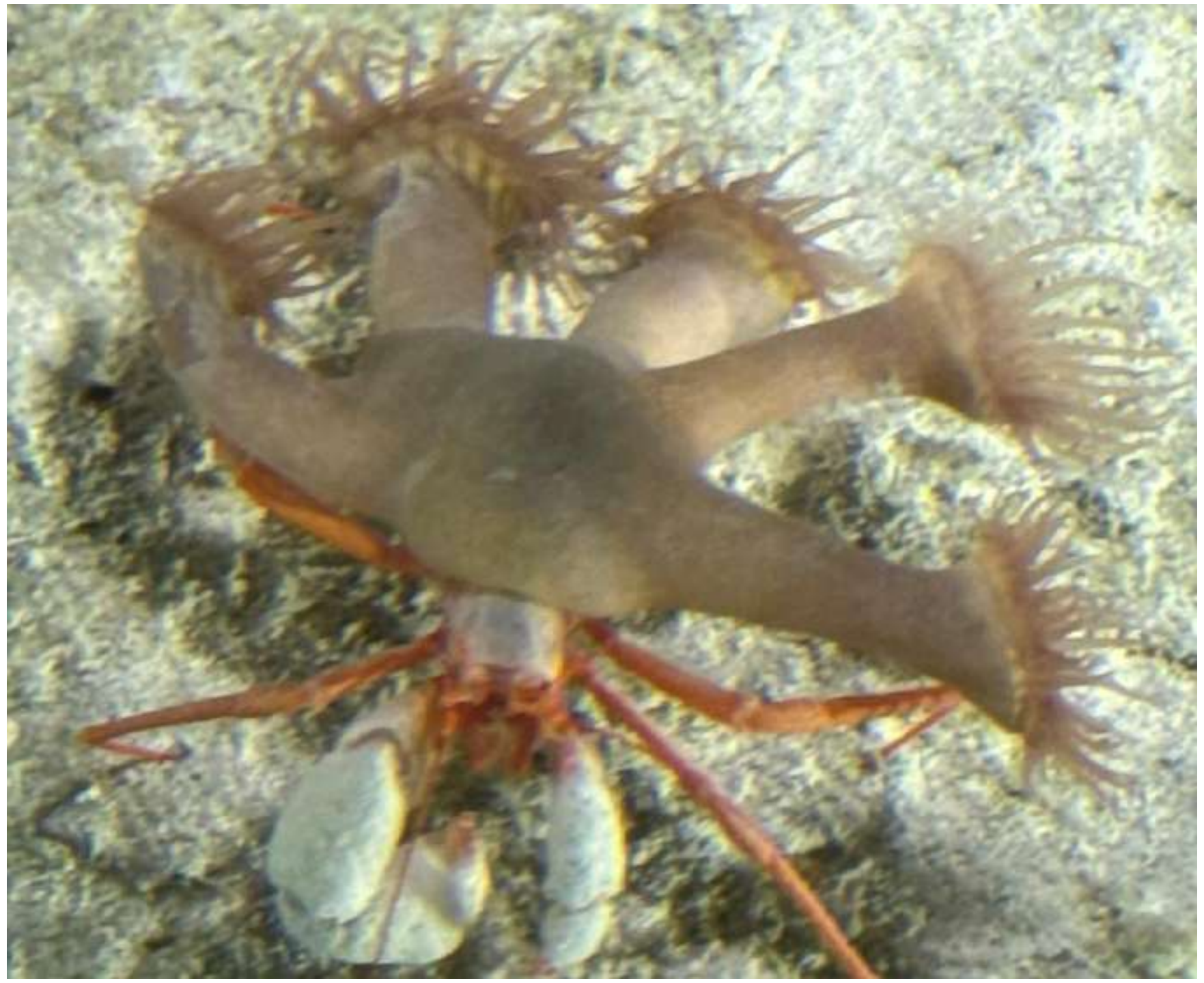

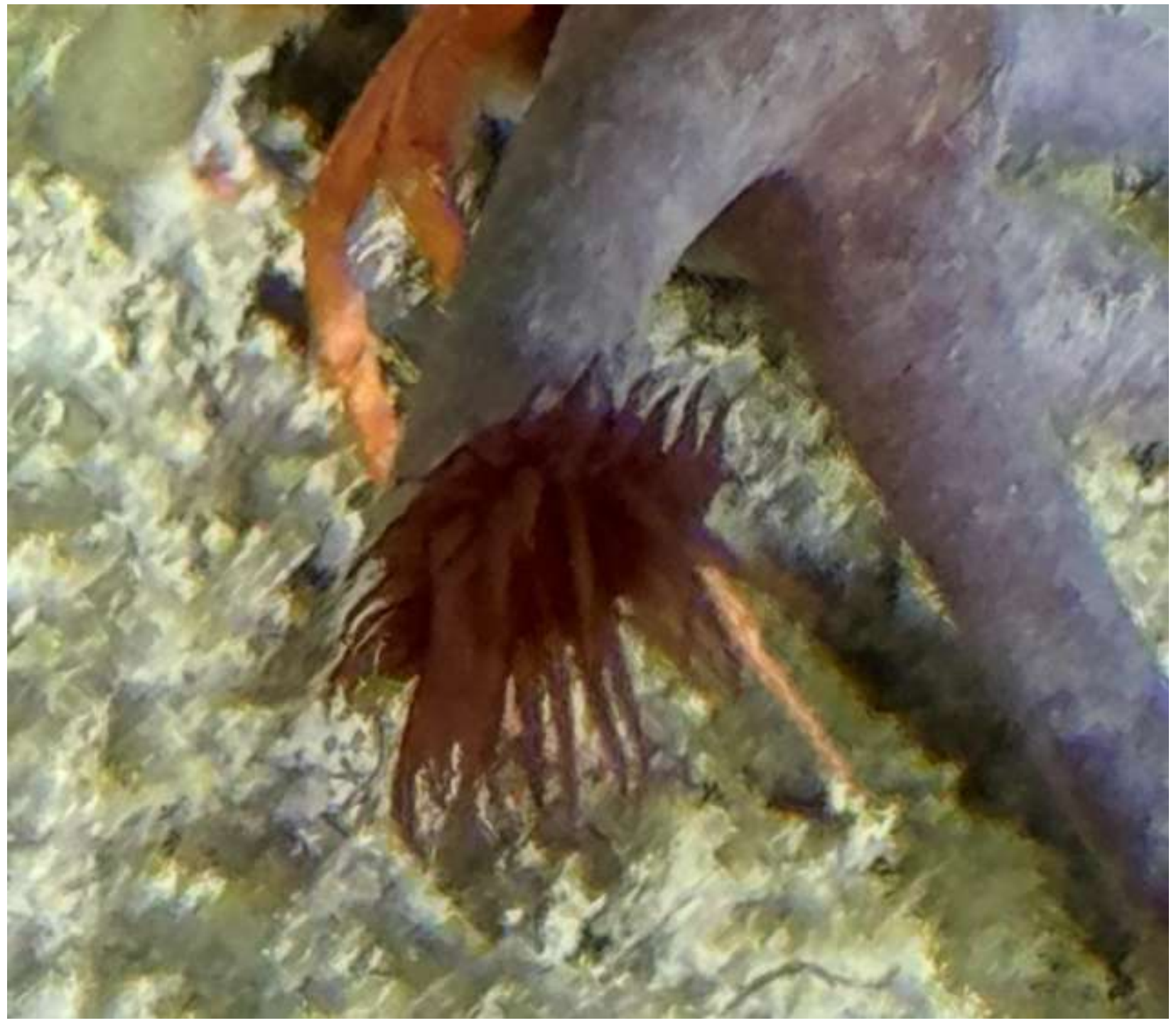

Red medium thin tentacles; possibly up to 50 (uncertain). Reddish stolon and coenenchyma (external organic layer connecting the polyps). Oral disc not visible.

Hexacorallia; Zoantharia; Epizoanthidae
Epizoanthus sp. indet.2

NC4_OM1_4500_NOR_106

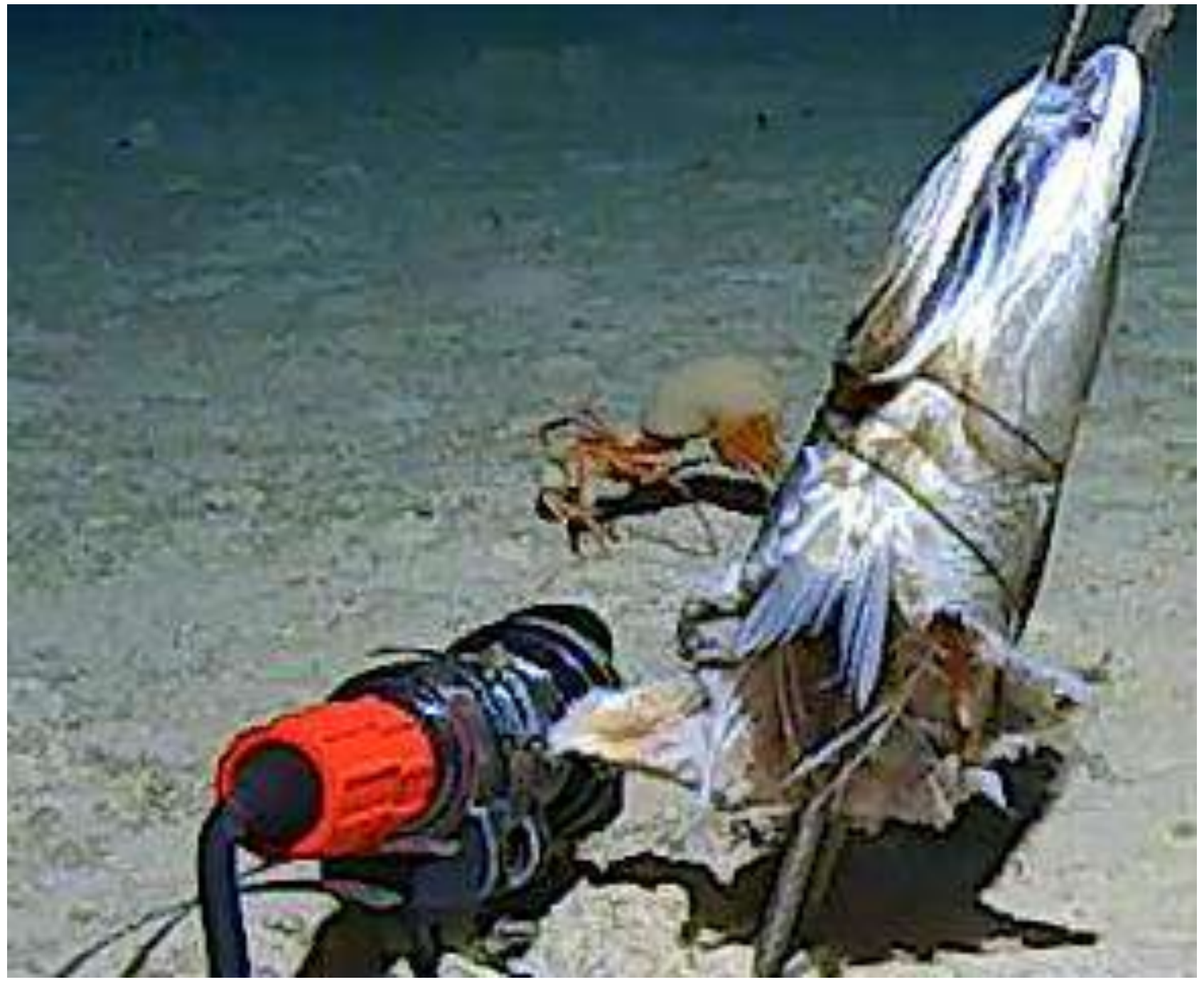

Red medium thin tentacles; possibly up to 50 (uncertain). Reddish stolon and coenenchyma (external organic layer connecting the polyps). One Polyp

Hexacorallia; Zoantharia; Zoantharia gen. indet.1

NC2_BK1_3200_IMG_7631

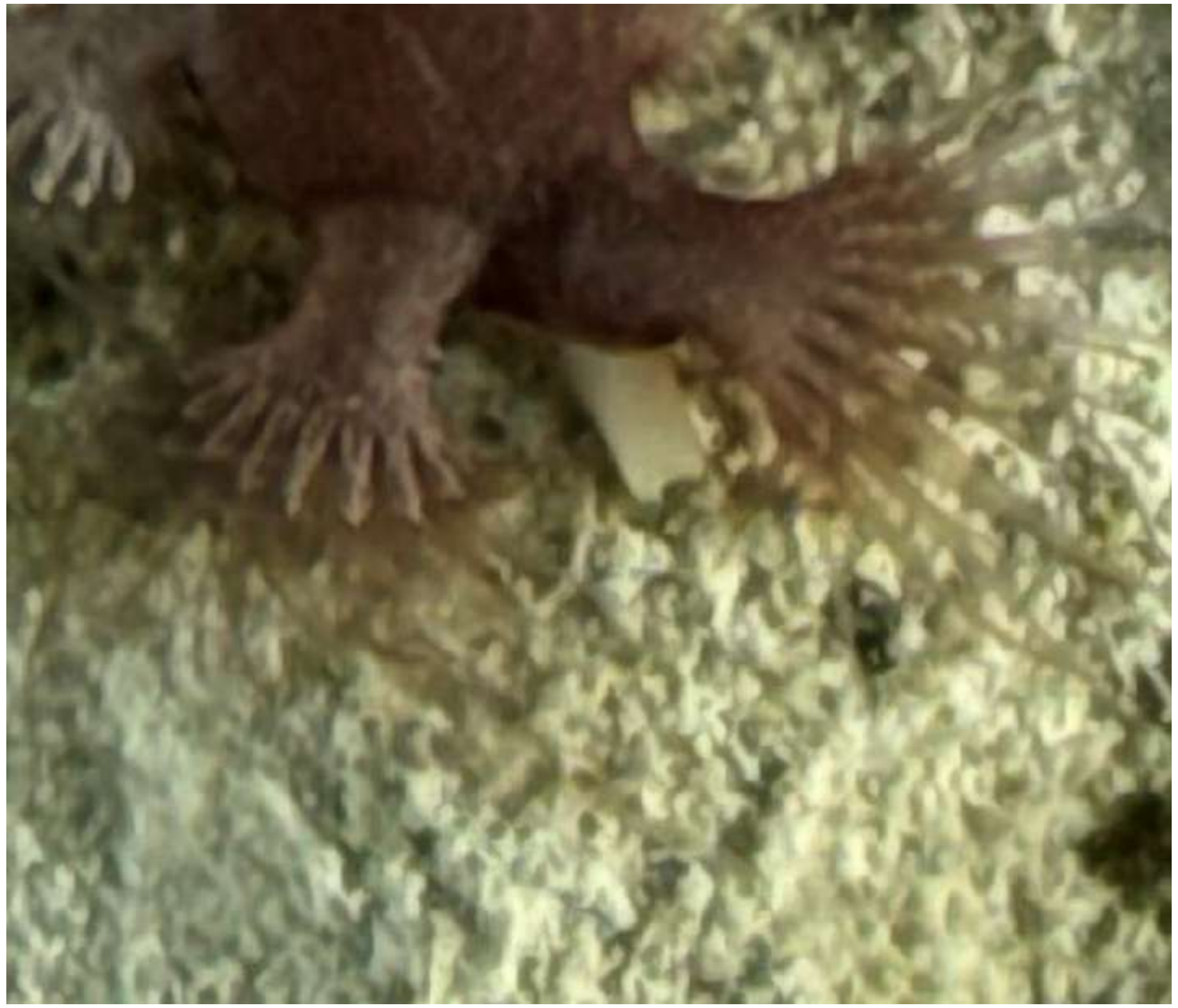

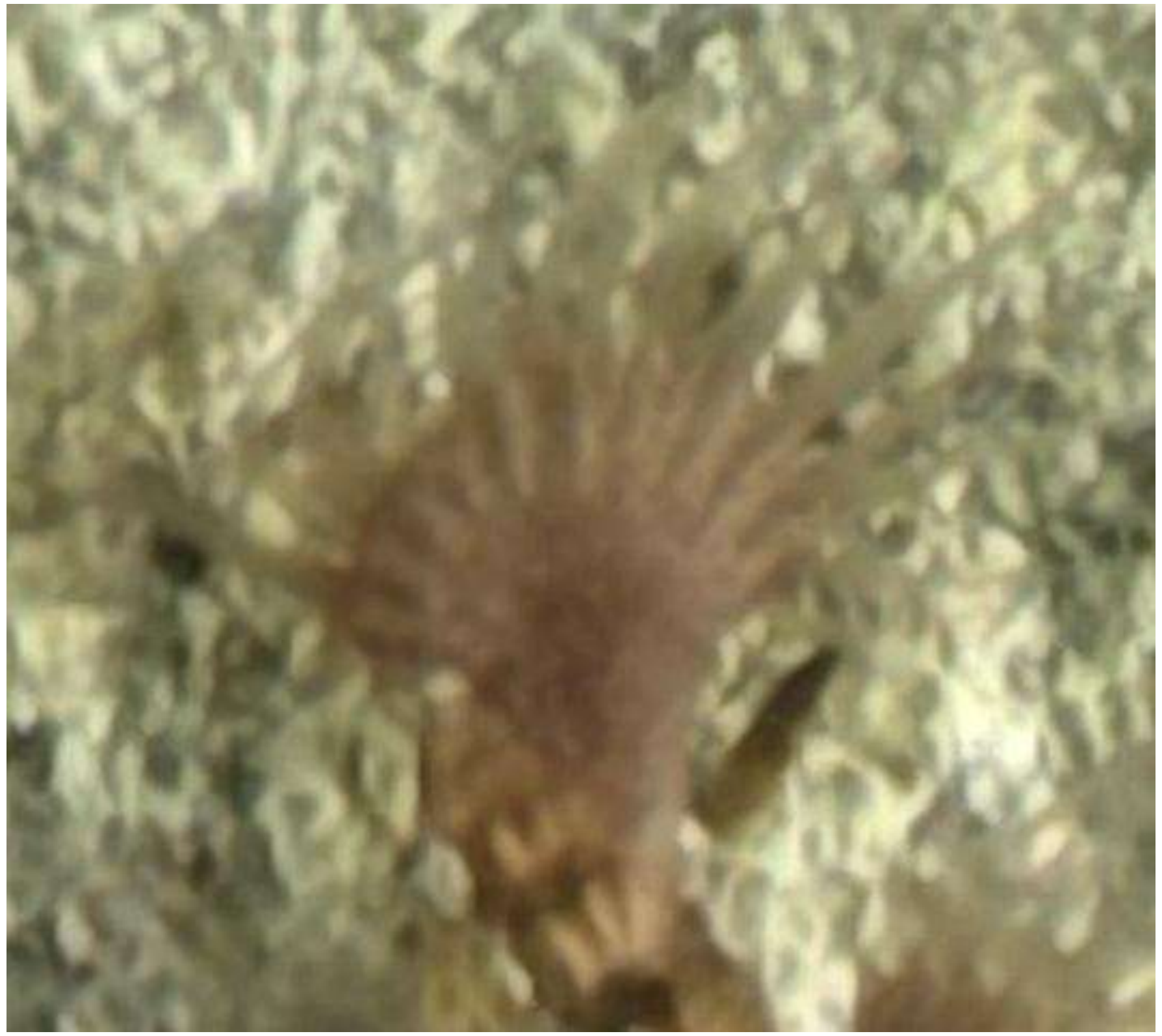

Red thin tentacles; possibly up to 20 (uncertain). Reddish stolon and coenenchyma (external organic layer connecting the polyps). Oral disc not visible.

Hexacorallia; Antipatharia; *Bathypathes* sp. indet. 1

NC4_BK6_4700

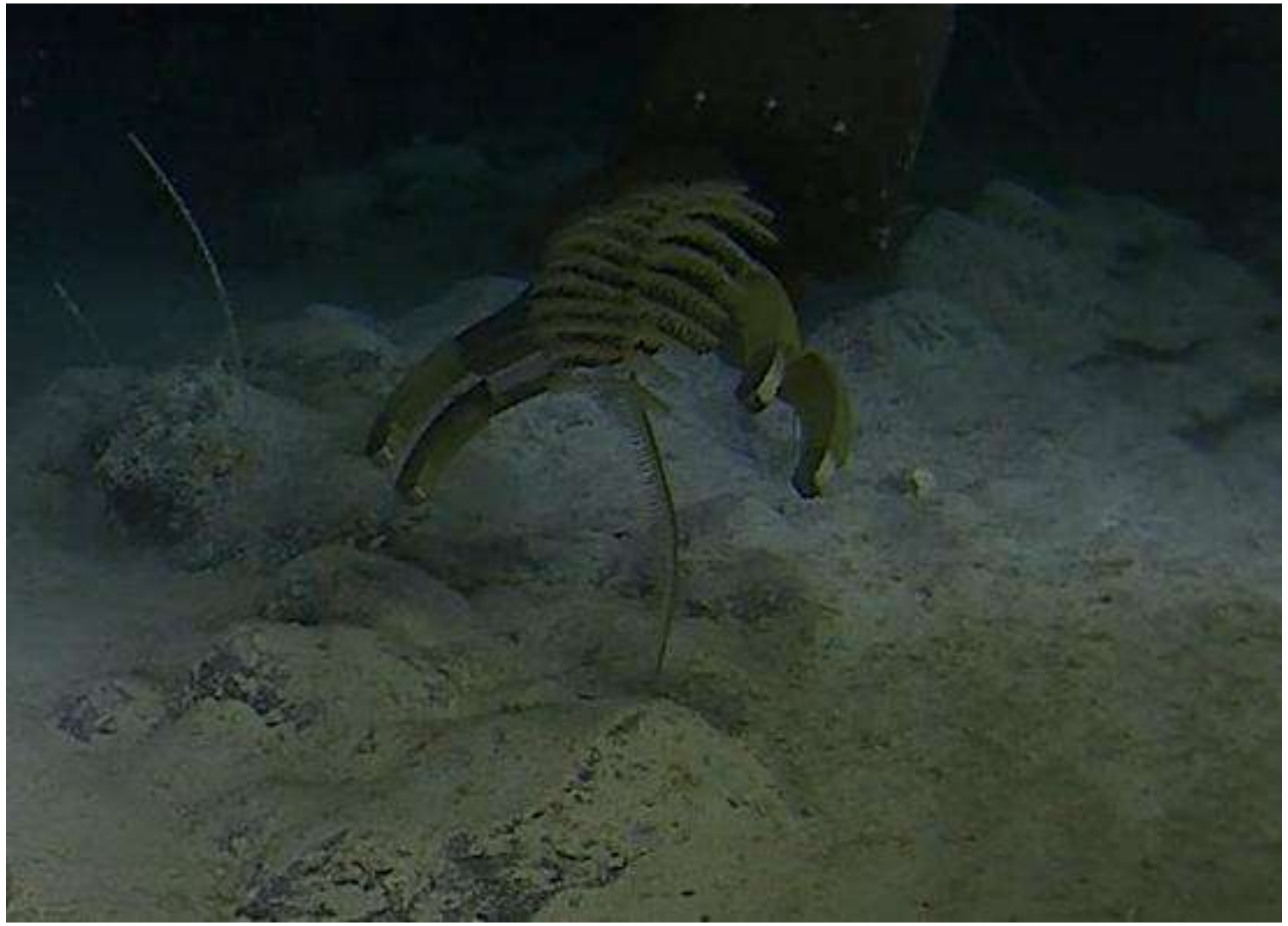

Hexacorallia; Antipatharia; Schizopathidae gen. indet. 1

NC2_BK5_4600_IMG_0441

NC2_BK5_4600_IMG_0447

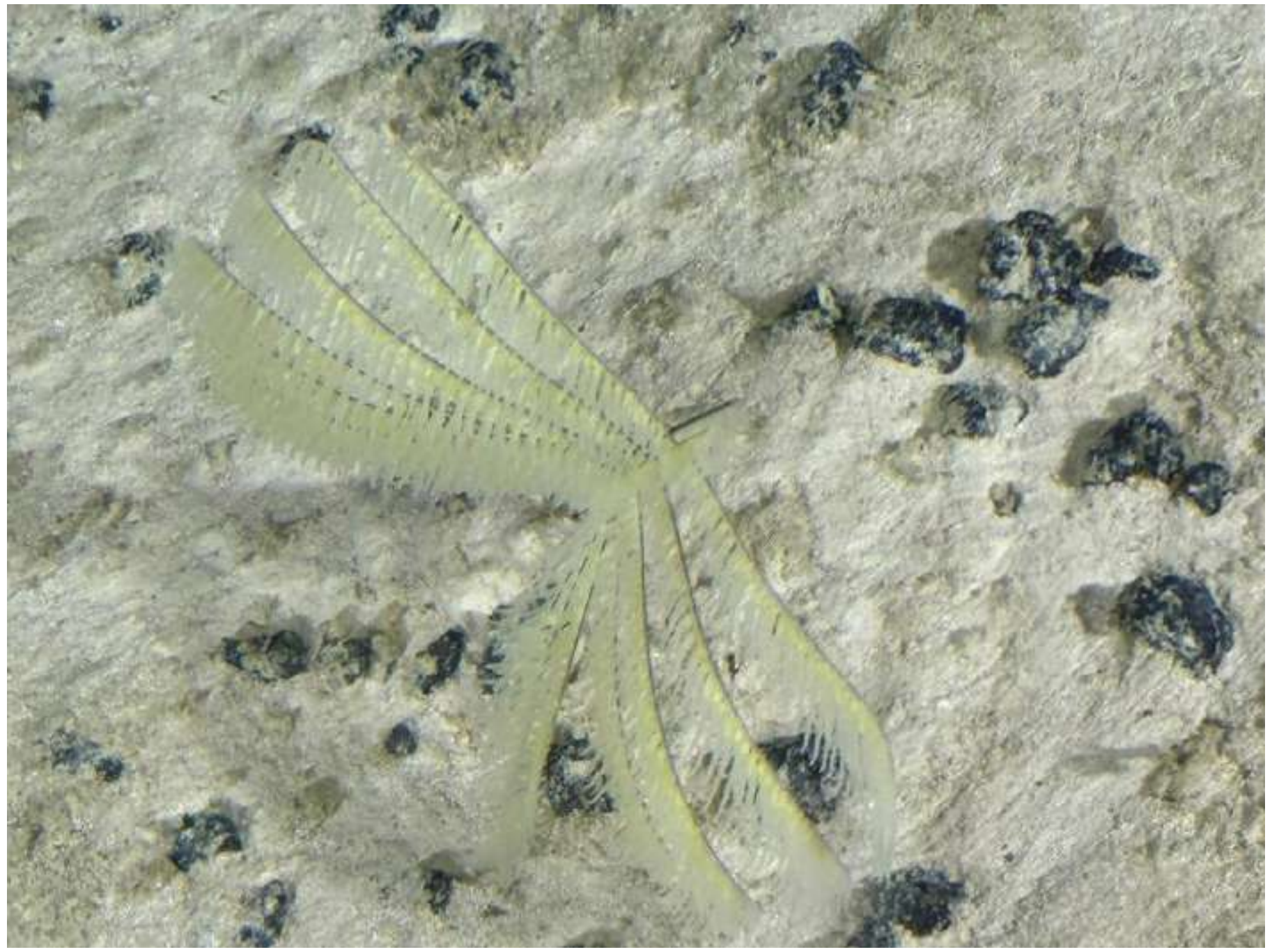

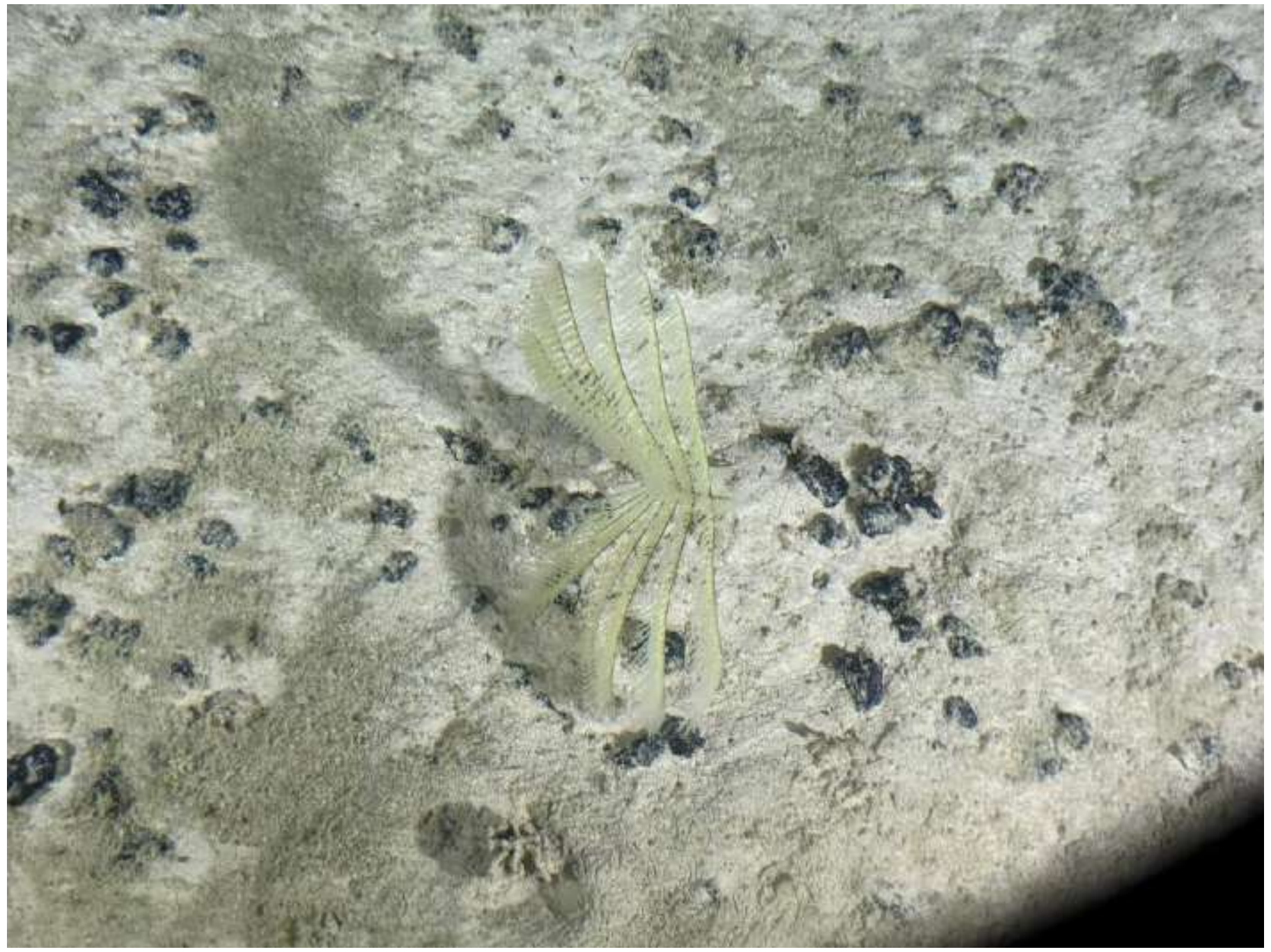

Corallum attaches to hard substrate. Pinnules are simple and are arranged parallel in two lateral rows of 7 pinnules. Pinnules lengthen and then shorten distally. Stem un-pinnulated. ; cf Bathypathes sp. *ID provided by Erika Gress.*

Hexacorallia; Antipatharia; Schizopathidae gen. indet. 2

NC4_BK2_4700_NOR_0056

NC4_BK2_4700_IMG_4877

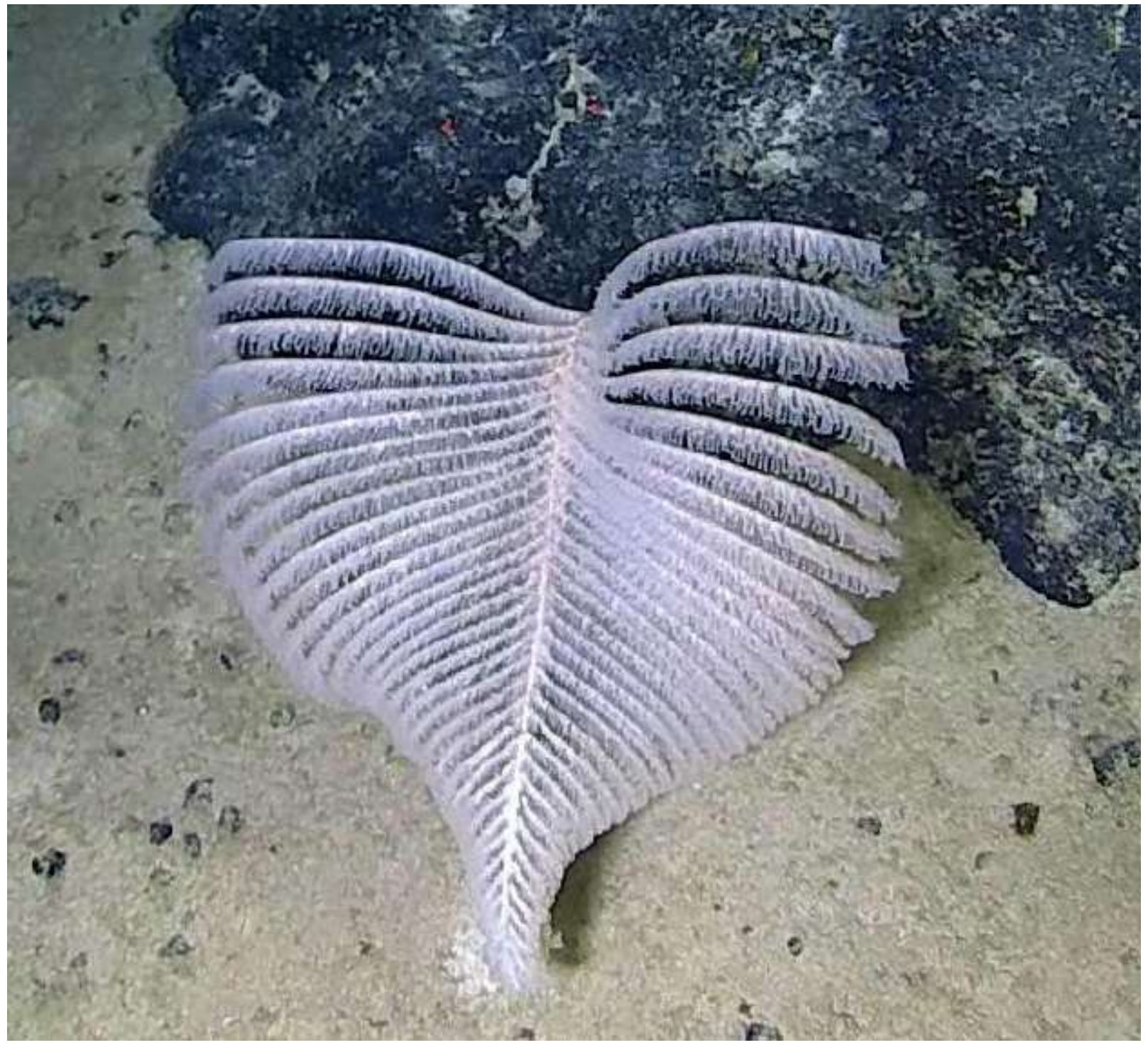

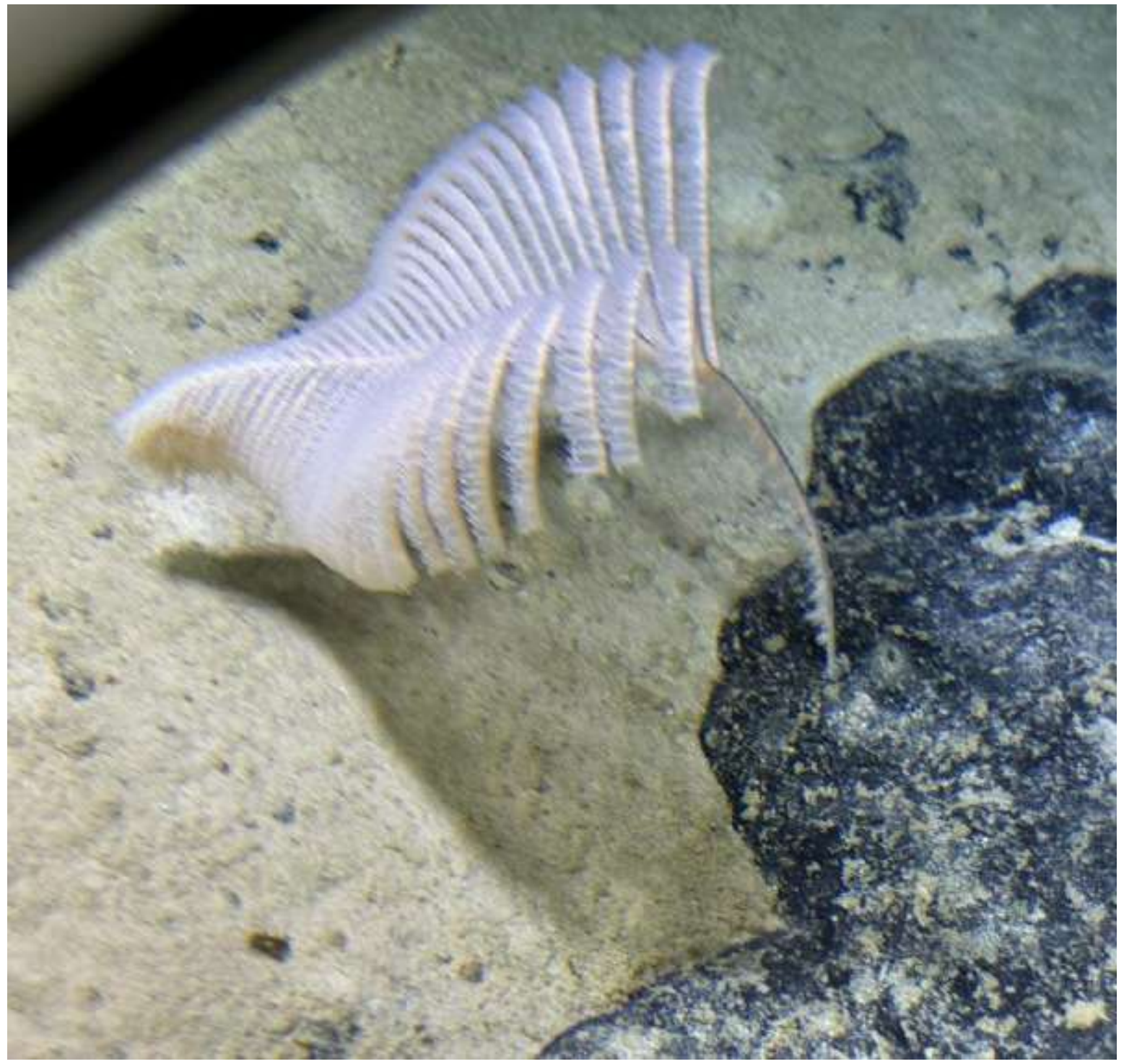

Corallum attaches to hard substrate. Pinnules are simple and are arranged parallel in two lateral rows of multiple pinnules. Pinnules shorten distally. Stem is pinnulated. ID provided by Erika Gress.

Hexacorallia; Antipatharia; Schizopathidae gen. indet. 3

NC2_BK5_4600_IMG_0515

NC2_BK5_4600_IMG_0520

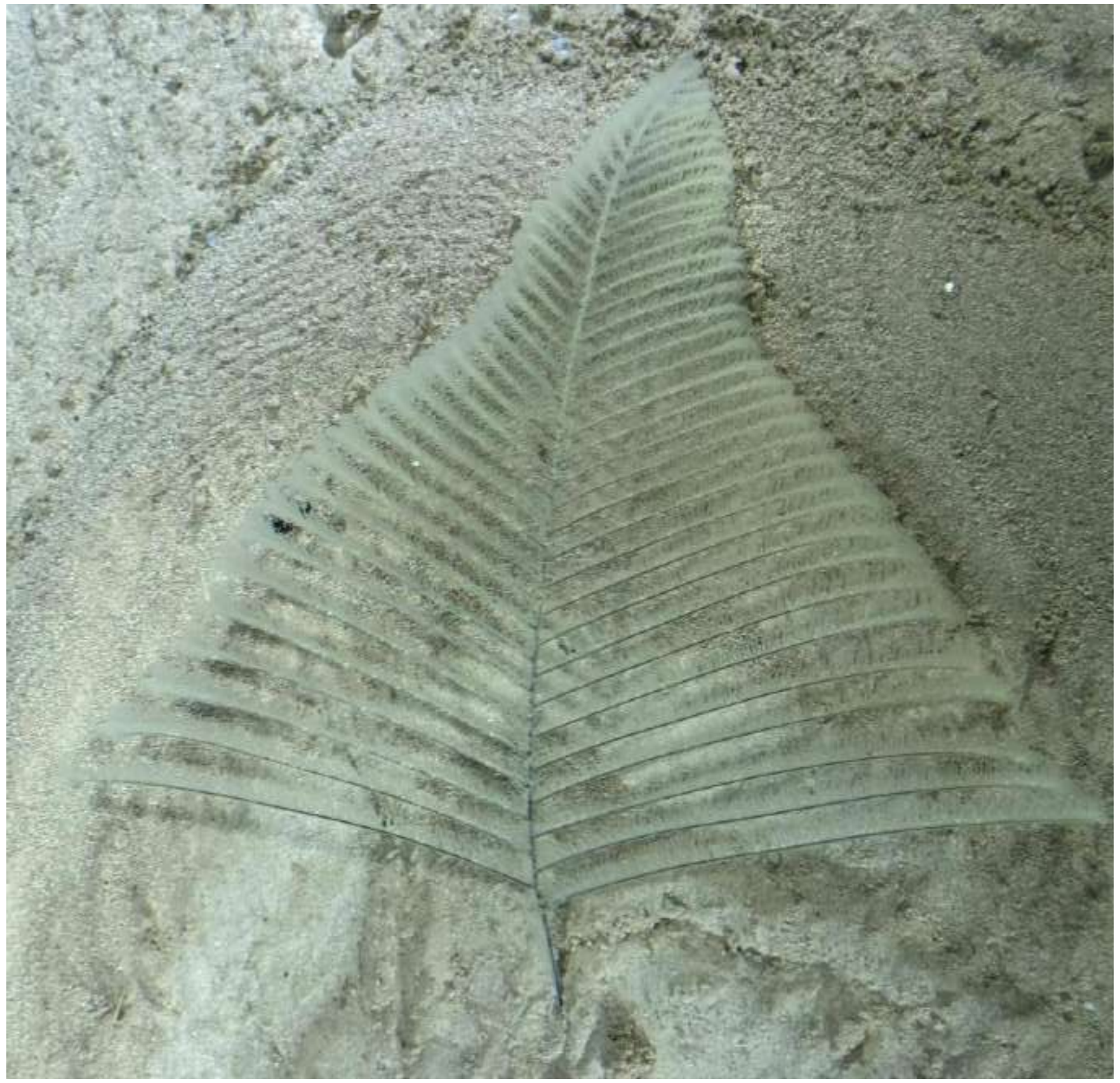

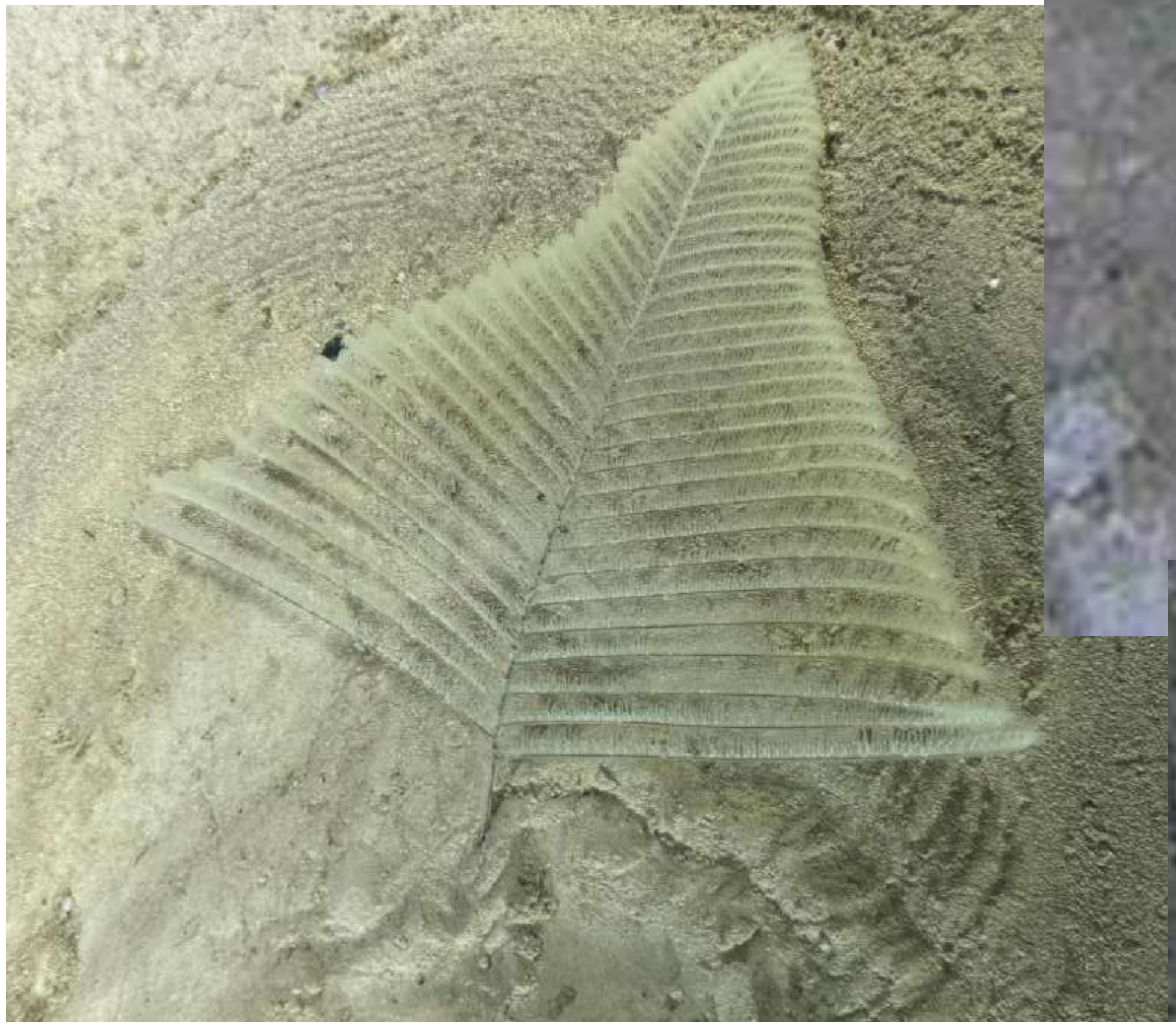

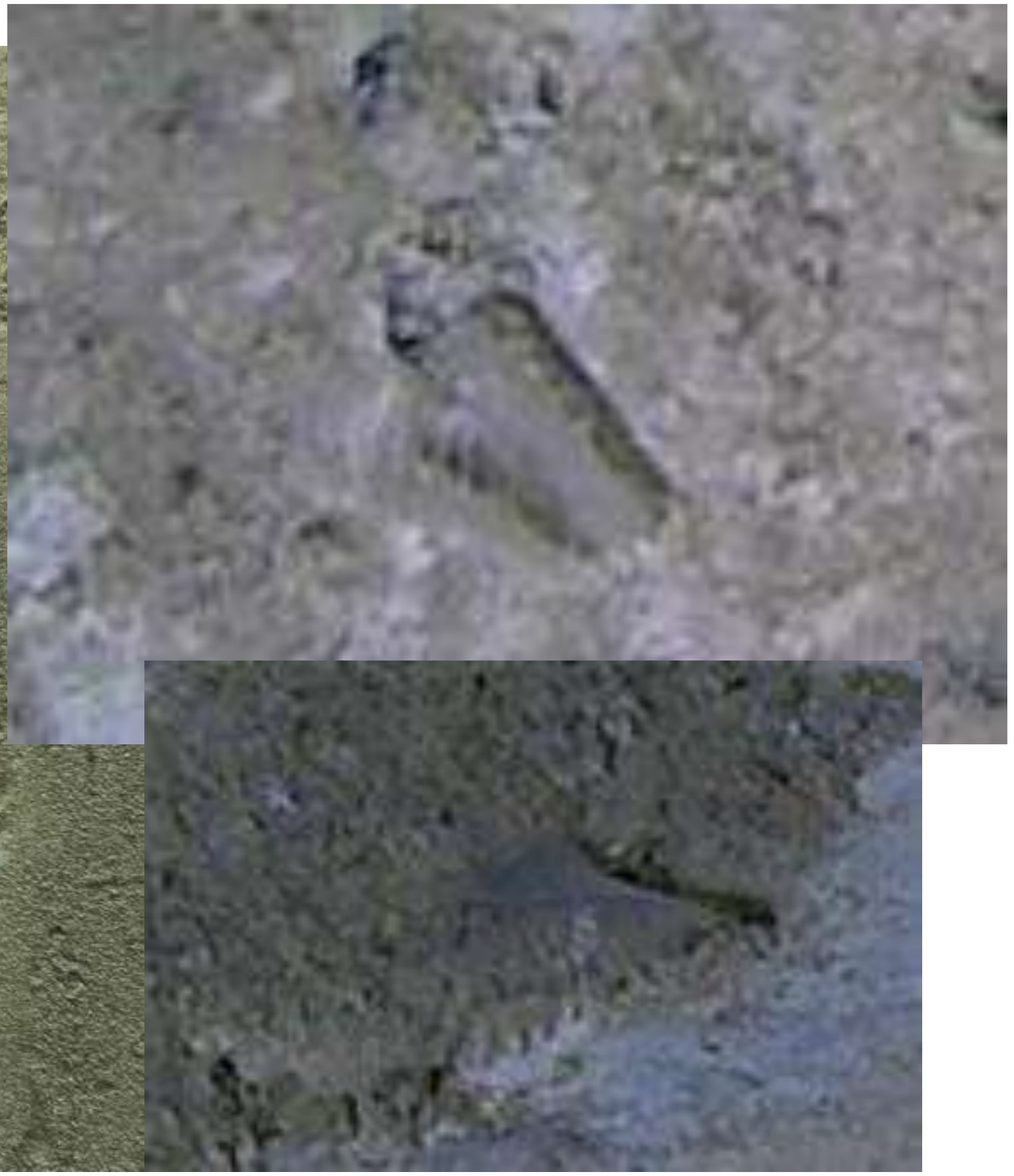

Corallum attaches to soft substrate. Pinnules are simple and are arranged in two alternating rows of multiple pinnules. Pinnules shorten distally. Stem is un-pinnulated. ID provided by Erika Gress.

Hexacorallia; Antipatharia; Cladopathidae; *Abyssopathes* sp. indet. 1

NC3_BK2_6000

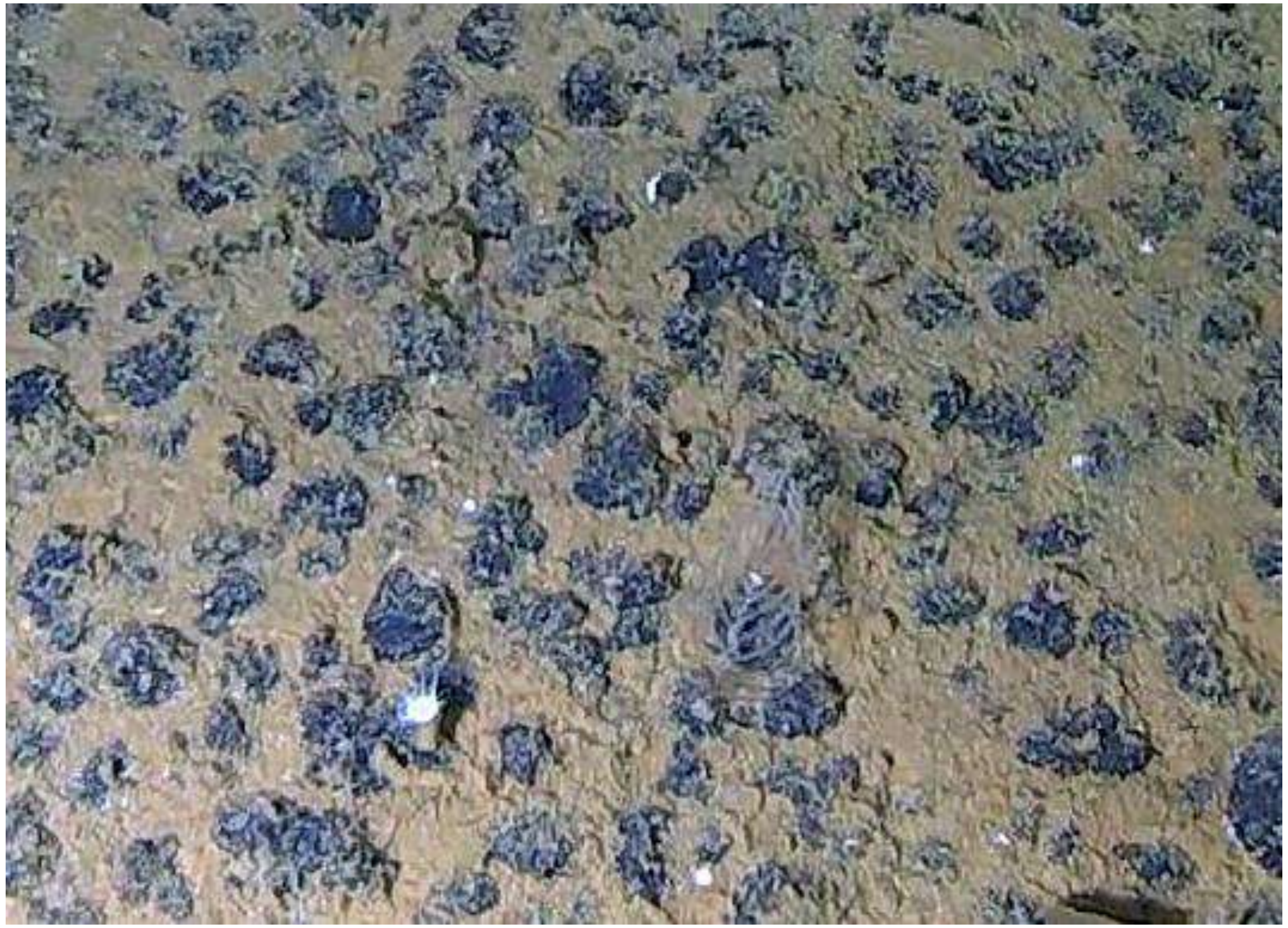

- Identification remains uncertain due to image limitations
- Colony appears to exhibit a funnel-like morphology
- This is consistent with reported colony forms in *Abyssopathes*
- The genus is characteristic of deep-water habitats, including seamounts and areas with polymetallic nodules or cobalt-rich crusts
- Colonies may form harp-shaped or funnel-like structures

Hexacorallia; Antipatharia; Antipatharia fam. inc. 1

NC4_BK4_4700_NOR0090

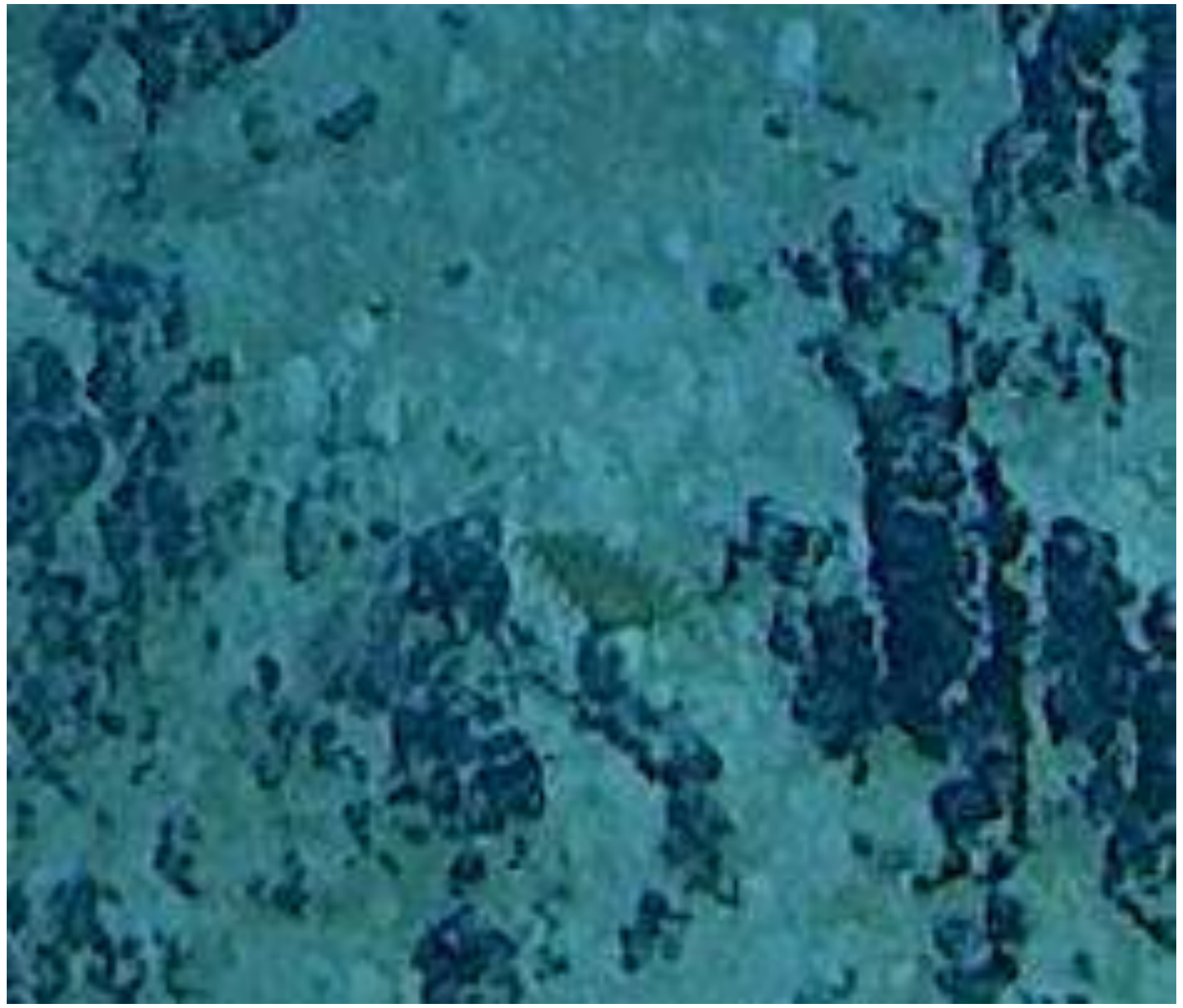

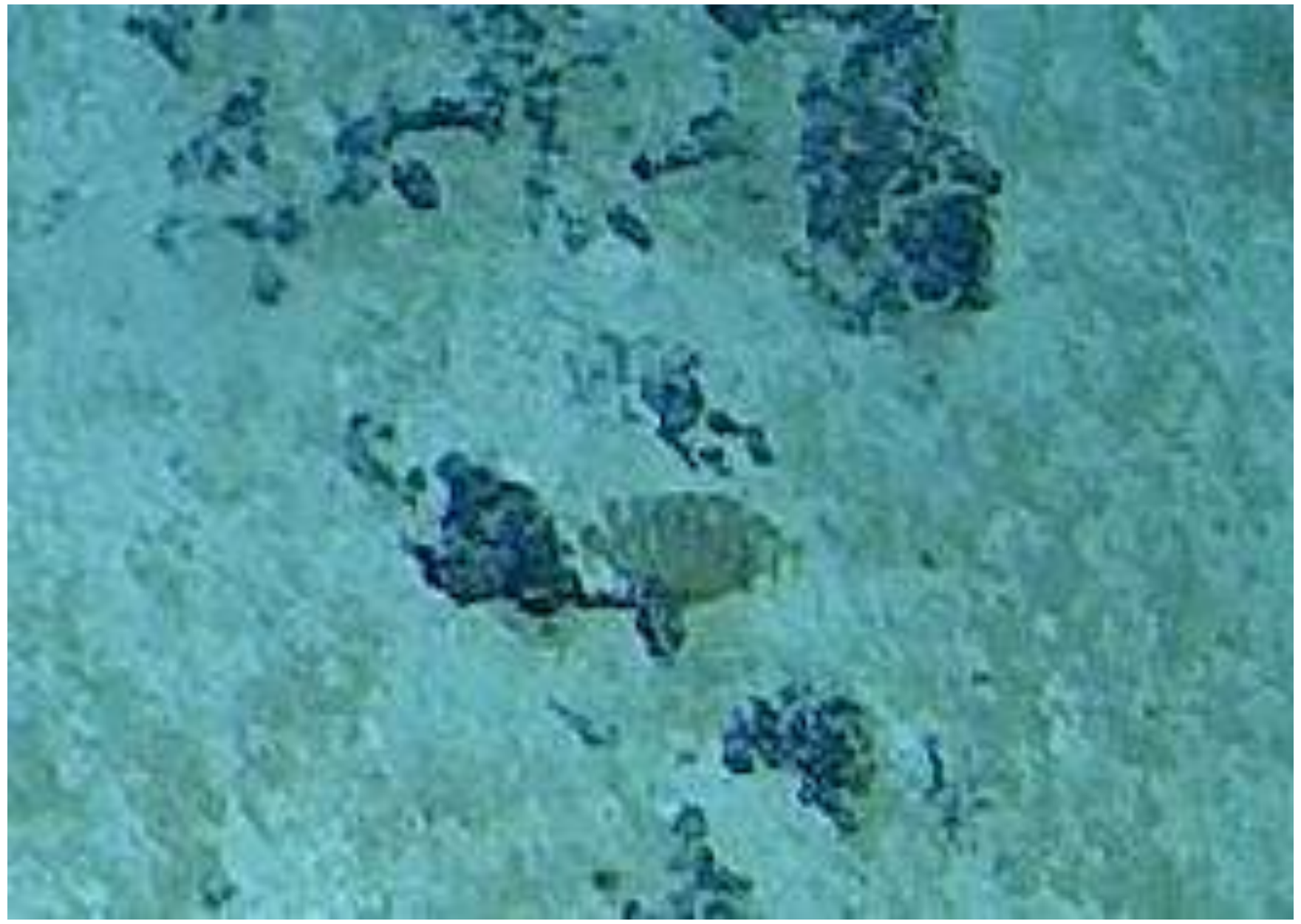

Triangular 'harp' like shape. No clearer reference imagery available

Hexacorallia; Ceriantharia; Ceriantharia fam. indet. 1

NC2_BK1_3200_7119

NC2_BK2_3800_7818

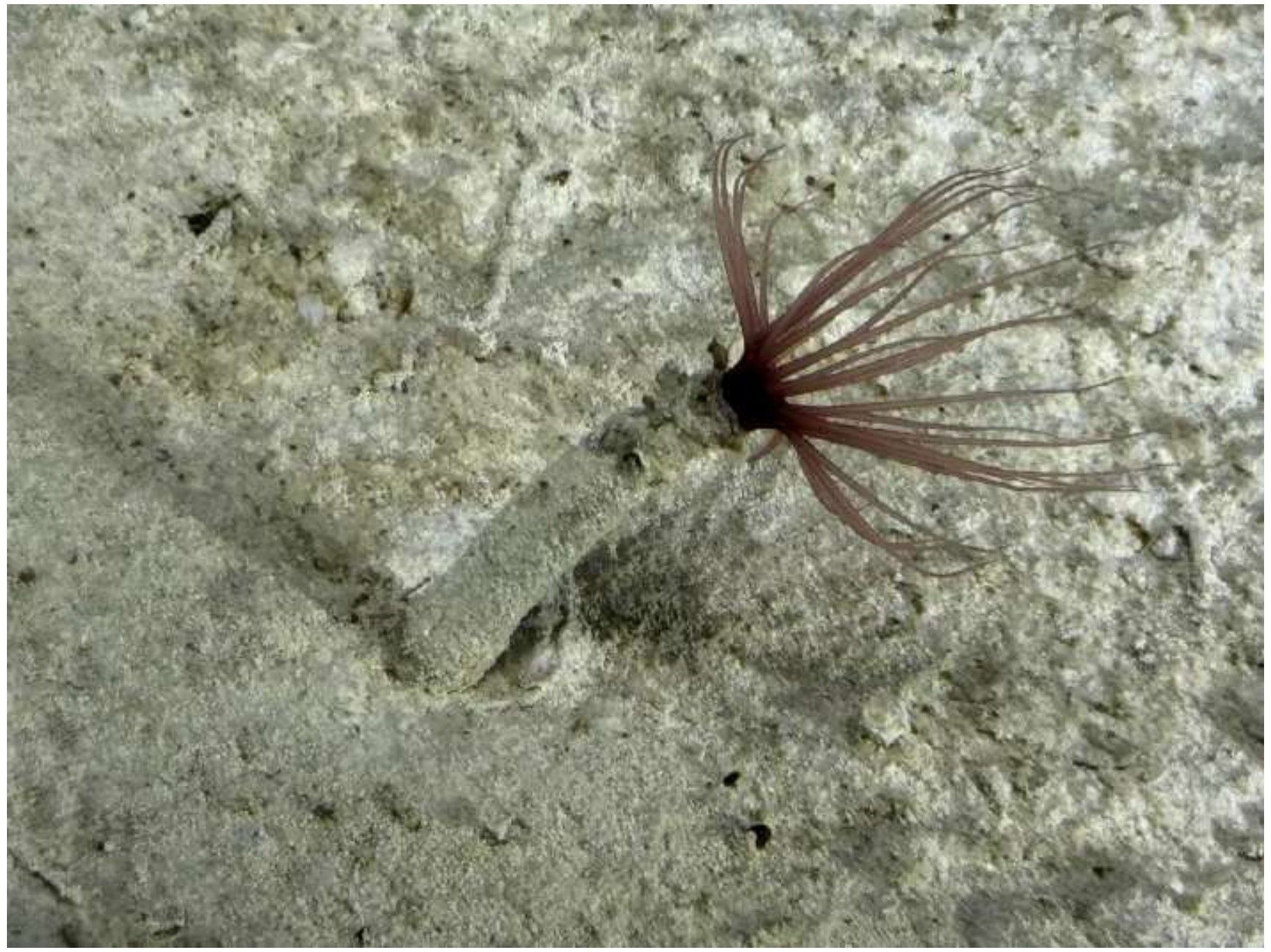

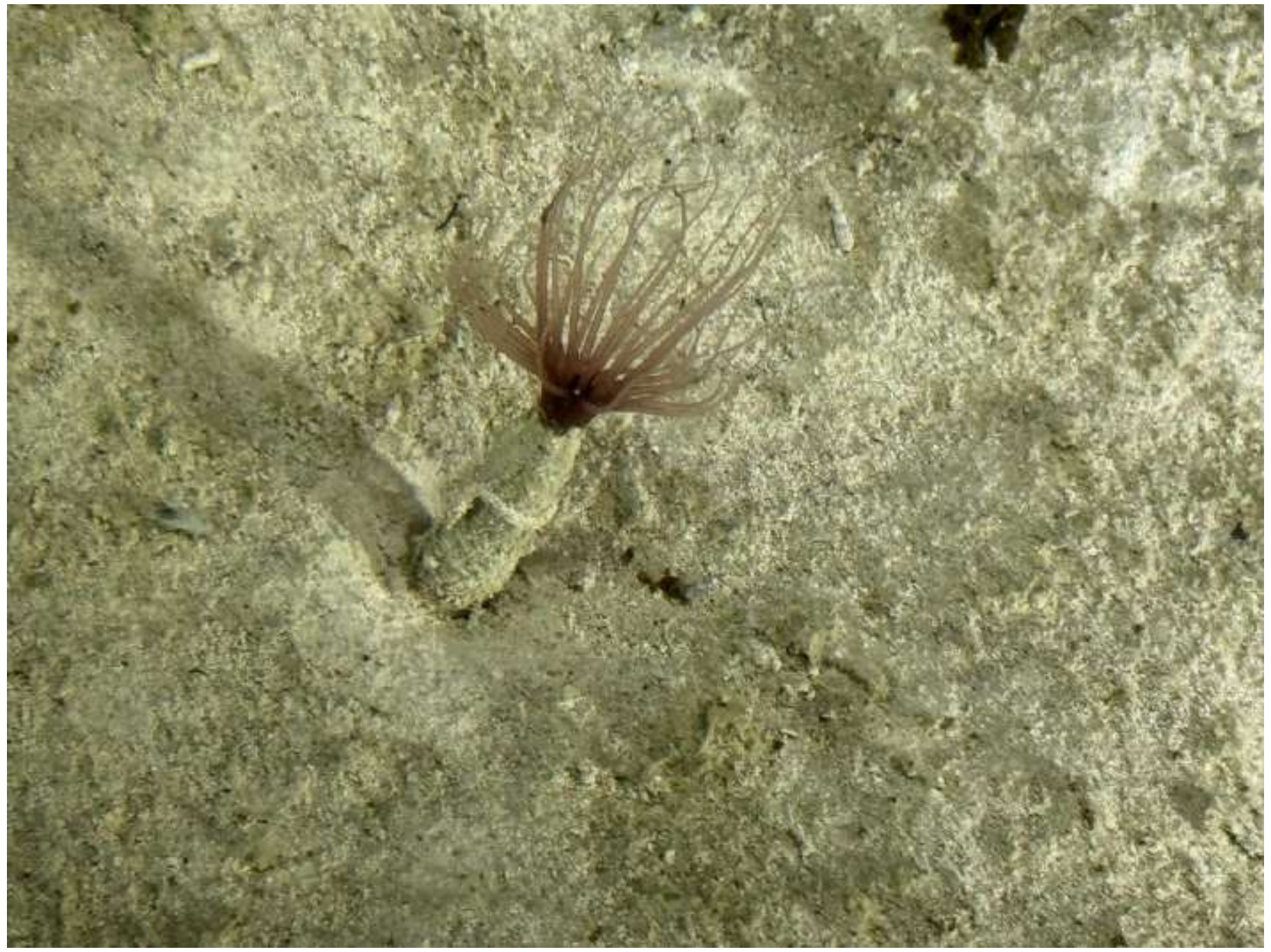

Found on soft sediments. Dark reddish-brown medium tentacles; up to 26. Column within sediment coated tube. Oral disc same colour as tentacles.

Hexacorallia; Ceriantharia; Ceriantharia fam. indet. 2

NC4_BK2_4700_NOR_0061

NC4_BK2_4700_IMG_4981

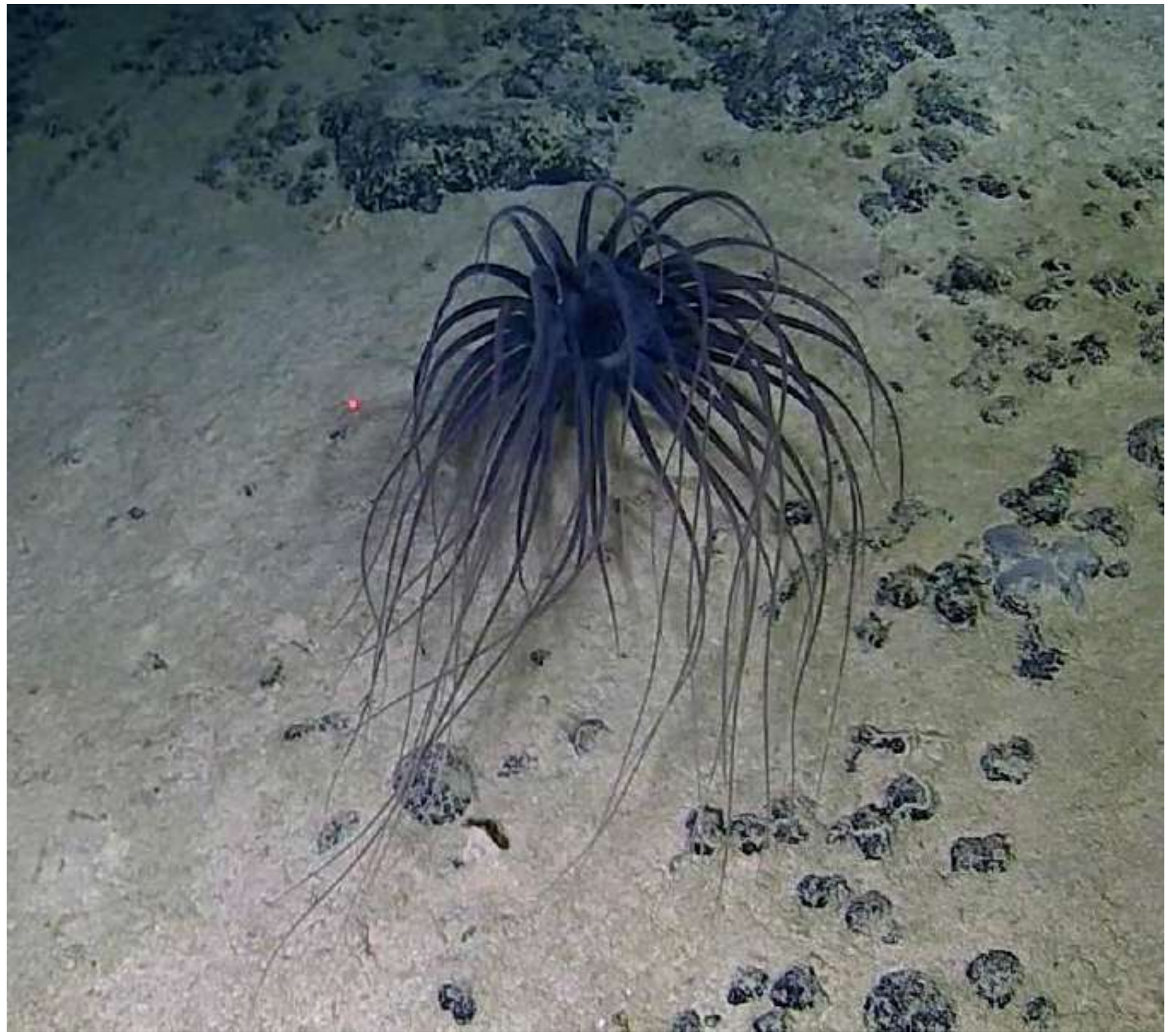

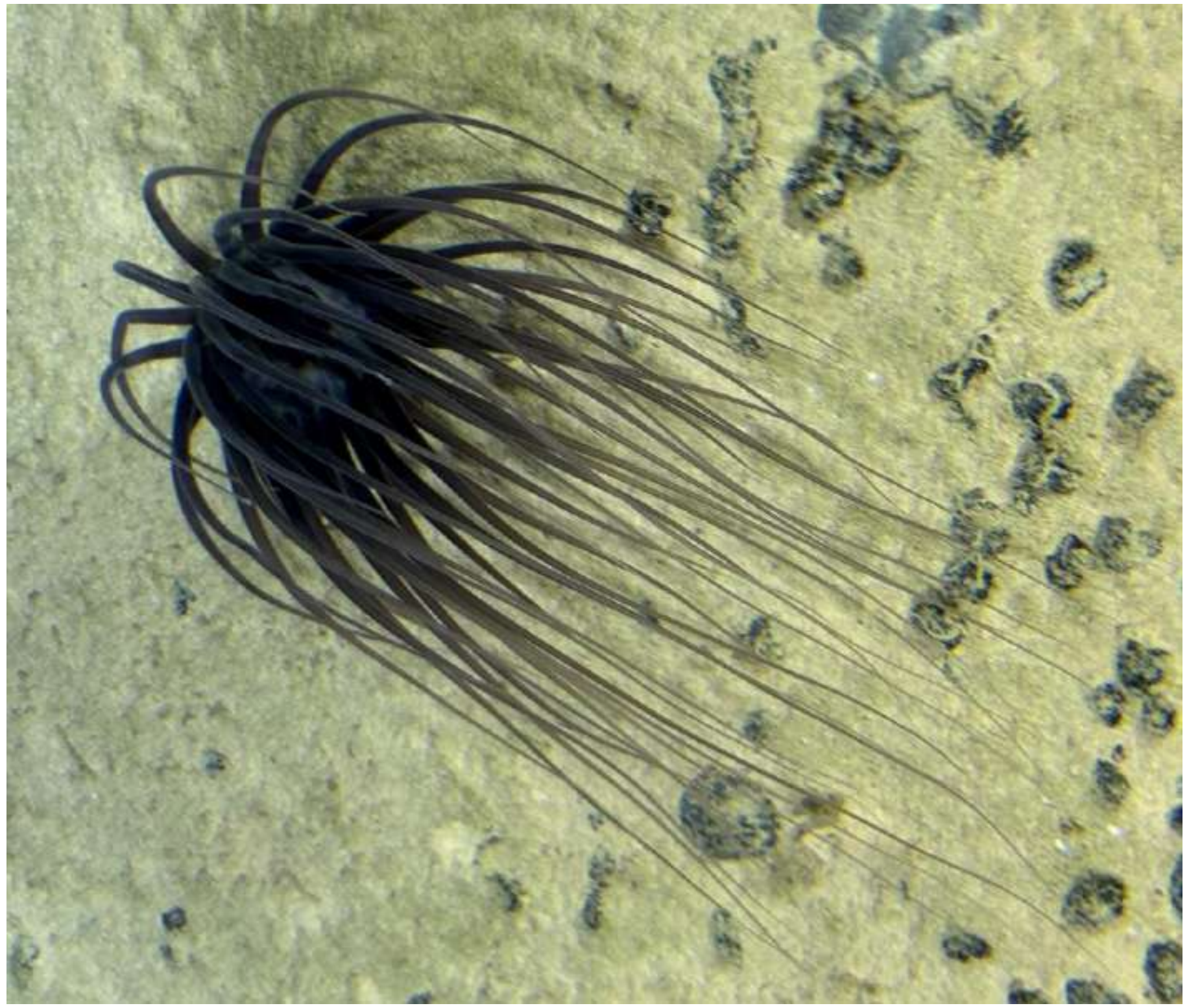

Found on soft sediments. Dark purple colouration with multiple, long tentacles. Oral disc same colour as tentacles. Cf. Relicanthus daphneae

Hexacorallia; Corallimorpharia; Corallimorphidae; Corallimorphidae gen. inc. 1

NC1_BK2_6700_IMG_4972

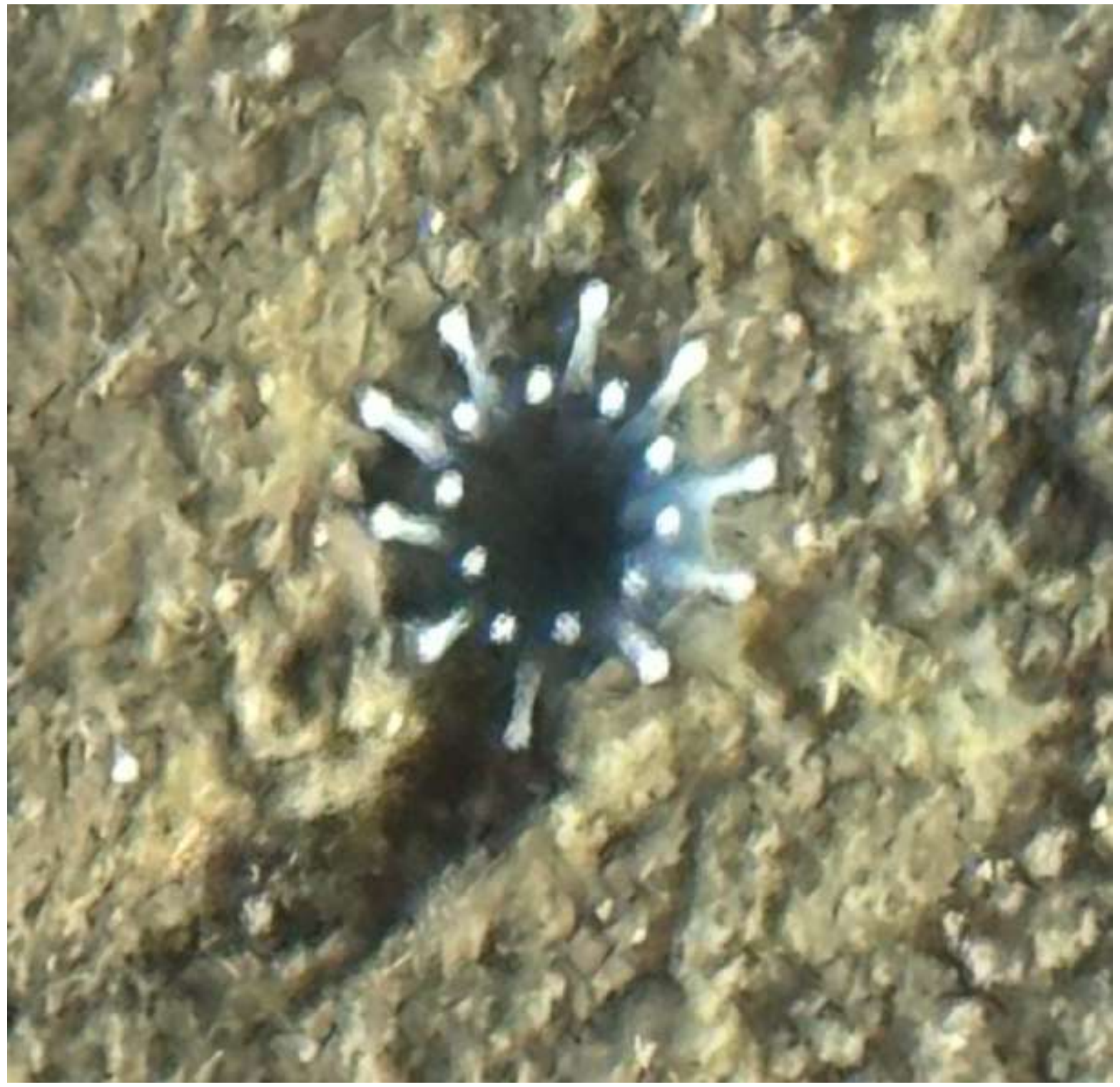

Found on both soft sediments. Dark blue with short tentacles; 2 rows of up to 20. Column is fleshy, cup-shaped and embedded within the sediment. Oral disc is concave.

NC4_BK4_4700_NOR_0052

Hexacorallia; Corallimorpharia; Corallimorphidae; Corallimorphidae gen. inc. 2

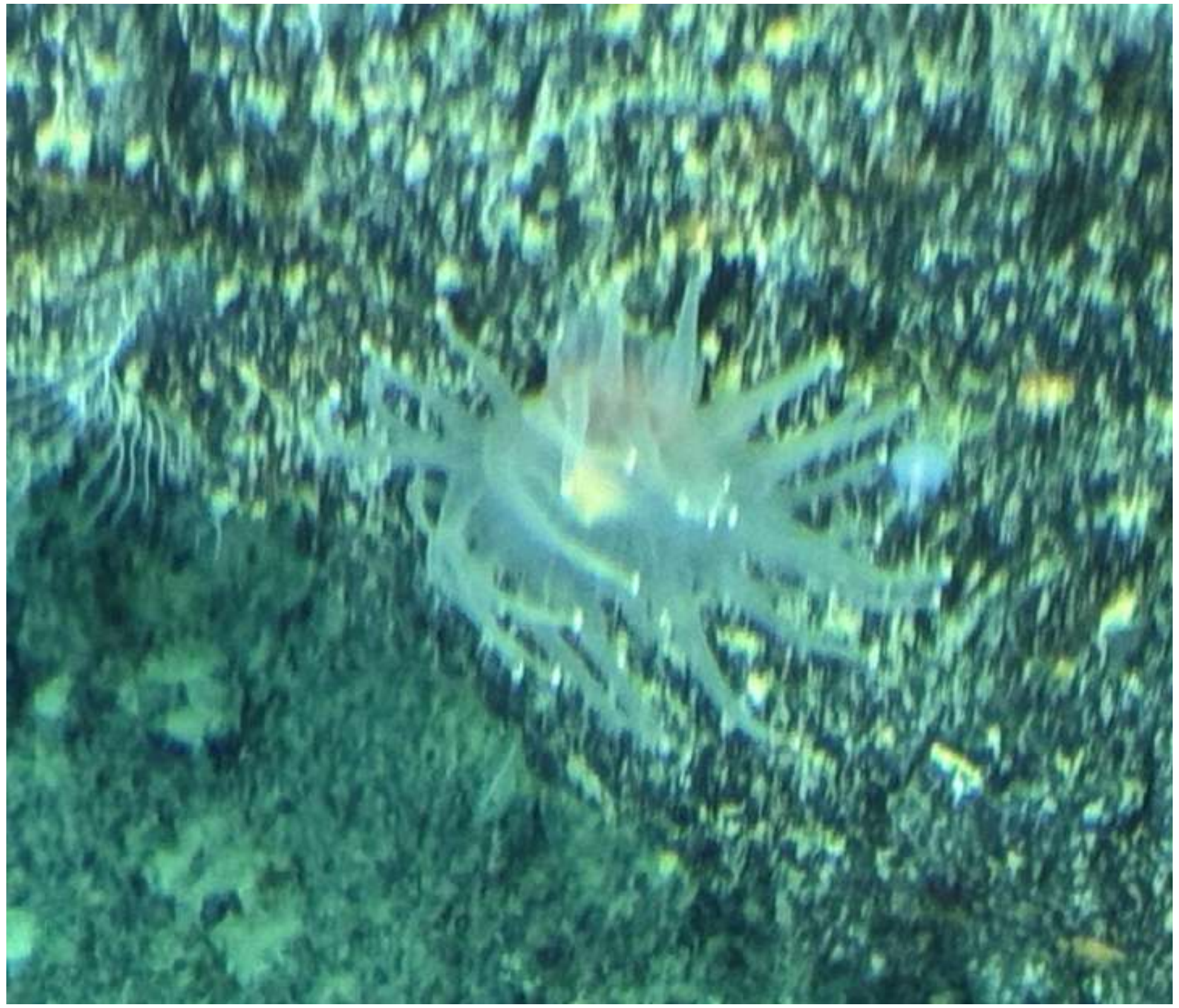

Found on hard substrate. Really translucent, colored tips at end of tentacles. Stalk also translucent.

Hydrozoa; Anthoathecata; Candelabridae; Candelabridae gen. inc. 1

NC4_BK3_6900_NOR_0079

NC4_BK3_6900_IMG_5793

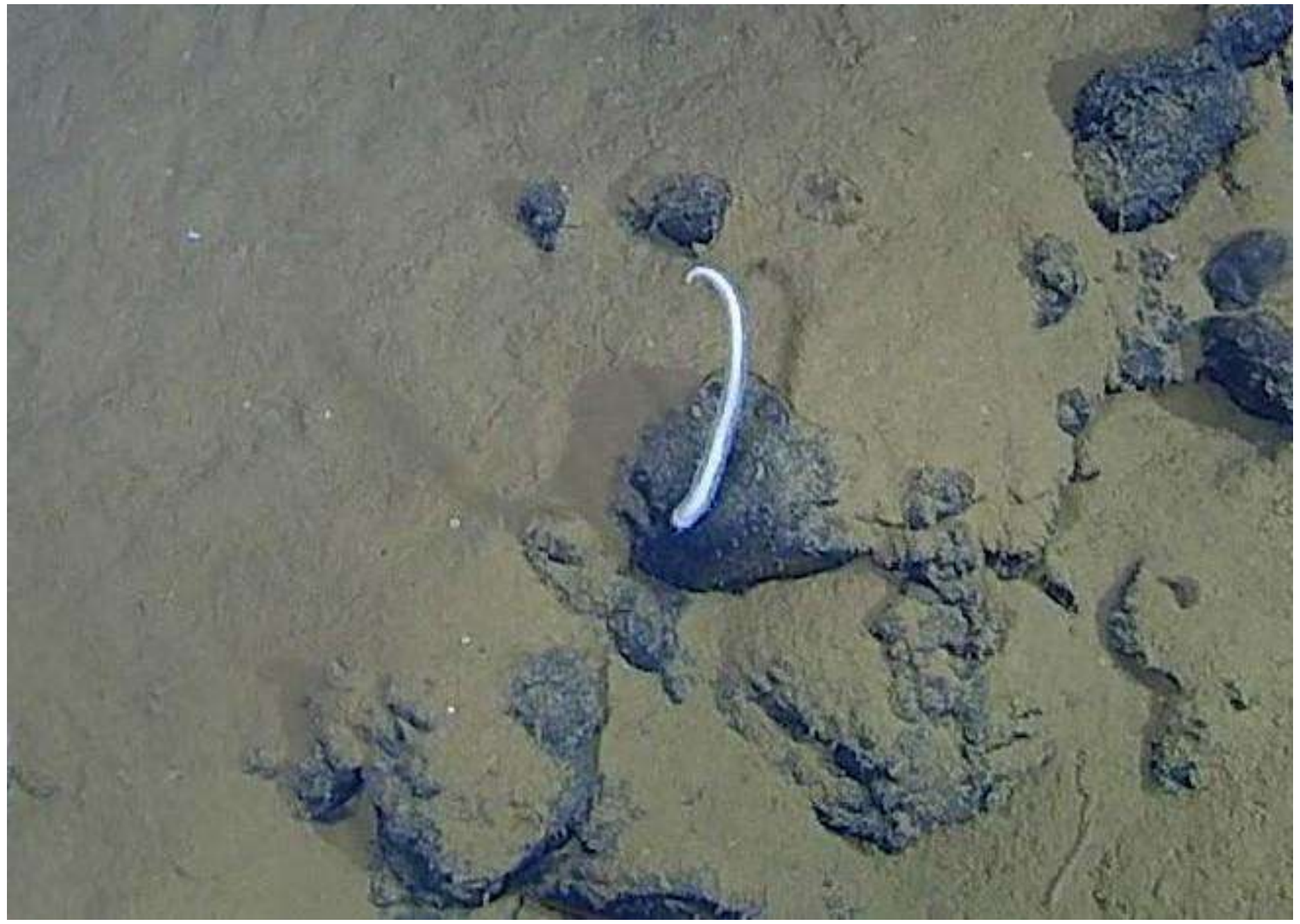

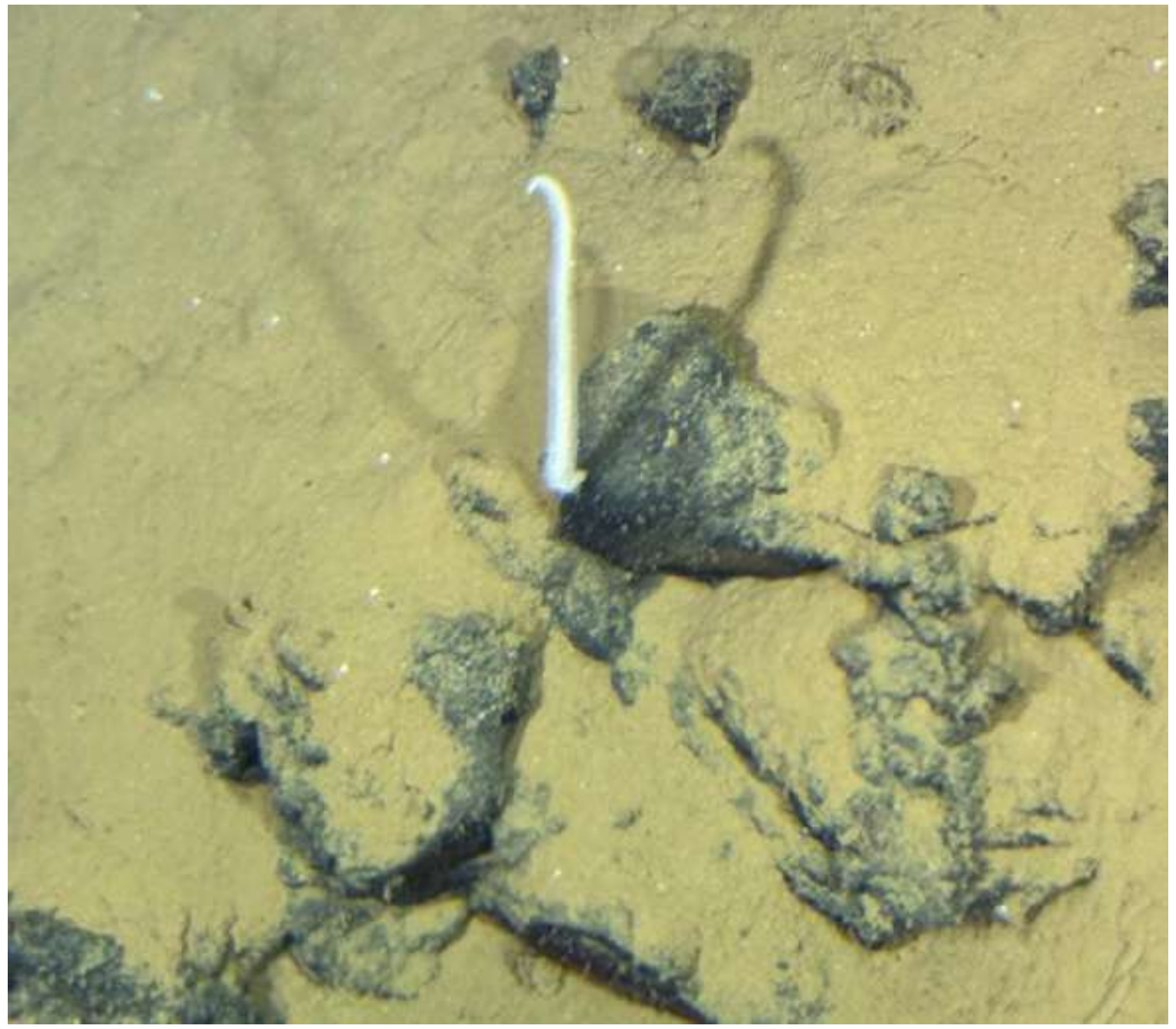

Attaches to hard substrates. Elongated, curved, white hydranth covered in transluscent, capitate tentacles

Hydrozoa;
Hydrozoa ord. inc. 1

NC2_BK2_3800_IMG_7877
NC2_BK1_3200_IMG_6962
NC2_BK5_4600_IMG_9934

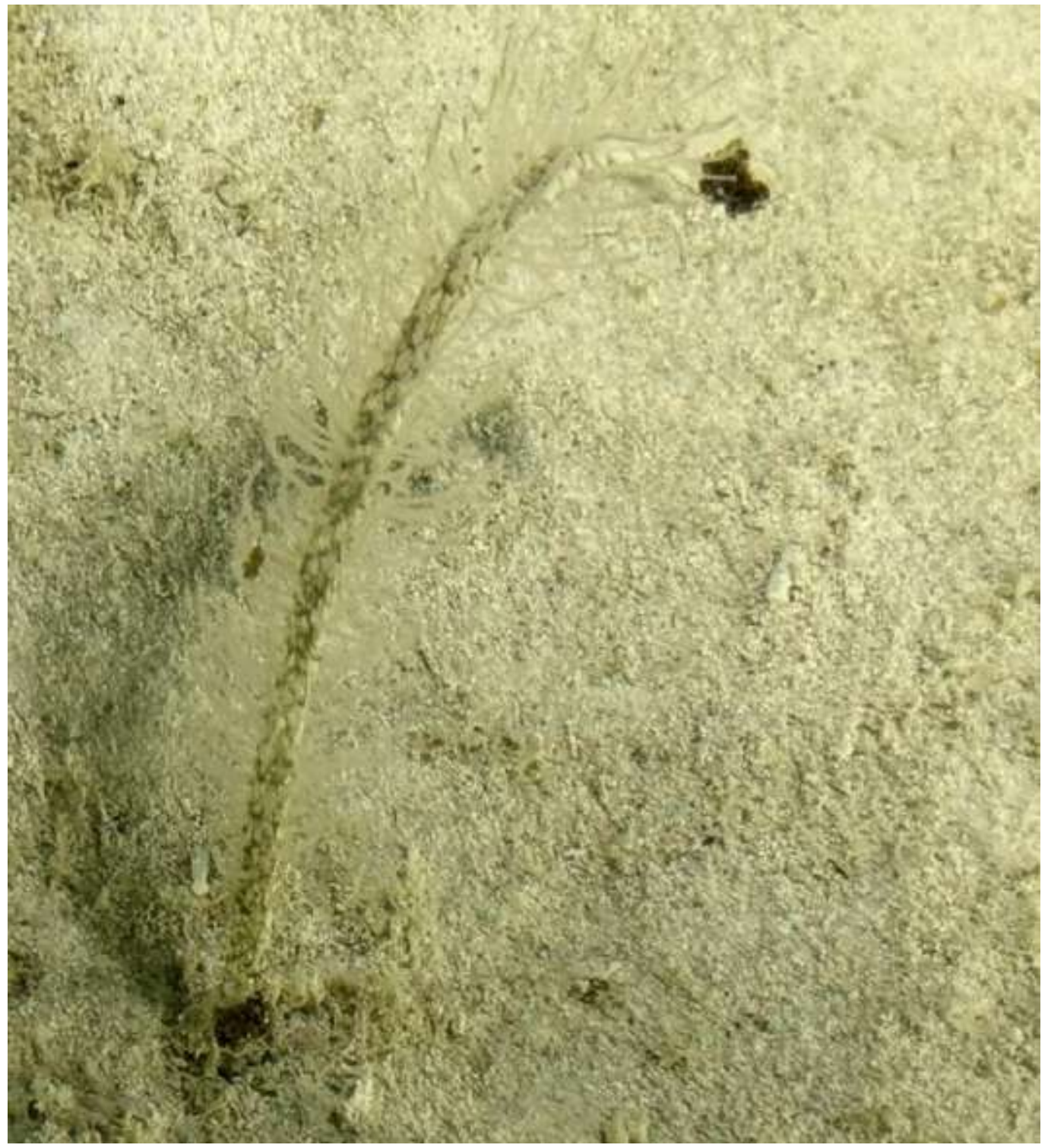

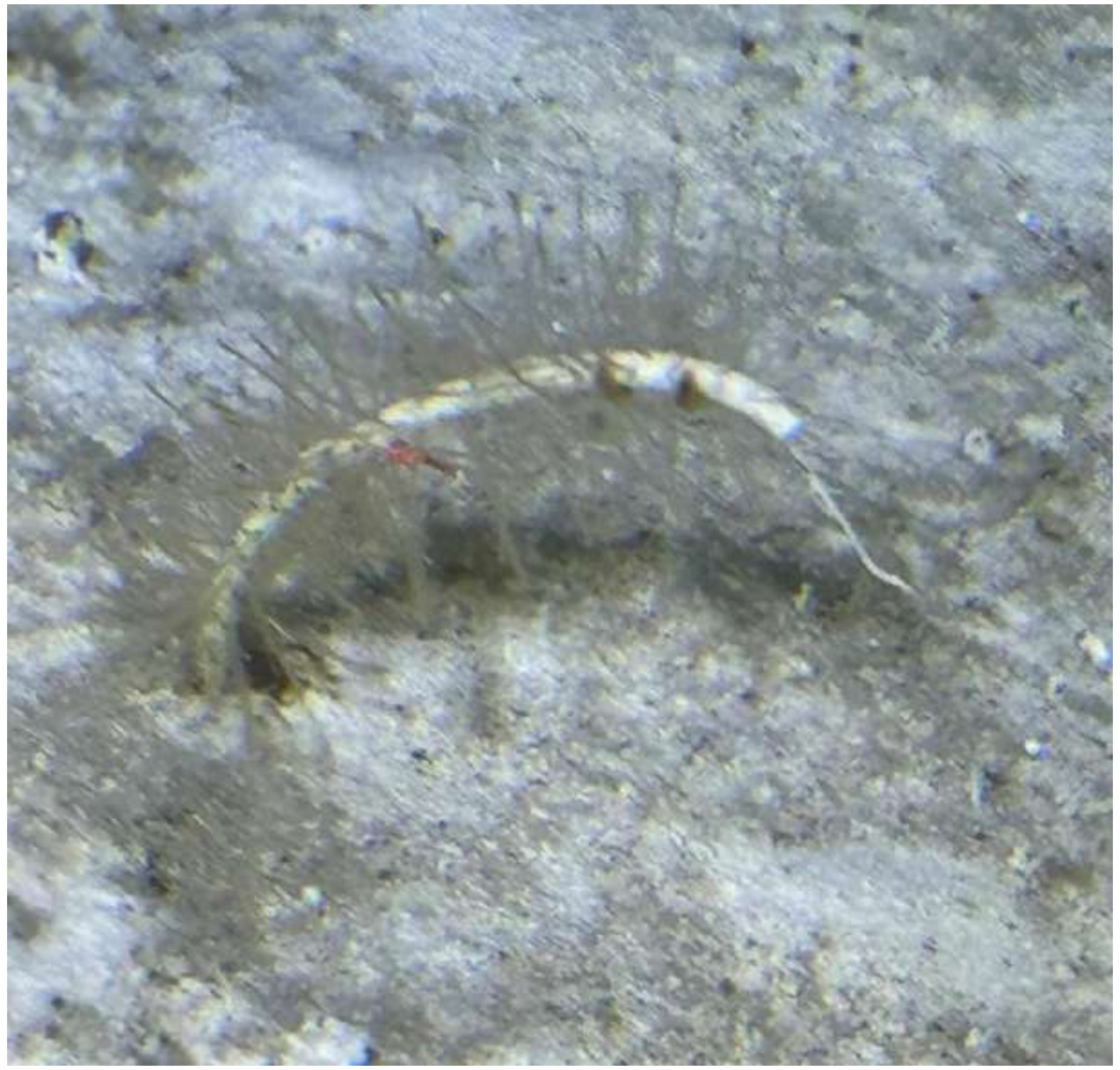

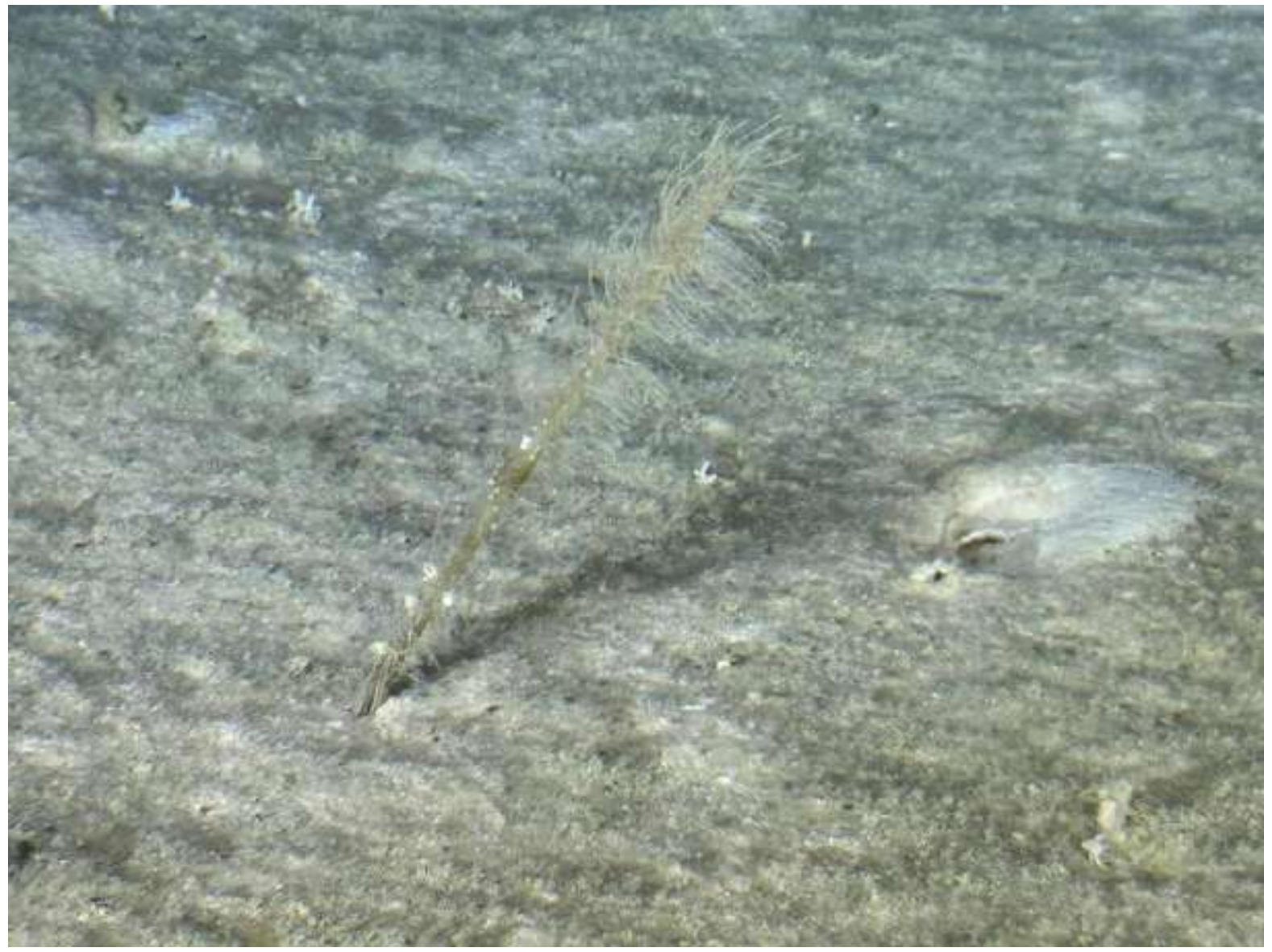

Attaches to organic surfaces including sabellid tubes. Thin, translucent tentacles extending from attachment to sabellid tube; hydrozoans on sabellid tube. Could be Anthoathecata: Hydractiniidae

Hydrozoa;
Hydrozoa ord. inc. 2

NC2_BK2_3800_IMG_8290

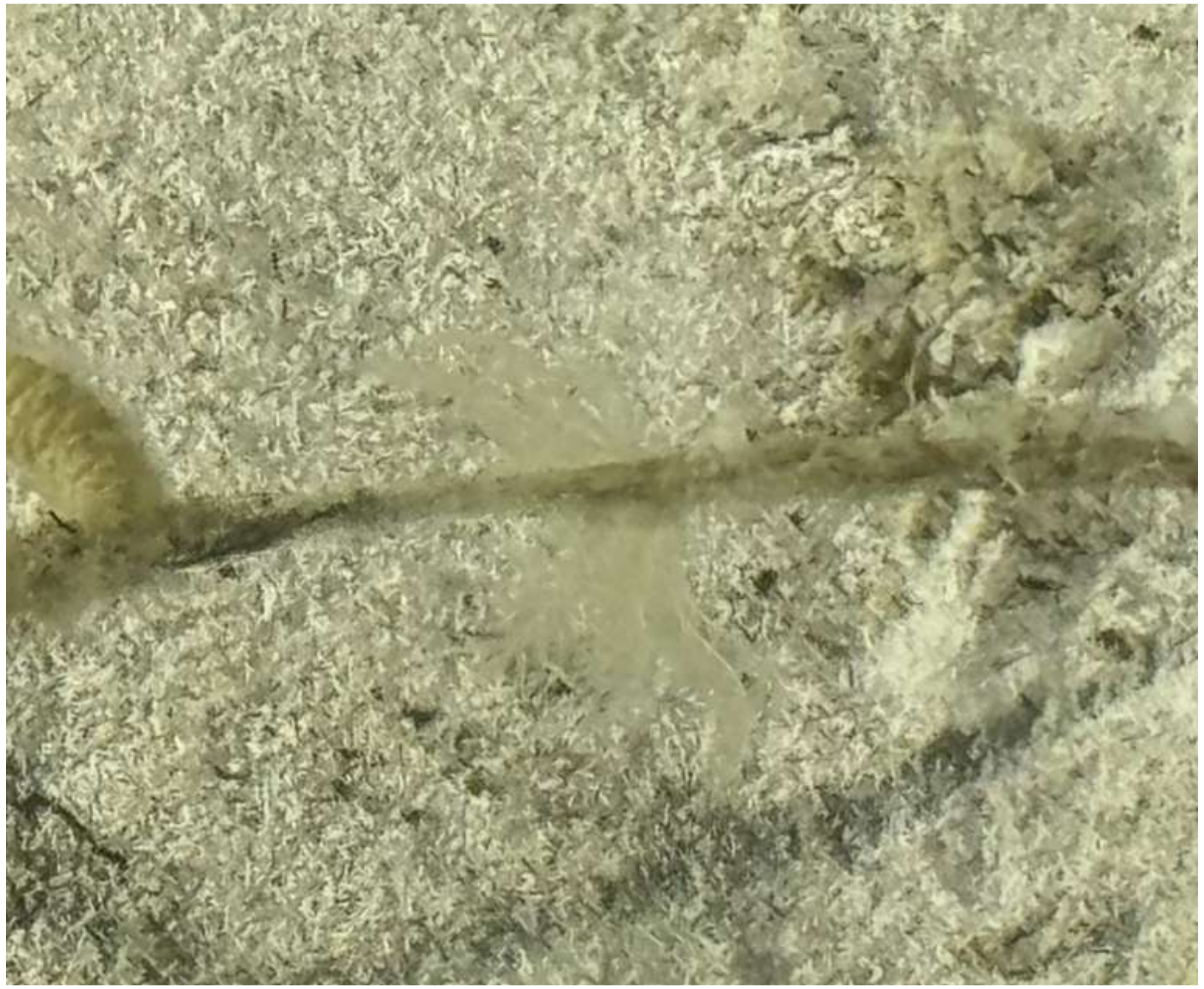

Attaches to organic surfaces including hexactinellid stalks. Fan-like, translucent growth pattern ; hydrozoans growing on hexactinellid stalk

Hydrozoa; Anthoathecata; Corymorphidae; *Branchioceriantus* sp. inc. 1

NC3_BK1_8000_IMG_1182
NC3_BK1_8000_IMG_1149
NC4_BK3_6900

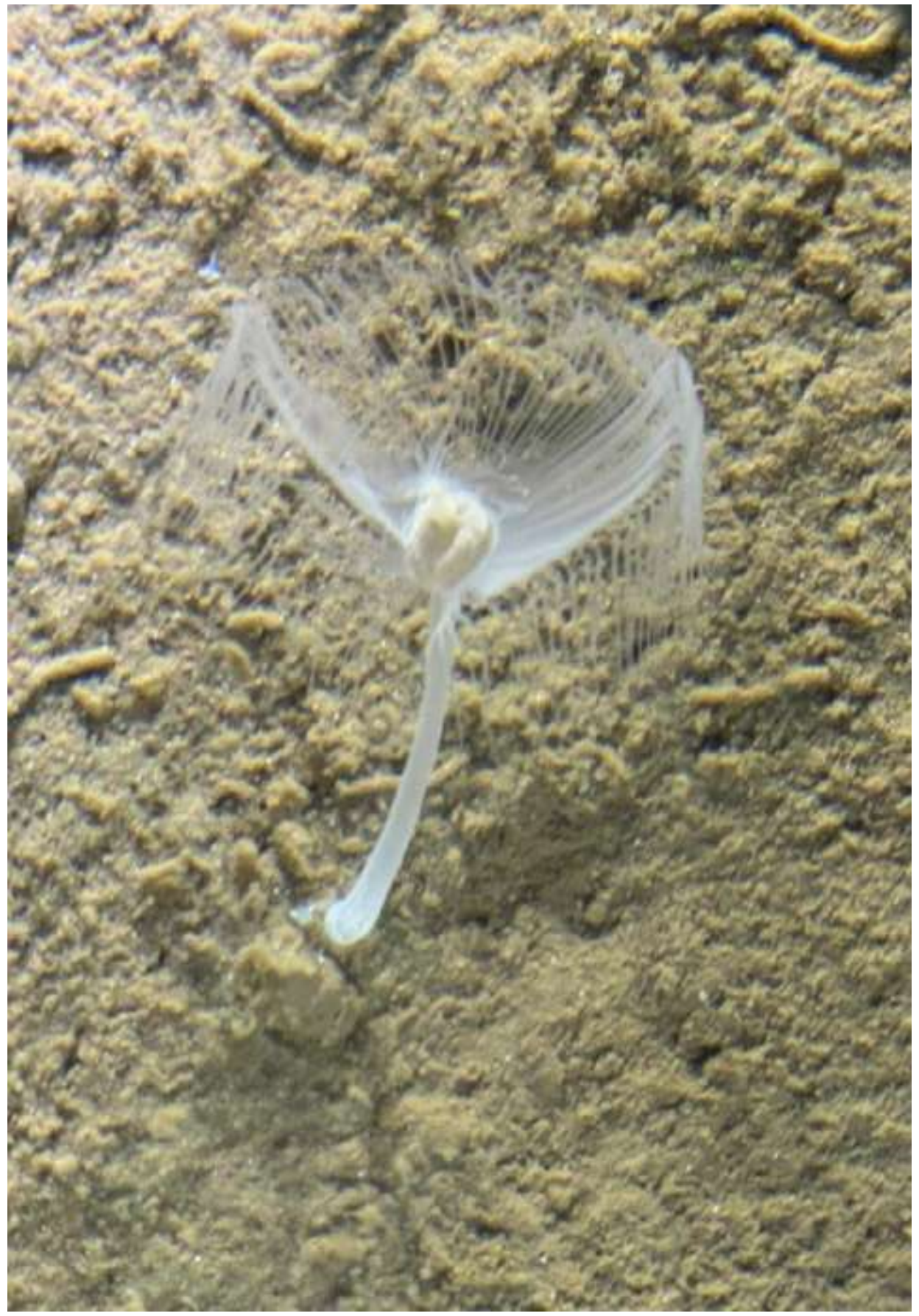
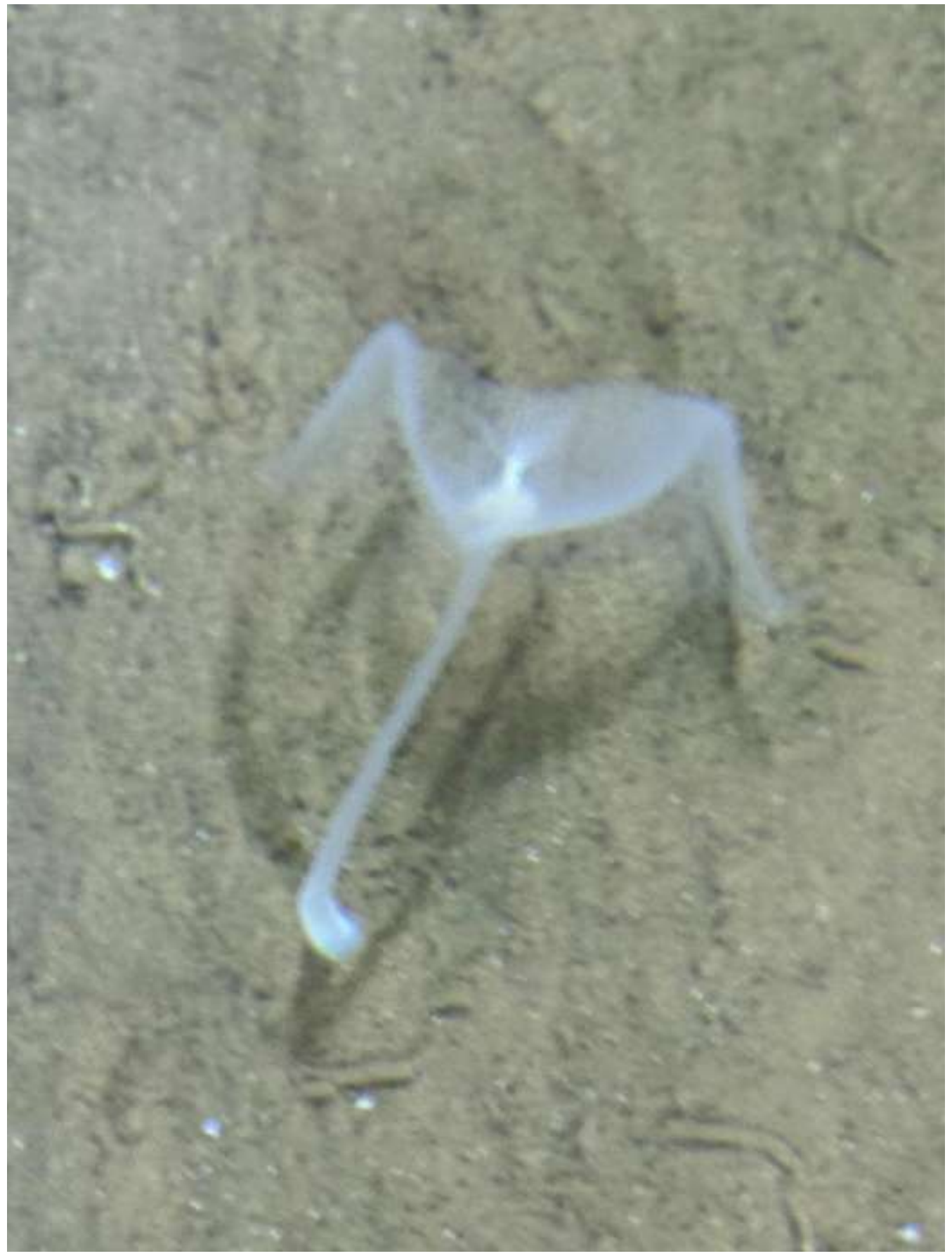
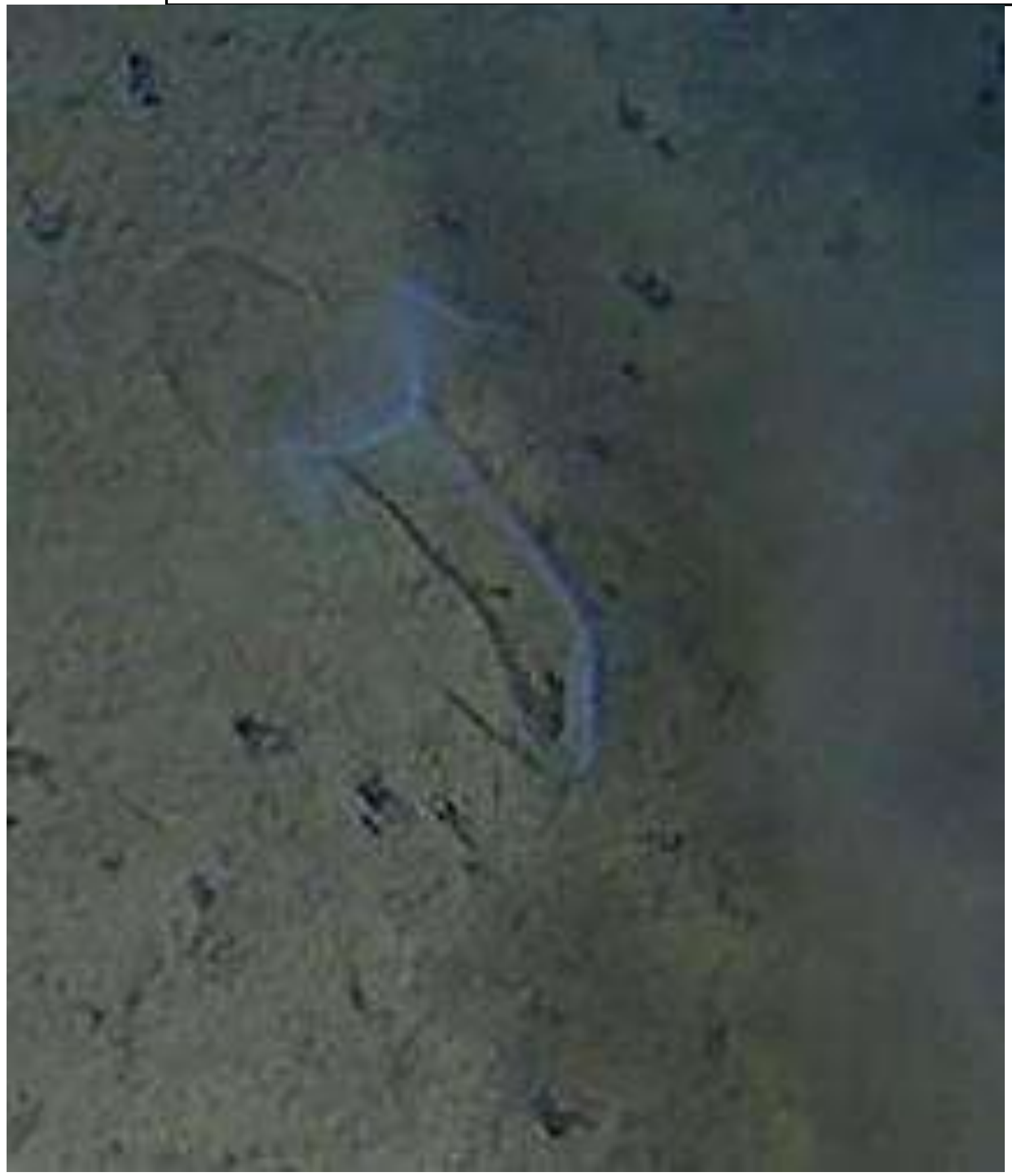

Attaches to soft sediment. Wineglass-shaped, translucent growth pattern.

Hydrozoa; Anthoathecata; Corymorphidae; *Branchiocerianthus* sp. inc. 2

NC4_BK3_6900

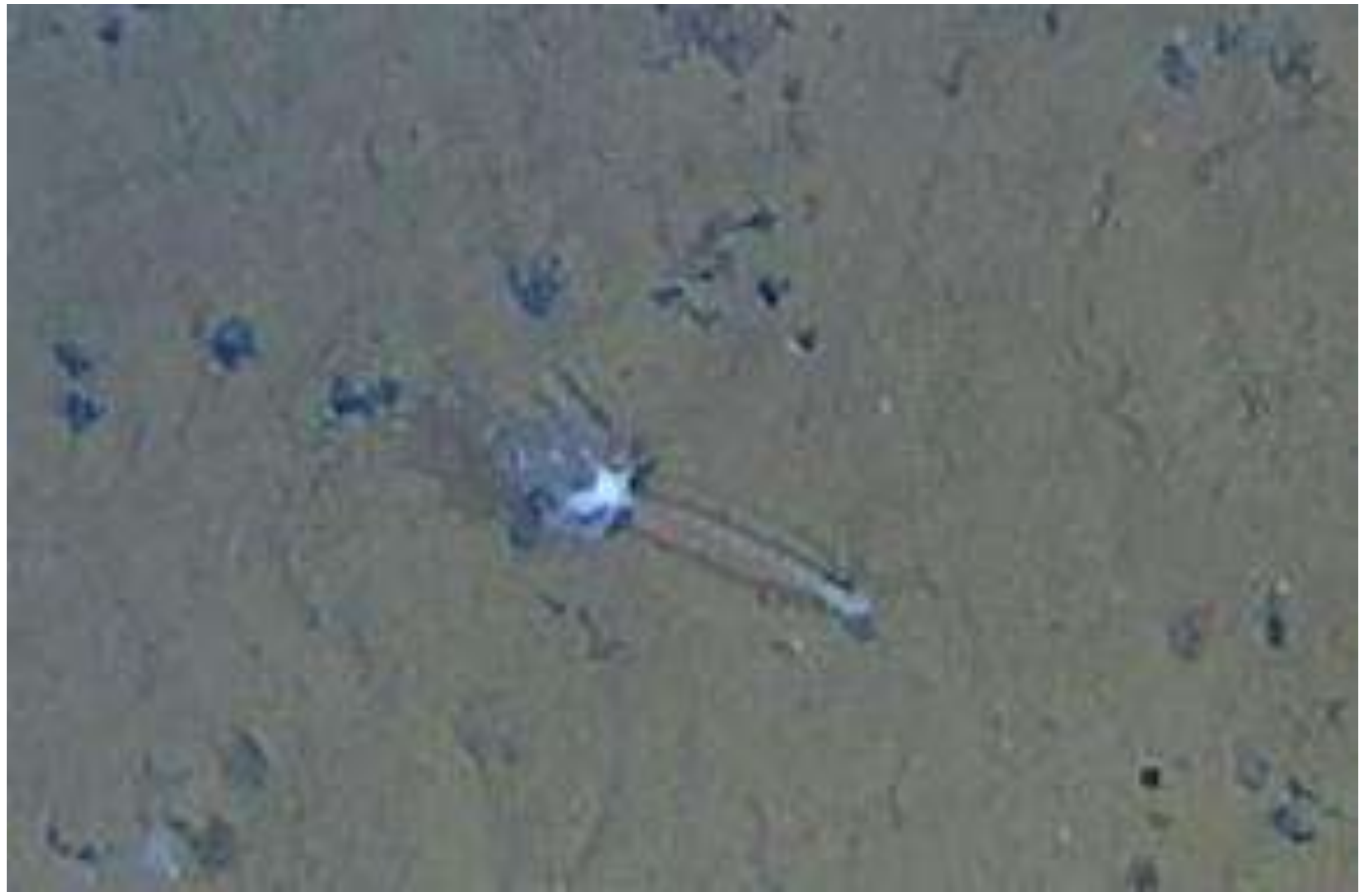

Dark stalk, white tentacles. Similar to branchiocerianthus in jpt dataset

Octocorallia; Scleralcyonacea; Pennatuloidea; Kophobelemnidae; *Kophobelemnon* gen. indet. 1

NC2_BK2_3800_IMG_8115
NC2_BK2_3800_IMG_8117

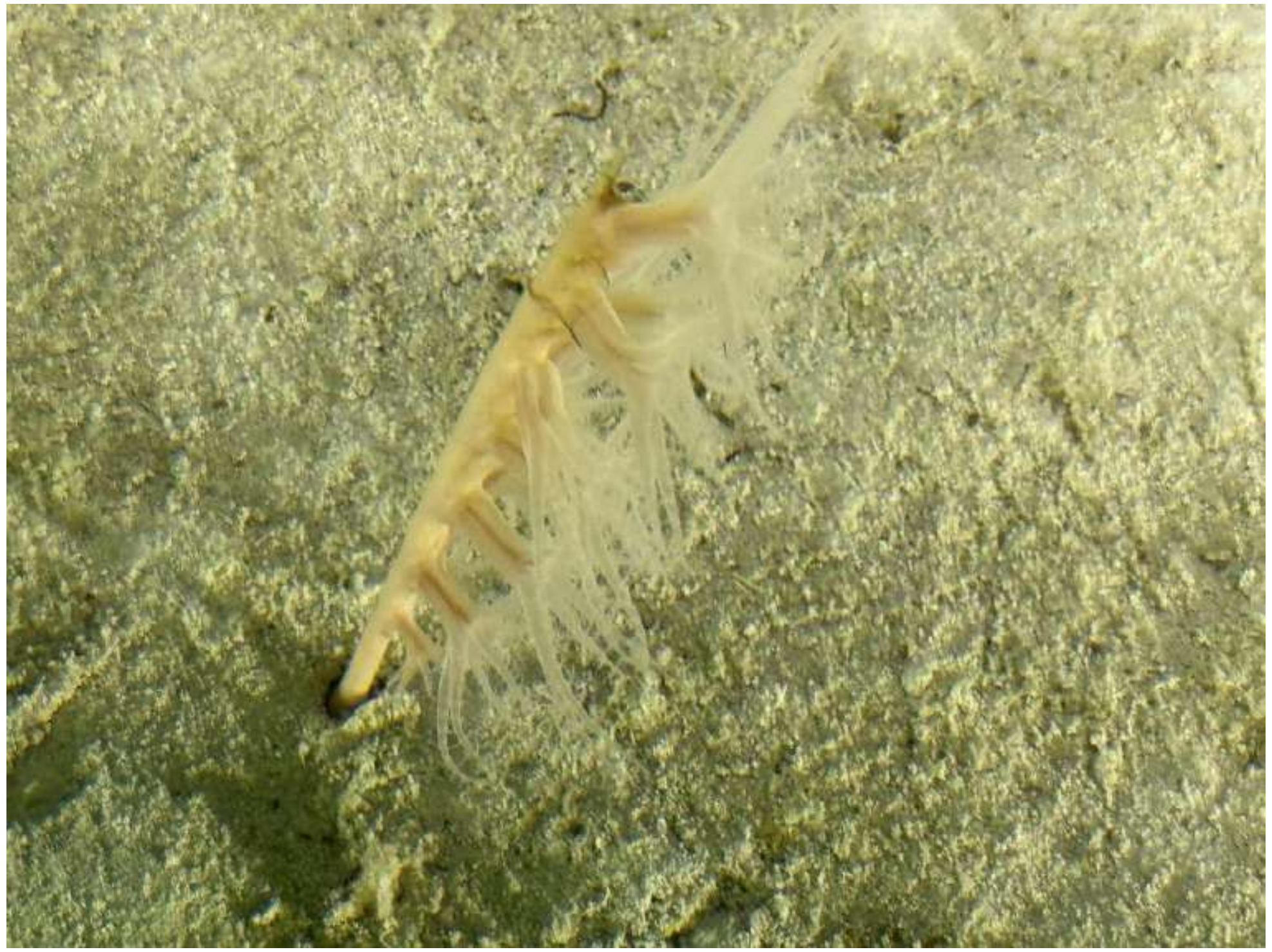
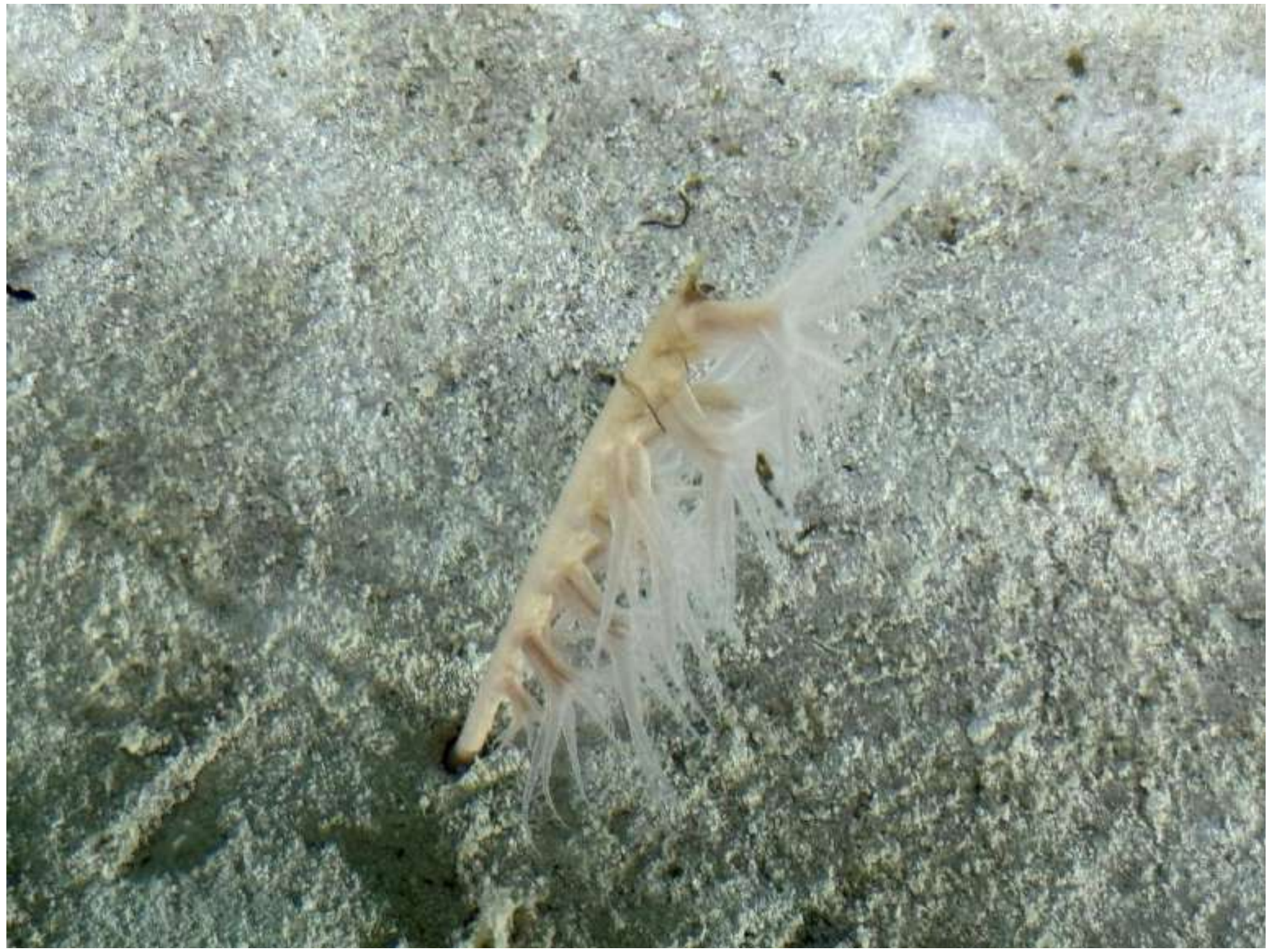

Attaches to soft sediments. Umbellulidae-like polyps grow unidirectionally and increase in size distally from the attachment.

Octocorallia; Scleralcyonacea; Umbellulidae; Umbellula; Umbellula sp. indet. 1

NC2_BK1_3200_NOR_0057
NC2_BK1_3200_IMG_7719
NC2_BK2_3800_IMG_8010

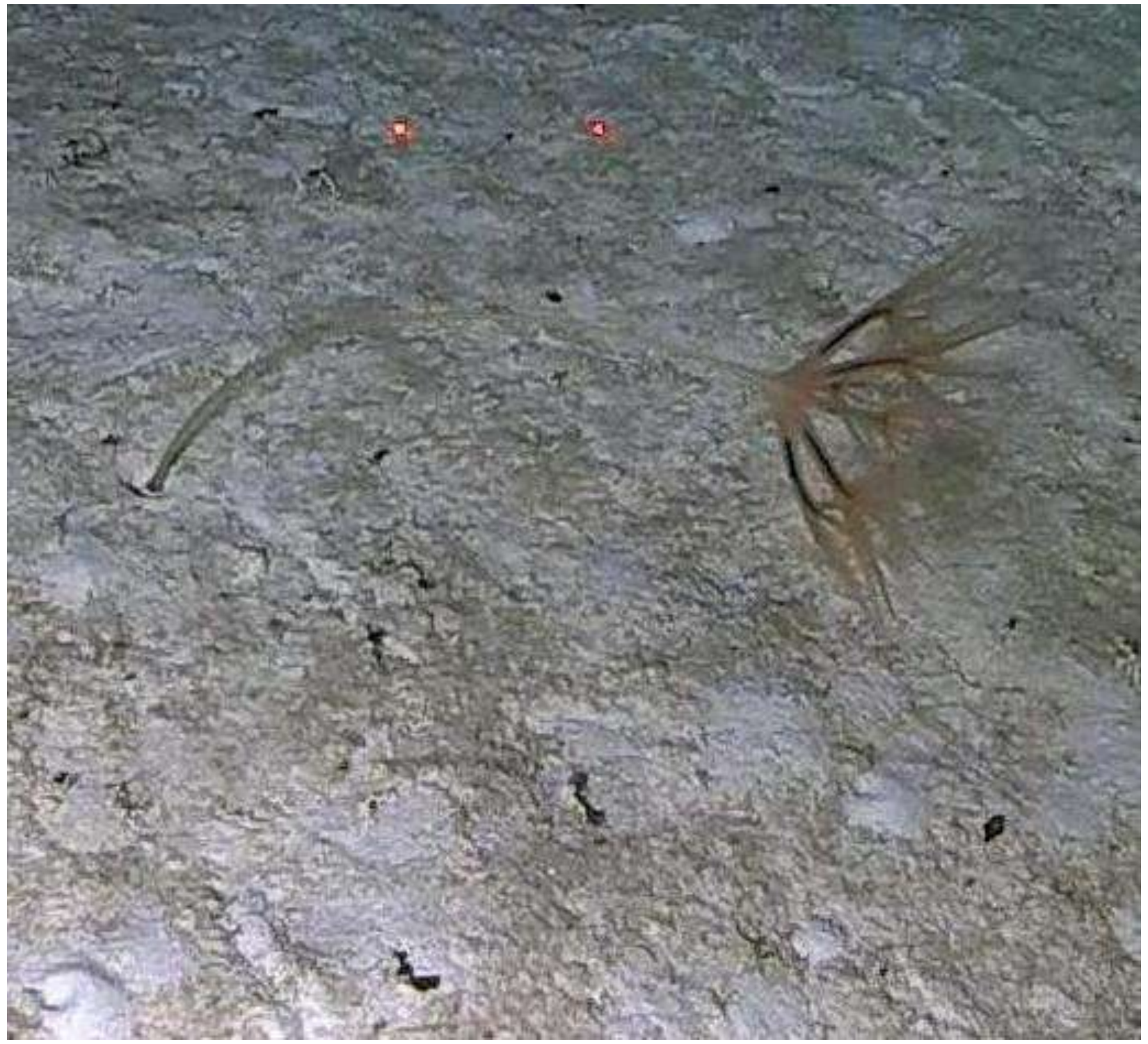

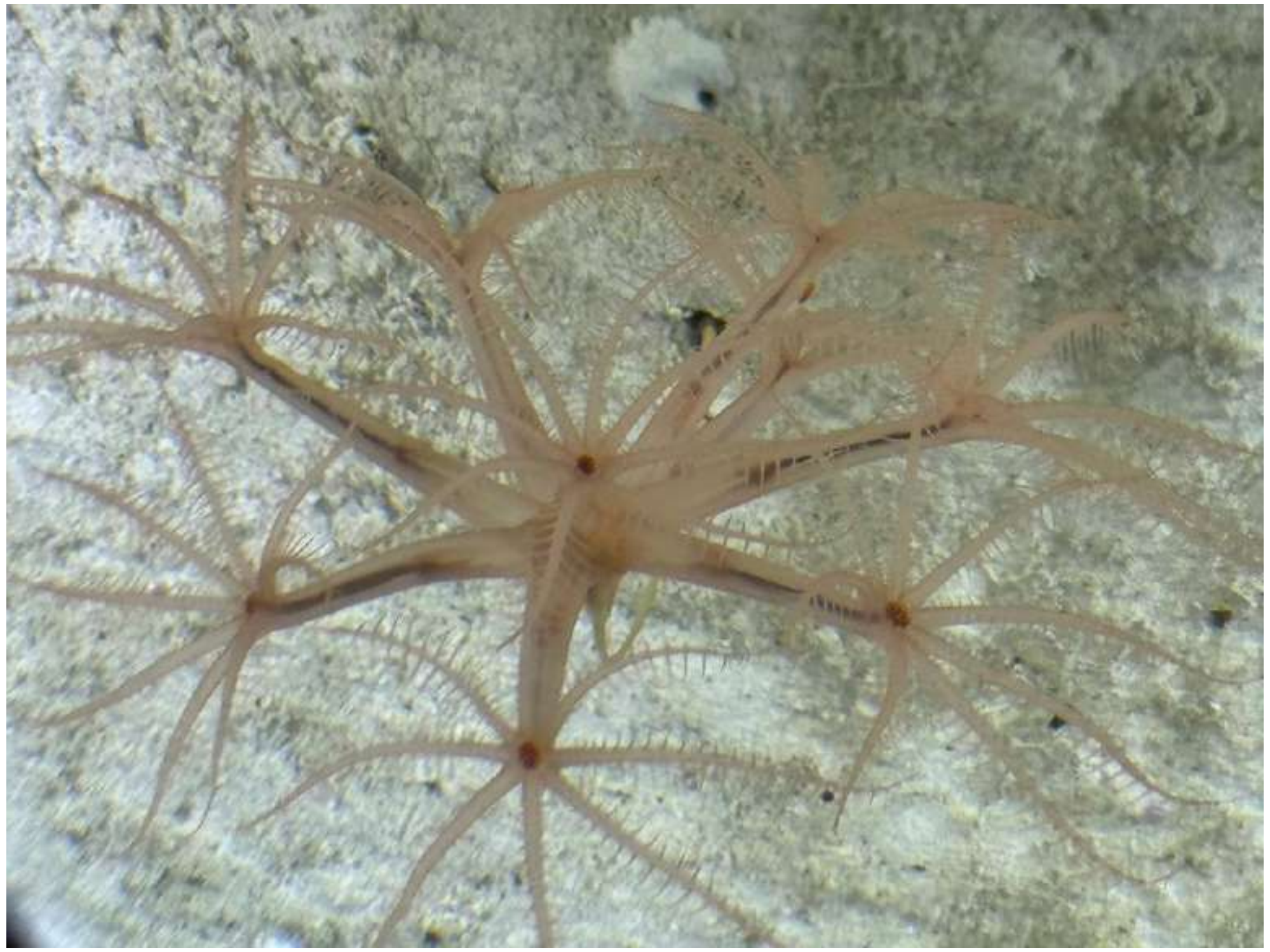

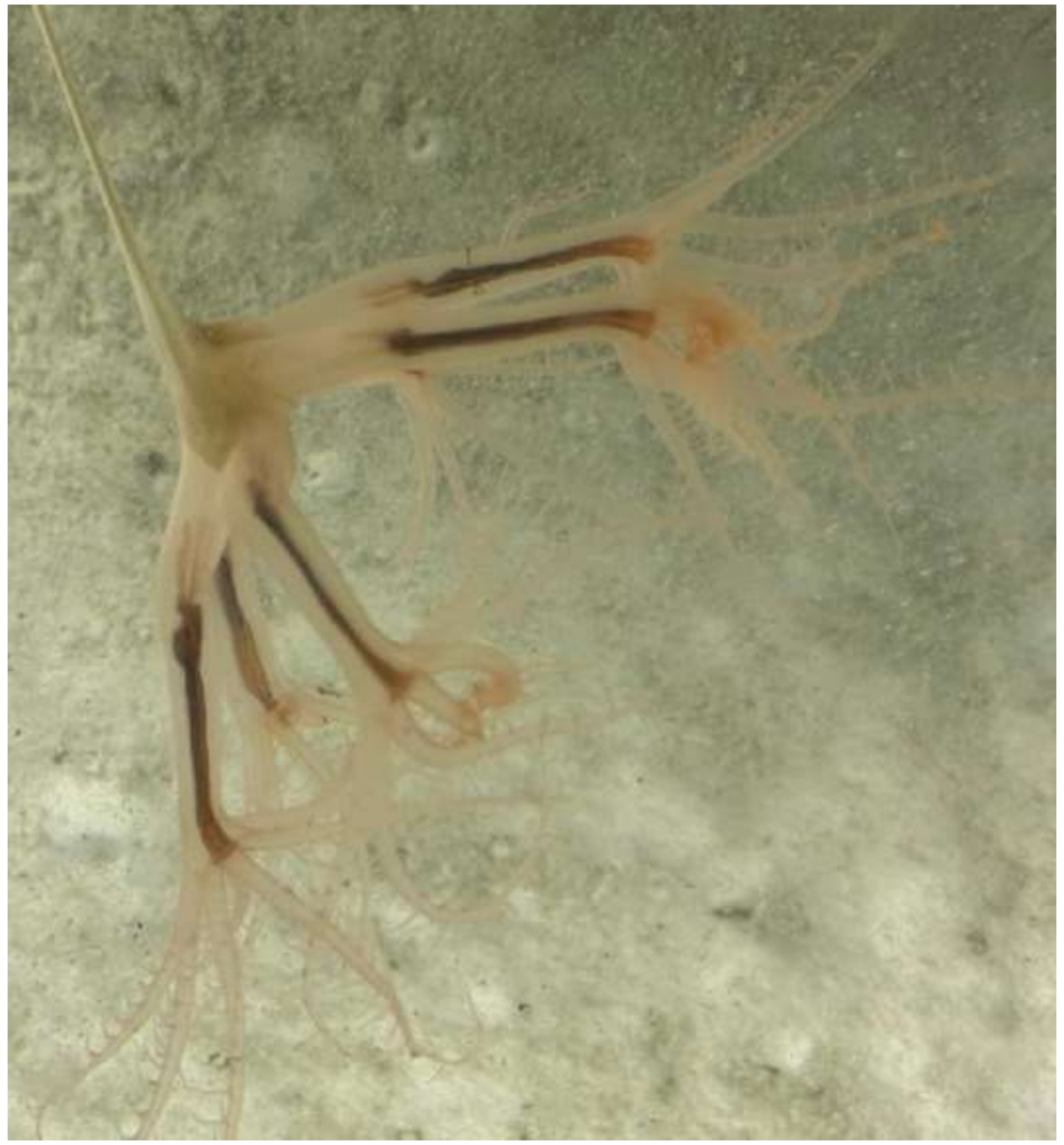

Attaches to soft substrates. Stem is long and flexible. 9 polyps grow in flower-like arrangement.

Octocorallia; Scleralcyonacea; Umbellulidae; Umbellula; Umbellula sp. indet. 2

NC4_BK3_6900_NOR_0056

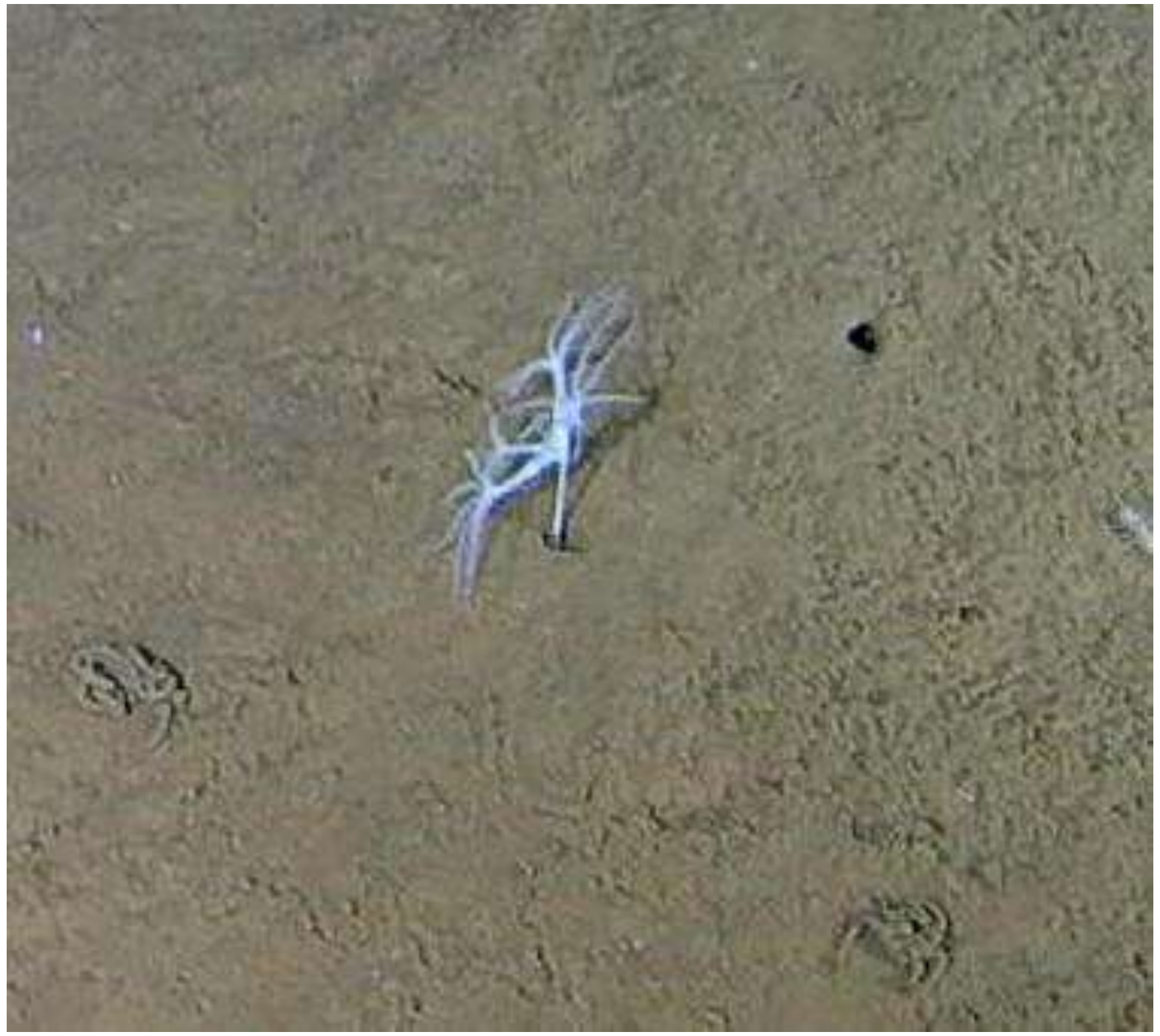

Attaches to soft substrates. Pale blue/ white colouration. Stem is short. 8-9 polyps grow in flower-like arrangement.

- Colony appears partially buried in the sediment
- Partial burial is documented in several pennatuloid taxa
- This behaviour is not commonly reported for *Umbellula*, but is plausible in this case
- A shortened rachis is considered less likely than partial burial

Octocorallia; Scleralcyonacea; Scleroptilidae; *Calibelemnon* sp. indet. 1

NC2_BK1_3200_IMG_6887
NC2_BK1_3200_IMG_6880

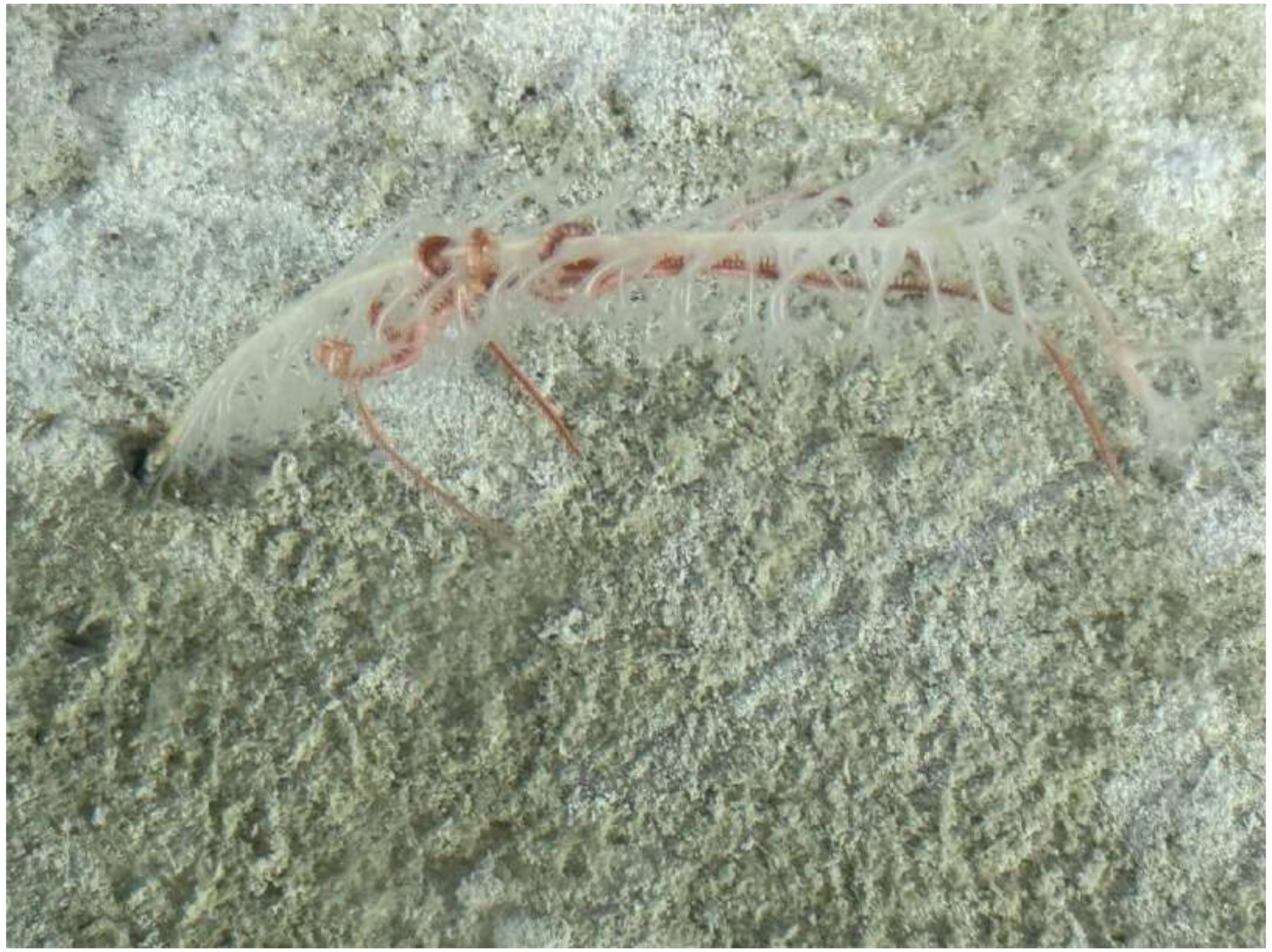

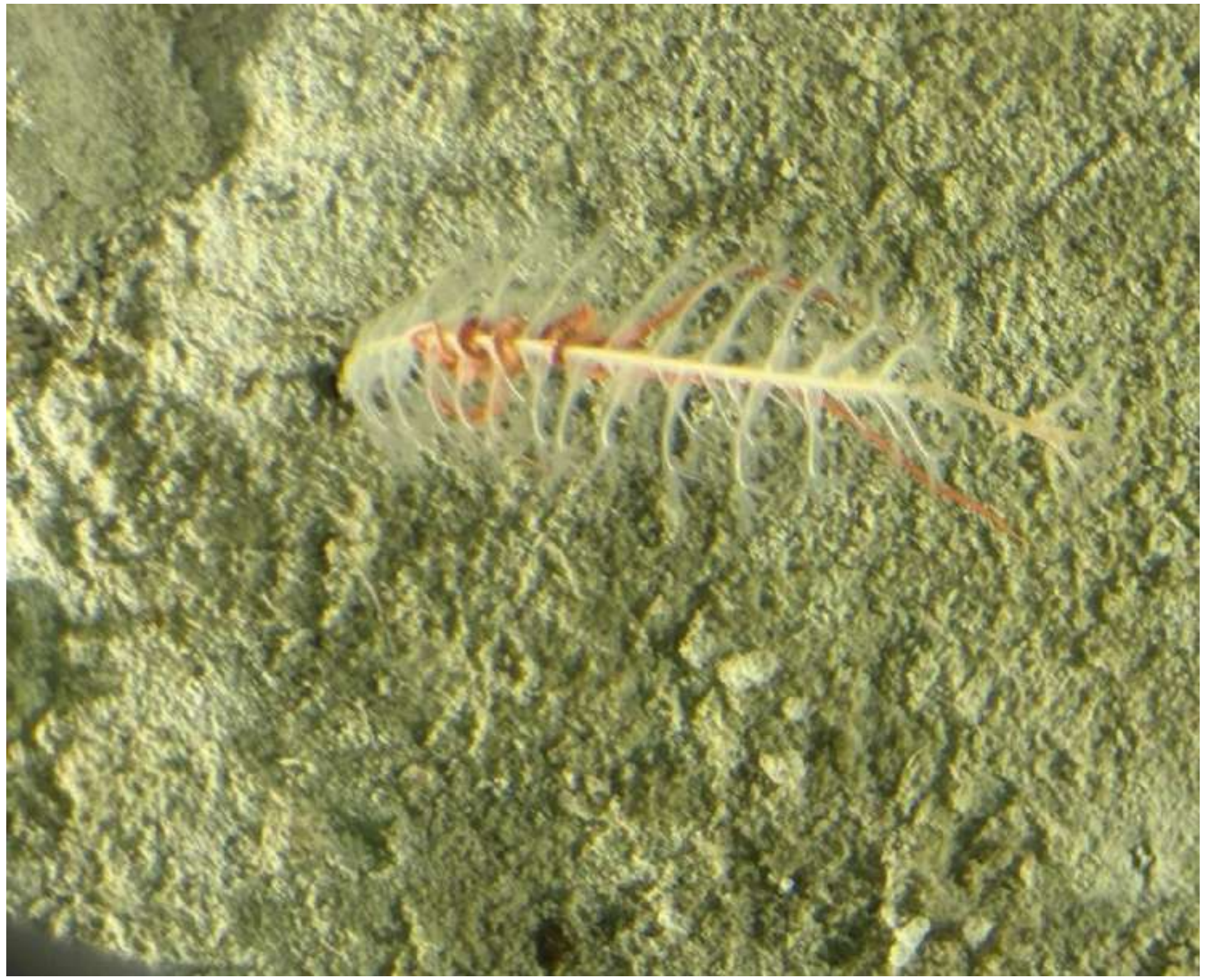

Attaches to soft substrates. Stem is long and translucent. Polyps grow laterally along the entirety of the exposed stem. Polyps shorten distally. ID confirmed by Raissa Hogan.

- Polyps arranged along the length of the rachis, more or less in pairs
- Polyps separated by distinct bare regions

Octocorallia; Scleralcyonacea; Scleroptilidae
Scleroptilum sp. indet. 1

NC2_BK1_3200_IMG_7548
NC2_BK1_3200_IMG_7555

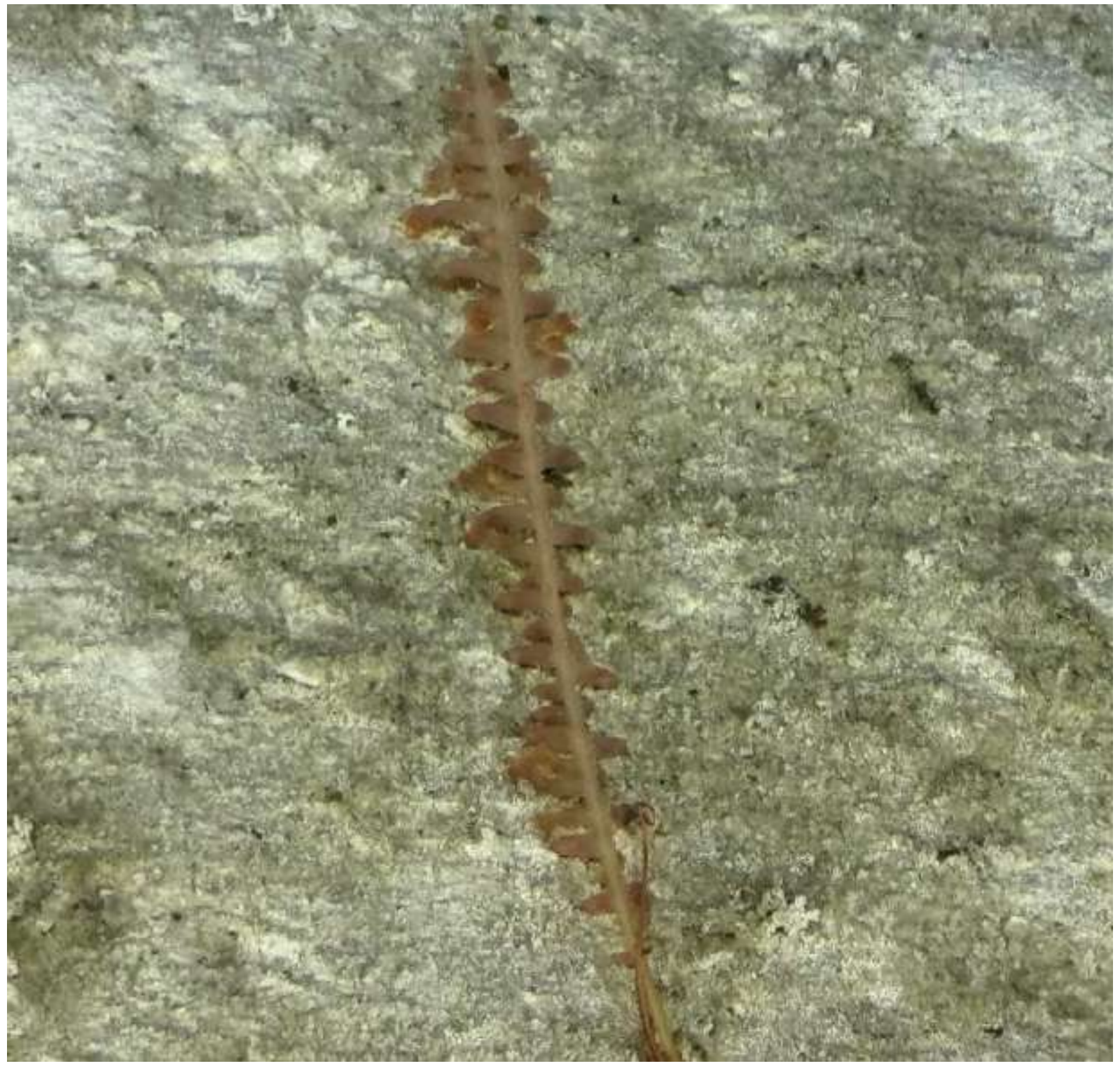

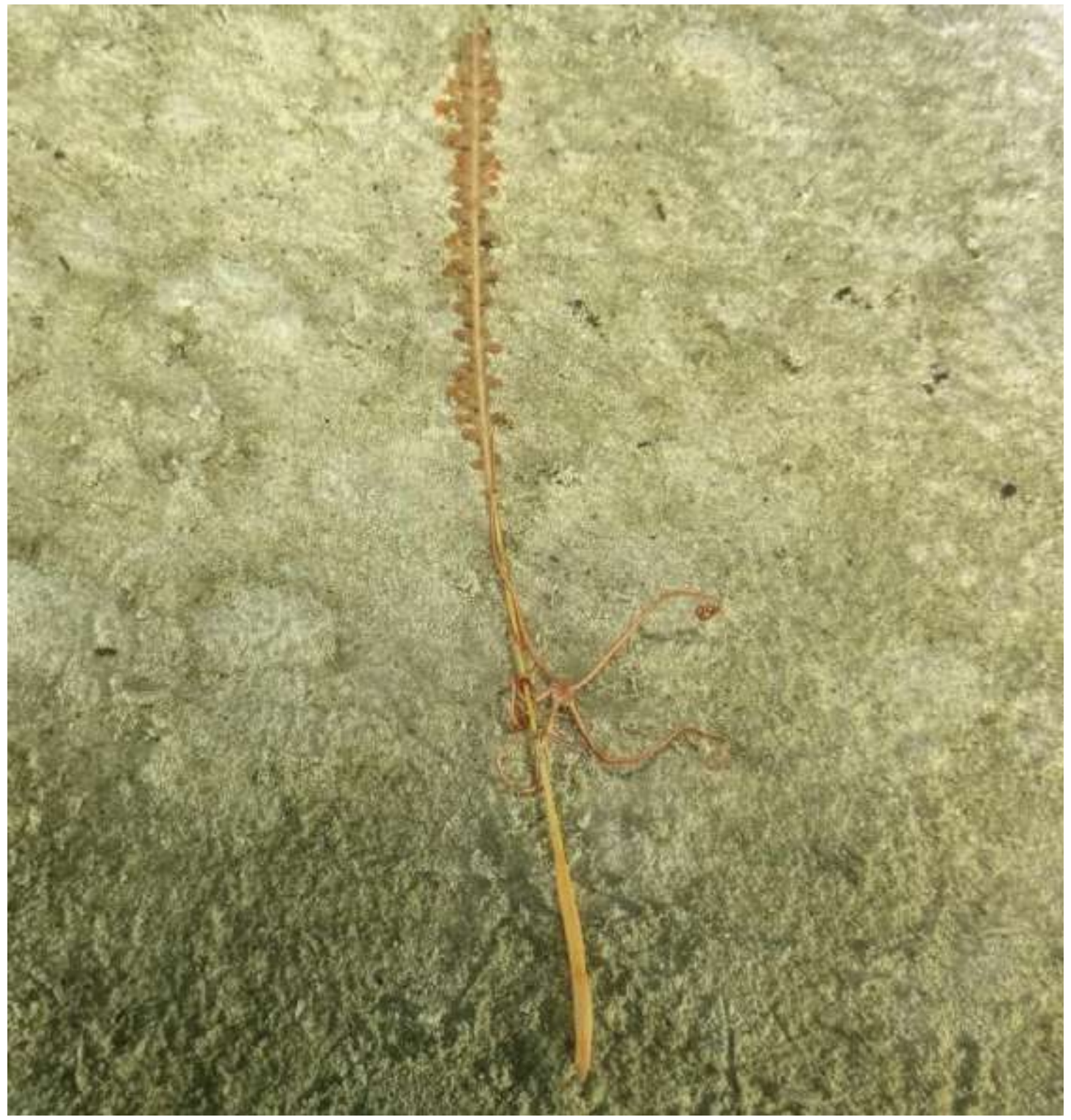

Attaches to soft substrates. Attachment is small. Large, fleshy polyps alternately arranged laterally along the stalk. Not branched. No nodes develop. Superfamily Pennatuloidea

- Colony elongate
- Bilateral symmetry
- Axis present
- Polyp leaves absent

Octocorallia; Scleralcyonacea; Chrysogorgiidae; *Chrysogorgia* sp. indet. 1

NC4_BK6_4700_NOR_0060

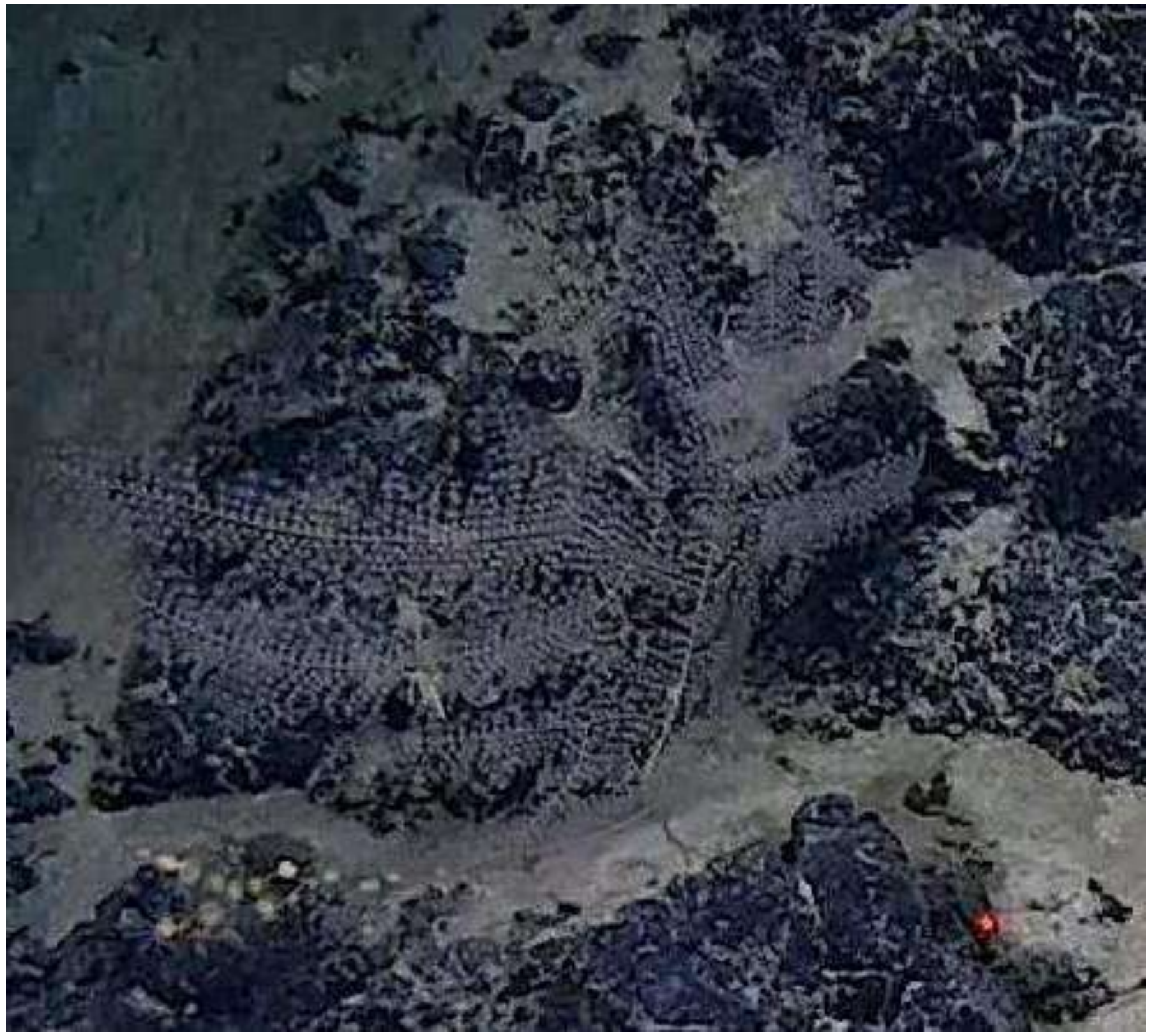

Attaches to hard substrates. Branching occurs low on the stalk. Rigid structure that develops into an arborescent shape. Nodes develop at < 45-degree angle.

Octocorallia; Scleralcyonacea; Scleralcyonacea fam. indet. 1

NC4_BK4_4700_IMG_6056

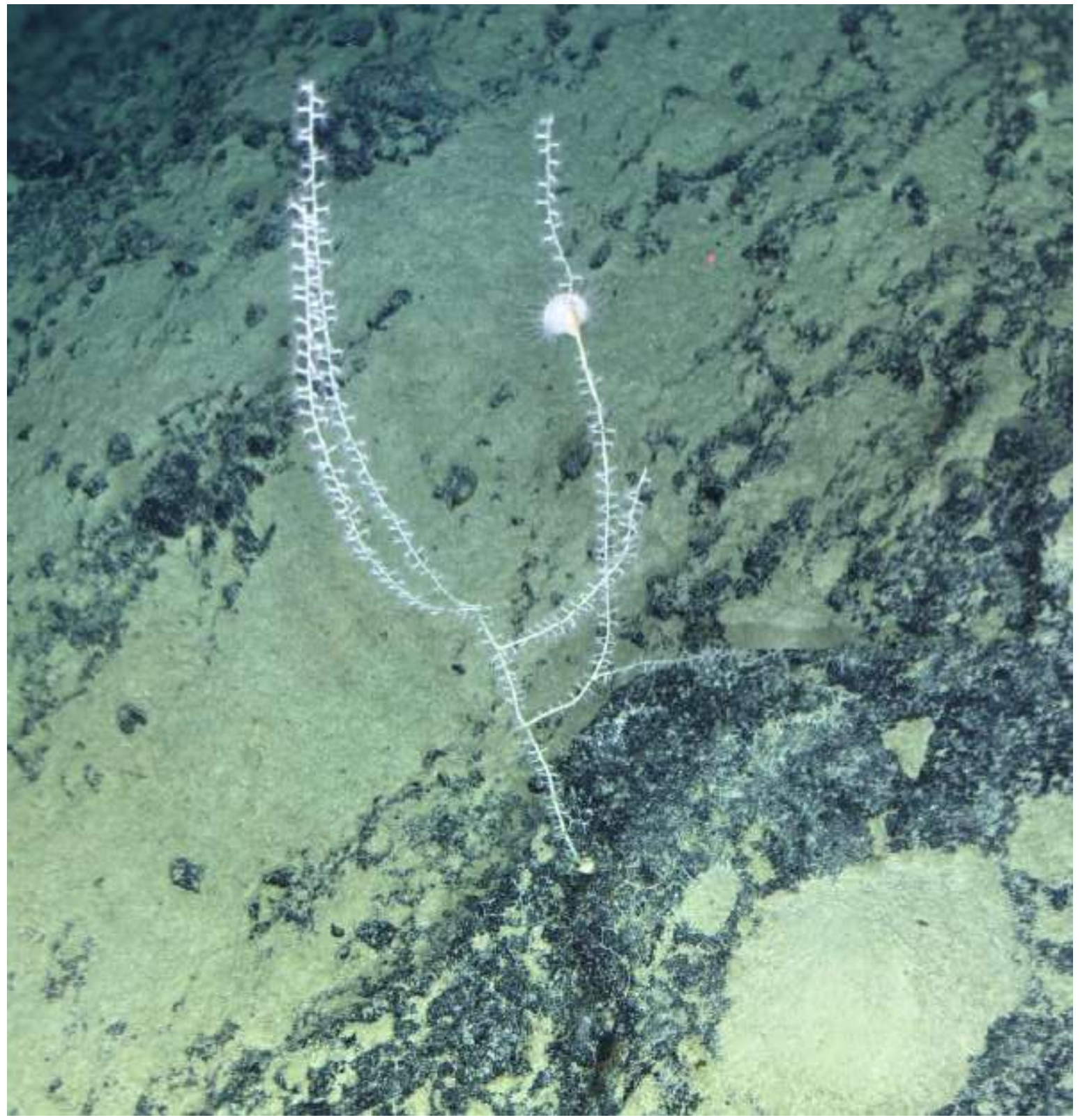

Attaches to hard substrates. Attachment is small. Polyps alternately arranges laterally along the stalk. Rigid structure that develops into an arborescent shape that branches higher on the stem. Nodes develop at roughly 90 degrees. Possibly Keratoisididae

Octocorallia
Scleralcyonacea fam. indet. 2

NC4_BK2_4700_NOR_0072
NC4_BK2_4700_IMG_5431
NC4_BK2_4700_IMG_5251

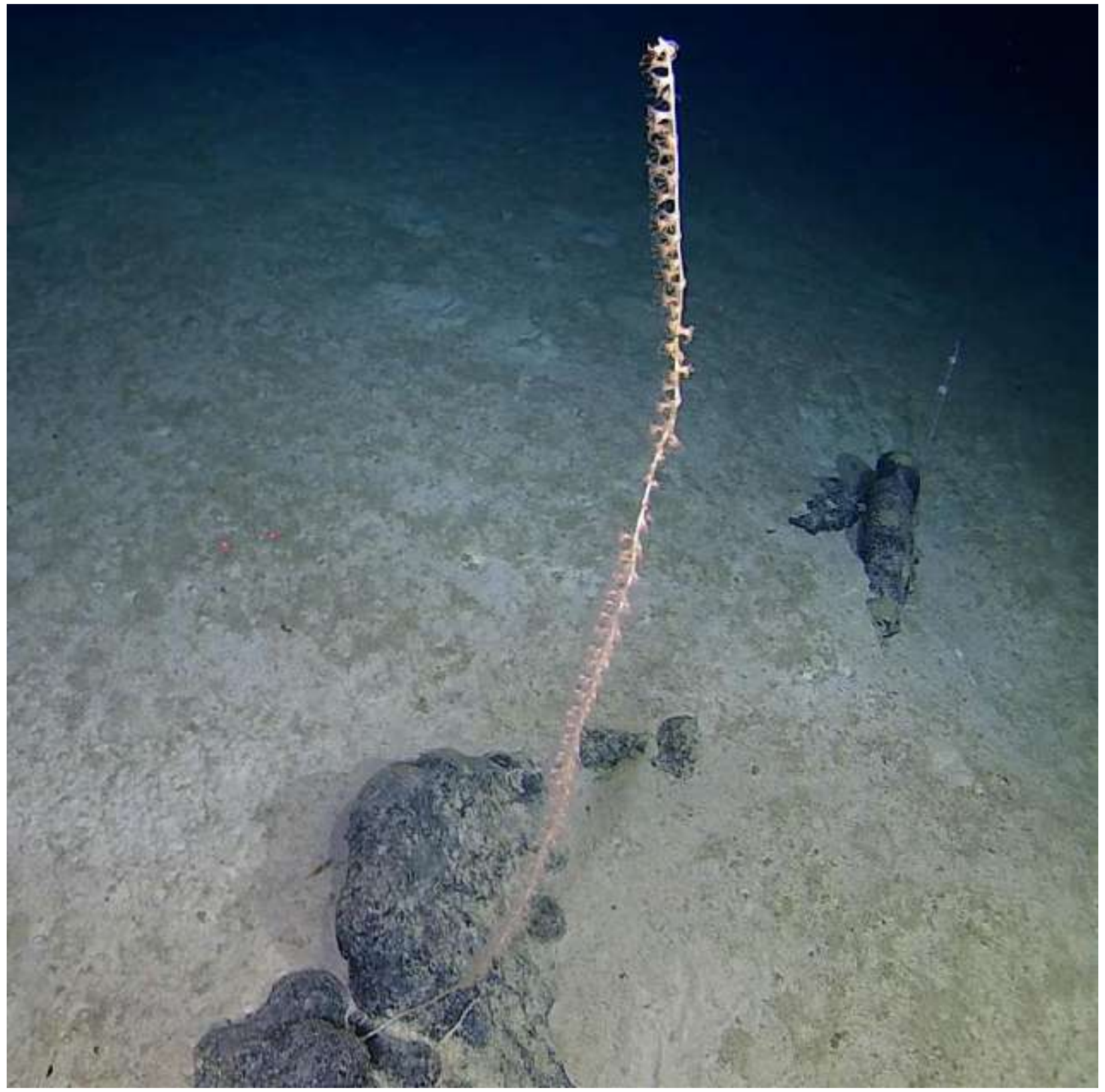
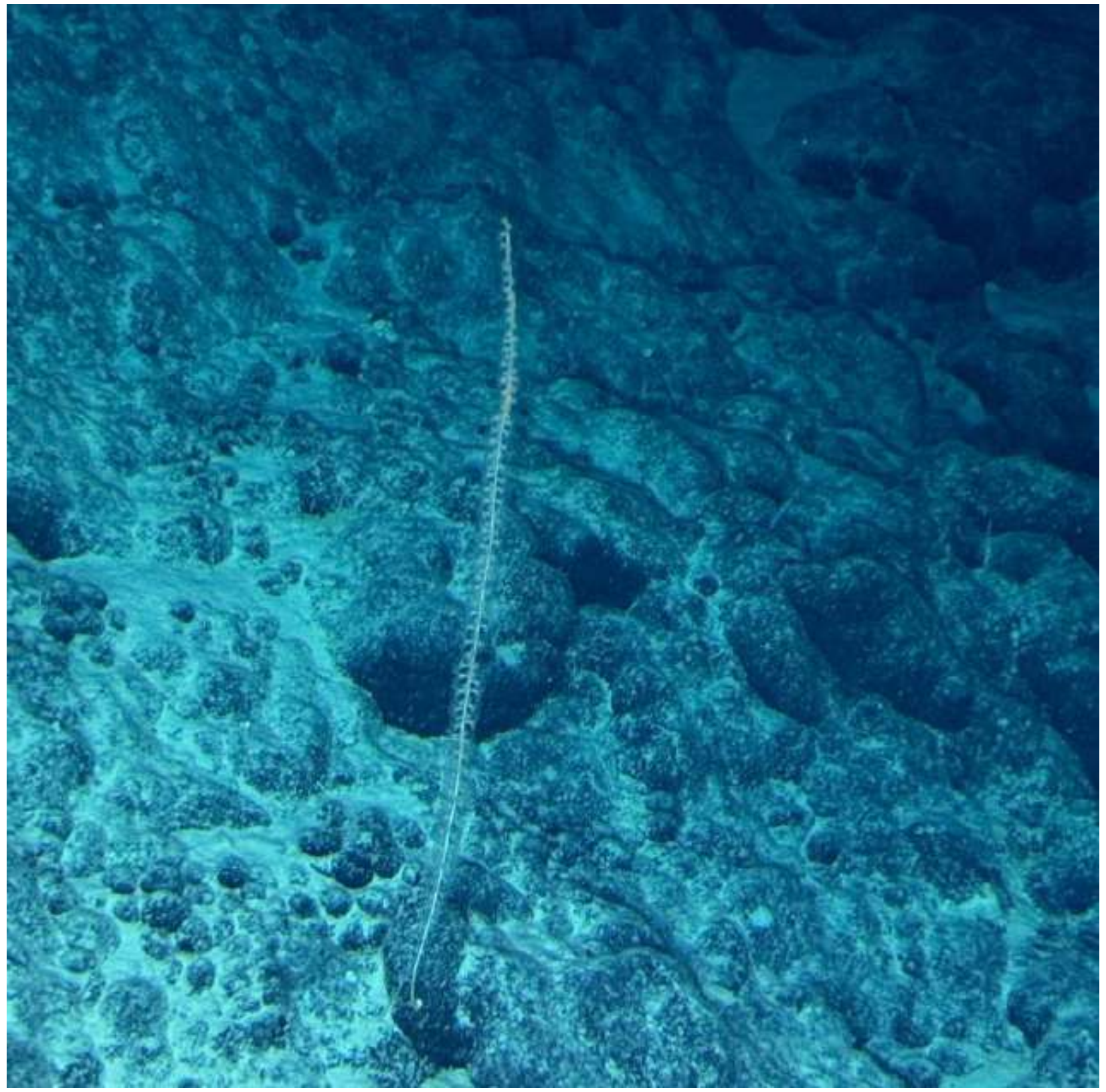
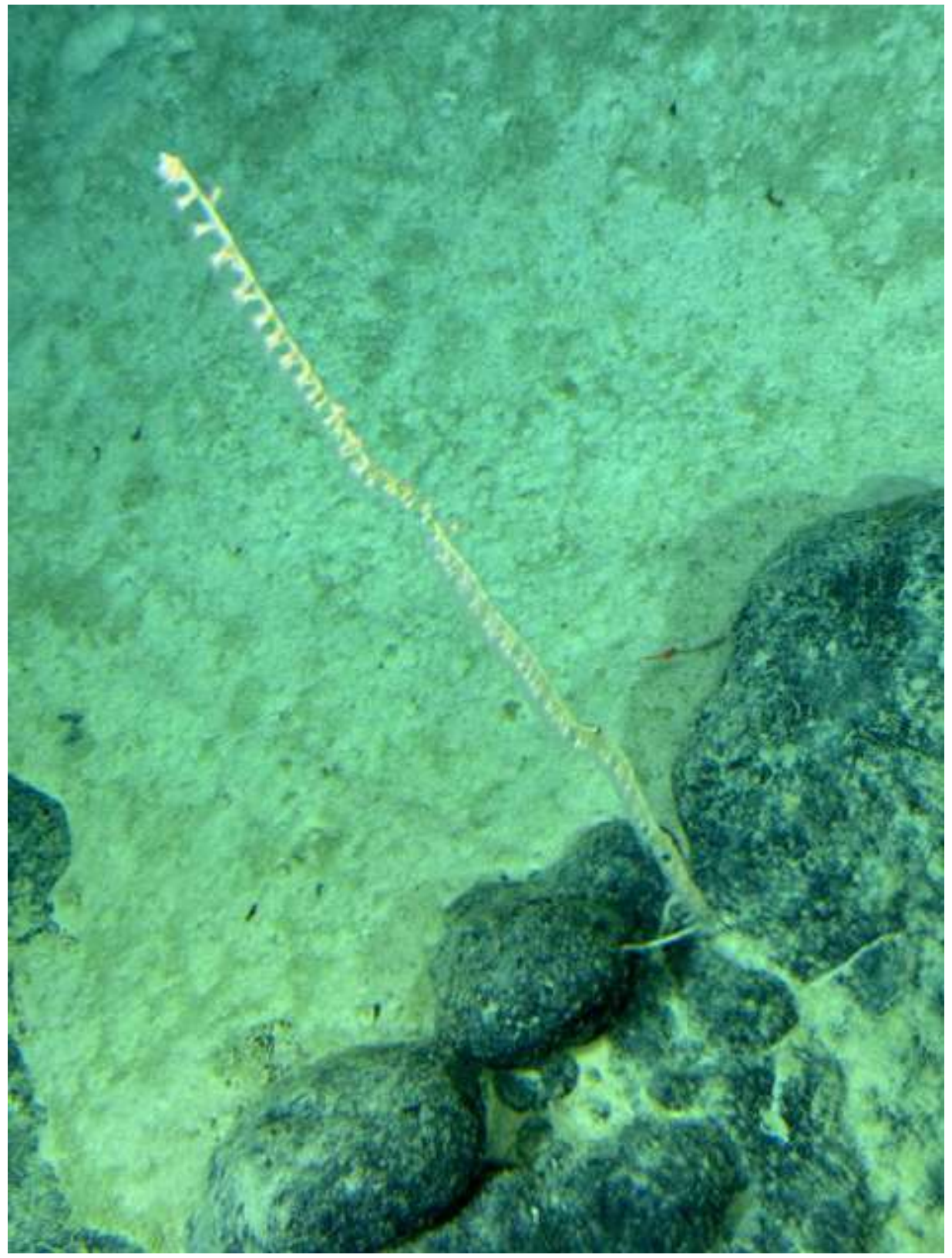

Attaches to hard substrates. Attachment is small. Polyps alternately arranged laterally along the stalk. Rigid structure that develops into a single strand. Not branched. No nodes develop.

Octocorallia
Scleralcyonacea fam. indet. 3

NC4_BK6_4700_NOR_0065
NC4_BK6_4700_IMG_6852

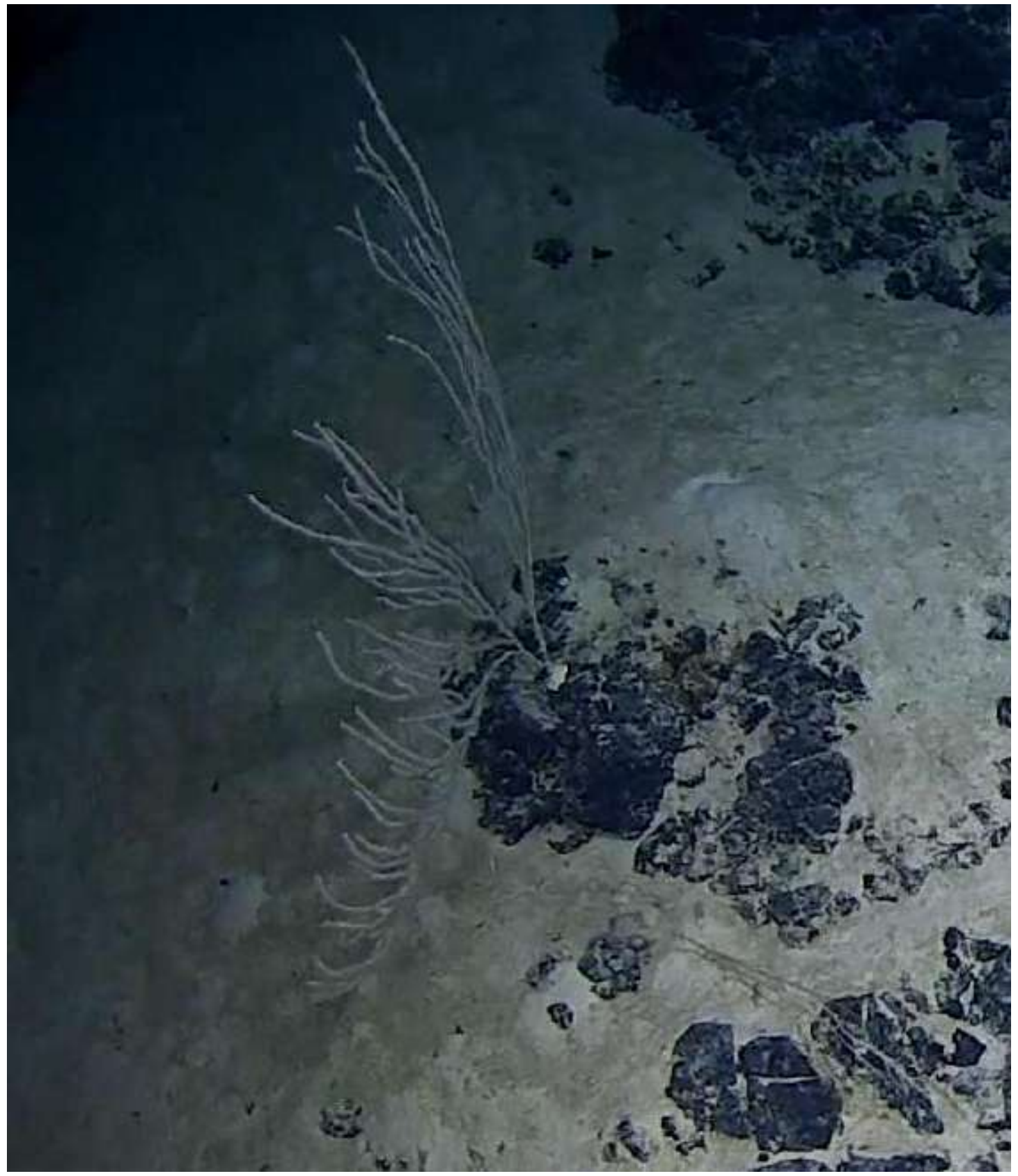

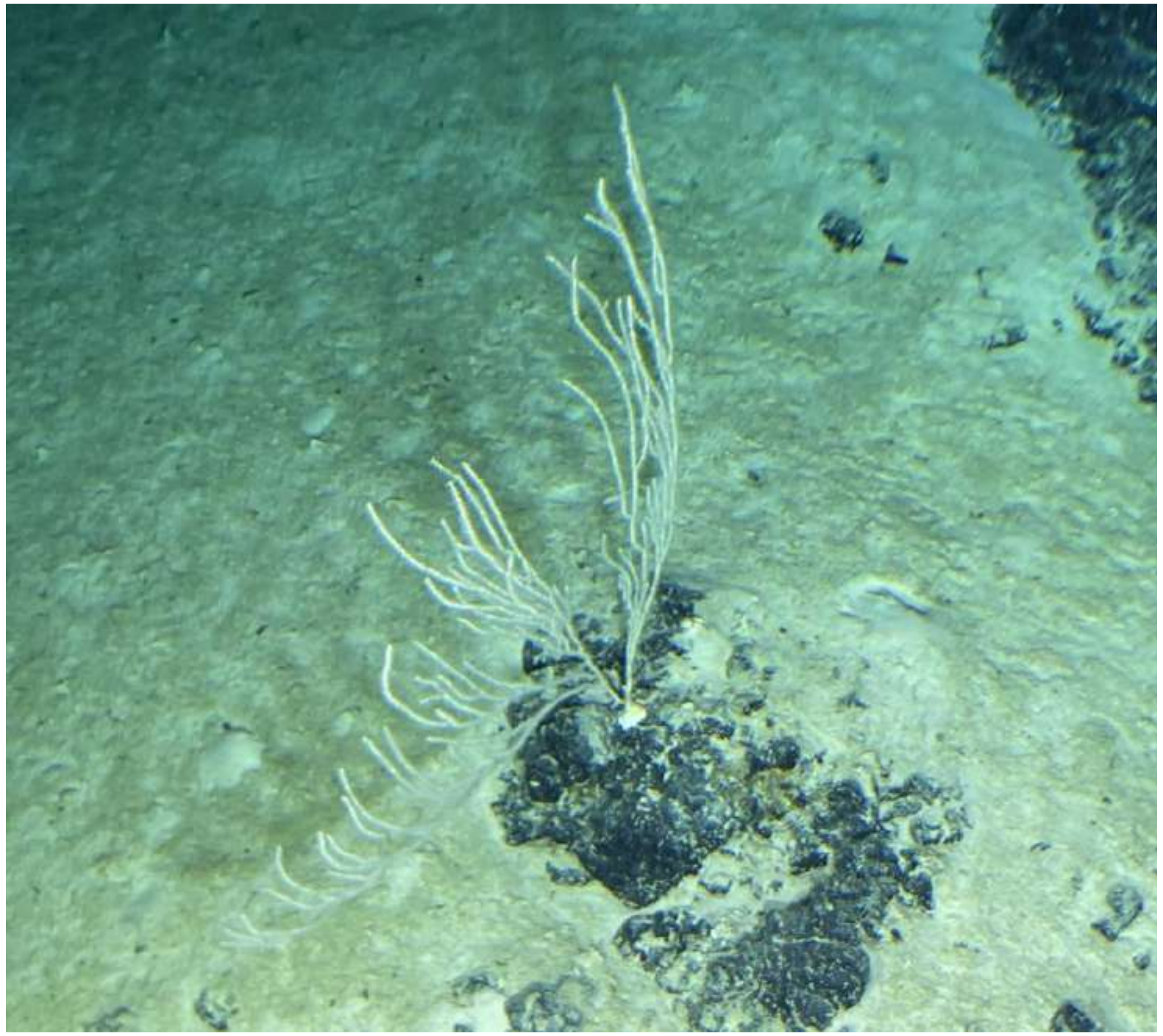

Attaches to hard substrates. Attachment is large. Polyps are small and arranged laterally along the stalk. Rigid structure that develops into an arborescent shape and branches at the base. Nodes develop at < 45-degree angle.

Octocorallia
Scleralcyonacea fam. indet. 4

NC4_BK4_4700_NOR_0057

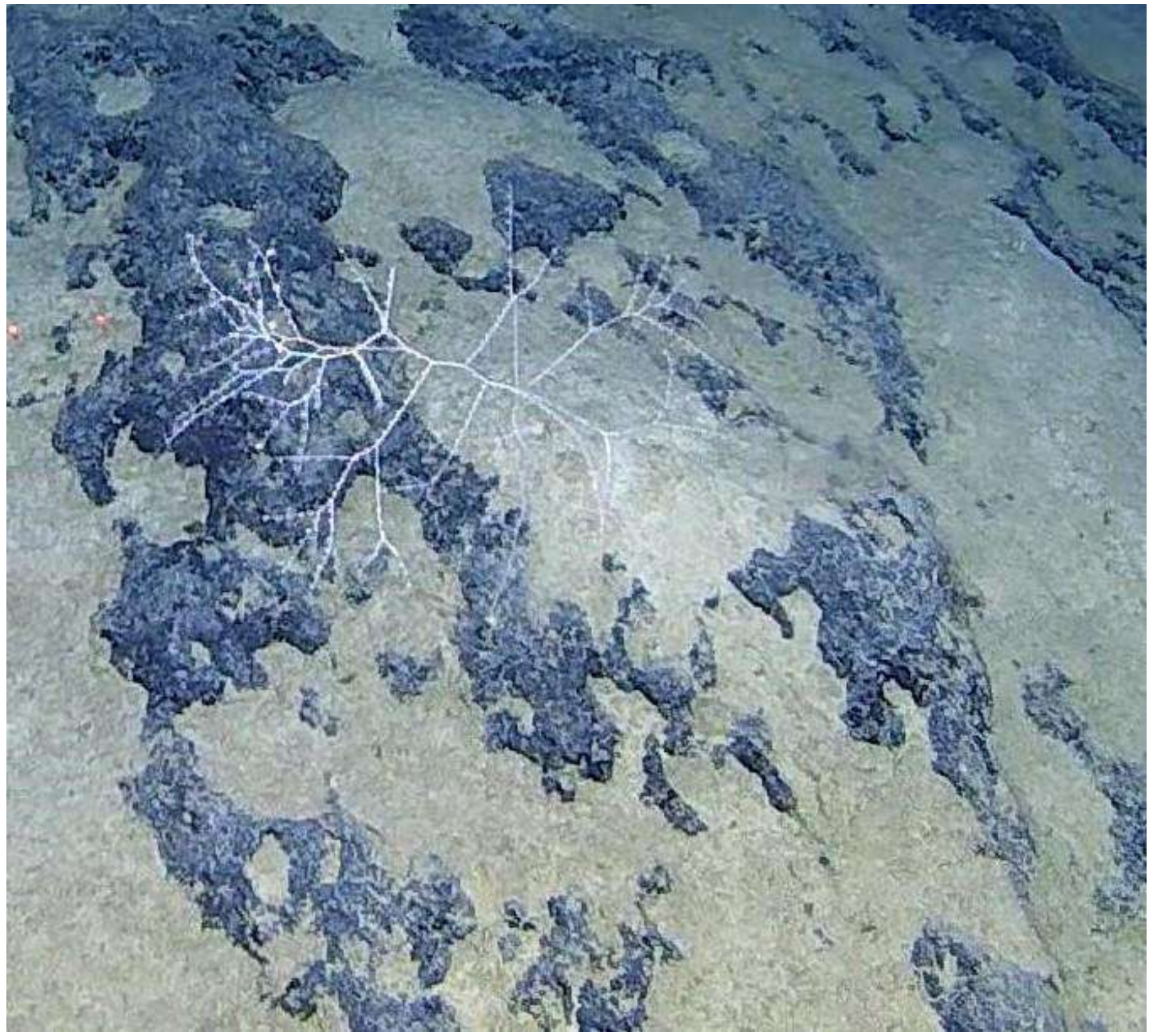

Attaches to hard substrates. Attachment is small. Branching occurs high on the stalk. Rigid structure that develops into an arborescent shape and branches near the base. Nodes develop at < 45-degree angle.

Octocorallia
Scleralcyonacea fam. indet. 5

NC2_BK5_4600_IMG_9712

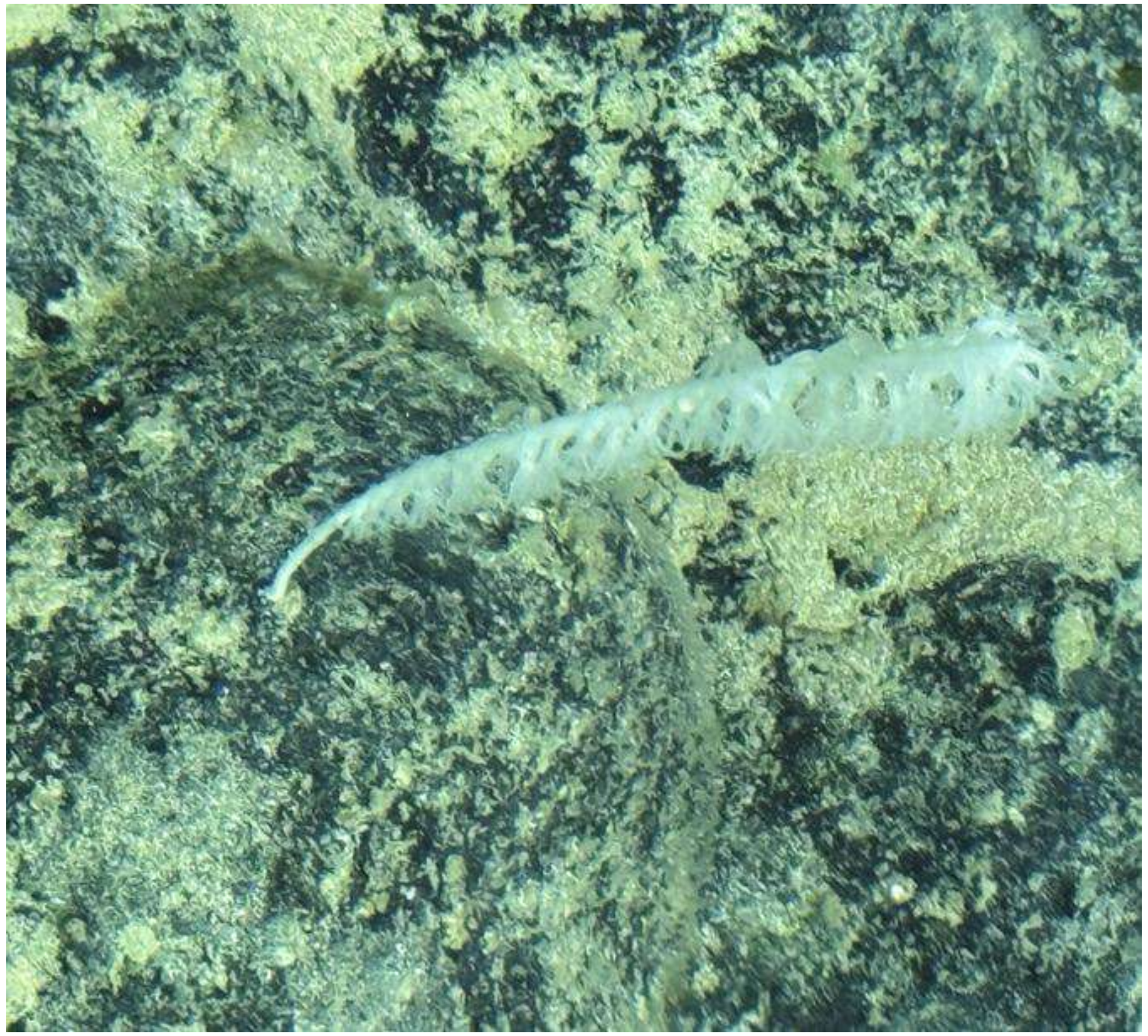

Attaches to hard substrates. Attachment is small. Large, fleshy polyps alternately arranged laterally along the stalk similar in shape to Umbellulidae polyps. Not branched. No nodes develop. Not a pennatuloid, possibly a bamboo coral.

Hydrozoa; Hydrozoa spp

NC2_CR5_5500_NOR_0088

NC2_OM2_3400_NOR_0037

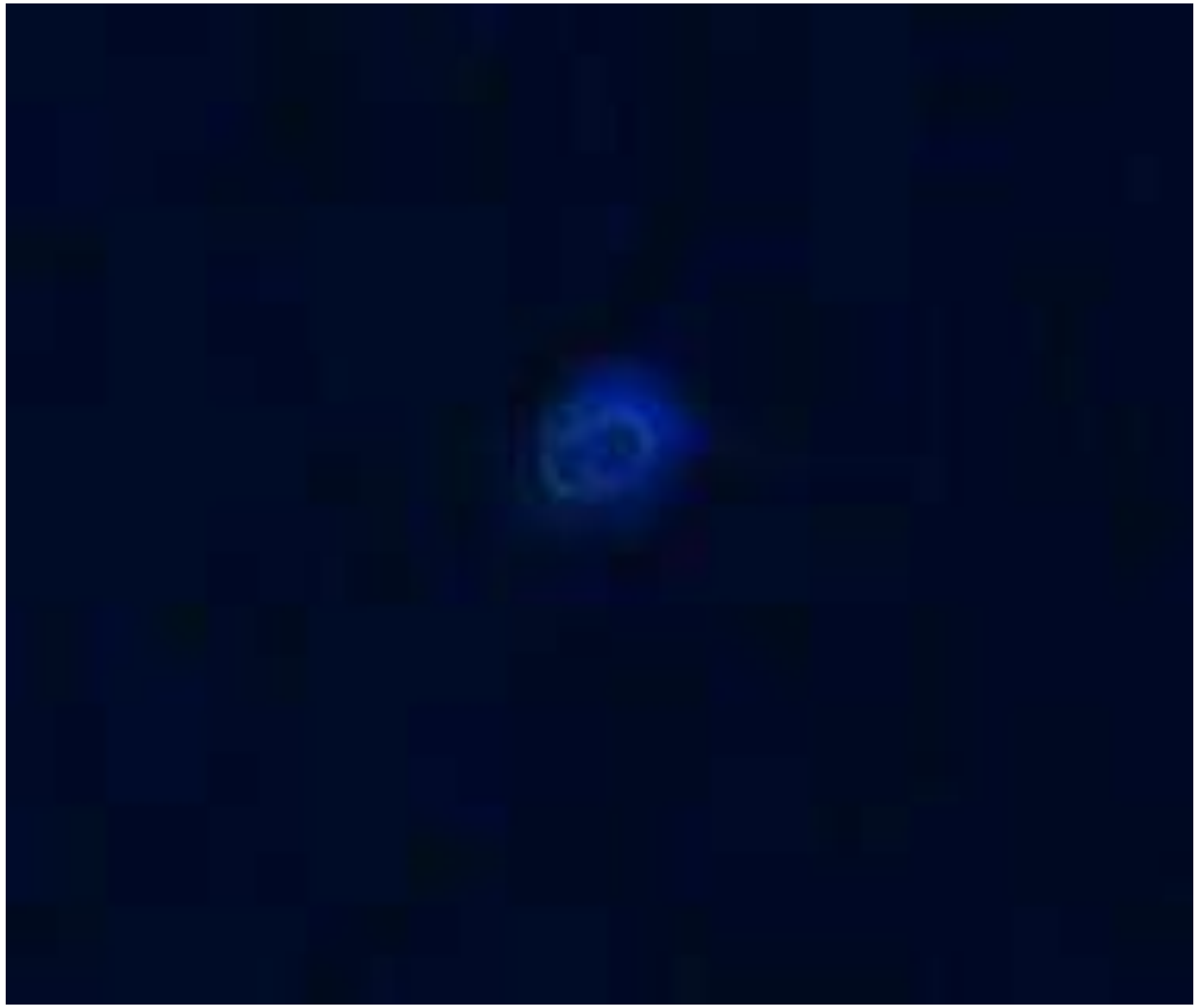

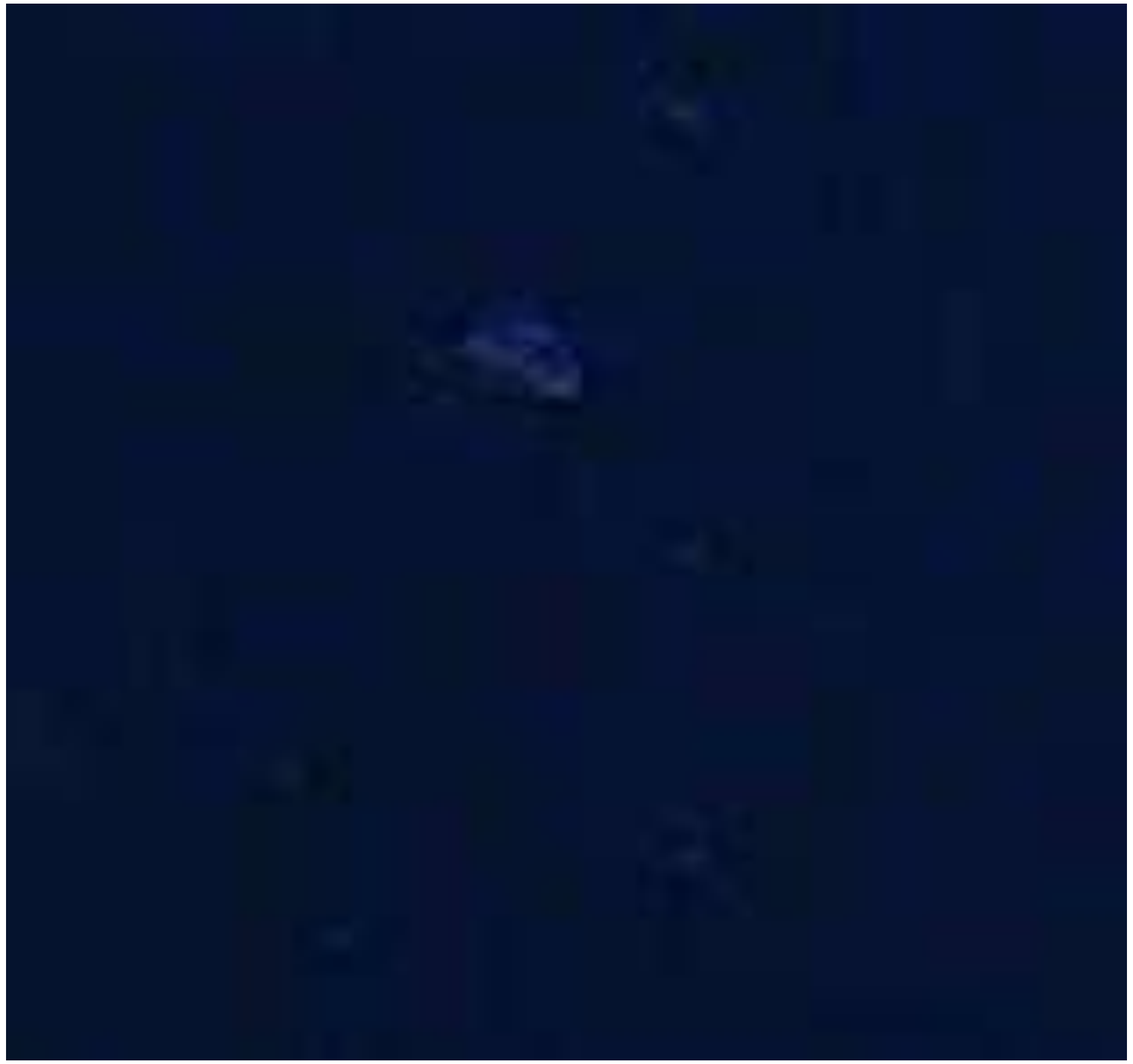

Dome shaped bell is translucent with multiple tentacles around the margin of the bell near the interradial stomach pouch.

Hydrozoa; Narcomedusae; Aeginidae; Aeginidae gen. indet. 1

NC1_MA4_7000_NOR_0051

NC1_OM8_7900_NOR_0092

NC4_CR5_6900_NOR_0053

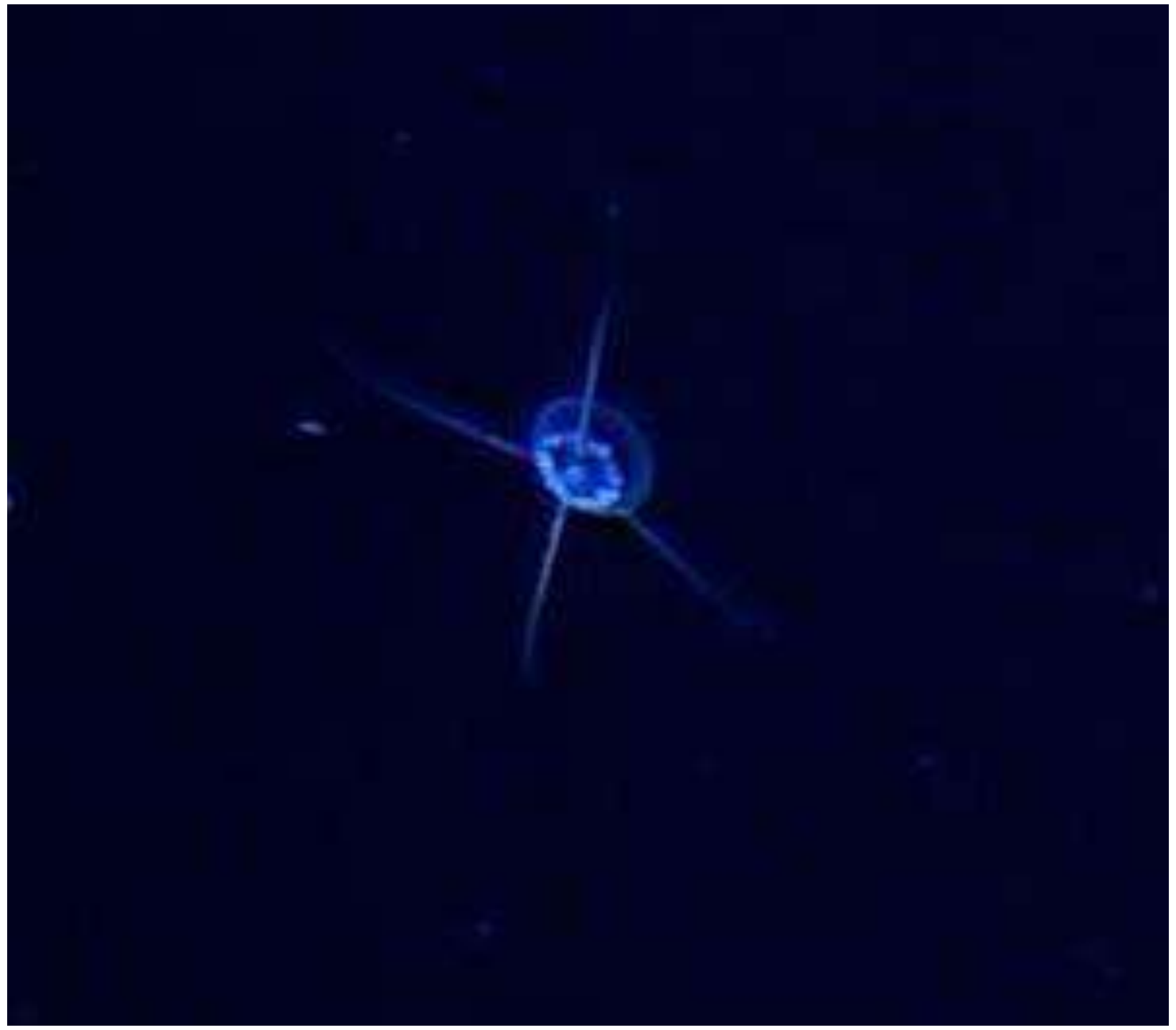

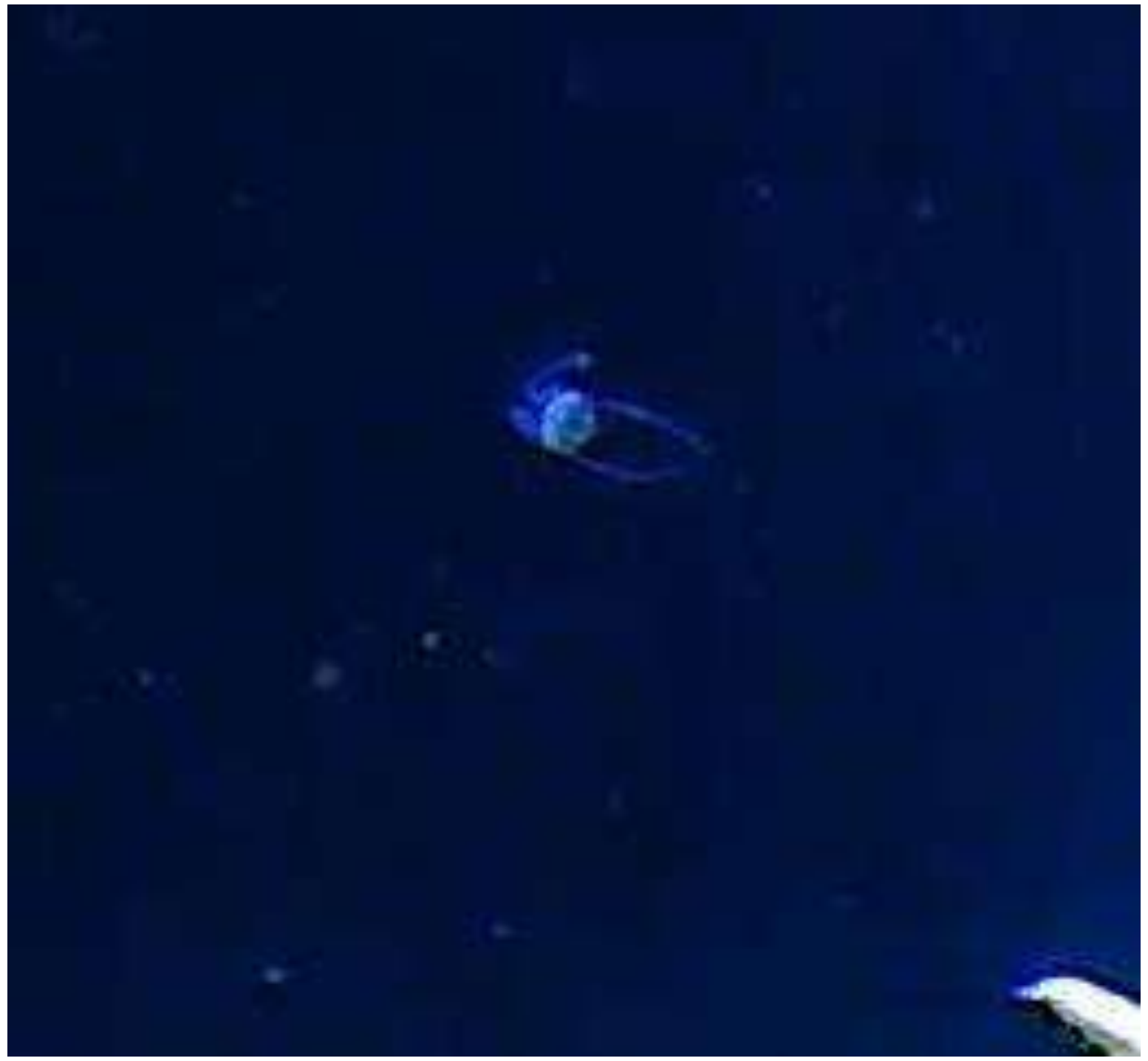

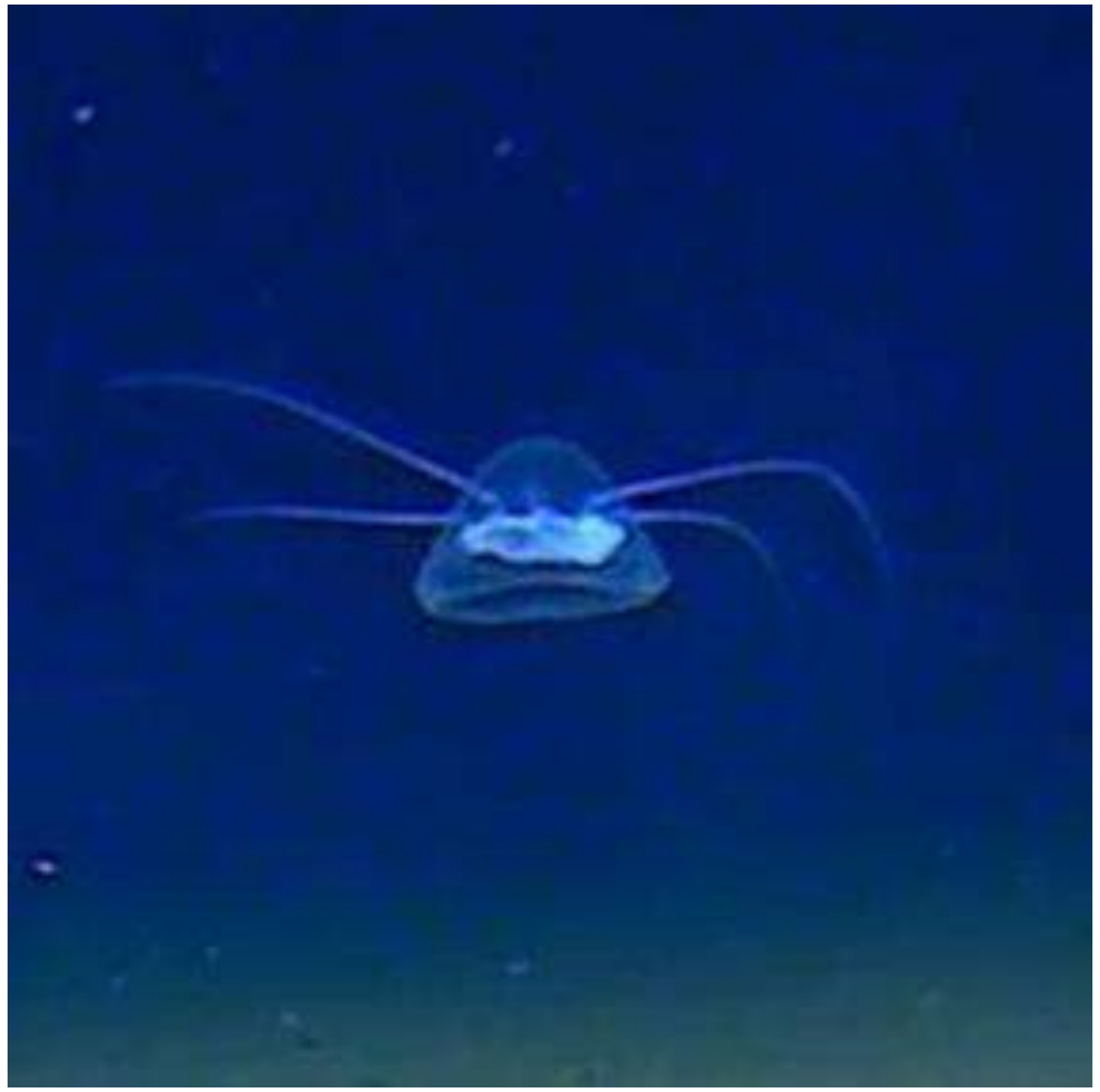

Dome shaped bell is translucent with 4 non-contractile tentacles arranged outward above the margin of the bell near the interradial stomach pouch.

Hydrozoa; Narcomedusae; Aeginidae; Aeginidae gen. indet. 2

NC3_BK2_6000

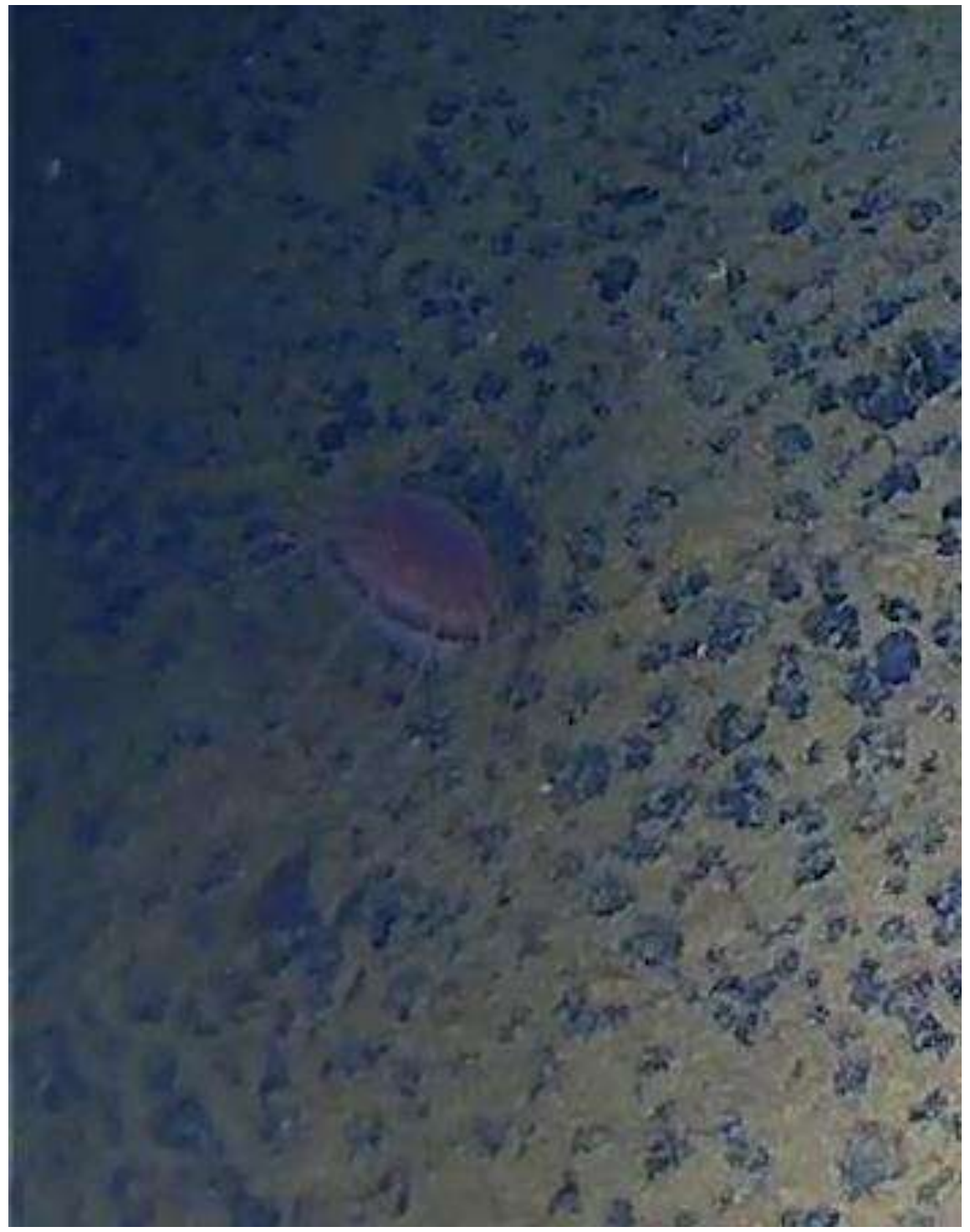

Dome shaped bell is translucent with 4 non-contractile tentacles arranged outward above the margin of the bell near the interradial stomach pouch. Red colour

Hydrozoa; Narcomedusae; Cuninidae; Cuninidae gen. indet. 1

NC3_CR3_5500_NOR_0102

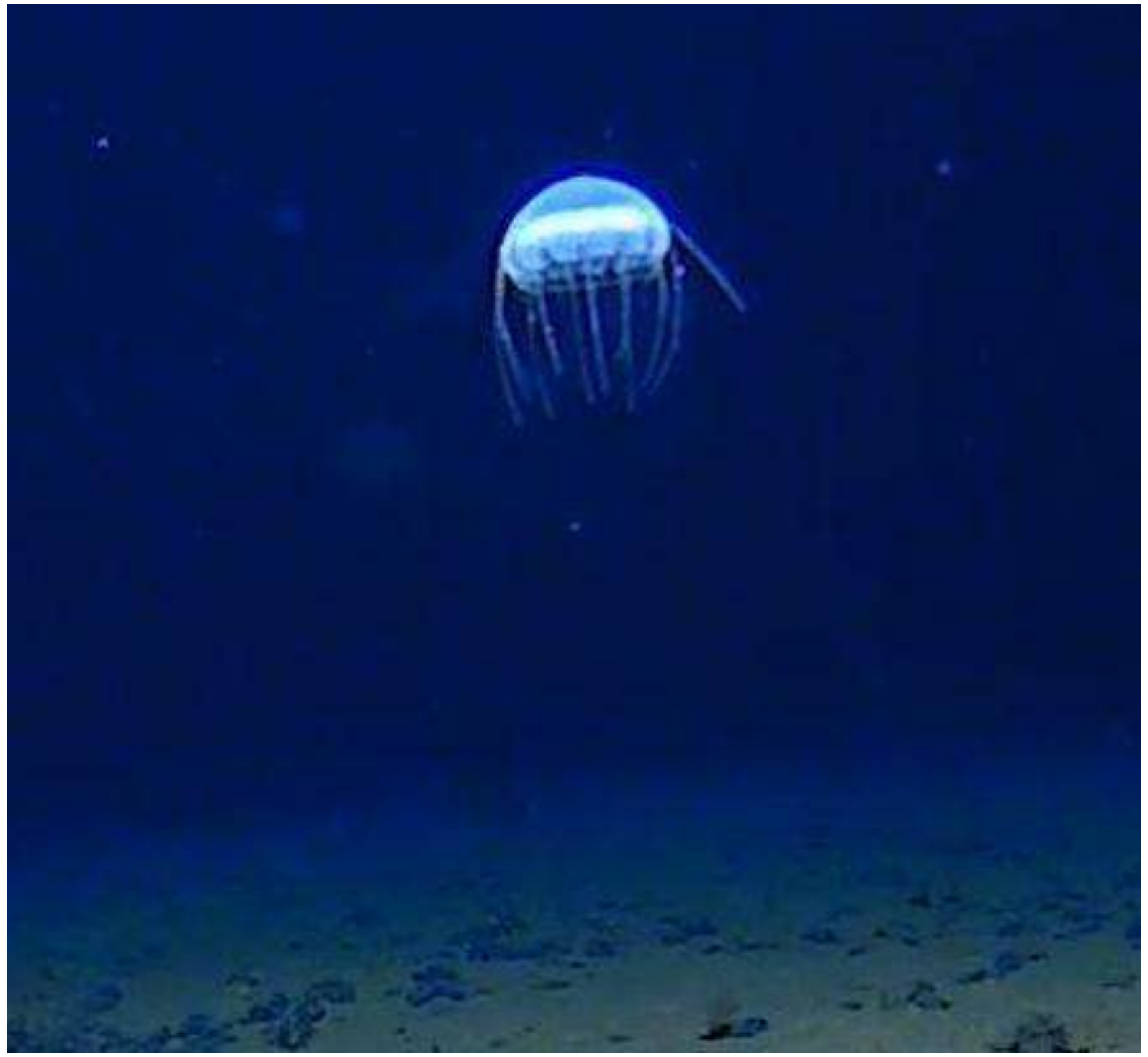

Domed shaped bell is semi-transparent. Multiple tentacles extend from above the bell margin near the interradial stomach pouch. No secondary tentacles are present.

Scyphozoa; Semaeostomeae; Ulmaridae; Ulmaridae gen. indet. 1

NC1_BK3_8000_IMG_5737

NC2_BK3_7400_IMG_9119

NC2_BK3_7400_IMG_9147

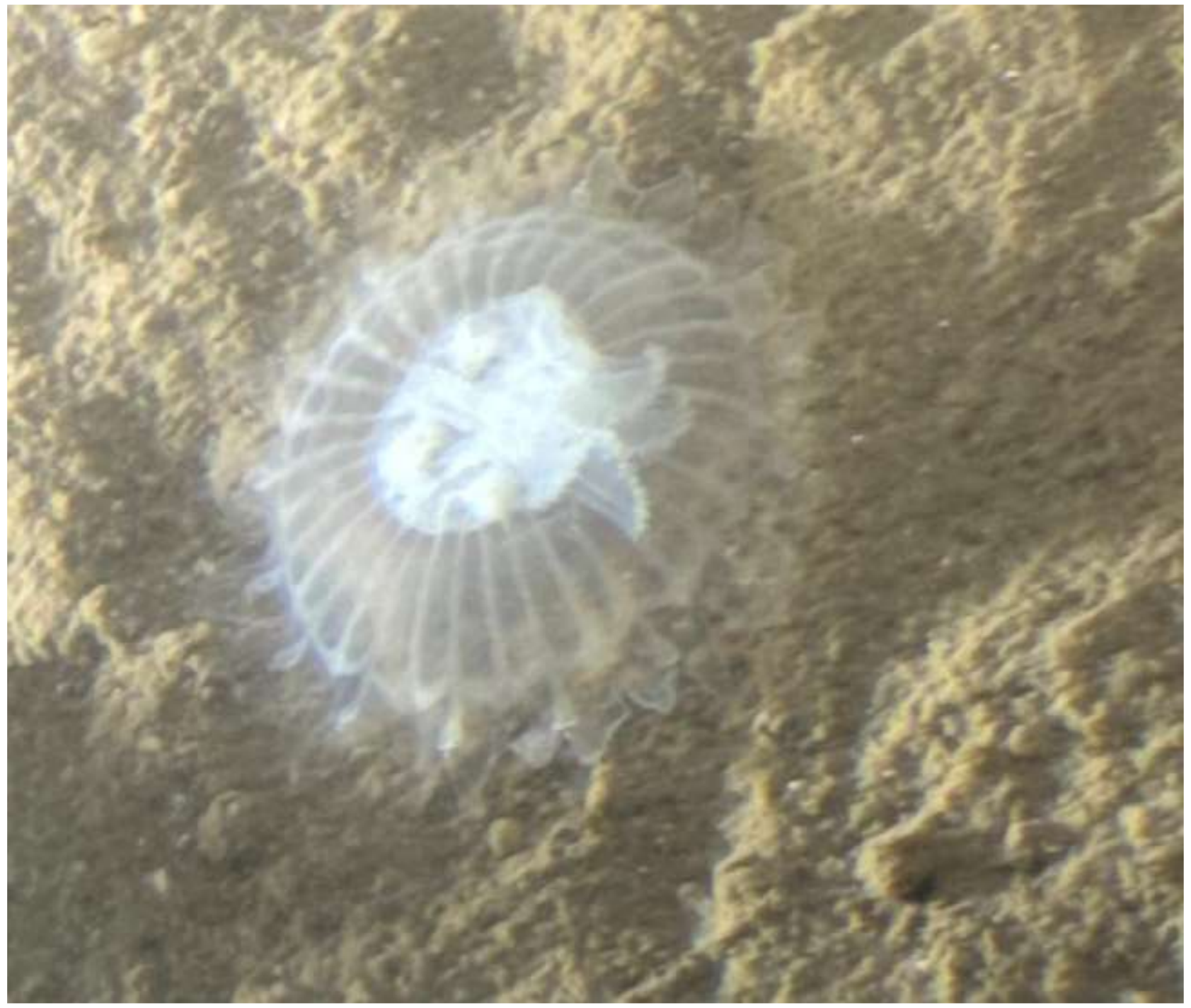

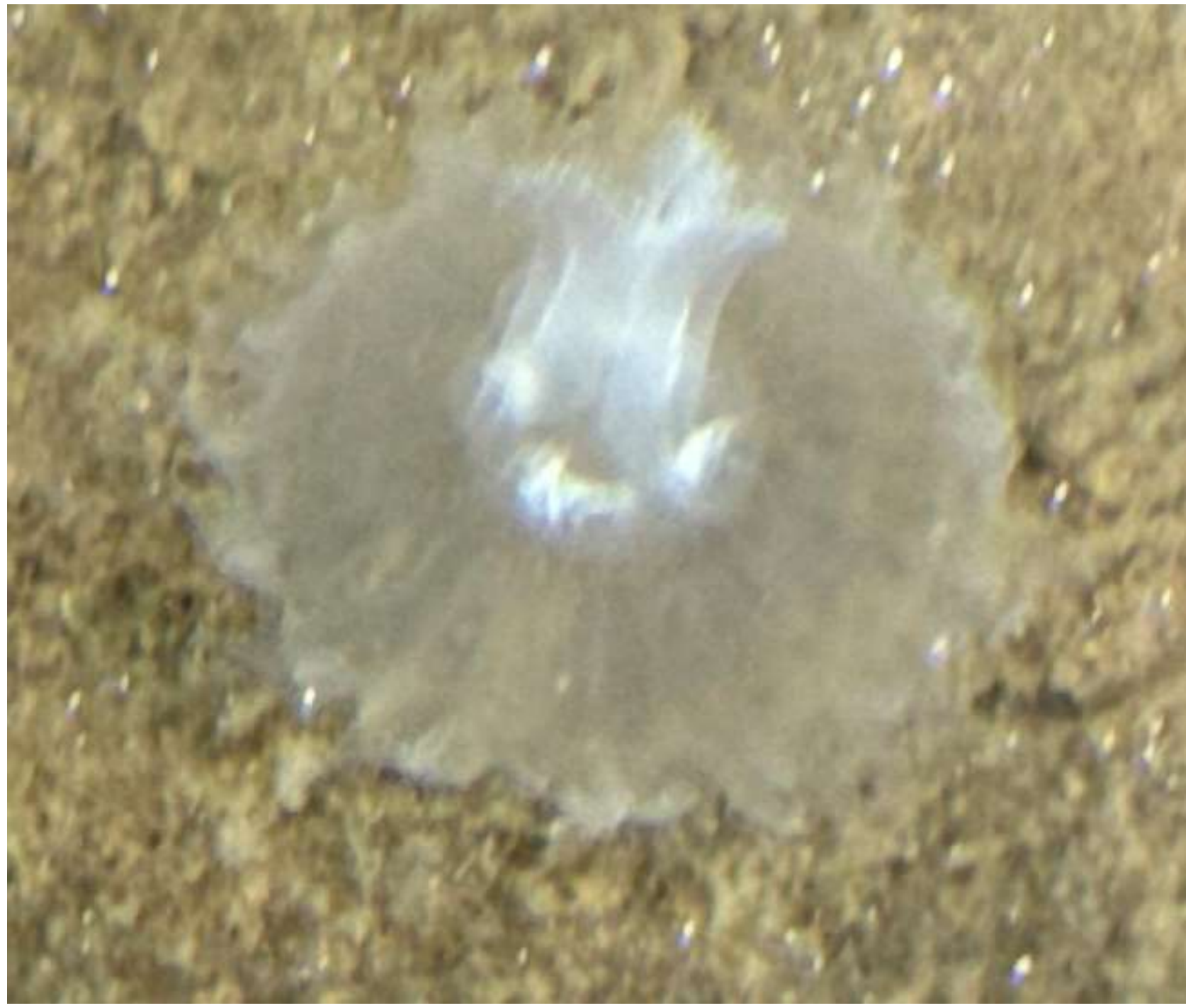

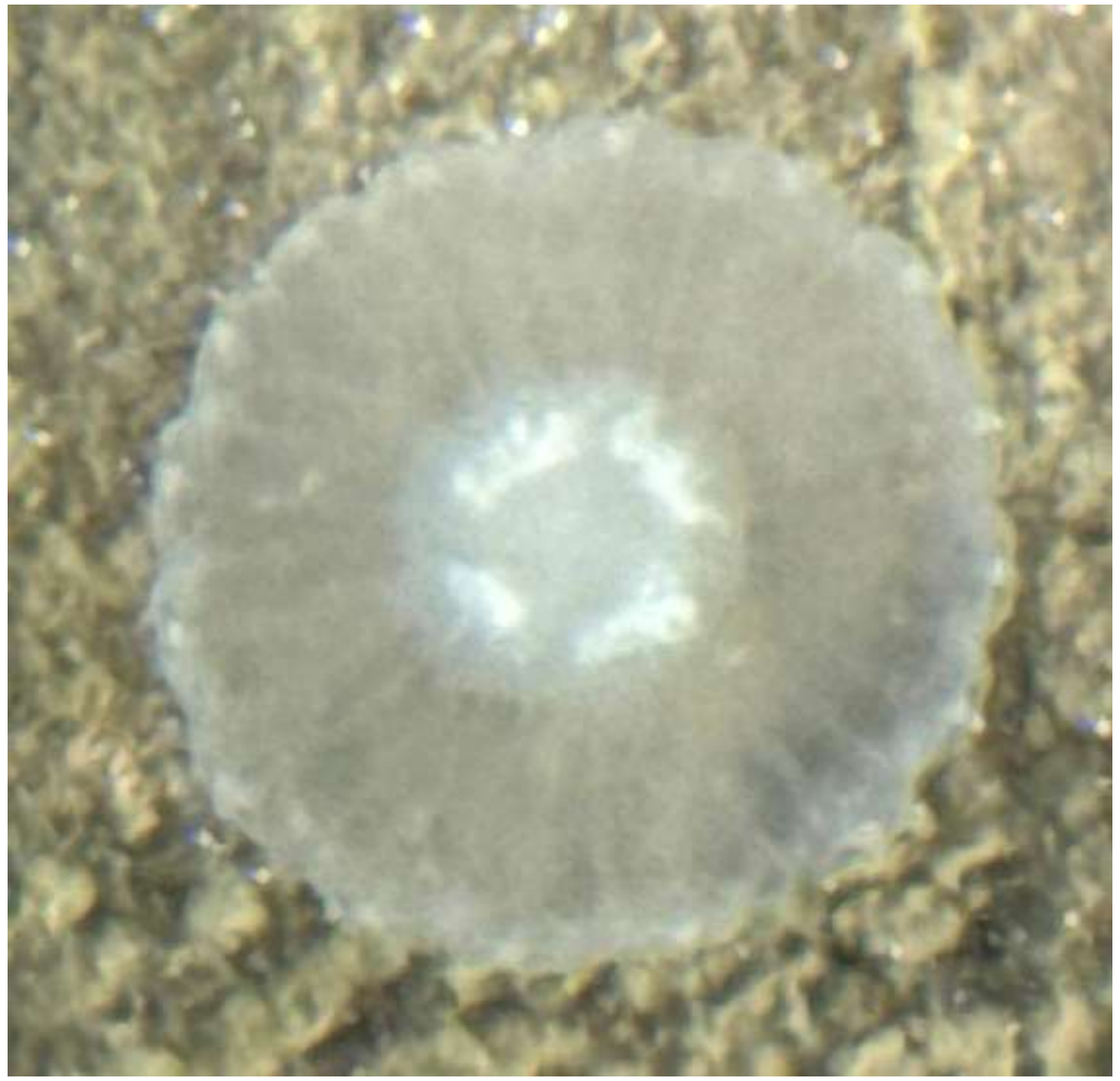

Bell is translucent with stomach arms short and tentacles absent. Radial canals appear as 4 distinct, white "bars" that form a circle at the top of the bell.

Scyphozoa; Semaeostomeae; Ulmaridae; Ulmaridae gen. indet. 2

NC1_BK1_6200 _IMG_4758

NC1_BK1_6200_NOR_0091

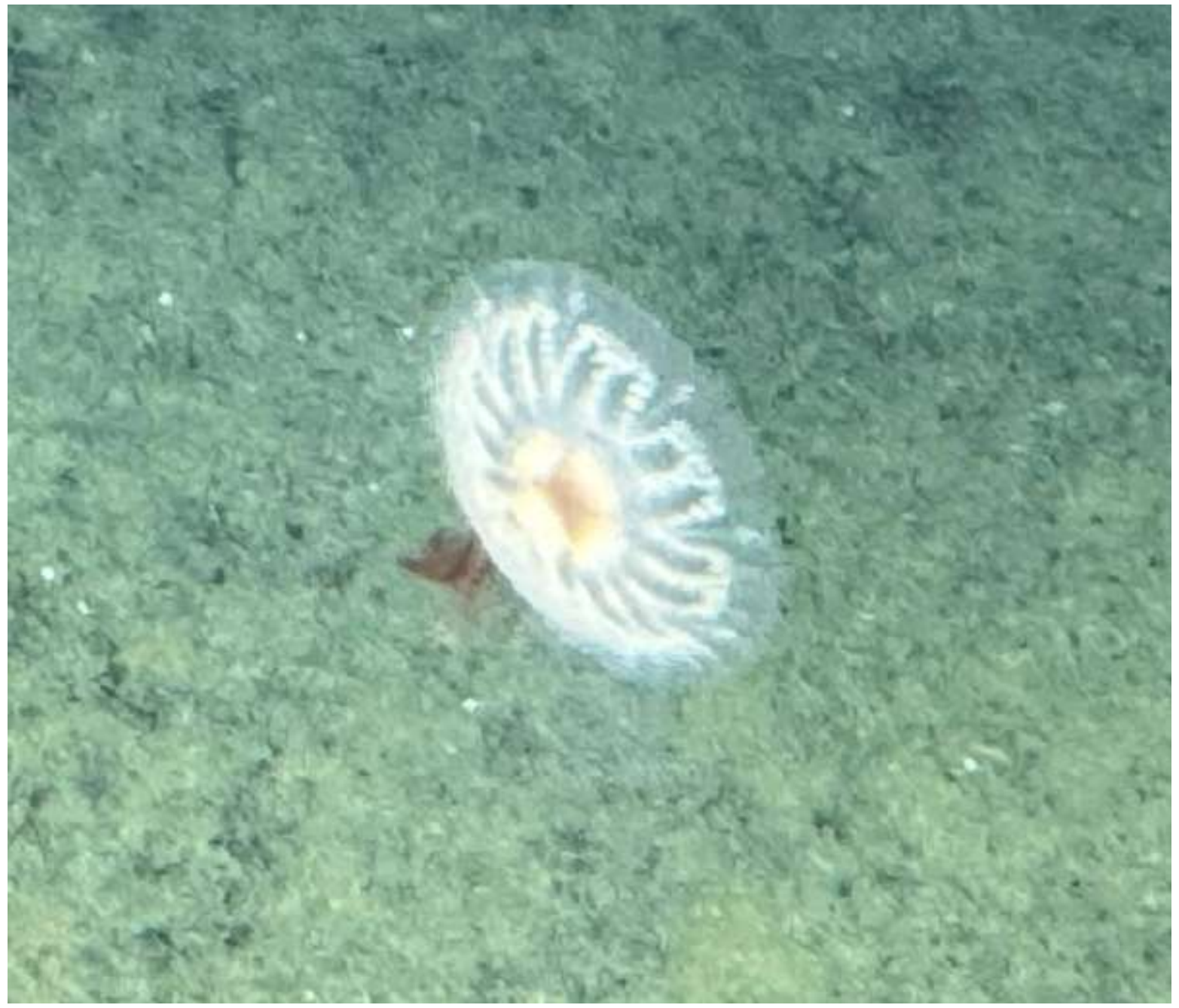

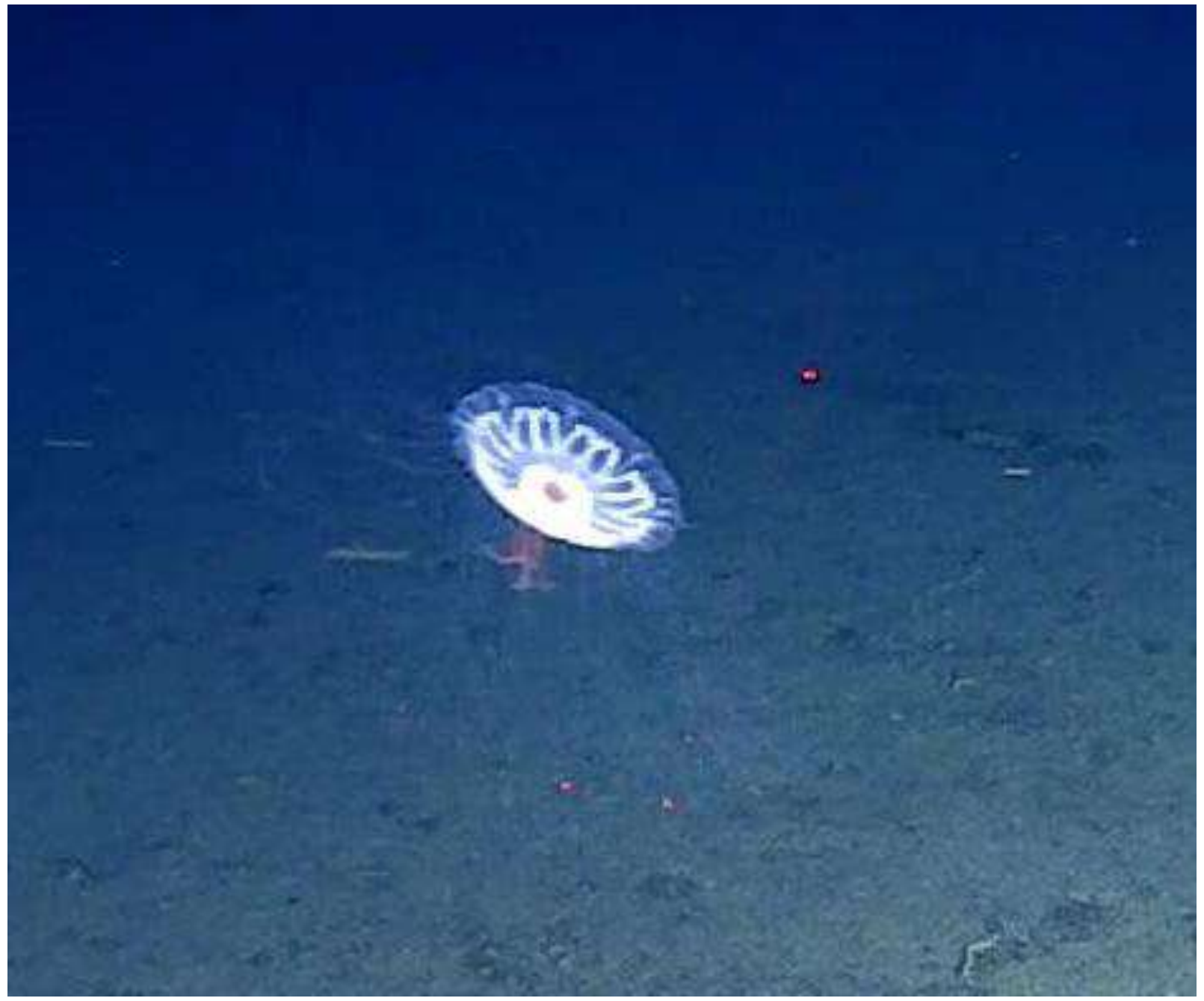

Bell is translucent with stomach arms short and tentacles absent. Stomach arms appear brownish red. Radial canals appear as 4 distinct, yellowish “bars” that form a circle at the top of the bell. Strong yellowish coloured lobes present.

Scyphozoa; Semaeostomeae; Ulmaridae; Ulmaridae gen. indet. 3

NC2_BK1_3200_IMG_7362

NC2_BK1_3200_IMG_7684

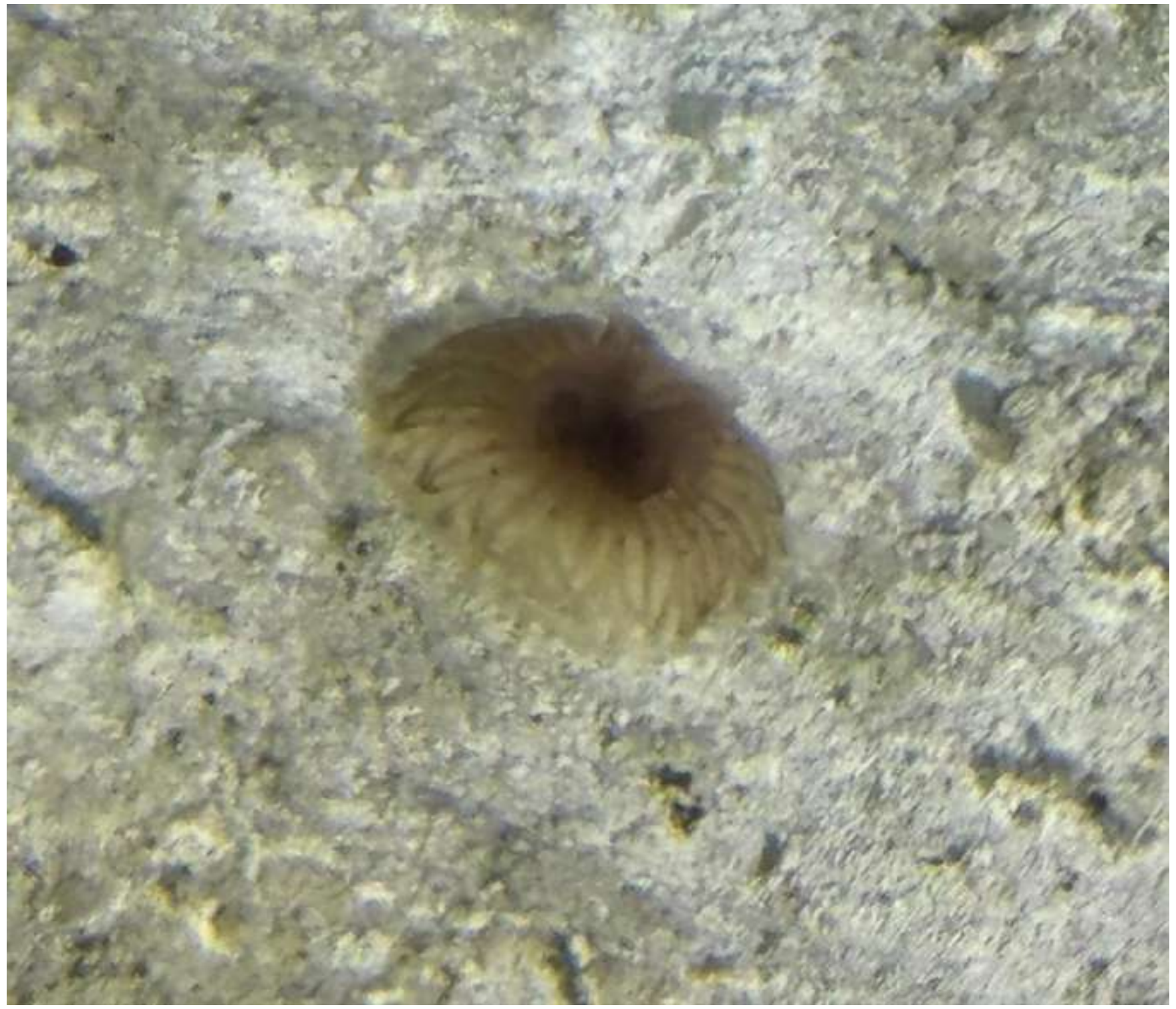

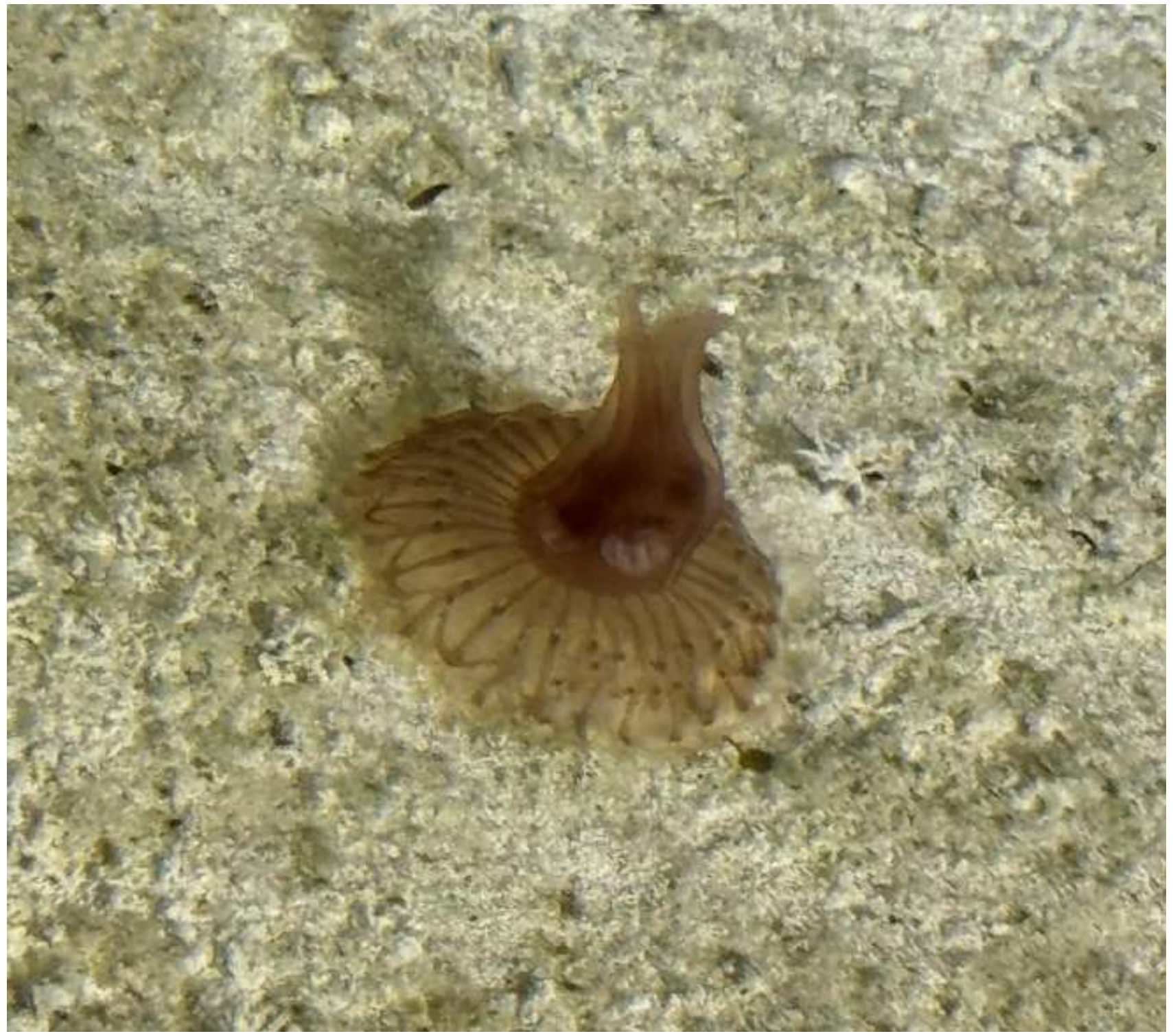

Bell is dark brown and semi-translucent with stomach arms short and tentacles absent. Stomach arms appear brown. Radial canals appear as a dark mass that forms at the top of the bell.

Scyphozoa; Semaeostomeae; Ulmaridae; Ulmaridae sp. indet. 4

NC2_BK1_3200_IMG_6945

NC2_BK1_3200_IMG_7512

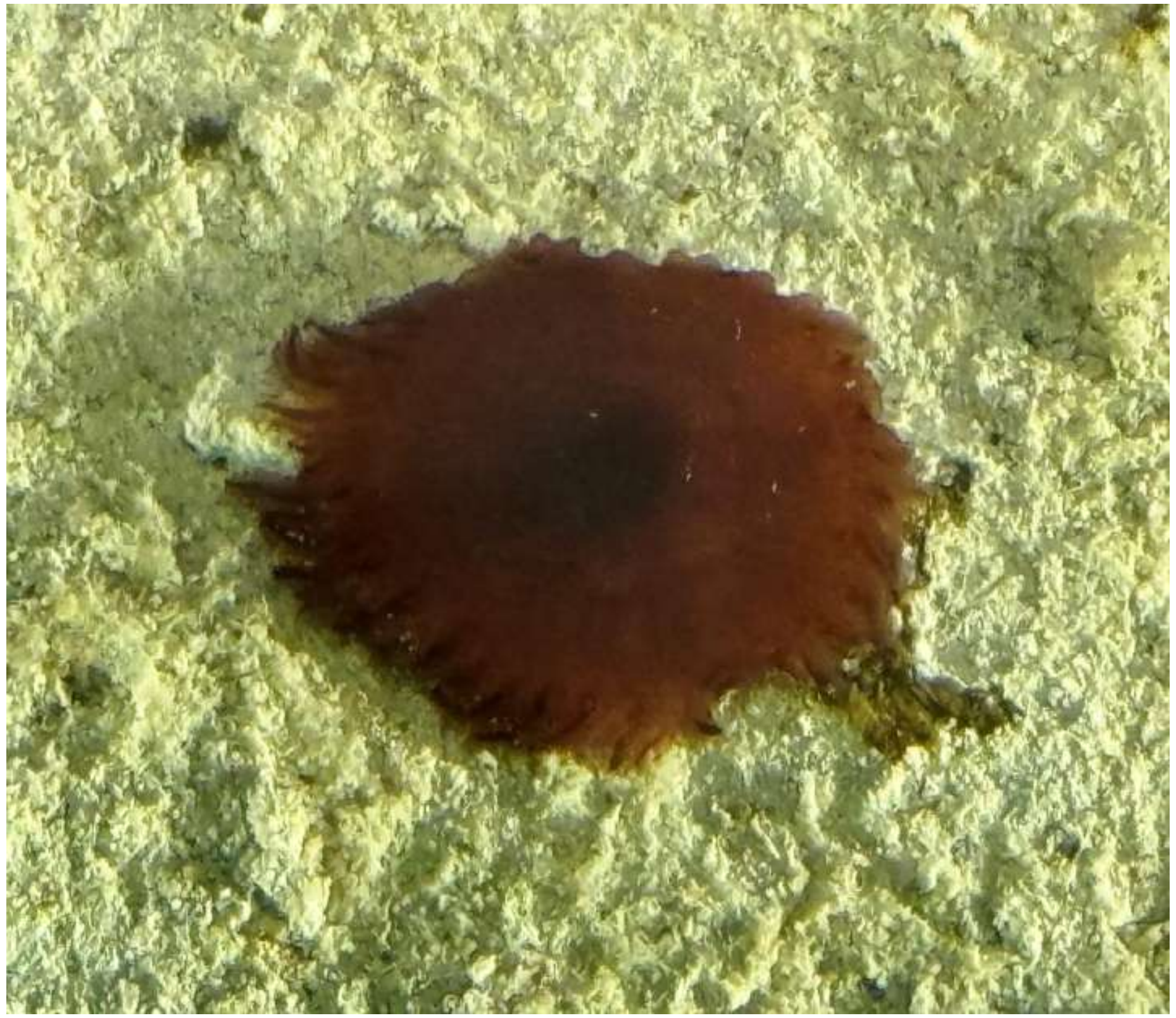

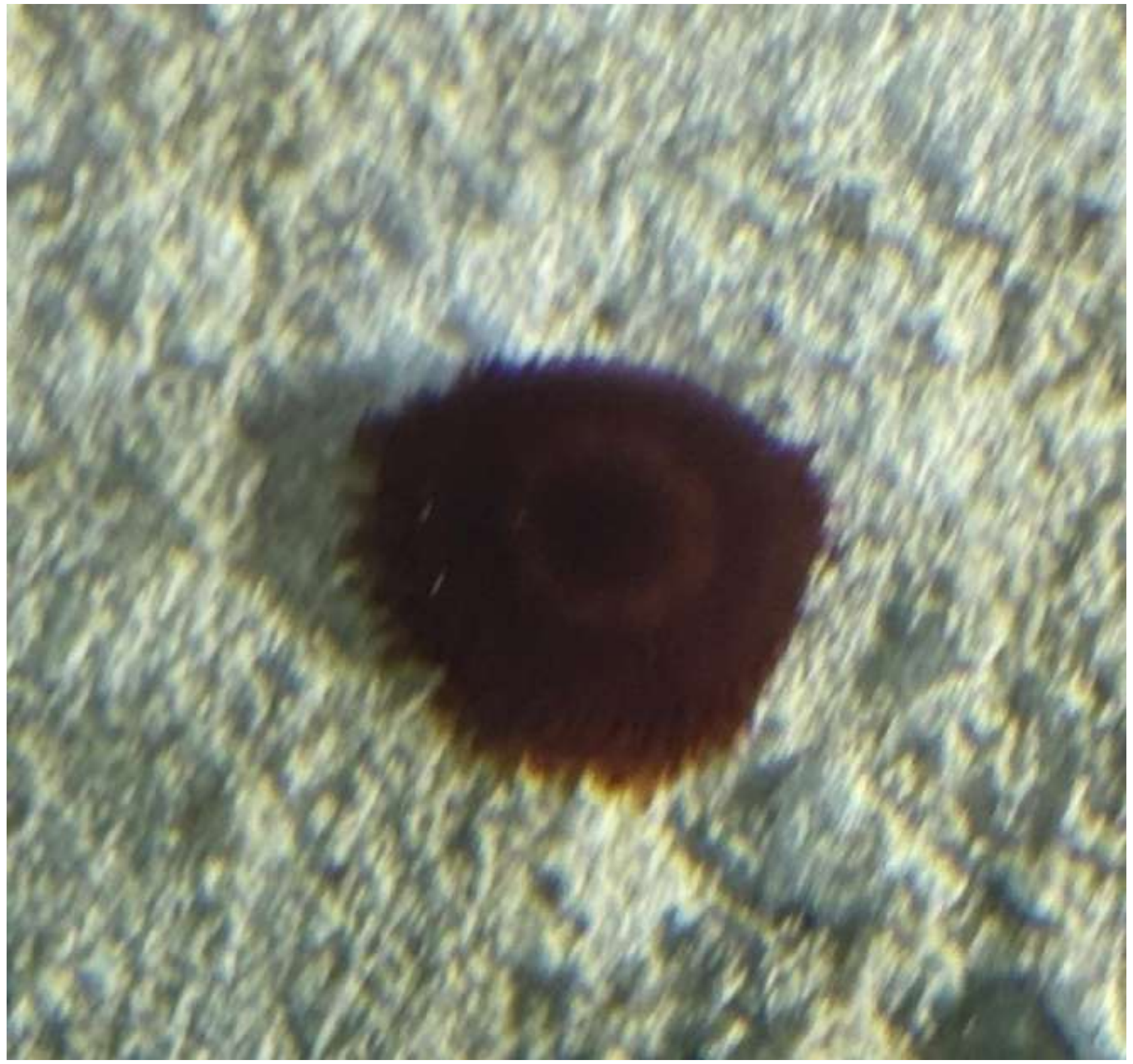

Bell is dark red-brown and with stomach arms short and tentacles absent. Radial canals appear as a dark mass that forms at the top of the bell.

Hydrozoa; Trachymedusae; Trachymedusae spp.

NC4_OM10_5300_NOR_0025

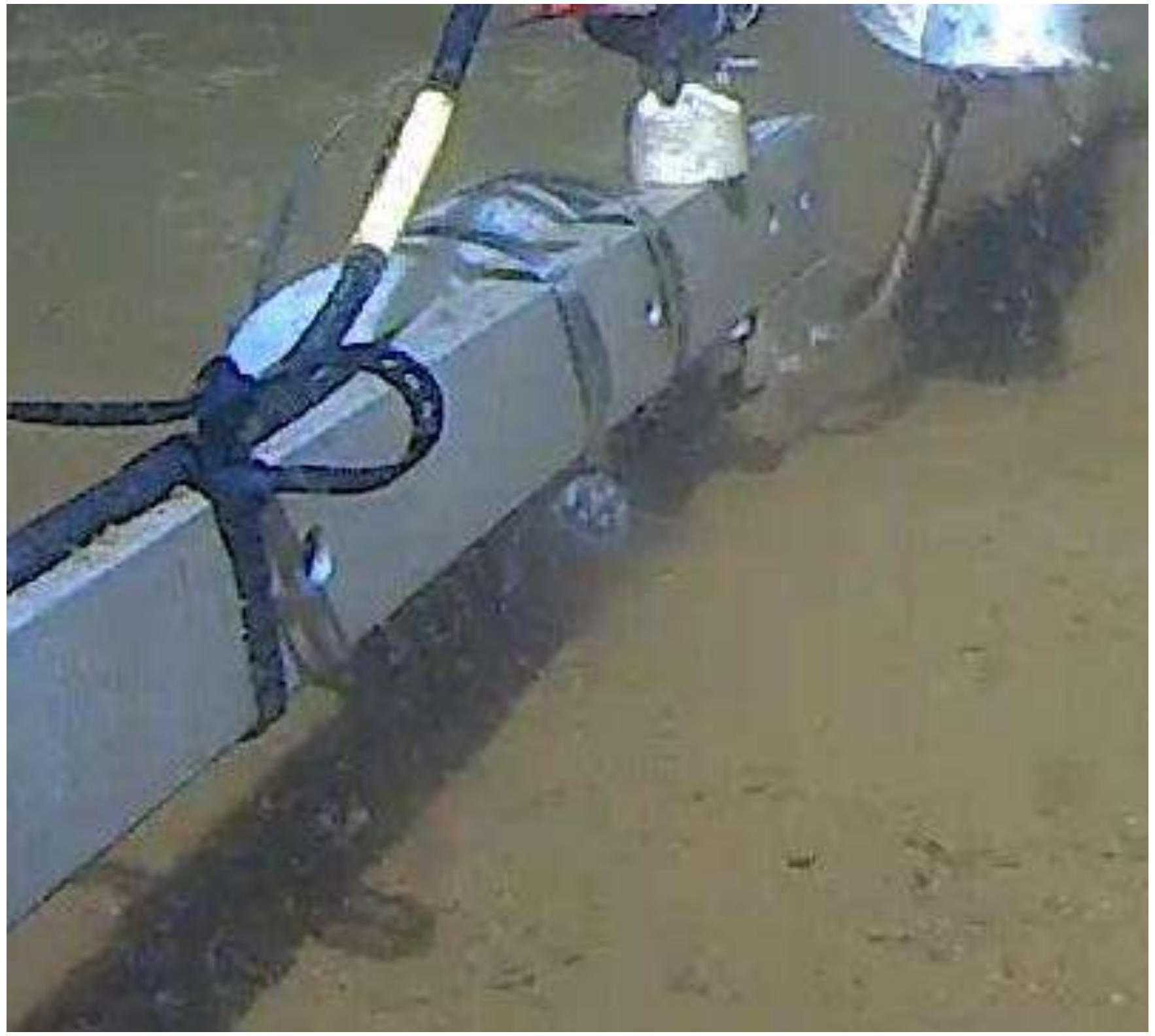

Bell is translucent. Radial canals appear 6-8 white masses that encompass the bell.

Hydrozoa; Trachymedusae; Trachymedusae gen. indet. 1

NC4_BK6_4700_NOR_0051

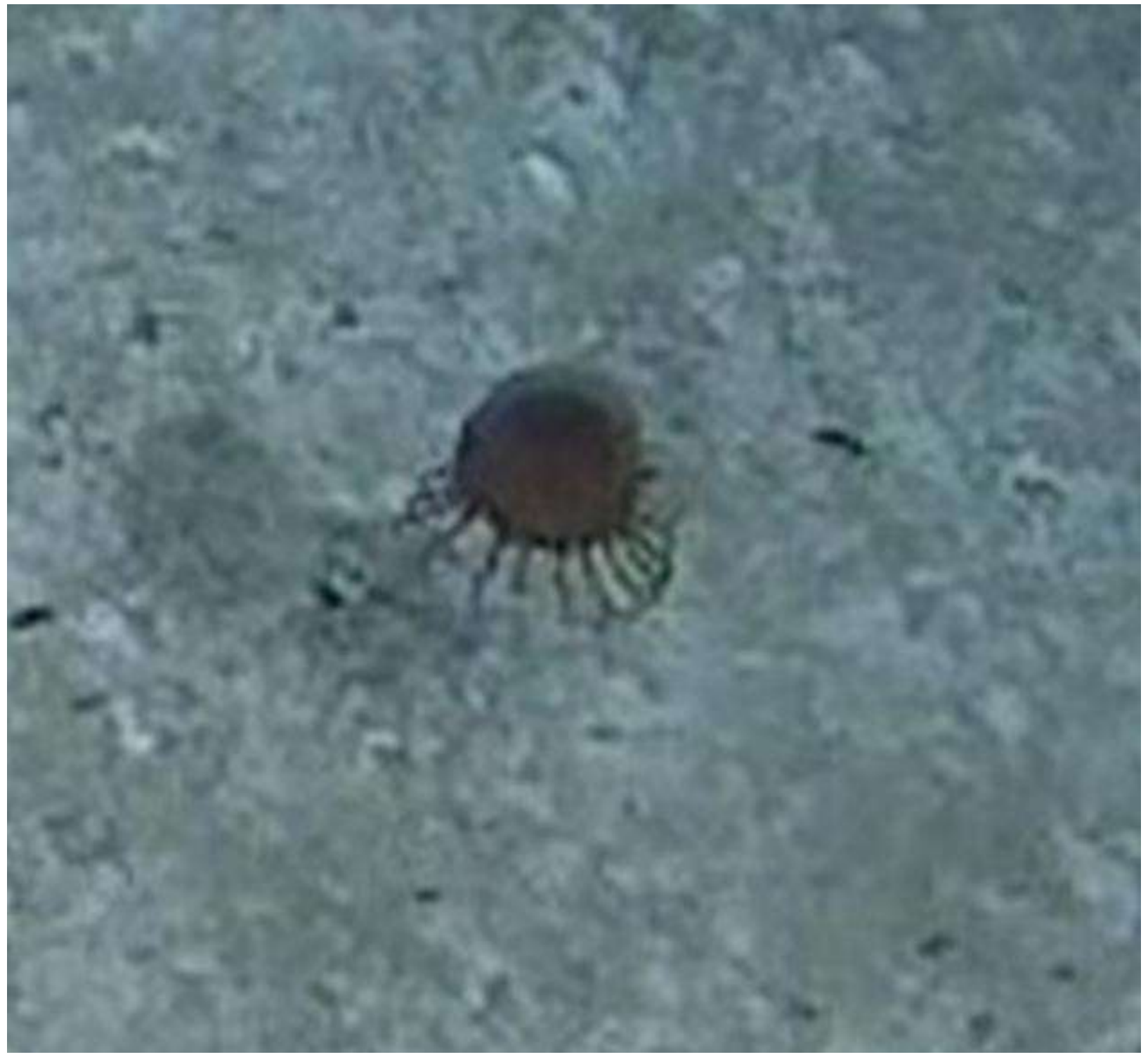

Bell is semi-translucent, red and domed. Reddish lobes apparent around the bell with bright radial canals present around the bell . The bell margin is lined with thick tentacles of moderate length.

Hydrozoa; Trachymedusae; Rhopalonematidae; *Arctapodema* sp. indet. 1

NC4_OM9_5700_NOR_0038

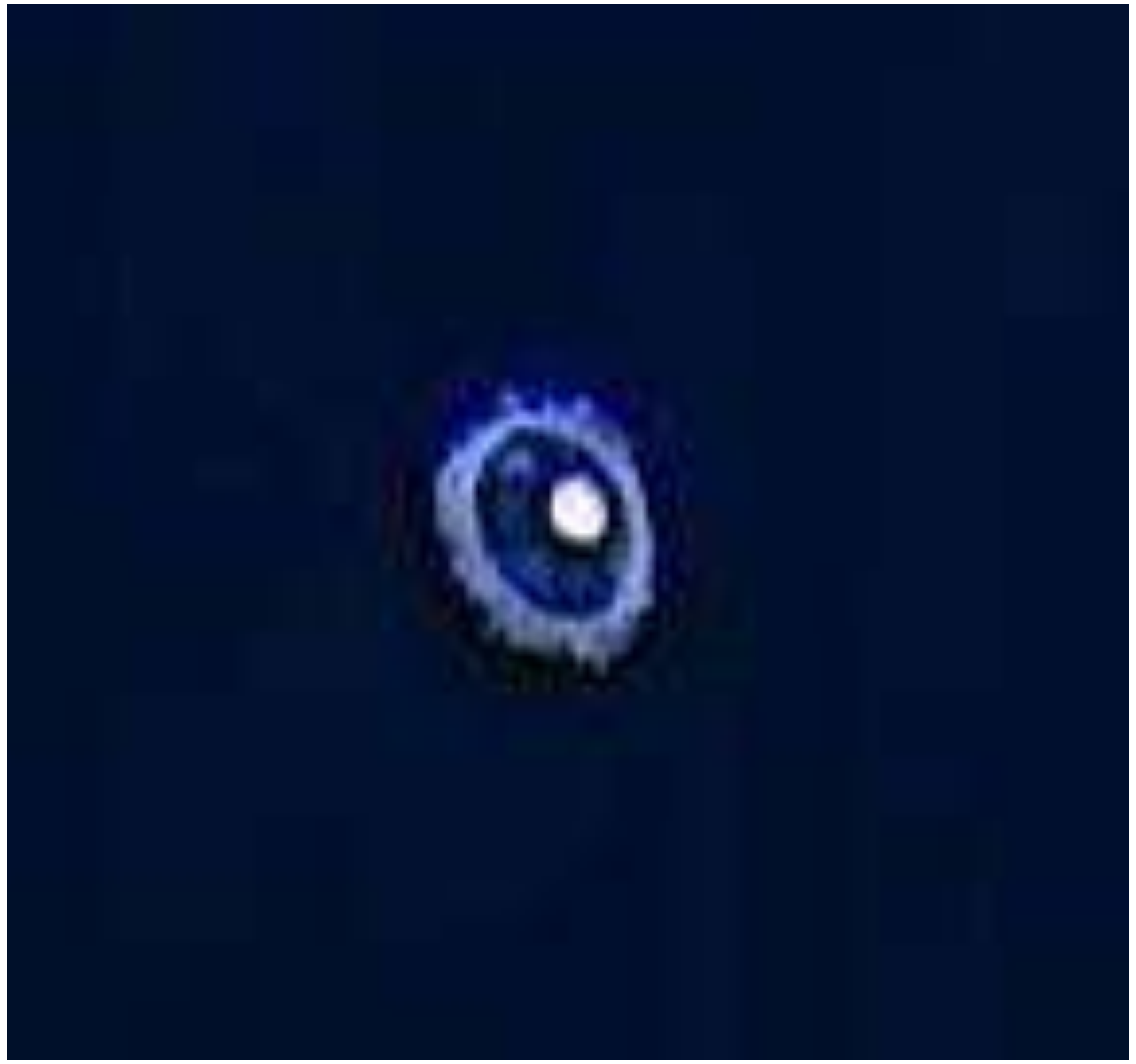

Bell is translucent and held in a wider, flatter position. With faint 6-8 radial canals observed around the bell. The bell margin is lined with small, thick, marginal tentacles.

Behavioural diagnostic feature: Swimming pattern is sporadic and irregular.

Hydrozoa; Trachymedusae; Rhopalonematidae; Rhopalonematidae spp

NC3_OM7_3500_NOR_0045

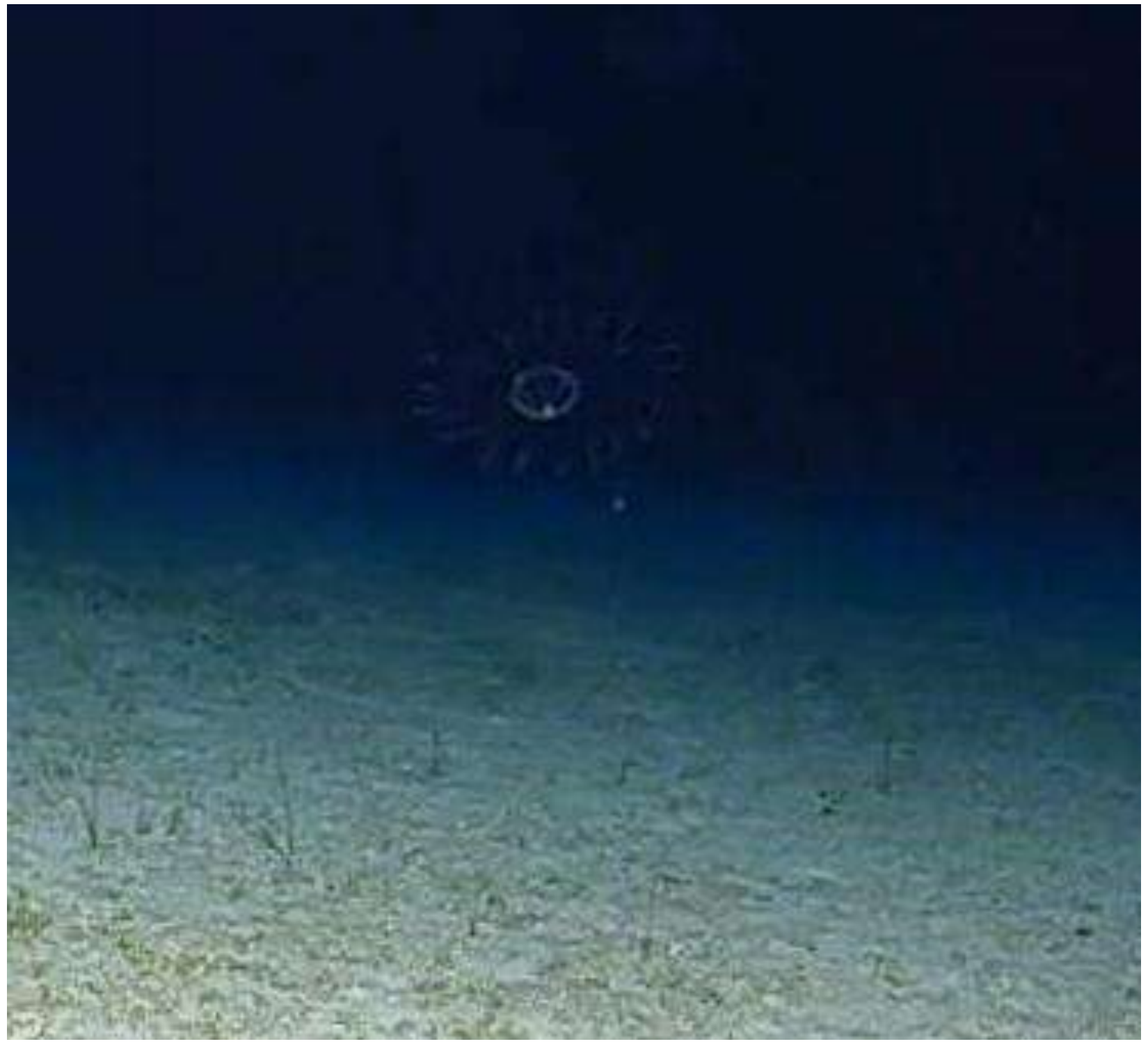

Bell is translucent. Faint radial canals observed around the bell. The bell margin is lined with long, thin marginal tentacles.

Hydrozoa; Trachymedusae; Rhopalonematidae; Rhopalonematidae sp. indet. 1

NC1_OM2_6000_NOR_0130
NC2_MA6_5700_NOR_0035
NC4_OM11_4500_NOR_0114

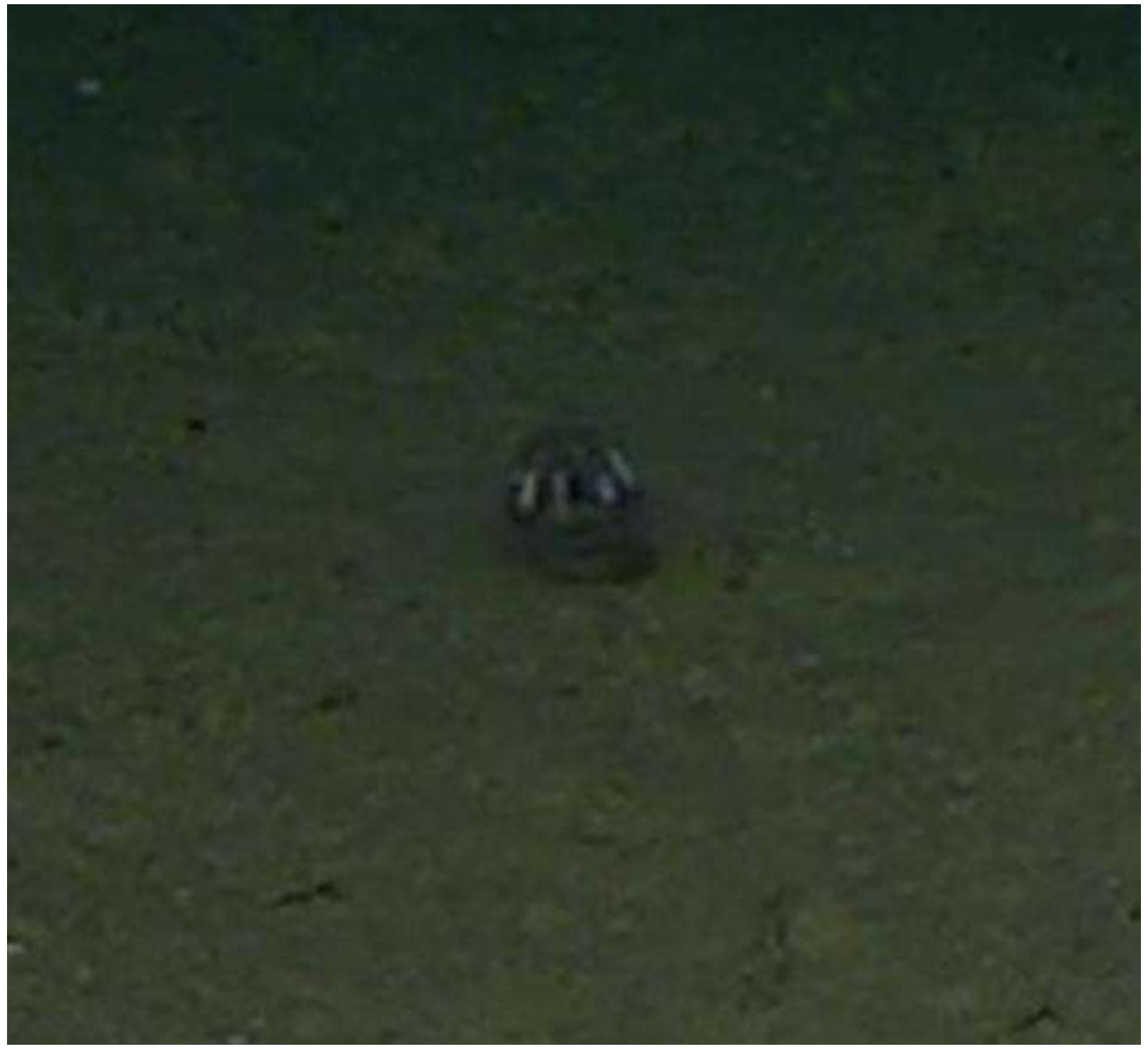

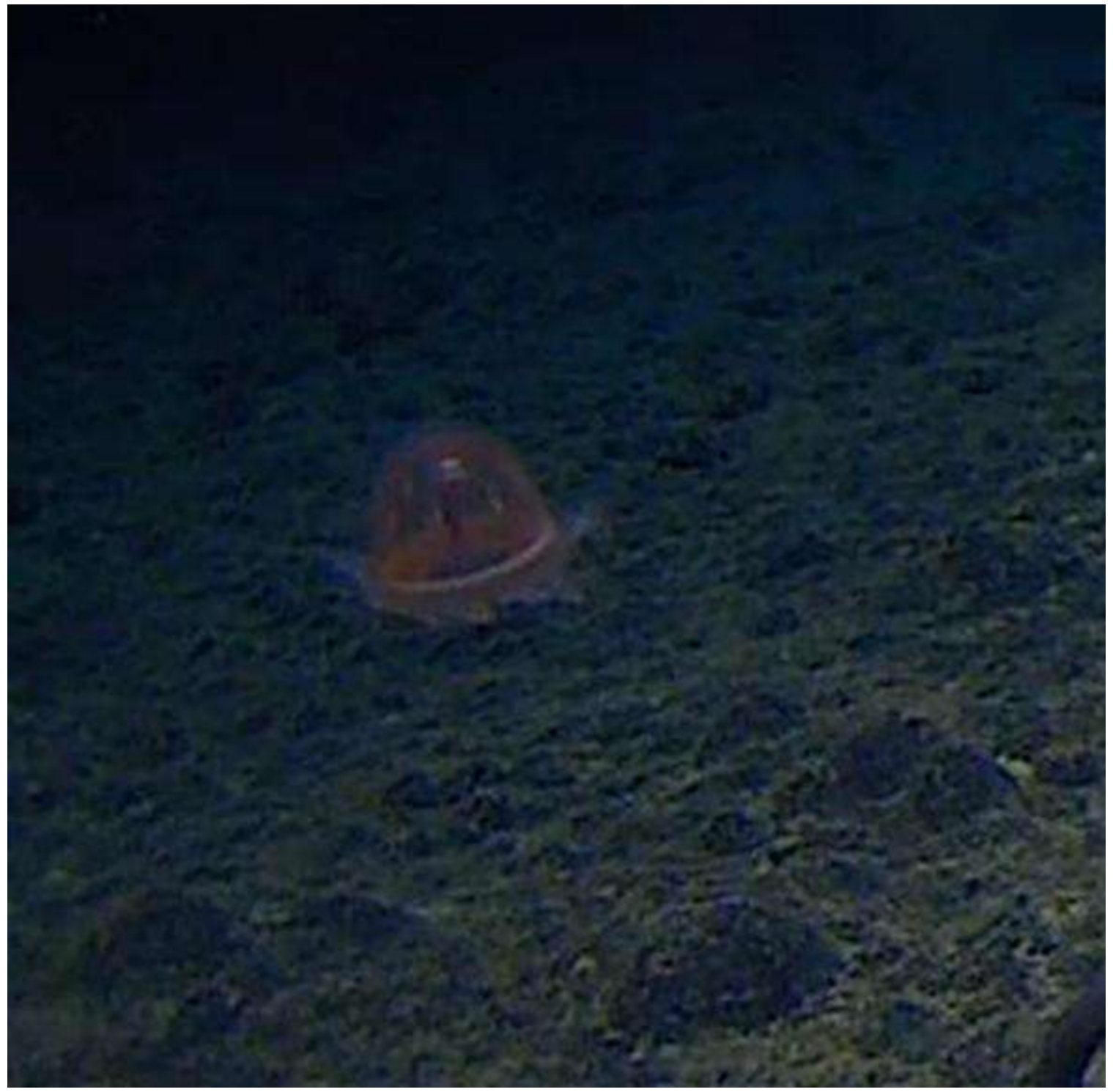

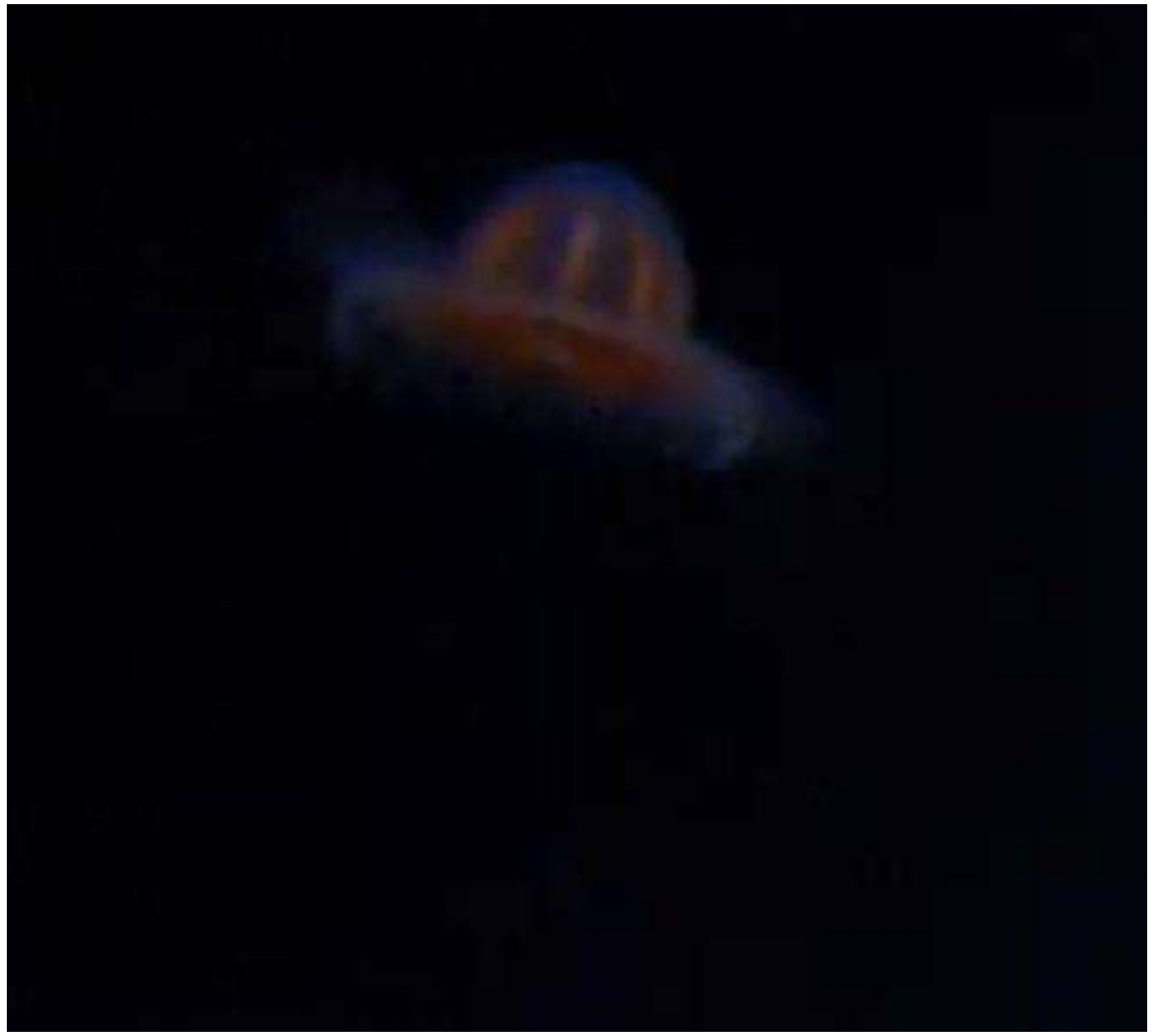

Bell is semi-translucent, red and domed. Reddish lobes apparent around the bell with bright radial canals present around the bell . The bell margin is lined with thick tentacles of moderate length.

Hydrozoa; Trachymedusae; Rhopalonematidae; Rhopalonematidae gen. indet. 2

NC3_OM1_5600_NOR_0078
NC4_CR4_5100_NOR_0059
NC1_CR1_6200_NOR_0058

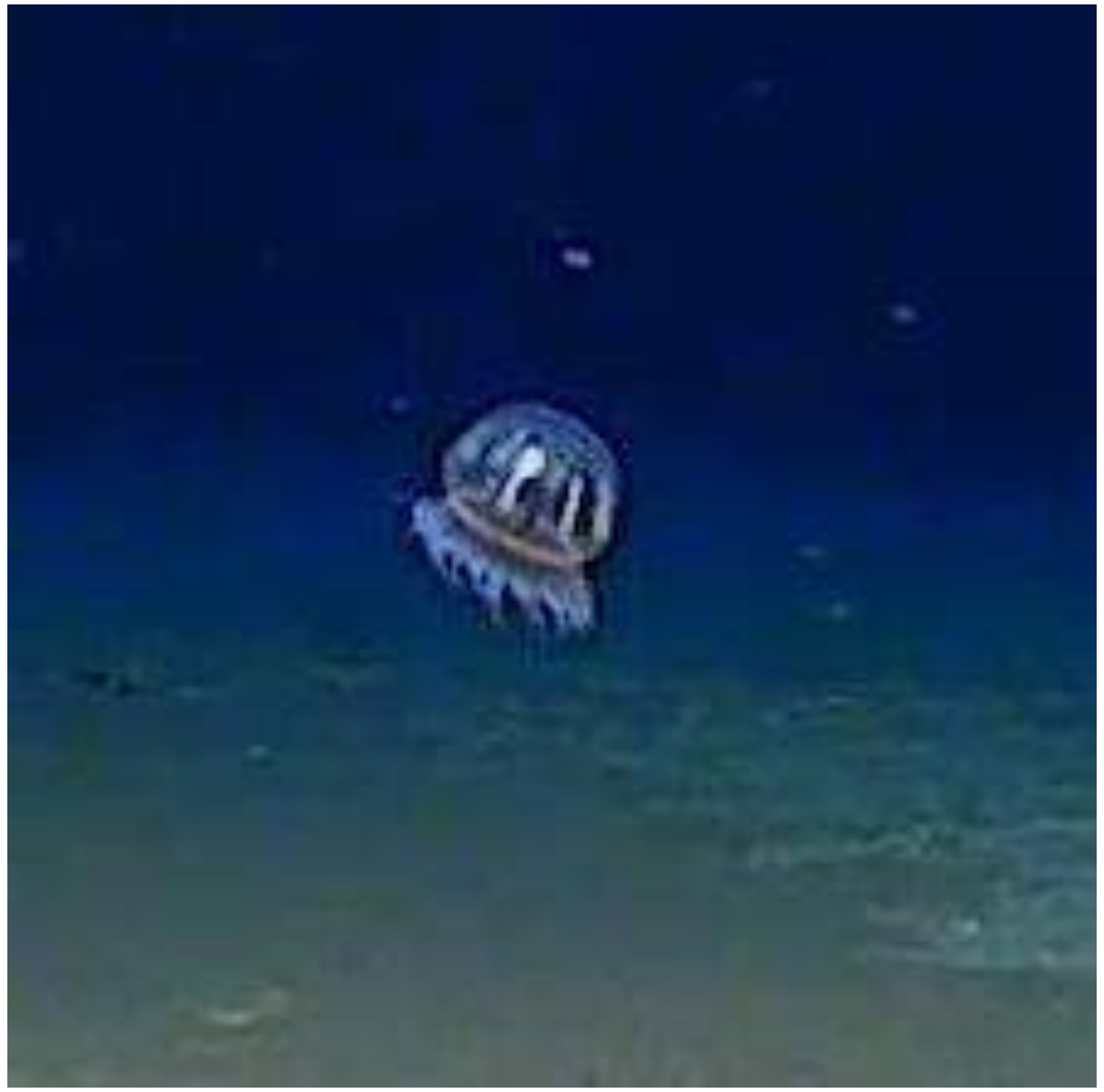

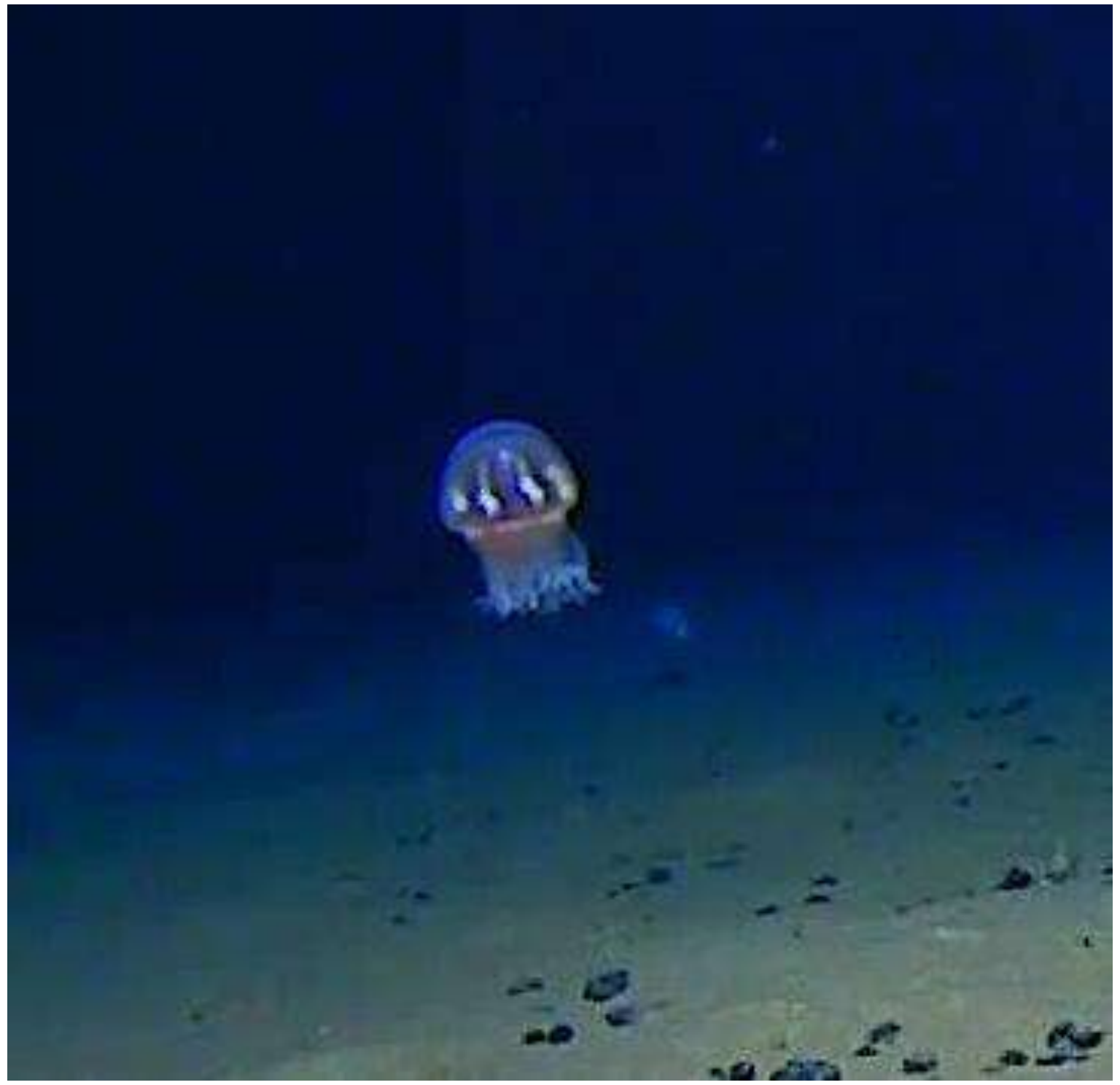

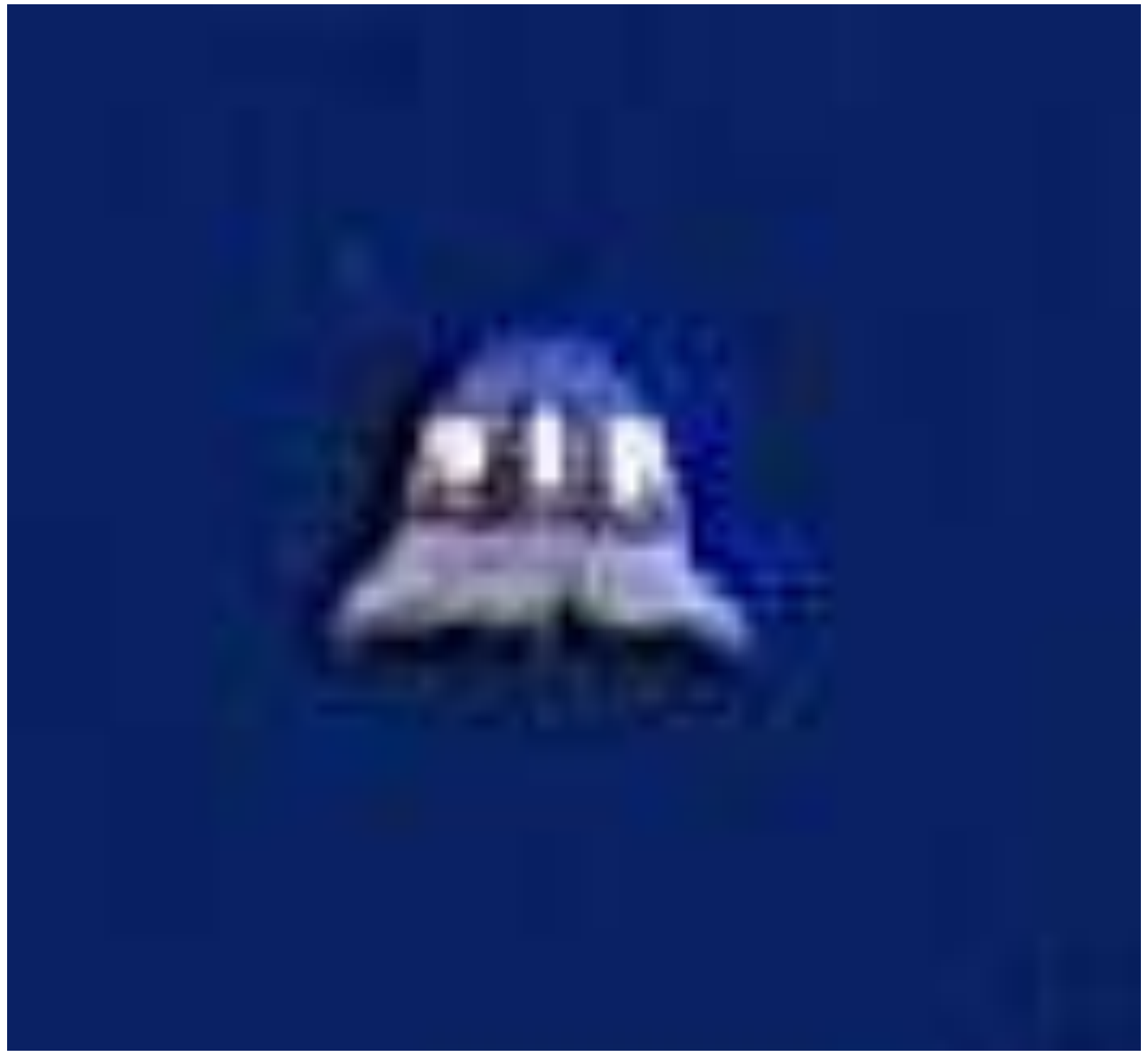

Bell is translucent, domed and bears a reddish tint. Bright radial canals present around the bell . The bell margin is lined with a thick mass of tentacles of moderate length.

Hydrozoa; Leptothecata; Tiarannidae; Tiarannidae gen. indet. 1

NC4_MA8_5500_NOR_0068
NC4_MA8_5500_NOR_0068

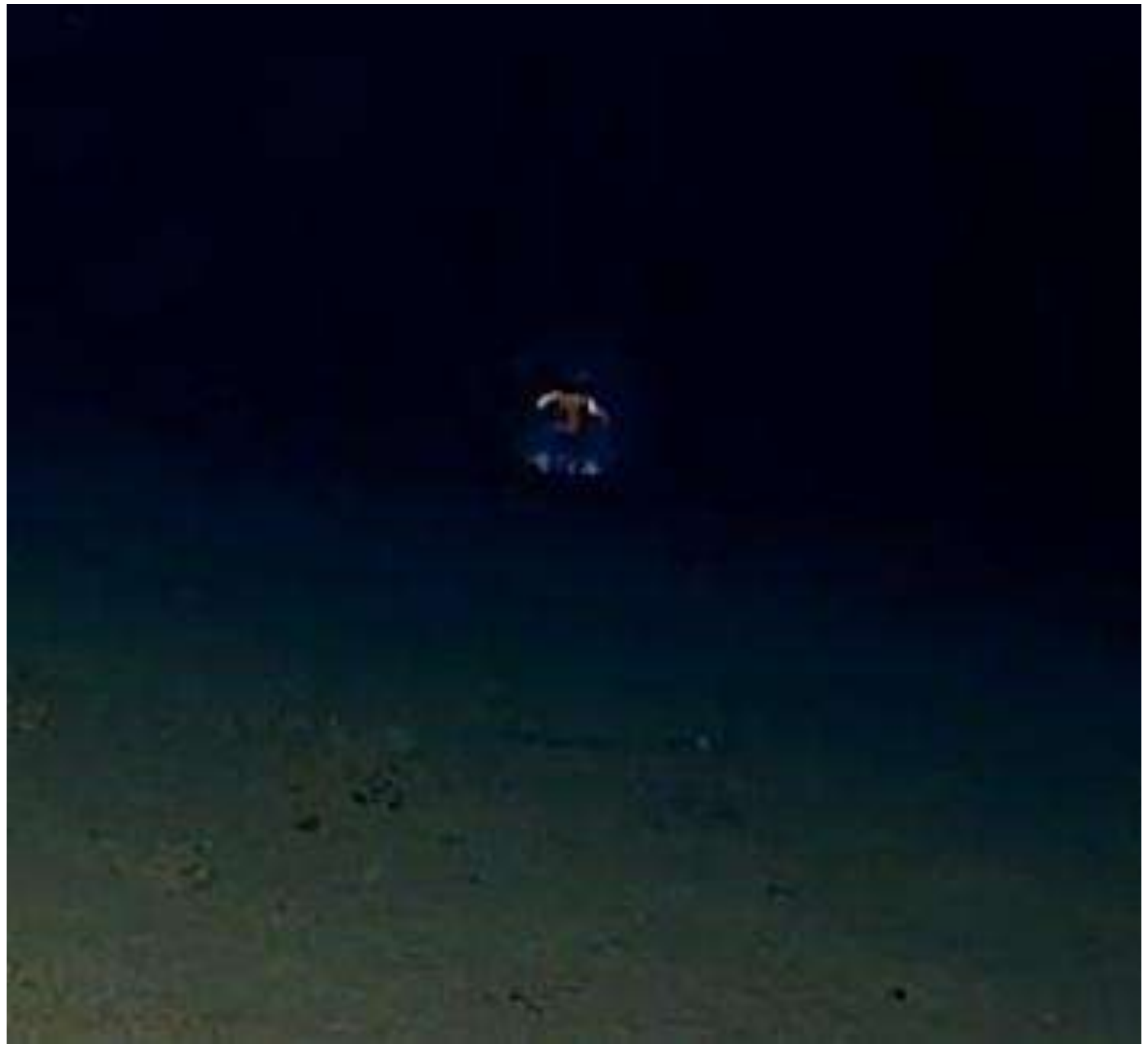
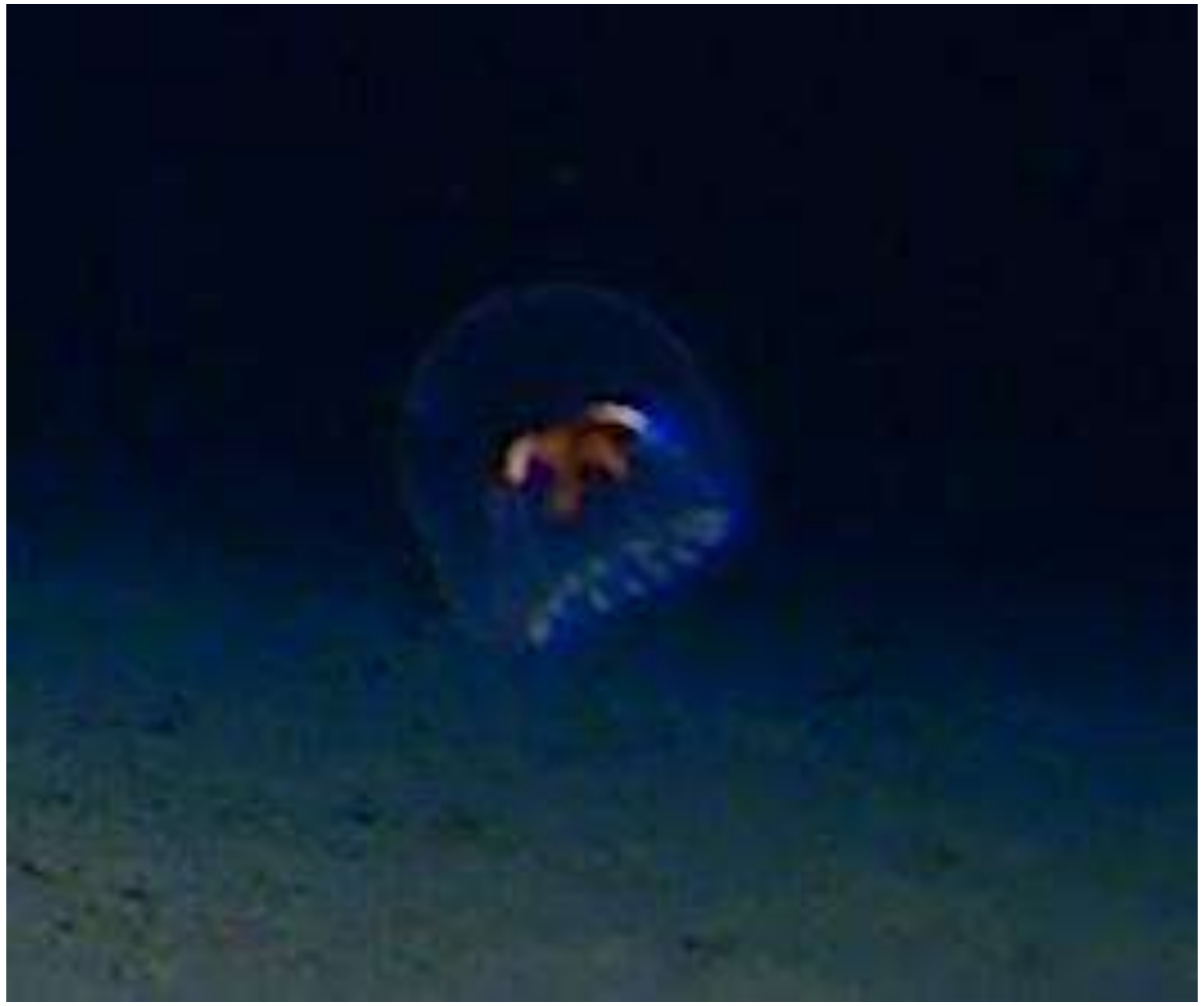

Bell is translucent and domed. Bright radial canals present around the top of the bell . Gonads are bright red. The bell margin lacks apparent tentacles.

See Video Clip: https://uniwa.sharepoint.com/:v:/r/teams/EXT-MUDSRC/Shared%20Documents/Cruises/2024_02_Nova%20Canton/Data/Video%20Analysis/Representative%20images/Cnidaria/Cnidaria_Hydrozoa_Leptothecata_Tiarannidae_NC4_MA8_5500_1_01_NOR_0068.avi_0003890.mp4?csf=1&web=1&e=AfLWI8

Hydrozoa; Siphonophorae; Calycophorae; Calycophorae sp indet. 1

NC2_OM1_3100_NOR_0094

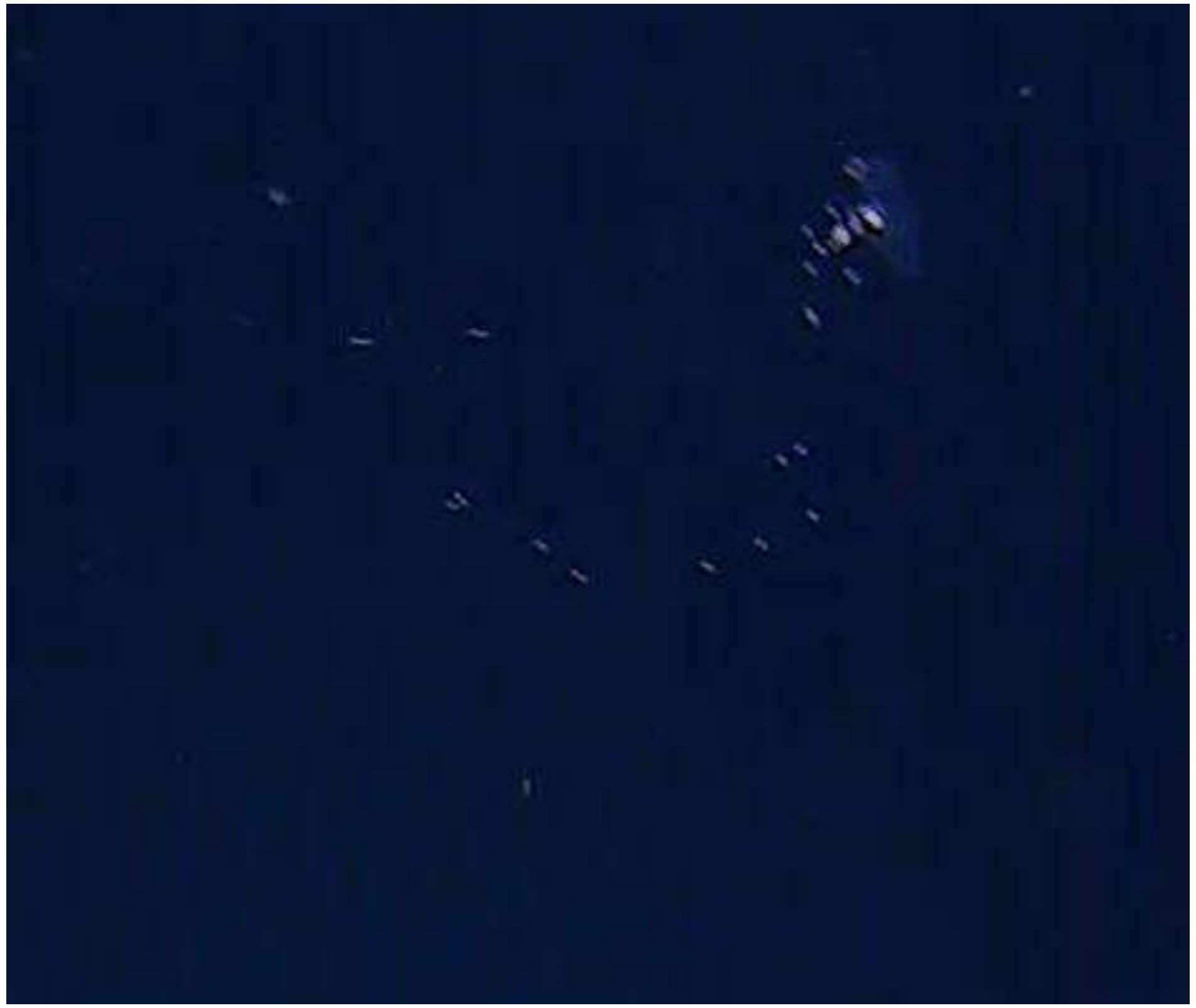

Body is translucent with multiple bright coloured nectophores along the body. Possible pneumatophore present at anterior end. Possibly the genus Kephyes or Clausophyes based on the 45-degree orientation of the siphosome.

Ctenophora

Tentaculata; Cydippida; Cydippida fam. indet. 1

NC2_BK3_7400_IMG_8677
NC2_BK3_7400_IMG_8678

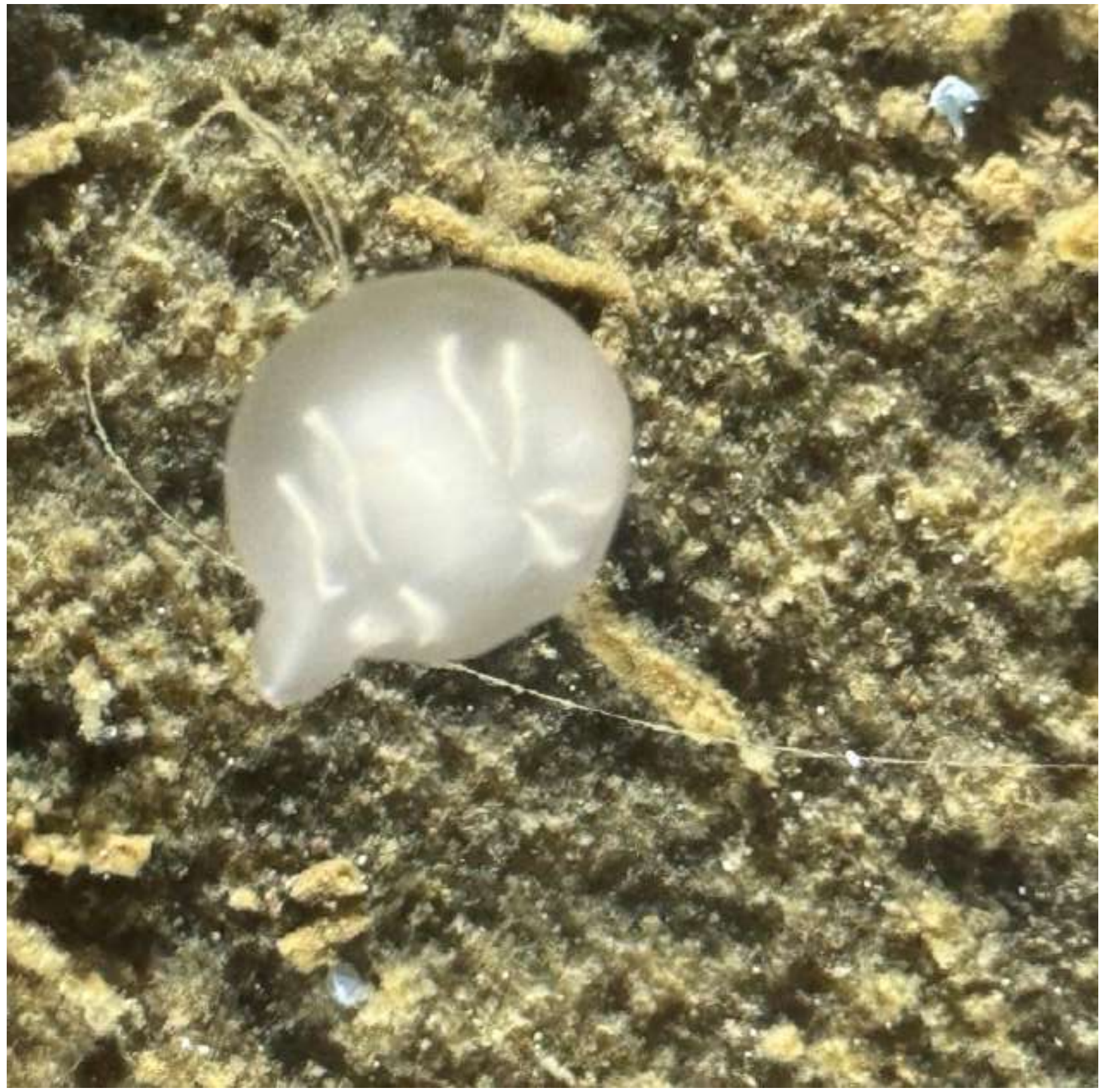

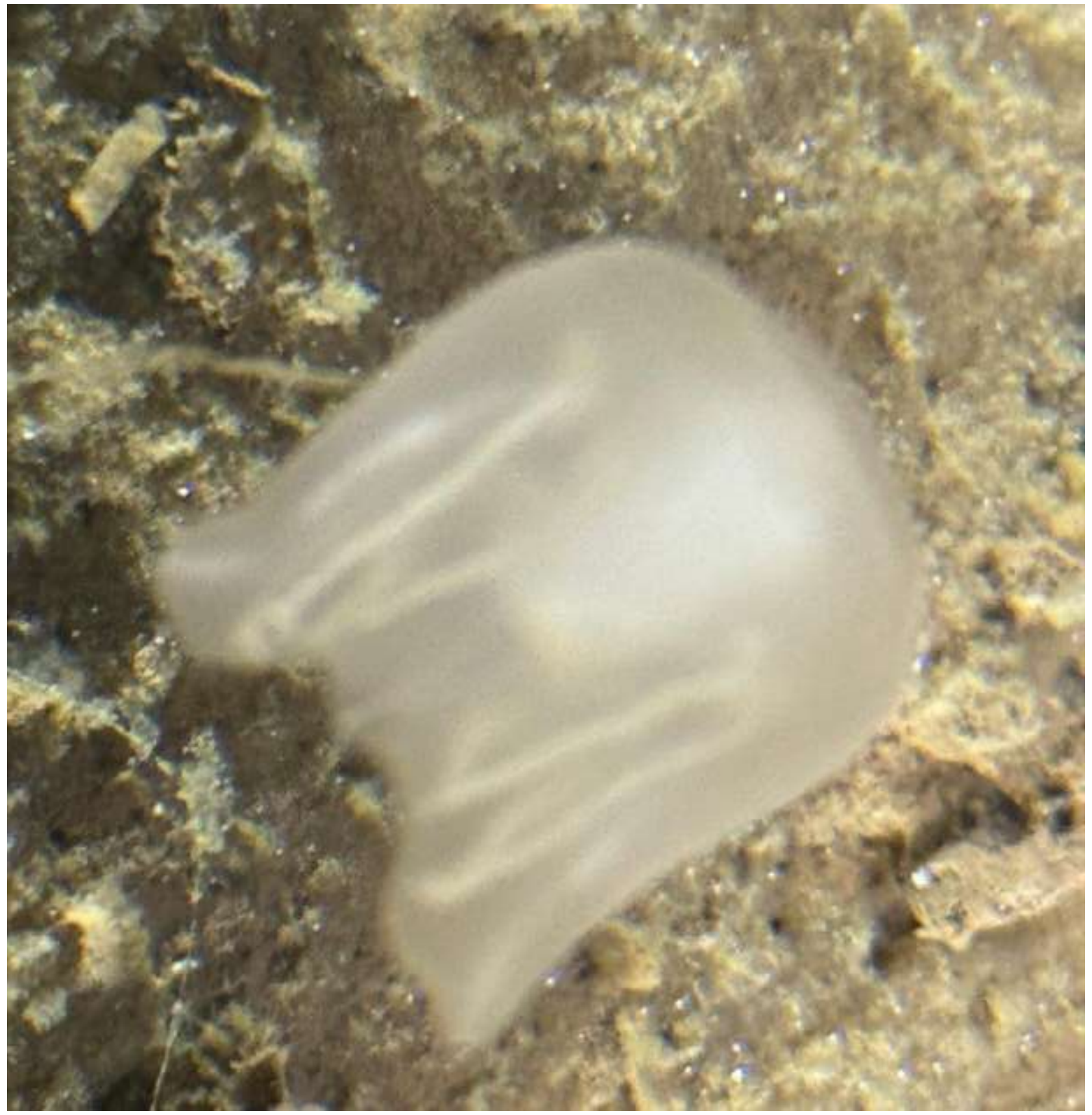

Bell is transparent and generally round with a flattened "brim" extending into two rounded points. 1- 2 tentacles long and trail behind the bell.

Tentaculata; Cydippida; Cydippida fam indet. 2

NC2_BK3_7400_IMG_8981
NC4_OM5_6900_NOR_0084

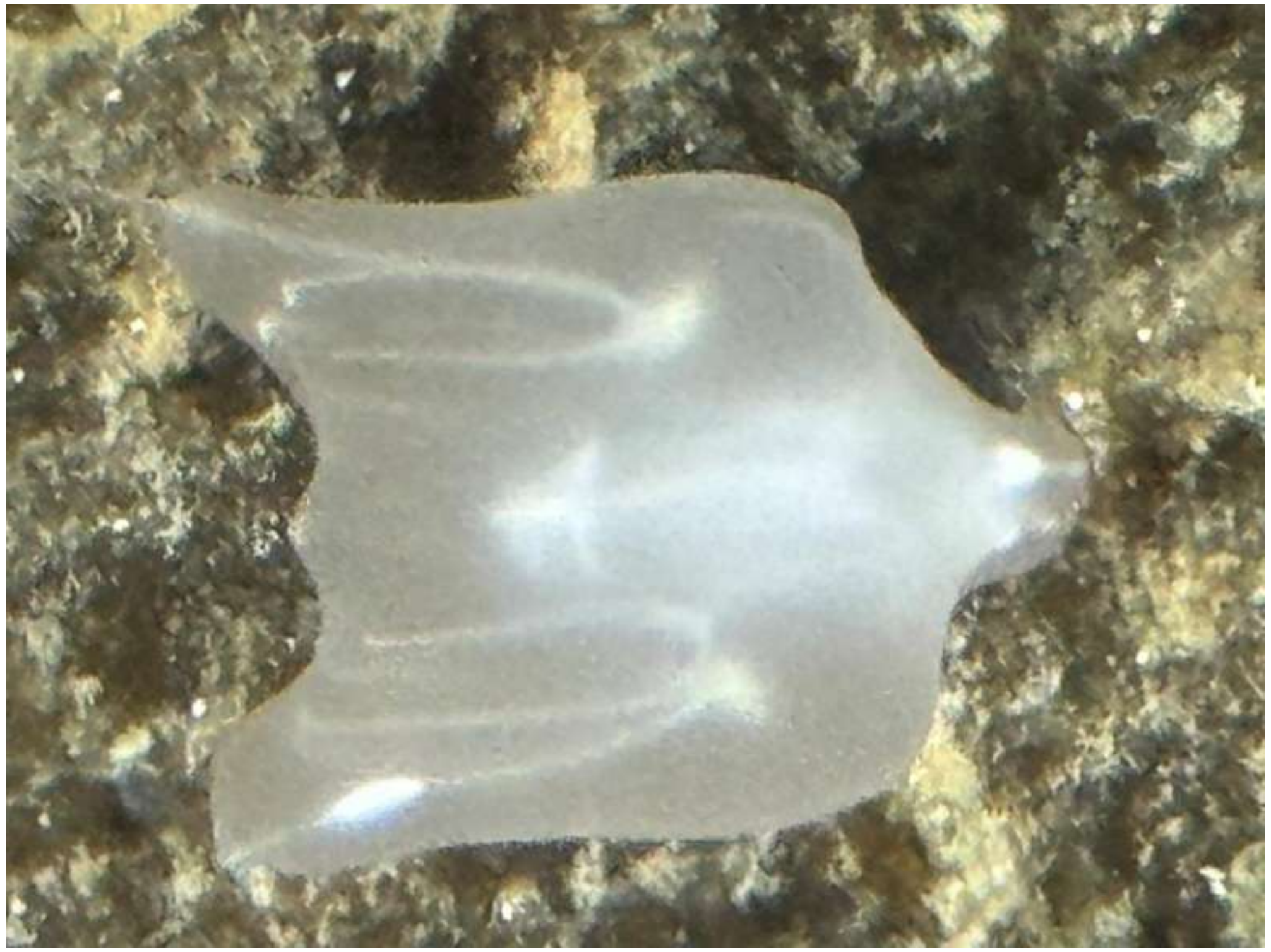

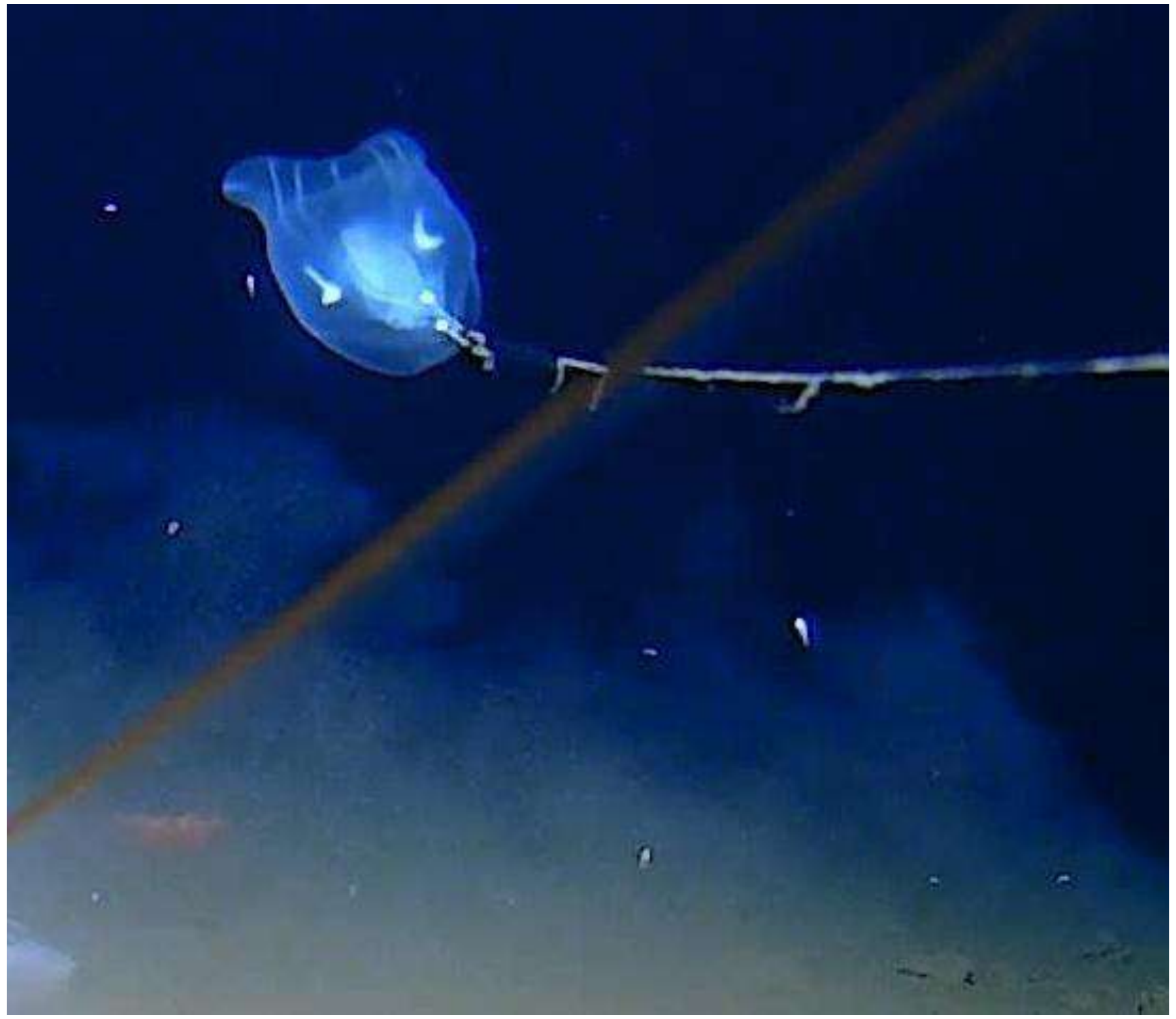

Bell is transparent and generally square with a flattened "brim" extending into two rounded points. Opposite end also has a rounded point-like protrusion. 1- 2 tentacles long and trail behind the bell.

NC1_CR8_7000_NOR_0065

Tentaculata; Cydippida; Cydippida fam indet. 3

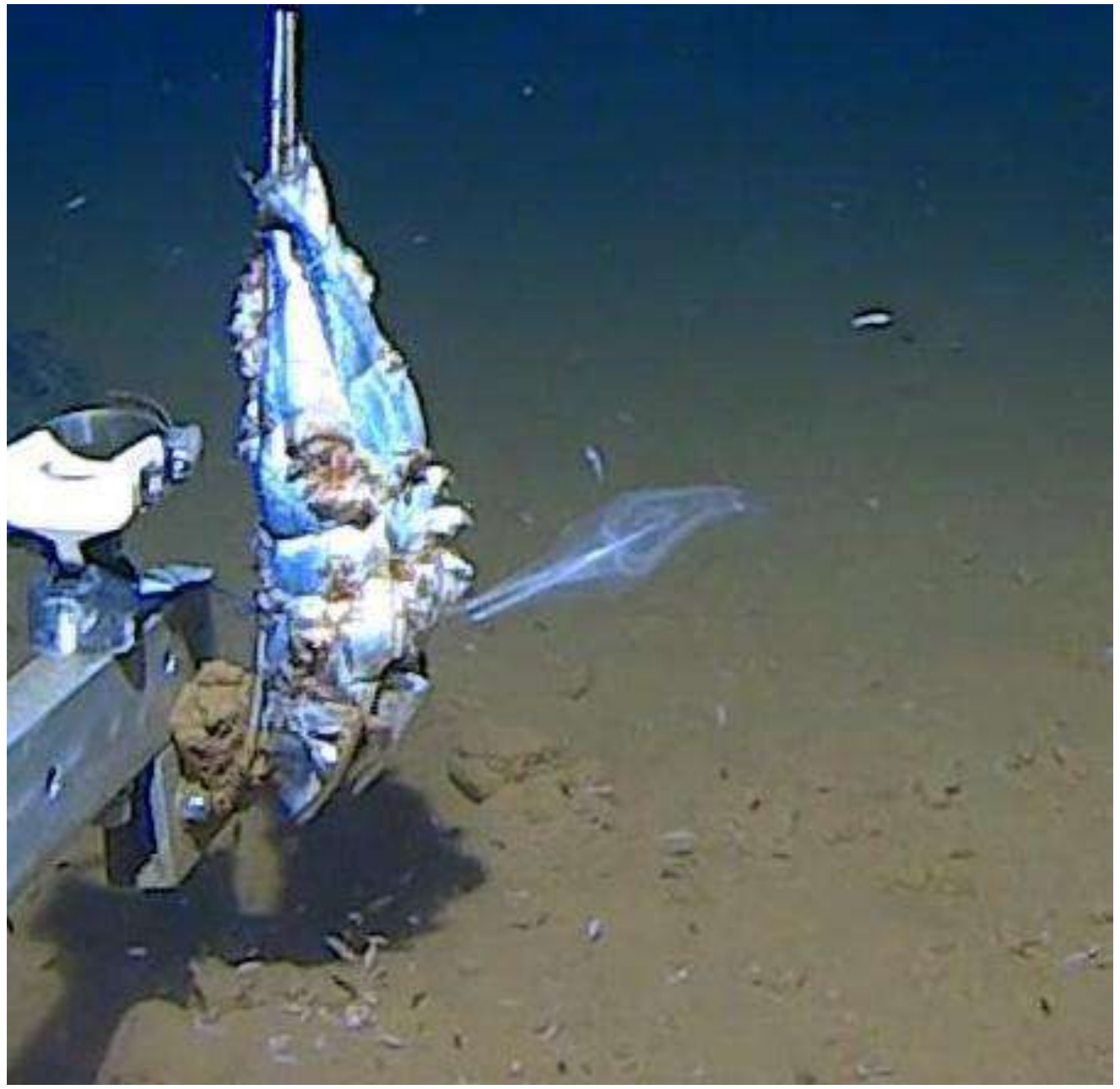

Bell is transparent and generally rhomboid and flattened. No tentacles apparent.

Tentaculata; Cydippida; Cydippida fam indet. 4

NC1_BK2_6700_IMG_5061
NC1_BK1_6200_IMG_0063

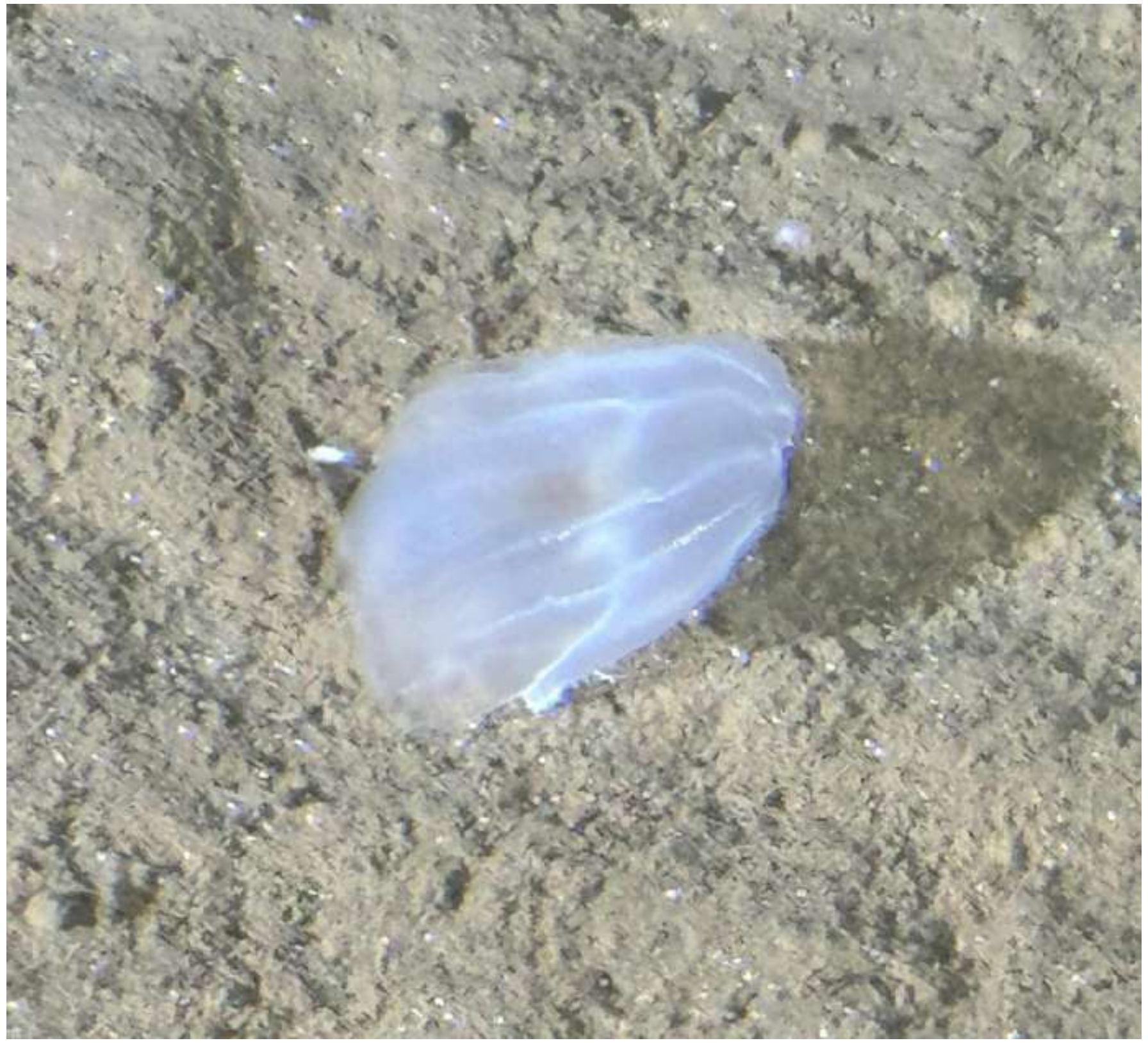

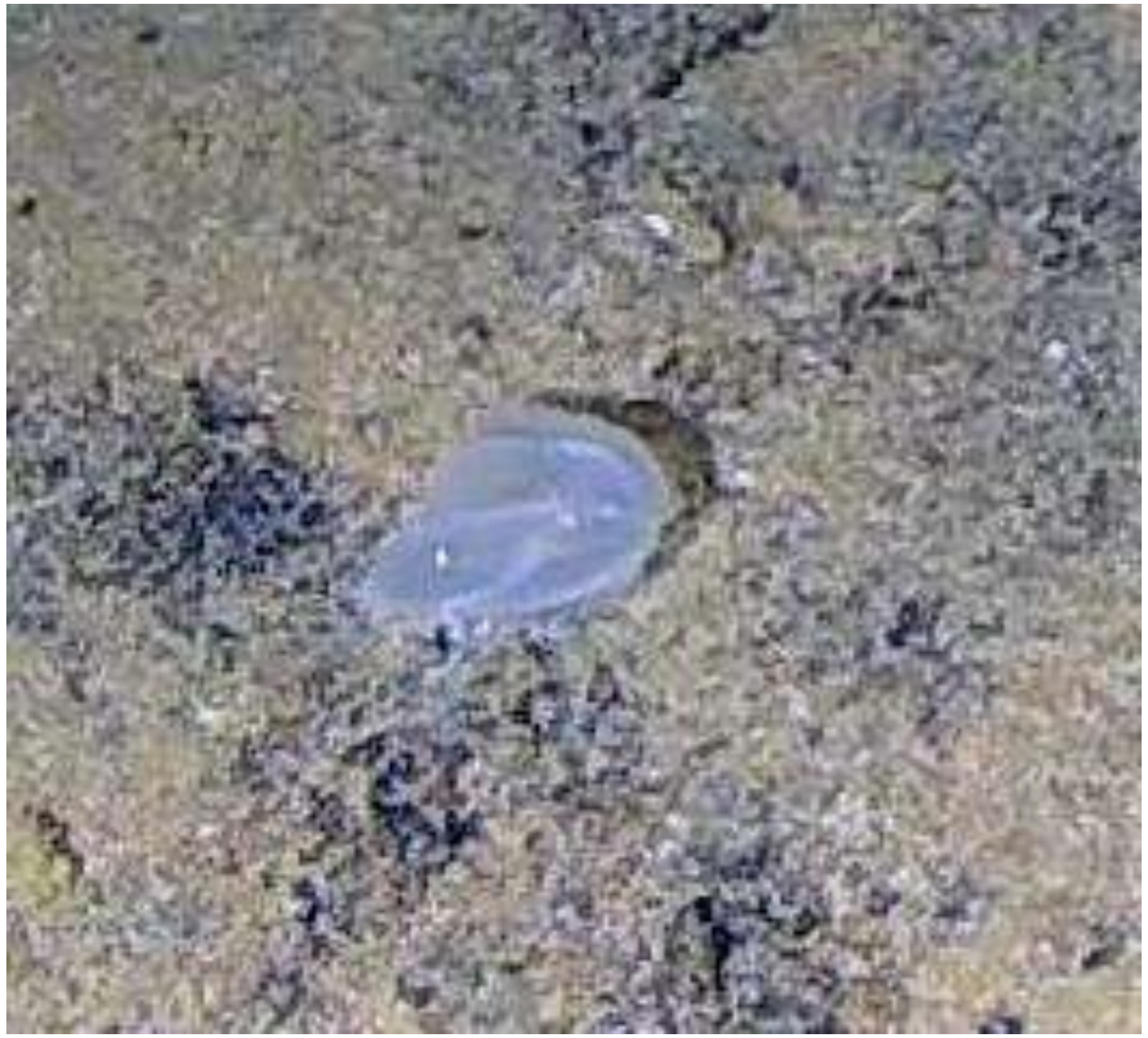

Bell is transparent and generally ovoid in shape. No tentacles apparent

NC3_OM6_4600_NOR_0086

Tentaculata; Cydippida; Cydippida fam inc. 5

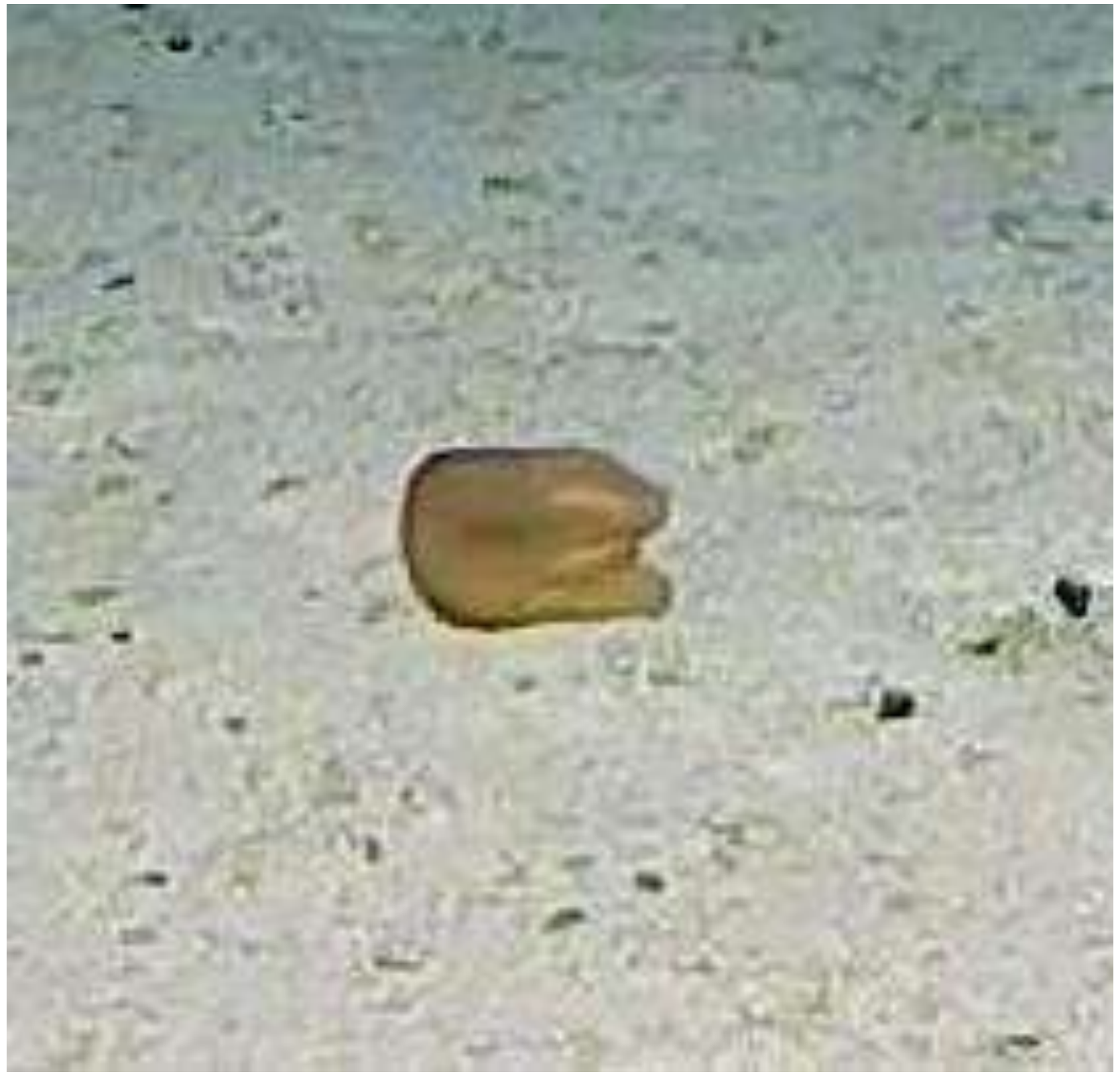

Bell is transparent brown and generally round with a flattened "brim" extending into two rounded points. No tentacles apparent

Porifera: Demospongiae

Porifera, Demospongiae
Demospongiae ord. inc. 1

NCT3_BK2_6000_NOR_0074 – 03:04

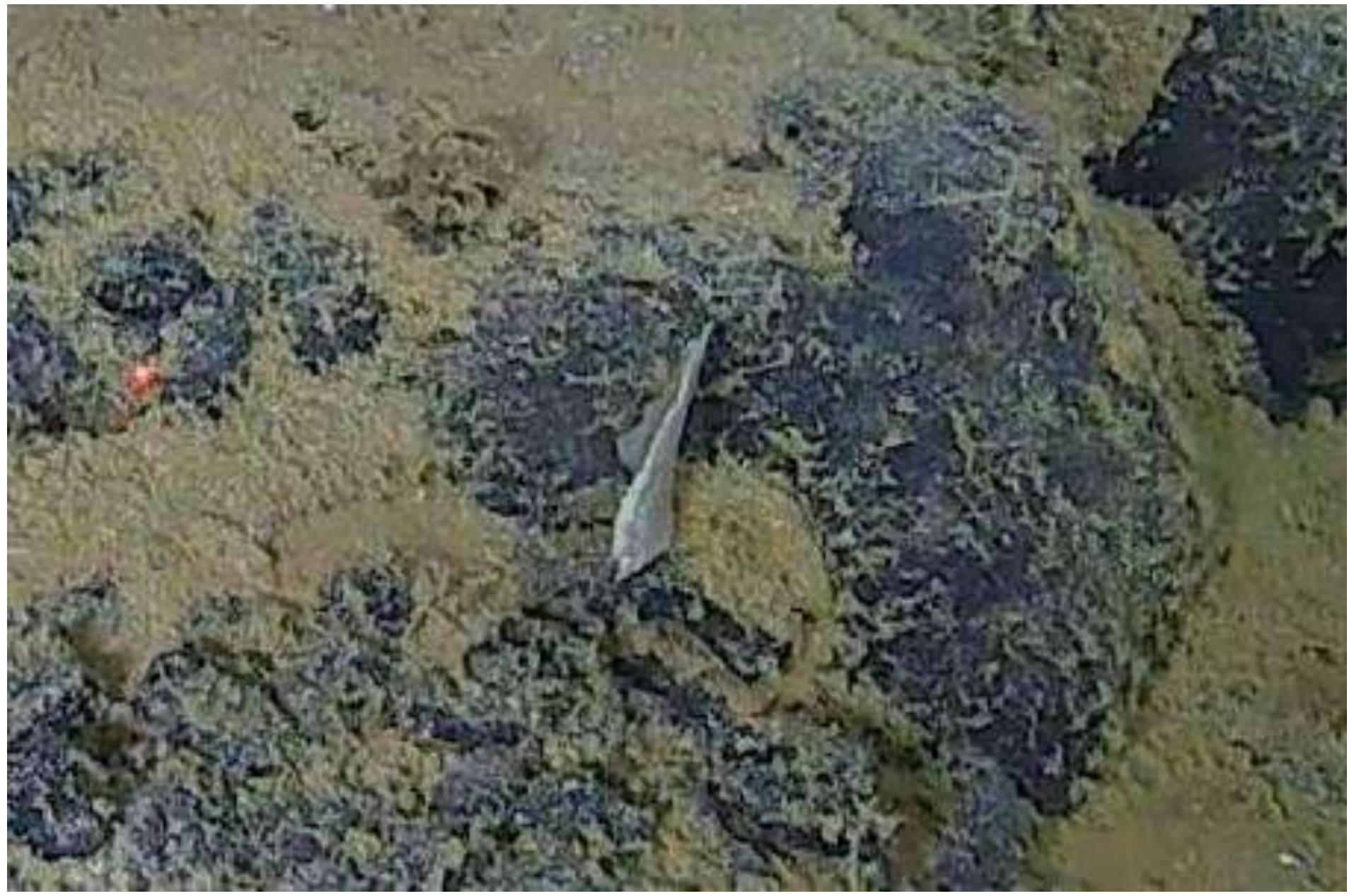

Fan shaped sponge showing an opaque white color, observed on rocks. Tetractinellida or Poecilosclerida.

Demospongiae, Poecilosclerida
Cladorhizidae spp.

NC2_BK5_4600 – NOR_0060 ~ around 02:20

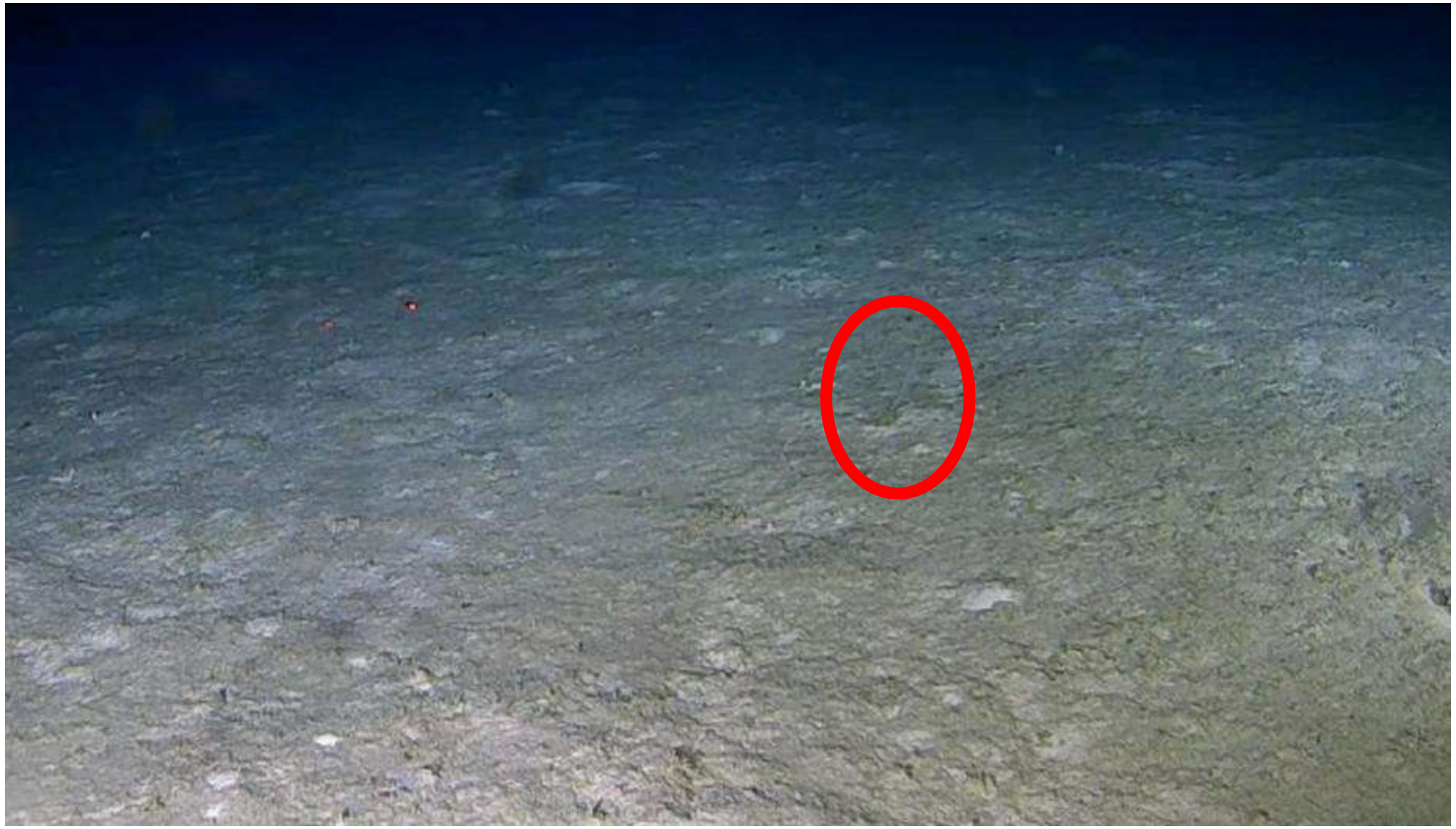

Erect thin specimen who lives on soft sediments. Body is white/bluish, with a hairy aspect given from the sponge feeding spines. Observation of "sticks" with similar size, but brownish/yellowish can represent either recently dead specimen or polychete tubes (in this case, for sabellids check the tip, a small white/bluish tuft made by the termination of the crown should be visible. As the organization of the spines is unclear in these observation, they are not categorized as "Cladorhizidae gen. indet. 1" but rather binned into this.

Demospongiae, Poecilosclerida
Cladorhizidae gen. indet. 1

NCT2_BK3_7400 - IMG_8578

NC1_BK2_6700

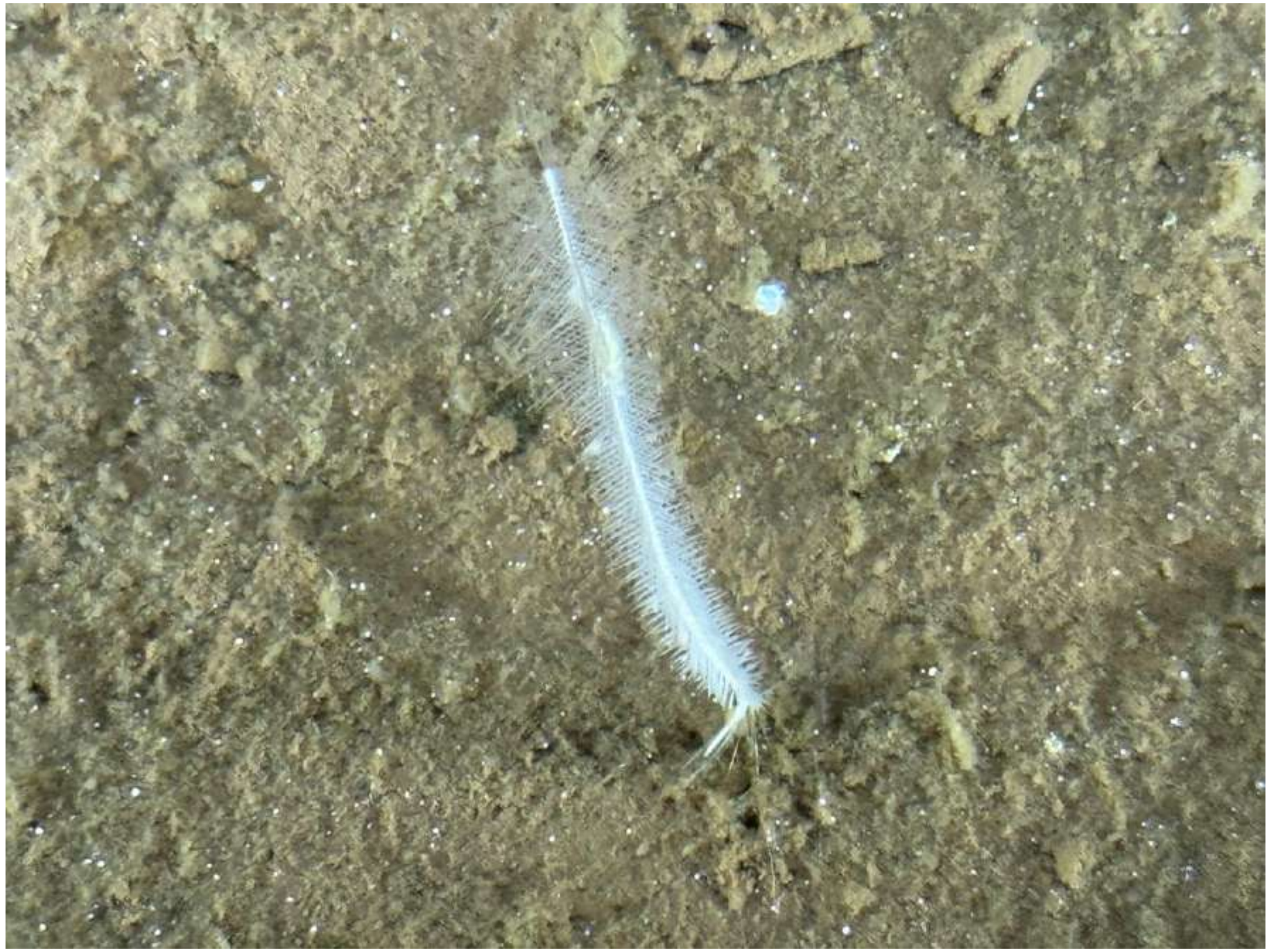

Erect thin specimen who lives on soft sediments. Body is white/bluish, with a hairy aspect given from the sponge feeding spines. The spines are organized in 4 lines with a 90-degree angle between them. *Asbestopluma*-like. Not in AM Matrix.

Demospongiae, Poecilosclerida
Cladorhizidae gen. indet. 2

NCT3_BK2_6000 - IMG_2214

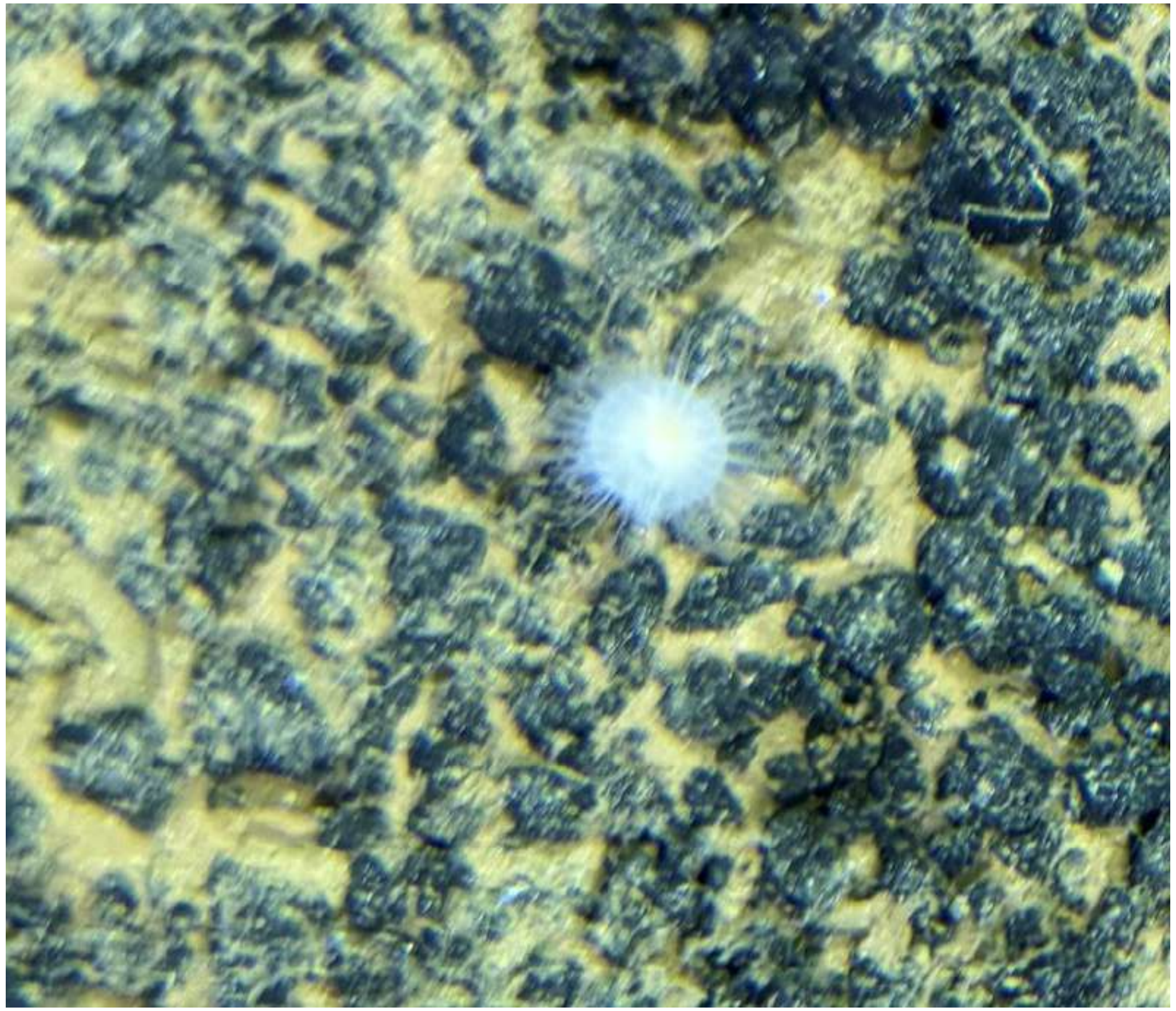

Erect sponges with a spherical body for buoyancy on top of a short stalk. From the main body multiple spines depart towards all direction, more or less on a horizontal plane.

Demospongiae, Poecilosclerida
Cladorhizidae gen. indet. 3

NC3_BK2_6000_NOR_0096 ~around 02:05

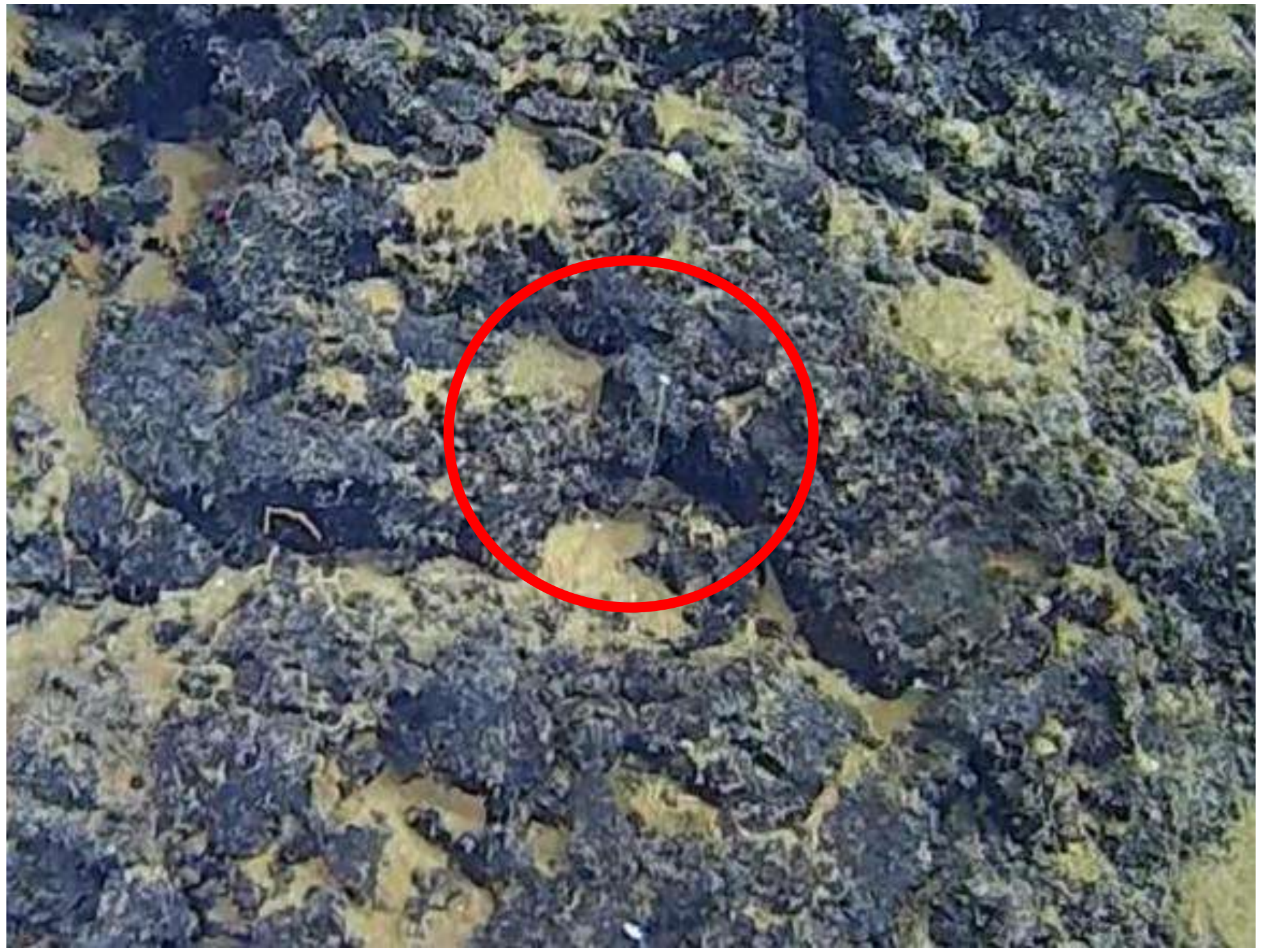

Erect carnivorous sponge with a long very thin stalk supporting a small disk-shaped body, from which spines departs towards the substrates, giving this morphotype the typical parasol appearance. *Axoniderma*-like.

Demospongiae, Poecilosclerida
Cladorhizidae gen. indet. 4

NC2_BK4_NOR_0087 – *00:13*

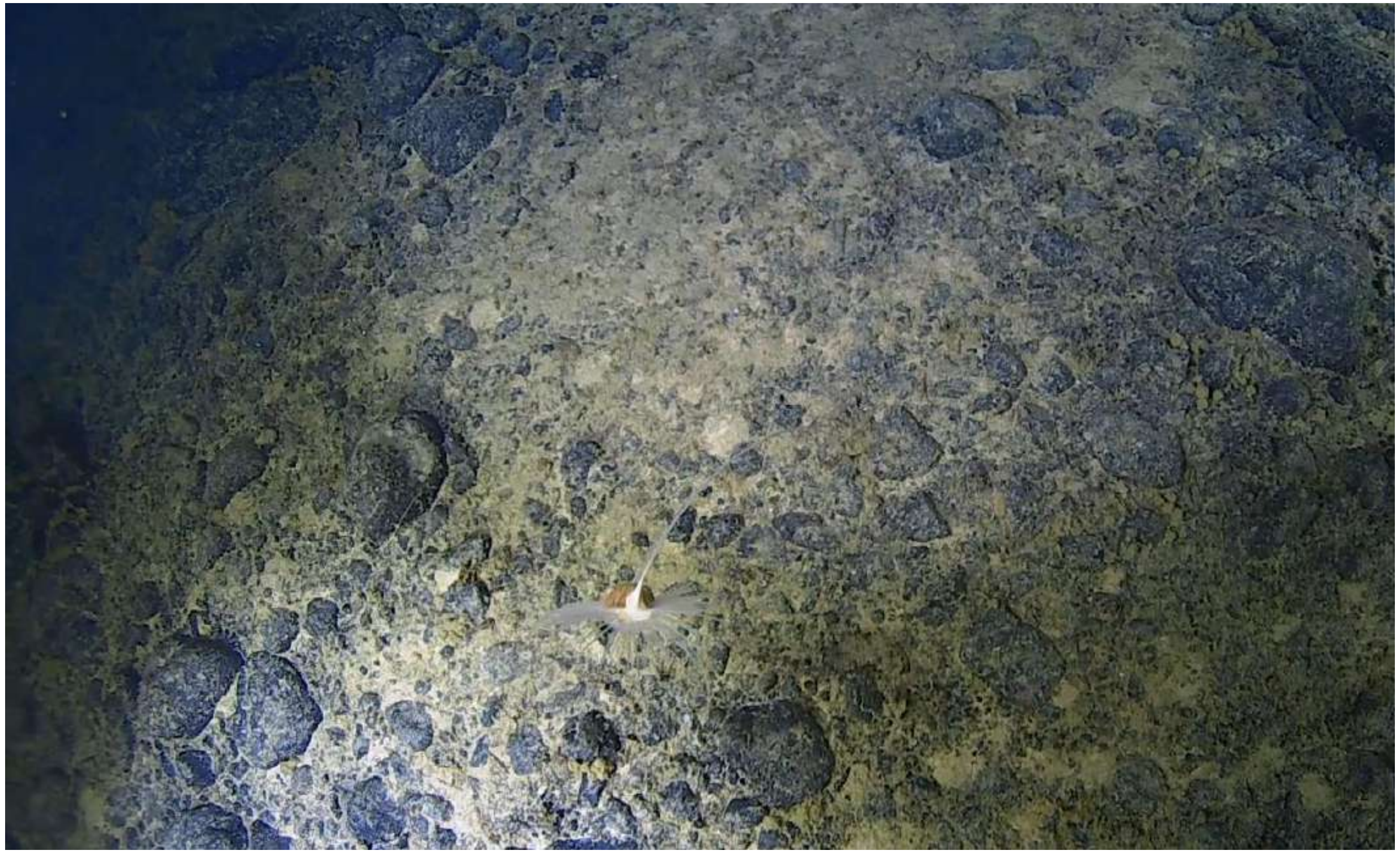

Erect parasol carnivorous sponge with a long very thin stalk supporting a small disk-shaped body, from which spines. Bigger and with longer and thicker spines than gen. indet. 3. *Axoniderma*-like.

Demospongiae, Poecilosclerida
Chondrocladia sp. indet. 1

NC2_BK5_4600 - IMG_9878

NC2_BK5_4600 - IMG_9891

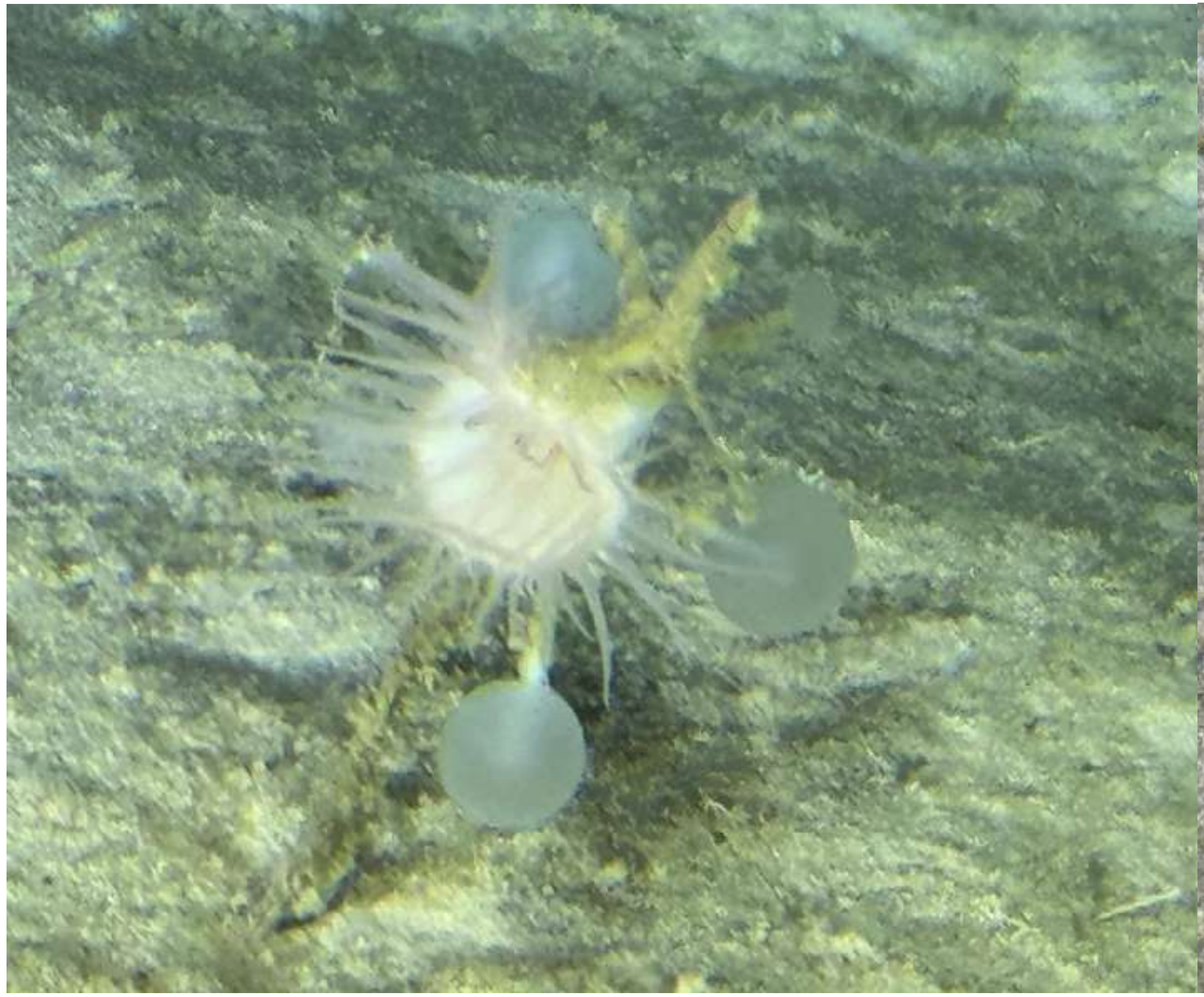

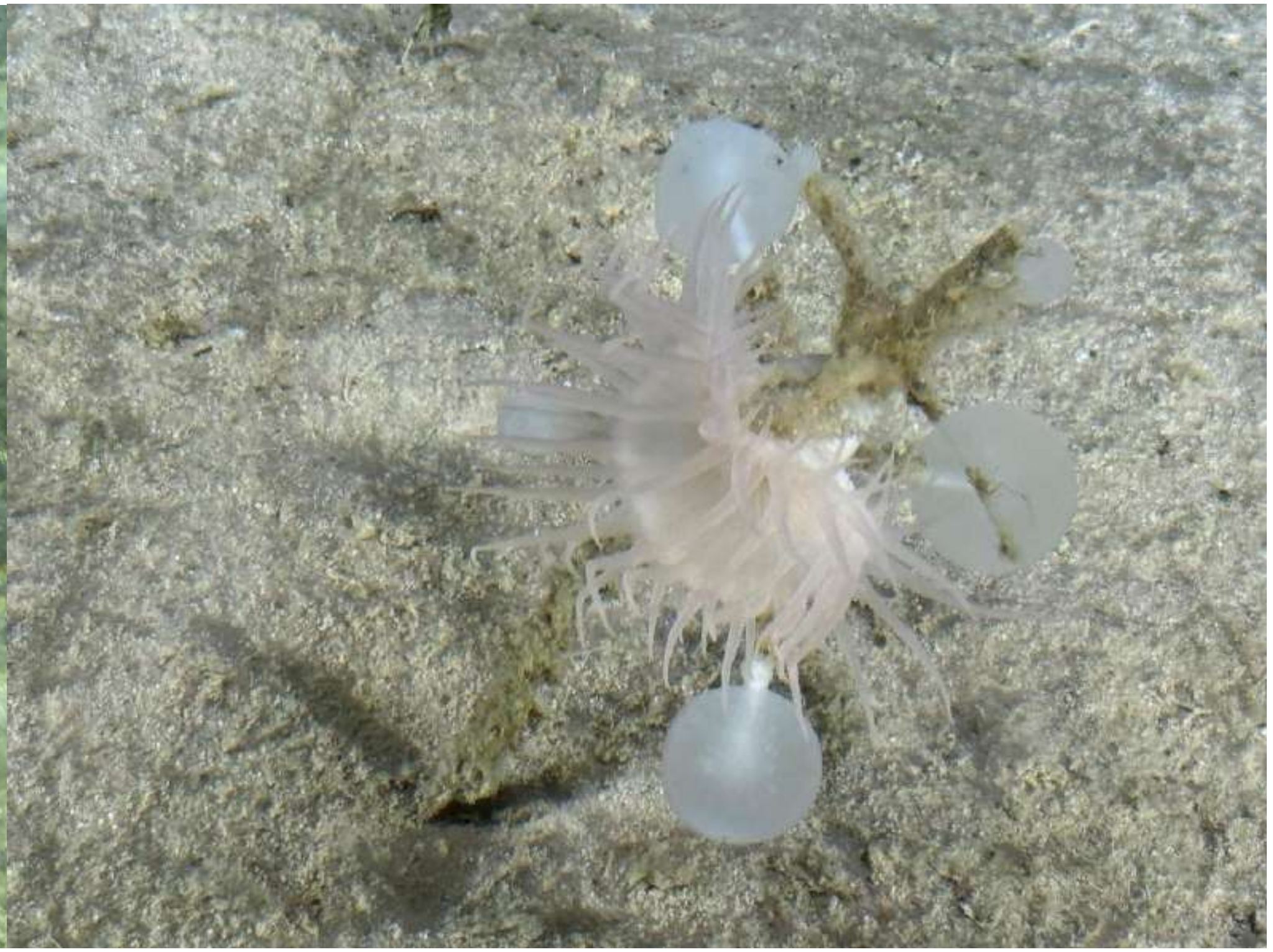

Ping-pong erect sponge. Rigid thick central body with small, buoyant, ball-like appendages. The central body is usually covered in sediments, hydrozoans, and foraminifera, and sometimes having also Venus flytrap sea anemones (fam. Actinoscyphidae).

Demospongiae, Poecilosclerida
Chondrocladia sp. indet. 2

NCT3_BK2_6000_NOR_0074 – 01:34

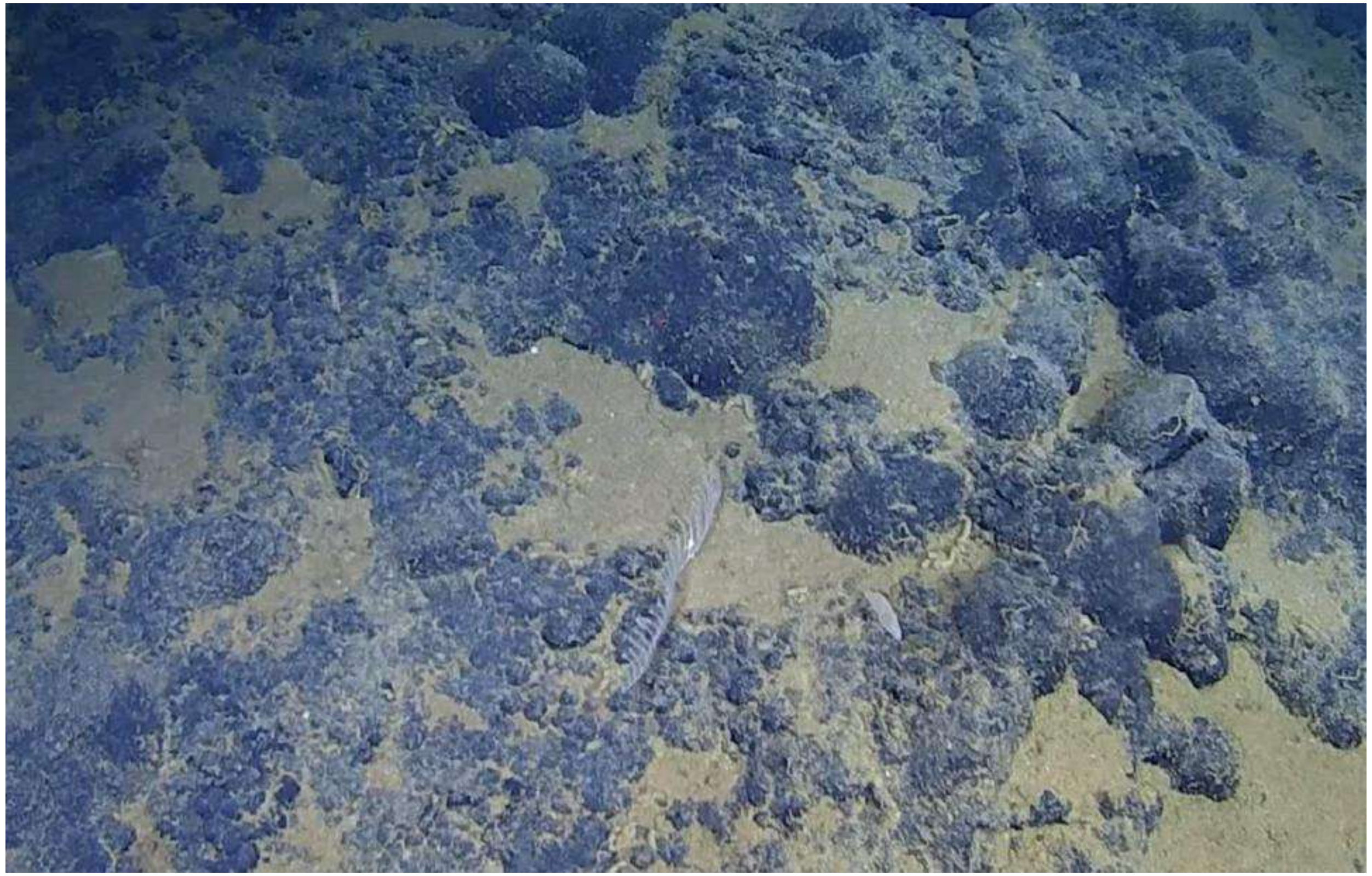

Harp carnivorous sponge. Two main branches develop from the attachment parallel to the ground; extending from each of them at regular intervals are upright branches that end in small white spheres buoys (some are bigger than the others; maybe for reproduction?). Some branching bryozoans have a similar morphology, but lack the spheres, and they are way smaller than these sponges.

Porifera: Hexactinellida

Porifera, Hexactinellida
Hexactinellida ord. inc. 1

NCT2_BK4_5800_NOR_0053 – 02:25

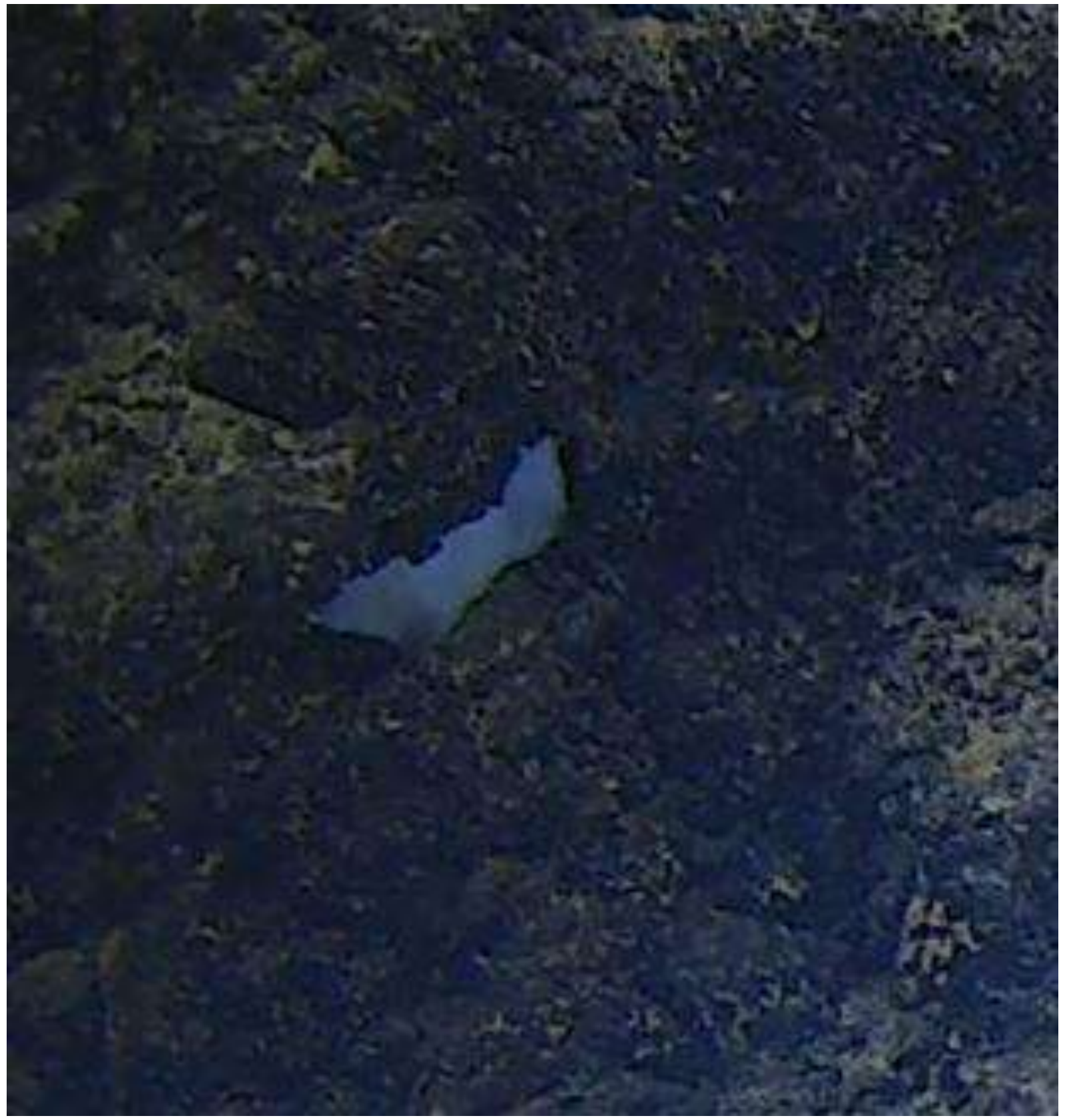

Specimen observed on hard substrate, uncertain ID and morphology, exhibiting a blueish colour with smooth appearance; could either be flat, following the rock profile, or a bit more massive.

Porifera, Hexactinellida
Hexactinellida ord. inc. 2

NCT3_BK2_6000_NOR_0093 – 01:13

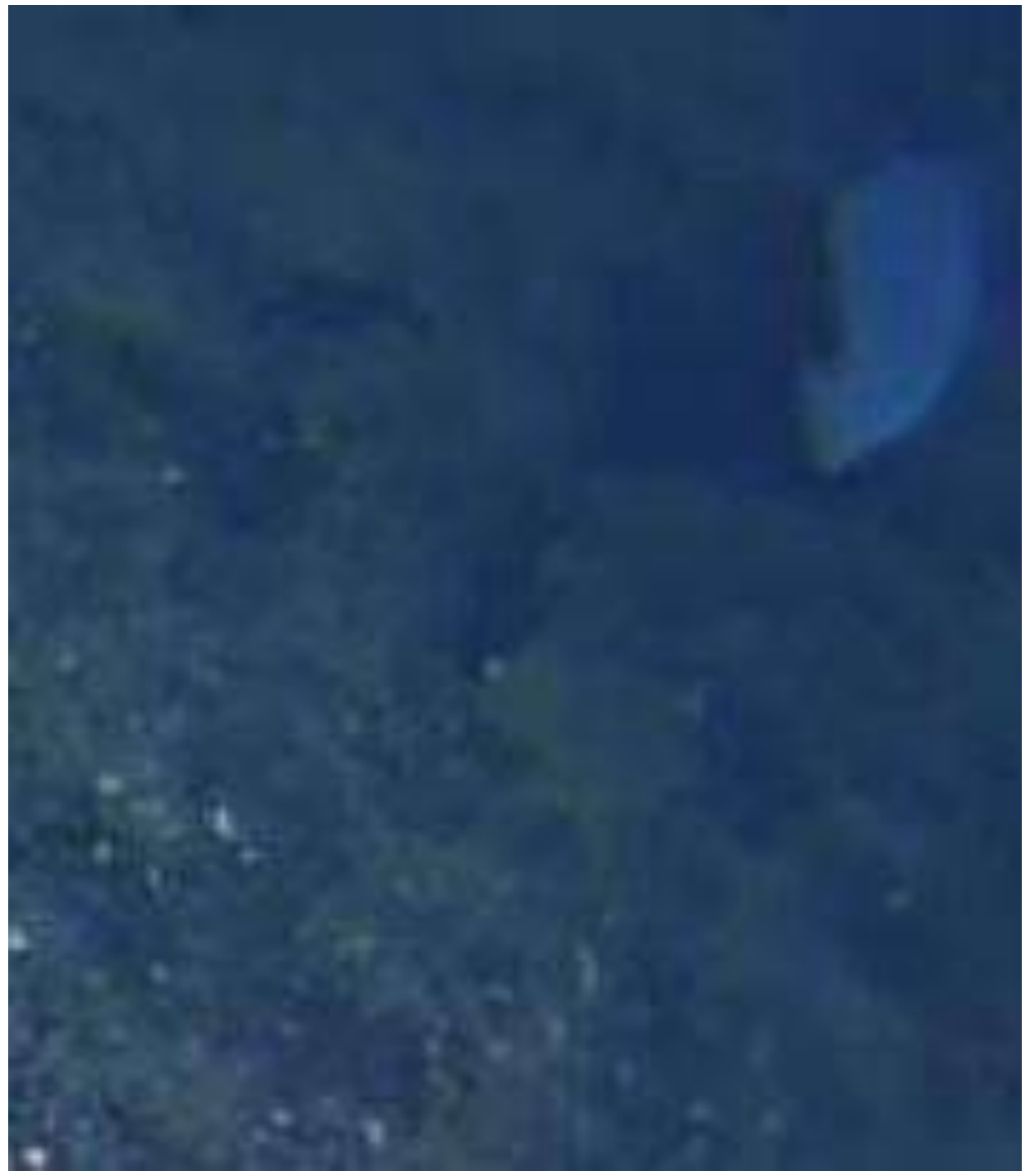

Erect/Barrel sponge with a very large attachment. Euplectellidae?

Porifera, Hexactinellida
Hexactinellida ord. inc. 3

NCT3_BK3_6000_NOR_0068 – 02:43

NCT3_BK3_6000_NOR_0068 – 04:56

NCT3_BK3_6000 – IMG_2762

NCT3_BK3_6000_NOR_0078 – 02:50

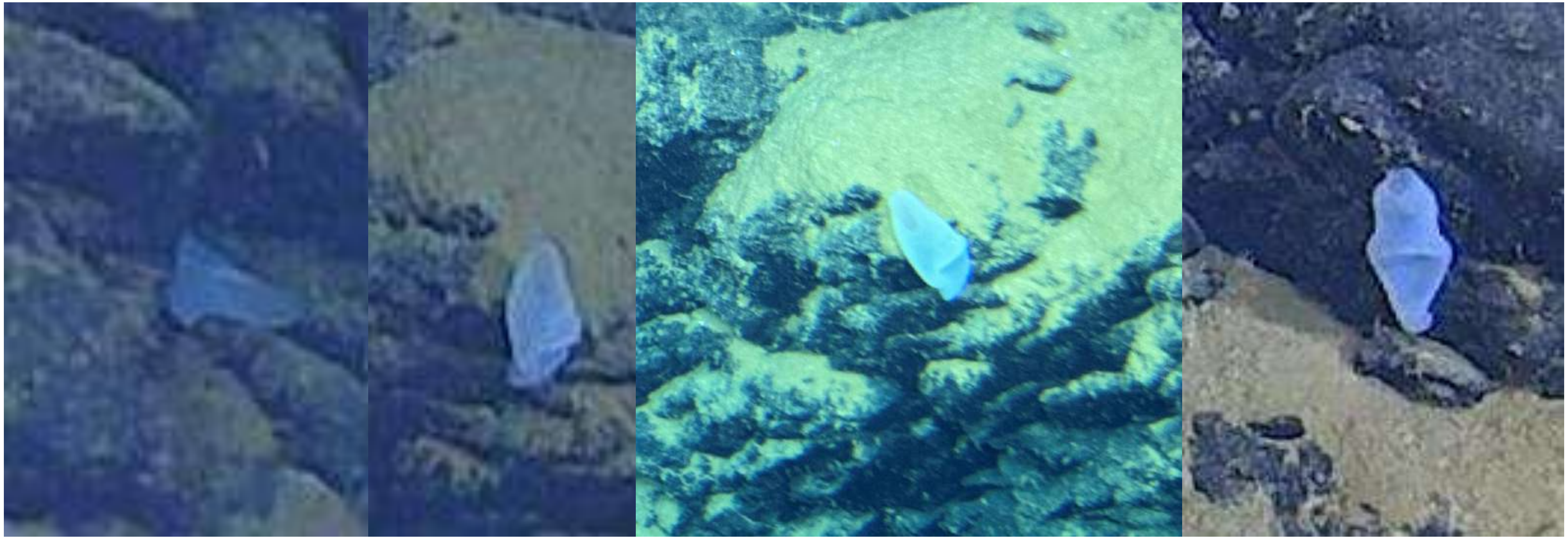

Erect barrel sponge ending with an oval osculum. Main body has a very smooth appearance, and it's separated from the attachment by a "fold. This morphotype may contain multiple genera sharing a close morphology. They could be juveniles of euplectellids or a de-inflated morphology of other morphotypes.

Porifera, Hexactinellida
Hexactinellida ord. inc. 4

NCT3_BK3_6000_NOR_0070 - 1:55

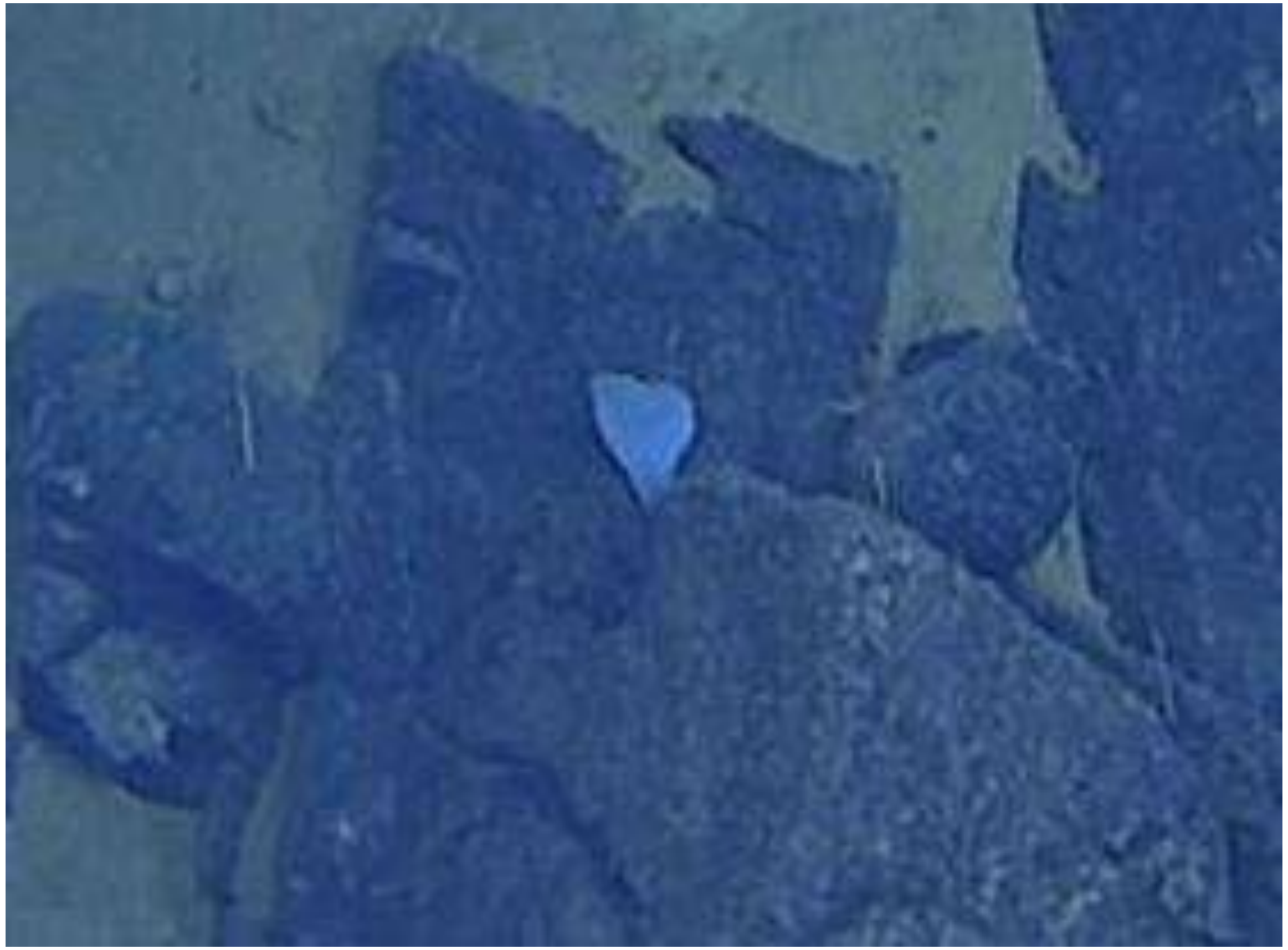

Funnel sponge with a thick peduncle. Traces of sediment on the edge. Thick peduncle similar to Hexactinellida ord. inc. 2, body texture close to Euplectellidae gen. inc. 5.

Porifera, Hexactinellida
Hexactinellida ord. inc. 5

NCT3_BK3_6000_NOR_0078 - 01:30

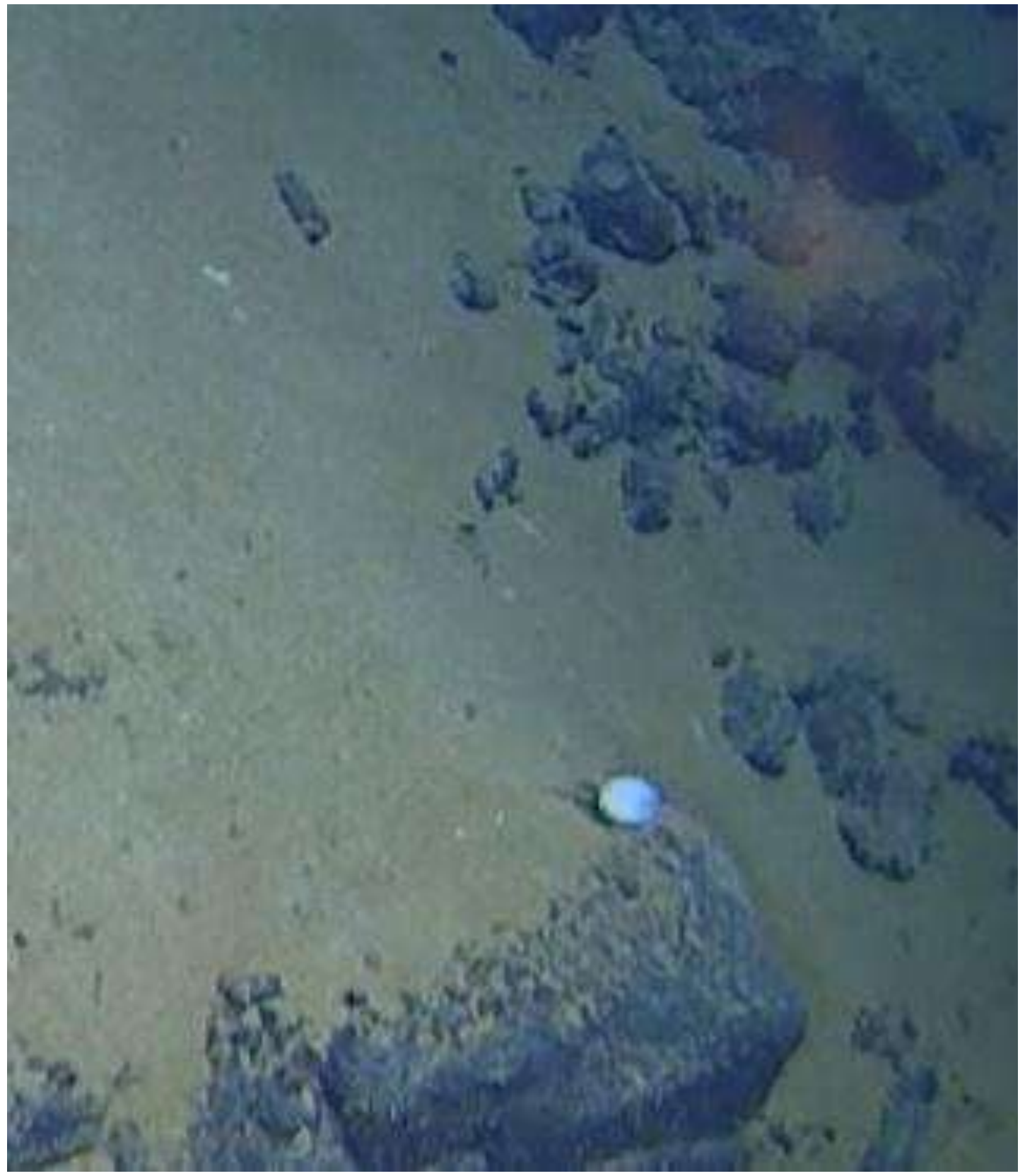

Short stalked hexactinellid with a globular body. Attached to substrate covered in sand, or in the fine sediment? *Amphidiscella, Hyalonema, Sympagella, Coryonema*?

Porifera, Hexactinellida
Hexactinellida ord. inc. 6

NCT4_BK2_4700_NOR_0066 – 01:02

NCT4_BK4_4700_NOR_0052 – 01:02

NCT4_BK4_4700 – IMG_6033

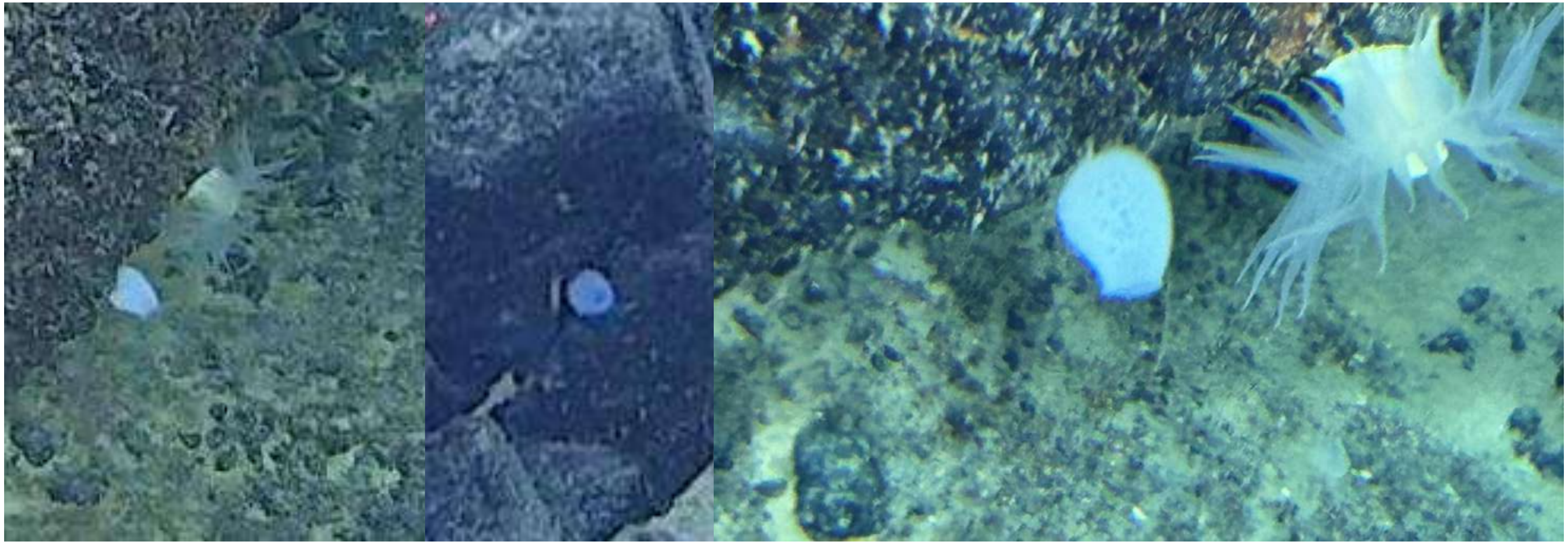

Small very short stalked glass sponges attached to hard substrate. Lyssacinosida?

Hexactinellida, Amphidiscosida
Hyalonematidae, Hyalonema sp. indet. 1

NCT2_BK2_3800_NOR_0069 – 00:01

NCT2_BK2_3800_NOR_0088 – 01:30

NCT2_BK5_4600_NOR_0072 – 04:30

NCT2_BK5_4600 – IMG_0207

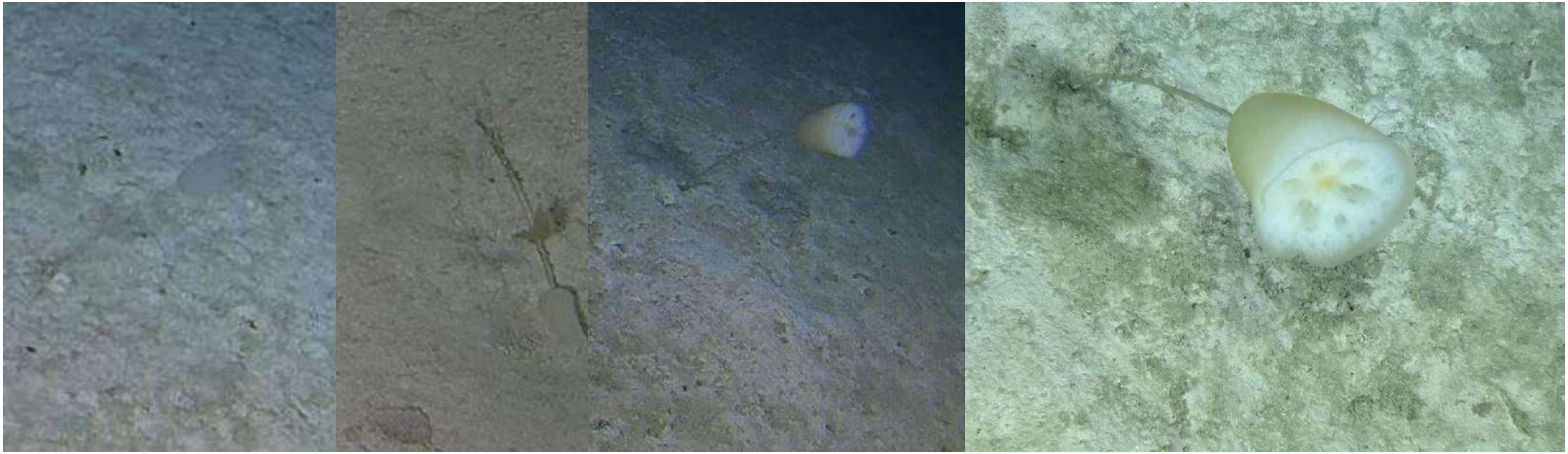

Stalked sponge with bell-shaped body and flat apical part, second picture likely same morphospecies but with contracted body.

Hexactinellida, Amphidiscosida
Hyalonematidae, Hyalonema gen. inc. 2

NCT2_BK4_5300_NOR_0058 – 00:50

NCT3_BK2_6000_NOR_0061 – 00:07

NCT2_BK4_5300 – IMG_9367

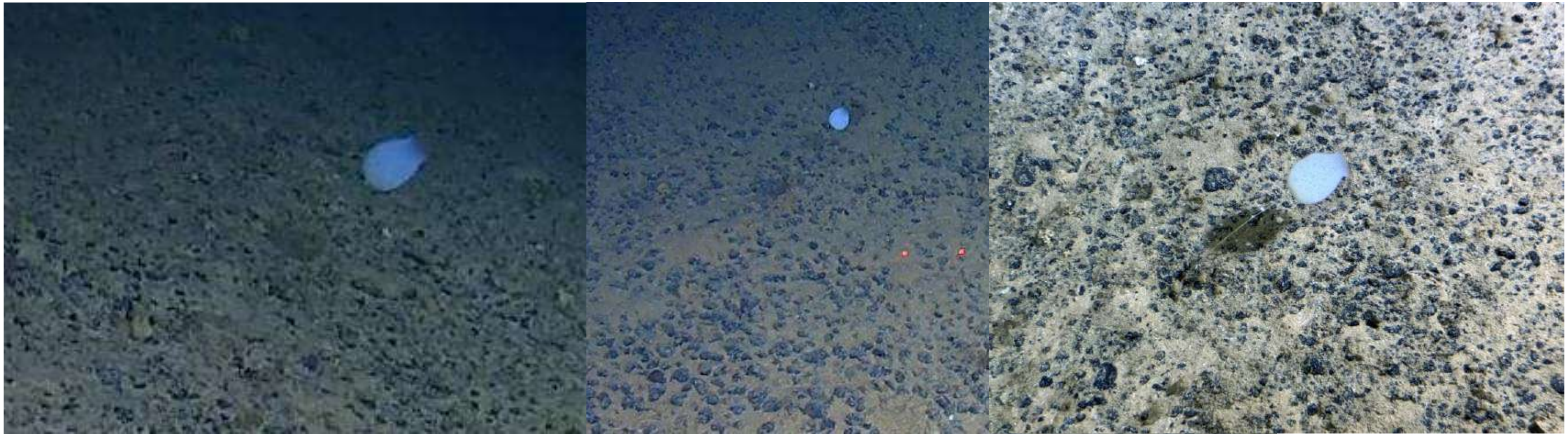

Thin stalked sponge with bell-shaped smooth body, apical part with a small bottleneck; growing on fine sediment spaces between nodules rather than nodules themselves? Could be *Hyalostylus*.

Hexactinellida, Amphidiscosida
Hyalonematidae, Hyalonema sp. indet. 3

NCT2_BK5_4600_NOR_0062 – 04:47

NCT2_BK5_4600_NOR_0064 – 02:14

NCT2_BK5_4600 – IMG_0179

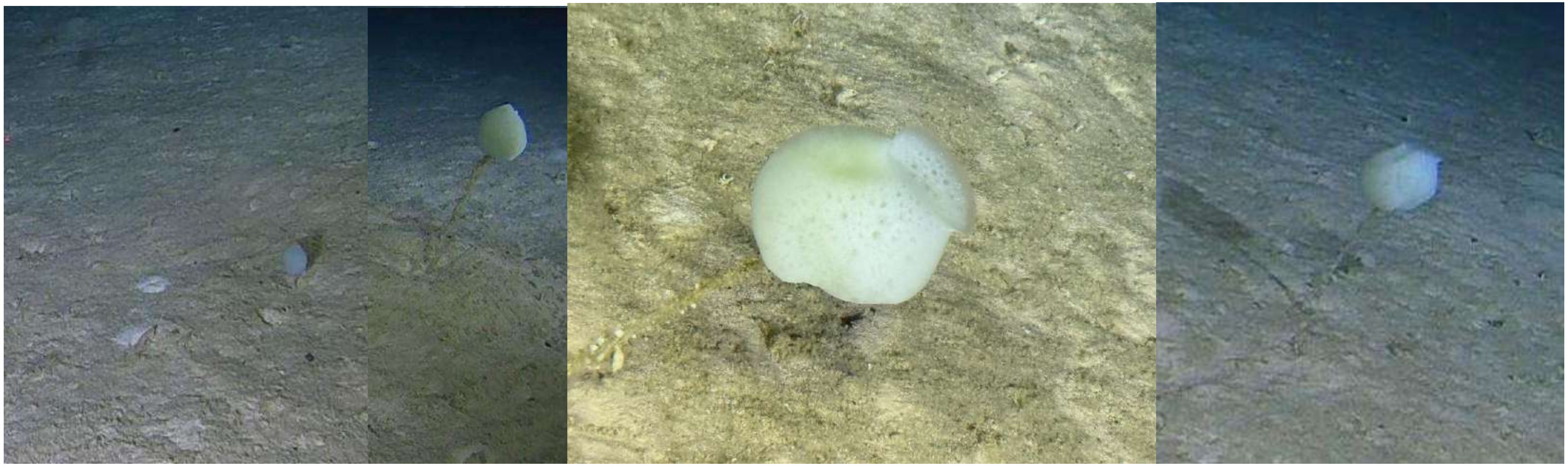

Rounded stalked sponge with osculum having a defined edge, looking pointed from certain angles. First picture as a juvenile? Very similar to sp. indet. 4.

Hexactinellida, Amphidiscosida
Hyalonematidae, Hyalonema sp. indet. 4

NCT2_BK5_4600 – IMG_9970

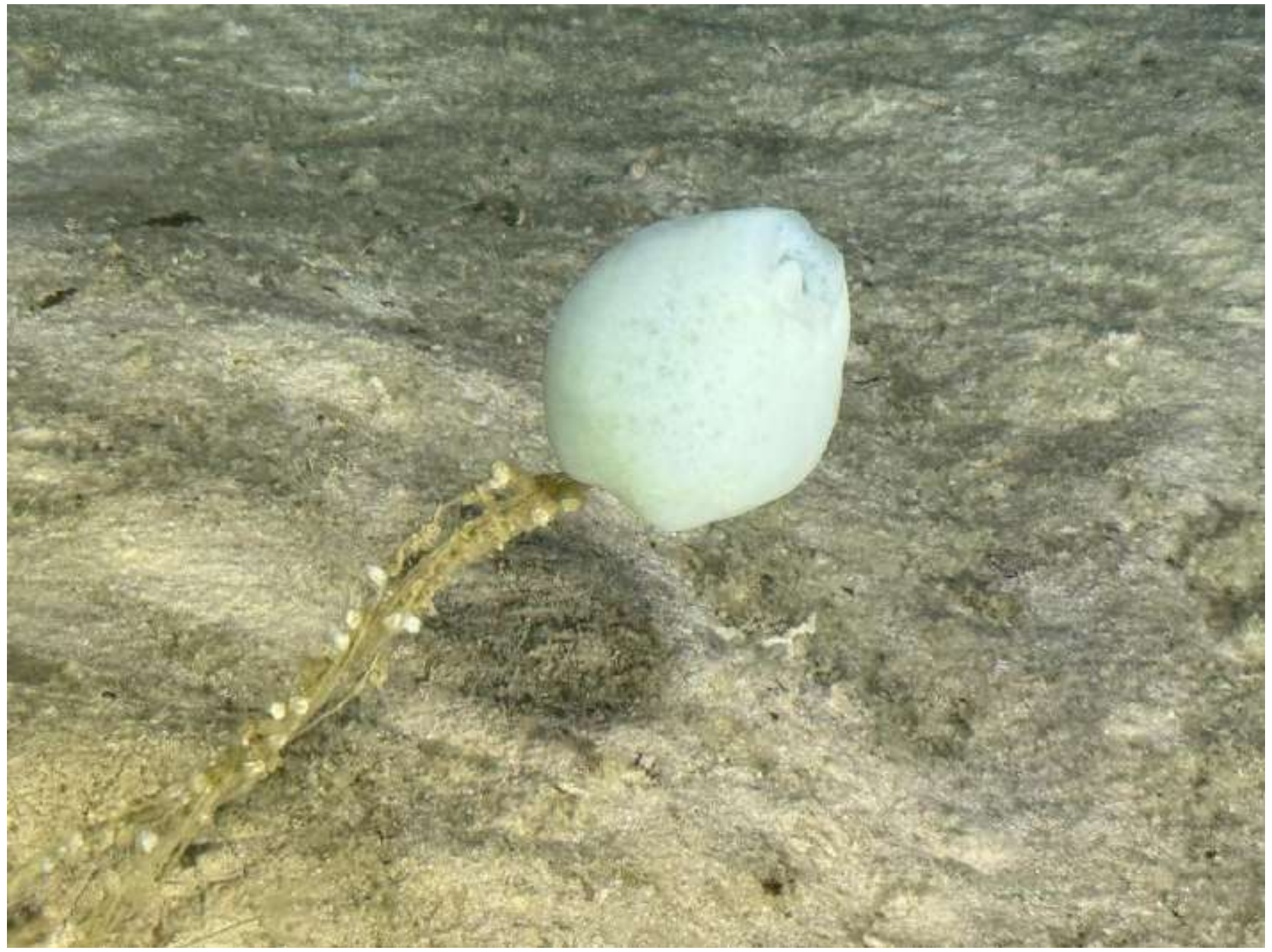

Rounded stalked sponge with osculum lacking a defined edge but having a very short central gastral cone. This morphotype can only be distinguished from iPhone pics, as otherwise the cone is not recognizable. Very similar to sp. indet. 3.

Hexactinellida, Lyssacinosida
Lyssacinosida fam. inc. 1

NCT2_BK2_3800_NOR_0086 – 02:58

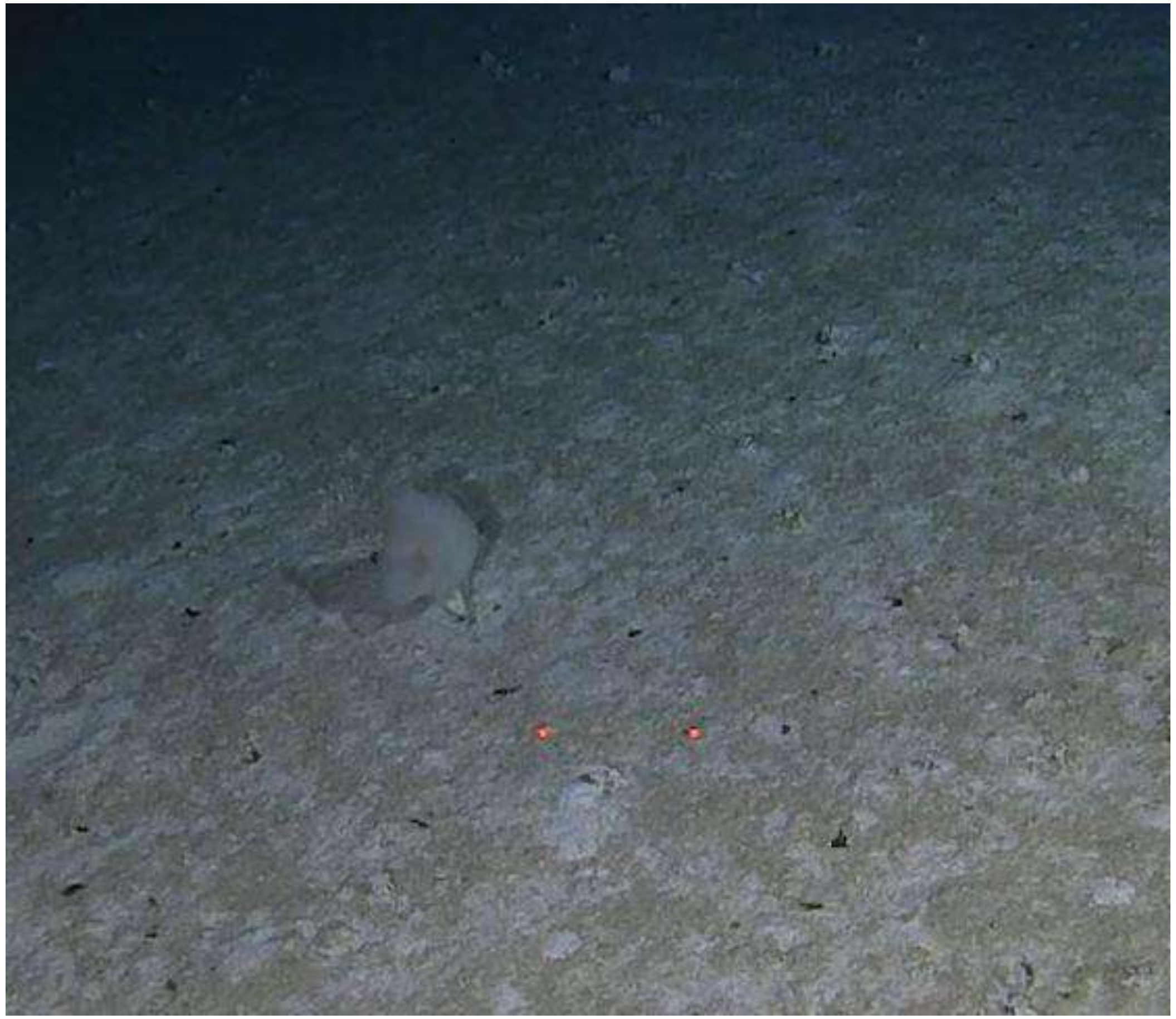

Short stalked specimen observed on fine sediments. Main body is a large bell with a central osculum. Euplectellidae?

Hexactinellida, Lyssacinosida
Lyssacinosida fam. inc. 2

NCT1_BK2_6700_NOR_0080 – 00:33

NCT1_BK2_6700 – IMG_5526

NCT4_BK3_6900_NOR_0061 – 01:29

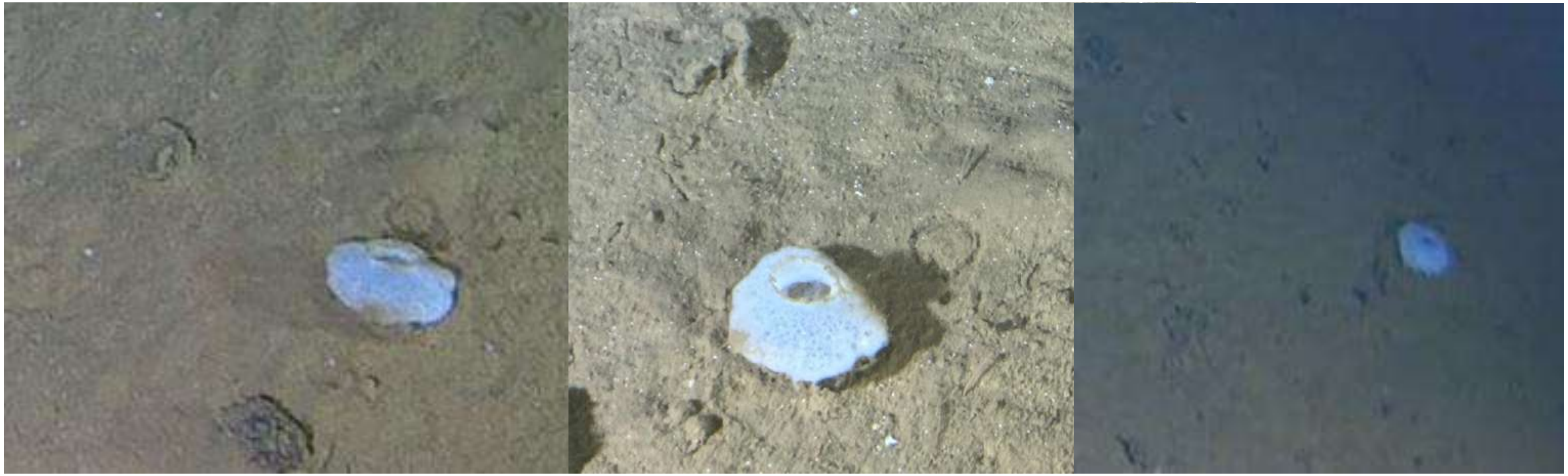

Nest shaped sponge observed on hadal soft sediments. The osculum is surrounded by a small neck covered in sediment. The atrium appear to be opening directly on the substrate. Base of the nest and body texture smoother than Euplectellidae gen. inc. 1., with larger openings over the body.

Hexactinellida, Lyssacinosida
Lyssacinosida fam. inc. 3

NCT1_BK2_6700_NOR_0066 – 03:30

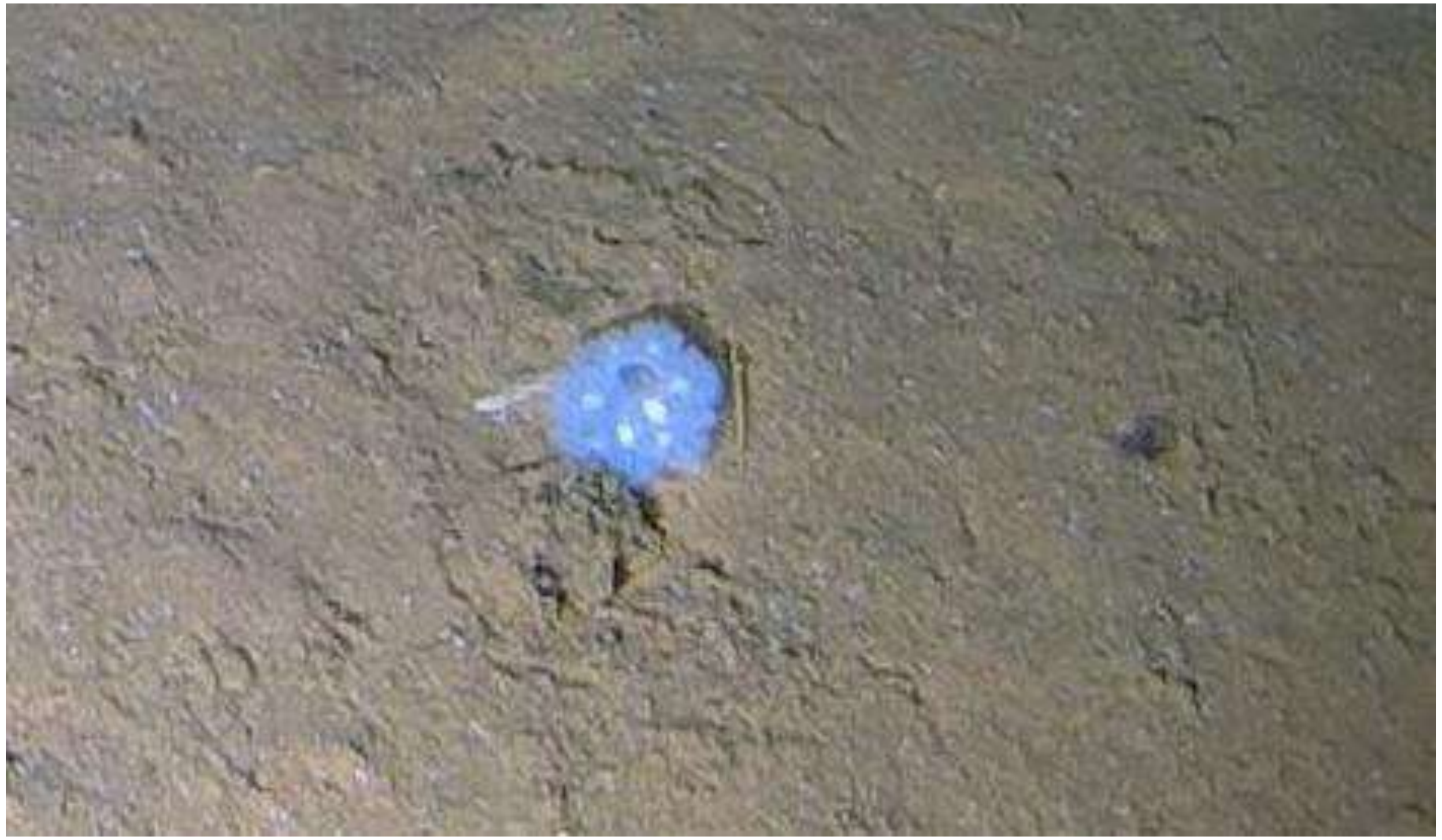

Found on hadal soft sediment, is a short barrel morphology with osculum well defined.
Similar to Lyssacinosida 4 and 5 from Tonga. Euplectellidae or Aulocalycidae?

Hexactinellida, Lyssacinosida
Lyssacinosida fam. inc. 4

NCT3_BK2_6000_NOR_0087 – 00:26

NCT2_BK4_5800_NOR_0070 – 02:15

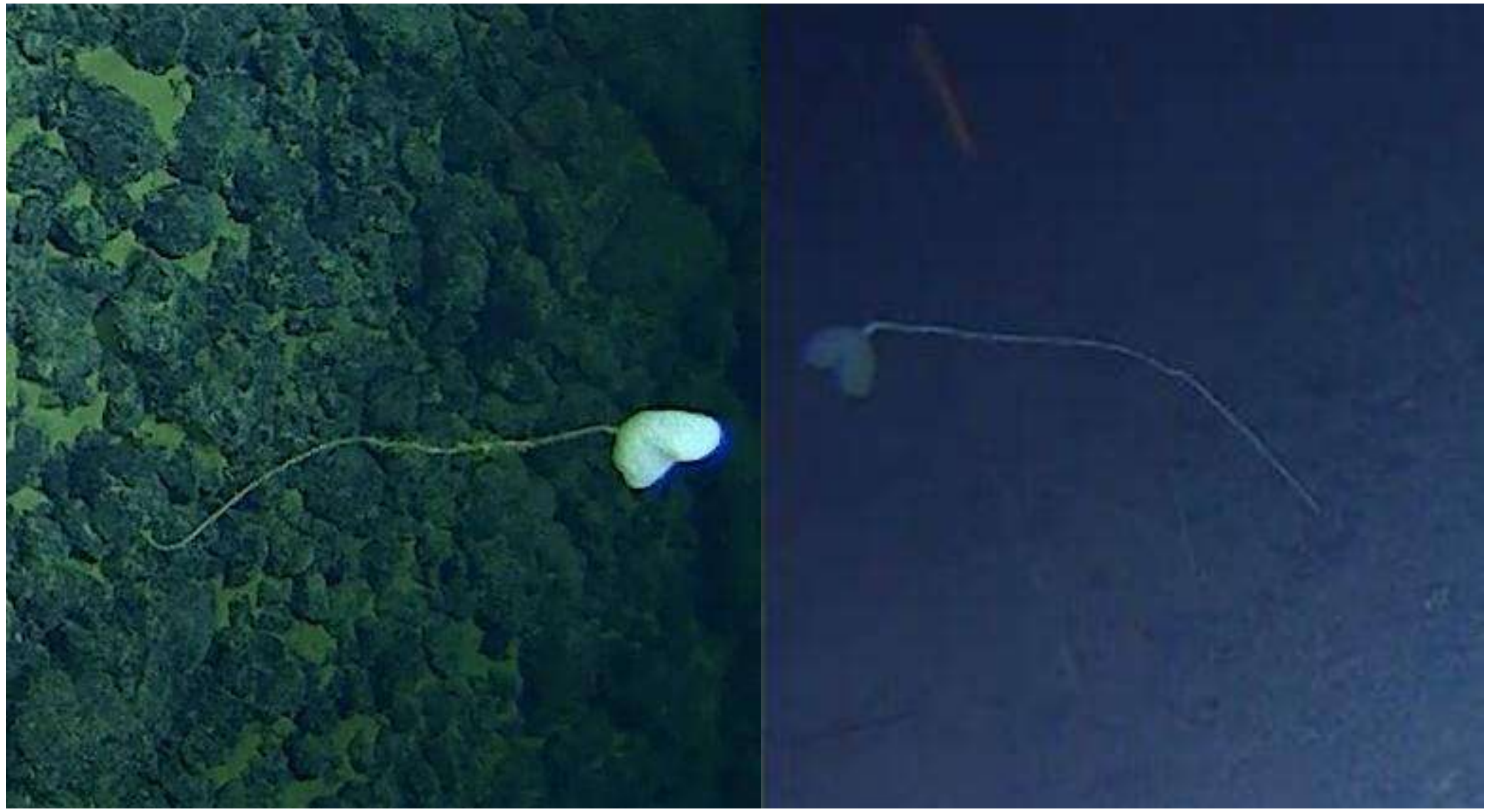

Very long stalked sponge with a lobate massive body. Found on rocky sloped areas. *Caulophacus* or stalked Euplectellidae?

Hexactinellida, Lyssacinosida
Lyssacinosida fam. inc. 5

NCT2_BK4_5300_NOR_0064 – 04:59

NCT2_BK4_5300 – IMG_9424

NCT3_BK3_6000 – IMG_3464

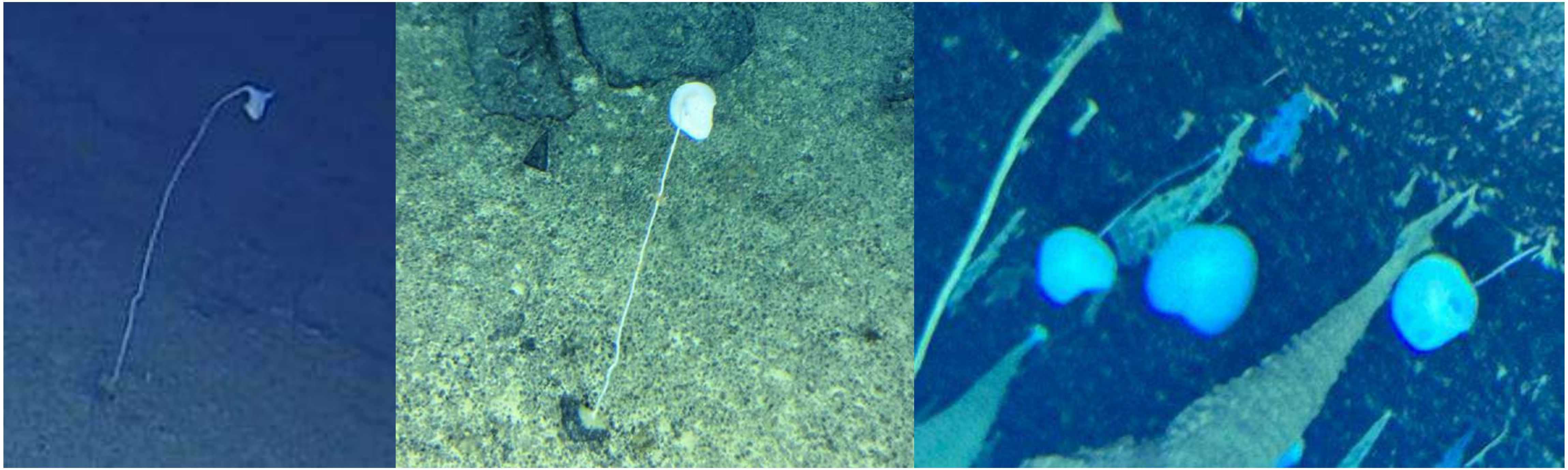

Very long stalked sponge with a flat body that looked folded/convex. Found on rocky sloped areas. Juveniles in last photo? *Caulophacus* or stalked Euplectellidae?

Hexactinellida, Lyssacinosida
Lyssacinosida fam. inc. 6

NCT3_BK3_6000 – IMG_3226

NCT3_BK3_6000_NOR_0079 – 02:15

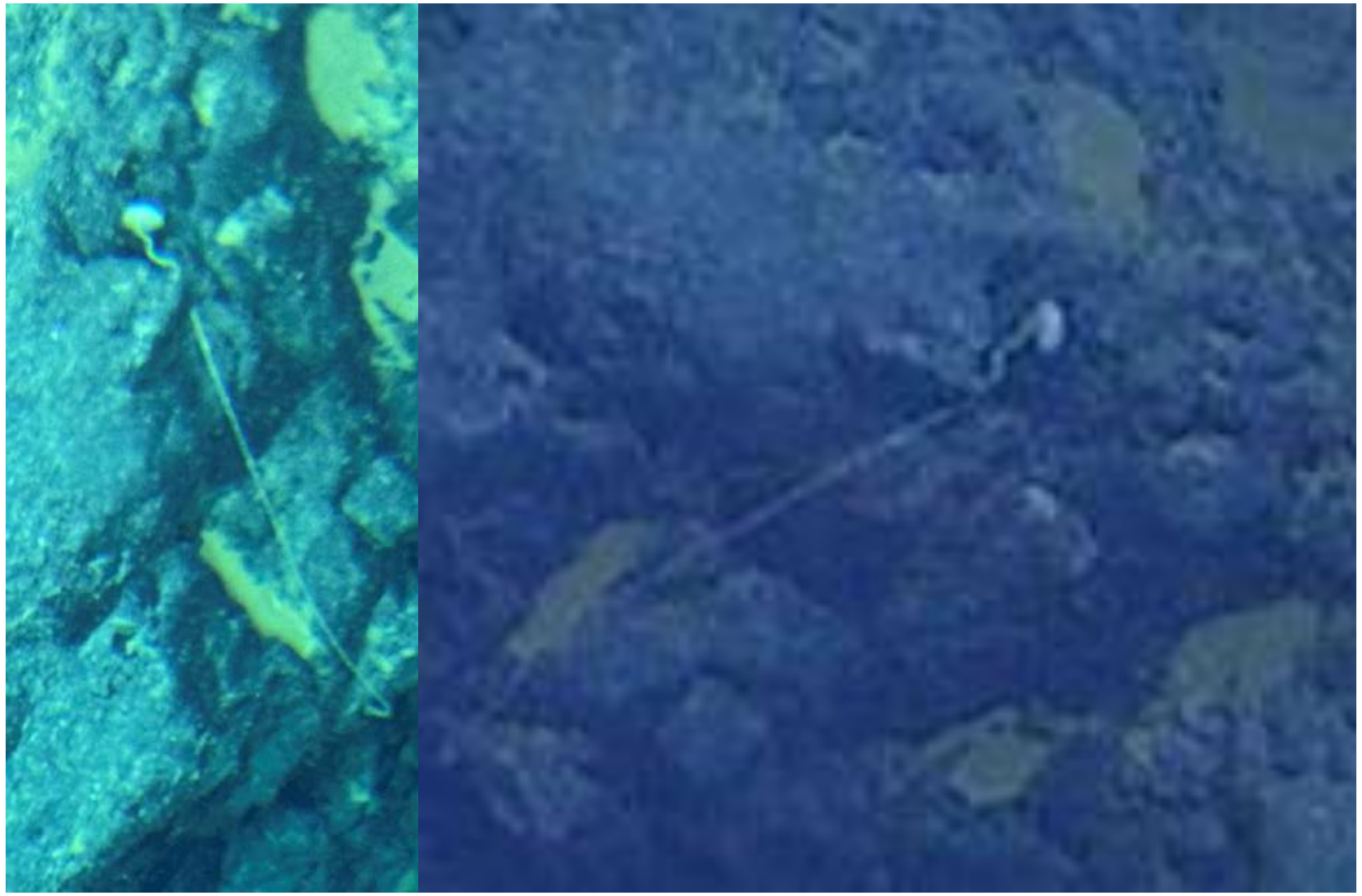

Very long stalked sponge with a mushroom-like body. Found on rocky sloped areas. Thicker stalk than *Caulophacis* sp. 1. *Caulophacus* or stalked Euplectellidae?

Hexactinellida, Lyssacinosida
Lyssacinosida fam. inc. 7

NCT3_BK3_6000_NOR_0080 – 02:10

NCT4_BK4_4700 – IMG_6080

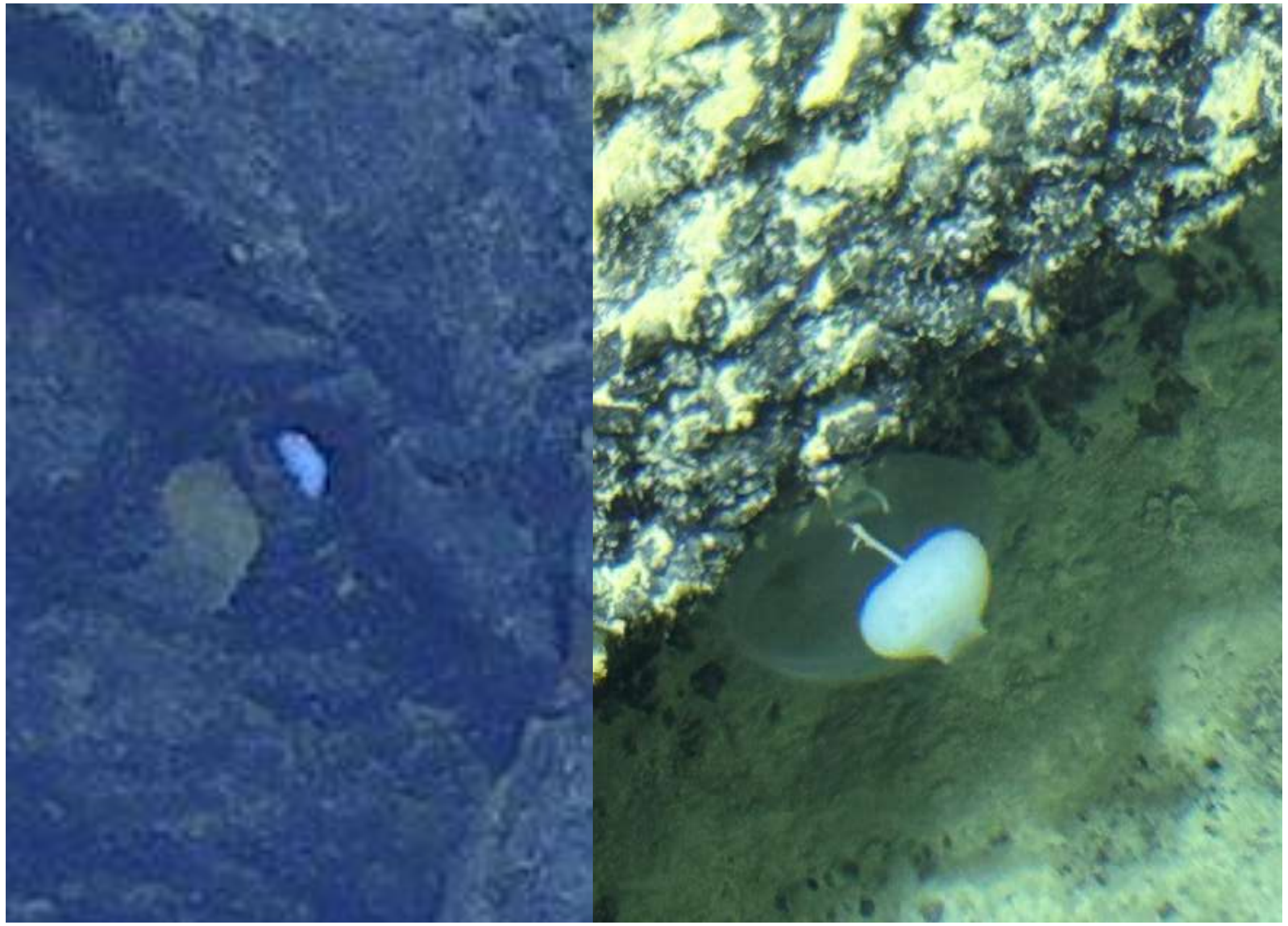

Short stalked sponge with a mushroom-like body, with more or less defined osculum edge. (*This is almost a bin-category for mushroom short stalked sponges on hard substrate*)

Hexactinellida, Lyssacinosida
Lyssacinosida fam. inc. 8

NCT4_BK6_4600_NOR_0084 – 04:44

NCT4_BK6_4600 – IMG_7006

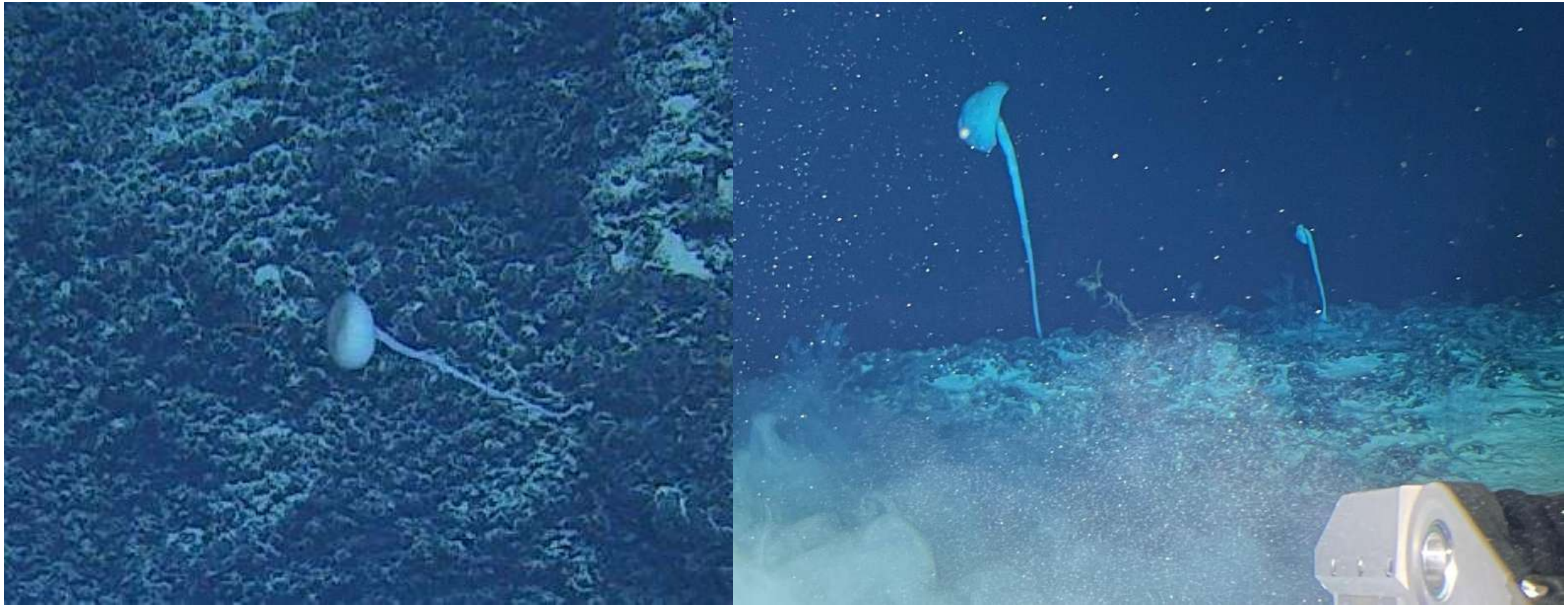

Long stalked sponge with a massive rounded, mushroom-like body.
Euplectellidae or *Caulophacus*?

Hexactinellida, Lyssacinosida
Euplectellidae gen. inc. 1

NCT1_BK2_6700_NOR_0074 – 01:27

NCT1_BK2_6700_NOR_0077 – 01:10

NCT1_BK2_6700 – IMG_5166

NCT1_BK2_6700 – IMG_5189

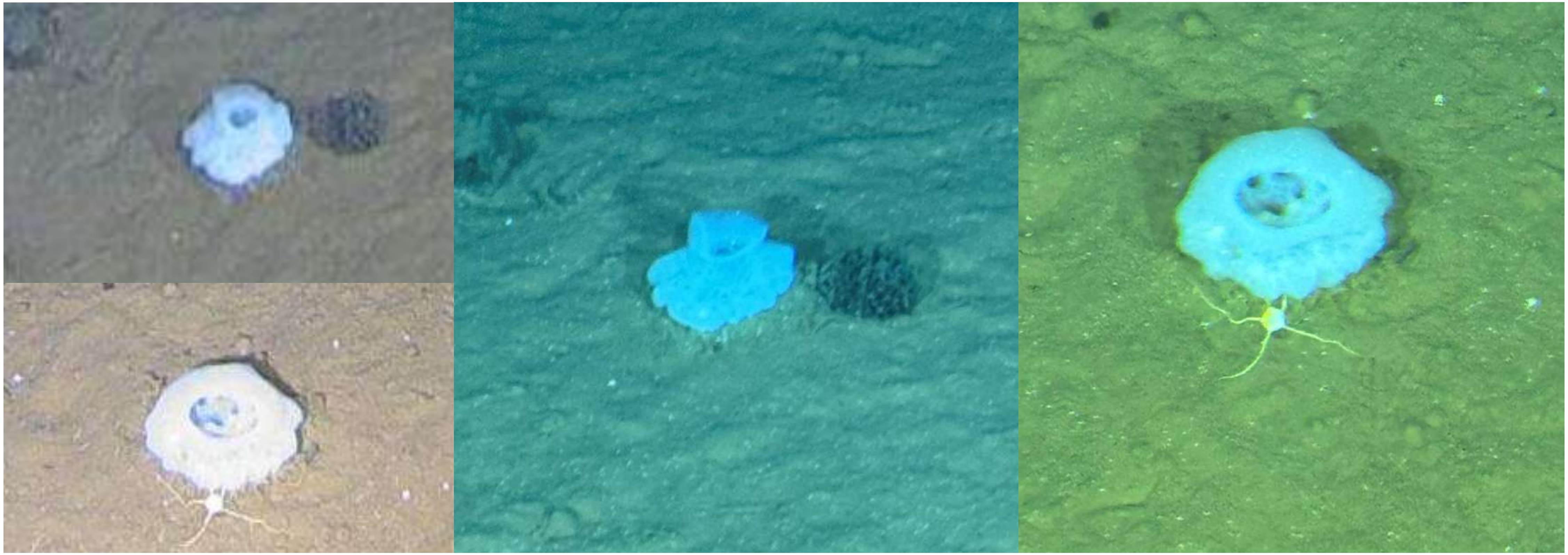

Nest shaped sponge observed on hadal soft sediments. Can a more or less developed “neck” around the osculum. The atrium does not open directly on the substrate, as a certain level of structure can be observed from the openings. Body texture and base of the nest not smooth but forming small bumps (double check adjective). *Placopegma* or *Chaunangium*.

Hexactinellida, Lyssacinosida
Euplectellidae gen. inc. 2

NCT3_BK2_6000_NOR_0086 – 03:56

NCT3_BK2_6000 – IMG_2295

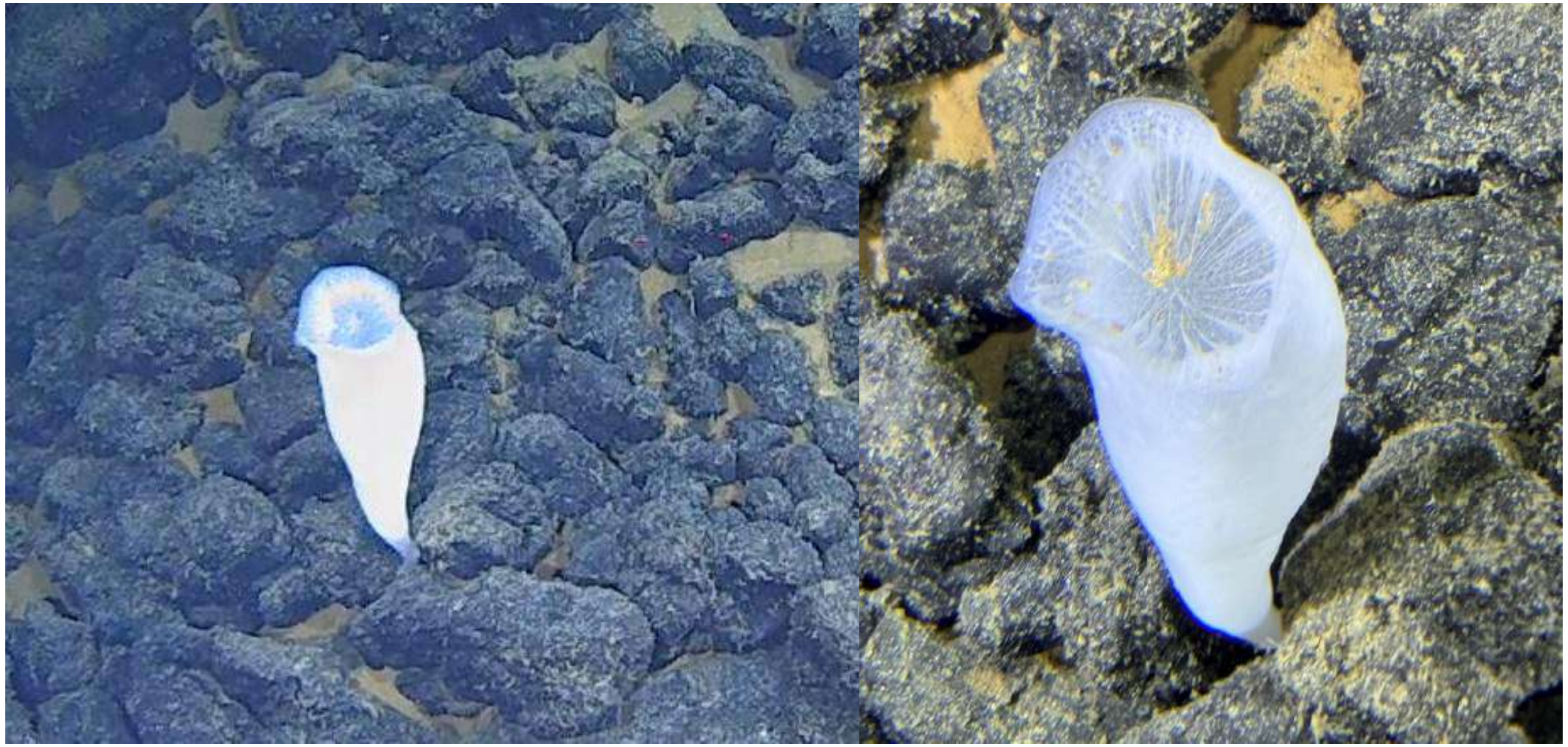

Venus' flower basket sponge attached to the rock and with a visible sieve plate. Osculum opens after a small restriction.

Hexactinellida, Lyssacinosida
Euplectellidae gen. inc. 3

NCT3_BK2_6000_NOR_0086 – 03:56

NCT3_BK2_6000_NOR_0087 – 00:38

NCT3_BK2_6000 – IMG_2299

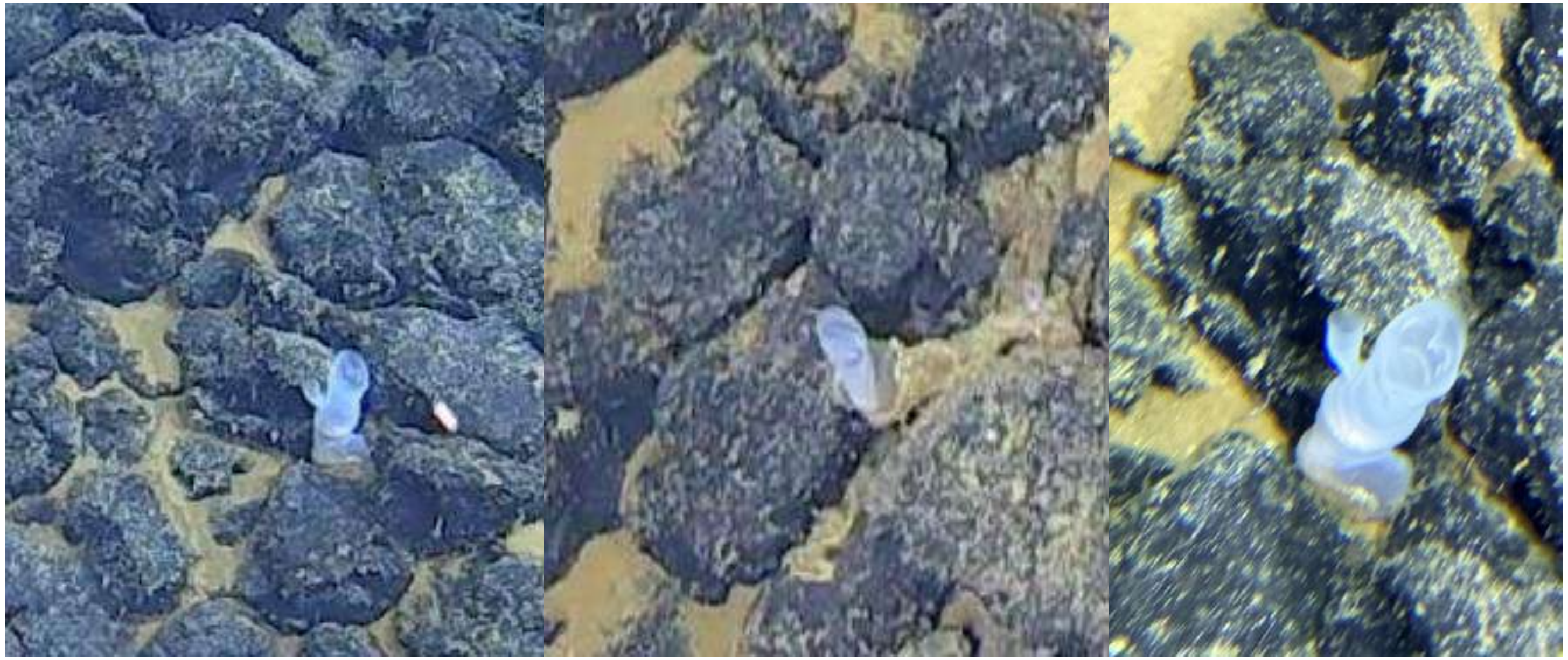

Small barrel sponge with thin body walls, lacks a sieve plate and the osculum is not round but hearth-shaped within the opening. In one case was observed with a secondary digitation developing from the side

Hexactinellida, Lyssacinosida
Euplectellidae gen. inc. 4

NCT3_BK2_6000_NOR_0086 – 03:56

NCT3_BK2_6000 – IMG_2389

NCT3_BK3_6000 – IMG_2990

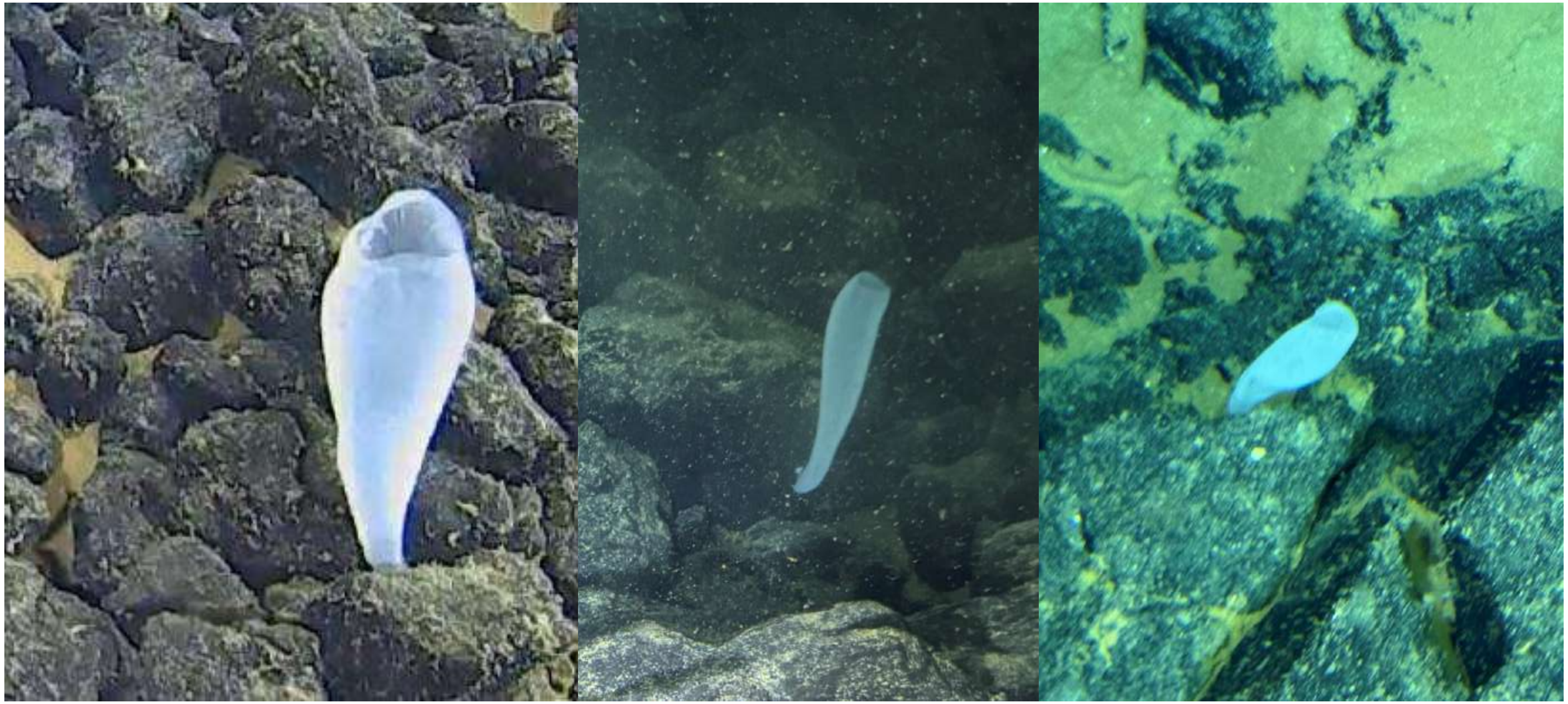

Venus’ flower basket sponge attached to the rock and with a barely visible sieve plate, without a well developed osculum edge as in gen. inc. 2.

Hexactinellida, Lyssacinosida
Euplectellidae gen. inc. 5

NCT3_BK2_6000_NOR_0086 – 03:56

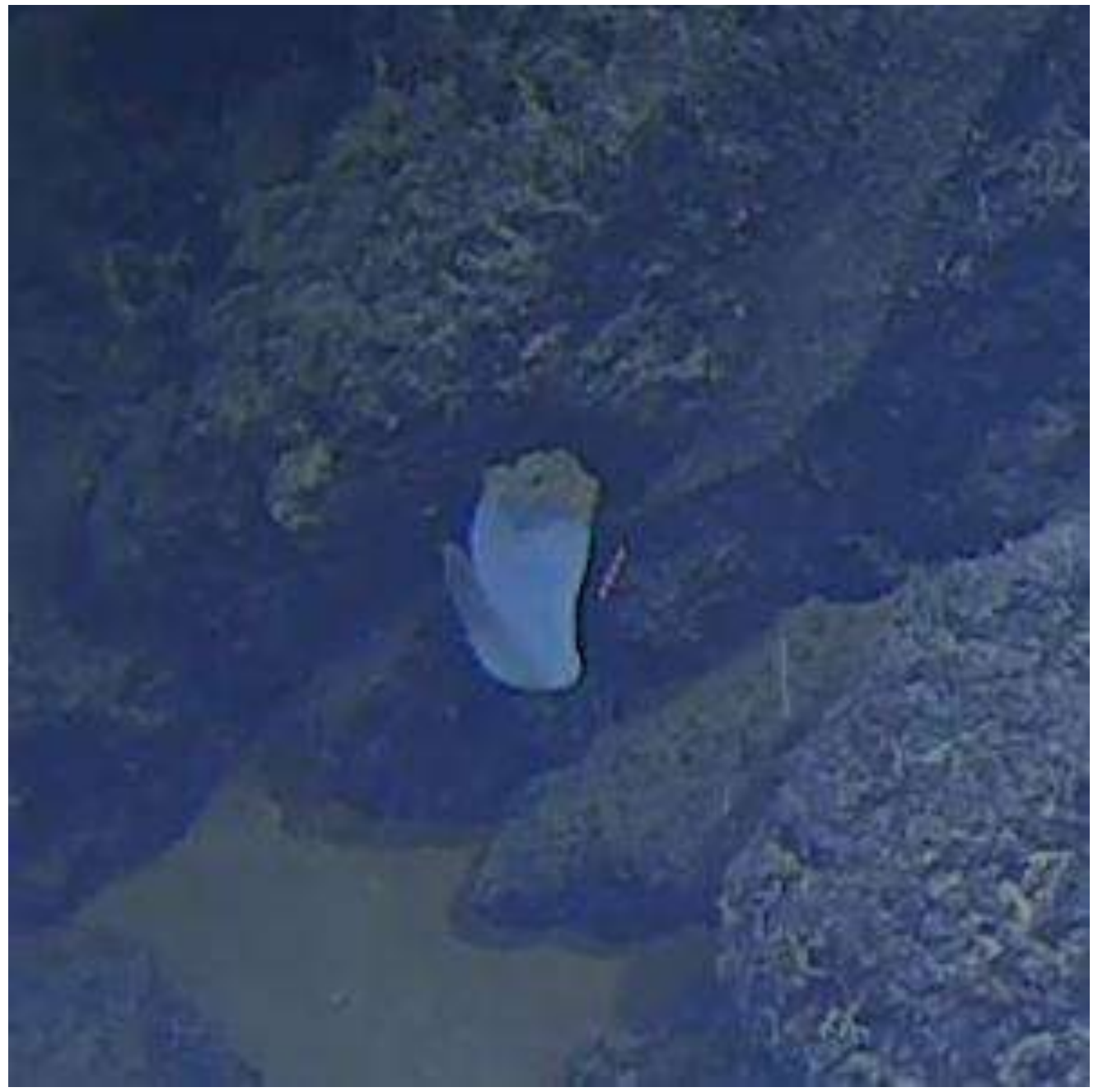

Barrel-like sponge, close to a Venus' flower basket morphology but lacking a visible sieve plate and with a peculiar very long peduncle.

Hexactinellida, Lyssacinosida Euplectellidae gen. inc. 6

NCT3_BK2_6000_NOR_0094 – 02:23

NCT3_BK2_6000 – IMG_2307

NCT3_BK3_6000 – IMG_2776

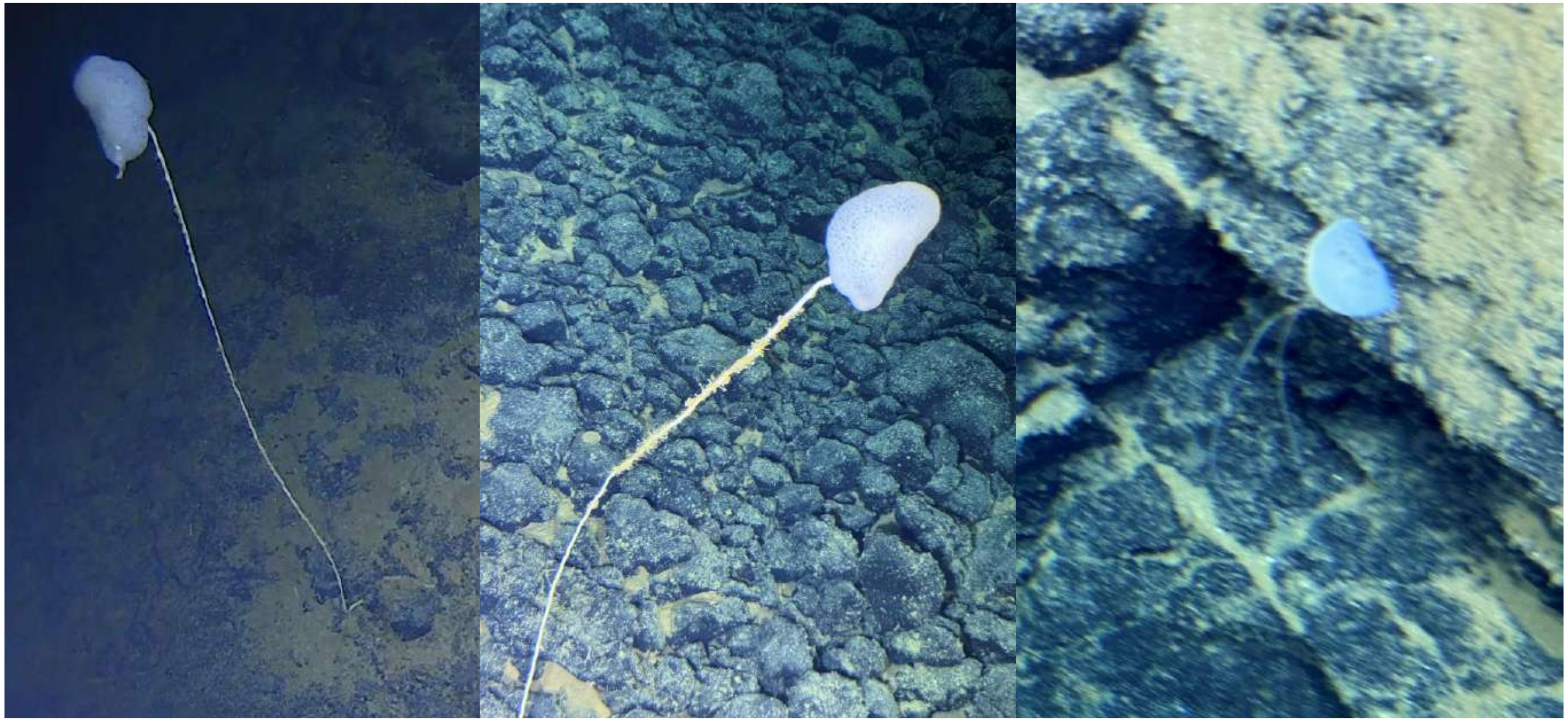

Very long stalked sponge with a flat body, different from Lyssacinosida fam. inc. 5. Found on rocky sloped areas. Texture different from rossellid sponges. Subfamily Bolosominae.

Hexactinellida, Lyssacinosida
Euplectellidae gen. inc. 7

NCT3_BK3_6000_NOR_0073 – 02:31

NCT3_BK3_6000_NOR_0074 – 03:31

NCT3_BK3_6000 – IMG_3010

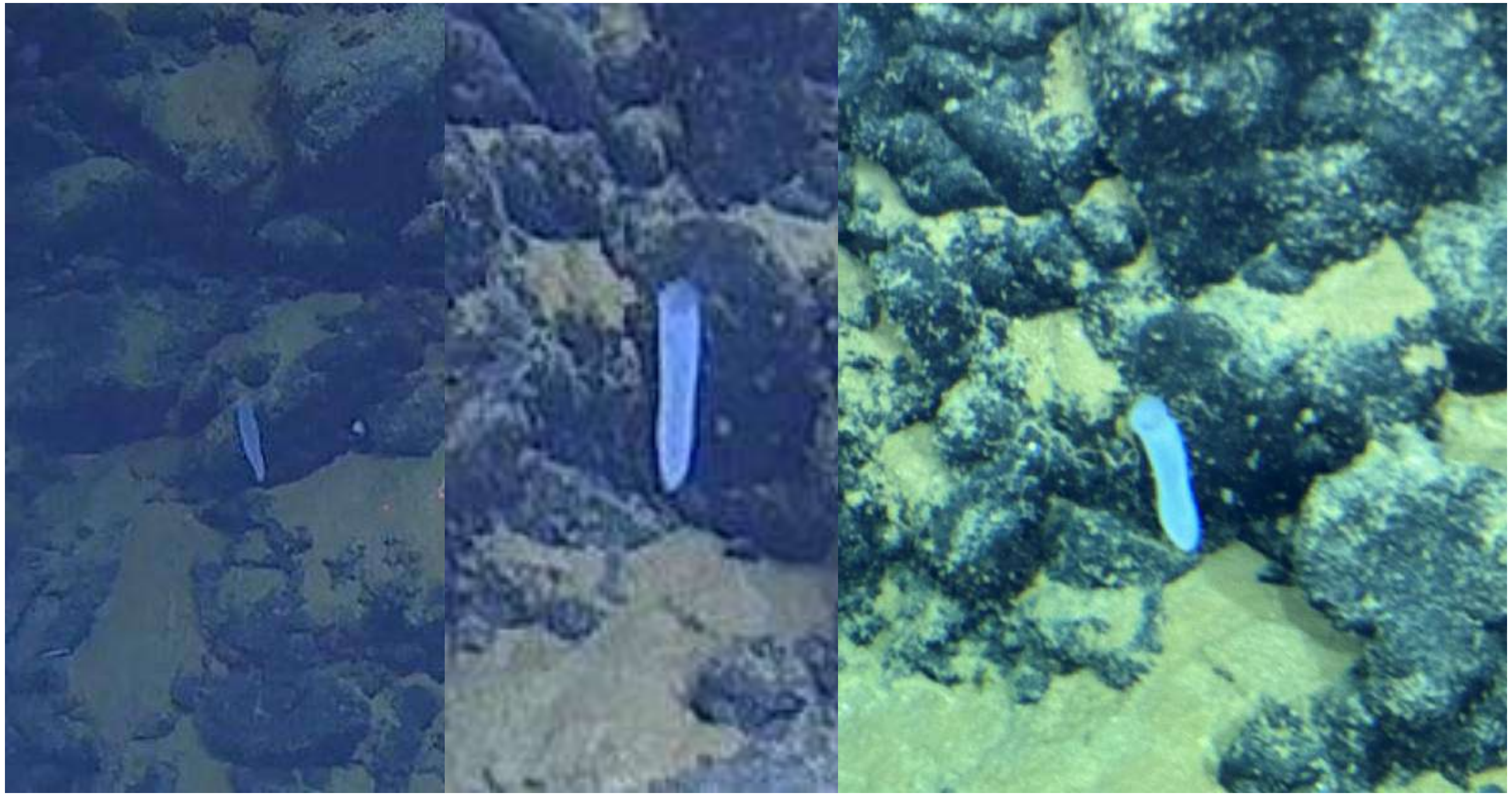

Very long and straight barrel sponge found on subvertical hard substrate. Unclear the presence of a sieve plate covering the obsculum. As it shows the same diameter all over its height, it’s separated from gen. inc. 2 and 4.

Porifera, Hexactinellida
Euplectellidae; *Docosaccus* gen. inc. 1

NCT3_BK2_6000_NOR_0064 – 00:01

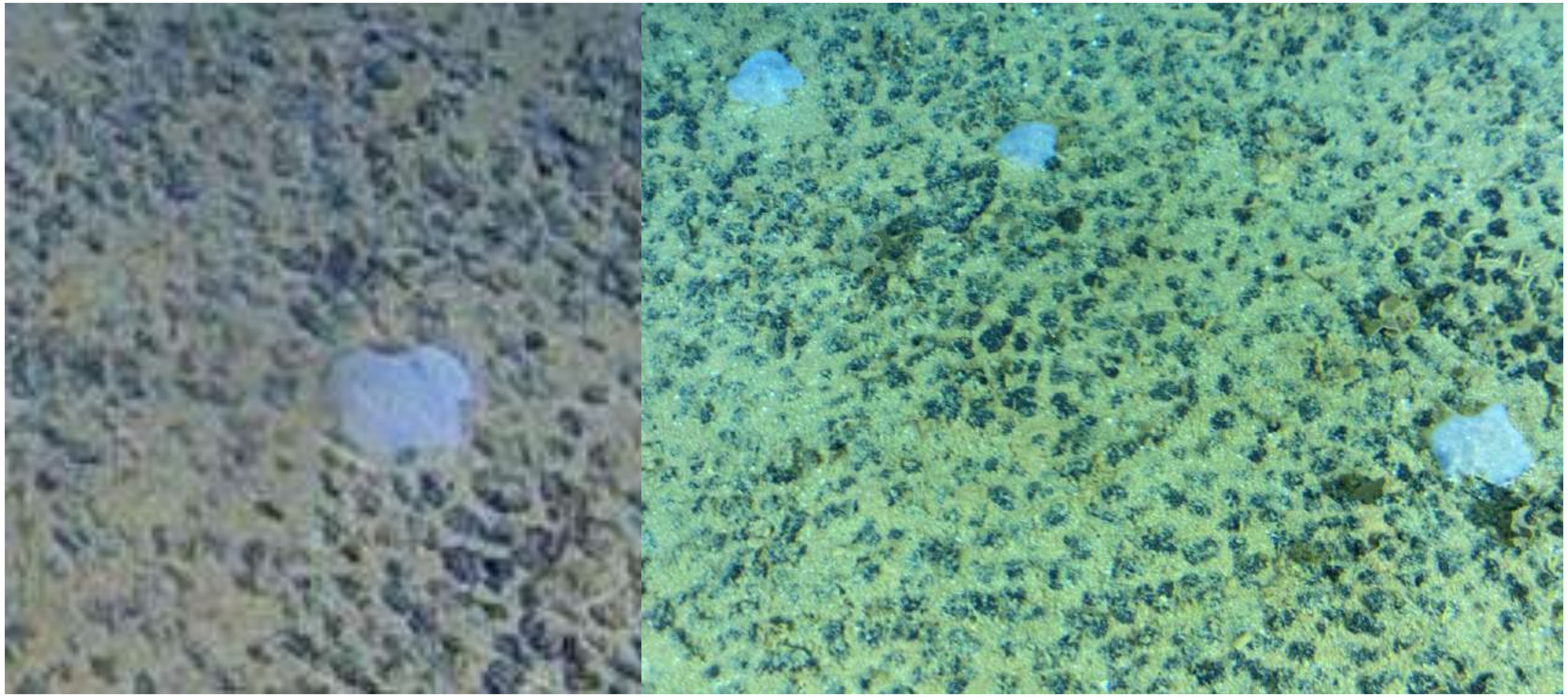

Semi transparent to bluish irregular plate-shaped sponge, growing horizontally on nodules, showing small white spots.

Hexactinellida, Lyssacinosida
Euplectellidae; Holascus sp. indet. 1

NCT3_BK2_6000_NOR_0055 – 00:22

NCT3_BK2_6000 - IMG_1340

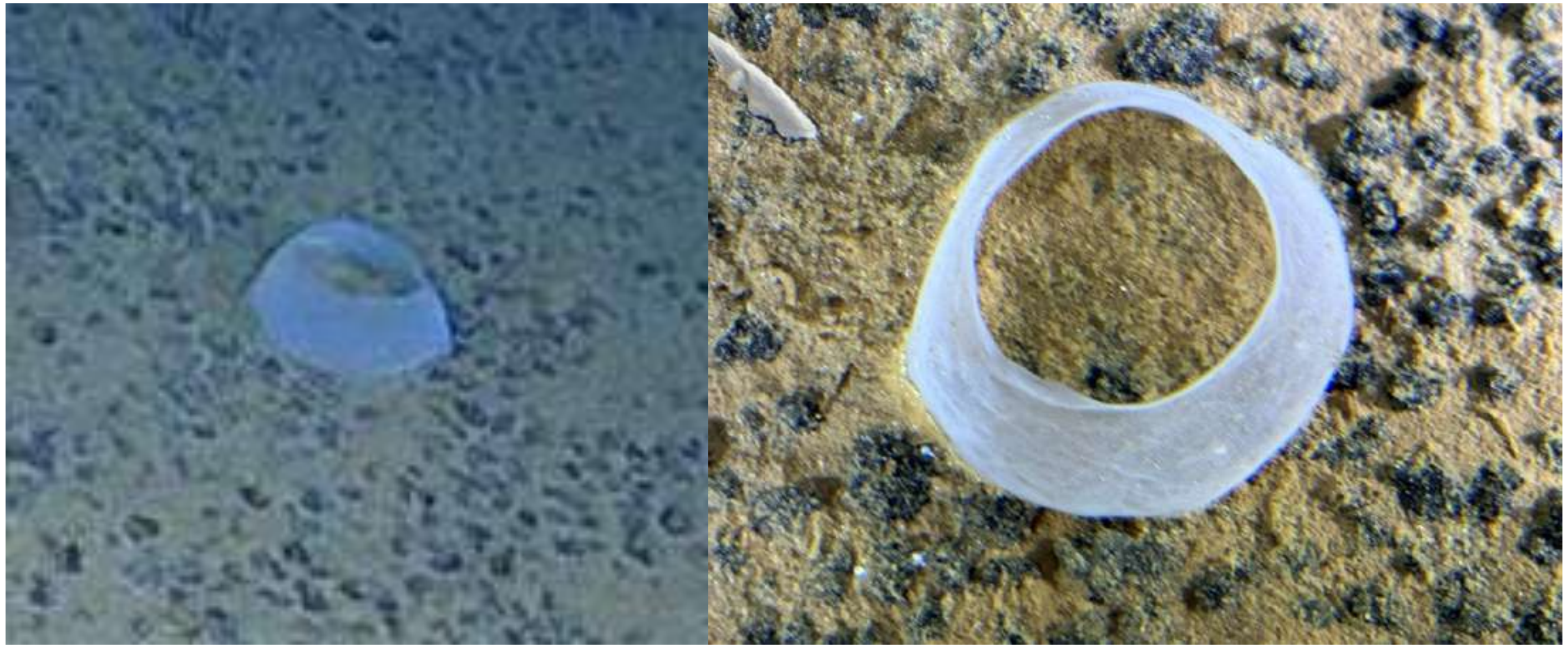

Nest shaped sponge with very thin and smooth body walls. Atrium is open on the substrate.

Hexactinellida, Lyssacinosida
Euplectellidae; Holascus sp. indet. 2

NCT3_BK2_6000_NOR_0063 – 04:49

NCT3_BK2_6000 - IMG_1570

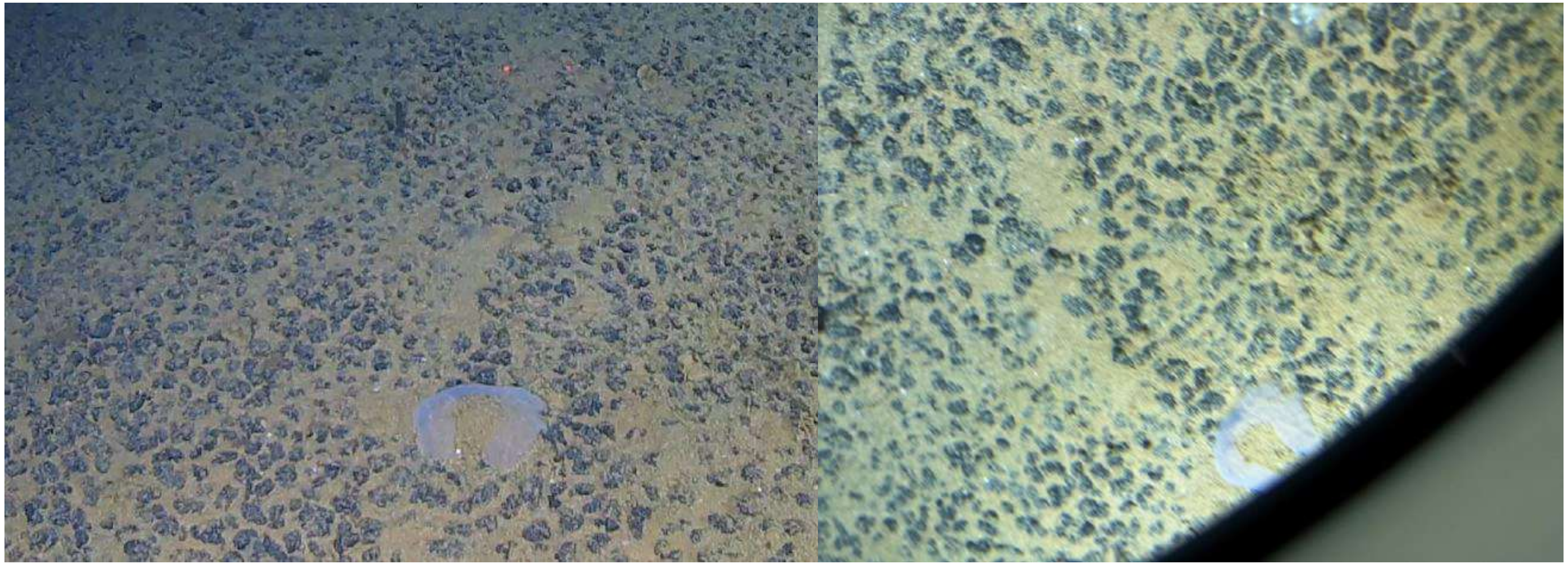

Nest shaped sponge with very thin and smooth body walls not closing completely. Atrium is open on the substrate. Could be a damaged or growing form of sp. indet. 1

Hexactinellida, Lyssacinosida
Euplectellidae; Hyalostylus sp. indet. 1

NCT3_BK2_6000_NOR_0078 – 01:25

NCT3_BK2_6000 - IMG_2087

NCT3_BK2_6000 - IMG_2314

NCT3_BK3_6000_NOR_0075 – 03:20

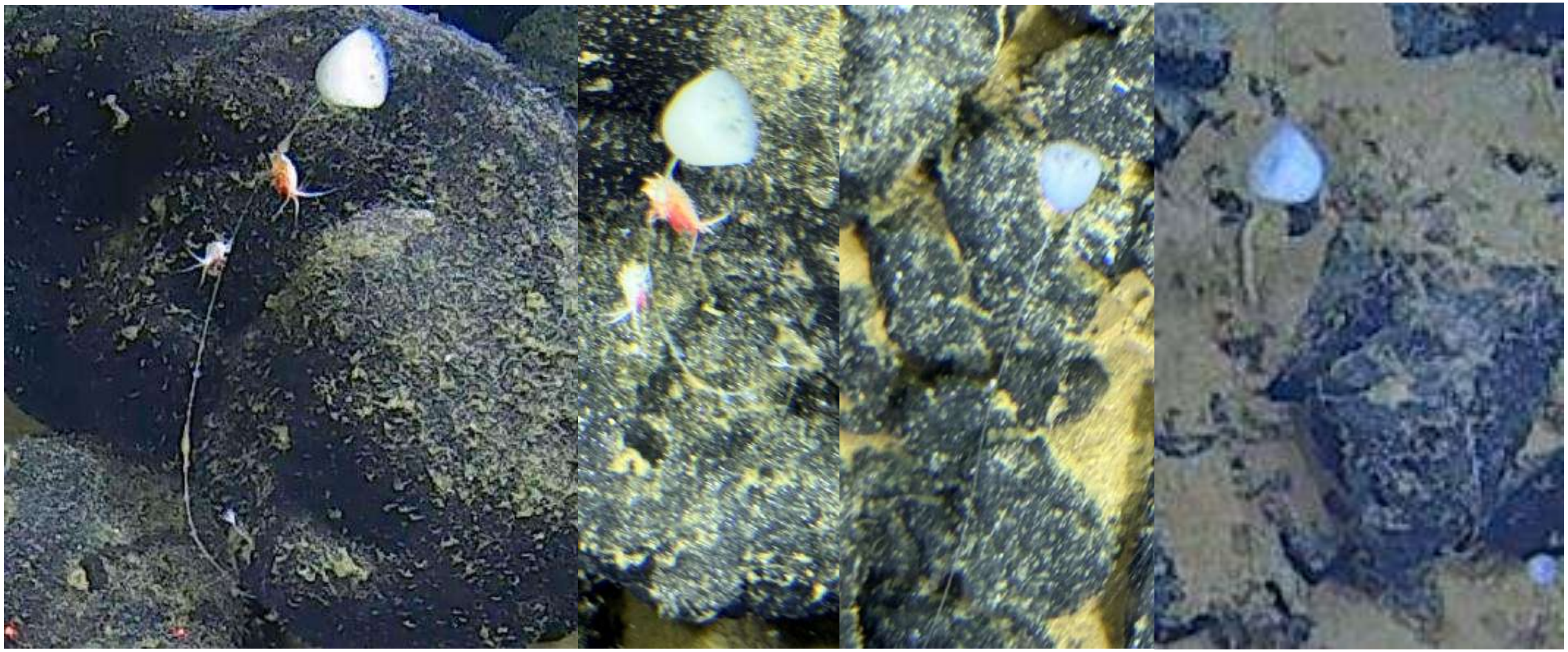

Thin stalked sponge found on hard substrate, with a bell-shaped body ending with multiple oscula. *Amathillopsis* amphipods are commonly attached to the stalk.

Hexactinellida, Lyssacinosida
Euplectellidae; Hyalostylus sp. indet. 2

NCT3_BK2_6000_NOR_0087 – 03:46

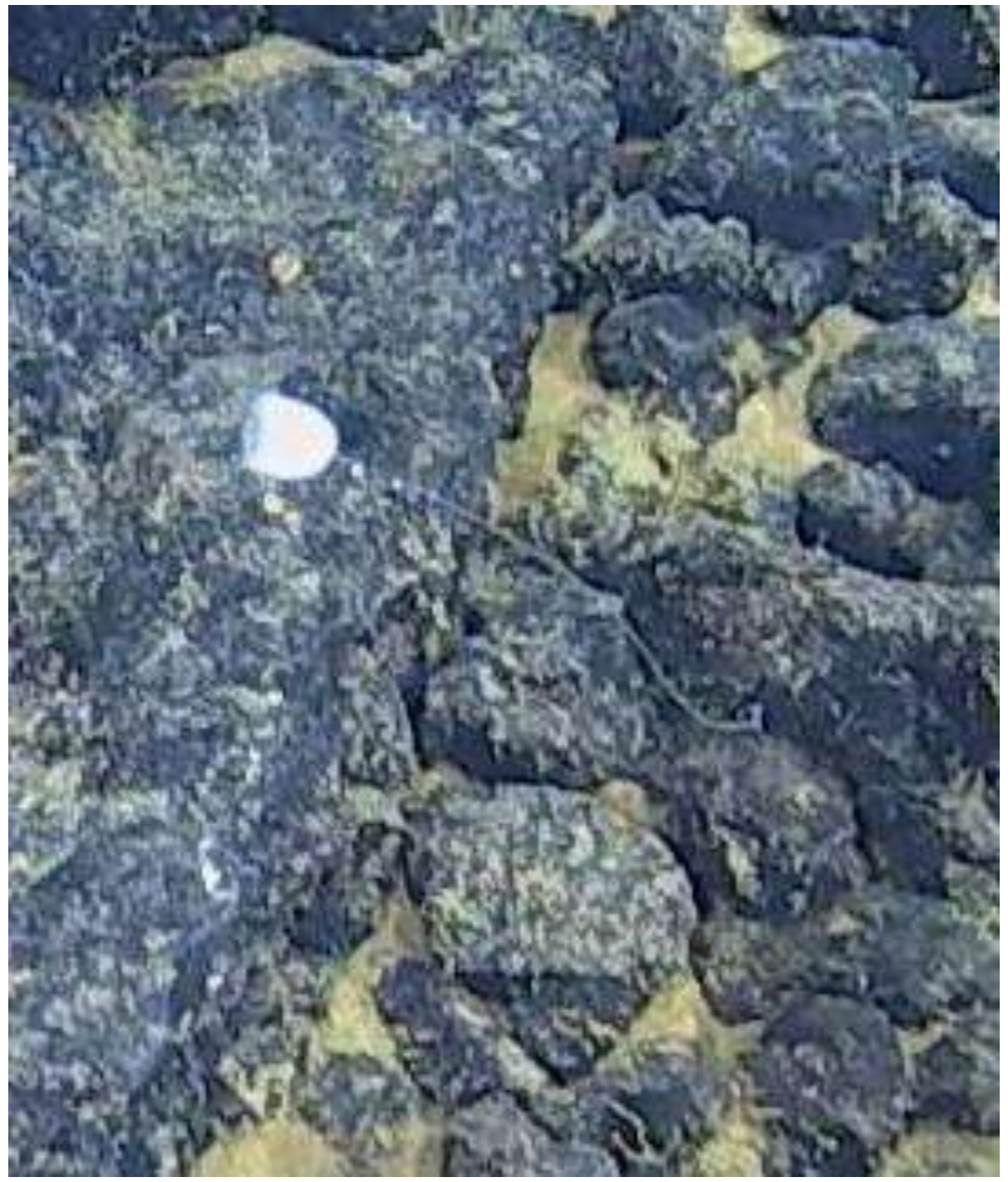

Stalked sponge found on hard substrate, with a cylindrical body. Stalk is thicker than sp. indet. 1. Central major osculum and small openings surrounding it?

Hexactinellida, Lyssacinosida
Euplectellidae; Hyalostylus sp. indet. 3

NCT3_BK2_6000_NOR_0096 – 04:25

NCT4_BK6_4600_NOR_0065 – 01:16

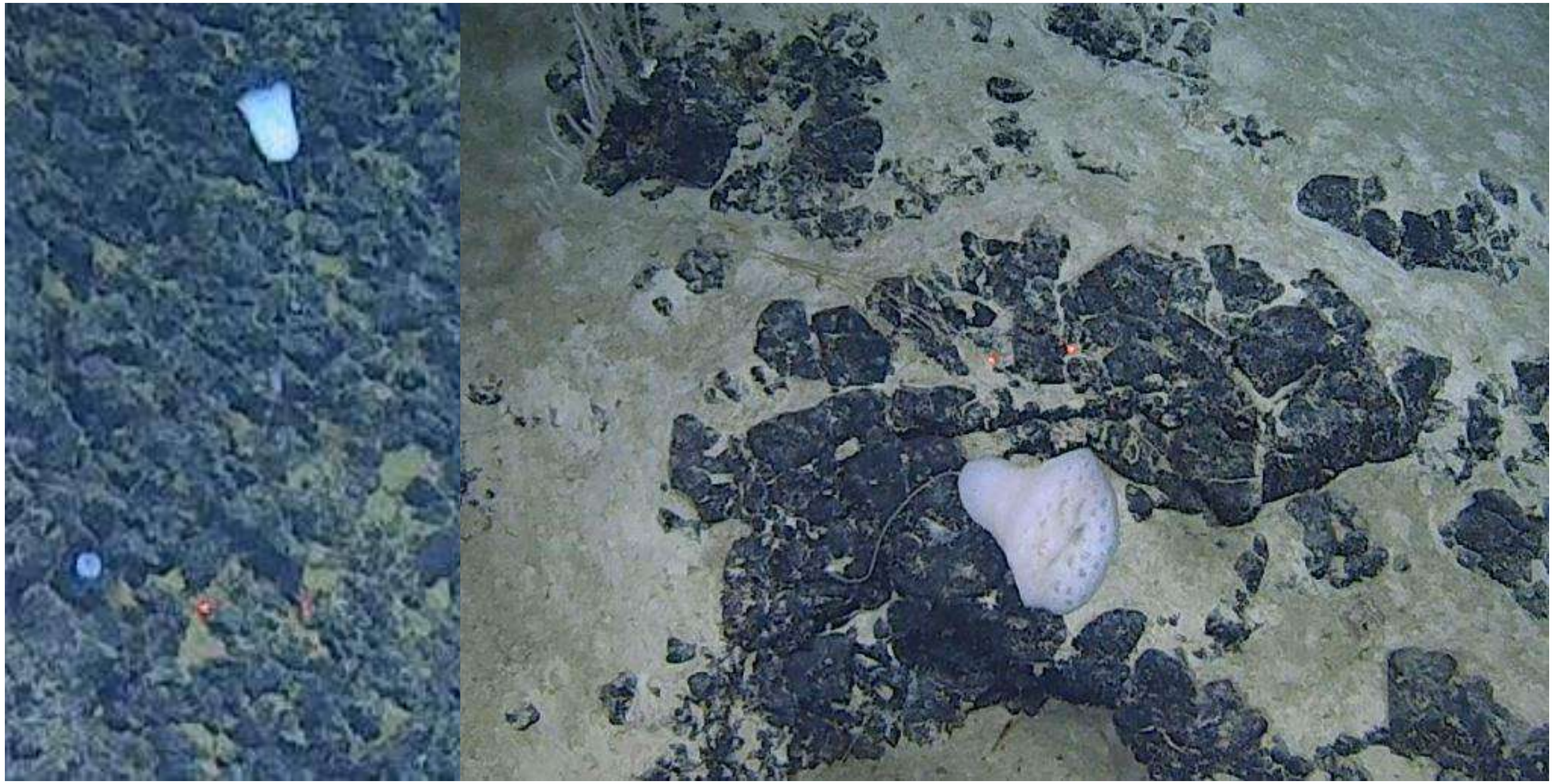

Thin stalked sponge found on hard substrate, with an elongated and thinner bell-shaped body compared to *Hyalostylus* sp. indet 1.

Hexactinellida, Lyssacinosida
Rossellidae; Caulophacus sp. indet. 1

NCT3_BK2_6000_NOR_0087 – 00:38

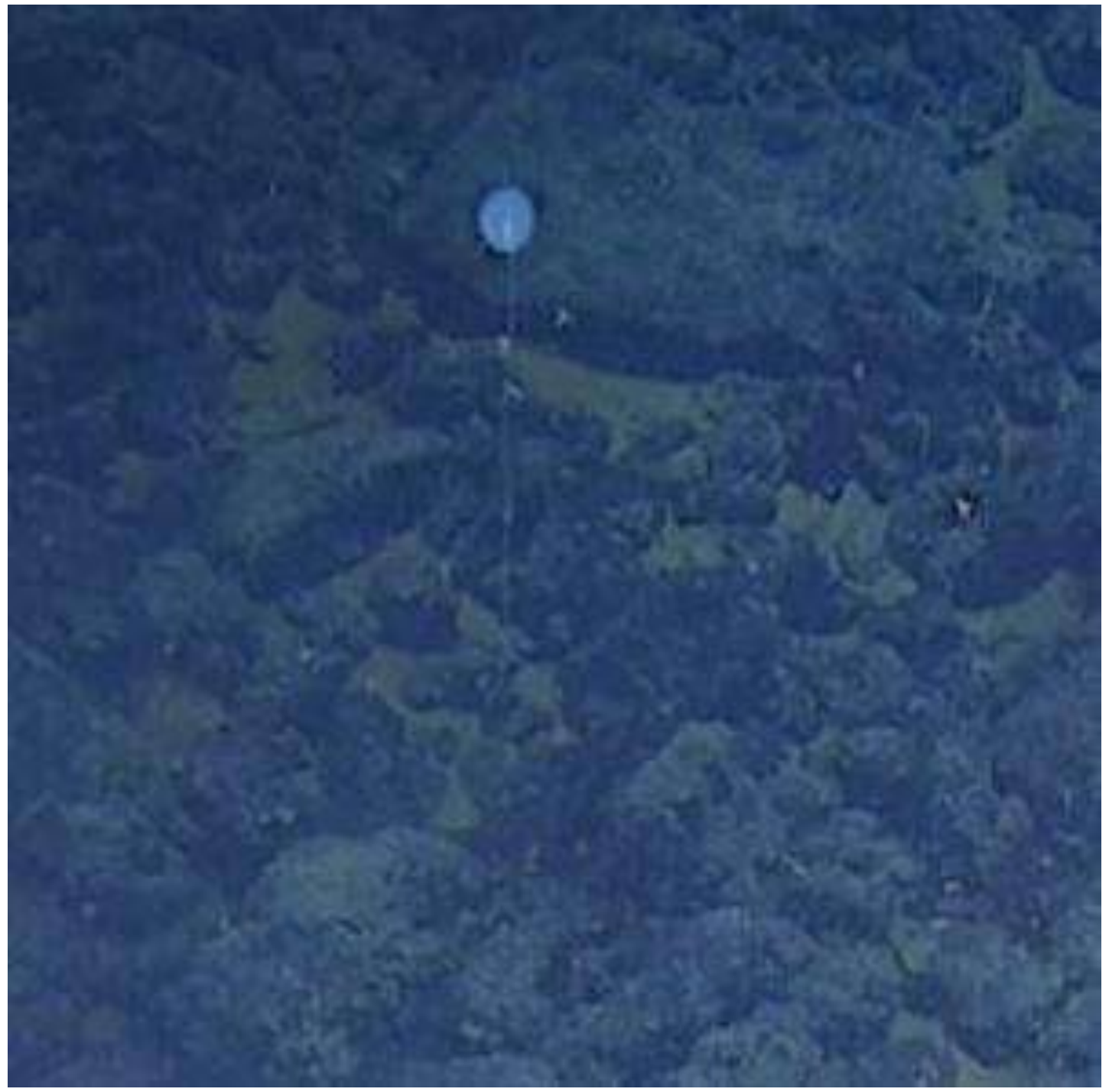

Very long thin stalked sponge with a rounded body

Hexactinellida, Sceptrulophora
Euretidae fam. inc. 1

NCT3_BK2_6000 – IMG_2481

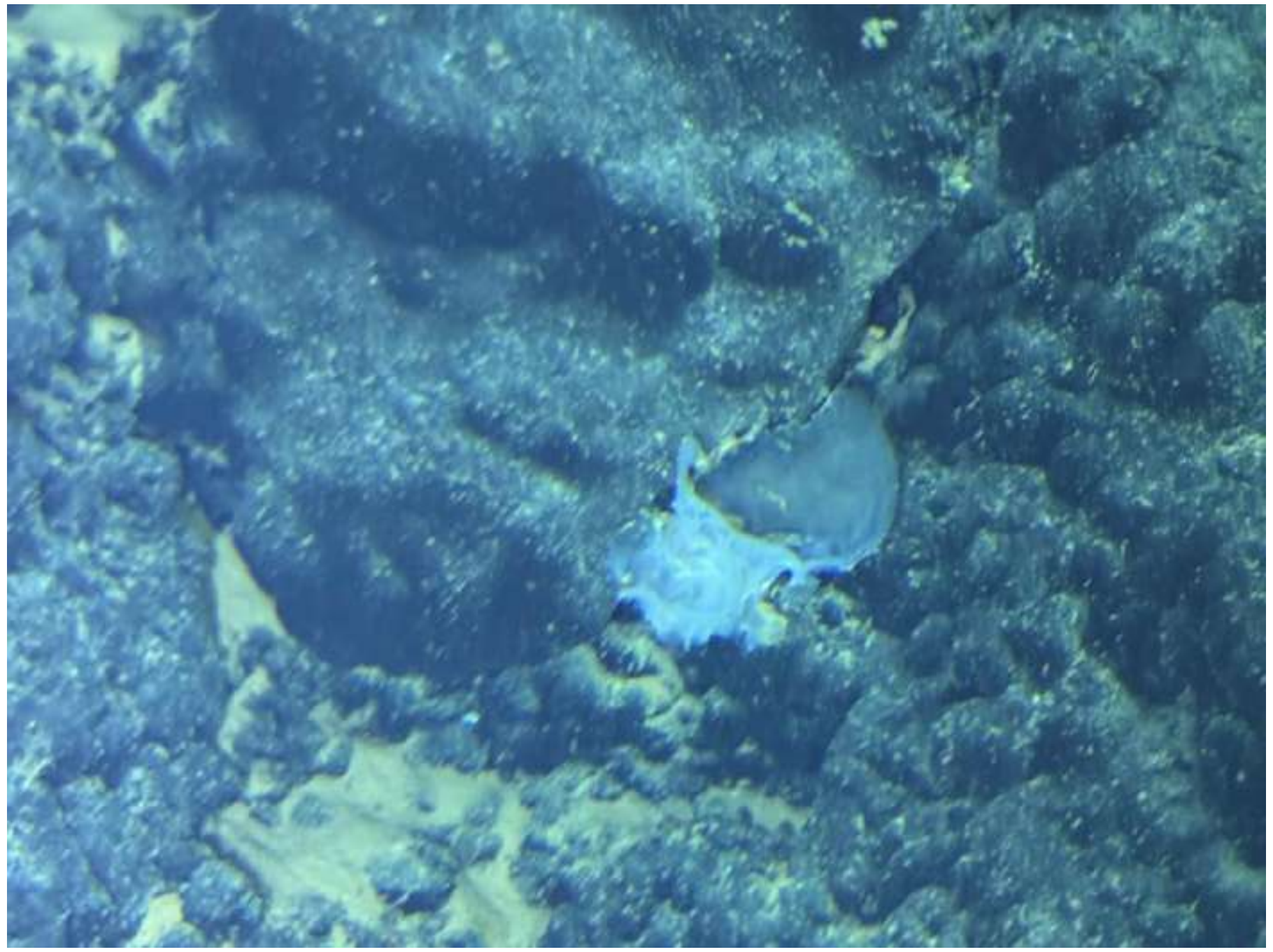

Irregular funnel morphology, *Chonelasma?* Not found in video [double check EM].

Hexactinellida, Sceptrulophora
Euretidae gen. inc. 2

NCT3_BK3_6000 – IMG_3237

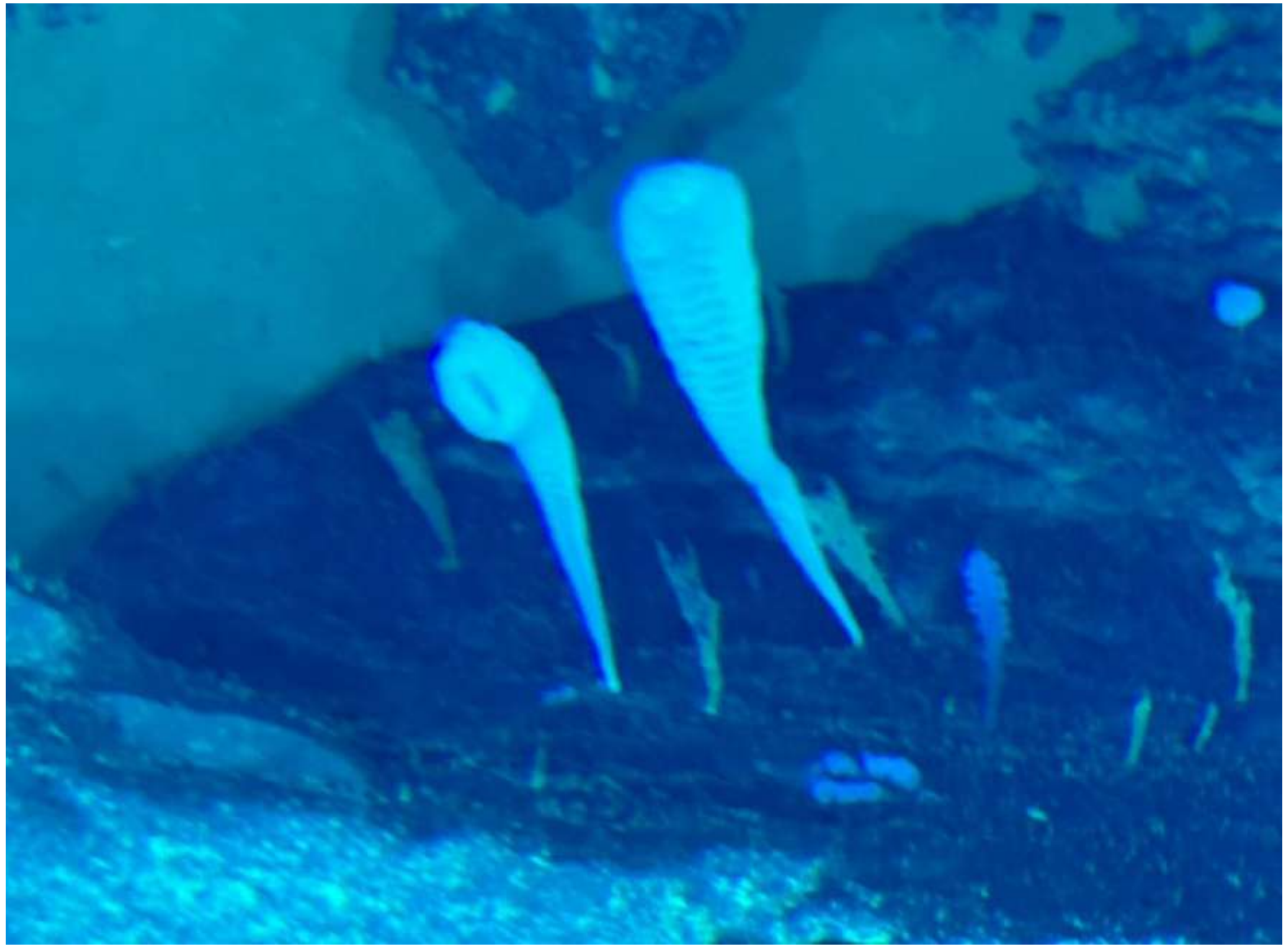

Long funnel morphology, almost a barrel, but with a very small attachment. Radial structures can be observed on the side. *Lefroyella*?

Hexactinellida, Sceptrulophora
Euretidae gen. inc. 3

NCT3_BK3_6000 – IMG_3533

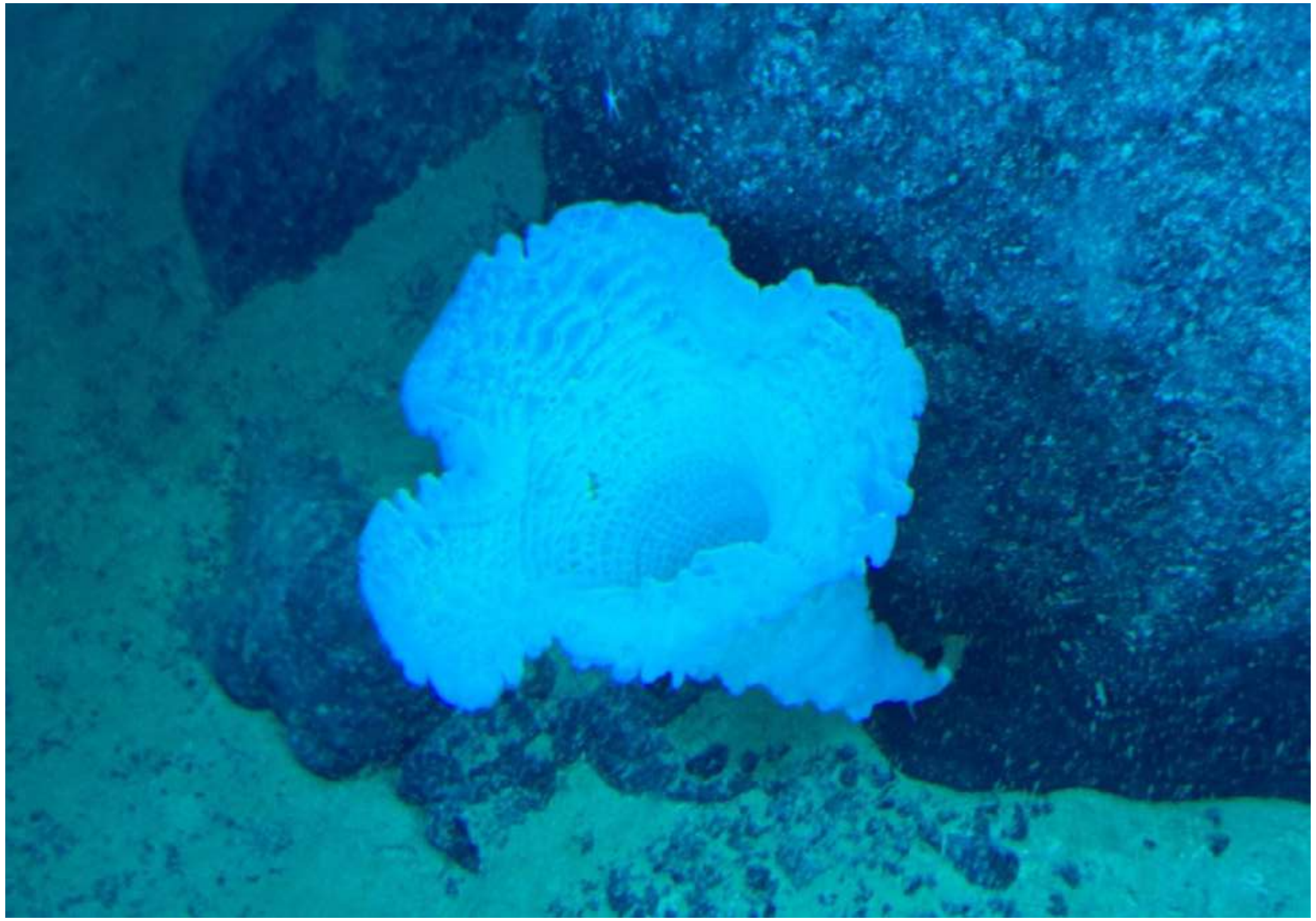

Long funnel morphology, with a very small attachment area; osculum surrounde by a flowering structure on four sides. Radial structures can be observed on the side.
Lefroyella?

Hexactinellida, Sceptrulophora
Euretidae; Periphragella gen. inc. 1

NCT3_BK2_6000_NOR_0097 – 01:51

NCT3_BK2_6000 – IMG_2498

NCT3_BK2_6000 – IMG_2509

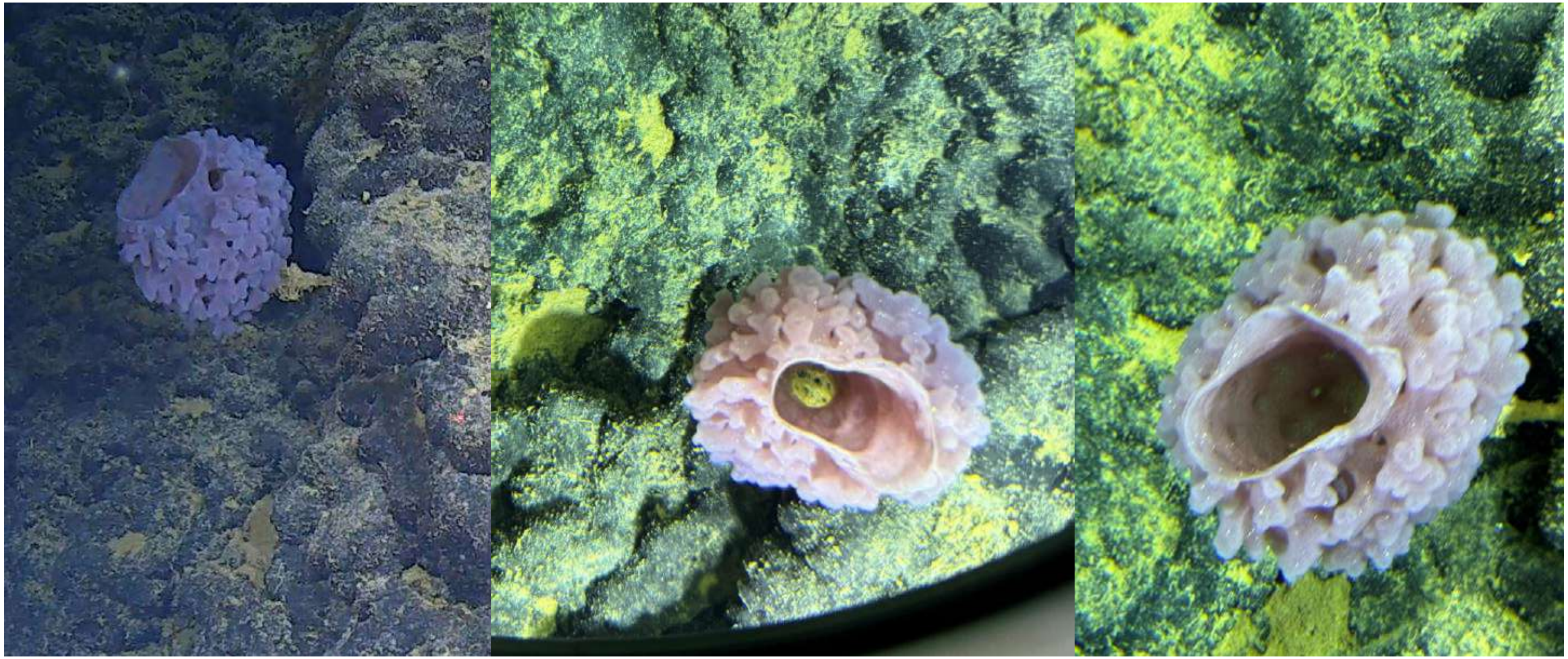

Pink coloured barrel sponge with a defined osculum and many dichotomous short tubes departing from the body. This morphotype have a short peduncle that looks covered in forams, not sharing the same pink colour of the main body.

Hexactinellida, Sceptrulophora
Euretidae; *Periphragella* gen. inc. 2

NCT3_BK3_6000 – IMG_2981

NCT3_BK3_6000_NOR_0074 – 00:36

NCT3_BK3_6000 – IMG_3028

NCT3_BK3_6000 – IMG_3402

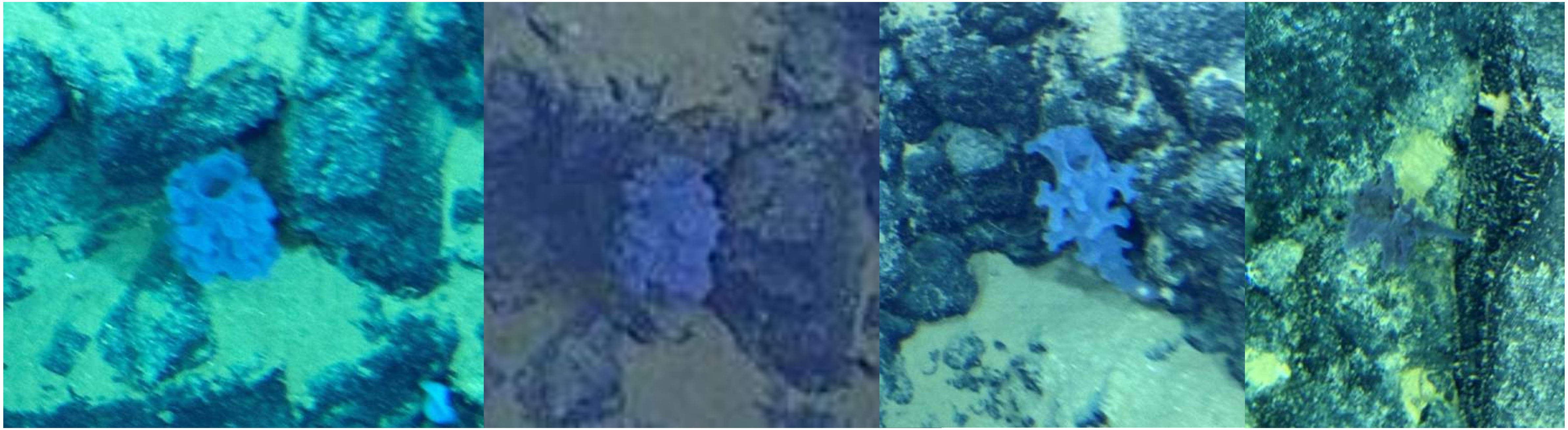

Complex funnel sponge with a thin attachment developing into an open atrium, with dichotomous short tubes departing from the main body. Developing forms have a more elongated morphology, thinner tubes and osculum, and sometimes the osculum looks “incomplete”. Compared to *Periphragella* 1, this has a body taller than wider, a shorter and smaller attachment, and a blue colour. They share a similar osculum edge.

Hexactinellida, Sceptrulophora
Farreaidae gen. inc. 1

NCT3_BK3_6000 – IMG_3237

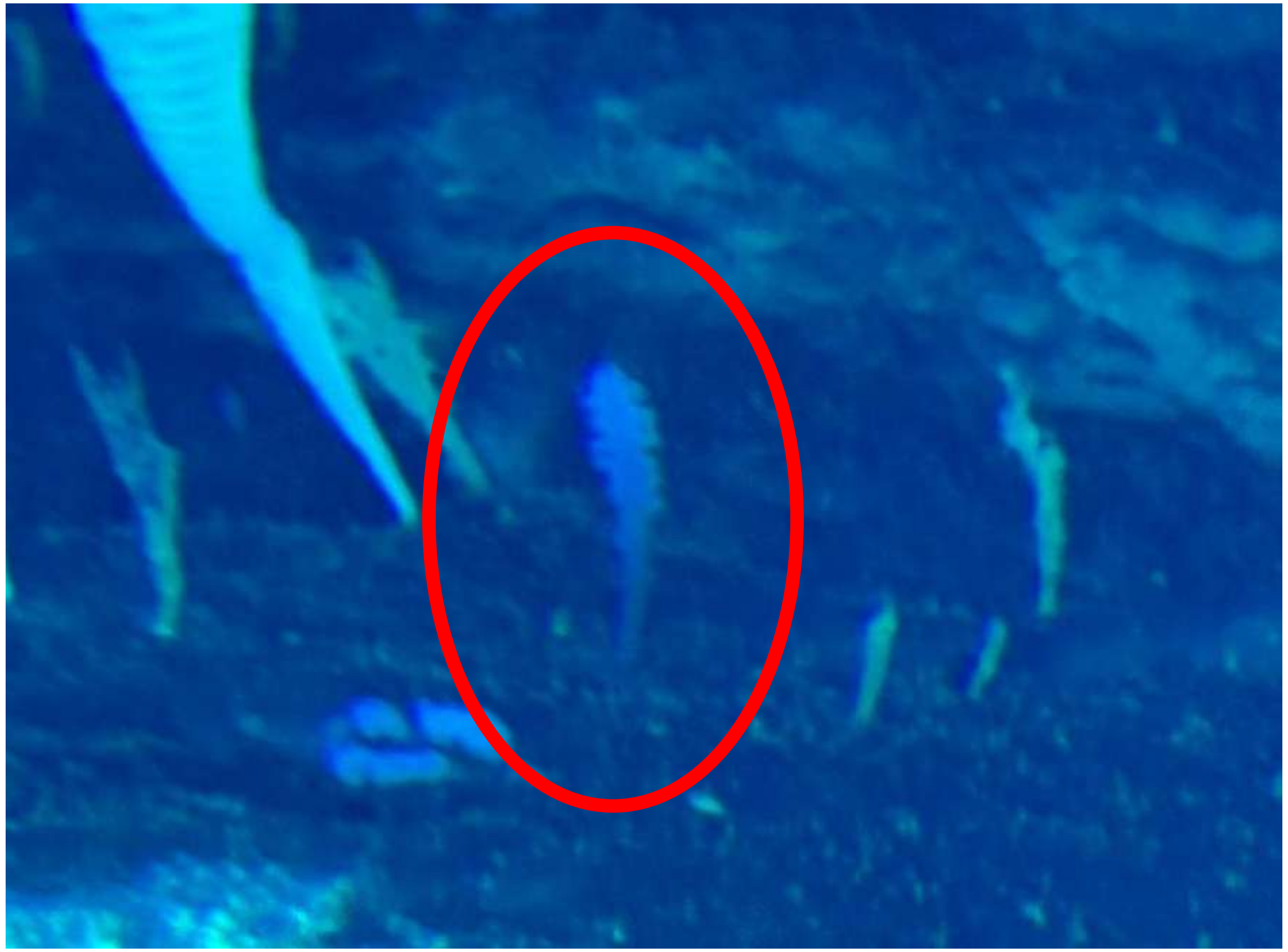

Erect sponge with digitations from the side growing from the central part. Looks flat, different from juveniles of Periphragella 2, which are more 3-dimensional.

Unidentified Hexactinellida Examples

PORIFERA
Stalked Hexactinellida spp.

NCT3_BK2_6000_NOR_0082 – 03:33

NCT3_BK2_6000_NOR_0083 – 00:41

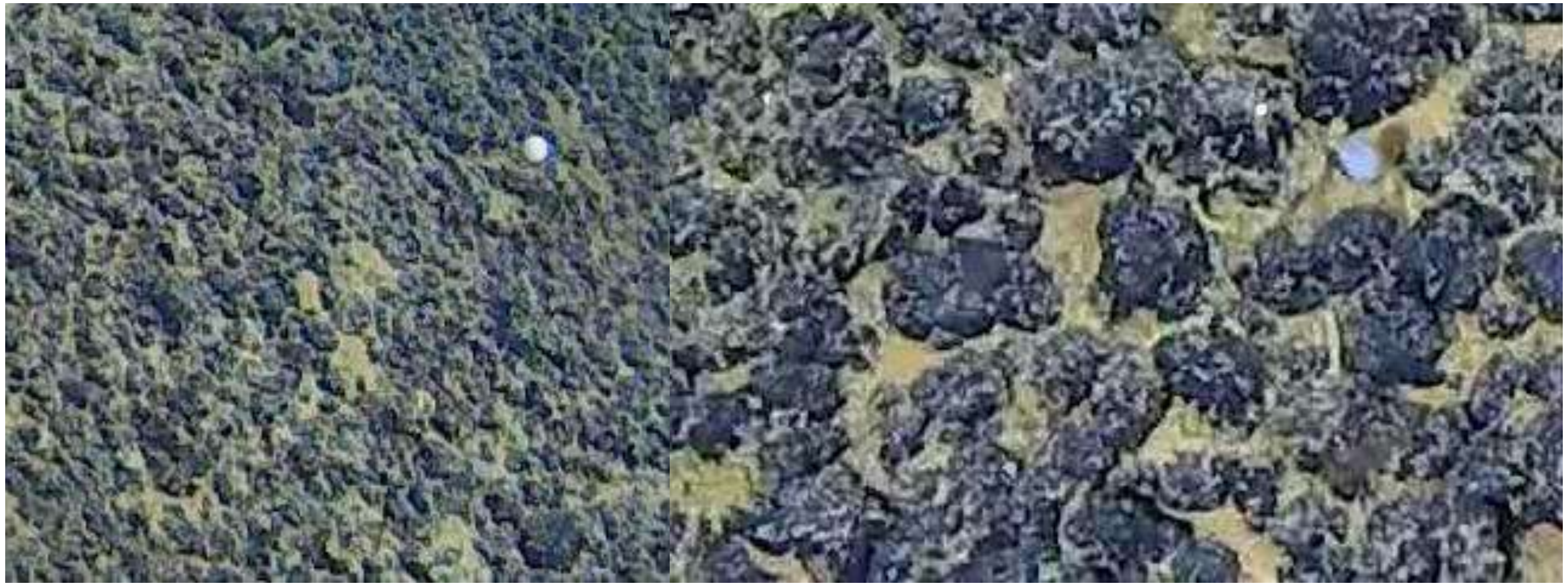

Bin morphotaxa. Bad quality pictures of stalked specimen, no proper mtp assigned.

PORIFERA
Hexactinellida spp.

NCT2_BK4_5800_NOR_0052 – 01:25

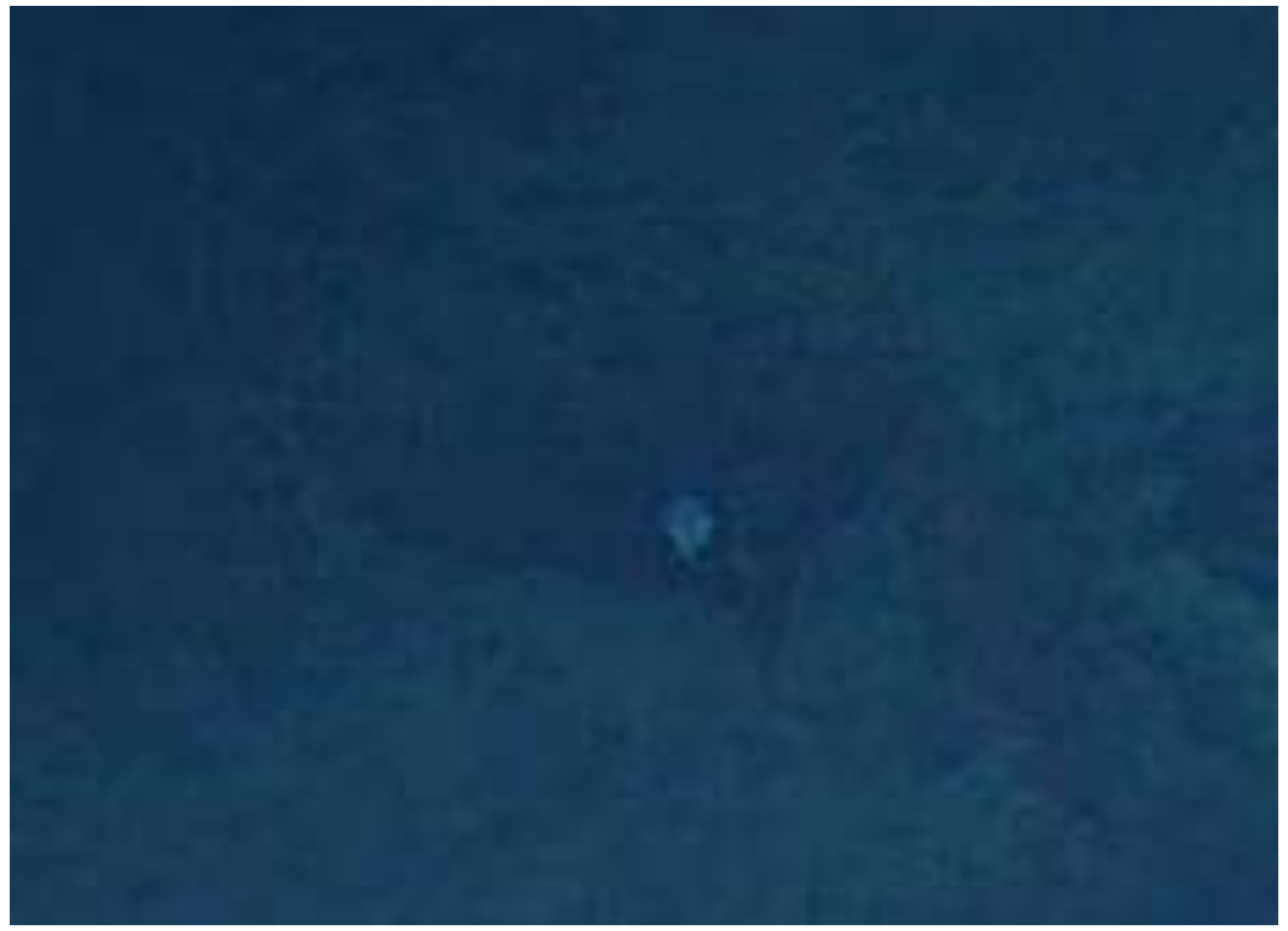

Bin morphotaxa. Bad quality pictures, no proper mtp assigned.

Chaetognatha

Chaetognatha; Chaetognatha spp

NC2_OM7_5000_NOR_0085
NC1_CR1_6200_NOR_0029
NC1_OM2_6000_NOR_0052

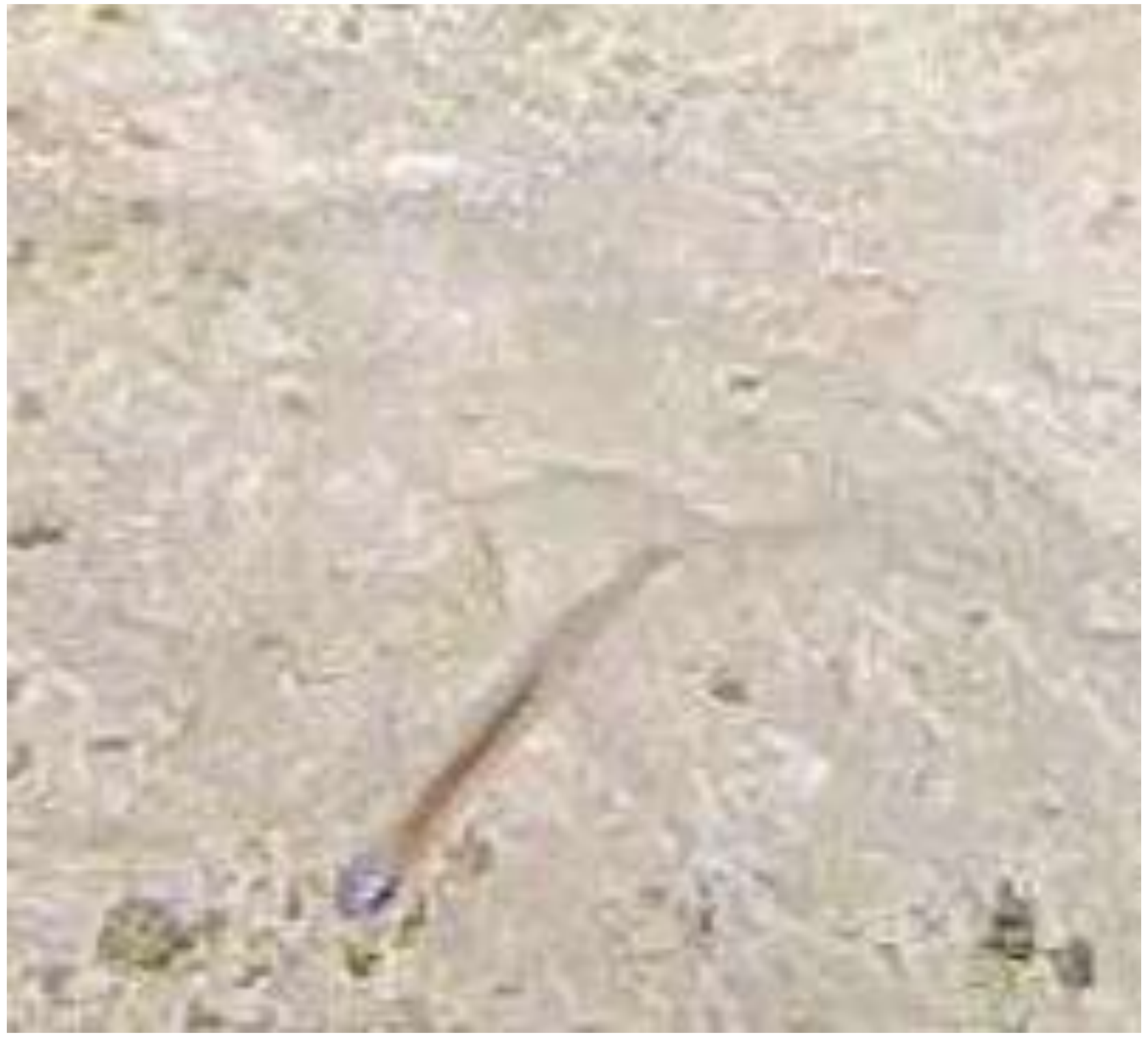
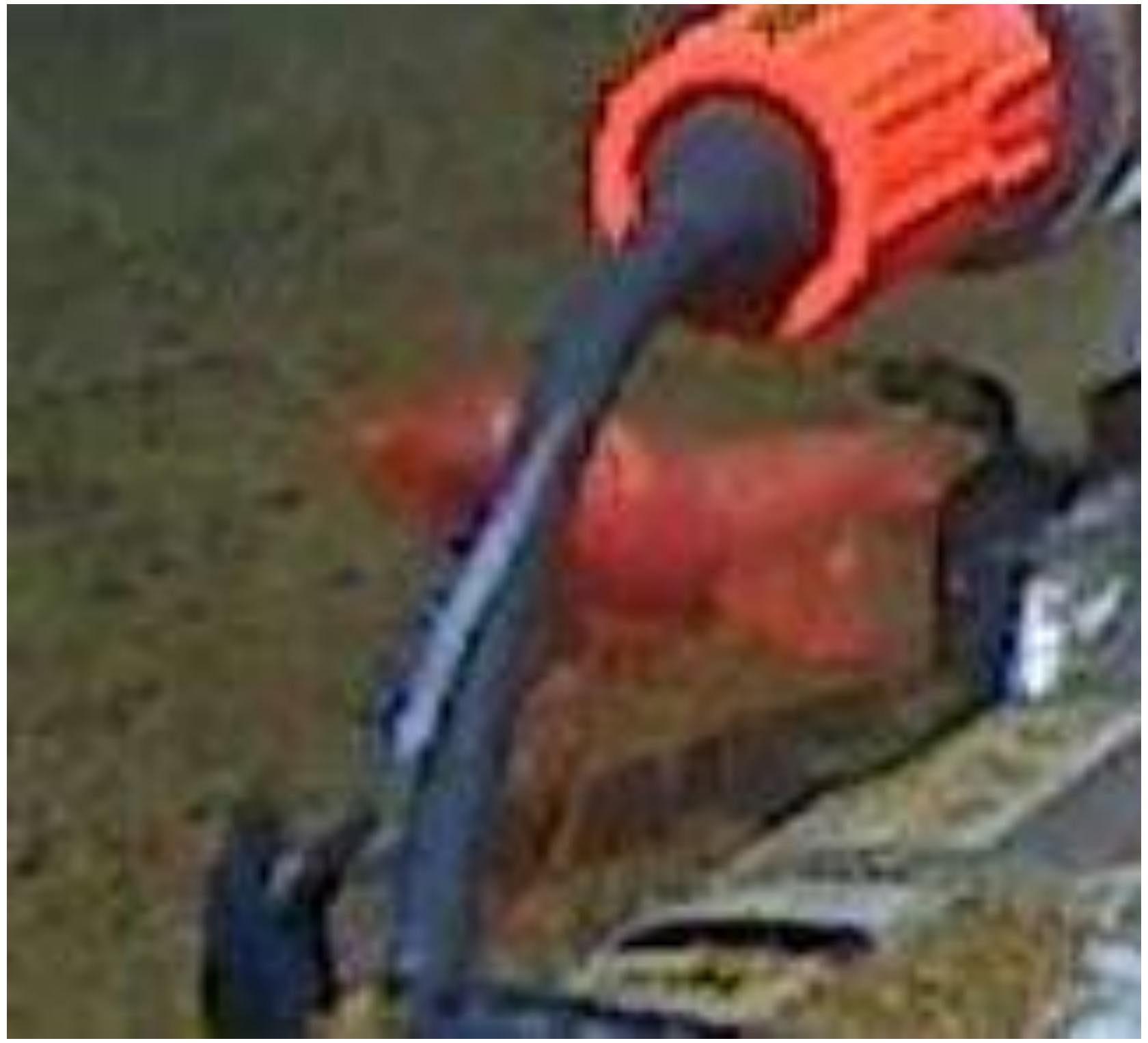
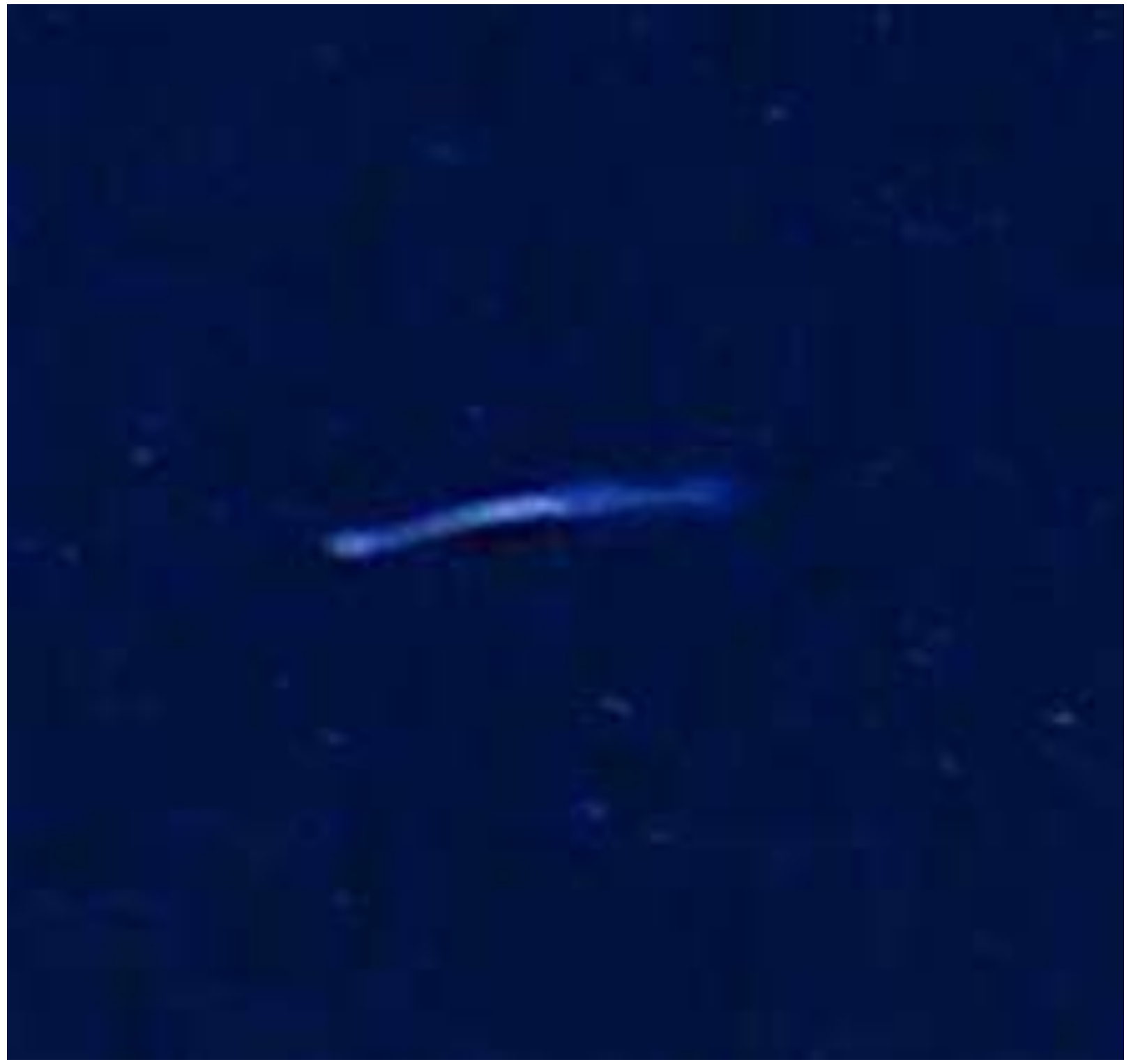

Body is pale-translucent and thin. The “head” is spade shaped and proportionally large in relation to the trunk.

Hemichordata

Enteropneusta; Torquaratoridae; Torquaratoridae gen. indet. 1

NC4_BK2_4700_NOR_0071

NC4_BK2_4700_NOR_0082

NC4_BK2_4700_IMG_5204

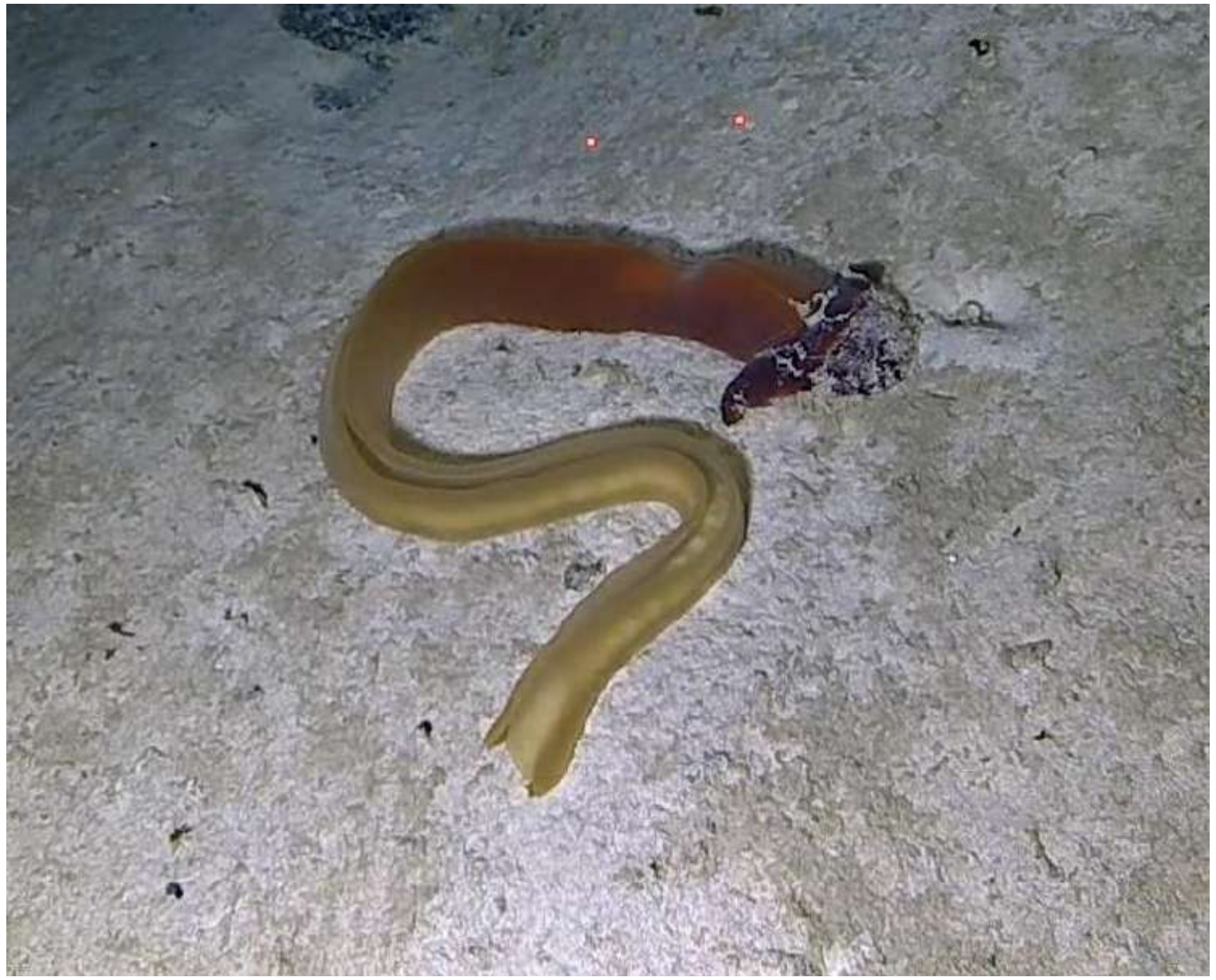
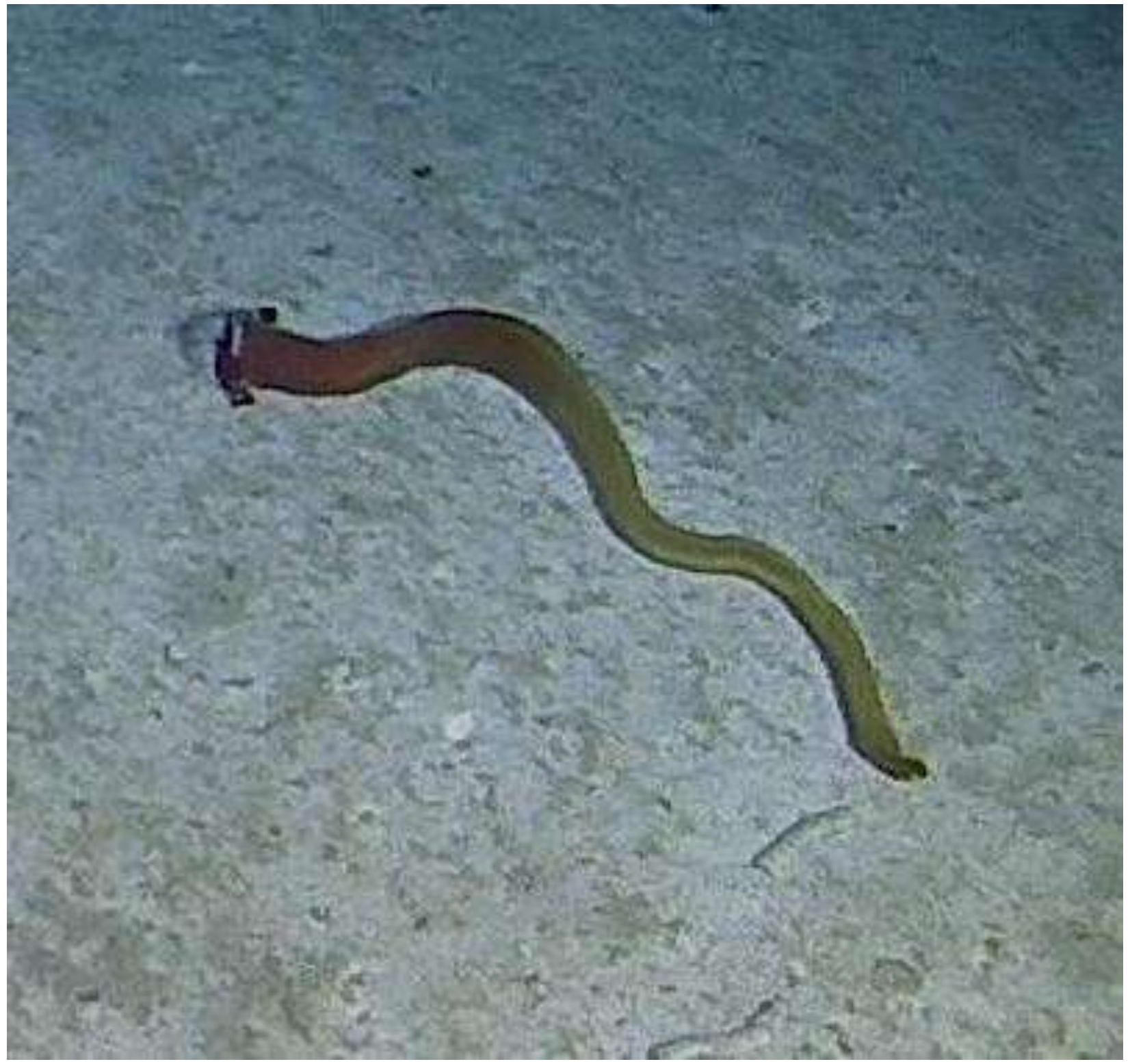
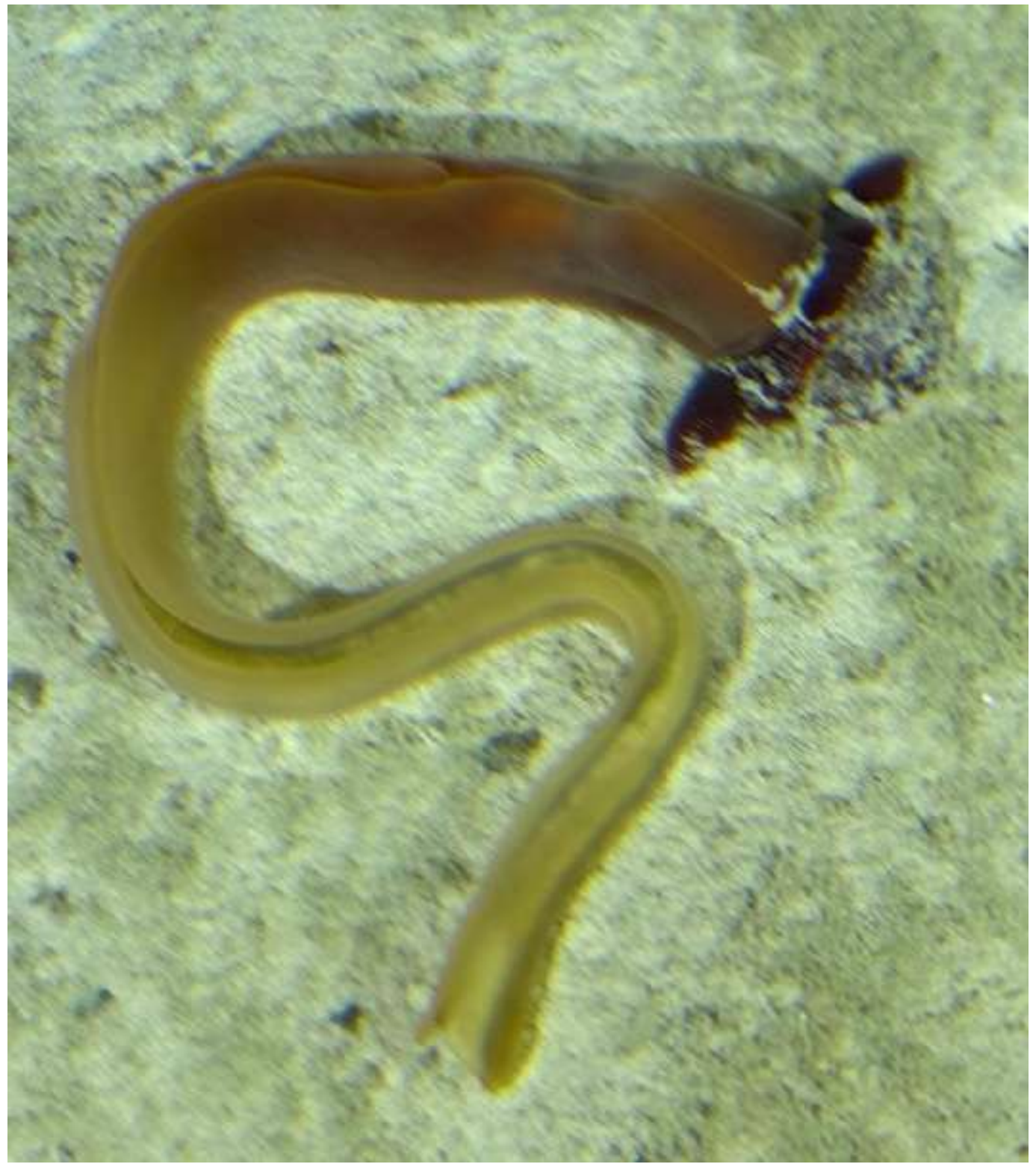

Body is large, partially translucent, and brightly coloured, with anterior end thick and red-orange that tapers to a yellow and slender posterior end. Lateral, gonad-holding folds apparent on both sides of the animal extend from the anterior to posterior end. Mouth is surrounded by two symmetrical triangular lateral collar lips and one rounded midventral collar lip.

Enteropneusta; Torquaratoridae; Torquaratoridae gen. indet. 2

NC1_BK2_6700_NOR_0076
NC1_BK2_6700_NOR_0079

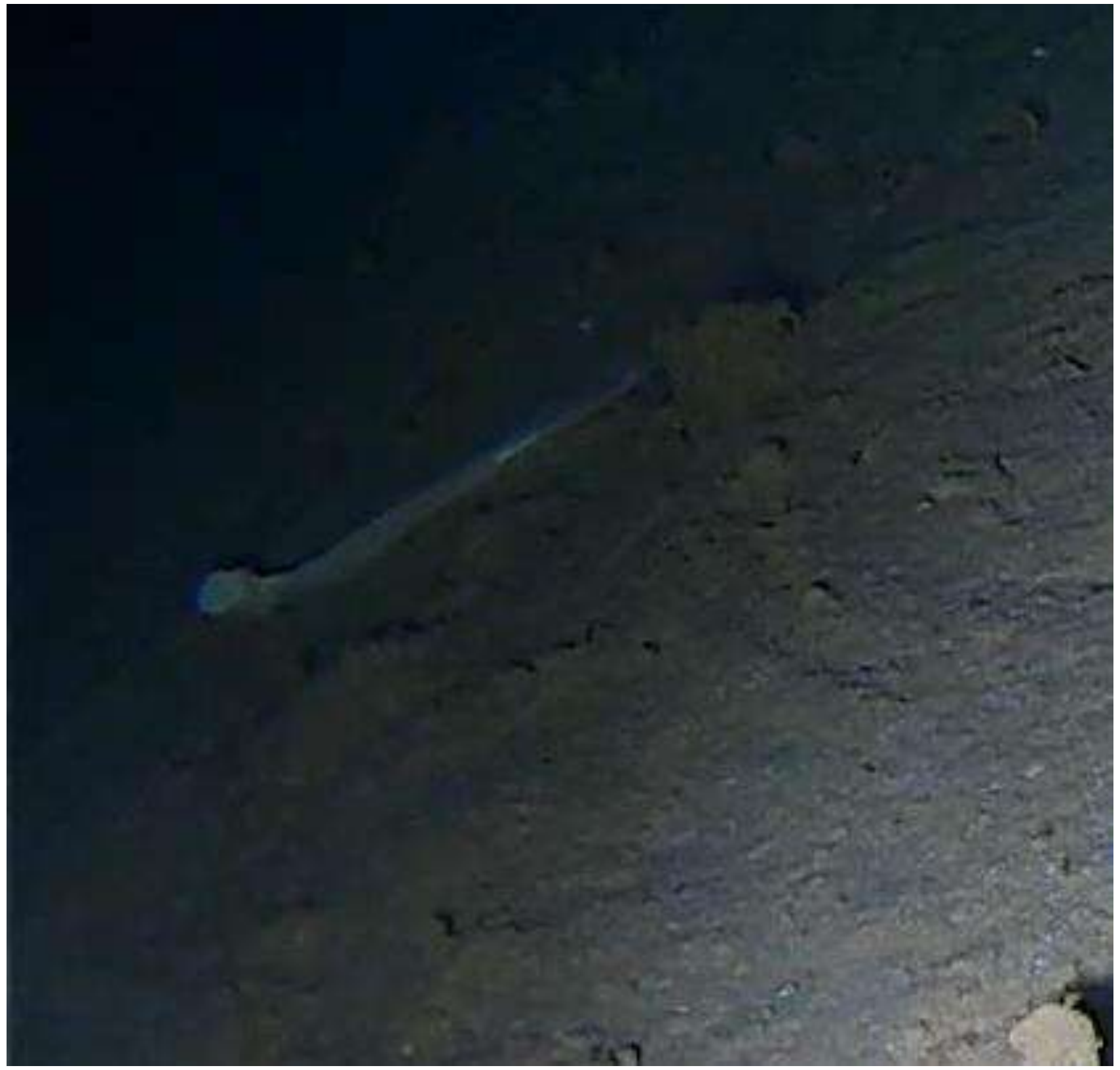

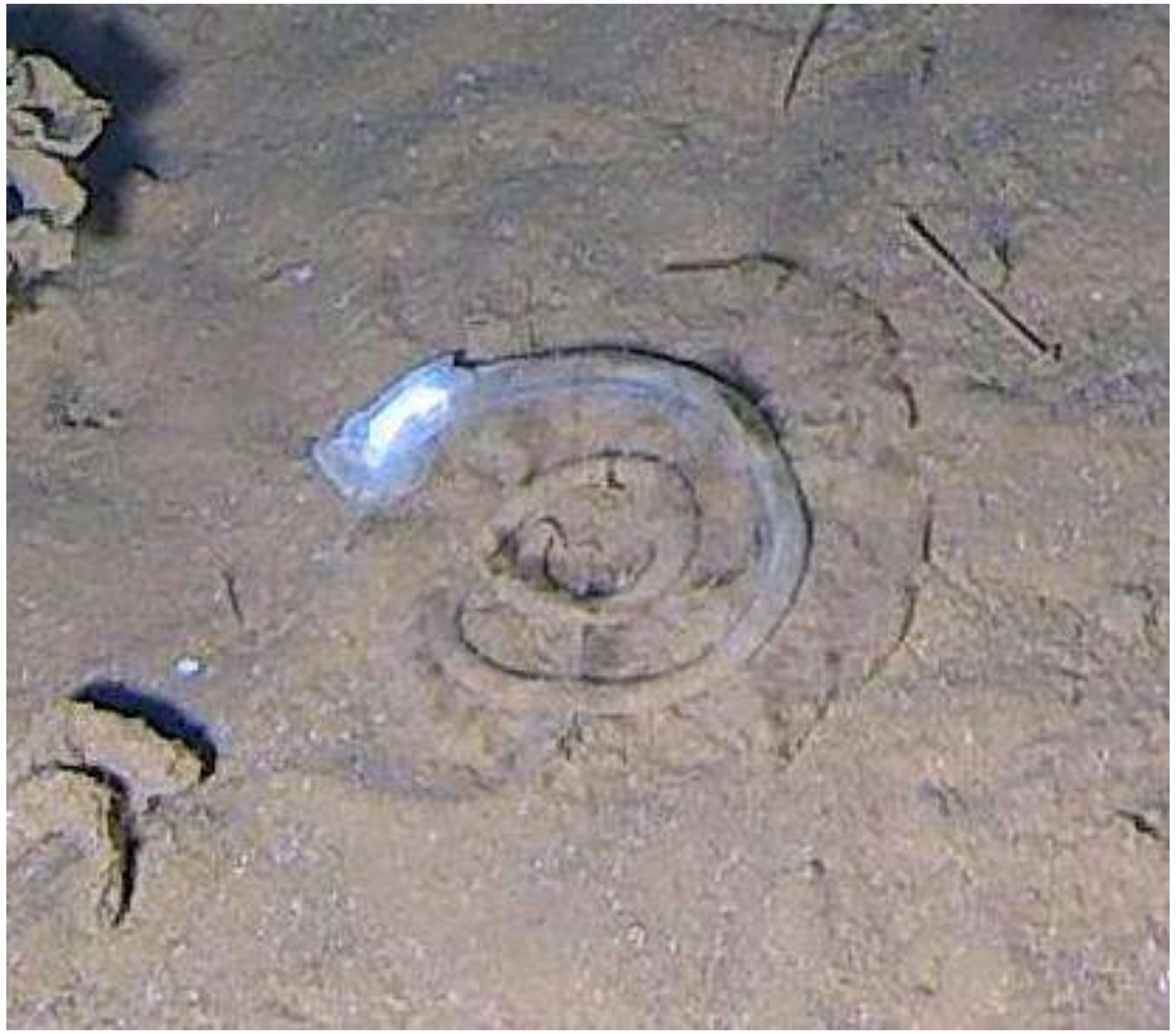

Body is large, and translucent. Lateral, gonad-holding folds apparent on both sides of the animal extend from the anterior to posterior end. Mouth is surrounded by two symmetrical triangular lateral collar lips and one rounded midventral collar lip.

Annelida

NC1_OM2_6000_NOR_0037

Annelida; Annelida spp. 1

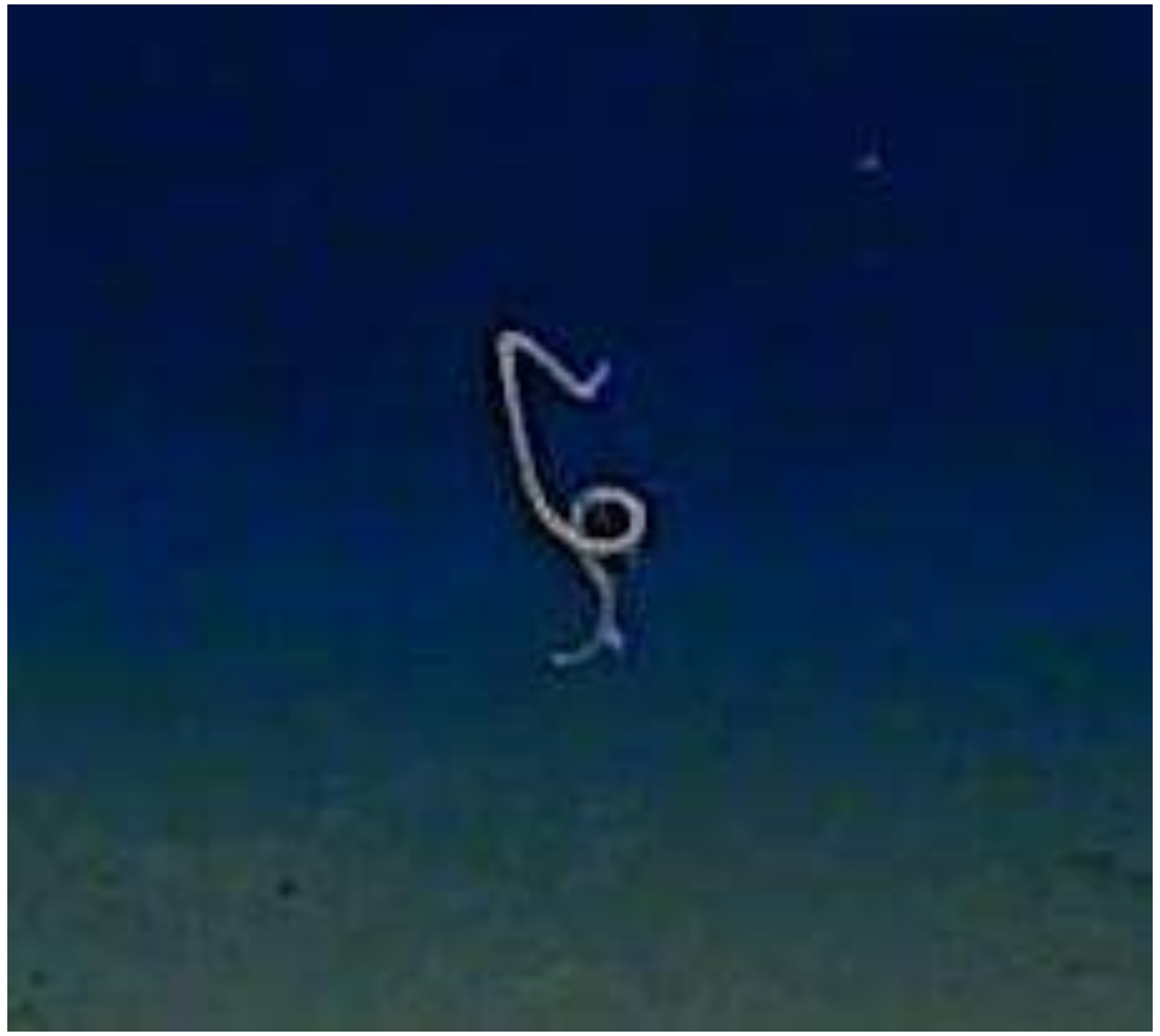

Body is elongated and cylindrical, reminiscent to typical garden worms in shape.

Behavioural Diagnostic: Swimming pattern characterised by erratic looping through the water column.

NC3_MA3_6100_NOR_0084

Annelida spp. 2

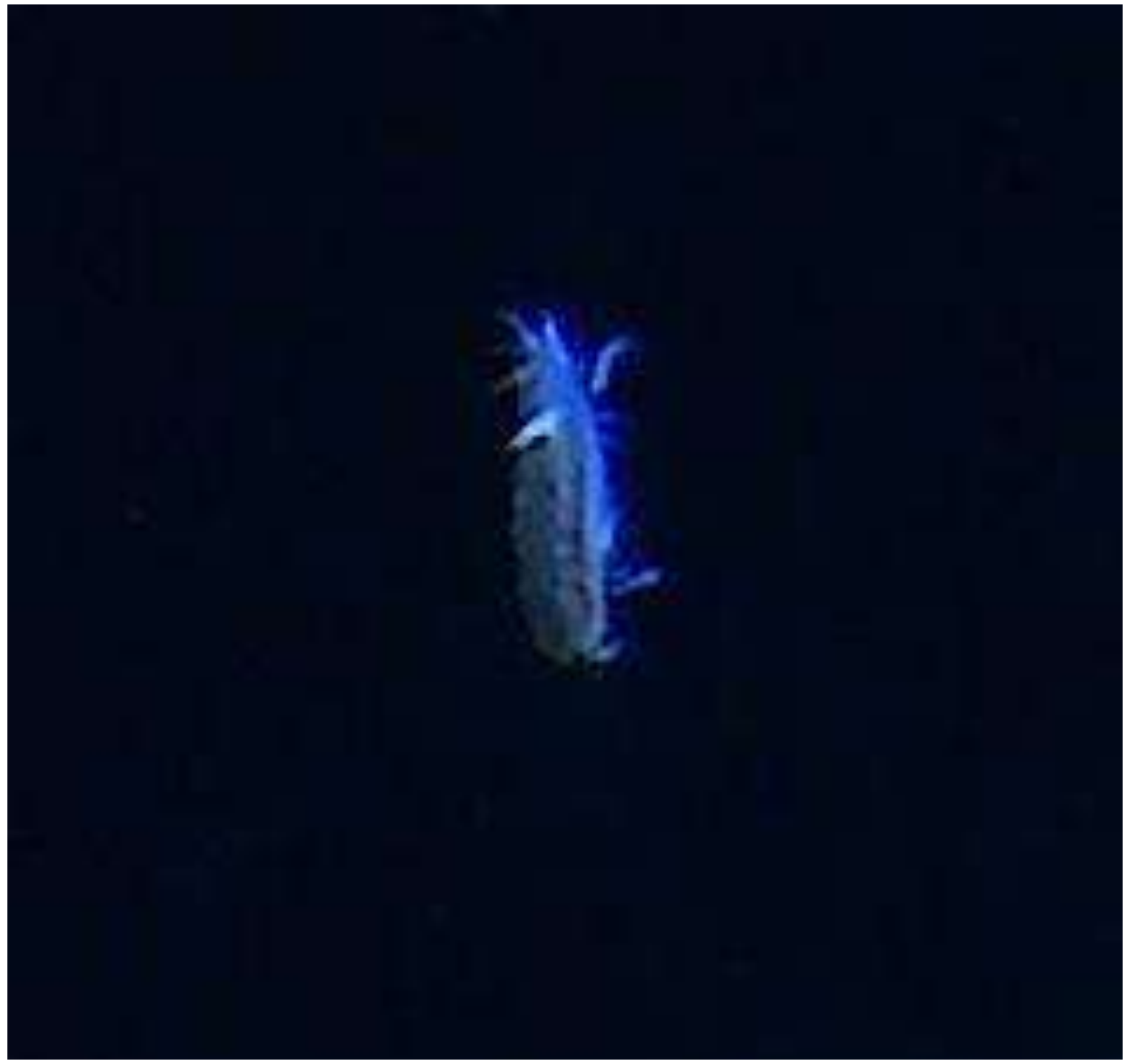

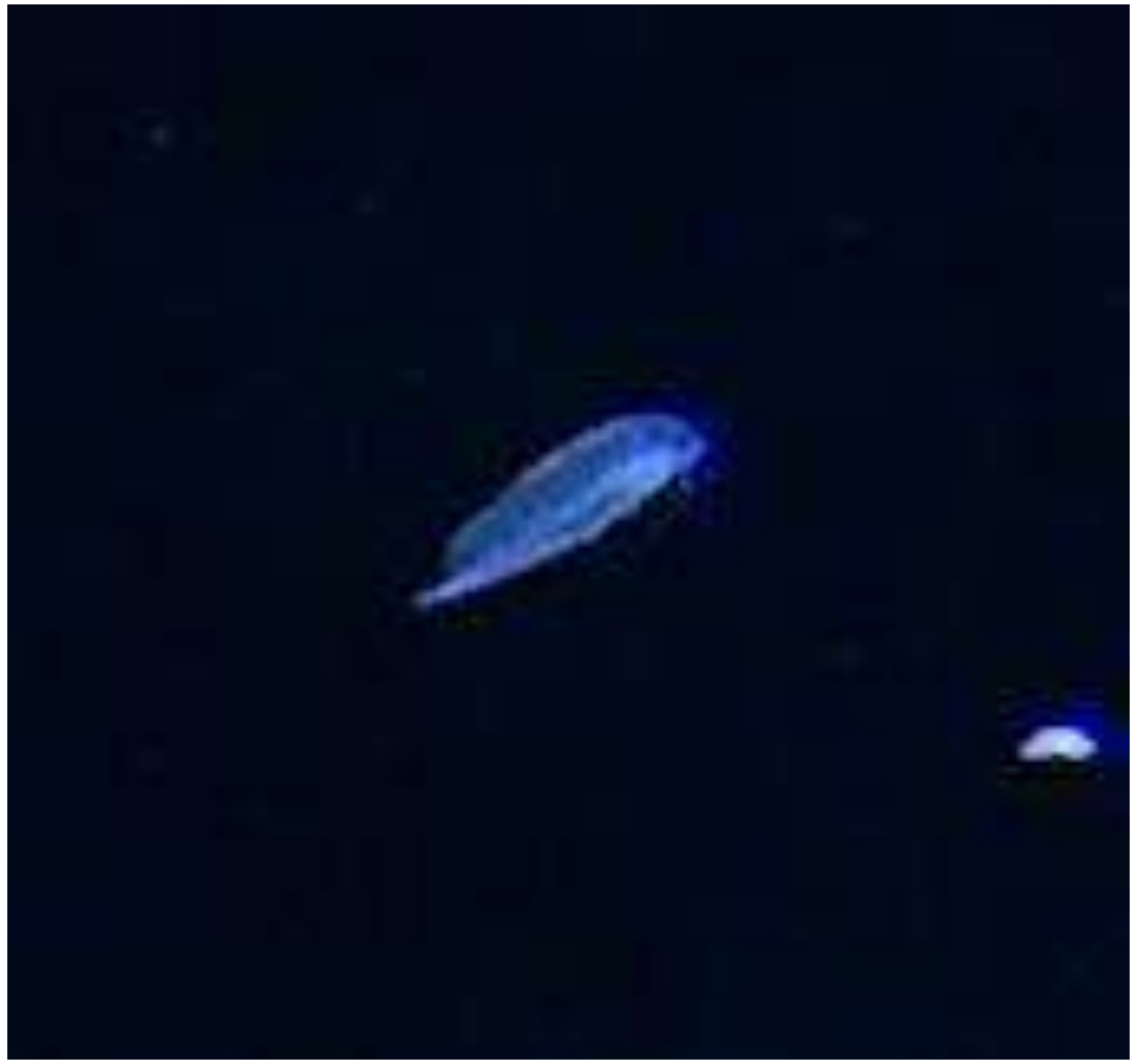

Chaetae visible, but cannot be distinguished as polynoid, acrocirrid or flabelligerid

Polychaeta; Echiura; Echiuroidea; Echiuroidea fam indet.1

NC3_BK4_8000_IMG_3758

NC1_MA6_7800_NOR_0126

NC1_BK3_8000_IMG_5588

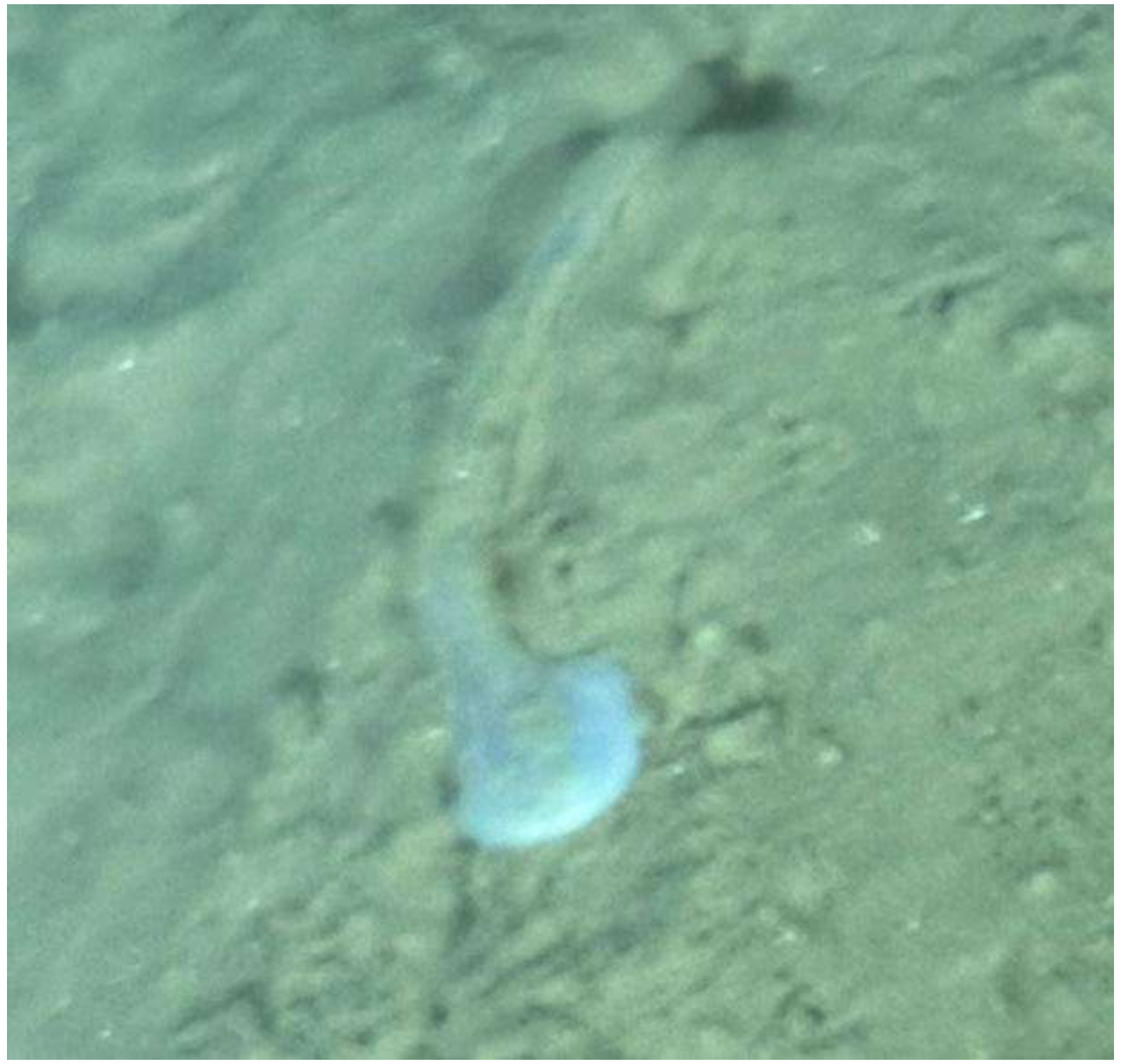

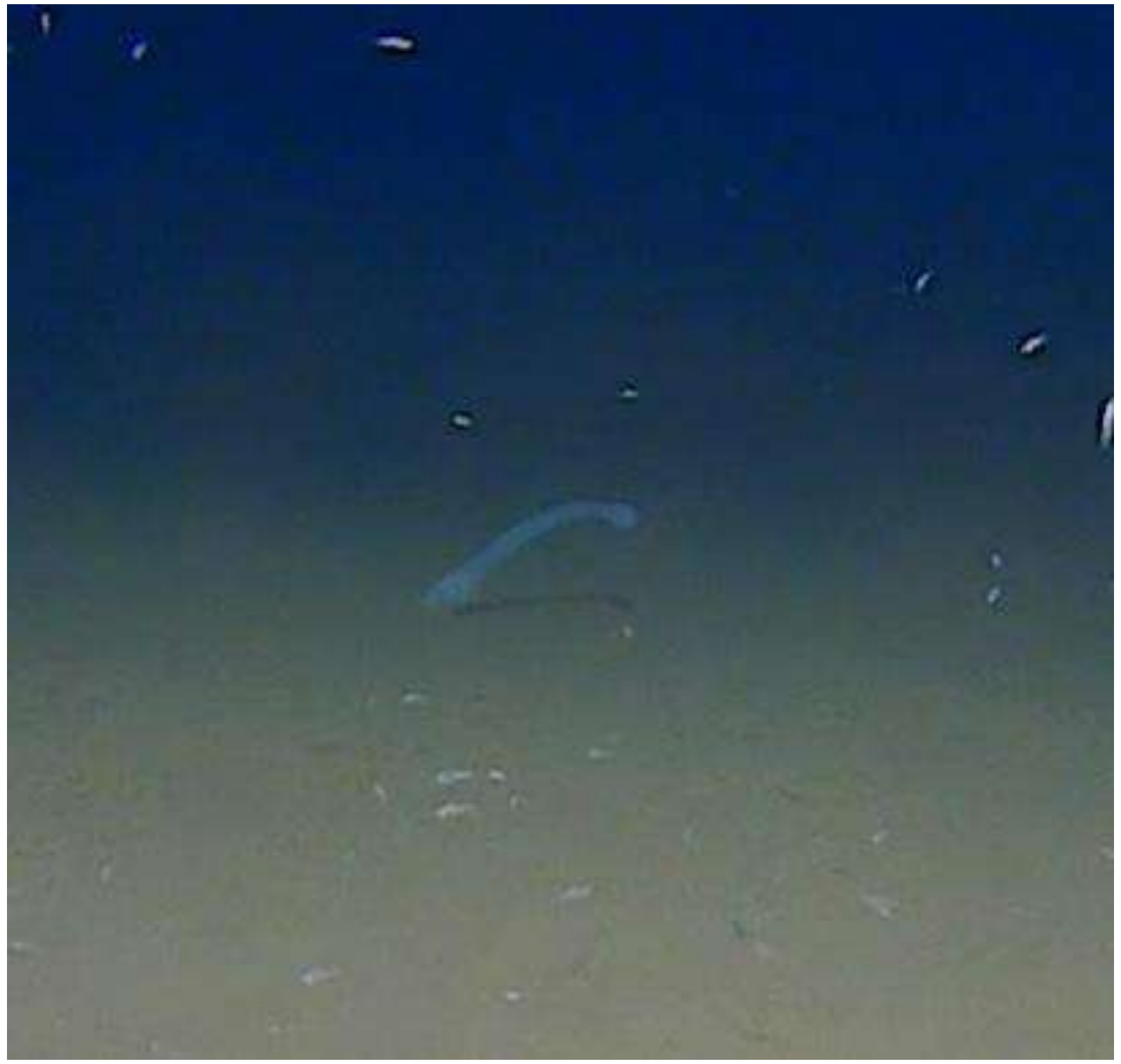

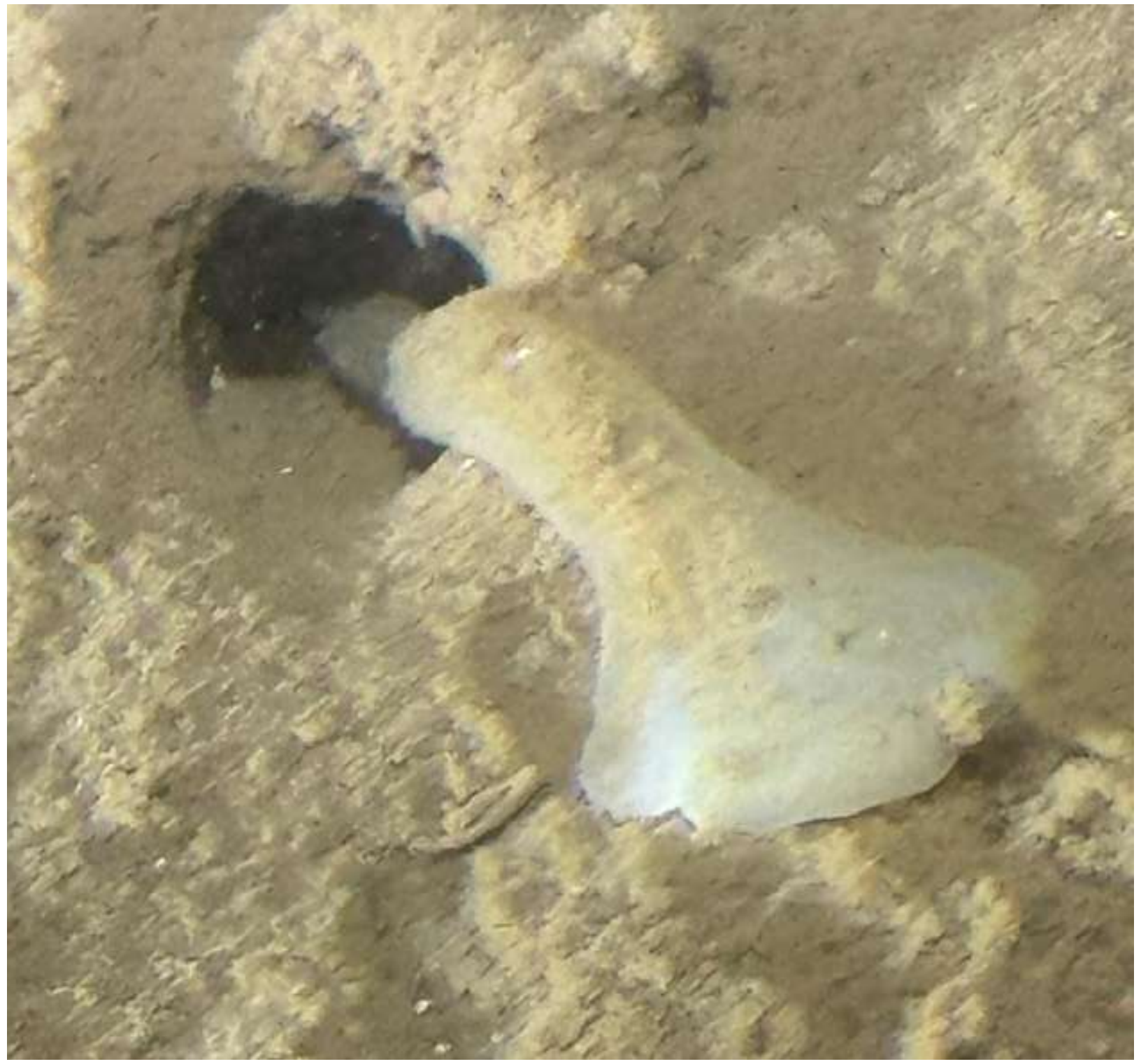

Body is translucent and long with a thickened trunk. The anterior end is flattened into a widened spoon-shape.

Behavioural Diagnostic: Observed moving across the sediment in an "inch-worm" style

Polychaeta; Echiura; Echiuroidea; Echiuroidea fam indet. 2

NC3_BK4_8000_IMG_4317

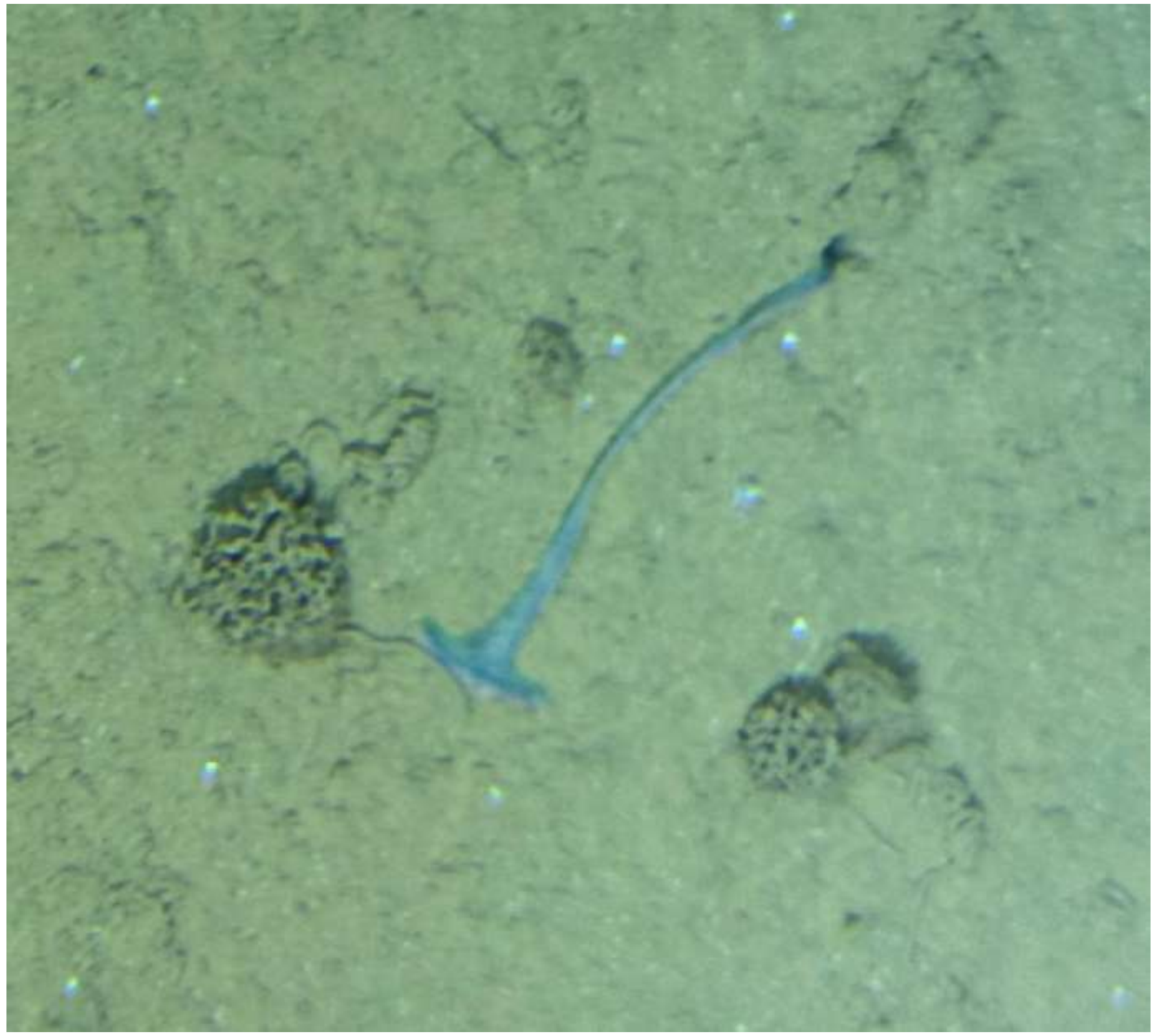

Body is long, blue and translucent. Anterior end is wide and flattened into a pickaxe-like shape.

Brett-Reminiscent of an acorn worm without the sections, however, keep as spoon worm

Polychaeta; Echiura; Echiuroidea; Echiuroidea fam. indet 3

NC1_OM2_6000_NOR_0078

NC3_CR1_4400_NOR_0204

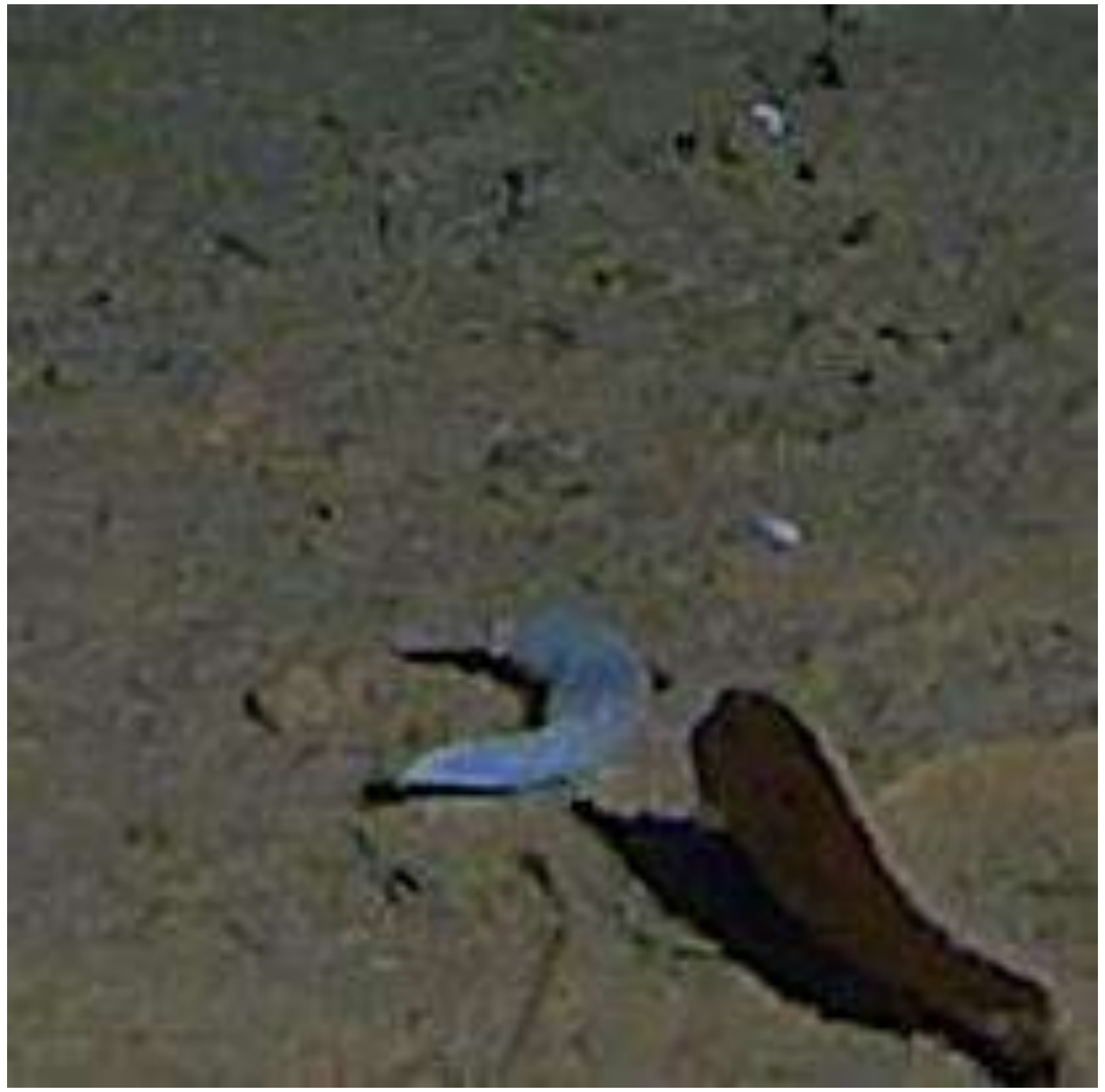

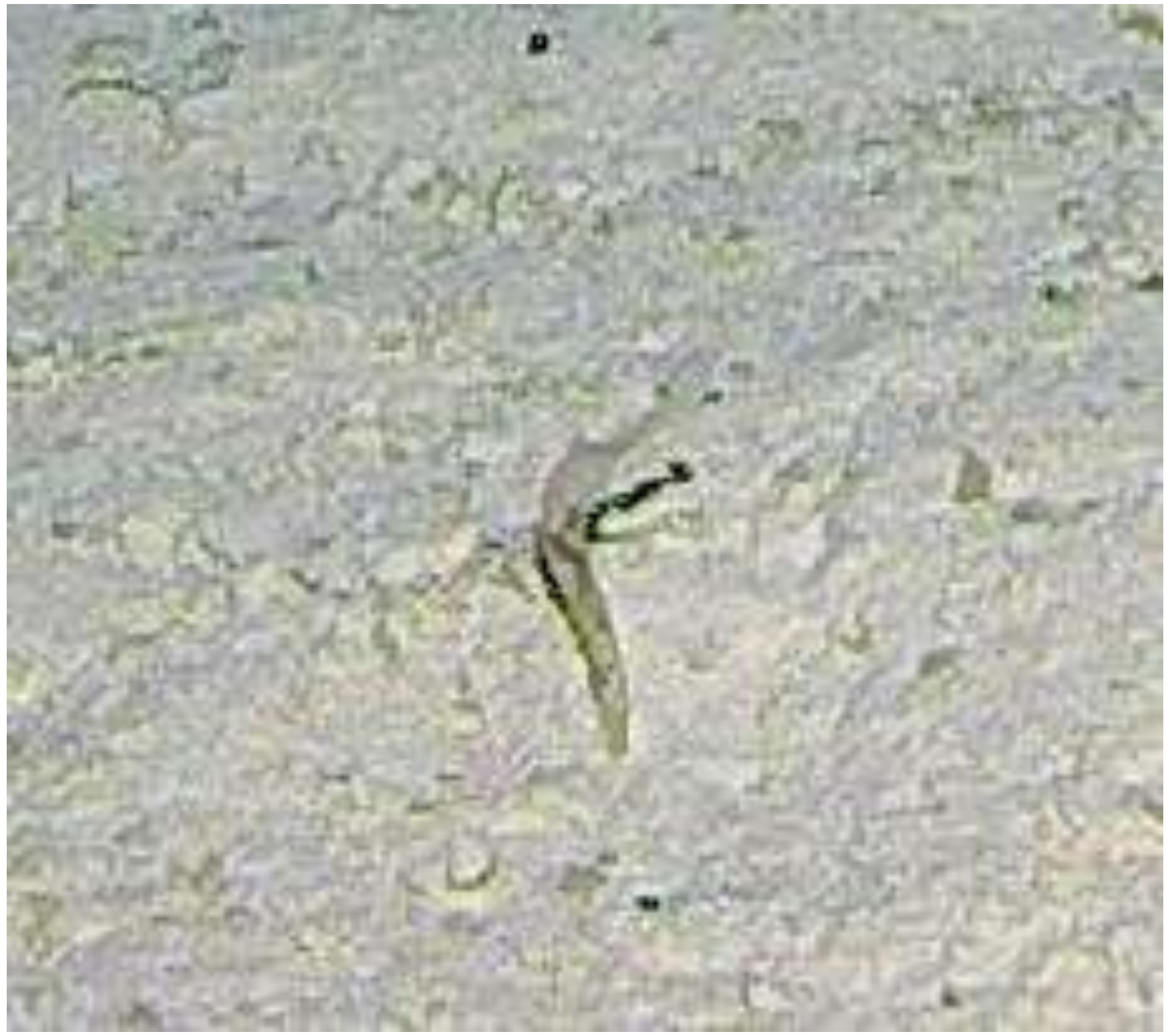

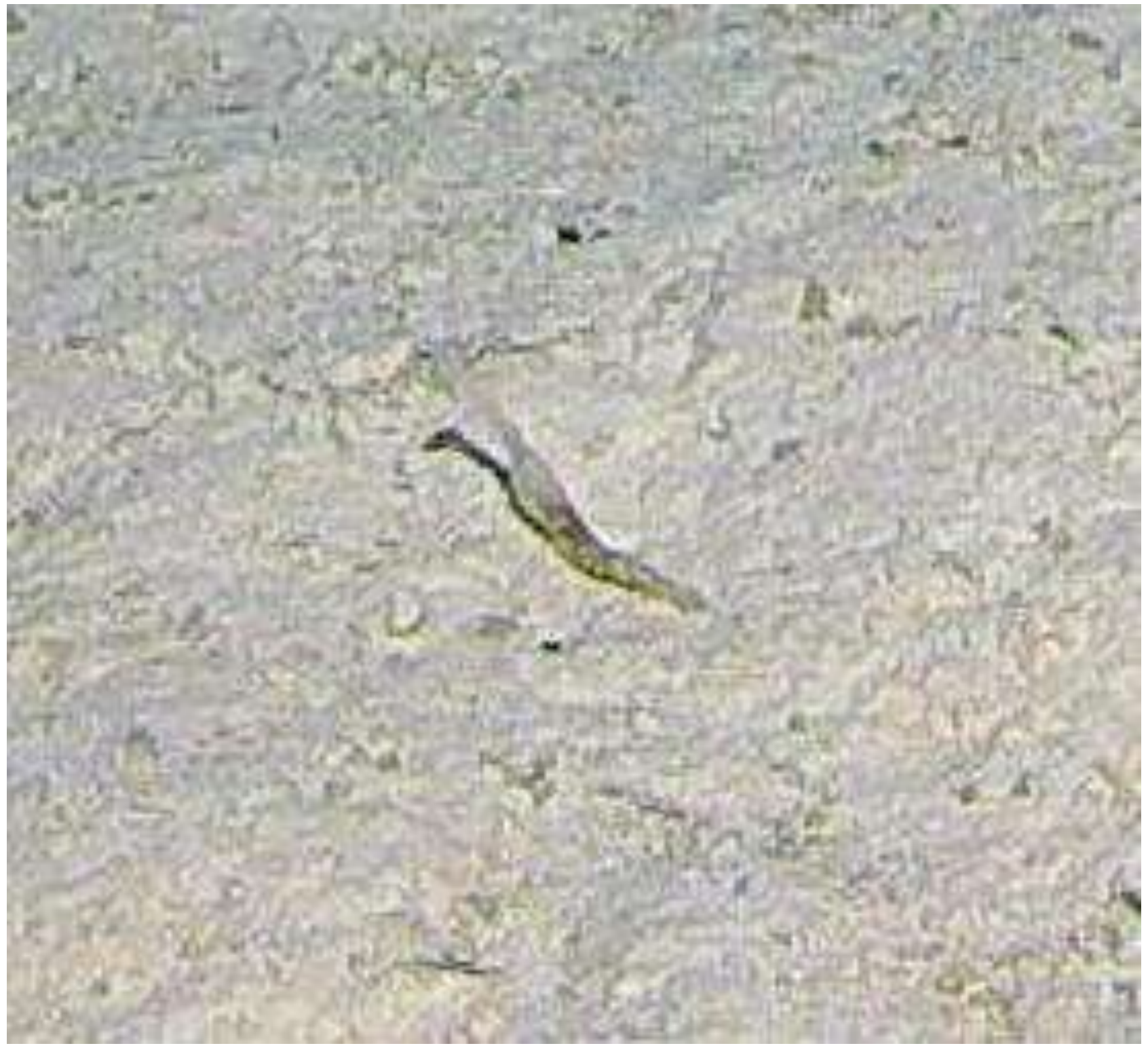

Body is small, translucent and cylindrical with tapering proboscis. ID mostly based on movement pattern/jumping

See video clip: *https://uniwa.sharepoint.com/:v:/r/teams/EXT-MUDSRC/Shared%20Documents/Cruises/2024_02_Nova%20Canton/Data/Video%20Analysis/Representative%20images/Annelida/Annelida_Polychaeta_Echiuroidea_spp_NC3_CR1_4400__01_NOR_0204.avi_0000026.mp4?csf=1&web=1&e=v2Wmne*

See video clip: *https://uniwa.sharepoint.com/:v:/r/teams/EXT-MUDSRC/Shared%20Documents/Cruises/2024_02_Nova%20Canton/Data/Video%20Analysis/2_Imagery/Representative%20images/Annelida/Annelida_Polychaeta_Echiuroidea_spp_NC1_OM2_6000_1_01_NOR_0078.avi_0000000.mp4?csf=1&web=1&e=m1TLsg*

Polychaeta; Echiura; Echiuroidea; Echiuroidea fam. indet 4

NC3_OM3_6700

NC4_BK1_5600

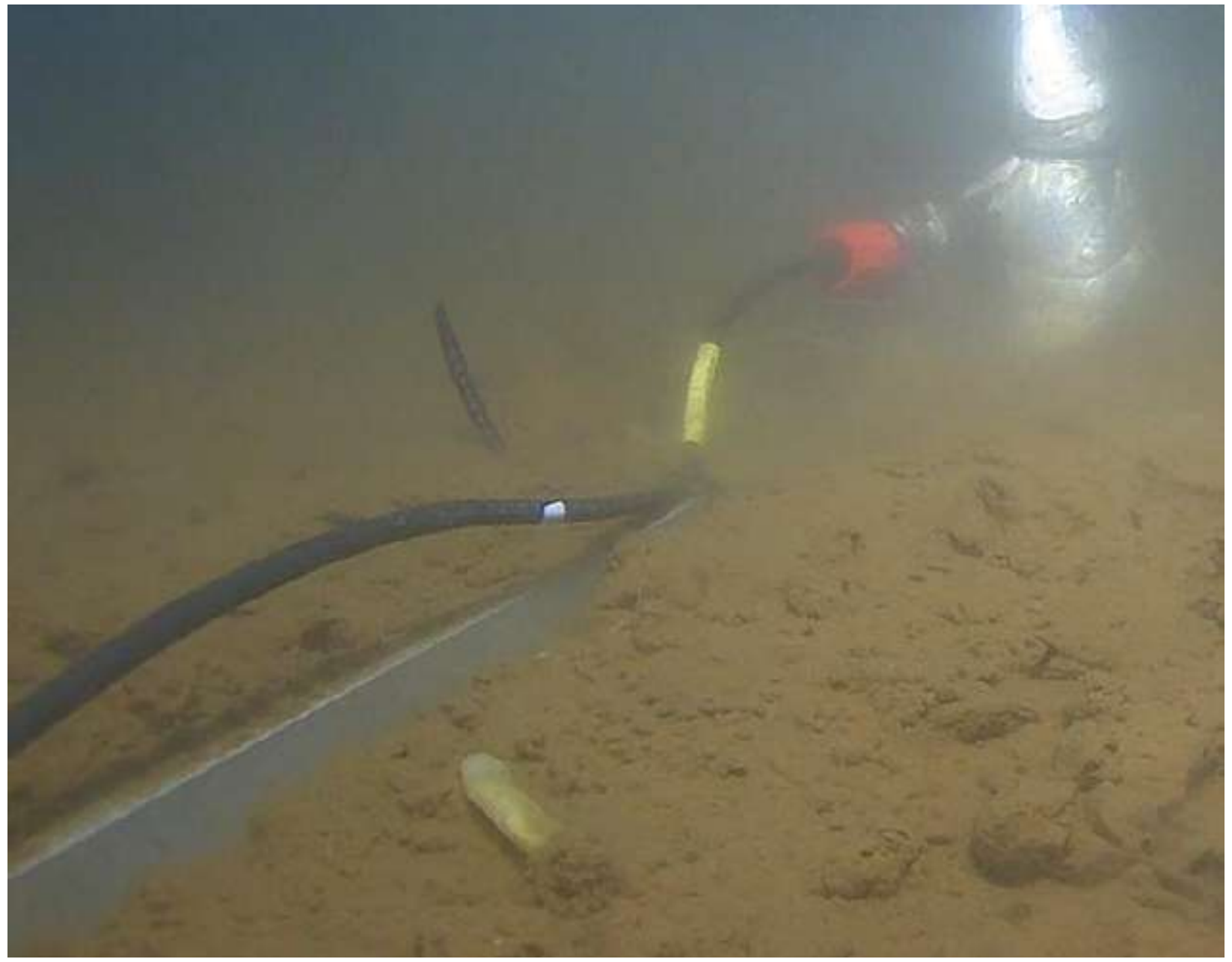

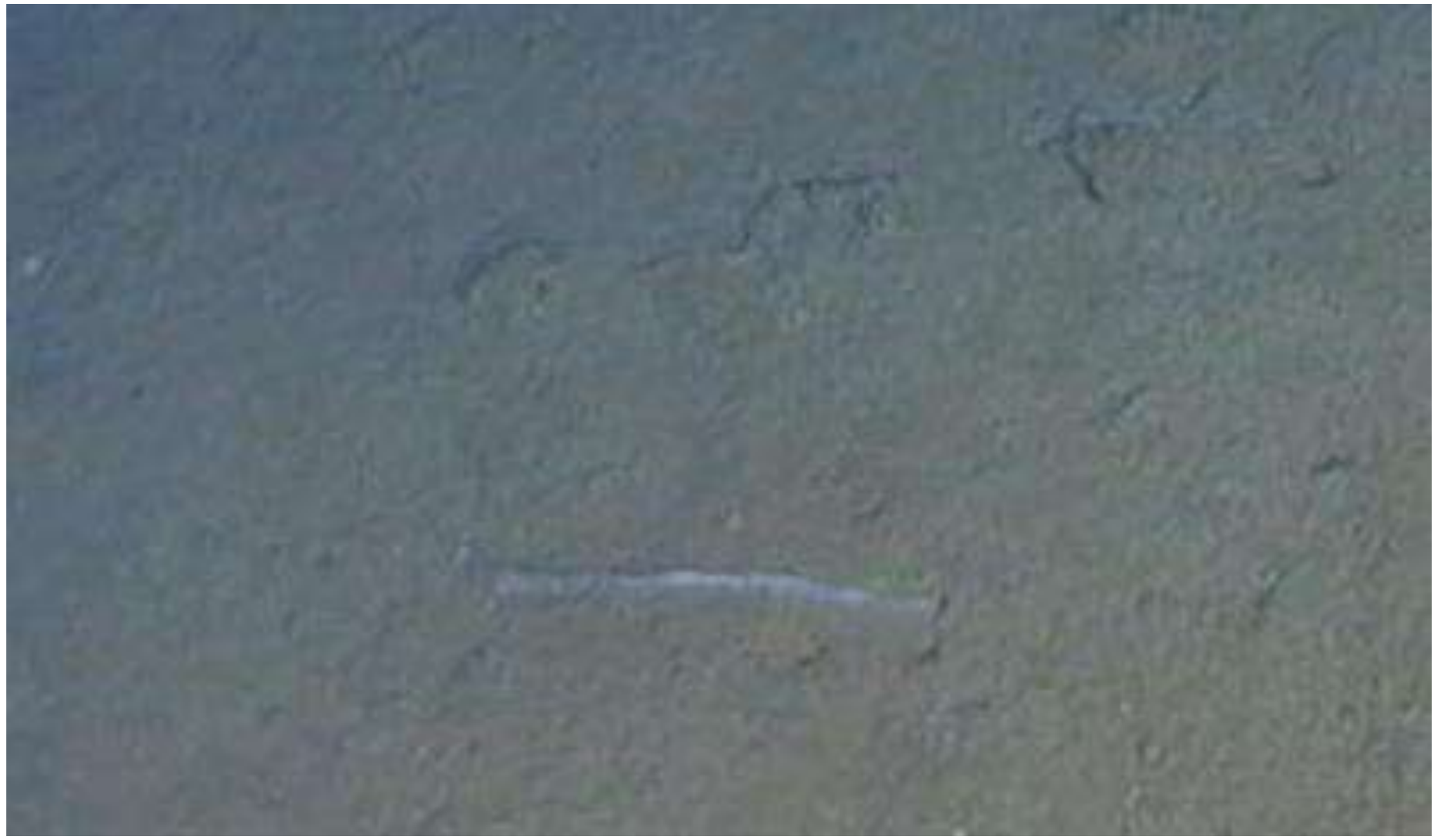

Body is white, translucent. Rouded proboscis

Polychaeta; Echiura; Echiuroidea; Echiuroidea fam indet. 5

NC2_BK3_7400_IMG_8814
NC2_BK3_7400_IMG_8816

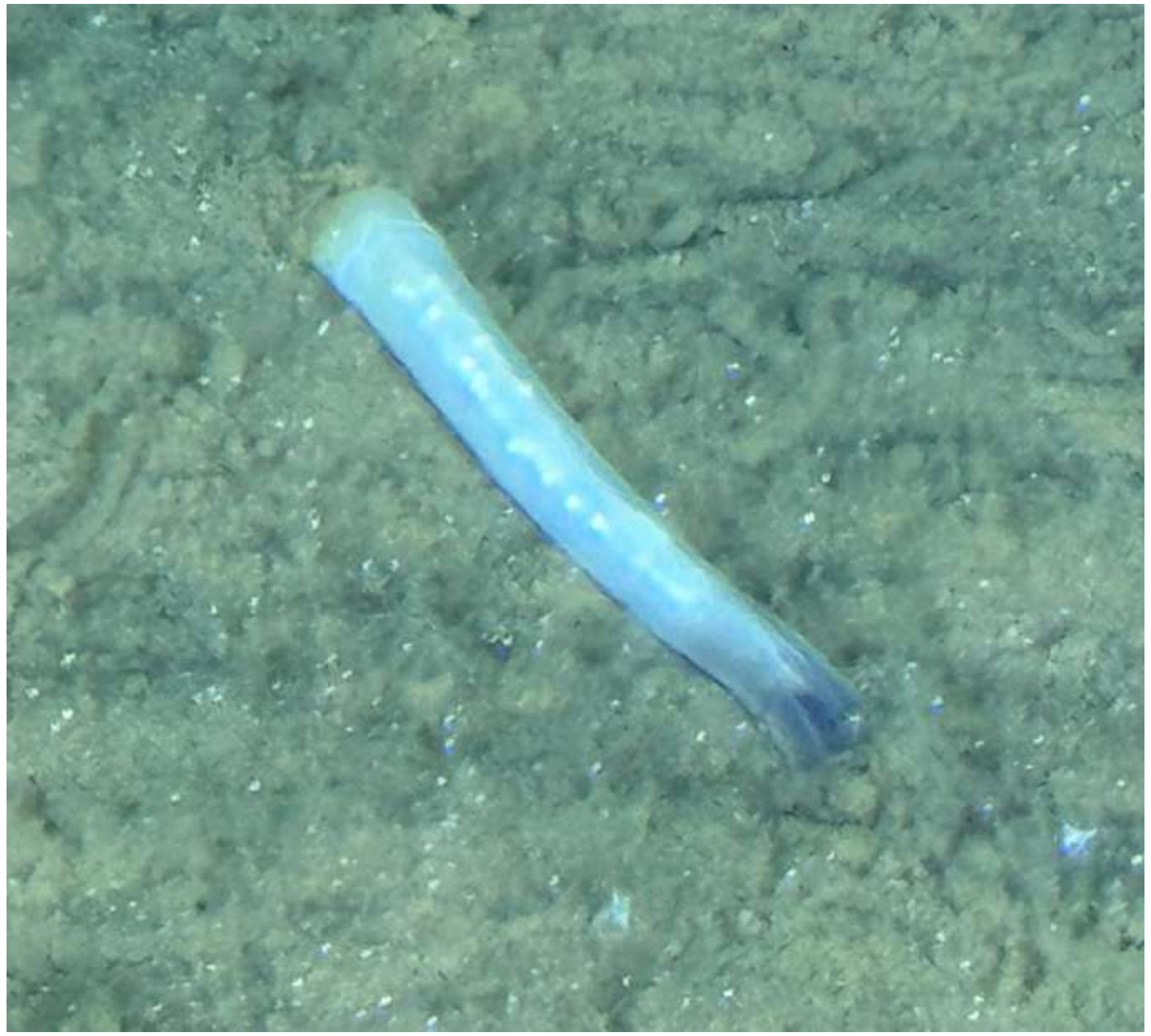

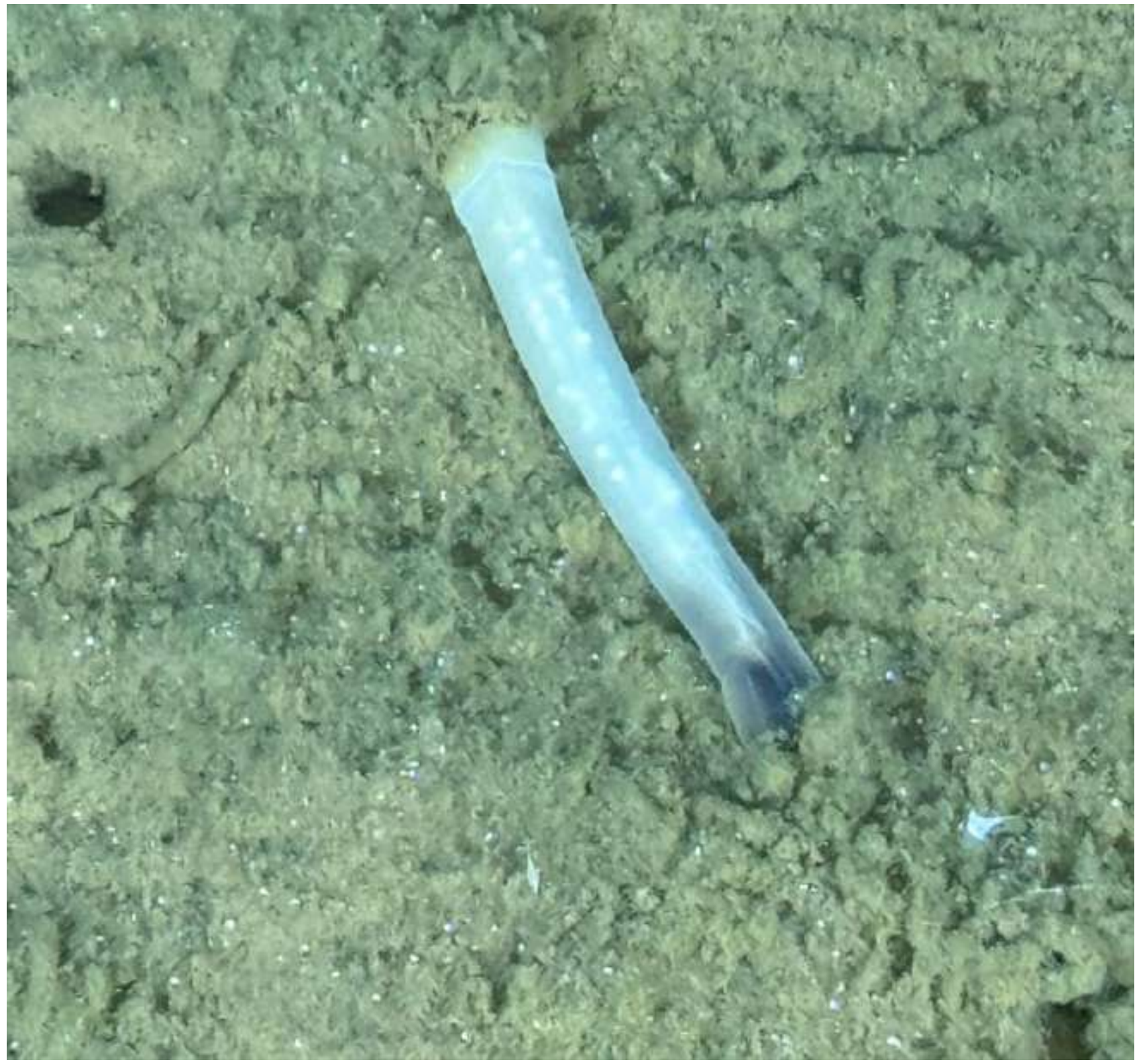

Body is translucent and gelatinous in appearance, with anterior end thick and white that tapers to a deep purple posterior end. Profile appears straight. Purple posterior end bears notable folds around terminal opening.

Polychaeta; Phyllodocida; Hesionidae; Hesionidae gen. indet. 1

NC1_BK2_6700_IMG_5225

NC1_BK2_6700_IMG_4987

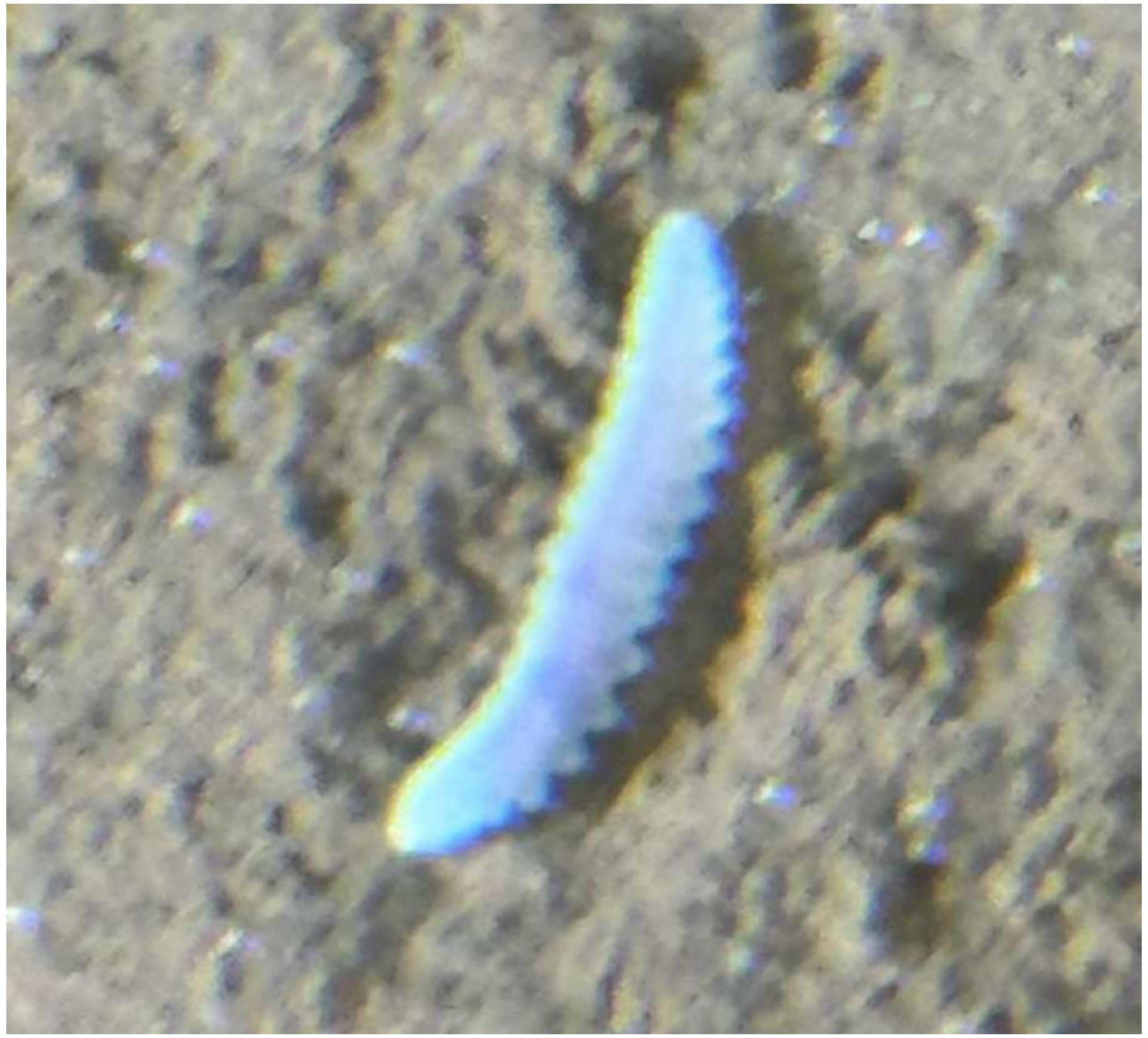

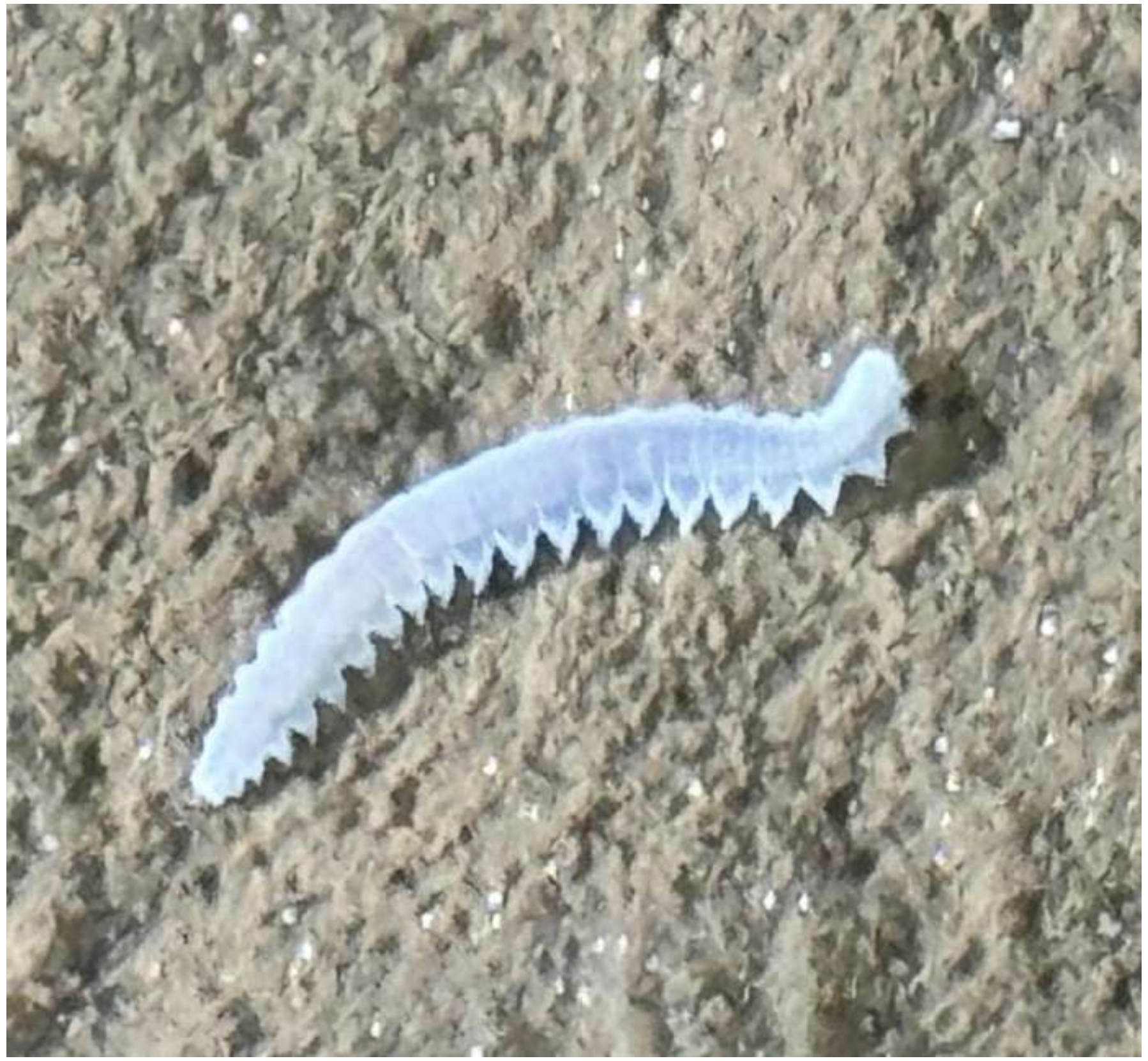

Body is white, segmented and dorso-ventrally flattened. Approximately 20 tentacular segments are triangular and line the flanks.

Polychaeta; Phyllodocida; Polynoidae; Macellicephalinae spp.

NC2_BK3_7400_NOR_0085

NC1_CR3_7000_NOR_0070

NC1_CR8_7000_NOR_0113

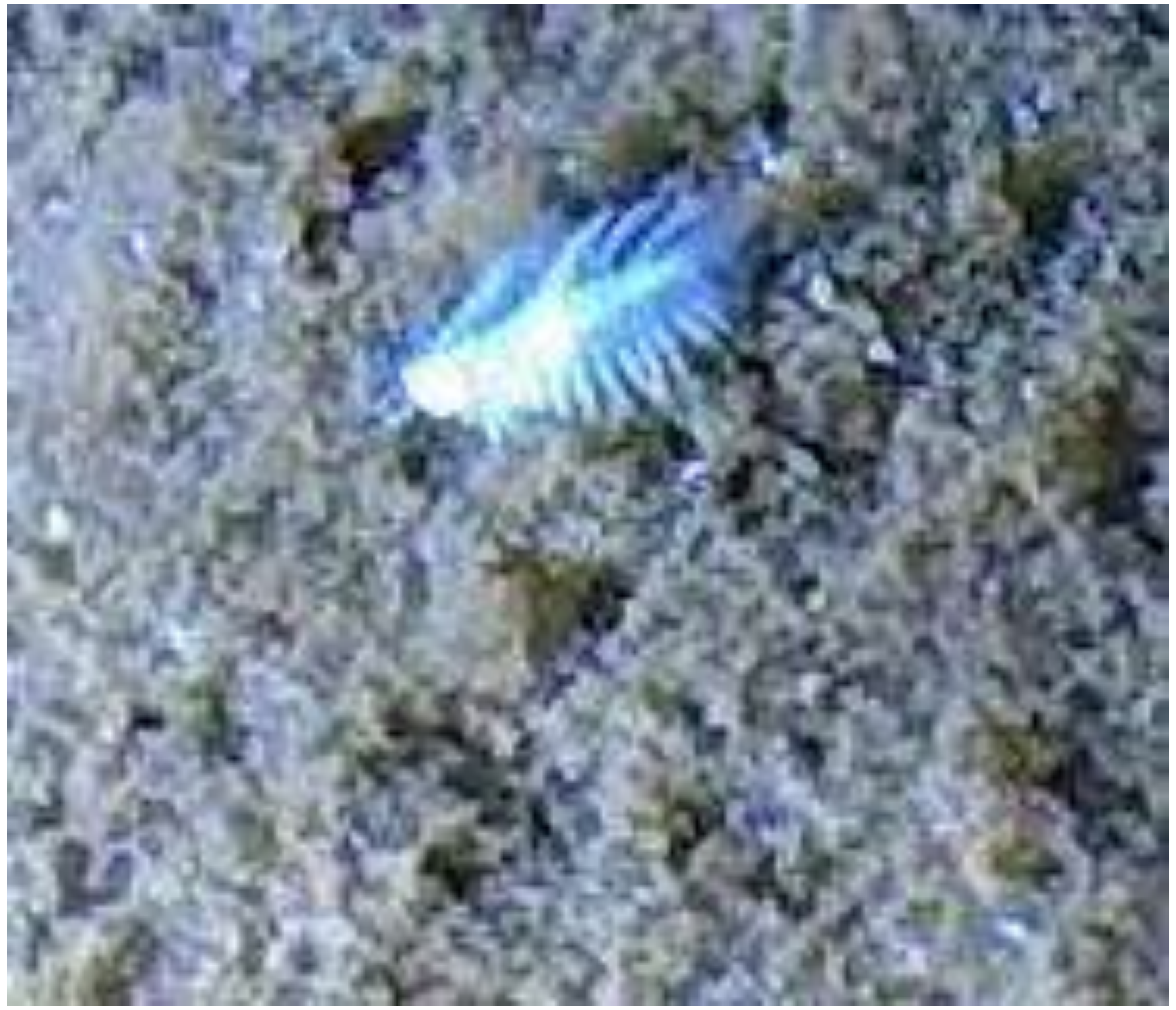

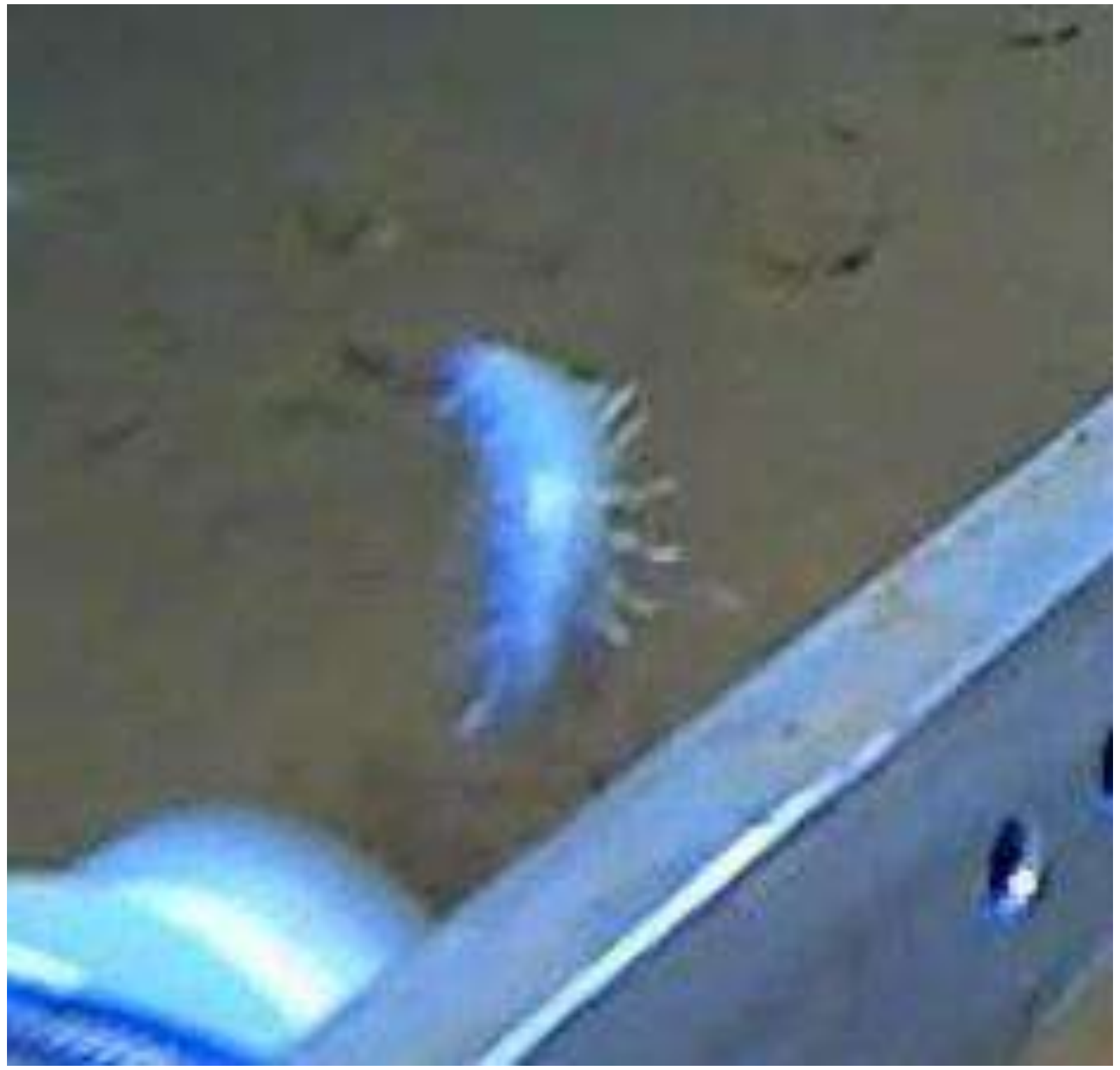

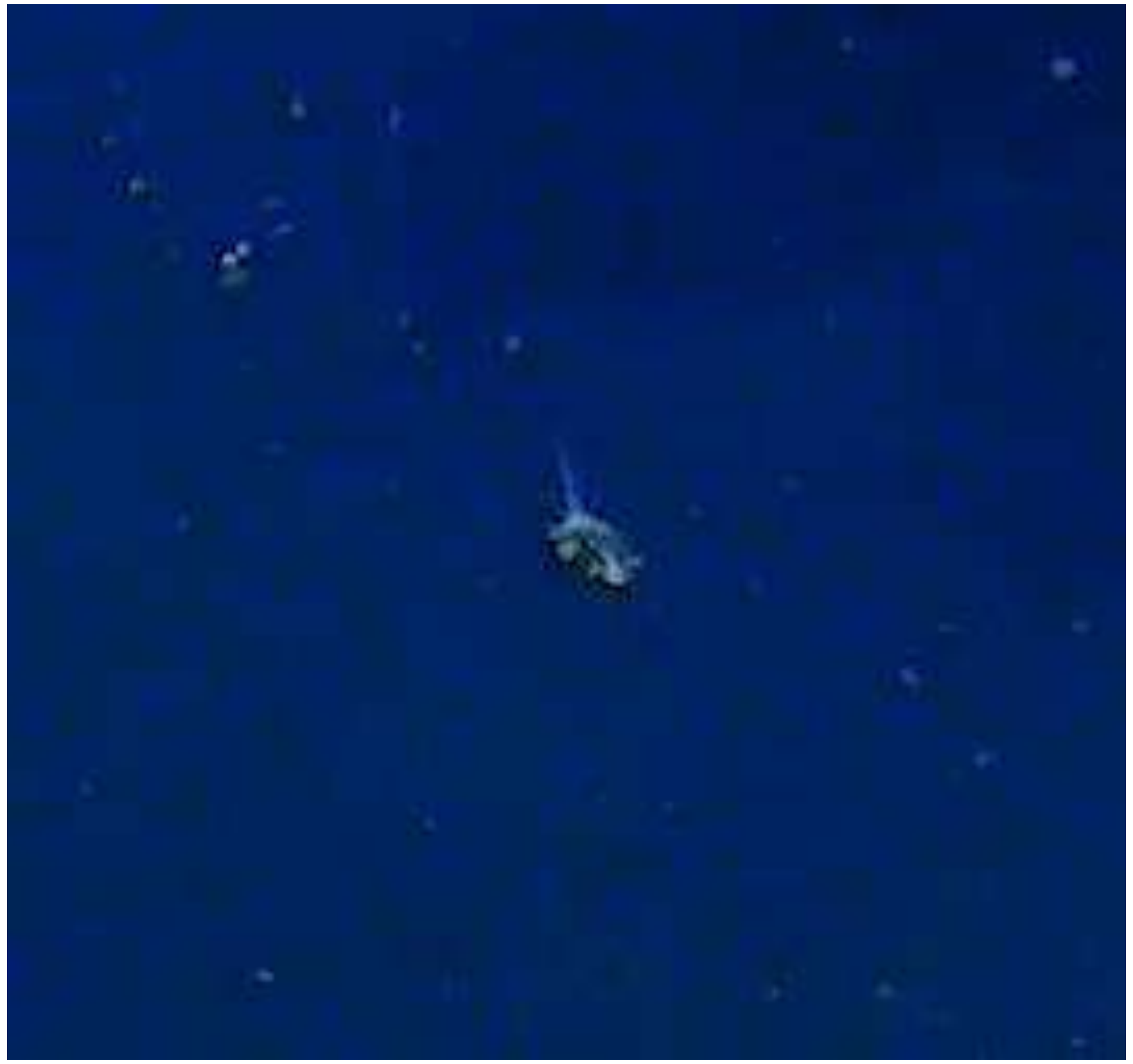

Body is whitish in colour and lined with parapodia from the anterior to the posterior. Large spine like projections extend outward from the posterior in a "fan-like" fashion.

See video clip(s): *https://uniwa.sharepoint.com/:v:/r/teams/EXT-MUDSRC/Shared%20Documents/Cruises/2024_02_Nova%20Canton/Data/Video%20Analysis/Representative%20images/Annelida/Annelida_Polychaeta_Phyllodocida_Polynoidae_sp1_NC1_CR8_7000_1_01_NOR_0113.avi_0000926.mp4?csf=1&web=1&e=xqeazT*

Polychaeta; Phyllodocida; Polynoidae; Macellicephalinae gen. indet. 1

NC2_BK3_7400_IMG_8559

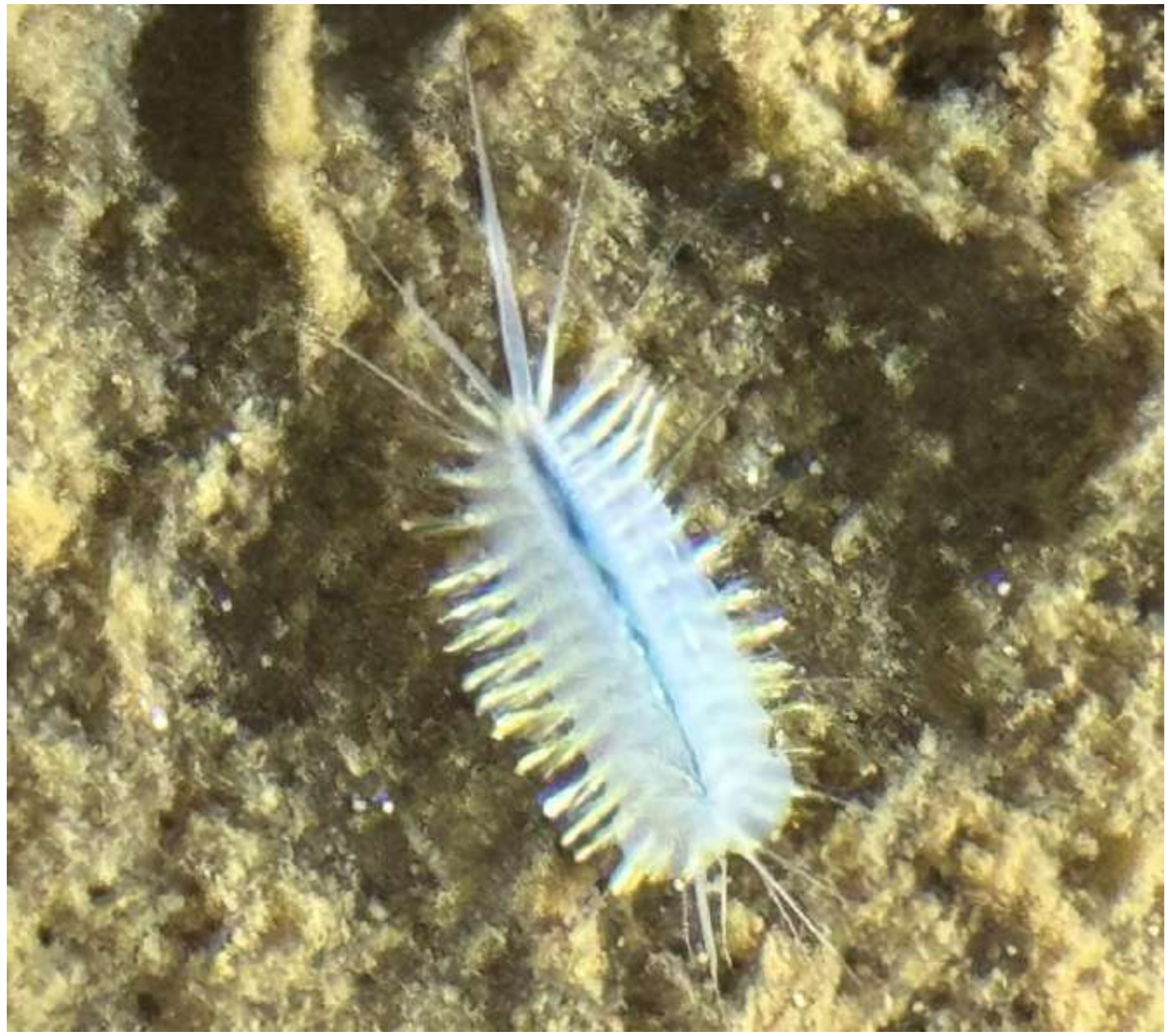

Body is whitish in colour with a blue dorsal stripe and lined with parapodia from the anterior to the posterior . Large spine like projections extend outward from the posterior in a "fan-like" fashion.

Polychaeta; Sabellida; Sabellidae; Sabellida fam. indet.1

NC2_BK2_3800_IMG_8046

NC2_BK2_3800_IMG_8055

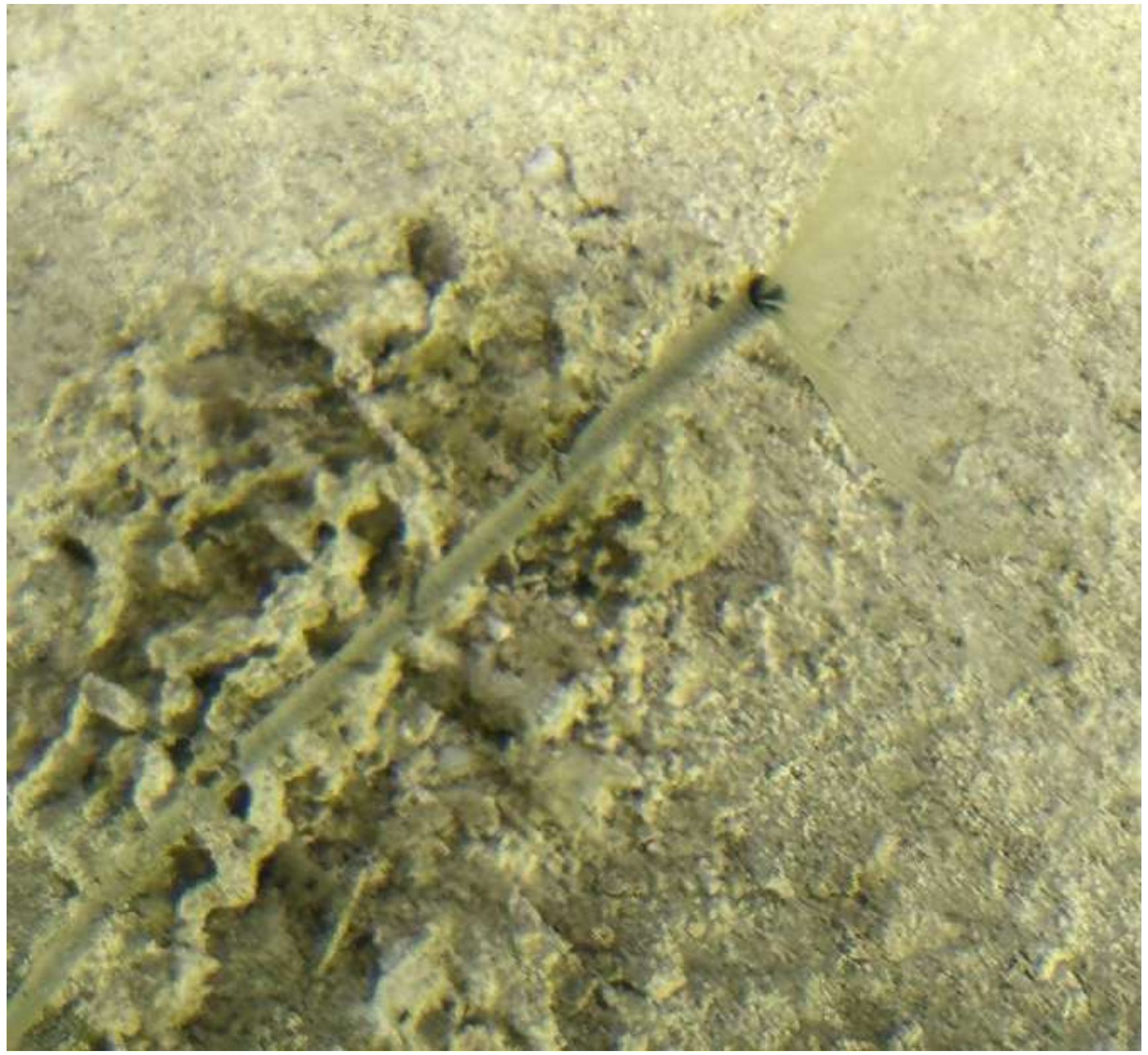

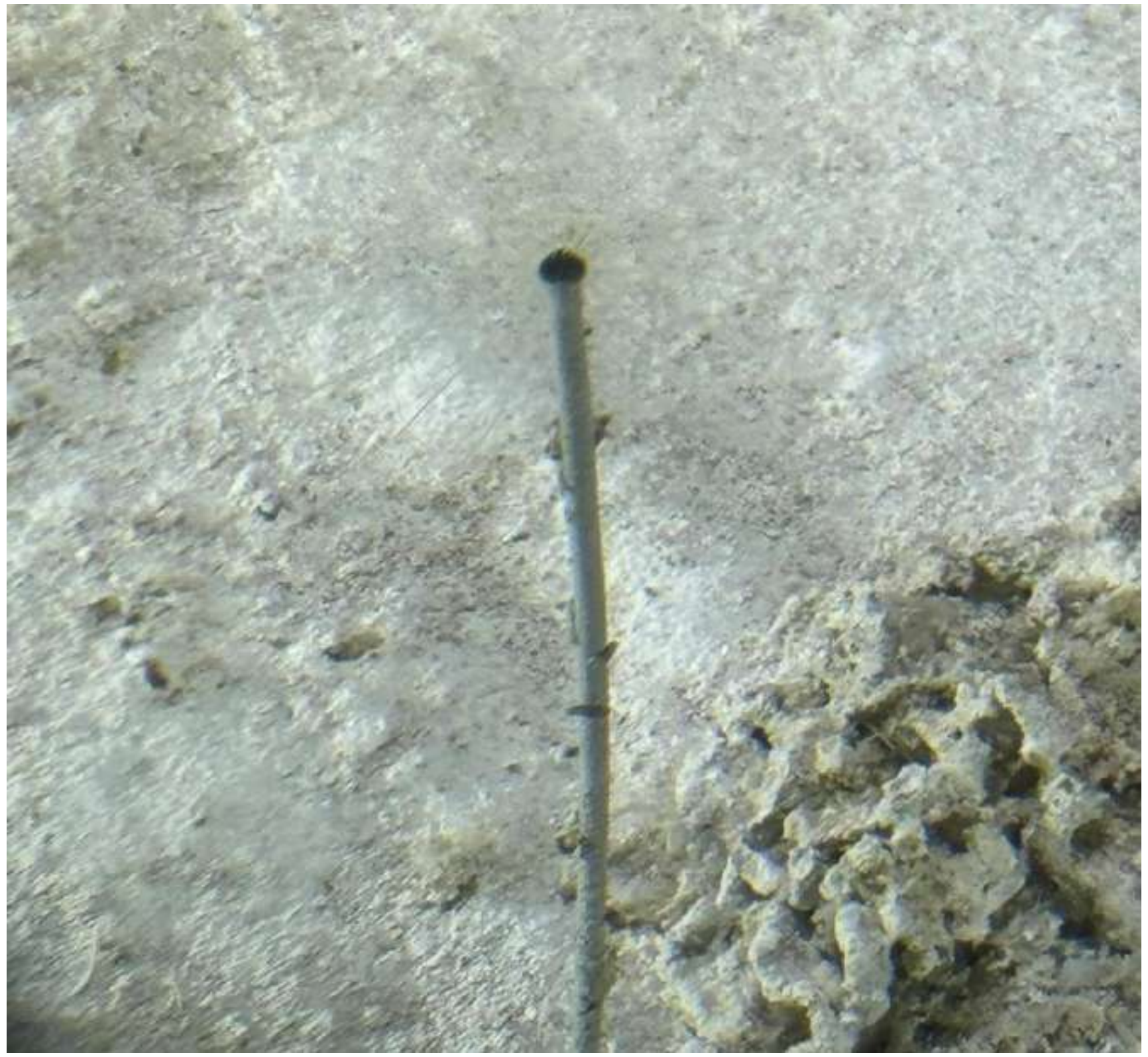

Body is encased within a rigid, vertically oriented tube. Fan-like filter feeding mouthparts are translucent are radially arranged.

Polychaeta; Sabellida; Sabellidae; Sabellida/Terebellida spp.

NC2_BK2_3800_IMG_7877

NC2_BK1_3200_IMG_6962

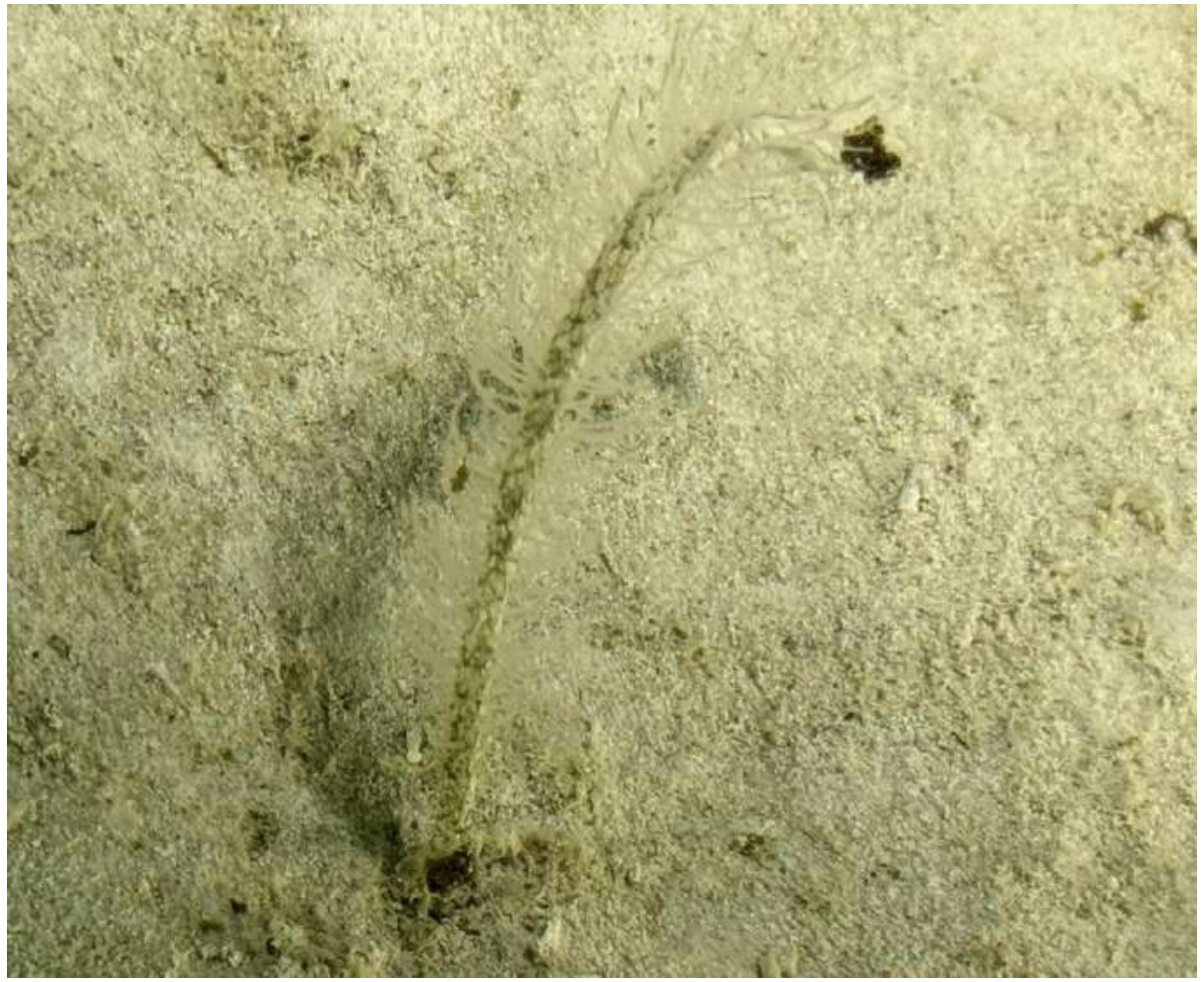

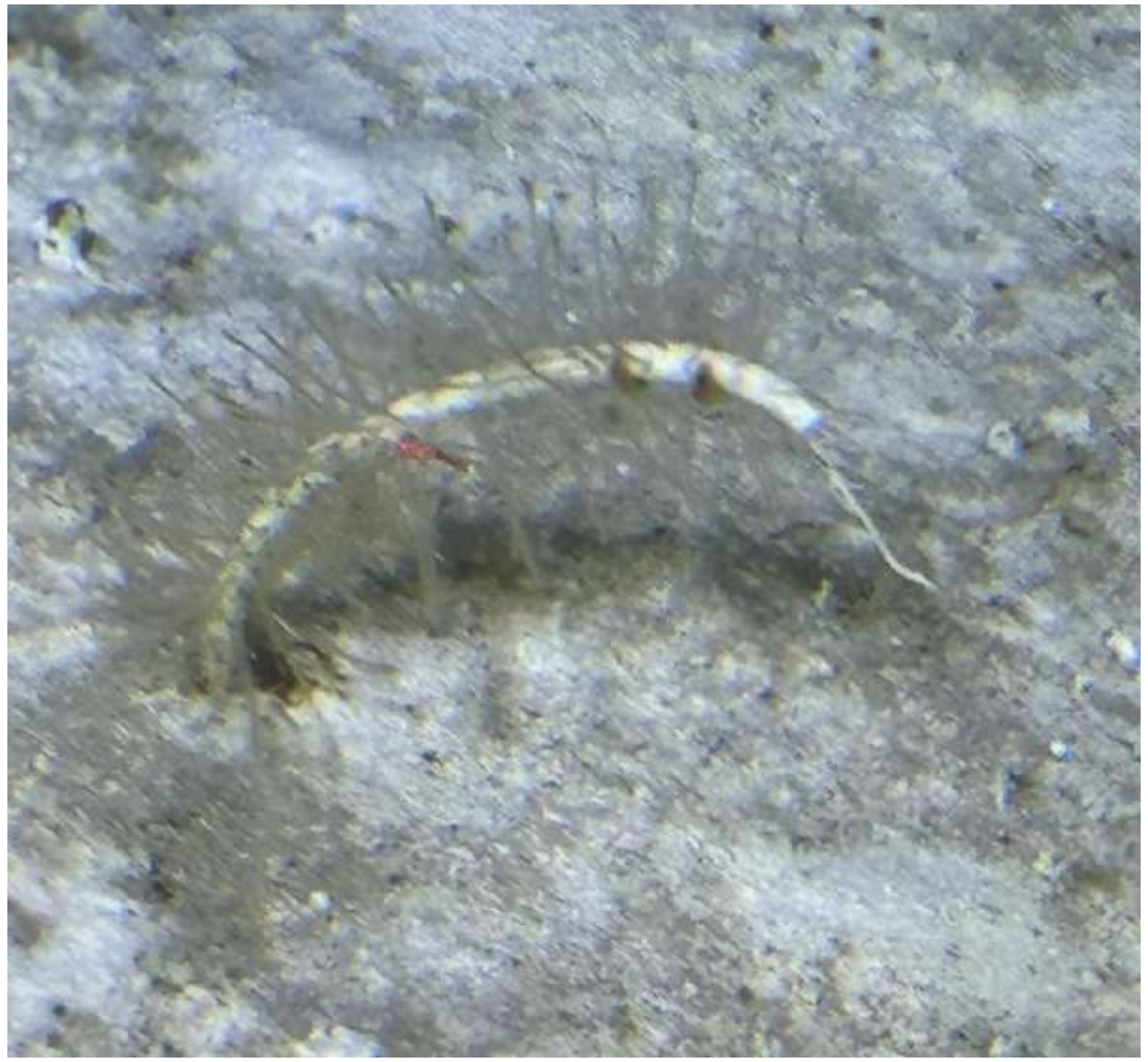

Body is encased within a rigid, curved tube. Observed covered in hydrozoans.

Polychaeta; Eunicida; Onuphidae; Onuphidae gen. indet. 1

NC1_BK2_6700_IMG_5219

NC1_BK2_6700_IMG_5051

NC1_BK2_6700_IMG_5047

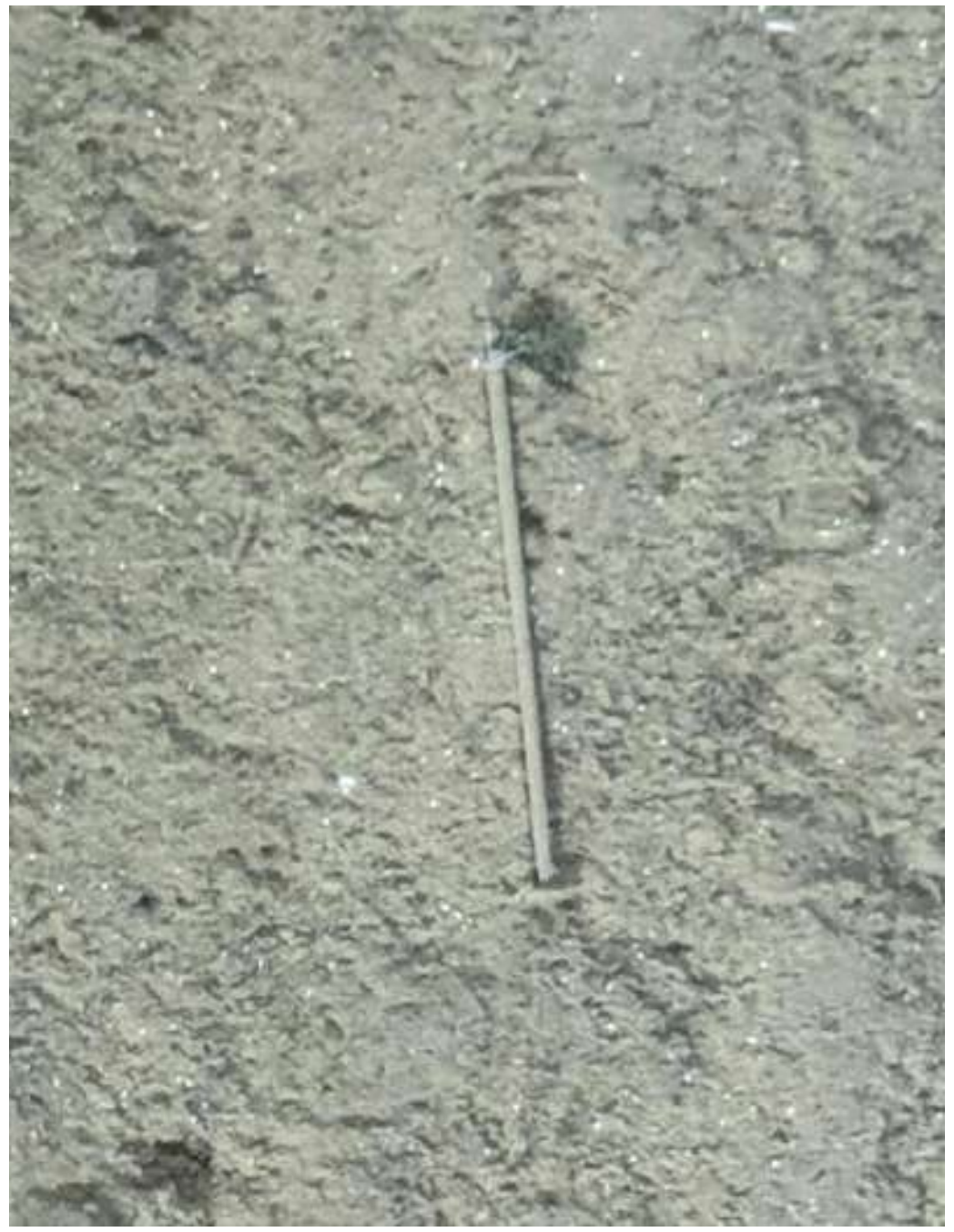

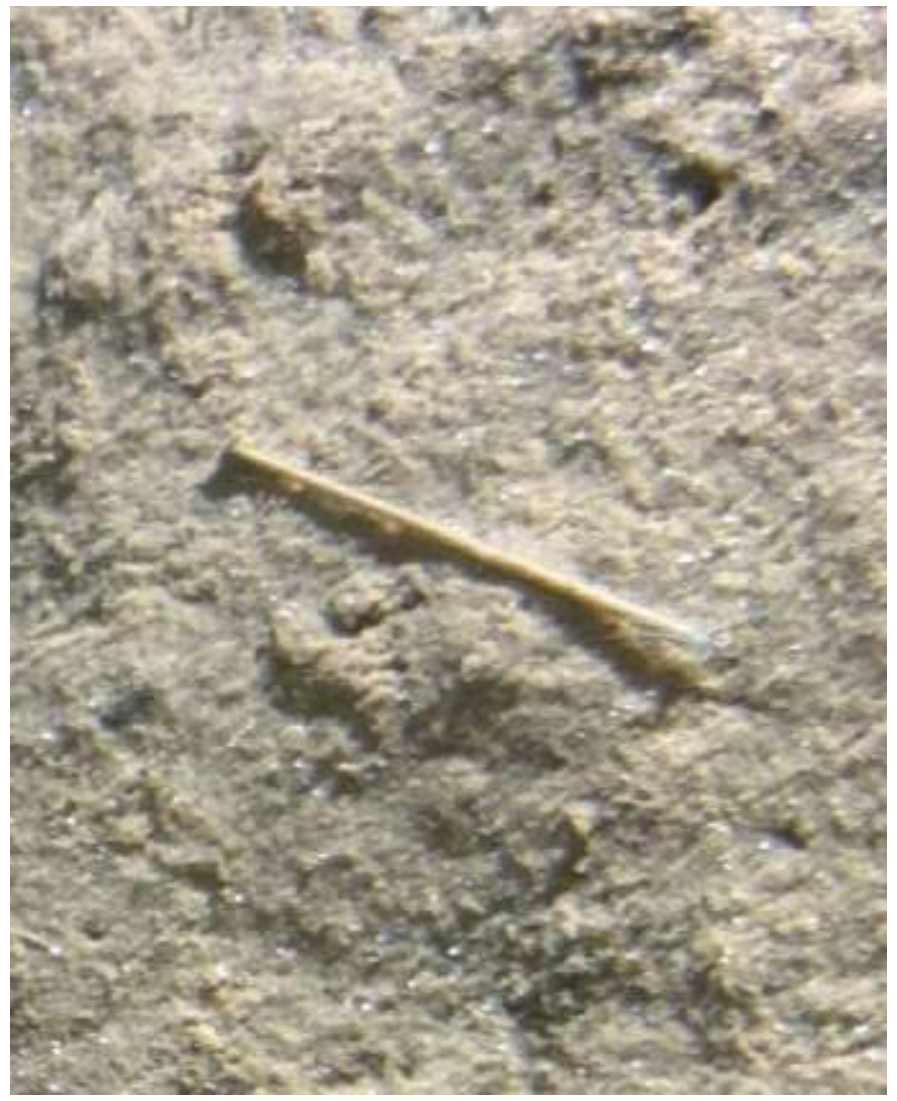

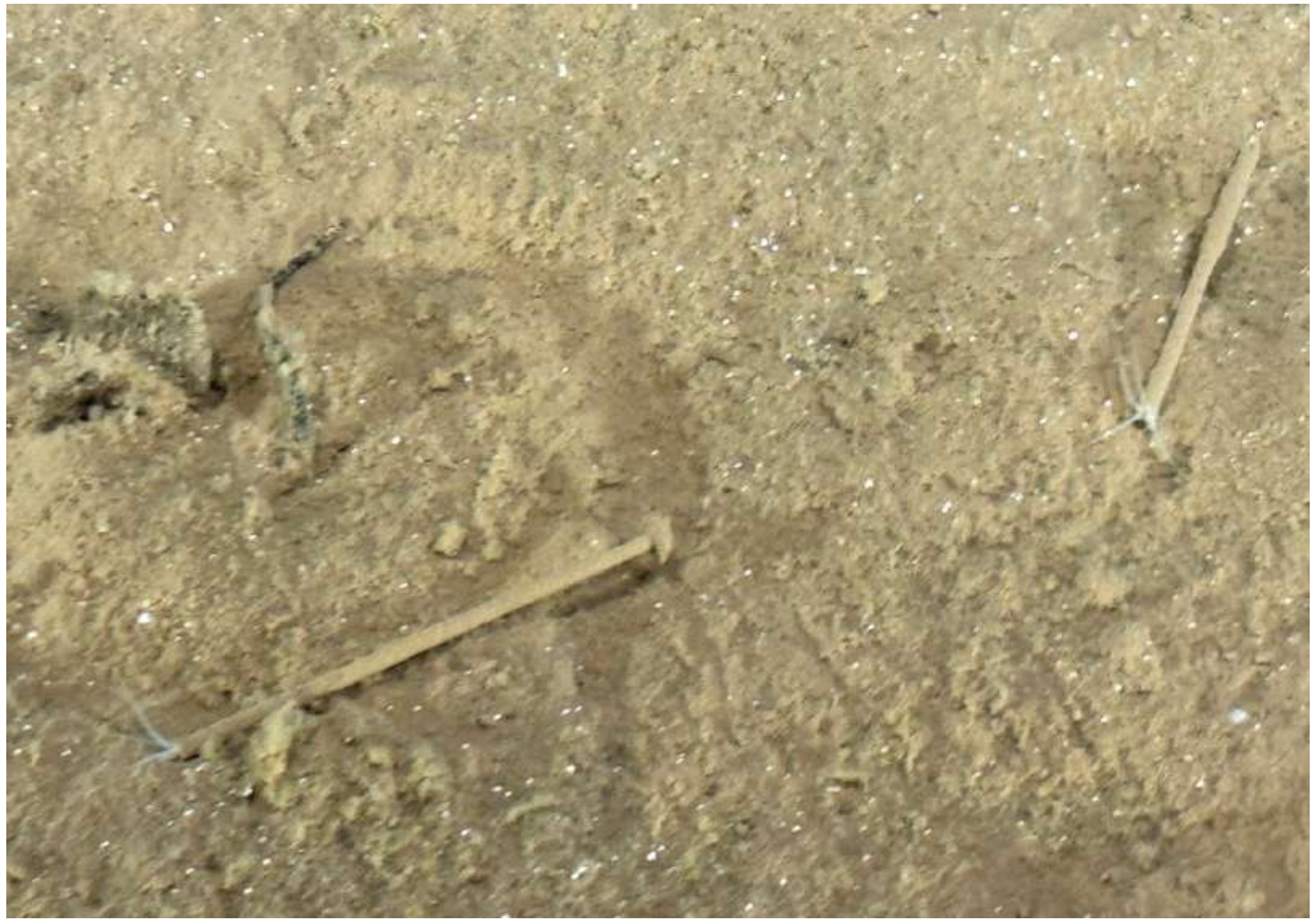

Body is encased within a rigid, curved tube.

Polychaeta; Phyllodocida; Polynoidae; Admetellinae gen. indet.

NC1_BK2_6700_IMG_4908

NC1_BK2_6700_IMG_4907

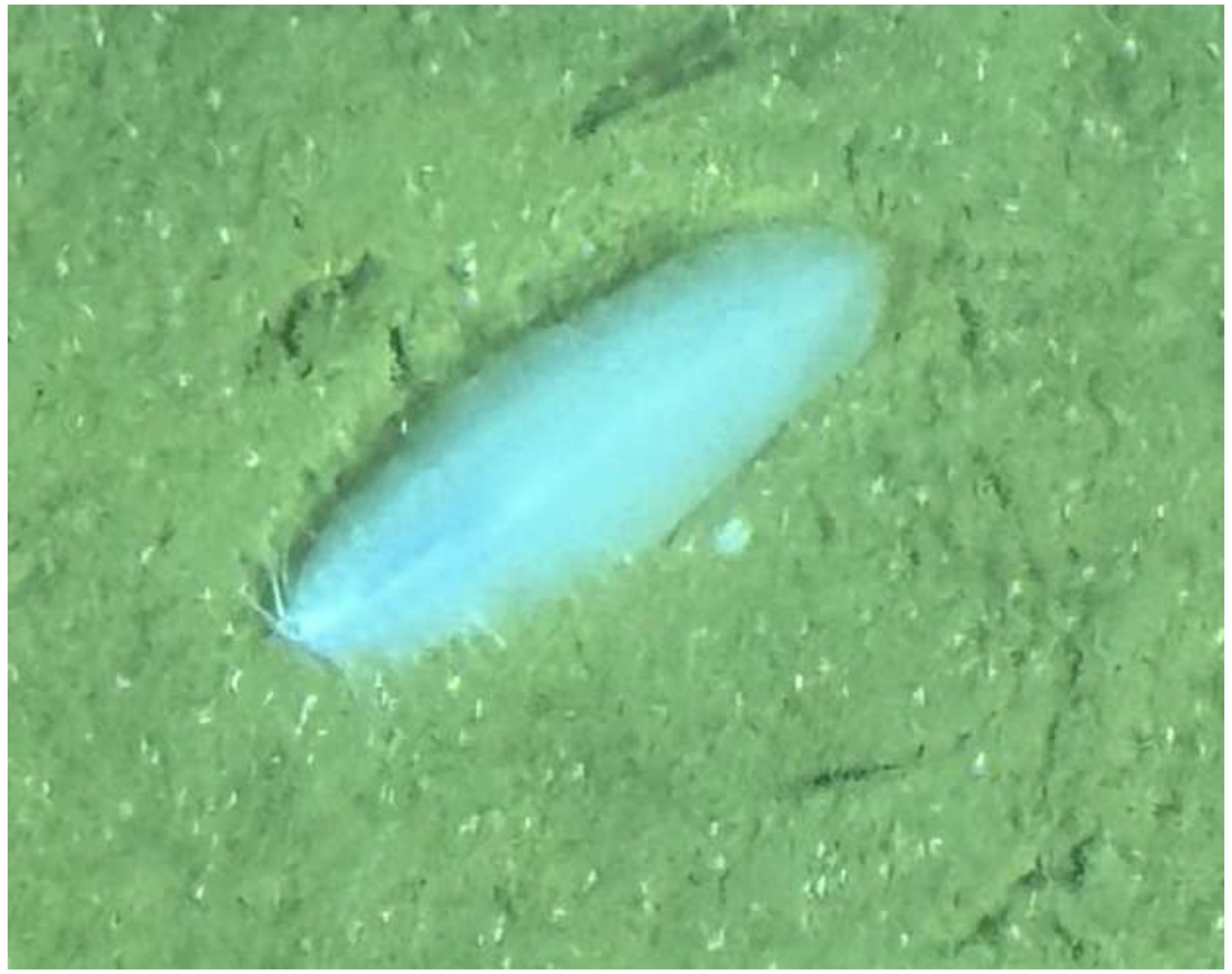

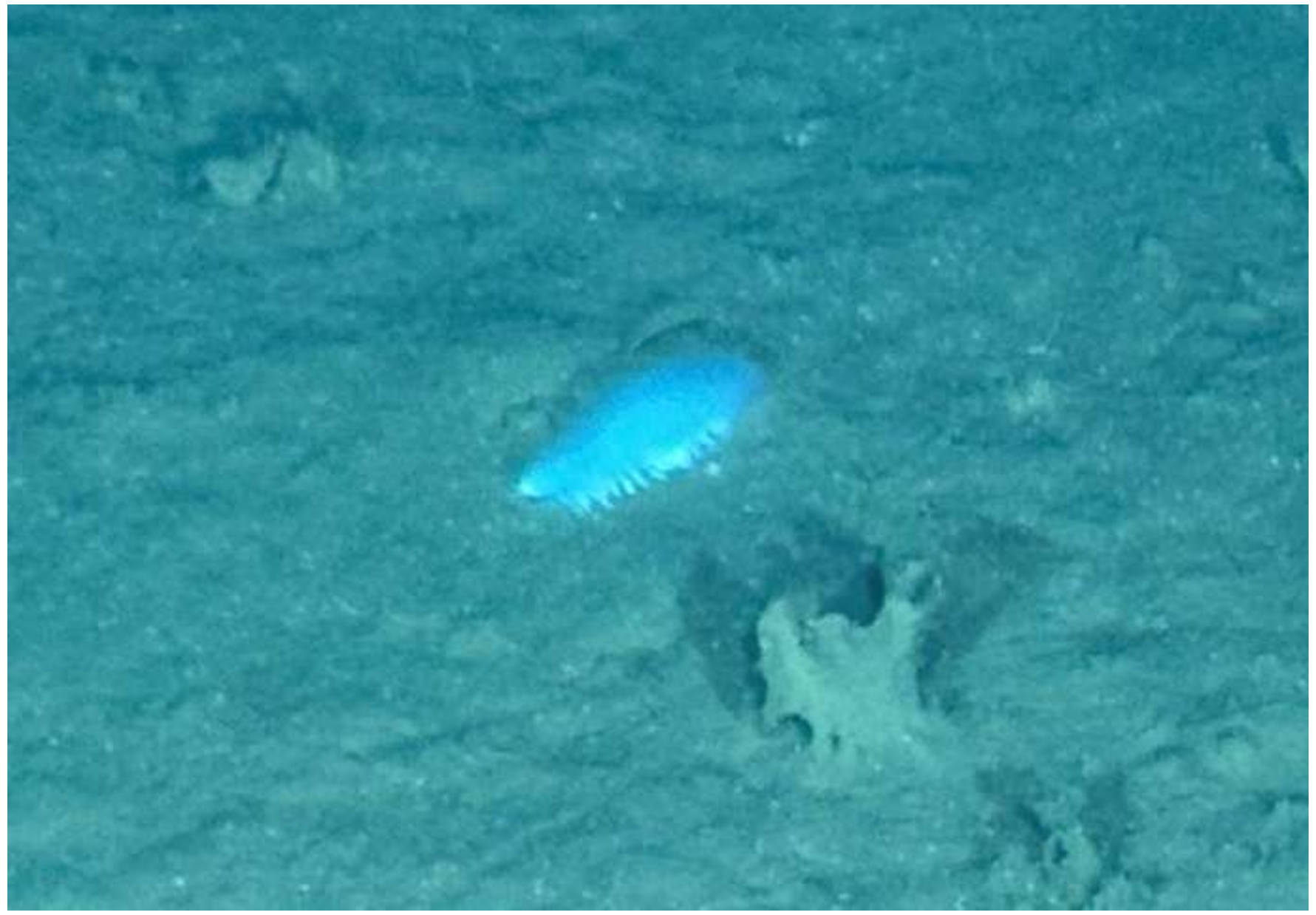

.

Polychaeta; Terebellida; Acrocirridae; Acrocirridae spp.

NC3_CR1_4400_NOR_0204
NC4_BK3_6900_NOR_0061
NC4_OM5_6900_NOR_0031

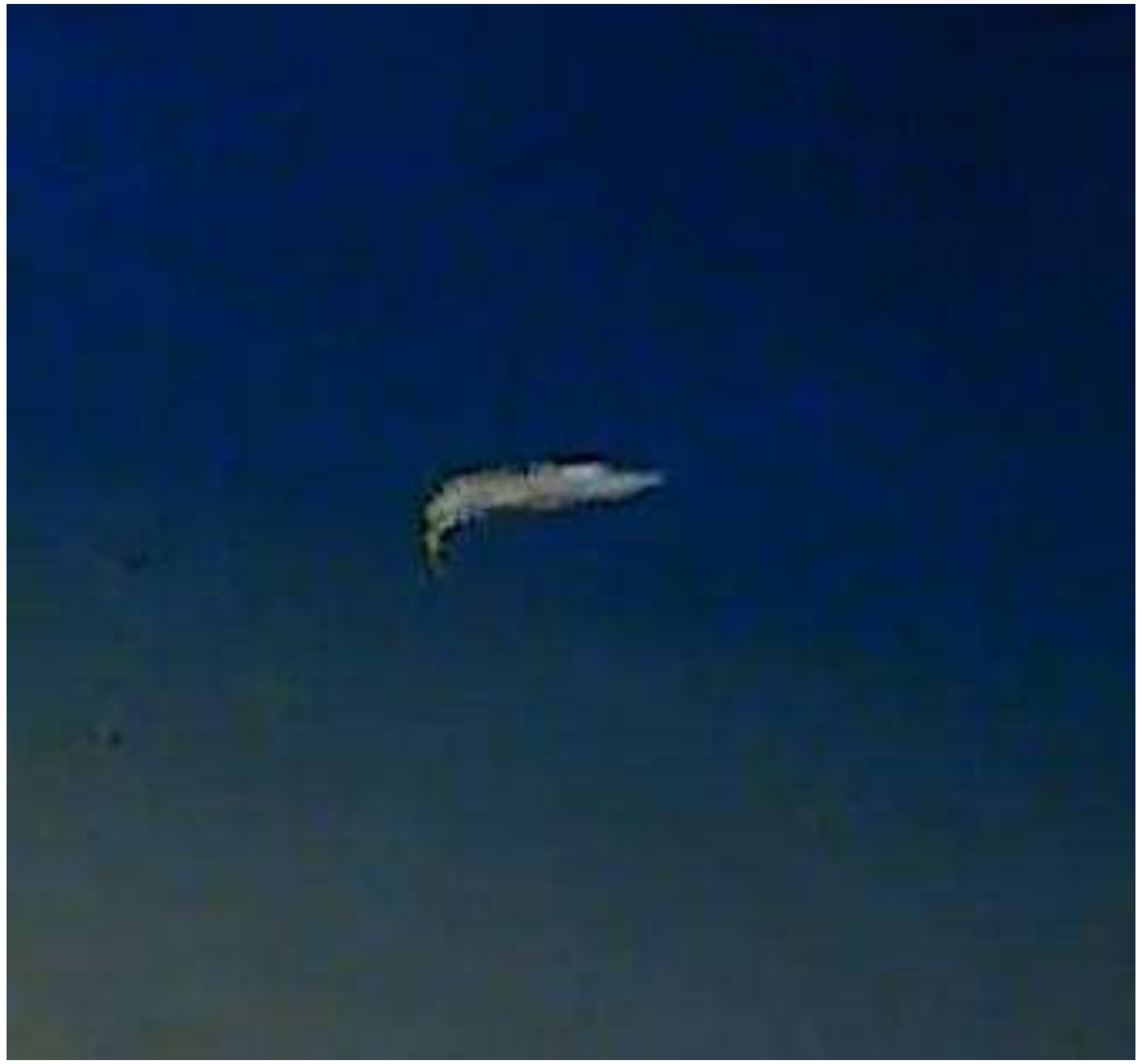

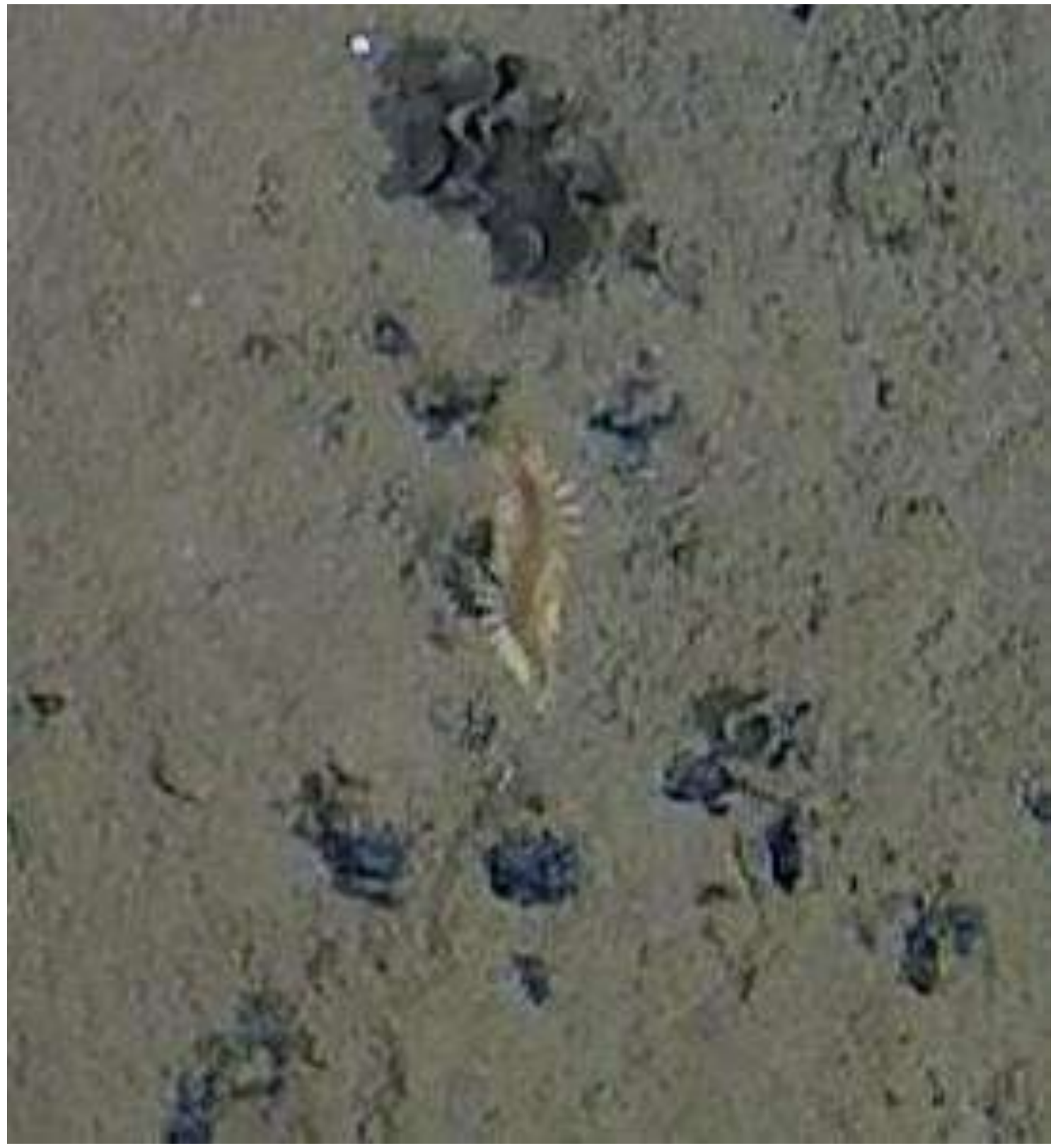

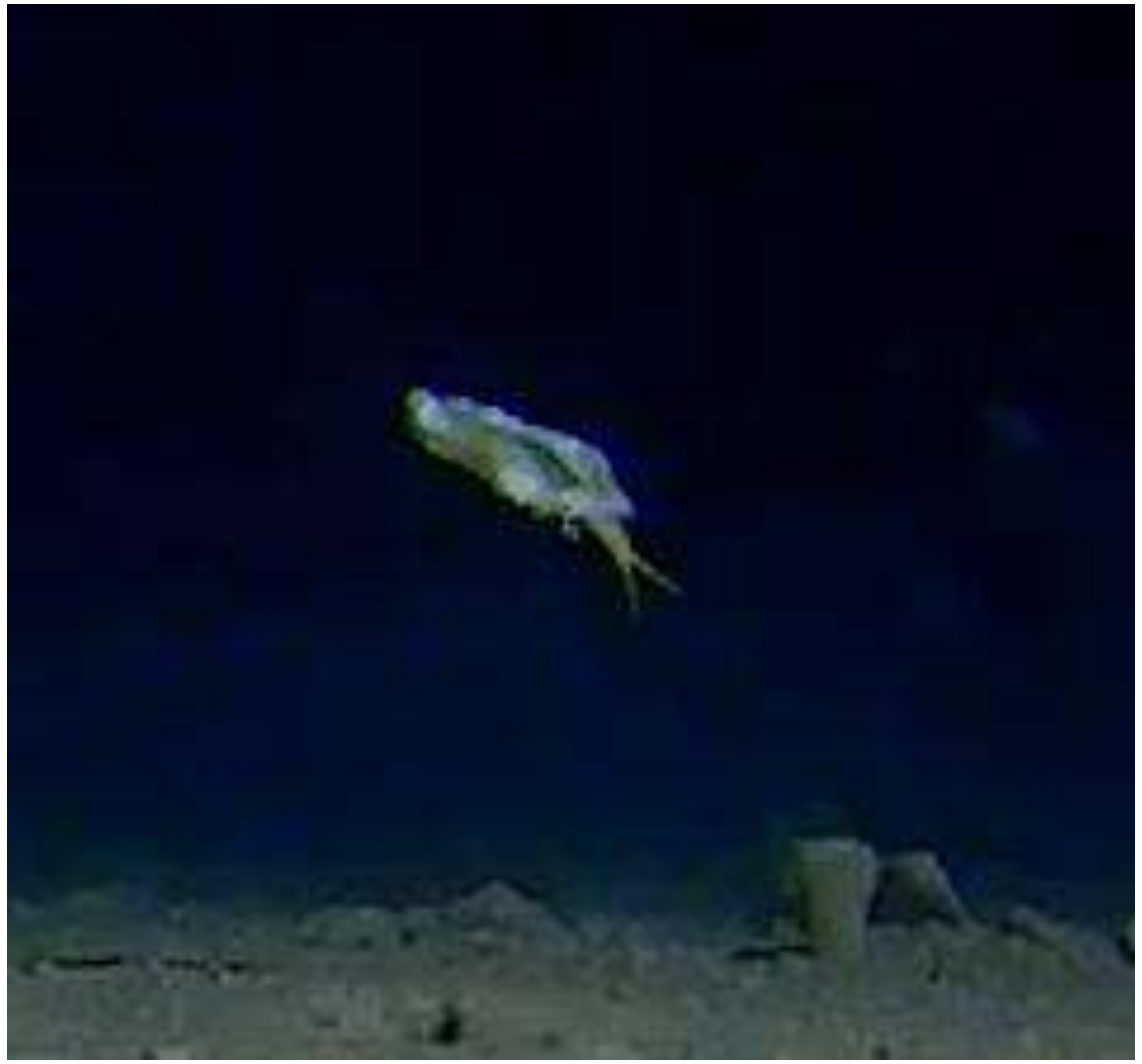

Body has a brownish colouration and is lined on both sides from anterior to posterior end with parapodia. The anterior end is tipped with two antennae like projection. The movement is assisted by the steady undulation of parapodia along with the lateral undulation of the torso.

Polychaeta; Terebellida; Flabelligeridae; Flabelligeridae spp.

NC4_BK1_4700_NOR_0057

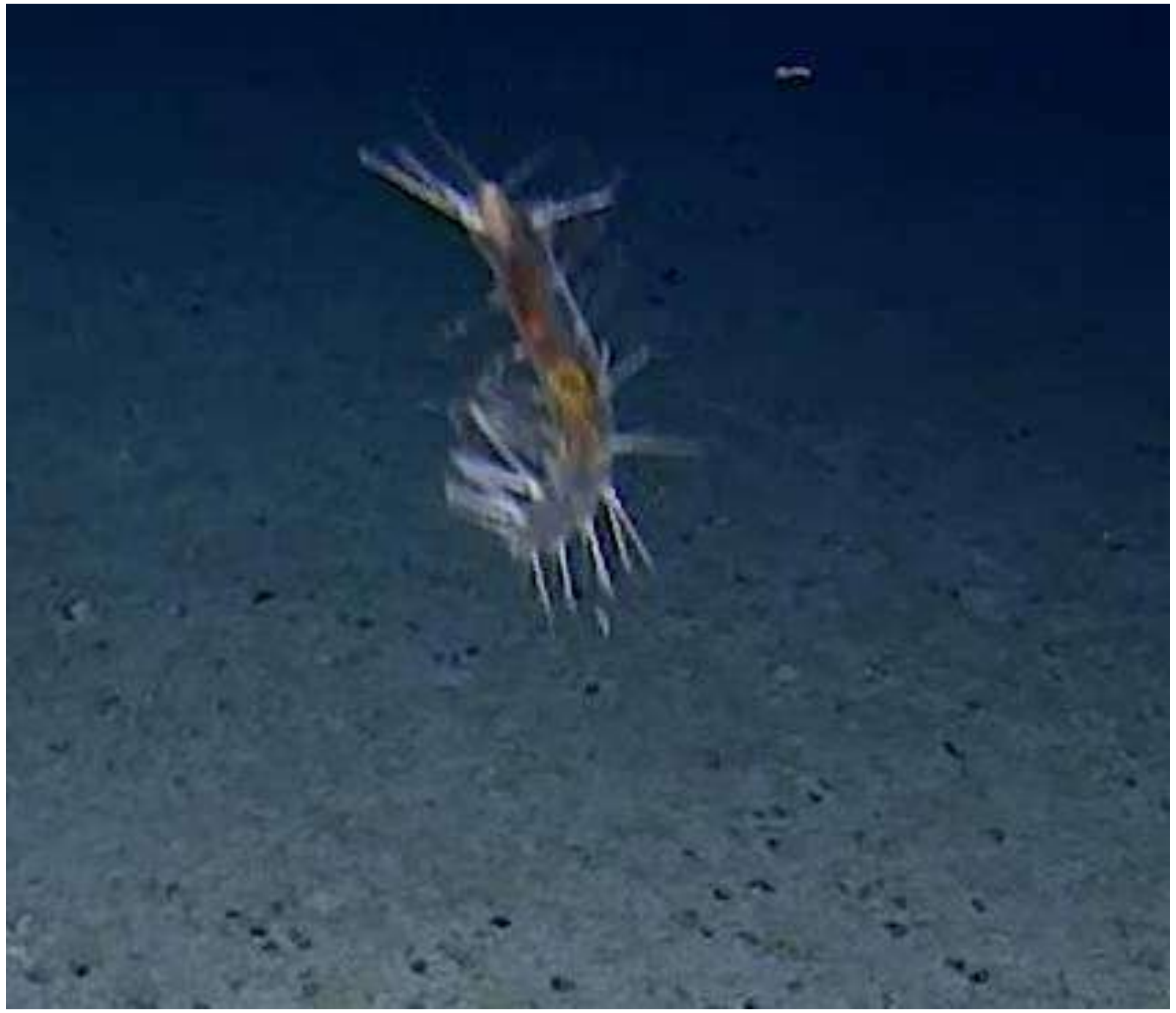

Body is transparent with internal organs visible. Elongated body bears multiple parapodia. Antennae are small and are centred between 4 large, prominent feeding appendages on the anterior end.

Nemertea

NC1_OM5_7500_NOR_0109

Nemertea; Nemertea class indet. 1

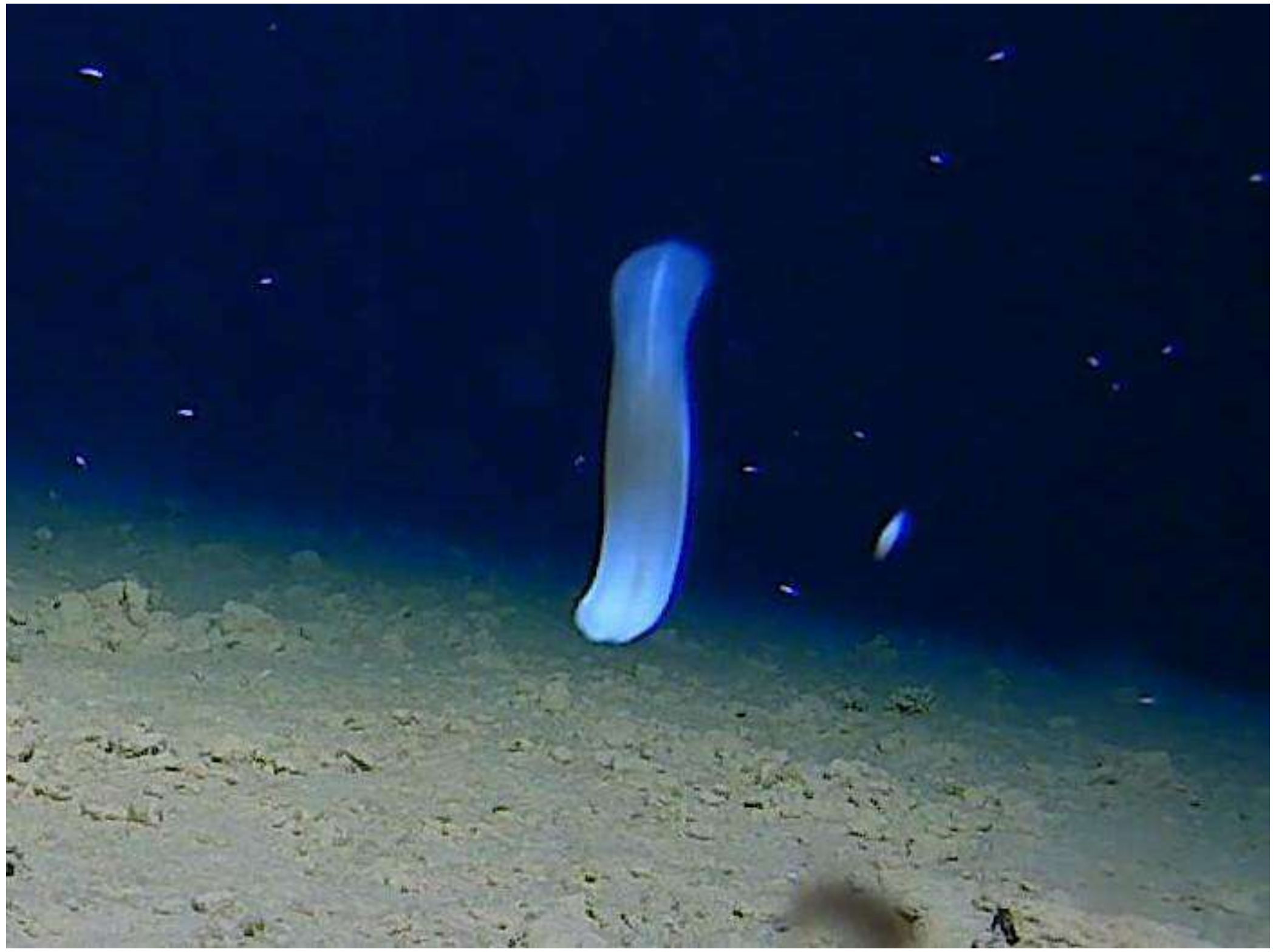

Worm-like in appearance with flattened, smooth, featureless body with rounded terminal ends. Skin is pale-translucent.

NC1_MA5_7300_NOR_0041

Nemertea; Nemertea class indet.2

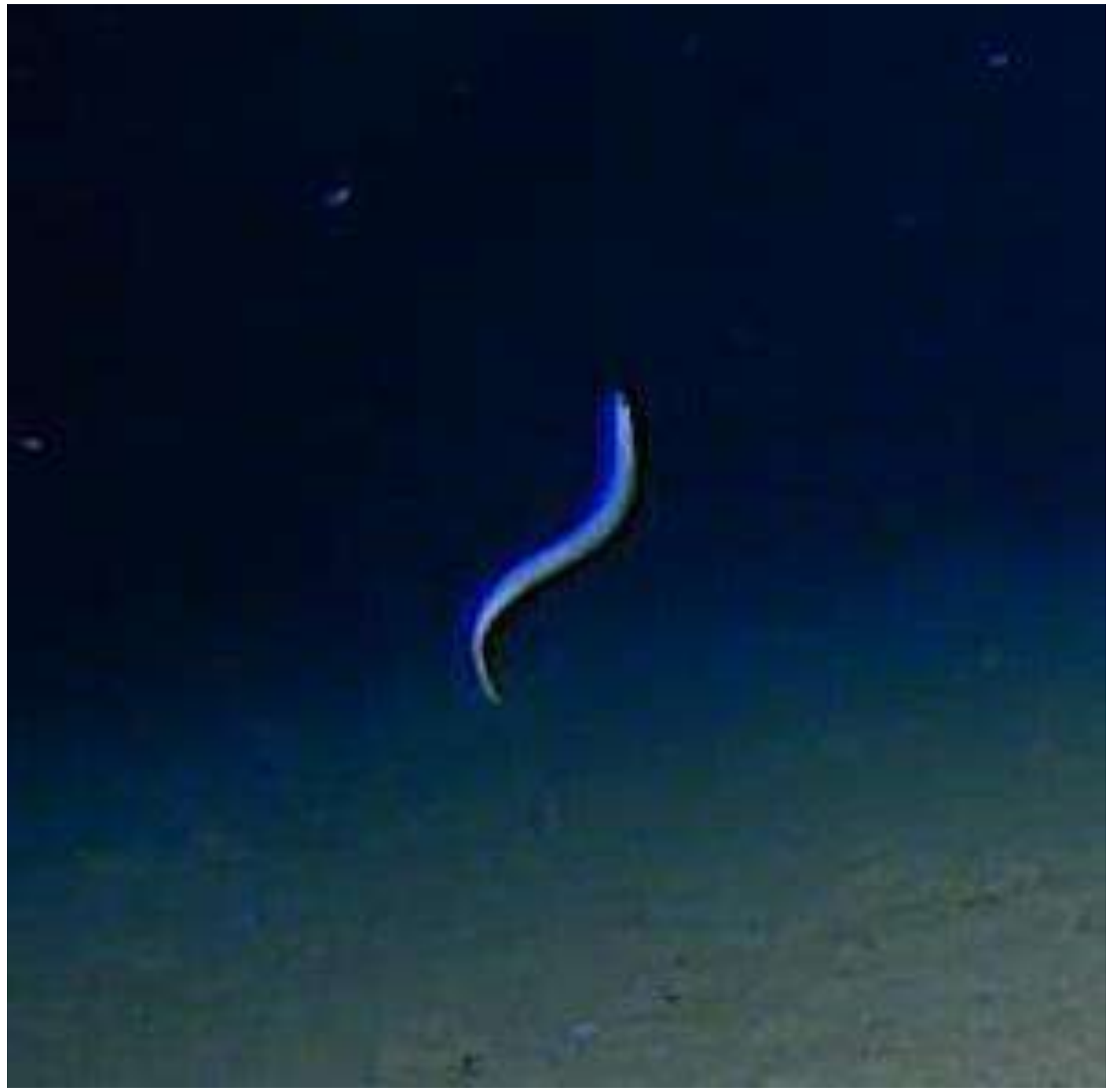

Narrow, dorsal-ventrally flattened, smooth, featureless body. Narrower than sp.1. Swim pattern is an undulating "S" pattern. Skin is translucent.

Nemertea; Nemertea class indet. 3

NC4_OM8_5500_NOR_0097

NC3_MA3_6100_NOR_0087

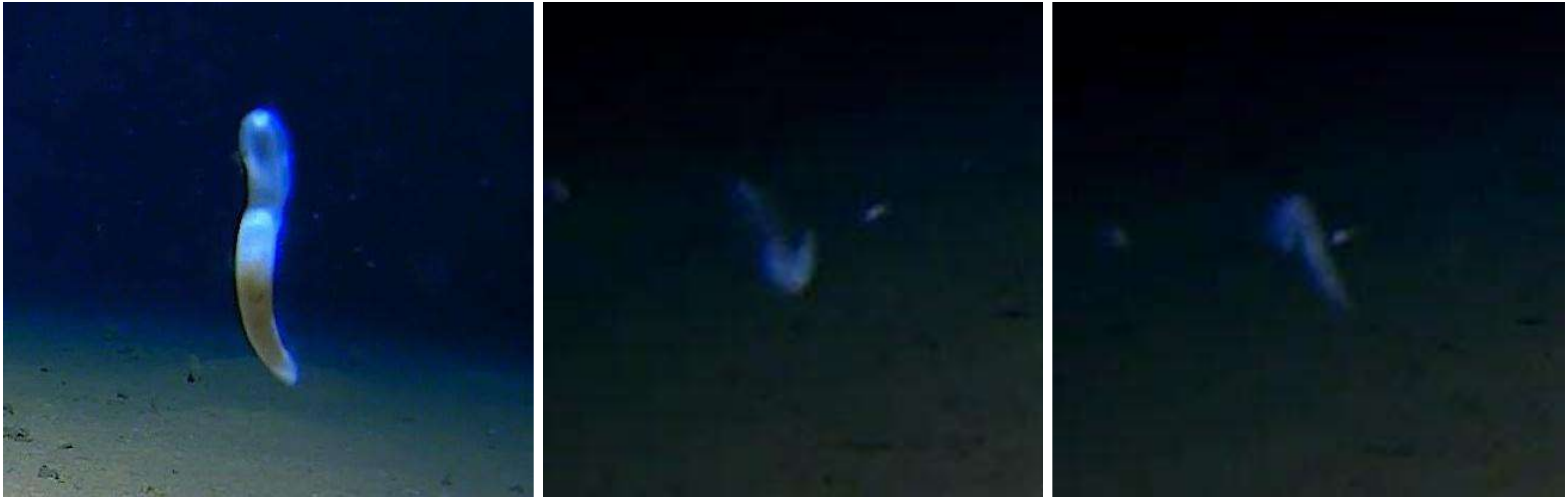

Smooth with rounded, flattened terminal end. Body is pale and semi-translucent. Swim pattern is characterised by the simultaneous flapping of both ends as the animal moves through the water column. Skin is pale and semi-translucent.

Platyhelminthes

Platyhelminthes; Platyhelminthes class indet. 1

NC2_BK4_5300_IMG_9377

NC2_BK4_5300_IMG_9382

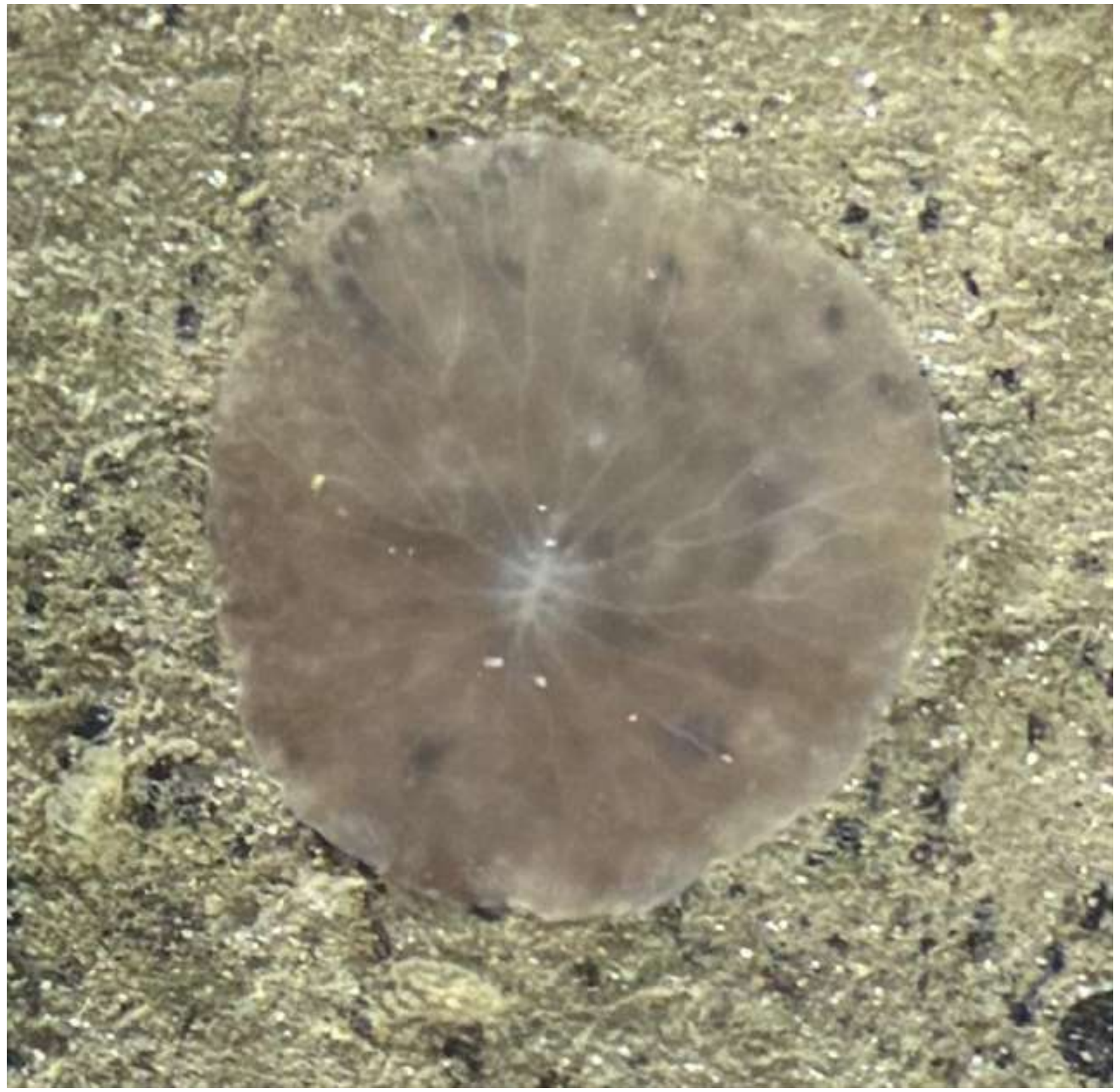

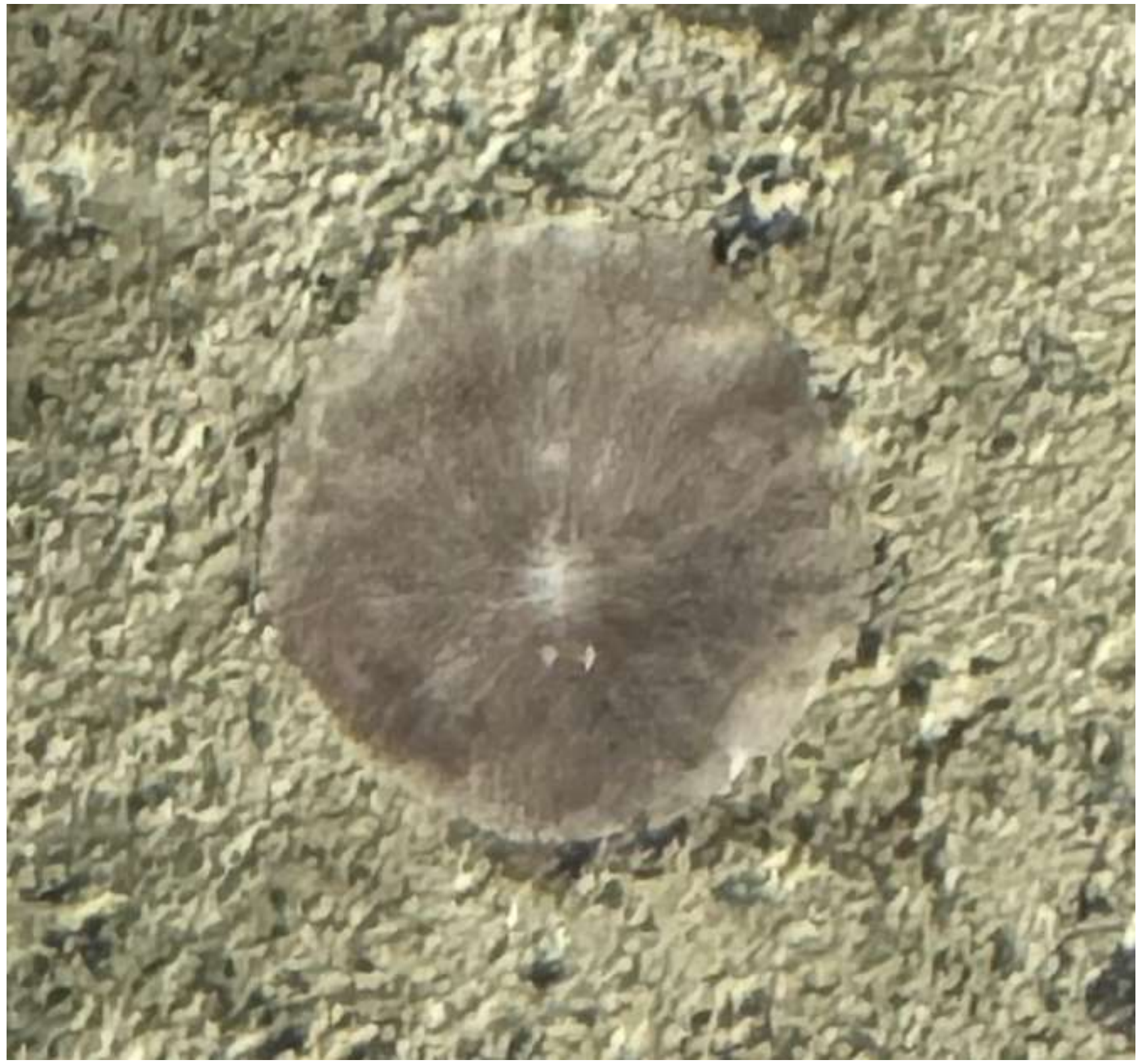

Body is translucent and pinkish in colouration with pale vascular internal structure apparent. Overall body shape is round and flattened.

Bryozoa

Bryozoa; Bryozoa sp 1

NC2_BK4_5300_IMG_9476

NC2_BK4_5300_NOR_0075

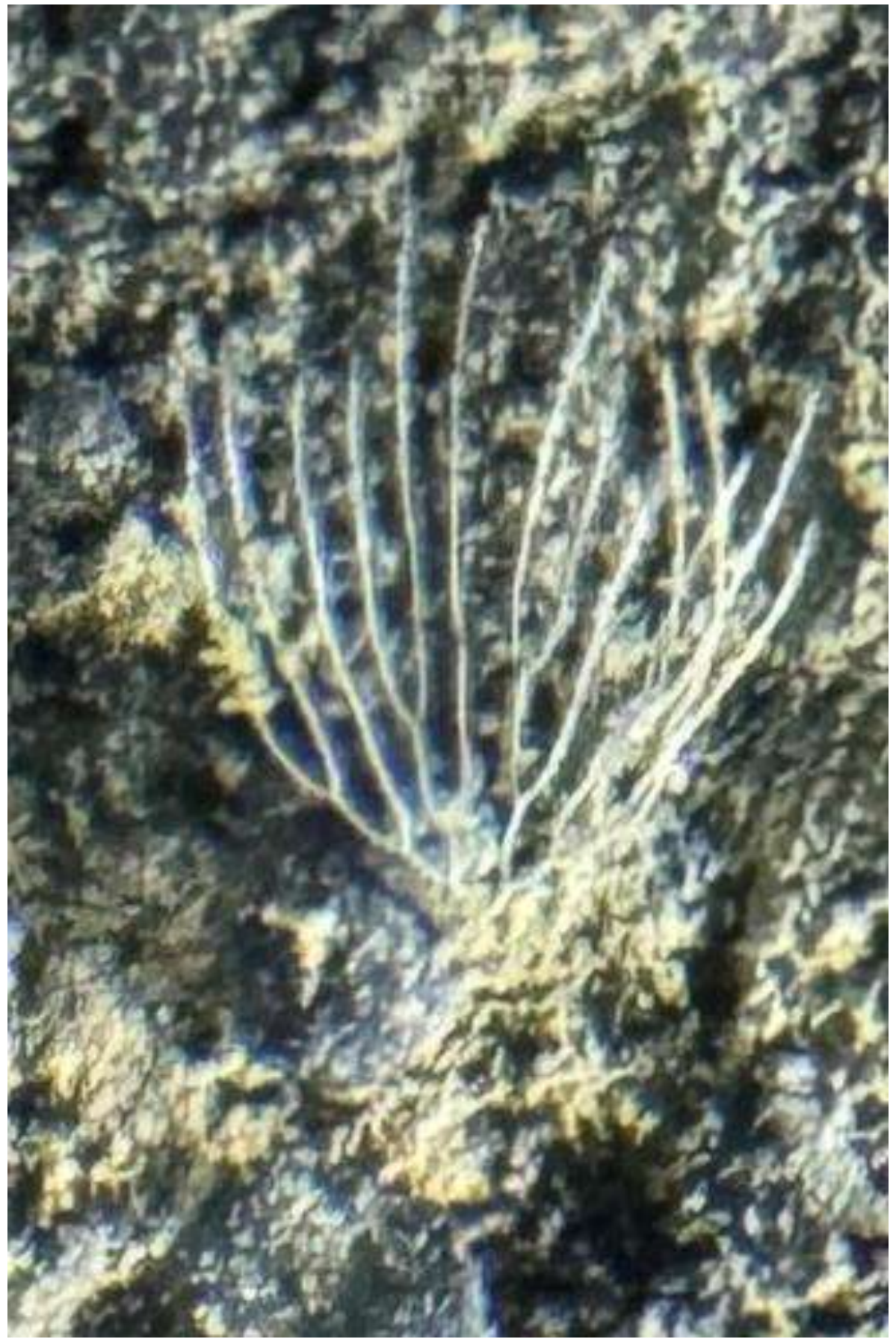

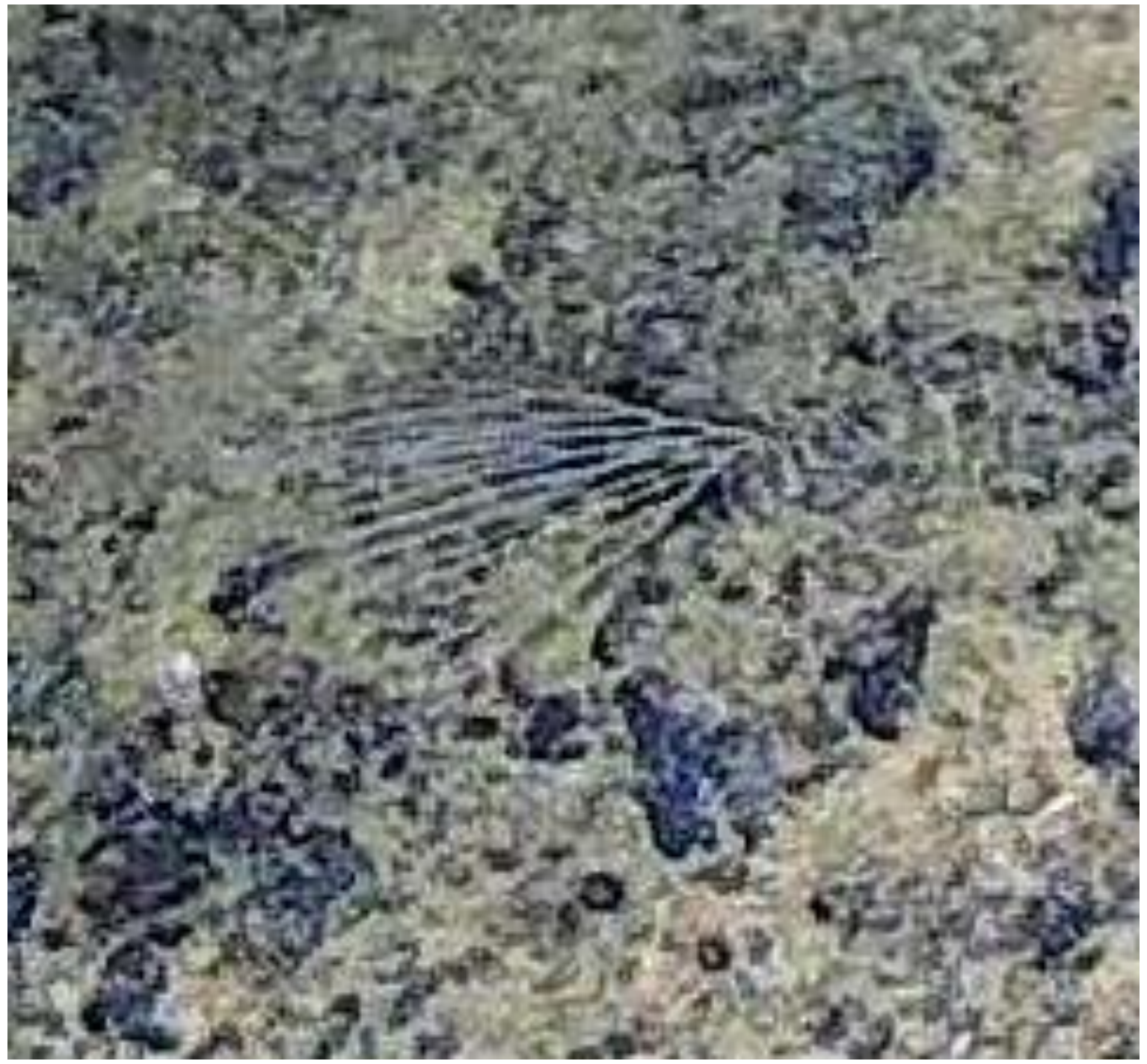

Upward branching structure

Keep in mind there may be other morphs in this dive.

Bryozoa; Bryozoa sp 2

NC2_BK1_3200

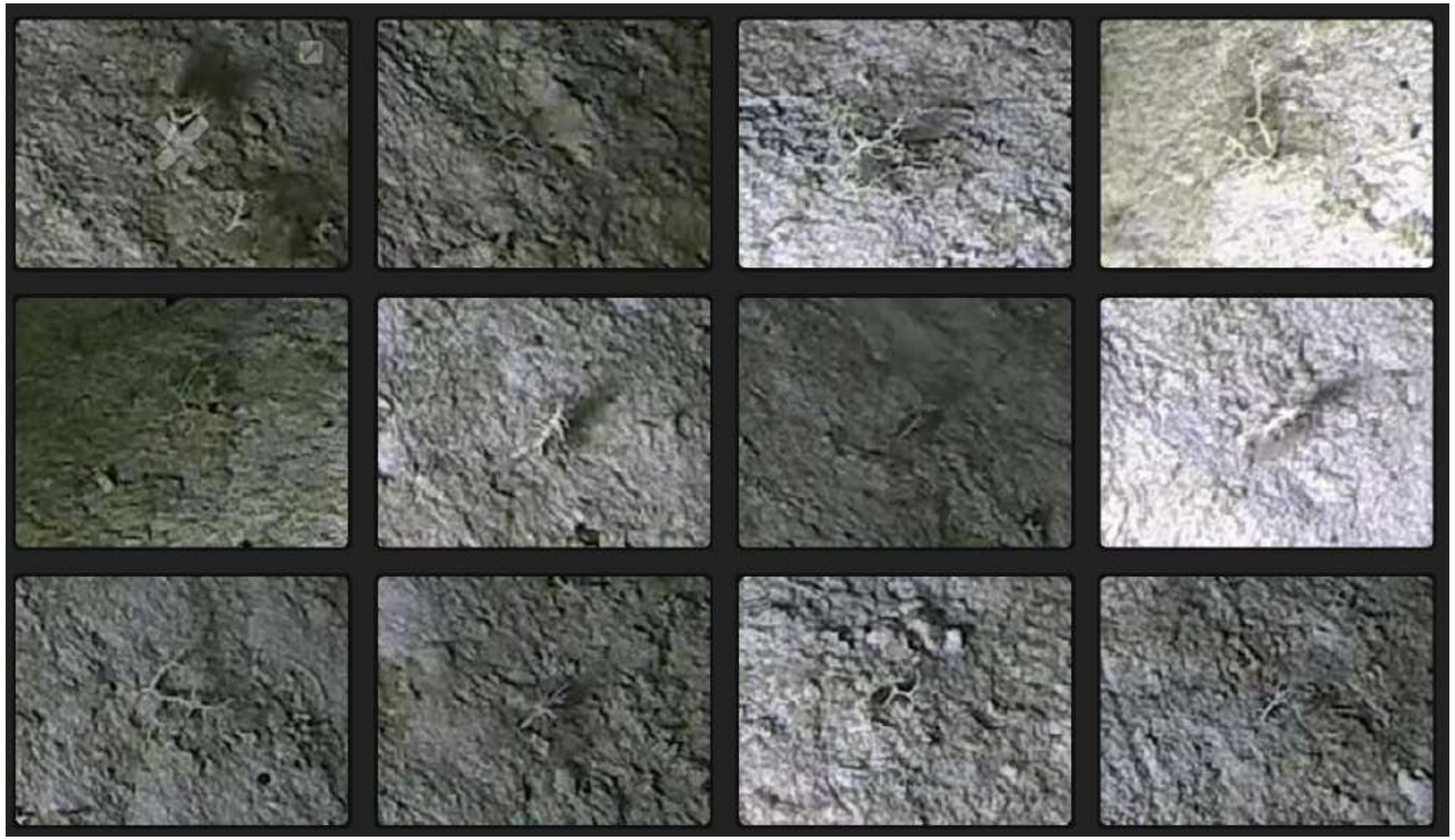

Retaria

Radiozoa; Radiozoa sp. 1

NC2_BK1_3200

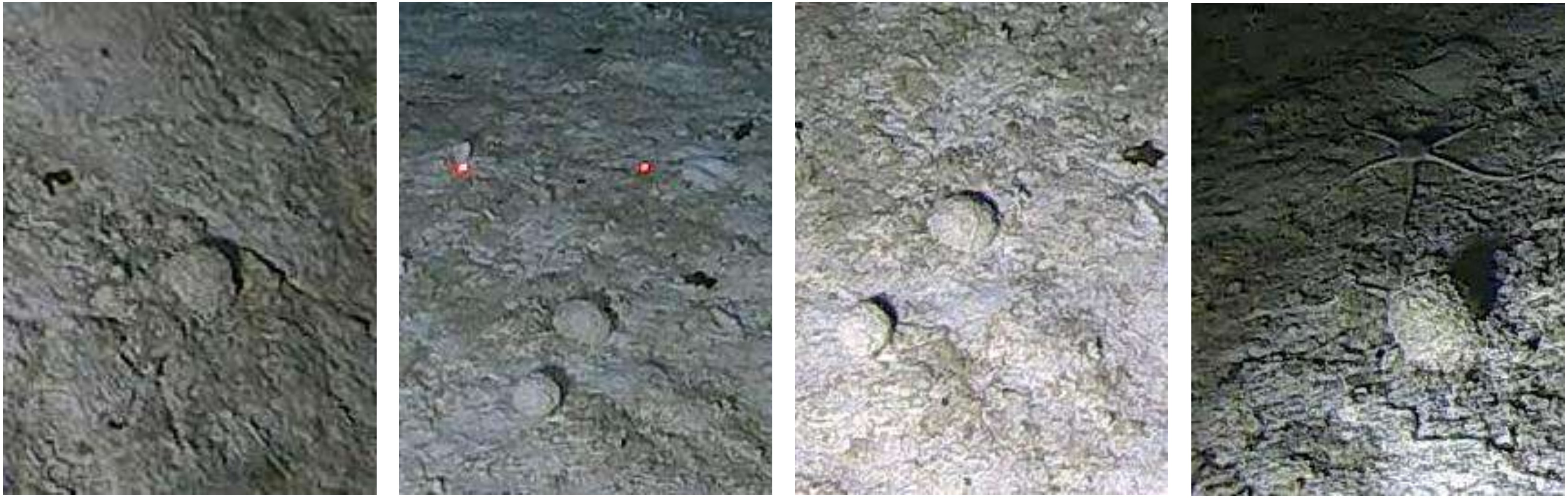

Rounded, meatball-like structure

Radiozoa sp. 2

NC2_BK4_5300_IMG_9470

NC2_BK4_5300_IMG_9433

NC2_BK2_3800_IMG_7945

NC2_BK2_3800_IMG_8066

NC2_BK2_3800_IMG_8152

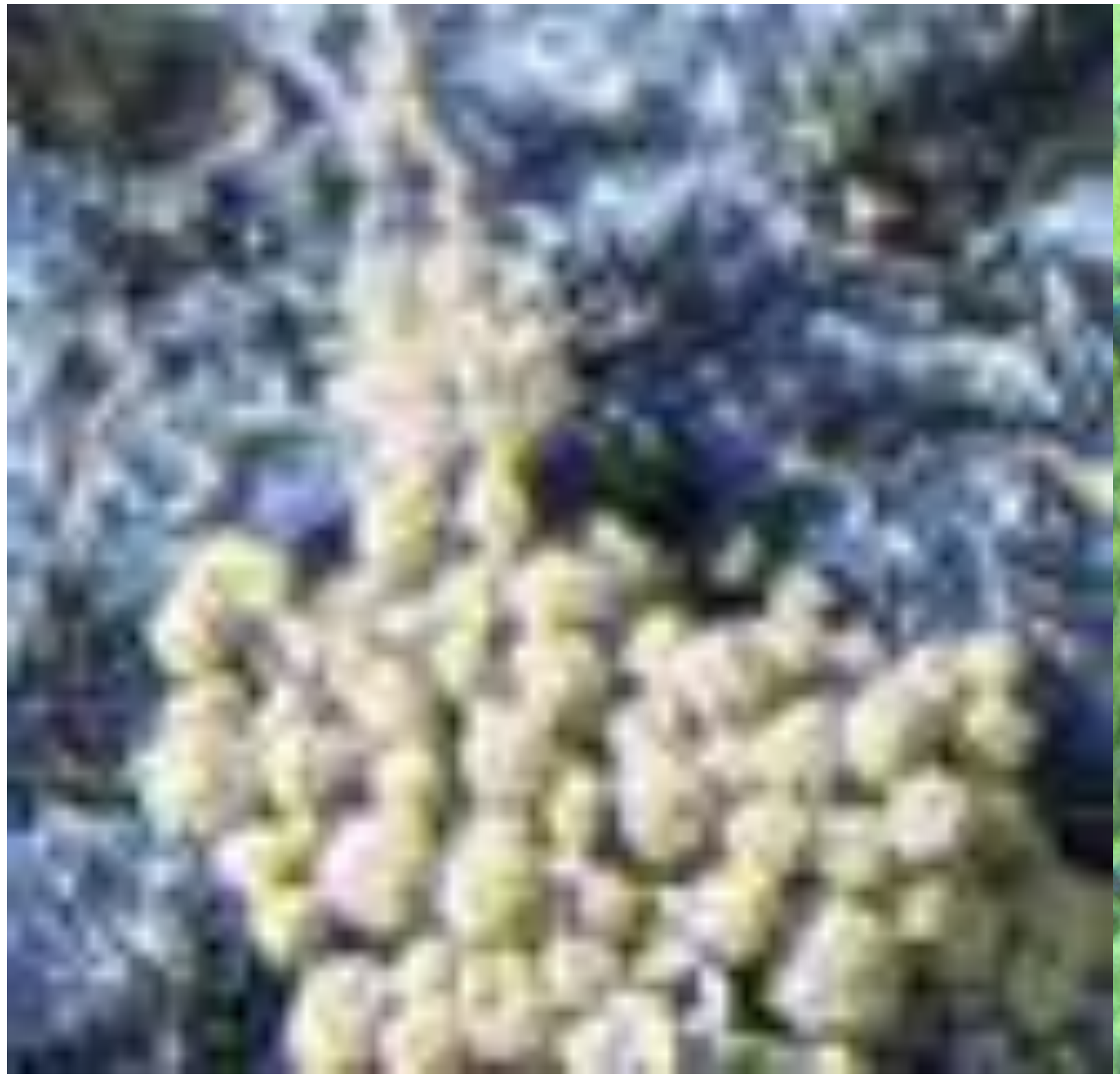

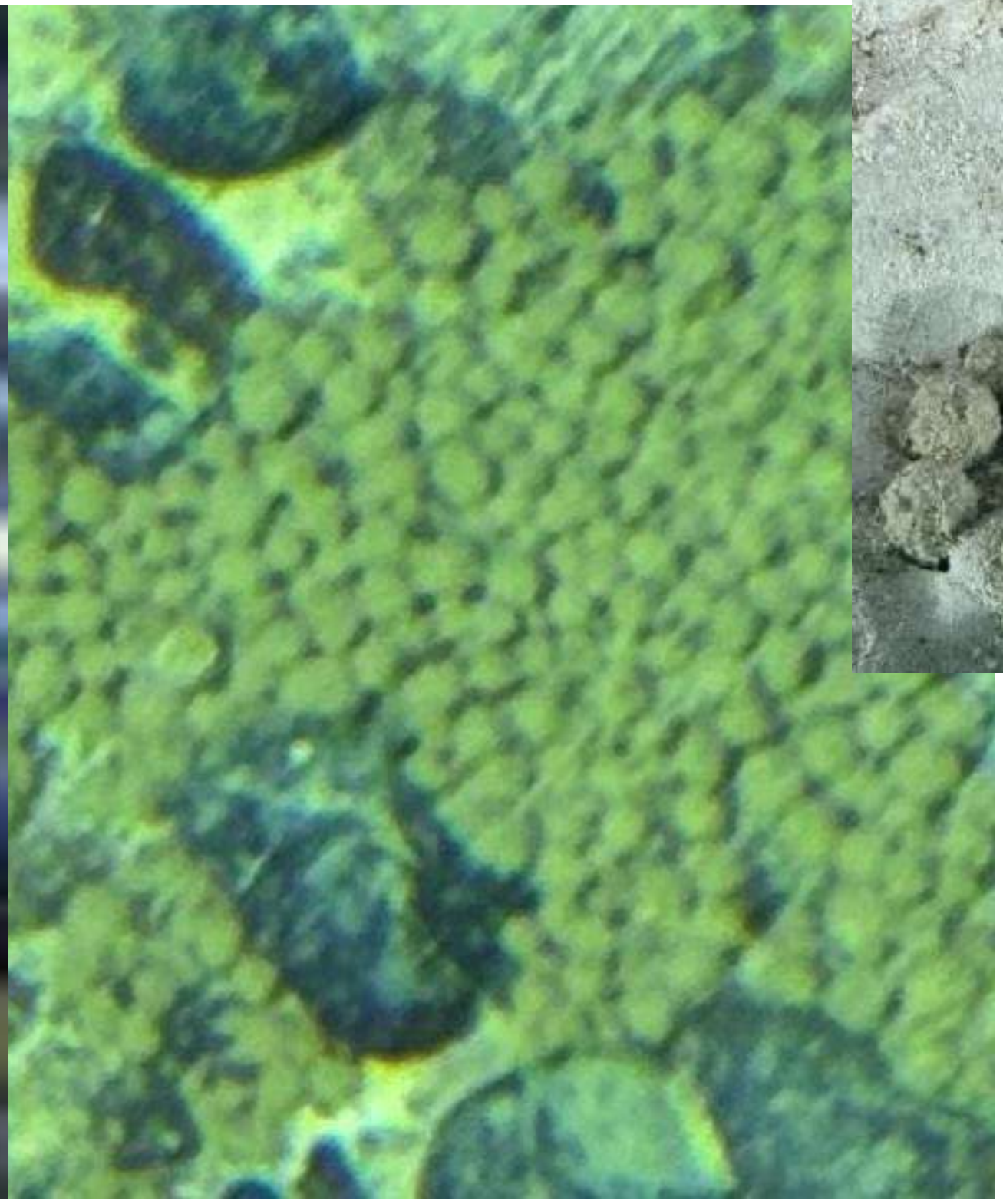

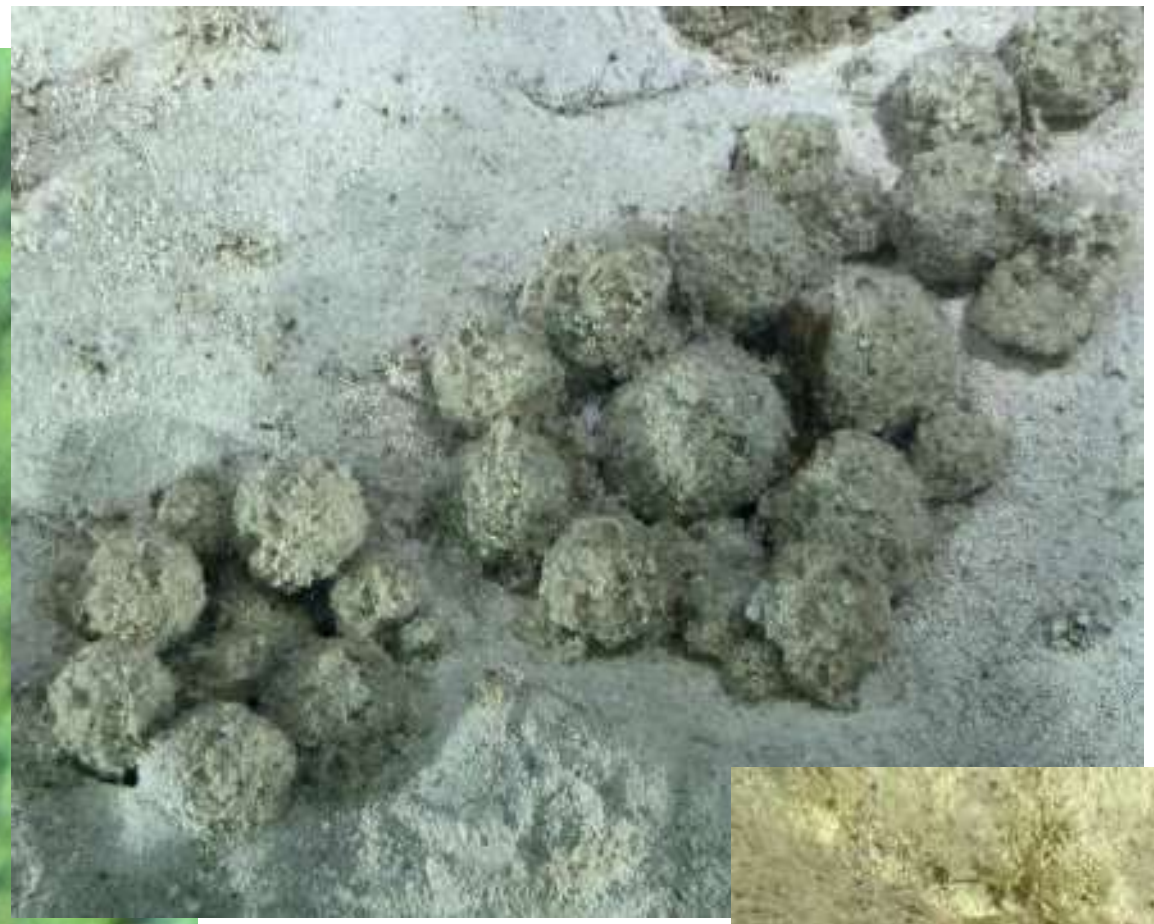

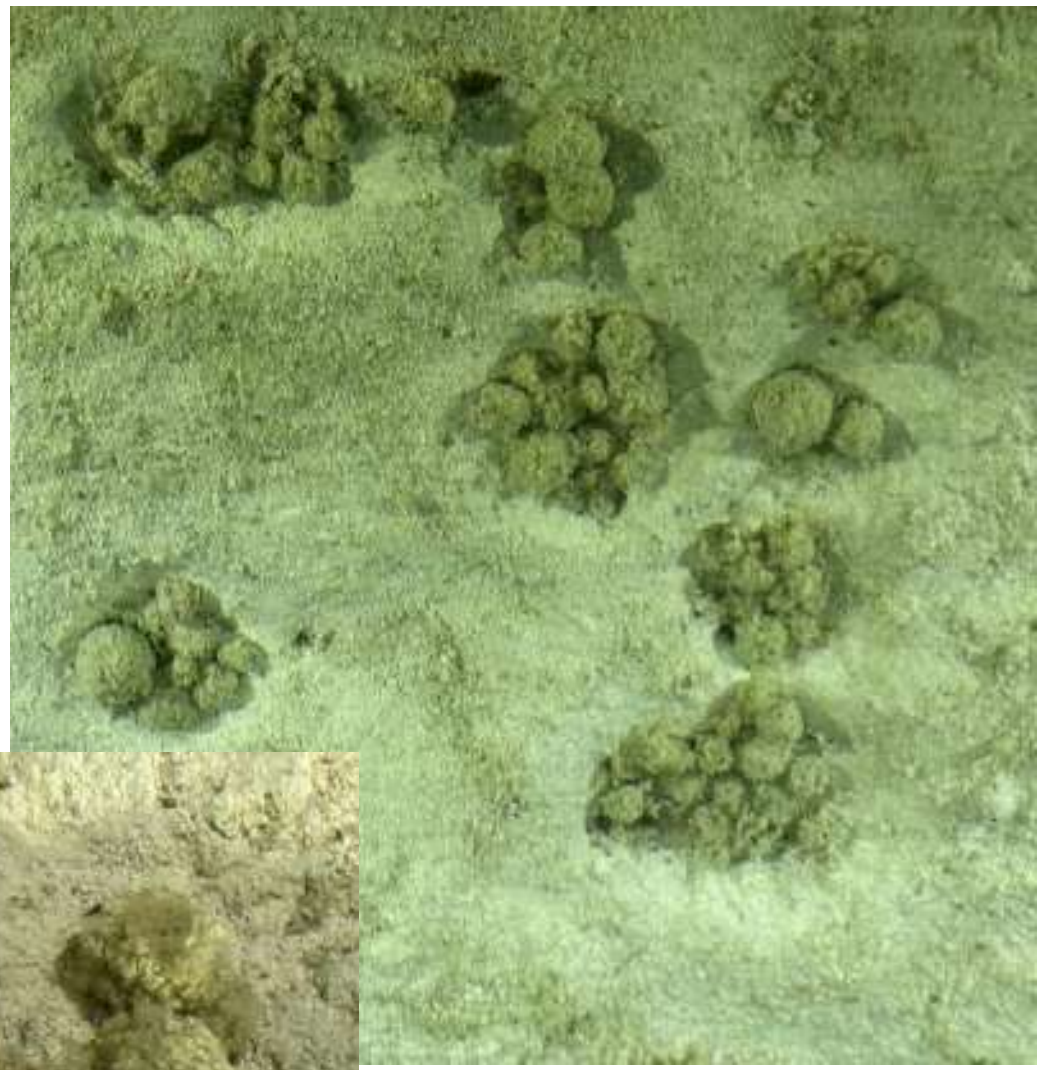

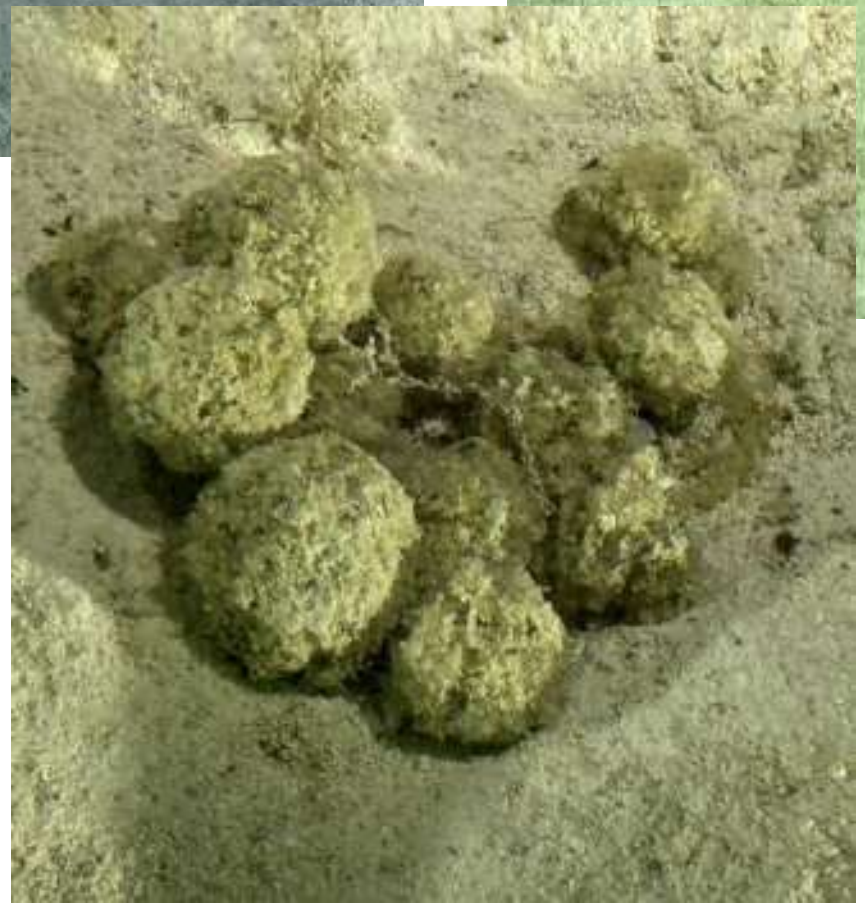

Small, rounded masses. Irregular, filamentous texture.

Radiozoa sp. 3

NC1_BK2_6700_IMG_5164

NC1_BK2_6700_IMG_5183

NC1_BK2_6700_IMG_5184

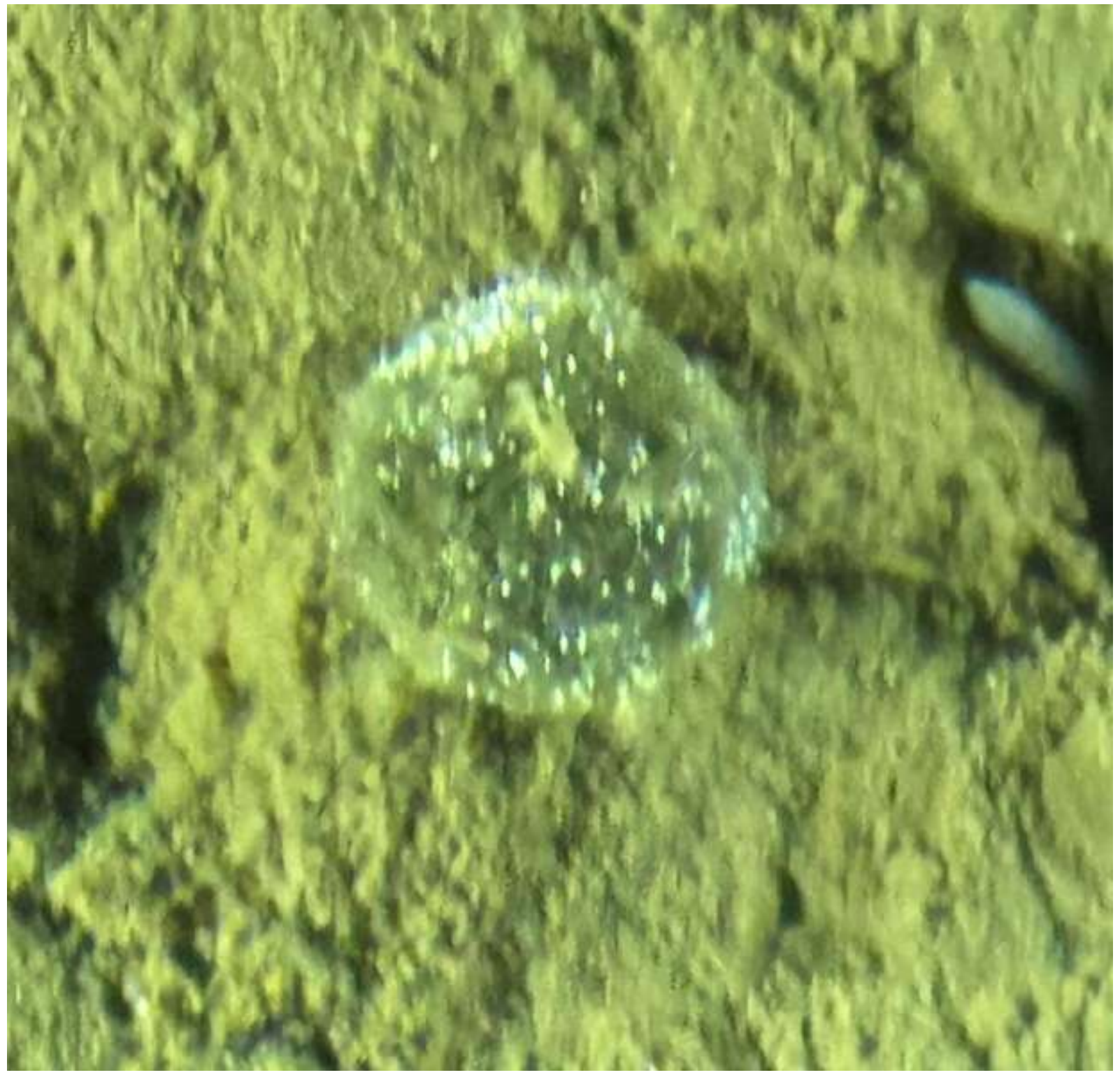
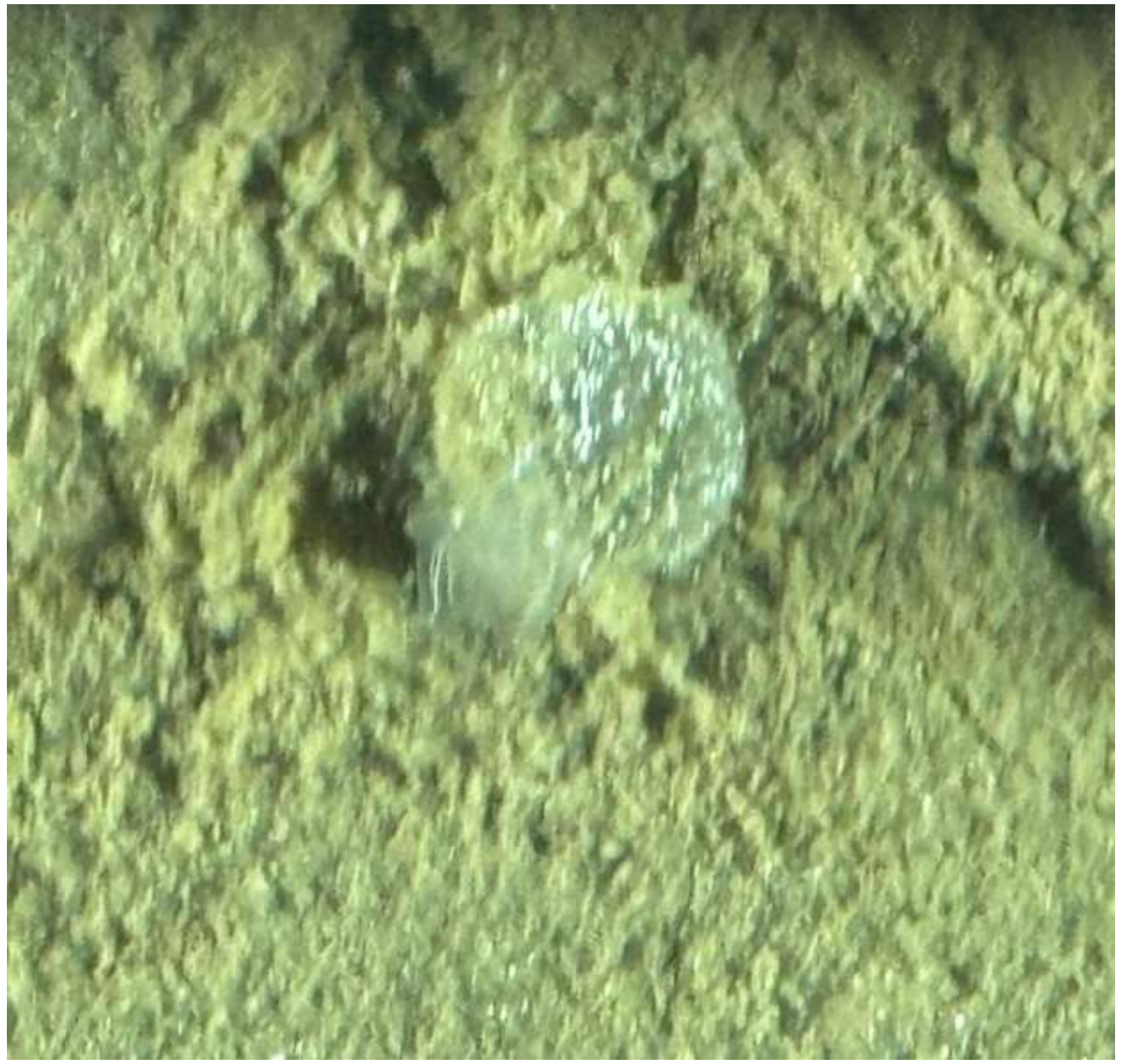
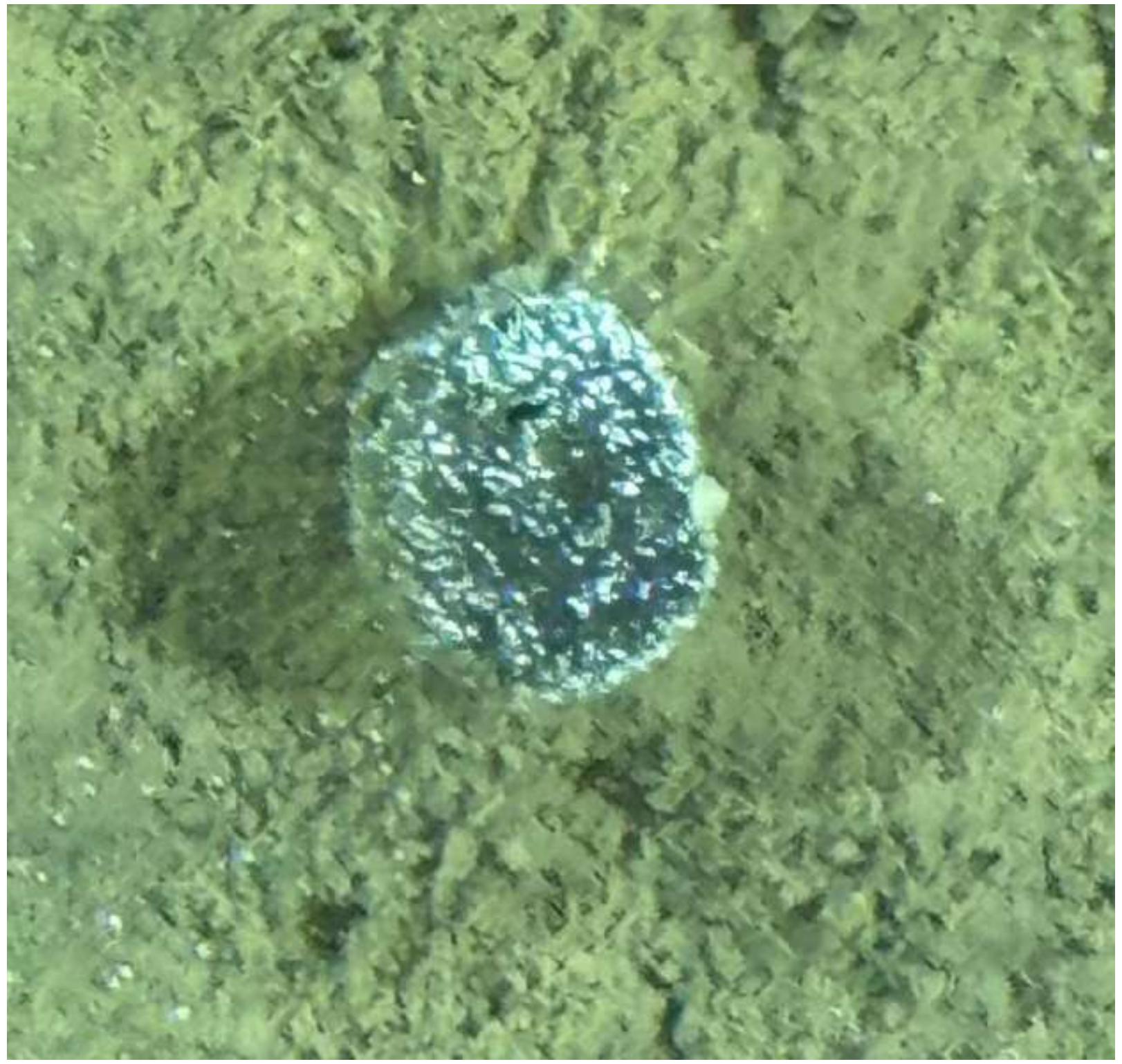

Rounded, body is generally transparent and covered in white spots. Observed resting on soft sediments.

Cercozoa Tuscaridium; Tuscaridium spp

NC3_MA8_2800_NOR_0121

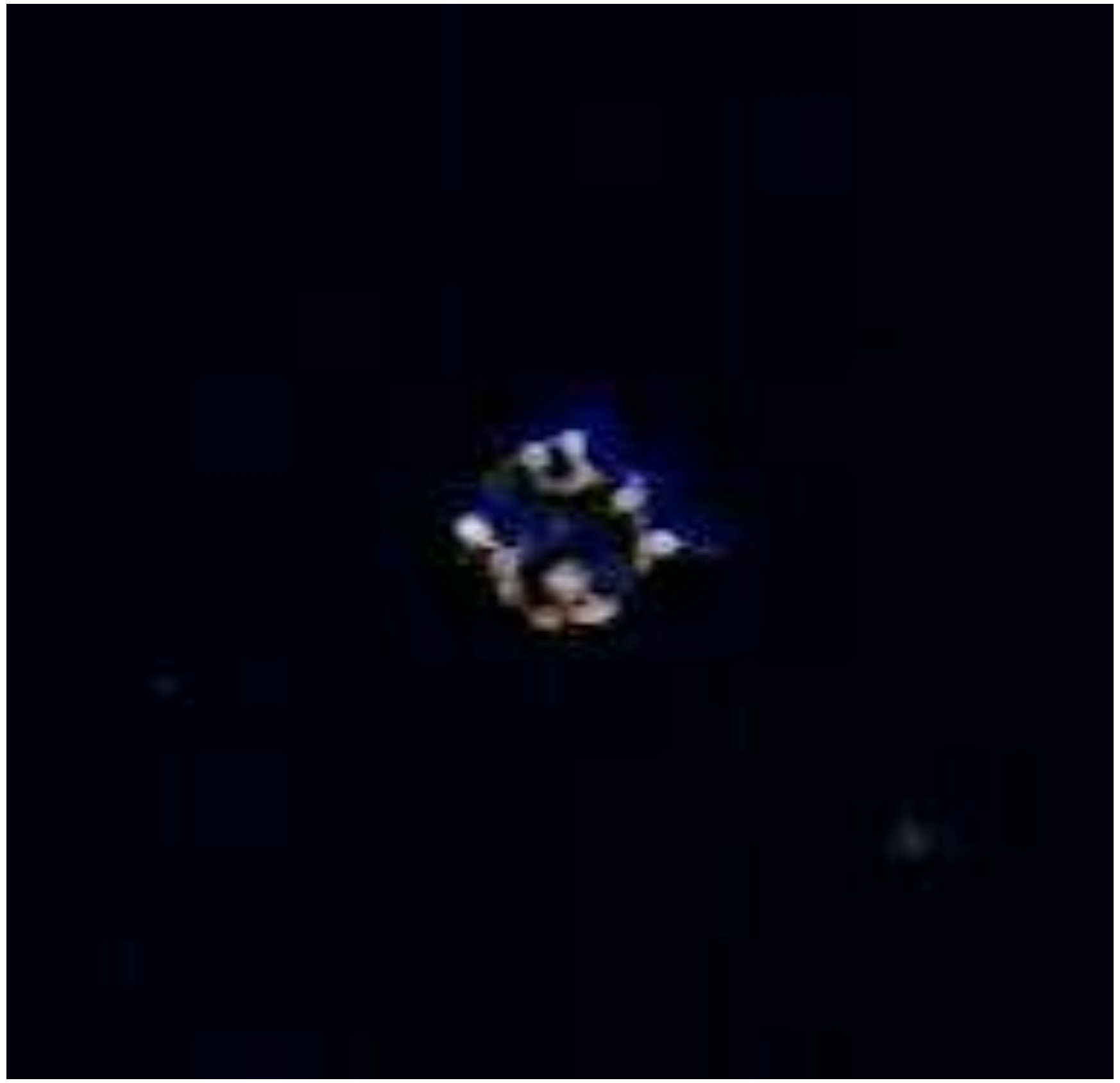

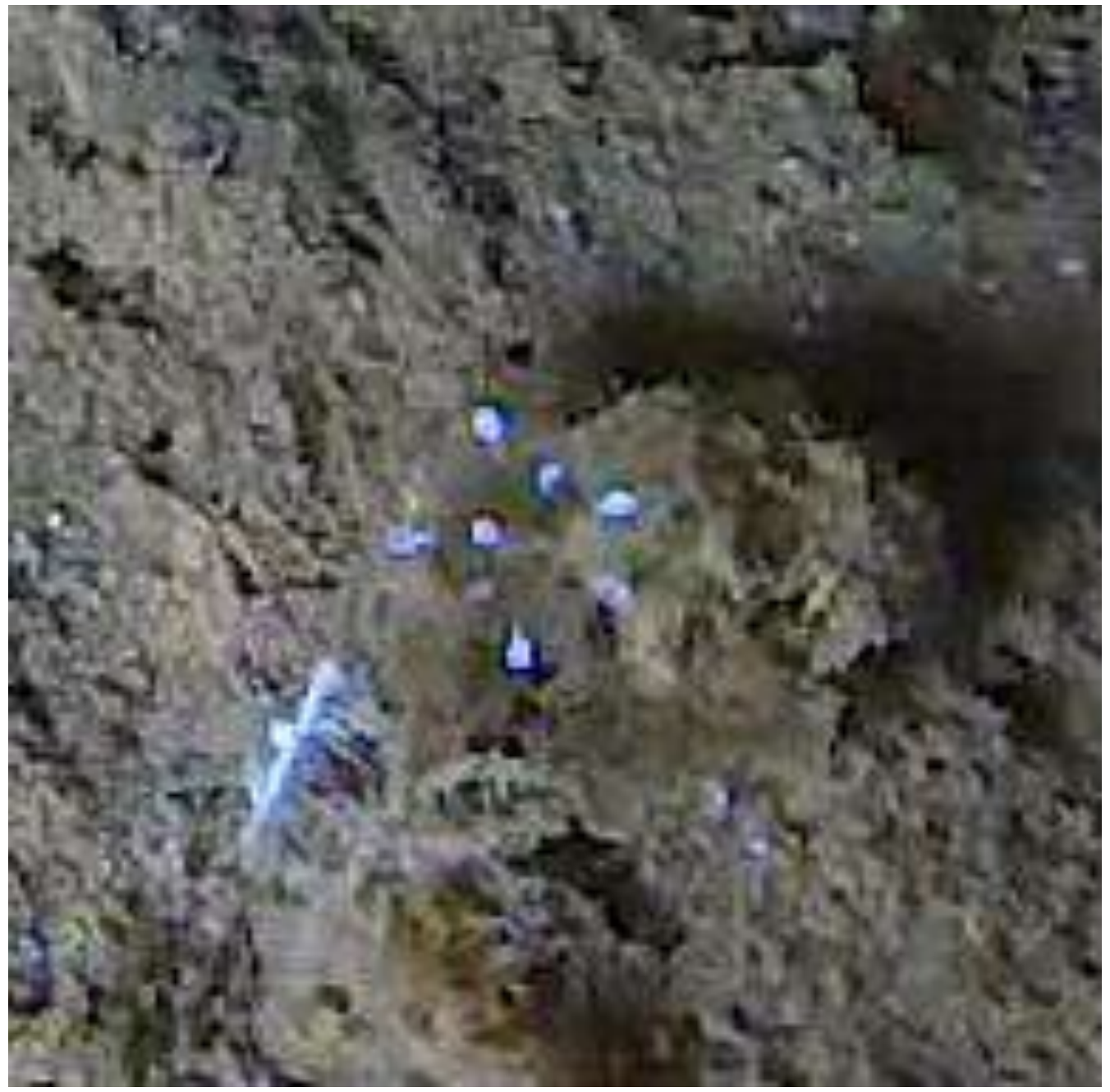

Body is round and translucent with multiple pale-coloured pseudopods covering the outer surface.

Foraminifera; Monothalamea

Examples here are presented as illustrative only; xenophyophoroidea were not annotated in the dataset.

Foraminifera; Monothalamea sp. 1

NC1_OM5_7500_NOR_0109
NC2_BK2_3800_IMG_7950
NC2_BK3_7400_IMG_8763

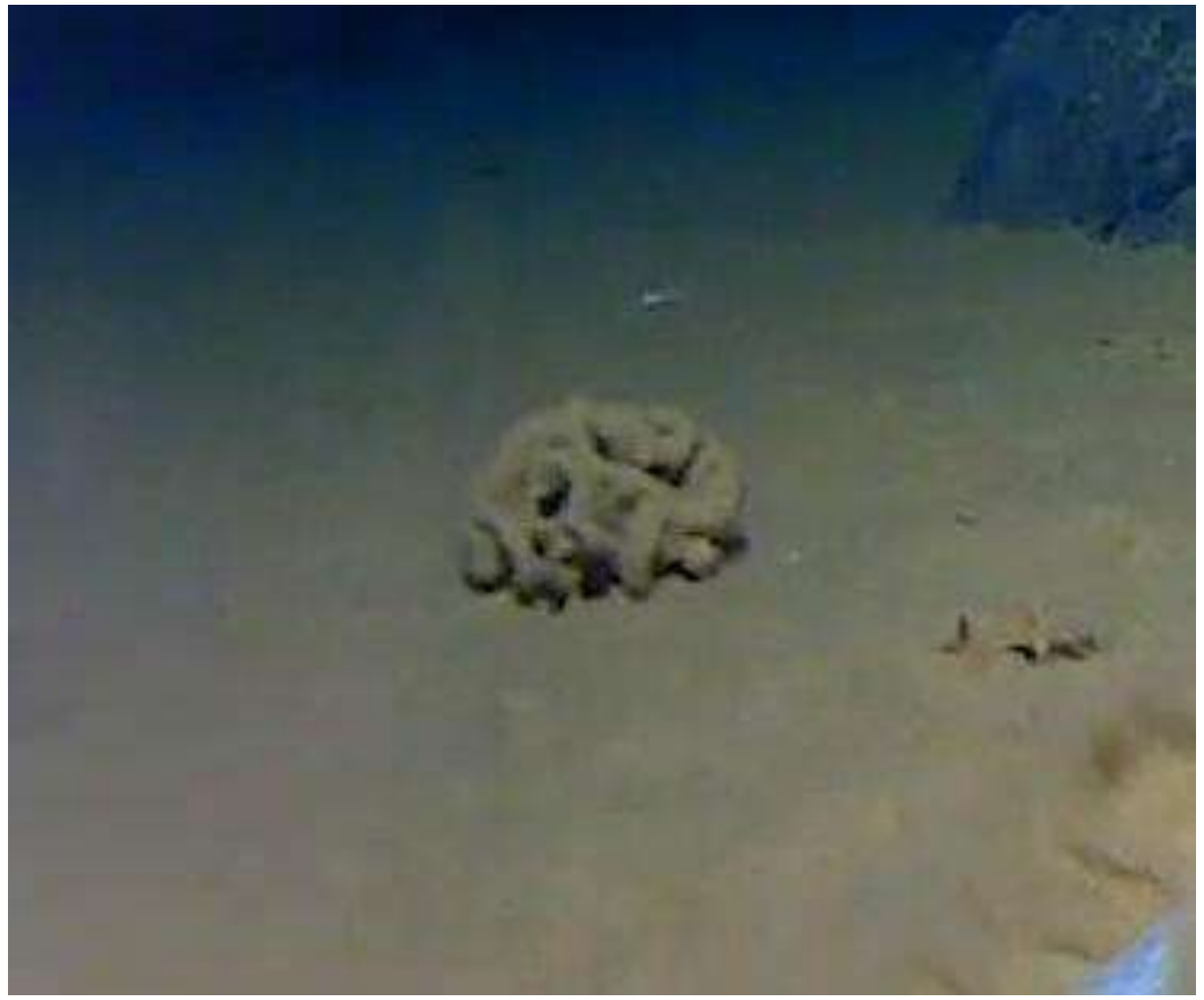
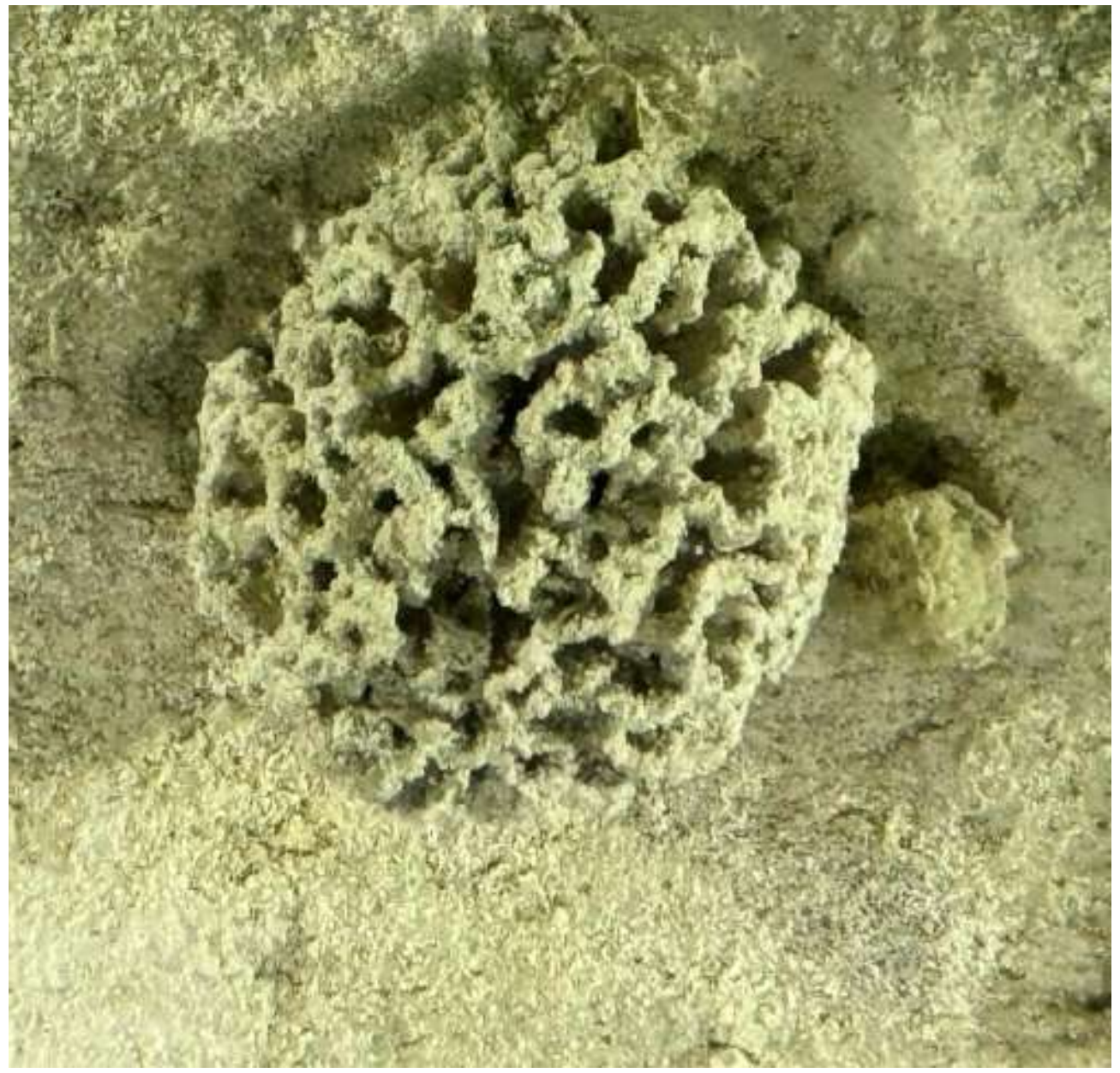
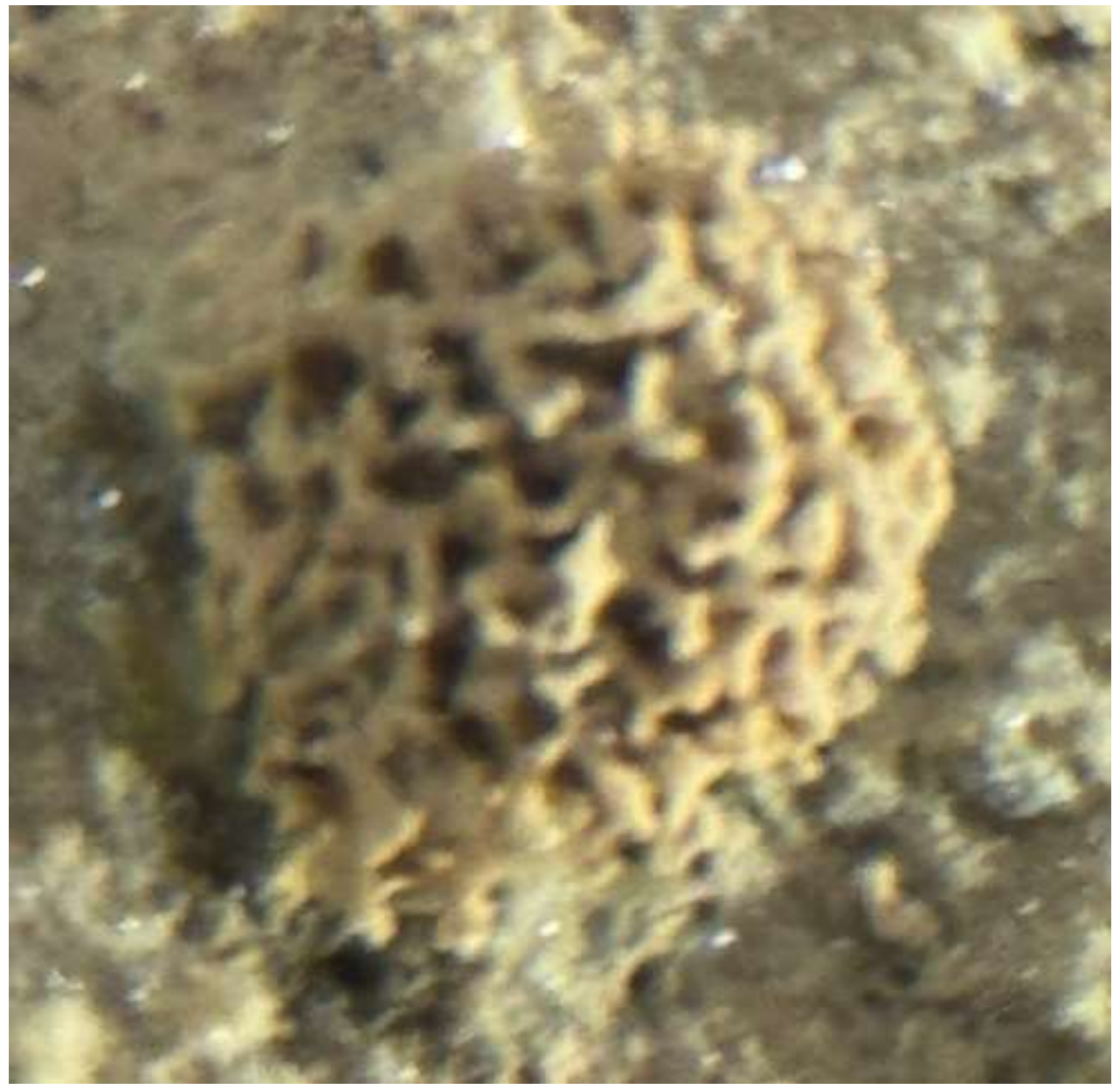

Overall rounded structure with “cerebral-like” structure.

NC2_BK4_5300_IMG_9468

Foraminifera; Monothalamea sp. 2

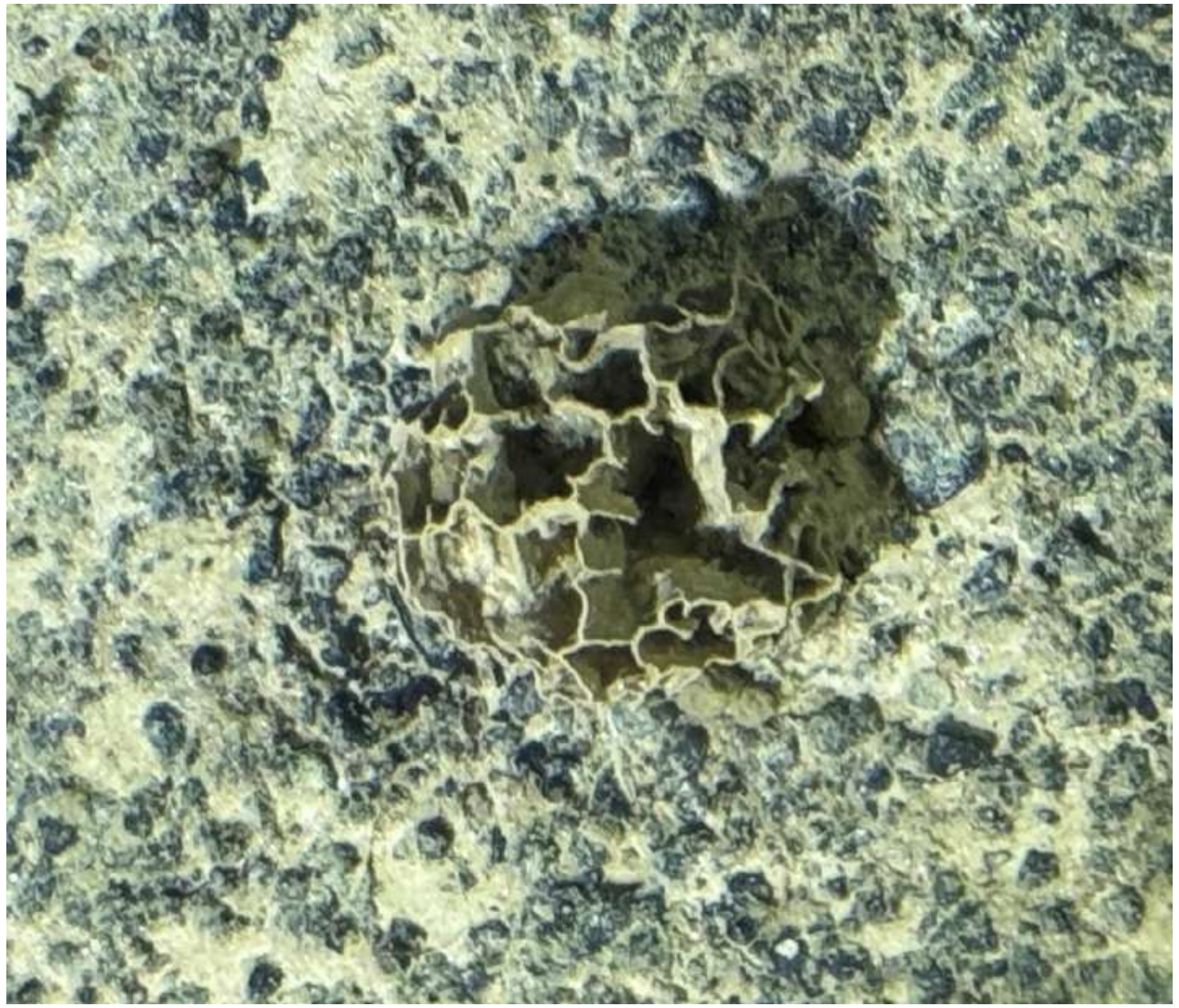

Overall rounded, "cerebral-like" structure with thin "walls"

Foraminifera; Monothalamea sp. 3

NC1_BK2_6700_IMG_5144
NC2_BK2_3800_IMG_8028

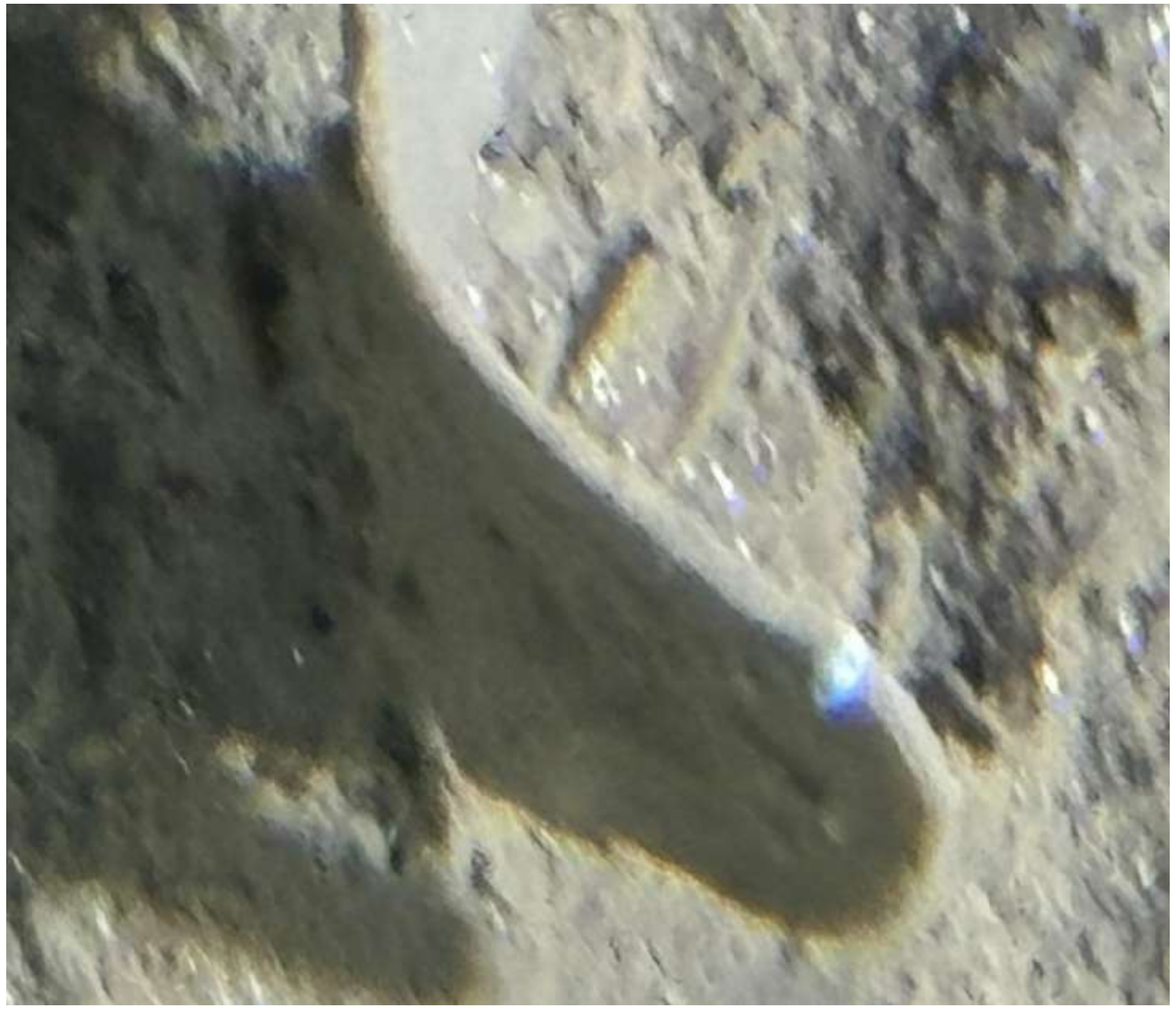

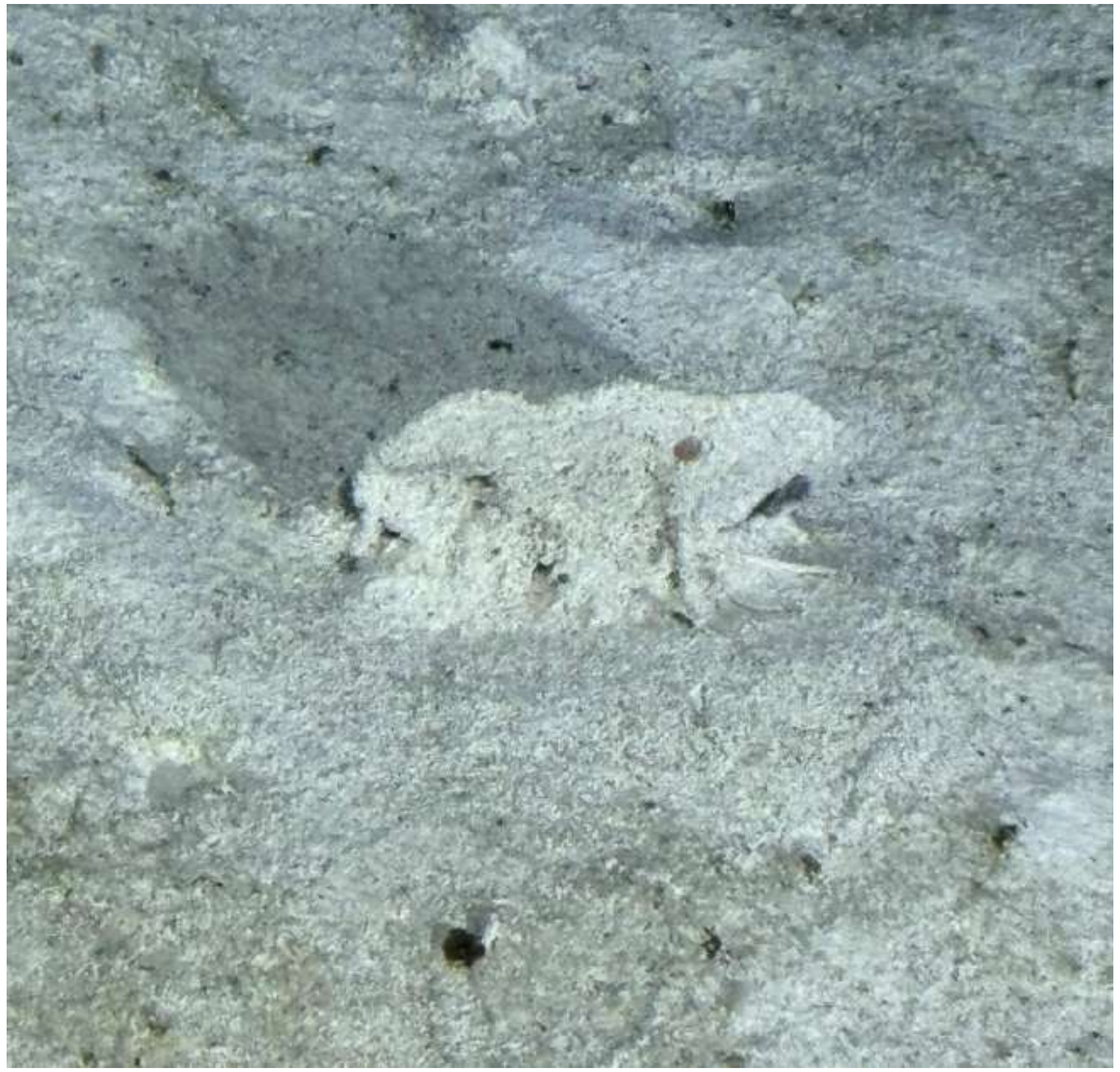

Flattened, “fan-like” structure

Foraminifera; Monothalamea sp. 4

NC1_BK3_8000_IMG_5881
NC3_BK4_8000_IMG_3814

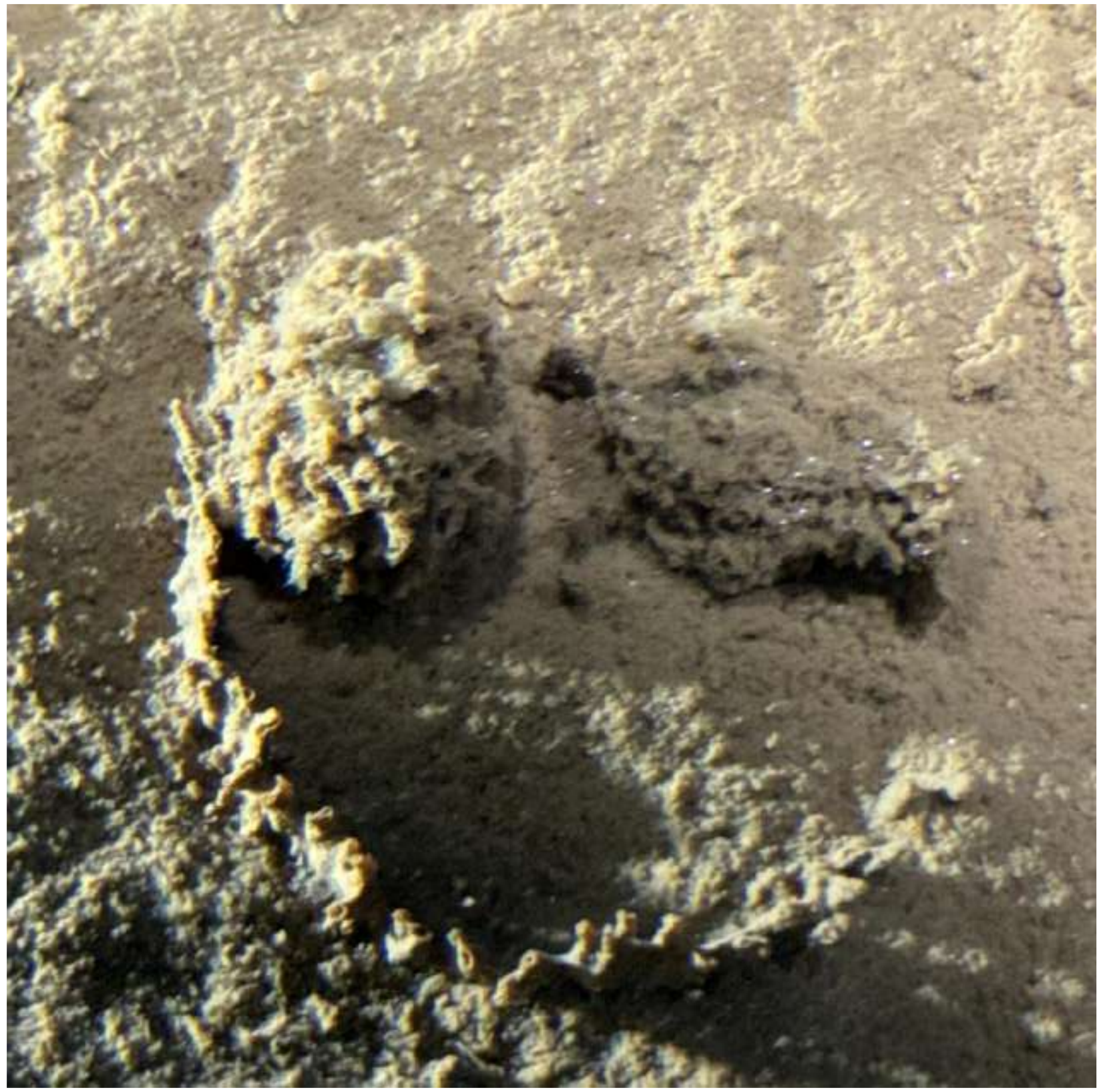

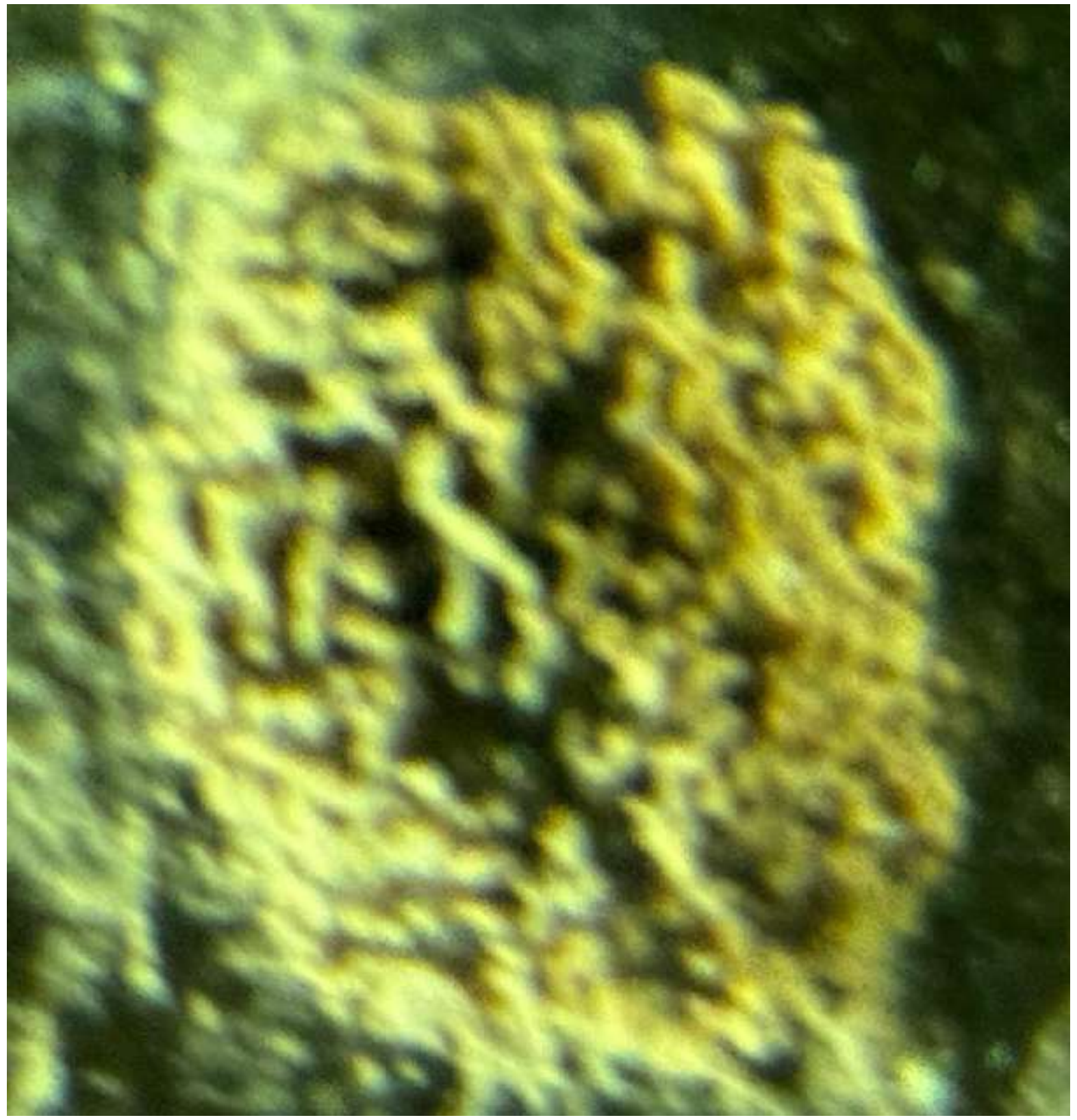

Ovoid shape with tightly bound, irregular "branches". Covered with a filamentous texture.

NC2_BK3_7400_IMG_8759

Foraminifera; Monothalamea sp. 5

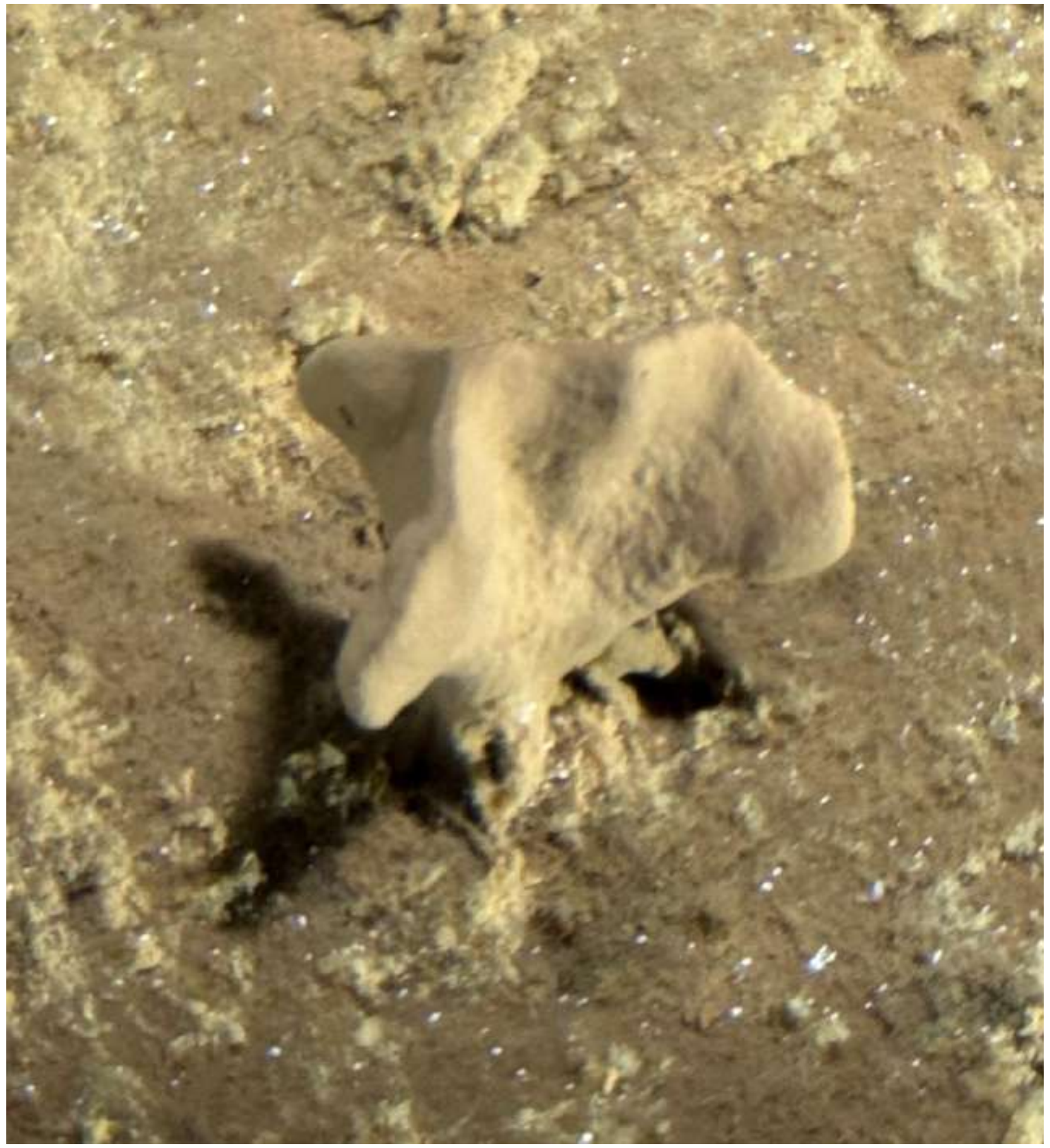

Wavy, flattened structure. Wall thicker than sp.4

Foraminifera: Monothalamea sp. 6

NC2_BK3_7400 _IMG_6022
NC1_BK1_8000 _IMG_0595

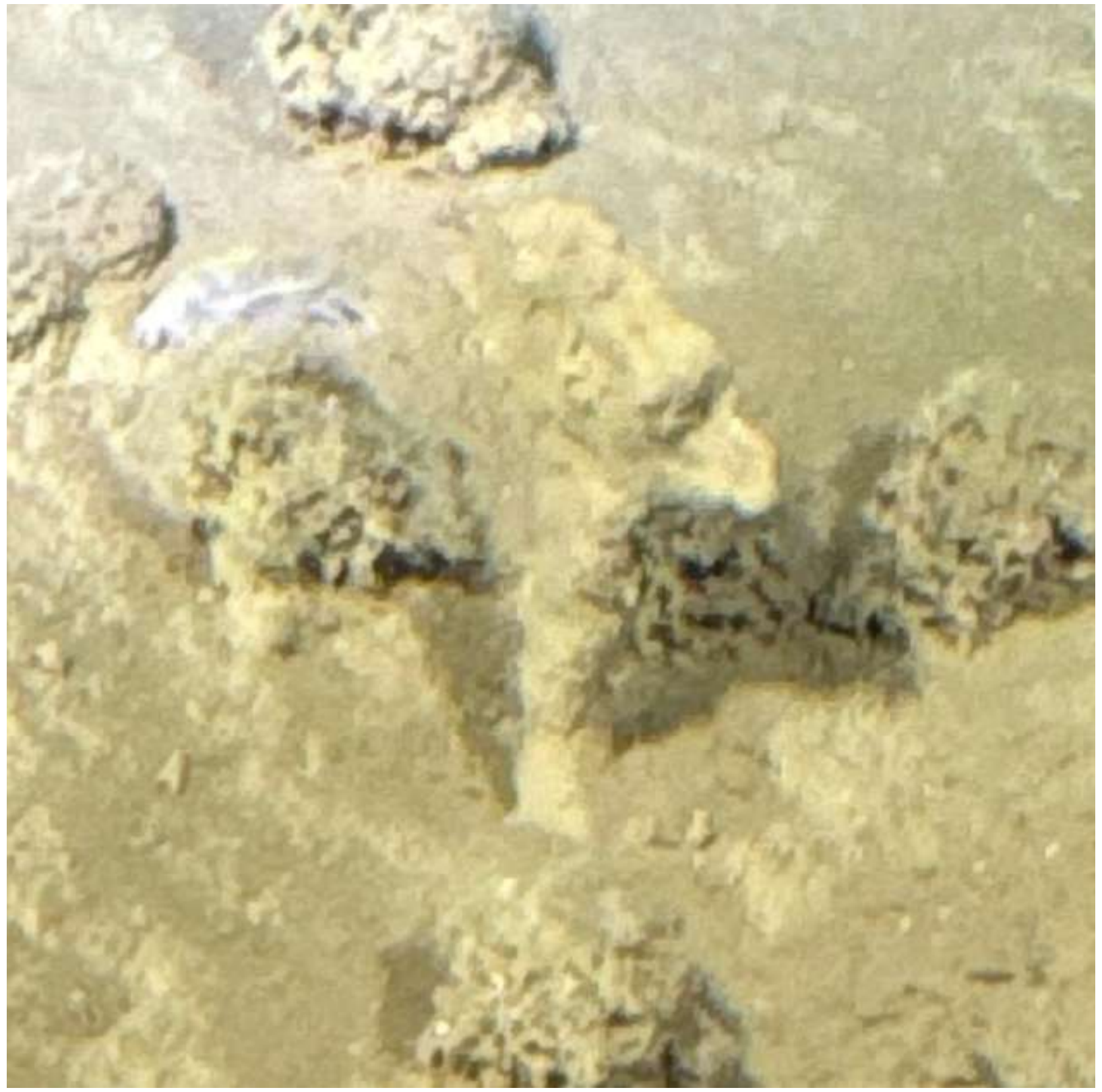

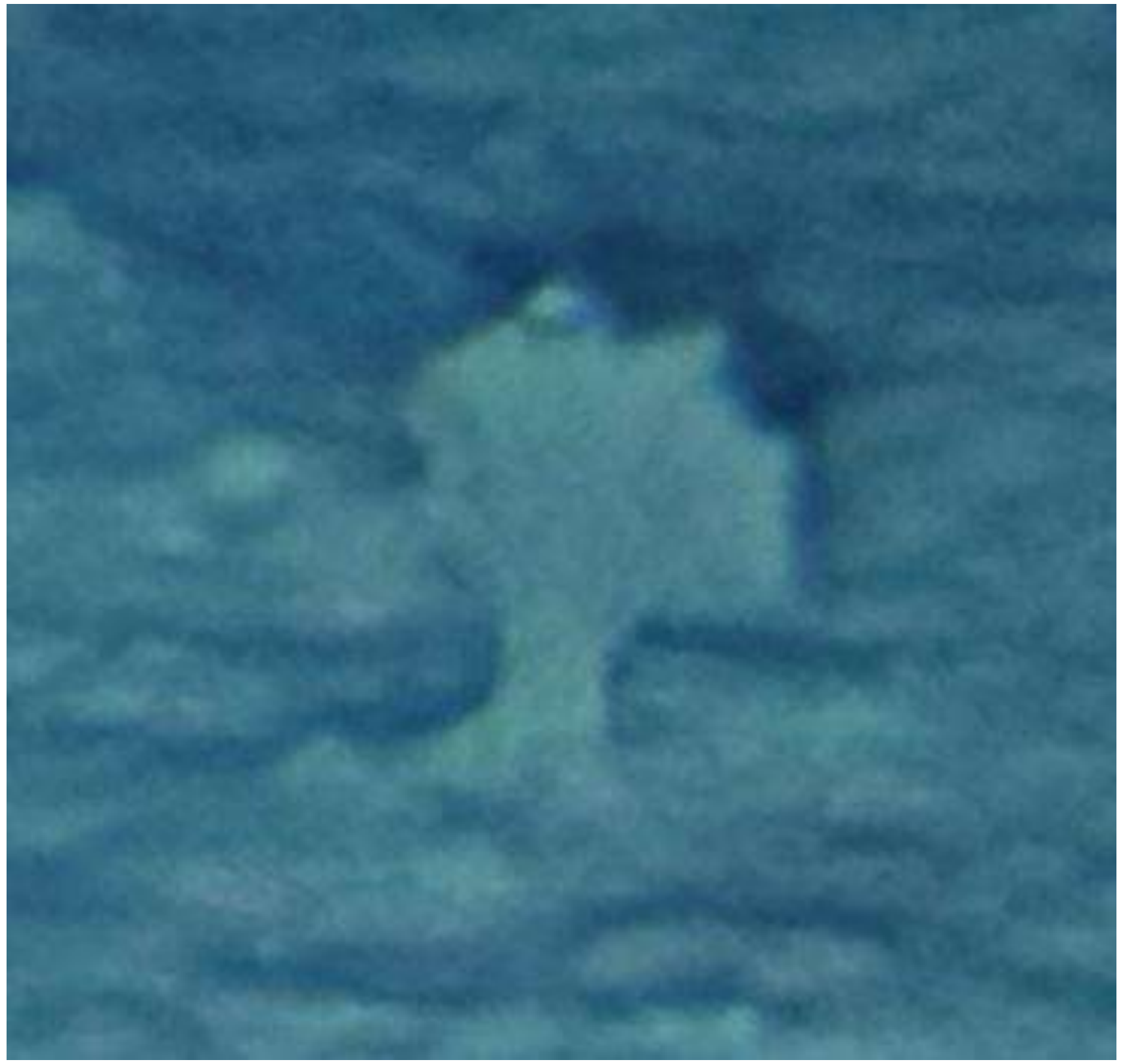

Wavy, palmate structure with notably tall trunk.

Foraminifera; Monothalamea sp. 7

NC2_BK1_3200 _IMG_6047
NC2_BK3_7400_IMG_8850

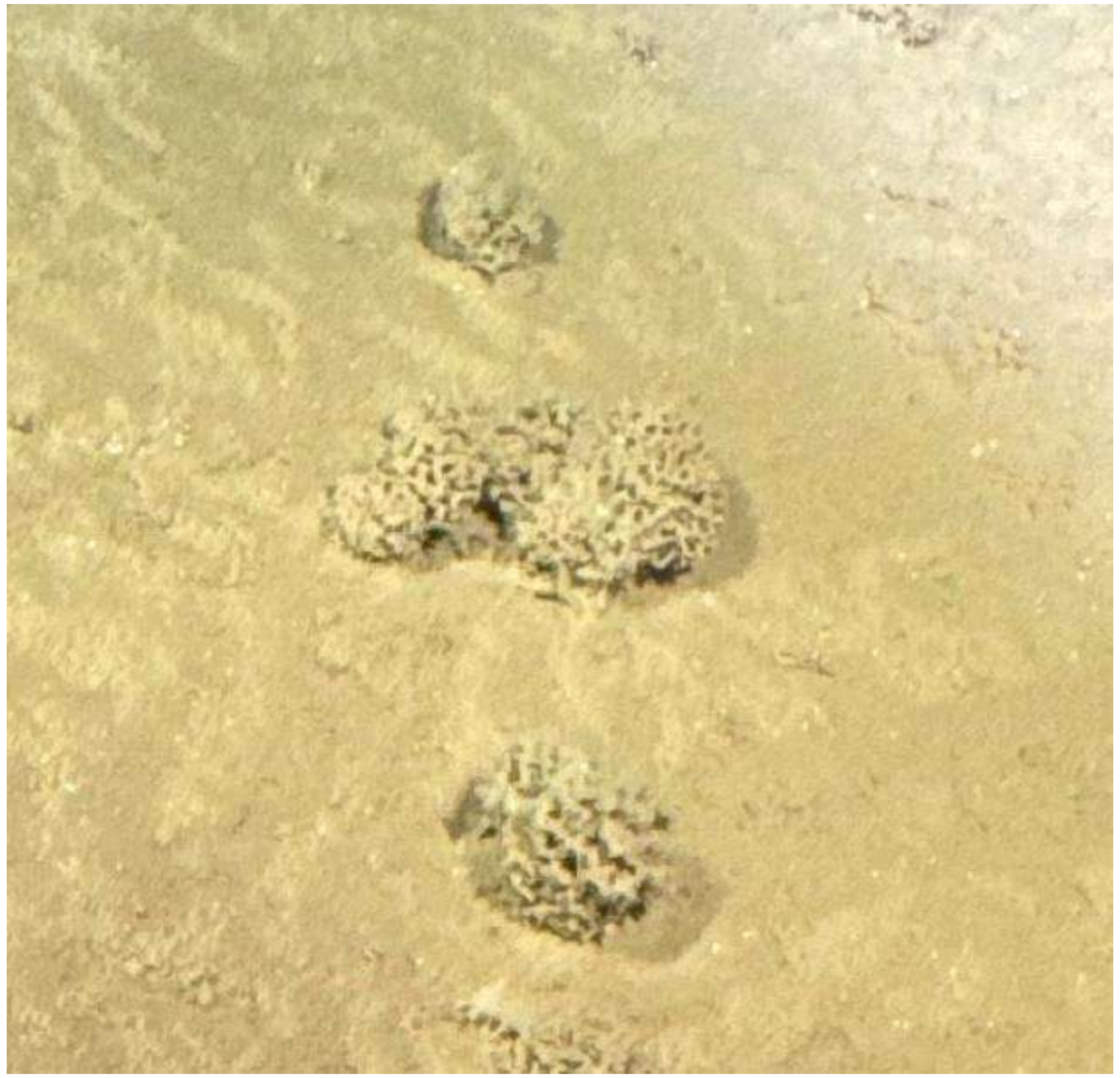

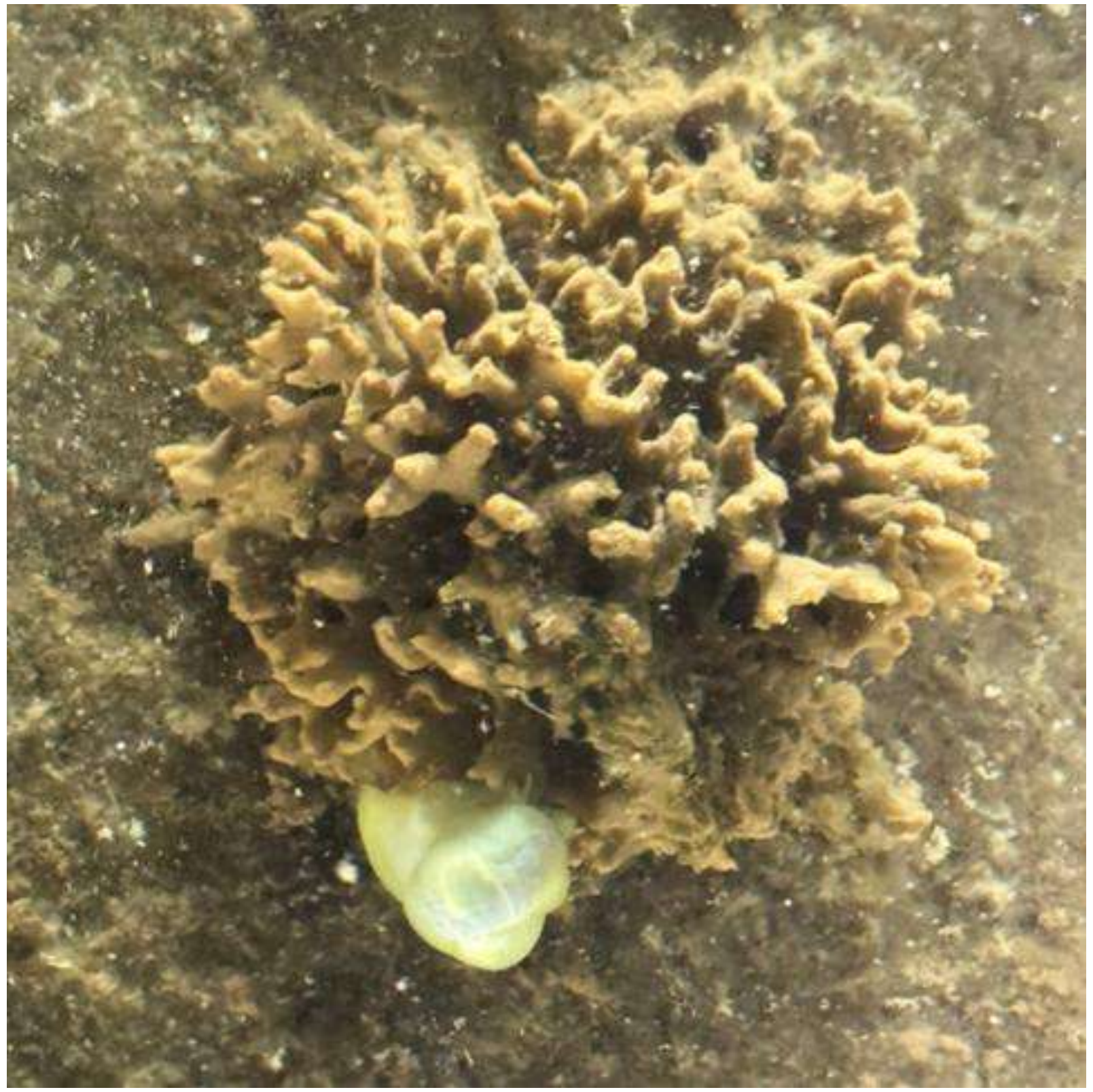

generally rounded shape, with "branches" reminiscent of finger corals.

NC3_BK1_8000_IMG_0613

Foraminifera; Monothalamea sp. 8

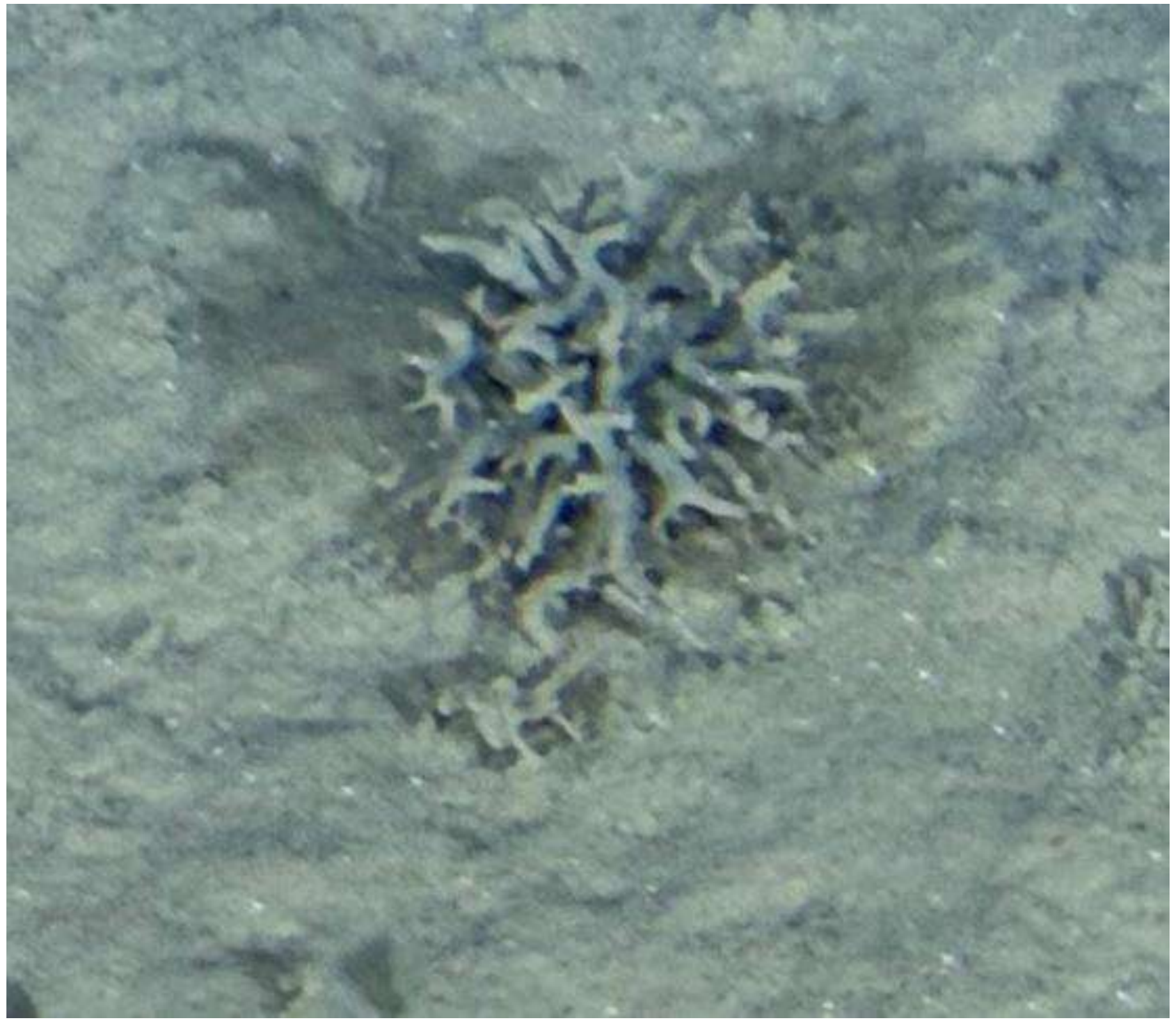

Shrub or bush-like shape with antler-like branches.

Foraminifera; Monothalamea sp. 9

NC2_BK1_3200_IMG_6954
NC2_BK2_3800_IMG_7858

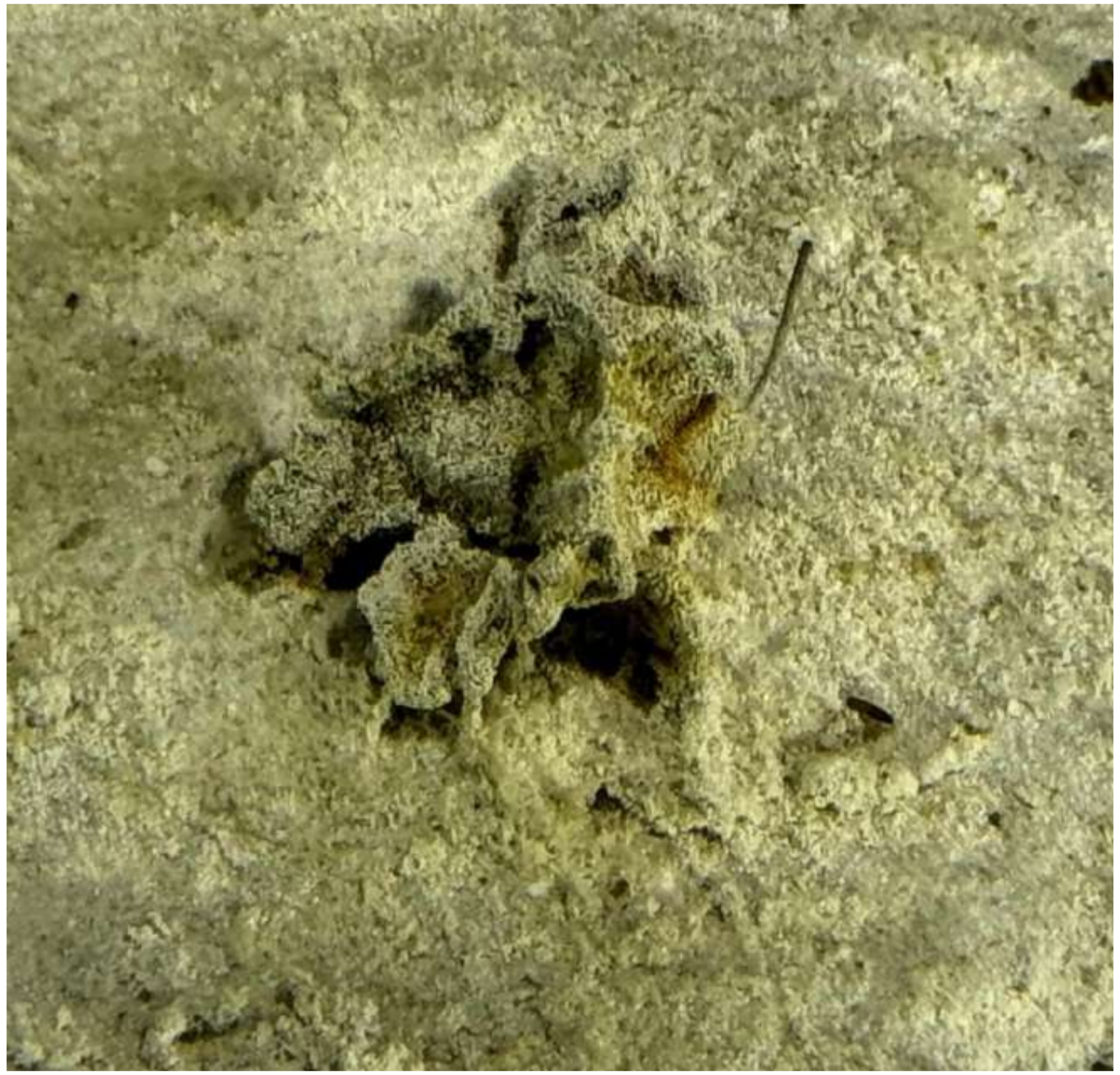

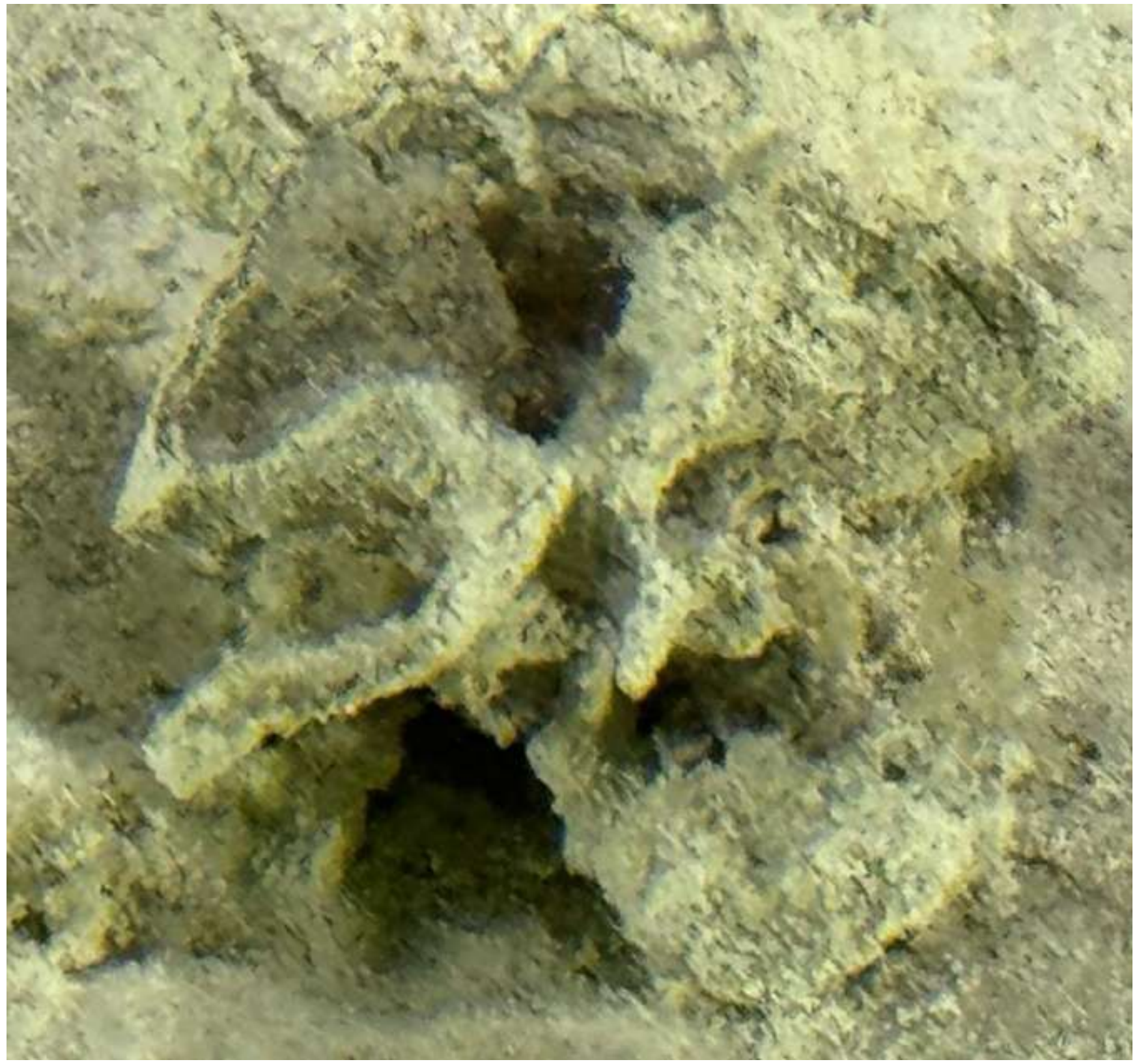

Rippled, irregular mass.

NC3_BK4_8000_IMG_3739

Foraminifera; Monothalamea sp. 10

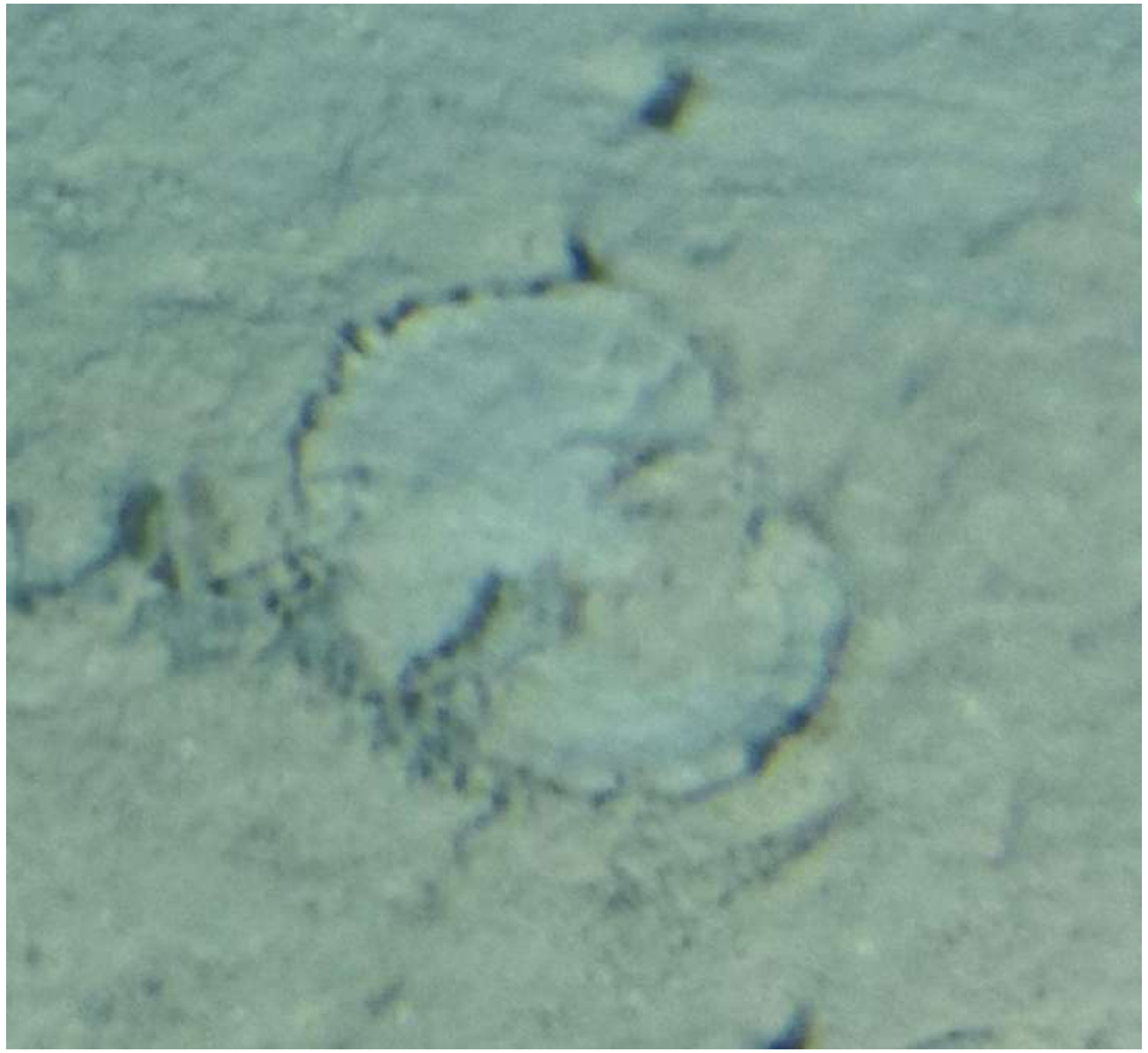

Dorsally flattened structure resembling two or semi-circles on the ocean floor surrounded by small mounds.

Foraminifera; Monothalamea sp. 11

NC2_BK4_5300_IMG_9540

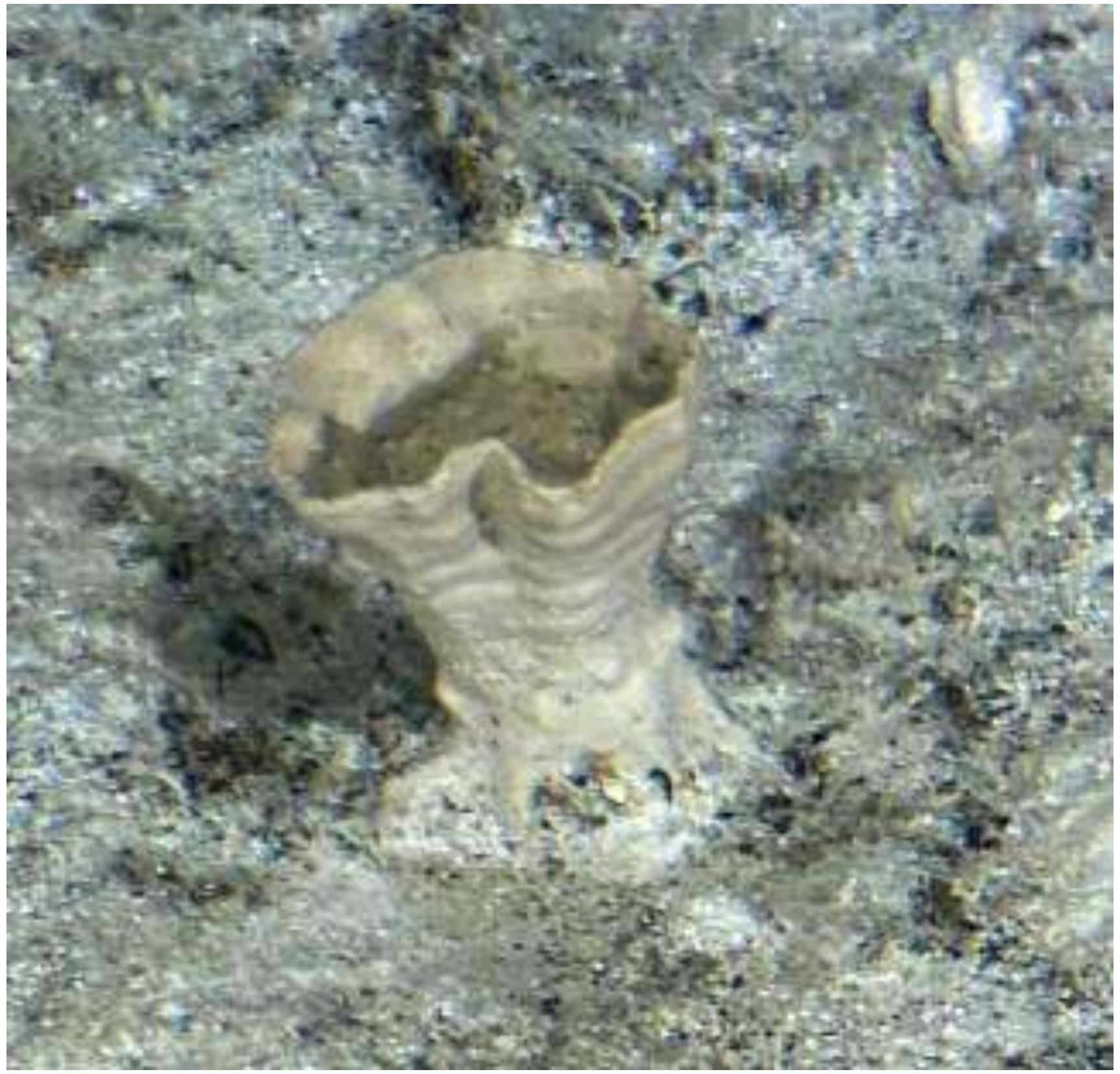

Wide cup-like structure

NC2_BK5_4600_IMG_9897

Foraminifera; Monothalamea sp. 12

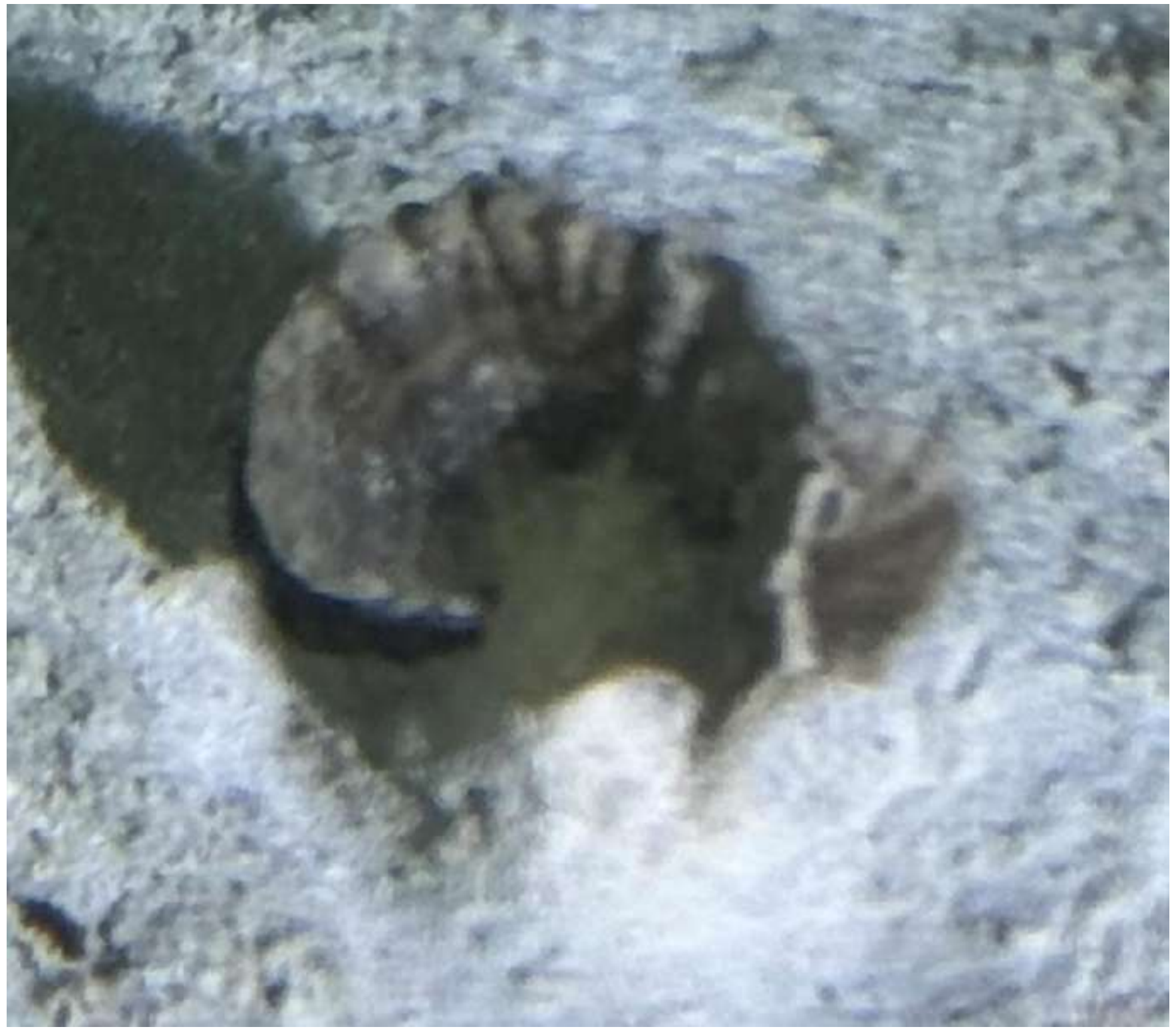

Dark coloured, rounded, wavy wall-like structure.

Foraminifera; Monothalamea sp. 13

NC1_BK1_6200_IMG_4290
NC1_BK1_6200_IMG_4298
NC1_BK1_6200_IMG_4771

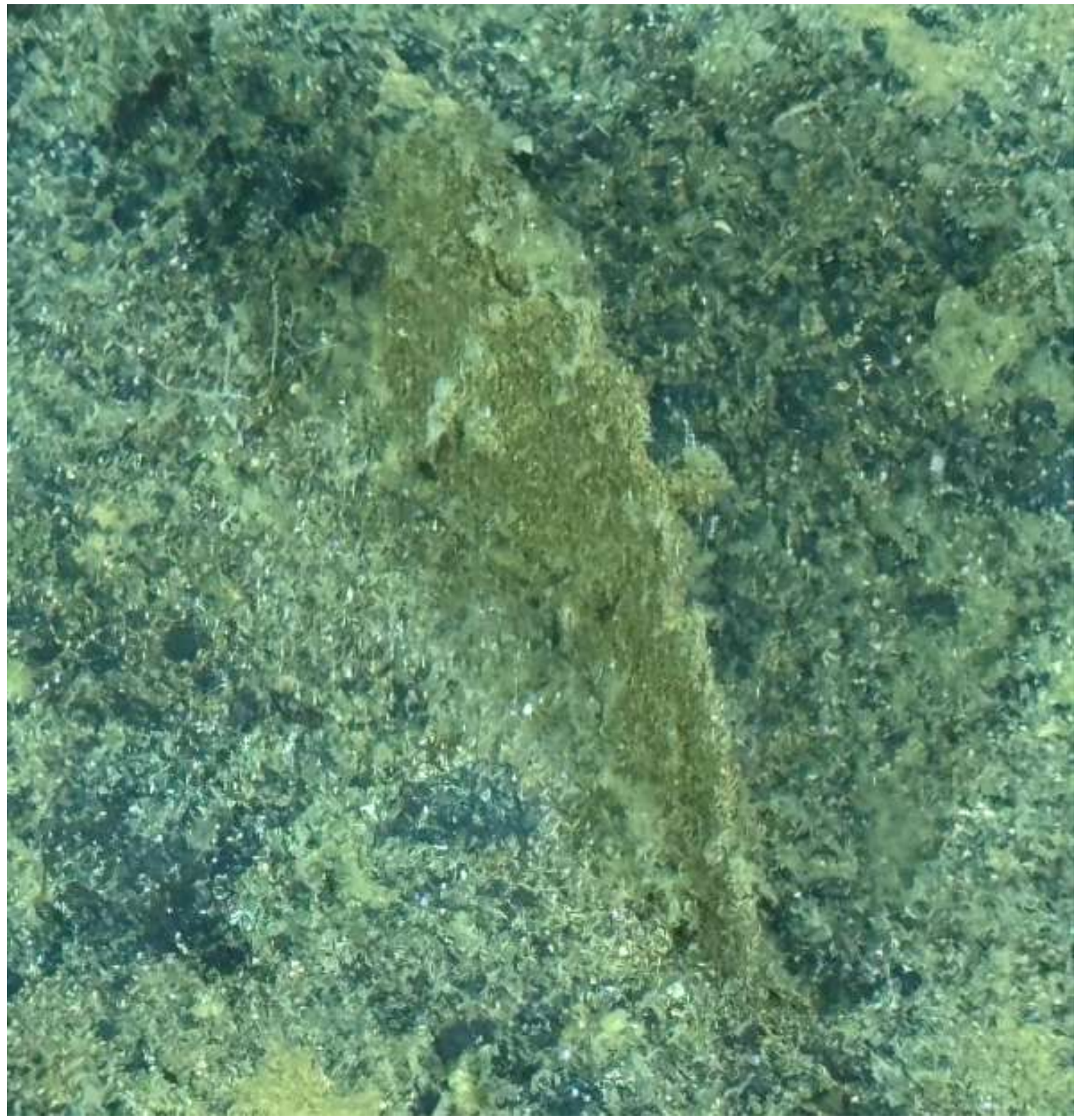
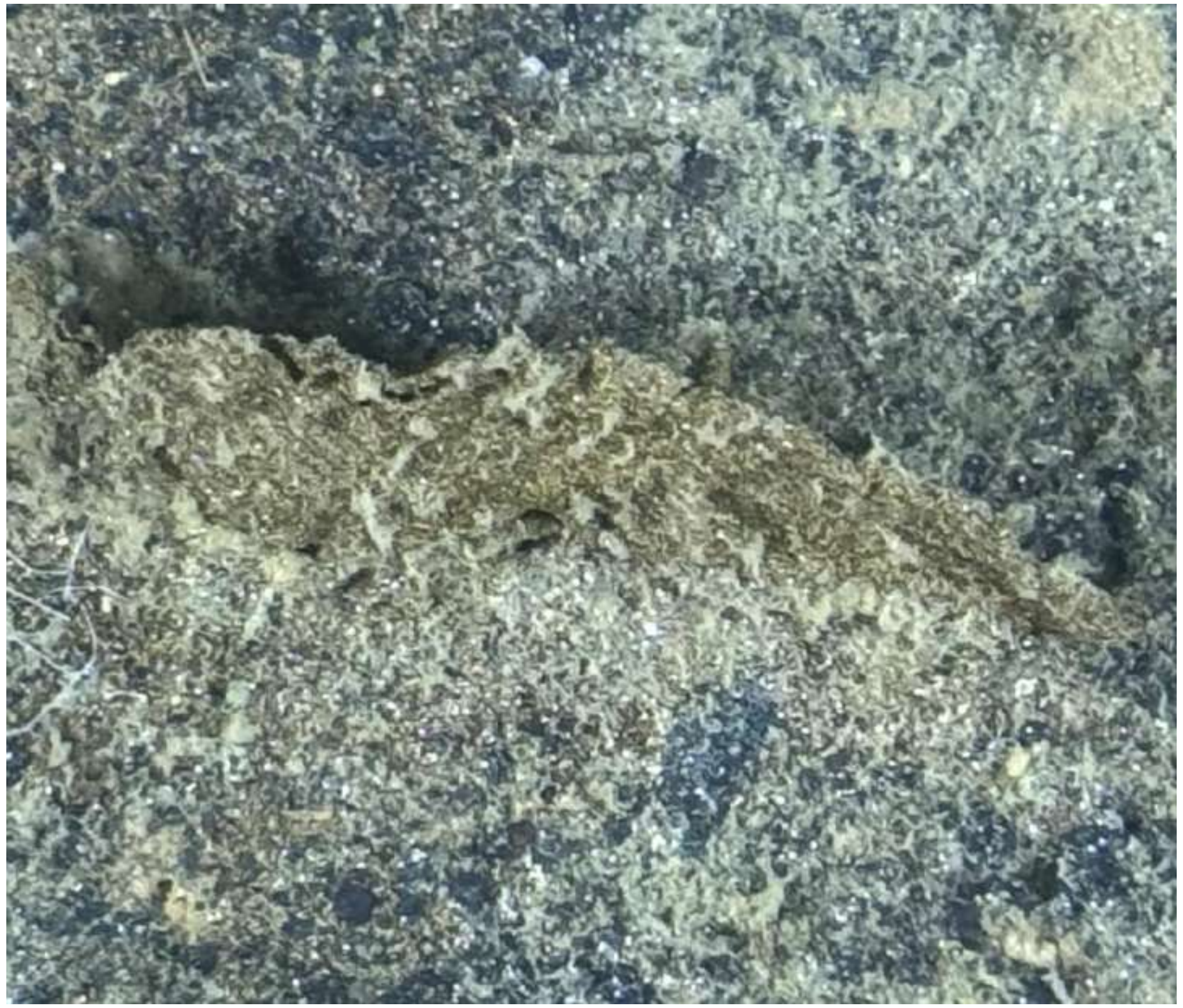
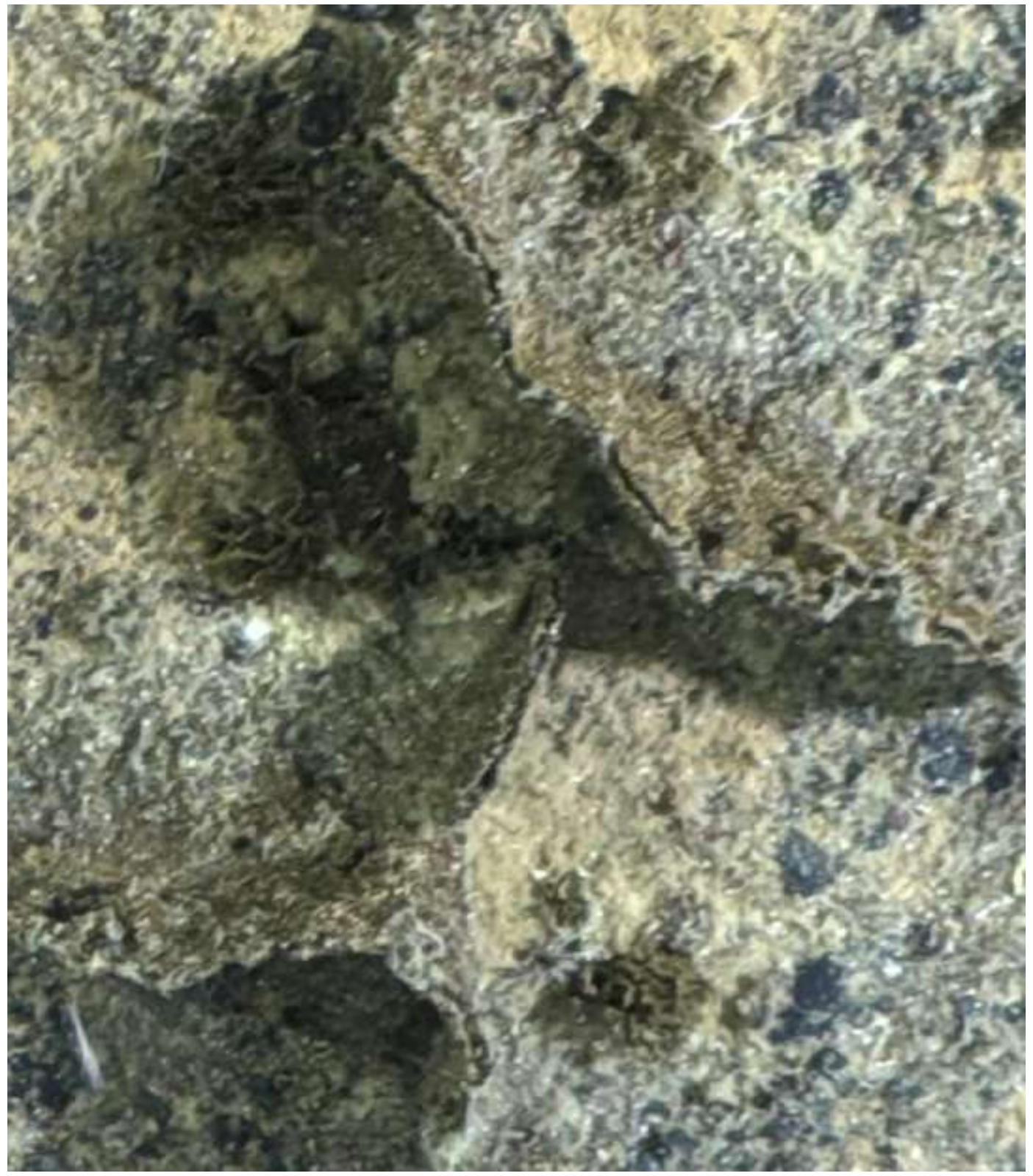

Thin test wall structure with dark interior.

Foraminifera; Monothalamea sp. 14

NC2_BK4_5300

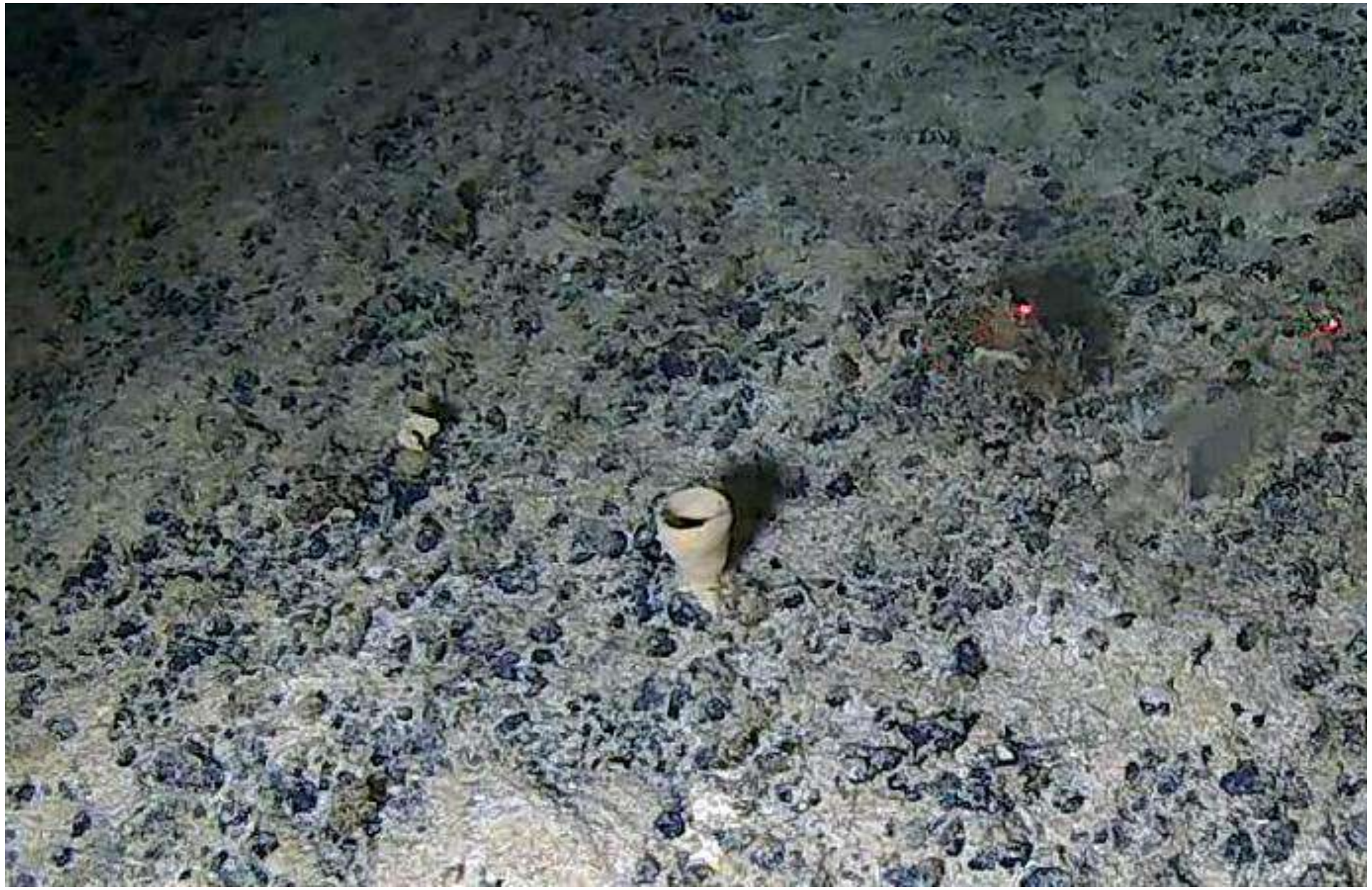

Foraminifera; Monothalamea sp. 15

NC2_BK4_5300

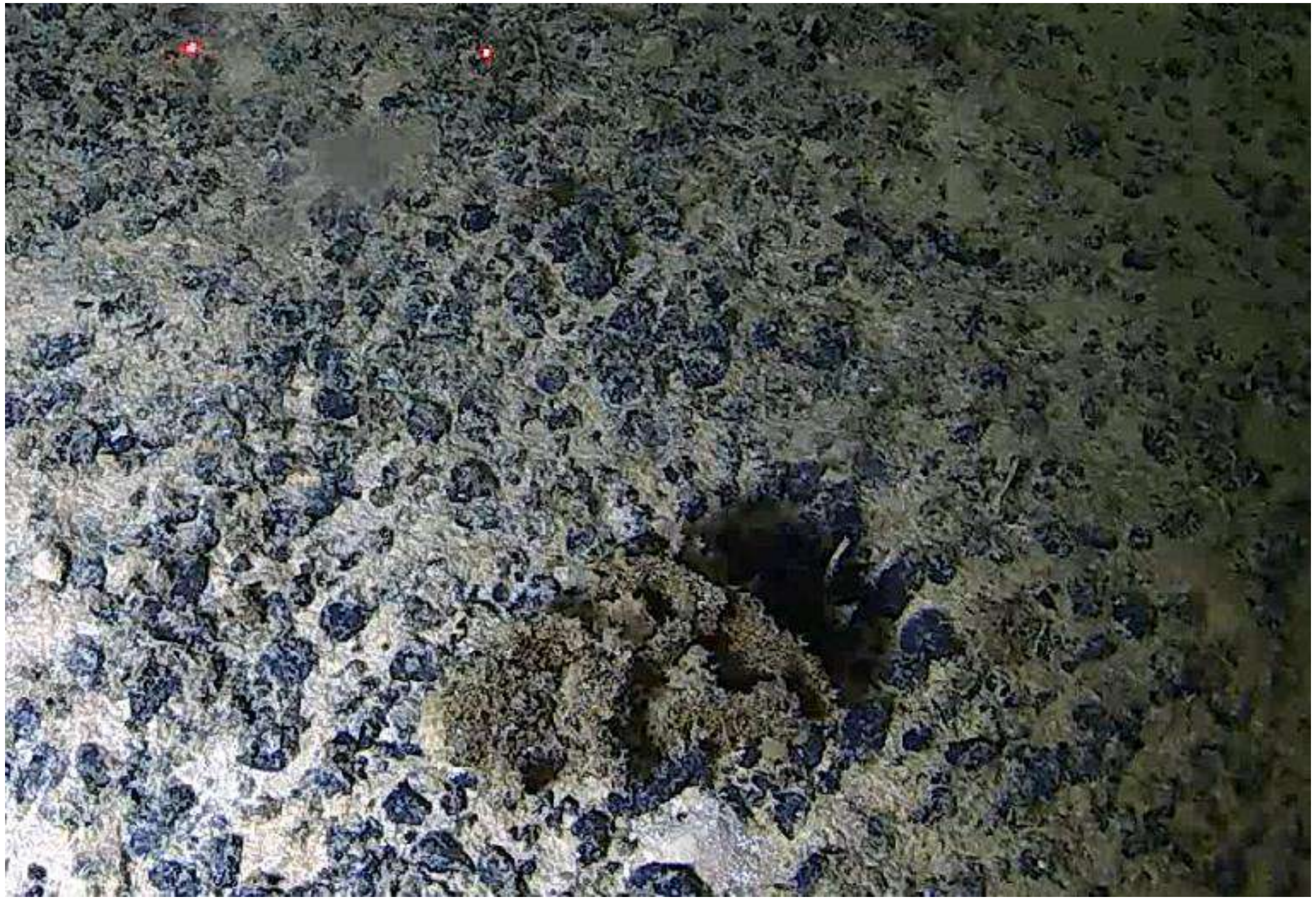

Animalia

incertae sedis

Animalia incertae sedis; Animalia incertae sedis 1

NC3_BK2_6000_IMG_1941

NC3_BK2_6000_NOR_0079

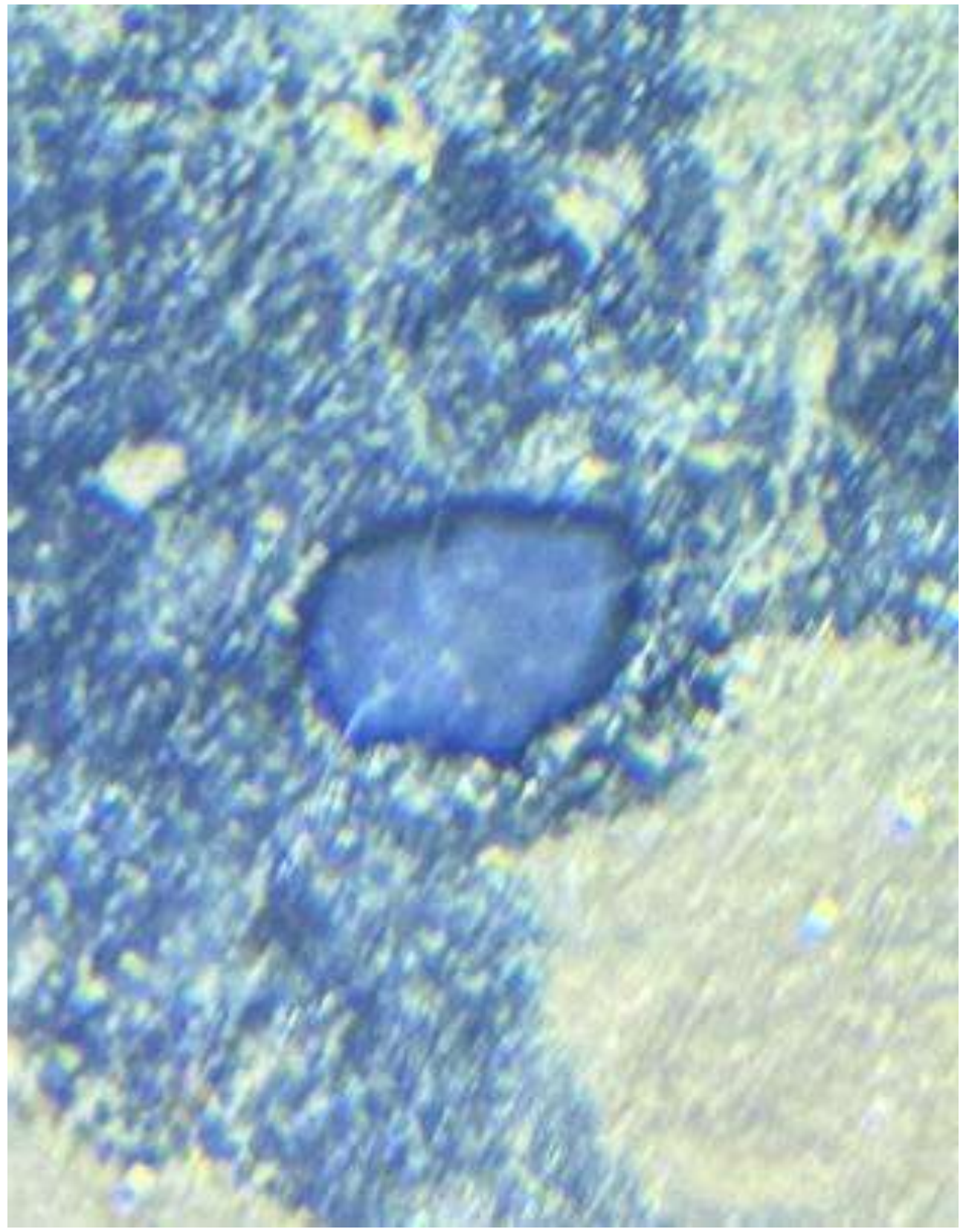

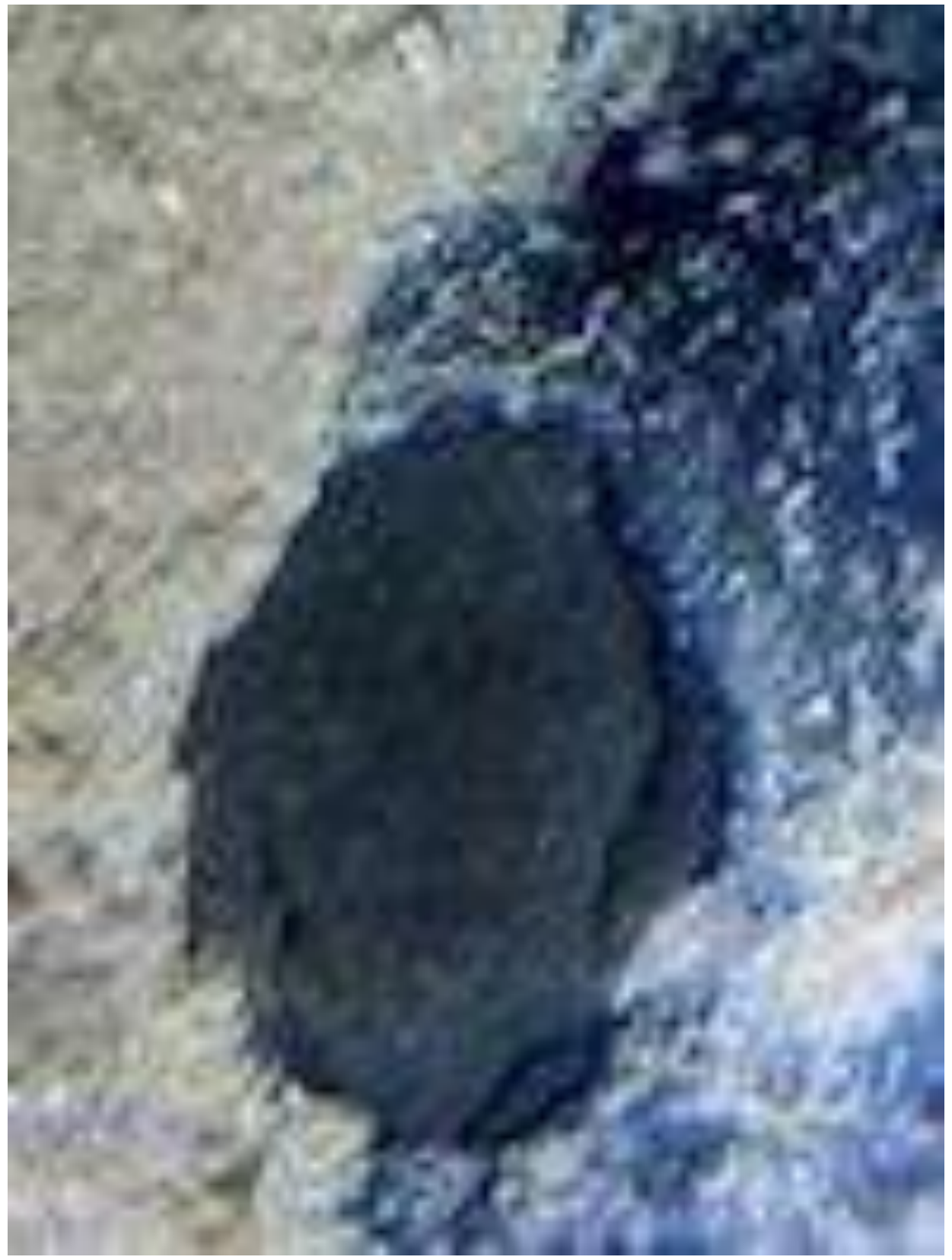

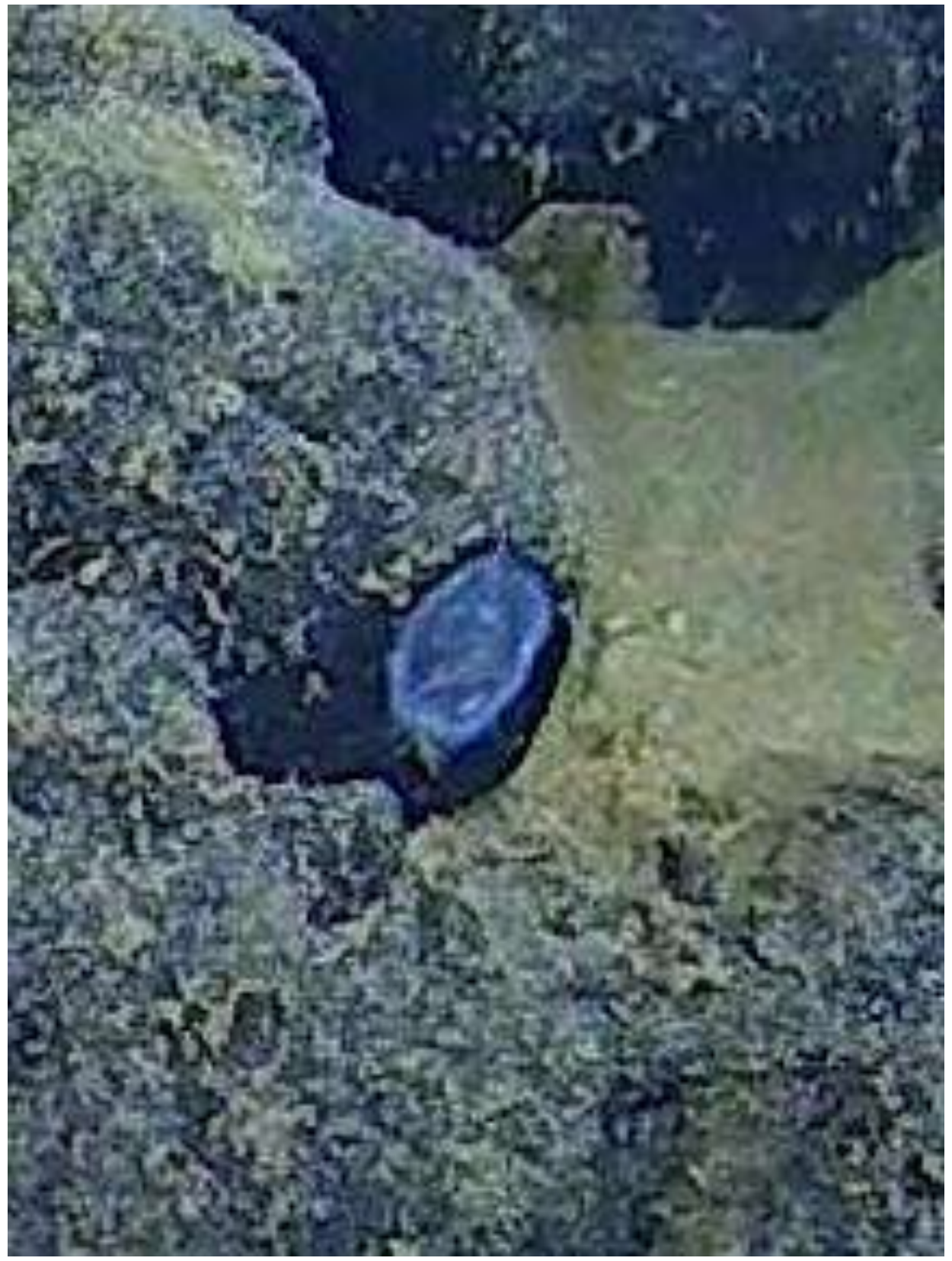

Body is dark and greyish-purple in colouration. Three papillae like appendages protrude from the dorsal side of the animal. Overall body shape is round and flattened. Cf. Platyhelminthes
